# Identification of biomarkers related to amino acid metabolism in nonobstructive azoospermia by bioinformatics

**DOI:** 10.1097/MD.0000000000048183

**Published:** 2026-05-01

**Authors:** Qing Hua Guo, Zhong Jun Ding, Dong Mei Liu, Xiao Shan Ren, XiaoYong Chen

**Affiliations:** a Andrology Department, Lanzhou University First Hospital, Lanzhou, Gansu, China; b Center for Reproductive Medicine, Gansu Provincial Maternal and Child Care Hospital, Lanzhou, Gansu, China.

**Keywords:** amino acid metabolism, biomarkers, infertility, nonobstructive azoospermia

## Abstract

Nonobstructive azoospermia (NOA) is one of the most severe types of male infertility. Amino acid metabolism (AAM) is strongly associated with various diseases. This study intended to identify biomarkers related to AAM in NOA. Biomarkers were screened from GSE9210, GSE108886, and AAM-related genes through a series of bioinformatics analyses. Then, the diagnostic efficacy of the biomarkers was evaluated. Furthermore, the mechanisms of action of these biomarkers were investigated through enrichment analysis, immune infiltration analysis, construction of regulatory networks, prediction of potential drugs, and prediction of related diseases. Finally, reverse transcription-quantitative polymerase chain reaction was performed for verification. Three biomarkers (*AKT1*, *ASNS*, and *SHC1*) were derived, which demonstrated good diagnostic efficacy for NOA. Meanwhile, *AKT1*, *ASNS*, and *SHC1* might play significant roles in the development of male germ cells through pathways such as male gamete generation, sexual reproduction, and gamete generation. Immune infiltration analysis revealed these 3 biomarkers were closely associated with T follicular helper cells, resting natural killer cells, and regulatory T cells. And *AKT1*, *ASNS*, and *SHC1* were regulated by 24 transcription factors and 15 microRNAs. Remarkably, sodium selenite, aflodac, and genistein were commonly predicted by the biomarkers. In addition, lung neoplasms were associated with *AKT1*, lipoidosis was associated with *ASNS*, and drug-induced acute liver injury was associated with *SHC1*. Ultimately, *AKT1* and *SHC1* were significantly upregulated in NOA. This study identified 3 biomarkers as being associated with NOA, providing valuable clues to help treat and predict NOA.

## 1. Introduction

Infertility affects 8% to 12% of couples globally, with male factors accounting for approximately 50% of cases.^[1]^ Notably, the prevalence of male infertility has been increasing at an annual rate of 0.29%,^[2,3]^ a trend potentially attributed to environmental factors, evolving lifestyles, and occupational stressors. Azoospermia is classified into obstructive (OA) and nonobstructive azoospermia (NOA) subtypes based on the presence or absence of sperm production defects. Nonobstructive azoospermia approximately 60% of clinical cases of azoospermia.^[4]^ As one of the most severe subtypes of male infertility, its core feature is the complete absence of sperm in the ejaculate, and this phenotype is not due to obstruction of the sperm transport ducts but rather results from congenital or acquired disorders of spermatogenesis within the testis itself.^[5]^

The diagnosis of NOA requires the integration of physical examinations, ultrasound, serum hormone testing (such as follicle-stimulating hormone), and invasive surgical exploration (such as testicular biopsy or microdissection testicular sperm extraction) for confirmation.^[6,7]^ In addition, genetic testing (e.g., karyotype analysis, Y chromosome microdeletion analysis) serves as an essential auxiliary tool.^[8,9]^ However, this diagnostic framework has limitations, including its invasive nature and the risk of sampling errors. Even microdissection testicular sperm extraction, considered the gold standard, achieves a sperm retrieval rate of only about 50%, indicating that over half of the patients will face surgical failure.^[10]^ The etiology of NOA is highly heterogeneous, with approximately 50% of cases classified as “idiopathic.”^[11,12]^ Known causes encompass genetic, congenital, endocrine, and various other factors.^[13]^ Advances in technologies such as whole-exome sequencing have uncovered additional monogenic causes (e.g., variants in genes like *CFAP47* and *HFM1*), highlighting the genetic complexity of NOA and the necessity for precise diagnosis and treatment.^[14,15]^ Furthermore, NOA imposes significant psychological and social burdens on patients, often leading to anxiety, depression, and strain on partner relationships and quality of life. These challenges, combined with economic pressures, constitute the comprehensive burden of the disease.^[16]^ Therefore, there is an urgent need to develop noninvasive molecular diagnostic tools to optimize the diagnostic process, reduce invasiveness, and enable early intervention.

Amino acids, recognized as the fundamental building blocks of proteins, function as signaling molecules and serve critical roles in maintaining energy balance and metabolic homeostasis. The metabolic processing of amino acid metabolism (AAM), constituting 1 of 3 major biochemical cascades in human physiology, encompasses pathways such as glutamine cycling, serine biosynthesis, and glycine catabolism.^[17]^ AAM dysregulation is implicated in diverse diseases, including male reproductive disorders. Specifically, AAM pathways regulate semen quality parameters such as sperm motility and DNA integrity.^[18–20]^ Various metabolic pathways, such as glycometabolism, lipid metabolism, and AAM, were disrupted in patients with NOA.^[21]^ In NOA, AAM disrupts the function of KASH5, a protein critical for meiosis, ultimately leading to the manifestation of azoospermia.^[3]^ AAM is critically implicated in the pathogenesis of NOA. Nevertheless, the identification of key biomarkers linked to AAM in NOA remains an area of limited understanding. Comprehensive studies are imperative to unravel the specific molecular pathways by which AAM-associated genes influence the onset and advancement of NOA.

This study acquired datasets related to NOA and AAM-related genes (AAMRGs) from public databases. By employing a range of bioinformatics approaches, we identified potential biomarkers associated with AAM in NOA and constructed a nomogram model based on these biomarkers. Furthermore, we investigated the potential biological functions, immune characteristics, molecular regulatory networks, and potential drug targets of these biomarkers. The mRNA expression levels of these biomarkers were also validated in clinical samples. These findings provide new insights into the diagnosis of NOA, with the potential to improve treatment outcomes for patients.

## 2. Materials and methods

### 2.1. Collation of gene expression data

GSE9210 and GSE108886 raw data were obtained from the Gene Expression Omnibus (http://www.ncbi.nlm.nih.gov/geo) database. GSE9210 (platform GPL887) was a training set, including 11 OA (OA group) as well as 47 NOA group samples of testis specimens.^[22]^ GSE108886 (platform GPL10558) was a validation set for gene expression, including 3 OA as well as 8 NOA samples of the testicular tissue.^[23]^ AAMRGs were obtained from the literature, and these genes were merged and deduplicated, resulting in a total of 2352 AAMRGs.^[24–26]^

### 2.2. Identification and functional enrichment analysis of candidate genes

Differential expression analysis of NOA versus OA samples in GSE9210 was performed to obtain differentially expressed genes (DEGs) via “limma” (v 3.54.0),^[27]^ where log_2_fold change > 0.5, *P*.adj < .05.^[28]^ In addition, DEGs were sorted by log_2_fold change and created a volcano plot and heat map to visualize the top 10 up- and downregulated DEGs by “ggplot2” (v 3.3.6)^[29]^ as well as “pheatmap” (v 1.0.12).^[30]^ Candidate genes were defined as DEGs that overlapped with AAMRGs. DEGs were gained by generating Venn diagrams with “VennDiagram” (v 1.7.3)^[31]^ Gene Ontology (GO) functional annotation, including biological processes (BPs), molecular functions (MFs), and cellular components (CCs), along with Kyoto Encyclopedia of Genes and Genomes (KEGG) analysis of candidate genes, were carried out using the “clusterProfiler” (v 4.6.2)^[32]^ (*P* < .05). Furthermore, the top 5 functions in BPs, MFs, and CCs, as well as the top 10 KEGG pathways, were displayed in order of significance based on the smallest *P* value to the largest.

### 2.3. Selection and validation of biomarkers

To explore the interplay among candidate genes, a protein–protein interaction (PPI) network was constructed using the Search Tool for Retrieval of Interacting Genes (https://string-db.org/) database (confidence = 0.4).^[33]^ The visualization of the PPI network was achieved using Cytoscape (v 3.10.2; Cytoscape Consortium, San Diego).^[34]^ Subsequently, the “Cytohubba” plugin within Cytoscape software (v 3.10.2) was utilized with the maximal clique centrality, maximum neighborhood component, and degree algorithms to filter candidate biomarkers. Specifically, genes were sorted by their interaction scores from highest to lowest, and the top 20 genes from each of the 3 algorithms were intersected. The resulting intersected genes were then selected, with the top 10 being designated as candidate biomarkers for further analysis. The expression of candidate biomarkers was analyzed in GSE9210 and GSE108886 and demonstrated by “ggplot2” (v 3.3.6). Receiver operating characteristic (ROC) curves of the candidate biomarkers were evaluated using the “pROC” (v 1.18.0).^[35]^ In both datasets, candidate biomarkers that showed significant inter-group differences with consistent trends and an area under the curve (AUC) >0.7 were identified as biomarkers.

### 2.4. Nomogram model construction of biomarkers

In the GSE9210, based on the expression levels of the biomarkers and using the presence or absence of disease as the outcome event, a nomogram model for the biomarkers was constructed using the “rms” (v 6.5-0).^[36]^ Subsequently, calibration curves were plotted by “regplot” (v 1.1; https://rdrr.io/cran/regplot/) to assess the predictive ability of the nomogram model. The closer the slope of the calibration curve was to 1, the better the accuracy of the model’s predictions. For a deeper evaluation of the nomogram model’s clinical effectiveness, decision curve analysis curves were charted utilizing the “rmda” (v 1.6).^[37]^ The ROC curve was plotted utilizing the “pROC” (v 1.18.0), and the forecasting accuracy of the nomogram model for NOA samples was evaluated via the AUC > 0.7.

### 2.5. Gene set enrichment analysis of biomarkers

To further identify markedly altered bioprocesses of the biomarkers in GSE9210, Spearman correlation analysis was performed between each biomarker and the remaining genes via “psych” (v 2.2.9).^[38]^ Subsequently, all biomarkers were ranked according to the magnitude of the correlation coefficients (cor). Using the ranking results, gene set enrichment analysis was conducted by “clusterProfiler” (v 4.6.2; *P* < .05 as well as normalized enrichment score > 1).^[39]^ The gene set (“c2.cp.kegg.v7.4.symbols.gmt”) was obtained from the Molecular Signatures Database (https://www.gsea-msigdb.org/gsea/msigdb). Finally, the top 5 signaling pathways were visualized using the “clusterProfiler” (v 4.6.2).

### 2.6. Immune infiltration analysis

In GSE9210, the proportion of 22 immune cells^[40]^ in NOA versus OA samples was estimated by cell-type identification by estimating relative subsets of RNA transcripts algorithm. Then, the Wilcoxon test (*P* < .05) was applied to assess differences in immune cell abundance in NOA versus OA groups with the “ggpubr” (v 0.6.0).^[41]^ The outcomes were visualized by plotting box plots with the “ggplot2” (v 3.3.6). Spearman correlation analysis was performed by “psych” (v 2.2.9) to calculate the associations between differential immune cells and between differential immune cells versus biomarkers. The correlation heat map was generated via “psych” (v 2.2.9), with a threshold set at cor > 0.3 and *P* value < .05.

### 2.7. Regulatory network construction, gene–disease association analysis, and potential drug prediction

Transcription factors (TFs) of biomarkers were acquired using the NetworkAnalyst (https://networkanalyst.ca/NetworkAnalyst/) database and JASPAR (https://jaspar.elixir.no/) database. The microRNAs (miRNAs) were acquired by miRDB (https://www.mirdb.org), miRanda (https://ngdc.cncb.ac.cn/databasecommons/database/id/1426), and TargetScan (https://targetscan.org) databases by “multiMiR” (v 1.18.0),^[42]^ and the intersection of the results predicted by 3 databases was taken as the key miRNAs. Cytoscape software (v 3.10.2) was employed to visualize both the TFs-biomarkers regulatory network and the miRNA-biomarkers regulatory network. Furthermore, to identify diseases associated with the biomarkers, the DisGeNET (http://www.disgenet.org) database was utilized to search for diseases related to the biomarkers. The diseases were sorted by their association scores in descending order, and the top 10 diseases associated with each biomarker were selected for visualization by Cytoscape software (v 3.10.2). To further explore potential drugs interacting with the biomarkers, the Drug Signatures Database (https://dsigdb.tanlab.org/DSigDBv1.0/) was searched for drugs related to biomarkers (*P*.adjust < .05), and the top 10 drugs predicted by the biomarkers were visualized with the help of Cytoscape software (v 3.10.2). Moreover, to explore the binding ability between biomarkers and drugs, molecular docking was carried out. Toxic drugs did not undergo molecular docking. The 3-dimensional structures of biomarkers and drugs were obtained from Protein Data Bank (https://www.rcsb.org/) and PubChem (https://pubchem.ncbi.nlm.nih.gov/) databases, respectively. The structure of the protein that could not be found was obtained from the AlphaFold database (https://alphafold.ebi.ac.uk/). Then, biomarkers and drugs were subjected to molecular docking via the CB-DOCK2 website (https://cadd.labshare.cn/cb-dock2/index.php).

### 2.8. Reverse transcription-quantitative polymerase chain reaction

To verify the reverse transcription-quantitative polymerase chain reaction (RT-qPCR) results of the biomarker, researchers collected tissue samples from 5 OA tissue samples and 5 NOA recipients.^[43,44]^ The inclusion criteria for all participants are as follows: aged between 20 and 40 years, a relatively healthy lifestyle (including no excessive alcohol consumption, no smoking, moderate physical activity), no history of substance abuse, no exposure to toxic substances in the work environment, no adherence to a high-fat or high-sugar diet, and no major systemic diseases. Patients with NOA included in the study met the following criteria: no sperm detected in at least 3 semen analyses according to the World Health Organization laboratory manual for human semen (5th edition) and confirmed by microscopic examination after centrifugation; meeting the diagnostic criteria for NOA based on physical examination, imaging analysis, and hormone levels. The OA group consisted of men hospitalized for assisted reproduction due to OA who had successfully fathered a child. There were no statistically significant differences between the 2 groups in terms of age, lifestyle habits, medication use, and work environment. In addition, all participants had a normal karyotype, with no Y-chromosome microdeletions, anatomical abnormalities, varicocele, prostatitis, genital infections, testicular trauma, or hematospermia.^[44]^

To validate the biomarkers identified through bioinformatic analysis, this study performed RT-qPCR assays using independent clinical samples. A total of 5 OA tissue samples and 5 NOA tissue samples were collected to verify the expression levels of the target biomarkers. The RT-qPCR experiment was approved by the Ethics Committee of the First Hospital of Lanzhou University (Approval No: LDYYLL-2025-837), and written informed consent was obtained from all participants. Total RNA was extracted from the 5 pairs of tissue samples using TRIzol reagent (Ambion, Austin), and RNA quality was assessed with a Nanodrop N50 spectrophotometer (Thermo Fisher Scientific, Waltham). Total RNA was reverse-transcribed into complementary DNA (cDNA) using the SweScript First Strand cDNA Synthesis Kit (Servicebio, Wuhan, China). The reaction system consisted of 4 μL of 5× reaction buffer, 1 μL of primer, 1 μL of SweScript RT I Enzyme Mix, 0.1 ng to 5 μg of total RNA, and RNase-free water to a total volume of 20 μL. The reaction conditions were as follows: 25°C for 5 minutes, 50°C for 15 minutes, 85°C for 5 seconds, followed by holding at 4°C. Quantitative polymerase chain reaction was performed according to the manufacturer’s instructions. The reaction system included 3 μL of cDNA, 5 μL of 2× Universal Blue SYBR Green qPCR Master Mix (Servicebio, Wuhan, China), and 1 μL of 10 μM forward and reverse primers. The amplification conditions were the following: pre-denaturation at 95°C for 1 minute, followed by 40 cycles of denaturation at 95°C for 20 seconds, annealing at 55°C for 20 seconds, and extension at 72°C for 30 seconds. Each sample was run in triplicate. Primer specificity was verified using melt curve analysis, which showed a single peak for all primers. Glyceraldehyde-3-phosphate dehydrogenase (*GAPDH*) was used as the internal reference gene, and the relative gene expression levels were calculated using the 2^−△△Ct^ method. The △Ct value was the difference between the Ct value of the target gene and that of the internal reference gene, and the △△Ct value was the difference between the △Ct values of the OA group and the NOA group. The GraphPad Prism 10 (GraphPad Software, San Diego)^[45]^ was employed to plot and calculate the *P* value. The detailed sequences of the primers are provided in Table S1, Supplemental Digital Content, https://links.lww.com/MD/R751.

### 2.9. Statistical analysis

R software (v 4.2.2) was used for all data analyses. The Wilcoxon test was performed to analyze variation between the NOA and OA groups. Differences between RT-qPCR experimental groups were obtained through *t* test. Statistical significance was set at *P* < .05.

## 3. Results

### 3.1. GO functional annotation and KEGG enrichment analysis of 115 candidate genes

**Figure 1.**
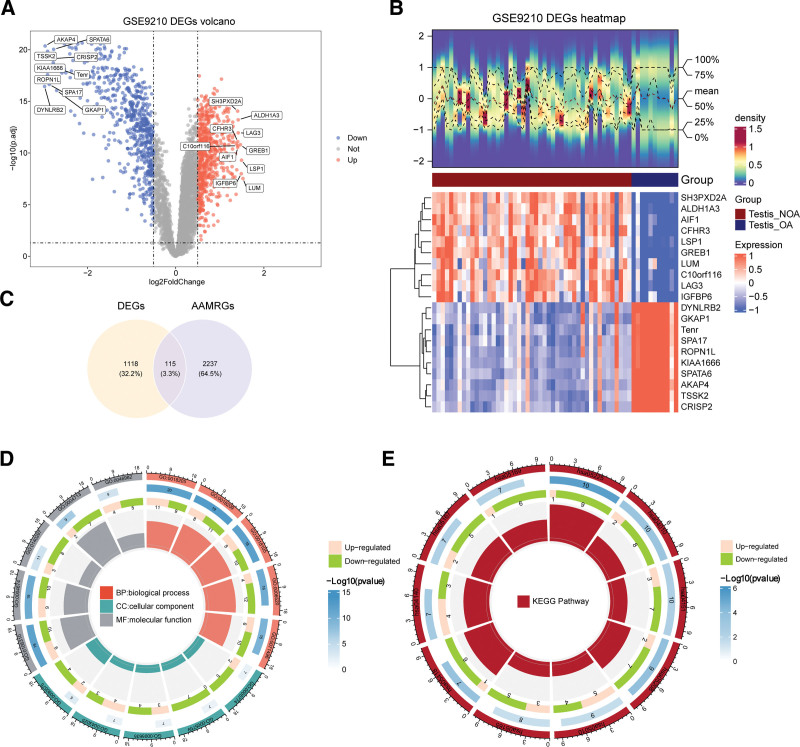
GO functional annotation and KEGG enrichment analysis of 115 candidate genes. (A) Volcano plot of DEGs between NOA group and OA group. (B) Heatmap of DEGs between NOA group and OA group. (C) Venn diagram of candidate genes. (D) Candidate gene GO enrichment results. (E) Enrichment results of candidate genes in KEGG pathways. DEGs = differentially expressed genes, GO = Gene Ontology, KEGG = Kyoto Encyclopedia of Genes and Genomes, NOA = nonobstructive azoospermia, OA = obstructive azoospermia.

In GSE9210, 1233 DEGs were screened in NOA versus OA groups, comprising 620 genes (*SH3PXD2A*, *ALDH1A3*, *LAG3*, etc) with upregulated expression, and 613 genes (*SPATA6*, *AKAP4*, *CRISP2*, etc) with downregulated expression (Fig. 1A and B). Therefore, the 1233 DEGs and 2352 AAMRGs were intersected, resulting in the identification of 115 candidate genes (Fig. 1C). A total of 1074 GO entries were enriched by 115 candidate genes, which included 908 in BPs (such as peptidyl-lysine modification, peptidyl-serine modification, and peptidyl-serine phosphorylation), 54 in CCs (such as transcription repressor complex, histone acetyltransferase complex, and chromatoid body), and 112 in MFs (such as protein serine kinase activity, protein serine/threonine kinase activity, and protein tyrosine kinase activity; Fig. 1D, Table S2, Supplemental Digital Content, https://links.lww.com/MD/R751). As revealed by the KEGG enrichment analysis, the 115 candidate genes were mostly associated with hepatocellular carcinoma, MAPK, and PI3K-Akt signaling pathway (Fig. 1E, Table S3, Supplemental Digital Content, https://links.lww.com/MD/R751).

### 3.2. Biomarkers acquired by PPI network, expression verification, and ROC

**Figure 2.**
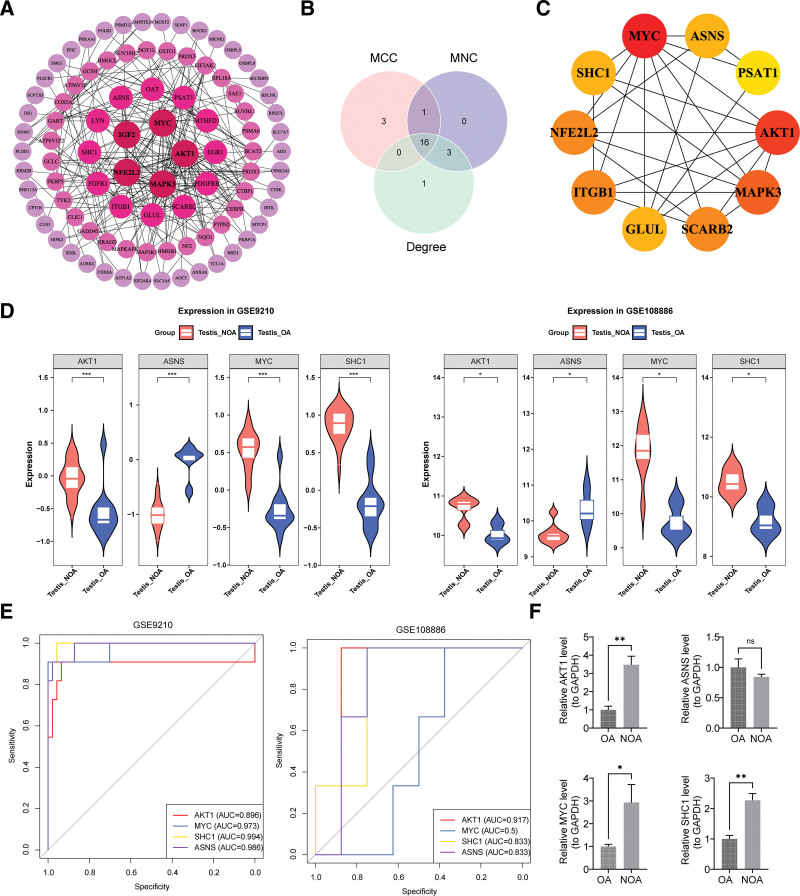
Biomarkers acquired by PPI network, expression verification, and ROC. (A) The PPI network of candidate genes. (B) Intersection of the top 20 candidate genes from the 3 algorithms. (C) Candidate biomarkers’ PPI network. (D) Expression analysis of candidate biomarkers. ns represents insignificant, *P < .05, **P < .01, ***P < .001, ****P < .0001. (E) ROC curve. (F) Comparison of expression differences. ns represents insignificant,*P < .05, **P < .01, ***P < .001, ****P < .0001. AUC = area under the curve, MCC = maximal clique centrality, MNC = maximum neighborhood component, NOA = nonobstructive azoospermia, OA = obstructive azoospermia, PPI = protein–protein interaction, ROC = receiver operating characteristic.

The PPI network contained 90 nodes and 252 edges, with AKT1, MAPK3, NFE2L2, insulin-like growth factor 2, and MYC exhibiting stronger interactions with other proteins than the rest of the network (Fig. 2A). Based on the 3 algorithms, 16 intersecting genes were obtained (Fig. 2B). The top 10 genes with the highest interaction scores were selected as candidate biomarkers, which were *AKT1*, *MYC*, *MAPK3*, *NFE2L2*, *SHC1*, *SCARB2*, *GLUL*, *PSAT1*, *ITGB1*, and *ASNS* (Fig. 2C). Among them, *AKT1*, *ASNS*, *MYC*, and *SHC1* showed consistent expression trends in GSE9210 and GSE108886 (*P* < .05; Fig. 2D). In both datasets, the AUC values for *AKT1*, *ASNS*, and *SHC1* were all >0.7; therefore, they were identified as biomarkers (Fig. 2E). In addition, RT-qPCR showed that *AKT1* and *SHC1* were significantly upregulated in NOA (*P* < .05). Unfortunately, *ASNS* was downregulated in the NOA group, but the difference was not significant (*P* > .05; Fig. 2F).

### 3.3. Nomogram model of AKT1, ASNS, and SHC1

**Figure 3.**
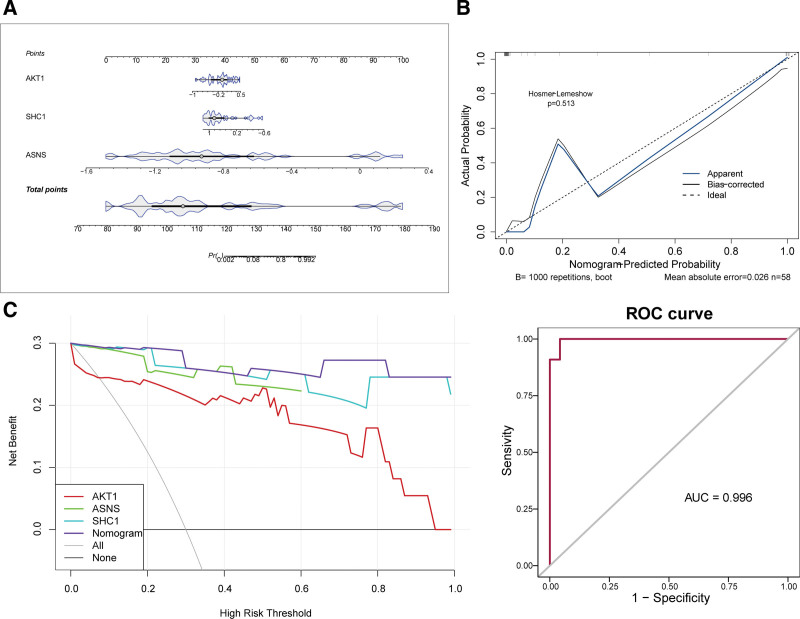
Nomogram model of *AKT1*, *ASNS*, and *SHC1*. (A) Nomogram. (B) Validation of the nomogram model – calibration curve. (C) Validation of the line graph model – decision curve and ROC curve. AUC = area under the curve, ROC = receiver operating characteristic.

*AKT1*, *ASNS*, and *SHC1* were the biomarkers to construct the NOA diagnostic nomogram (Fig. 3A). Most of the slopes of the calibration curves were close to 1, indicating that the nomogram model had predictive accuracy for NOA (Fig. 3B). Decision curve analysis curve showed biomarker and nomogram model curves mostly above the “All” and “None,” confirming the good performance of the nomogram (Fig. 3C). In addition, the ROC curve revealed that the nomogram model displayed a noteworthy diagnostic value for NOA (AUC = 0.996; Fig. 3C).

### 3.4. Gene set enrichment analysis of AKT1, ASNS, and SHC1

**Figure 4.**
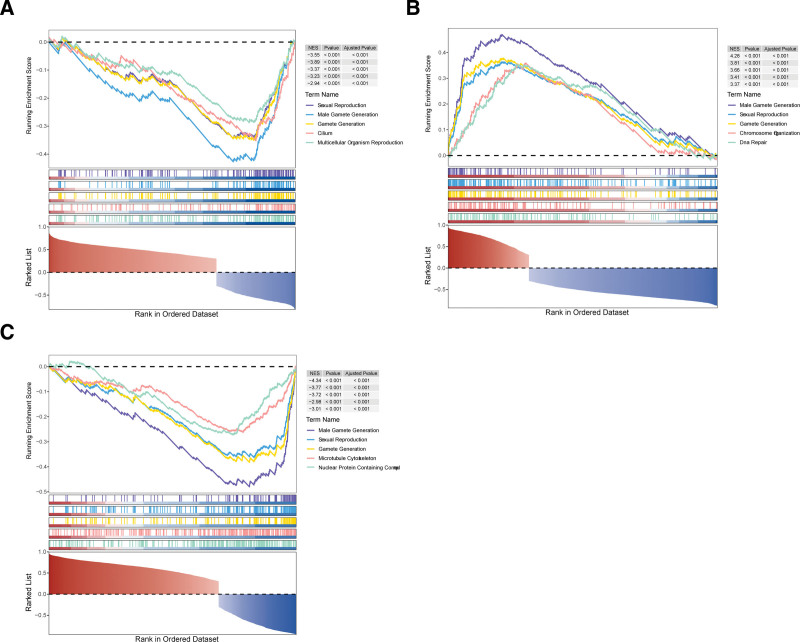
Gene set enrichment analysis of the biomarkers. (A) *AKT1*. (B) *ASNS*. (C) *SHC1*. NES = normalized enrichment score.

*AKT1*, *ASNS*, and *SHC1* were, respectively, enriched in 223, 509, and 643 pathways (Tables S4–S6, Supplemental Digital Content, https://links.lww.com/MD/R751). The top 5 signaling pathways, sexual reproduction, male gamete generation, and female gamete generation were commonly enriched by *AKT1*, *ASNS*, and *SHC1* (Fig. 4A–C). This suggested that *AKT1*, *ASNS*, and *SHC1* might play a crucial role in the development of germ cells, particularly male germ cells.

### 3.5. Immune infiltration analysis of AKT1, ASNS, and SHC1

**Figure 5.**
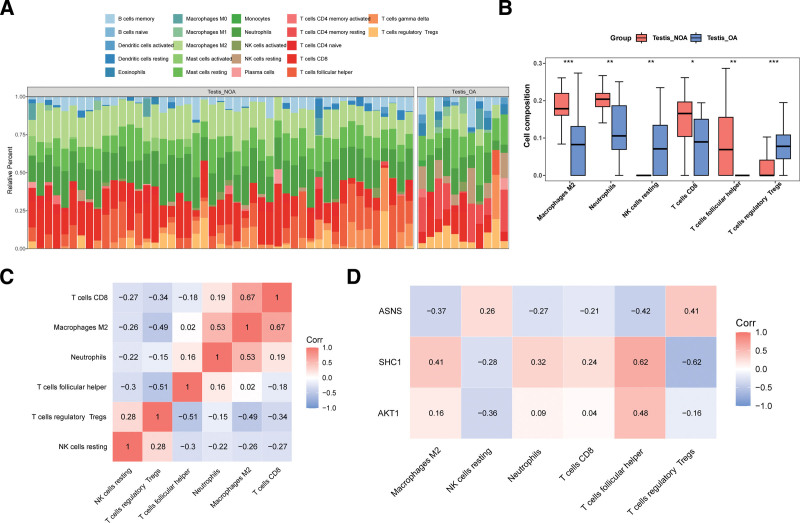
Immune infiltration analysis of *AKT1*, *ASNS*, and *SHC1*. (A) The proportion of 22 types of immune cells in each sample. (B) Box plot of enrichment scores of 12 types of immune infiltrating cells based on NOA/OA groups. ns represents insignificant,*P < .05, **P < .01, ***P < .001. (C) Heatmap showing the correlations among the 6 types of immune cells. (D) Heatmap showing the correlations between 3 biomarkers and 6 types of immune cells. NOA = nonobstructive azoospermia, OA = obstructive azoospermia.

In GSE9210, the proportions of immune cell infiltration, such as M2 macrophages, in the NOA versus OA group were shown in Figure 5A. Wilcoxon test revealed 6 immune cells exhibited significant differences; among them, M2 macrophages, CD8 T cells, neutrophils, and T follicular helper (Tfh) cells were upregulated in the NOA group (Fig. 5B). Spearman correlation analysis revealed that 6 differential immune cells mostly showed significant negative correlations, in which CD8 T cells showed the strongest positive correlation with M2 macrophages (cor = 0.67, *P* < .05), while Tfh cells showed the strongest negative correlation with regulatory T cells (Tregs, cor = −0.51, *P* < .05; Fig. 5C). Besides, *AKT1* showed the strongest positive correlation with Tfh cells (cor = 0.48, *P* < .05) and the strongest negative correlation with resting natural killer cells (cor = −0.36, *P* < .05). *ASNS* exhibited the strongest positive correlation with Tregs (cor = 0.41, *P* < .05) and the strongest negative correlation with Tfh cells (cor = −0.42, *P* < .05). *SHC1* had the strongest positive correlation with Tfh cells (cor = 0.62, *P* < .05) and the strongest negative correlation with Tregs (cor = −0.62, *P* < .05; Fig. 5D).

### 3.6. Regulatory network of AKT1, ASNS, and SHC1

**Figure 6.**
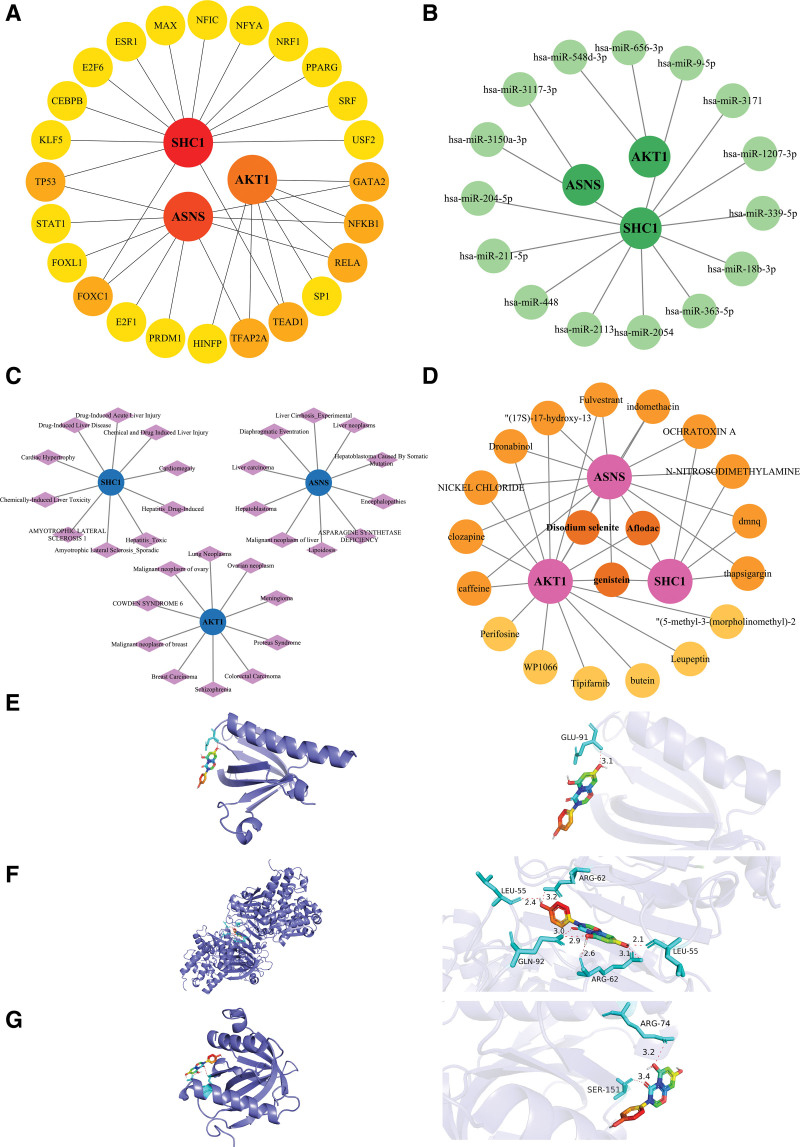
Regulatory network of *AKT1*, *ASNS*, and *SHC1*. (A) Biological marker – transcription factor regulatory network diagram. (B) Regulatory network diagram of biomarker – miRNA. (C) Biological markers – predictive drug network. (D) Association analysis diagram of genes and diseases.

In this study, we identified the TFs associated with biomarkers and found that *AKT1* was regulated by 7 TFs (e.g., *GATA2*, *NFKB1*, and *RFLA*), *ASNS* was regulated by 10 TFs (e.g., *TP53*, *STAT1*, and *FOXL1*), as well as *SHC1* was regulated by 14 TFs (e.g., *E2F6*, *MAX*, and *NFYA*; Fig. 6A). A total of 2 key miRNAs (e.g., hsa-miR-548d-3p) for *AKT1*, 1 key miRNA (e.g., hsa-miR-3117-3p) for *ASNS*, and 12 key miRNAs (e.g., hsa-miR-204-5p) for *SHC1* were predicted by databases (Fig. 6B). In addition, disease association analysis indicated that, for instance, lung neoplasms were related to *AKT1*, lung lipoidosis was associated with *ASNS*, and drug-induced acute liver injury was related to *SHC1* (Fig. 6C). Remarkably, disodium selenite, aflodac, and genistein were predicted for the biomarkers (Fig. 6D). Among them, disodium selenite and aflodac were toxic substances, so genistein was subjected to molecular docking with the biomarkers. The binding energies of *AKT1* (Fig. 6E), *ASNS* (Fig. 6F), and *SHC1* (Fig. 6G) with genistein were −6.1, −9.0, and −7.1 kcal/mol, respectively (Table 1). The binding of *ASNS* with genistein was the strongest, suggesting that *ASNS* might be a potential drug target.

**Table 1:** The binding ability between biomarkers and drugs.

Biomarkers	Drugs	Binding energy (kcal/mol)
*AKT1*	Genistein	−6.1
*ASNS*		−9
*SHC1*		−7.1

## 4. Discussion

NOA, accounting for 60% to 70% of azoospermia cases, presents significant diagnostic and therapeutic challenges due to its reliance on invasive procedures and only 50% sperm retrieval success rate despite advanced microsurgical techniques.^[46,47]^ Emerging evidence indicates that dysregulated AAM contributes to NOA pathogenesis through mechanisms such as meiotic protein (e.g., KASH5) dysfunction, although specific AAM-related biomarkers remain unidentified.^[3]^ Our bioinformatics study identified 3 AAM-associated diagnostic biomarkers (*AKT1*, *ASNS*, *SHC1*), which were validated by nomogram modeling (AUC > 0.9) and shown to be functionally enriched in gametogenesis pathways. Multi-omics analyses revealed *SHC1*’s immunomodulatory role in T-cell regulation and constructed transcriptional regulatory networks (TF-miRNA), while also screening 382 potential targeted drugs, thereby providing novel mechanistic insights and therapeutic avenues for NOA.

*AKT1* (protein kinase Bα) is a serine/threonine kinase. As the central node of the PI3K/AKT/mTOR pathway, it plays a key role in the regulation of cell proliferation, survival, and other processes.^[48]^ As a key mediator of intracellular signaling, *AKT1* activation is triggered by diverse upstream stimuli, including insulin, platelet-derived growth factor, insulin-like growth factor 1, epidermal growth factor, cytokines, and nutrient availability. In humans, the AKT kinase family comprises 3 isoforms (AKT1-3), all of which have been reported to be upregulated in various cancers where they function as oncogenes promoting tumor proliferation.^[49]^ Beyond its well-characterized role in oncogenesis, emerging evidence implicates *AKT1* in other pathological processes. For instance, *AKT1*-mediated mitochondrial autophagy in macrophages has been shown to confer apoptosis resistance and pro-fibrotic polarization, contributing to pulmonary fibrosis.^[50]^ In the field of reproduction, the association between *AKT1* and reproductive function has increasingly drawn attention. Studies have shown that the ratio of p-PTEN/PTEN to p-AKT1(S473)/AKT1 in fertile women is higher than that in infertile women.^[51]^ Animal research further suggests that the organophosphorus pesticide chlorpyrifos can induce male infertility by inducing reactive oxygen species and disrupting the PI3K-AKT pathway.^[52]^ However, there was previously a lack of direct evidence linking *AKT1* to human infertility. This study provides new direct insights by identifying *AKT1* as a biomarker related to NOA. This discovery expands the biological function of *AKT1* from areas such as cancer to male reproductive pathophysiology, suggesting that it may be involved in the NOA process through metabolic regulation. Mechanistically, *AKT1* can affect spermatogenesis by regulating multiple processes such as the proliferation, apoptosis, energy metabolism, and DNA damage repair of germ cells.^[53]^ Studies have shown that the activity of the PI3K/AKT pathway in the testicular tissue of NOA patients is downregulated, which may damage the self-renewal of spermatogonial stem cells and hinder the meiotic process.^[54]^ Animal model studies provide support for this mechanism: in a rat model of NOA, the traditional Chinese medicine “Shengjing Capsule” can upregulate the expression of integrin α6/β1 and PI3K/AKT pathway proteins, accompanied by an increase in sperm production in the testis, indicating that the activation of *AKT1* helps restore impaired spermatogenic function.^[54]^ These findings collectively provide potential experimental evidence for therapeutic strategies targeting *AKT1*. ASNS (asparagine synthetase) is a key metabolic enzyme that plays a crucial role in cellular AAM and is expressed in periportal hepatocytes.^[55]^ *ASNS* expression levels are associated with the progression and poor prognosis of certain solid tumors, such as clear cell renal cell carcinoma, and may influence T-lymphocyte infiltration in the tumor microenvironment.^[56]^ In addition, mutations in the *ASNS* gene have been linked to asparagine synthetase deficiency, a condition characterized by developmental delay, intellectual disability, and neurological abnormalities.^[57]^ Although in vitro studies suggest that *ASNS* is involved in adaptive responses to cellular metabolic stress, its precise regulatory mechanisms in vivo remain incompletely understood.^[55]^ In this study, we observed significant upregulation of *ASNS* in testicular tissues of patients with NOA, and this upregulation was linked to alterations in the immune microenvironment (e.g., Treg infiltration). Although there is no direct research clearly indicating that *ASNS* gene variations are the causative factor of NOA, multiple multi-omics integrated analysis studies have suggested a potential association between the 2. For instance, through the combined analysis of transcriptome and proteome data, it was found that there are widespread metabolic pathway disorders in the testicular tissues of NOA patients, with particularly significant alterations in AAM, oxidative phosphorylation, and glycolysis pathways.^[58,59]^ These metabolic abnormalities may disrupt the functional integrity of supporting cells (Sertoli cells), thereby affecting their ability to provide nutritional support and regulation for germ cells. However, the exact role of *ASNS* in NOA requires further experimental validation, such as through gene knockout or overexpression models to investigate its effects on germ cell metabolism and immune regulation.

SHC1 (Src homology 2 domain-containing transforming protein 1) is a pivotal signaling adaptor protein that plays central regulatory roles in diverse biological processes. As a key mediator of receptor tyrosine kinase signaling pathways, *SHC1* facilitates the transduction of various growth factors and cytokine signals through its SH2 domains.^[60,61]^ Extensive studies have demonstrated *SHC1*’s crucial involvement in tumorigenesis, with its aberrant expression and hyperphosphorylation being closely associated with the progression of multiple malignancies,^[62]^ rendering it a potential diagnostic biomarker and therapeutic target for cancers.^[63,64]^ Notably, beyond its oncogenic functions, *SHC1* also participates in physiological processes such as neuronal apoptosis regulation^[65]^ and immune cell signaling.^[66]^ The latest research, through multidimensional analysis, identified *SHC1* and fibroblast growth factor receptor 1 as novel immune-related oxidative stress biomarkers in NOA.^[67]^ Existing studies have confirmed that abnormalities in key processes such as oxidative phosphorylation, reactive oxygen species metabolism, and MAPK signaling pathways can induce mitochondrial dysfunction and accumulation of DNA damage, thereby triggering germ cell apoptosis or meiotic arrest.^[68]^ From a molecular mechanism perspective, *SHC1* can mediate signal transmission between upstream receptor tyrosine kinases and downstream effector molecules through its PTB and SH2 domains, thereby regulating the activity of PI3K/AKT and RAS/RAF/MEK/ERK pathways. The PI3K/AKT pathway plays a crucial role in maintaining the self-renewal of spermatogonial stem cells and promoting meiotic initiation, and the inhibition of this pathway has been proven to be closely related to the occurrence of NOA.^[69]^ Therefore, the abnormal expression of *SHC1* may interfere with the homeostasis of the above key signaling axes, disrupt the normal physiological balance of the spermatogenic epithelium, and ultimately participate in the pathological process of NOA. In our study, we similarly observed significant upregulation of *SHC1* in testicular tissues of NOA patients, showing close correlation with alterations in the immune microenvironment. Compared with previous reports, our discoveries further expand the understanding of *SHC1*’s role in male reproductive disorders. These findings not only validate the significance of *SHC1* in male infertility research but also suggest its potential involvement in NOA pathogenesis through multifaceted mechanisms encompassing “oxidative stress-immune modulation-metabolic reprogramming.”

However, the role of *SHC1* in NOA remains understudied. Our findings reveal that *SHC1* expression is markedly upregulated in testicular tissues of NOA patients, exhibiting exceptional diagnostic accuracy. In addition, functional analysis highlights oxidative stress and immune dysregulation as key pathways associated with *SHC1* activity, potentially underpinning its contribution to NOA pathophysiology.

Our study revealed that *AKT1*, *ASNS*, and *SHC1* are significantly enriched in key biological processes, including male gamete generation, sexual reproduction, chromosome organization, and DNA repair. This finding suggests these genes may play critical roles in male germ cell development. The results align well with existing research: first, the collagen α3(IV) chain, a key component of the testicular basement membrane, can activate the mTORC1/rpS6/Akt1/2-Cdc42-EB1 signaling pathway through MMP9-mediated proteolytic cleavage of its C-terminal NC1 domain, thereby regulating blood–testis barrier remodeling and sperm release.^[70]^ Second, environmental pollutants like ATMBPA can affect testicular Leydig cell function via the ESR1-CDK2/4-AKT pathway.^[71]^ Third, meiotic defects leading to homologous recombination disorders (e.g., DSB repair deficiency) have been confirmed as important pathogenic mechanisms of NOA.^[72]^ Notably, among approximately 15% of global infertility cases attributed to male factors, common phenotypes include oligoasthenoteratozoospermia. While high-throughput sequencing has identified pathogenic genes such as *TEX11* and *DNAH1*, most genetic mechanisms still require further elucidation through expanded clinical cohorts and animal models.^[73]^ Collectively, this evidence supports *AKT1*, *ASNS*, and *SHC1* as crucial molecular nodes regulating male reproduction, whose functional abnormalities may contribute to NOA pathogenesis by affecting key processes like meiosis and blood-testis barrier homeostasis.

Immune infiltration analysis revealed an association between *SHC1* expression levels and Tfh cells and Tregs abundance, suggesting that *SHC1* expression regulation may play a significant role in sustaining normal germ cell homeostasis. Furthermore, we constructed regulatory networks involving TF-mRNA and mRNA–miRNA interactions for the 3 biomarkers to identify their potential regulatory mechanisms within cells. By predicting potential targeted drugs for the biomarkers and retrieving disease associations through literature mining, we elucidated the relationships between these biomarkers and various diseases. These findings lay a solid foundation for future in-depth investigations into these biomarkers. Overall, our study offers valuable insights for the diagnosis and clinical management of NOA patients while providing a theoretical framework to advance understanding of the pathogenesis of NOA. The methodology and findings presented here have been carefully differentiated from existing literature to ensure originality.

In this study, we leveraged a comprehensive array of advanced bioinformatics methodologies to identify 3 key biomarkers (*AKT1*, *ASNS*, and *SHC1*) associated with NOA. While these findings may offer valuable insights for diagnostic strategies and therapeutic interventions in NOA patients, as well as provide a theoretical basis for unraveling the molecular mechanisms underlying this condition, several limitations warrant acknowledgment. First, the limited sample size derived from Gene Expression Omnibus datasets may constrain the statistical power of our bioinformatics analysis, necessitating validation through larger-scale cohort studies to confirm the robustness of the identified biomarkers. Second, while our computational pipeline highlights the utility of systems biology approaches in biomarker discovery, bridging the gap between these computational discoveries and their clinical translation remains a critical challenge requiring further investigation. Specifically, the development of efficient pathways to integrate these findings into diagnostic workflows or therapeutic modalities demands interdisciplinary collaboration. In addition, experimental validation through in vitro cellular models and in vivo animal studies is imperative to corroborate the functional relevance of these biomarkers in germ cell biology and pathophysiological processes. Such studies would not only strengthen the biological plausibility of our computational predictions but also avoid potential redundancies with prior studies by focusing on mechanistic exploration rather than mere biomarker identification.

## Acknowledgments

We would like to express our sincere gratitude to all individuals and organizations who supported and assisted us throughout this research. Special thanks to the following authors: Zhong Jun Ding. In conclusion, we extend our thanks to everyone who has supported and assisted us along the way. Without your support, this research would not have been possible.

## Author contributions

**Conceptualization:** Qing Hua Guo, XiaoYong Chen.

**Resources:** Zhong Jun Ding, XiaoYong Chen.

**Supervision:** Zhong Jun Ding.

**Validation:** Zhong Jun Ding.

**Investigation:** Dong Mei Liu.

**Methodology:** Dong Mei Liu.

**Software:** Dong Mei Liu.

**Data curation:** Xiao Shan Ren.

**Visualization:** Xiao Shan Ren.

**Project administration:** XiaoYong Chen.

**Writing – original draft:** Qing Hua Guo.

**Writing – review & editing:** Qing Hua Guo.

## Supplementary Material
