# Supplementary material for: Identification of biomarkers related to amino acid metabolism in nonobstructive azoospermia by bioinformatics
Source: Medicine (Baltimore). 2026 May 1;105(18):e48183. doi: 10.1097/MD.0000000000048183 (PMC13138492; doi:10.1097/MD.0000000000048183)
Supplement: Supplementary file 1 [file medi-105-e48183-s001.pdf]

TableS1: Specific primer sequences

primer	sequence
AKT1 F	GGAGGTTTTTGGGCTTGCG
AKT1 R	CTCTGATGCACCAGCTGACA
ASNS F	GACCTGCTTACGCCCAGATT
ASNS R	ATCATCACTGCCAAACAGCG
MYC F	ACAACCGAAAATGCACCAGC
MYC R	GTCGTTTCCGCAACAAGTCC
SHC1 F	ATCATCGCCAACCACCACAT
SHC1 R	TCCTCCTCATCCCATGCTGA
GAPDH F	ATGGGCAGCCGTTAGGAAAG
GAPDH R	AGGAAAAGCATCACCCGGAG

Table S2: The results of GO functions significantly enriched with candidate genes

ONTOLOGY	ID	Description	GeneRatio	BgRatio	pvalue	p.adjust	qvalue	geneID	Count	richFactor
BP	GO:0018209	peptidyl-serine modification	19/115	332/18903	1.40E-13	4.29E-10	3.18E-10	TSSK2/PRKAA1/DYRK4/MTCP1/MAPKAPK2/CSNK2A2/GADD45A/MAST2/HIPK2/PRKCH/MAD2L2/MKNK1/CLK1/AKT1/ROCK2/TCL1A/GALNT1/UCN/SMG1	19	0.572289157
BP	GO:0018105	peptidyl-serine phosphorylation	18/115	313/18903	5.99E-13	7.22E-10	5.35E-10	TSSK2/PRKAA1/DYRK4/MTCP1/MAPKAPK2/CSNK2A2/GADD45A/MAST2/HIPK2/PRKCH/MAD2L2/MKNK1/CLK1/AKT1/ROCK2/TCL1A/UCN/SMG1	18	0.575079872
BP	GO:0018205	peptidyl-lysine modification	20/115	414/18903	7.06E-13	7.22E-10	5.35E-10	PRKAA1/RNF113A/ZMPSTE24/CTBP1/PLOD1/DR1/SENP1/SUV39H2/DOT1L/SAE1/SUPT3H/BLOC1S1/BRD1/GCSH/ZBED1/PIWIL2/RUVBL1/NNMT/MAGEA2B/EGR1	20	0.483091787
BP	GO:1901605	alpha-amino acid metabolic process	15/115	205/18903	1.87E-12	1.43E-09	1.06E-09	GLUL/ASNS/SLC7A7/HMGCL/SLC25A12/BCAT2/CLN3/GCSH/GCLC/GART/ADI1/MTHFD1/OAT/SDSL/PSAT1	15	0.731707317
BP	GO:1901607	alpha-amino acid biosynthetic process	10/115	71/18903	1.58E-11	9.68E-09	7.17E-09	GLUL/ASNS/SLC25A12/BCAT2/CLN3/ADI1/MTHFD1/OAT/SDSL/PSAT1	10	1.408450704
BP	GO:0006520	cellular amino acid metabolic process	16/115	289/18903	2.27E-11	1.16E-08	8.59E-09	GLUL/ASNS/SLC7A7/HMGCL/SLC25A12/BCAT2/CLN3/GCSH/GCLC/GART/ADI1/MTHFD1/OAT/HNMT/SDSL/PSAT1	16	0.553633218
BP	GO:0008652	cellular amino acid biosynthetic process	10/115	79/18903	4.73E-11	2.07E-08	1.53E-08	GLUL/ASNS/SLC25A12/BCAT2/CLN3/ADI1/MTHFD1/OAT/SDSL/PSAT1	10	1.265822785
BP	GO:0071496	cellular response to external stimulus	16/115	328/18903	1.49E-10	5.71E-08	4.23E-08	SH3GLB1/PRKAA1/GLUL/ASNS/GADD45A/EIF2AK2/EIF2AK4/CTSK/NFE2L2/RRAGD/MAPK3/GCLC/MAP3K5/FOLR2/ATP1A2/LYN	16	0.487804878
BP	GO:0006575	cellular modified amino acid metabolic process	12/115	180/18903	1.01E-09	3.45E-07	2.55E-07	CPT1B/OSBPL5/CTSK/NFE2L2/PLSCR1/GCLC/GART/GSTO1/MTHFD1/OSBPL8/MGST2/GAMT	12	0.666666667
BP	GO:0031668	cellular response to extracellular stimulus	13/115	262/18903	7.20E-09	2.21E-06	1.64E-06	SH3GLB1/PRKAA1/GLUL/ASNS/EIF2AK2/EIF2AK4/CTSK/NFE2L2/RRAGD/MAPK3/MAP3K5/FOLR2/LYN	13	0.496183206
BP	GO:0009267	cellular response to starvation	11/115	175/18903	9.66E-09	2.47E-06	1.83E-06	SH3GLB1/PRKAA1/GLUL/ASNS/EIF2AK2/EIF2AK4/CTSK/NFE2L2/RRAGD/MAPK3/MAP3K5	11	0.628571429
BP	GO:0018108	peptidyl-tyrosine phosphorylation	15/115	380/18903	1.04E-08	2.47E-06	1.83E-06	NF2/SHC1/DYRK4/EIF2AK2/FGFR1/TYK2/PDGFRB/TESK2/MAPK3/HIPK2/IGF2/PTPN2/IBTK/LYN/CLK1	15	0.394736842
BP	GO:0018212	peptidyl-tyrosine modification	15/115	382/18903	1.11E-08	2.47E-06	1.83E-06	NF2/SHC1/DYRK4/EIF2AK2/FGFR1/TYK2/PDGFRB/TESK2/MAPK3/HIPK2/IGF2/PTPN2/IBTK/LYN/CLK1	15	0.392670157
BP	GO:0042398	cellular modified amino acid biosynthetic process	7/115	46/18903	1.13E-08	2.47E-06	1.83E-06	NFE2L2/PLSCR1/GCLC/GART/MTHFD1/MGST2/GAMT	7	1.52173913
BP	GO:0046777	protein autophosphorylation	12/115	226/18903	1.33E-08	2.72E-06	2.01E-06	TSSK2/MAPKAPK2/EIF2AK2/EIF2AK4/FGFR1/PDGFRB/MAPK3/LYN/MKNK1/CLK1/AKT1/SMG1	12	0.530973451
BP	GO:0031669	cellular response to nutrient levels	12/115	231/18903	1.70E-08	3.25E-06	2.41E-06	SH3GLB1/PRKAA1/GLUL/ASNS/EIF2AK2/EIF2AK4/CTSK/NFE2L2/RRAGD/MAPK3/MAP3K5/FOLR2	12	0.519480519

BP	GO:0031667	response to nutrient levels	16/115	474/18903	2.91E-08	5.25E-06	3.89E-06	SH3GLB1/PRKAA1/GLUL/ASNS/EIF2AK2/EIF2AK4/CTSK/NFE2L2/RRAGD/MAPK3/GCLC/MAP3K5/FOLR2/NQO1/AKT1/UCN	16	0.337552743
BP	GO:0042594	response to starvation	11/115	211/18903	6.66E-08	1.13E-05	8.41E-06	SH3GLB1/PRKAA1/GLUL/ASNS/EIF2AK2/EIF2AK4/CTSK/NFE2L2/RRAGD/MAPK3/MAP3K5	11	0.521327014
BP	GO:0046394	carboxylic acid biosynthetic process	13/115	326/18903	9.48E-08	1.53E-05	1.13E-05	PRKAA1/GLUL/ASNS/SLC25A12/BCAT2/CLN3/ADI1/MTHFD1/OAT/SDSL/MGST2/PSAT1/GAMT	13	0.398773006
BP	GO:0016053	organic acid biosynthetic process	13/115	328/18903	1.02E-07	1.56E-05	1.16E-05	PRKAA1/GLUL/ASNS/SLC25A12/BCAT2/CLN3/ADI1/MTHFD1/OAT/SDSL/MGST2/PSAT1/GAMT	13	0.396341463
BP	GO:0009069	serine family amino acid metabolic process	6/115	41/18903	1.68E-07	2.45E-05	1.82E-05	GCSH/GCLC/GART/MTHFD1/SDSL/PSAT1	6	1.463414634
BP	GO:0031098	stress-activated protein kinase signaling cascade	11/115	246/18903	3.14E-07	4.37E-05	3.24E-05	MAPKAPK2/ZMPSTE24/GADD45A/EIF2AK2/HMGB1/MYC/MAPK3/HIPK2/MAP3K5/LYN/PRDX1	11	0.447154472
BP	GO:0070302	regulation of stress-activated protein kinase signaling cascade	10/115	198/18903	3.64E-07	4.85E-05	3.59E-05	ZMPSTE24/GADD45A/EIF2AK2/HMGB1/MYC/MAPK3/HIPK2/MAP3K5/LYN/PRDX1	10	0.505050505
BP	GO:1905039	carboxylic acid transmembrane transport	9/115	155/18903	4.45E-07	5.60E-05	4.15E-05	CPT1B/SLC7A7/SLC25A12/CLN3/ITGB1/FOLR2/SLC1A4/ATP1A2/AKT1	9	0.580645161
BP	GO:1903825	organic acid transmembrane transport	9/115	156/18903	4.70E-07	5.60E-05	4.15E-05	CPT1B/SLC7A7/SLC25A12/CLN3/ITGB1/FOLR2/SLC1A4/ATP1A2/AKT1	9	0.576923077
BP	GO:0009064	glutamine family amino acid metabolic process	7/115	78/18903	4.75E-07	5.60E-05	4.15E-05	GLUL/ASNS/SLC7A7/SLC25A12/CLN3/GCLC/OAT	7	0.897435897
BP	GO:0034198	cellular response to amino acid starvation	6/115	49/18903	5.02E-07	5.70E-05	4.23E-05	SH3GLB1/EIF2AK2/EIF2AK4/RRAGD/MAPK3/MAP3K5	6	1.224489796
BP	GO:1990928	response to amino acid starvation	6/115	51/18903	6.41E-07	7.01E-05	5.20E-05	SH3GLB1/EIF2AK2/EIF2AK4/RRAGD/MAPK3/MAP3K5	6	1.176470588
BP	GO:0018210	peptidyl-threonine modification	8/115	120/18903	7.00E-07	7.40E-05	5.48E-05	DYRK4/GALNT11/CSNK2A2/HIPK2/CLK1/AKT1/ROCK2/GALNT1	8	0.666666667
BP	GO:0043543	protein acylation	11/115	272/18903	8.49E-07	8.67E-05	6.42E-05	PRKAA1/GLUL/ZMPSTE24/CTBP1/DR1/SUPT3H/BLOC1S1/BRD1/PIWIL2/RUVBL1/MAGEA2B	11	0.404411765
BP	GO:0016570	histone modification	14/115	474/18903	1.13E-06	0.000111891	8.29E-05	ZMPSTE24/CTBP1/DR1/SUV39H2/DOT1L/AURKC/SUPT3H/PRMT2/IGF2/BRD1/PIWIL2/RUVBL1/NNMT/UCN	14	0.29535865
BP	GO:0006473	protein acetylation	10/115	230/18903	1.43E-06	0.000136938	0.000101456	PRKAA1/ZMPSTE24/CTBP1/DR1/SUPT3H/BLOC1S1/BRD1/PIWIL2/RUVBL1/MAGEA2B	10	0.434782609
BP	GO:0043434	response to peptide hormone	13/115	421/18903	1.72E-06	0.000159885	0.000118458	SHC1/PRKAA1/CTSK/TYK2/NFE2L2/IGF2/GCLC/PTPN2/LYN/OSBPL8/EGR1/AKT1/ROCK2	13	0.308788599
BP	GO:0071375	cellular response to peptide hormone stimulus	11/115	306/18903	2.67E-06	0.000240736	0.00017836	SHC1/PRKAA1/TYK2/NFE2L2/IGF2/GCLC/PTPN2/LYN/OSBPL8/AKT1/ROCK2	11	0.359477124
BP	GO:0009084	glutamine family amino	4/115	17/18903	2.91E-06	0.000254935	0.00018888	GLUL/SLC25A12/CLN3/OAT	4	2.352941176

		acid biosynthetic process								
BP	GO:0018394	peptidyl-lysine acetylation	9/115	197/18903	3.28E-06	0.000279264	0.000206905	PRKAA1/ZMPSTE24/CTBP1/DR1/SUPT3H/BLOC1S1/BRD1/PIWIL2/RUVBL1	9	0.456852792
BP	GO:0034614	cellular response to reactive oxygen species	8/115	150/18903	3.77E-06	0.000312026	0.000231178	PRKAA1/NFE2L2/MAPK3/MAP3K5/PRDX1/PRDX3/NQO1/AKT1	8	0.533333333
BP	GO:0000302	response to reactive oxygen species	9/115	204/18903	4.36E-06	0.000351923	0.000260737	PRKAA1/NFE2L2/PDGFRB/MAPK3/MAP3K5/PRDX1/PRDX3/NQO1/AKT1	9	0.441176471
BP	GO:0045454	cell redox homeostasis	5/115	41/18903	4.80E-06	0.00037768	0.000279821	NFE2L2/GCLC/PRDX1/PRDX3/NQO1	5	1.219512195
BP	GO:0010907	positive regulation of glucose metabolic process	5/115	42/18903	5.43E-06	0.000415299	0.000307692	IGF2/SLC25A12/PTPN2/NNMT/AKT1	5	1.19047619
BP	GO:0032868	response to insulin	10/115	269/18903	5.78E-06	0.000415299	0.000307692	SHC1/PRKAA1/CTSK/IGF2/GCLC/PTPN2/LYN/OSBPL8/EGR1/AKT1	10	0.371747212
BP	GO:0036294	cellular response to decreased oxygen levels	8/115	159/18903	5.80E-06	0.000415299	0.000307692	PRKAA1/SUV39H2/NFE2L2/MYC/HIPK2/EGR1/AKT1/ROCK2	8	0.503144654
BP	GO:0009063	cellular amino acid catabolic process	7/115	113/18903	5.82E-06	0.000415299	0.000307692	GLUL/HMGCL/BCAT2/GCSH/OAT/HNMT/SDSL	7	0.619469027
BP	GO:0015807	L-amino acid transport	6/115	76/18903	6.87E-06	0.000478871	0.000354792	SLC7A7/SLC25A12/CLN3/ITGB1/SLC1A4/ATP1A2	6	0.789473684
BP	GO:0009308	amine metabolic process	7/115	117/18903	7.33E-06	0.00049953	0.000370099	PAOX/SLC7A7/SLC44A1/CLN3/NNMT/HNMT/AOC3	7	0.598290598
BP	GO:0045913	positive regulation of carbohydrate metabolic process	6/115	78/18903	7.99E-06	0.000520104	0.000385342	PRKAA1/IGF2/SLC25A12/PTPN2/NNMT/AKT1	6	0.769230769
BP	GO:1902475	L-alpha-amino acid transmembrane transport	6/115	78/18903	7.99E-06	0.000520104	0.000385342	SLC7A7/SLC25A12/CLN3/ITGB1/SLC1A4/ATP1A2	6	0.769230769
BP	GO:0010506	regulation of autophagy	11/115	344/18903	8.14E-06	0.000520104	0.000385342	ATP6V1E2/SH3GLB1/PRKAA1/ZMPSTE24/CSNK2A2/HMGB1/RRAGD/MAPK3/CLN3/AKT1/SMG1	11	0.319767442
BP	GO:0009066	aspartate family amino acid metabolic process	5/115	48/18903	1.06E-05	0.00066395	0.000491916	ASNS/SLC25A12/ADI1/MTHFD1/SDSL	5	1.041666667
BP	GO:0071453	cellular response to oxygen levels	8/115	175/18903	1.17E-05	0.000718347	0.000532218	PRKAA1/SUV39H2/NFE2L2/MYC/HIPK2/EGR1/AKT1/ROCK2	8	0.457142857
BP	GO:0006979	response to oxidative stress	12/115	434/18903	1.34E-05	0.000808495	0.000599009	PRKAA1/NFE2L2/PDGFRB/MAPK3/GCLC/MAP3K5/PRDX1/PRDX3/NQO1/RRM2B/AKT1/UCN	12	0.276497696
BP	GO:0051403	stress-activated MAPK cascade	9/115	239/18903	1.56E-05	0.000920142	0.000681727	MAPKAPK2/GADD45A/EIF2AK2/HMGB1/MYC/MAPK3/HIPK2/MAP3K5/PRDX1	9	0.376569038
BP	GO:1901653	cellular response to peptide	11/115	373/18903	1.73E-05	0.001003064	0.000743163	SHC1/PRKAA1/TYK2/NFE2L2/IGF2/GCLC/PTPN2/LYN/OSBPL8/AKT1/ROCK2	11	0.294906166
BP	GO:0046395	carboxylic acid catabolic process	9/115	243/18903	1.78E-05	0.001010806	0.0007489	GLUL/CPT1B/HMGCL/BCAT2/GCSH/OAT/HNMT/SDSL/AKT1	9	0.37037037
BP	GO:0006109	regulation of carbohydrate metabolic	8/115	186/18903	1.82E-05	0.001016129	0.000752843	PRKAA1/ZMPSTE24/SCARB2/IGF2/SLC25A12/PTPN2/NNMT/AKT1	8	0.430107527

		process								
BP	GO:0016054	organic acid catabolic process	9/115	247/18903	2.03E-05	0.001109129	0.000821746	GLUL/CPT1B/HMGCL/BCAT2/GCSH/OAT/HNMT/SDSL/AKT1	9	0.36437247
BP	GO:0005996	monosaccharide metabolic process	9/115	248/18903	2.09E-05	0.001125008	0.000833511	PRKAA1/ZMPSTE24/IGF2/SLC25A12/GCLC/PTPN2/GSTO1/NNMT/AKT1	9	0.362903226
BP	GO:0010676	positive regulation of cellular carbohydrate metabolic process	5/115	56/18903	2.28E-05	0.001203783	0.000891875	IGF2/SLC25A12/PTPN2/NNMT/AKT1	5	0.892857143
BP	GO:1901606	alpha-amino acid catabolic process	6/115	95/18903	2.49E-05	0.001287086	0.000953594	GLUL/HMGCL/BCAT2/GCSH/OAT/SDSL	6	0.631578947
BP	GO:0072203	cell proliferation involved in metanephros development	3/115	10/18903	2.55E-05	0.001287086	0.000953594	PDGFRB/MYC/EGR1	3	3
BP	GO:0032872	regulation of stress-activated MAPK cascade	8/115	195/18903	2.56E-05	0.001287086	0.000953594	GADD45A/EIF2AK2/HMGB1/MYC/MAPK3/HIPK2/MAP3K5/PRDX1	8	0.41025641
BP	GO:0033135	regulation of peptidyl-serine phosphorylation	7/115	143/18903	2.71E-05	0.001315067	0.000974324	PRKAA1/MTCP1/GADD45A/MAD2L2/AKT1/TCL1A/UCN	7	0.48951049
BP	GO:0015813	L-glutamate transmembrane transport	4/115	29/18903	2.75E-05	0.001315067	0.000974324	SLC25A12/ITGB1/SLC1A4/ATP1A2	4	1.379310345
BP	GO:0051938	L-glutamate import	4/115	29/18903	2.75E-05	0.001315067	0.000974324	SLC25A12/ITGB1/SLC1A4/ATP1A2	4	1.379310345
BP	GO:0015849	organic acid transport	10/115	324/18903	2.90E-05	0.001365713	0.001011848	PRKAA1/CPT1B/SLC7A7/SLC25A12/CLN3/ITGB1/FOLR2/SLC1A4/ATP1A2/AKT1	10	0.308641975
BP	GO:0098869	cellular oxidant detoxification	6/115	100/18903	3.33E-05	0.001546452	0.001145756	NFE2L2/GSTO1/PRDX1/PRDX3/NQO1/MGST2	6	0.6
BP	GO:0090311	regulation of protein deacetylation	5/115	61/18903	3.46E-05	0.001584201	0.001173724	PRKAA1/CTBP1/DR1/NNMT/UCN	5	0.819672131
BP	GO:0009895	negative regulation of catabolic process	10/115	332/18903	3.56E-05	0.00160539	0.001189423	PRKAA1/MAPKAPK2/CSNK2A2/SECISBP2/HIPK2/SLC25A12/MAD2L2/NQO1/AKT1/SMG1	10	0.301204819
BP	GO:0071456	cellular response to hypoxia	7/115	151/18903	3.85E-05	0.001709791	0.001266773	PRKAA1/SUV39H2/NFE2L2/MYC/HIPK2/EGR1/ROCK2	7	0.463576159
BP	GO:0070482	response to oxygen levels	10/115	337/18903	4.04E-05	0.001769249	0.001310825	PRKAA1/PLOD1/SUV39H2/NFE2L2/PDGFRB/MYC/HIPK2/EGR1/AKT1/ROCK2	10	0.296735905
BP	GO:1903955	positive regulation of protein targeting to mitochondrion	4/115	32/18903	4.10E-05	0.001769249	0.001310825	SH3GLB1/PRKAA1/CSNK2A2/SAE1	4	1.25
BP	GO:0003333	amino acid transmembrane transport	6/115	104/18903	4.15E-05	0.001769249	0.001310825	SLC7A7/SLC25A12/CLN3/ITGB1/SLC1A4/ATP1A2	6	0.576923077
BP	GO:0062012	regulation of small	10/115	339/18903	4.25E-05	0.001783306	0.001321239	PRKAA1/SLC7A7/ZMPSTE24/IGF2/SLC25A12/CLN3/PTPN	10	0.294985251

		molecule metabolic process						2/NNMT/EGR1/AKT1		
BP	GO:0010675	regulation of cellular carbohydrate metabolic process	7/115	155/18903	4.55E-05	0.001883598	0.001395545	ZMPSTE24/SCARB2/IGF2/SLC25A12/PTPN2/NNMT/AKT1	7	0.451612903
BP	GO:0010906	regulation of glucose metabolic process	6/115	107/18903	4.88E-05	0.001993268	0.001476799	ZMPSTE24/IGF2/SLC25A12/PTPN2/NNMT/AKT1	6	0.560747664
BP	GO:0006536	glutamate metabolic process	4/115	34/18903	5.24E-05	0.002084801	0.001544615	GLUL/SLC25A12/GCLC/OAT	4	1.176470588
BP	GO:0033238	regulation of cellular amine metabolic process	4/115	34/18903	5.24E-05	0.002084801	0.001544615	PAOX/SLC7A7/CLN3/NNMT	4	1.176470588
BP	GO:0043542	endothelial cell migration	9/115	281/18903	5.55E-05	0.002179865	0.001615047	GLUL/GADD45A/FGFR1/HMGB1/NFE2L2/CLN3/ITGB1/AKT1/ROCK2	9	0.320284698
BP	GO:0018107	peptidyl-threonine phosphorylation	6/115	111/18903	5.99E-05	0.002324618	0.001722294	DYRK4/CSNK2A2/HIPK2/CLK1/AKT1/ROCK2	6	0.540540541
BP	GO:1903749	positive regulation of establishment of protein localization to mitochondrion	4/115	36/18903	6.59E-05	0.002524951	0.001870719	SH3GLB1/PRKAA1/CSNK2A2/SAE1	4	1.111111111
BP	GO:0051881	regulation of mitochondrial membrane potential	5/115	70/18903	6.74E-05	0.002552387	0.001891047	GCLC/CLIC1/PRDX3/AKT1/TCL1A	5	0.714285714
BP	GO:1990748	cellular detoxification	6/115	115/18903	7.30E-05	0.002728806	0.002021754	NFE2L2/GSTO1/PRDX1/PRDX3/NQO1/MGST2	6	0.52173913
BP	GO:0002262	myeloid cell homeostasis	7/115	169/18903	7.86E-05	0.002902283	0.002150282	HMGB1/HIPK2/BRD1/PTPN2/MTHFD1/LYN/PRDX1	7	0.414201183
BP	GO:0097193	intrinsic apoptotic signaling pathway	9/115	298/18903	8.70E-05	0.003175484	0.002352695	NFE2L2/MYC/HIPK2/CEBPB/PTPN2/MAP3K5/RPS27L/RRM2B/AKT1	9	0.302013423
BP	GO:0072331	signal transduction by p53 class mediator	7/115	173/18903	9.10E-05	0.003281877	0.002431521	ZMPSTE24/MYC/HIPK2/RPS27L/RRM2B/MAGEA2B/AKT1	7	0.404624277
BP	GO:0045862	positive regulation of proteolysis	10/115	372/18903	9.21E-05	0.003281877	0.002431521	SENP1/HMGB1/NFE2L2/MYC/GCLC/MAP3K5/RPS27L/LYN/AKT1/ROCK2	10	0.268817204
BP	GO:0010950	positive regulation of endopeptidase activity	7/115	174/18903	9.43E-05	0.003324393	0.002463021	SENP1/HMGB1/MYC/MAP3K5/RPS27L/LYN/ROCK2	7	0.402298851
BP	GO:0031056	regulation of histone modification	7/115	175/18903	9.78E-05	0.003406552	0.002523892	ZMPSTE24/CTBP1/DR1/IGF2/PIWIL2/NNMT/UCN	7	0.4
BP	GO:0140467	integrated stress response signaling	4/115	40/18903	0.000100318	0.00345073	0.002556623	EIF2AK4/NFE2L2/CEBPB/PTPN2	4	1
BP	GO:0008286	insulin receptor signaling pathway	6/115	122/18903	0.000101293	0.00345073	0.002556623	SHC1/PRKAA1/IGF2/PTPN2/OSBPL8/AKT1	6	0.491803279
BP	GO:0043534	blood vessel endothelial cell migration	7/115	177/18903	0.000104967	0.003531413	0.0026164	GADD45A/FGFR1/HMGB1/NFE2L2/CLN3/ITGB1/AKT1	7	0.395480226
BP	GO:0097237	cellular response to	6/115	123/18903	0.000105965	0.003531413	0.0026164	NFE2L2/GSTO1/PRDX1/PRDX3/NQO1/MGST2	6	0.487804878

		toxic substance								
BP	GO:0036293	response to decreased oxygen levels	9/115	309/18903	0.00011455	0.003722543	0.002758007	PRKAA1/PLOD1/SUV39H2/NFE2L2/MYC/HIPK2/EGR1/AKT1/ROCK2	9	0.291262136
BP	GO:0051283	negative regulation of sequestering of calcium ion	6/115	125/18903	0.000115823	0.003722543	0.002758007	FKBP1A/IBTK/ANXA6/GSTO1/ATP1A2/LYN	6	0.48
BP	GO:0006750	glutathione biosynthetic process	3/115	16/18903	0.000115941	0.003722543	0.002758007	NFE2L2/GCLC/MGST2	3	1.875
BP	GO:0016573	histone acetylation	7/115	180/18903	0.000116557	0.003722543	0.002758007	ZMPSTE24/CTBP1/DR1/SUPT3H/BRD1/PIWIL2/RUVBL1	7	0.388888889
BP	GO:0042770	signal transduction in response to DNA damage	7/115	181/18903	0.000120644	0.003813348	0.002825284	ZMPSTE24/GADD45A/EIF2AK4/DOT1L/HIPK2/RPS27L/MAD2L2	7	0.386740331
BP	GO:0044282	small molecule catabolic process	10/115	385/18903	0.000122025	0.003817649	0.002828471	GLUL/CPT1B/HMGCL/SLC25A12/BCAT2/GCSH/OAT/HNMT/SDSL/AKT1	10	0.25974026
BP	GO:0051282	regulation of sequestering of calcium ion	6/115	127/18903	0.000126395	0.003914419	0.002900167	FKBP1A/IBTK/ANXA6/GSTO1/ATP1A2/LYN	6	0.472440945
BP	GO:0018393	internal peptidyl-lysine acetylation	7/115	186/18903	0.000142865	0.004359522	0.003229941	ZMPSTE24/CTBP1/DR1/SUPT3H/BRD1/PIWIL2/RUVBL1	7	0.376344086
BP	GO:0042542	response to hydrogen peroxide	6/115	130/18903	0.000143675	0.004359522	0.003229941	PRKAA1/NFE2L2/PDGFRB/MAP3K5/PRDX3/NQO1	6	0.461538462
BP	GO:1903214	regulation of protein targeting to mitochondrion	4/115	44/18903	0.000146248	0.004359522	0.003229941	SH3GLB1/PRKAA1/CSNK2A2/SAE1	4	0.909090909
BP	GO:0009636	response to toxic substance	8/115	250/18903	0.000146455	0.004359522	0.003229941	NFE2L2/PDGFRB/GSTO1/LYN/PRDX1/PRDX3/NQO1/MGST2	8	0.32
BP	GO:0051208	sequestering of calcium ion	6/115	131/18903	0.000149833	0.004414862	0.003270942	FKBP1A/IBTK/ANXA6/GSTO1/ATP1A2/LYN	6	0.458015267
BP	GO:0006006	glucose metabolic process	7/115	188/18903	0.000152634	0.004414862	0.003270942	PRKAA1/ZMPSTE24/IGF2/SLC25A12/PTPN2/NNMT/AKT1	7	0.372340426
BP	GO:0006475	internal protein amino acid acetylation	7/115	188/18903	0.000152634	0.004414862	0.003270942	ZMPSTE24/CTBP1/DR1/SUPT3H/BRD1/PIWIL2/RUVBL1	7	0.372340426
BP	GO:0070304	positive regulation of stress-activated protein kinase signaling cascade	6/115	132/18903	0.000156198	0.004475721	0.003316032	GADD45A/EIF2AK2/HMGB1/HIPK2/MAP3K5/LYN	6	0.454545455
BP	GO:1901214	regulation of neuron death	9/115	325/18903	0.000167419	0.004667263	0.003457944	HIPK2/CEBPB/CLN3/GCLC/MAP3K5/NQO1/EGR1/AKT1/UCN	9	0.276923077
BP	GO:0019184	nonribosomal peptide biosynthetic process	3/115	18/18903	0.000167449	0.004667263	0.003457944	NFE2L2/GCLC/MGST2	3	1.666666667
BP	GO:0042402	cellular biogenic amine catabolic process	3/115	18/18903	0.000167449	0.004667263	0.003457944	PAOX/SLC44A1/HNMT	3	1.666666667
BP	GO:0010952	positive regulation of	7/115	194/18903	0.000185237	0.005116546	0.003790815	SENP1/HMGB1/MYC/MAP3K5/RPS27L/LYN/ROCK2	7	0.360824742

		peptidase activity								
BP	GO:0090068	positive regulation of cell cycle process	8/115	260/18903	0.000191467	0.005241404	0.003883321	AURKC/PDGFRB/IGF2/PLSCR1/PIWIL2/RRM2B/AKT1/ROCK2	8	0.307692308
BP	GO:0042772	DNA damage response	3/115	19/18903	0.000197965	0.005371346	0.003979595	HIPK2/RPS27L/MAD2L2	3	1.578947368
BP	GO:1901983	regulation of protein acetylation	5/115	88/18903	0.000200103	0.005381706	0.00398727	PRKAA1/ZMPSTE24/CTBP1/PIWIL2/MAGEA2B	5	0.568181818
BP	GO:0071214	cellular response to abiotic stimulus	9/115	335/18903	0.000209806	0.005545402	0.004108551	ZMPSTE24/GADD45A/EIF2AK4/MYC/MAPK3/CLN3/GCLC/ATP1A2/EGR1	9	0.268656716
BP	GO:0104004	cellular response to environmental stimulus	9/115	335/18903	0.000209806	0.005545402	0.004108551	ZMPSTE24/GADD45A/EIF2AK4/MYC/MAPK3/CLN3/GCLC/ATP1A2/EGR1	9	0.268656716
BP	GO:0033674	positive regulation of kinase activity	11/115	496/18903	0.000221677	0.005759836	0.004267424	MTCP1/FGFR1/PDGFRB/IGF2/MAP3K5/LYN/OSBPL8/EGR1/AKT1/TCL1A/UCN	11	0.221774194
BP	GO:0043410	positive regulation of MAPK cascade	11/115	496/18903	0.000221677	0.005759836	0.004267424	SHC1/GADD45A/EIF2AK2/FGFR1/HMGB1/PDGFRB/MAPK3/HIPK2/IGF2/MAP3K5/ROCK2	11	0.221774194
BP	GO:0006525	arginine metabolic process	3/115	20/18903	0.00023187	0.005915622	0.004382845	SLC7A7/CLN3/OAT	3	1.5
BP	GO:0009310	amine catabolic process	3/115	20/18903	0.00023187	0.005915622	0.004382845	PAOX/SLC44A1/HNMT	3	1.5
BP	GO:0030003	cellular cation homeostasis	11/115	499/18903	0.000233461	0.005915622	0.004382845	ATP6V1F/MYC/MAPK3/FKBP1A/CLN3/IBTK/ANXA6/GSTO1/ATP1A2/LYN/SLC24A3	11	0.220440882
BP	GO:1903747	regulation of establishment of protein localization to mitochondrion	4/115	50/18903	0.000241241	0.006062655	0.004491781	SH3GLB1/PRKAA1/CSNK2A2/SAE1	4	0.8
BP	GO:0051235	maintenance of location	9/115	342/18903	0.000244509	0.006093356	0.004514526	FKBP1A/PTPN2/IBTK/ANXA6/GSTO1/ATP1A2/LYN/OSBPL8/AKT1	9	0.263157895
BP	GO:0045860	positive regulation of protein kinase activity	10/115	420/18903	0.000246437	0.006093356	0.004514526	MTCP1/FGFR1/PDGFRB/IGF2/MAP3K5/OSBPL8/EGR1/AKT1/TCL1A/UCN	10	0.238095238
BP	GO:0006835	dicarboxylic acid transport	5/115	93/18903	0.000259138	0.006295578	0.004664352	SLC25A12/ITGB1/FOLR2/SLC1A4/ATP1A2	5	0.537634409
BP	GO:0043281	regulation of cysteine-type endopeptidase activity involved in apoptotic process	7/115	205/18903	0.000259544	0.006295578	0.004664352	SENP1/HMGB1/MYC/MAP3K5/RPS27L/PRDX3/AKT1	7	0.341463415
BP	GO:0062197	cellular response to chemical stress	9/115	345/18903	0.000260776	0.006295578	0.004664352	PRKAA1/NFE2L2/MAPK3/CLN3/MAP3K5/PRDX1/PRDX3/NQO1/AKT1	9	0.260869565
BP	GO:0009067	aspartate family amino acid biosynthetic process	3/115	21/18903	0.000269318	0.006450999	0.004779502	ASNS/ADI1/MTHFD1	3	1.428571429
BP	GO:0006865	amino acid transport	6/115	147/18903	0.000280092	0.006657078	0.004932185	SLC7A7/SLC25A12/CLN3/ITGB1/SLC1A4/ATP1A2	6	0.408163265
BP	GO:0032869	cellular response to insulin stimulus	7/115	208/18903	0.000283502	0.006660386	0.004934636	SHC1/PRKAA1/IGF2/GCLC/PTPN2/OSBPL8/AKT1	7	0.336538462

BP	GO:0043648	dicarboxylic acid metabolic process	5/115	95/18903	0.000286131	0.006660386	0.004934636	GLUL/SLC25A12/GCLC/MTHFD1/OAT	5	0.526315789
BP	GO:0051348	negative regulation of transferase activity	8/115	276/18903	0.000286749	0.006660386	0.004934636	NF2/GADD45A/PTPN2/IBTK/LYN/MAD2L2/PRDX3/AKT1	8	0.289855072
BP	GO:0044154	histone H3-K14 acetylation	3/115	22/18903	0.000310462	0.007103555	0.005262977	DR1/BRD1/PIWIL2	3	1.363636364
BP	GO:0072111	cell proliferation involved in kidney development	3/115	22/18903	0.000310462	0.007103555	0.005262977	PDGFRB/MYC/EGR1	3	1.363636364
BP	GO:0042149	cellular response to glucose starvation	4/115	54/18903	0.000325139	0.007384259	0.005470948	SH3GLB1/PRKAA1/ASNS/NFE2L2	4	0.740740741
BP	GO:0062013	positive regulation of small molecule metabolic process	6/115	152/18903	0.000335191	0.007556587	0.005598625	PRKAA1/IGF2/SLC25A12/PTPN2/NNMT/AKT1	6	0.394736842
BP	GO:0006874	cellular calcium ion homeostasis	8/115	284/18903	0.000347237	0.007753985	0.005744876	FKBP1A/CLN3/IBTK/ANXA6/GSTO1/ATP1A2/LYN/SLC24A3	8	0.281690141
BP	GO:0002931	response to ischemia	4/115	55/18903	0.000349005	0.007753985	0.005744876	MAP3K5/NQO1/EGR1/ROCK2	4	0.727272727
BP	GO:0019430	removal of superoxide radicals	3/115	23/18903	0.000355452	0.007805261	0.005782866	NFE2L2/PRDX1/NQO1	3	1.304347826
BP	GO:0006816	calcium ion transport	10/115	440/18903	0.000356405	0.007805261	0.005782866	ZMPSTE24/PDGFRB/FKBP1A/IBTK/ANXA6/GSTO1/ATP1A2/LYN/UCN/SLC24A3	10	0.227272727
BP	GO:0098754	detoxification	6/115	154/18903	0.00035947	0.007816555	0.005791233	NFE2L2/GSTO1/PRDX1/PRDX3/NQO1/MGST2	6	0.38961039
BP	GO:0045787	positive regulation of cell cycle	9/115	362/18903	0.000370872	0.008007693	0.005932846	ASNS/AURKC/PDGFRB/IGF2/PLSCR1/PIWIL2/RRM2B/AKT1/ROCK2	9	0.248618785
BP	GO:0034599	cellular response to oxidative stress	8/115	288/18903	0.000381171	0.008172513	0.00605496	PRKAA1/NFE2L2/MAPK3/MAP3K5/PRDX1/PRDX3/NQO1/AKT1	8	0.277777778
BP	GO:0035249	synaptic transmission	5/115	102/18903	0.000397688	0.008321711	0.0061655	GLUL/CLN3/SLC1A4/ATP1A2/UCN	5	0.490196078
BP	GO:0046942	carboxylic acid transport	8/115	290/18903	0.000399124	0.008321711	0.0061655	PRKAA1/SLC7A7/SLC25A12/CLN3/ITGB1/FOLR2/SLC1A4/ATP1A2	8	0.275862069
BP	GO:0006749	glutathione metabolic process	4/115	57/18903	0.000400459	0.008321711	0.0061655	NFE2L2/GCLC/GSTO1/MGST2	4	0.701754386
BP	GO:0010332	response to gamma radiation	4/115	57/18903	0.000400459	0.008321711	0.0061655	PRKAA1/ZMPSTE24/MYC/EGR1	4	0.701754386
BP	GO:0010631	epithelial cell migration	9/115	366/18903	0.0004017	0.008321711	0.0061655	GLUL/GADD45A/FGFR1/HMGB1/NFE2L2/CLN3/ITGB1/AKT1/ROCK2	9	0.245901639
BP	GO:0070997	neuron death	9/115	368/18903	0.00041789	0.008599002	0.006370943	HIPK2/CEBPB/CLN3/GCLC/MAP3K5/NQO1/EGR1/AKT1/UCN	9	0.244565217
BP	GO:0090132	epithelium migration	9/115	369/18903	0.000426185	0.008606502	0.0063765	GLUL/GADD45A/FGFR1/HMGB1/NFE2L2/CLN3/ITGB1/AKT1/ROCK2	9	0.243902439
BP	GO:1903829	positive regulation of protein localization	10/115	451/18903	0.000432631	0.008606502	0.0063765	SH3GLB1/PRKAA1/GLUL/CSNK2A2/SAE1/CLN3/ITGB1/PRKCH/AKT1/ROCK2	10	0.22172949
BP	GO:0046425	regulation of receptor	5/115	104/18903	0.000434881	0.008606502	0.0063765	NF2/GADD45A/DOT1L/TYK2/PTPN2	5	0.480769231

		signaling pathway via JAK-STAT								
BP	GO:0009314	response to radiation	10/115	452/18903	0.000440187	0.008606502	0.0063765	PRKAA1/CPT1B/ZMPSTE24/GADD45A/EIF2AK4/MYC/ITGB1/ATP1A2/EGR1/AKT1	10	0.221238938
BP	GO:0016241	regulation of macroautophagy	6/115	160/18903	0.000440653	0.008606502	0.0063765	ATP6V1E2/SH3GLB1/MAPK3/CLN3/AKT1/SMG1	6	0.375
BP	GO:1901796	regulation of signal transduction by p53 class mediator	5/115	105/18903	0.000454432	0.008606502	0.0063765	ZMPSTE24/MYC/HIPK2/RRM2B/AKT1	5	0.476190476
BP	GO:0051090	regulation of DNA-binding transcription factor activity	10/115	454/18903	0.000455629	0.008606502	0.0063765	PSMA6/EIF2AK2/EIF2AK4/PRMT2/MAPK3/HIPK2/PRKCH/MAD2L2/PRDX3/AKT1	10	0.220264317
BP	GO:1903078	positive regulation of protein localization to plasma membrane	4/115	59/18903	0.000457115	0.008606502	0.0063765	CLN3/ITGB1/PRKCH/AKT1	4	0.677966102
BP	GO:0001666	response to hypoxia	8/115	296/18903	0.000457161	0.008606502	0.0063765	PRKAA1/PLOD1/SUV39H2/NFE2L2/MYC/HIPK2/EGR1/ROCK2	8	0.27027027
BP	GO:0048872	homeostasis of number of cells	8/115	296/18903	0.000457161	0.008606502	0.0063765	HMGB1/HIPK2/BRD1/PTPN2/MTHFD1/LYN/PRDX1/AKT1	8	0.27027027
BP	GO:0006658	phosphatidylserine metabolic process	3/115	25/18903	0.000457554	0.008606502	0.0063765	OSBPL5/PLSCR1/OSBPL8	3	1.2
BP	GO:0033233	regulation of protein sumoylation	3/115	25/18903	0.000457554	0.008606502	0.0063765	SAE1/MAGEA2B/EGR1	3	1.2
BP	GO:0071450	cellular response to oxygen radical	3/115	25/18903	0.000457554	0.008606502	0.0063765	NFE2L2/PRDX1/NQO1	3	1.2
BP	GO:0071451	cellular response to superoxide	3/115	25/18903	0.000457554	0.008606502	0.0063765	NFE2L2/PRDX1/NQO1	3	1.2
BP	GO:0051651	maintenance of location in cell	7/115	226/18903	0.000467294	0.008728277	0.006466722	FKBP1A/IBTK/ANXA6/GSTO1/ATP1A2/LYN/AKT1	7	0.309734513
BP	GO:0090130	tissue migration	9/115	374/18903	0.000469721	0.008728277	0.006466722	GLUL/GADD45A/FGFR1/HMGB1/NFE2L2/CLN3/ITGB1/AKT1/ROCK2	9	0.240641711
BP	GO:0019318	hexose metabolic process	7/115	229/18903	0.000505532	0.009281198	0.006876378	PRKAA1/ZMPSTE24/IGF2/SLC25A12/PTPN2/NNMT/AKT1	7	0.305676856
BP	GO:0071902	positive regulation of protein serine/threonine kinase activity	7/115	229/18903	0.000505532	0.009281198	0.006876378	MTCP1/FGFR1/PDGFRB/IGF2/MAP3K5/AKT1/TCL1A	7	0.305676856
BP	GO:0051054	positive regulation of DNA metabolic process	8/115	301/18903	0.000510605	0.00930231	0.006892019	HMGB1/PDGFRB/MYC/MAPK3/MAD2L2/RUVBL1/AKT1/UCN	8	0.265780731
BP	GO:0033138	positive regulation of peptidyl-serine phosphorylation	5/115	108/18903	0.000517076	0.00930231	0.006892019	MTCP1/MAD2L2/AKT1/TCL1A/UCN	5	0.462962963

BP	GO:0044106	cellular amine metabolic process	5/115	108/18903	0.000517076	0.00930231	0.006892019	PAOX/SLC7A7/CLN3/NNMT/HNMT	5	0.462962963
BP	GO:0010594	regulation of endothelial cell migration	7/115	230/18903	0.000518818	0.00930231	0.006892019	GLUL/GADD45A/FGFR1/HMGB1/NFE2L2/AKT1/ROCK2	7	0.304347826
BP	GO:0007009	plasma membrane organization	6/115	166/18903	0.00053549	0.009545417	0.007072136	SH3GLB1/CLN3/PLSCR1/ANXA6/FOLR2/AKT1	6	0.361445783
BP	GO:0006767	water-soluble vitamin metabolic process	4/115	62/18903	0.000552493	0.009791573	0.007254511	GCLC/GSTO1/NNMT/PSAT1	4	0.64516129
BP	GO:0006766	vitamin metabolic process	5/115	110/18903	0.000562306	0.009908213	0.007340929	GCLC/GSTO1/NQO1/NNMT/PSAT1	5	0.454545455
BP	GO:0071356	cellular response to tumor necrosis factor	7/115	234/18903	0.000574773	0.009990625	0.007401987	CTSK/NFE2L2/MAPK3/PTPN2/MAP3K5/AKT1/TCL1A	7	0.299145299
BP	GO:0009081	branched-chain amino acid metabolic process	3/115	27/18903	0.000576758	0.009990625	0.007401987	HMGCL/BCAT2/SDSL	3	1.111111111
BP	GO:1905564	positive regulation of vascular endothelial cell proliferation	3/115	27/18903	0.000576758	0.009990625	0.007401987	FGFR1/HMGB1/IGF2	3	1.111111111
BP	GO:2000116	regulation of cysteine-type endopeptidase activity	7/115	235/18903	0.000589486	0.010153734	0.007522834	SENP1/HMGB1/MYC/MAP3K5/RPS27L/PRDX3/AKT1	7	0.29787234
BP	GO:0055074	calcium ion homeostasis	8/115	309/18903	0.000606579	0.010340069	0.007660888	FKBP1A/CLN3/IBTK/ANXA6/GSTO1/ATP1A2/LYN/SLC24A3	8	0.258899676
BP	GO:0034250	positive regulation of cellular amide metabolic process	6/115	170/18903	0.000607049	0.010340069	0.007660888	EIF2AK4/NFE2L2/PIWIL2/RPS27L/ROCK2/UCN	6	0.352941176
BP	GO:0016925	protein sumoylation	4/115	64/18903	0.000623435	0.010560506	0.007824208	SAE1/ZBED1/MAGEA2B/EGR1	4	0.625
BP	GO:0032259	methylation	9/115	390/18903	0.000634337	0.010686131	0.007917283	ZMPSTE24/SUV39H2/DOT1L/MYC/PRMT2/PIWIL2/NNMT/HNMT/GAMT	9	0.230769231
BP	GO:0000303	response to superoxide	3/115	28/18903	0.000643116	0.010756065	0.007969097	NFE2L2/PRDX1/NQO1	3	1.071428571
BP	GO:0007259	receptor signaling pathway via JAK-STAT	6/115	172/18903	0.000645504	0.010756065	0.007969097	NF2/GADD45A/DOT1L/TYK2/PTPN2/LYN	6	0.348837209
BP	GO:0015800	acidic amino acid transport	4/115	65/18903	0.000661224	0.010958455	0.008119046	SLC25A12/ITGB1/SLC1A4/ATP1A2	4	0.615384615
BP	GO:0033673	negative regulation of kinase activity	7/115	240/18903	0.00066761	0.011004796	0.00815338	NF2/GADD45A/PTPN2/IBTK/LYN/PRDX3/AKT1	7	0.291666667
BP	GO:1904892	regulation of receptor signaling pathway via STAT	5/115	115/18903	0.000688367	0.011286275	0.008361926	NF2/GADD45A/DOT1L/TYK2/PTPN2	5	0.434782609
BP	GO:0071900	regulation of protein serine/threonine kinase activity	9/115	395/18903	0.000694533	0.011326799	0.00839195	MTCP1/GADD45A/FGFR1/PDGFRB/IGF2/MAP3K5/LYN/AKT1/TCL1A	9	0.227848101
BP	GO:0000305	response to oxygen	3/115	29/18903	0.000714154	0.011585171	0.008583376	NFE2L2/PRDX1/NQO1	3	1.034482759

		radical								
BP	GO:1904377	positive regulation of protein localization to cell periphery	4/115	67/18903	0.000741626	0.011967502	0.008866642	CLN3/ITGB1/PRKCH/AKT1	4	0.597014925
BP	GO:0019058	viral life cycle	8/115	319/18903	0.000746552	0.011983914	0.008878802	CTBP1/EIF2AK2/EIF2AK4/HMGB1/SCARB2/PLSCR1/ITGB1/ZBED1	8	0.250783699
BP	GO:0043200	response to amino acid	5/115	118/18903	0.000773507	0.012351938	0.009151469	RRAGD/CEBPB/GCLC/LYN/NQO1	5	0.423728814
BP	GO:0042987	amyloid precursor protein catabolic process	4/115	68/18903	0.000784313	0.012459597	0.009231233	FKBP1A/CLN3/LYN/ROCK2	4	0.588235294
BP	GO:0072503	cellular divalent inorganic cation homeostasis	8/115	323/18903	0.000809359	0.012791204	0.009476918	FKBP1A/CLN3/IBTK/ANXA6/GSTO1/ATP1A2/LYN/SLC24A3	8	0.247678019
BP	GO:0001667	ameboidal-type cell migration	10/115	492/18903	0.000846515	0.013309817	0.009861155	GLUL/GADD45A/FGFR1/HMGB1/NFE2L2/CLN3/ITGB1/ANXA6/AKT1/ROCK2	10	0.203252033
BP	GO:0051101	regulation of DNA binding	5/115	121/18903	0.000866277	0.013551053	0.010039885	ZMPSTE24/DOT1L/HMGB1/HIPK2/MAD2L2	5	0.41322314
BP	GO:0000413	protein peptidyl-prolyl isomerization	3/115	31/18903	0.000870785	0.013552421	0.010040898	PPIC/FKBP1A/FKBP5	3	0.967741935
BP	GO:1903320	regulation of protein modification by small protein conjugation or removal	7/115	252/18903	0.000888863	0.013742834	0.010181974	SAE1/FKBP1A/GCLC/MAD2L2/MAGEA2B/EGR1/AKT1	7	0.277777778
BP	GO:0097696	receptor signaling pathway via STAT	6/115	183/18903	0.000891984	0.013742834	0.010181974	NF2/GADD45A/DOT1L/TYK2/PTPN2/LYN	6	0.327868852
BP	GO:0006476	protein deacetylation	5/115	122/18903	0.000898969	0.013781195	0.010210395	PRKAA1/CTBP1/DR1/NNMT/UCN	5	0.409836066
BP	GO:0034612	response to tumor necrosis factor	7/115	254/18903	0.000930786	0.014154763	0.01048717	CTSK/NFE2L2/MAPK3/PTPN2/MAP3K5/AKT1/TCL1A	7	0.275590551
BP	GO:0072655	establishment of protein localization to mitochondrion	5/115	123/18903	0.000932571	0.014154763	0.01048717	SH3GLB1/PRKAA1/CSNK2A2/SAE1/AKT1	5	0.406504065
BP	GO:0032200	telomere organization	6/115	185/18903	0.000943672	0.014252698	0.010559729	DOT1L/MYC/MAPK3/MAD2L2/RUVBL1/SMG1	6	0.324324324
BP	GO:0051209	release of sequestered calcium ion into cytosol	5/115	124/18903	0.000967099	0.014404312	0.010672059	FKBP1A/IBTK/GSTO1/ATP1A2/LYN	5	0.403225806
BP	GO:0009266	response to temperature stimulus	6/115	186/18903	0.000970365	0.014404312	0.010672059	PRKAA1/MAPKAPK2/EIF2AK4/GCLC/LYN/AKT1	6	0.322580645
BP	GO:0046626	regulation of insulin receptor signaling pathway	4/115	72/18903	0.000972502	0.014404312	0.010672059	PRKAA1/IGF2/PTPN2/OSBPL8	4	0.555555556
BP	GO:1900076	regulation of cellular response to insulin stimulus	4/115	72/18903	0.000972502	0.014404312	0.010672059	PRKAA1/IGF2/PTPN2/OSBPL8	4	0.555555556

BP	GO:0015980	energy derivation by oxidation of organic compounds	8/115	333/18903	0.000985136	0.014521278	0.010758718	COX5A/COX8A/BLOC1S1/MYC/IGF2/SLC25A12/AKT1/UCN	8	0.24024024
BP	GO:0043280	positive regulation of cysteine-type endopeptidase activity involved in apoptotic process	5/115	125/18903	0.001002569	0.014707537	0.010896716	SENP1/HMGB1/MYC/MAP3K5/RPS27L	5	0.4
BP	GO:0071260	cellular response to mechanical stimulus	4/115	73/18903	0.001024098	0.01495183	0.011077711	GADD45A/MAPK3/GCLC/ATP1A2	4	0.547945205
BP	GO:0000096	sulfur amino acid metabolic process	3/115	33/18903	0.001047648	0.015223169	0.011278744	GCLC/ADI1/MTHFD1	3	0.909090909
BP	GO:0006839	mitochondrial transport	6/115	189/18903	0.001053946	0.015242438	0.011293021	SH3GLB1/PRKAA1/CPT1B/CSNK2A2/SAE1/GCLC	6	0.317460317
BP	GO:0006875	cellular metal ion homeostasis	9/115	420/18903	0.001069802	0.015399126	0.011409109	MYC/FKBP1A/CLN3/IBTK/ANXA6/GSTO1/ATP1A2/LYN/SLC24A3	9	0.214285714
BP	GO:2000756	regulation of peptidyl-lysine acetylation	4/115	74/18903	0.001077587	0.015438706	0.011438435	PRKAA1/ZMPSTE24/CTBP1/PIWIL2	4	0.540540541
BP	GO:0016032	viral process	9/115	421/18903	0.001087685	0.015471162	0.011462481	CTBP1/EIF2AK2/EIF2AK4/HMGB1/SCARB2/PLSCR1/ITGB1/ZBED1/GALNT1	9	0.213776722
BP	GO:0034976	response to endoplasmic reticulum stress	7/115	261/18903	0.001089945	0.015471162	0.011462481	EIF2AK2/EIF2AK4/NFE2L2/CEBPB/PTPN2/MAP3K5/DERL3	7	0.268199234
BP	GO:0070585	protein localization to mitochondrion	5/115	128/18903	0.00111479	0.0157509	0.011669737	SH3GLB1/PRKAA1/CSNK2A2/SAE1/AKT1	5	0.390625
BP	GO:0043536	positive regulation of blood vessel endothelial cell migration	4/115	75/18903	0.001133008	0.01593488	0.011806047	FGFR1/HMGB1/NFE2L2/AKT1	4	0.533333333
BP	GO:0034405	response to fluid shear stress	3/115	34/18903	0.001143966	0.016015529	0.011865799	NFE2L2/PDGFRB/AKT1	3	0.882352941
BP	GO:0019079	viral genome replication	5/115	130/18903	0.001194608	0.016498509	0.012223635	CTBP1/EIF2AK2/EIF2AK4/PLSCR1/ZBED1	5	0.384615385
BP	GO:0031929	TOR signaling	5/115	130/18903	0.001194608	0.016498509	0.012223635	PRKAA1/ZMPSTE24/RRAGD/AKT1/SMG1	5	0.384615385
BP	GO:0032874	positive regulation of stress-activated MAPK cascade	5/115	130/18903	0.001194608	0.016498509	0.012223635	GADD45A/EIF2AK2/HMGB1/HIPK2/MAP3K5	5	0.384615385
BP	GO:0098656	anion transmembrane transport	7/115	266/18903	0.001216181	0.016695664	0.012369706	SLC25A12/CLN3/ITGB1/CLIC1/FOLR2/SLC1A4/ATP1A2	7	0.263157895
BP	GO:0052548	regulation of endopeptidase activity	9/115	428/18903	0.001219775	0.016695664	0.012369706	SENP1/HMGB1/MYC/MAP3K5/RPS27L/LYN/PRDX3/AKT1/ROCK2	9	0.210280374
BP	GO:0010595	positive regulation of endothelial cell migration	5/115	131/18903	0.001236068	0.016843481	0.012479223	FGFR1/HMGB1/NFE2L2/AKT1/ROCK2	5	0.381679389
BP	GO:0030968	endoplasmic reticulum	4/115	77/18903	0.001249794	0.016955175	0.012561976	EIF2AK2/NFE2L2/PTPN2/DERL3	4	0.519480519

		unfolded protein response								
BP	GO:0043966	histone H3 acetylation	4/115	78/18903	0.001311234	0.017513227	0.012975433	DR1/SUPT3H/BRD1/PIWIL2	4	0.512820513
BP	GO:0051966	regulation of synaptic transmission	4/115	78/18903	0.001311234	0.017513227	0.012975433	GLUL/CLN3/ATP1A2/UCN	4	0.512820513
BP	GO:1903533	regulation of protein targeting	4/115	78/18903	0.001311234	0.017513227	0.012975433	SH3GLB1/PRKAA1/CSNK2A2/SAE1	4	0.512820513
BP	GO:0001101	response to acid chemical	5/115	133/18903	0.001322168	0.017513227	0.012975433	RRAGD/CEBPB/GCLC/LYN/NQO1	5	0.37593985
BP	GO:0035601	protein deacylation	5/115	133/18903	0.001322168	0.017513227	0.012975433	PRKAA1/CTBP1/DR1/NNMT/UCN	5	0.37593985
BP	GO:0003018	vascular process in circulatory system	7/115	270/18903	0.001325202	0.017513227	0.012975433	SLC44A1/GCLC/SLC1A4/ATP1A2/ROCK2/UCN/SLC24A3	7	0.259259259
BP	GO:0046326	positive regulation of glucose import	3/115	37/18903	0.001465844	0.019187137	0.014215622	NFE2L2/OSBPL8/AKT1	3	0.810810811
BP	GO:0060416	response to growth hormone	3/115	37/18903	0.001465844	0.019187137	0.014215622	TYK2/LYN/AKT1	3	0.810810811
BP	GO:0045936	negative regulation of phosphate metabolic process	9/115	440/18903	0.001476242	0.019187137	0.014215622	NF2/GADD45A/FKBP1A/PTPN2/IBTK/LYN/PRDX3/AKT1/ROCK2	9	0.204545455
BP	GO:0022408	negative regulation of cell-cell adhesion	6/115	202/18903	0.001481833	0.019187137	0.014215622	NF2/HMGB1/CEBPB/PTPN2/MAD2L2/AKT1	6	0.297029703
BP	GO:0010563	negative regulation of phosphorus metabolic process	9/115	441/18903	0.001499438	0.019187137	0.014215622	NF2/GADD45A/FKBP1A/PTPN2/IBTK/LYN/PRDX3/AKT1/ROCK2	9	0.204081633
BP	GO:0072507	divalent inorganic cation homeostasis	8/115	356/18903	0.001506602	0.019187137	0.014215622	FKBP1A/CLN3/IBTK/ANXA6/GSTO1/ATP1A2/LYN/SLC24A3	8	0.224719101
BP	GO:0034101	erythrocyte homeostasis	5/115	137/18903	0.001507561	0.019187137	0.014215622	HIPK2/BRD1/PTPN2/LYN/PRDX1	5	0.364963504
BP	GO:0098732	macromolecule deacylation	5/115	137/18903	0.001507561	0.019187137	0.014215622	PRKAA1/CTBP1/DR1/NNMT/UCN	5	0.364963504
BP	GO:0072332	intrinsic apoptotic signaling pathway by p53 class mediator	4/115	81/18903	0.001508187	0.019187137	0.014215622	MYC/HIPK2/RPS27L/RRM2B	4	0.49382716
BP	GO:0051279	regulation of release of sequestered calcium ion into cytosol	4/115	82/18903	0.001578173	0.019535398	0.014473646	FKBP1A/GSTO1/ATP1A2/LYN	4	0.487804878
BP	GO:0072337	modified amino acid transport	3/115	38/18903	0.001584477	0.019535398	0.014473646	SLC25A12/FOLR2/SLC1A4	3	0.789473684
BP	GO:0006547	histidine metabolic process	2/115	10/18903	0.001599278	0.019535398	0.014473646	MTHFD1/HNMT	2	2
BP	GO:0009396	folic acid-containing compound biosynthetic process	2/115	10/18903	0.001599278	0.019535398	0.014473646	GART/MTHFD1	2	2

BP	GO:0010998	regulation of translational initiation by eIF2 alpha phosphorylation	2/115	10/18903	0.001599278	0.019535398	0.014473646	EIF2AK2/EIF2AK4	2	2
BP	GO:0032070	regulation of deoxyribonuclease activity	2/115	10/18903	0.001599278	0.019535398	0.014473646	HMGB1/AKT1	2	2
BP	GO:0051933	amino acid neurotransmitter reuptake	2/115	10/18903	0.001599278	0.019535398	0.014473646	ITGB1/ATP1A2	2	2
BP	GO:0060368	regulation of Fc receptor mediated stimulatory signaling pathway	2/115	10/18903	0.001599278	0.019535398	0.014473646	PLSCR1/LYN	2	2
BP	GO:0060397	growth hormone receptor signaling pathway via JAK-STAT	2/115	10/18903	0.001599278	0.019535398	0.014473646	TYK2/LYN	2	2
BP	GO:1902959	regulation of aspartic-type endopeptidase activity involved in amyloid precursor protein catabolic process	2/115	10/18903	0.001599278	0.019535398	0.014473646	LYN/ROCK2	2	2
BP	GO:0010038	response to metal ion	8/115	360/18903	0.001616306	0.019665057	0.014569709	PRKAA1/NFE2L2/MAPK3/SLC25A12/PLSCR1/GCLC/NQO1/AKT1	8	0.222222222
BP	GO:0002831	regulation of response to biotic stimulus	8/115	361/18903	0.001644699	0.019931408	0.014767048	ZMPSTE24/EIF2AK4/HMGB1/NFE2L2/MAPK3/PLSCR1/PTPN2/LYN	8	0.221606648
BP	GO:0043618	regulation of transcription from RNA polymerase II promoter in response to stress	3/115	39/18903	0.001708958	0.020547705	0.015223658	NFE2L2/CEBPB/EGR1	3	0.769230769
BP	GO:0060251	regulation of glial cell proliferation	3/115	39/18903	0.001708958	0.020547705	0.015223658	NF2/PRKCH/LYN	3	0.769230769
BP	GO:0008306	associative learning	4/115	85/18903	0.001801653	0.021577615	0.015986711	CLN3/ITGB1/ATP1A2/UCN	4	0.470588235
BP	GO:0010212	response to ionizing radiation	5/115	143/18903	0.001820628	0.02163584	0.016029849	PRKAA1/ZMPSTE24/GADD45A/MYC/EGR1	5	0.34965035
BP	GO:2001056	positive regulation of cysteine-type endopeptidase activity	5/115	143/18903	0.001820628	0.02163584	0.016029849	SENP1/HMGB1/MYC/MAP3K5/RPS27L	5	0.34965035
BP	GO:0010762	regulation of fibroblast migration	3/115	40/18903	0.001839388	0.021774378	0.016132492	CLN3/ITGB1/AKT1	3	0.75
BP	GO:0010918	positive regulation of mitochondrial	2/115	11/18903	0.001946911	0.022696489	0.016815677	AKT1/TCL1A	2	1.818181818

		membrane potential								
BP	GO:0061418	regulation of transcription from RNA polymerase II promoter in response to hypoxia	2/115	11/18903	0.001946911	0.022696489	0.016815677	NFE2L2/EGR1	2	1.818181818
BP	GO:0070778	L-aspartate transmembrane transport	2/115	11/18903	0.001946911	0.022696489	0.016815677	SLC25A12/SLC1A4	2	1.818181818
BP	GO:0072584	caveolin-mediated endocytosis	2/115	11/18903	0.001946911	0.022696489	0.016815677	MAPK3/CLN3	2	1.818181818
BP	GO:0010232	vascular transport	4/115	87/18903	0.001962278	0.022696489	0.016815677	SLC44A1/SLC1A4/ATP1A2/SLC24A3	4	0.459770115
BP	GO:0150104	transport across blood-brain barrier	4/115	87/18903	0.001962278	0.022696489	0.016815677	SLC44A1/SLC1A4/ATP1A2/SLC24A3	4	0.459770115
BP	GO:0052547	regulation of peptidase activity	9/115	459/18903	0.001969721	0.022696489	0.016815677	SENP1/HMGB1/MYC/MAP3K5/RPS27L/LYN/PRDX3/AKT1/ROCK2	9	0.196078431
BP	GO:0015711	organic anion transport	8/115	372/18903	0.001983907	0.022696489	0.016815677	PRKAA1/SLC7A7/SLC25A12/CLN3/ITGB1/FOLR2/SLC1A4/ATP1A2	8	0.215053763
BP	GO:0051098	regulation of binding	8/115	372/18903	0.001983907	0.022696489	0.016815677	ZMPSTE24/DOT1L/HMGB1/MAPK3/HIPK2/FKBP1A/MAD2L2/AKT1	8	0.215053763
BP	GO:2001233	regulation of apoptotic signaling pathway	8/115	374/18903	0.002051118	0.023378174	0.017320733	CSNK2A2/FGFR1/NFE2L2/MYC/GCLC/PTPN2/RRM2B/AKT1	8	0.213903743
BP	GO:0010632	regulation of epithelial cell migration	7/115	292/18903	0.002068697	0.023491206	0.017404478	GLUL/GADD45A/FGFR1/HMGB1/NFE2L2/AKT1/ROCK2	7	0.239726027
BP	GO:0006469	negative regulation of protein kinase activity	6/115	216/18903	0.002078901	0.02351996	0.017425782	NF2/GADD45A/PTPN2/IBTK/LYN/AKT1	6	0.277777778
BP	GO:0044272	sulfur compound biosynthetic process	5/115	148/18903	0.002115903	0.023792326	0.017627576	NFE2L2/GCLC/ADI1/MTHFD1/MGST2	5	0.337837838
BP	GO:0070296	sarcoplasmic reticulum calcium ion transport	3/115	42/18903	0.002118495	0.023792326	0.017627576	ZMPSTE24/GSTO1/ATP1A2	3	0.714285714
BP	GO:1901215	negative regulation of neuron death	6/115	218/18903	0.002177161	0.024361957	0.018049612	HIPK2/CEBPB/CLN3/GCLC/AKT1/UCN	6	0.275229358
BP	GO:0044262	cellular carbohydrate metabolic process	7/115	296/18903	0.002233025	0.024829979	0.018396366	ZMPSTE24/SCARB2/IGF2/SLC25A12/PTPN2/NNMT/AKT1	7	0.236486486
BP	GO:0007612	learning	5/115	150/18903	0.002243283	0.024829979	0.018396366	EIF2AK4/CLN3/ITGB1/ATP1A2/UCN	5	0.333333333
BP	GO:0043535	regulation of blood vessel endothelial cell migration	5/115	150/18903	0.002243283	0.024829979	0.018396366	GADD45A/FGFR1/HMGB1/NFE2L2/AKT1	5	0.333333333
BP	GO:0009086	methionine biosynthetic process	2/115	12/18903	0.00232702	0.025480867	0.018878604	ADI1/MTHFD1	2	1.666666667
BP	GO:0072110	glomerular mesangial cell proliferation	2/115	12/18903	0.00232702	0.025480867	0.018878604	PDGFRB/EGR1	2	1.666666667
BP	GO:1905245	regulation of	2/115	12/18903	0.00232702	0.025480867	0.018878604	LYN/ROCK2	2	1.666666667

		aspartic-type peptidase activity								
BP	GO:0010828	positive regulation of glucose transmembrane transport	3/115	44/18903	0.002422567	0.026338976	0.019514371	NFE2L2/OSBPL8/AKT1	3	0.681818182
BP	GO:0031063	regulation of histone deacetylation	3/115	44/18903	0.002422567	0.026338976	0.019514371	CTBP1/DR1/UCN	3	0.681818182
BP	GO:0050679	positive regulation of epithelial cell proliferation	6/115	224/18903	0.002493073	0.026864208	0.019903512	GLUL/FGFR1/HMGB1/MYC/IGF2/AKT1	6	0.267857143
BP	GO:0044070	regulation of anion transport	4/115	93/18903	0.002503215	0.026864208	0.019903512	PRKAA1/CEBPB/ITGB1/ATP1A2	4	0.430107527
BP	GO:0070301	cellular response to hydrogen peroxide	4/115	93/18903	0.002503215	0.026864208	0.019903512	PRKAA1/NFE2L2/MAP3K5/NQO1	4	0.430107527
BP	GO:0000723	telomere maintenance	5/115	154/18903	0.002514686	0.026864208	0.019903512	MYC/MAPK3/MAD2L2/RUVBL1/SMG1	5	0.324675325
BP	GO:0050905	neuromuscular process	5/115	154/18903	0.002514686	0.026864208	0.019903512	ZMPSTE24/HIPK2/CLN3/GSTO1/UCN	5	0.324675325
BP	GO:0008631	intrinsic apoptotic signaling pathway in response to oxidative stress	3/115	45/18903	0.002584196	0.027321188	0.020242086	NFE2L2/MAP3K5/AKT1	3	0.666666667
BP	GO:0010712	regulation of collagen metabolic process	3/115	45/18903	0.002584196	0.027321188	0.020242086	PDGFRB/ITGB1/UCN	3	0.666666667
BP	GO:0043620	regulation of DNA-templated transcription in response to stress	3/115	45/18903	0.002584196	0.027321188	0.020242086	NFE2L2/CEBPB/EGR1	3	0.666666667
BP	GO:0042982	amyloid precursor protein metabolic process	4/115	95/18903	0.002704176	0.028182548	0.020880262	FKBP1A/CLN3/LYN/ROCK2	4	0.421052632
BP	GO:0031349	positive regulation of defense response	7/115	307/18903	0.002737275	0.028182548	0.020880262	HMGB1/MAPK3/CEBPB/PLSCR1/LYN/MGST2/UCN	7	0.228013029
BP	GO:0044403	biological process involved in symbiotic interaction	7/115	307/18903	0.002737275	0.028182548	0.020880262	EIF2AK4/HMGB1/SCARB2/PLSCR1/ITGB1/ZBED1/ROCK2	7	0.228013029
BP	GO:0006521	regulation of cellular amino acid metabolic process	2/115	13/18903	0.002739204	0.028182548	0.020880262	SLC7A7/CLN3	2	1.538461538
BP	GO:0006534	cysteine metabolic process	2/115	13/18903	0.002739204	0.028182548	0.020880262	GCLC/MTHFD1	2	1.538461538
BP	GO:0072537	fibroblast activation	2/115	13/18903	0.002739204	0.028182548	0.020880262	PDGFRB/GCLC	2	1.538461538
BP	GO:1990253	cellular response to leucine starvation	2/115	13/18903	0.002739204	0.028182548	0.020880262	EIF2AK4/RRAGD	2	1.538461538

BP	GO:2001198	regulation of dendritic cell differentiation	2/115	13/18903	0.002739204	0.028182548	0.020880262	HMGB1/CEBPB	2	1.538461538
BP	GO:0044788	modulation by host of viral process	3/115	46/18903	0.002752338	0.028222966	0.020910207	EIF2AK4/ZBED1/ROCK2	3	0.652173913
BP	GO:0010876	lipid localization	9/115	483/18903	0.002773093	0.02834101	0.020997666	PRKAA1/CPT1B/OSBPL5/SCARB2/CLN3/PLSCR1/PTPN2/OSBPL8/AKT1	9	0.186335404
BP	GO:0046330	positive regulation of JNK cascade	4/115	96/18903	0.002808688	0.028514694	0.021126347	GADD45A/HMGB1/HIPK2/MAP3K5	4	0.416666667
BP	GO:1903321	negative regulation of protein modification by small protein conjugation or removal	4/115	96/18903	0.002808688	0.028514694	0.021126347	GCLC/MAD2L2/MAGEA2B/AKT1	4	0.416666667
BP	GO:0050920	regulation of chemotaxis	6/115	230/18903	0.00284232	0.028760899	0.021308759	FGFR1/HMGB1/PDGFRB/MAPK3/PTPN2/LYN	6	0.260869565
BP	GO:0006417	regulation of translation	9/115	486/18903	0.002889524	0.02914237	0.021591388	EIF2AK2/EIF2AK4/SECISBP2/PIWIL2/RPS27L/MKNK1/AKT1/ROCK2/UCN	9	0.185185185
BP	GO:0097028	dendritic cell differentiation	3/115	47/18903	0.002927078	0.029424331	0.02180029	HMGB1/CEBPB/LYN	3	0.638297872
BP	GO:0006650	glycerophospholipid metabolic process	7/115	311/18903	0.00294086	0.029466268	0.021831362	OSBPL5/SLC44A1/PDGFRB/CLN3/PLSCR1/OSBPL8/SMG1	7	0.225080386
BP	GO:1902600	proton transmembrane transport	5/115	160/18903	0.002965472	0.029616085	0.02194236	ATP6V1E2/COX5A/ATP6V1F/COX8A/ATP1A2	5	0.3125
BP	GO:2001020	regulation of response to DNA damage stimulus	7/115	312/18903	0.002993526	0.029799195	0.022078025	ZMPSTE24/HMGB1/SUPT3H/MYC/MAD2L2/RUVBL1/SMG1	7	0.224358974
BP	GO:0060485	mesenchyme development	7/115	313/18903	0.003046913	0.030172991	0.022354968	FGFR1/PDGFRB/MYC/MAPK3/ANXA6/MAD2L2/ROCK2	7	0.223642173
BP	GO:0022407	regulation of cell-cell adhesion	9/115	490/18903	0.003050759	0.030172991	0.022354968	NF2/HMGB1/TYK2/CEBPB/IGF2/PTPN2/LYN/MAD2L2/AKT1	9	0.183673469
BP	GO:0009743	response to carbohydrate	6/115	234/18903	0.003094725	0.030402739	0.022525187	PRKAA1/GLUL/GCLC/LYN/NQO1/EGR1	6	0.256410256
BP	GO:0050890	cognition	7/115	314/18903	0.003101027	0.030402739	0.022525187	EIF2AK4/CEBPB/CLN3/ITGB1/SLC1A4/ATP1A2/UCN	7	0.222929936
BP	GO:0072593	reactive oxygen species metabolic process	6/115	235/18903	0.003160364	0.030402739	0.022525187	GADD45A/NFE2L2/PDGFRB/PRDX1/PRDX3/NQO1	6	0.255319149
BP	GO:0006563	L-serine metabolic process	2/115	14/18903	0.003183066	0.030402739	0.022525187	SDSL/PSAT1	2	1.428571429
BP	GO:0006596	polyamine biosynthetic process	2/115	14/18903	0.003183066	0.030402739	0.022525187	PAOX/NNMT	2	1.428571429
BP	GO:0035791	platelet-derived growth factor receptor-beta signaling pathway	2/115	14/18903	0.003183066	0.030402739	0.022525187	PDGFRB/PTPN2	2	1.428571429
BP	GO:0042754	negative regulation of circadian rhythm	2/115	14/18903	0.003183066	0.030402739	0.022525187	SUV39H2/PIWIL2	2	1.428571429

BP	GO:0043558	regulation of translational initiation in response to stress	2/115	14/18903	0.003183066	0.030402739	0.022525187	EIF2AK2/EIF2AK4	2	1.428571429
BP	GO:0043650	dicarboxylic acid biosynthetic process	2/115	14/18903	0.003183066	0.030402739	0.022525187	SLC25A12/MTHFD1	2	1.428571429
BP	GO:0044793	negative regulation by host of viral process	2/115	14/18903	0.003183066	0.030402739	0.022525187	EIF2AK4/ZBED1	2	1.428571429
BP	GO:0090239	regulation of histone H4 acetylation	2/115	14/18903	0.003183066	0.030402739	0.022525187	ZMPSTE24/CTBP1	2	1.428571429
BP	GO:0001776	leukocyte homeostasis	4/115	100/18903	0.00325443	0.0308919	0.022887603	HMGB1/MTHFD1/LYN/AKT1	4	0.4
BP	GO:0034620	cellular response to unfolded protein	4/115	100/18903	0.00325443	0.0308919	0.022887603	EIF2AK2/NFE2L2/PTPN2/DERL3	4	0.4
BP	GO:0018208	peptidyl-proline modification	3/115	49/18903	0.003296687	0.031196422	0.023113221	PPIC/FKBP1A/FKBP5	3	0.612244898
BP	GO:0032204	regulation of telomere maintenance	4/115	101/18903	0.003372963	0.031722405	0.023502919	MYC/MAPK3/RUVBL1/SMG1	4	0.396039604
BP	GO:1905477	positive regulation of protein localization to membrane	4/115	101/18903	0.003372963	0.031722405	0.023502919	CLN3/ITGB1/PRKCH/AKT1	4	0.396039604
BP	GO:0089718	amino acid import across plasma membrane	3/115	50/18903	0.003491715	0.032723297	0.024244472	ITGB1/SLC1A4/ATP1A2	3	0.6
BP	GO:0009416	response to light stimulus	7/115	321/18903	0.003500731	0.032723297	0.024244472	PRKAA1/CPT1B/EIF2AK4/MYC/ITGB1/ATP1A2/AKT1	7	0.218068536
BP	GO:0070665	positive regulation of leukocyte proliferation	5/115	167/18903	0.003562093	0.032795541	0.024297997	HMGB1/TYK2/MAPK3/IGF2/LYN	5	0.299401198
BP	GO:0043255	regulation of carbohydrate biosynthetic process	4/115	103/18903	0.003618784	0.032795541	0.024297997	IGF2/PTPN2/NNMT/AKT1	4	0.388349515
BP	GO:0048144	fibroblast proliferation	4/115	103/18903	0.003618784	0.032795541	0.024297997	ZMPSTE24/PDGFRB/MYC/PRDX1	4	0.388349515
BP	GO:1903076	regulation of protein localization to plasma membrane	4/115	103/18903	0.003618784	0.032795541	0.024297997	CLN3/ITGB1/PRKCH/AKT1	4	0.388349515
BP	GO:0009071	serine family amino acid catabolic process	2/115	15/18903	0.003658211	0.032795541	0.024297997	GCSH/SDSL	2	1.333333333
BP	GO:0033689	negative regulation of osteoblast proliferation	2/115	15/18903	0.003658211	0.032795541	0.024297997	NF2/EIF2AK2	2	1.333333333
BP	GO:0042532	negative regulation of tyrosine phosphorylation of STAT protein	2/115	15/18903	0.003658211	0.032795541	0.024297997	NF2/PTPN2	2	1.333333333
BP	GO:0042559	pteridine-containing	2/115	15/18903	0.003658211	0.032795541	0.024297997	GART/MTHFD1	2	1.333333333

		compound biosynthetic process								
BP	GO:0045725	positive regulation of glycogen biosynthetic process	2/115	15/18903	0.003658211	0.032795541	0.024297997	IGF2/AKT1	2	1.333333333
BP	GO:0045989	positive regulation of striated muscle contraction	2/115	15/18903	0.003658211	0.032795541	0.024297997	GSTO1/UCN	2	1.333333333
BP	GO:0071380	cellular response to prostaglandin E stimulus	2/115	15/18903	0.003658211	0.032795541	0.024297997	PRKAA1/AKT1	2	1.333333333
BP	GO:0072075	metanephric mesenchyme development	2/115	15/18903	0.003658211	0.032795541	0.024297997	PDGFRB/MYC	2	1.333333333
BP	GO:0090280	positive regulation of calcium ion import	2/115	15/18903	0.003658211	0.032795541	0.024297997	PDGFRB/UCN	2	1.333333333
BP	GO:1901722	regulation of cell proliferation involved in kidney development	2/115	15/18903	0.003658211	0.032795541	0.024297997	MYC/EGR1	2	1.333333333
BP	GO:0101023	vascular endothelial cell proliferation	3/115	51/18903	0.003693663	0.032920843	0.024390833	FGFR1/HMGB1/IGF2	3	0.588235294
BP	GO:1905562	regulation of vascular endothelial cell proliferation	3/115	51/18903	0.003693663	0.032920843	0.024390833	FGFR1/HMGB1/IGF2	3	0.588235294
BP	GO:0006626	protein targeting to mitochondrion	4/115	104/18903	0.00374614	0.033195563	0.024594372	SH3GLB1/PRKAA1/CSNK2A2/SAE1	4	0.384615385
BP	GO:0015914	phospholipid transport	4/115	104/18903	0.00374614	0.033195563	0.024594372	OSBPL5/SCARB2/PLSCR1/OSBPL8	4	0.384615385
BP	GO:0007159	leukocyte cell-cell adhesion	8/115	414/18903	0.003815439	0.033712204	0.024977147	HMGB1/TYK2/CEBPB/IGF2/ITGB1/PTPN2/LYN/AKT1	8	0.193236715
BP	GO:2000058	regulation of ubiquitin-dependent protein catabolic process	5/115	170/18903	0.003842479	0.033853563	0.025081879	CSNK2A2/NFE2L2/HIPK2/GCLC/AKT1	5	0.294117647
BP	GO:0002762	negative regulation of myeloid leukocyte differentiation	3/115	52/18903	0.003902605	0.034284772	0.025401359	MYC/PTPN2/LYN	3	0.576923077
BP	GO:0031330	negative regulation of cellular catabolic process	6/115	247/18903	0.004031687	0.035271907	0.026132721	MAPKAPK2/CSNK2A2/SECISBP2/HIPK2/AKT1/SMG1	6	0.24291498
BP	GO:2001242	regulation of intrinsic apoptotic signaling pathway	5/115	172/18903	0.004037978	0.035271907	0.026132721	NFE2L2/MYC/PTPN2/RRM2B/AKT1	5	0.290697674

BP	GO:2000677	regulation of transcription regulatory region DNA binding	3/115	53/18903	0.004118613	0.035763555	0.02649698	ZMPSTE24/DOT1L/MAD2L2	3	0.566037736
BP	GO:0032963	collagen metabolic process	4/115	107/18903	0.004146392	0.035763555	0.02649698	CTSK/PDGFRB/ITGB1/UCN	4	0.373831776
BP	GO:0006544	glycine metabolic process	2/115	16/18903	0.00416425	0.035763555	0.02649698	GCSH/GART	2	1.25
BP	GO:0006555	methionine metabolic process	2/115	16/18903	0.00416425	0.035763555	0.02649698	ADI1/MTHFD1	2	1.25
BP	GO:0045838	positive regulation of membrane potential	2/115	16/18903	0.00416425	0.035763555	0.02649698	AKT1/TCL1A	2	1.25
BP	GO:0070875	positive regulation of glycogen metabolic process	2/115	16/18903	0.00416425	0.035763555	0.02649698	IGF2/AKT1	2	1.25
BP	GO:1902106	negative regulation of leukocyte differentiation	4/115	108/18903	0.004285982	0.036706199	0.027195378	HMGB1/MYC/PTPN2/LYN	4	0.37037037
BP	GO:0010959	regulation of metal ion transport	8/115	423/18903	0.004339322	0.03697731	0.027396242	PDGFRB/FKBP1A/ITGB1/GSTO1/ATP1A2/LYN/AKT1/UCN	8	0.189125296
BP	GO:0014009	glial cell proliferation	3/115	54/18903	0.004341758	0.03697731	0.027396242	NF2/PRKCH/LYN	3	0.555555556
BP	GO:0010634	positive regulation of epithelial cell migration	5/115	176/18903	0.004450174	0.037795659	0.028002552	FGFR1/HMGB1/NFE2L2/AKT1/ROCK2	5	0.284090909
BP	GO:0010761	fibroblast migration	3/115	55/18903	0.00457211	0.038617323	0.028611317	CLN3/ITGB1/AKT1	3	0.545454545
BP	GO:1902991	regulation of amyloid precursor protein catabolic process	3/115	55/18903	0.00457211	0.038617323	0.028611317	FKBP1A/LYN/ROCK2	3	0.545454545
BP	GO:0000097	sulfur amino acid biosynthetic process	2/115	17/18903	0.004700795	0.038783646	0.028734544	ADI1/MTHFD1	2	1.176470588
BP	GO:0010763	positive regulation of fibroblast migration	2/115	17/18903	0.004700795	0.038783646	0.028734544	ITGB1/AKT1	2	1.176470588
BP	GO:0072109	glomerular mesangium development	2/115	17/18903	0.004700795	0.038783646	0.028734544	PDGFRB/EGR1	2	1.176470588
BP	GO:0072224	metanephric glomerulus development	2/115	17/18903	0.004700795	0.038783646	0.028734544	PDGFRB/EGR1	2	1.176470588
BP	GO:0098712	L-glutamate import across plasma membrane	2/115	17/18903	0.004700795	0.038783646	0.028734544	ITGB1/ATP1A2	2	1.176470588
BP	GO:1902036	regulation of hematopoietic stem cell differentiation	2/115	17/18903	0.004700795	0.038783646	0.028734544	EIF2AK2/NFE2L2	2	1.176470588
BP	GO:1990822	basic amino acid transmembrane transport	2/115	17/18903	0.004700795	0.038783646	0.028734544	SLC7A7/CLN3	2	1.176470588

BP	GO:0032496	response to lipopolysaccharide	7/115	339/18903	0.004709423	0.038783646	0.028734544	MAPKAPK2/HMGB1/MAPK3/CEBPB/LYN/PRDX3/AKT1	7	0.206489676
BP	GO:0001503	ossification	8/115	429/18903	0.004718297	0.038783646	0.028734544	ZMPSTE24/CTSK/MAPK3/CEBPB/IGF2/CLIC1/AKT1/SLC24A3	8	0.186480186
BP	GO:0031331	positive regulation of cellular catabolic process	8/115	429/18903	0.004718297	0.038783646	0.028734544	SH3GLB1/PRKAA1/HMGB1/NFE2L2/MAPK3/GCLC/AKT1/ROCK2	8	0.186480186
BP	GO:0070371	ERK1 and ERK2 cascade	7/115	340/18903	0.004784762	0.03922481	0.0290614	SHC1/HMGB1/PDGFRB/MYC/MAPK3/PTPN2/LYN	7	0.205882353
BP	GO:0043470	regulation of carbohydrate catabolic process	3/115	56/18903	0.004809733	0.039324375	0.029135167	PRKAA1/SCARB2/SLC25A12	3	0.535714286
BP	GO:0042391	regulation of membrane potential	8/115	431/18903	0.004850124	0.039549148	0.0293017	ZMPSTE24/CLN3/GCLC/CLIC1/ATP1A2/PRDX3/AKT1/TCL1A	8	0.185614849
BP	GO:0035265	organ growth	5/115	180/18903	0.004891546	0.039781116	0.029473564	ZMPSTE24/PDGFRB/IGF2/ANXA6/AKT1	5	0.277777778
BP	GO:0009410	response to xenobiotic stimulus	8/115	432/18903	0.00491709	0.039883066	0.029549098	PRKAA1/NFE2L2/MYC/GCLC/GSTO1/LYN/NQO1/NNMT	8	0.185185185
BP	GO:0006869	lipid transport	8/115	433/18903	0.004984765	0.040325306	0.02987675	PRKAA1/CPT1B/OSBPL5/SCARB2/CLN3/PLSCR1/OSBPL8/AKT1	8	0.184757506
BP	GO:0038066	p38MAPK cascade	3/115	57/18903	0.005054692	0.040577993	0.030063964	MAPKAPK2/GADD45A/MAP3K5	3	0.526315789
BP	GO:0097553	calcium ion transmembrane import into cytosol	5/115	182/18903	0.005123514	0.040577993	0.030063964	FKBP1A/IBTK/GSTO1/ATP1A2/LYN	5	0.274725275
BP	GO:0070588	calcium ion transmembrane transport	7/115	345/18903	0.005175198	0.040577993	0.030063964	FKBP1A/IBTK/ANXA6/GSTO1/ATP1A2/LYN/SLC24A3	7	0.202898551
BP	GO:0009408	response to heat	4/115	114/18903	0.005190587	0.040577993	0.030063964	MAPKAPK2/GCLC/LYN/AKT1	4	0.350877193
BP	GO:0030004	cellular monovalent inorganic cation homeostasis	4/115	114/18903	0.005190587	0.040577993	0.030063964	ATP6V1F/MAPK3/CLN3/ATP1A2	4	0.350877193
BP	GO:1903707	negative regulation of hemopoiesis	4/115	114/18903	0.005190587	0.040577993	0.030063964	HMGB1/MYC/PTPN2/LYN	4	0.350877193
BP	GO:0071466	cellular response to xenobiotic stimulus	5/115	183/18903	0.005242377	0.040577993	0.030063964	PRKAA1/NFE2L2/MYC/GSTO1/NQO1	5	0.273224044
BP	GO:0071216	cellular response to biotic stimulus	6/115	261/18903	0.005260101	0.040577993	0.030063964	ZMPSTE24/HMGB1/MAPK3/CEBPB/LYN/AKT1	6	0.229885057
BP	GO:0006595	polyamine metabolic process	2/115	18/18903	0.005267463	0.040577993	0.030063964	PAOX/NNMT	2	1.111111111
BP	GO:0006978	DNA damage response	2/115	18/18903	0.005267463	0.040577993	0.030063964	HIPK2/RPS27L	2	1.111111111
BP	GO:0010958	regulation of amino acid import across plasma membrane	2/115	18/18903	0.005267463	0.040577993	0.030063964	ITGB1/ATP1A2	2	1.111111111

BP	GO:0015802	basic amino acid transport	2/115	18/18903	0.005267463	0.040577993	0.030063964	SLC7A7/CLN3	2	1.111111111
BP	GO:0036499	PERK-mediated unfolded protein response	2/115	18/18903	0.005267463	0.040577993	0.030063964	NFE2L2/PTPN2	2	1.111111111
BP	GO:0045722	positive regulation of gluconeogenesis	2/115	18/18903	0.005267463	0.040577993	0.030063964	PTPN2/NNMT	2	1.111111111
BP	GO:0046653	tetrahydrofolate metabolic process	2/115	18/18903	0.005267463	0.040577993	0.030063964	GART/MTHFD1	2	1.111111111
BP	GO:0051580	regulation of neurotransmitter uptake	2/115	18/18903	0.005267463	0.040577993	0.030063964	ITGB1/ATP1A2	2	1.111111111
BP	GO:0060644	mammary gland epithelial cell differentiation	2/115	18/18903	0.005267463	0.040577993	0.030063964	CEBPB/AKT1	2	1.111111111
BP	GO:1902001	fatty acid transmembrane transport	2/115	18/18903	0.005267463	0.040577993	0.030063964	CPT1B/AKT1	2	1.111111111
BP	GO:1903789	regulation of amino acid transmembrane transport	2/115	18/18903	0.005267463	0.040577993	0.030063964	ITGB1/ATP1A2	2	1.111111111
BP	GO:0050732	negative regulation of peptidyl-tyrosine phosphorylation	3/115	58/18903	0.005307051	0.040780497	0.030213998	NF2/PTPN2/IBTK	3	0.517241379
BP	GO:0001938	positive regulation of endothelial cell proliferation	4/115	115/18903	0.005352829	0.041029433	0.030398433	FGFR1/HMGB1/IGF2/AKT1	4	0.347826087
BP	GO:0043414	macromolecule methylation	7/115	348/18903	0.005420703	0.041446071	0.030707118	ZMPSTE24/SUV39H2/DOT1L/MYC/PRMT2/PIWIL2/NNMT	7	0.201149425
BP	GO:0043666	regulation of phosphoprotein phosphatase activity	3/115	59/18903	0.005566869	0.042247575	0.031300947	PDGFRB/FKBP1A/ROCK2	3	0.508474576
BP	GO:0046324	regulation of glucose import	3/115	59/18903	0.005566869	0.042247575	0.031300947	NFE2L2/OSBPL8/AKT1	3	0.508474576
BP	GO:0051785	positive regulation of nuclear division	3/115	59/18903	0.005566869	0.042247575	0.031300947	PDGFRB/IGF2/PIWIL2	3	0.508474576
BP	GO:0042060	wound healing	8/115	442/18903	0.005626539	0.042594983	0.031558339	NFE2L2/PDGFRB/PLSCR1/ITGB1/CLIC1/ANXA6/MAP3K5/LYN	8	0.180995475
BP	GO:0050730	regulation of peptidyl-tyrosine phosphorylation	6/115	266/18903	0.005759148	0.043384638	0.032143389	NF2/SHC1/IGF2/PTPN2/IBTK/LYN	6	0.22556391
BP	GO:0051091	positive regulation of DNA-binding	6/115	266/18903	0.005759148	0.043384638	0.032143389	PSMA6/EIF2AK2/HIPK2/PRKCH/PRDX3/AKT1	6	0.22556391

		transcription factor activity								
BP	GO:0046686	response to cadmium ion	3/115	60/18903	0.005834205	0.043531813	0.03225243	MAPK3/GCLC/AKT1	3	0.5
BP	GO:0003417	growth plate cartilage development	2/115	19/18903	0.005863874	0.043531813	0.03225243	ZMPSTE24/ANXA6	2	1.052631579
BP	GO:0036003	positive regulation of transcription from RNA polymerase II promoter in response to stress	2/115	19/18903	0.005863874	0.043531813	0.03225243	NFE2L2/CEBPB	2	1.052631579
BP	GO:0072074	kidney mesenchyme development	2/115	19/18903	0.005863874	0.043531813	0.03225243	PDGFRB/MYC	2	1.052631579
BP	GO:0090043	regulation of tubulin deacetylation	2/115	19/18903	0.005863874	0.043531813	0.03225243	PRKAA1/DR1	2	1.052631579
BP	GO:1902176	negative regulation of oxidative stress-induced intrinsic apoptotic signaling pathway	2/115	19/18903	0.005863874	0.043531813	0.03225243	NFE2L2/AKT1	2	1.052631579
BP	GO:0051924	regulation of calcium ion transport	6/115	268/18903	0.005968161	0.044198989	0.032746736	PDGFRB/FKBP1A/GSTO1/ATP1A2/LYN/UCN	6	0.223880597
BP	GO:0071478	cellular response to radiation	5/115	189/18903	0.005996926	0.044305	0.032825279	ZMPSTE24/GADD45A/EIF2AK4/MYC/EGR1	5	0.264550265
BP	GO:0070663	regulation of leukocyte proliferation	6/115	269/18903	0.006074722	0.044771866	0.033171177	HMGB1/TYK2/MAPK3/CEBPB/IGF2/LYN	6	0.223048327
BP	GO:0051302	regulation of cell division	5/115	190/18903	0.006129735	0.045068986	0.033391311	SH3GLB1/DR1/AURKC/MYC/IGF2	5	0.263157895
BP	GO:0035967	cellular response to topologically incorrect protein	4/115	120/18903	0.00621507	0.045370014	0.033614341	EIF2AK2/NFE2L2/PTPN2/DERL3	4	0.333333333
BP	GO:0042752	regulation of circadian rhythm	4/115	120/18903	0.00621507	0.045370014	0.033614341	PRKAA1/SUV39H2/PIWIL2/ROCK2	4	0.333333333
BP	GO:0051897	positive regulation of protein kinase B signaling	4/115	120/18903	0.00621507	0.045370014	0.033614341	FGFR1/IGF2/ITGB1/OSBPL8	4	0.333333333
BP	GO:0001780	neutrophil homeostasis	2/115	20/18903	0.006489653	0.046938544	0.034776454	HMGB1/MTHFD1	2	1
BP	GO:0048643	positive regulation of skeletal muscle tissue development	2/115	20/18903	0.006489653	0.046938544	0.034776454	PRKAA1/IGF2	2	1
BP	GO:0071379	cellular response to prostaglandin stimulus	2/115	20/18903	0.006489653	0.046938544	0.034776454	PRKAA1/AKT1	2	1
BP	GO:0002237	response to molecule of bacterial origin	7/115	360/18903	0.006491175	0.046938544	0.034776454	MAPKAPK2/HMGB1/MAPK3/CEBPB/LYN/PRDX3/AKT1	7	0.194444444

BP	GO:0007611	learning or memory	6/115	273/18903	0.006514897	0.046999232	0.034821418	EIF2AK4/CEBPB/CLN3/ITGB1/ATP1A2/UCN	6	0.21978022
BP	GO:0002761	regulation of myeloid leukocyte differentiation	4/115	122/18903	0.006584341	0.047388706	0.035109976	MYC/CEBPB/PTPN2/LYN	4	0.327868852
BP	GO:0061448	connective tissue development	6/115	274/18903	0.006628475	0.047594625	0.03526254	ZMPSTE24/CTSK/PDGFRB/MAPK3/ANXA6/EGR1	6	0.218978102
BP	GO:0031663	lipopolysaccharide-mediated signaling pathway	3/115	63/18903	0.006681878	0.047754404	0.035380919	MAPK3/LYN/AKT1	3	0.476190476
BP	GO:0070059	intrinsic apoptotic signaling pathway in response to endoplasmic reticulum stress	3/115	63/18903	0.006681878	0.047754404	0.035380919	CEBPB/PTPN2/MAP3K5	3	0.476190476
BP	GO:0071675	regulation of mononuclear cell migration	4/115	123/18903	0.006774316	0.04830245	0.035786963	HMGB1/MAPK3/LYN/AKT1	4	0.325203252
BP	GO:0007042	lysosomal lumen acidification	2/115	21/18903	0.007144425	0.050471904	0.037394297	ATP6V1F/CLN3	2	0.952380952
BP	GO:0009083	branched-chain amino acid catabolic process	2/115	21/18903	0.007144425	0.050471904	0.037394297	HMGCL/BCAT2	2	0.952380952
BP	GO:0043555	regulation of translation in response to stress	2/115	21/18903	0.007144425	0.050471904	0.037394297	EIF2AK2/EIF2AK4	2	0.952380952
BP	GO:0060252	positive regulation of glial cell proliferation	2/115	21/18903	0.007144425	0.050471904	0.037394297	PRKCH/LYN	2	0.952380952
BP	GO:1904375	regulation of protein localization to cell periphery	4/115	125/18903	0.007165091	0.050501537	0.037416252	CLN3/ITGB1/PRKCH/AKT1	4	0.32
BP	GO:0071248	cellular response to metal ion	5/115	198/18903	0.007267691	0.050652085	0.037527792	PRKAA1/NFE2L2/MAPK3/NQO1/AKT1	5	0.252525253
BP	GO:0071897	DNA biosynthetic process	5/115	198/18903	0.007267691	0.050652085	0.037527792	PDGFRB/MYC/MAPK3/MAD2L2/RRM2B	5	0.252525253
BP	GO:0031100	animal organ regeneration	3/115	65/18903	0.007285574	0.050652085	0.037527792	CSNK2A2/CEBPB/NNMT	3	0.461538462
BP	GO:0035065	regulation of histone acetylation	3/115	65/18903	0.007285574	0.050652085	0.037527792	ZMPSTE24/CTBP1/PIWIL2	3	0.461538462
BP	GO:0040014	regulation of multicellular organism growth	3/115	65/18903	0.007285574	0.050652085	0.037527792	ZMPSTE24/IGF2/GAMT	3	0.461538462
BP	GO:1905953	negative regulation of lipid localization	3/115	65/18903	0.007285574	0.050652085	0.037527792	PTPN2/OSBPL8/AKT1	3	0.461538462
BP	GO:0032103	positive regulation of response to external stimulus	8/115	464/18903	0.007461105	0.051755087	0.038344999	FGFR1/HMGB1/PDGFRB/MAPK3/CEBPB/PLSCR1/LYN/MGST2	8	0.172413793
BP	GO:0050854	regulation of antigen	3/115	66/18903	0.007599144	0.052593621	0.038966263	PTPN2/PRKCH/LYN	3	0.454545455

		receptor-mediated signaling pathway								
BP	GO:0030218	erythrocyte differentiation	4/115	128/18903	0.007778733	0.052863654	0.039166329	HIPK2/BRD1/PTPN2/LYN	4	0.3125
BP	GO:0006338	chromatin remodeling	7/115	373/18903	0.007821072	0.052863654	0.039166329	CTBP1/DR1/SUV39H2/DOT1L/HMGB1/MYC/RUVBL1	7	0.18766756
BP	GO:0006907	pinocytosis	2/115	22/18903	0.007827821	0.052863654	0.039166329	MAPKAPK2/CLN3	2	0.909090909
BP	GO:0009070	serine family amino acid biosynthetic process	2/115	22/18903	0.007827821	0.052863654	0.039166329	MTHFD1/PSAT1	2	0.909090909
BP	GO:0010881	regulation of cardiac muscle contraction by regulation of the release of sequestered calcium ion	2/115	22/18903	0.007827821	0.052863654	0.039166329	GSTO1/ATP1A2	2	0.909090909
BP	GO:0010888	negative regulation of lipid storage	2/115	22/18903	0.007827821	0.052863654	0.039166329	PTPN2/OSBPL8	2	0.909090909
BP	GO:0032069	regulation of nuclease activity	2/115	22/18903	0.007827821	0.052863654	0.039166329	HMGB1/AKT1	2	0.909090909
BP	GO:0046628	positive regulation of insulin receptor signaling pathway	2/115	22/18903	0.007827821	0.052863654	0.039166329	IGF2/OSBPL8	2	0.909090909
BP	GO:0050765	negative regulation of phagocytosis	2/115	22/18903	0.007827821	0.052863654	0.039166329	HMGB1/PLSCR1	2	0.909090909
BP	GO:0090042	tubulin deacetylation	2/115	22/18903	0.007827821	0.052863654	0.039166329	PRKAA1/DR1	2	0.909090909
BP	GO:2000810	regulation of bicellular tight junction assembly	2/115	22/18903	0.007827821	0.052863654	0.039166329	PRKCH/ROCK2	2	0.909090909
BP	GO:0098739	import across plasma membrane	5/115	202/18903	0.007888677	0.053157546	0.039384071	ITGB1/FOLR2/SLC1A4/ATP1A2/AKT1	5	0.247524752
BP	GO:0050863	regulation of T cell activation	7/115	376/18903	0.008154667	0.054829405	0.040622741	HMGB1/TYK2/CEBPB/IGF2/PTPN2/LYN/AKT1	7	0.186170213
BP	GO:1903037	regulation of leukocyte cell-cell adhesion	7/115	377/18903	0.008268169	0.055470908	0.041098026	HMGB1/TYK2/CEBPB/IGF2/PTPN2/LYN/AKT1	7	0.185676393
BP	GO:1903131	mononuclear cell differentiation	8/115	473/18903	0.008329663	0.055761453	0.041313289	HMGB1/CTSK/MYC/CEBPB/ITGB1/PTPN2/LYN/EGR1	8	0.169133192
BP	GO:0043270	positive regulation of ion transport	6/115	289/18903	0.008509945	0.056671062	0.041987212	PDGFRB/CEBPB/ITGB1/GSTO1/AKT1/UCN	6	0.207612457
BP	GO:0034695	response to prostaglandin E	2/115	23/18903	0.008539475	0.056671062	0.041987212	PRKAA1/AKT1	2	0.869565217
BP	GO:0044827	modulation by host of viral genome replication	2/115	23/18903	0.008539475	0.056671062	0.041987212	EIF2AK4/ZBED1	2	0.869565217
BP	GO:0097164	ammonium ion metabolic process	2/115	23/18903	0.008539475	0.056671062	0.041987212	SLC44A1/HNMT	2	0.869565217
BP	GO:0042326	negative regulation of	7/115	381/18903	0.008733877	0.057835998	0.042850305	NF2/GADD45A/PTPN2/IBTK/LYN/PRDX3/AKT1	7	0.183727034

		phosphorylation								
BP	GO:0032722	positive regulation of chemokine production	3/115	70/18903	0.008932619	0.059024591	0.043730926	EIF2AK2/HMGB1/EGR1	3	0.428571429
BP	GO:0043467	regulation of generation of precursor metabolites and energy	4/115	134/18903	0.009107633	0.060051621	0.044491845	PRKAA1/IGF2/SLC25A12/AKT1	4	0.298507463
BP	GO:0051222	positive regulation of protein transport	6/115	294/18903	0.009214714	0.060319621	0.044690405	SH3GLB1/PRKAA1/GLUL/CSNK2A2/SAE1/CLN3	6	0.204081633
BP	GO:0007623	circadian rhythm	5/115	210/18903	0.009239744	0.060319621	0.044690405	PRKAA1/SUV39H2/PIWIL2/EGR1/ROCK2	5	0.238095238
BP	GO:0031396	regulation of protein ubiquitination	5/115	210/18903	0.009239744	0.060319621	0.044690405	FKBP1A/GCLC/MAD2L2/MAGEA2B/AKT1	5	0.238095238
BP	GO:0006123	mitochondrial electron transport	2/115	24/18903	0.009279024	0.060319621	0.044690405	COX5A/COX8A	2	0.833333333
BP	GO:0050855	regulation of B cell receptor signaling pathway	2/115	24/18903	0.009279024	0.060319621	0.044690405	PRKCH/LYN	2	0.833333333
BP	GO:1901863	positive regulation of muscle tissue development	2/115	24/18903	0.009279024	0.060319621	0.044690405	PRKAA1/IGF2	2	0.833333333
BP	GO:2000379	positive regulation of reactive oxygen species metabolic process	3/115	71/18903	0.009285995	0.060319621	0.044690405	GADD45A/NFE2L2/PDGFRB	3	0.422535211
BP	GO:0009612	response to mechanical stimulus	5/115	211/18903	0.009419157	0.061055253	0.04523543	GADD45A/MAPK3/GCLC/ATP1A2/UCN	5	0.236966825
BP	GO:0010565	regulation of cellular ketone metabolic process	4/115	136/18903	0.009581476	0.061971674	0.0459144	SLC7A7/CLN3/EGR1/AKT1	4	0.294117647
BP	GO:0016051	carbohydrate biosynthetic process	5/115	212/18903	0.009600961	0.061971674	0.0459144	IGF2/SLC25A12/PTPN2/NNMT/AKT1	5	0.235849057
BP	GO:0001819	positive regulation of cytokine production	8/115	486/18903	0.009715875	0.062495617	0.046302586	MAPKAPK2/EIF2AK2/HMGB1/TYK2/CEBPB/EGR1/ROCK2/UCN	8	0.164609053
BP	GO:0006644	phospholipid metabolic process	7/115	389/18903	0.009722899	0.062495617	0.046302586	OSBPL5/SLC44A1/PDGFRB/CLN3/PLSCR1/OSBPL8/SMG1	7	0.179948586
BP	GO:0045637	regulation of myeloid cell differentiation	5/115	214/18903	0.0099718	0.063507977	0.047052637	MYC/CEBPB/BRD1/PTPN2/LYN	5	0.23364486
BP	GO:0006541	glutamine metabolic process	2/115	25/18903	0.010046109	0.063507977	0.047052637	GLUL/ASNS	2	0.8
BP	GO:0018195	peptidyl-arginine modification	2/115	25/18903	0.010046109	0.063507977	0.047052637	ART5/PRMT2	2	0.8
BP	GO:0060396	growth hormone receptor signaling pathway	2/115	25/18903	0.010046109	0.063507977	0.047052637	TYK2/LYN	2	0.8

BP	GO:0071378	cellular response to growth hormone stimulus	2/115	25/18903	0.010046109	0.063507977	0.047052637	TYK2/LYN	2	0.8
BP	GO:1900078	positive regulation of cellular response to insulin stimulus	2/115	25/18903	0.010046109	0.063507977	0.047052637	IGF2/OSBPL8	2	0.8
BP	GO:1901984	negative regulation of protein acetylation	2/115	25/18903	0.010046109	0.063507977	0.047052637	CTBP1/MAGEA2B	2	0.8
BP	GO:1902993	positive regulation of amyloid precursor protein catabolic process	2/115	25/18903	0.010046109	0.063507977	0.047052637	LYN/ROCK2	2	0.8
BP	GO:0006801	superoxide metabolic process	3/115	74/18903	0.010394746	0.065041412	0.048188749	NFE2L2/PRDX1/NQO1	3	0.405405405
BP	GO:0030330	DNA damage response	3/115	74/18903	0.010394746	0.065041412	0.048188749	ZMPSTE24/HIPK2/RPS27L	3	0.405405405
BP	GO:0035924	cellular response to vascular endothelial growth factor stimulus	3/115	74/18903	0.010394746	0.065041412	0.048188749	MAPKAPK2/PDGFRB/AKT1	3	0.405405405
BP	GO:0043967	histone H4 acetylation	3/115	74/18903	0.010394746	0.065041412	0.048188749	ZMPSTE24/CTBP1/RUVBL1	3	0.405405405
BP	GO:2000573	positive regulation of DNA biosynthetic process	3/115	74/18903	0.010394746	0.065041412	0.048188749	PDGFRB/MYC/MAPK3	3	0.405405405
BP	GO:0042180	cellular ketone metabolic process	5/115	218/18903	0.010742822	0.065815011	0.048761902	SLC7A7/CLN3/NQO1/EGR1/AKT1	5	0.229357798
BP	GO:0043491	protein kinase B signaling	5/115	218/18903	0.010742822	0.065815011	0.048761902	FGFR1/IGF2/ITGB1/OSBPL8/AKT1	5	0.229357798
BP	GO:0043523	regulation of neuron apoptotic process	5/115	218/18903	0.010742822	0.065815011	0.048761902	HIPK2/CEBPB/CLN3/GCLC/NQO1	5	0.229357798
BP	GO:0032890	regulation of organic acid transport	3/115	75/18903	0.010780652	0.065815011	0.048761902	ITGB1/ATP1A2/AKT1	3	0.4
BP	GO:0046323	glucose import	3/115	75/18903	0.010780652	0.065815011	0.048761902	NFE2L2/OSBPL8/AKT1	3	0.4
BP	GO:0071479	cellular response to ionizing radiation	3/115	75/18903	0.010780652	0.065815011	0.048761902	ZMPSTE24/GADD45A/EGR1	3	0.4
BP	GO:0006986	response to unfolded protein	4/115	141/18903	0.010835517	0.065815011	0.048761902	EIF2AK2/NFE2L2/PTPN2/DERL3	4	0.283687943
BP	GO:0046328	regulation of JNK cascade	4/115	141/18903	0.010835517	0.065815011	0.048761902	GADD45A/HMGB1/HIPK2/MAP3K5	4	0.283687943
BP	GO:0070555	response to interleukin-1	4/115	141/18903	0.010835517	0.065815011	0.048761902	MAPK3/CEBPB/GCLC/EGR1	4	0.283687943
BP	GO:0010882	regulation of cardiac muscle contraction by calcium ion signaling	2/115	26/18903	0.010840372	0.065815011	0.048761902	GSTO1/ATP1A2	2	0.769230769

BP	GO:0032515	negative regulation of phosphoprotein phosphatase activity	2/115	26/18903	0.010840372	0.065815011	0.048761902	FKBP1A/ROCK2	2	0.769230769
BP	GO:0035751	regulation of lysosomal lumen pH	2/115	26/18903	0.010840372	0.065815011	0.048761902	ATP6V1F/CLN3	2	0.769230769
BP	GO:0046426	negative regulation of receptor signaling pathway via JAK-STAT	2/115	26/18903	0.010840372	0.065815011	0.048761902	NF2/PTPN2	2	0.769230769
BP	GO:0050995	negative regulation of lipid catabolic process	2/115	26/18903	0.010840372	0.065815011	0.048761902	PRKAA1/AKT1	2	0.769230769
BP	GO:0060314	regulation of ryanodine-sensitive calcium-release channel activity	2/115	26/18903	0.010840372	0.065815011	0.048761902	FKBP1A/GSTO1	2	0.769230769
BP	GO:0046486	glycerolipid metabolic process	7/115	399/18903	0.011071727	0.067086789	0.049704155	OSBPL5/SLC44A1/PDGFRB/CLN3/PLSCR1/OSBPL8/SMG1	7	0.175438596
BP	GO:0006809	nitric oxide biosynthetic process	3/115	76/18903	0.011174772	0.067577615	0.050067804	NQO1/AKT1/ROCK2	3	0.394736842
BP	GO:0050866	negative regulation of cell activation	5/115	221/18903	0.011347224	0.068460382	0.050721841	HMGB1/CEBPB/GCLC/PTPN2/LYN	5	0.226244344
BP	GO:0045727	positive regulation of translation	4/115	143/18903	0.011365406	0.068460382	0.050721841	EIF2AK4/PIWIL2/RPS27L/UCN	4	0.27972028
BP	GO:0007162	negative regulation of cell adhesion	6/115	308/18903	0.01140947	0.068591047	0.050818649	NF2/HMGB1/CEBPB/PTPN2/MAD2L2/AKT1	6	0.194805195
BP	GO:0071222	cellular response to lipopolysaccharide	5/115	222/18903	0.011553746	0.068757768	0.050942172	HMGB1/MAPK3/CEBPB/LYN/AKT1	5	0.225225225
BP	GO:0010827	regulation of glucose transmembrane transport	3/115	77/18903	0.011577136	0.068757768	0.050942172	NFE2L2/OSBPL8/AKT1	3	0.38961039
BP	GO:0010508	positive regulation of autophagy	4/115	144/18903	0.0116365	0.068757768	0.050942172	SH3GLB1/PRKAA1/HMGB1/MAPK3	4	0.277777778
BP	GO:0050921	positive regulation of chemotaxis	4/115	144/18903	0.0116365	0.068757768	0.050942172	FGFR1/HMGB1/PDGFRB/MAPK3	4	0.277777778
BP	GO:0006760	folic acid-containing compound metabolic process	2/115	27/18903	0.011661461	0.068757768	0.050942172	GART/MTHFD1	2	0.740740741
BP	GO:0009065	glutamine family amino acid catabolic process	2/115	27/18903	0.011661461	0.068757768	0.050942172	GLUL/OAT	2	0.740740741
BP	GO:0032967	positive regulation of collagen biosynthetic process	2/115	27/18903	0.011661461	0.068757768	0.050942172	PDGFRB/UCN	2	0.740740741
BP	GO:0042133	neurotransmitter	2/115	27/18903	0.011661461	0.068757768	0.050942172	CLN3/HNMT	2	0.740740741

		metabolic process								
BP	GO:0048641	regulation of skeletal muscle tissue development	2/115	27/18903	0.011661461	0.068757768	0.050942172	PRKAA1/IGF2	2	0.740740741
BP	GO:0060259	regulation of feeding behavior	2/115	27/18903	0.011661461	0.068757768	0.050942172	EIF2AK4/UCN	2	0.740740741
BP	GO:1904951	positive regulation of establishment of protein localization	6/115	310/18903	0.011750727	0.069151114	0.051233599	SH3GLB1/PRKAA1/GLUL/CSNK2A2/SAE1/CLN3	6	0.193548387
BP	GO:0051783	regulation of nuclear division	4/115	145/18903	0.011911724	0.069964266	0.051836058	PDGFRB/IGF2/PIWIL2/MAD2L2	4	0.275862069
BP	GO:0055117	regulation of cardiac muscle contraction	3/115	78/18903	0.011987772	0.070142193	0.051967883	GSTO1/ATP1A2/UCN	3	0.384615385
BP	GO:0072665	protein localization to vacuole	3/115	78/18903	0.011987772	0.070142193	0.051967883	SH3GLB1/SCARB2/ROCK2	3	0.384615385
BP	GO:0033157	regulation of intracellular protein transport	5/115	225/18903	0.012188674	0.071181854	0.052738161	SH3GLB1/PRKAA1/CSNK2A2/SAE1/DERL3	5	0.222222222
BP	GO:0030099	myeloid cell differentiation	7/115	407/18903	0.012245082	0.071375325	0.052881502	MYC/HIPK2/CEBPB/BRD1/PTPN2/LYN/PRDX3	7	0.171990172
BP	GO:0006305	DNA alkylation	3/115	79/18903	0.012406707	0.071420249	0.052914786	ZMPSTE24/MYC/PIWIL2	3	0.379746835
BP	GO:0006306	DNA methylation	3/115	79/18903	0.012406707	0.071420249	0.052914786	ZMPSTE24/MYC/PIWIL2	3	0.379746835
BP	GO:0072524	pyridine-containing compound metabolic process	3/115	79/18903	0.012406707	0.071420249	0.052914786	NQO1/NNMT/PSAT1	3	0.379746835
BP	GO:0035296	regulation of tube diameter	4/115	147/18903	0.012474649	0.071420249	0.052914786	GCLC/ATP1A2/ROCK2/UCN	4	0.272108844
BP	GO:0097746	blood vessel diameter maintenance	4/115	147/18903	0.012474649	0.071420249	0.052914786	GCLC/ATP1A2/ROCK2/UCN	4	0.272108844
BP	GO:1903038	negative regulation of leukocyte cell-cell adhesion	4/115	147/18903	0.012474649	0.071420249	0.052914786	HMGB1/CEBPB/PTPN2/AKT1	4	0.272108844
BP	GO:0003416	endochondral bone growth	2/115	28/18903	0.012509026	0.071420249	0.052914786	ZMPSTE24/ANXA6	2	0.714285714
BP	GO:0010714	positive regulation of collagen metabolic process	2/115	28/18903	0.012509026	0.071420249	0.052914786	PDGFRB/UCN	2	0.714285714
BP	GO:0010996	response to auditory stimulus	2/115	28/18903	0.012509026	0.071420249	0.052914786	ATP1A2/UCN	2	0.714285714
BP	GO:1903959	regulation of anion transmembrane transport	2/115	28/18903	0.012509026	0.071420249	0.052914786	ITGB1/ATP1A2	2	0.714285714

BP	GO:1904385	cellular response to angiotensin	2/115	28/18903	0.012509026	0.071420249	0.052914786	NFE2L2/ROCK2	2	0.714285714
BP	GO:1903050	regulation of proteolysis involved in protein catabolic process	5/115	227/18903	0.0126249	0.071873849	0.053250855	CSNK2A2/NFE2L2/HIPK2/GCLC/AKT1	5	0.220264317
BP	GO:0070372	regulation of ERK1 and ERK2 cascade	6/115	315/18903	0.012635357	0.071873849	0.053250855	SHC1/HMGB1/PDGFRB/MAPK3/PTPN2/LYN	6	0.19047619
BP	GO:0015748	organophosphate ester transport	4/115	148/18903	0.012762392	0.072328083	0.053587394	OSBPL5/SCARB2/PLSCR1/OSBPL8	4	0.27027027
BP	GO:0035150	regulation of tube size	4/115	148/18903	0.012762392	0.072328083	0.053587394	GCLC/ATP1A2/ROCK2/UCN	4	0.27027027
BP	GO:0008344	adult locomotory behavior	3/115	80/18903	0.012833966	0.072465819	0.053689442	ZMPSTE24/HIPK2/ATP1A2	3	0.375
BP	GO:0071230	cellular response to amino acid stimulus	3/115	80/18903	0.012833966	0.072465819	0.053689442	RRAGD/CEBPB/GCLC	3	0.375
BP	GO:0016236	macroautophagy	6/115	317/18903	0.013001998	0.073279646	0.054292401	ATP6V1E2/SH3GLB1/MAPK3/CLN3/AKT1/SMG1	6	0.189274448
BP	GO:0009411	response to UV	4/115	149/18903	0.01305435	0.0734021	0.054383126	PRKAA1/EIF2AK4/MYC/AKT1	4	0.268456376
BP	GO:0071241	cellular response to inorganic substance	5/115	229/18903	0.013071607	0.0734021	0.054383126	PRKAA1/NFE2L2/MAPK3/NQO1/AKT1	5	0.218340611
BP	GO:0033555	multicellular organismal response to stress	3/115	81/18903	0.013269573	0.073533011	0.054480117	ATP1A2/AKT1/UCN	3	0.37037037
BP	GO:0001890	placenta development	4/115	150/18903	0.013350543	0.073533011	0.054480117	CEBPB/IGF2/PRDX3/AKT1	4	0.266666667
BP	GO:0015740	C4-dicarboxylate transport	2/115	29/18903	0.01338272	0.073533011	0.054480117	SLC25A12/SLC1A4	2	0.689655172
BP	GO:0042104	positive regulation of activated T cell proliferation	2/115	29/18903	0.01338272	0.073533011	0.054480117	HMGB1/IGF2	2	0.689655172
BP	GO:0043247	telomere maintenance in response to DNA damage	2/115	29/18903	0.01338272	0.073533011	0.054480117	MAD2L2/RUVBL1	2	0.689655172
BP	GO:0044030	regulation of DNA methylation	2/115	29/18903	0.01338272	0.073533011	0.054480117	ZMPSTE24/MYC	2	0.689655172
BP	GO:0048265	response to pain	2/115	29/18903	0.01338272	0.073533011	0.054480117	AKT1/UCN	2	0.689655172
BP	GO:0072012	glomerulus vasculature development	2/115	29/18903	0.01338272	0.073533011	0.054480117	PDGFRB/EGR1	2	0.689655172
BP	GO:0090025	regulation of monocyte chemotaxis	2/115	29/18903	0.01338272	0.073533011	0.054480117	HMGB1/LYN	2	0.689655172
BP	GO:0090312	positive regulation of protein deacetylation	2/115	29/18903	0.01338272	0.073533011	0.054480117	CTBP1/NNMT	2	0.689655172
BP	GO:0097421	liver regeneration	2/115	29/18903	0.01338272	0.073533011	0.054480117	CSNK2A2/CEBPB	2	0.689655172
BP	GO:1902175	regulation of oxidative stress-induced intrinsic apoptotic signaling	2/115	29/18903	0.01338272	0.073533011	0.054480117	NFE2L2/AKT1	2	0.689655172

		pathway								
BP	GO:0046718	viral entry into host cell	4/115	151/18903	0.013650993	0.074872887	0.055472822	HMGB1/SCARB2/PLSCR1/ITGB1	4	0.264900662
BP	GO:0042509	regulation of tyrosine phosphorylation of STAT protein	3/115	82/18903	0.01371355	0.074947851	0.055528363	NF2/PTPN2/LYN	3	0.365853659
BP	GO:0046209	nitric oxide metabolic process	3/115	82/18903	0.01371355	0.074947851	0.055528363	NQO1/AKT1/ROCK2	3	0.365853659
BP	GO:2001234	negative regulation of apoptotic signaling pathway	5/115	233/18903	0.013996893	0.076360272	0.056574816	CSNK2A2/NFE2L2/GCLC/RRM2B/AKT1	5	0.214592275
BP	GO:0006094	gluconeogenesis	3/115	83/18903	0.014165919	0.07655459	0.056718785	SLC25A12/PTPN2/NNMT	3	0.361445783
BP	GO:0031507	heterochromatin formation	3/115	83/18903	0.014165919	0.07655459	0.056718785	CTBP1/DOT1L/HMGB1	3	0.361445783
BP	GO:2001057	reactive nitrogen species metabolic process	3/115	83/18903	0.014165919	0.07655459	0.056718785	NQO1/AKT1/ROCK2	3	0.361445783
BP	GO:0040013	negative regulation of locomotion	7/115	419/18903	0.014170319	0.07655459	0.056718785	NF2/GADD45A/HMGB1/NFE2L2/PTPN2/OSBPL8/AKT1	7	0.167064439
BP	GO:0071219	cellular response to molecule of bacterial origin	5/115	234/18903	0.014234931	0.07655459	0.056718785	HMGB1/MAPK3/CEBPB/LYN/AKT1	5	0.213675214
BP	GO:0005979	regulation of glycogen biosynthetic process	2/115	30/18903	0.0142822	0.07655459	0.056718785	IGF2/AKT1	2	0.666666667
BP	GO:0010962	regulation of glucan biosynthetic process	2/115	30/18903	0.0142822	0.07655459	0.056718785	IGF2/AKT1	2	0.666666667
BP	GO:0042744	hydrogen peroxide catabolic process	2/115	30/18903	0.0142822	0.07655459	0.056718785	PRDX1/PRDX3	2	0.666666667
BP	GO:0070498	interleukin-1-mediated signaling pathway	2/115	30/18903	0.0142822	0.07655459	0.056718785	MAPK3/EGR1	2	0.666666667
BP	GO:0071480	cellular response to gamma radiation	2/115	30/18903	0.0142822	0.07655459	0.056718785	ZMPSTE24/EGR1	2	0.666666667
BP	GO:0043524	negative regulation of neuron apoptotic process	4/115	154/18903	0.014578073	0.077587296	0.05748391	HIPK2/CEBPB/CLN3/GCLC	4	0.25974026
BP	GO:0045834	positive regulation of lipid metabolic process	4/115	154/18903	0.014578073	0.077587296	0.05748391	PRKAA1/PDGFRB/LYN/AKT1	4	0.25974026
BP	GO:0051092	positive regulation of NF-kappaB transcription factor activity	4/115	154/18903	0.014578073	0.077587296	0.05748391	PSMA6/EIF2AK2/PRKCH/PRDX3	4	0.25974026
BP	GO:0010921	regulation of phosphatase activity	3/115	84/18903	0.014626698	0.077587296	0.05748391	PDGFRB/FKBP1A/ROCK2	3	0.357142857
BP	GO:0031397	negative regulation of	3/115	84/18903	0.014626698	0.077587296	0.05748391	GCLC/MAD2L2/AKT1	3	0.357142857

BP	GO:0051851	protein ubiquitination modulation by host of symbiont process	3/115	84/18903	0.014626698	0.077587296	0.05748391	EIF2AK4/ZBED1/ROCK2	3	0.357142857
BP	GO:0060537	muscle tissue development	7/115	422/18903	0.014683741	0.077755356	0.057608425	PRKAA1/ZMPSTE24/PDGFRB/IGF2/FKBP1A/ITGB1/EGR1	7	0.165876777
BP	GO:0034764	positive regulation of transmembrane transport	5/115	237/18903	0.014965375	0.079110071	0.058612124	NFE2L2/ITGB1/GSTO1/OSBPL8/AKT1	5	0.210970464
BP	GO:0002507	tolerance induction	2/115	31/18903	0.015207125	0.079159671	0.058648872	HMGB1/LYN	2	0.64516129
BP	GO:0010880	regulation of release of sequestered calcium ion into cytosol by sarcoplasmic reticulum	2/115	31/18903	0.015207125	0.079159671	0.058648872	GSTO1/ATP1A2	2	0.64516129
BP	GO:0033688	regulation of osteoblast proliferation	2/115	31/18903	0.015207125	0.079159671	0.058648872	NF2/EIF2AK2	2	0.64516129
BP	GO:0034694	response to prostaglandin	2/115	31/18903	0.015207125	0.079159671	0.058648872	PRKAA1/AKT1	2	0.64516129
BP	GO:0061437	renal system vasculature development	2/115	31/18903	0.015207125	0.079159671	0.058648872	PDGFRB/EGR1	2	0.64516129
BP	GO:0061440	kidney vasculature development	2/115	31/18903	0.015207125	0.079159671	0.058648872	PDGFRB/EGR1	2	0.64516129
BP	GO:0072525	pyridine-containing compound biosynthetic process	2/115	31/18903	0.015207125	0.079159671	0.058648872	NNMT/PSAT1	2	0.64516129
BP	GO:0098868	bone growth	2/115	31/18903	0.015207125	0.079159671	0.058648872	ZMPSTE24/ANXA6	2	0.64516129
BP	GO:1900745	positive regulation of p38MAPK cascade	2/115	31/18903	0.015207125	0.079159671	0.058648872	GADD45A/MAP3K5	2	0.64516129
BP	GO:0050670	regulation of lymphocyte proliferation	5/115	239/18903	0.015466072	0.080371147	0.059546447	HMGB1/TYK2/CEBPB/IGF2/LYN	5	0.209205021
BP	GO:0090316	positive regulation of intracellular protein transport	4/115	157/18903	0.015544151	0.080520308	0.059656959	SH3GLB1/PRKAA1/CSNK2A2/SAE1	4	0.25477707
BP	GO:0007260	tyrosine phosphorylation of STAT protein	3/115	86/18903	0.015573563	0.080520308	0.059656959	NF2/PTPN2/LYN	3	0.348837209
BP	GO:0019319	hexose biosynthetic process	3/115	86/18903	0.015573563	0.080520308	0.059656959	SLC25A12/PTPN2/NNMT	3	0.348837209
BP	GO:0045088	regulation of innate immune response	5/115	240/18903	0.015720578	0.081007213	0.060017704	HMGB1/NFE2L2/PLSCR1/PTPN2/LYN	5	0.208333333
BP	GO:0045333	cellular respiration	5/115	240/18903	0.015720578	0.081007213	0.060017704	COX5A/COX8A/BLOC1S1/SLC25A12/UCN	5	0.208333333
BP	GO:0044409	entry into host	4/115	158/18903	0.015874929	0.08166532	0.060505291	HMGB1/SCARB2/PLSCR1/ITGB1	4	0.253164557

BP	GO:0001892	embryonic placenta development	3/115	87/18903	0.01605968	0.082016305	0.060765333	CEBPB/IGF2/AKT1	3	0.344827586
BP	GO:0048145	regulation of fibroblast proliferation	3/115	87/18903	0.01605968	0.082016305	0.060765333	ZMPSTE24/PDGFRB/MYC	3	0.344827586
BP	GO:0022412	cellular process involved in reproduction in multicellular organism	7/115	430/18903	0.01611785	0.082016305	0.060765333	TSSK2/AURKC/MAST2/ITGB1/PIWIL2/FOLR2/AKT1	7	0.162790698
BP	GO:0007628	adult walking behavior	2/115	32/18903	0.016157159	0.082016305	0.060765333	ZMPSTE24/HIPK2	2	0.625
BP	GO:0043552	positive regulation of phosphatidylinositol 3-kinase activity	2/115	32/18903	0.016157159	0.082016305	0.060765333	PDGFRB/LYN	2	0.625
BP	GO:0046685	response to arsenic-containing substance	2/115	32/18903	0.016157159	0.082016305	0.060765333	GCLC/GSTO1	2	0.625
BP	GO:0061099	negative regulation of protein tyrosine kinase activity	2/115	32/18903	0.016157159	0.082016305	0.060765333	PTPN2/IBTK	2	0.625
BP	GO:1990776	response to angiotensin	2/115	32/18903	0.016157159	0.082016305	0.060765333	NFE2L2/ROCK2	2	0.625
BP	GO:0035051	cardiocyte differentiation	4/115	159/18903	0.016210112	0.082149096	0.060863717	ZMPSTE24/PDGFRB/MAPK3/ITGB1	4	0.251572327
BP	GO:0032944	regulation of mononuclear cell proliferation	5/115	243/18903	0.016500873	0.083484617	0.061853196	HMGB1/TYK2/CEBPB/IGF2/LYN	5	0.205761317
BP	GO:0006112	energy reserve metabolic process	3/115	88/18903	0.016554274	0.083616809	0.061951136	MYC/IGF2/AKT1	3	0.340909091
BP	GO:0032386	regulation of intracellular transport	6/115	336/18903	0.016865263	0.085047526	0.063011145	SH3GLB1/PRKAA1/CSNK2A2/SAE1/MAPK3/DERL3	6	0.178571429
BP	GO:0071229	cellular response to acid chemical	3/115	89/18903	0.017057357	0.085270465	0.063176319	RRAGD/CEBPB/GCLC	3	0.337078652
BP	GO:0002431	Fc receptor mediated stimulatory signaling pathway	2/115	33/18903	0.017131966	0.085270465	0.063176319	PLSCR1/LYN	2	0.606060606
BP	GO:0007035	vacuolar acidification	2/115	33/18903	0.017131966	0.085270465	0.063176319	ATP6V1F/CLN3	2	0.606060606
BP	GO:0060351	cartilage development involved in endochondral bone morphogenesis	2/115	33/18903	0.017131966	0.085270465	0.063176319	ZMPSTE24/ANXA6	2	0.606060606
BP	GO:0090279	regulation of calcium ion import	2/115	33/18903	0.017131966	0.085270465	0.063176319	PDGFRB/UCN	2	0.606060606
BP	GO:0090659	walking behavior	2/115	33/18903	0.017131966	0.085270465	0.063176319	ZMPSTE24/HIPK2	2	0.606060606
BP	GO:1902253	regulation of intrinsic	2/115	33/18903	0.017131966	0.085270465	0.063176319	MYC/RRM2B	2	0.606060606

		apoptotic signaling pathway by p53 class mediator								
BP	GO:1904893	negative regulation of receptor signaling pathway via STAT	2/115	33/18903	0.017131966	0.085270465	0.063176319	NF2/PTPN2	2	0.606060606
BP	GO:0001933	negative regulation of protein phosphorylation	6/115	338/18903	0.017313353	0.0858944	0.063638588	NF2/GADD45A/PTPN2/IBTK/LYN/AKT1	6	0.177514793
BP	GO:0006790	sulfur compound metabolic process	6/115	338/18903	0.017313353	0.0858944	0.063638588	NFE2L2/GCLC/ADI1/GSTO1/MTHFD1/MGST2	6	0.177514793
BP	GO:0030641	regulation of cellular pH	3/115	90/18903	0.017568942	0.086453536	0.064052848	ATP6V1F/MAPK3/CLN3	3	0.333333333
BP	GO:0045995	regulation of embryonic development	3/115	90/18903	0.017568942	0.086453536	0.064052848	DR1/NFE2L2/RUVBL1	3	0.333333333
BP	GO:0046364	monosaccharide biosynthetic process	3/115	90/18903	0.017568942	0.086453536	0.064052848	SLC25A12/PTPN2/NNMT	3	0.333333333
BP	GO:2000177	regulation of neural precursor cell proliferation	3/115	90/18903	0.017568942	0.086453536	0.064052848	NF2/ITGB1/LYN	3	0.333333333
BP	GO:0035966	response to topologically incorrect protein	4/115	163/18903	0.01759524	0.086453536	0.064052848	EIF2AK2/NFE2L2/PTPN2/DERL3	4	0.245398773
BP	GO:0040029	epigenetic regulation of gene expression	4/115	163/18903	0.01759524	0.086453536	0.064052848	CTBP1/DOT1L/HMGB1/IGF2	4	0.245398773
BP	GO:0006402	mRNA catabolic process	5/115	248/18903	0.017858008	0.0874643	0.064801717	MAPKAPK2/SECISBP2/AKT1/ROCK2/SMG1	5	0.201612903
BP	GO:0048863	stem cell differentiation	5/115	248/18903	0.017858008	0.0874643	0.064801717	EIF2AK2/NFE2L2/MAPK3/ITGB1/ANXA6	5	0.201612903
BP	GO:0055067	monovalent inorganic cation homeostasis	4/115	164/18903	0.017952703	0.087787859	0.065041439	ATP6V1F/MAPK3/CLN3/ATP1A2	4	0.243902439
BP	GO:0070828	heterochromatin organization	3/115	91/18903	0.018089038	0.08782039	0.065065541	CTBP1/DOT1L/HMGB1	3	0.32967033
BP	GO:1901216	positive regulation of neuron death	3/115	91/18903	0.018089038	0.08782039	0.065065541	MAP3K5/NQO1/EGR1	3	0.32967033
BP	GO:0035308	negative regulation of protein dephosphorylation	2/115	34/18903	0.018131216	0.08782039	0.065065541	FKBP1A/ROCK2	2	0.588235294
BP	GO:0042558	pteridine-containing compound metabolic process	2/115	34/18903	0.018131216	0.08782039	0.065065541	GART/MTHFD1	2	0.588235294
BP	GO:0051973	positive regulation of telomerase activity	2/115	34/18903	0.018131216	0.08782039	0.065065541	MYC/MAPK3	2	0.588235294
BP	GO:0055094	response to lipoprotein particle	2/115	34/18903	0.018131216	0.08782039	0.065065541	ITGB1/AKT1	2	0.588235294

BP	GO:0000082	G1/S transition of mitotic cell cycle	5/115	250/18903	0.018420958	0.089083055	0.066001041	MYC/PRMT2/ITGB1/RPS27L/AKT1	5	0.2
BP	GO:0001656	metanephros development	3/115	92/18903	0.018617657	0.089892496	0.066600751	PDGFRB/MYC/EGR1	3	0.326086957
BP	GO:0051250	negative regulation of lymphocyte activation	4/115	166/18903	0.018681156	0.089898121	0.066604918	HMGB1/CEBPB/PTPN2/LYN	4	0.240963855
BP	GO:2001022	positive regulation of response to DNA damage stimulus	4/115	166/18903	0.018681156	0.089898121	0.066604918	HMGB1/MYC/MAD2L2/RUVBL1	4	0.240963855
BP	GO:0050870	positive regulation of T cell activation	5/115	251/18903	0.018706784	0.089898121	0.066604918	HMGB1/TYK2/IGF2/LYN/AKT1	5	0.199203187
BP	GO:0033044	regulation of chromosome organization	5/115	252/18903	0.018995527	0.09035174	0.066941001	MYC/MAPK3/MAD2L2/RUVBL1/SMG1	5	0.198412698
BP	GO:0048762	mesenchymal cell differentiation	5/115	252/18903	0.018995527	0.09035174	0.066941001	FGFR1/MAPK3/ANXA6/MAD2L2/ROCK2	5	0.198412698
BP	GO:0098657	import into cell	5/115	252/18903	0.018995527	0.09035174	0.066941001	ITGB1/FOLR2/SLC1A4/ATP1A2/AKT1	5	0.198412698
BP	GO:0016571	histone methylation	4/115	167/18903	0.019052176	0.09035174	0.066941001	SUV39H2/DOT1L/PRMT2/NNMT	4	0.239520958
BP	GO:0001893	maternal placenta development	2/115	35/18903	0.01915458	0.09035174	0.066941001	PRDX3/AKT1	2	0.571428571
BP	GO:0006691	leukotriene metabolic process	2/115	35/18903	0.01915458	0.09035174	0.066941001	MAPKAPK2/MGST2	2	0.571428571
BP	GO:0014808	release of sequestered calcium ion into cytosol by sarcoplasmic reticulum	2/115	35/18903	0.01915458	0.09035174	0.066941001	GSTO1/ATP1A2	2	0.571428571
BP	GO:0016242	negative regulation of macroautophagy	2/115	35/18903	0.01915458	0.09035174	0.066941001	AKT1/SMG1	2	0.571428571
BP	GO:0043276	anoikis	2/115	35/18903	0.01915458	0.09035174	0.066941001	ITGB1/AKT1	2	0.571428571
BP	GO:0060218	hematopoietic stem cell differentiation	2/115	35/18903	0.01915458	0.09035174	0.066941001	EIF2AK2/NFE2L2	2	0.571428571
BP	GO:1905898	positive regulation of response to endoplasmic reticulum stress	2/115	35/18903	0.01915458	0.09035174	0.066941001	NFE2L2/PTPN2	2	0.571428571
BP	GO:0045814	negative regulation of gene expression	3/115	93/18903	0.019154805	0.09035174	0.066941001	CTBP1/DOT1L/HMGB1	3	0.322580645
BP	GO:0019216	regulation of lipid metabolic process	6/115	347/18903	0.019431581	0.091516477	0.067803947	PRKAA1/ZMPSTE24/PDGFRB/LYN/EGR1/AKT1	6	0.172910663
BP	GO:0070661	leukocyte proliferation	6/115	348/18903	0.019677386	0.092357341	0.068426938	HMGB1/TYK2/MAPK3/CEBPB/IGF2/LYN	6	0.172413793
BP	GO:0032436	positive regulation of proteasomal ubiquitin-dependent	3/115	94/18903	0.019700489	0.092357341	0.068426938	NFE2L2/GCLC/AKT1	3	0.319148936

		protein catabolic process								
BP	GO:0035304	regulation of protein dephosphorylation	3/115	94/18903	0.019700489	0.092357341	0.068426938	PDGFRB/FKBP1A/ROCK2	3	0.319148936
BP	GO:0007568	aging	4/115	169/18903	0.019807876	0.092719003	0.068694891	NFE2L2/PDGFRB/GCLC/NQO1	4	0.236686391
BP	GO:0021537	telencephalon development	5/115	255/18903	0.019879358	0.092770335	0.068732922	NF2/CSNK2A2/SECISBP2/GART/ATP1A2	5	0.196078431
BP	GO:0051402	neuron apoptotic process	5/115	255/18903	0.019879358	0.092770335	0.068732922	HIPK2/CEBPB/CLN3/GCLC/NQO1	5	0.196078431
BP	GO:0045765	regulation of angiogenesis	6/115	349/18903	0.019925309	0.092843459	0.068787099	GLUL/GADD45A/NFE2L2/HIPK2/ITGB1/ROCK2	6	0.171919771
BP	GO:0090257	regulation of muscle system process	5/115	256/18903	0.020179871	0.09314063	0.069007272	ANXA6/GSTO1/ATP1A2/ROCK2/UCN	5	0.1953125
BP	GO:0033687	osteoblast proliferation	2/115	36/18903	0.020201735	0.09314063	0.069007272	NF2/EIF2AK2	2	0.555555556
BP	GO:0070873	regulation of glycogen metabolic process	2/115	36/18903	0.020201735	0.09314063	0.069007272	IGF2/AKT1	2	0.555555556
BP	GO:0090218	positive regulation of lipid kinase activity	2/115	36/18903	0.020201735	0.09314063	0.069007272	PDGFRB/LYN	2	0.555555556
BP	GO:0098810	neurotransmitter reuptake	2/115	36/18903	0.020201735	0.09314063	0.069007272	ITGB1/ATP1A2	2	0.555555556
BP	GO:1903514	release of sequestered calcium ion into cytosol by endoplasmic reticulum	2/115	36/18903	0.020201735	0.09314063	0.069007272	GSTO1/ATP1A2	2	0.555555556
BP	GO:1903580	positive regulation of ATP metabolic process	2/115	36/18903	0.020201735	0.09314063	0.069007272	PRKAA1/SLC25A12	2	0.555555556
BP	GO:0045638	negative regulation of myeloid cell differentiation	3/115	95/18903	0.020254717	0.093244689	0.069084368	MYC/PTPN2/LYN	3	0.315789474
BP	GO:0006936	muscle contraction	6/115	351/18903	0.02042754	0.093899306	0.06956937	ANXA6/GSTO1/ATP1A2/ROCK2/UCN/GAMT	6	0.170940171
BP	GO:1901987	regulation of cell cycle phase transition	7/115	453/18903	0.020792616	0.095405727	0.070685466	EIF2AK4/DOT1L/PRMT2/RPS27L/MAD2L2/RRM2B/AKT1	7	0.154525386
BP	GO:0061097	regulation of protein tyrosine kinase activity	3/115	96/18903	0.020817492	0.095405727	0.070685466	SHC1/PTPN2/IBTK	3	0.3125
BP	GO:0007254	JNK cascade	4/115	172/18903	0.020975785	0.095844646	0.071010659	GADD45A/HMGB1/HIPK2/MAP3K5	4	0.23255814
BP	GO:1905475	regulation of protein localization to membrane	4/115	172/18903	0.020975785	0.095844646	0.071010659	CLN3/ITGB1/PRKCH/AKT1	4	0.23255814
BP	GO:0001662	behavioral fear response	2/115	37/18903	0.021272357	0.096480839	0.07148201	ATP1A2/UCN	2	0.540540541
BP	GO:0006734	NADH metabolic process	2/115	37/18903	0.021272357	0.096480839	0.07148201	SLC25A12/NQO1	2	0.540540541
BP	GO:0010923	negative regulation of	2/115	37/18903	0.021272357	0.096480839	0.07148201	FKBP1A/ROCK2	2	0.540540541

		phosphatase activity								
BP	GO:0071276	cellular response to cadmium ion	2/115	37/18903	0.021272357	0.096480839	0.07148201	MAPK3/AKT1	2	0.540540541
BP	GO:0071402	cellular response to lipoprotein particle stimulus	2/115	37/18903	0.021272357	0.096480839	0.07148201	ITGB1/AKT1	2	0.540540541
BP	GO:0006576	cellular biogenic amine metabolic process	3/115	97/18903	0.021388819	0.096580438	0.071555802	PAOX/NNMT/HNMT	3	0.309278351
BP	GO:0006942	regulation of striated muscle contraction	3/115	97/18903	0.021388819	0.096580438	0.071555802	GSTO1/ATP1A2/UCN	3	0.309278351
BP	GO:0060993	kidney morphogenesis	3/115	97/18903	0.021388819	0.096580438	0.071555802	ZMPSTE24/PDGFRB/MYC	3	0.309278351
BP	GO:1901342	regulation of vasculature development	6/115	355/18903	0.021457757	0.096749239	0.071680866	GLUL/GADD45A/NFE2L2/HIPK2/ITGB1/ROCK2	6	0.169014085
BP	GO:0006937	regulation of muscle contraction	4/115	174/18903	0.021777453	0.097902743	0.072535489	ANXA6/GSTO1/ATP1A2/UCN	4	0.229885057
BP	GO:0031214	biomineral tissue development	4/115	174/18903	0.021777453	0.097902743	0.072535489	ZMPSTE24/CEBPB/ROCK2/SLC24A3	4	0.229885057
BP	GO:0007040	lysosome organization	3/115	98/18903	0.021968699	0.098186632	0.072745821	ATP6V1F/SCARB2/CLN3	3	0.306122449
BP	GO:0032642	regulation of chemokine production	3/115	98/18903	0.021968699	0.098186632	0.072745821	EIF2AK2/HMGB1/EGR1	3	0.306122449
BP	GO:0048661	positive regulation of smooth muscle cell proliferation	3/115	98/18903	0.021968699	0.098186632	0.072745821	PDGFRB/MAP3K5/AKT1	3	0.306122449
BP	GO:0080171	lytic vacuole organization	3/115	98/18903	0.021968699	0.098186632	0.072745821	ATP6V1F/SCARB2/CLN3	3	0.306122449
BP	GO:0002209	behavioral defense response	2/115	38/18903	0.022366128	0.099527648	0.07373937	ATP1A2/UCN	2	0.526315789
BP	GO:0032094	response to food	2/115	38/18903	0.022366128	0.099527648	0.07373937	AKT1/UCN	2	0.526315789
BP	GO:0032885	regulation of polysaccharide biosynthetic process	2/115	38/18903	0.022366128	0.099527648	0.07373937	IGF2/AKT1	2	0.526315789
BP	GO:0006885	regulation of pH	3/115	99/18903	0.022557134	0.099977642	0.074072768	ATP6V1F/MAPK3/CLN3	3	0.303030303
BP	GO:0032602	chemokine production	3/115	99/18903	0.022557134	0.099977642	0.074072768	EIF2AK2/HMGB1/EGR1	3	0.303030303
BP	GO:0032635	interleukin-6 production	4/115	176/18903	0.022597686	0.099977642	0.074072768	MAPKAPK2/HMGB1/CEBPB/UCN	4	0.227272727
BP	GO:0032675	regulation of interleukin-6 production	4/115	176/18903	0.022597686	0.099977642	0.074072768	MAPKAPK2/HMGB1/CEBPB/UCN	4	0.227272727
BP	GO:0036473	cell death in response to oxidative stress	3/115	100/18903	0.023154125	0.102291853	0.075787352	NFE2L2/MAP3K5/AKT1	3	0.3
BP	GO:0032350	regulation of hormone metabolic process	2/115	39/18903	0.023482732	0.103297069	0.076532109	ZMPSTE24/EGR1	2	0.512820513
BP	GO:0042401	cellular biogenic amine	2/115	39/18903	0.023482732	0.103297069	0.076532109	PAOX/NNMT	2	0.512820513

		biosynthetic process								
BP	GO:0045616	regulation of keratinocyte differentiation	2/115	39/18903	0.023482732	0.103297069	0.076532109	PRKCH/ROCK2	2	0.512820513
BP	GO:2001243	negative regulation of intrinsic apoptotic signaling pathway	3/115	101/18903	0.02375967	0.104365544	0.077323735	NFE2L2/RRM2B/AKT1	3	0.297029703
BP	GO:0050867	positive regulation of cell activation	7/115	467/18903	0.024062098	0.105542765	0.07819593	HMGB1/TYK2/PDGFRB/IGF2/LYN/MAD2L2/AKT1	7	0.149892934
BP	GO:0046700	heterocycle catabolic process	7/115	468/18903	0.024308447	0.106175259	0.078664541	MAPKAPK2/SECISBP2/NFE2L2/HNMT/AKT1/ROCK2/SMG1	7	0.14957265
BP	GO:0016575	histone deacetylation	3/115	102/18903	0.024373769	0.106175259	0.078664541	CTBP1/DR1/UCN	3	0.294117647
BP	GO:1901019	regulation of calcium ion transmembrane transporter activity	3/115	102/18903	0.024373769	0.106175259	0.078664541	FKBP1A/GSTO1/ATP1A2	3	0.294117647
BP	GO:0000184	nuclear-transcribed mRNA catabolic process	2/115	40/18903	0.024621856	0.106175259	0.078664541	SECISBP2/SMG1	2	0.5
BP	GO:0000731	DNA synthesis involved in DNA repair	2/115	40/18903	0.024621856	0.106175259	0.078664541	MAD2L2/RRM2B	2	0.5
BP	GO:0009309	amine biosynthetic process	2/115	40/18903	0.024621856	0.106175259	0.078664541	PAOX/NNMT	2	0.5
BP	GO:0010863	positive regulation of phospholipase C activity	2/115	40/18903	0.024621856	0.106175259	0.078664541	FGFR1/PDGFRB	2	0.5
BP	GO:0032965	regulation of collagen biosynthetic process	2/115	40/18903	0.024621856	0.106175259	0.078664541	PDGFRB/UCN	2	0.5
BP	GO:0045823	positive regulation of heart contraction	2/115	40/18903	0.024621856	0.106175259	0.078664541	ATP1A2/UCN	2	0.5
BP	GO:0046627	negative regulation of insulin receptor signaling pathway	2/115	40/18903	0.024621856	0.106175259	0.078664541	PRKAA1/PTPN2	2	0.5
BP	GO:1901532	regulation of hematopoietic progenitor cell differentiation	2/115	40/18903	0.024621856	0.106175259	0.078664541	EIF2AK2/NFE2L2	2	0.5
BP	GO:2000758	positive regulation of peptidyl-lysine acetylation	2/115	40/18903	0.024621856	0.106175259	0.078664541	PRKAA1/PIWIL2	2	0.5
BP	GO:0043409	negative regulation of MAPK cascade	4/115	181/18903	0.024730102	0.106342907	0.078788751	NF2/MYC/PTPN2/LYN	4	0.220994475
BP	GO:0110148	biomineralization	4/115	181/18903	0.024730102	0.106342907	0.078788751	ZMPSTE24/CEBPB/ROCK2/SLC24A3	4	0.220994475
BP	GO:0070167	regulation of biomineral	3/115	103/18903	0.024996419	0.107337564	0.079525685	ZMPSTE24/CEBPB/ROCK2	3	0.291262136

		tissue development								
BP	GO:0030336	negative regulation of cell migration	6/115	368/18903	0.02504825	0.107409697	0.079579128	NF2/GADD45A/HMGB1/NFE2L2/OSBPL8/AKT1	6	0.163043478
BP	GO:0042129	regulation of T cell proliferation	4/115	182/18903	0.0251707	0.107784029	0.079856468	HMGB1/TYK2/CEBPB/IGF2	4	0.21978022
BP	GO:1901988	negative regulation of cell cycle phase transition	5/115	272/18903	0.025398615	0.108608304	0.080467168	EIF2AK4/DOT1L/PRMT2/RPS27L/MAD2L2	5	0.183823529
BP	GO:0044772	mitotic cell cycle phase transition	7/115	473/18903	0.02556636	0.109081574	0.080817811	MYC/PRMT2/ITGB1/RPS27L/MAD2L2/RRM2B/AKT1	7	0.147991543
BP	GO:0001936	regulation of endothelial cell proliferation	4/115	183/18903	0.025616025	0.109081574	0.080817811	FGFR1/HMGB1/IGF2/AKT1	4	0.218579235
BP	GO:0044000	movement in host	4/115	183/18903	0.025616025	0.109081574	0.080817811	HMGB1/SCARB2/PLSCR1/ITGB1	4	0.218579235
BP	GO:0072210	metanephric nephron development	2/115	41/18903	0.025783188	0.109489273	0.081119872	PDGFRB/EGR1	2	0.487804878
BP	GO:1903524	positive regulation of blood circulation	2/115	41/18903	0.025783188	0.109489273	0.081119872	ATP1A2/UCN	2	0.487804878
BP	GO:0044728	DNA methylation or demethylation	3/115	105/18903	0.026267354	0.111083734	0.082301198	ZMPSTE24/MYC/PIWIL2	3	0.285714286
BP	GO:0110149	regulation of biomineralization	3/115	105/18903	0.026267354	0.111083734	0.082301198	ZMPSTE24/CEBPB/ROCK2	3	0.285714286
BP	GO:1901655	cellular response to ketone	3/115	105/18903	0.026267354	0.111083734	0.082301198	PRKAA1/AKT1/ROCK2	3	0.285714286
BP	GO:1903039	positive regulation of leukocyte cell-cell adhesion	5/115	275/18903	0.026464931	0.111765121	0.082806032	HMGB1/TYK2/IGF2/LYN/AKT1	5	0.181818182
BP	GO:0042102	positive regulation of T cell proliferation	3/115	106/18903	0.026915629	0.113162928	0.083841658	HMGB1/TYK2/IGF2	3	0.283018868
BP	GO:1900077	negative regulation of cellular response to insulin stimulus	2/115	42/18903	0.026966423	0.113162928	0.083841658	PRKAA1/PTPN2	2	0.476190476
BP	GO:1900274	regulation of phospholipase C activity	2/115	42/18903	0.026966423	0.113162928	0.083841658	FGFR1/PDGFRB	2	0.476190476
BP	GO:0000075	cell cycle checkpoint signaling	4/115	186/18903	0.026980463	0.113162928	0.083841658	EIF2AK4/DOT1L/RPS27L/MAD2L2	4	0.215053763
BP	GO:0043405	regulation of MAP kinase activity	4/115	186/18903	0.026980463	0.113162928	0.083841658	FGFR1/PDGFRB/MAP3K5/LYN	4	0.215053763
BP	GO:0051051	negative regulation of transport	7/115	479/18903	0.027134098	0.113651839	0.084203889	HMGB1/PLSCR1/GSTO1/ATP1A2/DERL3/AKT1/UCN	7	0.146137787
BP	GO:0014013	regulation of gliogenesis	3/115	107/18903	0.027572434	0.115330262	0.085447422	NF2/PRKCH/LYN	3	0.280373832
BP	GO:0050673	epithelial cell proliferation	7/115	481/18903	0.027670972	0.115584743	0.085635965	GLUL/FGFR1/HMGB1/MYC/CEBPB/IGF2/AKT1	7	0.145530146

BP	GO:1903169	regulation of calcium ion transmembrane transport	4/115	188/18903	0.027913893	0.11644081	0.086270219	FKBP1A/GSTO1/ATP1A2/LYN	4	0.212765957
BP	GO:0016266	O-glycan processing	2/115	43/18903	0.028171255	0.116838024	0.086564512	GALNT11/GALNT1	2	0.465116279
BP	GO:0033574	response to testosterone	2/115	43/18903	0.028171255	0.116838024	0.086564512	NQO1/ROCK2	2	0.465116279
BP	GO:0042596	fear response	2/115	43/18903	0.028171255	0.116838024	0.086564512	ATP1A2/UCN	2	0.465116279
BP	GO:0051602	response to electrical stimulus	2/115	43/18903	0.028171255	0.116838024	0.086564512	BRD1/NQO1	2	0.465116279
BP	GO:0032006	regulation of TOR signaling	3/115	108/18903	0.028237761	0.116838024	0.086564512	PRKAA1/ZMPSTE24/RRAGD	3	0.277777778
BP	GO:0090398	cellular senescence	3/115	108/18903	0.028237761	0.116838024	0.086564512	ZMPSTE24/MAP3K5/MAGEA2B	3	0.277777778
BP	GO:0044843	cell cycle G1/S phase transition	5/115	280/18903	0.028305105	0.116958829	0.086654017	MYC/PRMT2/ITGB1/RPS27L/AKT1	5	0.178571429
BP	GO:0051896	regulation of protein kinase B signaling	4/115	189/18903	0.028387773	0.117142547	0.086790132	FGFR1/IGF2/ITGB1/OSBPL8	4	0.211640212
BP	GO:0061013	regulation of mRNA catabolic process	4/115	190/18903	0.028866441	0.118957671	0.088134945	MAPKAPK2/SECISBP2/AKT1/ROCK2	4	0.210526316
BP	GO:0001504	neurotransmitter uptake	2/115	44/18903	0.029397383	0.120176502	0.089037969	ITGB1/ATP1A2	2	0.454545455
BP	GO:0014075	response to amine	2/115	44/18903	0.029397383	0.120176502	0.089037969	NQO1/RRM2B	2	0.454545455
BP	GO:0032881	regulation of polysaccharide metabolic process	2/115	44/18903	0.029397383	0.120176502	0.089037969	IGF2/AKT1	2	0.454545455
BP	GO:0042771	intrinsic apoptotic signaling pathway in response to DNA damage by p53 class mediator	2/115	44/18903	0.029397383	0.120176502	0.089037969	HIPK2/RPS27L	2	0.454545455
BP	GO:0045840	positive regulation of mitotic nuclear division	2/115	44/18903	0.029397383	0.120176502	0.089037969	PDGFRB/IGF2	2	0.454545455
BP	GO:1901020	negative regulation of calcium ion transmembrane transporter activity	2/115	44/18903	0.029397383	0.120176502	0.089037969	GSTO1/ATP1A2	2	0.454545455
BP	GO:0007173	epidermal growth factor receptor signaling pathway	3/115	110/18903	0.029593948	0.120498066	0.089276213	SHC1/PTPN2/AKT1	3	0.272727273
BP	GO:0019395	fatty acid oxidation	3/115	110/18903	0.029593948	0.120498066	0.089276213	PRKAA1/CPT1B/AKT1	3	0.272727273
BP	GO:2000060	positive regulation of ubiquitin-dependent protein catabolic process	3/115	110/18903	0.029593948	0.120498066	0.089276213	NFE2L2/GCLC/AKT1	3	0.272727273
BP	GO:2000146	negative regulation of	6/115	383/18903	0.029666919	0.120634979	0.089377651	NF2/GADD45A/HMGB1/NFE2L2/OSBPL8/AKT1	6	0.156657963

		cell motility								
BP	GO:0072659	protein localization to plasma membrane	5/115	285/18903	0.03022483	0.122659389	0.090877523	CLN3/ITGB1/PRKCH/AKT1/ROCK2	5	0.175438596
BP	GO:0071347	cellular response to interleukin-1	3/115	111/18903	0.030284787	0.122659389	0.090877523	MAPK3/CEBPB/EGR1	3	0.27027027
BP	GO:1905818	regulation of chromosome separation	3/115	111/18903	0.030284787	0.122659389	0.090877523	CSNK2A2/PLSCR1/MAD2L2	3	0.27027027
BP	GO:0005978	glycogen biosynthetic process	2/115	45/18903	0.030644508	0.122818381	0.090995319	IGF2/AKT1	2	0.444444444
BP	GO:0009250	glucan biosynthetic process	2/115	45/18903	0.030644508	0.122818381	0.090995319	IGF2/AKT1	2	0.444444444
BP	GO:0030574	collagen catabolic process	2/115	45/18903	0.030644508	0.122818381	0.090995319	CTSK/ITGB1	2	0.444444444
BP	GO:0031295	T cell costimulation	2/115	45/18903	0.030644508	0.122818381	0.090995319	LYN/AKT1	2	0.444444444
BP	GO:0033003	regulation of mast cell activation	2/115	45/18903	0.030644508	0.122818381	0.090995319	PLSCR1/LYN	2	0.444444444
BP	GO:0047496	vesicle transport along microtubule	2/115	45/18903	0.030644508	0.122818381	0.090995319	BLOC1S1/CLN3	2	0.444444444
BP	GO:0051955	regulation of amino acid transport	2/115	45/18903	0.030644508	0.122818381	0.090995319	ITGB1/ATP1A2	2	0.444444444
BP	GO:1900744	regulation of p38MAPK cascade	2/115	45/18903	0.030644508	0.122818381	0.090995319	GADD45A/MAP3K5	2	0.444444444
BP	GO:0009060	aerobic respiration	4/115	194/18903	0.030829121	0.123236093	0.0913048	COX5A/COX8A/BLOC1S1/UCN	4	0.206185567
BP	GO:0009749	response to glucose	4/115	194/18903	0.030829121	0.123236093	0.0913048	PRKAA1/GLUL/GCLC/EGR1	4	0.206185567
BP	GO:1904659	glucose transmembrane transport	3/115	113/18903	0.031691904	0.126520022	0.09373784	NFE2L2/OSBPL8/AKT1	3	0.265486726
BP	GO:0046006	regulation of activated T cell proliferation	2/115	46/18903	0.031912334	0.126810243	0.093952863	HMGB1/IGF2	2	0.434782609
BP	GO:1901861	regulation of muscle tissue development	2/115	46/18903	0.031912334	0.126810243	0.093952863	PRKAA1/IGF2	2	0.434782609
BP	GO:2001239	regulation of extrinsic apoptotic signaling pathway in absence of ligand	2/115	46/18903	0.031912334	0.126810243	0.093952863	FGFR1/AKT1	2	0.434782609
BP	GO:0019221	cytokine-mediated signaling pathway	7/115	496/18903	0.031930042	0.126810243	0.093952863	RNF113A/TYK2/MAPK3/PTPN2/LYN/EGR1/AKT1	7	0.141129032
BP	GO:0007626	locomotory behavior	4/115	197/18903	0.032351715	0.128318706	0.095070473	ZMPSTE24/HIPK2/ATP1A2/EGR1	4	0.203045685
BP	GO:0002695	negative regulation of leukocyte activation	4/115	198/18903	0.032868912	0.130003755	0.096318915	HMGB1/CEBPB/PTPN2/LYN	4	0.202020202
BP	GO:0002819	regulation of adaptive immune response	4/115	198/18903	0.032868912	0.130003755	0.096318915	EIF2AK4/HMGB1/TYK2/MAD2L2	4	0.202020202
BP	GO:0006984	ER-nucleus signaling	2/115	47/18903	0.033200568	0.130003755	0.096318915	NFE2L2/PTPN2	2	0.425531915

		pathway								
BP	GO:0008542	visual learning	2/115	47/18903	0.033200568	0.130003755	0.096318915	ITGB1/ATP1A2	2	0.425531915
BP	GO:0031294	lymphocyte costimulation	2/115	47/18903	0.033200568	0.130003755	0.096318915	LYN/AKT1	2	0.425531915
BP	GO:0045646	regulation of erythrocyte differentiation	2/115	47/18903	0.033200568	0.130003755	0.096318915	BRD1/LYN	2	0.425531915
BP	GO:0051972	regulation of telomerase activity	2/115	47/18903	0.033200568	0.130003755	0.096318915	MYC/MAPK3	2	0.425531915
BP	GO:0061647	histone H3-K9 modification	2/115	47/18903	0.033200568	0.130003755	0.096318915	SUV39H2/PIWIL2	2	0.425531915
BP	GO:0070849	response to epidermal growth factor	2/115	47/18903	0.033200568	0.130003755	0.096318915	MAPK3/AKT1	2	0.425531915
BP	GO:1902895	positive regulation of miRNA transcription	2/115	47/18903	0.033200568	0.130003755	0.096318915	MYC/EGR1	2	0.425531915
BP	GO:0001935	endothelial cell proliferation	4/115	199/18903	0.033390949	0.130250189	0.096501496	FGFR1/HMGB1/IGF2/AKT1	4	0.201005025
BP	GO:0009746	response to hexose	4/115	199/18903	0.033390949	0.130250189	0.096501496	PRKAA1/GLUL/GCLC/EGR1	4	0.201005025
BP	GO:0032388	positive regulation of intracellular transport	4/115	199/18903	0.033390949	0.130250189	0.096501496	SH3GLB1/PRKAA1/CSNK2A2/SAE1	4	0.201005025
BP	GO:0051146	striated muscle cell differentiation	5/115	293/18903	0.033464022	0.130369368	0.096589795	ZMPSTE24/PDGFRB/IGF2/ITGB1/AKT1	5	0.170648464
BP	GO:0008645	hexose transmembrane transport	3/115	116/18903	0.033865978	0.131434289	0.097378788	NFE2L2/OSBPL8/AKT1	3	0.25862069
BP	GO:0034440	lipid oxidation	3/115	116/18903	0.033865978	0.131434289	0.097378788	PRKAA1/CPT1B/AKT1	3	0.25862069
BP	GO:0051702	biological process involved in interaction with symbiont	3/115	116/18903	0.033865978	0.131434289	0.097378788	EIF2AK4/ZBED1/ROCK2	3	0.25862069
BP	GO:0043393	regulation of protein binding	4/115	200/18903	0.03391783	0.131469113	0.097404588	MAPK3/HIPK2/FKBP1A/AKT1	4	0.2
BP	GO:0051216	cartilage development	4/115	201/18903	0.034449561	0.132090316	0.097864834	ZMPSTE24/CTSK/MAPK3/ANXA6	4	0.199004975
BP	GO:0006953	acute-phase response	2/115	48/18903	0.034508918	0.132090316	0.097864834	CEBPB/PLSCR1	2	0.416666667
BP	GO:0031057	negative regulation of histone modification	2/115	48/18903	0.034508918	0.132090316	0.097864834	CTBP1/UCN	2	0.416666667
BP	GO:0031641	regulation of myelination	2/115	48/18903	0.034508918	0.132090316	0.097864834	SLC25A12/AKT1	2	0.416666667
BP	GO:0035305	negative regulation of dephosphorylation	2/115	48/18903	0.034508918	0.132090316	0.097864834	FKBP1A/ROCK2	2	0.416666667
BP	GO:0045581	negative regulation of T cell differentiation	2/115	48/18903	0.034508918	0.132090316	0.097864834	HMGB1/PTPN2	2	0.416666667
BP	GO:0045981	positive regulation of nucleotide metabolic	2/115	48/18903	0.034508918	0.132090316	0.097864834	PRKAA1/SLC25A12	2	0.416666667

		process								
BP	GO:0046717	acid secretion	2/115	48/18903	0.034508918	0.132090316	0.097864834	PRKAA1/UCN	2	0.416666667
BP	GO:0048538	thymus development	2/115	48/18903	0.034508918	0.132090316	0.097864834	ZMPSTE24/MAPK3	2	0.416666667
BP	GO:1900544	positive regulation of purine nucleotide metabolic process	2/115	48/18903	0.034508918	0.132090316	0.097864834	PRKAA1/SLC25A12	2	0.416666667
BP	GO:0043406	positive regulation of MAP kinase activity	3/115	117/18903	0.034607519	0.132302559	0.098022083	FGFR1/PDGFRB/MAP3K5	3	0.256410256
BP	GO:0007088	regulation of mitotic nuclear division	3/115	118/18903	0.035357458	0.134833291	0.099897086	PDGFRB/IGF2/MAD2L2	3	0.254237288
BP	GO:1901800	positive regulation of proteasomal protein catabolic process	3/115	118/18903	0.035357458	0.134833291	0.099897086	NFE2L2/GCLC/AKT1	3	0.254237288
BP	GO:0051701	biological process involved in interaction with host	4/115	203/18903	0.035527584	0.135202946	0.100170961	HMGB1/SCARB2/PLSCR1/ITGB1	4	0.197044335
BP	GO:0051251	positive regulation of lymphocyte activation	6/115	400/18903	0.035542588	0.135202946	0.100170961	HMGB1/TYK2/IGF2/LYN/MAD2L2/AKT1	6	0.15
BP	GO:0045933	positive regulation of muscle contraction	2/115	49/18903	0.035837098	0.13581773	0.10062645	GSTO1/UCN	2	0.408163265
BP	GO:0050798	activated T cell proliferation	2/115	49/18903	0.035837098	0.13581773	0.10062645	HMGB1/IGF2	2	0.408163265
BP	GO:0070509	calcium ion import	2/115	49/18903	0.035837098	0.13581773	0.10062645	PDGFRB/UCN	2	0.408163265
BP	GO:0006401	RNA catabolic process	5/115	299/18903	0.036030448	0.136368199	0.101034289	MAPKAPK2/SECISBP2/AKT1/ROCK2/SMG1	5	0.16722408
BP	GO:0031032	actomyosin structure organization	4/115	204/18903	0.036073884	0.136368199	0.101034289	NF2/PDGFRB/ITGB1/ROCK2	4	0.196078431
BP	GO:0015749	monosaccharide transmembrane transport	3/115	119/18903	0.036115779	0.136368199	0.101034289	NFE2L2/OSBPL8/AKT1	3	0.25210084
BP	GO:1901654	response to ketone	4/115	205/18903	0.036625045	0.13812102	0.102332942	PRKAA1/NQO1/AKT1/ROCK2	4	0.195121951
BP	GO:0002821	positive regulation of adaptive immune response	3/115	120/18903	0.036882464	0.138920926	0.102925587	EIF2AK4/TYK2/MAD2L2	3	0.25
BP	GO:0071674	mononuclear cell migration	4/115	206/18903	0.037181072	0.139374898	0.103261932	HMGB1/MAPK3/LYN/AKT1	4	0.194174757
BP	GO:0032964	collagen biosynthetic process	2/115	50/18903	0.037184823	0.139374898	0.103261932	PDGFRB/UCN	2	0.4
BP	GO:0120009	intermembrane lipid transfer	2/115	50/18903	0.037184823	0.139374898	0.103261932	OSBPL5/OSBPL8	2	0.4
BP	GO:2000059	negative regulation of ubiquitin-dependent protein catabolic	2/115	50/18903	0.037184823	0.139374898	0.103261932	CSNK2A2/HIPK2	2	0.4

		process								
BP	GO:0048511	rhythmic process	5/115	302/18903	0.037358122	0.139853484	0.103616513	PRKAA1/SUV39H2/PIWIL2/EGR1/ROCK2	5	0.165562914
BP	GO:0000077	DNA damage checkpoint signaling	3/115	121/18903	0.037657494	0.140459704	0.104065658	EIF2AK4/DOT1L/RPS27L	3	0.247933884
BP	GO:0032355	response to estradiol	3/115	121/18903	0.037657494	0.140459704	0.104065658	PDGFRB/NQO1/UCN	3	0.247933884
BP	GO:2000278	regulation of DNA biosynthetic process	3/115	121/18903	0.037657494	0.140459704	0.104065658	PDGFRB/MYC/MAPK3	3	0.247933884
BP	GO:0034284	response to monosaccharide	4/115	207/18903	0.037741966	0.140603729	0.104172365	PRKAA1/GLUL/GCLC/EGR1	4	0.193236715
BP	GO:0009165	nucleotide biosynthetic process	5/115	304/18903	0.038259794	0.141896574	0.105130225	SLC25A12/GART/MTHFD1/RRM2B/NNMT	5	0.164473684
BP	GO:0006479	protein methylation	4/115	208/18903	0.03830773	0.141896574	0.105130225	SUV39H2/DOT1L/PRMT2/NNMT	4	0.192307692
BP	GO:0008213	protein alkylation	4/115	208/18903	0.03830773	0.141896574	0.105130225	SUV39H2/DOT1L/PRMT2/NNMT	4	0.192307692
BP	GO:0019722	calcium-mediated signaling	4/115	208/18903	0.03830773	0.141896574	0.105130225	PRKAA1/ZMPSTE24/GSTO1/ATP1A2	4	0.192307692
BP	GO:0022904	respiratory electron transport chain	3/115	122/18903	0.038440851	0.141896574	0.105130225	COX5A/COX8A/SLC25A12	3	0.245901639
BP	GO:0071901	negative regulation of protein serine/threonine kinase activity	3/115	122/18903	0.038440851	0.141896574	0.105130225	GADD45A/LYN/AKT1	3	0.245901639
BP	GO:0010665	regulation of cardiac muscle cell apoptotic process	2/115	51/18903	0.038551809	0.141896574	0.105130225	NFE2L2/MAP3K5	2	0.392156863
BP	GO:0044088	regulation of vacuole organization	2/115	51/18903	0.038551809	0.141896574	0.105130225	SH3GLB1/SCARB2	2	0.392156863
BP	GO:1901985	positive regulation of protein acetylation	2/115	51/18903	0.038551809	0.141896574	0.105130225	PRKAA1/PIWIL2	2	0.392156863
BP	GO:1903793	positive regulation of anion transport	2/115	51/18903	0.038551809	0.141896574	0.105130225	CEBPB/ITGB1	2	0.392156863
BP	GO:0046651	lymphocyte proliferation	5/115	305/18903	0.03871561	0.14232861	0.105450318	HMGB1/TYK2/CEBPB/IGF2/LYN	5	0.163934426
BP	GO:1901293	nucleoside phosphate biosynthetic process	5/115	306/18903	0.039174752	0.143844058	0.106573103	SLC25A12/GART/MTHFD1/RRM2B/NNMT	5	0.163398693
BP	GO:0002224	toll-like receptor signaling pathway	3/115	123/18903	0.039232514	0.143883838	0.106602576	MAPKAPK2/HMGB1/LYN	3	0.243902439
BP	GO:0010823	negative regulation of mitochondrion organization	2/115	52/18903	0.039937777	0.145426632	0.107745622	GCLC/AKT1	2	0.384615385
BP	GO:0046622	positive regulation of organ growth	2/115	52/18903	0.039937777	0.145426632	0.107745622	IGF2/AKT1	2	0.384615385
BP	GO:0051452	intracellular pH reduction	2/115	52/18903	0.039937777	0.145426632	0.107745622	ATP6V1F/CLN3	2	0.384615385

BP	GO:0051496	positive regulation of stress fiber assembly	2/115	52/18903	0.039937777	0.145426632	0.107745622	NF2/ROCK2	2	0.384615385
BP	GO:0061462	protein localization to lysosome	2/115	52/18903	0.039937777	0.145426632	0.107745622	SCARB2/ROCK2	2	0.384615385
BP	GO:0086009	membrane repolarization	2/115	52/18903	0.039937777	0.145426632	0.107745622	ZMPSTE24/ATP1A2	2	0.384615385
BP	GO:0055007	cardiac muscle cell differentiation	3/115	124/18903	0.040032464	0.145598497	0.107872955	ZMPSTE24/PDGFRB/ITGB1	3	0.241935484
BP	GO:1903706	regulation of hemopoiesis	6/115	413/18903	0.040510564	0.147035905	0.108937922	HMGB1/MYC/CEBPB/BRD1/PTPN2/LYN	6	0.14527845
BP	GO:0001822	kidney development	5/115	309/18903	0.040572169	0.147035905	0.108937922	ZMPSTE24/PDGFRB/MYC/RRM2B/EGR1	5	0.161812298
BP	GO:0006282	regulation of DNA repair	4/115	212/18903	0.040619508	0.147035905	0.108937922	HMGB1/SUPT3H/MAD2L2/RUVBL1	4	0.188679245
BP	GO:0042098	T cell proliferation	4/115	212/18903	0.040619508	0.147035905	0.108937922	HMGB1/TYK2/CEBPB/IGF2	4	0.188679245
BP	GO:0050678	regulation of epithelial cell proliferation	6/115	414/18903	0.04091008	0.147913095	0.109587825	GLUL/FGFR1/HMGB1/MYC/IGF2/AKT1	6	0.144927536
BP	GO:0042063	gliogenesis	5/115	310/18903	0.041044651	0.148079383	0.109711027	NF2/MAPK3/PRKCH/LYN/AKT1	5	0.161290323
BP	GO:0007033	vacuole organization	4/115	213/18903	0.041209639	0.148079383	0.109711027	SH3GLB1/ATP6V1F/SCARB2/CLN3	4	0.187793427
BP	GO:0006111	regulation of gluconeogenesis	2/115	53/18903	0.04134245	0.148079383	0.109711027	PTPN2/NNMT	2	0.377358491
BP	GO:0007632	visual behavior	2/115	53/18903	0.04134245	0.148079383	0.109711027	ITGB1/ATP1A2	2	0.377358491
BP	GO:0009409	response to cold	2/115	53/18903	0.04134245	0.148079383	0.109711027	PRKAA1/EIF2AK4	2	0.377358491
BP	GO:0010662	regulation of striated muscle cell apoptotic process	2/115	53/18903	0.04134245	0.148079383	0.109711027	NFE2L2/MAP3K5	2	0.377358491
BP	GO:0015804	neutral amino acid transport	2/115	53/18903	0.04134245	0.148079383	0.109711027	SLC25A12/SLC1A4	2	0.377358491
BP	GO:0048146	positive regulation of fibroblast proliferation	2/115	53/18903	0.04134245	0.148079383	0.109711027	PDGFRB/MYC	2	0.377358491
BP	GO:0038127	ERBB signaling pathway	3/115	126/18903	0.041657134	0.149032409	0.110417117	SHC1/PTPN2/AKT1	3	0.238095238
BP	GO:0008016	regulation of heart contraction	4/115	214/18903	0.041804645	0.149211924	0.110550119	ZMPSTE24/GSTO1/ATP1A2/UCN	4	0.186915888
BP	GO:0046474	glycerophospholipid biosynthetic process	4/115	214/18903	0.041804645	0.149211924	0.110550119	SLC44A1/CLN3/PLSCR1/SMG1	4	0.186915888
BP	GO:0032943	mononuclear cell proliferation	5/115	312/18903	0.041999653	0.149733648	0.110936661	HMGB1/TYK2/CEBPB/IGF2/LYN	5	0.16025641
BP	GO:0002688	regulation of leukocyte chemotaxis	3/115	127/18903	0.042481811	0.151276695	0.112079894	HMGB1/MAPK3/LYN	3	0.236220472
BP	GO:0010883	regulation of lipid storage	2/115	54/18903	0.042765554	0.152110426	0.1126976	PTPN2/OSBPL8	2	0.37037037
BP	GO:0006909	phagocytosis	5/115	314/18903	0.042968059	0.152476932	0.112969142	HMGB1/CLN3/PLSCR1/ITGB1/LYN	5	0.159235669

BP	GO:0019932	second-messenger-mediated signaling	5/115	314/18903	0.042968059	0.152476932	0.112969142	PRKAA1/ZMPSTE24/GSTO1/ATP1A2/UCN	5	0.159235669
BP	GO:0006304	DNA modification	3/115	128/18903	0.043314685	0.152998647	0.113355677	ZMPSTE24/MYC/PIWIL2	3	0.234375
BP	GO:0045471	response to ethanol	3/115	128/18903	0.043314685	0.152998647	0.113355677	PRKAA1/CTSK/NQO1	3	0.234375
BP	GO:0045739	positive regulation of DNA repair	3/115	128/18903	0.043314685	0.152998647	0.113355677	HMGB1/MAD2L2/RUVBL1	3	0.234375
BP	GO:0050868	negative regulation of T cell activation	3/115	128/18903	0.043314685	0.152998647	0.113355677	HMGB1/CEBPB/PTPN2	3	0.234375
BP	GO:0031570	DNA integrity checkpoint signaling	3/115	129/18903	0.044155732	0.154464325	0.114441588	EIF2AK4/DOT1L/RPS27L	3	0.23255814
BP	GO:0045931	positive regulation of mitotic cell cycle	3/115	129/18903	0.044155732	0.154464325	0.114441588	ASNS/RRM2B/AKT1	3	0.23255814
BP	GO:0051928	positive regulation of calcium ion transport	3/115	129/18903	0.044155732	0.154464325	0.114441588	PDGFRB/GSTO1/UCN	3	0.23255814
BP	GO:0003229	ventricular cardiac muscle tissue development	2/115	55/18903	0.044206816	0.154464325	0.114441588	ZMPSTE24/FKBP1A	2	0.363636364
BP	GO:0010518	positive regulation of phospholipase activity	2/115	55/18903	0.044206816	0.154464325	0.114441588	FGFR1/PDGFRB	2	0.363636364
BP	GO:0010659	cardiac muscle cell apoptotic process	2/115	55/18903	0.044206816	0.154464325	0.114441588	NFE2L2/MAP3K5	2	0.363636364
BP	GO:0042743	hydrogen peroxide metabolic process	2/115	55/18903	0.044206816	0.154464325	0.114441588	PRDX1/PRDX3	2	0.363636364
BP	GO:1903202	negative regulation of oxidative stress-induced cell death	2/115	55/18903	0.044206816	0.154464325	0.114441588	NFE2L2/AKT1	2	0.363636364
BP	GO:2000179	positive regulation of neural precursor cell proliferation	2/115	55/18903	0.044206816	0.154464325	0.114441588	ITGB1/LYN	2	0.363636364
BP	GO:0002573	myeloid leukocyte differentiation	4/115	218/18903	0.044233424	0.154464325	0.114441588	MYC/CEBPB/PTPN2/LYN	4	0.183486239
BP	GO:1902105	regulation of leukocyte differentiation	5/115	317/18903	0.044445853	0.155029563	0.114860369	HMGB1/MYC/CEBPB/PTPN2/LYN	5	0.157728707
BP	GO:0001505	regulation of neurotransmitter levels	4/115	219/18903	0.044852803	0.156238001	0.115755693	CLN3/ITGB1/ATP1A2/HNMT	4	0.182648402
BP	GO:0010948	negative regulation of cell cycle process	5/115	318/18903	0.044945178	0.156238001	0.115755693	EIF2AK4/DOT1L/PRMT2/RPS27L/MAD2L2	5	0.157232704
BP	GO:0072001	renal system development	5/115	318/18903	0.044945178	0.156238001	0.115755693	ZMPSTE24/PDGFRB/MYC/RRM2B/EGR1	5	0.157232704
BP	GO:0034968	histone lysine methylation	3/115	130/18903	0.045004927	0.156268525	0.115778308	SUV39H2/DOT1L/NNMT	3	0.230769231
BP	GO:0060326	cell chemotaxis	5/115	319/18903	0.045447871	0.157628025	0.116785552	FGFR1/HMGB1/PDGFRB/MAPK3/LYN	5	0.156739812

BP	GO:0043551	regulation of phosphatidylinositol 3-kinase activity	2/115	56/18903	0.045665967	0.157671007	0.116817397	PDGFRB/LYN	2	0.357142857
BP	GO:0051438	regulation of ubiquitin-protein transferase activity	2/115	56/18903	0.045665967	0.157671007	0.116817397	MAD2L2/MAGEA2B	2	0.357142857
BP	GO:1903170	negative regulation of calcium ion transmembrane transport	2/115	56/18903	0.045665967	0.157671007	0.116817397	GSTO1/ATP1A2	2	0.357142857
BP	GO:2000630	positive regulation of miRNA metabolic process	2/115	56/18903	0.045665967	0.157671007	0.116817397	MYC/EGR1	2	0.357142857
BP	GO:0034219	carbohydrate transmembrane transport	3/115	132/18903	0.046727662	0.160974169	0.119264688	NFE2L2/OSBPL8/AKT1	3	0.227272727
BP	GO:0051983	regulation of chromosome segregation	3/115	132/18903	0.046727662	0.160974169	0.119264688	CSNK2A2/PLSCR1/MAD2L2	3	0.227272727
BP	GO:0022409	positive regulation of cell-cell adhesion	5/115	322/18903	0.04697618	0.161316565	0.119518367	HMGB1/TYK2/IGF2/LYN/AKT1	5	0.155279503
BP	GO:0010658	striated muscle cell apoptotic process	2/115	57/18903	0.04714274	0.161316565	0.119518367	NFE2L2/MAP3K5	2	0.350877193
BP	GO:0043388	positive regulation of DNA binding	2/115	57/18903	0.04714274	0.161316565	0.119518367	HMGB1/HIPK2	2	0.350877193
BP	GO:0045071	negative regulation of viral genome replication	2/115	57/18903	0.04714274	0.161316565	0.119518367	EIF2AK2/PLSCR1	2	0.350877193
BP	GO:0050879	multicellular organismal movement	2/115	57/18903	0.04714274	0.161316565	0.119518367	HIPK2/GSTO1	2	0.350877193
BP	GO:0050881	musculoskeletal movement	2/115	57/18903	0.04714274	0.161316565	0.119518367	HIPK2/GSTO1	2	0.350877193
BP	GO:0070374	positive regulation of ERK1 and ERK2 cascade	4/115	223/18903	0.047379021	0.161944346	0.119983486	SHC1/HMGB1/PDGFRB/MAPK3	4	0.179372197
BP	GO:0002244	hematopoietic progenitor cell differentiation	3/115	133/18903	0.047601149	0.162161246	0.120144185	EIF2AK2/NFE2L2/LYN	3	0.22556391
BP	GO:0030168	platelet activation	3/115	133/18903	0.047601149	0.162161246	0.120144185	PLSCR1/CLIC1/LYN	3	0.22556391
BP	GO:0035303	regulation of dephosphorylation	3/115	133/18903	0.047601149	0.162161246	0.120144185	PDGFRB/FKBP1A/ROCK2	3	0.22556391
BP	GO:0006605	protein targeting	5/115	325/18903	0.048534875	0.164890905	0.122166572	SH3GLB1/PRKAA1/CSNK2A2/SAE1/SCARB2	5	0.153846154
BP	GO:0045620	negative regulation of	2/115	58/18903	0.048636872	0.164890905	0.122166572	HMGB1/PTPN2	2	0.344827586

		lymphocyte differentiation								
BP	GO:0048008	platelet-derived growth factor receptor signaling pathway	2/115	58/18903	0.048636872	0.164890905	0.122166572	PDGFRB/PTPN2	2	0.344827586
BP	GO:0060350	endochondral bone morphogenesis	2/115	58/18903	0.048636872	0.164890905	0.122166572	ZMPSTE24/ANXA6	2	0.344827586
BP	GO:0007596	blood coagulation	4/115	225/18903	0.048671321	0.164890905	0.122166572	NFE2L2/PLSCR1/CLIC1/LYN	4	0.177777778
BP	GO:0051304	chromosome separation	3/115	135/18903	0.049372227	0.166896634	0.123652603	CSNK2A2/PLSCR1/MAD2L2	3	0.222222222
BP	GO:1900180	regulation of protein localization to nucleus	3/115	135/18903	0.049372227	0.166896634	0.123652603	NF2/GLUL/AKT1	3	0.222222222
BP	GO:0048705	skeletal system morphogenesis	4/115	227/18903	0.049983053	0.168775374	0.125044549	ZMPSTE24/FGFR1/ANXA6/MTHFD1	4	0.176211454
CC	GO:0017053	transcription repressor complex	4/115	77/19869	0.001040987	0.108627608	0.092526399	CTBP1/DR1/HMGB1/MYC	4	0.519480519
CC	GO:0000123	histone acetyltransferase complex	4/115	91/19869	0.001932406	0.108627608	0.092526399	DR1/SUPT3H/BRD1/RUVBL1	4	0.43956044
CC	GO:0033391	chromatoid body	2/115	12/19869	0.002110328	0.108627608	0.092526399	PIWIL2/SMG1	2	1.666666667
CC	GO:0031248	protein acetyltransferase complex	4/115	101/19869	0.002824517	0.108627608	0.092526399	DR1/SUPT3H/BRD1/RUVBL1	4	0.396039604
CC	GO:1902493	acetyltransferase complex	4/115	101/19869	0.002824517	0.108627608	0.092526399	DR1/SUPT3H/BRD1/RUVBL1	4	0.396039604
CC	GO:0022626	cytosolic ribosome	4/115	105/19869	0.003249066	0.108627608	0.092526399	RPL39L/RPL18A/EIF2AK4/RPS27L	4	0.380952381
CC	GO:0090575	RNA polymerase II transcription regulator complex	6/115	249/19869	0.003293511	0.108627608	0.092526399	DR1/NFE2L2/SUPT3H/MYC/HIPK2/CEBPB	6	0.240963855
CC	GO:0090571	RNA polymerase II transcription repressor complex	2/115	15/19869	0.003319476	0.108627608	0.092526399	DR1/MYC	2	1.333333333
CC	GO:0042470	melanosome	4/115	110/19869	0.003838431	0.108627608	0.092526399	ITGB1/ANXA6/SLC1A4/PRDX1	4	0.363636364
CC	GO:0048770	pigment granule	4/115	110/19869	0.003838431	0.108627608	0.092526399	ITGB1/ANXA6/SLC1A4/PRDX1	4	0.363636364
CC	GO:0033178	proton-transporting two-sector ATPase complex	2/115	19/19869	0.005324992	0.136997511	0.116691203	ATP6V1E2/ATP6V1F	2	1.052631579
CC	GO:0005844	polysome	3/115	66/19869	0.006632363	0.156413216	0.133229037	PSMA6/RPL18A/EIF2AK4	3	0.454545455
CC	GO:0005751	mitochondrial respiratory chain complex IV	2/115	25/19869	0.009133349	0.188807248	0.160821498	COX5A/COX8A	2	0.8
CC	GO:0005840	ribosome	5/115	232/19869	0.011303368	0.188807248	0.160821498	RPL39L/RPL18A/EIF2AK2/EIF2AK4/RPS27L	5	0.215517241
CC	GO:0045277	respiratory chain complex IV	2/115	28/19869	0.011378986	0.188807248	0.160821498	COX5A/COX8A	2	0.714285714
CC	GO:0045121	membrane raft	6/115	326/19869	0.011789939	0.188807248	0.160821498	MAPK3/CLN3/PLSCR1/ITGB1/ATP1A2/LYN	6	0.18404908

CC	GO:0098857	membrane microdomain	6/115	327/19869	0.011955143	0.188807248	0.160821498	MAPK3/CLN3/PLSCR1/ITGB1/ATP1A2/LYN	6	0.183486239
CC	GO:0005901	caveola	3/115	82/19869	0.012008942	0.188807248	0.160821498	MAPK3/CLN3/ATP1A2	3	0.365853659
CC	GO:0008305	integrin complex	2/115	31/19869	0.01384122	0.206161335	0.175603294	ITGB1/LYN	2	0.64516129
CC	GO:0036464	cytoplasmic ribonucleoprotein granule	5/115	248/19869	0.0147206	0.208296496	0.177421974	PSMA6/HIPK2/PIWIL2/ROCK2/SMG1	5	0.201612903
CC	GO:0005774	vacuolar membrane	7/115	461/19869	0.01778161	0.226074502	0.192564854	SH3GLB1/ATP6V1F/BLOC1S1/SCARB2/CLN3/ANXA6/LYN	7	0.151843818
CC	GO:0035770	ribonucleoprotein granule	5/115	265/19869	0.019046927	0.226074502	0.192564854	PSMA6/HIPK2/PIWIL2/ROCK2/SMG1	5	0.188679245
CC	GO:0043202	lysosomal lumen	3/115	98/19869	0.019301736	0.226074502	0.192564854	CTSK/PDGFRB/SCARB2	3	0.306122449
CC	GO:0097386	glial cell projection	2/115	38/19869	0.020384123	0.226074502	0.192564854	GLUL/ITGB1	2	0.526315789
CC	GO:0070461	SAGA-type complex	2/115	39/19869	0.021405803	0.226074502	0.192564854	DR1/SUPT3H	2	0.512820513
CC	GO:0005759	mitochondrial matrix	7/115	483/19869	0.022291759	0.226074502	0.192564854	SHC1/BLOC1S1/HMGCL/BCAT2/GCSH/OAT/PRDX3	7	0.144927536
CC	GO:0016605	PML body	3/115	105/19869	0.02311174	0.226074502	0.192564854	HIPK2/ZBED1/MAGEA2B	3	0.285714286
CC	GO:0005635	nuclear envelope	7/115	488/19869	0.023418268	0.226074502	0.192564854	ZMPSTE24/SENP1/MAPK3/CLIC1/ZBED1/OSBPL8/MGST2	7	0.143442623
CC	GO:0070069	cytochrome complex	2/115	42/19869	0.024595224	0.226074502	0.192564854	COX5A/COX8A	2	0.476190476
CC	GO:0043025	neuronal cell body	7/115	497/19869	0.0255443	0.226074502	0.192564854	PRKAA1/GLUL/BRD1/SLC1A4/ATP1A2/NQO1/UCN	7	0.14084507
CC	GO:0044853	plasma membrane raft	3/115	113/19869	0.027930241	0.226074502	0.192564854	MAPK3/CLN3/ATP1A2	3	0.265486726
CC	GO:0061695	transferase complex	5/115	295/19869	0.028574413	0.226074502	0.192564854	PRKAA1/CSNK2A2/SUPT3H/MAP3K5/MAD2L2	5	0.169491525
CC	GO:0043209	myelin sheath	2/115	47/19869	0.03030951	0.226074502	0.192564854	GLUL/ITGB1	2	0.425531915
CC	GO:0098562	cytoplasmic side of membrane	4/115	204/19869	0.030892577	0.226074502	0.192564854	BLOC1S1/FKBP1A/LYN/AKT1	4	0.196078431
CC	GO:1905368	peptidase complex	3/115	118/19869	0.031192435	0.226074502	0.192564854	PSMA6/PSMD12/SUPT3H	3	0.254237288
CC	GO:0031965	nuclear membrane	5/115	305/19869	0.032317538	0.226074502	0.192564854	ZMPSTE24/SENP1/CLIC1/ZBED1/OSBPL8	5	0.163934426
CC	GO:0005769	early endosome	6/115	414/19869	0.033367073	0.226074502	0.192564854	NF2/BLOC1S1/MAPK3/CLN3/PRDX3/AOC3	6	0.144927536
CC	GO:0005782	peroxisomal matrix	2/115	50/19869	0.03396579	0.226074502	0.192564854	PAOX/HMGCL	2	0.4
CC	GO:0014704	intercalated disc	2/115	50/19869	0.03396579	0.226074502	0.192564854	ITGB1/ATP1A2	2	0.4
CC	GO:0016469	proton-transporting two-sector ATPase complex	2/115	50/19869	0.03396579	0.226074502	0.192564854	ATP6V1E2/ATP6V1F	2	0.4
CC	GO:0031907	microbody lumen	2/115	50/19869	0.03396579	0.226074502	0.192564854	PAOX/HMGCL	2	0.4
CC	GO:0005765	lysosomal membrane	6/115	418/19869	0.034721767	0.226074502	0.192564854	ATP6V1F/BLOC1S1/SCARB2/CLN3/ANXA6/LYN	6	0.14354067
CC	GO:0098852	lytic vacuole membrane	6/115	418/19869	0.034721767	0.226074502	0.192564854	ATP6V1F/BLOC1S1/SCARB2/CLN3/ANXA6/LYN	6	0.14354067
CC	GO:0005925	focal adhesion	6/115	422/19869	0.036111668	0.226074502	0.192564854	SENP1/PDGFRB/SCARB2/MAPK3/ITGB1/ANXA6	6	0.142180095
CC	GO:0098636	protein complex involved in cell adhesion	2/115	52/19869	0.036493978	0.226074502	0.192564854	ITGB1/LYN	2	0.384615385
CC	GO:0016363	nuclear matrix	3/115	126/19869	0.036809645	0.226074502	0.192564854	PSMA6/CEBPB/RUVBL1	3	0.238095238
CC	GO:0140534	endoplasmic reticulum protein-containing complex	3/115	127/19869	0.037545942	0.226074502	0.192564854	FKBP1A/MAP3K5/DERL3	3	0.236220472

CC	GO:0032154	cleavage furrow	2/115	54/19869	0.039092408	0.229530795	0.195508841	NF2/ITGB1	2	0.37037037
CC	GO:0030055	cell-substrate junction	6/115	432/19869	0.039742081	0.229530795	0.195508841	SENP1/PDGFRB/SCARB2/MAPK3/ITGB1/ANXA6	6	0.138888889
CC	GO:0001669	acrosomal vesicle	3/115	133/19869	0.042121378	0.233732354	0.199087629	TSSK2/ATP6V1E2/CSNK2A2	3	0.22556391
CC	GO:0005793	endoplasmic reticulum-Golgi intermediate compartment	3/115	133/19869	0.042121378	0.233732354	0.199087629	HMGB1/PTPN2/GALNT1	3	0.22556391
CC	GO:1902911	protein kinase complex	3/115	136/19869	0.044509606	0.240462046	0.20481982	PRKAA1/CSNK2A2/MAP3K5	3	0.220588235
CC	GO:0000502	proteasome complex	2/115	59/19869	0.045883217	0.240462046	0.20481982	PSMA6/PSMD12	2	0.338983051
CC	GO:0022625	cytosolic large ribosomal subunit	2/115	59/19869	0.045883217	0.240462046	0.20481982	RPL39L/RPL18A	2	0.338983051
MF	GO:0106310	protein serine kinase activity	19/115	363/18432	1.06E-12	4.38E-10	3.35E-10	TSSK2/PRKAA1/DYRK4/MAPKAPK2/CSNK2A2/EIF2AK2/EIF2AK4/AURKC/MAST2/TESK2/MAPK3/HIPK2/MAP3K5/PRKCH/MKNK1/CLK1/AKT1/ROCK2/SMG1	19	0.523415978
MF	GO:0004674	protein serine/threonine kinase activity	19/115	430/18432	2.04E-11	4.21E-09	3.23E-09	TSSK2/PRKAA1/DYRK4/MAPKAPK2/CSNK2A2/EIF2AK2/EIF2AK4/AURKC/MAST2/TESK2/MAPK3/HIPK2/MAP3K5/PRKCH/MKNK1/CLK1/AKT1/ROCK2/SMG1	19	0.441860465
MF	GO:0004713	protein tyrosine kinase activity	9/115	138/18432	2.04E-07	2.81E-05	2.15E-05	DYRK4/EIF2AK2/FGFR1/TYK2/PDGFRB/TESK2/HIPK2/LYN/CLK1	9	0.652173913
MF	GO:0004712	protein serine/threonine/tyrosine kinase activity	6/115	45/18432	3.45E-07	3.56E-05	2.73E-05	DYRK4/AURKC/TESK2/MAPK3/CLK1/AKT1	6	1.333333333
MF	GO:0072341	modified amino acid binding	7/115	94/18432	2.00E-06	0.000165043	0.000126502	OSBPL5/HMGB1/SCARB2/ANXA6/FOLR2/OSBPL8/MGST2	7	0.744680851
MF	GO:0016879	ligase activity	5/115	48/18432	1.20E-05	0.000822215	0.000630211	GLUL/ASNS/GCLC/GART/MTHFD1	5	1.041666667
MF	GO:0001786	phosphatidylserine binding	5/115	61/18432	3.90E-05	0.002295598	0.001759529	OSBPL5/HMGB1/SCARB2/ANXA6/OSBPL8	5	0.819672131
MF	GO:0016741	transferase activity	8/115	225/18432	8.40E-05	0.004324942	0.003314979	SUV39H2/DOT1L/PRMT2/GCSH/GART/NNMT/HNMT/GAMT	8	0.355555556
MF	GO:0004715	non-membrane spanning protein tyrosine kinase activity	4/115	45/18432	0.000175868	0.007226372	0.005538865	EIF2AK2/TYK2/LYN/CLK1	4	0.888888889
MF	GO:0016209	antioxidant activity	5/115	85/18432	0.000190948	0.007226372	0.005538865	GSTO1/PRDX1/PRDX3/NQO1/MGST2	5	0.588235294
MF	GO:0140297	DNA-binding transcription factor binding	11/115	476/18432	0.000192937	0.007226372	0.005538865	PSMA6/CTBP1/HMGB1/NFE2L2/MYC/PRMT2/HIPK2/CEBPB/PTPN2/MAD2L2/MAGEA2B	11	0.231092437
MF	GO:0015485	cholesterol binding	4/115	51/18432	0.000286605	0.009057608	0.006942469	OSBPL5/SCARB2/ANXA6/OSBPL8	4	0.784313725
MF	GO:0046982	protein heterodimerization activity	9/115	341/18432	0.000287685	0.009057608	0.006942469	DR1/GADD45A/SAE1/SUPT3H/RRAGD/CEBPB/ITGB1/ATP1A2/AOC3	9	0.263929619
MF	GO:0009931	calcium-dependent protein serine/threonine	3/115	22/18432	0.000334155	0.009057608	0.006942469	MAPKAPK2/PRKCH/MKNK1	3	1.363636364

		kinase activity								
MF	GO:0061629	RNA polymerase II-specific DNA-binding transcription factor binding	9/115	350/18432	0.000348124	0.009057608	0.006942469	PSMA6/CTBP1/HMGB1/NFE2L2/PRMT2/HIPK2/CEBPB/PTPN2/MAD2L2	9	0.257142857
MF	GO:0010857	calcium-dependent protein kinase activity	3/115	23/18432	0.000382535	0.009057608	0.006942469	MAPKAPK2/PRKCH/MKNK1	3	1.304347826
MF	GO:0008170	N-methyltransferase activity	5/115	99/18432	0.000388527	0.009057608	0.006942469	SUV39H2/DOT1L/PRMT2/NNMT/HNMT	5	0.505050505
MF	GO:0005496	steroid binding	5/115	101/18432	0.000426048	0.009057608	0.006942469	OSBPL5/SCARB2/ANXA6/ATP1A2/OSBPL8	5	0.495049505
MF	GO:0008483	transaminase activity	3/115	24/18432	0.000435201	0.009057608	0.006942469	BCAT2/OAT/PSAT1	3	1.25
MF	GO:0005178	integrin binding	6/115	156/18432	0.00043969	0.009057608	0.006942469	NF2/HMGB1/IGF2/ITGB1/PTPN2/LYN	6	0.384615385
MF	GO:0016769	transferase activity	3/115	25/18432	0.000492305	0.009619094	0.007372837	BCAT2/OAT/PSAT1	3	1.2
MF	GO:0000287	magnesium ion binding	7/115	225/18432	0.000528146	0.009619094	0.007372837	TSSK2/GLUL/MAST2/HMGCL/PLSCR1/GCLC/MAP3K5	7	0.311111111
MF	GO:0008757	S-adenosylmethionine-dependent methyltransferase activity	6/115	162/18432	0.000536988	0.009619094	0.007372837	SUV39H2/DOT1L/PRMT2/NNMT/HNMT/GAMT	6	0.37037037
MF	GO:0032934	sterol binding	4/115	61/18432	0.000570514	0.009748377	0.007471929	OSBPL5/SCARB2/ANXA6/OSBPL8	4	0.655737705
MF	GO:0016874	ligase activity	6/115	165/18432	0.000591528	0.009748377	0.007471929	GLUL/ASNS/SAE1/GCLC/GART/MTHFD1	6	0.363636364
MF	GO:0015078	proton transmembrane transporter activity	5/115	136/18432	0.001629364	0.023378672	0.017919268	ATP6V1E2/COX5A/ATP6V1F/COX8A/SLC25A12	5	0.367647059
MF	GO:0005528	FK506 binding	2/115	10/18432	0.001680687	0.023378672	0.017919268	FKBP1A/FKBP5	2	2
MF	GO:0016595	glutamate binding	2/115	10/18432	0.001680687	0.023378672	0.017919268	GLUL/GCLC	2	2
MF	GO:0016742	hydroxymethyl-	2/115	10/18432	0.001680687	0.023378672	0.017919268	GCSH/GART	2	2
MF	GO:0003755	peptidyl-prolyl cis-trans isomerase activity	3/115	38/18432	0.00170233	0.023378672	0.017919268	PPIC/FKBP1A/FKBP5	3	0.789473684
MF	GO:0022853	active ion transmembrane transporter activity	7/115	278/18432	0.001805576	0.023996682	0.01839296	ATP6V1E2/COX5A/ATP6V1F/COX8A/SLC25A12/ATP1A2/SLC24A3	7	0.251798561
MF	GO:0003713	transcription coactivator activity	7/115	281/18432	0.001918618	0.024702207	0.01893373	CTBP1/HMGB1/SUPT3H/PRMT2/HIPK2/ZBED1/RUVBL1	7	0.24911032
MF	GO:0000099	sulfur amino acid transmembrane transporter activity	2/115	11/18432	0.002045808	0.025259385	0.019360796	SLC25A12/SLC1A4	2	1.818181818
MF	GO:0016859	cis-trans isomerase activity	3/115	41/18432	0.00212212	0.025259385	0.019360796	PPIC/FKBP1A/FKBP5	3	0.731707317
MF	GO:0008168	methyltransferase activity	6/115	212/18432	0.002145822	0.025259385	0.019360796	SUV39H2/DOT1L/PRMT2/NNMT/HNMT/GAMT	6	0.283018868
MF	GO:0019842	vitamin binding	5/115	149/18432	0.002428656	0.026508708	0.020318376	PLOD1/FOLR2/OAT/SDSL/PSAT1	5	0.33557047
MF	GO:0043178	alcohol binding	4/115	90/18432	0.002432318	0.026508708	0.020318376	OSBPL5/SCARB2/ANXA6/OSBPL8	4	0.444444444
MF	GO:0005527	macrolide binding	2/115	12/18432	0.002444978	0.026508708	0.020318376	FKBP1A/FKBP5	2	1.666666667

MF	GO:0001046	core promoter sequence-specific DNA binding	3/115	44/18432	0.002601012	0.02747736	0.021060828	DR1/MYC/CEBPB	3	0.681818182
MF	GO:0005161	platelet-derived growth factor receptor binding	2/115	14/18432	0.00334374	0.034440522	0.026397947	PDGFRB/LYN	2	1.428571429
MF	GO:0015399	primary active transmembrane transporter activity	5/115	169/18432	0.004166806	0.040677456	0.031178428	ATP6V1E2/COX5A/ATP6V1F/COX8A/ATP1A2	5	0.295857988
MF	GO:0005159	insulin-like growth factor receptor binding	2/115	16/18432	0.004373568	0.040677456	0.031178428	SHC1/IGF2	2	1.25
MF	GO:0019829	ATPase-coupled cation transmembrane transporter activity	3/115	53/18432	0.00441756	0.040677456	0.031178428	ATP6V1E2/ATP6V1F/ATP1A2	3	0.566037736
MF	GO:0022804	active transmembrane transporter activity	8/115	417/18432	0.004627593	0.040677456	0.031178428	ATP6V1E2/COX5A/ATP6V1F/COX8A/SLC25A12/SLC1A4/ATP1A2/SLC24A3	8	0.191846523
MF	GO:0043539	protein serine/threonine kinase activator activity	3/115	54/18432	0.004656386	0.040677456	0.031178428	MTCP1/IGF2/TCL1A	3	0.555555556
MF	GO:0004601	peroxidase activity	3/115	55/18432	0.004902886	0.040677456	0.031178428	PRDX1/PRDX3/MGST2	3	0.545454545
MF	GO:0030170	pyridoxal phosphate binding	3/115	55/18432	0.004902886	0.040677456	0.031178428	OAT/SDSL/PSAT1	3	0.545454545
MF	GO:0004697	protein kinase C activity	2/115	17/18432	0.004936584	0.040677456	0.031178428	PRKCH/SMG1	2	1.176470588
MF	GO:0015172	acidic amino acid transmembrane transporter activity	2/115	17/18432	0.004936584	0.040677456	0.031178428	SLC25A12/SLC1A4	2	1.176470588
MF	GO:0015556	C4-dicarboxylate transmembrane transporter activity	2/115	17/18432	0.004936584	0.040677456	0.031178428	SLC25A12/SLC1A4	2	1.176470588
MF	GO:0070279	vitamin B6 binding	3/115	56/18432	0.005157128	0.041661507	0.031932683	OAT/SDSL/PSAT1	3	0.535714286
MF	GO:0016684	oxidoreductase activity	3/115	57/18432	0.00541918	0.042936583	0.032910002	PRDX1/PRDX3/MGST2	3	0.526315789
MF	GO:0042054	histone methyltransferase activity	3/115	59/18432	0.005966968	0.04611975	0.035349834	SUV39H2/DOT1L/PRMT2	3	0.508474576
MF	GO:0004129	cytochrome-c oxidase activity	2/115	19/18432	0.006156763	0.04611975	0.035349834	COX5A/COX8A	2	1.052631579
MF	GO:0004653	polypeptide N-acetylgalactosaminyltransferase activity	2/115	19/18432	0.006156763	0.04611975	0.035349834	GALNT11/GALNT1	2	1.052631579
MF	GO:0016675	oxidoreductase activity	2/115	20/18432	0.006813112	0.04924565	0.037745772	COX5A/COX8A	2	1
MF	GO:0035173	histone kinase activity	2/115	20/18432	0.006813112	0.04924565	0.037745772	PRKAA1/AURKC	2	1
MF	GO:0005548	phospholipid transporter activity	3/115	64/18432	0.007477355	0.053115006	0.040711553	OSBPL5/PLSCR1/OSBPL8	3	0.46875
MF	GO:0030145	manganese ion binding	3/115	65/18432	0.007804027	0.054495917	0.041769993	GLUL/HMGCL/GALNT1	3	0.461538462

MF	GO:0015179	L-amino acid transmembrane transporter activity	3/115	66/18432	0.008139014	0.054598814	0.041848861	SLC7A7/SLC25A12/SLC1A4	3	0.454545455
MF	GO:0005158	insulin receptor binding	2/115	22/18432	0.008216326	0.054598814	0.041848861	SHC1/IGF2	2	0.909090909
MF	GO:0050321	tau-protein kinase activity	2/115	22/18432	0.008216326	0.054598814	0.041848861	PRKAA1/ROCK2	2	0.909090909
MF	GO:0035035	histone acetyltransferase binding	2/115	23/18432	0.008962402	0.058611262	0.044924319	CEBPB/EGR1	2	0.869565217
MF	GO:0042625	ATPase-coupled ion transmembrane transporter activity	2/115	24/18432	0.009737602	0.060786241	0.046591396	ATP6V1E2/ATP6V1F	2	0.833333333
MF	GO:0044769	ATPase activity	2/115	24/18432	0.009737602	0.060786241	0.046591396	ATP6V1E2/ATP6V1F	2	0.833333333
MF	GO:0046961	proton-transporting ATPase activity	2/115	24/18432	0.009737602	0.060786241	0.046591396	ATP6V1E2/ATP6V1F	2	0.833333333
MF	GO:0043177	organic acid binding	4/115	137/18432	0.010698598	0.065788392	0.050425441	GLUL/PLOD1/GCLC/FOLR2	4	0.291970803
MF	GO:0004364	glutathione transferase activity	2/115	26/18432	0.011373836	0.066813431	0.051211112	GSTO1/MGST2	2	0.769230769
MF	GO:0017025	TBP-class protein binding	2/115	26/18432	0.011373836	0.066813431	0.051211112	DR1/RUVBL1	2	0.769230769
MF	GO:0072349	modified amino acid transmembrane transporter activity	2/115	26/18432	0.011373836	0.066813431	0.051211112	SLC25A12/SLC1A4	2	0.769230769
MF	GO:0070851	growth factor receptor binding	4/115	140/18432	0.011513965	0.066813431	0.051211112	SHC1/PDGFRB/PLSCR1/LYN	4	0.285714286
MF	GO:0004683	calmodulin-dependent protein kinase activity	2/115	27/18432	0.012234109	0.068952389	0.052850579	MAPKAPK2/MKNK1	2	0.740740741
MF	GO:0009678	pyrophosphate hydrolysis-driven proton transmembrane transporter activity	2/115	27/18432	0.012234109	0.068952389	0.052850579	ATP6V1E2/ATP6V1F	2	0.740740741
MF	GO:0001618	virus receptor activity	3/115	77/18432	0.012384652	0.068952389	0.052850579	SCARB2/PLSCR1/ITGB1	3	0.38961039
MF	GO:0140272	exogenous protein binding	3/115	78/18432	0.012822528	0.070211137	0.053815383	SCARB2/PLSCR1/ITGB1	3	0.384615385
MF	GO:0016645	oxidoreductase activity	2/115	28/18432	0.013121984	0.070211137	0.053815383	PAOX/MTHFD1	2	0.714285714
MF	GO:0070273	phosphatidylinositol-4-phosphate binding	2/115	28/18432	0.013121984	0.070211137	0.053815383	OSBPL5/OSBPL8	2	0.714285714
MF	GO:0046875	ephrin receptor binding	2/115	29/18432	0.014037091	0.073206093	0.056110955	SHC1/LYN	2	0.689655172
MF	GO:0051861	glycolipid binding	2/115	29/18432	0.014037091	0.073206093	0.056110955	CLN3/LYN	2	0.689655172
MF	GO:0001968	fibronectin binding	2/115	31/18432	0.015947527	0.081067374	0.062136465	CTSK/ITGB1	2	0.64516129
MF	GO:0005310	dicarboxylic acid transmembrane transporter activity	2/115	31/18432	0.015947527	0.081067374	0.062136465	SLC25A12/SLC1A4	2	0.64516129

MF	GO:0015171	amino acid transmembrane transporter activity	3/115	85/18432	0.016134769	0.081067374	0.062136465	SLC7A7/SLC25A12/SLC1A4	3	0.352941176
MF	GO:0051427	hormone receptor binding	2/115	32/18432	0.01694213	0.084098285	0.064459595	TYK2/UCN	2	0.625
MF	GO:0005154	epidermal growth factor receptor binding	2/115	33/18432	0.017962513	0.087065355	0.066733793	SHC1/PLSCR1	2	0.606060606
MF	GO:0019956	chemokine binding	2/115	33/18432	0.017962513	0.087065355	0.066733793	HMGB1/ITGB1	2	0.606060606
MF	GO:0008376	acetylgalactosaminyltransferase activity	2/115	34/18432	0.019008319	0.090016408	0.068995714	GALNT11/GALNT1	2	0.588235294
MF	GO:0043531	ADP binding	2/115	34/18432	0.019008319	0.090016408	0.068995714	GCLC/RUVBL1	2	0.588235294
MF	GO:0008276	protein methyltransferase activity	3/115	92/18432	0.019883753	0.092046137	0.070551459	SUV39H2/DOT1L/PRMT2	3	0.326086957
MF	GO:0051219	phosphoprotein binding	3/115	92/18432	0.019883753	0.092046137	0.070551459	SHC1/MAPK3/LYN	3	0.326086957
MF	GO:0015248	sterol transporter activity	2/115	36/18432	0.021174804	0.096933548	0.074297559	OSBPL5/OSBPL8	2	0.555555556
MF	GO:0016409	palmitoyltransferase activity	2/115	37/18432	0.02229479	0.099985319	0.076636678	GLUL/CPT1B	2	0.540540541
MF	GO:0031406	carboxylic acid binding	4/115	171/18432	0.022326819	0.099985319	0.076636678	GLUL/PLOD1/GCLC/FOLR2	4	0.233918129
MF	GO:0042626	ATPase-coupled transmembrane transporter activity	3/115	98/18432	0.023447462	0.10387478	0.079617869	ATP6V1E2/ATP6V1F/ATP1A2	3	0.306122449
MF	GO:0043028	cysteine-type endopeptidase regulator activity involved in apoptotic process	2/115	40/18432	0.025797637	0.113070493	0.086666193	RPS27L/PRDX3	2	0.5
MF	GO:0008022	protein C-terminus binding	4/115	181/18432	0.026796469	0.116212055	0.089074135	PRKAA1/CTBP1/SAE1/PRDX3	4	0.220994475
MF	GO:0015298	solute:cation antiporter activity	2/115	42/18432	0.028248601	0.121233581	0.092923031	SLC25A12/SLC24A3	2	0.476190476
MF	GO:0005543	phospholipid binding	7/115	474/18432	0.029065126	0.122800215	0.094123824	SHC1/OSBPL5/HMGB1/SCARB2/ANXA6/OSBPL8/AKT1	7	0.147679325
MF	GO:0018024	histone-lysine N-methyltransferase activity	2/115	43/18432	0.029507819	0.122800215	0.094123824	SUV39H2/DOT1L	2	0.465116279
MF	GO:0048156	tau protein binding	2/115	43/18432	0.029507819	0.122800215	0.094123824	PRKAA1/ROCK2	2	0.465116279
MF	GO:0015175	neutral amino acid transmembrane transporter activity	2/115	44/18432	0.030789095	0.125726188	0.096366521	SLC25A12/SLC1A4	2	0.454545455
MF	GO:0001221	transcription coregulator binding	3/115	109/18432	0.030821226	0.125726188	0.096366521	CTBP1/NFE2L2/MYC	3	0.275229358
MF	GO:0001784	phosphotyrosine residue	2/115	46/18432	0.033416553	0.134976664	0.103456819	SHC1/MAPK3	2	0.434782609

		binding								
MF	GO:0030295	protein kinase activator activity	3/115	115/18432	0.035298636	0.141194546	0.108222697	MTCP1/IGF2/TCL1A	3	0.260869565
MF	GO:0016829	lyase activity	4/115	199/18432	0.036108069	0.143043506	0.109639887	HMGB1/HMGCL/SDSL/MGST2	4	0.201005025
MF	GO:1990782	protein tyrosine kinase binding	3/115	119/18432	0.03846015	0.150910304	0.115669625	SHC1/ITGB1/PTPN2	3	0.25210084
MF	GO:0120013	lipid transfer activity	2/115	50/18432	0.038922342	0.151283067	0.11595534	OSBPL5/OSBPL8	2	0.4
MF	GO:0019209	kinase activator activity	3/115	121/18432	0.040093414	0.154378379	0.118327833	MTCP1/IGF2/TCL1A	3	0.247933884
MF	GO:0140296	general transcription initiation factor binding	2/115	53/18432	0.043261599	0.165034988	0.126495903	DR1/RUVBL1	2	0.377358491
MF	GO:0016597	amino acid binding	2/115	54/18432	0.044746415	0.169133237	0.129637126	GLUL/GCLC	2	0.37037037
MF	GO:1901682	sulfur compound transmembrane transporter activity	2/115	55/18432	0.046249944	0.173227063	0.132774959	SLC25A12/SLC1A4	2	0.363636364
MF	GO:0019887	protein kinase regulator activity	4/115	218/18432	0.047732027	0.177167524	0.135795241	MTCP1/IGF2/IBTK/TCL1A	4	0.183486239
MF	GO:0019838	growth factor binding	3/115	132/18432	0.049692399	0.182797039	0.140110147	SHC1/FGFR1/PDGFRB	3	0.227272727

Table S3: The results of KEGG pathways significantly enriched with candidate genes

ID	Description	GeneRatio	BgRatio	pvalue	p.adjust	qvalue	geneID	Count	richFactor
hsa05225	Hepatocellular carcinoma	10/86	170/8842	5.32E-06	0.001277024	0.000873753	SHC1/GADD45A/NFE2L2/MYC/MAPK3/IGF2/GSTO1/NQO1/AKT1/MGST2	10	0.588235294
hsa04010	MAPK signaling pathway	10/86	300/8842	0.000628104	0.015358595	0.010508512	MAPKAPK2/GADD45A/FGFR1/PDGFRB/MYC/MAPK3/IGF2/MAP3K5/MKNK1/AKT1	10	0.333333333
hsa04151	PI3K-Akt signaling pathway	10/86	362/8842	0.002584875	0.036492358	0.024968456	PRKAA1/MTCP1/FGFR1/PDGFRB/MYC/MAPK3/IGF2/ITGB1/AKT1/TCL1A	10	0.276243094
hsa05208	Chemical carcinogenesis - reactive oxygen species	9/86	226/8842	0.000330341	0.015358595	0.010508512	COX5A/COX8A/NFE2L2/MAPK3/GSTO1/MAP3K5/NQO1/AKT1/MGST2	9	0.398230088
hsa05010	Alzheimer disease	9/86	391/8842	0.013392307	0.089282045	0.061087715	PSMA6/COX5A/CSNK2A2/EIF2AK2/PSMD12/COX8A/MAPK3/MAP3K5/AKT1	9	0.230179028
hsa05165	Human papillomavirus infection	8/86	333/8842	0.015398786	0.090616409	0.062000701	ATP6V1E2/ATP6V1F/EIF2AK2/TYK2/PDGFRB/MAPK3/ITGB1/AKT1	8	0.24024024
hsa05418	Fluid shear stress and atherosclerosis	7/86	141/8842	0.000424813	0.015358595	0.010508512	PRKAA1/NFE2L2/GSTO1/MAP3K5/NQO1/AKT1/MGST2	7	0.496453901
hsa04140	Autophagy - animal	7/86	169/8842	0.001244461	0.021333619	0.014596687	SH3GLB1/PRKAA1/EIF2AK4/HMGB1/RRAGD/MAPK3/AKT1	7	0.414201183
hsa05167	Kaposi sarcoma-associated herpesvirus infection	7/86	196/8842	0.002896156	0.038615414	0.026421073	MAPKAPK2/EIF2AK2/TYK2/MYC/MAPK3/LYN/AKT1	7	0.357142857
hsa05169	Epstein-Barr virus infection	7/86	203/8842	0.003519533	0.041329709	0.028278222	GADD45A/EIF2AK2/PSMD12/TYK2/MYC/LYN/AKT1	7	0.344827586
hsa05205	Proteoglycans in cancer	7/86	204/8842	0.00361635	0.041329709	0.028278222	FGFR1/MYC/MAPK3/IGF2/ITGB1/AKT1/ROCK2	7	0.343137255
hsa05020	Prion disease	7/86	278/8842	0.018284636	0.096250674	0.065855724	PSMA6/COX5A/CSNK2A2/PSMD12/COX8A/MAPK3/EGR1	7	0.251798561
hsa05220	Chronic myeloid leukemia	6/86	77/8842	9.69E-05	0.007751617	0.005303738	SHC1/CTBP1/GADD45A/MYC/MAPK3/AKT1	6	0.779220779
hsa05224	Breast cancer	6/86	148/8842	0.00311108	0.039297851	0.026888003	SHC1/GADD45A/FGFR1/MYC/MAPK3/AKT1	6	0.405405405
hsa04218	Cellular senescence	6/86	157/8842	0.004161658	0.04427008	0.030290055	MAPKAPK2/GADD45A/MYC/MAPK3/HIPK2/AKT1	6	0.382165605
hsa04150	mTOR signaling pathway	6/86	158/8842	0.004292814	0.04427008	0.030290055	ATP6V1E2/PRKAA1/ATP6V1F/RRAGD/MAPK3/AKT1	6	0.379746835
hsa05160	Hepatitis C	6/86	159/8842	0.004427008	0.04427008	0.030290055	EIF2AK2/EIF2AK4/TYK2/MYC/MAPK3/AKT1	6	0.377358491
hsa04510	Focal adhesion	6/86	203/8842	0.013974548	0.090616409	0.062000701	SHC1/PDGFRB/MAPK3/ITGB1/AKT1/ROCK2	6	0.295566502
hsa05417	Lipid and atherosclerosis	6/86	216/8842	0.018448046	0.096250674	0.065855724	NFE2L2/MAPK3/MAP3K5/LYN/AKT1/ROCK2	6	0.277777778
hsa04810	Regulation of actin cytoskeleton	6/86	230/8842	0.024273763	0.109918926	0.075207686	FGFR1/PDGFRB/MAPK3/ITGB1/AKT1/ROCK2	6	0.260869565
hsa04714	Thermogenesis	6/86	235/8842	0.026623717	0.114954278	0.078652927	COX5A/PRKAA1/CPT1B/FGFR1/COX8A/MAP3K5	6	0.255319149
hsa04014	Ras signaling pathway	6/86	238/8842	0.028104233	0.114954278	0.078652927	SHC1/FGFR1/PDGFRB/MAPK3/IGF2/AKT1	6	0.25210084
hsa05012	Parkinson disease	6/86	271/8842	0.04806734	0.140684898	0.096258088	PSMA6/COX5A/PSMD12/COX8A/NFE2L2/MAP3K5	6	0.221402214
hsa00260	Glycine, serine and threonine metabolism	5/86	41/8842	4.40E-05	0.00528579	0.003616593	GCSH/SDSL/AOC3/PSAT1/GAMT	5	1.219512195
hsa00270	Cysteine and methionine metabolism	5/86	52/8842	0.00014047	0.008428215	0.005766674	BCAT2/GCLC/ADI1/SDSL/PSAT1	5	0.961538462
hsa05230	Central carbon metabolism in cancer	5/86	71/8842	0.000608981	0.015358595	0.010508512	FGFR1/PDGFRB/MYC/MAPK3/AKT1	5	0.704225352
hsa05218	Melanoma	5/86	73/8842	0.000691953	0.015358595	0.010508512	GADD45A/FGFR1/PDGFRB/MAPK3/AKT1	5	0.684931507
hsa01230	Biosynthesis of amino acids	5/86	75/8842	0.000783116	0.015358595	0.010508512	GLUL/ASNS/BCAT2/SDSL/PSAT1	5	0.666666667
hsa01524	Platinum drug resistance	5/86	75/8842	0.000783116	0.015358595	0.010508512	MAPK3/GSTO1/MAP3K5/AKT1/MGST2	5	0.666666667
hsa05214	Glioma	5/86	76/8842	0.000831924	0.015358595	0.010508512	SHC1/GADD45A/PDGFRB/MAPK3/AKT1	5	0.657894737
hsa04722	Neurotrophin signaling pathway	5/86	120/8842	0.006152027	0.05678794	0.038854906	SHC1/MAPKAPK2/MAPK3/MAP3K5/AKT1	5	0.416666667
hsa04611	Platelet activation	5/86	125/8842	0.007291033	0.064531116	0.044152869	MAPK3/ITGB1/LYN/AKT1/ROCK2	5	0.4

hsa04210	Apoptosis	5/86	136/8842	0.01030096	0.079749371	0.054565359	GADD45A/CTSK/MAPK3/MAP3K5/AKT1	5	0.367647059
hsa04910	Insulin signaling pathway	5/86	138/8842	0.010927647	0.081822417	0.055983759	SHC1/PRKAA1/MAPK3/MKNK1/AKT1	5	0.362318841
hsa05162	Measles	5/86	139/8842	0.011250582	0.081822417	0.055983759	CSNK2A2/EIF2AK2/EIF2AK4/TYK2/AKT1	5	0.35971223
hsa05226	Gastric cancer	5/86	150/8842	0.015242667	0.090616409	0.062000701	SHC1/GADD45A/MYC/MAPK3/AKT1	5	0.333333333
hsa01240	Biosynthesis of cofactors	5/86	153/8842	0.016477106	0.091965245	0.062923588	BCAT2/GCLC/MTHFD1/NQO1/PSAT1	5	0.326797386
hsa04932	Non-alcoholic fatty liver disease	5/86	157/8842	0.018224484	0.096250674	0.065855724	COX5A/PRKAA1/COX8A/MAP3K5/AKT1	5	0.318471338
hsa04630	JAK-STAT signaling pathway	5/86	168/8842	0.023650572	0.109156486	0.074686017	TYK2/PDGFRB/MYC/PTPN2/AKT1	5	0.297619048
hsa04530	Tight junction	5/86	170/8842	0.024738083	0.109947034	0.075226918	NF2/PRKAA1/ITGB1/MAP3K5/ROCK2	5	0.294117647
hsa04310	Wnt signaling pathway	5/86	174/8842	0.027008889	0.114954278	0.078652927	CTBP1/CSNK2A2/MYC/RUVBL1/ROCK2	5	0.287356322
hsa04062	Chemokine signaling pathway	5/86	193/8842	0.039595369	0.130908243	0.089568798	SHC1/MAPK3/LYN/AKT1/ROCK2	5	0.259067358
hsa05202	Transcriptional misregulation in cancer	5/86	193/8842	0.039595369	0.130908243	0.089568798	GADD45A/DOT1L/SUPT3H/MYC/CEBPB	5	0.259067358
hsa00480	Glutathione metabolism	4/86	58/8842	0.002373293	0.036492358	0.024968456	GCLC/GSTO1/RRM2B/MGST2	4	0.689655172
hsa05213	Endometrial cancer	4/86	59/8842	0.002527239	0.036492358	0.024968456	GADD45A/MYC/MAPK3/AKT1	4	0.677966102
hsa01521	EGFR tyrosine kinase inhibitor resistance	4/86	80/8842	0.00752863	0.064531116	0.044152869	SHC1/PDGFRB/MAPK3/AKT1	4	0.5
hsa04012	ErbB signaling pathway	4/86	86/8842	0.009674897	0.079749371	0.054565359	SHC1/MYC/MAPK3/AKT1	4	0.465116279
hsa05210	Colorectal cancer	4/86	87/8842	0.010067391	0.079749371	0.054565359	GADD45A/MYC/MAPK3/AKT1	4	0.459770115
hsa04520	Adherens junction	4/86	93/8842	0.012640109	0.086675032	0.059303969	CSNK2A2/FGFR1/MAPK3/ROCK2	4	0.430107527
hsa05222	Small cell lung cancer	4/86	93/8842	0.012640109	0.086675032	0.059303969	GADD45A/MYC/ITGB1/AKT1	4	0.430107527
hsa05215	Prostate cancer	4/86	98/8842	0.015079122	0.090616409	0.062000701	FGFR1/PDGFRB/MAPK3/AKT1	4	0.408163265
hsa05231	Choline metabolism in cancer	4/86	99/8842	0.015600116	0.090616409	0.062000701	SLC44A1/PDGFRB/MAPK3/AKT1	4	0.404040404
hsa04620	Toll-like receptor signaling pathway	4/86	109/8842	0.021439554	0.105010061	0.071848989	CTSK/TYK2/MAPK3/AKT1	4	0.366972477
hsa05145	Toxoplasmosis	4/86	111/8842	0.022748008	0.109156486	0.074686017	TYK2/MAPK3/ITGB1/AKT1	4	0.36036036
hsa04668	TNF signaling pathway	4/86	119/8842	0.028463902	0.114954278	0.078652927	MAPK3/CEBPB/MAP3K5/AKT1	4	0.336134454
hsa04071	Sphingolipid signaling pathway	4/86	122/8842	0.030808982	0.114954278	0.078652927	MAPK3/MAP3K5/AKT1/ROCK2	4	0.327868852
hsa04919	Thyroid hormone signaling pathway	4/86	122/8842	0.030808982	0.114954278	0.078652927	MYC/MAPK3/ATP1A2/AKT1	4	0.327868852
hsa04068	FoxO signaling pathway	4/86	133/8842	0.040363375	0.130908243	0.089568798	PRKAA1/GADD45A/MAPK3/AKT1	4	0.30075188
hsa00190	Oxidative phosphorylation	4/86	138/8842	0.045206088	0.140684898	0.096258088	ATP6V1E2/COX5A/ATP6V1F/COX8A	4	0.289855072
hsa05135	Yersinia infection	4/86	138/8842	0.045206088	0.140684898	0.096258088	MAPK3/ITGB1/AKT1/ROCK2	4	0.289855072
hsa04915	Estrogen signaling pathway	4/86	139/8842	0.046212177	0.140684898	0.096258088	SHC1/MAPK3/FKBP5/AKT1	4	0.287769784
hsa04371	Apelin signaling pathway	4/86	140/8842	0.047230771	0.140684898	0.096258088	PRKAA1/MAPK3/EGR1/AKT1	4	0.285714286
hsa04380	Osteoclast differentiation	4/86	142/8842	0.049305438	0.141695398	0.096949483	CTSK/TYK2/MAPK3/AKT1	4	0.281690141
hsa05216	Thyroid cancer	3/86	37/8842	0.005437912	0.052203952	0.035718494	GADD45A/MYC/MAPK3	3	0.810810811
hsa04370	VEGF signaling pathway	3/86	60/8842	0.02041655	0.102082751	0.069846093	MAPKAPK2/MAPK3/AKT1	3	0.5
hsa00310	Lysine degradation	3/86	63/8842	0.023207294	0.109156486	0.074686017	PLOD1/SUV39H2/DOT1L	3	0.476190476
hsa05221	Acute myeloid leukemia	3/86	68/8842	0.028296717	0.114954278	0.078652927	MYC/MAPK3/AKT1	3	0.441176471
hsa04664	Fc epsilon RI signaling pathway	3/86	69/8842	0.029380182	0.114954278	0.078652927	MAPK3/LYN/AKT1	3	0.434782609
hsa04920	Adipocytokine signaling pathway	3/86	70/8842	0.030485433	0.114954278	0.078652927	PRKAA1/CPT1B/AKT1	3	0.428571429
hsa04917	Prolactin signaling pathway	3/86	71/8842	0.031612427	0.114954278	0.078652927	SHC1/MAPK3/AKT1	3	0.422535211

hsa05120	Epithelial cell signaling in Helicobacter pylori infection	3/86	71/8842	0.031612427	0.114954278	0.078652927	ATP6V1E2/ATP6V1F/LYN	3	0.422535211
hsa05223	Non-small cell lung cancer	3/86	73/8842	0.033931436	0.120374948	0.082361807	GADD45A/MAPK3/AKT1	3	0.410958904
hsa05212	Pancreatic cancer	3/86	77/8842	0.038827826	0.130908243	0.089568798	GADD45A/MAPK3/AKT1	3	0.38961039
hsa05100	Bacterial invasion of epithelial cells	3/86	78/8842	0.040105333	0.130908243	0.089568798	SHC1/ITGB1/MAD2L2	3	0.384615385
hsa00983	Drug metabolism - other enzymes	3/86	81/8842	0.044064704	0.140684898	0.096258088	GSTO1/RRM2B/MGST2	3	0.37037037
hsa04146	Peroxisome	3/86	83/8842	0.046809044	0.140684898	0.096258088	PAOX/HMGCL/PRDX1	3	0.361445783
hsa00290	Valine, leucine and isoleucine biosynthesis	2/86	4/8842	0.000553986	0.015358595	0.010508512	BCAT2/SDSL	2	5
hsa00670	One carbon pool by folate	2/86	20/8842	0.015857872	0.090616409	0.062000701	GART/MTHFD1	2	1
hsa04966	Collecting duct acid secretion	2/86	28/8842	0.030008843	0.114954278	0.078652927	ATP6V1E2/ATP6V1F	2	0.714285714
hsa01523	Antifolate resistance	2/86	30/8842	0.034106235	0.120374948	0.082361807	GART/FOLR2	2	0.666666667
hsa00630	Glyoxylate and dicarboxylate metabolism	2/86	31/8842	0.036232151	0.126024875	0.086227546	GLUL/GCSH	2	0.64516129
hsa00512	Mucin type O-glycan biosynthesis	2/86	36/8842	0.047587849	0.140684898	0.096258088	GALNT11/GALNT1	2	0.555555556
hsa00250	Alanine, aspartate and glutamate metabolism	2/86	37/8842	0.049996417	0.141695398	0.096949483	GLUL/ASNS	2	0.540540541
hsa03271	Virion - Rotavirus	1/86	2/8842	0.019359101	0.098854983	0.06763762	ITGB1	1	5

Table S4: GSEA results of AKT1

	ID	Description	setSize	enrichmentScore	NES	pvalue	p.adjust	qvalue	rank	leading_edge	core_enrichment
GOBP_SEXUAL_REPRODUCTION	GOBP_SEXUAL_REPRODUCTION	GOBP_SEXUAL_REPRODUCTION	157	-0.344805172	-3.548826062	5.82E-14	2.56E-10	2.13E-10	689	tags=54%, list=25%, signal=42%	PARP11/RAD54B/FOXJ1/PRSS21/FZR1/PIWIL2/AURKC/CCDC42/BOLL/UBE3A/RUVBL1/HSPA2/TRIP13/TIAL1/LYZL6/TNP2/CSNK2A2/RPL39L/FKBP6/TCP11/CDC25C/RMI1/SPAG4/PAFAH1B1/HSF2/CATSPER2/MAST2/PPP1CC/CKS2/TUBG1/WNT3/ACRBP/PTTG1/SMC3/STAG3/KLHL10/TDRKH/PTTG2/CCNB2/ACTL7A/IQCF1/SETX/PAQR7/PLEKHA1/RAD51/SMC1B/TOP3A/TSSK2/ELL3/MYBL1/SPATA6/SPA17/AKAP4/MKRN2/TESK2/ROPN1L/BBS4/AFF4/ARID4B/MKKS/DAZAP1/CCIN/ZW10/SFMBT1/TTC21A/RFX2/IQCG/ZPBP2/STRBP/RAD50/IFT20/MYCBPAP/TBPL1/SPACA3/SPAG8/SRPK1/MYCBP/HSPA1L/TCFL5/LZTFL1/TDRD7/DDX20/ANAPC10/DZIP1
GOBP_MALE_GAMETE_GENERATION	GOBP_MALE_GAMETE_GENERATION	GOBP_MALE_GAMETE_GENERATION	97	-0.427342929	-3.891875862	1.78E-13	3.91E-10	3.26E-10	689	tags=62%, list=25%, signal=48%	PARP11/FOXJ1/PRSS21/PIWIL2/CCDC42/BOLL/RUVBL1/HSPA2/TRIP13/TNP2/CSNK2A2/RPL39L/FKBP6/TCP11/CDC25C/SPAG4/PAFAH1B1/HSF2/CATSPER2/MAST2/PPP1CC/ACRBP/PTTG1/KLHL10/TDRKH/ACTL7A/IQCF1/SETX/PLEKHA1/TSSK2/ELL3/MYBL1/SPATA6/SPA17/AKAP4/MKRN2/TESK2/ROPN1L/BBS4/AFF4/ARID4B/MKKS/DAZAP1/CCIN/SFMBT1/TTC21A/RFX2/IQCG/ZPBP2/STRBP/IFT20/MYCBPAP/TBPL1/SPAG8/SRPK1/MYCBP/TCFL5/LZTFL1/TDRD7/DZIP1
GOBP_GAMETE_GENERATION	GOBP_GAMETE_GENERATION	GOBP_GAMETE_GENERATION	121	-0.350601857	-3.372001123	8.52E-11	1.25E-07	1.04E-07	460	tags=45%, list=17%, signal=39%	CSNK2A2/RPL39L/FKBP6/TCP11/CDC25C/SPAG4/PAFAH1B1/HSF2/CATSPER2/MAST2/PPP1CC/WNT3/ACRBP/PTTG1/KLHL10/TDRKH/CCNB2/ACTL7A/IQCF1/SETX/PAQR7/PLEKHA1/TSSK2/ELL3/MYBL1/SPATA6/SPA17/AKAP4/MKRN2/TESK2/ROPN1L/BBS4/AFF4/ARID4B/MKKS/DAZAP1/CCIN/SFMBT1/TTC21A/RFX2/IQCG/ZPBP2/STRBP/IFT20/MYCBPAP/TBPL1/SPAG8/SRPK1/MYCBP/TCFL5/LZTFL1/TDRD7/DDX20/DZIP1
GOCC_CILIUM	GOCC_CILIUM	GOCC_CILIUM	102	-0.352373369	-3.23084862	2.46E-09	2.70E-06	2.25E-06	437	tags=43%, list=16%, signal=38%	IFT122/TCP11/SPAG4/PAFAH1B1/TULP2/STARD10/CATSPER2/CEP290/TUBG1/CEP250/DNAH8/ACTL7A/ARFGEF2/DNAH17/DYNLL2/SPATA6/SPA17/AKAP4/SPAG5/ROPN1L/BBS4/ATP1B3/MKKS/WDR54/DNAI1/CLUAP1/TTC21A/NME7/CCDC96/IQCG/IQCD/IFT20/KIF3A/C20orf85/SPACA3/SPAG8/DYNLRB2/LZTFL1/SPATA7/DNALI1/GPI/DZIP1/IFT

											74/EFHC1
GOBP_MULTICELLULAR_ORGANISM_REPRODUCTION	GOBP_MULTICELLULAR_ORGANISM_REPRODUCTION	GOBP_MULTICELLULAR_ORGANISM_REPRODUCTION	146	-0.288255896	-2.940899328	1.46E-08	1.29E-05	1.07E-05	511	tags=40%, list=19%, signal=35%	TIAL1/LYZL6/TNP2/CSNK2A2/RPL39L/FKBP6/TCP11/CDC25C/SPAG4/PAFAH1B1/HSF2/CATSPER2/MAST2/PPP1CC/WNT3/ACRBP/PTTG1/KLHL10/TDRKH/CCNB2/ACTL7A/IQCF1/SETX/PAQR7/PLEKHA1/TSSK2/ELL3/MYBL1/SPATA6/SPA17/AKAP4/MKRN2/TESK2/ROPN1L/BBS4/AFF4/ARID4B/MKKS/DAZAP1/CCIN/SFMBT1/TTC21A/RFX2/IQCG/ZPBP2/STRBP/IFT20/MYCBPAP/TBPL1/SPACA3/SPAG8/SRPK1/MYCBP/TCFL5/LZTFL1/TDRD7/DDX20/PCNA/DZIP1
GOCC_CHROMOSOMAL_REGION	GOCC_CHROMOSOMAL_REGION	GOCC_CHROMOSOMAL_REGION	64	-0.415144589	-3.318784707	1.82E-08	1.33E-05	1.11E-05	850	tags=70%, list=31%, signal=50%	POT1/SMARCC1/NUDCD2/SMC6/BUB1B/UHRF2/THOC7/CBX1/NUP107/CHEK2/RECQL4/HAT1/AURKC/TFIP11/RAD17/BLM/CENPM/CDCA8/CENPH/SUV39H2/PINX1/ZNF330/TPR/DYNC1LI1/PAFAH1B1/PPP1CC/SMC3/ATR/STAG3/BRD7/NDE1/SETX/RAD51/SMC1B/CENPE/SPAG5/ZW10/SMCHD1/ITGB3BP/C1orf112/WRN/RAD50/AHCTF1/PCNA/SUGT1
GOBP_SPERMATID_DIFFERENTIATION	GOBP_SPERMATID_DIFFERENTIATION	GOBP_SPERMATID_DIFFERENTIATION	38	-0.506087364	-3.337593338	2.96E-08	1.86E-05	1.55E-05	615	tags=68%, list=23%, signal=54%	CCDC42/HSPA2/TRIP13/TNP2/TCP11/PAFAH1B1/CATSPER2/MAST2/ACRBP/KLHL10/ACTL7A/IQCF1/TSSK2/AKAP4/ROPN1L/BBS4/AFF4/MKKS/TTC21A/RFX2/IQCG/ZPBP2/STRBP/TBPL1/SRPK1/DZIP1
GOBP_CILIUM_OR_FLAGELLUM_DEPENDENT_CELL_MOTILITY	GOBP_CILIUM_OR_FLAGELLUM_DEPENDENT_CELL_MOTILITY	GOBP_CILIUM_OR_FLAGELLUM_DEPENDENT_CELL_MOTILITY	19	-0.64517841	-3.198498404	7.25E-08	3.98E-05	3.32E-05	405	tags=74%, list=15%, signal=63%	CATSPER2/DNAH8/IQCF1/DNAH17/AKAP4/ROPN1L/BBS4/MKKS/DNAI1/TTC21A/IQCG/LZTFL1/DZIP1/EFHC1
GOBP_CILIUM_MOVEMENT	GOBP_CILIUM_MOVEMENT	GOBP_CILIUM_MOVEMENT	21	-0.626618012	-3.259524886	1.14E-07	4.57E-05	3.81E-05	405	tags=71%, list=15%, signal=61%	CATSPER2/ZMYND10/DNAH8/IQCF1/DNAH17/SPA17/AKAP4/ROPN1L/BBS4/MKKS/DNAI1/TTC21A/IQCG/LZTFL1/DZIP1
GOCC_MOTILE_CILIUM	GOCC_MOTILE_CILIUM	GOCC_MOTILE_CILIUM	37	-0.502378303	-3.257909378	1.13E-07	4.57E-05	3.81E-05	431	tags=65%, list=16%, signal=55%	CAPZB/LYZL6/TCP11/SPAG4/PAFAH1B1/STARD10/CATSPER2/DNAH8/ACTL7A/DNAH17/SPATA6/SPA17/AKAP4/ROPN1L/BBS4/ATP1B3/MKKS/DNAI1/IQCG/IQCD/IFT20/SPACA3/DNALI1/IFT74
GOBP_CHROMOSOME_ORGANIZATION	GOBP_CHROMOSOME_ORGANIZATION	GOBP_CHROMOSOME_ORGANIZATION	107	-0.308154329	-2.894641223	1.09E-07	4.57E-05	3.81E-05	867	tags=61%, list=32%, signal=43%	YLPM1/KPNB1/POT1/SMARCC1/TUBG2/SMC6/BUB1B/PDCD6IP/MAPRE1/ERCC3/ESPL1/GTF2H2/NUP107/RAD54B/RFC1/MAD2L1BP/CHEK2/RECQL4/HAT1/AURKC/PPHLN1/KIF4A/TFIP11/RUVBL1/HSPA2/CHD1L/TRIP13/DHX30/RAE1/DOT1L/NUSAP1/BLM/CDCA8/CENPH/KIF23/PINX1/TPR/DYNC1LI1/MAPK3/TUBG1/PTTG1/SMC3/ATR/STAG3/

GOBP_DNA_REPAIR	GOBP_DNA_REPAIR	GOBP_DNA_REPAIR	102	-0.31664323	-2.903245344	1.68E-07	6.17E-05	5.14E-05	828	tags=59%, list=30%, signal=43%	PTTG2/BRD7/TOP1MT/SETX/RAD51/SMC1B/TOP3A/CENPE/ATF7IP/SPAG5/NUP155/ZW10/SMCHD1/RFC4/ITGB3BP/CDK5RAP2/WRN/RAD50/KATNB1/PCNA/SUGT1 BARD1/SMARCC1/VCP/POLE/SMC6/ENY2/ERCC3/GTF2H2/RAD54B/CCDC117/ALKBH3/RFC1/CHEK2/FZR1/RECQL4/CEBPG/TFIP11/CDC7/RUVBL1/CHD1L/TRAIP/TRIP13/RAD17/POLB/PAXIP1/UBE2N/DOT1L/BLM/MBD4/TIMELESS/RCHY1/NHEJ1/RMI1/RNF138/GTF2H1/CDC14B/UBE2T/ATXN3/PTTG1/SMC3/ATR/SUPT3H/BRD7/TAF9/SETX/RAD51/TOP3A/TAF6/MDC1/NUDT1/USP1/SMCHD1/TAF10/RFC4/C1orf112/WRN/UBE2D3/RAD50/CHAF1B/PCNA
GOBP_REPRODUCTION	GOBP_REPRODUCTION	GOBP_REPRODUCTION	234	-0.217912638	-2.525489675	6.02E-07	0.000203521	0.000169708	448	tags=32%, list=16%, signal=29%	RPL39L/FKBP6/TCP11/CDC25C/RMI1/SPAG4/PAFAH1B1/HSF2/NCOA4/CATSPER2/MAST2/PPP1CC/CKS2/TUBG1/TESC/WNT3/ACRBP/PTTG1/SMC3/STAG3/KLHL10/TDRKH/PTTG2/CCNB2/ACTL7A/IQCF1/SETX/PAQR7/PLEKHA1/RAD51/SMC1B/TOP3A/TSSK2/ELL3/NUDT1/MYBL1/SPATA6/SPA17/AKAP4/FKBP4/MKRN2/TESK2/ROPN1L/BBS4/AFF4/ARID4B/MKKS/DAZAP1/CCIN/ZW10/DNAI1/SFMBT1/TTC21A/RFX2/IQCG/ZPBP2/STRBP/RAD50/EIF2B4/IFT20/MYCBPAP/TBPL1/SPACA3/SPAG8/SRPK1/MYCBP/HSPA1L/TCFL5/LZTFL1/TDRD7/DDX20/PCNA/ANAPC10/DZIP1
GOBP_RESPONSE_TO_WOUNDING	GOBP_RESPONSE_TO_WOUNDING	GOBP_RESPONSE_TO_WOUNDING	108	0.320059697	2.593729008	6.83E-07	0.000214189	0.000178604	994	tags=58%, list=36%, signal=39%	RHOC/KLKB1/PPARA/ENTPD2/EXTL3/ITPK1/CLDN3/TNF/TIMP1/SDC4/BAX/TSPO/STAT3/VPS4A/HSPB1/FCER1G/GATA4/RAC1/SYK/ADRA2B/KREMEN1/CLIC1/ADIPOR2/CTSG/WNT3A/VASH1/PTN/DGKG/HPS6/TOR1A/AGER/NRP1/ITGB1/KCNB1/ANXA6/SERPINE2/FGF1/SPRR3/PLAT/ADAM17/F2RL3/GNA12/GLI1/CHMP7/ST3GAL4/ILK/VWF/COL3A1/MSX2/RTN4RL2/MERTK/DCBLD2/EDN1/LRG1/IL17A/NFE2L2/NFIA/CHMP6/NF1/F10/SERPINA1/YAP1/CLDN4
GOBP_MICROTUBULE_BASED_MOVEMENT	GOBP_MICROTUBULE_BASED_MOVEMENT	GOBP_MICROTUBULE_BASED_MOVEMENT	55	-0.403849193	-3.032984679	7.84E-07	0.000229384	0.000191275	464	tags=49%, list=17%, signal=42%	KIF23/DYNC1LI1/IFT122/PAFAH1B1/CATSPER2/ZMYND10/DNAH8/NDE1/IQCF1/DNAH17/SPA17/CENPE/AKAP4/ROPN1L/BBS4/MKKS/DNAI1/CLUAP1/TTC21A/IQCG/IFT20/KIF3A/KATNB1/DYNLRB2/LZTFL1/DZIP1/IFT74
GOMF_MOLECUL	GOMF_MOLECUL	GOMF_MOLECULA	189	0.271066518	2.574310488	8.35E-07	0.000229	0.000191	1530	tags=77%,	LRP6/PPARA/EXTL3/CLDN3/RXRB/GPRC5B/SDC

AR_TRANSDUCER_ACTIVITY	AR_TRANSDUCER_ACTIVITY	R_TRANSDUCER_ACTIVITY					384	275		list=56%, signal=36%	4/TSPO/P2RX2/STAT3/XCR1/FCER1G/PLXNB2/ADRA2B/TNFRSF4/GPR39/ADIPOR1/ADIPOR2/OR2T4/MRGPRF/OR4D5/M6PR/TAAR5/CD79A/DERL1/CHRNB2/PDGFRB/AGER/NRP1/PTCH2/ITGB1/OR8H2/OR11L1/GPR37/FGFR1/OR5F1/OR52N2/PTPRC/HCRTR1/GHSR/FZD4/GPR78/CNTNAP1/LRP1/OR8B8/CD4/IL1R1/CRHR2/F2RL3/OR52M1/OR3A3/IFNGR1/RRH/BCAM/SMO/MTNR1A/SQSTM1/MCHR1/OR51S1/OR4K17/PLXNA1/GRIK5/OR1D4/SPN/EPHA8/OR7G2/RTN4RL2/OR2B11/GFRA3/MERTK/OR6V1/GRM1/DCBLD2/TAAR6/IGF2R/OR5V1/HLA-DRB3/OR6Y1/CD300A/CLDN4/NFAM1/OR52E4/CHRNA7/PROKR2/OR6X1/PIGR/SSTR3/GAL/SCARB2/TEK/OR51L1/OPN1LW/GPR12/VAC14/SLC1A5/MAS1L/CRHR1/OR6C4/OR4M1/HLA-DQB1/RGMB/CCR5/LAG3/GP1BB/TAS2R9/ROR2/IFNAR2/ERBB2/OR4D1/IL9R/GPR119/OR10H4/FOLR2/CR2/ENG/P2RY8/ANTXR2/LTBP1/OR8G1/OR6B2/OR6K2/HRH3/LRP8/C3AR1/HLA-DOB/CELSR2/TAS2R39/EPHA4/OR51A7/OR6M1/OR9K2/OR8D1/KISS1R/OSCAR/P2RY10/KIR2DL4/GRIN2C/GPR83/OR52D1/IFNAR1/NR1H2/MRGPRX3/OR13C4/OPN1MW/GRIN1
GOBP_DEVELOPMENTAL_PROCESS_INVOLVED_IN_REPRODUCTION	GOBP_DEVELOPMENTAL_PROCESS_INVOLVED_IN_REPRODUCTION	GOBP_DEVELOPMENTAL_PROCESS_INVOLVED_IN_REPRODUCTION	163	-0.24970179	-2.601572638	9.06E-07	0.000234034	0.000195153	460	tags=35%, list=17%, signal=31%	CSNK2A2/RPL39L/FKBP6/TCP11/CDC25C/SPAG4/PAFAH1B1/HSF2/NCOA4/CATSPER2/MAST2/PPP1CC/TESC/ACRBP/PTTG1/KLHL10/TDRKH/ACTL7A/IQCF1/SETX/PAQR7/PLEKHA1/TSSK2/ELL3/NUDT1/MYBL1/SPATA6/SPA17/AKAP4/FKBP4/MKRN2/TESK2/ROPN1L/BBS4/AFF4/ARID4B/MKKS/DAZAP1/CCIN/SFMBT1/TTC21A/RFX2/IQCG/ZPBP2/STRBP/EIF2B4/IFT20/MYCBPAP/TBPL1/SPAG8/SRPK1/MYCBP/TCFL5/LZTFL1/TDRD7/DDX20/DZIP1
GOCC_CHROMOSOME_CENTROMERIC_REGION	GOCC_CHROMOSOME_CENTROMERIC_REGION	GOCC_CHROMOSOME_CENTROMERIC_REGION	42	-0.431968306	-3.00175845	1.16E-06	0.000283394	0.000236312	826	tags=71%, list=30%, signal=51%	SMARCC1/NUDCD2/SMC6/BUB1B/UHRF2/CBX1/NUP107/AURKC/CENPM/CDCA8/CENPH/SUV39H2/PINX1/ZNF330/TPR/DYNC1LI1/PAFAH1B1/PPP1CC/SMC3/STAG3/BRD7/NDE1/SMC1B/CENPE/SPAG5/ZW10/ITGB3BP/C1orf112/AHCTF1/SUGT1
GOCC_NUCLEAR_CHROMOSOME	GOCC_NUCLEAR_CHROMOSOME	GOCC_NUCLEAR_CHROMOSOME	32	-0.486587406	-3.006351946	1.34E-06	0.000308859	0.000257546	576	tags=62%, list=21%, signal=50%	RUVBL1/HSPA2/BLM/TIMELESS/PINX1/POLA2/FKBP6/TUBG1/SMC3/STAG3/SETX/PRIM1/RAD51/SMC1B/ARID4B/SMCHD1/SAP30/RAD50/ACTR6/PCNA
GOBP_MICROTUB	GOBP_MICROTUB	GOBP_MICROTUB	150	-0.253017392	-2.57495779	1.51E-06	0.000331	0.000276	517	tags=38%,	GSK3B/RAE1/RANBP1/NUSAP1/CDCA8/CENPH/K

ULE_BASED_PROCESS	ULE_BASED_PROCESS	ULE_BASED_PROCESS					255	221		list=19%, signal=33%	IF23/TPR/KATNA1/DYNC1LI1/IFT122/WDR62/SLK/PAFAH1B1/CATSPER2/CNTN2/CEP350/TUBG1/CEP250/CDC14B/ATXN3/SMC3/ZMYND10/DNAH8/CCNB2/NDE1/IQCF1/PRKAA1/DNAH17/DYNLL2/SPA17/CENPE/AKAP4/FKBP4/KIAA0753/SPAG5/ROPN1L/BBS4/MKKS/ZW10/DNAI1/CLUAP1/TTC21A/CDK5RAP2/IQCG/PARD6A/IFT20/KIF3A/KATNB1/DYNLRB2/LZTFL1/SPATA7/SUGT1/CEP63/DZIP1/IFT74/EFHC1
GOCC_CONDENSED_CHROMOSOME	GOCC_CONDENSED_CHROMOSOME	GOCC_CONDENSED_CHROMOSOME	47	-0.407024135	-2.92862689	1.82E-06	0.000379689	0.000316609	826	tags=68%, list=30%, signal=48%	SMARCC1/NUDCD2/SMC6/NOL6/BUB1B/NUP107/AURKC/HSPA2/BLM/CENPM/CENPH/PINX1/TPR/DYNC1LI1/FKBP6/PAFAH1B1/PPP1CC/TUBG1/SMC3/STAG3/BRD7/NDE1/RAD51/SMC1B/CENPE/SPAG5/ZW10/ITGB3BP/C1orf112/RAD50/AHCTF1/SUGT1
GOMF_SIGNALING_RECEPTOR_BINDING	GOMF_SIGNALING_RECEPTOR_BINDING	GOMF_SIGNALING_RECEPTOR_BINDING	238	0.23724721	2.382959889	3.04E-06	0.000579959	0.000483606	1572	tags=76%, list=58%, signal=36%	DDX54/LRP6/ARHGEF1/WNT10A/ABL1/TNF/MMP14/TIMP1/GPRC5B/TMED1/STUB1/AP2M1/ARF4/ITGA3/STAT3/TAP1/PRMT2/TRIP6/LTB/BOK/SYK/ADAM15/TACC1/GNA11/SEMA6C/TRAK1/ITGAM/MTSS1/ANG/FYN/UCN/DNAJC14/CTSG/WNT3A/ITGB1BP1/RASL11B/MSN/CDK5/CMTM7/CXCL13/DERL1/PTN/GDF1/PDGFRB/PTCH2/APLN/ITGA9/RABEP2/ITGB1/ITGB7/FGFR1/FLT3LG/SHC1/PTPRC/EFNB1/REEP2/SERPINE2/GPRASP2/NTF3/GRIPAP1/MICA/FBN1/SMAD7/SLURP1/LRP1/FGF1/FGF3/CD4/BCAP31/IL1R1/RLN3/ISG15/PLAT/ADAM17/CCL22/THY1/AMN/S100A4/ARR3/ELMO2/GNA12/DIAPH1/TYK2/SCP2/TSHB/ARRB2/WNT2B/NPFF/TNXB/SMO/SQSTM1/MCHR1/HMGB1/VWF/COL3A1/GFRA3/UCN3/ITGA6/EDN1/LRG1/IL17A/GALNT11/SLA/RALA/SEMA4B/AIP/HLA-A/SIX3/UNC93B1/MDM2/SNX17/SELPLG/LHB/PCSK1N/SSTR3/GAL/HLA-E/SHANK1/SOCS1/WNT1/HLA-B/PTPN1/TYROBP/DHH/TRAF1/HLA-F/LAG3/IGFBP4/PALM/ERBB2/FRS2/TGFBI/TNFSF10/SH3BP1/ARNT2/IGF2/GNAI1/JAKMIP1/EDA/CR2/ENG/CD177/PYCARD/ACTN2/DAB2/BMP4/ITGB8/HLA-C/ADAMTS5/TRAF4/EMP2/EFNA2/AAK1/LYN/OGN/ADIPOQ/VEGFB/PLSCR1/EPHA4/CLSTN3/CPNE3/AQP1/C3/CCL5/BTN2A2/SEMA3C/FAM3C/TRAF5/NR1H2/TGFA/PTPN2/NPTN/TGFBRAP1/CCL8/ATXN2/WIPI1/LYNX1/IGHG1/FNTA/SH2B2/IGFBP6/DLG4

GOCC_CATALYTIC_COMPLEX	GOCC_CATALYTIC_COMPLEX	GOCC_CATALYTIC_COMPLEX	313	-0.184913601	-2.244599587	2.91E-06	0.000579959	0.000483606	812	tags=44%, list=30%, signal=35%	VCP/POLE/SMC6/PPP2R2B/TEX10/BUB1B/ENY2/ENO3/PIGP/NCK1/POLR1C/POP4/DDA1/ERCC3/SHARPIN/PHF21A/RANBP2/PSENEN/ADRM1/PFKM/GTF2H2/RNF7/CDK5R1/BCKDHA/ANKRD9/CRADD/DNTTIP1/ATP6V0A2/RFC1/KCTD10/CLPX/FZR1/ATG12/LSM3/PIGH/DCUN1D3/POLRMT/NDUFB6/TFIP11/UBE3A/MTA3/PSMC2/PPP3CB/POLR3E/RUVBL1/PAF1/RMND5B/KLHDC2/RRAGD/UXS1/KLHL8/SF3A1/ASB1/FBXO39/PAXIP1/GSK3B/UBE2N/BLM/SUZ12/ATP6V1F/RCHY1/RCOR3/CSNK2A2/TPR/POLA2/NDUFS7/DYNC1LI1/FBXL2/PLRG1/NHEJ1/NDUFC2/RMI1/PPWD1/PAFAH1B1/FBXO24/APH1B/PPP1CC/CKS2/PMPCB/TBK1/PRKAG2/GTF2H1/RNMT/SUPT3H/DR1/BRD7/DNAH8/CCNB2/C15orf48/FBXO7/TAF9/WDR26/PCGF6/PRIM1/PRKAA1/RAD51/TOP3A/TSSK2/TAF6/SAE1/DNAH17/PIK3CG/DYNLL2/UBE2D2/AKAP4/NDUFS6/DERL3/EXOSC8/NDUFA12/ARID4B/FBXL18/DNAI1/COX5A/SAP30/TSN/TAF10/RFC4/PSMD12/COX6A1/FBXO15/UBE2D3/PARD6A/TBPL1/BRD1/DERL2/CCNH/DYNLRB2/PSMA6/CAB39/ATP6V1E2/DNALI1/ACTR6/PCNA/SUGT1/ANAPC10/PSMF1/POLG2/CACYBP/PNPT1
HP_ABNORMAL_MALE_GERM_CELL_MORPHOLOGY	HP_ABNORMAL_MALE_GERM_CELL_MORPHOLOGY	HP_ABNORMAL_MALE_GERM_CELL_MORPHOLOGY	10	-0.772142595	-2.82914732	3.50E-06	0.000640851	0.000534381	631	tags=100%, list=23%, signal=77%	FKBP6/CATSPER2/KLHL10/DNAH8/DNAH17/TTC21A/DNALI1/DZIP1/IFT74
HP_ABNORMAL_SPERM_MOTILITY	HP_ABNORMAL_SPERM_MOTILITY	HP_ABNORMAL_SPERM_MOTILITY	14	-0.678460224	-2.892098424	6.14E-06	0.001078258	0.000899119	679	tags=86%, list=25%, signal=65%	FOXJ1/ALG9/CATSPER2/KLHL10/ZMYND10/DNAH8/DNAH17/DNAI1/TTC21A/DNALI1/DZIP1/IFT74
GOBP_REGULATION_OF_SYNAPSE_ASSEMBLY	GOBP_REGULATION_OF_SYNAPSE_ASSEMBLY	GOBP_REGULATION_OF_SYNAPSE_ASSEMBLY	13	0.673425728	2.64340454	6.69E-06	0.001119773	0.000933737	619	tags=85%, list=23%, signal=66%	EIF4G1/LRFN3/LRRN3/CHD4/WNT3A/CHRNB2/CLSTN1/ABI3/GHSR/NTNG2/AMIGO3
GOBP_DNA_METABOLIC_PROCESS	GOBP_DNA_METABOLIC_PROCESS	GOBP_DNA_METABOLIC_PROCESS	167	-0.22826413	-2.391214749	7.13E-06	0.001119773	0.000933737	682	tags=43%, list=25%, signal=34%	RAD54B/CCDC117/ALKBH3/RFC1/CHEK2/FZR1/RECQL4/CEBPG/POLRMT/TFIP11/ENDOG/CDC7/RUVBL1/CHD1L/TRAIP/TRIP13/RAD17/POLB/PAXIP1/UBE2N/DOT1L/BLM/MBD4/TIMELESS/RCHY1/GTPBP4/PINX1/POLA2/NHEJ1/RMI1/MAPK3/RNF138/GTF2H1/CDC14B/UBE2T/ATXN3/PTTG1/SMC3/TFDP1/ATR/SUPT3H/BRD7/TOP1MT/TAF9/SETX/PRIM1/RAD51/TOP3A/TAF6/MDC1/NUDT1/FAF1/ATF7IP/USP1/ARID4B/USP37/SMCHD1/TSN/TAF1

											0/RFC4/KPNA2/C1orf112/DONSON/WRN/UBE2D3/RAD50/CHAF1B/RLF/GFER/PCNA/GMNN/POLG2
GOBP_GENERATION_OF_NEURONS	GOBP_GENERATION_OF_NEURONS	GOBP_GENERATION_OF_NEURONS	244	0.229338806	2.31631274	7.14E-06	0.001119773	0.000933737	1086	tags=55%, list=40%, signal=36%	AKT1/CTTN/EIF4G1/LRP6/BAG5/PBX3/WNT10A/DAB1/DLX1/ISL2/ABL1/MAPK8IP2/ITM2C/GPRC5B/ACTL6B/CUL7/BRSK1/SDC4/BAX/TSPO/ARF4/RP1L1/GDI1/ITGA3/STAT3/MOV10/PLXNB2/CDK5R2/RAC1/ADRA2B/OPCML/KIF13B/PPP2R5B/TRIO/BRSK2/SEMA6C/TRAK1/KREMEN1/HMG20B/FYN/ARHGAP4/UCN/WNT3A/HEYL/CDK5/EMX1/ATF4/PTN/DGKG/CHRNB2/TOR1A/ETV5/AGER/NRP1/BLOC1S1/STX3/ITGB1/RAPH1/EIF2AK4/GPR37/KCNB1/FGFR1/EFNB1/IRX3/CDH23/ABI3/PBX2/FZD4/NTF3/NKX2-8/CNTNAP1/GLI2/NTNG2/PITX3/B3GNT2/AMIGO3/RAPGEF1/RAB8A/ADAM17/PAK6/NHLH2/THY1/TBC1D23/MCF2/WNT2B/ATCAY/DNM2/TNXB/SMO/RAB6B/PLXNA1/NEUROG1/EPHA8/HMGB1/CLN8/COL3A1/NKX2-5/RTN4RL2/GFRA3/ENC1/CDH4/ABI1/EOMES/EHMT2/RAB11A/ITGA6/EDN1/MINK1/SF3A2/NFE2L2/SEMA4B/FBXW8/NFIA/VAPA/NEUROD6/EP300/NF1/SDC2/SIX3/MDM2/PQBP1/CHRNA7/BCL2/MTPN/SCARB2/DGUOK/HS6ST1/TRIOBP/CPNE5/SHANK1/AUTS2/ATP1B2/WNT1
GOBP_CELL_CYCLE_CHECKPOINT_SIGNALING	GOBP_CELL_CYCLE_CHECKPOINT_SIGNALING	GOBP_CELL_CYCLE_CHECKPOINT_SIGNALING	40	-0.411904933	-2.812265479	9.05E-06	0.001282917	0.001069776	828	tags=70%, list=30%, signal=50%	BARD1/E2F1/BUB1B/CRADD/MAD2L1BP/CHEK2/TIPRL/FZR1/RINT1/TRIP13/RAD17/DOT1L/BLM/TIMELESS/CDCA8/TPR/DYNC1LI1/CRY1/CDC14B/ATR/RAD51/MDC1/ZW10/CDK5RAP2/DONSON/RAD50/INTS7/CEP63
GOBP_CELLULAR_PROCESS_INVOLVED_IN_REPRODUCTION_IN_MULTICELLULAR_ORGANISM	GOBP_CELLULAR_PROCESS_INVOLVED_IN_REPRODUCTION_IN_MULTICELLULAR_ORGANISM	GOBP_CELLULAR_PROCESS_INVOLVED_IN_REPRODUCTION_IN_MULTICELLULAR_ORGANISM	73	-0.317963299	-2.66539082	8.81E-06	0.001282917	0.001069776	570	tags=47%, list=21%, signal=38%	HSPA2/TRIP13/TIAL1/LYZL6/TNP2/TCP11/PAFAH1B1/CATSPER2/MAST2/ACRBP/KLHL10/TDRKH/CCNB2/ACTL7A/IQCF1/PAQR7/TSSK2/MYBL1/AKAP4/ROPN1L/BBS4/AFF4/MKKS/TTC21A/RFX2/IQCG/ZPBP2/STRBP/TBPL1/SPACA3/SRPK1/TDRD7/DDX20/DZIP1
GOBP_WOUND_HEALING	GOBP_WOUND_HEALING	GOBP_WOUND_HEALING	79	0.337312232	2.516236075	8.93E-06	0.001282917	0.001069776	994	tags=61%, list=36%, signal=40%	RHOC/KLKB1/PPARA/ENTPD2/ITPK1/CLDN3/TNF/TIMP1/SDC4/VPS4A/HSPB1/FCER1G/GATA4/SYK/ADRA2B/CLIC1/ADIPOR2/CTSG/WNT3A/DGKG/HPS6/TOR1A/ITGB1/ANXA6/SERPINE2/FGF1/SPRR3/PLAT/ADAM17/F2RL3/GNA12/CHMP7/ST3GAL4/ILK/VWF/COL3A1/MSX2/MERTK/DCBLD2/EDN1/LRG1/NFE2L2/CHMP6/NF1/F10/SERPINA1/YAP1/CLDN4

GOBP_POSITIVE_REGULATION_OF_SIGNALING	GOBP_POSITIVE_REGULATION_OF_SIGNALING	GOBP_POSITIVE_REGULATION_OF_SIGNALING	300	0.210629185	2.221593225	9.56E-06	0.001312806	0.0010947	1531	tags=72%, list=56%, signal=35%	AKT1/RHOC/DOK4/MAGED1/EXTL3/PTP4A3/ABL1/MAPK8IP2/TNF/RXRB/ITM2C/GPRC5B/CDH3/BAX/GADD45A/SLC35B2/P2RX2/PSMD9/STAT3/LSM14A/NADK/TRIP6/LTB/BOK/GATA4/RAC1/SYK/LTBR/ADRA2B/RNF185/PPP2R5B/LFNG/ING4/GNA11/APAF1/KLF2/ADIPOR1/PPP2R1A/MBD2/IRAK1/OSBP/TRIM16/FYN/BBC3/UCN/CC2D1A/GAPDH/TBL1X/LMCD1/WNT3A/ITGB1BP1/OCIAD2/PHB2/PTN/TRIM8/LY86/MID1/PDGFRB/CLSTN1/AGER/NRP1/STX3/ITGB1/NEK6/EIF2AK4/GPR37/FGFR1/NET1/SHC1/PTPRC/RWDD3/HAVCR2/SERPINE2/HCRTR1/GHSR/RGS14/NTF3/FGF1/LIMS2/CANT1/EIF2AK2/BAD/CD4/BCAP31/FBXW11/TSPAN5/IL1R1/CRHR2/ADAM17/RELA/NFKB1/CCL22/F2RL3/DIRAS1/S100A4/CTSK/GLI1/PLEKHA4/FAM107A/IQGAP3/TNIP2/TYK2/ARRB2/DAPK3/MIER1/TNXB/SMO/SQSTM1/ILK/EPHA8/CARD9/HMGB1/VWF/COL3A1/GATA5/TMEM101/MSX2/JRK/UCN3/GRM1/EDN1/MINK1/LRG1/CXXC5/RALA/OCIAD1/MEN1/VAPA/EP300/NF1/F10/CD300A/YAP1/NFAM1/CHRNA7/MAP2K3/UBE2B/ARHGEF3/GAL/TSHZ3/HCLS1/SHANK1/AUTS2/TEK/WNT1/RPS15/MOS/PTPN1/PUM1/RBP4/TYROBP/DHH/CA2/ITPR3/WNK1/IGFBP4/PRMT5/PPP3CA/ROR2/ERBB2/FRS2/TM2D3/TNFSF10/CASP4/CTH/IGF2/EDA/ENG/TTC23/P2RY8/ECE1/CTSC/PYCARD/HIPK2/SOX9/H19/DAB2/MYC/FIS1/BMP4/AGPAT1/TRAF4/EMP2/ABAT/AAK1/TRIM41/LYN/ADIPOQ/EPHA4/CLSTN3/SCHIP1/PRDM15/SPIN1/CAMTA1/IQGAP1/MMD2/TRPM4/ADA/TRIM44/C3/P2RY10/SRPX/GRIN2C/MAZ/CCL5/ITPR1/XBP1/TRAF5/PJA2/TGFA/CYFIP1/PTPN2/NPTN/GRIN1/CCL8
GOBP_NEUROGENESIS	GOBP_NEUROGENESIS	GOBP_NEUROGENESIS	289	0.214485172	2.232923488	1.01E-05	0.001338544	0.001116162	1086	tags=53%, list=40%, signal=35%	AKT1/CTTN/EIF4G1/LRP6/BAG5/PBX3/CSK/WNT10A/DAB1/DLX1/ISL2/ABL1/MAPK8IP2/NDRG1/TNF/MMP14/ITM2C/GPRC5B/CLCN2/ACTL6B/CUL7/BRSK1/SDC4/BAX/TSPO/ARF4/RP1L1/GDI1/ITGA3/STAT3/MOV10/PLXNB2/BOK/LAMC3/CDK5R2/RAC1/ADRA2B/OPCML/KIF13B/PPP2R5B/TRIO/BRSK2/SEMA6C/TRAK1/KREMEN1/HMG20B/FYN/ARHGAP4/UCN/WNT3A/PRX/HEYL/CDK5/EMX1/ATF4/PTN/DGKG/CHRNB2/TOR1A/ETV5/AGER/NRP1/BLOC1S1/STX3/ITGB1/RAPH1/EIF2AK4/GPR3

											7/KCNB1/FGFR1/NAP1L1/EFNB1/IRX3/SERPINE2/CDH23/ABI3/PBX2/RGS14/FZD4/NTF3/NKX2-8/CNTNAP1/LRP1/GLI2/NTNG2/PITX3/B3GNT2/AMIGO3/RAPGEF1/RAB8A/ADAM17/RELA/PAK6/NHLH2/THY1/TBC1D23/MCF2/IFNGR1/WNT2B/ATCAY/DNM2/TNXB/SMO/RAB6B/PLXNA1/NEUROG1/ILK/EPHA8/HMGB1/CLN8/COL3A1/NKX2-5/RTN4RL2/SIRT2/GFRA3/ENC1/CDH4/ABI1/EOMES/EHMT2/RAB11A/ITGA6/EDN1/MINK1/SF3A2/NFE2L2/SEMA4B/FBXW8/NFIA/VAPA/NEUROD6/EP300/NF1/SDC2/SIX3/YAP1/MDM2/PQBP1/CHRNA7/EIF2B1/BCL2/MTPN/MED12/SCARB2/DGUOK/HS6ST1/TRIOBP/CPNE5/SHANK1/AUTS2/ATP1B2/WNT1
GOCC_ACROSOMAL_VESICLE	GOCC_ACROSOMAL_VESICLE	GOCC_ACROSOMAL_VESICLE	29	-0.49856468	-2.950633408	1.21E-05	0.001519323	0.001266906	509	tags=62%, list=19%, signal=51%	LYZL6/CSNK2A2/TCP11/POMT1/ACRBP/ACTL7A/IQCF1/MORN3/TSSK2/NUDT1/ZPBP2/IFT20/SH3GL3/SPACA3/SPAG8/ATP6V1E2/TRIP11/IFT74
GOBP_CELL_CYCLE_PROCESS	GOBP_CELL_CYCLE_PROCESS	GOBP_CELL_CYCLE_PROCESS	239	-0.19028059	-2.204972086	1.21E-05	0.001519323	0.001266906	665	tags=38%, list=24%, signal=32%	VRK1/MAD2L1BP/CHEK2/TIPRL/USP8/FZR1/PIWIL2/AURKC/DCUN1D3/KIF4A/CCDC42/MTA3/CDC7/RINT1/PAF1/HSPA2/TRIP13/RAD17/CEP72/PPP6C/PAXIP1/RAE1/DOT1L/AURKAIP1/RANBP1/NUSAP1/BLM/TIMELESS/CENPM/CAPN3/CDCA8/CENPH/KIF23/CSNK2A2/PINX1/TPR/DYNC1LI1/WDR62/PLRG1/CDC25C/RMI1/PAFAH1B1/CPSF3/CKS2/CRY1/TUBG1/CEP250/CDC14B/MYH10/PTTG1/SMC3/TFDP1/ATR/STAG3/TDRKH/PTTG2/BRD7/CCNB2/NDE1/FBXO7/RAD51/SMC1B/TOP3A/MDC1/MYBL1/CENPE/KIAA0753/SPAG5/BBS4/USP37/ZW10/TAF10/ITGB3BP/CDK5RAP2/C1orf112/SH3GLB1/DONSON/WRN/RAD50/PARD6A/KLF11/AHCTF1/KIF3A/SRPK1/KATNB1/CCNH/INTS7/PCNA/GMNN/SUGT1/CEP63/EFHC1
GOCC_CILIARY_PLASM	GOCC_CILIARY_PLASM	GOCC_CILIARY_PLASM	18	-0.586226501	-2.825775259	1.58E-05	0.001932103	0.001611108	326	tags=61%, list=12%, signal=54%	DNAH8/ARFGEF2/DNAH17/WDR54/DNAI1/NME7/CCDC96/SPAG8/SPATA7/DNALI1/EFHC1
GOCC_MICROTUBULE_ORGANIZING_CENTER	GOCC_MICROTUBULE_ORGANIZING_CENTER	GOCC_MICROTUBULE_ORGANIZING_CENTER	146	-0.231751758	-2.36442202	2.00E-05	0.002256293	0.001881438	594	tags=39%, list=22%, signal=32%	KIFC3/VPS37A/RUVBL1/RRAGD/CEP72/GSK3B/RANBP1/UBN1/KIF23/KATNA1/DYNC1LI1/IFT122/WDR62/SPECC1/PAFAH1B1/SLC1A4/CEP290/CEP350/TUBG1/CEP250/CDC14B/CCHCR1/ZMYND10/AKAP11/CCNB2/NDE1/CCDC77/ARFGEF2/RAD51/TSSK2/DYNLL2/KIAA0753/SPAG5/CCDC112/BBS4/MKKS/DNAI1/CLUAP1/NME7/CDK5RAP2/CCDC96/WRN/IQCD/PARD6A/TBCCD1/IFT20/HMMR/KIF

											3A/KATNB1/DYNLRB2/RAB3IP/SPATA7/PCNA/CEP63/DZIP1/IFT74/EFHC1
GOCC_INTRACELLULAR_PROTEIN_CONTAINING_COMPLEX	GOCC_INTRACELLULAR_PROTEIN_CONTAINING_COMPLEX	GOCC_INTRACELLULAR_PROTEIN_CONTAINING_COMPLEX	162	-0.221086258	-2.297375438	1.93E-05	0.002256293	0.001881438	783	tags=47%, list=29%, signal=36%	BUB1B/ENY2/POLR1C/POP4/DDA1/ERCC3/SHARPIN/ADRM1/GTF2H2/RNF7/ANKRD9/CNOT6/KCTD10/FZR1/DCUN1D3/POLRMT/UBE3A/PSMC2/PPP3CB/POLR3E/RUVBL1/PAF1/RMND5B/KLHDC2/KLHL8/ASB1/FBXO39/GSK3B/UBE2N/RCHY1/CNOT7/KIF23/CSNK2A2/POLA2/FBXL2/NHEJ1/TRAPPC2/FBXO24/CKS2/PRKAG2/GTF2H1/RNMT/SUPT3H/DR1/FBXO7/TAF9/WDR26/PCGF6/PRIM1/PRKAA1/RAD51/CNOT10/TSSK2/TAF6/UBE2D2/AKAP4/DERL3/FBXL18/TSN/TAF10/PSMD12/FBXO15/NCBP2/UBE2D3/TBPL1/BRD1/DERL2/CCNH/PSMA6/WDR41/SUGT1/ANAPC10/PSMF1/DZIP1/POLG2/CACYBP
GOBP_CELL_ADHESION	GOBP_CELL_ADHESION	GOBP_CELL_ADHESION	266	0.216869161	2.217391344	1.97E-05	0.002256293	0.001881438	1490	tags=71%, list=55%, signal=36%	AKT1/CTTN/LRP6/CSK/PPARA/DAB1/ABL1/SIGLEC12/CLDN3/TNF/MMP14/LRFN3/ACTL6B/SDC4/CDH3/ITGA3/LGALS3BP/ARHGEF7/HSPB1/TRIP6/PLXNB2/LAMC3/RAC1/SYK/OPCML/MAGI1/ADAM15/PPP2R1A/INPPL1/ITGAM/MTSS1/IRAK1/CLDN15/FYN/CLIC1/CTSG/WNT3A/ITGB1BP1/MSN/CDK5/CXCL13/SERPINB8/TOR1A/PKP3/CLSTN1/AGER/NRP1/ITGA9/STX3/ITGB1/ITGB7/SHC1/PTPRC/EFNB1/HAVCR2/SERPINE2/PCDHGA8/CDH23/FZD4/FBN1/COL6A3/SMAD7/SLURP1/CNTNAP1/LIMS2/GLI2/NTNG2/BAD/BCR/AMIGO3/CD4/ADAM17/RELA/STAB1/F2RL3/THY1/MYADM/TRO/FAM107A/RSU1/TYK2/SART1/ST3GAL4/ATP1B1/BCAM/DAPK3/TNXB/FBLN2/PLXNA1/ILK/SPN/EPHA8/HMGB1/VWF/COL3A1/GATA5/MERTK/SPOCK2/COL15A1/SCGB1A1/CDH4/ITGA6/PARVA/CLDN5/MINK1/LRG1/HLA-DRB3/NF1/CD6/HLA-A/CD300A/CLDN4/FLOT2/MYO10/SELPLG/BCL2/HLA-E/TRIOBP/MMRN2/ATP1B2/SOCS1/TEK/WNT1/ZNF703/UMOD/PKP2/SNAI2/HLA-DRB5/KLHL25/HLA-DQB1/RGMB/WNK1/LAG3/NFASC/PPP3CA/GP1BB/AIF1/CDH11/COL4A6/CHST2/ERBB2/CHRD/TFE3/TGFBI/FOLR2/IGF2/ADAM12/EDA/ENG/CD177/PYCARD/GNE/SOX9/ACTN2/DAB2/AZGP1/HLA-DMB/BMP4/ITGB8/HLA-DOB/CEBPB/CELSR2/RCC2/EMP2/MCAM/HOXD3/LYN/VEZT/ADIPOQ/ALOX5/EPHA4/CLASP2/CLSTN3/METAP1/OMG/TNFR

											SF21/PERP/RAB1A/ADA/APOD/PCDHB16/SRPX/FXYD5/TMEM47/AMIGO2/PODXL/CCL5/BTN2A2/CGREF1
GOBP_REGULATION_OF_PROTEIN_LOCALIZATION_TO_MEMBRANE	GOBP_REGULATION_OF_PROTEIN_LOCALIZATION_TO_MEMBRANE	GOBP_REGULATION_OF_PROTEIN_LOCALIZATION_TO_MEMBRANE	39	0.440870808	2.598967778	2.27E-05	0.002496037	0.002081352	582	tags=56%, list=21%, signal=45%	AKT1/CSK/TNF/MMP14/AP2M1/GDI1/ITGA3/VPS4A/ITGAM/FYN/BBC3/WNT3A/ITGB1BP1/CDK5/CLN3/STX3/ITGB1/KCNB1/ABI3/GHSR/GRIPAP1/LRP1
GOBP_CELL_MORPHOGENESIS	GOBP_CELL_MORPHOGENESIS	GOBP_CELL_MORPHOGENESIS	156	0.261558426	2.370418836	2.45E-05	0.002564944	0.002138811	1072	tags=58%, list=39%, signal=38%	CTTN/DAB1/ISL2/ABL1/CLDN3/MAPK8IP2/CUL7/BRSK1/CDH3/HEG1/GDI1/MOV10/ARHGEF7/PLXNB2/LAMC3/CDK5R2/RAC1/KIF13B/TRIO/BRSK2/SEMA6C/TRAK1/KLF2/CAP1/FYN/ARHGAP4/BRWD3/WNT3A/MSN/CDK5/EMX1/ATF4/PTN/CHRNB2/ZMYM3/NRP1/ITGB1/BIN3/RAPH1/EIF2AK4/EFNB1/CDH23/ABI3/FZD4/NTF3/NKX2-8/CNTNAP1/GLI2/COL27A1/NTNG2/B3GNT2/RAB8A/ADAM17/PAK6/THY1/ZMPSTE24/GNA12/RHOG/DIAPH1/MCF2/DNM2/DAPK3/SMO/PLXNA1/ILK/EPHA8/RHOU/GFRA3/CDH4/ABI1/EDN1/PARVA/MINK1/SEMA4B/FBXW8/NFIA/EP300/SDC2/UNC93B1/YAP1/CDC42EP5/CLDN4/TBC1D20/MYO10/PQBP1/CHRNA7/BCL2/TRIOBP/CPNE5/SHANK1/AUTS2
GOBP_DNA_DAMAGE_RESPONSE	GOBP_DNA_DAMAGE_RESPONSE	GOBP_DNA_DAMAGE_RESPONSE	154	-0.228153853	-2.368819669	2.40E-05	0.002564944	0.002138811	704	tags=44%, list=26%, signal=35%	MAPKAPK2/CRADD/CBX1/RAD54B/CCDC117/ALKBH3/RFC1/VRK1/CHEK2/TIPRL/VAV3/FZR1/RECQL4/CEBPG/TFIP11/ENDOG/CDC7/RINT1/RUVBL1/CHD1L/TRAIP/TRIP13/RAD17/POLB/PAXIP1/UBE2N/DOT1L/BLM/GNL1/MBD4/TIMELESS/RCHY1/NHEJ1/RMI1/MAPK3/RNF138/CRY1/GTF2H1/CDC14B/UBE2T/ATXN3/PTTG1/SMC3/ATR/SUPT3H/BRD7/TAF9/SETX/RAD51/TOP3A/TAF6/MDC1/ELL3/NUDT1/USP1/SMCHD1/TAF10/RFC4/C1orf112/DONSON/WRN/UBE2D3/RAD50/CHAF1B/MDM4/INTS7/PCNA/CEP63
GOBP_NEURON_DEVELOPMENT	GOBP_NEURON_DEVELOPMENT	GOBP_NEURON_DEVELOPMENT	193	0.240587821	2.296732358	2.51E-05	0.002564944	0.002138811	1079	tags=56%, list=40%, signal=36%	AKT1/CTTN/BAG5/PBX3/DAB1/ISL2/ABL1/MAPK8IP2/ITM2C/ACTL6B/CUL7/BRSK1/SDC4/TSPO/ARF4/RP1L1/GDI1/ITGA3/MOV10/PLXNB2/CDK5R2/RAC1/OPCML/KIF13B/PPP2R5B/TRIO/BRSK2/SEMA6C/TRAK1/KREMEN1/FYN/ARHGAP4/UCN/WNT3A/CDK5/EMX1/PTN/DGKG/CHRNB2/TOR1A/AGER/NRP1/BLOC1S1/STX3/ITGB1/RAPH1/EIF2AK4/GPR37/KCNB1/EFNB1/CDH23/ABI3/PBX2/FZD4/NTF3/NKX2-8/CNTNAP1/GLI2/NTNG2/PITX3/B3GNT2/AMIGO3/RAPGEF1/RAB8A/ADAM17/PAK6/

GOBP_REGULATION_OF_CELL_JUNCTION_ASSEMBLY	GOBP_REGULATION_OF_CELL_JUNCTION_ASSEMBLY	GOBP_REGULATION_OF_CELL_JUNCTION_ASSEMBLY	38	0.435929611	2.565061686	2.91E-05	0.002841646	0.002369542	770	tags=63%, list=28%, signal=46%	NHLH2/THY1/TBC1D23/MCF2/ATCAY/DNM2/TNXB/SMO/RAB6B/PLXNA1/NEUROG1/EPHA8/HMGB1/RTN4RL2/GFRA3/ENC1/CDH4/ABI1/RAB11A/ITGA6/EDN1/MINK1/SF3A2/NFE2L2/SEMA4B/FBXW8/VAPA/NEUROD6/EP300/SDC2/MDM2/PQBP1/CHRNA7/BCL2/SCARB2/DGUOK/HS6ST1/TRIOBP/CPNE5/SHANK1/AUTS2/ATP1B2 EIF4G1/ABL1/CLDN3/TNF/MMP14/LRFN3/SDC4/RAC1/LRRN3/CHD4/WNT3A/ITGB1BP1/CHRNB2/CLSTN1/NRP1/IRX3/ABI3/GHSR/NTNG2/AMIGO3/RAPGEF1/THY1/FAM107A/DAPK3
GOBP_GERM_CELL_DEVELOPMENT	GOBP_GERM_CELL_DEVELOPMENT	GOBP_GERM_CELL_DEVELOPMENT	60	-0.327506479	-2.563937829	2.88E-05	0.002841646	0.002369542	643	tags=52%, list=24%, signal=40%	PIWIL2/CCDC42/HSPA2/TRIP13/TIAL1/TNP2/TCP11/PAFAH1B1/CATSPER2/ACRBP/KLHL10/TDRKH/ACTL7A/IQCF1/PAQR7/TSSK2/AKAP4/ROPN1L/BBS4/AFF4/MKKS/TTC21A/RFX2/IQCG/ZPBP2/STRBP/TBPL1/SRPK1/TDRD7/DDX20/DZIP1
GOBP_BEHAVIOR	GOBP_BEHAVIOR	GOBP_BEHAVIOR	116	0.279566859	2.327737376	3.27E-05	0.003103993	0.002588303	1119	tags=61%, list=41%, signal=38%	AKT1/EIF4G1/VWA1/GNG7/PBX3/VDAC1/PPARA/DAB1/ABL1/MAPK8IP2/BRSK1/TSPO/ARF4/SLC16A1/ITGA3/P2RX2/STAT3/SGSH/MBD2/FYN/MMP17/UCN/CDK5/PTN/CHRNB2/CLN3/AGER/APLN/ITGB1/EIF2AK4/PI3/GPR37/NPTX2/SERPINE2/CDH23/HCRTR1/GHSR/ATAD1/RGS14/FZD4/NTF3/SLURP1/PITX3/PAK6/APRT/NHLH2/ZMPSTE24/ARRB2/MTNR1A/MCHR1/NEUROG1/WFS1/CLN8/NTAN1/EGR1/AAAS/EHMT2/GRM1/CLDN5/ABHD12/EP300/TMOD1/NF1/ALDH1A3/CHRNA7/BCL2/GAL/SHANK1/ATP1B2/PUM1/CRHR1
GOCC_NUCLEAR_PROTEIN_CONTAINING_COMPLEX	GOCC_NUCLEAR_PROTEIN_CONTAINING_COMPLEX	GOCC_NUCLEAR_PROTEIN_CONTAINING_COMPLEX	215	-0.197070347	-2.203192745	3.39E-05	0.003103993	0.002588303	853	tags=48%, list=31%, signal=36%	KPNB1/POT1/PRPF40A/LSM6/BOP1/BARD1/SMARCC1/E2F1/POLE/DHX15/NOL6/TEX10/CREB3/BUB1B/ENY2/POLR1C/TCF12/ERCC3/PHF21A/RANBP2/GTF2H2/THOC7/PRPF38A/PARP11/NUP107/DNTTIP1/FZR1/LSM3/XPOT/CEBPG/TFIP11/MTA3/POLR3E/RUVBL1/PAF1/TLE4/CPSF2/SF3A1/PRPF40B/CLMN/PAXIP1/RAE1/INTS6/RANBP1/SUZ12/RGPD5/TIMELESS/RCOR3/TPR/POLA2/PLRG1/NHEJ1/PPWD1/SPAG4/CPSF3/UTP18/PRKRIP1/GTF2H1/RNMT/TFDP1/ATR/SUPT3H/DR1/NFYB/BRD7/CEBPZ/PDCD7/TAF9/PCGF6/NUP88/PRIM1/DHX16/RAD51/TAF6/SAE1/ELL3/GEMIN4/CSTF1/EXOSC8/AFF4/NUP155/ARID4B/SAP30/TAF10/RFC4/RAD50/BUD13/TBPL1/AHCTF1/BRD1/PRPF18/STAT4/CCNH/INTS7/SMAD2/DDX20/ACTR6/PCNA/ANAPC10/XP

											O4/SNUPN/NR1H3/ZC3H8
GOCC_MICROTUBULE_CYTOSKELETON	GOCC_MICROTUBULE_CYTOSKELETON	GOCC_MICROTUBULE_CYTOSKELETON	236	-0.184954467	-2.140342504	3.35E-05	0.003103993	0.002588303	594	tags=35%, list=22%, signal=30%	KIFC3/VPS37A/CDC7/RUVBL1/HSPA2/RRAGD/CEP72/POLB/GSK3B/RAE1/MAPKBP1/RANBP1/NUSAP1/UBN1/CDCA8/KIF23/PINX1/TPR/KATNA1/DYNC1LI1/IFT122/WDR62/SPECC1/PAFAH1B1/SLC1A4/HSPH1/MAST2/CEP290/CEP350/TUBG1/CEP250/CDC14B/SMC3/CCHCR1/DR1/ZMYND10/DNAH8/AKAP11/CCNB2/NDE1/CCDC77/ARFGEF2/RAD51/TSSK2/DNAH17/DYNLL2/CENPE/FKBP4/KIAA0753/SPAG5/CCDC112/BBS4/MKKS/ZW10/DNAI1/CLUAP1/NME7/CDK5RAP2/CCDC96/C1orf112/IQCG/STRBP/LYST/WRN/IQCD/PARD6A/TBCCD1/IFT20/HMMR/KIF3A/SPAG8/KATNB1/DYNLRB2/LZTFL1/RAB3IP/SPATA7/DNALI1/PCNA/CEP63/DZIP1/IFT74/EFHC1
GOCC_SYNAPSE	GOCC_SYNAPSE	GOCC_SYNAPSE	251	0.209693485	2.123745577	3.97E-05	0.003558339	0.002967166	665	tags=37%, list=24%, signal=31%	AKT1/CTTN/ATP2B3/EIF4G1/LRP6/FXR2/VDAC1/KCNJ9/SCN2B/CLTB/ATP1A3/CTBP1/ABL1/MAPK8IP2/LRFN3/BRSK1/AP2M1/ARF4/GDI1/SLC16A1/ITGA3/P2RX2/GNB1/RAB11FIP3/ARHGEF7/SLC32A1/PURA/PRR12/TMUB1/RAC1/KCTD12/GDI2/TRIO/GNA11/SYNGR2/PPP2R1A/PRIMA1/FYN/CNIH3/UCN/CHD4/SNTA1/CPLX2/WNT3A/KCNMB1/SLC29A1/CDK5/PHB2/ADD1/PTN/CACNB3/CHRNB2/TOR1A/SYP/CLN3/CLSTN1/AGER/NRP1/KCNAB2/STX3/ITGB1/GPR37/KCNB1/EFNB1/NPTX2/SERPINE2/CDH23/HCRTR1/ABI3/GHSR/ATAD1/RGS14/FZD4/NTF3/GRIPAP1/CAD/SV2A/CNTNAP1/DNAJC5/ARHGDIA/SLC6A17/NTNG2/BCR/RGS12/RAB8A/ELAVL1/CRHR2/PLAT/RELA/SNCB/PAK6/PRR7
GOCC_PLASMA_MEMBRANE_PROTEIN_COMPLEX	GOCC_PLASMA_MEMBRANE_PROTEIN_COMPLEX	GOCC_PLASMA_MEMBRANE_PROTEIN_COMPLEX	104	0.294051444	2.369434644	4.12E-05	0.003621423	0.003019769	1349	tags=72%, list=49%, signal=38%	LRP6/KCNAB3/GNG7/SCN2B/CLTB/ATP1A3/CDH3/AP2M1/ITGA3/GNB1/FCER1G/RAC1/SYK/GNA11/ITGAM/CNIH3/CALM3/SNTA1/WNT3A/KCNMB1/GJB1/CD79A/CACNB3/CHRNB2/KCNB2/KCNAB2/ITGA9/ITGB1/LIME1/KCNB1/ITGB7/SHC1/C8A/CDH23/ABCD4/LRP1/CD4/GJA4/GNA12/CACNA1E/TYK2/ATP1B1/KCNS2/KCNG1/KCNH4/GRIK5/HMGB1/KCNS3/C9/CDH4/GRM1/ITGA6/APH1A/HLA-DRB3/ABHD12/HLA-A/FLOT2/CHRNA7/HLA-E/ATP1B2/HLA-B/HLA-DRB5/HLA-DQB1/TRAF1/HLA-F/GP1BB/CDH11/ERBB2/GJC1/GNAI1/HLA-DMB/ITGB8/HLA-C/HLA-DOB/TRAF4
GOCC_MALE_GE	GOCC_MALE_GER	GOCC_MALE_GER	16	-0.578310725	-2.636568246	4.28E-05	0.003688	0.003076	570	tags=69%,	HSPA2/TRIP13/ACTL7A/RAD51/DAZAP1/TSN/TA

RM_CELL_NUCLEUS	M_CELL_NUCLEUS	M_CELL_NUCLEUS					921	053		list=21%, signal=55%	F10/TBPL1/SPAG8/TCFL5/PCNA
GOBP_MUSCLE_TISSUE_DEVELOPMENT	GOBP_MUSCLE_TISSUE_DEVELOPMENT	GOBP_MUSCLE_TISSUE_DEVELOPMENT	82	0.311350145	2.347259827	4.38E-05	0.003703597	0.00308829	1341	tags=74%, list=49%, signal=39%	SOX15/MAML1/PPARA/ABL1/CFL2/HEG1/P2RX2/GATA4/MEF2D/HMG20B/MYH11/VGLL4/WNT3A/HEYL/CDK5/PDGFRB/ITGB1/BIN3/POPDC2/IRX3/SMAD7/CNTNAP1/FGF3/MYOD1/ZMPSTE24/GLI1/EMD/MYL3/ARRB2/SMO/MAPK11/COL3A1/GATA5/NKX2-5/EGR1/MSX2/SIRT2/EOMES/HSD17B1/EDN1/EP300/NF1/YAP1/ELN/BCL2/MTPN/PKP2/RBP4/KEL/PPP3CA/FRS2/GJC1/FOSL2/IGF2/WT1/ENG/SOX9/ACTN2/BMP4/BTG2/VAMP5
GOBP_CELL_CYCLE	GOBP_CELL_CYCLE	GOBP_CELL_CYCLE	325	-0.169451595	-2.098992711	5.17E-05	0.004283656	0.00357198	665	tags=36%, list=24%, signal=31%	VRK1/MAD2L1BP/CHEK2/TIPRL/USP8/FZR1/PIWIL2/AURKC/DCUN1D3/KIF4A/CCDC42/BOLL/MTA3/CDC7/RINT1/RUVBL1/PAF1/HSPA2/MAPK6/TRIP13/RAD17/CEP72/PPP6C/PAXIP1/RAE1/DOT1L/AURKAIP1/RANBP1/NUSAP1/BLM/TIMELESS/CENPM/CAPN3/CDCA8/GTPBP4/CENPH/KIF23/SUV39H2/CSNK2A2/PINX1/TPR/KATNA1/DYNC1LI1/FKBP6/WDR62/PLRG1/CDC25C/RMI1/PAFAH1B1/MAPK3/ZMYND11/CPSF3/PPP1CC/CKS2/CRY1/TUBG1/CEP250/GTF2H1/CDC14B/NF2/MYH10/PTTG1/SMC3/TFDP1/ATR/STAG3/DR1/TDRKH/PTTG2/BRD7/CCNB2/NDE1/FBXO7/PDCD2L/RAD51/BIRC8/SMC1B/TOP3A/TAF6/MDC1/MYBL1/C6orf89/CENPE/KIAA0753/SPAG5/BBS4/USP37/ZW10/TAF10/ITGB3BP/SENP5/CDK5RAP2/C1orf112/SH3GLB1/DONSON/WRN/RAD50/EPB41L2/PARD6A/KLF11/AHCTF1/CHAF1B/KIF3A/SPAG8/SRPK1/KATNB1/MDM4/CCNH/INTS7/PCNA/GMNN/SUGT1/ANAPC10/CEP63/ASNS/PNPT1/EFHC1
GOBP_POSITIVE_REGULATION_OF_PROTEIN_LOCALIZATION_TO_MEMBRANE	GOBP_POSITIVE_REGULATION_OF_PROTEIN_LOCALIZATION_TO_MEMBRANE	GOBP_POSITIVE_REGULATION_OF_PROTEIN_LOCALIZATION_TO_MEMBRANE	21	0.530754553	2.544435954	5.31E-05	0.004318333	0.003600896	793	tags=76%, list=29%, signal=54%	AKT1/TNF/ITGA3/ITGAM/FYN/BBC3/WNT3A/ITGB1BP1/CDK5/CLN3/STX3/ITGB1/KCNB1/LRP1/RHOG/SQSTM1
GOBP_MICROTUBULE_CYTOSKELETON_ORGANIZATION	GOBP_MICROTUBULE_CYTOSKELETON_ORGANIZATION	GOBP_MICROTUBULE_CYTOSKELETON_ORGANIZATION	117	-0.243097819	-2.331907767	5.62E-05	0.004486255	0.00374092	817	tags=52%, list=30%, signal=38%	HOOK2/TUBG2/VCP/PDCD6IP/RBM14/MAPRE1/ESPL1/SPAST/SSNA1/CDK5R1/MAP6D1/FOXJ1/CHEK2/AURKC/KIF4A/CCDC42/CEP72/GSK3B/RAE1/RANBP1/NUSAP1/CDCA8/CENPH/KIF23/TPR/KATNA1/DYNC1LI1/WDR62/SLK/PAFAH1B1/CNTN2/CEP350/TUBG1/CEP250/CDC14B/ATXN3/SMC3/ZMYND10/DNAH8/CCNB2/NDE1/PRKAA1/DNAH17

											/CENPE/FKBP4/KIAA0753/SPAG5/BBS4/ZW10/DNAI1/CLUAP1/CDK5RAP2/IQCG/PARD6A/KIF3A/KATNB1/SPATA7/SUGT1/CEP63/DZIP1/EFHC1
GOCC_PRESYNAPTIC_ACTIVE_ZONE	GOCC_PRESYNAPTIC_ACTIVE_ZONE	GOCC_PRESYNAPTIC_ACTIVE_ZONE	16	0.583485617	2.478936693	5.95E-05	0.004665226	0.003890157	607	tags=75%, list=22%, signal=59%	CTBP1/LRFN3/BRSK1/ITGA3/SLC32A1/TRIO/PHB2/SYP/STX3/SV2A/CNTNAP1/NTNG2
GOBP_G_PROTEIN_COUPLED_RECEPTOR_SIGNALING_PATHWAY	GOBP_G_PROTEIN_COUPLED_RECEPTOR_SIGNALING_PATHWAY	GOBP_G_PROTEIN_COUPLED_RECEPTOR_SIGNALING_PATHWAY	166	0.238379032	2.198290729	6.32E-05	0.00486892	0.00406001	1825	tags=89%, list=67%, signal=31%	AKT1/CRTC3/GNG7/ARHGEF1/ENTPD2/GPRC5B/GNB1/XCR1/RAC1/KCTD12/ADRA2B/GPR39/GNA11/UCN/CALM3/OR2T4/MRGPRF/OR4D5/TAAR5/RGS6/DGKG/GRK1/SYP/PDGFRB/APLN/OR8H2/OR11L1/GPR37/RGS11/OR5F1/OR52N2/HCRTR1/GHSR/RGS14/FZD4/GPR78/OR8B8/RGS12/CRHR2/RELA/CCL22/F2RL3/OR52M1/ARR3/OR3A3/GNA12/TSHB/RRH/ARRB2/NPFF/SMO/MTNR1A/MCHR1/OR51S1/OR4K17/OR1D4/OR7G2/OR2B11/OR6V1/UCN3/GRM1/TAAR6/EDN1/UBQLN2/IGF2R/OR5V1/OR6Y1/OR52E4/PROKR2/LHB/PCSK1N/OR6X1/SSTR3/GAL/OR51L1/OPN1LW/GPR12/MAS1L/CRHR1/OR6C4/OR4M1/CA2/RGS10/ITPR3/CCR5/PRMT5/PPP3CA/TAS2R9/PALM/OR4D1/FRS2/GPR119/OR10H4/GNAI1/P2RY8/ECE1/OR8G1/ACTN2/OR6B2/OR6K2/HRH3/C3AR1/PDE4A/CELSR2/TAS2R39/YWHAB/OR51A7/OR6M1/OR9K2/OR8D1/KISS1R/ADA/C3/P2RY10/GPR83/OR52D1/CCL5/ITPR1/MRGPRX3/OR13C4/OPN1MW/CCL8/HTR1B/OR1E2/ROCK2/GPR135/RPH3AL/OR10A4/FZD9/PDCL/OR2H1/UCN2/NMUR1/GIPR/PENK/CXCR3/GPR157/FFAR2/RXFP3/PRKAR1A/INS/OR10T2/ABHD6/OR4S1/CCL21/OR13C8/CCR1/GPR21
GOBP_POSITIVE_REGULATION_OF_INTRACELLULAR_SIGNAL_TRANSDUCTION	GOBP_POSITIVE_REGULATION_OF_INTRACELLULAR_SIGNAL_TRANSDUCTION	GOBP_POSITIVE_REGULATION_OF_INTRACELLULAR_SIGNAL_TRANSDUCTION	159	0.245889359	2.239418331	6.75E-05	0.005114849	0.004265081	1531	tags=76%, list=56%, signal=36%	AKT1/RHOC/DOK4/EXTL3/PTP4A3/ABL1/MAPK8IP2/TNF/GPRC5B/BAX/GADD45A/SLC35B2/P2RX2/TRIP6/LTB/BOK/GATA4/RAC1/SYK/LTBR/ADRA2B/ING4/IRAK1/FYN/BBC3/UCN/CC2D1A/GAPDH/LMCD1/ITGB1BP1/PHB2/TRIM8/MID1/PDGFRB/AGER/NRP1/ITGB1/NEK6/GPR37/FGFR1/NET1/SHC1/PTPRC/HAVCR2/HCRTR1/NTF3/FGF1/CANT1/EIF2AK2/BAD/CD4/BCAP31/FBXW11/RELA/CCL22/F2RL3/DIRAS1/S100A4/IQGAP3/TNIP2/ARRB2/MIER1/ILK/EPHA8/CARD9/HMGB1/VWF/COL3A1/TMEM101/GRM1/EDN1/MINK1/CXXC5/VAPA/EP300/F10/CHRNA7/MAP2K3/ARHGEF3/HCLS1/AUTS2/TEK/RPS15/MOS/PTPN1/IGFBP4/PPP3CA/ROR2/E

											RBB2/FRS2/TNFSF10/CTH/IGF2/EDA/ENG/P2RY8/PYCARD/HIPK2/SOX9/H19/DAB2/MYC/FIS1/BMP4/TRAF4/LYN/ADIPOQ/SCHIP1/CAMTA1/IQGAP1/MMD2/ADA/TRIM44/P2RY10/MAZ/CCL5/TRAF5/PJA2/TGFA/PTPN2/CCL8
GOBP_ORGANIC_ACID_CATABOLIC_PROCESS	GOBP_ORGANIC_ACID_CATABOLIC_PROCESS	GOBP_ORGANIC_ACID_CATABOLIC_PROCESS	52	0.375937457	2.471512973	7.65E-05	0.005698358	0.004751647	485	tags=44%, list=18%, signal=37%	AKT1/ECHS1/ABCD1/ECH1/PPARA/SDSL/HMGCL/DCXR/GOT2/BCAT2/SLC16A1/AIG1/MTHFS/ALDH4A1/ILVBL/PGD/LPIN2/GLUD2/SORD/ACAA1/FAH/GPT2/ALDH6A1
GOCC_CHROMOSOME	GOCC_CHROMOSOME	GOCC_CHROMOSOME	303	-0.163929057	-1.972250419	7.95E-05	0.005818856	0.004852126	687	tags=36%, list=25%, signal=30%	CBX1/NUP107/DNTTIP1/FOXJ1/RFC1/CHEK2/DNTTIP2/RECQL4/HAT1/CEBPG/AURKC/PPHLN1/KIF4A/TFIP11/MTA3/NAP1L4/NANOG/RUVBL1/HSPA2/CHD1L/TRAIP/TRIP13/RAD17/PAXIP1/NUSAP1/BLM/RFX4/PINK1/SUZ12/TIMELESS/TNP2/CENPM/CDCA8/CENPH/SUV39H2/CSNK2A2/PINX1/ZNF330/TPR/POLA2/DYNC1LI1/SPHK2/FKBP6/NHEJ1/RMI1/PAFAH1B1/HSF2/ZMYND11/ZBTB32/RNF138/PPP1CC/TUBG1/SMC3/TFDP1/ATR/SUPT3H/STAG3/GRHL1/DR1/NFYB/BRD7/TOP1MT/NDE1/TAF9/SETX/PRIM1/PRKAA1/RAD51/SMC1B/TOP3A/TAF6/MDC1/ELL3/ZHX3/CENPE/SPAG5/EXOSC8/AFF4/ARID4B/ZW10/SMCHD1/SAP30/TAF10/RFC4/ITGB3BP/RFX2/EBNA1BP2/C1orf112/DONSON/WRN/RAD50/CCDC86/KLF11/AHCTF1/BRD1/CHAF1B/STAT4/SRPK1/PPP1R7/INTS7/TCFL5/SMAD2/ACTR6/PCNA/SUGT1/PWP1/NR1H3/ZC3H8/PAWR
GOBP_ORGANIC_ACID_METABOLIC_PROCESS	GOBP_ORGANIC_ACID_METABOLIC_PROCESS	GOBP_ORGANIC_ACID_METABOLIC_PROCESS	175	0.235481857	2.181900929	8.23E-05	0.005926	0.004941469	617	tags=37%, list=23%, signal=30%	AKT1/ECHS1/ABCD1/VDAC1/ECH1/PPARA/SDSL/HMGCL/CYP7A1/DCXR/PRMT3/GOT2/BCAT2/ACO1/MECR/IDH3G/DHTKD1/MGST2/GAMT/PLA2G4D/SLC16A1/AIG1/MTHFS/ZBTB7A/STAT3/OSBPL1A/ACY1/ASAH2/LARS2/SYK/ALDH4A1/GSS/PTGES/PDK2/ILVBL/ADIPOR1/OSBP/ADIPOR2/GAPDH/PGD/LTC4S/MID1IP1/LPIN2/GLUD2/SORD/ACAA1/FAH/ATF4/PSAT1/CLN3/GALK1/GPT2/PRKAB2/ALDH6A1/SLC6A8/ABCC10/CYP4B1/ABCD4/GHSR/CAD/ALOX5AP/HSD17B8/PYCR2/ICMT
GOCC_CONDENSED_CHROMOSOME_CENTROMERIC_REGION	GOCC_CONDENSED_CHROMOSOME_CENTROMERIC_REGION	GOCC_CONDENSED_CHROMOSOME_CENTROMERIC_REGION	28	-0.468583521	-2.768882114	8.55E-05	0.006057739	0.005051321	826	tags=75%, list=30%, signal=53%	SMARCC1/NUDCD2/BUB1B/NUP107/AURKC/CENPM/CENPH/PINX1/TPR/DYNC1LI1/PAFAH1B1/PPP1CC/BRD7/NDE1/CENPE/SPAG5/ZW10/ITGB3BP/C1orf112/AHCTF1/SUGT1
GOBP_CELL_JUNCTION_ASSEMBL	GOBP_CELL_JUNCTION_ASSEMBLY	GOBP_CELL_JUNCTION_ASSEMBLY	79	0.311912867	2.32676534	8.88E-05	0.006190966	0.005162415	675	tags=47%, list=25%,	CTTN/RHOC/EIF4G1/ABL1/CLDN3/TNF/MMP14/LRFN3/SDC4/CDH3/ARHGEF7/TRIP6/PLXNB2/RAC

Y										signal=36%	1/LSR/LRRN3/CLDN15/CHD4/WNT3A/ITGB1BP1/GJB1/CDK5/CHRNB2/PKP3/CLSTN1/NRP1/IRX3/ABI3/GHSR/SMAD7/CNTNAP1/NTNG2/BCR/AMIGO3/RAPGEF1/GJA4/THY1
GOMF_OXIDOREDUCTASE_ACTIVITY_ACTING_ON_CH_OH_GROUP_OF_DONORS	GOMF_OXIDOREDUCTASE_ACTIVITY_ACTING_ON_CH_OH_GROUP_OF_DONORS	GOMF_OXIDOREDUCTASE_ACTIVITY_ACTING_ON_CH_OH_GROUP_OF_DONORS	24	0.491576959	2.470675177	9.10E-05	0.006245784	0.005208125	1328	tags=96%, list=49%, signal=50%	KCNAB3/CTBP1/DCXR/IDH3G/AKR7A3/PGD/RDH5/SORD/KCNAB2/AKR7A2/HSD17B8/ADHFE1/CBR3/DHRS4/CBR1/HSD17B1/AKR1A1/IMPDH1/IMPDH2/AKR1C1/GPD1/IDH3B/LDHA
GOCC_POTASSIUM_CHANNEL_COMPLEX	GOCC_POTASSIUM_CHANNEL_COMPLEX	GOCC_POTASSIUM_CHANNEL_COMPLEX	14	0.607618211	2.459163671	9.76E-05	0.00659931	0.005502917	843	tags=86%, list=31%, signal=60%	KCNAB3/CALM3/KCNMB1/KCNB2/KCNAB2/KCNB1/KCNS2/KCNG1/KCNH4/GRIK5/KCNS3/CCDC51
GOBP_PROTEIN_MODIFICATION_BY_SMALL_PROTEIN_REMOVAL	GOBP_PROTEIN_MODIFICATION_BY_SMALL_PROTEIN_REMOVAL	GOBP_PROTEIN_MODIFICATION_BY_SMALL_PROTEIN_REMOVAL	18	-0.532860051	-2.568534085	0.000109508	0.007224057	0.00602387	812	tags=83%, list=30%, signal=59%	VCP/ENY2/USP31/ZRANB1/USP8/USP48/USP30/ATXN3/ITCH/SENP1/USP1/USP37/SENP5/USP6/USPL1
GOMF_ATP_DEPENDENT_ACTIVITY	GOMF_ATP_DEPENDENT_ACTIVITY	GOMF_ATP_DEPENDENT_ACTIVITY	97	-0.252521638	-2.299752262	0.000110178	0.007224057	0.00602387	694	tags=49%, list=25%, signal=38%	ERCC3/ABCA5/SPAST/TOR2A/RAD54B/ATP6V0A2/RFC1/CLPX/RECQL4/KIF4A/KIFC3/PSMC2/RUVBL1/HSPA2/CHD1L/TRIP13/RAD17/DHX30/BLM/ATP6V1F/KIF23/KATNA1/MYO9A/HSPH1/CLPB/MYH10/SMC3/DNAH8/SETX/DHX16/RAD51/SMC1B/SAE1/DNAH17/DDX52/ATAD2/CENPE/ATF7IP/SMCHD1/RFC4/WRN/RAD50/HSPA14/KIF3A/HSPA1L/DYNLRB2/ATP6V1E2/DDX20
GOBP_MEIOTIC_CELL_CYCLE	GOBP_MEIOTIC_CELL_CYCLE	GOBP_MEIOTIC_CELL_CYCLE	43	-0.369746726	-2.588847677	0.000112513	0.007228848	0.006027865	682	tags=58%, list=25%, signal=44%	RAD54B/FZR1/PIWIL2/AURKC/BOLL/HSPA2/TRIP13/FKBP6/CDC25C/RMI1/CKS2/TUBG1/PTTG1/SMC3/STAG3/TDRKH/PTTG2/CCNB2/RAD51/SMC1B/TOP3A/MYBL1/ZW10/RAD50/ANAPC10
GOCC_CENTROSOME	GOCC_CENTROSOME	GOCC_CENTROSOME	112	-0.238980514	-2.282292784	0.000113542	0.007228848	0.006027865	594	tags=40%, list=22%, signal=33%	KIFC3/VPS37A/RRAGD/CEP72/GSK3B/RANBP1/UBN1/KIF23/KATNA1/DYNC1LI1/WDR62/PAFAH1B1/SLC1A4/CEP290/CEP350/TUBG1/CEP250/CDC14B/ZMYND10/CCNB2/NDE1/CCDC77/DYNLL2/KIAA0753/SPAG5/CCDC112/BBS4/MKKS/DNAI1/CLUAP1/NME7/CDK5RAP2/WRN/PARD6A/TBCCD1/IFT20/HMMR/KIF3A/KATNB1/DYNLRB2/PCNA/CEP63/DZIP1/IFT74/EFHC1
GOBP_MEIOSIS_I_CELL_CYCLE_PROCESS	GOBP_MEIOSIS_I_CELL_CYCLE_PROCESS	GOBP_MEIOSIS_I_CELL_CYCLE_PROCESS	21	-0.485031794	-2.523025469	0.000116119	0.007287299	0.006076605	755	tags=71%, list=28%, signal=52%	ESPL1/RAD54B/PIWIL2/HSPA2/TRIP13/CDC25C/RMI1/CKS2/PTTG1/STAG3/PTTG2/CCNB2/RAD51/MYBL1/RAD50
GOBP_DNA_TEMPLATED_DNA_REPLICATION	GOBP_DNA_TEMPLATED_DNA_REPLICATION	GOBP_DNA_TEMPLATED_DNA_REPLICATION	33	-0.405769652	-2.526012447	0.000120704	0.007386873	0.006159636	671	tags=70%, list=25%, signal=53%	BARD1/POLE/NOC3L/RFC1/RECQL4/POLRMT/ENDOG/CDC7/TRAIP/POLB/BLM/TIMELESS/POLA2/ATR/PRIM1/RAD51/RFC4/DONSON/WRN/RAD50/

											PCNA/GMNN/POLG2
GOBP_ORGANELLE_FISSION	GOBP_ORGANELLE_FISSION	GOBP_ORGANELLE_FISSION	91	-0.261342165	-2.34692849	0.000121069	0.007386873	0.006159636	783	tags=52%, list=29%, signal=38%	BUB1B/PDCD6IP/MAPRE1/ESPL1/SPAST/REEP3/RAD54B/MAD2L1BP/CHEK2/FZR1/PIWIL2/AURKC/KIF4A/HSPA2/TRIP13/AURKAIP1/RANBP1/NUSAP1/PINK1/CDCA8/KIF23/PINX1/TPR/DYNC1LI1/CDC25C/RMI1/DDHD1/CKS2/TUBG1/CDC14B/PTTG1/SMC3/STAG3/TDRKH/PTTG2/CCNB2/NDE1/RAD51/PEX11G/MYBL1/CENPE/SPAG5/ZW10/CDK5RAP2/MTFR1/RAD50/KATNB1
GOMF_DNA_HELICASE_ACTIVITY	GOMF_DNA_HELICASE_ACTIVITY	GOMF_DNA_HELICASE_ACTIVITY	15	-0.574875625	-2.5405077	0.000126969	0.00764074	0.006371327	756	tags=80%, list=28%, signal=58%	ERCC3/RAD54B/RECQL4/RUVBL1/CHD1L/DHX30/BLM/SETX/RAD51/RFC4/WRN/RAD50
GOBP_CELLULAR_CATABOLIC_PROCESS	GOBP_CELLULAR_CATABOLIC_PROCESS	GOBP_CELLULAR_CATABOLIC_PROCESS	269	0.196841053	2.011730453	0.000133167	0.007905423	0.006592036	485	tags=28%, list=18%, signal=26%	AKT1/CTTN/EIF4G1/FXR2/ECHS1/ABCD1/VDAC1/GTPBP1/ECH1/PPARA/SDSL/ENTPD2/HMGCL/ENTPD8/ABL1/DCXR/DCPS/TNF/TIMP1/STUB1/GOT2/BCAT2/ITPA/EXOSC7/BAX/TSPO/MGST2/BLVRA/PLA2G4D/SLC16A1/AIG1/NT5C/MTHFS/STAT3/CDC37/MOV10/VPS4A/HSPB1/ENTPD6/FASTK/HBM/ASAH2/AUP1/BOK/GATA4/ALDH4A1/RNF185/SGSH/APAF1/ILVBL/PNPLA2/ANG/PRIMA1/SLC25A5/GAPDH/PGD/NTHL1/LPIN2/GLUD2/SORD/WDR6/ACAA1/FAH/CDK5/ZFYVE1/PHB2/TRIM8/MEFV/GRAMD1A/SEC22B/CLN3/PKP3/DKC1/SND1/GPT2/ALDH6A1
HP_DECREASED_FERTILITY_IN_MALES	HP_DECREASED_FERTILITY_IN_MALES	HP_DECREASED_FERTILITY_IN_MALES	27	-0.449864626	-2.616764616	0.000142583	0.008241685	0.006872432	505	tags=63%, list=18%, signal=52%	FOXJ1/AURKC/BLM/FKBP6/CATSPER2/UBE2T/STAG3/KLHL10/ZMYND10/DNAH8/RAD51/DNAH17/DNAI1/TTC21A/DNALI1/DZIP1/IFT74
GOBP_DNA_TEMPLATED_DNA_REPLICATION_MAINTENANCE_OF_FIDELITY	GOBP_DNA_TEMPLATED_DNA_REPLICATION_MAINTENANCE_OF_FIDELITY	GOBP_DNA_TEMPLATED_DNA_REPLICATION_MAINTENANCE_OF_FIDELITY	12	-0.637944638	-2.514840418	0.00014115	0.008241685	0.006872432	828	tags=92%, list=30%, signal=64%	BARD1/POLE/TRAIP/BLM/TIMELESS/ATR/RAD51/DONSON/WRN/RAD50/PCNA
GOBP_CELL_ACTIVATION	GOBP_CELL_ACTIVATION	GOBP_CELL_ACTIVATION	185	0.227909574	2.155149291	0.00014491	0.008267406	0.006893879	1356	tags=65%, list=50%, signal=35%	AKT1/CRTC3/SOX15/CSK/ENTPD2/ABL1/SUPT6H/NDRG1/TNF/MMP14/ACTL6B/SDC4/BAX/ZBTB7A/STAT3/HSPB1/PURA/FCER1G/SYK/LTBR/ADRA2B/TNFRSF4/KIF13B/LFNG/PRAM1/ITGAM/FYN/CLIC1/SLC25A5/CPLX2/CTSG/WNT3A/CD79A/MSN/CMTM7/PHB2/DGKG/CHRNB2/AGER/ITGB1/EIF2AK4/PTPRC/EFNB1/HAVCR2/SERPINE2/MICA/SMAD7/SLURP1/LRP1/GLI2/BAD/BCR/CD4/PLAT/ADAM17/PRR7/F2RL3/THY1/IFNGR1/TNIP2/TYK2/SART1/CEBPA/SMO/ILK/SPN/HMGB1/VWF/COL3

											A1/EGR1/MERTK/SCGB1A1/EOMES/EDN1/IL17A/HLA-DRB3/MEN1/EP300/CD6/HLA-A/CD300A/NFAM1/MDM2/FLOT2/CHRNA7/SELPLG/RPS6KA1/BCL2/IMPDH2/GAL/HLA-E/SOCS1/WNT1/HLA-DRB5/KLHL25/TYROBP/SLC11A1/HLA-DQB1/HLA-F/LAG3/HSH2D/PRMT5/PPP3CA/GP1BB/AIF1/ERBB2/PRDX1/FOSL2/IGF2/CR2/CTSC/CD177/PYCARD/MAFB/HLA-DMB/BMP4/ITGB8/POU2AF1/HLA-DOB/CEBPB/EMP2
GOBP_ORGANELLE_ASSEMBLY	GOBP_ORGANELLE_ASSEMBLY	GOBP_ORGANELLE_ASSEMBLY	191	-0.189651726	-2.076827411	0.000147502	0.008307373	0.006927207	662	tags=38%, list=24%, signal=31%	CHEK2/IQCB1/ATG12/LSM3/AURKC/KIF4A/CCDC42/VPS37C/OGFOD1/VPS37A/TBC1D1/CEP72/EIF5B/DHX30/ATG9A/GSK3B/RFX4/PINK1/CAPN3/CDCA8/CNOT7/CENPH/KIF23/TPR/IFT122/WDR62/PAFAH1B1/NIP7/CEP290/TBC1D3/CEP350/CEP250/CDC14B/ACRBP/NF2/EDC3/SMC3/STX18/ZMYND10/DNAH8/CCNB2/ACTL7A/PRKAA1/EIF5/DNAH17/CSRP2/SPATA6/CENPE/AKAP4/KIAA0753/SPAG5/BBS4/MKKS/EIF2S1/WDR54/DNAI1/CLUAP1/RFX2/CDK5RAP2/CCDC96/IQCG/ZPBP2/SH3GLB1/RAB8B/IFT20/TBPL1/KIF3A/RAB3IP/SUGT1/CEP63/DZIP1/IFT74
GOBP_INTEGRIN_MEDIATED_SIGNALING_PATHWAY	GOBP_INTEGRIN_MEDIATED_SIGNALING_PATHWAY	GOBP_INTEGRIN_MEDIATED_SIGNALING_PATHWAY	29	0.445441161	2.404570287	0.000160396	0.008919261	0.007437437	898	tags=69%, list=33%, signal=47%	CCM2/ABL1/TIMP1/ITGA3/SYK/ADAM15/PRAM1/ITGAM/ITGB1BP1/PTN/NRP1/ITGA9/ITGB1/ITGB7/LIMS2/ISG15/THY1/ILK/COL3A1/ITGA6
GOBP_CILIUM_ORGANIZATION	GOBP_CILIUM_ORGANIZATION	GOBP_CILIUM_ORGANIZATION	75	-0.282969662	-2.35840427	0.000167546	0.008975962	0.007484718	390	tags=35%, list=14%, signal=31%	CEP290/TBC1D3/CEP350/CEP250/CDC14B/ZMYND10/DNAH8/DNAH17/SPATA6/AKAP4/KIAA0753/BBS4/MKKS/WDR54/DNAI1/CLUAP1/TTC21A/RFX2/CCDC96/IQCG/RAB8B/IFT20/KIF3A/RAB3IP/DZIP1/IFT74
GOMF_SALT_TRANSMEMBRANE_TRANSPORTER_ACTIVITY	GOMF_SALT_TRANSMEMBRANE_TRANSPORTER_ACTIVITY	GOMF_SALT_TRANSMEMBRANE_TRANSPORTER_ACTIVITY	89	0.279901523	2.168043139	0.00016656	0.008975962	0.007484718	1702	tags=89%, list=62%, signal=35%	SEC61A1/ATP2B3/KCNAB3/KCNJ9/SCN2B/ATP1A3/CLCN2/SLC16A1/SLC12A9/CLIC1/SLC25A5/ATP7B/ATP2A3/KCNMB1/SLC29A1/SLC41A3/SLC12A8/SLC25A22/CACNB3/KCNB2/KCNAB2/CLIC5/TRPM2/SLC6A8/KCNB1/ANXA6/SLC5A11/CACNA1E/KCNS2/KCNG1/KCNH4/GRIK5/KCNS3/ATP2A1/CDC51/SLC25A29/SLC23A3/KCNK5/SLC30A9/CLCA4/CLDN4/CHRNA7/SLC17A8/SLC13A4/SLC26A1/ABCG2/SLC34A2/SLC4A5/SLC9A8/ITPR3/AQP6/SLC25A12/KCNK7/SLC25A23/SLC44A1/SLC10A5/KCNH6/SLC34A3/KCNJ1/AQP1/CLCN7/CACNA1B/SLC16A11/TRPM4/TPCN1/GRIN2C/ITPR1/SLC17A7/

GOBP_MONOATOMIC_ION_TRANSPORT	GOBP_MONOATOMIC_ION_TRANSPORT	GOBP_MONOATOMIC_ION_TRANSPORT	213	0.219846148	2.144324972	0.00016393	0.008975962	0.007484718	1184	tags=57%, list=43%, signal=35%	GRIN1/ANXA9/SLC34A1/SLC13A2/HTR1B/SLC30A3/TMCO3/HCN3/CLCC1/KCNMB3/ATP1A2AKT1/SEC61A1/ATP2B3/KCNAB3/VDAC1/KCNJ9/SCN2B/ATP1A3/ABL1/SFXN3/CLCN2/BAX/TSPO/SLC16A1/P2RX2/COX8A/SLC12A9/TOMM40/STIM1/SLC32A1/XCR1/CABP5/CLDN15/FYN/CLIC1/SLC25A5/UCN/CALM3/ATP7B/SNTA1/ATP2A3/WNT3A/KCNMB1/CDK5/SLC41A3/SLC12A8/PHB2/ATF4/SLC25A22/CACNB3/ATP13A1/CHRNB2/KCNB2/PDGFRB/KCNAB2/CLIC5/TRPM2/ITGB1/SLC6A8/LIME1/KCNB1/ABCC10/PTPRC/ANXA6/SERPINE2/CDH23/KLHL3/OTOP2/SLC6A17/COX7A1/CLNS1A/CD4/STEAP3/SLC5A11/F2RL3/GJA4/THY1/ZMPSTE24/SLC22A7/COX15/DIAPH1/CDK2/CACNA1E/PPIF/ATP1B1/KCNS2/KCNG1/DNM2/KCNH4/MCHR1/GRIK5/LASP1/WFS1/KCNS3/ATP2A1/NKX2-5/CCDC51/EDN1/TMEM163/KCNK5/SLC30A9/CLCA4/COMMD9/CLDN4/CHRNA7/OXSR1/SLC17A8/BCL2/SLC13A4/GAL/OTOP3/SCARA5/SLC26A1/ATP1B2/CNKSR3/SLC31A1/UMOD/PKP2/CRHR1/SLC34A2/SLC4A5/SLC9A8/SLC11A1/CA2/ITPR3/MAOB/WNK1/KEL/CCR5/CYBRD1/PPP3CA/GP1BB
GOCC_EXTERNAL_ENCAPSULATING_STRUCTURE	GOCC_EXTERNAL_ENCAPSULATING_STRUCTURE	GOCC_EXTERNAL_ENCAPSULATING_STRUCTURE	93	0.285812535	2.227713603	0.00016972	0.008982869	0.007490478	1420	tags=74%, list=52%, signal=37%	VWA1/MMP23B/ENTPD2/MMP14/TIMP1/LGALS3BP/CPA3/ITIH5/LAMC3/LRRN3/ANG/ORM2/MMP17/PRELP/CTSG/ADAMTSL2/SERPINB8/CD248/PI3/ANXA6/EMILIN3/SERPINE2/FBN1/COL6A3/FGF1/COL27A1/PLAT/S100A4/MGP/ADAMTS7/WNT2B/BCAM/TNXB/COL4A2/FBLN2/SPN/VWF/COL3A1/RTN4RL2/SPOCK2/COL15A1/MUC2/ANGPTL4/PCOLCE/SERPINH1/SDC2/SERPINA1/ELN/HTRA1/PODN/MMP15/FBN3/MMRN2/MMP28/NYX/COL4A6/MMP11/TGFBI/LTBP1/CTSC/COL4A5/AZGP1/ADAMTS5/CBLN4/OGN/ADIPOQ/PLSCR1/PZP/EMID1
GOBP_CELL_GROWTH	GOBP_CELL_GROWTH	GOBP_CELL_GROWTH	86	0.294329918	2.254140448	0.00017261	0.009027067	0.007527333	656	tags=44%, list=24%, signal=35%	AKT1/CTTN/EIF4G1/PPARA/EXTL3/ABL1/MMP14/GDI1/SFN/PRMT2/CCDC85B/GATA4/ADAM15/ING4/SEMA6C/ADIPOR1/PPP2R1A/TSPYL2/ARHGAP4/UCN/VGLL4/WNT3A/SERTAD3/CDK5/EMX1/CLSTN1/NRP1/PTCH2/ITGB1/BIN3/RAPH1/EIF2AK4/NET1/SERPINE2/SMAD7/ENO1/ADAM17/PAK6
GOBP_MONOCARBOXYLIC_ACID_	GOBP_MONOCARBOXYLIC_ACID_C	GOBP_MONOCARBOXYLIC_ACID_CA	29	0.442195535	2.387049826	0.00018056	0.009331785	0.007781425	383	tags=48%, list=14%,	AKT1/ECHS1/ABCD1/ECH1/PPARA/DCXR/SLC16A1/AIG1/ILVBL/PGD/LPIN2/SORD/ACAA1/FAH

CATABOLIC_PROCESS	ATABOLIC_PROCESS	TABOLIC_PROCESS								signal=42%	
GOBP_ANIMAL_ORGAN_MORPHOGENESIS	GOBP_ANIMAL_ORGAN_MORPHOGENESIS	GOBP_ANIMAL_ORGAN_MORPHOGENESIS	183	0.220268878	2.074445496	0.000184061	0.009402086	0.007840047	1366	tags=66%, list=50%, signal=35%	ANKRD11/MDFI/FAM20C/PBX3/PPARA/WNT10A/MAGED1/DLX1/ABL1/TNF/MMP14/BAX/GAMT/HEG1/TLE2/STAT3/STIM1/GATA4/RAC1/SYK/MEF2D/ADAM15/LFNG/INPPL1/PAX4/LZTS2/WNT3A/HEYL/MSN/EMX1/PHB2/ATF4/PTN/NRP1/FGFR1/ANXA6/IRX3/CDH23/PBX2/KLHL3/FBN1/SMAD7/FGF1/LIMS2/GLI2/COL27A1/FGF3/PITX3/BCR/AHDC1/FBXW11/RELA/CDX4/NFKB1/THY1/ZMPSTE24/MGP/GLI1/PLEKHA4/MYL3/WNT2B/FOXE3/SMO/PLXNA1/NEUROG1/PAX9/ILK/COL3A1/GATA5/NKX2-5/MSX2/ETV7/CRIP1/ITGA6/EDN1/PARVA/CLDN5/TREH/SERPINH1/ZNF22/EP300/NF1/ALDH1A3/SIX3/YAP1/ELN/MDM2/TBC1D20/RPS6KA1/BCL2/MED12/TRIOBP/TEK/WNT1/PKP2/SNAI2/RBP4/AQP6/PPP3CA/FRS2/MAN2A1/PHACTR4/FOSL2/IGF2/WT1/EDA/ENG/FHL1/CTNNBIP1/HIPK2/SOX9/DAB2/MYC/MAFB/TMEM119/BMP4/ADAMTS5/CEBPB/CELSR2/EMP2/HOXD3
GOBP_REGULATION_OF_BLOOD_VESSEL_ENDOTHELIAL_CELL_MIGRATION	GOBP_REGULATION_OF_BLOOD_VESSEL_ENDOTHELIAL_CELL_MIGRATION	GOBP_REGULATION_OF_BLOOD_VESSEL_ENDOTHELIAL_CELL_MIGRATION	18	0.540533935	2.42851646	0.00018692	0.009438397	0.007870325	1074	tags=89%, list=39%, signal=54%	AKT1/ABL1/TNF/GADD45A/HSPB1/ITGB1BP1/VASH1/NRP1/FGFR1/ADAM17/HMGB1/CARD10/NFE2L2/NF1/MAP2K3/MMRN2
GOCC_RECEPTOR_COMPLEX	GOCC_RECEPTOR_COMPLEX	GOCC_RECEPTOR_COMPLEX	59	0.341473899	2.341395532	0.000192066	0.009588035	0.007995103	1375	tags=80%, list=50%, signal=40%	GPRC5B/ITGA3/P2RX2/PLXNB2/KCTD12/SYK/ITGAM/CNIH3/CD79A/CHRNB2/PDGFRB/NRP1/ITGA9/ITGB1/LIME1/GPR37/ITGB7/FGFR1/LRP1/CD4/AMN/TYK2/MTNR1A/PLXNA1/GRIK5/GFRA3/MERTK/GRM1/ITGA6/ABHD12/AIP/CHRNA7/PIGR/TEK/ITPR3/TRAF1/GP1BB/ROR2/ERBB2/GPR119/ARNT2/CR2/ENG/LRP8/ITGB8/TRAF4/LYN
GOBP_FERTILIZATION	GOBP_FERTILIZATION	GOBP_FERTILIZATION	24	-0.456069535	-2.530768161	0.000199021	0.009714412	0.008100484	603	tags=62%, list=22%, signal=49%	UBE3A/LYZL6/TNP2/CATSPER2/ACRBP/KLHL10/TDRKH/ACTL7A/IQCF1/SPA17/AKAP4/ZPBP2/SPACA3/SPAG8/HSPA1L
GOBP_MONOATOMIC_CATION_TRANSPORT	GOBP_MONOATOMIC_CATION_TRANSPORT	GOBP_MONOATOMIC_CATION_TRANSPORT	182	0.227121779	2.133375544	0.000197143	0.009714412	0.008100484	1184	tags=58%, list=43%, signal=35%	AKT1/SEC61A1/ATP2B3/KCNAB3/VDAC1/KCNJ9/SCN2B/ATP1A3/ABL1/BAX/TSPO/SLC16A1/P2RX2/COX8A/SLC12A9/STIM1/SLC32A1/XCR1/CABP5/FYN/SLC25A5/UCN/CALM3/ATP7B/SNTA1/ATP2A3/WNT3A/KCNMB1/CDK5/SLC41A3/SLC12A8/PHB2/ATF4/SLC25A22/CACNB3/ATP13A1/CHRNB2/KCNB2/PDGFRB/KCNAB2/TRPM2/ITGB1/SLC6A

											8/LIME1/KCNB1/PTPRC/ANXA6/SERPINE2/CDH23/KLHL3/OTOP2/SLC6A17/COX7A1/CD4/STEAP3/SLC5A11/F2RL3/GJA4/THY1/ZMPSTE24/COX15/DIAPH1/CDK2/CACNA1E/PPIF/ATP1B1/KCNS2/KCNG1/DNM2/KCNH4/MCHR1/WFS1/KCNS3/ATP2A1/NKX2-5/CCDC51/EDN1/TMEM163/KCNK5/SLC30A9/COMMD9/CHRNA7/OXSR1/SLC17A8/BCL2/SLC13A4/GAL/OTOP3/SCARA5/ATP1B2/CNKSR3/SLC31A1/UMOD/PKP2/CRHR1/SLC34A2/SLC4A5/SLC9A8/SLC11A1/ITPR3/WNK1/KEL/CCR5/CYBRD1/PPP3CA/GP1BB
GOMF_UBIQUITIN_LIKE_PROTEIN_PEPTIDASE_ACTIVITY	GOMF_UBIQUITIN_LIKE_PROTEIN_PEPTIDASE_ACTIVITY	GOMF_UBIQUITIN_LIKE_PROTEIN_PEPTIDASE_ACTIVITY	14	-0.600095157	-2.558048646	0.000214763	0.010199054	0.008504608	749	tags=86%, list=27%, signal=63%	USP31/ZRANB1/USP8/USP48/USP30/ATXN3/SENP1/USP1/USP37/SENP5/USP6/USPL1
GOCC_RNA_POLYMERASE_COMPLEX	GOCC_RNA_POLYMERASE_COMPLEX	GOCC_RNA_POLYMERASE_COMPLEX	22	-0.470288342	-2.5148674	0.000215548	0.010199054	0.008504608	782	tags=73%, list=29%, signal=52%	ENY2/POLR1C/ERCC3/GTF2H2/POLRMT/POLR3E/PAF1/POLA2/GTF2H1/SUPT3H/TAF9/PRIM1/TAF6/TAF10/TBPL1/CCNH
GOBP_SINGLE_FERTILIZATION	GOBP_SINGLE_FERTILIZATION	GOBP_SINGLE_FERTILIZATION	16	-0.535543597	-2.441589238	0.000215914	0.010199054	0.008504608	603	tags=69%, list=22%, signal=54%	UBE3A/LYZL6/TNP2/ACTL7A/IQCF1/SPA17/AKAP4/ZPBP2/SPACA3/SPAG8/HSPA1L
GOBP_TRANSMEMBRANE_TRANSPORT	GOBP_TRANSMEMBRANE_TRANSPORT	GOBP_TRANSMEMBRANE_TRANSPORT	242	0.198052868	1.998956863	0.000222291	0.010388538	0.008662612	1471	tags=68%, list=54%, signal=35%	AKT1/SEC61A1/ATP2B3/KCNAB3/ABCD1/VDAC1/KCNJ9/SCN2B/ATP1A3/ABL1/MAPK8IP2/SFXN3/TNF/PRAF2/SLC35D2/CLCN2/BAX/TIMM17B/SLC16A1/SLC35B2/P2RX2/COX8A/SLC12A9/TOMM40/STIM1/SLC14A2/SLC32A1/TAP1/XCR1/CABP5/RNF185/FYN/CLIC1/SLC25A5/CNIH3/CALM3/ATP7B/SNTA1/ATP2A3/WNT3A/KCNMB1/GJB1/SLC29A1/SLC41A3/SLC12A8/PHB2/SLC25A22/CACNB3/ATP13A1/CHRNB2/KCNB2/CLN3/KCNAB2/CLIC5/TRPM2/ITGB1/SLC7A4/SLC6A8/LIME1/KCNB1/ABCC10/PTPRC/ANXA6/ABCD4/OTOP2/SV2A/SLC6A17/COX7A1/BCR/STEAP3/SLC5A11/F2RL3/GJA4/THY1/SLC22A7/COX15/DIAPH1/CACNA1E/PPIF/ATP1B1/KCNS2/KCNG1/KCNH4/GRIK5/TOMM20/LASP1/RNF5/CLN8/KCNS3/ATP2A1/CCDC51/GRM1/SLC25A29/EDN1/TMEM163/SLC23A3/MINK1/SLC2A1/NFE2L2/KCNK5/SLC30A9/CLCA4/CLDN4/CHRNA7/OXSR1/SLC17A8/BCL2/SLC13A4/GAL/OTOP3/SCARA5/SLC26A1/SHANK1/ATP1B2/SLC31A1/UMOD/ABCG2/SLC1A5/CRHR1/SLC34A2/SLC4A5/SLC9A8/SLC11A1/CA2/SLC29A2/ITPR3/WNK1/KEL

											/CCR5/CYBRD1/AQP6/PPP3CA/GP1BB/SLC2A13/OAZ2/GJC1/SLC25A12/KCNK7/ABCB7/FHL1/ACTN2/SLC25A23/SLC44A1/AZGP1/SLC10A5/SLC25A36/SLC22A8/LYN/KCNH6/SLC34A3/KCNJ1/ADIPOQ/SLC6A19/AQP1/FXN/CLCN7/CACNA1B/SLC29A3/SLC16A11/TRPM4/C3/SLC22A11/TPCN1/GRIN2C/FXYD5
HP_X_LINKED_INHERITANCE	HP_X_LINKED_INHERITANCE	HP_X_LINKED_INHERITANCE	48	0.358816378	2.302477172	0.000224762	0.010393463	0.008666719	1298	tags=77%, list=48%, signal=41%	ATP2B3/ABCD1/GDI1/FAM50A/LAS1L/TBL1X/SSR4/BRWD3/GJB1/MSN/AP1S2/SYP/MID1/DKC1/SLC6A8/EFNB1/GPRASP2/BCAP31/RNF113A/ARR3/EMD/PRPS1/BCORL1/UBQLN2/PGK1/PQBP1/MED12/OPN1LW/LAGE3/NYX/COL4A6/TFE3/ABCB7/EDA/FHL1/COL4A5/PHKA2
GOCC_CELL_SURFACE	GOCC_CELL_SURFACE	GOCC_CELL_SURFACE	142	0.242768974	2.129085512	0.000229932	0.010521769	0.008773709	1532	tags=76%, list=56%, signal=35%	LRP6/TNF/GPRC5B/LRFN3/SDC4/HEG1/ITGA3/SLC32A1/XCR1/FCER1G/ENTPD6/PLXNB2/ADRA2B/TNFRSF4/ADAM15/ITGAM/IRAK1/WNT3A/CD79A/MSN/PHB2/HM13/CHRNB2/CLSTN1/AGER/CD248/ITGA9/ITGB1/GPR37/KCNB1/ITGB7/FLT3LG/PTPRC/EFNB1/HAVCR2/GHSR/FZD4/MICA/CD4/IL1R1/ENO1/PLAT/ADAM17/THY1/CD1B/CTSK/CYP2W1/ADAMTS7/AAMP/BCAM/SPN/HMGB1/RTN4RL2/GFRA3/ITGA6/DCBLD2/IL17A/COLEC11/IGF2R/RALA/F10/CD6/SDC2/HLA-A/CHRNA7/SCARA5/HLA-E/ATP1B2/TEK/WNT1/HLA-B/UMOD/ABCG2/TYROBP/CCR5/HLA-F/LAG3/ROR2/IL9R/FOLR2/ENG/ANTXR2/ECE1/LMAN2/TFPI/AZGP1/LRP8/ITGB8/HLA-C/VAMP5/PROM2/EMP2/MCAM/MPZL1/LYN/ADIPOQ/EPHA4/CLSTN3/KISS1R/ADA/C3/SLC22A11/SRPX/BTN2A2/TGFA/NPTN/GRIN1/LIPG
GOBP_POSITIVE_REGULATION_OF_CELL_ADHESION	GOBP_POSITIVE_REGULATION_OF_CELL_ADHESION	GOBP_POSITIVE_REGULATION_OF_CELL_ADHESION	94	0.279058678	2.1862305	0.000240244	0.010880338	0.009072705	1386	tags=72%, list=51%, signal=37%	AKT1/CSK/ABL1/TNF/ACTL6B/SDC4/ITGA3/ARHGEF7/RAC1/SYK/MAGI1/IRAK1/FYN/CTSG/WNT3A/ITGB1BP1/CXCL13/AGER/NRP1/STX3/PTPRC/EFNB1/HAVCR2/SMAD7/LIMS2/GLI2/BAD/CD4/RELA/THY1/MYADM/RSU1/TYK2/SART1/ST3GAL4/FBLN2/ILK/SPN/HMGB1/SPOCK2/ITGA6/HLA-DRB3/CD6/HLA-A/FLOT2/MYO10/HLA-E/TRIOBP/SOCS1/TEK/HLA-DRB5/KLHL25/HLA-DQB1/PPP3CA/AIF1/CHST2/ERBB2/CHRD/TFE3/IGF2/PYCARD/DAB2/HLA-DMB/HLA-DOB/EMP2/LYN/ALOX5/EPHA4
GOBP_EPITHELIU	GOBP_EPITHELIU	GOBP_EPITHELIU	219	0.208313074	2.048377342	0.0002551	0.011438	0.009537	1320	tags=63%,	AKT1/RHOC/LRP6/FAM20C/VDAC1/WNT10A/MA

M_DEVELOPMENT	M_DEVELOPMENT	M_DEVELOPMENT				64	133	83		list=48%, signal=35%	GED1/EXTL3/CCM2/ITPK1/ABL1/CLDN3/LBH/TNF/MMP14/CLCN2/BRSK1/SDC4/CDH3/BAX/HEG1/SFN/FOXQ1/SAFB2/TAGLN/LCE1A/PLXNB2/GATA4/RAC1/LFNG/BRSK2/LSR/APAF1/KLF2/MTSS1/TRIM16/LZTS2/WNT3A/HEYL/MSN/EMX1/ADAMTSL2/PHB2/ADD1/ATF4/TOR1A/SSBP3/NRP1/PTCH2/ITGB1/MSI1/IRX3/SERPINE2/CDH23/KLHL3/FZD4/SMAD7/FGF1/KRT14/GLI2/COL27A1/PITX3/BAD/BCR/RAPGEF1/SPRR3/ADAM17/RELA/KRT3/GJA4/ZMPSTE24/MYADM/GLI1/PLEKHA4/IQGAP3/DEAF1/DNASE1L2/WNT2B/CEBPA/FOXE3/SMO/PLXNA1/ILK/CNFN/GATA5/NKX2-5/MSX2/KRT85/CBR1/ABI1/EDN1/CLDN5/LRG1/LCE1D/IL17A/PGK1/RALA/EP300/TMOD1/NF1/ALDH1A3/SIX3/YAP1/CLDN4/TBC1D20/BCL2/MED12/GAL/TRIOBP/MMRN2/AKR1C1/WNT1/ELOVL1/ZNF703/UMOD/SNAI2/MAF/SLC4A5/CA2/PPP3CA/SOX21/CHRD/FRS2/VASP/PHACTR4/FOSL2/SH3BP1/WT1/EDA/ENG/CTNNBIP1/ECE1/SOX9/DAB2/MYC/MAFB/BMP4
GOMF_G_PROTEIN_COUPLED_RECEPTOR_ACTIVITY	GOMF_G_PROTEIN_COUPLED_RECEPTOR_ACTIVITY	GOMF_G_PROTEIN_COUPLED_RECEPTOR_ACTIVITY	97	0.275781278	2.178905146	0.000263307	0.011683922	0.009742784	1836	tags=94%, list=67%, signal=32%	GPRC5B/XCR1/ADRA2B/GPR39/OR2T4/MRGPRF/OR4D5/TAAR5/PDGFRB/OR8H2/OR11L1/GPR37/OR5F1/OR52N2/HCRTR1/GHSR/FZD4/GPR78/OR8B8/CRHR2/F2RL3/OR52M1/OR3A3/RRH/SMO/MTNR1A/MCHR1/OR51S1/OR4K17/OR1D4/OR7G2/OR2B11/OR6V1/GRM1/TAAR6/OR5V1/OR6Y1/OR52E4/PROKR2/OR6X1/SSTR3/GAL/OR51L1/OPN1LW/GPR12/MAS1L/CRHR1/OR6C4/OR4M1/CCR5/TAS2R9/OR4D1/GPR119/OR10H4/P2RY8/OR8G1/OR6B2/OR6K2/HRH3/C3AR1/CELSR2/TAS2R39/OR51A7/OR6M1/OR9K2/OR8D1/KISS1R/P2RY10/GPR83/OR52D1/MRGPRX3/OR13C4/OPN1MW/HTR1B/OR1E2/GPR135/OR10A4/FZD9/OR2H1/NMUR1/GIPR/CXCR3/GPR157/FFAR2/RXFP3/OR10T2/OR4S1/OR13C8/CCR1/GPR21/OR8S1
GOCC_CENTRIOLE	GOCC_CENTRIOLE	GOCC_CENTRIOLE	24	-0.448442757	-2.488446532	0.000286532	0.012587343	0.010496113	436	tags=54%, list=16%, signal=46%	WDR62/CEP290/CEP350/TUBG1/CEP250/CCHCR1/TSSK2/KIAA0753/BBS4/IFT20/KIF3A/CEP63/DZIP1
GOBP_BLOOD_VESSEL_ENDOTHELIAL_CELL_MIGRATION	GOBP_BLOOD_VESSEL_ENDOTHELIAL_CELL_MIGRATION	GOBP_BLOOD_VESSEL_ENDOTHELIAL_CELL_MIGRATION	22	0.490511786	2.3922333	0.000291308	0.012670463	0.010565423	1074	tags=82%, list=39%, signal=50%	AKT1/ABL1/TNF/GADD45A/HSPB1/ITGB1BP1/VASH1/CLN3/NRP1/ITGB1/FGFR1/ADAM17/HMGB1/CARD10/NFE2L2/NF1/MAP2K3/MMRN2

GOBP_REGULATION_OF_MULTICELLULAR_ORGANISMAL_DEVELOPMENT	GOBP_REGULATION_OF_MULTICELLULAR_ORGANISMAL_DEVELOPMENT	GOBP_REGULATION_OF_MULTICELLULAR_ORGANISMAL_DEVELOPMENT	238	0.201648707	2.025401185	0.000294724	0.01269336	0.010584517	1524	tags=71%, list=56%, signal=34%	AKT1/SOX15/FAM20C/PPARA/DAB1/EXTL3/DLX1/CCM2/ABL1/TNF/MMP14/RXRB/CLCN2/ACTL6B/CUL7/CDH3/TSPO/GDI1/GADD45A/SFN/STAT3/STIM1/HSPB1/PLXNB2/GATA4/SYK/SEMA6C/TRAK1/LRRN3/KLF2/TRIM16/ARHGAP4/VGLL4/WNT3A/HEYL/CDK5/VASH1/ADD1/ATF4/CXCL13/PTN/CLSTN1/ETV5/AGER/NRP1/PTCH2/ITGB1/NAP1L1/PTPRC/SERPINE2/GHSR/RGS14/FZD4/FBN1/SMAD7/FGF1/GLI2/PITX3/BAD/AMIGO3/CD4/OAS2/ISG15/MYOD1/RELA/STAB1/THY1/ZMPSTE24/CTSK/MGP/GLI1/MCF2/ADAMTS7/SART1/WNT2B/CEBPA/FOXE3/SMO/COL4A2/PLXNA1/PIAS3/MAPK11/HMGB1/TFPT/NKX2-5/MSX2/SIRT2/CDH4/EDN1/CLDN5/LRG1/IL17A/PGK1/ANGPTL4/PURB/NFE2L2/SEMA4B/FBXW8/NF1/YAP1/NFAM1/CHRNA7/GAL/HCLS1/MMRN2/SOCS1/TEK/WNT1/HLA-B/SNAI2/MAF/KLHL25/RBP4/ULK1/TYROBP/KLF10/WNK1/LAG3/PRMT5/PPP3CA/ROR2/ZFYVE27/ERBB2/MAN2A1/TFE3/CHODL/ADAM12/TCTA/WT1/ENG/CTNNBIP1/CTSC/FAIM/HIPK2/SOX9/MYC/MAFB/TMEM119/BMP4/LRP8/BTG2/ITGB8/C3AR1/EHMT1/CEBPB/EMP2/LYN/ADIPOQ/ALOX5/VEGFB/EPHA4/CLASP2/CLSTN3/TWIST1/AQP1/TNFRSF21/TRPM4/ADA/C3/ZNF488/PLAG1/AMIGO2/BTN2A2/SEMA3C/XBP1/CYFIP1/NOTUM/PTPN2/NPTN
GOCC_SIDE_OF_MEMBRANE	GOCC_SIDE_OF_MEMBRANE	GOCC_SIDE_OF_MEMBRANE	118	0.24932716	2.083799345	0.0003216	0.013716411	0.011437601	1375	tags=69%, list=50%, signal=36%	GNG7/TNF/AP2M1/HEG1/ITGA3/GNB1/XCR1/FCER1G/SYK/OPCML/TNFRSF4/GNA11/ITGAM/FYN/MMP17/CD79A/HM13/CHRNB2/KCNAB2/CD248/SAMD10/ITGA9/BLOC1S1/ITGB1/PTPN7/PTPRC/MICA/NTNG2/CD4/BCAP31/IL1R1/THY1/CD1B/CTSK/GNA12/PLEKHA4/TYK2/CHMP7/BCAM/SPPL2B/SPN/RTN4RL2/GFRA3/ITGA6/IL17A/COLEC11/CD52/TREH/HLA-DRB3/F10/HLA-A/PTP4A1/CHRNA7/HLA-E/ATP1B2/HLA-B/UMOD/PTPN1/ABCG2/HLA-DRB5/ITPR3/HLA-DQB1/RGMB/TRAF1/CCR5/HLA-F/LAG3/PPP3CA/PALM/IL9R/FOLR2/GNAI1/ENG/ECE1/CD177/TFPI/AZGP1/HLA-C/MCAM/EFNA2/LYN
GOBP_NEGATIVE_REGULATION_OF_CELL_CYCLE_P	GOBP_NEGATIVE_REGULATION_OF_CELL_CYCLE_P	GOBP_NEGATIVE_REGULATION_OF_CELL_CYCLE_PRO	61	-0.285712027	-2.265568089	0.000332826	0.014058697	0.01172302	828	tags=57%, list=30%, signal=41%	BARD1/E2F1/BUB1B/RBM14/ESPL1/CRADD/MAD2L1BP/CHEK2/TIPRL/FZR1/DCUN1D3/RINT1/TRIP13/RAD17/DOT1L/AURKAIP1/BLM/TIMELESS/CD

ROCESS	ROCESS	CESS									CA8/PINX1/TPR/DYNC1LI1/CRY1/CDC14B/ATR/BRD7/FBXO7/RAD51/MDC1/ZW10/CDK5RAP2/DONSON/RAD50/INTS7/CEP63
GOCC_MICROTUBULE_ASSOCIATED_COMPLEX	GOCC_MICROTUBULE_ASSOCIATED_COMPLEX	GOCC_MICROTUBULE_ASSOCIATED_COMPLEX	32	-0.387116534	-2.391776957	0.00034396	0.014390641	0.011999815	631	tags=56%, list=23%, signal=44%	AURKC/KIF4A/KIFC3/CDCA8/KIF23/TPR/KATNA1/DYNC1LI1/PAFAH1B1/DNAH8/NDE1/DNAH17/DYNLL2/DNAI1/KIF3A/KATNB1/DYNLRB2/DNALI1
GOCC_CATION_CHANNEL_COMPLEX	GOCC_CATION_CHANNEL_COMPLEX	GOCC_CATION_CHANNEL_COMPLEX	23	0.461764769	2.275304962	0.000352235	0.014597796	0.012172555	843	tags=70%, list=31%, signal=49%	KCNAB3/SCN2B/CALM3/KCNMB1/CACNB3/KCNB2/KCNAB2/KCNB1/CACNA1E/KCNS2/KCNG1/KCNH4/GRIK5/KCNS3/ATP2A1/CCDC51
GOBP_INTERLEUKIN_1_PRODUCTION	GOBP_INTERLEUKIN_1_PRODUCTION	GOBP_INTERLEUKIN_1_PRODUCTION	19	0.507782426	2.335772422	0.000360455	0.014798874	0.012340226	1013	tags=84%, list=37%, signal=53%	TNF/STAT3/HSPB1/TRIM16/ORM2/MEFV/AGER/HAVCR2/GHSR/RELA/ARRB2/HMGB1/EGR1/RAD21/IL17A/CHRNA7
GOBP_POSITIVE_REGULATION_OF_MULTICELLULAR_ORGANISMAL_PROCESS	GOBP_POSITIVE_REGULATION_OF_MULTICELLULAR_ORGANISMAL_PROCESS	GOBP_POSITIVE_REGULATION_OF_MULTICELLULAR_ORGANISMAL_PROCESS	285	0.18653452	1.933100206	0.000368263	0.014979445	0.012490798	1395	tags=63%, list=51%, signal=34%	AKT1/CTTN/SOX15/FAM20C/CSK/MAGED1/ABL1/LBH/TNF/MMP14/RXRB/GPRC5B/CLCN2/ACTL6B/CUL7/TSPO/HEG1/GDI1/STAT3/STIM1/HSPB1/FCER1G/LTB/PLXNB2/GATA4/SYK/ADRA2B/TNFRSF4/TRAK1/LRRN3/ADIPOR1/ITGAM/IRAK1/TRIM16/FYN/ORM2/UCN/ADIPOR2/GAPDH/WNT3A/GPSM3/ATF4/PTN/CHRNB2/MEFV/CLSTN1/ETV5/AGER/NRP1/APLN/ITGB1/PRKAB2/NAP1L1/PTPRC/EFNB1/HAVCR2/SERPINE2/GHSR/RGS14/FZD4/PRAP1/SMAD7/LRP1/FGF1/GLI2/EIF2AK2/BAD/AMIGO3/CD4/OAS2/IL1R1/ISG15/ENO1/PLAT/ADAM17/RELA/THY1/GLI1/IFNGR1/IQGAP3/TNIP2/TYK2/SART1/ST3GAL4/ARRB2/WNT2B/CEBPA/TNXB/SMO/PLXNA1/SPN/CARD9/MAPK11/HMGB1/GATA5/ATP2A1/NKX2-5/EGR1/MSX2/CDH4/RAD21/EDN1/LRG1/IL17A/ANGPTL4/NFE2L2/FBXW8/HLA-DRB3/CD6/HLA-A/UNC93B1/YAP1/NFAM1/BCL7C/FLOT2/PQBP1/CHRNA7/RPS6KA1/BCL2/MTPN/GAL/HLA-E/HCLS1/MMRN2/SOCS1/TEK/WNT1/ZNF703/HLA-DRB5/KLHL25/TYROBP/SLC11A1/HLA-DQB1/KLF10/WNK1/HLA-F/PRMT5/PPP3CA/GP1BB/AIF1/ROR2/ZFYVE27/EBF2/FRS2/MAN2A1/CHODL/IGF2/ADAM12/WT1/ENG/CTNNBIP1/CTSC/CD177/PYCARD/FAIM/HIPK2/SOX9/H19/DAB2/MYC/TMEM119/HLA-DMB/BMP4/LRP8/AGPAT1/ITGB8/C3AR1/EHMT1/POU2AF1/HLA-DOB/CEBPB/EMP2/ABAT/LYN/ADIPOQ/ALOX5/VEGFB/EPHA4/CLSTN3/TWIST1
GOBP_REGULATI	GOBP_REGULATI	GOBP_REGULATIO	11	0.627089238	2.273661632	0.0003721	0.014998	0.012506	480	tags=73%,	AKT1/SDC4/ARHGEF7/RAC1/ARHGAP4/ITGB1BP

ON_OF_FIBROBLAST_MIGRATION	ON_OF_FIBROBLAST_MIGRATION	N_OF_FIBROBLAST_MIGRATION				55	856	983		list=18%, signal=60%	1/CLN3/ITGB1
GOBP_REGULATION_OF_PROTEIN_LOCALIZATION_TO_CELL_PERIPHERY	GOBP_REGULATION_OF_PROTEIN_LOCALIZATION_TO_CELL_PERIPHERY	GOBP_REGULATION_OF_PROTEIN_LOCALIZATION_TO_CELL_PERIPHERY	31	0.412217595	2.262138671	0.000377005	0.0150562	0.0125548	582	tags=55%, list=21%, signal=44%	AKT1/EIF4G1/CSK/TNF/MMP14/AP2M1/ITGA3/VPS4A/WNT3A/CDK5/CLN3/STX3/ITGB1/ABI3/GHSR/GRIPAP1/LRP1
HP_FETAL_ONSET	HP_FETAL_ONSET	HP_FETAL_ONSET	38	-0.358910718	-2.36697872	0.000398539	0.015643922	0.013044879	716	tags=58%, list=26%, signal=43%	PYCR1/VRK1/FZR1/RECQL4/LBR/TRAIP/CPSF3/CLPB/THSD1/PRKAG2/WNT3/CHRNG/NUP88/PRIM1/CENPE/KIAA0753/NUP155/PSMD12/DEF6/SLC25A19/ASNS/TRIP11
GOBP_MULTICELLULAR_ORGANISMAL_RESPONSE_TO_STRESS	GOBP_MULTICELLULAR_ORGANISMAL_RESPONSE_TO_STRESS	GOBP_MULTICELLULAR_ORGANISMAL_RESPONSE_TO_STRESS	18	0.525422807	2.360625029	0.000398843	0.015643922	0.013044879	151	tags=44%, list=6%, signal=42%	AKT1/EIF4G1/VWA1/GNG7/VDAC1/MAPK8IP2/TSPO/P2RX2
GOMF_CATALYTIC_ACTIVITY_ACTING_ON_DNA	GOMF_CATALYTIC_ACTIVITY_ACTING_ON_DNA	GOMF_CATALYTIC_ACTIVITY_ACTING_ON_DNA	43	-0.344955243	-2.415265689	0.00041839	0.016265384	0.013563094	682	tags=60%, list=25%, signal=46%	ISG20/POLE/ERCC3/RAD54B/ALKBH3/RFC1/RECQL4/ENDOG/RUVBL1/CHD1L/RAD17/DHX30/POLB/BLM/MBD4/CRY1/SMC3/TOP1MT/SETX/RAD51/TOP3A/RFC4/WRN/RAD50/PCNA/POLG2
GOCC_GERM_CELL_NUCLEUS	GOCC_GERM_CELL_NUCLEUS	GOCC_GERM_CELL_NUCLEUS	18	-0.499359768	-2.407053374	0.000431377	0.016623148	0.013861419	570	tags=61%, list=21%, signal=49%	HSPA2/TRIP13/ACTL7A/RAD51/DAZAP1/TSN/TAF10/TBPL1/SPAG8/TCFL5/PCNA
GOCC_CHROMOSOME_TELOMERIC_REGION	GOCC_CHROMOSOME_TELOMERIC_REGION	GOCC_CHROMOSOME_TELOMERIC_REGION	26	-0.430979288	-2.449608597	0.000441933	0.016881824	0.01407712	850	tags=73%, list=31%, signal=51%	POT1/SMC6/THOC7/CBX1/CHEK2/RECQL4/HAT1/TFIP11/RAD17/BLM/PINX1/PPP1CC/ATR/SETX/RAD51/SMCHD1/WRN/RAD50/PCNA
GOBP_POSITIVE_REGULATION_OF_LOCOMOTION	GOBP_POSITIVE_REGULATION_OF_LOCOMOTION	GOBP_POSITIVE_REGULATION_OF_LOCOMOTION	103	0.259639396	2.100655038	0.000455475	0.01724916	0.014383428	1506	tags=76%, list=55%, signal=35%	AKT1/RHOC/ABL1/CLDN3/TNF/MMP14/ITGA3/STAT3/ARHGEF7/HSPB1/TRIP6/RAC1/SEMA6C/ITGB1BP1/GPSM3/CXCL13/PTN/PDGFRB/AGER/NRP1/STX3/ITGB1/FGFR1/PTPRC/FZD4/NTF3/FGF1/IL1R1/ADAM17/THY1/MYADM/FAM107A/AAMP/DAPK3/SMO/SPN/HMGB1/GRB7/SPOCK2/RAB11A/ITGA6/EDN1/NFE2L2/SEMA4B/F10/CLDN4/MDM2/PTP4A1/OXSR1/MAP2K3/BCL2/TEK/ZNF703/SNAI2/WNK1/PPP3CA/AIF1/ROR2/PYCARD/SOX9/DAB2/BMP4/C3AR1/MCAM/LYN/VEGFB/EPHA4/CLASP2/TWIST1/CPNE3/AQP1/PHPT1/MALAT1/MAZ/PODXL/CCL5/SEMA3C/XBP1
GOMF_CYSTEINE_TYPE_PEPTIDASE_ACTIVITY	GOMF_CYSTEINE_TYPE_PEPTIDASE_ACTIVITY	GOMF_CYSTEINE_TYPE_PEPTIDASE_ACTIVITY	19	-0.481022105	-2.384686794	0.000465617	0.017334377	0.014454486	755	tags=74%, list=28%, signal=54%	ESPL1/USP31/ZRANB1/USP8/CAPN3/USP48/USP30/ATXN3/SENP1/USP1/USP37/SENP5/USP6/USPL1
GOMF_CATALYTIC_ACTIVITY_ACT	GOMF_CATALYTIC_ACTIVITY_ACT	GOMF_CATALYTIC_ACTIVITY_ACTI	97	-0.236012733	-2.149403198	0.00046546	0.017334377	0.014454486	818	tags=51%, list=30%,	ISG20/POLE/DUS3L/DHX15/POLR1C/POP4/ERCC3/DUS1L/CNOT6/RAD54B/ALKBH3/RFC1/METTL8/

ING_ON_A_NUCLEIC_ACID	ING_ON_A_NUCLEIC_ACID	NG_ON_A_NUCLEIC_ACID								signal=37%	PIWIL2/RECQL4/POLRMT/METTL6/TRIT1/ENDOG/RUVBL1/CHD1L/RAD17/DHX30/POLB/BLM/MBD4/CNOT7/CPSF3/CRY1/SMC3/RNMT/TOP1MT/SETX/PRIM1/DHX16/RAD51/TOP3A/NUDT1/DDX52/EXOSC8/NUDT4/RFC4/WRN/RAD50/DDX20/PCNA/POLG2/PNPT1/TRPT1
HP_ANTENATAL_ONSET	HP_ANTENATAL_ONSET	HP_ANTENATAL_ONSET	48	-0.323624975	-2.342656247	0.000473565	0.017482101	0.014577668	665	tags=52%, list=24%, signal=40%	VRK1/FZR1/RECQL4/LBR/TRAIP/NDUFC2/CPSF3/CLPB/THSD1/PRKAG2/WNT3/CHRNG/NUP88/PRIM1/TOP3A/CENPE/KIAA0753/NUP155/SEC24D/PSMD12/DEF6/GLUL/SLC25A19/ASNS/TRIP11
GOBP_DNA_REPLICATION	GOBP_DNA_REPLICATION	GOBP_DNA_REPLICATION	57	-0.30306626	-2.33109701	0.000489942	0.017797887	0.01484099	671	tags=51%, list=25%, signal=39%	RFC1/RECQL4/POLRMT/ENDOG/CDC7/RUVBL1/TRAIP/RAD17/POLB/BLM/TIMELESS/GTPBP4/POLA2/RMI1/SMC3/ATR/TOP1MT/PRIM1/RAD51/FAF1/USP37/RFC4/DONSON/WRN/RAD50/CHAF1B/PCNA/GMNN/POLG2
GOCC_COLLAGEN_CONTAINING_EXTRACELLULAR_MATRIX	GOCC_COLLAGEN_CONTAINING_EXTRACELLULAR_MATRIX	GOCC_COLLAGEN_CONTAINING_EXTRACELLULAR_MATRIX	73	0.290859165	2.111969691	0.000490222	0.017797887	0.01484099	1648	tags=86%, list=60%, signal=35%	VWA1/MMP23B/ENTPD2/TIMP1/LGALS3BP/CPA3/ITIH5/LAMC3/ANG/ORM2/PRELP/CTSG/SERPINB8/ANXA6/EMILIN3/SERPINE2/FBN1/COL6A3/COL27A1/PLAT/S100A4/MGP/WNT2B/BCAM/TNXB/COL4A2/FBLN2/SPN/VWF/COL3A1/COL15A1/MUC2/ANGPTL4/PCOLCE/SERPINH1/SDC2/SERPINA1/ELN/HTRA1/PODN/MMRN2/MMP28/COL4A6/TGFBI/LTBP1/CTSC/COL4A5/AZGP1/ADAMTS5/CBLN4/OGN/ADIPOQ/PLSCR1/PZP/SRPX/NAV2/ANXA9/CFP/C1QC/CDH2/LOXL2/EMILIN2/FGL2
GOBP_REGULATION_OF_PROTEIN_LOCALIZATION_TO_PLASMA_MEMBRANE	GOBP_REGULATION_OF_PROTEIN_LOCALIZATION_TO_PLASMA_MEMBRANE	GOBP_REGULATION_OF_PROTEIN_LOCALIZATION_TO_PLASMA_MEMBRANE	26	0.451095729	2.32531014	0.000502218	0.018083974	0.015079547	582	tags=58%, list=21%, signal=46%	AKT1/CSK/TNF/MMP14/AP2M1/ITGA3/VPS4A/WNT3A/CDK5/CLN3/STX3/ITGB1/ABI3/GRIPAP1/LRP1
GOMF_ATP_DEPENDENT_ACTIVITY_ACTING_ON_DNA	GOMF_ATP_DEPENDENT_ACTIVITY_ACTING_ON_DNA	GOMF_ATP_DEPENDENT_ACTIVITY_ACTING_ON_DNA	21	-0.449557844	-2.338498017	0.000525251	0.018759566	0.015642898	756	tags=67%, list=28%, signal=49%	ERCC3/RAD54B/RFC1/RECQL4/RUVBL1/CHD1L/RAD17/DHX30/BLM/SETX/RAD51/RFC4/WRN/RAD50
GOMF_KINASE_BINDING	GOMF_KINASE_BINDING	GOMF_KINASE_BINDING	162	0.222745254	2.041184394	0.00054257	0.019157148	0.015974427	832	tags=44%, list=30%, signal=32%	AKT1/RHOC/BAG5/CSK/VDAC1/MAML1/PPARA/ABL1/MAPK8IP2/GPRC5B/STUB1/BRSK1/SDC4/GADD45A/SFN/RHOBTB2/STAT3/DBF4B/CDC37/ARHGEF7/HSPB1/TRIP6/CDK5R2/GATA4/RAC1/SYK/WWC3/KIF13B/BRSK2/PRAM1/ADIPOR1/IRAK1/CALM3/ITGB1BP1/MSN/ATF4/PTN/TRIM8/PDGFRB/NRP1/ITGB1/PRKAB2/NEK6/LIME1/FLT3LG/SHC1/PTPRC/RGS14/SV2A/MTCP1/BAD/CD4/RAB8

											A/ELAVL1/RELA/PRR7/THY1/DIRAS1/PPP1R15A/ELMO2/AP1B1/RHOG/TNIP2/ATP1B1/ARRB2/CEBPA/SQSTM1/ILK/GRB7/TFPT/RHOU
GOBP_CELL_JUNCTION_ORGANIZATION	GOBP_CELL_JUNCTION_ORGANIZATION	GOBP_CELL_JUNCTION_ORGANIZATION	143	0.231489155	2.033930343	0.000545104	0.019157148	0.015974427	689	tags=39%, list=25%, signal=31%	AKT1/CTTN/RHOC/EIF4G1/CSK/CCM2/ABL1/CLDN3/TNF/MMP14/LRFN3/SDC4/CDH3/HEG1/ARF4/ITGA3/P2RX2/ARHGEF7/TRIP6/PLXNB2/RAC1/LSR/LRRN3/ITGAM/MTSS1/CLDN15/FYN/CHD4/SNTA1/WNT3A/ITGB1BP1/GJB1/CDK5/ADD1/CACNB3/CHRNB2/CLN3/PKP3/CLSTN1/NRP1/ITGB1/IRX3/ABI3/GHSR/GRIPAP1/SMAD7/CNTNAP1/LIMS2/NTNG2/BCR/AMIGO3/RAPGEF1/SNCB/GJA4/THY1/MYADM
GOBP_FIBROBLAST_MIGRATION	GOBP_FIBROBLAST_MIGRATION	GOBP_FIBROBLAST_MIGRATION	14	0.561453914	2.272326672	0.00055066	0.019198808	0.016009166	807	tags=79%, list=30%, signal=56%	AKT1/SDC4/ARHGEF7/RAC1/ARHGAP4/ITGB1BP1/CLN3/CD248/ITGB1/GNA12/ILK
GOBP_POSITIVE_REGULATION_OF_HYDROLASE_ACTIVITY	GOBP_POSITIVE_REGULATION_OF_HYDROLASE_ACTIVITY	GOBP_POSITIVE_REGULATION_OF_HYDROLASE_ACTIVITY	103	0.257523993	2.083540028	0.000570186	0.019723034	0.016446298	723	tags=44%, list=26%, signal=33%	AKT1/RHOC/CLDN3/TNF/BAX/ARF4/RALGDS/STAT3/ARHGEF7/BOK/SYK/APAF1/ANG/FYN/BBC3/CALM3/GRTP1/ATP2A3/RGS6/PHB2/CXCL13/MEFV/PDGFRB/AGER/ITGB1/ARHGAP27/RGS11/FGFR1/NET1/PTPRC/TBC1D2B/NTF3/BAD/BCR/BCAP31/RAPGEF1/PRR7/CCL22/THY1/CTSK/PPP1R15A/GNA12/RHOG/EVI5/RSU1
GOBP_COLLAGEN_METABOLIC_PROCESS	GOBP_COLLAGEN_METABOLIC_PROCESS	GOBP_COLLAGEN_METABOLIC_PROCESS	20	0.490247756	2.276252963	0.000582066	0.019976672	0.016657797	1320	tags=95%, list=48%, signal=49%	MMP23B/CYP7A1/MMP14/ADAM15/MMP17/UCN/ITGB1/CBX8/LARP6/CTSK/TNXB/PCOLCE/SERPINH1/MMP15/MMP28/MMP11/FOSL2/ENG/BMP4
GOBP_CHROMOSOME_SEGREGATION	GOBP_CHROMOSOME_SEGREGATION	GOBP_CHROMOSOME_SEGREGATION	69	-0.27735975	-2.278636865	0.000600564	0.020451753	0.01705395	826	tags=57%, list=30%, signal=40%	SMARCC1/TUBG2/SMC6/BUB1B/PDCD6IP/MAPRE1/ESPL1/MAD2L1BP/CHEK2/AURKC/KIF4A/TRIP13/NUSAP1/CENPM/CDCA8/CENPH/KIF23/CSNK2A2/PINX1/TPR/DYNC1LI1/TUBG1/PTTG1/SMC3/STAG3/PTTG2/BRD7/CCNB2/NDE1/TOP3A/CENPE/SPAG5/ZW10/ITGB3BP/CDK5RAP2/C1orf112/SRPK1/KATNB1/CEP63
GOBP_PROTEIN_RNA_COMPLEX_ORGANIZATION	GOBP_PROTEIN_RNA_COMPLEX_ORGANIZATION	GOBP_PROTEIN_RNA_COMPLEX_ORGANIZATION	27	-0.415713759	-2.418116457	0.000616267	0.02082507	0.017365245	604	tags=56%, list=22%, signal=44%	TFIP11/RUVBL1/SF3A1/DHX30/ATR/TAF9/SETX/EIF5/GEMIN4/ZNHIT3/BUD13/PRPF18/SRPK1/GEMIN6/DDX20
GOBP_SPERM_MOTILITY	GOBP_SPERM_MOTILITY	GOBP_SPERM_MOTILITY	14	-0.5602233	-2.388085349	0.000627481	0.021042176	0.017546281	405	tags=64%, list=15%, signal=55%	CATSPER2/IQCF1/AKAP4/ROPN1L/DNAI1/TTC21A/IQCG/LZTFL1/DZIP1
GOCC_CILIARY_BASAL_BODY	GOCC_CILIARY_BASAL_BODY	GOCC_CILIARY_BASAL_BODY	24	-0.425497822	-2.361123161	0.000655718	0.021693303	0.018089232	437	tags=50%, list=16%, signal=42%	IFT122/CEP290/TUBG1/CEP250/SPAG5/BBS4/MKKS/CCDC96/IQCD/IFT20/SPATA7/DZIP1
GOBP_CELL_CEL	GOBP_CELL_CELL	GOBP_CELL_CELL	169	0.21763818	2.003425455	0.0006567	0.021693	0.018089	1652	tags=79%,	AKT1/LRP6/CSK/PPARA/DAB1/ABL1/CLDN3/TNF

L_ADHESION	_ADHESION	_ADHESION				74	303	232		list=60%, signal=33%	/LRFN3/ACTL6B/SDC4/CDH3/ITGA3/HSPB1/PLXNB2/SYK/MAGI1/ITGAM/IRAK1/CLDN15/FYN/CLIC1/CTSG/WNT3A/MSN/CXCL13/SERPINB8/PKP3/CLSTN1/AGER/ITGA9/ITGB1/ITGB7/SHC1/PTPRC/EFNB1/HAVCR2/SERPINE2/PCDHGA8/CDH23/SMAD7/LIMS2/GLI2/NTNG2/BAD/AMIGO3/CD4/RELA/F2RL3/THY1/MYADM/TRO/TYK2/SART1/ST3GAL4/TNXB/ILK/SPN/HMGB1/GATA5/SCGB1A1/CDH4/ITGA6/PARVA/CLDN5/MINK1/LRG1/HLA-DRB3/CD6/HLA-A/CD300A/CLDN4/FLOT2/MYO10/SELPLG/BCL2/HLA-E/ATP1B2/SOCS1/WNT1/ZNF703/UMOD/PKP2/HLA-DRB5/KLHL25/HLA-DQB1/WNK1/LAG3/NFASC/PPP3CA/AIF1/CDH11/CHST2/ERBB2/IGF2/CD177/PYCARD/SOX9/HLA-DMB/BMP4/HLA-DOB/CEBPB/CELSR2/LYN/VEZT/ADIPOQ/ALOX5/CLSTN3/METAP1/TNFRSF21/PERP/ADA/PCDHB16/FXYD5/TMEM47/AMIGO2/PODXL/CCL5/BTN2A2/XBP1/PTPN2/NPTN/ANXA9/PTPN23/DLG4/HAS2/CITED2/HLA-DMA/CD34/CDH2/CYP1B1/EMILIN2/FGL2/CD276
GOBP_POSITIVE_REGULATION_OF_CELL_JUNCTION_ASSEMBLY	GOBP_POSITIVE_REGULATION_OF_CELL_JUNCTION_ASSEMBLY	GOBP_POSITIVE_REGULATION_OF_CELL_JUNCTION_ASSEMBLY	16	0.530963872	2.255798238	0.000718374	0.023204548	0.019349402	916	tags=81%, list=34%, signal=54%	ABL1/CLDN3/SDC4/RAC1/LRRN3/ITGB1BP1/CLSTN1/NRP1/IRX3/AMIGO3/THY1/CLDN5/IL17A
GOCC_ENDOPLASMIC_RETICULUM_LUMEN	GOCC_ENDOPLASMIC_RETICULUM_LUMEN	GOCC_ENDOPLASMIC_RETICULUM_LUMEN	58	0.314078957	2.13498794	0.000717415	0.023204548	0.019349402	1360	tags=76%, list=50%, signal=39%	VWA1/PDIA5/FAM20C/TIMP1/PNPLA2/RDH5/WNT3A/MBTPS1/CALU/TOR1A/FBN1/COL6A3/COL27A1/CD4/TSPAN5/SUMF1/ADAM17/PDIA6/CYP2W1/FUCA2/ADAMTS7/TXNDC5/COL4A2/WFS1/COL3A1/TGOLN2/COL15A1/EDN1/MEN1/SERPINH1/GPX7/F10/SDC2/SERPINA1/WNT1/IGFBP4/COL4A6/LTBP1/CTSC/COL4A5/FMO1/BMP4/ADAMTS5/ARSI
GOBP_REGULATION_OF_HYDROLASE_ACTIVITY	GOBP_REGULATION_OF_HYDROLASE_ACTIVITY	GOBP_REGULATION_OF_HYDROLASE_ACTIVITY	138	0.228801311	1.990654832	0.000712926	0.023204548	0.019349402	723	tags=41%, list=26%, signal=31%	AKT1/RHOC/ABL1/CLDN3/TNF/TIMP1/BAX/ARF4/SFN/RALGDS/STAT3/ARHGEF7/PLXNB2/BOK/SYK/APAF1/ANG/FYN/BBC3/CALM3/GRTP1/GAPDH/ATP2A3/ITGB1BP1/RGS6/PHB2/CXCL13/SERPINB8/MEFV/PDGFRB/AGER/IFI6/ITGB1/ARHGAP27/RGS11/FGFR1/NET1/PTPRC/SERPINE2/TBC1D2B/NTF3/LRP1/BAD/BCR/OAS2/BCAP31/RAPGEF1/PRR7/CCL22/THY1/CTSK/PPP1R15A/GNA12/RHOG/EVI5/RSU1
GOBP_REGULATI	GOBP_REGULATI	GOBP_REGULATIO	96	0.25860882	2.038433838	0.0007491	0.024020	0.020030	1268	tags=67%,	AKT1/CTTN/EIF4G1/SOX15/PPARA/EXTL3/ABL1/

ON_OF_GROWTH	ON_OF_GROWTH	N_OF_GROWTH				17	963	18		list=46%, signal=37%	MMP14/GAMT/GDI1/SFN/STAT3/CCDC85B/WWC3/ADAM15/ING4/SEMA6C/ADIPOR1/PPP2R1A/TSPYL2/ARHGAP4/UCN/VGLL4/WNT3A/SERTAD3/CDK5/CLSTN1/NRP1/PTCH2/NET1/SERPINE2/GHSR/SMAD7/ENO1/MYOD1/ADAM17/ZMPSTE24/GLI1/FAM107A/DNM2/NUBP1/SMO/MAPK11/WFS1/NKX2-5/CDH4/DCBLD2/EDN1/SEMA4B/YAP1/RPS6KA1/BCL2/MTPN/CPNE5/BCL2L1/RBP4/ULK1/IGFBP4/ZFYVE27/ERBB2/FOSL2/IGF2/WT1/FHL1
GOBP_MONOCARBOXYLIC_ACID_METABOLIC_PROCESS	GOBP_MONOCARBOXYLIC_ACID_METABOLIC_PROCESS	GOBP_MONOCARBOXYLIC_ACID_METABOLIC_PROCESS	116	0.240708449	2.004193396	0.000766228	0.024391585	0.020339228	383	tags=29%, list=14%, signal=26%	AKT1/ECHS1/ABCD1/VDAC1/ECH1/PPARA/CYP7A1/DCXR/PRMT3/MECR/DHTKD1/GAMT/PLA2G4D/SLC16A1/AIG1/MTHFS/ZBTB7A/STAT3/OSBPL1A/ASAH2/PTGES/PDK2/ILVBL/ADIPOR1/OSBP/ADIPOR2/GAPDH/PGD/LTC4S/MID1IP1/LPIN2/SORD/ACAA1/FAH
GOBP_SKIN_DEVELOPMENT	GOBP_SKIN_DEVELOPMENT	GOBP_SKIN_DEVELOPMENT	54	0.33636771	2.224143655	0.000786868	0.024868426	0.020736847	1313	tags=76%, list=48%, signal=40%	WNT10A/EXTL3/TNF/CDH3/ITGA3/SFN/FOXQ1/LCE1A/LTB/LSR/TRIM16/PTCH2/KRT14/GLI2/BCR/AHDC1/SPRR3/RELA/KRT3/ZMPSTE24/DNASE1L2/SMO/CNFN/COL3A1/MSX2/KRT85/ITGA6/LCE1D/IL17A/NF1/YAP1/CLDN4/BCL2/GAL/ELOVL1/PPP3CA/SOX21/FOSL2/EDA/SOX9/MAFB
GOBP_GROWTH	GOBP_GROWTH	GOBP_GROWTH	147	0.225686342	1.994181156	0.000808554	0.025371281	0.021156159	903	tags=47%, list=33%, signal=33%	AKT1/CTTN/EIF4G1/SOX15/PPARA/EXTL3/CCM2/ABL1/MMP14/GAMT/HEG1/GDI1/SFN/STAT3/PRMT2/CCDC85B/GATA4/WWC3/ADAM15/ING4/SEMA6C/KLF2/ADIPOR1/PPP2R1A/TSPYL2/ARHGAP4/UCN/VGLL4/LZTS2/WNT3A/SERTAD3/CDK5/EMX1/PTN/CLSTN1/NRP1/PTCH2/ITGB1/BIN3/RAPH1/EIF2AK4/NET1/ANXA6/SERPINE2/GHSR/SMAD7/FGF1/GLI2/COL27A1/ENO1/MYOD1/ADAM17/PAK6/ZMPSTE24/GLI1/FAM107A/DNM2/NUBP1/SMO/PLXNA1/MAPK11/WFS1/COL3A1/NKX2-5/MSX2/CDH4/EHMT2/DCBLD2/EDN1
GOBP_REGULATION_OF_MONOATOMIC_ION_TRANSPORT	GOBP_REGULATION_OF_MONOATOMIC_ION_TRANSPORT	GOBP_REGULATION_OF_MONOATOMIC_ION_TRANSPORT	93	0.262277823	2.044276583	0.000837868	0.026104624	0.021767667	1183	tags=62%, list=43%, signal=37%	AKT1/KCNAB3/VDAC1/KCNJ9/SCN2B/ABL1/CLCN2/BAX/TSPO/P2RX2/STIM1/CABP5/FYN/CLIC1/UCN/CALM3/SNTA1/KCNMB1/PHB2/ATF4/CACNB3/KCNB2/PDGFRB/KCNAB2/CLIC5/ITGB1/LIME1/KCNB1/SERPINE2/CD4/F2RL3/THY1/DIAPH1/CACNA1E/PPIF/ATP1B1/KCNS2/KCNG1/KCNH4/MCHR1/WFS1/KCNS3/ATP2A1/NKX2-5/EDN1/KCNK5/OXSR1/BCL2/GAL/ATP1B2/CNKSR3/PKP2/CRHR1/CA2/MAOB/WNK1/KEL/PPP3CA
GOBP_NON_MOTI	GOBP_NON_MOTI	GOBP_NON_MOTIL	11	-0.599658818	-2.275351019	0.0008681	0.026668	0.022238	437	tags=100%,	TTC8/TMEM107/MAPRE1/IFT122/CEP290/CEP350/

LE_CILIUM_ASSEMBLY	LE_CILIUM_ASSEMBLY	E_CILIUM_ASSEMBLY				2	902	197		list=16%, signal=84%	CEP250/BBS4/MKKS/IFT74
GOBP_AXON_DEVELOPMENT	GOBP_AXON_DEVELOPMENT	GOBP_AXON_DEVELOPMENT	89	0.259589182	2.010709126	0.000866874	0.026668902	0.022238197	1243	tags=64%, list=46%, signal=36%	CTTN/DAB1/ISL2/ABL1/BRSK1/TSPO/GDI1/PLXNB2/CDK5R2/RAC1/KIF13B/TRIO/BRSK2/SEMA6C/TRAK1/KREMEN1/FYN/ARHGAP4/WNT3A/CDK5/PTN/CHRNB2/NRP1/ITGB1/RAPH1/EFNB1/NKX2-8/CNTNAP1/GLI2/NTNG2/B3GNT2/RAB8A/ADAM17/THY1/MCF2/DNM2/SMO/PLXNA1/NEUROG1/EPHA8/RTN4RL2/GFRA3/CDH4/EDN1/SEMA4B/NEUROD6/BCL2/AUTS2/ULK1/KEL/NFASC/MTR/CDH11/ZFYVE27/ERBB2/VASP/CHODL
HP_IMPAIRMENT_IN_PERSONALITY_FUNCTIONING	HP_IMPAIRMENT_IN_PERSONALITY_FUNCTIONING	HP_IMPAIRMENT_IN_PERSONALITY_FUNCTIONING	124	0.242967293	2.05856571	0.000876059	0.026725881	0.022285709	1184	tags=60%, list=43%, signal=36%	AKT1/EIF4G1/CDC42BPB/ATP1A3/UBE2L3/HMBS/STUB1/GNB1/FKBP5/PURA/PRR12/ALDH4A1/GNA11/ITGAM/IRAK1/ANG/ATP7B/LAS1L/GLUD2/EIF2AK1/CHRNB2/TOR1A/PSAT1/CLN3/PDGFRB/FGFR1/CDH23/TBC1D2B/DNAJC5/GLI2/SLC6A17/BCR/NHLH2/SETBP1/LGI3/DEAF1/TSHB/CCNF/SMO/SQSTM1/BAZ1B/WFS1/DISP1/GALT/JRK/DAO/RAD21/UBQLN2/SLC2A1/PGK1/MEN1/CLCA4/EP300/NF1/AIP/SERPINA1/SIX3/ELN/HTRA1/PQBP1/CHRNA7/PROKR2/EIF2B1/IMPDH2/MED12/DGUOK/HS6ST1/HLA-B/GALC/TYROBP/SLC11A1/HLA-DQB1/NFASC/ALAD/GP1BB
GOCC_9PLUS2_MOTILE_CILIUM	GOCC_9PLUS2_MOTILE_CILIUM	GOCC_9PLUS2_MOTILE_CILIUM	23	-0.427812168	-2.347905874	0.000904433	0.027401197	0.02284883	567	tags=57%, list=21%, signal=45%	CAPZB/LYZL6/TCP11/DNAH8/DNAH17/SPATA6/SPA17/AKAP4/ATP1B3/DNAI1/IQCG/SPACA3/DNALI1
GOBP_POSITIVE_REGULATION_OF_CANONICAL_NF_KAPPAB_SIGNAL_TRANSDUCTION	GOBP_POSITIVE_REGULATION_OF_CANONICAL_NF_KAPPAB_SIGNAL_TRANSDUCTION	GOBP_POSITIVE_REGULATION_OF_CANONICAL_NF_KAPPAB_SIGNAL_TRANSDUCTION	37	0.37177795	2.167698035	0.000910976	0.0274104	0.022856504	959	tags=65%, list=35%, signal=43%	RHOC/ABL1/TNF/GPRC5B/SLC35B2/LTB/LTBR/IRAK1/FYN/CC2D1A/GAPDH/TRIM8/NEK6/CANT1/CD4/FBXW11/RELA/S100A4/TNIP2/MIER1/CARD9/TMEM101/CXXC5/VAPA
HP_PRIMARY_MICROCEPHALY	HP_PRIMARY_MICROCEPHALY	HP_PRIMARY_MICROCEPHALY	21	-0.434907995	-2.262292826	0.000942782	0.028174439	0.023493607	417	tags=52%, list=15%, signal=45%	SLC1A4/CLPB/SMC3/NDE1/CENPE/TPRKB/CDK5RAP2/KATNB1/SLC25A19/CEP63/ASNS
GOBP_RNA_3_END_PROCESSING	GOBP_RNA_3_END_PROCESSING	GOBP_RNA_3_END_PROCESSING	14	-0.546463007	-2.32942882	0.001009419	0.028794646	0.024010775	575	tags=71%, list=21%, signal=57%	PAF1/CPSF2/INTS6/CPSF3/ZNF473/CSTF1/EXOSC8/NCBP2/INTS7/PNPT1
GOBP_EXTRINSIC_APOPTOTIC_SIGNALING_PATHWAY	GOBP_EXTRINSIC_APOPTOTIC_SIGNALING_PATHWAY	GOBP_EXTRINSIC_APOPTOTIC_SIGNALING_PATHWAY	37	0.368353963	2.147734047	0.000998428	0.028794646	0.024010775	1320	tags=81%, list=48%, signal=42%	AKT1/CTTN/TNF/ITM2C/BCL2L2/BAX/LTB/BOK/LTBR/PPP2R1A/NRP1/IFI6/FGFR1/PTPRC/MKNK2/BAD/IFI27/RELA/DEDD/ITGA6/NF1/YAP1/BCL2/BCL2L1/SNAI2/TRAF1/TNFSF10/PYCARD/FAIM/B

											MP4
GOBP_SMALL_MOLECULE_CATABOLIC_PROCESS	GOBP_SMALL_MOLECULE_CATABOLIC_PROCESS	GOBP_SMALL_MOLECULE_CATABOLIC_PROCESS	74	0.282816141	2.066234685	0.000985285	0.028794646	0.024010775	734	tags=45%, list=27%, signal=34%	AKT1/ECHS1/ABCD1/ECH1/PPARA/SDSL/HMGCL/CYP7A1/DCXR/GOT2/BCAT2/FUT6/SLC16A1/AIG1/MTHFS/ALDH4A1/ILVBL/PGD/LPIN2/GLUD2/SORD/ACAA1/FAH/GALK1/GPT2/ALDH6A1/ABCD4/BAD/GDA/ENO1/ADHFE1/SCP2/CYP2W1
GOBP_LOCALIZATION_WITHIN_MEMBRANE	GOBP_LOCALIZATION_WITHIN_MEMBRANE	GOBP_LOCALIZATION_WITHIN_MEMBRANE	136	0.230286849	2.006169533	0.001007693	0.028794646	0.024010775	714	tags=41%, list=26%, signal=32%	AKT1/SEC61A1/LRP6/CSK/TNF/MMP14/DENND4C/AP2M1/BAX/ZDHHC22/GDI1/ITGA3/RAB11FIP3/TOMM40/VPS4A/RAC1/PRAM1/ITGAM/TM9SF3/FYN/BBC3/WNT3A/EXOC6/ITGB1BP1/CDK5/PTN/CACNB3/HPS6/ATP13A1/KCNB2/CLN3/PKP3/CLSTN1/AGER/STX3/ITGB1/KCNB1/ITGB7/PTPRC/REEP2/ABI3/GHSR/ATAD1/GRIPAP1/CMTM6/LRP1/ICMT/VPS37B/TSPAN5/RAB8A/GOLGA7/THY1/AMN/MYADM/RHOG/OSBPL5
GOBP_NEGATIVE_REGULATION_OF_MOLECULAR_FUNCTION	GOBP_NEGATIVE_REGULATION_OF_MOLECULAR_FUNCTION	GOBP_NEGATIVE_REGULATION_OF_MOLECULAR_FUNCTION	110	0.244306006	1.981298331	0.001001028	0.028794646	0.024010775	1027	tags=53%, list=38%, signal=34%	AKT1/LRP6/MDFI/BAG5/PPARA/ABL1/NFKBIL1/TNF/TIMP1/BAX/HEG1/GADD45A/SFN/HSPB1/PRMT2/TNFRSF4/ADAM15/IRAK1/CALM3/GAPDH/ATP2A3/ITGB1BP1/HEYL/PHB2/SERPINB8/CLN3/IFI6/EIF2AK4/PTPRC/RWDD3/HAVCR2/SERPINE2/RGS14/SMAD7/LRP1/NFKB1/THY1/PPP1R15A/ZNF593/PPIF/ARRB2/CEBPA/SMO/GNL3L/PNKP/WFS1/MSX2/SPOCK2/EOMES/ANGPTL4/MEN1/SERPINH1/CHMP6/NF1/SERPINA1/CD300A/OXSR1/RPS6KA1
GOBP_LOCOMOTION	GOBP_LOCOMOTION	GOBP_LOCOMOTION	213	0.200412588	1.954774834	0.000970809	0.028794646	0.024010775	1494	tags=70%, list=55%, signal=35%	AKT1/RHOC/ABL1/CLDN3/TNF/MMP14/TIMP1/SDC4/ITGA3/GADD45A/STAT3/ARHGEF7/HSPB1/XCR1/FCER1G/TRIP6/PLXNB2/RAC1/SYK/ADAM15/SEMA6C/ADIPOR1/ARHGAP4/CTSG/WNT3A/ITGB1BP1/GPSM3/MSN/CDK5/CMTM7/VASH1/CXCL13/PTN/CLN3/PDGFRB/AGER/NRP1/ITGA9/STX3/TRPM2/ITGB1/GPR37/FGFR1/PTPRC/SERPINE2/ABI3/GHSR/FZD4/NTF3/SMAD7/SLURP1/LRP1/FGF1/NTNG2/FGF3/BCR/IL1R1/ADAM17/CCL22/THY1/S100A4/MYADM/ELMO2/GNA12/RHOG/FAM107A/AAMP/ST3GAL4/ARRB2/DAPK3/TNXB/SMO/PLXNA1/SPN/HMGB1/GRB7/COL3A1/SPOCK2/ARHGDIB/CARD10/RAB11A/ITGA6/EDN1/PARVA/CLDN5/MINK1/NFE2L2/RALA/SEMA4B/EP300/NF1/F10/CD300A/CLDN4/MDM2/PTP4A1/OXSR1/PODN/MAP2K3/BCL2/IFITM1/MMRN2/ATP1B2/TEK/ZNF703/SNAI2/LSP1/MMP28/WNK1/CCR5/PPP3CA/AIF

											1/CDH11/ROR2/CHST2/CHRD/SPATA13/SH3BP1/FOLR2/ARHGAP18/ENG/PYCARD/SOX9/DAB2/BMP4/C3AR1/RCC2/EMP2/MCAM/LYN/ADIPOQ/ALOX5/VEGFB/EPHA4/CLASP2/TWIST1/CPNE3/AQP1/TRPM4/PHPT1/ADA/APOD/MALAT1/GRIN2C/MAZ/PODXL/PLEKHG3/CCL5/SEMA3C/WAS
GOBP_POSITIVE_REGULATION_OF_DEVELOPMENTAL_PROCESS	GOBP_POSITIVE_REGULATION_OF_DEVELOPMENTAL_PROCESS	GOBP_POSITIVE_REGULATION_OF_DEVELOPMENTAL_PROCESS	233	0.192690433	1.925474721	0.001002556	0.028794646	0.024010775	1475	tags=69%, list=54%, signal=35%	AKT1/EIF4G1/SOX15/FAM20C/MAML1/MAGED1/DAB1/DLX1/ABL1/LBH/TNF/MMP14/RXRB/GPRC5B/CLCN2/ACTL6B/CUL7/BAX/TSPO/GDI1/SFN/STAT3/STIM1/ARHGEF7/HSPB1/PLXNB2/GATA4/SYK/ADRA2B/TRAK1/LRRN3/HMG20B/TRIM16/CLIC1/WNT3A/HEYL/ADD1/ATF4/PTN/CCDC3/SSBP3/CLSTN1/ETV5/AGER/NRP1/PTCH2/ITGB1/FGFR1/NAP1L1/PTPRC/IRX3/SERPINE2/GHSR/RGS14/FZD4/SMAD7/FGF1/GLI2/BAD/AMIGO3/CD4/ISG15/MYOD1/RELA/NFKB1/GLI1/IQGAP3/SART1/ARRB2/WNT2B/CEBPA/TNXB/SMO/PLXNA1/NEUROG1/ILK/MAPK11/HMGB1/GATA5/NKX2-5/MSX2/SIRT2/CDH4/EDN1/CLDN5/LRG1/IL17A/ANGPTL4/NFE2L2/RALA/FBXW8/YAP1/BCL7C/MDM2/CHRNA7/RPS6KA1/CDS1/BCL2/IFITM1/GAL/HCLS1/CPNE5/SHANK1/MMRN2/SOCS1/TEK/WNT1/ZNF703/SNAI2/MIXL1/KLHL25/TYROBP/KLF10/WNK1/PRMT5/PPP3CA/ROR2/ZFYVE27/FRS2/MAN2A1/TFE3/CHODL/CTH/IGF2/ADAM12/WT1/ENG/CTNNBIP1/FAIM/HIPK2/SOX9/DAB2/LRP3/MYC/FIS1/TMEM119/BMP4/LRP8/ITGB8/C3AR1/CEBPB/EMP2/HOXD3/LYN/ADIPOQ/ALOX5/VEGFB/EPHA4/CLASP2/CLSTN3/TWIST1/AQP1/JUND/MMD2/TRPM4/ADA/C3/ZNF488/PLAG1/AMIGO2
GOMF_METAL_ION_TRANSMEMBRANE_TRANSPORTER_ACTIVITY	GOMF_METAL_ION_TRANSMEMBRANE_TRANSPORTER_ACTIVITY	GOMF_METAL_ION_TRANSMEMBRANE_TRANSPORTER_ACTIVITY	73	0.278592097	2.022896769	0.001027212	0.029113162	0.024276374	1702	tags=89%, list=62%, signal=34%	SEC61A1/ATP2B3/KCNAB3/KCNJ9/SCN2B/ATP1A3/SLC12A9/ATP7B/ATP2A3/KCNMB1/SLC29A1/SLC41A3/SLC12A8/CACNB3/ATP13A1/KCNB2/KCNAB2/TRPM2/SLC6A8/KCNB1/ANXA6/SLC5A11/CACNA1E/KCNS2/KCNG1/KCNH4/GRIK5/KCNS3/ATP2A1/CCDC51/KCNK5/SLC30A9/CHRNA7/SLC17A8/SLC13A4/SLC31A1/SLC34A2/SLC4A5/SLC9A8/SLC11A1/ITPR3/KCNK7/SLC10A5/KCNH6/SLC34A3/KCNJ1/AQP1/CACNA1B/TRPM4/TPCN1/GRIN2C/ITPR1/SLC17A7/GRIN1/ANXA9/SLC34A1/SLC13A2/HTR1B/SLC30A3/CNNM2/NIPA2/TMCO3/HCN3/KCNMB3/ATP1A2

GOBP_MICROTUBULE_CYTOSKELETON_ORGANIZATION_INVOLVED_IN_MITOSIS	GOBP_MICROTUBULE_CYTOSKELETON_ORGANIZATION_INVOLVED_IN_MITOSIS	GOBP_MICROTUBULE_CYTOSKELETON_ORGANIZATION_INVOLVED_IN_MITOSIS	38	-0.341776457	-2.253980058	0.001057824	0.029411523	0.024525165	853	tags=66%, list=31%, signal=46%	KPNB1/TUBG2/VCP/PDCD6IP/MAPRE1/ESPL1/SPAST/CHEK2/AURKC/KIF4A/RAE1/NUSAP1/CDCA8/CENPH/KIF23/TPR/WDR62/PAFAH1B1/TUBG1/SMC3/NDE1/CENPE/ZW10/CDK5RAP2/EFHC1
GOBP_SYNAPSE_ASSEMBLY	GOBP_SYNAPSE_ASSEMBLY	GOBP_SYNAPSE_ASSEMBLY	22	0.458973716	2.238421662	0.00105371	0.029411523	0.024525165	619	tags=59%, list=23%, signal=46%	EIF4G1/LRFN3/PLXNB2/LRRN3/CHD4/WNT3A/CDK5/CHRNB2/CLSTN1/ABI3/GHSR/NTNG2/AMIGO3
GOBP_SMALL_MOLECULE_METABOLIC_PROCESS	GOBP_SMALL_MOLECULE_METABOLIC_PROCESS	GOBP_SMALL_MOLECULE_METABOLIC_PROCESS	319	0.176695312	1.89536843	0.001053165	0.029411523	0.024525165	680	tags=33%, list=25%, signal=28%	AKT1/PDXK/ECHS1/ABCD1/NME4/VDAC1/GTPBP1/ECH1/PPARA/SDSL/ENTPD2/HMGCL/CYP7A1/ENTPD8/ITPK1/DCXR/UCKL1/PRMT3/SFXN3/PGLS/TNF/GOT2/CLCN2/BCAT2/ITPA/ACO1/MECR/IDH3G/TSPO/DHTKD1/MGST2/GAMT/PLA2G4D/NDUFB7/FUT6/SLC16A1/AIG1/NT5C/MTHFS/ZBTB7A/STAT3/ACOT9/NANS/OSBPL1A/NADK/ENTPD6/ACY1/ASAH2/LARS2/SYK/ALDH4A1/GSS/PTGES/PDK2/ILVBL/ADIPOR1/INPPL1/OSBP/PNPLA2/ADIPOR2/GAPDH/PGD/NTHL1/RDH5/LTC4S/MID1IP1/LPIN2/GLUD2/SORD/ACAA1/FAH/ATF4/MBTPS1/DCTD/PSAT1/CLN3/KCNAB2/GALK1/TFF3/GPT2/PRKAB2/ALDH6A1/SLC6A8/CLYBL/ABCC10/CYP4B1/ABCD4/GHSR/AKR7A2/CAD/FGF1/ALOX5AP/HSD17B8/PYCR2/BAD/GDA/ICMT/KHK/ENO1/APRT/PTGES2/TMEM86B/NFKB1/AMN/HKDC1/ZMPSTE24
GOBP_CIRCULATORY_SYSTEM_PROCESS	GOBP_CIRCULATORY_SYSTEM_PROCESS	GOBP_CIRCULATORY_SYSTEM_PROCESS	101	0.253049316	2.02928795	0.001069376	0.029545724	0.024637071	1384	tags=70%, list=51%, signal=36%	ATP2B3/PPARA/SCN2B/ATP1A3/PTP4A3/ABL1/TNF/HEG1/SLC16A1/P2RX2/CPA3/GATA4/ADRA2B/GNA11/KLF2/FYN/UCN/CALM3/SNTA1/CTSG/ATP2A3/PCSK5/KCNMB1/ITGB1BP1/SLC29A1/AGER/APLN/POPDC2/IRX3/SMAD7/LRP1/SLC6A17/BCR/ZMPSTE24/GNA12/MYL3/ATP1B1/ATP2A1/NKX2-5/EDN1/CLDN5/SLC2A1/KLK1/YAP1/ELN/MDM2/CHRNA7/MAP2K3/ATP1B2/TEK/UMOD/PKP2/ABCG2/SLC1A5/SLC4A5/SLC29A2/WNK1/KEL/SLC2A13/GJC1/CTNNBIP1/ECE1/LRP3/SLC44A1/C3AR1/EMP2/ABAT/SLC22A8/KCNH6/ADIPOQ/VEGFB
GOBP_REGULATION_OF_CELL_GROWTH	GOBP_REGULATION_OF_CELL_GROWTH	GOBP_REGULATION_OF_CELL_GROWTH	69	0.295451748	2.110507111	0.0010904 4	0.029737388	0.024796892	1070	tags=61%, list=39%, signal=38%	AKT1/CTTN/EIF4G1/PPARA/EXTL3/ABL1/MMP14/GDI1/SFN/CCDC85B/ADAM15/ING4/SEMA6C/ADIPOR1/PPP2R1A/TSPYL2/ARHGAP4/UCN/VGLL4/WNT3A/SERTAD3/CDK5/CLSTN1/NRP1/PTCH2/NET1/SERPINE2/SMAD7/ENO1/ADAM17/FAM107A/DNM2/NUBP1/CDH4/DCBLD2/EDN1/SEMA4B/YA

											P1/RPS6KA1/BCL2/MTPN/CPNE5
GOBP_MUSCLE_STRUCTURE_DEVELOPMENT	GOBP_MUSCLE_STRUCTURE_DEVELOPMENT	GOBP_MUSCLE_STRUCTURE_DEVELOPMENT	119	0.242055938	2.024656591	0.001088534	0.029737388	0.024796892	1347	tags=66%, list=49%, signal=35%	AKT1/SOX15/MAML1/PPARA/CFL2/SUPT6H/TNF/MMP14/ACTL6B/HEG1/P2RX2/TAGLN/GATA4/MEF2D/HMG20B/MYH11/WNT3A/HEYL/CDK5/SMTN/PDGFRB/ITGB1/BIN3/POPDC2/IRX3/OBSCN/COL6A3/SMAD7/CNTNAP1/FGF3/MYOD1/ZMPSTE24/PDLIM2/TMOD4/EMD/MYL3/ARRB2/SMO/NEUROG1/MAPK11/COL3A1/NKX2-5/EGR1/DISP1/SIRT2/EOMES/HSD17B1/EDN1/EP300/TMOD1/NF1/SPEG/ELN/MDM2/FLOT2/BCL2/MTPN/WNT1/PKP2/MBNL1/KEL/PDLIM3/MEF2B/PPP3CA/FRS2/CHODL/CTH/IGF2/ADAM12/WT1/ENG/FHL1/SOX9/ACTN2/TMEM119/BMP4/BTG2/VAMP5/ADAMTS5
GOBP_RESPONSE_TO_GROWTH_FACTOR	GOBP_RESPONSE_TO_GROWTH_FACTOR	GOBP_RESPONSE_TO_GROWTH_FACTOR	127	0.229726379	1.951338378	0.001096621	0.029737388	0.024796892	1330	tags=65%, list=49%, signal=35%	AKT1/FAM20C/PPARA/WNT10A/DLX1/PTP4A3/ABL1/STUB1/ITGA3/ZBTB7A/STAT3/HSPB1/GATA4/PPP2R5B/APAF1/FYN/ITGB1BP1/RASL11B/HEYL/ADAMTSL2/CXCL13/PDGFRB/NRP1/APLN/ITGB1/FGFR1/SHC1/FZD4/NTF3/CAD/FBN1/SMAD7/FGF1/FGF3/PITX3/RAPGEF1/ADAM17/RELA/CTSK/EMD/ADAMTS7/ARRB2/TNXB/COL4A2/PAX9/ILK/COL3A1/GATA5/EGR1/MSX2/SCGB1A1/PEG10/ELK1/EDN1/CLDN5/LRG1/MEN1/NFIA/EP300/MDM2/HTRA1/MAP2K3/MMRN2/WNT1/ZNF703/PTPN1/SNAI2/RGMB/ZFYVE27/ERBB2/CHRD/FRS2/EHD4/ENG/LTBP1/HIPK2/SOX9/DAB2/MYC/BMP4/LRP8/ITGB8
GOCC_CONDENSED_NUCLEAR_CHROMOSOME	GOCC_CONDENSED_NUCLEAR_CHROMOSOME	GOCC_CONDENSED_NUCLEAR_CHROMOSOME	13	-0.538361337	-2.20715683	0.001124592	0.030308799	0.02527337	570	tags=69%, list=21%, signal=55%	HSPA2/BLM/FKBP6/TUBG1/SMC3/STAG3/RAD51/SMC1B/RAD50
GOBP_POSITIVE_REGULATION_OF_PROTEIN_LOCALIZATION_TO_PLASMA_MEMBRANE	GOBP_POSITIVE_REGULATION_OF_PROTEIN_LOCALIZATION_TO_PLASMA_MEMBRANE	GOBP_POSITIVE_REGULATION_OF_PROTEIN_LOCALIZATION_TO_PLASMA_MEMBRANE	12	0.57663894	2.185565801	0.001157984	0.030830448	0.025708354	793	tags=83%, list=29%, signal=59%	AKT1/TNF/ITGA3/WNT3A/CLN3/STX3/ITGB1/LRP1/RHOG/SQSTM1
GOCC_TRANSFERASE_COMPLEX	GOCC_TRANSFERASE_COMPLEX	GOCC_TRANSFERASE_COMPLEX	166	-0.180780274	-1.875547974	0.00115197	0.030830448	0.025708354	839	tags=46%, list=31%, signal=34%	DAD1/KRTCAP2/BARD1/POLE/SMC6/TEX10/BUB1B/ENY2/PIGP/POLR1C/DDA1/ERCC3/SHARPIN/RANBP2/PFKM/GTF2H2/RNF7/CDK5R1/ANKRD9/KCTD10/FZR1/ATG12/PIGH/DCUN1D3/POLRMT/POLR3E/RUVBL1/PAF1/RMND5B/KLHDC2/KLHL8/ASB1/FBXO39/PAXIP1/UBE2N/SUZ12/RCHY1/CSNK2A2/POLA2/FBXL2/FBXO24/CKS2/TBK1/PRKAG

											2/GTF2H1/RNMT/SUPT3H/DR1/CCNB2/FBXO7/TAF9/WDR26/PCGF6/PRIM1/PRKAA1/RAD51/TSSK2/TAF6/PIK3CG/UBE2D2/AKAP4/DERL3/FBXL18/TAF10/FBXO15/UBE2D3/PARD6A/TBPL1/BRD1/DERL2/CCNH/CAB39/PCNA/SUGT1/ANAPC10/POLG2/CACYBP
GOMF_CYTOSKELETAL_MOTOR_ACTIVITY	GOMF_CYTOSKELETAL_MOTOR_ACTIVITY	GOMF_CYTOSKELETAL_MOTOR_ACTIVITY	19	-0.455554689	-2.258431038	0.001182618	0.031296632	0.026097087	620	tags=63%, list=23%, signal=49%	KIF4A/KIFC3/KIF23/MYO9A/MYH10/SMC3/DNAH8/DNAH17/CENPE/DNAI1/KIF3A/DYNLRB2
GOBP_MOLTING_CYCLE	GOBP_MOLTING_CYCLE	GOBP_MOLTING_CYCLE	20	0.471156099	2.18760913	0.001217629	0.031839555	0.02654981	1289	tags=90%, list=47%, signal=48%	WNT10A/TNF/CDH3/FOXQ1/PTCH2/KRT14/GLI2/RELA/ZMPSTE24/DNASE1L2/SMO/MSX2/NF1/BCL2/GAL/SOX21/EDA/SOX9
GOBP_INORGANIC_ION_HOMEOSTASIS	GOBP_INORGANIC_ION_HOMEOSTASIS	GOBP_INORGANIC_ION_HOMEOSTASIS	92	0.2593048	2.012370966	0.001212589	0.031839555	0.02654981	1702	tags=86%, list=62%, signal=33%	ATP2B3/ATP1A3/ABL1/ACO1/BAX/SLC12A9/STIM1/XCR1/BOK/CALM3/ATP7B/ATP2A3/EIF2AK1/SLC12A8/ATF4/ATP13A1/CLN3/TRPM2/LIME1/PTPRC/ANXA6/CDH23/HCRTR1/KLHL3/CCDC115/SV2A/BCAP31/F2RL3/THY1/DIAPH1/ATP1B1/NUBP1/WFS1/ATP2A1/GRM1/EDN1/SLC30A9/CHRNA7/SLC17A8/BCL2/SCARA5/ATP1B2/SLC31A1/UMOD/GPR12/SLC34A2/SLC11A1/ITPR3/WNK1/KEL/CCR5/CYBRD1/GP1BB/ABCB7/SLC25A23/MYC/FIS1/FECH/LYN/SLC34A3/FXN/TPCN1/CCL5/ITPR1/IBTK/SLC17A7/NPTN/GRIN1/CCL8/GSTO1/SLC34A1/ATG5/HTR1B/SCO1/FKBP1A/CNNM2/FZD9/SCO2/ATP1A2
GOBP_RHO_PROTEIN_SIGNAL_TRANSDUCTION	GOBP_RHO_PROTEIN_SIGNAL_TRANSDUCTION	GOBP_RHO_PROTEIN_SIGNAL_TRANSDUCTION	29	0.405136137	2.186996632	0.00122786	0.0319171	0.026614472	883	tags=66%, list=32%, signal=45%	ARHGEF1/ABL1/HEG1/ITGA3/RHOBTB2/RAC1/TNFAIP1/ARHGAP4/NRP1/ITGB1/NET1/ARHGDIA/BCR/F2RL3/GNA12/RHOG/RHOU/COL3A1/ARHGDIB
GOBP_STRIATED_MUSCLE_TISSUE_DEVELOPMENT	GOBP_STRIATED_MUSCLE_TISSUE_DEVELOPMENT	GOBP_STRIATED_MUSCLE_TISSUE_DEVELOPMENT	45	0.342794264	2.13995874	0.001277027	0.032760558	0.0273178	1320	tags=76%, list=48%, signal=40%	SOX15/MAML1/PPARA/ABL1/HEG1/GATA4/MYH11/VGLL4/WNT3A/PDGFRB/ITGB1/IRX3/SMAD7/FGF3/ZMPSTE24/GLI1/MYL3/ARRB2/MAPK11/GATA5/NKX2-5/MSX2/SIRT2/EDN1/YAP1/MTPN/PKP2/RBP4/FRS2/GJC1/WT1/ENG/ACTN2/BMP4
GOBP_POSITIVE_REGULATION_OF_MAPK_CASCADE	GOBP_POSITIVE_REGULATION_OF_MAPK_CASCADE	GOBP_POSITIVE_REGULATION_OF_MAPK_CASCADE	68	0.300711306	2.13067671	0.001282681	0.032760558	0.0273178	1349	tags=72%, list=49%, signal=37%	DOK4/ABL1/MAPK8IP2/TNF/GADD45A/GATA4/SYK/LTBR/ADRA2B/IRAK1/PHB2/MID1/PDGFRB/AGER/NRP1/GPR37/FGFR1/SHC1/PTPRC/HAVCR2/HCRTR1/NTF3/FGF1/EIF2AK2/CD4/CCL22/DIRAS1/IQGAP3/ARRB2/EPHA8/CARD9/HMGB1/GRM1/EDN1/MINK1/CHRNA7/MAP2K3/TEK/MOS/PTPN1/IGFBP4/ROR2/ERBB2/FRS2/IGF2/PYCARD/HIPK2/BMP4/TRAF4

GOCC_POSTSYNAPSE	GOCC_POSTSYNAPSE	GOCC_POSTSYNAPSE	117	0.23451545	1.952077446	0.001272213	0.032760558	0.0273178	665	tags=39%, list=24%, signal=31%	AKT1/CTTN/EIF4G1/FXR2/CLTB/ABL1/MAPK8IP2/LRFN3/AP2M1/ARF4/ITGA3/P2RX2/RAB11FIP3/ARHGEF7/PURA/PRR12/TMUB1/RAC1/KCTD12/TRIO/FYN/CNIH3/SNTA1/CPLX2/SLC29A1/CDK5/PHB2/ADD1/CHRNB2/CLSTN1/AGER/NRP1/STX3/ITGB1/KCNB1/ABI3/GHSR/ATAD1/RGS14/GRIPAP1/SLC6A17/BCR/RAB8A/ELAVL1/PAK6/PRR7
GOBP_EPITHELIAL_CELL_PROLIFERATION	GOBP_EPITHELIAL_CELL_PROLIFERATION	GOBP_EPITHELIAL_CELL_PROLIFERATION	81	0.270050613	2.025177716	0.001296699	0.032845454	0.027388592	1395	tags=73%, list=51%, signal=37%	AKT1/MAGED1/TNF/MMP14/BCL2L2/CDH3/BAX/SFN/STAT3/PURA/MTSS1/ANG/WNT3A/ITGB1BP1/VASH1/PHB2/PTN/CDK4/NRP1/APLN/FGFR1/GHSR/NKX2-8/SLURP1/FGF1/LIMS2/BAD/ADAM17/GLI1/IQGAP3/KLF9/DEAF1/FOXE3/SMO/HMGB1/NKX2-5/LRG1/IL17A/NF1/YAP1/RPS6KA1/MMRN2/TEK/ZNF703/BCL2L1/SNAI2/IGFBP4/POLD4/ERBB2/FRS2/IGF2/SOX9/DAB2/MYC/BMP4/CEBPB/ALOX5/VEGFB/TWIST1
GOBP_CELL_MORPHOGENESIS_INVOLVED_IN_NEURON_DIFFERENTIATION	GOBP_CELL_MORPHOGENESIS_INVOLVED_IN_NEURON_DIFFERENTIATION	GOBP_CELL_MORPHOGENESIS_INVOLVED_IN_NEURON_DIFFERENTIATION	101	0.251079729	2.013493163	0.001300958	0.032845454	0.027388592	1219	tags=63%, list=45%, signal=36%	CTTN/DAB1/ISL2/ABL1/MAPK8IP2/CUL7/BRSK1/GDI1/PLXNB2/CDK5R2/RAC1/KIF13B/TRIO/BRSK2/SEMA6C/TRAK1/FYN/ARHGAP4/WNT3A/CDK5/PTN/CHRNB2/NRP1/ITGB1/RAPH1/EFNB1/CDH23/ABI3/FZD4/NKX2-8/CNTNAP1/GLI2/NTNG2/B3GNT2/RAB8A/ADAM17/THY1/MCF2/DNM2/SMO/PLXNA1/EPHA8/GFRA3/CDH4/ABI1/EDN1/MINK1/SEMA4B/FBXW8/SDC2/PQBP1/CHRNA7/BCL2/TRIOBP/SHANK1/AUTS2/ULK1/KEL/NFASC/PPP3CA/CDH11/ZFYVE27/ERBB2/VASP
GOBP_LEUKOCYTE_CELL_CELL_ADHESION	GOBP_LEUKOCYTE_CELL_CELL_ADHESION	GOBP_LEUKOCYTE_CELL_CELL_ADHESION	82	0.26360098	1.98728024	0.001312692	0.032952326	0.027477709	1521	tags=78%, list=56%, signal=36%	AKT1/CSK/PPARA/ABL1/TNF/ACTL6B/SDC4/SYK/IRAK1/FYN/CTSG/MSN/AGER/ITGB1/ITGB7/PTPRC/EFNB1/HAVCR2/SMAD7/GLI2/BAD/CD4/RELA/THY1/TYK2/SART1/ST3GAL4/SPN/HMGB1/SCGB1A1/LRG1/HLA-DRB3/CD6/HLA-A/CD300A/FLOT2/SELPLG/HLA-E/SOCS1/UMOD/HLA-DRB5/KLHL25/HLA-DQB1/WNK1/LAG3/PPP3CA/AIF1/CHST2/ERBB2/IGF2/CD177/PYCARD/HLA-DMB/BMP4/HLA-DOB/CEBPB/LYN/ALOX5/TNFRSF21/ADA/CCL5/BTN2A2/XBP1/PTPN2
GOBP_CALCIUM_ION_TRANSPORT	GOBP_CALCIUM_ION_TRANSPORT	GOBP_CALCIUM_ION_TRANSPORT	72	0.280136601	2.02359215	0.001352772	0.033765491	0.028155776	838	tags=49%, list=31%, signal=35%	SEC61A1/ATP2B3/VDAC1/ABL1/BAX/TSPO/P2RX2/STIM1/XCR1/CABP5/FYN/UCN/CALM3/ATP2A3/WNT3A/CDK5/CACNB3/CHRNB2/PDGFRB/TRPM2/LIME1/PTPRC/ANXA6/CDH23/CD4/F2RL3/GJA4/THY1/ZMPSTE24/DIAPH1/CACNA1E/ATP1B1/MCHR1/WFS1/ATP2A1

GOBP_POSITIVE_REGULATION_OF_RESPONSE_TO_EXTERNAL_STIMULUS	GOBP_POSITIVE_REGULATION_OF_RESPONSE_TO_EXTERNAL_STIMULUS	GOBP_POSITIVE_REGULATION_OF_RESPONSE_TO_EXTERNAL_STIMULUS	94	0.252681331	1.979582347	0.001369694	0.033994733	0.028346933	1385	tags=70%, list=51%, signal=36%	NFKBIL1/TNF/GPRC5B/MGST2/LSM14A/HSPB1/RAC1/SYK/RNF185/IRAK1/FYN/GPSM3/PHB2/CXCL13/PTN/LY86/MEFV/PDGFRB/AGER/NRP1/STX3/FGFR1/HAVCR2/GHSR/NTF3/ZNFX1/ALOX5AP/EIF2AK2/PLAT/ADAM17/RELA/TNIP2/ARRB2/CEBPA/CARD9/HMGB1/SIRT2/EDN1/IL17A/COLEC11/EP300/UNC93B1/CD300A/NPLOC4/FLOT2/PQBP1/OXSR1/MAPKAPK3/HLA-E/MMRN2/PUM1/TYROBP/WNK1/HLA-F/LAG3/AIF1/LRSAM1/CASP4/CTSC/PYCARD/C3AR1/CEBPB/TRIM41/LYN/VEGFB/PLSCR1
GOBP_REGULATION_OF_CELL_DIFFERENTIATION	GOBP_REGULATION_OF_CELL_DIFFERENTIATION	GOBP_REGULATION_OF_CELL_DIFFERENTIATION	257	0.182767518	1.860074979	0.001397108	0.034480323	0.028751848	1395	tags=64%, list=51%, signal=34%	AKT1/EIF4G1/FAM20C/MAML1/PPARA/DAB1/EXTL3/DLX1/ISL2/ABL1/SUPT6H/LBH/TNF/MMP14/GPRC5B/CLCN2/ACTL6B/CUL7/TSPO/GDI1/SFN/STAT3/CCDC85B/PLXNB2/SYK/ADRA2B/TRIO/GNA11/SEMA6C/TRAK1/ADIPOR1/PPP2R1A/HMG20B/TRIM16/CLIC1/ARHGAP4/CHD4/WNT3A/HEYL/CDK5/ADD1/ATF4/PTN/CCDC3/ETV5/AGER/NRP1/PTCH2/ITGB1/EIF2AK4/FGFR1/NAP1L1/PTPRC/IRX3/SERPINE2/RGS14/FZD4/NTF3/FBN1/SMAD7/GLI2/EIF2AK2/PITX3/BAD/CD4/ISG15/MYOD1/RELA/NFKB1/THY1/GLI1/MCF2/ADAMTS7/SART1/ARRB2/CEBPA/FOXE3/TNXB/SMO/PLXNA1/NEUROG1/PIAS3/ILK/MAPK11/HMGB1/GATA5/NKX2-5/MSX2/SMYD5/SIRT2/SPOCK2/CDH4/EDN1/CLDN5/LRG1/IL17A/PURB/NFE2L2/SEMA4B/FBXW8/OCIAD1/MEN1/JDP2/NF1/SIX3/YAP1/NFAM1/BCL7C/MDM2/FLOT2/RPS6KA1/CDS1/BCL2/IFITM1/HCLS1/SOCS1/WNT1/HLA-B/ZNF703/PKP2/SNAI2/MAF/MIXL1/KLHL25/ULK1/TYROBP/KLF10/LAG3/PRMT5/PPP3CA/ROR2/ZFYVE27/ERBB2/CHRD/FRS2/MAN2A1/TFE3/MMP11/FOSL2/CHODL/CTH/IGF2/TCTA/ENG/CTNNBIP1/FAIM/SOX9/DAB2/LRP3/MYC/MAFB/TMEM119/BMP4/LRP8/BTG2/CEBPB/EMP2/HOXD3/LYN/ADIPOQ/ALOX5/EPHA4/CLASP2/TWIST1
GOBP_REGULATION_OF_CELL_CYCLE_PHASE_TRANSITION	GOBP_REGULATION_OF_CELL_CYCLE_PHASE_TRANSITION	GOBP_REGULATION_OF_CELL_CYCLE_PHASE_TRANSITION	89	-0.228502123	-2.009540226	0.001411512	0.034641181	0.028885981	664	tags=44%, list=24%, signal=34%	MAD2L1BP/CHEK2/TIPRL/FZR1/DCUN1D3/MTA3/CDC7/RINT1/PAF1/HSPA2/TRIP13/RAD17/PAXIP1/DOT1L/BLM/TIMELESS/CDCA8/PINX1/TPR/DYNC1LI1/PLRG1/CDC25C/CPSF3/CRY1/CDC14B/TFDP1/ATR/BRD7/FBXO7/RAD51/MDC1/CENPE/ZW10/CDK5RAP2/DONSON/RAD50/CCNH/INTS7/CEP63

GOBP_REGULATION_OF_INTRACELLULAR_SIGNAL_TRANSDUCTION	GOBP_REGULATION_OF_INTRACELLULAR_SIGNAL_TRANSDUCTION	GOBP_REGULATION_OF_INTRACELLULAR_SIGNAL_TRANSDUCTION	292	0.174776031	1.827670567	0.001435685	0.035038702	0.029217459	1110	tags=50%, list=41%, signal=33%	AKT1/DUSP9/RHOC/DOK4/BAG5/CSK/PPARA/ARHGEF1/EXTL3/PTP4A3/ABL1/MAPK8IP2/LBH/TNF/GPRC5B/BCL2L2/DENND4C/BAX/HEG1/ITGA3/GADD45A/SLC35B2/P2RX2/HSPB1/TRIP6/LTB/BOK/GATA4/RAC1/TNFAIP1/SYK/LTBR/ADRA2B/WWC3/ING4/TRIO/PDK2/ADIPOR1/PPP2R1A/IRAK1/FYN/BBC3/ARHGAP4/UCN/CALM3/CC2D1A/GAPDH/VGLL4/LMCD1/ITGB1BP1/PHB2/ATF4/DGKG/TRIM8/MID1/PDGFRB/AGER/NRP1/ITGB1/NEK6/LIME1/GPR37/ARHGAP27/FGFR1/NET1/SHC1/PTPRC/HAVCR2/HCRTR1/OBSCN/RGS14/NTF3/PRAP1/SMAD7/LRP1/FGF1/ARHGDIA/CANT1/EIF2AK2/BAD/BCR/CD4/BCAP31/FBXW11/NKIRAS2/ENO1/RELA/PAK6/CCL22/F2RL3/DIRAS1/ZMPSTE24/S100A4/MYADM/GNA12/MCF2/IQGAP3/PSD4/TNIP2/PPIF/ARHGEF17/ARRB2/MIER1/ARFGAP1/PHLDA3/SQSTM1/ILK/EPHA8/CARD9/HMGB1/VWF/WFS1/RHOU/COL3A1/TMEM101/ARHGDIB/GRM1/EDN1/MINK1/SLA/CXXC5/NFE2L2/MEN1/VAPA/EP300/NF1/AIP/F10/CD300A/RGL2/MDM2/CHRNA7/RPS6KA1/MAP2K3/UBE2B/BCL2/ARHGEF3/HCLS1/AUTS2/TEK/WNT1/CNKSR3/RPS15/MOS/PTPN1/BCL2L1/SNAI2
GOCC_MEMBRANE_PROTEIN_COMPLEX	GOCC_MEMBRANE_PROTEIN_COMPLEX	GOCC_MEMBRANE_PROTEIN_COMPLEX	211	0.191283441	1.871564347	0.001477735	0.035865686	0.02990705	1087	tags=51%, list=40%, signal=33%	SEC61A1/LRP6/KCNAB3/GNG7/VDAC1/SCN2B/CLTB/ATP1A3/CLCN2/CDH3/DENND4C/AP2M1/BAX/NDUFB7/TIMM17B/ITGA3/TMED3/GNB1/COX8A/TOMM40/TAP1/FCER1G/RAC1/SYK/GNA11/COPE/ITGAM/CLIC1/SLC25A5/CNIH3/CALM3/SNTA1/CPLX2/SSR4/WNT3A/KCNMB1/GJB1/CD79A/PHB2/HM13/DERL1/AP1S2/CACNB3/CHRNB2/KCNB2/SEC22B/KCNAB2/ITGA9/STT3A/CLIC5/STX3/ITGB1/COPZ1/LIME1/KCNB1/ITGB7/PEF1/SHC1/C8A/CDH23/ABCD4/CCDC115/LRP1/COX7A1/CD4/VPS37B/GJA4/AP1B1/GNA12/CACNA1E/CHCHD3/TYK2/PPIF/CHMP7/ATP1B1/KCNS2/KCNG1/KCNH4/DNAJC11/GRIK5/TOMM20/HMGB1/KCNS3/ATP2A1/C9/CCDC51/CDH4/GRM1/ITGA6/IGF2R/APH1A/HLA-DRB3/BET1L/CHMP6/ABHD12/HLA-A/NPLOC4/CLDN4/FLOT2/NDUFS1/CHRNA7/SLC17A8/BCL2/VPS11/HLA-E/ATP1B2/SOCS1/HLA-B
GOCC_GOLGI_APPARATUS	GOCC_GOLGI_APPARATUS	GOCC_GOLGI_APPARATUS	273	0.17951805	1.837312389	0.001486844	0.0358885	0.029926074	1240	tags=57%, list=45%,	DDX54/CTTN/B4GALNT4/LRP6/FAM20C/CLTB/ATP1A3/EXTL3/GAL3ST4/MMP14/ITM2C/SLC35D2/

										signal=34%	TMED1/CUL7/SDC4/ACO1/DENND4C/MGAT4B/ZDHHC22/ARF4/GDI1/FUT6/TMED3/SLC35B2/RAB11FIP3/ENTPD6/ASAH2/BOK/RAC1/GDI2/LTBR/LFNG/TRAPPC1/AKR7A3/COPE/INPPL1/ST6GALNAC6/TAF11/OSBP/TRIP10/PRELP/ATP7B/BACE2/M6PR/WNT3A/PCSK5/OCIAD2/EXTL1/ZFYVE1/B4GALT7/AP1S2/MBTPS1/CALU/SEC22B/MID1/CLN3/A4GALT/PDGFRB/CLSTN1/CLIC5/COPZ1/B3GAT3/GALNT2/SEC23IP/AKR7A2/LRP1/RAB20/DNAJC5/CANT1/B3GNT2/BCAP31/SPRR3/RAB8A/GOLGA7/ADAM17/PTGES2/CD1B/PPP1R15A/AP1B1/WDR81/TBC1D23/SCAMP3/SART1/ST3GAL4/OSBPL11/LIMK2/DNM2/SPPL2B/SMO/ARFGAP1/RAB6B/TRRAP/KCNS3/RHOU/GALT/HS3ST4/TGOLN2/MUC2/RAB11A/MINK1/SLC2A1/GALNT11/PRDM2/IGF2R/NFE2L2/APH1A/FBXW8/HLA-DRB3/OCIAD1/BET1L/VAPA/CHID1/F10/SDC2/HLA-A/SERPINA1/ARF5/RNF122/UNC93B1/COMMD9/SNX17/TPST2/TBC1D20/LHB/ZDHHC16/PCSK1N/HLA-E/SCARB2/HS6ST1/B3GNT8/FYCO1/NAGPA/WNT1/HLA-B/UMOD/MIF4GD/VAC14/B3GALNT1/HLA-DRB5/SLC9A8/DHH/CREB3L4/HLA-DQB1/KEL/HLA-F/ZDHHC14/PRMT5/COPZ2/CHST2/ZDHHC4/CCDC91/MAN2A1/MMP11/TGFBI/VPS13B
GOBP_POSITIVE_REGULATION_OF_CELL_DIFFERENTIATION	GOBP_POSITIVE_REGULATION_OF_CELL_DIFFERENTIATION	GOBP_POSITIVE_REGULATION_OF_CELL_DIFFERENTIATION	155	0.221083042	1.997062705	0.00151796	0.036439328	0.030385389	1468	tags=72%, list=54%, signal=35%	AKT1/EIF4G1/FAM20C/MAML1/DAB1/DLX1/TNF/MMP14/GPRC5B/CLCN2/ACTL6B/CUL7/TSPO/GDI1/SFN/STAT3/PLXNB2/SYK/ADRA2B/TRAK1/HMG20B/TRIM16/CLIC1/WNT3A/HEYL/ADD1/PTN/CDC3/ETV5/AGER/NRP1/PTCH2/ITGB1/FGFR1/NAP1L1/PTPRC/IRX3/SERPINE2/RGS14/FZD4/SMAD7/GLI2/BAD/CD4/ISG15/MYOD1/RELA/NFKB1/SART1/ARRB2/CEBPA/TNXB/SMO/PLXNA1/NEUROG1/ILK/MAPK11/HMGB1/GATA5/NKX2-5/MSX2/SIRT2/CDH4/EDN1/CLDN5/LRG1/IL17A/FBXW8/YAP1/MDM2/RPS6KA1/CDS1/BCL2/IFITM1/HCLS1/SOCS1/ZNF703/SNAI2/KLHL25/TYROBP/KLF10/PRMT5/PPP3CA/ROR2/ZFYVE27/MAN2A1/TFE3/CHODL/CTH/ENG/CTNNBIP1/FAIM/SOX9/DAB2/LRP3/TMEM119/BMP4/LRP8/CEBPB/EMP2/HOXD3/LYN/ADIPOQ/EPHA4/TWIST1/JUND/MMD2/TRPM4/ADA/ZNF488/PLAG1
GOBP_REGULATI	GOBP_REGULATI	GOBP_REGULATIO	43	0.359752443	2.221149155	0.0015478	0.036954	0.030815	619	tags=49%,	AKT1/EIF4G1/ABL1/TNF/LRFN3/ARF4/LRRN3/FY

ON_OF_SYNAPSE_STRUCTURE_OR_ACTIVITY	ON_OF_SYNAPSE_STRUCTURE_OR_ACTIVITY	N_OF_SYNAPSE_STRUCTURE_OR_ACTIVITY				48	862	273		list=23%, signal=38%	N/CHD4/WNT3A/CDK5/CHRNB2/CLN3/CLSTN1/ITGB1/ABI3/GHSR/GRIPAP1/CNTNAP1/NTNG2/AMIGO3
GOBP_SKIN_EPIDERMIS_DEVELOPMENT	GOBP_SKIN_EPIDERMIS_DEVELOPMENT	GOBP_SKIN_EPIDERMIS_DEVELOPMENT	23	0.427419123	2.106069837	0.001561329	0.037075246	0.030915657	1289	tags=87%, list=47%, signal=46%	WNT10A/TNF/CDH3/SFN/FOXQ1/LSR/GLI2/RELA/ZMPSTE24/DNASE1L2/SMO/MSX2/NF1/CLDN4/BCL2/GAL/ELOVL1/SOX21/EDA/SOX9
GOMF_INORGANIC_MOLECULAR_ENTITY_TRANSMEMBRANE_TRANSPORTER_ACTIVITY	GOMF_INORGANIC_MOLECULAR_ENTITY_TRANSMEMBRANE_TRANSPORTER_ACTIVITY	GOMF_INORGANIC_MOLECULAR_ENTITY_TRANSMEMBRANE_TRANSPORTER_ACTIVITY	109	0.240280698	1.955960768	0.001574348	0.03718338	0.031005825	1702	tags=85%, list=62%, signal=33%	SEC61A1/ATP2B3/KCNAB3/KCNJ9/SCN2B/ATP1A3/CLCN2/SLC16A1/COX8A/SLC12A9/SLC32A1/CLIC1/SLC25A5/ATP7B/ATP2A3/KCNMB1/SLC29A1/SLC41A3/SLC12A8/SLC25A22/CACNB3/ATP13A1/KCNB2/KCNAB2/CLIC5/TRPM2/SLC6A8/KCNB1/ABCC10/ANXA6/OTOP2/COX7A1/SLC5A11/COX15/CACNA1E/KCNS2/KCNG1/KCNH4/GRIK5/KCNS3/ATP2A1/CCDC51/KCNK5/SLC30A9/CLCA4/CLDN4/CHRNA7/SLC17A8/SLC13A4/OTOP3/SLC26A1/SLC31A1/SLC34A2/SLC4A5/SLC9A8/SLC11A1/ITPR3/AQP6/SLC2A13/SLC25A12/KCNK7/SLC25A23/SLC10A5/SLC22A8/KCNH6/SLC34A3/KCNJ1/AQP1/CLCN7/CACNA1B/TRPM4/SLC22A11/TPCN1/GRIN2C/ITPR1/SLC17A7/COX7B/GRIN1/ANXA9/SLC34A1/SLC13A2/HTR1B/ATP6V1G2/SLC30A3/ATP6V0A1/CNNM2/NIPA2/TMCO3/OTOP1/HCN3/CLCC1/KCNMB3/ATP1A2
GOBP_DNA_INTEGRITY_CHECKPOINT_SIGNALING	GOBP_DNA_INTEGRITY_CHECKPOINT_SIGNALING	GOBP_DNA_INTEGRITY_CHECKPOINT_SIGNALING	28	-0.389039823	-2.298854655	0.001606167	0.03773204	0.031463333	703	tags=68%, list=26%, signal=51%	BARD1/E2F1/CRADD/CHEK2/TIPRL/FZR1/RINT1/RAD17/DOT1L/BLM/TIMELESS/CRY1/CDC14B/ATR/RAD51/MDC1/DONSON/INTS7/CEP63
GOMF_CYSTEINE_TYPE_ENDOPEPTIDASE_ACTIVITY	GOMF_CYSTEINE_TYPE_ENDOPEPTIDASE_ACTIVITY	GOMF_CYSTEINE_TYPE_ENDOPEPTIDASE_ACTIVITY	13	-0.527744532	-2.163630388	0.001617258	0.037790508	0.031512087	755	tags=77%, list=28%, signal=56%	ESPL1/USP8/CAPN3/USP48/USP30/SENP1/USP1/USP37/SENP5/USP6
GOBP_DNA_CONFORMATION_CHANGE	GOBP_DNA_CONFORMATION_CHANGE	GOBP_DNA_CONFORMATION_CHANGE	23	-0.411646484	-2.259185851	0.001659731	0.038577766	0.032168552	756	tags=65%, list=28%, signal=48%	ERCC3/GTF2H2/RAD54B/RECQL4/RUVBL1/CHD1L/DHX30/BLM/TOP1MT/SETX/RAD51/TOP3A/RFC4/WRN/RAD50
GOBP_REGULATION_OF_APOPTOTIC_SIGNALING_PATHWAY	GOBP_REGULATION_OF_APOPTOTIC_SIGNALING_PATHWAY	GOBP_REGULATION_OF_APOPTOTIC_SIGNALING_PATHWAY	75	0.273417642	2.019033521	0.001704122	0.039401087	0.032855089	1320	tags=69%, list=48%, signal=37%	AKT1/CTTN/BAG5/MAGED1/MAPK8IP2/TNF/ITM2C/BCL2L2/BAX/HSPB1/LTB/ASAH2/BOK/GATA4/LTBR/APAF1/PPP2R1A/FYN/BBC3/SLC25A5/ATF4/NRP1/IFI6/FGFR1/PTPRC/BAD/BCAP31/ENO1/RELA/CTSK/PPIF/MNT/WFS1/ITGA6/NFE2L2/NF1/YAP1/MDM2/BCL2/WNT1/PTPN1/BCL2L1/SNAI2/TRAF1/TNFSF10/CTH/CTSC/PYCARD/FAIM/MYC/FIS1/BMP4
GOBP_MITOTIC_C	GOBP_MITOTIC_C	GOBP_MITOTIC_C	168	-0.172237956	-1.807708051	0.0017340	0.039884	0.033257	665	tags=38%,	VRK1/MAD2L1BP/CHEK2/USP8/FZR1/AURKC/DC

ELL_CYCLE	ELL_CYCLE	ELL_CYCLE				94	173	916		list=24%, signal=30%	UN1D3/KIF4A/MTA3/CDC7/RINT1/HSPA2/TRIP13/RAD17/PPP6C/RAE1/AURKAIP1/RANBP1/NUSAP1/BLM/CDCA8/CENPH/KIF23/PINX1/TPR/DYNC1LI1/WDR62/PLRG1/CDC25C/PAFAH1B1/CPSF3/CKS2/TUBG1/CEP250/CDC14B/MYH10/SMC3/TFDP1/BRD7/CCNB2/NDE1/FBXO7/RAD51/MDC1/MYBL1/CENPE/SPAG5/BBS4/USP37/ZW10/TAF10/CDK5RAP2/C1orf112/DONSON/RAD50/KLF11/KATNB1/CCNH/PCNA/GMNN/ANAPC10/ASNS/EFHC1
GOBP_NEGATIVE_REGULATION_OF_TOR_SIGNALING	GOBP_NEGATIVE_REGULATION_OF_TOR_SIGNALING	GOBP_NEGATIVE_REGULATION_OF_TOR_SIGNALING	11	-0.572690172	-2.173020937	0.001744815	0.039921723	0.033289227	603	tags=82%, list=22%, signal=64%	YWHAZ/UBE3A/ENDOG/UBE2N/MAPK3/TBK1/ATXN3/PRKAA1/TSSK2
GOBP_CELLULAR_RESPONSE_TO_BIOTIC_STIMULUS	GOBP_CELLULAR_RESPONSE_TO_BIOTIC_STIMULUS	GOBP_CELLULAR_RESPONSE_TO_BIOTIC_STIMULUS	36	0.360481027	2.089822641	0.001763947	0.040150364	0.033479882	854	tags=61%, list=31%, signal=43%	AKT1/ABL1/NFKBIL1/TNF/TSPO/SYK/APAF1/IRAK1/CTSG/CXCL13/LY86/CDK4/HAVCR2/GHSR/BCR/RELA/NFKB1/ZMPSTE24/TNIP2/HMGB1/WFS1/HMGCS2
GOBP_MOLTING_CYCLE_PROCESS	GOBP_MOLTING_CYCLE_PROCESS	GOBP_MOLTING_CYCLE_PROCESS	17	0.495484949	2.157238094	0.001801228	0.040787595	0.034011246	1289	tags=94%, list=47%, signal=50%	WNT10A/TNF/CDH3/FOXQ1/GLI2/RELA/ZMPSTE24/DNASE1L2/SMO/MSX2/NF1/BCL2/GAL/SOX21/EDA/SOX9
GOBP_REGULATION_OF_MUSCLE_CELL_DIFFERENTIATION	GOBP_REGULATION_OF_MUSCLE_CELL_DIFFERENTIATION	GOBP_REGULATION_OF_MUSCLE_CELL_DIFFERENTIATION	20	0.454931765	2.112278467	0.001871462	0.041900891	0.034939581	1320	tags=90%, list=48%, signal=47%	MAML1/PPARA/SUPT6H/MMP14/WNT3A/MYOD1/ARRB2/MAPK11/NKX2-5/EDN1/MDM2/BCL2/FRS2/CTH/IGF2/ENG/TMEM119/BMP4
HP_X_LINKED_RECESSIVE_INHERITANCE	HP_X_LINKED_RECESSIVE_INHERITANCE	HP_X_LINKED_RECESSIVE_INHERITANCE	36	0.358177506	2.076468404	0.001880569	0.041900891	0.034939581	1298	tags=78%, list=48%, signal=41%	ATP2B3/ABCD1/FAM50A/LAS1L/SSR4/BRWD3/MSN/AP1S2/SYP/MID1/DKC1/SLC6A8/GPRASP2/BCAP31/EMD/PRPS1/BCORL1/PGK1/PQBP1/MED12/OPN1LW/LAGE3/NYX/COL4A6/ABCB7/EDA/FHL1/PHKA2
GOBP_REGULATION_OF_CELLULAR_COMPONENT_SIZE	GOBP_REGULATION_OF_CELLULAR_COMPONENT_SIZE	GOBP_REGULATION_OF_CELLULAR_COMPONENT_SIZE	75	0.271726967	2.006548847	0.001898083	0.041900891	0.034939581	1290	tags=69%, list=47%, signal=38%	CTTN/ABL1/CFL2/AP2M1/GDI1/SLC12A9/RAC1/SEMA6C/ARPC5/ARHGAP4/UCN/SPTAN1/WNT3A/MSN/CDK5/SLC12A8/ADD1/CLN3/NRP1/COTL1/BLOC1S1/CLNS1A/SSH3/MYADM/PPP1R15A/TMOD4/IQGAP3/DNM2/CLN8/CCDC51/CDH4/RAB11A/EDN1/SEMA4B/PEX11B/TMOD1/CDC42EP5/ELN/FCHSD1/OXSR1/MTPN/HCLS1/TRIOBP/ULK1/WNK1/KEL/ZFYVE27/VASP/SH3BP1/ARHGAP18/PYCARD/ACTN2
GOBP_POSITIVE_REGULATION_OF_PHOSPHORYLATION	GOBP_POSITIVE_REGULATION_OF_PHOSPHORYLATION	GOBP_POSITIVE_REGULATION_OF_PHOSPHORYLATION	108	0.236297094	1.914925974	0.001889558	0.041900891	0.034939581	1421	tags=71%, list=52%, signal=36%	AKT1/EIF4G1/FXR2/ABL1/CLDN3/TNF/CDK2AP1/GPRC5B/RAC1/SYK/ADRA2B/IRAK1/OSBP/ANG/FYN/UCN/CALM3/WNT3A/ITGB1BP1/PHB2/CLN3/PDGFRB/AGER/NRP1/FGFR1/PTPRC/NTF3/FGF1/

											MTCP1/FGF3/BAD/CD4/ADAM17/DIRAS1/IQGAP3/ARRB2/LIMK2/SQSTM1/ILK/SPN/EPHA8/EGR1/MERTK/ABI1/CARD10/ITGA6/EDN1/CD6/CD300A/CHRNA7/MAP2K3/BCL2/HCLS1/TEK/WNT1/PTPN1/SLC11A1/ROR2/ERBB2/EHD4/IGF2/ENG/HIPK2/SOX9/DAB2/TMEM119/BMP4/LRP8/TRAF4/PROM2/EMP2/LYN/ADIPOQ/VEGFB/EPHA4/IQGAP1/MMD2
GOBP_RESPONSE_TO_ENDOGENOUS_STIMULUS	GOBP_RESPONSE_TO_ENDOGENOUS_STIMULUS	GOBP_RESPONSE_TO_ENDOGENOUS_STIMULUS	305	0.167492735	1.780930402	0.001886476	0.041900891	0.034939581	1330	tags=59%, list=49%, signal=34%	AKT1/DDX54/LRP6/FAM20C/CSK/PPARA/WNT10A/ATP1A3/UBE2L3/DLX1/PTP4A3/ABL1/LBH/TNF/MMP14/RXRB/TIMP1/STUB1/BCL2L2/DENND4C/TSPO/ITGA3/GNB1/ZBTB7A/STAT3/SAFB2/HSPB1/PRMT2/FCER1G/GATA4/SYK/PPP2R5B/GNA11/PDK2/APAF1/KLF2/ADIPOR1/INPPL1/ANG/TRIM16/FYN/UCN/ADIPOR2/LPIN2/ITGB1BP1/RASL11B/HEYL/CDK5/ADAMTSL2/PHB2/CXCL13/ARPC1B/CHRNB2/PDGFRB/AGER/NRP1/APLN/TRPM2/GPT2/ITGB1/FGFR1/SHC1/PTPRC/MSI1/HCRTR1/GHSR/FZD4/NTF3/CAD/FBN1/SMAD7/LRP1/FGF1/FGF3/PITX3/KHK/RAPGEF1/RAB8A/MYOD1/CRHR2/ADAM17/RELA/NFKB1/ZMPSTE24/CTSK/DIAPH1/FAM107A/TYK2/EMD/KLF9/TSHB/ADAMTS7/ARRB2/TNXB/COL4A2/PAX9/ILK/EPHA8/CARD9/GRB7/COL3A1/GATA5/EGR1/MSX2/SIRT2/HMGCS2/SCGB1A1/PEG10/UCN3/ELK1/EHMT2/EDN1/CLDN5/LRG1/SLC2A1/NFE2L2/MEN1/NFIA/EP300/YAP1/CLDN4/MDM2/HTRA1/CHRNA7/LHB/MAP2K3/EIF2B1/BCL2/SSTR3/VPS11/GAL/HCLS1/MMRN2/SOCS1/AKR1C1/TEK/WNT1/ZNF703/PTPN1/BCL2L1/SNAI2/MAS1L/CRHR1/SLC34A2/DHH/CA2/RGS10/RGMB/MAOB/PRMT5/ALAD/PPP3CA/GPD1/PALM/COL4A6/ZFYVE27/ERBB2/CHRD/FRS2/EHD4/TNFSF10/CASP4/FOSL2/FOLR2/IGF2/GNAI1/WT1/ENG/LTBP1/HIPK2/SOX9/ACTN2/TFPI/DAB2/HRH3/MYC/BMP4/LRP8/BTG2/ITGB8
GOBP_POSITIVE_REGULATION_OF_PHOSPHORUS_METABOLIC_PROCESS	GOBP_POSITIVE_REGULATION_OF_PHOSPHORUS_METABOLIC_PROCESS	GOBP_POSITIVE_REGULATION_OF_PHOSPHORUS_METABOLIC_PROCESS	115	0.235862486	1.949681586	0.001910408	0.041962118	0.034990636	1421	tags=71%, list=52%, signal=36%	AKT1/EIF4G1/FXR2/PPARA/ABL1/CLDN3/TNF/CDK2AP1/GPRC5B/RAC1/SYK/ADRA2B/IRAK1/OSBP/ANG/FYN/UCN/CALM3/WNT3A/ITGB1BP1/PHB2/CLN3/PDGFRB/AGER/NRP1/FGFR1/PTPRC/NTF3/FGF1/MTCP1/FGF3/BAD/CD4/ENO1/ADAM17/DIRAS1/PPP1R15A/GNA12/IQGAP3/ARRB2/LIMK2/SQSTM1/ILK/SPN/EPHA8/EGR1/MERTK/ABI1/CAR

HP_ABNORMAL_GASTROINTESTINAL_MOTILITY	HP_ABNORMAL_GASTROINTESTINAL_MOTILITY	HP_ABNORMAL_GASTROINTESTINAL_MOTILITY	22	0.445567385	2.173038783	0.001932328	0.042232412	0.035216024	1288	tags=86%, list=47%, signal=46%	D10/ITGA6/EDN1/CD6/CD300A/CHRNA7/MAP2K3/BCL2/HCLS1/TEK/WNT1/PTPN1/SLC11A1/ROR2/ERBB2/EHD4/IGF2/ENG/HIPK2/SOX9/DAB2/TMEM119/BMP4/LRP8/TRAF4/PROM2/EMP2/LYN/ADIPOQ/VEGFB/EPHA4/CAMTA1/IQGAP1/MMD2ATP1A3/HMBS/STAT3/MYH11/GMPPA/FAH/CDK4/SLC6A8/SMO/WFS1/AAAS/RAD21/CLCA4/SERPINA1/MDM2/SLC11A1/COL4A6/WT1/COL4A5
GOBP_POSITIVE_REGULATION_OF_CELL_CELL_ADHESION	GOBP_POSITIVE_REGULATION_OF_CELL_CELL_ADHESION	GOBP_POSITIVE_REGULATION_OF_CELL_CELL_ADHESION	66	0.29182744	2.046924607	0.002023816	0.04401298	0.036700773	1652	tags=86%, list=60%, signal=35%	AKT1/CSK/ABL1/TNF/ACTL6B/SYK/MAGI1/IRAK1/FYN/CTSG/WNT3A/CXCL13/AGER/PTPRC/EFNB1/HAVCR2/SMAD7/GLI2/BAD/CD4/RELA/THY1/TYK2/SART1/ST3GAL4/SPN/HMGB1/HLA-DRB3/CD6/HLA-A/FLOT2/MYO10/HLA-E/SOCS1/HLA-DRB5/KLHL25/HLA-DQB1/PPP3CA/AIF1/CHST2/IGF2/PYCARD/HLA-DMB/HLA-DOB/LYN/ALOX5/ADA/PODXL/CCL5/BTN2A2/XBP1/PTPN23/HAS2/CITED2/HLA-DMA/EMILIN2/CD276
GOBP_PROTEIN_LOCALIZATION_TO_ORGANELLE	GOBP_PROTEIN_LOCALIZATION_TO_ORGANELLE	GOBP_PROTEIN_LOCALIZATION_TO_ORGANELLE	176	-0.172314944	-1.850883162	0.002042387	0.044058941	0.036739098	738	tags=40%, list=27%, signal=31%	ZFAND6/RANBP2/TIMM23/KPNA6/BANP/PEX10/ARL1/NUP107/HK1/VRK1/DNAJB6/PPHLN1/VPS37C/VPS37A/RTN4/PPP3CB/PAF1/BCAP29/CEP72/GSK3B/ANK2/DNAJC15/PINK1/RGPD5/CSNK2A2/PINX1/TPR/POLA2/IFT122/PLRG1/TULP2/NCOA4/CEP350/PMPCB/CEP250/NF2/ATR/ZMYND10/AKAP11/LAMP3/FBXO7/NUP88/CAMLG/PRKAA1/SAE1/MON1A/KIAA0753/SPAG5/BBS4/NUP155/CSE1L/ZW10/TTC21A/KPNA2/GLUL/MTX2/SH3GLB1/RER1/WRN/RAB8B/UBE2D3/HACL1/PARD6A/IFT20/HSPA1L/LZTFL1/SPATA7/SRP19/DZIP1/SNUPN/OSBPL8
GOBP_DEFENSE_RESPONSE	GOBP_DEFENSE_RESPONSE	GOBP_DEFENSE_RESPONSE	269	0.172565378	1.763631217	0.002045988	0.044058941	0.036739098	1452	tags=64%, list=53%, signal=33%	AKT1/EIF4G1/ABCD1/KLKB1/GNG7/VDAC1/PPARA/EXTL3/NFKBIL1/MAPK8IP2/TNF/DEFB105A/TIMP1/GPRC5B/TMED1/MGST2/STAT3/LGALS3BP/CDC37/MOV10/LSM14A/TAP1/XCR1/FCER1G/RAC1/SYK/TNFRSF4/RNF185/ADAM15/PTGES/ITGAM/IRAK1/ANG/IFITM3/FYN/ORM2/UCN/GAPDH/CTSG/GPSM3/EIF2AK1/PHB2/CXCL13/PTN/TRIM8/LY86/MEFV/AGER/COTL1/IFI6/ITGB1/EIF2AK4/PI3/SHC1/C8A/PTPRC/HAVCR2/ABI3/GHSR/ZNFX1/MICA/LRP1/RAB20/ALOX5AP/EIF2AK2/BCR/IFI27/CD4/OAS2/IL1R1/ISG15/NKIRAS2/ADAM17/RELA/STAB1/NFKB1/CCL22/ZMPSTE24/IFNGR1/CUEDC2/TNIP2/TYK2/ATP1B1/ARRB2/DAPK3/CEBPA/

											SMO/SPN/CARD9/HMGB1/C9/SIRT2/SCGB1A1/TFEB/EDN1/IL17A/COLEC11/NFE2L2/TBKBP1/ABHD12/EP300/CHID1/CD6/HLA-A/SERPINA1/UNC93B1/CD300A/NPLOC4/NFAM1/FLOT2/PRSS3/PQBP1/CHRNA7/MAP2K3/BCL2/IFITM1/MAPKAPK3/HLA-E/MMRN2/DLEC1/TEK/MPEG1/HLA-B/UMOD/PTPN1/PUM1/BCL2L1/TYROBP/PLA2G4C/SLC11A1/LSP1/CCR5/HLA-F/LAG3/AIF1/IFNAR2/CHST2/LRSAM1/PRDX1/IFIT3/ILF3/PARP4/CASP4/FOSL2/FOLR2/CR2/CTNNBIP1/CTSC/CD177/PYCARD/H19/LRP8/UBL7/C3AR1/HLA-C/ADAMTS5/CEBPB/TRAF4/TRIM41/LYN/ADIPOQ/ALOX5/PLSCR1/AQP1/RHBDD3/DEFB123/RHBDF2/RAB1A/ADA/TRIM44/TARBP2/APOD/C3
GOBP_CELLULAR_RESPONSE_TO_LIPID	GOBP_CELLULAR_RESPONSE_TO_LIPID	GOBP_CELLULAR_RESPONSE_TO_LIPID	101	0.243850034	1.955515799	0.002067176	0.044298065	0.036938494	731	tags=42%, list=27%, signal=32%	AKT1/DDX54/LRP6/PPARA/ATP1A3/UBE2L3/CYP7A1/ABL1/NFKBIL1/LBH/TNF/RXRB/BCL2L2/TSPO/GNB1/ZBTB7A/SAFB2/PRMT2/SYK/ADAM15/IRAK1/CTSG/WNT3A/KCNMB1/HEYL/MSN/PHB2/CXCL13/LY86/GRAMD1A/CDK4/HAVCR2/GHSR/FZD4/BAD/BCR/MYOD1/RELA/NFKB1/FAM107A/TNIP2/KLF9
HP_INFERTILITY	HP_INFERTILITY	HP_INFERTILITY	29	-0.38674525	-2.288857394	0.002149495	0.044981103	0.037508055	679	tags=59%, list=25%, signal=45%	FOXJ1/AURKC/TRIP13/BLM/FKBP6/CATSPER2/STAG3/KLHL10/ZMYND10/DNAH8/DNAH17/DNAI1/TTC21A/GGPS1/DNALI1/DZIP1/IFT74
GOMF_PEPTIDE_BINDING	GOMF_PEPTIDE_BINDING	GOMF_PEPTIDE_BINDING	64	0.291866784	2.041694519	0.002117518	0.044981103	0.037508055	1423	tags=77%, list=52%, signal=38%	SEC61A1/MAML1/PFDN1/CLTB/ATP1A3/MAPK8IP2/ITM2C/AP2M1/TAP1/ITGAM/ANG/PCSK5/CLSTN1/AGER/GPR37/HCRTR1/PPIH/GPRASP2/GHSR/FZD4/MICA/LRP1/CRHR2/RELA/CD1B/PPIF/MCHR1/TOMM20/CRIP1/HLA-DRB3/HLA-A/CHRNA7/SSTR3/PPIC/HLA-E/HLA-B/CRHR1/HLA-DRB5/HLA-DQB1/HLA-F/PPP3CA/ECE1/HLA-DMB/LRP8/HLA-C/HLA-DOB/EPHA4/CLSTN3/CACNA1B
GOBP_REGULATION_OF_DNA_BINDING_TRANSCRIPTION_FACTOR_ACTIVITY	GOBP_REGULATION_OF_DNA_BINDING_TRANSCRIPTION_FACTOR_ACTIVITY	GOBP_REGULATION_OF_DNA_BINDING_TRANSCRIPTION_FACTOR_ACTIVITY	62	0.291955207	2.030053914	0.002145564	0.044981103	0.037508055	1086	tags=61%, list=40%, signal=38%	AKT1/CRTC3/LRP6/MDFI/NFKBIL1/TNF/ZBTB7A/STAT3/PRMT2/SYK/TNFRSF4/IRAK1/HEYL/PHB2/TRIM8/AGER/EIF2AK4/RWDD3/HAVCR2/FZD4/SMAD7/EIF2AK2/RELA/NHLH2/ARRB2/SMO/NEUROG1/CARD9/WFS1/EOMES/EDN1/MEN1/EP300/NFAM1/FLOT2/MTPN/HCLS1/WNT1
GOCC_SPINDLE	GOCC_SPINDLE	GOCC_SPINDLE	88	-0.226112882	-1.978856958	0.002141935	0.044981103	0.037508055	823	tags=52%, list=30%, signal=38%	NUDCD2/TUBG2/RASSF1/SMC6/BUB1B/MAPRE1/ESPL1/SPAST/CBX1/CCDC117/MAD2L1BP/IQCB1/AURKC/KIF4A/CDC7/HSPA2/POLB/RAE1/MAPKBP1/NUSAP1/CDCA8/KIF23/PINX1/TPR/KATNA1/D

											YNC1LI1/WDR62/PAFAH1B1/CEP350/TUBG1/CDC14B/SMC3/DR1/NDE1/CENPE/SPAG5/ZW10/CDK5RAP2/C1orf112/TBCCD1/HMMR/KIF3A/SPAG8/KATNB1/CEP63/EFHC1
GOBP_CALCIUM_ION_HOMEOSTASIS	GOBP_CALCIUM_ION_HOMEOSTASIS	GOBP_CALCIUM_ION_HOMEOSTASIS	60	0.281051648	1.926104883	0.002150246	0.044981103	0.037508055	1579	tags=82%, list=58%, signal=35%	ATP2B3/ABL1/BAX/STIM1/XCR1/BOK/CALM3/ATP7B/ATP2A3/ATF4/ATP13A1/CLN3/TRPM2/LIME1/PTPRC/ANXA6/CDH23/HCRTR1/SV2A/BCAP31/F2RL3/THY1/DIAPH1/ATP1B1/WFS1/ATP2A1/GRM1/EDN1/CHRNA7/BCL2/UMOD/GPR12/ITPR3/KEL/CCR5/GP1BB/SLC25A23/FIS1/LYN/TPCN1/CCL5/ITPR1/IBTK/NPTN/GRIN1/CCL8/GSTO1/ATG5/HTR1B
GOBP_REGULATION_OF_EPITHELIAL_CELL_PROLIFERATION	GOBP_REGULATION_OF_EPITHELIAL_CELL_PROLIFERATION	GOBP_REGULATION_OF_EPITHELIAL_CELL_PROLIFERATION	66	0.291051043	2.041478833	0.002165339	0.045082152	0.037592315	1395	tags=76%, list=51%, signal=38%	AKT1/MAGED1/TNF/CDH3/BAX/SFN/STAT3/MTSS1/ANG/WNT3A/VASH1/PHB2/PTN/CDK4/NRP1/APLN/FGFR1/GHSR/NKX2-8/SLURP1/FGF1/LIMS2/BAD/ADAM17/GLI1/IQGAP3/KLF9/DEAF1/FOXE3/SMO/HMGB1/NKX2-5/LRG1/NF1/YAP1/MMRN2/TEK/ZNF703/SNAI2/POLD4/ERBB2/FRS2/IGF2/SOX9/DAB2/MYC/BMP4/ALOX5/VEGFB/TWIST1
HP_ATONIC_SEIZURE	HP_ATONIC_SEIZURE	HP_ATONIC_SEIZURE	17	0.490061228	2.133624344	0.00218378	0.045251636	0.037733641	502	tags=59%, list=18%, signal=48%	AP2M1/GAMT/GNB1/COX8A/MTHFS/SLC32A1/ALDH4A1/SLC25A22/TRIM8/KCNB1
HP_GRAY_MATTER_HETEROTOPIA	HP_GRAY_MATTER_HETEROTOPIA	HP_GRAY_MATTER_HETEROTOPIA	19	-0.437192063	-2.167397565	0.002196267	0.045296718	0.037771234	436	tags=58%, list=16%, signal=49%	WDR62/PAFAH1B1/POMT1/TUBG1/ARFGEF2/SPEN/DHX16/CENPE/CDK5RAP2/KATNB1/CEP63
GOBP_POSITIVE_REGULATION_OF_LYMPHOCYTE_ACTIVATION	GOBP_POSITIVE_REGULATION_OF_LYMPHOCYTE_ACTIVATION	GOBP_POSITIVE_REGULATION_OF_LYMPHOCYTE_ACTIVATION	57	0.302205945	2.045711627	0.002220936	0.045591462	0.03801701	1506	tags=81%, list=55%, signal=37%	AKT1/CSK/ABL1/MMP14/ACTL6B/SYK/TNFRSF4/FYN/WNT3A/CHRNB2/AGER/PTPRC/EFNB1/HAVCR2/GLI2/BAD/CD4/THY1/TNIP2/TYK2/SART1/SPN/HMGB1/HLA-DRB3/CD6/HLA-A/FLOT2/BCL2/HLA-E/SOCS1/HLA-DRB5/KLHL25/TYROBP/HLA-DQB1/HLA-F/PPP3CA/AIF1/IGF2/PYCARD/HLA-DMB/HLA-DOB/LYN/ADA/CCL5/BTN2A2/XBP1
GOBP_REGULATION_OF_RHO_PROTEIN_SIGNAL_TRANSDUCTION	GOBP_REGULATION_OF_RHO_PROTEIN_SIGNAL_TRANSDUCTION	GOBP_REGULATION_OF_RHO_PROTEIN_SIGNAL_TRANSDUCTION	18	0.474407255	2.131421829	0.002268133	0.046343758	0.038644321	883	tags=72%, list=32%, signal=49%	ABL1/HEG1/ITGA3/RAC1/TNFAIP1/NRP1/ITGB1/NET1/ARHGDIA/BCR/F2RL3/COL3A1/ARHGDIB
GOCC_NUCLEOLUS	GOCC_NUCLEOLUS	GOCC_NUCLEOLUS	177	-0.166798658	-1.791244642	0.002307087	0.04692145	0.039126036	604	tags=33%, list=22%, signal=28%	TFIP11/ZCCHC9/MRPS31/NANOG/PAF1/TRAIP/RAD17/ERGIC2/RAE1/MAPKBP1/NUSAP1/BLM/PA2G4/SUZ12/TNP2/CAPN3/CDCA8/GTPBP4/CENPH/PINX1/ZNF330/PLRG1/NHEJ1/SPECC1/NIP7/NARF/PPP1CC/UTP18/PRKRIP1/CDC14B/UBE2T/ATXN3/NF2/RNMT/STAG3/PNMA1/ZFP91/AKAP11/SETX/

GOBP_MEIOTIC_CELL_CYCLE_PROCESS	GOBP_MEIOTIC_CELL_CYCLE_PROCESS	GOBP_MEIOTIC_CELL_CYCLE_PROCESS	30	-0.366278101	-2.22187931	0.002374702	0.047894354	0.039937305	682	tags=63%, list=25%, signal=48%	RAD51/ELL3/GEMIN4/CARF/DDX52/C6orf89/EXOSC8/AFF4/ZNF692/SENP5/EBNA1BP2/MTX2/WRN/CCDC86/SURF2/RGS22/LRRC34/SRP19/ACTR6/PWP1 TUBG2/ESPL1/RAD54B/FZR1/PIWIL2/HSPA2/TRIP13/CDC25C/RMI1/CKS2/TUBG1/PTTG1/STAG3/TDRKH/PTTG2/CCNB2/RAD51/MYBL1/RAD50
GOBP_LYMPHOCYTE_ACTIVATION	GOBP_LYMPHOCYTE_ACTIVATION	GOBP_LYMPHOCYTE_ACTIVATION	134	0.212828562	1.841175521	0.002387631	0.047894354	0.039937305	1356	tags=64%, list=50%, signal=34%	AKT1/CSK/ABL1/SUPT6H/MMP14/ACTL6B/SDC4/BAX/ZBTB7A/STAT3/PURA/FCER1G/SYK/TNFRSF4/KIF13B/LFNG/FYN/SLC25A5/CTSG/WNT3A/CD79A/MSN/CMTM7/PHB2/CHRNB2/AGER/ITGB1/EIF2AK4/PTPRC/EFNB1/HAVCR2/MICA/SMAD7/GLI2/BAD/CD4/ADAM17/PRR7/THY1/TNIP2/TYK2/SART1/SPN/HMGB1/EGR1/MERTK/SCGB1A1/EOMES/HLA-DRB3/MEN1/EP300/CD6/HLA-A/CD300A/NFAM1/FLOT2/CHRNA7/BCL2/IMPDH2/GAL/HLA-E/SOCS1/WNT1/HLA-DRB5/KLHL25/TYROBP/SLC11A1/HLA-DQB1/HLA-F/LAG3/HSH2D/PPP3CA/AIF1/ERBB2/PRDX1/FOSL2/IGF2/CR2/PYCARD/MAFB/HLA-DMB/BMP4/POU2AF1/HLA-DOB/CEBPB/EMP2
GOBP_RNA_PROCESSING	GOBP_RNA_PROCESSING	GOBP_RNA_PROCESSING	157	-0.174836027	-1.799458649	0.002377473	0.047894354	0.039937305	847	tags=46%, list=31%, signal=34%	PRPF40A/LSM6/RBBP6/BOP1/BARD1/ISG20/DUS3L/DHX15/NOL6/ENY2/POP4/RBM14/THOC7/DUS1L/CPEB1/PRPF38A/METTL8/DNTTIP2/PIWIL2/LSM3/THADA/ZRANB2/METTL6/TRIT1/TFIP11/PAF1/CPSF2/SF3A1/PRPF40B/INTS6/PA2G4/GTPBP4/FKBP6/PLRG1/PPWD1/CPSF3/ZNF473/UTP18/PRKRIP1/RNMT/SUPT3H/TDRKH/PDCD7/TAF9/SETX/DHX16/GEMIN4/CSTF1/DDX52/KHDRBS3/EXOSC8/TPRKB/NUP155/DAZAP1/TSN/TAF10/ELAVL4/EBNA1BP2/ZNHIT3/NCBP2/BUD13/PRPF18/SRPK1/INTS7/GEMIN6/IWS1/TDRD7/SMAD2/DDX20/PWP1/PNPT1/TRPT1
GOBP_POSITIVE_REGULATION_OF_LEUKOCYTE_CELL_CELL_ADHESION	GOBP_POSITIVE_REGULATION_OF_LEUKOCYTE_CELL_CELL_ADHESION	GOBP_POSITIVE_REGULATION_OF_LEUKOCYTE_CELL_CELL_ADHESION	55	0.304865632	2.036546434	0.00247327	0.049163233	0.040995376	1506	tags=80%, list=55%, signal=37%	AKT1/CSK/ABL1/TNF/ACTL6B/SYK/IRAK1/FYN/AGER/PTPRC/EFNB1/HAVCR2/GLI2/BAD/CD4/RELA/THY1/TYK2/SART1/ST3GAL4/SPN/HMGB1/HLA-DRB3/CD6/HLA-A/FLOT2/HLA-E/SOCS1/HLA-DRB5/KLHL25/HLA-DQB1/PPP3CA/AIF1/CHST2/IGF2/PYCARD/HLA-DMB/HLA-DOB/LYN/ALOX5/ADA/CCL5/BTN2A2/XBP1
GOBP_REGULATION_OF_RESPONS	GOBP_REGULATION_OF_RESPONS	GOBP_REGULATION_OF_RESPONSE_	178	0.2003937	1.866458817	0.002468315	0.049163233	0.040995376	1523	tags=71%, list=56%,	AKT1/SOX15/KLKB1/PPARA/EXTL3/CLDN3/NFKBIL1/TNF/RXRB/GPRC5B/BAX/MGST2/STAT3/CD

E_TO_EXTERNAL_STIMULUS	E_TO_EXTERNAL_STIMULUS	TO_EXTERNAL_STIMULUS								signal=33%	C37/LSM14A/HSPB1/RAC1/SYK/RNF185/PTGES/SEMA6C/KREMEN1/IRAK1/FYN/UCN/WNT3A/GPSM3/PHB2/CXCL13/PTN/GRK1/LY86/MEFV/PDGFRB/AGER/NRP1/STX3/EIF2AK4/FGFR1/PTPRC/HAVCR2/SERPINE2/GHSR/NTF3/ZNFX1/MICA/FGF1/ALOX5AP/EIF2AK2/BCR/IL1R1/ISG15/MYOD1/PLAT/ADAM17/RELA/NFKB1/ZMPSTE24/TNIP2/ST3GAL4/ARRB2/CEBPA/SPN/CARD9/HMGB1/SIRT2/SCGB1A1/EDN1/IL17A/COLEC11/NFE2L2/SEMA4B/ABHD12/EP300/HLA-A/UNC93B1/CD300A/NPLOC4/FLOT2/PQBP1/CHRNA7/OXSR1/BCL2/MAPKAPK3/HLA-E/MMRN2/TEK/HLA-B/PTPN1/PUM1/SNAI2/TYROBP/MMP28/WNK1/HLA-F/LAG3/AIF1/LRSAM1/CASP4/CTSC/PYCARD/TFPI/LRP8/C3AR1/CEBPB/TRIM41/LYN/ADIPOQ/ALOX5/VEGFB/PLSCR1/EPHA4/CLASP2/RHBDD3/RHBDF2/ADA/TRIM44/TARBP2/C3/KIR2DL4/CCL5/SEMA3C/PJA2/NR1H2/PTPN2/CHD8
GOBP_PERIPHERAL_NERVOUS_SYSTEM_DEVELOPMENT	GOBP_PERIPHERAL_NERVOUS_SYSTEM_DEVELOPMENT	GOBP_PERIPHERAL_NERVOUS_SYSTEM_DEVELOPMENT	19	0.459741769	2.114787929	0.00249301	0.049332405	0.041136442	1208	tags=84%, list=44%, signal=47%	AKT1/ISL2/NDRG1/PRX/CDK5/NTF3/CNTNAP1/RELA/NHLH2/ILK/SIRT2/GFRA3/NF1/MED12/NFASC/ERBB2
GOCC_LATERAL_PLASMA_MEMBRANE	GOCC_LATERAL_PLASMA_MEMBRANE	GOCC_LATERAL_PLASMA_MEMBRANE	15	0.522373806	2.169338428	0.002515632	0.049556821	0.041323574	1079	tags=87%, list=40%, signal=53%	CLDN3/SLC16A1/PPP2R1A/CLDN15/SNTA1/GJB1/KCNB1/GNA12/IQGAP3/ATP1B1/CLDN5/CLDN4/ATP1B2

Table S5: GSEA results of ASNS

	ID	Description	setSize	enrichmentScore	NES	pvalue	p.adjust	qvalue	rank	leading_edge	core_enrichment
GOBP_MALE_GAMETE_GENERATION	GOBP_MALE_GAMETE_GENERATION	GOBP_MALE_GAMETE_GENERATION	108	0.470434618	4.280175848	8.70E-18	4.20E-14	3.37E-14	640	tags=56%, list=20%, signal=47%	TDRD7/SRPK1/MYCBP/TBPL1/MYCBPAP/TTC21A/TCFL5/LZTFL1/DZIP1/SPA17/DAZAP1/RFX2/ROPN1L/PTTG1/STRBP/SPATA6/SPAG8/CCIN/SETX/AKAP4/CSNK2A2/CATSPER2/TSSK2/IQCF1/FKBP6/RPL39L/SFMBT1/ZPBP2/PAFAH1B1/ACTL7A/MAST2/ELL3/MYBL1/ACRBP/KLHL10/TCP11/TESK2/CDC25C/HSPA2/PRSS21/PIWIL2/PLEKHA1/RUVBL1/PPP1CC/TRIP13/CCDC42/TDRKH/FOXJ1/HSF2/AFF4/SPOCD1/IQCG/IFT20/BBS4/INHBB/PARP11/MKKS/BOLL/TNP2/SPAG4/MKRN2
GOBP_SEXUAL_REPRODUCTION	GOBP_SEXUAL_REPRODUCTION	GOBP_SEXUAL_REPRODUCTION	183	0.364473122	3.806532257	1.10E-16	2.66E-13	2.13E-13	640	tags=45%, list=20%, signal=38%	TDRD7/SRPK1/MYCBP/DDX20/TBPL1/MYCBPAP/TTC21A/TCFL5/CCNB2/LZTFL1/DZIP1/SPA17/HSPA1L/DAZAP1/RFX2/PTTG2/ROPN1L/PTTG1/STRBP/STAG3/SPATA6/SPAG8/CCIN/SETX/AKAP4/SPACA3/CSNK2A2/CKS2/CATSPER2/TSSK2/RAD51/IQCF1/FKBP6/RPL39L/SFMBT1/ZPBP2/PAFAH1B1/ACTL7A/MAST2/ELL3/MYBL1/ACRBP/SMC1B/KLHL10/TCP11/TESK2/WNT3/CDC25C/AURKC/HSPA2/PRSS21/PIWIL2/SMC3/ANAPC10/PLEKHA1/RUVBL1/PPP1CC/TRIP13/CCDC42/TDRKH/FOXJ1/LYZL6/HSF2/AFF4/FZR1/SPOCD1/UBE3A/TUBG1/IQCG/TIAL1/IFT20/BBS4/TOP3A/RAD50/INHBB/PARP11/ESPL1/MKKS/BOLL/TNP2/SPAG4/TUBG2/MKRN2
GOBP_GAMETE_GENERATION	GOBP_GAMETE_GENERATION	GOBP_GAMETE_GENERATION	138	0.37834017	3.662104682	5.43E-14	8.74E-11	6.99E-11	658	tags=48%, list=21%, signal=40%	TDRD7/SRPK1/MYCBP/DDX20/TBPL1/MYCBPAP/TTC21A/TCFL5/CCNB2/LZTFL1/DZIP1/SPA17/DAZAP1/RFX2/ROPN1L/PTTG1/STRBP/SPATA6/SPAG8/CCIN/SETX/AKAP4/CSNK2A2/CATSPER2/TSSK2/IQCF1/FKBP6/R

											PL39L/SFMBT1/ZPBP2/PAFAH1B1/ACTL7A/MAST2/ELL3/MYBL1/ACRBP/KLHL10/TCP11/TESK2/WNT3/CDC25C/HSPA2/PRSS21/PIWIL2/PLEKHA1/RUVBL1/PPP1CC/TRIP13/CCDC42/TDRKH/FOXJ1/HSF2/AFF4/SPOCD1/IQCG/TIAL1/IFT20/BBS4/INHBB/PARP11/MKKS/BOLL/TNP2/SPAG4/MKRN2/PAQR7
GOBP_CHROMOSOME_ORGANIZATION	GOBP_CHROMOSOME_ORGANIZATION	GOBP_CHROMOSOME_ORGANIZATION	129	0.35944482	3.405050256	6.87E-12	8.29E-09	6.64E-09	923	tags=61%, list=29%, signal=45%	SPAG5/RFC4/SUGT1/SMCHD1/PTTG2/PTTG1/STAG3/NUP155/SETX/CENPE/RAD51/PCNA/CDCA8/SMC1B/DHX30/DOT1L/KATNB1/RAE1/TOP1MT/AURKC/CENPH/RECQL4/HSPA2/ITGB3BP/TPR/SMC3/BLM/RUVBL1/ATF7IP/TRIP13/TFIP11/BRD7/MAPK3/PINX1/KIF23/CDK5RAP2/MAD2L1BP/DYNC1LI1/TUBG1/WRN/TRIM28/BUB1B/CHD1L/TOP3A/GTF2H2/RAD50/CHEK2/KIF4A/ESPL1/KPNB1/SMC6/TPX2/TUBG2/YLPM1/ATR/ERCC3/ZW10/PPHLN1/RFC1/MAD2L2/KIF22/AKAP8L/MCM5/NUSAP1/NUP107/SMARCC1/POT1/PARP1/MAP3K4/HAT1/CDC16/MCM3/BCCIP/HMGA1/UBE2C/HMGB3/PDCD6IP/RIF1/MAPRE1
GOBP_DNA_REPAIR	GOBP_DNA_REPAIR	GOBP_DNA_REPAIR	126	0.354703926	3.371274134	1.85E-11	1.78E-08	1.43E-08	808	tags=53%, list=25%, signal=41%	RFC4/C1orf112/SMCHD1/NUDT1/PTTG1/MDC1/SETX/USP1/CHAF1B/RAD51/CDC14B/UBE2D3/PCNA/POLB/DOT1L/TRAIP/UBE2T/RNF138/RECQL4/PAXIP1/SMC3/BLM/RUVBL1/TRIP13/SUPT3H/UBE2N/RAD17/TFIP11/BRD7/CCDC117/TAF6/ATXN3/TIMELESS/FZR1/WRN/TRIM28/CHD1L/CDC7/CEBPG/TAF10/TOP3A/GTF2H2/RAD50/CHEK2/BARD1/POLE/SMC6/GTF2H1/ATR/ERCC3/NHEJ1/RFC1/CDK7/MAD2L2/RMI1/RCHY1/KIF22/MBD4/FANCC/WDR33/TAF9/MCM5/SMARCC1/POT1/VCP/TEX264/PARP1

GOCC_NUCLEAR_PROTEIN_CONTAINING_COMPLEX	GOCC_NUCLEAR_PROTEIN_CONTAINING_COMPLEX	GOCC_NUCLEAR_PROTEIN_CONTAINING_COMPLEX	251	0.264049014	2.958745578	2.99E-11	2.41E-08	1.93E-08	964	tags=53%, list=30%, signal=40%	NR1H3/DDX20/TBPL1/RFC4/NUP88/BUD13/PRKRIP1/BRD1/CCNH/INTS7/SAE1/NUP155/DHX16/GEMIN4/CPSF3/RAD51/AHCTF1/PCNA/CLMN/ELL3/RANBP1/STAT4/SAP30/PRPF18/SNUPN/RAE1/DR1/TLE4/EXOSC8/PRPF40B/SF3A1/TPR/PAXIP1/PRIM1/ANAPC10/RUVBL1/SUZ12/CSTF1/SUPT3H/PCGF6/ACTR6/TFIP11/BRD7/UTP18/POLA2/SMAD2/PAF1/INTS6/RGPD5/PRPF38A/ZC3H8/TAF6/AFF4/TIMELESS/NFYB/FZR1/PDCD7/PHF21A/TEX10/TRIM28/BUB1B/CEBPZ/TFDP1/CEBPG/TAF10/GTF2H2/RAD50/UBE2S/DNTTIP1/PARP11/BARD1/BOP1/POLE/KPNB1/XPO4/GTF2H1/NUP93/THOC5/SPAG4/E2F1/EXOSC4/CPSF2/ATR/POLR3E/ERCC3/NOL6/NHEJ1/CDK7/RCOR3/CHD5/CREB3/MED4/ARID4B/LSM3/PRPF40A/GTF2E1/BRD9/FANCC/BAZ1A/WDR33/TAF9/SAP130/SETD1A/MCM5/NUP107/SMARCC1/PPWD1/POT1/CSTF3/RANBP2/CPSF1/SUPT5H/CDC16/SMG7/BRMS1/MCM3/BCCIP/HMGA1/UBE2C/XPOT/DHX35/SIRT1/PLRG1/PRPF6/ENY2/CTNNBL1/POLR1C/TRIP4/DHX15/NUP37/LUC7L/RNMT/UBAP2L
HP_ABNORMAL_MALE_GERM_CELL_MORPHOLOGY	HP_ABNORMAL_MALE_GERM_CELL_MORPHOLOGY	HP_ABNORMAL_MALE_GERM_CELL_MORPHOLOGY	10	0.914160401	3.288730103	1.17E-10	7.73E-08	6.19E-08	284	tags=100%, list=9%, signal=91%	TTC21A/DNALI1/DZIP1/IFT74/CATSPER2/FKBP6/KLHL10/DNAH17/AURKC/DNAH8
GOBP_CELL_ADHESION	GOBP_CELL_ADHESION	GOBP_CELL_ADHESION	310	-0.256052693	-2.795988463	1.28E-10	7.73E-08	6.19E-08	1319	tags=58%, list=41%, signal=38%	MYOC/PDGFRA/DLG4/ENG/FYN/CLSTN1/TMEM47/BCAM/TNXB/PCDHB14/CLDN4/CD276/CLASP2/TNF/CBFB/CDH9/IL15/PTPN2/LRP6/THY1/SYK/PARVB/CCR1/CTSG/VEZT/APOD/AMIGO3/STX3/SELPLG/PODXL/NF1/ACTN2/CLDN5/AGR2/COL4A6/PLXNB2/BCR/TRIOBP/SOCS1/OMG/SRPX/CYP1B1/ATP1B1/VWF/BCL2/TNFRSF21/ABI3BP/WNT1/PCDHB4/SNAI2/CLSTN3/FGL2/ZNF703/TSC1/

											WNT3A/NRP2/ETS1/CXCL13/PLAU/HSPB1/FXYD5/CXCR3/ADIPOQ/CCL5/EMP2/CD4/SERPINB8/BAD/CD6/PPARA/HLA-DMA/ILK/ITGB8/HLA-A/PARVG/COL15A1/FAM107A/CD34/CITED2/PCDHB16/SPN/NFASC/BTN2A2/CDH4/CORO2B/MINK1/SMAD7/ALOX5/HLA-E/XBP1/RELA/CCL21/ITGAM/TRO/TGFBI/FOLR2/PYCARD/SERPINE2/CEBPB/MYADM/INPPL1/NRP1/AIF1/BMP4/TEK/EDA/PKP2/HLA-DOB/COL6A3/SLURP1/LRG1/CLDN3/DAB2/CDH11/HLA-DRB5/LYN/HLA-DMB/GNE/CNTNAP1/STAB1/DAB1/AGER/FZD4/RAB1A/CDH3/PLXNA1/FLOT2/ADAM12/LAG3/RSU1/CD177/CHRD/COL3A1/HAS2/HLA-DQB1/IGF2/AKT1/SCGB1A1/CSK/AMIGO2/RAC1/ARHGEF7/PTPRC/RGMB/LRFN3/PERP/TFE3/CTTN/PPP3CA/HLA-DRB3/ITGA3/PPP2R1A/GLI2/ITGB1/CDK5/ADAM15/F2RL3/MAGI1/MSN/ST3GAL4/IRAK1/LAMC3/HMGB1/MMP14/CLIC1/HAVCR2/TOR1A/ITGA9/ITGB7/TYK2/FBN1/PARVA/CLDN15/TRIP6/SHC1/PCDHGA8/EFNB1/SIGLEC12/ITGB1BP1/ABL1/LGALS3BP
GOBP_MULTICELLULAR_ORGANISM_REPRODUCTION	GOBP_MULTICELLULAR_ORGANISM_REPRODUCTION	GOBP_MULTICELLULAR_ORGANISM_REPRODUCTION	169	0.304488336	3.116601603	1.73E-10	9.20E-08	7.37E-08	388	tags=33%, list=12%, signal=30%	TDRD7/SRPK1/MYCBP/DDX20/TBPL1/MYCBPAP/TTC21A/TCFL5/CCNB2/LZTFL1/DZIP1/SPA17/DAZAP1/RFX2/ROPN1L/PTTG1/STRBP/SPATA6/SPAG8/CCIN/SETX/AKAP4/SPACA3/CSNK2A2/CATSPER2/TSSK2/IQCF1/FKBP6/RPL39L/SFMBT1/ZPBP2/PAFAH1B1/PCNA/ACTL7A/MAST2/ELL3/MYBL1/ACRBP/KLHL10/TCP11/TESK2/WNT3/CDC25C/HSPA2/PRSS21/PIWIL2/PLEKHA1/RUVBL1/PPP1CC/TRIP13/CCDC42/TDRKH/FOXJ1/LYZL6/HSF2
GOMF_MOLECULA	GOMF_MOLECULA	GOMF_MOLECULAR_T	241	-0.280838607	-2.899454198	1.90E-10	9.20E-08	7.37E-08	1627	tags=70%, list=51%,	OR2A12/IFNAR1/P2RY8/TRPV1/GPR

R_TRANSDUCER_ACTIVITY	R_TRANSDUCER_ACTIVITY	RANSDUCER_ACTIVITY								signal=37%	3/SIGLEC7/TAS2R10/CD300A/CNR2/CX3CR1/REG4/IL21R/GABRR1/LTBP1/OR2J2/CD7/OR4P4/OR4M1/CHRNA7/OR5D18/TAS2R60/OR13C8/CR2/VAC14/IL9R/OR1D2/MCHR1/OR10A4/OR1F1/MERTK/EXTL3/OR5AS1/GRIA4/OR11L1/OR52N2/PDGFRA/OR52B6/OR51L1/ENG/RRH/BCAM/MRGPRX2/CLDN4/TAS2R41/OR4D1/TMEM63A/LRP6/OR4K14/GPRC5B/CCR1/BTN1A1/OR6B2/RXFP3/GPR119/OR2B11/GPR12/SSTR3/KISS1R/FSHR/TAS2R40/SQSTM1/CHRNA2/OR8A1/RARG/GPR21/TAAR6/OR4S1/FFAR2/PLXNB2/NR1H2/TAAR5/GPR15/OR8S1/OR7D2/OR51S1/OR8D1/NRP2/IL1R1/CXCR3/HTR1B/CD4/CCR5/OR10J5/PPARA/OR10T2/GPR83/CD79A/GPR135/OR6M1/RXRB/M6PR/SPN/C3AR1/OR1D4/OR52M1/KIR2DL4/OR8G1/OR6K2/FOLR2/OR6Y1/TAS2R9/OR7G2/GPR78/OR1E2/OR4K17/PTCH2/MRGPRX3/OR8B8/TNFRSF4/NRP1/OR9K2/TEK/GHSR/OR13C4/HLA-DOB/CRHR2/HRH3/DCBLD2/CLDN3/CNTNAP1/MRGPRF/AGER/SMO/MTNR1A/ADIPOR2/FZD4/PLXNA1/IGF2R/OR6V1/GRM1/OR6C4/OR10H4/LAG3/XCR1/ANTXR2/OR52D1/PROKR2/TAS2R39/P2RX2/HLA-DQB1/OR2T4/OR51A7/GRIK5/GFRA3/OR6X1/OR4D5/PTPRC/RGMB/ADRA2B/FCER1G/OR8H2/HLA-DRB3/MAS1L/OR3A3/GPR39/PDGFRB/ITGB1/SCARB2/OR5F1/F2RL3/OR5V1/OR52E4/ADIPOR1/IFNGR1/CHRNB2/STAT3/FGFR1/DERL1/TSPO
GOBP_SPERMATID_DIFFERENTIATION	GOBP_SPERMATID_DIFFERENTIATION	GOBP_SPERMATID_DIFFERENTIATION	40	0.542701849	3.562022454	6.30E-10	2.77E-07	2.22E-07	618	tags=65%, list=19%, signal=53%	SRPK1/TBPL1/TTC21A/DZIP1/RFX2/ROPN1L/STRBP/AKAP4/CATSPER2/TSSK2/IQCF1/ZPBP2/PAFAH1B1/ACTL7A/MAST2/ACRBP/KLHL10/TCP11/HSPA2/TRIP13/CCDC42/AFF4/IQC

											G/BBS4/MKKS/TNP2
GOCC_CILIUM	GOCC_CILIUM	GOCC_CILIUM	110	0.352786935	3.211527401	7.08E-10	2.85E-07	2.28E-07	518	tags=41%, list=16%, signal=36%	KIF3A/EFHC1/SPAG5/TTC21A/DNALI1/LZTFL1/DZIP1/DYNLRB2/IQCD/SPA17/IFT74/DYNLL2/ROPN1L/CCDC96/SPATA6/SPAG8/AKAP4/SPACA3/C20orf85/CLUAP1/CATSPER2/SPATA7/PAFAH1B1/ACTL7A/WDR54/TCP11/DNAH17/IFT122/GPI/DNAH8/ARFGEF2/CEP250/IQCE/LYZL6/STARD10/TULP2/CAPZB/SSNA1/DNAI1/TUBG1/IQCG/IFT20/NME7/BBS4/IQCB1
GOBP_CELL_CYCLE	GOBP_CELL_CYCLE	GOBP_CELL_CYCLE	382	0.215851693	2.635822982	1.02E-09	3.78E-07	3.02E-07	742	tags=37%, list=23%, signal=32%	ASNS/SRPK1/GMNN/KIF3A/EFHC1/SPAG5/DONSON/KLF11/MDM4/CCNB2/C1orf112/SUGT1/FBXO7/SH3GLB1/PTTG2/PTTG1/CCNH/STAG3/INTS7/SPAG8/MDC1/PARD6A/CSNK2A2/CHAF1B/CKS2/CPSF3/CENPE/CENPM/RAD51/FKBP6/CDC14B/AHCTF1/PAFAH1B1/PCNA/WDR62/MYBL1/CDCA8/SMC1B/RANBP1/DOT1L/KATNA1/KATNB1/CDC25C/SUV39H2/CEP63/RAE1/CRY1/NDE1/DR1/AURKC/NF2/CENPH/PNPT1/HSPA2/PDCD2L/ITGB3BP/TPR/PAXIP1/PIWIL2/SMC3/ANAPC10/CEP250/BLM/RUVBL1/PPP1CC/PPP6C/TRIP13/GTPBP4/ZMYND11/AURKAIP1/CEP72/RAD17/C6orf89/CCDC42/TDRKH/BRD7/MAPK3/PAF1/MAGEA4/PINX1/KIF23/BANP/CDK5RAP2/VRK1/USP37/TAF6/MYH10/SENP5/MAD2L1BP/TIMELESS/FZR1/SSNA1/KIAA0753/DYNC1LI1/TUBG1/WRN/BUB1B/EPB41L2/BBS4/CDC7/TFDP1/TAF10/MAPK6/TOP3A/RAD50/UBE2S/CHEK2/CAPN3/RBM14/KIF4A/BARD1/BOP1/POLE/ESPL1/KPNB1/SMC6/BIRC8/GTF2H1/BOLL/THOC5/ZBTB17/TPX2/TUBG2/E2F1/GAK/RASSF1/ATR/ERCC3/ZW10/CDK7/MAD2L2/ZC3HC1/RMI1/RINT1/HERC5/KIF22/PRPF40A/AKAP8L/USP8/

GOBP_DNA_METABOLIC_PROCESS	GOBP_DNA_METABOLIC_PROCESS	GOBP_DNA_METABOLIC_PROCESS	202	0.272389742	2.924324675	1.44E-09	4.34E-07	3.48E-07	818	tags=46%, list=26%, signal=37%	TUSC2/DCUN1D3 GMNN/KPNA2/DONSON/TSN/RFC4/C1orf112/SMCHD1/NUDT1/PTTG1/POLG2/MDC1/SETX/USP1/CHAF1B/FAF1/RAD51/CDC14B/UBE2D3/PCNA/GFER/POLB/DOT1L/TRAIP/UBE2T/RNF138/TOP1MT/RECQL4/PAXIP1/PRIM1/SMC3/RLF/BLM/RUVBL1/POLRMT/ATF7IP/TRIP13/SUPT3H/GTPBP4/UBE2N/RAD17/TFIP11/BRD7/POLA2/MAPK3/PINX1/CCDC117/USP37/TAF6/ATXN3/TIMELESS/FZR1/SPOCD1/WRN/TRIM28/CHD1L/CDC7/TFDP1/CEBPG/TAF10/TOP3A/GTF2H2/RAD50/CHEK2/BARD1/POLE/TTF1/SMC6/GTF2H1/EXOSC4/YLPM1/ATR/ERCC3/NHEJ1/RFC1/CDK7/MAD2L2/RMI1/ARID4B/RCHY1/KIF22/MBD4/FANCC/BAZ1A/WDR33/TAF9/ENDOG/MCM5/SMARCC1/POT1/VCP/TEX264/PARP1/MAP3K4
GOBP_G_PROTEIN_COUPLED_RECEPTOR_SIGNALING_PATHWAY	GOBP_G_PROTEIN_COUPLED_RECEPTOR_SIGNALING_PATHWAY	GOBP_G_PROTEIN_COUPLED_RECEPTOR_SIGNALING_PATHWAY	203	-0.291153101	-2.88457509	1.35E-09	4.34E-07	3.48E-07	1673	tags=74%, list=52%, signal=38%	ADM/P2RY10/RAMP2/ECE1/DGKG/ROCK2/OR2A12/P2RY8/PDCL/GPR3/TAS2R10/CNR2/CX3CR1/ARR3/NPFF/ARHGEF1/OR2J2/OR4P4/OR4M1/OR5D18/TAS2R60/OR13C8/PRKAR1A/OR1D2/MCHR1/PALM/OR10A4/OR1F1/OR5AS1/PCSK1N/CCL8/OR11L1/UCN/OR52N2/YWHAB/OR52B6/OR51L1/RRH/MRGPRX2/TAS2R41/OR4D1/GHRH/CCL22/OR4K14/GPRC5B/CCR1/OR6B2/RXFP3/GPR119/SYP/OR2B11/GPR12/SSTR3/KISS1R/FSHR/TAS2R40/RGS12/OR8A1/ACTN2/GPR21/TAAR6/ARRB2/OR4S1/FFAR2/TAAR5/GPR15/OR8S1/OR7D2/OR51S1/RPH3AL/OR8D1/ABHD6/CXCR3/HTR1B/CCL5/CCR5/OR10J5/OR10T2/GPR83/INS/GPR135/OR6M1/RGS14/C3AR1/OR1D4/OR52M1/OR8G1/TSHB/FRS2/RELA/CCL21/OR6K2/OR6Y1/TAS2R9/OR7G2/GPR78/UCN3/OR1E2/G

											NB1/GNAI1/OR4K17/MRGPRX3/OR8B8/OR9K2/GHSR/OR13C4/ITPR1/CRHR2/RGS11/HRH3/CALM3/ENTPD2/MRGPRF/SMO/MTNR1A/FZD4/IGF2R/OR6V1/GRM1/RGS10/OR6C4/OR10H4/XCR1/OR52D1/PROKR2/TAS2R39/OR2T4/OR51A7/AKT1/GRK1/OR6X1/OR4D5/RAC1/GNA12/RGS6/ADRA2B/PPP3CA/OR8H2/MAS1L/OR3A3/GPR39/PDGFRB/UBQLN2/GNG7/OR5F1/F2RL3/OR5V1/APLN/OR52E4/CRTC3/KCTD12
GOCC_MICROTUBULE_CYTOSKELETON	GOCC_MICROTUBULE_CYTOSKELETON	GOCC_MICROTUBULE_CYTOSKELETON	272	0.247693185	2.823061663	1.27E-09	4.34E-07	3.48E-07	794	tags=42%, list=25%, signal=34%	KIF3A/EFHC1/SPAG5/DNALI1/CCNB2/TBCCD1/LZTFL1/C1orf112/DZIP1/DYNLRB2/IQCD/CCDC112/IFT74/DYNLL2/STRBP/CCDC96/RAB3IP/SPAG8/HMMR/PARD6A/CLUAP1/SPATA7/CENPE/TSSK2/RAD51/ZMYND10/CDC14B/PAFAH1B1/PCNA/WDR62/MAST2/CDCA8/RANBP1/CCHCR1/POLB/KATNA1/CCDC77/DNAH17/IFT122/KATNB1/CEP63/RAE1/SPECC1/NDE1/DR1/AURKC/HSPA2/TPR/DNAH8/UBN1/MAPKBP1/HSPH1/ARFGEF2/SMC3/CEP250/RUVBL1/RRAGD/CEP72/FKBP4/CEP350/MAP2K2/PINX1/KIF23/CDK5RAP2/CCDC117/LYST/MAPKAPK2/MAD2L1BP/SSNA1/KIAA0753/DNAI1/DYNC1LI1/TUBG1/WRN/IQCG/KIFC3/IFT20/NME7/BUB1B/BBS4/IQCB1/CDC7/MAP6D1/KIF4A/VPS37A/SLC1A4/ESPL1/RASSF7/SMC6/MKKS/NUP93/RASSF3/TPX2/TUBG2/E2F1/CBX1/RASSF1/KIFAP3/ZW10/HOOK2/KBTBD8/MAD2L2/AKAP11/GSK3B/KIF22/CEP290/RAB28/RAB11FIP4/NUSAP1/MARK1/PACRG/TUBB/DCUN1D5/ZNF415
GOCC_CHROMOSOMAL_REGION	GOCC_CHROMOSOMAL_REGION	GOCC_CHROMOSOMAL_REGION	80	0.404049465	3.388654027	1.57E-09	4.46E-07	3.57E-07	960	tags=66%, list=30%, signal=48%	SPAG5/C1orf112/SUGT1/SMCHD1/STAG3/SETX/CENPE/CENPM/RAD51/AHCTF1/PAFAH1B1/PCNA/CDCA8/SMC1B/SUV39H2/NDE1/AURKC/CE

										NPH/RECQL4/ITGB3BP/TPR/SMC3/BLM/PPP1CC/RAD17/TFIP11/BRD7/PINX1/DYNC1LI1/WRN/BUB1B/RAD50/CHEK2/SMC6/THOC5/CBX1/ATR/ZW10/KIF22/BAZ1A/MCM5/NUP107/SMARCC1/POT1/PARP1/HAT1/TP53BP1/MCM3/RIF1/ZNF330/CSNK1A1/NUP37/UHRF2
GOBP_MICROTUBULE_BASED_PROCESS	GOBP_MICROTUBULE_BASED_PROCESS	GOBP_MICROTUBULE_BASED_PROCESS	168	0.290756845	2.979392065	2.86E-09	7.61E-07	6.10E-07	680	tags=42%, list=21%, signal=35%	KIF3A/EFHC1/SPAG5/TTC21A/CCNB2/LZTFL1/DZIP1/DYNLRB2/SUGT1/SPA17/IFT74/DYNLL2/ROPN1L/AKAP4/PARD6A/CLUAP1/CATSPER2/SPATA7/CENPE/IQCF1/ZMYND10/CDC14B/PAFAH1B1/WDR62/CDCA8/RANBP1/KATNA1/DNAH17/IFT122/KATNB1/CEP63/RAE1/NDE1/AURKC/CENPH/PRKAA1/TPR/DNAH8/SMC3/CEP250/CEP72/FKBP4/CEP350/CCDC42/FOXJ1/KIF23/CDK5RAP2/ATXN3/SSNA1/KIAA0753/DNAI1/DYNC1LI1/TUBG1/IQCG/KIFC3/IFT20/BBS4/CHEK2/MAP6D1/RBM14/KIF4A/CNTN2/ESPL1/KPNB1/MKKS/TPX2/TUBG2/SLK/KIFAP3/ZW10/HOOK2
GOCC_CATALYTIC_COMPLEX	GOCC_CATALYTIC_COMPLEX	GOCC_CATALYTIC_COMPLEX	364	0.216490364	2.613872885	2.99E-09	7.61E-07	6.10E-07	809	tags=40%, list=25%, signal=34%	TBPL1/TSN/DNALI1/FBXO15/RFC4/CCNB2/ATP6V1E2/DYNLRB2/SUGT1/FBXO7/PSMA6/DYNLL2/BRD1/CACYBP/CCNH/POLG2/SAE1/AKAP4/PARD6A/C15orf48/CSNK2A2/CKS2/DERL2/COX5A/APH1B/TSSK2/RAD51/UBE2D3/PSMD12/PAFAH1B1/PCNA/FBXO24/FBXL18/PSMF1/DNAH17/SAP30/DR1/PNPT1/PRKAA1/EXOSC8/SF3A1/TPR/DNAH8/ASB1/PAXIP1/PRIM1/ANAPC10/BLM/RUVBL1/POLRMT/PPP1CC/ATP6V1F/SUZ12/SUPT3H/UBE2N/PCGF6/RRAGD/UBE2D2/ACTR6/TFIP11/BRD7/POLA2/PAF1/RMND5B/PRKAG2/WDR26/TAF6/UXS1/FBXO39/FZR1/FBXL2/DNAI1/DYNC1LI1/UBE3A/PHF21A/TEX10/ADRM1/BUB1B/ENO3/CAB39/KCTD10/T

											AF10/TOP3A/SHARPIN/GTF2H2/NDUFS7/UBE2S/DNTTIP1/NOXO1/BARD1/POLE/TBK1/SMC6/CLPX/DERL3/TRAF2/GTF2H1/ATP6V0A2/PIK3CG/NDUFS6/EXOSC4/POLR3E/PPP3CB/PMPCB/ERCC3/NHEJ1/RFC1/CDK7/NDUFA12/KBTBD8/COX6A1/RCOR3/MAD2L2/BCKDHA/NDUFB6/RMI1/KRTCAP2/CHD5/ARID4B/RCHY1/GSK3B/DDA1/PSENEN/LSM3/GTF2E1/BRD9/ATG12/DCUN1D3/BAZ1A/RMND5A/TAF9/KLHDC2/PSMD8/NDUFC2/KLHL8/SAP130/SETD1A/RNF7/PIGP/SMARCC1/PIGH/DCUN1D5/PPWD1/PPP2R3B/RANBP2/VCP/GNG4
GOBP_DEVELOPMENTAL_PROCESS_INVOLVED_IN_REPRODUCTION	GOBP_DEVELOPMENTAL_PROCESS_INVOLVED_IN_REPRODUCTION	GOBP_DEVELOPMENTAL_PROCESS_INVOLVED_IN_REPRODUCTION	188	0.27843675	2.920968465	3.27E-09	7.69E-07	6.16E-07	623	tags=38%, list=19%, signal=32%	TDRD7/SRPK1/MYCBP/DDX20/TBPL1/MYCBPAP/TTC21A/TCFL5/LZTFL1/DZIP1/SPA17/EIF2B4/NUDT1/DAZAP1/RFX2/ROPN1L/PTTG1/STRBP/SPATA6/SPAG8/CCIN/SETX/AKAP4/CSNK2A2/CATSPER2/TSSK2/IQCF1/FKBP6/RPL39L/SFMBT1/ZPBP2/PAFAH1B1/ACTL7A/MAST2/ELL3/MYBL1/ACRBP/KLHL10/TCP11/TESK2/TESC/CDC25C/HSPA2/PRSS21/ASB1/PIWIL2/PLEKHA1/RUVBL1/PPP1CC/TRIP13/DNAJB6/NCOA4/FKBP4/CCDC42/TDRKH/FOXJ1/HSF2/AFF4/SPOCD1/UBE3A/IQCG/TIAL1/IFT20/BBS4/SPINT2/INHBB/PARP11/MKKS/BOLL/TNP2/SPAG4
GOBP_REPRODUCTION	GOBP_REPRODUCTION	GOBP_REPRODUCTION	275	0.234322905	2.683119244	3.34E-09	7.69E-07	6.16E-07	640	tags=34%, list=20%, signal=30%	TDRD7/SRPK1/MYCBP/DDX20/TBPL1/MYCBPAP/TTC21A/TCFL5/CCNB2/LZTFL1/DZIP1/SPA17/EIF2B4/NUDT1/HSPA1L/DAZAP1/RFX2/PTTG2/ROPN1L/PTTG1/STRBP/STAG3/SPATA6/SPAG8/CCIN/SETX/AKAP4/SPACA3/CSNK2A2/CKS2/CATSPER2/TSSK2/RAD51/IQCF1/FKBP6/RPL39L/SFMBT1/ZPBP2/PAFAH1B1/PCNA/ACTL7A/MAST2/ELL3/MYBL1/ACRBP/SMC1B/KLHL10/TCP11/TESK2/TESC/

												WNT3/CDC25C/AURKC/HSPA2/PRSS21/ASB1/PIWIL2/SMC3/ANAPC10/PLEKHA1/RUVBL1/PPP1CC/TRIP13/DNAJB6/NCOA4/FKBP4/CCDC42/TDRKH/FOXJ1/LYZL6/HSF2/AFF4/FZR1/SPOCD1/DNAI1/UBE3A/TUBG1/IQCG/TRIM28/TIAL1/IFT20/BBS4/TOP3A/SPINT2/RAD50/INHBB/PARP11/ESPL1/MKKS/BOLL/TNP2/SPAG4/TUBG2/MKRN2
GOMF_G_PROTEIN_COUPLED_RECEPTOR_ACTIVITY	GOMF_G_PROTEIN_COUPLED_RECEPTOR_ACTIVITY	GOMF_G_PROTEIN_COUPLED_RECEPTOR_ACTIVITY	131	-0.332569123	-2.940839533	5.29E-09	1.16E-06	9.30E-07	1759	tags=82%, list=55%, signal=38%		GPR37/HCRTR1/GLP1R/NPBWR2/OR10H3/P2RY10/RAMP2/OR2A12/P2RY8/GPR3/TAS2R10/CNR2/CX3CR1/OR2J2/OR4P4/OR4M1/OR5D18/TAS2R60/OR13C8/OR1D2/MCHR1/OR10A4/OR1F1/OR5AS1/OR11L1/OR52N2/OR52B6/OR51L1/RRH/MRGPRX2/TAS2R41/OR4D1/OR4K14/GPRC5B/CCR1/OR6B2/RXFP3/GPR119/OR2B11/GPR12/SSTR3/KISS1R/FSHR/TAS2R40/OR8A1/GPR21/TAAR6/OR4S1/FFAR2/TAAR5/GPR15/OR8S1/OR7D2/OR51S1/OR8D1/CXCR3/HTR1B/CCR5/OR10J5/OR10T2/GPR83/GPR135/OR6M1/C3AR1/OR1D4/OR52M1/OR8G1/OR6K2/OR6Y1/TAS2R9/OR7G2/GPR78/OR1E2/OR4K17/MRGPRX3/OR8B8/OR9K2/GHSR/OR13C4/CRHR2/HRH3/MRGPRF/SMO/MTNR1A/FZD4/OR6V1/GRM1/OR6C4/OR10H4/XCR1/OR52D1/PROKR2/TAS2R39/OR2T4/OR51A7/OR6X1/OR4D5/ADRA2B/OR8H2/MAS1L/OR3A3/GPR39/PDGFRB/OR5F1/F2RL3/OR5V1/OR52E4
GOCC_MOTILE_CILIUM	GOCC_MOTILE_CILIUM	GOCC_MOTILE_CILIUM	37	0.534983131	3.303992987	1.24E-08	2.61E-06	2.09E-06	647	tags=68%, list=20%, signal=55%		DNALI1/IQCD/SPA17/IFT74/ROPN1L/SPATA6/AKAP4/SPACA3/CATSPER2/PAFAH1B1/ACTL7A/TCP11/DNAH17/DNAH8/LYZL6/STARD10/CAPZB/SSNA1/DNAI1/IQCG/IFT20/BBS4/MKKS/SPAG4/ATP1B3
GOBP_POSITIVE_REGULATION_OF_C	GOBP_POSITIVE_REGULATION_OF_C	GOBP_POSITIVE_REGULATION_OF_CELL_A	106	-0.347491689	-2.848416474	1.86E-08	3.74E-06	2.99E-06	1257	tags=66%, list=39%, signal=41%		CD276/TNF/CBFB/IL15/THY1/SYK/CTSG/STX3/PODXL/AGR2/TRIOBP/S

ELL_ADHESION	ELL_ADHESION	DHESION									OCS1/ABI3BP/TSC1/WNT3A/ETS1/CXCL13/CCL5/EMP2/CD4/BAD/CD6/HLA-DMA/ILK/HLA-A/CITED2/SPN/BTN2A2/SMAD7/ALOX5/HLA-E/XBP1/RELA/CCL21/PYCARD/MYADM/NRP1/AIF1/TEK/HLA-DOB/DAB2/HLA-DRB5/LYN/HLA-DMB/AGER/FLOT2/RSU1/CHRD/HAS2/HLA-DQB1/IGF2/AKT1/CSK/RAC1/ARHGEF7/PTPRC/TFE3/PPP3CA/HLA-DRB3/ITGA3/GLI2/MAGI1/ST3GAL4/IRAK1/HMGB1/HAVCR2/TYK2/EFNB1/ITGB1BP1/ABL1
GOBP_MICROTUBULE_BASED_MOVEMENT	GOBP_MICROTUBULE_BASED_MOVEMENT	GOBP_MICROTUBULE_BASED_MOVEMENT	59	0.421145103	3.194299175	2.12E-08	3.93E-06	3.15E-06	594	tags=53%, list=19%, signal=44%	KIF3A/TTC21A/LZTFL1/DZIP1/DYNLRB2/SPA17/IFT74/ROPN1L/AKAP4/CLUAP1/CATSPER2/CENPE/IQCF1/ZMYND10/PAFAH1B1/DNAH17/IFT122/KATNB1/NDE1/DNAH8/KIF23/SSNA1/DNAI1/DYNC1LI1/IQCG/KIFC3/IFT20/BBS4/KIF4A/KPNB1/MKKS
GOBP_DNA_DAMAGE_RESPONSE	GOBP_DNA_DAMAGE_RESPONSE	GOBP_DNA_DAMAGE_RESPONSE	186	0.268296778	2.811076574	2.11E-08	3.93E-06	3.15E-06	898	tags=50%, list=28%, signal=38%	DONSON/MDM4/RFC4/C1orf112/SMCHD1/NUDT1/PTTG1/INTS7/MDC1/SETX/USP1/CHAF1B/RAD51/CDC14B/UBE2D3/PCNA/ELL3/POLB/DOT1L/TRAIP/GNL1/CEP63/UBE2T/RNF138/CRY1/RECQL4/PAXIP1/SMC3/BLM/RUVBL1/TRIP13/SUPT3H/UBE2N/RAD17/TFIP11/BRD7/MAPK3/VRK1/CCDC117/MAPKAPK2/TAF6/ATXN3/TIMELESS/FZR1/WRN/TRIM28/CHD1L/CDC7/CEBPG/TAF10/TOP3A/GTF2H2/RAD50/CHEK2/BARD1/POLE/SMC6/GTF2H1/THOC5/E2F1/CBX1/ATR/ERCC3/NHEJ1/RFC1/CDK7/MAD2L2/RMI1/RINT1/RCHY1/KIF22/MBD4/FANCC/WDR33/TAF9/ENDOG/SETD1A/MCM5/SMARCC1/DCUN1D5/POT1/VCP/TEX264/PARP1/USP10/TRAF6/TP53BP1/RBBP6/HIPK1/VAV3/MCM3/POLI/BCCIP
GOCC_INTRACELLULAR_PROTEIN_C	GOCC_INTRACELLULAR_PROTEIN_C	GOCC_INTRACELLULAR_PROTEIN_CONTAI	192	0.25504935	2.692723137	3.21E-08	5.53E-06	4.43E-06	905	tags=48%, list=28%, signal=37%	TBPL1/TSN/FBXO15/CNOT10/DZIP1/SUGT1/FBXO7/PSMA6/NCBP2/BRD

ONTAINING_COMPLEX	ONTAINING_COMPLEX	NING_COMPLEX									1/CACYBP/CCNH/POLG2/WDR41/AKAP4/CSNK2A2/CKS2/DERL2/TSSK2/RAD51/UBE2D3/PSMD12/FBXO24/FBXL18/PSMF1/DR1/PRKAA1/ASB1/PRIM1/ANAPC10/RUVBL1/POLRMT/SUPT3H/UBE2N/PCGF6/UBE2D2/POLA2/PAF1/KIF23/RMND5B/PRKAG2/WDR26/TAF6/FBXO39/FZR1/FBXL2/UBE3A/ADRM1/BUB1B/CNOT7/KCTD10/TAF10/SHARPIN/GTF2H2/UBE2S/BARD1/POLE/DERL3/TRAF2/GTF2H1/POLR3E/PPP3CB/ERCC3/NHEJ1/CDK7/KBTBD8/CNOT6/MAD2L2/RCHY1/GSK3B/DDA1/GTF2E1/DCUN1D3/RMND5A/TAF9/KLHDC2/PSMD8/KLHL8/RNF7/DCUN1D5/DNAJB11/VCP/TRAPPC3/CDC16/ANKRD9/TRAPPC2/KLHL7/CUL2/MCM3/PSMC2/FBXO11/UBE2C/POP4
GOBP_CELL_CYCLE_PROCESS	GOBP_CELL_CYCLE_PROCESS	GOBP_CELL_CYCLE_PROCESS	280	0.22705015	2.587047668	3.12E-08	5.53E-06	4.43E-06	742	tags=38%, list=23%, signal=32%	SRPK1/GMNN/KIF3A/EFHC1/SPAG5/DONSON/KLF11/CCNB2/C1orf112/SUGT1/FBXO7/SH3GLB1/PTTG2/PTTG1/CCNH/STAG3/INTS7/MDC1/PARD6A/CSNK2A2/CKS2/CPSF3/CENPE/CENPM/RAD51/CDC14B/AHCTF1/PAFAH1B1/PCNA/WDR62/MYBL1/CDCA8/SMC1B/RANBP1/DOT1L/KATNB1/CDC25C/CEP63/RAE1/CRY1/NDE1/AURKC/CENPH/HSPA2/ITGB3BP/TPR/PAXIP1/PIWIL2/SMC3/CEP250/BLM/PPP6C/TRIP13/AURKAIP1/CEP72/RAD17/CCDC42/TDRKH/BRD7/PAF1/PINX1/KIF23/CDK5RAP2/VRK1/USP37/MYH10/MAD2L1BP/TIMELESS/FZR1/KIAA0753/DYNC1LI1/TUBG1/WRN/BUB1B/BBS4/CDC7/TFDP1/TAF10/TOP3A/RAD50/UBE2S/CHEK2/CAPN3/RBM14/KIF4A/BARD1/POLE/ESPL1/KPNB1/SMC6/THOC5/ZBTB17/TPX2/TUBG2/E2F1/ATR/ERCC3/ZW10/CDK7/MAD2L2/RMI1/RINT1/KIF22/PRPF40A/AKAP8L/USP8/DCU

											N1D3
GOMF_OLFACTORY_RECEPTOR_ACTIVITY	GOMF_OLFACTORY_RECEPTOR_ACTIVITY	GOMF_OLFACTORY_RECEPTOR_ACTIVITY	58	-0.433846354	-2.941003668	4.26E-08	6.91E-06	5.54E-06	1508	tags=86%, list=47%, signal=46%	OR2J2/OR4P4/OR4M1/OR5D18/OR13C8/OR1D2/OR10A4/OR1F1/OR5AS1/OR11L1/OR52N2/OR52B6/OR51L1/OR4D1/OR4K14/OR6B2/OR2B11/OR8A1/OR4S1/OR8S1/OR7D2/OR51S1/OR8D1/OR10J5/OR10T2/OR6M1/OR1D4/OR52M1/OR8G1/OR6K2/OR6Y1/OR7G2/OR1E2/OR4K17/OR8B8/OR9K2/OR13C4/OR6V1/OR6C4/OR10H4/OR52D1/OR2T4/OR51A7/OR6X1/OR4D5/OR8H2/OR3A3/OR5F1/OR5V1/OR52E4
GOBP_SENSORY_PERCEPTION_OF_CHEMICAL_STIMULUS	GOBP_SENSORY_PERCEPTION_OF_CHEMICAL_STIMULUS	GOBP_SENSORY_PERCEPTION_OF_CHEMICAL_STIMULUS	78	-0.37757974	-2.836916536	4.29E-08	6.91E-06	5.54E-06	1508	tags=79%, list=47%, signal=43%	OR2J2/OR4P4/OR4M1/OR5D18/TAS2R60/OR13C8/OR1D2/OR10A4/OR1F1/OR5AS1/OR11L1/OR52N2/OR52B6/OR51L1/TAS2R41/OR4D1/OR4K14/OR6B2/OR2B11/TAS2R40/REEP2/OR8A1/OR4S1/TAAR5/OR8S1/OR7D2/OR51S1/OR8D1/OR10J5/TTC8/B3GNT2/OR10T2/OR6M1/OR1D4/OR52M1/OR8G1/OR6K2/OR6Y1/TAS2R9/OR7G2/OR1E2/GNB1/OR4K17/OR8B8/OR9K2/OR13C4/NAV2/OR6V1/OR6C4/OR10H4/OR52D1/TAS2R39/P2RX2/OR2T4/OR51A7/OR6X1/OR4D5/OR8H2/OR3A3/OR5F1/OR5V1/OR52E4
GOCC_MICROTUBULE_ORGANIZING_CENTER	GOCC_MICROTUBULE_ORGANIZING_CENTER	GOCC_MICROTUBULE_ORGANIZING_CENTER	169	0.270082142	2.764435731	4.94E-08	7.46E-06	5.97E-06	680	tags=40%, list=21%, signal=33%	KIF3A/EFHC1/SPAG5/CCNB2/TBCCD1/DZIP1/DYNLRB2/IQCD/CCDC112/IFT74/DYNLL2/CCDC96/RAB3IP/HMMR/PARD6A/CLUAP1/SPATA7/TSSK2/RAD51/ZMYND10/CDC14B/PAFAH1B1/PCNA/WDR62/RANBP1/CCHCR1/KATNA1/CCDC77/IFT122/KATNB1/CEP63/SPECC1/NDE1/AURKC/UBN1/ARFGEF2/CEP250/RUVBL1/RRAGD/CEP72/CEP350/KIF23/CDK5RAP2/MAPKAPK2/SSNA1/KIAA0753/DNAI1/DYNC1LI1/TUBG1/WRN/KIFC3/IFT20/NME7/BUB1B/BBS4/IQCB1/VPS37A/SLC1A4/ESPL1/RASSF7/MKKS/NUP93/TUBG2/E2F1/RASSF1/K

											IFAP3/HOOK2
GOMF_SIGNALING_RECEPTOR_BINDING	GOMF_SIGNALING_RECEPTOR_BINDING	GOMF_SIGNALING_RECEPTOR_BINDING	279	-0.238977803	-2.570539059	4.86E-08	7.46E-06	5.97E-06	1575	tags=66%, list=49%, signal=37%	MTSS1/SIX3/CX3CR1/ARR3/BMP2/S100B/TMED1/NPFF/ARHGEF1/S100A12/ITGA5/ATXN2/GRIPAP1/TAP2/PTPN1/LTA/TRAF5/CR2/SEMA4B/MCHR1/RLN3/PALM/WNT2B/SH2B2/MICA/PCSK1N/CCL8/LTB/SRI/GMFG/SH3BP1/UCN/MYOC/PDGFRA/DLG4/ENG/FYN/SNX17/DIAPH1/TNXB/CD276/TNF/GHRH/IL15/PTPN2/LRP6/THY1/SYK/CCL22/ANXA7/GPRC5B/BTN1A1/CTSG/IGFBP6/EFNA2/SSTR3/SELPLG/WNT11/SQSTM1/TRAK2/REEP2/BTNL2/AAK1/CSHL1/ACTN2/INHBA/AGR2/TACC1/ARRB2/HLA-C/NR1H2/ELMO2/FKBP1A/SOCS2/SOCS1/TRAK1/VWF/ANGPTL2/TGFA/AMN/FAM3C/WNT1/CLSTN3/WNT3A/IL1R1/GUCA2A/CXCL13/UNC93B1/MDM2/ANKRA2/NFATC4/LYNX1/ADIPOQ/CCL5/ADAMTS5/EMP2/CD4/OGN/ITGB8/INS/HLA-A/FNTA/HLA-F/TIMP1/TGFBRAP1/BTN2A2/TSHB/SMAD7/FRS2/HLA-E/CCL21/HLA-B/VEGFB/ITGAM/IGFBP4/TGFBI/PYCARD/SERPINE2/PLSCR1/UCN3/WIPI1/CPNE3/GNAI1/PTCH2/DNAJC14/S100A4/BMP4/EDA/SLURP1/ISG15/LRG1/DAB2/ANG/LYN/SMO/TNFSF10/DDX54/TYROBP/NTF3/FGF1/LAG3/CD177/COL3A1/SLA/IGF2/SCP2/TAP1/GFRA3/RALA/PRMT2/PTPRC/GNA12/IL17A/ITGA3/PDGFRB/GPRASP2/STUB1/AP2M1/ARF4/ITGB1/CDK5/ADAM15/MSN/APLN/FLT3LG/HMGB1/GALNT11/MMP14/TRAF1/ITGA9/ITGB7/TYK2/FBN1/STAT3/FGFR1/PTN/DERL1/TRIP6/SHC1/EFNB1/RABEP2/ITGB1BP1/ABL1
GOBP_REGULATION_OF_CELL_ADHESION	GOBP_REGULATION_OF_CELL_ADHESION	GOBP_REGULATION_OF_CELL_ADHESION	172	-0.286604599	-2.69077723	5.78E-08	8.46E-06	6.77E-06	1272	tags=61%, list=40%, signal=39%	TNXB/CD276/CLASP2/TNF/CBFB/IL15/PTPN2/THY1/SYK/CTSG/APOD/STX3/PODXL/NF1/AGR2/PLXNB2/TR

											IOBP/SOCS1/CYP1B1/BCL2/TNFRSF21/ABI3BP/WNT1/SNAI2/FGL2/ZNF703/TSC1/WNT3A/ETS1/CXCL13/PLAU/FXYD5/CXCR3/ADIPOQ/CCL5/EMP2/CD4/BAD/CD6/PPARA/HLA-DMA/ILK/HLA-A/FAM107A/CITED2/SPN/BTN2A2/CORO2B/MINK1/SMAD7/ALOX5/HLA-E/XBP1/RELA/CCL21/TGFBI/PYCARD/SERPINE2/CEBPB/MYADM/NRP1/AIF1/BMP4/TEK/PKP2/HLA-DOB/DAB2/HLA-DRB5/LYN/HLA-DMB/DAB1/AGER/FZD4/PLXNA1/FLOT2/LAG3/RSU1/CHRD/HAS2/HLA-DQB1/IGF2/AKT1/SCGB1A1/CSK/RAC1/ARHGEF7/PTPRC/LRFN3/TFE3/PPP3CA/HLA-DRB3/ITGA3/PPP2R1A/GLI2/ADAM15/MAGI1/ST3GAL4/IRAK1/HMGB1/MMP14/HAVCR2/TYK2/EFNB1/ITGB1BP1/ABL1
GOBP_CELL_CELL_ADHESION	GOBP_CELL_CELL_ADHESION	GOBP_CELL_CELL_ADHESION	192	-0.265642174	-2.58965017	8.59E-08	1.22E-05	9.77E-06	1316	tags=60%, list=41%, signal=38%	PDGFRA/DLG4/FYN/CLSTN1/TMEM47/TNXB/PCDHB14/CLDN4/CD276/TNF/CBFB/CDH9/IL15/PTPN2/LRP6/THY1/SYK/CTSG/VEZT/AMIGO3/SELPLG/PODXL/CLDN5/PLXNB2/SOCS1/CYP1B1/BCL2/TNFRSF21/WNT1/PCDHB4/CLSTN3/FGL2/ZNF703/WNT3A/ETS1/CXCL13/HSPB1/FXYD5/ADIPOQ/CCL5/CD4/SERPINB8/BAD/CD6/PPARA/HLA-DMA/ILK/HLA-A/CD34/CITED2/PCDHB16/SPN/NFASC/BTN2A2/CDH4/MINK1/SMAD7/ALOX5/HLA-E/XBP1/RELA/CCL21/ITGAM/TRO/PYCARD/SERPINE2/CEBPB/MYADM/AIF1/BMP4/PKP2/HLA-DOB/LRG1/CLDN3/CDH11/HLA-DRB5/LYN/HLA-DMB/DAB1/AGER/CDH3/FLOT2/LAG3/CD177/HAS2/HLA-DQB1/IGF2/AKT1/SCGB1A1/CSK/AMIGO2/PTPRC/LRFN3/PERP/PPP3CA/HLA-DRB3/ITGA3/GLI2/ITGB1/F2RL3/MAGI1/MSN/ST3GAL4/IRAK1/HMGB1/CLIC1/HAVCR2/ITGA9/ITGB7/TYK2/

GOBP_POSITIVE_REGULATION_OF_LOCOMOTION	GOBP_POSITIVE_REGULATION_OF_LOCOMOTION	GOBP_POSITIVE_REGULATION_OF_LOCOMOTION	111	-0.329454013	-2.752525927	1.35E-07	1.86E-05	1.49E-05	1322	tags=66%, list=41%, signal=40%	PARVA/CLDN15/SHC1/PCDHGA8/EFNB1/ABL1 MAP2K3/MYOC/OXSR1/PDGFRA/CLDN4/CLASP2/TNF/THY1/CCR1/SLC26A5/STX3/PODXL/DUOX2/CYP1B1/BCL2/SNAI2/MALAT1/ZNF703/NRP2/IL1R1/ETS1/CXCL13/PLAU/HSPB1/MDM2/CCL5/INS/FAM107A/SPN/MAZ/C3AR1/XBP1/CCL21/VEGFB/GRB7/PYCARD/PTP4A1/MYADM/CPNE3/GPSM3/NRP1/AIF1/BMP4/TEK/CLDN3/DAB2/LYN/AGER/SMO/FZD4/NTF3/FGF1/RAB11A/HAS2/AKT1/RAC1/ARHGEF7/PTPRC/F10/PPP3CA/ITGA3/NFE2L2/PDGFRB/ITGB1/HMGB1/MMP14/RHOC/STAT3/FGFR1/PTN/TRIP6/ITGB1BP1/ABL1
GOBP_ORGANELLE_ASSEMBLY	GOBP_ORGANELLE_ASSEMBLY	GOBP_ORGANELLE_ASSEMBLY	207	0.242681873	2.62154441	1.56E-07	2.09E-05	1.68E-05	605	tags=35%, list=19%, signal=30%	TBPL1/KIF3A/SPAG5/CCNB2/DZIP1/SUGT1/SH3GLB1/RFX2/IFT74/CCDC96/RAB3IP/SPATA6/CSRP2/AKAP4/CLUAP1/CENPE/EIF5/ZMYND10/CDC14B/ZPBP2/PAFAH1B1/WDR62/ACTL7A/ACRBP/CDCA8/WDR54/DHX30/TBC1D3/DNAH17/IFT122/CEP63/AURKC/NF2/CENPH/PRKAA1/STX18/RAB8B/EDC3/TPR/DNAH8/SMC3/CEP250/CEP72/EIF2S1/CEP350/CCDC42/FOXJ1/KIF23/RFX4/CDK5RAP2/NIP7/KIAA0753/DNAI1/IQCG/IFT20/BBS4/MYOM2/IQCB1/CNOT7/ATG9A/PINK1/CHEK2/CAPN3/RBM14/KIF4A/TBC1D1/BOP1/VPS37A/KPNB1/ACTL8/MKKS/LRFN4
GOCC_CHROMOSOME	GOCC_CHROMOSOME	GOCC_CHROMOSOME	357	0.20082948	2.428977698	1.68E-07	2.19E-05	1.76E-05	808	tags=38%, list=25%, signal=32%	NR1H3/SRPK1/SPAG5/DONSON/KLF11/TCFL5/RFC4/PWP1/C1orf112/SUGT1/SMCHD1/CCDC86/RFX2/BRD1/STAG3/INTS7/MDC1/SETX/CSNK2A2/PPP1R7/CHAF1B/CENPE/CENPM/RAD51/FKBP6/AHCTF1/PAFAH1B1/PCNA/ELL3/CDCA8/SMC1B/STAT4/TRAIP/EBNA1BP2/SUV39H2/SAP30/RNF138/TOP1MT/PAWR/NDE1/DR1/

											AURKC/CENPH/PRKAA1/RECQL4/HSPA2/EXOSC8/ITGB3BP/TPR/PAXIP1/PRIM1/SMC3/BLM/RUVBL1/PPP1CC/TRIP13/SUZ12/SUPT3H/ZMYND11/RAD17/ACTR6/TFIP11/BRD7/POLA2/SMAD2/FOXJ1/HSF2/PINX1/RFX4/ZHX3/ZC3H8/NAP1L4/TAF6/AFF4/TIMELESS/NFYB/SPOCD1/DYNC1LI1/TUBG1/WRN/ZBTB32/TRIM28/GRHL1/BUB1B/CHD1L/TFDP1/CEBPG/TAF10/PINK1/TOP3A/RAD50/CHEK2/DNTTIP1/KIF4A/SPHK2/BOP1/POLE/SMC6/ARPC2/THOC5/TNP2/E2F1/EXOSC4/PRRX2/CBX1/ATR/NOL6/NHEJ1/KIFAP3/ZW10/PPHLN1/RFC1/GMEB1/MAD2L2/RMI1/CHD5/CREB3/ARID4B/KIF22/BRD9/DNTTIP2/AKAP8L/FANCC/BAZ1A/TAF9/SAP130/SETD1A/MCM5/NUSAP1/NUP107/SMARCC1/HMX1/POT1/VCP/TEX264/PARP1
GOBP_MICROTUBULE_CYTOSKELETON_ORGANIZATION	GOBP_MICROTUBULE_CYTOSKELETON_ORGANIZATION	GOBP_MICROTUBULE_CYTOSKELETON_ORGANIZATION	131	0.29256975	2.780378763	1.80E-07	2.29E-05	1.83E-05	680	tags=43%, list=21%, signal=35%	KIF3A/EFHC1/SPAG5/CCNB2/DZIP1/SUGT1/PARD6A/CLUAP1/SPATA7/CENPE/ZMYND10/CDC14B/PAFAH1B1/WDR62/CDCA8/RANBP1/KATNA1/DNAH17/KATNB1/CEP63/RAE1/NDE1/AURKC/CENPH/PRKAA1/TPR/DNAH8/SMC3/CEP250/CEP72/FKBP4/CEP350/CCDC42/FOXJ1/KIF23/CDK5RAP2/ATXN3/SSNA1/KIAA0753/DNAI1/DYNC1LI1/TUBG1/IQCG/BBS4/CHEK2/MAP6D1/RBM14/KIF4A/CNTN2/ESPL1/KPNB1/TPX2/TUBG2/SLK/ZW10/HOOK2
GOBP_CELL_CYCLE_CHECKPOINT_SIGNALING	GOBP_CELL_CYCLE_CHECKPOINT_SIGNALING	GOBP_CELL_CYCLE_CHECKPOINT_SIGNALING	45	0.461488239	3.165820794	2.22E-07	2.38E-05	1.91E-05	711	tags=62%, list=22%, signal=49%	DONSON/INTS7/MDC1/RAD51/CDC14B/CDCA8/DOT1L/CEP63/CRY1/TPR/BLM/TRIP13/RAD17/CDK5RAP2/MAD2L1BP/TIMELESS/FZR1/DYNC1LI1/BUB1B/RAD50/CHEK2/BARD1/THOC5/E2F1/ATR/ZW10/MAD2L2/RINT1
GOCC_CONDENSE	GOCC_CONDENSE	GOCC_CONDENSED_C	57	0.408549901	3.085593963	2.27E-07	2.38E-05	1.91E-05	786	tags=58%, list=25%,	SPAG5/C1orf112/SUGT1/STAG3/CEN

D_CHROMOSOME	D_CHROMOSOME	HROMOSOME								signal=44%	PE/CENPM/RAD51/FKBP6/AHCTF1/PAFAH1B1/SMC1B/NDE1/AURKC/CENPH/HSPA2/ITGB3BP/TPR/SMC3/BLM/PPP1CC/BRD7/PINX1/DYNC1LI1/TUBG1/BUB1B/RAD50/SMC6/NOL6/KIFAP3/ZW10/KIF22/NUP107/SMARCC1
GOBP_POSITIVE_REGULATION_OF_EPITHELIAL_CELL_PROLIFERATION	GOBP_POSITIVE_REGULATION_OF_EPITHELIAL_CELL_PROLIFERATION	GOBP_POSITIVE_REGULATION_OF_EPITHELIAL_CELL_PROLIFERATION	39	-0.496397086	-2.96903045	2.27E-07	2.38E-05	1.91E-05	963	tags=72%, list=30%, signal=51%	TGFA/ZNF703/WNT3A/NRP2/BAD/XBP1/VEGFB/MYC/YAP1/NRP1/BMP4/TEK/GHSR/LRG1/ANG/SMO/CDH3/FGF1/HAS2/IGF2/AKT1/IQGAP3/APLN/HMGB1/NKX2-5/STAT3/FGFR1/PTN
GOBP_POSITIVE_REGULATION_OF_CELL_CELL_ADHESION	GOBP_POSITIVE_REGULATION_OF_CELL_CELL_ADHESION	GOBP_POSITIVE_REGULATION_OF_CELL_CELL_ADHESION	74	-0.384605389	-2.860280738	2.02E-07	2.38E-05	1.91E-05	1291	tags=72%, list=40%, signal=44%	FYN/CD276/TNF/CBFB/IL15/THY1/SYK/CTSG/PODXL/SOCS1/WNT3A/ETS1/CXCL13/CCL5/CD4/BAD/CD6/HLA-DMA/HLA-A/CITED2/SPN/BTN2A2/SMAD7/ALOX5/HLA-E/XBP1/RELA/CCL21/PYCARD/AIF1/HLA-DOB/HLA-DRB5/LYN/HLA-DMB/AGER/FLOT2/HAS2/HLA-DQB1/IGF2/AKT1/CSK/PTPRC/PPP3CA/HLA-DRB3/GLI2/MAGI1/ST3GAL4/IRAK1/HMGB1/HAVCR2/TYK2/EFNB1/ABL1
GOBP_POSITIVE_REGULATION_OF_LEUKOCYTE_CELL_CELL_ADHESION	GOBP_POSITIVE_REGULATION_OF_LEUKOCYTE_CELL_CELL_ADHESION	GOBP_POSITIVE_REGULATION_OF_LEUKOCYTE_CELL_CELL_ADHESION	62	-0.409053781	-2.852196871	2.05E-07	2.38E-05	1.91E-05	910	tags=74%, list=28%, signal=54%	FYN/CD276/TNF/CBFB/IL15/THY1/SYK/SOCS1/ETS1/CCL5/CD4/BAD/CD6/HLA-DMA/HLA-A/SPN/BTN2A2/ALOX5/HLA-E/XBP1/RELA/CCL21/PYCARD/AIF1/HLA-DOB/HLA-DRB5/LYN/HLA-DMB/AGER/FLOT2/HAS2/HLA-DQB1/IGF2/AKT1/CSK/PTPRC/PPP3CA/HLA-DRB3/GLI2/ST3GAL4/IRAK1/HMGB1/HAVCR2/TYK2/EFNB1/ABL1
GOBP_LEUKOCYTE_CELL_CELL_ADHESION	GOBP_LEUKOCYTE_CELL_CELL_ADHESION	GOBP_LEUKOCYTE_CELL_CELL_ADHESION	91	-0.344016953	-2.736155975	2.27E-07	2.38E-05	1.91E-05	1257	tags=67%, list=39%, signal=42%	CD276/TNF/CBFB/IL15/PTPN2/THY1/SYK/CTSG/SELPLG/SOCS1/TNFRSF21/FGL2/ETS1/CCL5/CD4/BAD/CD6/PPARA/HLA-DMA/HLA-A/SPN/BTN2A2/SMAD7/ALOX5/HLA-E/XBP1/RELA/CCL21/PYCARD/CEBPB/AIF1/BMP4/HLA-DOB/LRG1/HLA-DRB5/LYN/HLA-DMB/AGER/FLOT2/LAG3/

GOBP_CELL_ACTIVATION	GOBP_CELL_ACTIVATION	GOBP_CELL_ACTIVATION	205	-0.258802391	-2.558774249	2.23E-07	2.38E-05	1.91E-05	1038	tags=50%, list=32%, signal=36%	CD177/HAS2/HLA-DQB1/IGF2/AKT1/SCGB1A1/CSK/PTPRC/PPP3CA/HLA-DRB3/GLI2/ITGB1/MSN/ST3GAL4/IRAK1/HMGB1/HAVCR2/ITGB7/TYK2/EFNB1/ABL1 BCR/GCLC/NDRG1/FKBP1A/SOCS1/VWF/BCL2/TNFRSF21/WNT1/FGL2/TSC1/WNT3A/HSPB1/MDM2/CPLX2/CCL5/LIPA/EMP2/CD4/BAD/CD6/HLA-DMA/ILK/ITGB8/INS/CD79A/HLA-A/MAFB/PRDX1/SPN/MEN1/HLA-F/FOSL2/HSH2D/BTN2A2/SMAD7/HLA-E/XBP1/CCL21/ITGAM/PYCARD/SERPINE2/CEBPB/PLSCR1/TNFRSF4/AIF1/BMP4/EOMES/HLA-DOB/SLURP1/BAX/ENTPD2/HLA-DRB5/LYN/HLA-DMB/LFNG/AIRE/AGER/SMO/FLOT2/TYROBP/LAG3/CEBPA/CD177/COL3A1/HLA-DQB1/IGF2/CTSC/AKT1/SCGB1A1/SLC11A1/CSK/PTPRC/LTBR/IL17A/ADRA2B/FCER1G/PPP3CA/HLA-DRB3/GLI2/SUPT6H/ITGB1/F2RL3/MSN/IMPDH2/HMGB1/PHB2/ZBTB7A/MMP14/CLIC1/HAVCR2/IFNGR1/CHRNB2/TYK2/CRTC3/SLC25A5/PURA/STAT3/EIF2AK4/EFNB1/ABL1/KIF13B
GOBP_NERVOUS_SYSTEM_PROCESS	GOBP_NERVOUS_SYSTEM_PROCESS	GOBP_NERVOUS_SYSTEM_PROCESS	255	-0.240321986	-2.532694445	2.24E-07	2.38E-05	1.91E-05	1648	tags=69%, list=51%, signal=36%	BRSK1/NEUROG1/AAAS/ROCK2/OR2A12/CHMP2B/TRPV1/PDCL/MAPK8IP2/RP1L1/KCNMB3/TAS2R10/KCNQ3/SIX3/CX3CR1/ARR3/S100B/GABRR1/NPFF/OR2J2/ITGA5/OR4P4/GJC1/OR4M1/CAV3/CHRNA7/OR5D18/SLC17A8/TAS2R60/OR13C8/OR1D2/ASCL1/TPPP/OR10A4/OR1F1/OR5AS1/OR11L1/PJA2/UCN/OR52N2/OR52B6/OR51L1/DLG4/RRH/FYN/CYFIP1/DIAPH1/MRGPRX2/TAS2R41/OR4D1/EML2/TNF/ATP1A2/CLN8/OR4K14/ATP2A1/NPTX2/OR6B2/SLC26A5/ATAD1/OR2B11/ZNF488/PRR4/BCHE/LUM/TAS2R40/ATXN1/REEP2/CHRNA2

											/OR8A1/NF1/CLDN5/RDH5/ARRB2/OR4S1/FXN/BCR/TAAR5/LCTL/TRIOBP/ATP8B1/OR8S1/TNFRSF21/OR7D2/OR51S1/IMPG2/OR8D1/SNAI2/TSC1/TSHZ3/CRYGA/NFATC4/OR10J5/OAT/TTC8/B3GNT2/OR10T2/INS/HIPK2/OR6M1/FAM107A/RGS14/OR1D4/OR52M1/BTG2/OR8G1/TMEM25/OR6K2/PBX3/TGFBI/OR6Y1/TAS2R9/SERPINE2/SEZ6/GSTO1/OR7G2/OR1E2/GNB1/OR4K17/OR8B8/OR9K2/GHSR/OR13C4/WFS1/ITPR1/SLURP1/NAV2/HRH3/CNTNAP1/CLIC5/NOB1/AGER/FZD4/CDH3/OR6V1/GRM1/NTF3/OR6C4/OR10H4/OR52D1/OTOR/TAS2R39/P2RX2/CTSC/OR2T4/OR51A7/GRIK5/AKT1/GRK1/OR6X1/CLN3/OR4D5/PPP3CA/SLC29A1/OR8H2/ITGA3/CBR3/OR3A3/ALDH1A3/HPS1/ARF4/ITGB1/CDK5/SCARB2/OR5F1/ST3GAL4/OR5V1/LAMC3/OR52E4/CHRNB2/ZMPSTE24/EIF2AK4/PTN/PTGES/ABL1/VDAC1
GOBP_LOCOMOTION	GOBP_LOCOMOTION	GOBP_LOCOMOTION	236	-0.24091892	-2.467525043	2.48E-07	2.55E-05	2.04E-05	1325	tags=58%, list=41%, signal=36%	SH3BP1/MAP2K3/MYOC/OXSR1/PDGFRA/ENG/ARHGAP18/TNXB/CLDN4/CLASP2/TNF/ATP1A2/SPATA13/PTPN2/THY1/SYK/CCL22/CCR1/CTSG/SULF1/SLC26A5/APOD/STX3/WNT11/PODXL/NF1/CLDN5/ARRB2/FFAR2/PLXNB2/BCR/ELMO2/DUOX2/CYP1B1/BCL2/SNAI2/MALAT1/ZNF703/WNT3A/NRP2/IL1R1/ETS1/CXCL13/PLAU/HSPB1/ABHD6/MDM2/CXCR3/ADIPOQ/CCL5/EMP2/CCR5/OR10J5/INS/FAM107A/CITED2/PLEKHG3/SPN/MAZ/ABI3/C3AR1/TIMP1/MINK1/SMAD7/ALOX5/XBP1/CCL21/VEGFB/GRB7/RHOG/FOLR2/PYCARD/SERPINE2/PTP4A1/MYADM/CPNE3/GPSM3/S100A4/NRP1/AIF1/BMP4/TEK/GHSR/SLURP1/CLDN3/DAB2/CDH11/LYN/AIRE/IFITM1/AGER/S

GOBP_POSITIVE_REGULATION_OF_SIGNALING	GOBP_POSITIVE_REGULATION_OF_SIGNALING	GOBP_POSITIVE_REGULATION_OF_SIGNALING	340	-0.212427417	-2.362889512	3.05E-07	3.07E-05	2.46E-05	1382	tags=56%, list=43%, signal=36%	MO/FZD4/PLXNA1/NTF3/FGF1/XCR1/RAB11A/TRPM2/PODN/CHRD/COL3A1/HAS2/AKT1/ARHGDIB/RALA/CLN3/LSP1/RAC1/ARHGEF7/PTPRC/GNA12/F10/FCER1G/PPP3CA/ITGA3/NFE2L2/PDGFRB/ITGB1/CDK5/ADAM15/MSN/ST3GAL4/HMGB1/MMP14/ADIPOR1/RHOC/ITGA9/PARVA/STAT3/FGFR1/PTN/GADD45A/TRIP6/ITGB1BP1/ABL1 EXTL3/AUTS2/LIMS2/JRK/CCL8/TM2D3/LTB/SRI/PJA2/UCN/MAP2K3/MYOC/PDGFRA/DLG4/ENG/GATA4/AVPI1/APAF1/FYN/CYFIP1/CLSTN1/TNXB/LTF/PTP4A3/TNF/CYLD/GHRH/PTPN2/SYK/CCL22/CANT1/GPRC5B/CCR1/FAM53B/TRIM16/SULF1/RPS15/NMI/MOS/IGFBP6/STX3/WNT11/FSHR/SQSTM1/AAK1/CSHL1/NF1/INHBA/AGR2/ARRB2/TM7SF3/FFAR2/MAP3K5/CC2D1A/FKBP1A/SRPX/CYP1B1/VWF/HAND2/TGFA/WNT1/RPH3AL/CLSTN3/WNT3A/OCIAD1/IL1R1/TSHZ3/NFATC4/ADIPOQ/CCL5/LIPA/EMP2/CD4/BAD/PRKCH/ILK/INS/HIPK2/RXRB/PSMD9/FAM107A/CITED2/RGS14/MEN1/MAZ/TTC23/KMO/MINK1/KLF2/FRS2/NET1/XBP1/RELA/CCL21/IGFBP4/ITM2C/PYCARD/SERPINE2/MYC/UCN3/RWDD3/YAP1/S100A4/MIER1/PUM1/NRP1/HCLS1/BMP4/H19/TEK/GHSR/EDA/ITPR1/IQGAP1/DOK4/CRHR2/BAX/LRG1/SYT12/DAB2/LYN/LFNG/TRIM41/AGER/SMO/TNFSF10/SLC35B2/CDH3/BBC3/GRM1/TYROBP/NTF3/FGF1/LY86/RNF185/OSBP/COL3A1/P2RX2/PLEKHA4/IGF2/CTSC/NEK6/AKT1/RALA/UBE2B/RAC1/PTPRC/F10/LTBR/VAPA/ADRA2B/IQGAP3/PPP3CA/PPP2R1A/CASP4/PDGFRB/MID1/ITGB1/EIF2AK2/LMCD1/F2RL3/FBX

												W11/IRAK1/HMGB1/ARHGEF3/NFKB1/PHB2/HAVCR2/ADIPOR1/RHOC/CTSK/OCIAD2/TYK2/TRIM8/TSPAN5/PPP2R5B/STAT3/MAGED1/FGFR1/EIF2AK4/PTN/GADD45A/NADK/TRIP6/SHC1/ING4/ITGB1BP1/ABL1/LSM14A/MBD2
GOBP_LYMPHOCYTE_ACTIVATION	GOBP_LYMPHOCYTE_ACTIVATION	GOBP_LYMPHOCYTE_ACTIVATION	146	-0.290718569	-2.647436426	3.12E-07	3.08E-05	2.46E-05	1011	tags=52%, list=32%, signal=37%	FKBP1A/SOCS1/BCL2/TNFRSF21/WNT1/FGL2/TSC1/WNT3A/CCL5/LIPA/EMP2/CD4/BAD/CD6/HLA-DMA/INS/CD79A/HLA-A/MAFB/PRDX1/SPN/MEN1/HLA-F/FOSL2/HSH2D/BTN2A2/SMAD7/HLA-E/XBP1/CCL21/PYCARD/CEBPB/TNFRSF4/AIF1/BMP4/EOMES/HLA-DOB/BAX/HLA-DRB5/LYN/HLA-DMB/LFNG/AIRE/AGER/FLOT2/TYROBP/LAG3/HLA-DQB1/IGF2/AKT1/SCGB1A1/SLC11A1/CSK/PTPRC/FCER1G/PPP3CA/HLA-DRB3/GLI2/SUPT6H/ITGB1/MSN/IMPDH2/HMGB1/PHB2/ZBTB7A/MMP14/HAVCR2/CHRNB2/TYK2/SLC25A5/PURA/STAT3/EIF2AK4/EFNB1/ABL1/KIF13B
GOCC_ACROSOMAL_VESICLE	GOCC_ACROSOMAL_VESICLE	GOCC_ACROSOMAL_VESICLE	31	0.522024915	3.037649002	3.69E-07	3.56E-05	2.85E-05	611	tags=65%, list=19%, signal=53%	ATP6V1E2/NUDT1/IFT74/SPAG8/SPACA3/CSNK2A2/TSSK2/IQCF1/ZPBP2/ACTL7A/ACRBP/TCP11/SH3GL3/MORN3/TRIP11/LYZL6/POMT1/IFT20/DPEP3/ATP6V0A2
HP_DECREASED_FERTILITY_IN_MALES	HP_DECREASED_FERTILITY_IN_MALES	HP_DECREASED_FERTILITY_IN_MALES	28	0.551740039	3.096100225	3.84E-07	3.64E-05	2.91E-05	469	tags=61%, list=15%, signal=52%	TTC21A/DNALI1/DZIP1/IFT74/STAG3/CATSPER2/RAD51/FKBP6/ZMYND10/KLHL10/DNAH17/UBE2T/AURKC/DNAH8/BLM/FOXJ1/DNAI1
GOCC_VACUOLE	GOCC_VACUOLE	GOCC_VACUOLE	179	-0.270443299	-2.574292389	4.04E-07	3.76E-05	3.01E-05	928	tags=48%, list=29%, signal=36%	ORM2/TSC1/OCIAD1/ABHD6/UNC93B1/LIPA/TRIP10/VPS11/OGN/HLA-DMA/SDC2/SIDT1/TPCN1/CHMP6/ABCD4/MANBA/CD34/CTBS/MAN2B2/M6PR/DNAJC13/HLA-F/C3AR1/ATG16L2/TMEM25/ABCC10/CYBRD1/ITM2C/PYCARD/PPT2/WIPI1/CPNE3/GNB1/GNAI1/VPS4A/GLB1/HLA-DOB/DAB2/HLA-DRB5/LYN/HLA-DMB/T

											FEB/SLC29A3/GALC/IFITM1/BBC3/ANXA6/SLC2A13/TRPM2/GRAMD1A/HAS2/HLA-DQB1/CTSC/FYCO1/PTGES2/SLC11A1/CLN3/CHMP7/TM9SF1/DNAJC5/VAPA/TFE3/HLA-DRB3/PDGFRB/AP2M1/HPS1/UBQLN2/SCARB2/ABCD1/ZFYVE1/MEFV/CTSK/OCIAD2/CAP1/PRELP/WDR81/IFITM3/PYGB/BLOC1S1/GDI2/HPS6/TRAPPC1/TXNDC5/FUCA2/CCDC115/SGSH
GOBP_SENSORY_PERCEPTION_OF_SMELL	GOBP_SENSORY_PERCEPTION_OF_SMELL	GOBP_SENSORY_PERCEPTION_OF_SMELL	64	-0.400016869	-2.828830604	5.62E-07	5.12E-05	4.10E-05	1508	tags=83%, list=47%, signal=45%	OR2J2/OR4P4/OR4M1/OR5D18/OR13C8/OR1D2/OR10A4/OR1F1/OR5AS1/OR11L1/OR52N2/OR52B6/OR51L1/OR4D1/OR4K14/OR6B2/OR2B11/OR8A1/OR4S1/OR8S1/OR7D2/OR51S1/OR8D1/OR10J5/TTC8/B3GNT2/OR10T2/OR6M1/OR1D4/OR52M1/OR8G1/OR6K2/OR6Y1/OR7G2/OR1E2/OR4K17/OR8B8/OR9K2/OR13C4/NAV2/OR6V1/OR6C4/OR10H4/OR52D1/OR2T4/OR51A7/OR6X1/OR4D5/OR8H2/OR3A3/OR5F1/OR5V1/OR52E4
GOCC_EXTERNAL_ENCAPSULATING_STRUCTURE	GOCC_EXTERNAL_ENCAPSULATING_STRUCTURE	GOCC_EXTERNAL_ENCAPSULATING_STRUCTURE	104	-0.317840179	-2.59871199	5.74E-07	5.13E-05	4.11E-05	1374	tags=67%, list=43%, signal=40%	WNT2B/MMRN2/ANXA4/MYOC/BCAM/TNXB/COL4A1/ANXA7/CTSG/SULF1/WNT11/LUM/ANGPTL4/COL4A6/ADAMTSL2/SRPX/VWF/ANGPTL2/ABI3BP/C1QC/IMPG2/FGL2/ORM2/ADIPOQ/ADAMTS5/SERPINB8/OGN/LRRN3/SDC2/COL15A1/SPN/MMP15/ELN/LOXL2/TIMP1/COL4A5/EMILIN3/VWA1/TGFBI/SERPINE2/MMP17/PLSCR1/S100A4/COL4A2/SERPINA1/COL6A3/NAV2/HTRA1/CPA3/PCOLCE/ENTPD2/ANG/ANXA6/FGF1/PODN/COL3A1/CTSC/PI3/MGP/COL27A1/FBN3/CD248/MUC2/LAMC3/MMP23B/MMP14/PRELP/FBN1/ITIH5/LGALS3BP
GOCC_NUCLEAR_CHROMOSOME	GOCC_NUCLEAR_CHROMOSOME	GOCC_NUCLEAR_CHROMOSOME	43	0.461795691	3.116999896	6.05E-07	5.31E-05	4.25E-05	891	tags=67%, list=28%, signal=49%	SMCHD1/STAG3/SETX/RAD51/FKBP6/PCNA/SMC1B/SAP30/HSPA2/PRIM1/SMC3/BLM/RUVBL1/ACTR6/PO

											LA2/PINX1/TIMELESS/TUBG1/RAD50/POLE/NOL6/KIFAP3/ARID4B/BAZ1A/SAP130/MCM5/PARP1/BRMS1/MCM3
GOBP_MONOATOMIC_ION_TRANSPORT	GOBP_MONOATOMIC_ION_TRANSPORT	GOBP_MONOATOMIC_ION_TRANSPORT	245	-0.233716232	-2.427473111	6.21E-07	5.36E-05	4.29E-05	1950	tags=81%, list=61%, signal=34%	SLC34A1/SLC4A5/SLC6A8/SLC12A1/BDKRB1/CACNA1C/GRM6/FGF13/GP1BB/ABCB1/GRIN1/CASK/SLC13A2/SNTA1/GAL/WWP1/KCNJ9/KCNG1/SLC4A1/CA2/AQP1/HCN4/HTR3A/CDH23/DNM2/SLC6A1/RASA3/SLC5A7/KCNMB1/COMMD9/SLC5A11/RAMP2/SLC25A22/KCNH7/ATG5/UMOD/TRPV1/KCNMB3/CLCA4/SLC12A3/KCNQ3/SLC22A2/CDK2/TMEM163/ATP1B2/TOMM40/GABRR1/TRPV5/OSTM1/KCNS3/SLC41A3/GJC1/CAV3/HCN3/CHRNA7/SLC17A8/SLC34A3/KLHL3/NIPA2/KCNJ6/MCHR1/SLC25A37/CCL8/GRIA4/SLC17A7/SRI/UCN/KCNAB3/OXSR1/FYN/DIAPH1/CLDN4/LTF/ATP1A2/TMEM63A/ATP6V1G2/STOM/THY1/CLCN6/KCNE3/CCR1/ATP2A1/SLC26A5/PLLP/CHRNA2/CACNA1B/ACTN2/CACNA1E/ATP2B3/SLC10A5/FXN/ATP7B/KCNS2/ATP8B1/FKBP1A/ATP6V0A1/ATP1B1/BCL2/SLC12A8/SLC22A11/CACNB3/TSC1/WNT3A/FXYD5/ABCB7/KCNAB2/HTR1B/FHL1/CCL5/CLCN2/IBTK/CD4/CCR5/SLC22A8/STIM1/SLC22A7/TPCN1/TMCO3/CNNM2/TMEM109/CSN2/SCN2B/ATP13A1/OTOP1/CCL21/SLC9A8/KCNJ1/ABCC10/CYBRD1/SERPINE2/GSTO1/SLC25A12/SLC32A1/WFS1/ITPR1/PKP2/KCNH4/BAX/HRH3/CALM3/CLCC1/LYN/CLIC5/SCARA5/SLC12A9/OTOP2/LIME1/COX7B/SLC25A23/GJA4/SLC6A17/KCNB2/ANXA6/SLC16A1/XCR1/MAOB/COX15/TRPM2/P2RX2/ATF4/GRIK5/KCNK5/AKT1/PPIF/SLC11A1/SLC34A2/CLNS1A/CABP5/PTPRC/SLC2

											6A1/ATP1A3/SFXN3/OTOP3/PPP3CA/SLC31A1/CCDC51/PDGFRB/SLC30A9/KCNB1/ITGB1/CDK5/F2RL3/LASP1/SEC61A1/PHB2/CLIC1/STEAP3/CHRNB2/COX7A1/NKX2-5/SLC25A5/ZMPSTE24/ATP2A3/TSPO/CLDN15/COX8A/ABL1/VDAC1
GOBP_T_CELL_ACTIVATION	GOBP_T_CELL_ACTIVATION	GOBP_T_CELL_ACTIVATION	110	-0.315892183	-2.638290551	6.65E-07	5.64E-05	4.51E-05	1011	tags=55%, list=32%, signal=39%	FKBP1A/SOCS1/BCL2/TNFRSF21/WNT1/FGL2/TSC1/CCL5/LIPA/CD4/BAD/CD6/HLA-DMA/INS/HLA-A/MAFB/SPN/MEN1/FOSL2/HSH2D/BTN2A2/SMAD7/HLA-E/XBP1/CCL21/PYCARD/CEBPB/TNFRSF4/AIF1/BMP4/EOMES/HLA-DOB/BAX/HLA-DRB5/LYN/HLA-DMB/LFNG/AIRE/AGER/FLOT2/LAG3/HLA-DQB1/IGF2/AKT1/SCGB1A1/SLC11A1/CSK/PTPRC/FCER1G/PPP3CA/HLA-DRB3/GLI2/MSN/HMGB1/HAVCR2/TYK2/STAT3/EIF2AK4/EFNB1/ABL1/KIF13B
GOBP_EPITHELIAL_CELL_PROLIFERATION	GOBP_EPITHELIAL_CELL_PROLIFERATION	GOBP_EPITHELIAL_CELL_PROLIFERATION	87	-0.354231798	-2.756182798	6.94E-07	5.78E-05	4.63E-05	963	tags=56%, list=30%, signal=40%	TGFA/SNAI2/ZNF703/WNT3A/NRP2/LIPA/BAD/CD34/LOXL2/ALOX5/FRS2/XBP1/VEGFB/IGFBP4/CEBPB/MYC/YAP1/NRP1/BMP4/TEK/GHSR/SLURP1/BAX/LRG1/DAB2/ANG/SMO/CDH3/FGF1/HAS2/IGF2/BCL2L1/AKT1/IL17A/IQGAP3/CDK4/APLN/HMGB1/PHB2/MMP14/NKX2-5/KLF9/PURA/STAT3/MAGED1/FGFR1/PTN/BCL2L2/ITGB1BP1
GOCC_COLLAGEN_CONTAINING_EXTRACELLULAR_MATRIX	GOCC_COLLAGEN_CONTAINING_EXTRACELLULAR_MATRIX	GOCC_COLLAGEN_CONTAINING_EXTRACELLULAR_MATRIX	84	-0.35216878	-2.717920501	7.09E-07	5.80E-05	4.65E-05	1374	tags=71%, list=43%, signal=42%	WNT2B/MMRN2/ANXA4/MYOC/BCAM/TNXB/COL4A1/ANXA7/CTSG/SULF1/LUM/ANGPTL4/COL4A6/SRPX/VWF/ANGPTL2/ABI3BP/C1QC/IMPG2/FGL2/ORM2/ADIPOQ/ADAMTS5/SERPINB8/OGN/SDC2/COL15A1/SPN/ELN/LOXL2/TIMP1/COL4A5/EMILIN3/VWA1/TGFBI/SERPINE2/PLSCR1/S100A4/COL4A2/SERPINA1/COL6A3/NAV2/HTRA1/CPA3/PCOLCE/ENTPD2/ANG/ANXA6/PODN/COL3A1/CTSC/MGP/COL27A1/MUC2/LAMC

											3/MMP23B/PRELP/FBN1/ITIH5/LGALS3BP
GOBP_CELLULAR_PROCESS_INVOLVED_IN_REPRODUCTION_IN_MULTICELLULAR_ORGANISM	GOBP_CELLULAR_PROCESS_INVOLVED_IN_REPRODUCTION_IN_MULTICELLULAR_ORGANISM	GOBP_CELLULAR_PROCESS_INVOLVED_IN_REPRODUCTION_IN_MULTICELLULAR_ORGANISM	83	0.334470347	2.825027821	7.69E-07	6.19E-05	4.95E-05	618	tags=43%, list=19%, signal=36%	TDRD7/SRPK1/DDX20/TBPL1/TTC21A/CCNB2/DZIP1/RFX2/ROPN1L/STRBP/AKAP4/SPACA3/CATSPER2/TSSK2/IQCF1/ZPBP2/PAFAH1B1/ACTL7A/MAST2/MYBL1/ACRBP/KLHL10/TCP11/HSPA2/PIWIL2/TRIP13/CCDC42/TDRKH/LYZL6/AFF4/IQCG/TIAL1/BBS4/INHBB/MKKS/TNP2
GOBP_GERM_CELL_DEVELOPMENT	GOBP_GERM_CELL_DEVELOPMENT	GOBP_GERM_CELL_DEVELOPMENT	66	0.368709741	2.912922122	7.97E-07	6.31E-05	5.05E-05	658	tags=48%, list=21%, signal=39%	TDRD7/SRPK1/DDX20/TBPL1/TTC21A/DZIP1/RFX2/ROPN1L/STRBP/AKAP4/CATSPER2/TSSK2/IQCF1/ZPBP2/PAFAH1B1/ACTL7A/ACRBP/KLHL10/TCP11/HSPA2/PIWIL2/TRIP13/CCDC42/TDRKH/AFF4/IQCG/TIAL1/BBS4/INHBB/MKKS/TNP2/PAQR7
GOCC_CHROMOSOME_TELOMERIC_REGION	GOCC_CHROMOSOME_TELOMERIC_REGION	GOCC_CHROMOSOME_TELOMERIC_REGION	32	0.495634901	2.924438669	1.17E-06	8.82E-05	7.06E-05	915	tags=75%, list=29%, signal=54%	SMCHD1/SETX/RAD51/PCNA/RECQL4/BLM/PPP1CC/RAD17/TFIP11/PINX1/WRN/RAD50/CHEK2/SMC6/THOC5/CBX1/ATR/MCM5/POT1/PARP1/HAT1/TP53BP1/MCM3/RIF1
GOBP_DETECTION_OF_STIMULUS	GOBP_DETECTION_OF_STIMULUS	GOBP_DETECTION_OF_STIMULUS	103	-0.314269209	-2.57003882	1.17E-06	8.82E-05	7.06E-05	1627	tags=77%, list=51%, signal=39%	OR2A12/TRPV1/KCNMB3/CDS1/ATF2/TAS2R10/OR2J2/OR4P4/OR4M1/CAV3/OR5D18/TAS2R60/OR13C8/OR1D2/OR10A4/OR1F1/EXTL3/OR5AS1/OR11L1/OR52N2/OR52B6/OR51L1/RRH/FYN/TAS2R41/OR4D1/TNF/OR4K14/OR6B2/OR2B11/TAS2R40/OR8A1/OR4S1/SRPX/OR8S1/OR7D2/OR51S1/OR8D1/OR10J5/STIM1/OR10T2/HLA-A/OR6M1/OR1D4/OR52M1/OR8G1/OTOP1/HLA-B/OR6K2/OR6Y1/TAS2R9/SERPINE2/OR7G2/YAP1/OR1E2/OR4K17/OR8B8/OR9K2/OR13C4/CALM3/FECH/SMO/OR6V1/OR6C4/OR10H4/OR52D1/TAS2R39/P2RX2/OR2T4/OR51A7/GRK1/OR6X1/OR4D5/OR8H2/OR3A3/OR5F1/OR5V1/OR52E4/TSPO
GOBP_POSITIVE_REGULATION_OF_M	GOBP_POSITIVE_REGULATION_OF_M	GOBP_POSITIVE_REGULATION_OF_MULTIC	319	-0.211215116	-2.331280764	1.13E-06	8.82E-05	7.06E-05	1375	tags=56%, list=43%, signal=36%	DHX37/WNT2B/ZFPM2/KLHL25/MMRN2/LTB/POU2AF1/UCN/ENG/GAT

ULTICELLULAR_ORGANISMAL_PROCESS	ULTICELLULAR_ORGANISMAL_PROCESS	ELLULAR_ORGANISMAL_PROCESS									A4/FYN/CYFIP1/CLSTN1/TNXB/LTF/CD276/TNF/CBFB/ATP1A2/GHRH/EHMT1/IL15/THY1/SYK/GPRC5B/CCR1/ATP2A1/TRIM16/SULF1/AMIGO3/ZNF488/SGIP1/WNT11/LUM/ANGPTL4/INHBA/GPR21/ARRB2/MYOG/FFAR2/PLXNB2/NR1H2/SOCS1/CYP1B1/TRAK1/HAND2/BCL2/GDI1/WNT1/CLSTN3/ZNF703/ORM2/WNT3A/IL1R1/ETS1/PLAU/HSPB1/UNC93B1/NFATC4/ADIPOQ/CCL5/CLCN2/EMP2/CD4/BAD/STIM1/CD6/PRKCH/LRRN3/HLA-DMA/ITGB8/INS/HLA-A/HIPK2/RXRB/CD34/CITED2/RGS14/SPN/HLA-F/C3AR1/LOXL2/KIR2DL4/BTN2A2/CDH4/SMAD7/ALOX5/FRS2/HLA-E/XBP1/RELA/CCL21/VEGFB/TMEM119/OAS2/ITGAM/FBXW8/FAM20C/PYCARD/SERPINE2/CEBPB/GSTO1/MYC/YAP1/CUL7/GPSM3/TNFRSF4/NRP1/AIF1/HCLS1/BMP4/H19/TEK/GHSR/HLA-DOB/ISG15/LRG1/DAB2/HLA-DRB5/LYN/HLA-DMB/AIRE/AGER/SMO/ADIPOR2/FZD4/RAB1A/PLXNA1/FLOT2/TYROBP/ADAM12/FGF1/CEBPA/CD177/PRKAB2/HLA-DQB1/PQBP1/IGF2/CTSC/PLAG1/ATF4/AKT1/SLC11A1/CSK/AMIGO2/PTPRC/IL17A/ADRA2B/FCER1G/IQGAP3/CTTN/PPP3CA/HLA-DRB3/PRAP1/GLI2/NFE2L2/ITGB1/EIF2AK2/EBF2/NAP1L1/ST3GAL4/APLN/IRAK1/HMGB1/MMP14/HAVCR2/MEFV/ADIPOR1/IFNGR1/CHRNB2/NKX2-5/TYK2/HEG1/STAT3/MAGED1/PTN/TSPO/EFNB1/ABL1
GOCC_SPINDLE	GOCC_SPINDLE	GOCC_SPINDLE	107	0.297144646	2.710403844	1.19E-06	8.85E-05	7.09E-05	789	tags=48%, list=25%, signal=37%	KIF3A/EFHC1/SPAG5/TBCCD1/C1orf112/SPAG8/HMMR/CENPE/CDC14B/PAFAH1B1/WDR62/CDCA8/POLB/KATNA1/KATNB1/CEP63/RAE1/NDE1/DR1/AURKC/HSPA2/TPR/MAPKBP1/SMC3/CEP350/PINX1/KIF23/CDK5R

											AP2/CCDC117/MAD2L1BP/DYNC1LI1/TUBG1/BUB1B/IQCB1/CDC7/KIF4A/ESPL1/SMC6/TPX2/TUBG2/CBX1/RASSF1/KIFAP3/ZW10/KBTBD8/MAD2L2/KIF22/RAB11FIP4/NUSAP1/TUBB/DCUN1D5
GOBP_POSITIVE_REGULATION_OF_RESPONSE_TO_EXTERNAL_STIMULUS	GOBP_POSITIVE_REGULATION_OF_RESPONSE_TO_EXTERNAL_STIMULUS	GOBP_POSITIVE_REGULATION_OF_RESPONSE_TO_EXTERNAL_STIMULUS	103	-0.31304004	-2.559986889	1.37E-06	0.000100583	8.05E-05	1326	tags=66%, list=41%, signal=40%	PJA2/OXSR1/PDGFRA/FYN/LTF/TNF/CYLD/IL15/MGST2/SYK/GPRC5B/CCR1/NMI/STX3/ARRB2/FFAR2/ETS1/CXCL13/PLAU/HSPB1/UNC93B1/CCL5/COLEC11/HLA-F/C3AR1/KIR2DL4/HLA-E/RELA/CCL21/VEGFB/PYCARD/CEBPB/PLSCR1/GPSM3/PUM1/NRP1/AIF1/GHSR/SIRT2/LYN/TRIM41/AGER/ZNFX1/FLOT2/TYROBP/NTF3/LAG3/LY86/CEBPA/RNF185/PQBP1/CTSC/RAC1/IL17A/CASP4/PDGFRB/EIF2AK2/ALOX5AP/IRAK1/HMGB1/PHB2/MAPKAPK3/HAVCR2/MEFV/NFKBIL1/FGFR1/PTN/LSM14A
GOBP_REGULATION_OF_CELL_CELL_ADHESION	GOBP_REGULATION_OF_CELL_CELL_ADHESION	GOBP_REGULATION_OF_CELL_CELL_ADHESION	106	-0.311864057	-2.55637399	1.65E-06	0.000118622	9.50E-05	1257	tags=64%, list=39%, signal=40%	CD276/TNF/CBFB/IL15/PTPN2/THY1/SYK/CTSG/PODXL/SOCS1/TNFRSF21/WNT1/FGL2/ZNF703/WNT3A/ETS1/CXCL13/FXYD5/ADIPOQ/CCL5/CD4/BAD/CD6/PPARA/HLA-DMA/HLA-A/CITED2/SPN/BTN2A2/MINK1/SMAD7/ALOX5/HLA-E/XBP1/RELA/CCL21/PYCARD/SERPINE2/CEBPB/MYADM/AIF1/BMP4/HLA-DOB/HLA-DRB5/LYN/HLA-DMB/AGER/FLOT2/LAG3/HAS2/HLA-DQB1/IGF2/AKT1/SCGB1A1/CSK/PTPRC/LRFN3/PPP3CA/HLA-DRB3/GLI2/MAGI1/ST3GAL4/IRAK1/HMGB1/HAVCR2/TYK2/EFNB1/ABL1
GOBP_CILIUM_MOVEMENT	GOBP_CILIUM_MOVEMENT	GOBP_CILIUM_MOVEMENT	22	0.578578447	2.932698142	1.84E-06	0.000129051	0.000103331	594	tags=68%, list=19%, signal=56%	TTC21A/LZTFL1/DZIP1/SPA17/ROPN1L/AKAP4/CATSPER2/IQCF1/ZMYND10/DNAH17/DNAH8/DNAI1/IQCG/BBS4/MKKS
GOCC_CENTROSOME	GOCC_CENTROSOME	GOCC_CENTROSOME	132	0.279401384	2.65957946	1.82E-06	0.000129051	0.000103331	634	tags=39%, list=20%, signal=33%	KIF3A/EFHC1/SPAG5/CCNB2/TBCCD1/DZIP1/DYNLRB2/CCDC112/IFT74/DYNLL2/HMMR/PARD6A/CLUAP

											1/ZMYND10/CDC14B/PAFAH1B1/PCNA/WDR62/RANBP1/KATNA1/CCDC77/KATNB1/CEP63/NDE1/AURKC/UBN1/CEP250/RRAGD/CEP72/CEP350/KIF23/CDK5RAP2/MAPKAPK2/SSNA1/KIAA0753/DNAI1/DYNC1LI1/TUBG1/WRN/KIFC3/IFT20/NME7/BBS4/IQCB1/VPS37A/SLC1A4/ESPL1/RASSF7/MKKS/NUP93/TUBG2/E2F1
GOBP_DEFENSE_RESPONSE	GOBP_DEFENSE_RESPONSE	GOBP_DEFENSE_RESPONSE	310	-0.207861836	-2.269764428	1.94E-06	0.000134184	0.00010744	1076	tags=46%, list=34%, signal=34%	INHBA/RBMS3/AGR2/TRIM4/ARRB2/FFAR2/HLA-C/BCR/NR1H2/MAP3K5/DUOX2/ATP1B1/COTL1/TRAFD1/BCL2/NQO1/C1QC/FBXO9/FGL2/ORM2/IL1R1/ETS1/CXCL13/UNC93B1/CUEDC2/CXCR3/NFATC4/ADIPOQ/CCL5/ADAMTS5/KIR2DS3/LIPA/CD4/CCR5/MPEG1/CD6/PPARA/COLEC11/INS/HLA-A/ABHD12/PARP4/PRDX1/SPN/HLA-F/UBL7/ABI3/C3AR1/KIR2DL4/FOSL2/TIMP1/ALOX5/HP/HLA-E/OTOP1/RELA/CCL21/HLA-B/OAS2/ITGAM/CFHR5/PLA2G4C/FOLR2/PYCARD/CEBPB/PLSCR1/GPSM3/TNFRSF4/PUM1/AIF1/RHBDF2/H19/TEK/GHSR/SERPINA1/ISG15/SIRT2/ANG/LYN/TFEB/TRIM41/STAB1/IFITM1/AGER/SMO/ZNFX1/RAB1A/IFIT3/FLOT2/TYROBP/LAG3/LY86/CEBPA/XCR1/RNF185/CD177/PQBP1/CTSC/BCL2L1/PI3/AKT1/SCGB1A1/DEFB105A/TAP1/IFI6/SLC11A1/LSP1/RAC1/PTPRC/IL17A/FCER1G/CASP4/NFE2L2/ITGB1/EIF2AK2/C8A/ADAM15/GNG7/ALOX5AP/ABCD1/IRAK1/HMGB1/NFKB1/PHB2/MAPKAPK3/HAVCR2/MEFV/IFNGR1/NFKBIL1/TYK2/TRIM8/IFITM3/STAT3/ZMPSTE24/EIF2AK4/PTN/PTGES/SHC1/EIF4G1/LSM14A/LGALS3BP/EIF2AK1/VDAC1
GOBP_POSITIVE_REGULATION_OF_IMMUNE_SYSTEM_	GOBP_POSITIVE_REGULATION_OF_IMMUNE_SYSTEM_	GOBP_POSITIVE_REGULATION_OF_IMMUNE_SYSTEM_PROCESS	196	-0.246453436	-2.408436914	2.39E-06	0.000162758	0.00013032	1268	tags=57%, list=40%, signal=37%	PRAM1/LTF/CD276/TNF/CBFB/CYLD/IL15/PTPN2/THY1/SYK/GPRC5B/CCR1/BTN1A1/CTSG/NMI/BTNL2/CD

PROCESS	PROCESS										1B/NF1/ARRB2/FFAR2/HLA-C/SOCS1/BCL2/TNFRSF21/CACNB3/C1QC/WNT3A/IL1R1/CXCL13/UNC93B1/CCL5/LIPA/CD4/BAD/CD6/PRKCH/COLEC11/HLA-DMA/CD79A/HLA-A/SPN/HLA-F/C3AR1/KIR2DL4/FOSL2/BTN2A2/HLA-E/XBP1/RELA/CCL21/HLA-B/VEGFB/ITGAM/CFHR5/CFHR3/ELF1/PYCARD/PLSCR1/GPSM3/TNFRSF4/PUM1/AIF1/HCLS1/HLA-DOB/BAX/SIRT2/HLA-DRB5/LYN/HLA-DMB/TRIM41/AGER/LIME1/ZNFX1/FLOT2/TYROBP/LAG3/CEBPA/RNF185/CD177/HLA-DQB1/PQBP1/IGF2/CTSC/AKT1/SLC11A1/EIF2B1/CSK/RAC1/PTPRC/IL17A/FCER1G/PPP3CA/HLA-DRB3/GLI2/EIF2AK2/C8A/IRAK1/HMGB1/NFKB1/PHB2/MAPKAPK3/MMP14/HAVCR2/MEFV/NFKBIL1/CHRNB2/TYK2/EIF2AK4/PTN/EFNB1/ABL1/LSM14A
GOBP_CYTOKINE_PRODUCTION	GOBP_CYTOKINE_PRODUCTION	GOBP_CYTOKINE_PRODUCTION	158	-0.265009335	-2.466416499	2.49E-06	0.000167265	0.000133929	1385	tags=63%, list=43%, signal=38%	MERTK/EXTL3/ANXA4/LTB/POU2AF1/UCN/MAP2K3/GATA4/TNXB/LTF/CD276/TNF/CYLD/IL15/SYK/GPRC5B/BTN1A1/TRIM16/SULF1/APOD/NMI/WNT11/LUM/BTNL2/INHBA/ARRB2/FFAR2/SOCS1/CYP1B1/TNFRSF21/SNAI2/ORM2/WNT3A/IL1R1/HSPB1/UNC93B1/CUEDC2/NFATC4/ADIPOQ/CD4/CD6/PPARA/ITGB8/INS/HLA-A/CD34/SPN/HLA-F/C3AR1/KIR2DL4/BTN2A2/SMAD7/KLF2/ALOX5/HLA-E/XBP1/RELA/HLA-B/OAS2/ELF1/PYCARD/CEBPB/GPSM3/AIF1/H19/GHSR/IQGAP1/ISG15/PCSK5/LYN/AIRE/AGER/RAB1A/CDH3/TYROBP/LAG3/COL3A1/PQBP1/ATF4/SCGB1A1/SLC11A1/CSK/RAC1/PTPRC/IL17A/FCER1G/EIF2AK2/ABCD1/IRAK1/HMGB1/NFKB1/HAVCR2/MEFV/IFNGR1/NFKBIL1/TYK2/HEG1/STAT3/TSPO/ABL1

GOBP_CHROMOSOME_SEGREGATION	GOBP_CHROMOSOME_SEGREGATION	GOBP_CHROMOSOME_SEGREGATION	88	0.313695871	2.721257365	2.67E-06	0.00017665	0.000141443	787	tags=49%, list=25%, signal=38%	SRPK1/SPAG5/CCNB2/C1orf112/PTTG2/PTTG1/STAG3/CSNK2A2/CENPE/CENPM/CDCA8/KATNB1/CEP63/NDE1/AURKC/CENPH/ITGB3BP/TPR/SMC3/TRIP13/BRD7/PINX1/KIF23/CDK5RAP2/MAD2L1BP/DYNC1LI1/TUBG1/BUB1B/TOP3A/CHEK2/KIF4A/ESPL1/KPNB1/SMC6/TPX2/TUBG2/ZW10/MAD2L2/KIF22/AKAP8L/NUSAP1/SMARCC1/TUBB
GOMF_ATP_DEPENDENT_ACTIVITY	GOMF_ATP_DEPENDENT_ACTIVITY	GOMF_ATP_DEPENDENT_ACTIVITY	109	0.287298893	2.617498382	2.81E-06	0.000183456	0.000146893	780	tags=45%, list=24%, signal=35%	DDX20/KIF3A/RFC4/ATP6V1E2/DYNLRB2/SMCHD1/HSPA1L/SAE1/DHX16/SETX/HSPA14/ATAD2/CENPE/RAD51/SMC1B/CLPB/DHX30/KATNA1/DNAH17/MYO9A/RECQL4/HSPA2/DNAH8/HSPH1/SMC3/BLM/RUVBL1/ATF7IP/ATP6V1F/TRIP13/RAD17/KIF23/MYH10/WRN/KIFC3/DDX52/CHD1L/RAD50/KIF4A/CLPX/ATP6V0A2/ERCC3/RFC1/CDK7/CHD5/KIF22/ATP8A2/MCM5/TOR2A
GOBP_DNA_REPLICATION	GOBP_DNA_REPLICATION	GOBP_DNA_REPLICATION	65	0.356350039	2.79177986	2.91E-06	0.000185153	0.000148252	901	tags=60%, list=28%, signal=44%	GMNN/DONSON/RFC4/POLG2/CHAF1B/FAF1/RAD51/PCNA/POLB/TRAIP/TOP1MT/RECQL4/PRIM1/SMC3/BLM/RUVBL1/POLRMT/GTPBP4/RAD17/POLA2/USP37/TIMELESS/WRN/CDC7/RAD50/BARD1/POLE/TTF1/ATR/RFC1/RMI1/BAZ1A/ENDOG/MCM5/PARP1/RBBP6/MCM3/POLI/HMGA1
GOBP_ORGANELLE_FISSION	GOBP_ORGANELLE_FISSION	GOBP_ORGANELLE_FISSION	108	0.291640549	2.653445954	2.88E-06	0.000185153	0.000148252	733	tags=44%, list=23%, signal=35%	SPAG5/CCNB2/PTTG2/PTTG1/STAG3/CKS2/CENPE/RAD51/CDC14B/MYBL1/CDCA8/RANBP1/KATNB1/CDC25C/NDE1/AURKC/HSPA2/MTFR1/TPR/PEX11G/PIWIL2/SMC3/DDHD1/TRIP13/AURKAIP1/TDRKH/PINX1/KIF23/CDK5RAP2/MAD2L1BP/FZR1/DYNC1LI1/TUBG1/BUB1B/PINK1/RAD50/UBE2S/CHEK2/KIF4A/ESPL1/KPNB1/TPX2/TUBG2/ZW10/MAD2L2/RMI1/KIF22/AKAP8L
GOBP_DETECTION	GOBP_DETECTION	GOBP_DETECTION_OF	78	-0.33683709	-2.530799744	3.21E-06	0.0002011	0.0001610	1508	tags=74%, list=47%,	OR2J2/OR4P4/OR4M1/OR5D18/TAS2

_OF_STIMULUS_INVOLVED_IN_SENSORY_PERCEPTION	_OF_STIMULUS_INVOLVED_IN_SENSORY_PERCEPTION	_STIMULUS_INVOLVED_IN_SENSORY_PERCEPTION					07	26		signal=40%	R60/OR13C8/OR1D2/OR10A4/OR1F1/OR5AS1/OR11L1/OR52N2/OR52B6/OR51L1/FYN/TAS2R41/OR4D1/TNF/OR4K14/OR6B2/OR2B11/TAS2R40/OR8A1/OR4S1/OR8S1/OR7D2/OR51S1/OR8D1/OR10J5/OR10T2/OR6M1/OR1D4/OR52M1/OR8G1/OR6K2/OR6Y1/TAS2R9/SERPINE2/OR7G2/OR1E2/OR4K17/OR8B8/OR9K2/OR13C4/OR6V1/OR6C4/OR10H4/OR52D1/TAS2R39/OR2T4/OR51A7/OR6X1/OR4D5/OR8H2/OR3A3/OR5F1/OR5V1/OR52E4
GOBP_POSITIVE_REGULATION_OF_CELL_ACTIVATION	GOBP_POSITIVE_REGULATION_OF_CELL_ACTIVATION	GOBP_POSITIVE_REGULATION_OF_CELL_ACTIVATION	78	-0.336606223	-2.529065142	3.26E-06	0.000202106	0.000161826	866	tags=53%, list=27%, signal=39%	CCL5/CD4/BAD/CD6/HLA-DMA/HLA-A/SPN/HLA-F/BTN2A2/HLA-E/XBP1/CCL21/ITGAM/PYCARD/TNFRSF4/AIF1/HLA-DOB/HLA-DRB5/LYN/HLA-DMB/AGER/FLOT2/TYROBP/CEBPA/CD177/HLA-DQB1/IGF2/CTSC/AKT1/CSK/PTPRC/PPP3CA/HLA-DRB3/GLI2/HMGB1/MMP14/HAVCR2/CHRNB2/TYK2/EFNB1/ABL1
HP_ABNORMAL_SPERM_MOTILITY	HP_ABNORMAL_SPERM_MOTILITY	HP_ABNORMAL_SPERM_MOTILITY	16	0.653736355	2.917223685	3.36E-06	0.00020281	0.000162389	469	tags=69%, list=15%, signal=59%	TTC21A/DNALI1/DZIP1/IFT74/CATSPER2/ZMYND10/KLHL10/DNAH17/DNAH8/FOXJ1/DNAI1
HP_INFERTILITY	HP_INFERTILITY	HP_INFERTILITY	31	0.487902692	2.839092699	3.32E-06	0.00020281	0.000162389	469	tags=55%, list=15%, signal=47%	TTC21A/DNALI1/DZIP1/IFT74/STAG3/CATSPER2/FKBP6/ZMYND10/KLHL10/DNAH17/AURKC/DNAH8/BLM/TRIP13/GGPS1/FOXJ1/DNAI1
GOCC_CHROMOSOME_CENTROMERIC_REGION	GOCC_CHROMOSOME_CENTROMERIC_REGION	GOCC_CHROMOSOME_CENTROMERIC_REGION	53	0.387994414	2.836134081	3.58E-06	0.000212855	0.000170429	786	tags=55%, list=25%, signal=42%	SPAG5/C1orf112/SUGT1/STAG3/CENPE/CENPM/AHCTF1/PAFAH1B1/CDCA8/SMC1B/SUV39H2/NDE1/AURKC/CENPH/ITGB3BP/TPR/SMC3/PPP1CC/BRD7/PINX1/DYNC1LI1/BUB1B/SMC6/CBX1/ZW10/KIF22/BAZ1A/NUP107/SMARCC1
GOBP_RESPONSE_TO_BACTERIUM	GOBP_RESPONSE_TO_BACTERIUM	GOBP_RESPONSE_TO_BACTERIUM	117	-0.293240634	-2.47781561	3.61E-06	0.000212855	0.000170429	871	tags=49%, list=27%, signal=37%	ADIPOQ/ADAMTS5/CD4/CCR5/FMO1/MPEG1/CD6/HLA-A/FKBP5/SPN/KMO/FOSL2/PRDX3/HP/HLA-E/XBP1/RELA/HLA-B/OAS2/PYCARD/CEBPB/GHSR/ALAD/ISG15/RNF5/LRG1/CLDN3/ANG/LYN/TFEB/TRIM41/STAB1/HERC6/ZNFX1/RAB1A/LY86/MA

											OB/PI3/AKT1/SCGB1A1/DEFB105A/SLC11A1/IL17A/FCER1G/CASP4/CDK4/IRAK1/HMGB1/NFKB1/MAPKAPK3/HAVCR2/OCIAD2/NFKBIL1/FUCA2/TSPO/SHC1/ABL1
GOBP_POSITIVE_REGULATION_OF_LYMPHOCYTE_ACTIVATION	GOBP_POSITIVE_REGULATION_OF_LYMPHOCYTE_ACTIVATION	GOBP_POSITIVE_REGULATION_OF_LYMPHOCYTE_ACTIVATION	67	-0.368791182	-2.651501317	3.98E-06	0.000228888	0.00018327	1007	tags=60%, list=31%, signal=42%	SOCS1/BCL2/WNT3A/CCL5/CD4/BAD/CD6/HLA-DMA/HLA-A/SPN/HLA-F/BTN2A2/HLA-E/XBP1/CCL21/PYCARD/TNFRSF4/AIF1/HLA-DOB/HLA-DRB5/LYN/HLA-DMB/AGER/FLOT2/TYROBP/HLA-DQB1/IGF2/AKT1/CSK/PTPRC/PPP3CA/HLA-DRB3/GLI2/HMGB1/MMP14/HAVCR2/CHRNB2/TYK2/EFNB1/ABL1
GOBP_HOMEOSTATIC_PROCESS	GOBP_HOMEOSTATIC_PROCESS	GOBP_HOMEOSTATIC_PROCESS	337	-0.20013992	-2.227079735	3.98E-06	0.000228888	0.00018327	1259	tags=51%, list=39%, signal=35%	LTF/MT1H/RAB20/TNF/ATP1A2/EHMT1/CLN8/ATP6V1G2/IL15/CREG1/PTPN2/THY1/SYK/CLCN6/KCNE3/CCR1/CROCC/ATP2A1/MPV17/SV2A/MT1E/GPR12/SGIP1/PRR4/PNPLA4/SQSTM1/IRX3/ANGPTL4/NF1/INHBA/GPR21/CLDN5/ATP2B3/NUBP1/FXN/MTHFD1/MT1B/AQP6/FFAR2/BCR/NR1H2/GCLC/ATP7B/FKBP1A/ATP6V0A1/ATP1B1/BCL2/SLC12A8/NQO1/RPH3AL/OCIAD1/ETS1/HSPB1/ABHD6/ABCB7/HTR1B/ADIPOQ/CCL5/ADAMTS5/IBTK/PDK2/LIPA/CCR5/OR10J5/BAD/FMO1/STIM1/INS/TPCN1/HIPK2/MAFB/RBP1/CD34/CITED2/CNNM2/PRDX1/EMX1/ACOX1/FOSL2/ATP13A1/ARMCX1/PRDX3/KLF2/ALOX5/XBP1/CCL21/SLC9A8/TMEM119/ABHD4/CYBRD1/CEBPB/GSTO1/MYC/YAP1/UBTF/HCLS1/BMP4/WFS1/ITPR1/BAX/ISG15/HEATR3/NOVA1/CLDN3/CALM3/FECH/ANG/LYN/SCARA5/SLC12A9/AGER/LIME1/SMO/ADIPOR2/SLC25A23/CDH3/ANXA6/GRM1/SLC16A1/CEBPA/XCR1/OSBP/TRPM2/COL3A1/HAS2/PRKAB2/ATF4/AKT1/SLC11A1/CLN3/CSK/SLC34A2/AKR1C1/CLNS1A/PTPRC/AT

GOBP_RESPONSE_TO_ENDOGENOUS_STIMULUS	GOBP_RESPONSE_TO_ENDOGENOUS_STIMULUS	GOBP_RESPONSE_TO_ENDOGENOUS_STIMULUS	351	-0.19404011	-2.174063398	4.29E-06	0.000243788	0.000195201	1324	tags=53%, list=41%, signal=35%	P1A3/IL17A/TFE3/PPP3CA/SLC29A1/SLC31A1/PPP2R1A/NFE2L2/CCDC51/GPRASP2/MUC2/BACE2/SLC30A9/KCNB1/ITGB1/EBF2/CFL2/F2RL3/ABCD1/HMGB1/ZBTB7A/ADIPOR1/TFF3/CTSK/OCIAD2/CRTC3/SLC25A5/STAT3/ATP2A3/CCDC115/PTGES/TSPO/NADK/ACO1/EIF4G1/ABL1/EIF2AK1 UCN/MAP2K3/PDGFRA/ENG/GATA4/APAF1/FYN/CYFIP1/DIAPH1/TNXB/CLDN4/PTP4A3/TNF/ATP1A2/PTPN2/LRP6/COL4A1/SYK/TRIM16/SULF1/UGCG/SLC26A5/SAFB2/CYP11A1/SSTR3/VPS18/BCHE/FSHR/EHD4/CSHL1/RARG/ACTN2/INHBA/GPR21/CLDN5/COL4A6/ARRB2/ADAMTSL2/MYOG/NR1H2/GCLC/EMD/FKBP1A/SOCS2/SOCS1/CYP1B1/ELK1/MYOD1/BCL2/NQO1/WNT1/SNAI2/ZNF703/TSC1/NRP2/ARPC1B/CXCL13/HSPB1/MDM2/UBE2D1/HTR1B/ADIPOQ/CCL5/PDK2/VPS11/DHCR24/PPARA/ILK/ITGB8/GPR83/INS/FNTA/HIPK2/RXRB/FAM107A/CITED2/MEN1/CAT/AANAT/BTG2/FOSL2/TIMP1/TGFBRAP1/TSHB/SMAD7/KLF2/FRS2/OTOP1/XBP1/RELA/CCL21/VEGFB/GRB7/FAM20C/FOLR2/CEBPB/MYC/UCN3/YAP1/INPPL1/CPNE3/GNB1/GNAI1/MSI1/NRP1/HCLS1/COL4A2/BMP4/TEK/GHSR/GLB1/ITPR1/TFPI/IQGAP1/ALAD/HTRA1/CRHR2/HRH3/LRG1/SIRT2/DAB2/FECH/RAPGEF1/ANG/LYN/TRIM41/AGER/PITX3/TNFSF10/DDX54/ADIPOR2/FZD4/RGS10/NTF3/FGF1/MAOB/TRPM2/CHRD/COL3A1/HAS2/IGF2/BCL2L1/AKT1/SCGB1A1/EIF2B1/CSK/PAX9/SLC34A2/AKR1C1/PRMT2/PTPRC/ATP1A3/RGMB/FCER1G/PPP3CA/ITGA3/CASP4/MAS1L/NFE2L2/NFIA/PDGFRB/S

											TUB1/ITGB1/CDK5/APLN/NFKB1/PHB2/ZBTB7A/MMP14/ADIPOR1/CTSK/UBE2L3/CHRNB2/DENND4C/KLF9/TYK2/PPP2R5B/FBN1/STAT3/ZMPSTE24/FGFR1/TSPO/SHC1/BCL2L2/ITGB1BP1/ABL1
GOBP_SENSORY_PERCEPTION	GOBP_SENSORY_PERCEPTION	GOBP_SENSORY_PERCEPTION	152	-0.261446552	-2.399365737	5.00E-06	0.000280582	0.000224661	1627	tags=72%, list=51%, signal=37%	OR2A12/TRPV1/PDCL/RP1L1/TAS2R10/KCNQ3/SIX3/ARR3/OR2J2/OR4P4/GJC1/OR4M1/CHRNA7/OR5D18/SLC17A8/TAS2R60/OR13C8/OR1D2/OR10A4/OR1F1/OR5AS1/OR11L1/UCN/OR52N2/OR52B6/OR51L1/RRH/FYN/DIAPH1/MRGPRX2/TAS2R41/OR4D1/EML2/TNF/CLN8/OR4K14/OR6B2/SLC26A5/OR2B11/PRR4/LUM/TAS2R40/REEP2/OR8A1/RDH5/OR4S1/FXN/TAAR5/LCTL/TRIOBP/ATP8B1/OR8S1/OR7D2/OR51S1/IMPG2/OR8D1/SNAI2/CRYGA/OR10J5/OAT/TTC8/B3GNT2/OR10T2/OR6M1/OR1D4/OR52M1/OR8G1/OR6K2/TGFBI/OR6Y1/TAS2R9/SERPINE2/OR7G2/OR1E2/GNB1/OR4K17/OR8B8/OR9K2/OR13C4/WFS1/NAV2/CLIC5/NOB1/FZD4/CDH3/OR6V1/GRM1/OR6C4/OR10H4/OR52D1/OTOR/TAS2R39/P2RX2/OR2T4/OR51A7/GRK1/OR6X1/OR4D5/OR8H2/OR3A3/HPS1/CDK5/SCARB2/OR5F1/OR5V1/LAMC3/OR52E4/CHRNB2/PTGES
GOBP_CILIUM_ORGANIZATION	GOBP_CILIUM_ORGANIZATION	GOBP_CILIUM_ORGANIZATION	73	0.338541971	2.734562049	5.13E-06	0.000284729	0.000227982	729	tags=49%, list=23%, signal=39%	KIF3A/TTC21A/DZIP1/RFX2/IFT74/CCDC96/RAB3IP/SPATA6/AKAP4/CLUAP1/ZMYND10/CDC14B/WDR54/TBC1D3/DNAH17/IFT122/RAB8B/DNAH8/CEP250/CEP350/CCDC42/FOXJ1/RFX4/SSNA1/KIAA0753/DNAI1/IQCG/IFT20/BBS4/IQCB1/TBC1D1/MKKS/TTC17/KIFAP3/GSK3B/CEP290
GOBP_DNA_TEMPLATED_DNA_REPLICATION	GOBP_DNA_TEMPLATED_DNA_REPLICATION	GOBP_DNA_TEMPLATED_DNA_REPLICATION	40	0.429347981	2.818024579	5.42E-06	0.00029419	0.000235557	808	tags=62%, list=25%, signal=47%	GMNN/DONSON/RFC4/POLG2/RAD51/PCNA/POLB/TRAIP/RECQL4/PRIM1/BLM/POLRMT/POLA2/TIMELESS/WRN/CDC7/RAD50/BARD1/POLE/

											ATR/RFC1/BAZ1A/ENDOG/MCM5/PARP1
GOBP_INORGANIC_ION_HOMEOSTASIS	GOBP_INORGANIC_ION_HOMEOSTASIS	GOBP_INORGANIC_ION_HOMEOSTASIS	105	-0.298721859	-2.450878834	5.37E-06	0.00029419	0.000235557	1989	tags=90%, list=62%, signal=35%	MT3/EDN1/SLC34A1/SLC12A1/BDKRB1/CACNA1C/GP1BB/VAPB/SCO1/GRIN1/BCAP31/CDH23/S100A14/HCRTR1/RASA3/BOK/ATG5/UMOD/NPTN/TRPV1/GPR3/FIS1/SLC12A3/ATP1B2/TRPV5/CAV3/CHRNA7/SLC17A8/SLC34A3/KLHL3/CCL8/SLC17A7/SRI/SYPL2/DIAPH1/MT1H/ATP1A2/THY1/KCNE3/CCR1/ATP2A1/SV2A/MT1E/GPR12/ATP2B3/NUBP1/FXN/MT1B/ATP7B/FKBP1A/ATP1B1/BCL2/SLC12A8/ABCB7/HTR1B/CCL5/IBTK/CCR5/STIM1/TPCN1/CNNM2/ATP13A1/CCL21/CYBRD1/GSTO1/MYC/WFS1/ITPR1/BAX/CALM3/FECH/LYN/SCARA5/SLC12A9/LIME1/SLC25A23/ANXA6/GRM1/XCR1/TRPM2/ATF4/SLC11A1/CLN3/SLC34A2/PTPRC/ATP1A3/SLC31A1/SLC30A9/F2RL3/ATP2A3/CCDC115/ACO1/ABL1/EIF2AK1
HP_FETAL_ONSET	HP_FETAL_ONSET	HP_FETAL_ONSET	41	0.433614293	2.867994178	5.54E-06	0.000294239	0.000235596	519	tags=51%, list=16%, signal=43%	ASNS/NUP88/SLC25A19/DEF6/NUP155/CPSF3/CENPE/PSMD12/CLPB/TRAIP/WNT3/RECQL4/PRIM1/TRIP11/PRKAG2/CHRNG/VRK1/PYCR1/FZR1/KIAA0753/THSD1
GOBP_REGULATION_OF_LOCOMOTION	GOBP_REGULATION_OF_LOCOMOTION	GOBP_REGULATION_OF_LOCOMOTION	201	-0.23860558	-2.358497567	5.49E-06	0.000294239	0.000235596	1325	tags=58%, list=41%, signal=36%	SH3BP1/MAP2K3/MYOC/OXSR1/PDGFRA/ENG/ARHGAP18/TNXB/CLDN4/CLASP2/TNF/SPATA13/PTPN2/THY1/CCR1/SULF1/SLC26A5/APOD/STX3/WNT11/PODXL/NF1/CLDN5/PLXNB2/BCR/DUOX2/CYP1B1/BCL2/SNAI2/MALAT1/ZNF703/WNT3A/NRP2/IL1R1/ETS1/CXCL13/PLAU/HSPB1/ABHD6/MDM2/CXCR3/ADIPOQ/CCL5/EMP2/INS/FAM107A/CITED2/PLEKHG3/SPN/MAZ/ABI3/C3AR1/TIMP1/MINK1/SMAD7/XBP1/CCL21/VEGFB/GRB7/PYCARD/SERPINE2/PTP4A1/MYADM/CPNE3/GPSM3/NRP1/AIF1/BMP4/TEK/GHSR/SLURP1/CLDN3/

										DAB2/CDH11/LYN/AIRE/IFITM1/AGER/SMO/FZD4/PLXNA1/NTF3/FGF1/RAB11A/PODN/CHRD/COL3A1/HAS2/AKT1/ARHGDIB/CLN3/RAC1/ARHGEF7/PTPRC/GNA12/F10/PPP3CA/ITGA3/NFE2L2/PDGFRB/ITGB1/CDK5/ADAM15/MSN/ST3GAL4/HMGB1/MMP14/ADIPOR1/RHOC/STAT3/FGFR1/PTN/GADD45A/TRIP6/ITGB1BP1/ABL1
GOBP_POSITIVE_REGULATION_OF_IMMUNE_RESPONSE	GOBP_POSITIVE_REGULATION_OF_IMMUNE_RESPONSE	GOBP_POSITIVE_REGULATION_OF_IMMUNE_RESPONSE	139	-0.27526251	-2.488168261	5.85E-06	0.000307129	0.000245918	1268	tags=60%, list=40%, signal=38%	PRAM1/LTF/CD276/TNF/CYLD/IL15/PTPN2/THY1/SYK/BTN1A1/CTSG/NMI/BTNL2/CD1B/ARRB2/FFAR2/HLA-C/BCL2/TNFRSF21/CACNB3/C1QC/IL1R1/UNC93B1/CCL5/LIPA/CD4/PRKCH/COLEC11/HLA-DMA/CD79A/HLA-A/HLA-F/C3AR1/KIR2DL4/FOSL2/BTN2A2/HLA-E/XBP1/RELA/HLA-B/ITGAM/CFHR5/CFHR3/ELF1/PYCARD/PLSCR1/PUM1/HLA-DOB/BAX/SIRT2/HLA-DRB5/LYN/HLA-DMB/TRIM41/LIME1/ZNFX1/FLOT2/TYROBP/LAG3/RNF185/CD177/HLA-DQB1/PQBP1/SLC11A1/EIF2B1/CSK/PTPRC/IL17A/FCER1G/HLA-DRB3/EIF2AK2/C8A/IRAK1/HMGB1/NFKB1/PHB2/MAPKAPK3/HAVCR2/MEFV/NFKBIL1/TYK2/EIF2AK4/ABL1/LSM14A
GOBP_MEIOTIC_CELL_CYCLE	GOBP_MEIOTIC_CELL_CYCLE	GOBP_MEIOTIC_CELL_CYCLE	50	0.394808796	2.812701525	6.47E-06	0.000336088	0.000269105	706	tags=52%, list=22%, signal=41%	CCNB2/PTTG2/PTTG1/STAG3/CKS2/RAD51/FKBP6/MYBL1/SMC1B/CDC25C/AURKC/HSPA2/PIWIL2/SMC3/ANAPC10/TRIP13/TDRKH/FZR1/TUBG1/TOP3A/RAD50/ESPL1/BOLL/TUBG2/ZW10/RMI1
GOCC_TRANSFERASE_COMPLEX	GOCC_TRANSFERASE_COMPLEX	GOCC_TRANSFERASE_COMPLEX	196	0.222418762	2.365011614	6.63E-06	0.000340888	0.000272948	903	tags=45%, list=28%, signal=35%	TBPL1/FBXO15/CCNB2/SUGT1/FBXO7/BRD1/CACYBP/CCNH/POLG2/AKAP4/PARD6A/CSNK2A2/CKS2/DERL2/TSSK2/RAD51/UBE2D3/PCNA/FBXO24/FBXL18/DR1/PRKAA1/ASB1/PAXIP1/PRIM1/ANAPC10/RUVBL1/POLRMT/SUZ12/SUPT3H/UBE2N/PC

											GF6/UBE2D2/POLA2/PAF1/RMND5B/PRKAG2/WDR26/TAF6/FBXO39/FZR1/FBXL2/TEX10/BUB1B/CAB39/KCTD10/TAF10/SHARPIN/GTF2H2/UBE2S/BARD1/POLE/TBK1/SMC6/DERL3/TRAF2/GTF2H1/PIK3CG/POLR3E/ERCC3/CDK7/KBTBD8/MAD2L2/KRTCAP2/RCHY1/DDA1/GTF2E1/ATG12/DCUN1D3/RMND5A/TAF9/KLHDC2/KLHL8/SETD1A/RNF7/PIGP/PIGH/DCUN1D5/RANBP2/CDC16/ANKRD9/CDK5R1/KLHL7/CUL2/MCM3/PFKM/BCCIP/FBXO11/UBE2C
GOBP_CILIUM_OR_FLAGELLUM_DEPENDENT_CELL_MOTILITY	GOBP_CILIUM_OR_FLAGELLUM_DEPENDENT_CELL_MOTILITY	GOBP_CILIUM_OR_FLAGELLUM_DEPENDENT_CELL_MOTILITY	19	0.605439438	2.948060725	7.63E-06	0.000387922	0.000310608	594	tags=74%, list=19%, signal=60%	EFHC1/TTC21A/LZTFL1/DZIP1/ROPN1L/AKAP4/CATSPER2/IQCF1/DNAH17/DNAH8/DNAI1/IQCG/BBS4/MKKS
GOBP_TRANSMEMBRANE_TRANSPORT	GOBP_TRANSMEMBRANE_TRANSPORT	GOBP_TRANSMEMBRANE_TRANSPORT	285	-0.206934469	-2.235008097	8.41E-06	0.00041855	0.000335132	1950	tags=78%, list=61%, signal=34%	SLC34A1/SLC4A5/SLC6A8/SLC12A1/BDKRB1/CACNA1C/C3/GRM6/FGF13/GP1BB/ABCB1/GRIN1/SLC13A2/SNTA1/GAL/WWP1/SLC16A12/KCNJ9/KCNG1/SLC4A1/SEC63/CA2/RALBP1/AQP1/HCN4/SLC7A4/HTR3A/SLC6A1/GLP1R/RASA3/ABCD3/SHANK1/SLC5A7/KCNMB1/ABCG2/ACSL1/FGF19/SLC5A11/SLC23A3/SLC25A22/KCNH7/ATG5/UMOD/SLC7A14/TRPV1/MAPK8IP2/OAZ2/KCNMB3/CLCA4/SLC12A3/KCNQ3/SLC22A2/TMEM163/ATP1B2/TOMM40/GABRR1/TRPV5/KCNS3/SLC41A3/GJC1/CAV3/HCN3/CHRNA7/SLC35A1/TAP2/SLC17A8/SLC34A3/NIPA2/KCNJ6/MFSD1/SLC25A37/GRIA4/SLC16A9/SLC17A7/SRI/KCNAB3/OXSR1/DLG4/FYN/DIAPH1/CLDN4/SLC25A36/LTF/TNF/ATP1A2/TMEM63A/CLN8/ATP6V1G2/STOM/THY1/CLCN6/KCNE3/ATP2A1/ACSL5/MPV17/SV2A/SLC35A4/SLC26A5/SLC25A20/CHRNA2/CACNA1B/ACTN2/CACNA1E/ATP2B3/SLC10A5/FXN/AQP6/BCR/ATP7B/KCNS

											2/ATP8B1/FKBP1A/ATP6V0A1/ATP1B1/BCL2/SLC12A8/SLC22A11/CACNB3/SLC29A2/TSC1/WNT3A/FXYD5/ABCB7/KCNAB2/ADIPOQ/FHL1/CLCN2/IBTK/CCR5/SLC22A8/STIM1/SLC22A7/INS/TPCN1/SLC16A11/ABCD4/TMCO3/CNNM2/TMEM109/SCN2B/ATP13A1/MINK1/OTOP1/CCL21/SLC9A8/KCNJ1/ABCC10/CYBRD1/GSTO1/SLC25A12/SLC32A1/GJB1/ITPR1/SLC44A1/KCNH4/BAX/RNF5/CALM3/CLCC1/LYN/SLC29A3/CLIC5/SCARA5/SLC12A9/OTOP2/LIME1/SLC35B2/COX7B/SLC25A23/GJA4/SLC6A17/KCNB2/ANXA6/GRM1/SLC16A1/XCR1/SLC2A13/RNF185/COX15/TRPM2/P2RX2/GRIK5/KCNK5/AKT1/TAP1/PPIF/SLC11A1/CLN3/SLC34A2/CABP5/PTPRC/SLC26A1/ATP1A3/SFXN3/OTOP3/PPP3CA/SLC29A1/SLC31A1/NFE2L2/TIMM17B/CCDC51/TOMM20/SLC30A9/KCNB1/ITGB1/F2RL3/CNIH3/ABCD1/LASP1/SEC61A1/PHB2/CLIC1/STEAP3/CHRNB2/COX7A1/SLC25A5/SLC35D2/ATP2A3/SLC14A2/PRAF2/COX8A/ABL1/VDAC1
GOBP_REGULATION_OF_IMMUNE_SYSTEM_PROCESS	GOBP_REGULATION_OF_IMMUNE_SYSTEM_PROCESS	GOBP_REGULATION_OF_IMMUNE_SYSTEM_PROCESS	281	-0.207917091	-2.233228085	8.38E-06	0.00041855	0.000335132	1268	tags=53%, list=40%, signal=35%	PRAM1/LTF/CD276/TNF/CBFB/CYLD/IL15/PTPN2/THY1/SYK/GPRC5B/CCR1/BTN1A1/CTSG/APOD/NMI/BTNL2/CD1B/NF1/RARG/INHBA/ARRB2/FFAR2/HLA-C/BCR/NR1H2/PIAS3/FKBP1A/PSG8/SOCS1/TRAFD1/BCL2/TNFRSF21/CACNB3/C1QC/SNAI2/FGL2/ORM2/WNT3A/IL1R1/ETS1/CXCL13/UNC93B1/CUEDC2/CXCR3/ADIPOQ/CCL5/LIPA/CD4/BAD/CD6/PRKCH/COLEC11/HLA-DMA/INS/CD79A/HLA-A/MAFB/SPN/HLA-F/C3AR1/KIR2DL4/FOSL2/BTN2A2/SMAD7/HLA-E/OTOP1/XBP1/RELA/CCL21/HLA-B/VEGFB/ITGAM/TCTA/CFHR5/CFHR3/ELF1/PYCARD/CEBPB/MYC/P

GOBP_BLOOD_VESSEL_MORPHOGENESIS	GOBP_BLOOD_VESSEL_MORPHOGENESIS	GOBP_BLOOD_VESSEL_MORPHOGENESIS	136	-0.269717267	-2.416037866	9.08E-06	0.000447587	0.000358382	1363	tags=61%, list=43%, signal=37%	LSCR1/INPPL1/GPSM3/TNFRSF4/PUM1/AIF1/HCLS1/RHBDF2/BMP4/HLA-DOB/BAX/ISG15/SIRT2/HLA-DRB5/LYN/HLA-DMB/AIRE/TRIM41/AGER/LIME1/ZNFX1/FLOT2/TYROBP/LAG3/CEBPA/RNF185/CD177/COL3A1/HLA-DQB1/PQBP1/IGF2/CTSC/AKT1/SCGB1A1/SLC11A1/EIF2B1/CSK/RAC1/PTPRC/IL17A/TFE3/FCER1G/PPP3CA/HLA-DRB3/GLI2/NFE2L2/SUPT6H/EIF2AK2/C8A/MSN/ST3GAL4/IRAK1/HMGB1/NFKB1/PHB2/MAPKAPK3/MMP14/HAVCR2/MEFV/NFKBIL1/CHRNB2/TYK2/FBN1/STAT3/EIF2AK4/PTN/EFNB1/ABL1/LSM14AZFPM2/MMRN2/CCM2/RNH1/PDGFRA/ENG/GATA4/E2F7/TNF/TBX6/COL4A1/THY1/SYK/SULF1/APOD/WNT11/ANGPTL4/NF1/CLDN5/CYP1B1/GPR15/HAND2/TGFA/NRP2/ETS1/CXCL13/HSPB1/CXCR3/NFATC4/EMP2/OR10J5/STIM1/ITGB8/HIPK2/COL15A1/CD34/CITED2/C3AR1/LOXL2/SMAD7/KLF2/ALOX5/XBP1/VEGFB/TGFBI/YAP1/CUL7/NRP1/COL4A2/BMP4/TEK/GHSR/BAX/LRG1/ANG/STAB1/SMO/ADIPOR2/FZD4/ADAM12/FGF1/COL3A1/HAS2/AKT1/COL27A1/HS6ST1/ADRA2B/SLC31A1/NFE2L2/PDGFRB/ITGB1/ADAM15/PGK1/APLN/MMP14/NKX2-5/HEG1/PARVA/STAT3/GADD45A/SHC1/ITGB1BP1/ABL1
GOBP_DNA_TEMPLATED_DNA_REPLICATION_MAINTENANCE_OF_FIDELITY	GOBP_DNA_TEMPLATED_DNA_REPLICATION_MAINTENANCE_OF_FIDELITY	GOBP_DNA_TEMPLATED_DNA_REPLICATION_MAINTENANCE_OF_FIDELITY	14	0.659699791	2.823556283	1.05E-05	0.0005105	0.000408756	652	tags=79%, list=20%, signal=63%	DONSON/RAD51/PCNA/TRAIP/BLM/TIMELESS/WRN/RAD50/BARD1/POLE/ATR
GOBP_INFLAMMATORY_RESPONSE	GOBP_INFLAMMATORY_RESPONSE	GOBP_INFLAMMATORY_RESPONSE	149	-0.26203446	-2.392047152	1.13E-05	0.00054686	0.00043787	928	tags=47%, list=29%, signal=35%	ORM2/IL1R1/ETS1/CXCL13/CUEDC2/CXCR3/NFATC4/ADIPOQ/CCL5/LIPA/CCR5/CD6/PPARA/INS/ABHD12/PARP4/SPN/C3AR1/FOSL2/TIMP1/A

										LOX5/HP/HLA-E/RELA/CCL21/ITGAM/PLA2G4C/FOLR2/PYCARD/CEBPB/PLSCR1/GPSM3/TNFRSF4/AIF1/RHBDF2/H19/TEK/GHSR/SERPINA1/SIRT2/LYN/STAB1/AGER/SMO/TYROBP/LY86/CEBPA/XCR1/CTSC/AKT1/SCGB1A1/SLC11A1/RAC1/PTPRC/IL17A/CASP4/NFE2L2/ITGB1/EIF2AK2/ALOX5AP/ABCD1/HMGB1/NFKB1/HAVCR2/MEFV/IFNGR1/STAT3/PTN/PTGES/EIF2AK1
GOCC_LYSOSOMAL_LUMEN	GOCC_LYSOSOMAL_LUMEN	GOCC_LYSOSOMAL_LUMEN	18	-0.592307962	-2.596813017	1.15E-05	0.000549585	0.000440051	860	tags=78%, list=27%, signal=57%	LIPA/OGN/SDC2/MANBA/MAN2B2/PPT2/GLB1/GALC/PDGFRB/SCARB2/CTSK/PRELP/TXNDC5/SGSH
GOBP_CELL_MOTILITY	GOBP_CELL_MOTILITY	GOBP_CELL_MOTILITY	322	-0.195088762	-2.151958435	1.22E-05	0.000578343	0.000463078	1357	tags=54%, list=42%, signal=35%	MMRN2/JAGN1/CCL8/RNH1/SH3BP1/MAP2K3/MYOC/OXSR1/PDGFRA/ENG/FYN/ARHGAP18/TNXB/CLDN4/PTP4A3/CLASP2/TNF/SPATA13/THY1/SYK/CCL22/PLTP/CCR1/CTSG/SULF1/SLC26A5/APOD/IGFBP6/SELPLG/WNT11/CEND1/PODXL/NF1/CLDN5/ARRB2/FFAR2/PLXNB2/BCR/ELMO2/PHACTR4/DUOX2/CYP1B1/GPR15/HAND2/BCL2/SNAI2/MALAT1/ZNF703/NRP2/IL1R1/ETS1/CXCL13/PLAU/HSPB1/ABHD6/MDM2/CXCR3/ADIPOQ/CCL5/EMP2/CCR5/ILK/SDC2/ITGB8/INS/FAM107A/CD34/CITED2/PLEKHG3/SPN/MAZ/CORO6/ABI3/C3AR1/LOXL2/NHLH2/TIMP1/MINK1/SMAD7/ALOX5/NET1/XBP1/CCL21/VEGFB/GRB7/SORD/RHOG/FOLR2/PYCARD/SERPINE2/PTP4A1/MYADM/CPNE3/GPSM3/NRP1/AIF1/BMP4/TEK/EOMES/IQGAP1/SLURP1/BAX/LRG1/CLDN3/CDK5R2/DAB2/CDH11/ANG/LYN/AIRE/DAB1/MIXL1/IFITM1/AGER/SMO/FZD4/RAB1A/PLXNA1/ANXA6/NTF3/FGF1/XCR1/RAB11A/CD177/TRPM2/PODN/CHRD/COL3A1/HAS2/AKT1/GFRA3/ARHGDIB/UBE2B/CLN3/RAC1/ARHGEF7/PT

											PRC/GNA12/F10/IL17A/CDC42BPB/FCER1G/CTTN/PPP3CA/ITGA3/NFE2L2/PDGFRB/CD248/ARF4/ITGB1/CDK5/ADAM15/MSN/ST3GAL4/HMGB1/PHB2/MMP14/ADIPOR1/RHOC/TOR1A/ITGA9/CAP1/ITGB7/TNFAIP1/PARVA/STAT3/FGFR1/PTN/GADD45A/TSPO/TRIP6/EFNB1/ITGB1BP1/ABL1/WWC3
GOBP_REGULATION_OF_IMMUNE_RESPONSE	GOBP_REGULATION_OF_IMMUNE_RESPONSE	GOBP_REGULATION_OF_IMMUNE_RESPONSE	181	-0.237467369	-2.260482078	1.35E-05	0.00063377	0.000507458	1268	tags=56%, list=40%, signal=36%	PRAM1/LTF/CD276/TNF/CYLD/IL15/PTPN2/THY1/SYK/BTN1A1/CTSG/NMI/BTNL2/CD1B/ARRB2/FFAR2/HLA-C/BCR/NR1H2/FKBP1A/TRAFD1/BCL2/TNFRSF21/CACNB3/C1QC/FGL2/IL1R1/CXCL13/UNC93B1/CCL5/LIPA/CD4/PRKCH/COLEC11/HLA-DMA/INS/CD79A/HLA-A/SPN/HLA-F/C3AR1/KIR2DL4/FOSL2/BTN2A2/SMAD7/HLA-E/OTOP1/XBP1/RELA/HLA-B/ITGAM/CFHR5/CFHR3/ELF1/PYCARD/PLSCR1/INPPL1/PUM1/RHBDF2/HLA-DOB/BAX/ISG15/SIRT2/HLA-DRB5/LYN/HLA-DMB/TRIM41/AGER/LIME1/ZNFX1/FLOT2/TYROBP/LAG3/RNF185/CD177/COL3A1/HLA-DQB1/PQBP1/AKT1/SLC11A1/EIF2B1/CSK/PTPRC/IL17A/FCER1G/HLA-DRB3/NFE2L2/SUPT6H/EIF2AK2/C8A/IRAK1/HMGB1/NFKB1/PHB2/MAPKAPK3/HAVCR2/MEFV/NFKBIL1/TYK2/EIF2AK4/ABL1/LSM14A
GOCC_MICROTUBULE_ASSOCIATED_COMPLEX	GOCC_MICROTUBULE_ASSOCIATED_COMPLEX	GOCC_MICROTUBULE_ASSOCIATED_COMPLEX	32	0.453363252	2.675019502	1.37E-05	0.000635718	0.000509018	565	tags=56%, list=18%, signal=47%	KIF3A/DNALI1/DYNLRB2/DYNLL2/PAFAH1B1/CDCA8/KATNA1/DNAH17/KATNB1/NDE1/AURKC/TPR/DNAH8/KIF23/DNAI1/DYNC1LI1/KIFC3/KIF4A
GOBP_BIOLOGICAL_PROCESS_INVOLVED_IN_INTERSPECIES_INTERACTION_BETWEEN_ORGANISMS	GOBP_BIOLOGICAL_PROCESS_INVOLVED_IN_INTERSPECIES_INTERACTION_BETWEEN_ORGANISMS	GOBP_BIOLOGICAL_PROCESS_INVOLVED_IN_INTERSPECIES_INTERACTION_BETWEEN_ORGANISMS	299	-0.199482011	-2.167198085	1.42E-05	0.000651041	0.000521287	1057	tags=45%, list=33%, signal=33%	TRIM4/ARRB2/FFAR2/HLA-C/BCR/NR1H2/MAP3K5/DUOX2/ATP1B1/GPR15/COTL1/TRAFD1/BCL2/NQO1/IFI44/C1QC/FBXO9/FGL2/CXCL13/HSPB1/UNC93B1/ADIPOQ/CCL5/ADAMTS5/CD4/CCR5/FMO1/MPEG1/CD6

											/PPARA/COLEC11/ITGB8/INS/TPCN1/HLA-A/CHMP6/FKBP5/PRDX1/SPN/HLA-F/KMO/UBL7/KIR2DL4/FOSL2/PRDX3/HP/HLA-E/OTOP1/XBP1/RELA/CCL21/HLA-B/OAS2/ITGAM/CFHR5/PYCARD/CEBPB/PLSCR1/TNFRSF4/PUM1/NRP1/AIF1/H19/GHSR/VPS4A/ALAD/BAX/ISG15/RNF5/LRG1/CLDN3/SIRT2/ANG/LYN/TFEB/TRIM41/STAB1/HERC6/IFITM1/ZNFX1/RAB1A/IFIT3/IGF2R/FLOT2/TYROBP/LAG3/LY86/MAOB/RNF185/CD177/PQBP1/BCL2L1/PI3/AKT1/SCGB1A1/DEFB105A/IFI6/SLC11A1/CHMP7/PTPRC/VAPA/IL17A/FCER1G/CASP4/NFE2L2/MUC2/ITGB1/EIF2AK2/C8A/ADAM15/SCARB2/ZFYVE1/CDK4/IRAK1/HMGB1/NFKB1/PHB2/MAPKAPK3/HAVCR2/MEFV/IFNGR1/OCIAD2/NFKBIL1/ITGB7/TYK2/TRIM8/IFITM3/ZMPSTE24/FUCA2/EIF2AK4/TSPO/SHC1/ZBED1/ABL1/LSM14A
HP_ANTENATAL_ONSET	HP_ANTENATAL_ONSET	HP_ANTENATAL_ONSET	50	0.381363583	2.716914984	1.47E-05	0.000663858	0.000531549	542	tags=48%, list=17%, signal=41%	ASNS/NUP88/SLC25A19/DEF6/NUP155/CPSF3/CENPE/PSMD12/CLPB/TRAIP/WNT3/RECQL4/GLUL/PRIM1/TRIP11/PRKAG2/CHRNG/VRK1/PYCR1/FZR1/KIAA0753/BUB1B/THSD1/TOP3A
GOBP_REGULATION_OF_EPITHELIAL_CELL_PROLIFERATION	GOBP_REGULATION_OF_EPITHELIAL_CELL_PROLIFERATION	GOBP_REGULATION_OF_EPITHELIAL_CELL_PROLIFERATION	68	-0.34827037	-2.509203265	1.47E-05	0.000663858	0.000531549	963	tags=56%, list=30%, signal=40%	TGFA/SNAI2/ZNF703/WNT3A/NRP2/BAD/ALOX5/FRS2/XBP1/VEGFB/MYC/YAP1/NRP1/BMP4/TEK/GHSR/SLURP1/BAX/LRG1/DAB2/ANG/SMO/CDH3/FGF1/HAS2/IGF2/AKT1/IQGAP3/CDK4/APLN/HMGB1/PHB2/NKX2-5/KLF9/STAT3/MAGED1/FGFR1/PTN
GOBP_MUSCLE_CELL_MIGRATION	GOBP_MUSCLE_CELL_MIGRATION	GOBP_MUSCLE_CELL_MIGRATION	24	-0.529518779	-2.60000691	1.50E-05	0.000669432	0.000536013	1082	tags=79%, list=34%, signal=53%	NF1/CYP1B1/BCL2/PLAU/MDM2/ADIPOQ/CCL5/NET1/NRP1/AIF1/BMP4/SMO/PLXNA1/HAS2/GNA12/NFE2L2/PDGFRB/PARVA/ITGB1BP1
GOMF_OXIDOREDUCTASE_ACTIVIT	GOMF_OXIDOREDUCTASE_ACTIVIT	GOMF_OXIDOREDUCTASE_ACTIVITY	154	-0.248782143	-2.300471063	1.57E-05	0.000690376	0.000552782	1469	tags=64%, list=46%, signal=37%	NSDHL/HBM/CYP17A1/GPD1/GPHN/PHF2/PPOX/FMO5/CYP4X1/PNPO/K

Y	Y										CNAB3/MECR/ALDH1L1/CYP7A1/CYP2A13/MGST2/PAM/DCXR/PGD/NDUFS1/CYP11A1/GPX5/FADS6/RDH5/FXN/MTHFD1/DUOX2/CYP1B1/DHRS4/NQO1/CYP2S1/HSDL2/DAO/LDHA/CYP2U1/CYP3A43/IDH3B/NDUFB7/KCNAB2/FMO1/PYCR2/DHCR24/HSD17B1/PRDX1/CAT/KMO/LOXL2/ACOX1/ALKBH4/PRDX3/ALOX5/ALDH4A1/SORD/CYBRD1/GLUD2/GSTO1/ALDH2/HR/PIPOX/BLVRA/STAB1/ADI1/COX7B/HSD17B8/MAOB/COX15/AKR7A2/PTGES2/AKR1C1/ALKBH6/DHRSX/CYP4B1/CBR3/AKR7A3/CYP2W1/ADHFE1/RSAD1/AKR1A1/ALDH1A3/CBR1/ACAA1/PDIA6/IMPDH2/PGK1/ALOX5AP/IDH3G/STEAP3/COX7A1/SUMF1/CCS/PDIA5/TXNDC5/PLOD1/LTC4S/PTGES/CTBP1/DHTKD1/COX8A/MSRB2
GOBP_RESPONSE_TO_OXYGEN_CONTAINING_COMPOUND	GOBP_RESPONSE_TO_OXYGEN_CONTAINING_COMPOUND	GOBP_RESPONSE_TO_OXYGEN_CONTAINING_COMPOUND	364	-0.187721905	-2.12142531	1.57E-05	0.000690376	0.000552782	1331	tags=53%, list=42%, signal=35%	SRI/UCN/PEF1/MAP2K3/PDGFRA/DLG4/GATA4/FYN/CYFIP1/CYP7A1/CLDN4/LTF/TNF/ATP1A2/ANKZF1/PTPN2/LRP6/COL4A1/MGST2/CTSG/TRIM16/MPV17/SLC26A5/APOD/CYP11A1/SSTR3/JUND/BCHE/WNT11/SQSTM1/CHRNA2/CSHL1/RARG/CACNA1B/ACTN2/INHBA/GPR21/CLDN5/COL4A6/ARRB2/FXN/MYOG/FFAR2/BCR/GCLC/MAP3K5/DUOX2/SOCS2/SOCS1/CYP1B1/ELK1/HAND2/MYOD1/BCL2/GDI1/NQO1/GYS2/WNT1/SNAI2/ZNF703/TSC1/WNT3A/ARPC1B/CXCL13/MDM2/NFATC4/HTR1B/ADIPOQ/PDK2/LIPA/CCR5/BAD/FMO1/STIM1/CD6/PPARA/INS/RXRB/ADD1/PRDX1/MMP15/CAT/AANAT/KMO/BTG2/FOSL2/TIMP1/PRDX3/TSHB/KLF2/HP/NET1/OTOP1/XBP1/RELA/CCL21/GRB7/FOLR2/PYCARD/CEBPB/UCN3/YAP1/INPPL1/CPNE3/GNB1/GNAI1/UBTF/SLC25A12/H19/T

											EK/GHSR/GLB1/ITPR1/COL6A3/IQGAP1/ALAD/CRHR2/HRH3/SYT12/CLDN3/PMVK/SIRT2/CALM3/FECH/RAPGEF1/LYN/TRIM41/AGER/PITX3/SMO/TNFSF10/FZD4/SLC25A23/IGF2R/RGS10/LY86/CEBPA/MAOB/OSBP/TRPM2/GRAMD1A/COL3A1/P2RX2/IGF2/BCL2L1/AKT1/SCGB1A1/PPIF/SLC11A1/EIF2B1/CSK/AKR1C1/PTPRC/ATP1A3/PPP3CA/SLC29A1/CASP4/MAS1L/NFE2L2/AKR1A1/TOMM20/GOT2/KCNB1/CDK5/ADAM15/MSN/ALOX5AP/CDK4/IRAK1/HMGB1/NFKB1/PHB2/MAPKAPK3/HAVCR2/ADIPOR1/CTSK/NFKBIL1/CHRNB2/DENND4C/KLF9/TYK2/CCS/ENTPD6/FBN1/STAT3/TSPO/NADK/SHC1/BCL2L2/EIF4G1/ABL1/MBD2
GOBP_MONOATOMIC_CATION_TRANSPORT	GOBP_MONOATOMIC_CATION_TRANSPORT	GOBP_MONOATOMIC_CATION_TRANSPORT	202	-0.224930763	-2.223629576	1.59E-05	0.000691777	0.000553904	1950	tags=80%, list=61%, signal=33%	SLC34A1/SLC4A5/SLC6A8/SLC12A1/BDKRB1/CACNA1C/GRM6/FGF13/GP1BB/GRIN1/CASK/SLC13A2/SNTA1/GAL/KCNJ9/KCNG1/AQP1/HCN4/CDH23/DNM2/SLC6A1/RASA3/SLC5A7/KCNMB1/COMMD9/SLC5A11/RAMP2/SLC25A22/KCNH7/ATG5/UMOD/TRPV1/KCNMB3/SLC12A3/KCNQ3/SLC22A2/CDK2/TMEM163/ATP1B2/TRPV5/KCNS3/SLC41A3/CAV3/HCN3/CHRNA7/SLC17A8/SLC34A3/KLHL3/NIPA2/KCNJ6/MCHR1/SLC25A37/CCL8/SLC17A7/SRI/UCN/KCNAB3/OXSR1/FYN/DIAPH1/LTF/ATP1A2/TMEM63A/ATP6V1G2/THY1/KCNE3/CCR1/ATP2A1/CACNA1B/ACTN2/CACNA1E/ATP2B3/SLC10A5/FXN/ATP7B/KCNS2/FKBP1A/ATP6V0A1/ATP1B1/BCL2/SLC12A8/CACNB3/TSC1/WNT3A/FXYD5/ABCB7/KCNAB2/FHL1/CCL5/IBTK/CD4/CCR5/STIM1/TPCN1/TMCO3/CNNM2/CSN2/SCN2B/ATP13A1/OTOP1/CCL21/SLC9A8/KCNJ1/CYBRD1/SERPINE2/GSTO1/S

											LC25A12/SLC32A1/WFS1/ITPR1/PKP2/KCNH4/BAX/CALM3/LYN/SCARA5/SLC12A9/OTOP2/LIME1/COX7B/SLC25A23/GJA4/SLC6A17/KCNB2/ANXA6/SLC16A1/XCR1/COX15/TRPM2/P2RX2/ATF4/KCNK5/AKT1/PPIF/SLC11A1/SLC34A2/CABP5/PTPRC/ATP1A3/OTOP3/PPP3CA/SLC31A1/CCDC51/PDGFRB/SLC30A9/KCNB1/ITGB1/CDK5/F2RL3/SEC61A1/PHB2/STEAP3/CHRNB2/COX7A1/NKX2-5/SLC25A5/ZMPSTE24/ATP2A3/TSPO/COX8A/ABL1/VDAC1
GOBP_MEIOSIS_I_CELL_CYCLE_PROCESS	GOBP_MEIOSIS_I_CELL_CYCLE_PROCESS	GOBP_MEIOSIS_I_CELL_CYCLE_PROCESS	21	0.545788396	2.720497981	1.61E-05	0.000696298	0.000557524	583	tags=62%, list=18%, signal=51%	CCNB2/PTTG2/PTTG1/STAG3/CKS2/RAD51/MYBL1/CDC25C/HSPA2/PIWIL2/TRIP13/RAD50/ESPL1
GOBP_REGULATION_OF_MULTICELLULAR_ORGANISMAL_DEVELOPMENT	GOBP_REGULATION_OF_MULTICELLULAR_ORGANISMAL_DEVELOPMENT	GOBP_REGULATION_OF_MULTICELLULAR_ORGANISMAL_DEVELOPMENT	268	-0.202034338	-2.14282003	1.64E-05	0.000702385	0.000562398	1460	tags=59%, list=46%, signal=35%	LTA/KLF10/PRMT6/SEMA4B/NCOA3/NOTUM/ASCL1/SLITRK2/TPPP/EXTL3/WNT2B/ZFPM2/KLHL25/MMRN2/SFN/CCM2/RNH1/ENG/GATA4/CYFIP1/CLSTN1/LTF/TDG/CLASP2/TNF/CBFB/EHMT1/IL15/PTPN2/THY1/SYK/CCR1/TRIM16/SULF1/MAFG/AMIGO3/ZNF488/FOXE3/LGI4/WNT11/ANGPTL4/NF1/RARG/INHBA/CLDN5/PLXNB2/PIAS3/SOCS1/CYP1B1/TRAK1/MYOD1/TNFRSF21/GDI1/WNT1/C1QC/SNAI2/CLSTN3/FGL2/WNT3A/ETS1/CXCL13/HSPB1/NFATC4/ADIPOQ/CLCN2/EMP2/CD4/OR10J5/BAD/STIM1/PRKCH/LRRN3/PPARA/ITGB8/HIPK2/MAFB/RXRB/CD34/CITED2/RGS14/ADD1/C3AR1/LOXL2/BTG2/BTN2A2/CDH4/SMAD7/KLF2/ALOX5/XBP1/RELA/HLA-B/VEGFB/TMEM119/OAS2/TCTA/FBXW8/FAM20C/SERPINE2/CEBPB/MYC/YAP1/CUL7/PTCH2/NRP1/HCLS1/COL4A2/BMP4/TEK/GHSR/ISG15/LRG1/SIRT2/TFPT/LYN/STAB1/DAB1/AGER/PITX3/SMO/FZD4/CDH3/PLXNA1/TYROBP/ADAM12/FGF1/LAG3/CEBPA/C

											TSC/PLAG1/ATF4/AKT1/MGP/AMIGO2/PTPRC/VGLL4/IL17A/TFE3/MAF/PPP3CA/GLI2/NFE2L2/ITGB1/CDK5/NAP1L1/PGK1/HMGB1/MMP14/CTSK/NKX2-5/FBN1/STAT3/ZMPSTE24/PTN/GADD45A/TSPO/ABL1
GOBP_NEGATIVE_REGULATION_OF_CELL_CYCLE_PROCESS	GOBP_NEGATIVE_REGULATION_OF_CELL_CYCLE_PROCESS	GOBP_NEGATIVE_REGULATION_OF_CELL_CYCLE_PROCESS	70	0.321652816	2.593147982	2.04E-05	0.00086518	0.000692747	742	tags=50%, list=23%, signal=39%	DONSON/FBXO7/INTS7/MDC1/RAD51/CDC14B/CDCA8/DOT1L/CEP63/CRY1/TPR/BLM/TRIP13/AURKAIP1/RAD17/BRD7/PINX1/CDK5RAP2/MAD2L1BP/TIMELESS/FZR1/DYNC1LI1/BUB1B/RAD50/CHEK2/RBM14/BARD1/ESPL1/THOC5/E2F1/ATR/ZW10/MAD2L2/RINT1/DCUN1D3
GOBP_PROTEIN_RNA_COMPLEX_ORGANIZATION	GOBP_PROTEIN_RNA_COMPLEX_ORGANIZATION	GOBP_PROTEIN_RNA_COMPLEX_ORGANIZATION	29	0.474776805	2.665260671	2.06E-05	0.00086673	0.000693989	369	tags=45%, list=12%, signal=40%	SRPK1/DDX20/BUD13/SETX/GEMIN4/EIF5/ZNHIT3/DHX30/PRPF18/GEMIN6/SF3A1/RUVBL1/TFIP11
GOBP_PERIPHERAL_NERVOUS_SYSTEM_DEVELOPMENT	GOBP_PERIPHERAL_NERVOUS_SYSTEM_DEVELOPMENT	GOBP_PERIPHERAL_NERVOUS_SYSTEM_DEVELOPMENT	21	-0.546711716	-2.51945033	2.12E-05	0.000883449	0.000707376	1132	tags=86%, list=35%, signal=56%	LGI4/NF1/NDRG1/HAND2/ILK/CITED2/NFASC/NHLH2/RELA/SIRT2/CNTNAP1/PRX/NTF3/AKT1/GFRA3/CDK5/MED12/ISL2
GOBP_RESPONSE_TO_LIPID	GOBP_RESPONSE_TO_LIPID	GOBP_RESPONSE_TO_LIPID	181	-0.232923168	-2.217225248	2.21E-05	0.000910809	0.000729282	1324	tags=58%, list=41%, signal=36%	UCN/MAP2K3/GATA4/CYP7A1/CLDN4/LTF/TNF/ATP1A2/LRP6/MGST2/SYK/CTSG/TRIM16/SAFB2/SSTR3/VPS18/BCHE/WNT11/RARG/INHBA/MYOG/FFAR2/BCR/SOCS2/CYP1B1/ELK1/HAND2/MYOD1/BCL2/NQO1/SNAI2/ZNF703/WNT3A/ARPC1B/CXCL13/MDM2/HTR1B/ADIPOQ/LIPA/CCR5/VPS11/BAD/FMO1/CD6/PPARA/GPR83/RXRB/FAM107A/MMP15/CAT/AANAT/KMO/FOSL2/PRDX3/TSHB/XBP1/RELA/CCL21/PYCARD/CEBPB/UCN3/YAP1/GNB1/GNAI1/GHSR/GLB1/TFPI/ALAD/PMVK/DAB2/FECH/LYN/TRIM41/SMO/DDX54/FZD4/IGF2R/LY86/MAOB/GRAMD1A/AKT1/SCGB1A1/SLC11A1/AKR1C1/PRMT2/PTPRC/ATP1A3/ADAM15/MSN/CDK4/IRAK1/HMGB1/NFKB1/PHB2/ZBTB7A/MAPKAPK3/HAVCR2/UBE2L3/NFKBIL1/KLF9/STAT3/TSPO/BC

											L2L2/ABL1/MBD2
GOCC_CILIARY_PLASM	GOCC_CILIARY_PLASM	GOCC_CILIARY_PLASM	19	0.584437655	2.845796934	2.26E-05	0.000926011	0.000741455	504	tags=63%, list=16%, signal=54%	EFHC1/DNALI1/CCDC96/SPAG8/SPATA7/WDR54/DNAH17/DNAH8/ARFGEF2/SSNA1/DNAI1/NME7
GOBP_LEUKOCYTE_PROLIFERATION	GOBP_LEUKOCYTE_PROLIFERATION	GOBP_LEUKOCYTE_PROLIFERATION	66	-0.342439883	-2.443787913	2.40E-05	0.000967341	0.000774547	978	tags=58%, list=31%, signal=41%	BCL2/TNFRSF21/WNT3A/CCL5/LIPA/EMP2/CD6/CD79A/HLA-A/SPN/FOSL2/BTN2A2/HLA-E/PYCARD/CEBPB/TNFRSF4/AIF1/BMP4/BAX/LYN/HLA-DMB/AGER/TYROBP/IGF2/BCL2L1/SCGB1A1/SLC11A1/PTPRC/PPP3CA/MSN/IMPDH2/HMGB1/HAVCR2/CHRNB2/TYK2/PURA/EFNB1/ABL1
GOCC_CELL_SURFACE	GOCC_CELL_SURFACE	GOCC_CELL_SURFACE	177	-0.236013147	-2.237696166	2.40E-05	0.000967341	0.000774547	1789	tags=74%, list=56%, signal=35%	RTN4RL2/CD209/IL2RG/SLC6A1/GPR37/ENO1/ADAMTS7/ADA/CLEC14A/TSPAN8/ABCG2/CD83/RAMP2/ECE1/ADAM17/UMOD/NPTN/TRPV1/TSPAN32/AOC3/TMIGD1/KCNQ3/CX3CR1/UMODL1/PSG2/BMP2/IL21R/ATP1B2/CD1A/ITGA5/CAV3/CHRNA7/IL9R/IGSF21/MICA/ANXA4/PDGFRA/ENG/CLSTN1/BCAM/LTF/CD276/TNF/LRP6/THY1/PROM2/GPRC5B/CCR1/LAYN/BTN1A1/SULF1/KISS1R/BTNL2/CD1B/MPZL1/HLA-C/PLXNB2/MAP3K5/DUOX2/PSG8/SRPX/SLC22A11/TGFA/WNT1/CLSTN3/WNT3A/IL1R1/PLAU/CXCR3/ADIPOQ/EMP2/CD4/CCR5/CD6/COLEC11/HLA-DMA/SDC2/ITGB8/CD79A/HLA-A/CD34/SPN/HLA-F/BTN2A2/HLA-E/HLA-B/ITGAM/FOLR2/TNFRSF4/SLC32A1/TEK/GHSR/TFPI/DCBLD2/LYN/SCARA5/AGER/FZD4/IGF2R/TYROBP/LAG3/XCR1/ANTXR2/GFRA3/RALA/PTPRC/F10/IL17A/LRFN3/ADRA2B/FCER1G/ITGA3/CYP2W1/CD248/KCNB1/ITGB1/ADAM15/HM13/MSN/IRAK1/FLT3LG/HMGB1/PHB2/HAVCR2/ITGA9/CTSK/ITGB7/CHRNB2/ENTPD6/HEG1/EFNB1
GOBP_VASCULATURE_DEVELOPME	GOBP_VASCULATURE_DEVELOPME	GOBP_VASCULATURE_DEVELOPMENT	153	-0.242403253	-2.22960939	2.55E-05	0.0010168 8	0.000814213	1698	tags=71%, list=53%, signal=35%	CLEC14A/CTSH/ADM/RAMP2/VASH1/FOXC1/ROCK2/PRRX1/ATF2/CX

NT	NT										3CR1/BMP2/EGR1/ITGA5/GJC1/CHRNA7/COL8A2/NSDHL/SVEP1/CDX4/ELK3/ZFPM2/MMRN2/CCM2/RNH1/PDGFRA/ENG/GATA4/E2F7/SNX17/TNF/TBX6/COL4A1/THY1/SYK/SULF1/APOD/WNT11/ANGPTL4/NF1/CLDN5/CYP1B1/GPR15/HAND2/TGFA/NRP2/ETS1/CXCL13/HSPB1/MDM2/CXCR3/NFATC4/LIPA/EMP2/OR10J5/STIM1/ITGB8/HIPK2/COL15A1/CD34/CITED2/C3AR1/LOXL2/SMAD7/KLF2/ALOX5/XBP1/VEGFB/FBXW8/TGFBI/YAP1/CUL7/NRP1/COL4A2/BMP4/TEK/GHSR/BAX/LRG1/ANG/STAB1/SMO/ADIPOR2/FZD4/GJA4/ADAM12/FGF1/COL3A1/HAS2/AKT1/COL27A1/HS6ST1/ADRA2B/SLC31A1/NFE2L2/PDGFRB/ITGB1/ADAM15/PGK1/APLN/MMP14/NKX2-5/HEG1/PARVA/STAT3/GADD45A/SHC1/ITGB1BP1/ABL1
GOBP_DOUBLE_STRAND_BREAK_REPAIR	GOBP_DOUBLE_STRAND_BREAK_REPAIR	GOBP_DOUBLE_STRAND_BREAK_REPAIR	56	0.342824317	2.576453096	2.64E-05	0.00104334	0.000835399	1112	tags=68%, list=35%, signal=45%	SMCHD1/SETX/RAD51/POLB/RNF138/RECQL4/BLM/RUVBL1/TRIP13/UBE2N/TFIP11/BRD7/TIMELESS/WRN/CDC7/TOP3A/RAD50/CHEK2/SMC6/ATR/NHEJ1/MAD2L2/RMI1/MCM5/SMARCC1/POT1/VCP/PARP1/TP53BP1/MCM3/RIF1/SIRT1/EP400/TWIST1/ERCC5/DDX11/FIGNL1/XRCC3
GOBP_POSITIVE_REGULATION_OF_CYTOKINE_PRODUCTION	GOBP_POSITIVE_REGULATION_OF_CYTOKINE_PRODUCTION	GOBP_POSITIVE_REGULATION_OF_CYTOKINE_PRODUCTION	96	-0.29882731	-2.400525411	2.78E-05	0.001092258	0.000874568	1345	tags=65%, list=42%, signal=39%	LTB/POU2AF1/UCN/GATA4/TNXB/CD276/TNF/IL15/SYK/GPRC5B/TRIM16/SULF1/WNT11/LUM/FFAR2/CYP1B1/ORM2/WNT3A/IL1R1/HSPB1/UNC93B1/NFATC4/ADIPOQ/CD4/CD6/INS/HLA-A/CD34/SPN/HLA-F/C3AR1/KIR2DL4/HLA-E/XBP1/RELA/OAS2/PYCARD/CEBPB/GPSM3/AIF1/H19/ISG15/AIRE/AGER/RAB1A/TYROBP/PQBP1/ATF4/SLC11A1/PTPRC/IL17A/FCER1G/EIF2AK2/IRAK1/HMGB1/HAVCR2/MEFV/IFNGR1/TYK2/HEG1/STAT3/ABL1

GOBP_REACTIVE_OXYGEN_SPECIES_METABOLIC_PROCESS	GOBP_REACTIVE_OXYGEN_SPECIES_METABOLIC_PROCESS	GOBP_REACTIVE_OXYGEN_SPECIES_METABOLIC_PROCESS	60	-0.357660498	-2.472839517	2.85E-05	0.001110489	0.000889166	1335	tags=72%, list=42%, signal=43%	IER3/FYN/TNF/SYK/MPV17/DCXR/NDUFS1/BCR/DUOX2/CYP1B1/DHRS4/BCL2/NQO1/ABCB7/LIPA/PPARA/INS/PRDX1/CAT/ACOX1/PRDX3/ALOX5/HP/ITGAM/H19/SIRT2/TYROBP/MAOB/CD177/IFI6/AKR1C1/RAC1/NFE2L2/PDGFRB/CBR1/ARF4/SH3PXD2B/ABCD1/CCS/SH3PXD2A/GADD45A/TSPO/VDAC1
GOBP_REGULATION_OF_RESPONSE_TO_EXTERNAL_STIMULUS	GOBP_REGULATION_OF_RESPONSE_TO_EXTERNAL_STIMULUS	GOBP_REGULATION_OF_RESPONSE_TO_EXTERNAL_STIMULUS	195	-0.227427259	-2.22242159	2.99E-05	0.001153428	0.000923547	983	tags=46%, list=31%, signal=34%	MYOD1/TRAFD1/BCL2/SNAI2/FGL2/WNT3A/IL1R1/ETS1/CXCL13/PLAU/HSPB1/UNC93B1/ADIPOQ/CCL5/PPARA/COLEC11/INS/HLA-A/ABHD12/RXRB/SPN/HLA-F/C3AR1/KIR2DL4/ALOX5/HLA-E/OTOP1/RELA/CCL21/HLA-B/VEGFB/PYCARD/SERPINE2/CEBPB/PLSCR1/GPSM3/PUM1/NRP1/AIF1/RHBDF2/TEK/GHSR/TFPI/BAX/ISG15/CLDN3/SIRT2/LYN/TRIM41/AGER/ZNFX1/FLOT2/TYROBP/NTF3/FGF1/LAG3/LY86/CEBPA/RNF185/PQBP1/CTSC/AKT1/SCGB1A1/GRK1/RAC1/PTPRC/IL17A/CASP4/NFE2L2/PDGFRB/EIF2AK2/ALOX5AP/ST3GAL4/IRAK1/HMGB1/NFKB1/PHB2/MAPKAPK3/HAVCR2/MEFV/KREMEN1/NFKBIL1/STAT3/ZMPSTE24/FGFR1/EIF2AK4/PTN/PTGES/LSM14A
GOBP_CELLULAR_RESPONSE_TO_OXYGEN_CONTAINING_COMPOUND	GOBP_CELLULAR_RESPONSE_TO_OXYGEN_CONTAINING_COMPOUND	GOBP_CELLULAR_RESPONSE_TO_OXYGEN_CONTAINING_COMPOUND	248	-0.210605871	-2.200620672	3.20E-05	0.001225836	0.000981524	1331	tags=55%, list=42%, signal=35%	SRI/MAP2K3/PDGFRA/DLG4/GATA4/FYN/CYFIP1/CYP7A1/LTF/TNF/ANKZF1/PTPN2/LRP6/COL4A1/CTSG/MPV17/CYP11A1/SSTR3/JUND/WNT11/SQSTM1/CSHL1/RARG/ACTN2/INHBA/GPR21/COL4A6/ARRB2/FXN/MYOG/FFAR2/BCR/GCLC/MAP3K5/SOCS2/SOCS1/CYP1B1/ELK1/HAND2/MYOD1/NQO1/WNT1/SNAI2/ZNF703/WNT3A/CXCL13/MDM2/NFATC4/HTR1B/ADIPOQ/PDK2/CCR5/BAD/CD6/INS/RXRB/ADD1/PRDX1/CAT/AANAT/KMO/PRDX3/KLF2/NET1/OTOP1/XBP1/RELA/GRB7/FOLR2/PYC

											ARD/CEBPB/YAP1/CPNE3/GNB1/GNAI1/UBTF/H19/GHSR/ITPR1/IQGAP1/CRHR2/HRH3/SYT12/SIRT2/FECH/RAPGEF1/LYN/TRIM41/AGER/SMO/FZD4/SLC25A23/RGS10/LY86/CEBPA/OSBP/TRPM2/GRAMD1A/COL3A1/IGF2/BCL2L1/AKT1/PPIF/CSK/AKR1C1/ATP1A3/PPP3CA/SLC29A1/CASP4/MAS1L/NFE2L2/AKR1A1/KCNB1/CDK5/ADAM15/MSN/ALOX5AP/CDK4/IRAK1/HMGB1/NFKB1/PHB2/HAVCR2/ADIPOR1/NFKBIL1/CHRNB2/DENND4C/KLF9/TYK2/CCS/FBN1/STAT3/TSPO/NADK/SHC1/BCL2L2/ABL1
GOBP_RNA_3_END_PROCESSING	GOBP_RNA_3_END_PROCESSING	GOBP_RNA_3_END_PROCESSING	19	0.576775943	2.808489831	3.27E-05	0.001240448	0.000993224	822	tags=79%, list=26%, signal=59%	NCBP2/INTS7/CPSF3/ZNF473/PNPT1/EXOSC8/CSTF1/PAF1/INTS6/BARD1/EXOSC4/CPSF2/WDR33/CSTF3/CPSF1
GOBP_SMALL_MOLECULE_METABOLIC_PROCESS	GOBP_SMALL_MOLECULE_METABOLIC_PROCESS	GOBP_SMALL_MOLECULE_METABOLIC_PROCESS	384	-0.176660271	-2.005054208	3.29E-05	0.001240448	0.000993224	1309	tags=50%, list=41%, signal=34%	MECR/NMNAT3/STARD4/FUT6/ALDH1L1/SNX17/CYP7A1/TNXB/PLA2G4D/CYP2A13/MCCC1/TDG/TNF/ATP1A2/CLN8/NME6/PTPN2/CSAD/DUSP12/MGST2/SYK/PLTP/NUDT8/ACSL5/DCXR/PGD/APOD/NDUFS1/CYP11A1/PNPLA4/CA11/GDA/FADS6/TMEM86B/RDH5/NEIL1/MTHFD1/EXTL2/NR1H2/GCLC/ATP8B1/CYP1B1/ATP1B1/DHRS4/GALM/SLC22A11/ATCAY/DERA/NQO1/AMN/SNAI2/CYP2S1/PSAT1/ASNSD1/DAO/LDHA/CYP2U1/IDH3B/ABHD6/NDUFB7/SEC14L2/KCNAB2/ELOVL1/ADIPOQ/GNPDA1/CLCN2/PDK2/LIPA/BAD/FMO1/OAT/PYCR2/DHCR24/PPARA/INS/BPHL/ABHD12/ABCD4/RBP1/HSD17B1/MAN2B2/CAT/KMO/ECHDC1/ACOX1/NANS/COQ10A/GCSH/ICMT/ALOX5/NTHL1/ALDH4A1/XBP1/PGM2/ABCC10/SORD/IGFBP4/PLA2G4C/GLUD2/PYCARD/CA14/GSTO1/PPT2/ALDH2/INPPL1/SLC25A12/H19/GHS

											R/GLB1/PIPOX/CLYBL/PMVK/SIRT2/DAB2/ENTPD2/FECH/GNE/ADI1/ADIPOR2/SLC16A1/HSD17B8/FGF1/CEBPA/ECHS1/OSBP/PRKAB2/IGF2/HYI/AKR7A2/ATF4/SDSL/AKT1/SCP2/PTGES2/ACY1/PRPS1/CLN3/UGT2A3/AKR1C1/ITPA/NFE2L1/APRT/SFXN3/CYP4B1/CYP2W1/SUCLG2/ADHFE1/AKR1A1/GALT/ALDH1A3/CBR1/GOT2/ACAA1/IMPDH2/PGK1/MBTPS1/ALOX5AP/ABCD1/IDH3G/NFKB1/ZBTB7A/ADIPOR1/TFF3/ENTPD8/NUDT16/UCKL1/OSBPL5/ENTPD6/ILVBL/NME4/STAT3/ZMPSTE24/AIG1/FUCA2/DCTD/ECH1/LTC4S/PTGES/BCAT2/TSPO/ITPK1/NADK/PGLS/ACO1/LARS2/DHTKD1/OSBPL1A/NT5C/GTPBP1/ACOT9/HMGCL/PDXK/VDAC1
GOBP_MRNA_PROCESSING	GOBP_MRNA_PROCESSING	GOBP_MRNA_PROCESSING	85	0.285820404	2.439124036	3.34E-05	0.001252148	0.001002592	961	tags=55%, list=30%, signal=40%	SRPK1/DDX20/BUD13/KHDRBS3/PRKRIP1/DAZAP1/NCBP2/DHX16/SETX/GEMIN4/CPSF3/ZNF473/PRPF18/GEMIN6/PNPT1/PRPF40B/SF3A1/CSTF1/TFIP11/IWS1/PAF1/ZRANB2/PRPF38A/CPEB1/ELAVL4/RBM14/DUS3L/BARD1/THOC5/CPSF2/LSM3/PRPF40A/AKAP8L/WDR33/PPWD1/CSTF3/CPSF1/ECD/SAFB/RBBP6/DHX35/PLRG1/PRPF6/CTNNBL1/DHX15/LUC7L/RNMT
GOBP_RECOMBINATIONAL_REPAIR	GOBP_RECOMBINATIONAL_REPAIR	GOBP_RECOMBINATIONAL_REPAIR	29	0.464530861	2.607742881	3.45E-05	0.001282158	0.00102662	1112	tags=79%, list=35%, signal=52%	SMCHD1/RAD51/RNF138/RECQL4/BLM/RUVBL1/UBE2N/TIMELESS/WRN/CDC7/TOP3A/SMC6/MAD2L2/RMI1/MCM5/PARP1/TP53BP1/MCM3/RIF1/EP400/ERCC5/FIGNL1/XRCC3
GOCC_MHC_PROTEIN_COMPLEX	GOCC_MHC_PROTEIN_COMPLEX	GOCC_MHC_PROTEIN_COMPLEX	11	-0.677843936	-2.450741793	4.03E-05	0.001486668	0.001190372	1040	tags=100%, list=32%, signal=68%	HLA-DMA/HLA-A/HLA-F/HLA-E/HLA-B/HLA-DOB/HLA-DRB5/HLA-DMB/HLA-DQB1/HLA-DRB3
GOCC_VACUOLAR_LUMEN	GOCC_VACUOLAR_LUMEN	GOCC_VACUOLAR_LUMEN	38	-0.418913849	-2.498357737	4.62E-05	0.001690408	0.001353505	928	tags=61%, list=29%, signal=44%	ORM2/LIPA/OGN/SDC2/MANBA/MAN2B2/PYCARD/PPT2/GLB1/GALC/CTSC/PTGES2/PDGFRB/SCARB2/CTSK/CAP1/PRELP/PYGB/GDI2/TRAPP

											C1/TXNDC5/FUCA2/SGSH
GOBP_MRNA_METABOLIC_PROCESS	GOBP_MRNA_METABOLIC_PROCESS	GOBP_MRNA_METABOLIC_PROCESS	137	0.229083659	2.217406305	4.66E-05	0.001691901	0.001354701	961	tags=50%, list=30%, signal=36%	NR1H3/SRPK1/DDX20/CNOT10/BUD13/KHDRBS3/PRKRIP1/DAZAP1/NCBP2/DHX16/SETX/SECISBP2/GEMIN4/CPSF3/ZNF473/PRPF18/GEMIN6/PNPT1/EXOSC8/EDC3/PRPF40B/SF3A1/CSTF1/TFIP11/IWS1/PAF1/ZRANB2/PRPF38A/MAPKAPK2/TAF6/CPEB1/ELAVL4/CNOT7/CEBPG/EDC4/TAF10/RBM14/DUS3L/BARD1/TRAF2/BOLL/THOC5/E2F1/EXOSC4/CPSF2/CNOT6/LSM3/PRPF40A/AKAP8L/WDR33/TAF9/PPWD1/DNAJB11/CSTF3/CPSF1/ECD/PAIP1/SMG7/SAFB/RBBP6/FASTKD1/DHX35/PLRG1/PRPF6/CTNNBL1/DHX15/LUC7L/RNMT
GOBP_MITOTIC_CELL_CYCLE	GOBP_MITOTIC_CELL_CYCLE	GOBP_MITOTIC_CELL_CYCLE	200	0.203319535	2.172360118	4.72E-05	0.001700264	0.001361397	742	tags=37%, list=23%, signal=30%	ASNS/GMNN/EFHC1/SPAG5/DONSON/KLF11/CCNB2/C1orf112/FBXO7/CCNH/MDC1/CKS2/CPSF3/CENPE/RAD51/CDC14B/PAFAH1B1/PCNA/WDR62/MYBL1/CDCA8/RANBP1/KATNB1/CDC25C/RAE1/NDE1/AURKC/CENPH/HSPA2/TPR/SMC3/ANAPC10/CEP250/BLM/PPP6C/TRIP13/AURKAIP1/RAD17/BRD7/PINX1/KIF23/CDK5RAP2/VRK1/USP37/MYH10/MAD2L1BP/FZR1/DYNC1LI1/TUBG1/BUB1B/BBS4/CDC7/TFDP1/TAF10/RAD50/UBE2S/CHEK2/KIF4A/BARD1/POLE/ESPL1/KPNB1/TPX2/TUBG2/E2F1/ERCC3/ZW10/CDK7/MAD2L2/RINT1/KIF22/AKAP8L/USP8/DCUN1D3
GOCC_CONDENSED_CHROMOSOME_CENTROMERIC_REGION	GOCC_CONDENSED_CHROMOSOME_CENTROMERIC_REGION	GOCC_CONDENSED_CHROMOSOME_CENTROMERIC_REGION	35	0.431045762	2.639143421	5.26E-05	0.00186742	0.001495239	505	tags=49%, list=16%, signal=41%	SPAG5/C1orf112/SUGT1/CENPE/CENPM/AHCTF1/PAFAH1B1/NDE1/AURKC/CENPH/ITGB3BP/TPR/PPP1CC/BRD7/PINX1/DYNC1LI1/BUB1B
GOBP_REGULATION_OF_T_CELL_ACTIVATION	GOBP_REGULATION_OF_T_CELL_ACTIVATION	GOBP_REGULATION_OF_T_CELL_ACTIVATION	73	-0.317117305	-2.358890344	5.24E-05	0.00186742	0.001495239	1291	tags=66%, list=40%, signal=40%	FYN/CD276/CBFB/IL15/PTPN2/THY1/SYK/CTSG/SOCS1/TNFRSF21/FGL2/CCL5/CD4/BAD/CD6/HLA-DMA/HLA-A/SPN/BTN2A2/SMAD7/HLA-E/XBP1/CCL21/PYCARD/CEBPB/AIF1/BMP4/HLA-DOB/HLA-DRB5/LYN/HL

											A-DMB/AGER/FLOT2/LAG3/HLA-DQB1/IGF2/AKT1/SCGB1A1/CSK/PTPRC/PPP3CA/HLA-DRB3/GLI2/HMGB1/HAVCR2/TYK2/EFNB1/ABL1
HP_REDUCED_SPERM_MOTILITY	HP_REDUCED_SPERM_MOTILITY	HP_REDUCED_SPERM_MOTILITY	11	0.683568059	2.591672432	5.85E-05	0.002017759	0.001615614	284	tags=64%, list=9%, signal=58%	TTC21A/DNALI1/CATSPER2/ZMYND10/KLHL10/DNAH17/DNAH8
GOBP_MRNA_3_END_PROCESSING	GOBP_MRNA_3_END_PROCESSING	GOBP_MRNA_3_END_PROCESSING	11	0.683225623	2.590374122	5.85E-05	0.002017759	0.001615614	822	tags=91%, list=26%, signal=68%	NCBP2/CPSF3/ZNF473/CSTF1/PAF1/BARD1/CPSF2/WDR33/CSTF3/CPSF1
GOCC_MALE_GERM_CELL_NUCLEUS	GOCC_MALE_GERM_CELL_NUCLEUS	GOCC_MALE_GERM_CELL_NUCLEUS	18	0.552622261	2.565446613	5.78E-05	0.002017759	0.001615614	330	tags=56%, list=10%, signal=50%	TBPL1/TSN/TCFL5/DAZAP1/SPAG8/RAD51/PCNA/ACTL7A/HSPA2/TRIP13
GOBP_DNA_RECOMBINATION	GOBP_DNA_RECOMBINATION	GOBP_DNA_RECOMBINATION	51	0.350200287	2.50724413	5.89E-05	0.002017759	0.001615614	915	tags=59%, list=29%, signal=43%	KPNA2/TSN/SMCHD1/SETX/RAD51/POLB/RNF138/RECQL4/PAXIP1/BLM/RUVBL1/TRIP13/UBE2N/TIMELESS/WRN/CDC7/TOP3A/RAD50/BARD1/SMC6/NHEJ1/MAD2L2/RMI1/ENDOG/MCM5/PARP1/TP53BP1/MCM3/HMGB3/RIF1
GOBP_SMOOTH_MUSCLE_CELL_MIGRATION	GOBP_SMOOTH_MUSCLE_CELL_MIGRATION	GOBP_SMOOTH_MUSCLE_CELL_MIGRATION	20	-0.542508935	-2.492117191	5.78E-05	0.002017759	0.001615614	1082	tags=80%, list=34%, signal=53%	NF1/CYP1B1/BCL2/PLAU/MDM2/ADIPOQ/CCL5/NRP1/AIF1/BMP4/PLXNA1/HAS2/GNA12/NFE2L2/PDGFRB/PARVA
GOBP_CELL_SUBSTRATE_ADHESION	GOBP_CELL_SUBSTRATE_ADHESION	GOBP_CELL_SUBSTRATE_ADHESION	84	-0.306365681	-2.364427552	5.97E-05	0.002030626	0.001625917	1280	tags=63%, list=40%, signal=39%	BCAM/TNXB/CLASP2/THY1/PARVB/APOD/NF1/ACTN2/AGR2/BCR/TRIOBP/VWF/BCL2/ABI3BP/WNT1/TSC1/PLAU/EMP2/ILK/ITGB8/PARVG/FAM107A/CD34/CORO2B/MINK1/CCL21/ITGAM/MYADM/NRP1/TEK/EDA/PKP2/DAB2/CDH11/FZD4/RAB1A/RSU1/COL3A1/HAS2/RAC1/ARHGEF7/CTTN/ITGA3/ITGB1/CDK5/ADAM15/MMP14/ITGA9/ITGB7/PARVA/TRIP6/ITGB1BP1/ABL1
GOBP_REGULATION_OF_CELL_CYCLE_PHASE_TRANSITION	GOBP_REGULATION_OF_CELL_CYCLE_PHASE_TRANSITION	GOBP_REGULATION_OF_CELL_CYCLE_PHASE_TRANSITION	104	0.260034959	2.358804711	6.10E-05	0.002059302	0.001648878	711	tags=41%, list=22%, signal=33%	DONSON/FBXO7/CCNH/INTS7/MDC1/CPSF3/CENPE/RAD51/CDC14B/CDCA8/DOT1L/CDC25C/CEP63/CRY1/HSPA2/TPR/PAXIP1/BLM/TRIP13/RAD17/BRD7/PAF1/PINX1/CDK5RAP2/MAD2L1BP/TIMELESS/FZR1/DYNC1LI1/BUB1B/CDC7/TFDP1/RAD50/CHEK2/BARD1/ESPL1/THOC5/E2F1/ATR/ERCC3/ZW10/CDK7/MAD2L2/RI

											NT1
HP_ABNORMALITY_OF_SKIN_ADNEXA_MORPHOLOGY	HP_ABNORMALITY_OF_SKIN_ADNEXA_MORPHOLOGY	HP_ABNORMALITY_OF_SKIN_ADNEXA_MORPHOLOGY	293	-0.190760124	-2.067518458	6.24E-05	0.002092158	0.001675186	614	tags=30%, list=19%, signal=27%	INPPL1/B4GALT7/CUL7/HR/SLC25A12/SLC32A1/BMP4/EDA/GLB1/SLURP1/HTRA1/ZNF462/HEATR3/CDH11/GNE/AIRE/SLC29A3/KRT85/SMO/COX7B/PNKP/CDH3/SLC6A17/PLXNA1/TRIO/ANTXR2/COL3A1/PROKR2/ANKRD11/STT3A/HLA-DQB1/PQBP1/IGF2/CTSC/PLAG1/AKT1/RALA/MGP/PRPS1/CLN3/PAX9/RAC1/ATP1A3/HS6ST1/PERP/TFE3/MAF/CDC42BPB/ITGA3/PPP2R1A/GLI2/PRR12/PDGFRB/MID1/GPRASP2/STUB1/AP2M1/SLC30A9/HPS1/CDK5/MED12/ABCD1/SETBP1/CDK4/IRAK1/MMP23B/NFKB1/MMP14/KREMEN1/CTSK/UBE2L3/SUMF1/TRIM8/WDR81/CCDC8/GMPPA/FBN1/PURA/HPS6/STAT3/ZMPSTE24/DPH5/FGFR1/KRT14/SGSH/RNF113A/CTBP1/EFNB1/ABL1
GOBP_HEMOPOIESIS	GOBP_HEMOPOIESIS	GOBP_HEMOPOIESIS	176	-0.228162381	-2.159831515	6.32E-05	0.002105384	0.001685776	949	tags=44%, list=30%, signal=33%	WNT1/C1QC/SNAI2/FGL2/TSC1/WNT3A/ETS1/ADIPOQ/LIPA/CD4/BAD/ITGB8/SSBP3/CD79A/HIPK2/MAFB/CD34/CITED2/SPN/MEN1/FOSL2/BTN2A2/PRDX3/SMAD7/KLF2/XBP1/HLA-B/MKNK2/TCTA/FAM20C/CEBPB/MYC/HCLS1/BMP4/TEK/EOMES/BAX/ISG15/HEATR3/FECH/LYN/LFNG/AIRE/MIXL1/HERC6/AGER/TYROBP/LAG3/CEBPA/TRPM2/ATF4/PTPRC/LTBR/IL17A/TFE3/MAF/FCER1G/PPP3CA/GLI2/NFE2L2/ITGB1/EIF2AK2/FLT3LG/HMGB1/ZBTB7A/MMP14/CTSK/NKX2-5/SLC25A5/FBN1/STAT3/SH3PXD2A/PTN/WDR7/DHTKD1/ABL1/EIF2AK1
GOBP_MEIOTIC_CELL_CYCLE_PROCESS	GOBP_MEIOTIC_CELL_CYCLE_PROCESS	GOBP_MEIOTIC_CELL_CYCLE_PROCESS	33	0.427281265	2.554192702	6.39E-05	0.00211403	0.00169698	706	tags=55%, list=22%, signal=43%	CCNB2/PTTG2/PTTG1/STAG3/CKS2/RAD51/MYBL1/CDC25C/HSPA2/PIWIL2/TRIP13/TDRKH/FZR1/TUBG1/RAD50/ESPL1/TUBG2/RMI1
GOBP_FERTILIZATION	GOBP_FERTILIZATION	GOBP_FERTILIZATION	28	0.462078384	2.592962061	6.70E-05	0.00220208	0.00176399	477	tags=50%, list=15%, signal=43%	SPA17/HSPA1L/SPAG8/AKAP4/SPACA3/CATSPER2/IQCF1/ZPBP2/ACTL

											7A/ACRBP/KLHL10/TDRKH/LYZL6/UBE3A
GOMF_CYTOSKELETAL_MOTOR_ACTIVITY	GOMF_CYTOSKELETAL_MOTOR_ACTIVITY	GOMF_CYTOSKELETAL_MOTOR_ACTIVITY	18	0.548720852	2.547335039	6.81E-05	0.002223101	0.001780031	565	tags=67%, list=18%, signal=55%	KIF3A/DYNLRB2/CENPE/DNAH17/MYO9A/DNAH8/SMC3/KIF23/MYH10/DNAI1/KIFC3/KIF4A
GOMF_CATALYTIC_ACTIVITY_ACTING_ON_A_NUCLEIC_ACID	GOMF_CATALYTIC_ACTIVITY_ACTING_ON_A_NUCLEIC_ACID	GOMF_CATALYTIC_ACTIVITY_ACTING_ON_A_NUCLEIC_ACID	109	0.249069435	2.269200682	6.90E-05	0.002236051	0.0017904	779	tags=41%, list=24%, signal=32%	DDX20/RFC4/NUDT1/POLG2/DHX16/SETX/CPSF3/RAD51/PCNA/DHX30/POLB/TOP1MT/CRY1/PNPT1/RECQL4/EXOSC8/PIWIL2/PRIM1/SMC3/BLM/RUVBL1/POLRMT/TRPT1/NUDT4/RAD17/METTL6/WRN/DDX52/CHD1L/CNOT7/DUS1L/TOP3A/RAD50/DUS3L/POLE/EXOSC4/ERCC3/RFC1/CDK7/CNOT6/CHD5/MBD4/ENDOG/MCM5/TRIT1
GOMF_CATALYTIC_ACTIVITY_ACTING_ON_DNA	GOMF_CATALYTIC_ACTIVITY_ACTING_ON_DNA	GOMF_CATALYTIC_ACTIVITY_ACTING_ON_DNA	50	0.354213532	2.523492268	6.97E-05	0.002244686	0.001797314	776	tags=52%, list=24%, signal=40%	RFC4/POLG2/SETX/RAD51/PCNA/DHX30/POLB/TOP1MT/CRY1/RECQL4/SMC3/BLM/RUVBL1/RAD17/WRN/CHD1L/TOP3A/RAD50/POLE/ERCC3/RFC1/CDK7/CHD5/MBD4/ENDOG/MCM5
GOCC_RNA_POLYMERASE_COMPLEX	GOCC_RNA_POLYMERASE_COMPLEX	GOCC_RNA_POLYMERASE_COMPLEX	26	0.470745614	2.573313111	7.07E-05	0.002262232	0.001811363	950	tags=73%, list=30%, signal=52%	TBPL1/CCNH/PRIM1/POLRMT/SUPT3H/POLA2/PAF1/TAF6/TAF10/GTF2H2/GTF2H1/POLR3E/ERCC3/CDK7/GTF2E1/TAF9/MCM3/ENY2/POLR1C
GOBP_REGULATION_OF_HEMOPOIESIS	GOBP_REGULATION_OF_HEMOPOIESIS	GOBP_REGULATION_OF_HEMOPOIESIS	71	-0.310899556	-2.26714922	7.33E-05	0.002322057	0.001859265	1259	tags=65%, list=39%, signal=40%	LTF/TNF/CBFB/IL15/PTPN2/SYK/CCR1/NF1/RARG/INHBA/PIAS3/SOCS1/C1QC/SNAI2/FGL2/ETS1/ADIPOQ/CD4/BAD/MAFB/BTN2A2/SMAD7/XBP1/HLA-B/TCTA/CEBPB/MYC/HCLS1/BMP4/ISG15/LYN/AGER/TYROBP/LAG3/CEBPA/PTPRC/IL17A/TFE3/PPP3CA/GLI2/HMGB1/MMP14/FBN1/STAT3/PTN/ABL1
GOBP_RNA_PROCESSING	GOBP_RNA_PROCESSING	GOBP_RNA_PROCESSING	172	0.213384725	2.202633171	7.36E-05	0.002322057	0.001859265	961	tags=48%, list=30%, signal=36%	TDRD7/SRPK1/DDX20/TSN/PWP1/BUD13/KHDRBS3/PRKRIP1/DAZAP1/NCBP2/INTS7/NUP155/DHX16/SETX/GEMIN4/CPSF3/FKBP6/ZNHIT3/ZNF473/EBNA1BP2/PRPF18/GEMIN6/PNPT1/EXOSC8/PRPF40B/SF3A1/PIWIL2/TRPT1/CSTF1/SUPT3H/GTPBP4/TDRKH/TFIP11/TPRKB/IWS1/UTP18/S

											MAD2/PAF1/ZRANB2/INTS6/PRPF38A/METTL6/CPEB1/ELAVL4/PDCD7/DDX52/DUS1L/TAF10/PA2G4/RBM14/DUS3L/BARD1/BOP1/THOC5/EXOSC4/CPSF2/NOL6/LSM3/PRPF40A/DNTTIP2/AKAP8L/WDR33/TAF9/THADA/TRIT1/PPWD1/CSTF3/CPSF1/ECD/SAFB/RBBP6/FASTKD1/POP4/YRDC/DHX35/PLRG1/PRPF6/ENY2/CTNNBL1/DDX49/DHX15/LUC7L/RNMT
GOBP_TAXIS	GOBP_TAXIS	GOBP_TAXIS	73	-0.31240688	-2.323851654	7.62E-05	0.002388529	0.001912489	1226	tags=63%, list=38%, signal=40%	PTPN2/SYK/CCL22/CCR1/CTSG/STX3/ARRB2/FFAR2/ELMO2/WNT3A/NRP2/CXCL13/PLAU/HSPB1/CXCR3/CCL5/CCR5/OR10J5/SPN/C3AR1/ALOX5/CCL21/VEGFB/RHOG/FOLR2/GPSM3/S100A4/NRP1/AIF1/BMP4/LYN/AGER/NTF3/FGF1/XCR1/TRPM2/RALA/LSP1/RAC1/FCER1G/PDGFRB/HMGB1/ITGA9/PARVA/FGFR1/PTN
GOMF_ATP_DEPENDENT_ACTIVITY_ACTING_ON_DNA	GOMF_ATP_DEPENDENT_ACTIVITY_ACTING_ON_DNA	GOMF_ATP_DEPENDENT_ACTIVITY_ACTING_ON_DNA	26	0.467526265	2.555714666	7.87E-05	0.002451006	0.001962514	776	tags=62%, list=24%, signal=47%	RFC4/SETX/RAD51/DHX30/RECQL4/BLM/RUVBL1/RAD17/WRN/CHD1L/RAD50/ERCC3/RFC1/CDK7/CHD5/MCM5
GOBP_TELOMERE_ORGANIZATION	GOBP_TELOMERE_ORGANIZATION	GOBP_TELOMERE_ORGANIZATION	31	0.429382855	2.498567338	7.92E-05	0.002452061	0.001963359	824	tags=65%, list=26%, signal=48%	RAD51/PCNA/DOT1L/RECQL4/BLM/RUVBL1/TFIP11/MAPK3/PINX1/WRN/RAD50/SMC6/YLPM1/ATR/RFC1/MAD2L2/POT1/PARP1/MAP3K4/HAT1
GOBP_MITOTIC_CELL_CYCLE_PROCESS	GOBP_MITOTIC_CELL_CYCLE_PROCESS	GOBP_MITOTIC_CELL_CYCLE_PROCESS	181	0.205267844	2.133563366	8.36E-05	0.002571019	0.002058608	742	tags=38%, list=23%, signal=31%	EFHC1/SPAG5/DONSON/KLF11/CCNB2/FBXO7/CCNH/MDC1/CKS2/CPSF3/CENPE/RAD51/CDC14B/PAFAH1B1/PCNA/WDR62/CDCA8/RANBP1/KATNB1/CDC25C/RAE1/NDE1/AURKC/CENPH/HSPA2/TPR/SMC3/BLM/PPP6C/TRIP13/AURKAIP1/RAD17/BRD7/PINX1/KIF23/CDK5RAP2/VRK1/USP37/MYH10/MAD2L1BP/FZR1/DYNC1LI1/TUBG1/BUB1B/BBS4/CDC7/TFDP1/TAF10/RAD50/UBE2S/CHEK2/KIF4A/BARD1/POLE/ESPL1/KPNB1/TPX2/TUBG2/E2F1/ERCC3/ZW10/

											CDK7/MAD2L2/RINT1/KIF22/AKAP8L/USP8/DCUN1D3
GOCC_SIDE_OF_MEMBRANE	GOCC_SIDE_OF_MEMBRANE	GOCC_SIDE_OF_MEMBRANE	148	-0.238328817	-2.174021948	8.54E-05	0.002610433	0.002090167	1844	tags=77%, list=58%, signal=34%	GNA11/BCAP31/MCAM/SLC4A1/PTPN7/RTN4RL2/CD209/IL2RG/RASA3/CD52/ADA/CLEC14A/ABCG2/CD83/TREH/ECE1/BDH1/UMOD/TRPV1/LSAMP/CFP/CNR2/CX3CR1/UMODL1/IL21R/ATP1B2/CD1A/ITGA5/CHRNA7/PTPN1/TRAF5/IL9R/PALM/IGSF21/MICA/PDGFRA/DLG4/ENG/FYN/BCAM/CD276/TNF/CYLD/THY1/SYK/CCR1/BTN1A1/EFNA2/CHMP4C/BTNL2/CD1B/HLA-C/MAP3K5/FKBP1A/OMG/SAMD10/SLC22A11/PRMT8/IL1R1/PLAU/ACP1/CXCR3/KCNAB2/LYNX1/CD4/CCR5/COLEC11/CD79A/HLA-A/CD34/SPN/HLA-F/BTN2A2/HLA-E/HLA-B/ITGAM/FOLR2/PTP4A1/MMP17/GNB1/GNAI1/TNFRSF4/TFPI/IQGAP1/HLA-DRB5/LYN/LAG3/XCR1/CD177/PLEKHA4/HLA-DQB1/GFRA3/CHMP7/PTPRC/GNA12/F10/RGMB/IL17A/FCER1G/PPP3CA/HLA-DRB3/ITGA3/CD248/AP2M1/ITGB1/GNG7/HM13/TRAF1/ITGA9/CTSK/CHRNB2/TYK2/BLOC1S1/HEG1
GOMF_EXTRACELLULAR_MATRIX_STRUCTURAL_CONSTITUENT	GOMF_EXTRACELLULAR_MATRIX_STRUCTURAL_CONSTITUENT	GOMF_EXTRACELLULAR_MATRIX_STRUCTURAL_CONSTITUENT	37	-0.411353441	-2.44037479	8.93E-05	0.002711918	0.002171426	1272	tags=76%, list=40%, signal=46%	TNXB/COL4A1/LUM/COL4A6/SRPX/VWF/ABI3BP/IMPG2/FGL2/ADIPOQ/CD4/OGN/COL15A1/ELN/COL4A5/EMILIN3/VWA1/TGFBI/COL4A2/COL6A3/PCOLCE/PODN/COL3A1/MGP/COL27A1/FBN3/PRELP/FBN1
GOBP_RESPONSE_TO_WOUNDING	GOBP_RESPONSE_TO_WOUNDING	GOBP_RESPONSE_TO_WOUNDING	124	-0.255359426	-2.192796018	9.05E-05	0.002733216	0.002188479	1646	tags=69%, list=51%, signal=35%	ADAM17/DGKG/VASH1/CHMP2B/TSPAN32/KLKB1/CX3CR1/MTR/PHLDB2/ITGA5/CAV3/WAS/ELK3/MERTK/EXTL3/PDGFRA/GATA4/CLDN4/CLASP2/TNF/SYK/CCR1/CTSG/APOD/CHMP4C/NF1/INHBA/DUOX2/VWF/BCL2/WNT1/WNT3A/PLAU/HSPB1/PPARA/SPRR3/ILK/INS/CHMP6/CD34/TIMP1/ALOX5/XBP1/SERPINE2/PLSCR1/YAP1/NRP1/VPS4A/TFPI/SERPI

											NA1/BAX/LRG1/DCBLD2/CLDN3/ENTPD2/LYN/AGER/ADIPOR2/ANXA6/TYROBP/FGF1/COL3A1/SLC11A1/CHMP7/RAC1/GNA12/F10/IL17A/ADRA2B/FCER1G/PPP3CA/NFE2L2/NFIA/KCNB1/ITGB1/F2RL3/ST3GAL4/CLIC1/RHOC/TOR1A/KREMEN1/HPS6/STAT3/PTN/TSPO/ITPK1
GOBP_CALCIUM_ION_HOMEOSTASIS	GOBP_CALCIUM_ION_HOMEOSTASIS	GOBP_CALCIUM_ION_HOMEOSTASIS	67	-0.325911235	-2.343206972	9.16E-05	0.002749236	0.002201306	1935	tags=91%, list=60%, signal=37%	BDKRB1/CACNA1C/GP1BB/VAPB/GRIN1/BCAP31/CDH23/S100A14/HCRTR1/RASA3/BOK/ATG5/UMOD/NPTN/TRPV1/GPR3/FIS1/TRPV5/CAV3/CHRNA7/CCL8/SRI/SYPL2/DIAPH1/ATP1A2/THY1/CCR1/ATP2A1/SV2A/GPR12/ATP2B3/ATP7B/FKBP1A/ATP1B1/BCL2/HTR1B/CCL5/IBTK/CCR5/STIM1/TPCN1/ATP13A1/CCL21/GSTO1/WFS1/ITPR1/BAX/CALM3/LYN/LIME1/SLC25A23/ANXA6/GRM1/XCR1/TRPM2/ATF4/CLN3/PTPRC/F2RL3/ATP2A3/ABL1
GOMF_INTEGRIN_BINDING	GOMF_INTEGRIN_BINDING	GOMF_INTEGRIN_BINDING	37	-0.410744012	-2.436759318	9.30E-05	0.002773181	0.002220479	1272	tags=76%, list=40%, signal=46%	TNXB/PTPN2/THY1/SYK/ANXA7/ACTN2/VWF/ADAMTS5/EMP2/ITGB8/ITGAM/TGFBI/ISG15/LYN/FGF1/CD177/COL3A1/IGF2/ITGA3/ITGB1/ADAM15/HMGB1/MMP14/ITGA9/ITGB7/FBN1/PTN/ITGB1BP1
GOBP_REGULATION_OF_INFLAMMATORY_RESPONSE	GOBP_REGULATION_OF_INFLAMMATORY_RESPONSE	GOBP_REGULATION_OF_INFLAMMATORY_RESPONSE	60	-0.341619916	-2.361936067	9.46E-05	0.002802243	0.002243748	1520	tags=78%, list=47%, signal=42%	MFHAS1/S100A12/CHRNA7/LTA/EXTL3/IER3/FYN/TNF/CYLD/IL15/PTPN2/MGST2/SYK/GPRC5B/NMI/FFAR2/BCR/IL1R1/ETS1/ADIPOQ/CCL5/PPARA/INS/ABHD12/SPN/ALOX5/HLA-E/RELA/PYCARD/CEBPB/GPSM3/RHBDF2/TEK/GHSR/LYN/AGER/CEBPA/CTSC/SCGB1A1/PTPRC/IL17A/CASP4/ALOX5AP/NFKB1/MEFV/STAT3/PTGES
GOCC_ENDOPLASMIC_RETICULUM_LUMEN	GOCC_ENDOPLASMIC_RETICULUM_LUMEN	GOCC_ENDOPLASMIC_RETICULUM_LUMEN	64	-0.333202973	-2.356337549	9.77E-05	0.002876375	0.002303106	949	tags=55%, list=30%, signal=39%	WNT1/WNT3A/ADAMTS5/CD4/TGOLN2/FMO1/SDC2/INS/COL15A1/MEN1/TIMP1/COL4A5/VWA1/FAM20C/IGFBP4/COL4A2/BMP4/WFS1/SERPINA1/COL6A3/COL3A1/CTSC/COL27

											A1/F10/CYP2W1/PDIA6/CALU/MBTPS1/TOR1A/SUMF1/TSPAN5/FBN1/PDIA5/TXNDC5/FUCA2
GOBP_T_CELL_PROLIFERATION	GOBP_T_CELL_PROLIFERATION	GOBP_T_CELL_PROLIFERATION	45	-0.375097707	-2.332027639	0.000101193	0.002962197	0.002371824	866	tags=58%, list=27%, signal=43%	CCL5/LIPA/CD6/HLA-A/SPN/BTN2A2/HLA-E/PYCARD/CEBPB/TNFRSF4/AIF1/BMP4/BAX/HLA-DMB/AGER/IGF2/SCGB1A1/SLC11A1/PTPRC/PPP3CA/MSN/HMGB1/HAVCR2/TYK2/EFNB1/ABL1
GOBP_MITOTIC_SISTER_CHROMATID_SEGREGATION	GOBP_MITOTIC_SISTER_CHROMATID_SEGREGATION	GOBP_MITOTIC_SISTER_CHROMATID_SEGREGATION	49	0.357213074	2.535237258	0.000107046	0.003113395	0.002492887	923	tags=63%, list=29%, signal=46%	SPAG5/CENPE/CDCA8/KATNB1/AURKC/TPR/SMC3/TRIP13/PINX1/KIF23/CDK5RAP2/MAD2L1BP/DYNC1LI1/TUBG1/BUB1B/CHEK2/KIF4A/ESPL1/KPNB1/TPX2/TUBG2/ZW10/MAD2L2/KIF22/AKAP8L/NUSAP1/CDC16/BCCIP/UBE2C/PDCD6IP/MAPRE1
GOBP_CELLULAR_RESPONSE_TO_LIPID	GOBP_CELLULAR_RESPONSE_TO_LIPID	GOBP_CELLULAR_RESPONSE_TO_LIPID	112	-0.268478237	-2.247310509	0.000108292	0.003113395	0.002492887	1285	tags=61%, list=40%, signal=38%	CYP7A1/LTF/TNF/ATP1A2/LRP6/SYK/CTSG/SAFB2/SSTR3/VPS18/WNT11/RARG/INHBA/MYOG/FFAR2/BCR/CYP1B1/ELK1/HAND2/MYOD1/SNAI2/ZNF703/WNT3A/CXCL13/CCR5/VPS11/BAD/CD6/PPARA/RXRB/FAM107A/KMO/XBP1/RELA/PYCARD/YAP1/GNB1/GNAI1/GHSR/TFPI/DAB2/FECH/LYN/TRIM41/SMO/DDX54/FZD4/LY86/GRAMD1A/AKT1/AKR1C1/PRMT2/ATP1A3/ADAM15/MSN/CDK4/IRAK1/HMGB1/NFKB1/PHB2/ZBTB7A/HAVCR2/UBE2L3/NFKBIL1/KLF9/TSPO/BCL2L2/ABL1
GOBP_ANIMAL_ORGAN_MORPHOGENESIS	GOBP_ANIMAL_ORGAN_MORPHOGENESIS	GOBP_ANIMAL_ORGAN_MORPHOGENESIS	209	-0.21016594	-2.091107562	0.000107677	0.003113395	0.002492887	1082	tags=47%, list=34%, signal=33%	NF1/RARG/INHBA/CLDN5/MTHFD1/AQP6/BCR/LCTL/TRIOBP/PHACTR4/PAX4/ETV7/FKBP1A/HAND2/BCL2/TGFA/WNT1/IMPG2/SNAI2/WNT3A/NRP2/MDM2/FHL1/ADAMTS5/LIPA/EMP2/STIM1/TTC8/PPARA/ILK/HIPK2/MAFB/CD34/CITED2/EMX1/ELN/FOSL2/SMAD7/FRS2/OTOP1/XBP1/RELA/TMEM119/PBX3/FAM20C/CEBPB/MYC/YAP1/INPPL1/NRP1/BMP4/TEK/EDA/PKP2/BAX/DAB2/LFNG/PITX3/SMO/PLXNA1/ANXA6/FGF1/

											ZNF22/COL3A1/HAS2/OTOR/AHDC1/PLEKHA4/ANKRD11/IGF2/PLAG1/ATF4/MGP/PAX9/RAC1/COL27A1/PERP/PPP3CA/GLI2/ALDH1A3/MED12/ADAM15/FBXW11/MSN/MEF2D/NFKB1/PHB2/MMP14/NKX2-5/FBN1/HEG1/PARVA/STAT3/ZMPSTE24/MAGED1/FGFR1/PTN/ABL1
GOBP_LEUKOCYTE_MIGRATION	GOBP_LEUKOCYTE_MIGRATION	GOBP_LEUKOCYTE_MIGRATION	77	-0.304902836	-2.295357851	0.000112166	0.003205684	0.002566783	1356	tags=66%, list=42%, signal=39%	JAGN1/CCL8/OXSR1/FYN/TNF/THY1/SYK/CCL22/CCR1/CTSG/APOD/SELPLG/NF1/FFAR2/BCR/GPR15/IL1R1/CXCL13/CXCR3/CCL5/EMP2/CCR5/CD34/SPN/C3AR1/ALOX5/CCL21/VEGFB/FOLR2/PYCARD/GPSM3/AIF1/LYN/AIRE/AGER/CD177/TRPM2/AKT1/RAC1/IL17A/FCER1G/ITGA3/ITGB1/MSN/ST3GAL4/HMGB1/MMP14/ITGA9/ITGB7/PTN/ABL1
GOBP_DNA_INTEGRITY_CHECKPOINT_SIGNALING	GOBP_DNA_INTEGRITY_CHECKPOINT_SIGNALING	GOBP_DNA_INTEGRITY_CHECKPOINT_SIGNALING	31	0.421613608	2.453358294	0.000115749	0.003288626	0.002633194	711	tags=58%, list=22%, signal=46%	DONSON/INTS7/MDC1/RAD51/CDC14B/DOT1L/CEP63/CRY1/BLM/RAD17/TIMELESS/FZR1/CHEK2/BARD1/THOC5/E2F1/ATR/RINT1
GOBP_POSITIVE_REGULATION_OF_LEUKOCYTE_PROLIFERATION	GOBP_POSITIVE_REGULATION_OF_LEUKOCYTE_PROLIFERATION	GOBP_POSITIVE_REGULATION_OF_LEUKOCYTE_PROLIFERATION	35	-0.409386614	-2.359007269	0.000117704	0.003324635	0.002662026	978	tags=63%, list=31%, signal=44%	BCL2/WNT3A/CCL5/CD6/HLA-A/SPN/HLA-E/PYCARD/TNFRSF4/AIF1/LYN/HLA-DMB/AGER/IGF2/BCL2L1/PTPRC/PPP3CA/HMGB1/HAVCR2/CHRNB2/TYK2/EFNB1
GOMF_OXIDOREDUCTASE_ACTIVITY_ACTING_ON_A_SULFUR_GROUP_OF_DONORS	GOMF_OXIDOREDUCTASE_ACTIVITY_ACTING_ON_A_SULFUR_GROUP_OF_DONORS	GOMF_OXIDOREDUCTASE_ACTIVITY_ACTING_ON_A_SULFUR_GROUP_OF_DONORS	13	-0.612179497	-2.382533497	0.000118705	0.003333415	0.002669057	720	tags=77%, list=22%, signal=60%	PRDX3/GSTO1/STAB1/PDIA6/PGK1/SUMF1/CCS/PDIA5/TXNDC5/MSRB2
GOCC_CENTRIOLE	GOCC_CENTRIOLE	GOCC_CENTRIOLE	25	0.467941849	2.504393165	0.000122518	0.003420578	0.002738848	518	tags=56%, list=16%, signal=47%	KIF3A/DZIP1/TSSK2/WDR62/CCHCR1/CEP63/CEP250/CEP350/SSNA1/KIAA0753/TUBG1/IFT20/BBS4/IQCB1
HP_OLIGOZOOSPERMIA	HP_OLIGOZOOSPERMIA	HP_OLIGOZOOSPERMIA	10	0.699850122	2.517739951	0.000127337	0.003494544	0.002798073	311	tags=70%, list=10%, signal=63%	DZIP1/IFT74/CATSPER2/FKBP6/KLHL10/DNAH17/BLM
GOBP_NUCLEAR_CHROMOSOME_SEGREGATION	GOBP_NUCLEAR_CHROMOSOME_SEGREGATION	GOBP_NUCLEAR_CHROMOSOME_SEGREGATION	66	0.307447907	2.428934496	0.000127164	0.003494544	0.002798073	923	tags=56%, list=29%, signal=41%	SPAG5/CCNB2/PTTG2/PTTG1/STAG3/CENPE/CDCA8/KATNB1/AURKC/TPR/SMC3/TRIP13/BRD7/PINX1/KIF23/CDK5RAP2/MAD2L1BP/DYNC1LI1/TUBG1/BUB1B/CHEK2/KIF4A/ES

											PL1/KPNB1/TPX2/TUBG2/ZW10/MAD2L2/KIF22/AKAP8L/NUSAP1/SMARCC1/CDC16/BCCIP/UBE2C/PDCD6IP/MAPRE1
GOBP_CALCIUM_ION_TRANSPORT	GOBP_CALCIUM_ION_TRANSPORT	GOBP_CALCIUM_ION_TRANSPORT	81	-0.295890068	-2.260918144	0.00012722	0.003494544	0.002798073	1352	tags=63%, list=42%, signal=37%	CCL8/SRI/UCN/FYN/DIAPH1/ATP1A2/THY1/CCR1/ATP2A1/CACNA1B/CACNA1E/ATP2B3/FKBP1A/ATP1B1/BCL2/CACNB3/WNT3A/CCL5/IBTK/CD4/CCR5/STIM1/TPCN1/CSN2/CCL21/GSTO1/WFS1/ITPR1/BAX/CALM3/LYN/LIME1/SLC25A23/GJA4/ANXA6/XCR1/TRPM2/P2RX2/CABP5/PTPRC/PPP3CA/PDGFRB/CDK5/F2RL3/SEC61A1/CHRNB2/ZMPSTE24/ATP2A3/TSPO/ABL1/VDAC1
GOBP_SISTER_CHROMATID_SEGREGATION	GOBP_SISTER_CHROMATID_SEGREGATION	GOBP_SISTER_CHROMATID_SEGREGATION	56	0.31898381	2.39728276	0.000128436	0.003504774	0.002806263	923	tags=59%, list=29%, signal=43%	SPAG5/CENPE/CDCA8/KATNB1/AURKC/TPR/SMC3/TRIP13/BRD7/PINX1/KIF23/CDK5RAP2/MAD2L1BP/DYNC1LI1/TUBG1/BUB1B/CHEK2/KIF4A/ESPL1/KPNB1/TPX2/TUBG2/ZW10/MAD2L2/KIF22/AKAP8L/NUSAP1/SMARCC1/CDC16/BCCIP/UBE2C/PDCD6IP/MAPRE1
GOMF_PEPTIDE_ANTIGEN_BINDING	GOMF_PEPTIDE_ANTIGEN_BINDING	GOMF_PEPTIDE_ANTIGEN_BINDING	17	-0.551937881	-2.350974044	0.000131944	0.00352616	0.00282338 7	829	tags=76%, list=26%, signal=57%	DHCR24/HLA-DMA/HLA-A/HLA-F/HLA-E/HLA-B/HLA-DOB/HLA-DRB5/HLA-DMB/HLA-DQB1/TAP1/HLA-DRB3/MAML1
GOBP_OSSIFICATION	GOBP_OSSIFICATION	GOBP_OSSIFICATION	81	-0.295655442	-2.259125347	0.000130935	0.00352616	0.002823387	1259	tags=60%, list=39%, signal=38%	LTF/TNF/CBFB/CCR1/RPS15/JUND/WNT11/NF1/MYOG/HAND2/BCL2/SNAI2/WNT3A/ILK/RXRB/MEN1/CAT/DNAJC13/FOSL2/SMAD7/ALOX5/TMEM119/OSTF1/FAM20C/CEBPB/LRP3/YAP1/INPPL1/BMP4/TEK/ISG15/CDH11/IFITM1/SMO/CEBPA/CHRD/IGF2/ATF4/AKT1/MGP/PPP3CA/GLI2/MEF2D/MMP14/CLIC1/KREMEN1/CTSK/ZMPSTE24/PTN
GOBP_REGULATION_OF_LEUKOCYTE_DIFFERENTIATION	GOBP_REGULATION_OF_LEUKOCYTE_DIFFERENTIATION	GOBP_REGULATION_OF_LEUKOCYTE_DIFFERENTIATION	59	-0.327950912	-2.24756521	0.000131676	0.00352616	0.002823387	1259	tags=66%, list=39%, signal=41%	LTF/TNF/CBFB/IL15/PTPN2/SYK/CCR1/NF1/INHBA/PIAS3/SOCS1/C1QC/FGL2/ADIPOQ/CD4/BAD/MAFB/BTN2A2/SMAD7/XBP1/HLA-B/TCTA/CEBPB/MYC/HCLS1/BMP4/LYN/AGE

											R/TYROBP/LAG3/PTPRC/IL17A/TFE3/PPP3CA/GLI2/HMGB1/MMP14/FBN1/ABL1
GOBP_MONOATOMIC_ION_TRANSMEMBRANE_TRANSPORT	GOBP_MONOATOMIC_ION_TRANSMEMBRANE_TRANSPORT	GOBP_MONOATOMIC_ION_TRANSMEMBRANE_TRANSPORT	193	-0.214685694	-2.101059469	0.00013214	0.00352616	0.002823387	1950	tags=80%, list=61%, signal=33%	SLC34A1/SLC4A5/SLC6A8/SLC12A1/BDKRB1/CACNA1C/GRM6/FGF13/GP1BB/ABCB1/GRIN1/SLC13A2/SNTA1/GAL/WWP1/KCNJ9/KCNG1/SLC4A1/AQP1/HCN4/HTR3A/SLC6A1/RASA3/KCNMB1/SLC25A22/KCNH7/ATG5/TRPV1/KCNMB3/CLCA4/SLC12A3/KCNQ3/TMEM163/ATP1B2/GABRR1/TRPV5/KCNS3/SLC41A3/GJC1/CAV3/HCN3/CHRNA7/SLC17A8/SLC34A3/NIPA2/KCNJ6/SLC25A37/GRIA4/SLC17A7/SRI/KCNAB3/OXSR1/FYN/DIAPH1/CLDN4/LTF/ATP1A2/TMEM63A/ATP6V1G2/STOM/THY1/CLCN6/KCNE3/ATP2A1/SLC26A5/CHRNA2/CACNA1B/ACTN2/CACNA1E/ATP2B3/FXN/ATP7B/KCNS2/ATP8B1/FKBP1A/ATP6V0A1/ATP1B1/BCL2/SLC12A8/CACNB3/WNT3A/FXYD5/ABCB7/KCNAB2/FHL1/CLCN2/IBTK/CCR5/STIM1/TPCN1/TMCO3/CNNM2/TMEM109/SCN2B/ATP13A1/OTOP1/CCL21/SLC9A8/KCNJ1/ABCC10/GSTO1/SLC25A12/SLC32A1/ITPR1/KCNH4/BAX/CALM3/CLCC1/LYN/CLIC5/SCARA5/SLC12A9/OTOP2/LIME1/COX7B/SLC25A23/SLC6A17/KCNB2/ANXA6/SLC16A1/XCR1/COX15/TRPM2/P2RX2/GRIK5/KCNK5/PPIF/SLC11A1/SLC34A2/CABP5/PTPRC/SLC26A1/ATP1A3/SFXN3/OTOP3/PPP3CA/SLC31A1/CCDC51/SLC30A9/KCNB1/ITGB1/F2RL3/LASP1/SEC61A1/PHB2/CLIC1/STEAP3/CHRNB2/COX7A1/SLC25A5/ATP2A3/COX8A/ABL1/VDAC1
GOBP_ACTIVATION_OF_IMMUNE_RESPONSE	GOBP_ACTIVATION_OF_IMMUNE_RESPONSE	GOBP_ACTIVATION_OF_IMMUNE_RESPONSE	97	-0.271531045	-2.186583967	0.000133011	0.003529894	0.002826377	1291	tags=62%, list=40%, signal=38%	FYN/PRAM1/LTF/CD276/TNF/CYLD/PTPN2/THY1/SYK/BTN1A1/NMI/BTNL2/ARRB2/FFAR2/BCL2/TNFRSF21

											/CACNB3/C1QC/UNC93B1/LIPA/PRKCH/COLEC11/CD79A/HLA-A/C3AR1/FOSL2/BTN2A2/RELA/CFHR5/CFHR3/ELF1/PYCARD/PLSCR1/PUM1/BAX/SIRT2/LYN/LIME1/ZNFX1/FLOT2/TYROBP/HLA-DQB1/PQBP1/EIF2B1/CSK/PTPRC/FCER1G/HLA-DRB3/EIF2AK2/C8A/IRAK1/HMGB1/NFKB1/PHB2/MAPKAPK3/HAVCR2/MEFV/NFKBIL1/ABL1/LSM14A
GOCC_ENDOSOME	GOCC_ENDOSOME	GOCC_ENDOSOME	225	-0.198239556	-2.015936377	0.000135032	0.003563958	0.002853652	1305	tags=53%, list=41%, signal=34%	MITD1/SBF2/FYN/SNX17/CLSTN1/RAB33A/MAGEL2/LTF/PTP4A3/TNF/ATP1A2/HPS3/IL15/CREG1/PTPN2/LRP6/CLCN6/CTSE/AP1G2/TBC1D17/VPS18/EHD4/CHMP4C/SQSTM1/TRAK2/CD1B/HLA-C/ATP7B/NDRG1/ATP6V0A1/TRAK1/RHOBTB2/GPR15/CLINT1/AMN/WNT3A/OCIAD1/ABHD6/UNC93B1/CD4/CCR5/VPS11/TGOLN2/HLA-DMA/INS/TPCN1/CD79A/HLA-A/TBC1D16/CHMP6/GPR135/M6PR/DNAJC13/HLA-F/KIR2DL4/TGFBRAP1/PRDX3/TMEM25/HLA-E/HLA-B/SLC9A8/PTP4A1/WIPI1/NRP1/VPS4A/HLA-DOB/RAPGEF1/HLA-DRB5/HLA-DMB/SLC29A3/APH1A/LLGL1/SMO/SCAMP3/RAB1A/IGF2R/ANXA6/FLOT2/RAB11A/RAB11FIP3/SLC2A13/ANTXR2/SLA/HLA-DQB1/FYCO1/SLC11A1/CLN3/FCHSD1/CHMP7/TBC1D2B/RAC1/HLA-DRB3/SLC31A1/TMUB1/AP2M1/BACE2/ITGB1/SCARB2/BET1L/IRAK1/HMGB1/MMP14/HAVCR2/CTSK/OCIAD2/STEAP3/WDR81/IFITM3/RHOU/TNFAIP1/BLOC1S1/HPS6/ZMPSTE24/TRAPPC1/DERL1/CCDC115/PRAF2/RABEP2/OSBPL1A/F8A1
GOBP_NEGATIVE_REGULATION_OF_MULTICELLULAR_ORGANISMAL_PR	GOBP_NEGATIVE_REGULATION_OF_MULTICELLULAR_ORGANISMAL_PR	GOBP_NEGATIVE_REGULATION_OF_MULTICELLULAR_ORGANISMAL_PROCESS	220	-0.207405648	-2.08760702	0.000137011	0.003596545	0.002879744	1678	tags=67%, list=52%, signal=34%	CD83/ADM/ACOT11/ATG5/ADAM17/NEUROG1/PPM1B/VASH1/FOXC1/ROCK2/NPTN/TRPV1/SOX15/TSPAN32/KLKB1/ATF2/CD300A/ARHGAP4/

OCESS	OCESS										CNR2/CX3CR1/BMP2/MCF2/DLX1/CYP51A1/CAV3/CHRNA7/SEMA4B/PRKAR1A/MERTK/EXTL3/ZFPM2/MICA/MMRN2/ANXA4/SRI/UCN/PDGFRA/LTF/TNF/CBFB/CYLD/ATP1A2/IL15/PTPN2/THY1/CCR1/CTSG/SULF1/APOD/NMI/FOXE3/WNT11/NF1/RARG/INHBA/CLDN5/ARRB2/FXN/BCR/NR1H2/GCLC/PIAS3/SOCS2/SOCS1/BCL2/TNFRSF21/GDI1/C1QC/SNAI2/FGL2/WNT3A/CXCL13/PLAU/ABHD6/CUEDC2/NFATC4/ADIPOQ/ADAMTS5/PPARA/INS/MAFB/CD34/CITED2/SPN/HLA-F/LOXL2/BTG2/TIMP1/BTN2A2/SMAD7/KLF2/ALOX5/FRS2/TMEM119/TCTA/PYCARD/SERPINE2/CEBPB/MYC/NRP1/COL4A2/BMP4/TEK/GHSR/TFPI/NOVA1/SIRT2/LYN/STAB1/DAB1/AGER/PITX3/SMO/CDH3/TYROBP/LAG3/CEBPA/RAB11FIP3/CTSC/ATF4/SCGB1A1/ARHGDIB/SLC11A1/CSK/RAC1/PTPRC/VGLL4/TFE3/PPP3CA/CDK5/PGK1/ABCD1/HMGB1/NFKB1/PHB2/HAVCR2/MEFV/ADIPOR1/KREMEN1/CTSK/IFNGR1/NFKBIL1/FBN1/PTN/GADD45A/TSPO/ITGB1BP1/WWC3
GOBP_REGULATION_OF_CELL_ACTIVATION	GOBP_REGULATION_OF_CELL_ACTIVATION	GOBP_REGULATION_OF_CELL_ACTIVATION	119	-0.24616658	-2.08234535	0.000144454	0.00377141	0.003019758	1385	tags=61%, list=43%, signal=36%	MERTK/MICA/KLHL25/PJA2/PDGFRA/FYN/PRAM1/CD276/TNF/CBFB/IL15/PTPN2/THY1/SYK/CTSG/INHBA/BCR/GCLC/SOCS1/BCL2/TNFRSF21/FGL2/WNT3A/CCL5/CD4/BAD/CD6/HLA-DMA/HLA-A/SPN/HLA-F/BTN2A2/SMAD7/HLA-E/XBP1/CCL21/ITGAM/PYCARD/SERPINE2/CEBPB/PLSCR1/TNFRSF4/AIF1/BMP4/HLA-DOB/HLA-DRB5/LYN/HLA-DMB/AGER/FLOT2/TYROBP/LAG3/CEBPA/CD177/HLA-DQB1/IGF2/CTSC/AKT1/SCGB1A1/CSK/PTPRC/FCER1G/PPP3CA/HLA-DRB3/GLI2/SUPT6H/HMGB1/MMP14/HAVCR2/CHRNB2/TYK2/E

											FNB1/ABL1
GOCC_MITOTIC_SPINDLE	GOCC_MITOTIC_SPINDLE	GOCC_MITOTIC_SPINDLE	43	0.376857108	2.543686722	0.000147104	0.003812141	0.003052371	825	tags=56%, list=26%, signal=42%	EFHC1/SPAG5/CENPE/CDC14B/WDR62/KATNA1/RAE1/DR1/TPR/MAPKBP1/SMC3/KIF23/CDK5RAP2/CCDC117/TUBG1/IQCB1/CDC7/ESPL1/SMC6/TPX2/KIF22/NUSAP1/TUBB/CDC16
GOBP_REGULATION_OF_LYMPHOCYTE_ACTIVATION	GOBP_REGULATION_OF_LYMPHOCYTE_ACTIVATION	GOBP_REGULATION_OF_LYMPHOCYTE_ACTIVATION	93	-0.279213336	-2.23051143	0.000147805	0.003812141	0.003052371	1257	tags=60%, list=39%, signal=38%	CD276/CBFB/IL15/PTPN2/THY1/SYK/CTSG/INHBA/SOCS1/BCL2/TNFRSF21/FGL2/WNT3A/CCL5/CD4/BAD/CD6/HLA-DMA/HLA-A/SPN/HLA-F/BTN2A2/SMAD7/HLA-E/XBP1/CCL21/PYCARD/CEBPB/TNFRSF4/AIF1/BMP4/HLA-DOB/HLA-DRB5/LYN/HLA-DMB/AGER/FLOT2/TYROBP/LAG3/HLA-DQB1/IGF2/AKT1/SCGB1A1/CSK/PTPRC/PPP3CA/HLA-DRB3/GLI2/SUPT6H/HMGB1/MMP14/HAVCR2/CHRNB2/TYK2/EFNB1/ABL1
GOCC_ANCHORING_JUNCTION	GOCC_ANCHORING_JUNCTION	GOCC_ANCHORING_JUNCTION	201	-0.209401757	-2.069832292	0.000148381	0.003812141	0.003052371	1325	tags=55%, list=41%, signal=35%	SH3BP1/YWHAB/DLG4/ENG/CYFIP1/LLGL2/TMEM47/CLDN4/CLASP2/CDH9/TLE2/ATP1A2/THY1/PARVB/LAYN/SV2A/VEZT/RPS15/STX3/PODXL/ACTN2/CLDN5/MPZL1/TRIOBP/DUOX2/NDRG1/ATP1B1/CCDC85B/PDLIM2/ARPC1B/PLAU/HSPB1/FHL1/FHOD1/PRKCH/ILK/ITGB8/PARVG/FAM107A/ADD1/CAT/NFASC/CDH4/CORO2B/SMAD7/FRS2/GRB7/RHOG/TSPAN9/YAP1/MYADM/CPNE3/NRP1/TEK/GJB1/PKP2/IQGAP1/CLDN3/SIRT2/DAB2/CDH11/RAI14/LYN/CNTNAP1/LLGL1/PRX/FZD4/CDH3/GJA4/IGF2R/ANXA6/FLOT2/RSU1/AKT1/RALA/CSK/ALKBH6/RAC1/ARHGEF7/PTPRC/GNA12/VAPA/PERP/CDC42BPB/IQGAP3/CTTN/PPP3CA/ITGA3/SLC31A1/PDGFRB/ITGB1/ADAM15/SCARB2/MAGI1/SH3PXD2B/MSN/CDK4/LASP1/MMP14/HAVCR2/CAP1/ITGB7/RHOU/HEG1/GDI2/PARVA/SH3PXD2A/CLDN15/TRIP6/SH

											C1/ITGB1BP1
GOBP_POSITIVE_REGULATION_OF_T_CELL_PROLIFERATION	GOBP_POSITIVE_REGULATION_OF_T_CELL_PROLIFERATION	GOBP_POSITIVE_REGULATION_OF_T_CELL_PROLIFERATION	24	-0.482925435	-2.371227455	0.00015024	0.003839467	0.003074251	866	tags=67%, list=27%, signal=49%	CCL5/CD6/HLA-A/SPN/HLA-E/PYCARD/AIF1/HLA-DMB/AGER/IGF2/PTPRC/PPP3CA/HMGB1/HAVCR2/TYK2/EFNB1
GOCC_CONDENSED_NUCLEAR_CHROMOSOME	GOCC_CONDENSED_NUCLEAR_CHROMOSOME	GOCC_CONDENSED_NUCLEAR_CHROMOSOME	16	0.560187797	2.499773947	0.000159279	0.004049039	0.003242055	672	tags=69%, list=21%, signal=55%	STAG3/RAD51/FKBP6/SMC1B/HSPA2/SMC3/BLM/TUBG1/RAD50/NOL6/KIFAP3
GOBP_PROTEIN_LOCALIZATION_TO_ORGANELLE	GOBP_PROTEIN_LOCALIZATION_TO_ORGANELLE	GOBP_PROTEIN_LOCALIZATION_TO_ORGANELLE	198	0.203033696	2.170141352	0.000161111	0.004074157	0.003262167	674	tags=34%, list=21%, signal=28%	KPNA2/SPAG5/TTC21A/NUP88/CSE1L/LZTFL1/DZIP1/FBXO7/HSPA1L/SH3GLB1/SAE1/NUP155/PARD6A/MTX2/CSNK2A2/HACL1/SPATA7/ZMYND10/UBE2D3/IFT122/SNUPN/NF2/PRKAA1/RAB8B/TPR/GLUL/DNAJC15/CEP250/OSBPL8/DNAJB6/CEP72/RER1/NCOA4/CEP350/POLA2/PAF1/LAMP3/PINX1/RGPD5/HK1/BANP/VRK1/TULP2/SRP19/BCAP29/KIAA0753/WRN/MON1A/TRIM28/IFT20/BUB1B/BBS4/CAMLG/PINK1/BARD1/VPS37A/KPNB1/ZFAND6/NUP93/TIMM23/GAK/ATR/PPP3CB/PMPCB/ARFRP1/ZW10/PPHLN1
GOBP_RESPONSE_TO_TOXIC_SUBSTANCE	GOBP_RESPONSE_TO_TOXIC_SUBSTANCE	GOBP_RESPONSE_TO_TOXIC_SUBSTANCE	58	-0.32551124	-2.206610114	0.000162273	0.004082187	0.003268596	1258	tags=64%, list=39%, signal=39%	MT1H/TNF/EHMT1/MGST2/MT1E/GPX5/MT1B/ATP7B/DUOX2/CYP1B1/BCL2/NQO1/MDM2/CCL5/SLC22A8/BPHL/PRDX1/CAT/SCN2B/PRDX3/HP/GSTO1/H19/ALAD/BAX/FECH/LYN/MAOB/ATF4/SLC11A1/NFE2L2/AKR1A1/MUC2/ALOX5AP/CCS/LTC4S/PTGES
GOBP_PROTEIN_DNA_COMPLEX_ASSEMBLY	GOBP_PROTEIN_DNA_COMPLEX_ASSEMBLY	GOBP_PROTEIN_DNA_COMPLEX_ASSEMBLY	27	0.444987107	2.468314547	0.000166138	0.004147468	0.003320867	858	tags=67%, list=27%, signal=49%	GMNN/SUGT1/CHAF1B/CENPE/RAD51/DR1/CENPH/ITGB3BP/UBN1/ATF7IP/NAP1L4/TAF6/TAF10/MED4/BAZ1A/TAF9/HAT1/ASF1B
GOBP_T_CELL_ACTIVATION_INVOLVED_IN_IMMUNE_RESPONSE	GOBP_T_CELL_ACTIVATION_INVOLVED_IN_IMMUNE_RESPONSE	GOBP_T_CELL_ACTIVATION_INVOLVED_IN_IMMUNE_RESPONSE	16	-0.570378515	-2.374974223	0.000166586	0.004147468	0.003320867	933	tags=81%, list=29%, signal=58%	FGL2/TSC1/SPN/MEN1/SMAD7/EOMES/HLA-DMB/SLC11A1/FCER1G/HMGB1/HAVCR2/STAT3/EIF2AK4
GOBP_INTEGRIN_MEDIATED_SIGNALING_PATHWAY	GOBP_INTEGRIN_MEDIATED_SIGNALING_PATHWAY	GOBP_INTEGRIN_MEDIATED_SIGNALING_PATHWAY	31	-0.430420702	-2.345503975	0.000177939	0.004407409	0.003529001	1373	tags=77%, list=43%, signal=45%	LIMS2/CCM2/PRAM1/THY1/SYK/PHACTR4/EMP2/ILK/ITGB8/TIMP1/ITGAM/NRP1/ISG15/DAB2/CD177/COL3

											A1/ITGA3/ITGB1/ADAM15/ITGA9/ITGB7/PTN/ITGB1BP1/ABL1
GOMF_DNA_HELICASE_ACTIVITY	GOMF_DNA_HELICASE_ACTIVITY	GOMF_DNA_HELICASE_ACTIVITY	17	0.540671988	2.497034168	0.000181926	0.004483168	0.003589661	776	tags=71%, list=24%, signal=54%	RFC4/SETX/RAD51/DHX30/RECQL4/BLM/RUVBL1/WRN/CHD1L/RAD50/ERCC3/MCM5
GOBP_CELL_DIVISION	GOBP_CELL_DIVISION	GOBP_CELL_DIVISION	142	0.212816544	2.092127839	0.000183459	0.004498001	0.003601537	583	tags=32%, list=18%, signal=27%	EFHC1/SPAG5/CCNB2/SH3GLB1/PTTG1/PARD6A/CKS2/CENPE/CDC14B/AHCTF1/PAFAH1B1/CDCA8/KATNA1/KATNB1/CDC25C/CEP63/RAE1/NDE1/DR1/AURKC/ITGB3BP/TPR/SMC3/ANAPC10/BLM/RUVBL1/PPP1CC/KIF23/VRK1/USP37/MYH10/SENP5/TIMELESS/FZR1/SSNA1/DYNC1LI1/TIAL1/BUB1B/EPB41L2/BBS4/CDC7/UBE2S/CHEK2/KIF4A/ESPL1
GOBP_MITOTIC_CELL_CYCLE_CHECKPOINT_SIGNALING	GOBP_MITOTIC_CELL_CYCLE_CHECKPOINT_SIGNALING	GOBP_MITOTIC_CELL_CYCLE_CHECKPOINT_SIGNALING	33	0.408566694	2.442321141	0.000186359	0.004546021	0.003639987	711	tags=58%, list=22%, signal=45%	DONSON/MDC1/CDC14B/CDCA8/TPR/BLM/TRIP13/RAD17/CDK5RAP2/MAD2L1BP/FZR1/DYNC1LI1/BUB1B/RAD50/CHEK2/BARD1/ZW10/MAD2L2/RINT1
GOBP_POSITIVE_REGULATION_OF_DEVELOPMENTAL_PROCESS	GOBP_POSITIVE_REGULATION_OF_DEVELOPMENTAL_PROCESS	GOBP_POSITIVE_REGULATION_OF_DEVELOPMENTAL_PROCESS	269	-0.183312075	-1.953398914	0.000198498	0.004817814	0.003857611	1375	tags=54%, list=43%, signal=34%	DHX37/WNT2B/ZFPM2/KLHL25/MMRN2/SFN/DLG4/ENG/GATA4/CYFIP1/CLSTN1/TNXB/LTF/CLASP2/TNF/CBFB/GHRH/IL15/SYK/HMG20B/GPRC5B/CCR1/TRIM16/AMIGO3/ZNF488/JUND/WNT11/IRX3/ANGPTL4/INHBA/GPR21/CLDN5/AGR2/ARRB2/MYOG/PLXNB2/MAP3K5/SOCS2/SOCS1/CYP1B1/TRAK1/MYOD1/BCL2/GDI1/WNT1/SNAI2/CLSTN3/ZNF703/WNT3A/ETS1/ZDHHC6/HSPB1/MDM2/ADIPOQ/CCDC3/GCM1/CLCN2/EMP2/CD4/BAD/STIM1/PRKCH/LRRN3/ILK/ITGB8/SSBP3/INS/HIPK2/RXRB/CD34/CITED2/RGS14/ADD1/C3AR1/LOXL2/BTN2A2/CDH4/SMAD7/ALOX5/FRS2/XBP1/RELA/VEGFB/TMEM119/FBXW8/FAM20C/SERPINE2/CEBPB/MYC/LRP3/YAP1/CUL7/PTCH2/NRP1/HCLS1/BMP4/TEK/GHSR/BAX/ISG15/LRG1/SIRT2/DAB2/LYN/DAB1/MIXL1/IFITM1/AGER/SMO/FZ

											D4/PLXNA1/TYROBP/ADAM12/FGF1/CEBPA/IGF2/PLAG1/ATF4/AKT1/RALA/AMIGO2/ARHGEF7/PTPRC/IL17A/TFE3/ADRA2B/IQGAP3/PPP3CA/GLI2/NFE2L2/ITGB1/NAP1L1/HMGB1/NFKB1/MMP14/CLIC1/NKX2-5/STAT3/MAGED1/FGFR1/PTN/MAML1/TSPO/EIF4G1/ABL1
GOBP_REGULATION_OF_FIBROBLAST_MIGRATION	GOBP_REGULATION_OF_FIBROBLAST_MIGRATION	GOBP_REGULATION_OF_FIBROBLAST_MIGRATION	11	-0.638380973	-2.308063621	0.000200856	0.004850668	0.003883918	364	tags=64%, list=11%, signal=57%	AKT1/CLN3/RAC1/ARHGEF7/GNA12/ITGB1/ITGB1BP1
GOCC_PLASMA_MEMBRANE_PROTEIN_COMPLEX	GOCC_PLASMA_MEMBRANE_PROTEIN_COMPLEX	GOCC_PLASMA_MEMBRANE_PROTEIN_COMPLEX	126	-0.249688527	-2.16144977	0.000207171	0.004978296	0.003986109	1614	tags=69%, list=50%, signal=36%	NOXA1/KCNMB3/TSPAN32/KCNQ3/BMP2/ATP1B2/C9/KCNS3/ITGA5/GJC1/CAV3/HCN3/CHRNA7/TRAF5/KCNJ6/GRIA4/KCNAB3/DLG4/CDH9/ATP1A2/LRP6/SYK/SGIP1/CHRNA2/CACNA1B/CACNA1E/HLA-C/KCNS2/DUOX2/FKBP1A/ATP1B1/CACNB3/WNT3A/ABHD6/KCNAB2/CD4/HLA-DMA/ITGB8/CD79A/HLA-A/ABHD12/ABCD4/HLA-F/SCN2B/CDH4/HLA-E/HLA-B/ITGAM/GNB1/GNAI1/GJB1/HLA-DOB/KCNH4/CDH11/CALM3/HLA-DRB5/LYN/HLA-DMB/APH1A/LIME1/CDH3/GJA4/KCNB2/GRM1/FLOT2/HLA-DQB1/GRIK5/RAC1/GNA12/ATP1A3/FCER1G/HLA-DRB3/ITGA3/AP2M1/KCNB1/ITGB1/C8A/GNG7/CNIH3/HMGB1/TRAF1/ITGA9/ITGB7/CHRNB2/TYK2/CLTB/SHC1
GOCC_VESICLE_MEMBRANE	GOCC_VESICLE_MEMBRANE	GOCC_VESICLE_MEMBRANE	251	-0.194602888	-2.0246869	0.000208843	0.004993624	0.003998382	1355	tags=55%, list=42%, signal=35%	ANXA4/GRIA4/SLC17A7/SRI/SYPL2/PEF1/DLG4/MITD1/AMPH/SBF2/SNX17/DIAPH1/SEC23IP/RAB20/FCAR/TMEM63A/ATP6V1G2/LRP6/STOM/CLCN6/ANXA7/GPRC5B/PAM/AP1G2/SV2A/SYP/VPS18/SGIP1/STX3/EHD4/CHMP4C/PLA1A/CD1B/SERPINB10/AQP6/HLA-C/NDRG1/ATP6V0A1/RHOBTB2/TGFA/CLINT1/AMN/WNT1/SNX24/RPH3AL/WNT3A/PLAU/ABHD6/MDM2/KCNAB2/CD4/VPS11/TGOLN2/MPEG1/HLA-DMA/TPCN1/

GOBP_REGULATION_OF_MONOATOMIC_ION_TRANSPORT	GOBP_REGULATION_OF_MONOATOMIC_ION_TRANSPORT	GOBP_REGULATION_OF_MONOATOMIC_ION_TRANSPORT	106	-0.26306327	-2.156350136	0.000210558	0.005009822	0.004011351	1331	tags=60%, list=42%, signal=36%	HLA-A/CHMP6/GPR135/MANBA/M6PR/DNAJC13/HLA-F/COPZ2/NFASC/C3AR1/KIR2DL4/HLA-E/HLA-B/SLC9A8/ITGAM/RHOG/PHACTR2/WIPI1/CPNE3/SLC32A1/VPS4A/WFS1/ITPR1/HLA-DOB/IQGAP1/DCST2/SYT12/APPBP2/DAB2/HLA-DRB5/LYN/HLA-DMB/SLC29A3/STAB1/APH1A/LLGL1/SMO/SCAMP3/FZD4/RAB1A/SLC6A17/IGF2R/ANXA6/FLOT2/TYROBP/RAB11A/RAB11FIP3/ANTXR2/CD177/TRPM2/HLA-DQB1/BCL2L1/TAP1/SLC11A1/RALA/CLN3/CHMP7/RAC1/PTPRC/DNAJC5/MS4A3/VAPA/FCER1G/HLA-DRB3/SLC31A1/AP2M1/BACE2/ITGB1/SCARB2/CNIH3/IRAK1/TOR1A/STEAP3/DENND4C/WDR81/IFITM3/RHOU/HPS6/ZMPSTE24/CLTB/PRAF2/COPZ1/SEC22B SRI/UCN/KCNAB3/OXSR1/FYN/DIAPH1/ATP1A2/STOM/THY1/KCNE3/CCR1/ATP2A1/SLC26A5/CACNA1B/ACTN2/CACNA1E/KCNS2/ATP8B1/FKBP1A/ATP1B1/BCL2/CACNB3/FXYD5/KCNAB2/HTR1B/FHL1/CCL5/CLCN2/CD4/STIM1/TPCN1/TMEM109/SCN2B/KCNJ1/SERPINE2/GSTO1/WFS1/PKP2/KCNH4/BAX/HRH3/CALM3/LYN/CLIC5/LIME1/KCNB2/MAOB/P2RX2/ATF4/KCNK5/AKT1/PPIF/CABP5/PPP3CA/PDGFRB/KCNB1/ITGB1/F2RL3/PHB2/CLIC1/NKX2-5/TSPO/ABL1/VDAC1
GOBP_POSITIVE_REGULATION_OF_MAPK_CASCADE	GOBP_POSITIVE_REGULATION_OF_MAPK_CASCADE	GOBP_POSITIVE_REGULATION_OF_MAPK_CASCADE	83	-0.287928941	-2.214789633	0.000216776	0.005132491	0.004109572	1352	tags=63%, list=42%, signal=37%	CCL8/PJA2/MAP2K3/PDGFRA/GATA4/AVPI1/TNF/SYK/CCL22/CCR1/MOS/IGFBP6/FSHR/INHBA/ARRB2/MAP3K5/HAND2/TGFA/CCL5/CD4/INS/HIPK2/MINK1/FRS2/CCL21/IGFBP4/PYCARD/NRP1/BMP4/TEK/IQGAP1/DOK4/AGER/GRM1/NTF3/FGF1/IGF2/PTPRC/LTBR/ADRA2B/IQGAP3/PDGFRB/MID1/EIF2AK2/IRAK1/HMG

											B1/PHB2/HAVCR2/FGFR1/GADD45A/SHC1/ABL1
GOBP_RNA_LOCALIZATION	GOBP_RNA_LOCALIZATION	GOBP_RNA_LOCALIZATION	39	0.377815175	2.418872991	0.000223372	0.005262855	0.004213955	958	tags=64%, list=30%, signal=45%	NUP88/NCBP2/NUP155/AHCTF1/ZNHIT3/SNUPN/RAE1/PNPT1/TPR/RUVBL1/IWS1/PARP11/KPNB1/NUP93/THOC5/ATR/NOL6/AKAP8L/NUP107/RANBP2/SMG7/XPOT/PRPF6/ENY2/NUP37
GOBP_LYMPHOCYTE_ACTIVATION_INVOLVED_IN_IMMUNE_RESPONSE	GOBP_LYMPHOCYTE_ACTIVATION_INVOLVED_IN_IMMUNE_RESPONSE	GOBP_LYMPHOCYTE_ACTIVATION_INVOLVED_IN_IMMUNE_RESPONSE	29	-0.447523886	-2.407037793	0.000228465	0.005356735	0.004289123	933	tags=66%, list=29%, signal=47%	FGL2/TSC1/SPN/MEN1/HLA-F/SMAD7/XBP1/EOMES/HLA-DMB/LFNG/SLC11A1/PTPRC/FCER1G/SUPT6H/HMGB1/HAVCR2/STAT3/EIF2AK4/ABL1
GOBP_POSITIVE_REGULATION_OF_ENDOTHELIAL_CELL_PROLIFERATION	GOBP_POSITIVE_REGULATION_OF_ENDOTHELIAL_CELL_PROLIFERATION	GOBP_POSITIVE_REGULATION_OF_ENDOTHELIAL_CELL_PROLIFERATION	20	-0.504475809	-2.317404848	0.000237325	0.005444582	0.004359463	684	tags=65%, list=21%, signal=51%	VEGFB/NRP1/BMP4/TEK/GHSR/LRG1/ANG/IGF2/AKT1/APLN/HMGB1/STAT3/FGFR1
GOBP_REGULATION_OF_DNA_METABOLIC_PROCESS	GOBP_REGULATION_OF_DNA_METABOLIC_PROCESS	GOBP_REGULATION_OF_DNA_METABOLIC_PROCESS	112	0.233901012	2.139371667	0.000234768	0.005444582	0.004359463	818	tags=43%, list=26%, signal=33%	GMNN/KPNA2/RFC4/SMCHD1/POLG2/USP1/FAF1/RAD51/PCNA/GFER/PAXIP1/SMC3/RLF/BLM/RUVBL1/SUPT3H/GTPBP4/UBE2N/RAD17/TFIP11/BRD7/MAPK3/PINX1/CCDC117/USP37/TAF6/TIMELESS/WRN/TRIM28/CDC7/TFDP1/CEBPG/TAF10/RAD50/BARD1/TTF1/SMC6/YLPM1/ATR/MAD2L2/BAZ1A/TAF9/ENDOG/MCM5/SMARCC1/POT1/PARP1/MAP3K4
GOBP_CELL_CYCLE_PHASE_TRANSITION	GOBP_CELL_CYCLE_PHASE_TRANSITION	GOBP_CELL_CYCLE_PHASE_TRANSITION	137	0.21349203	2.066487738	0.000234884	0.005444582	0.004359463	742	tags=39%, list=23%, signal=31%	DONSON/KLF11/CCNB2/FBXO7/CCNH/INTS7/MDC1/CKS2/CPSF3/CENPE/RAD51/CDC14B/CDCA8/DOT1L/CDC25C/CEP63/CRY1/HSPA2/TPR/PAXIP1/BLM/PPP6C/TRIP13/RAD17/BRD7/PAF1/PINX1/CDK5RAP2/USP37/MAD2L1BP/TIMELESS/FZR1/DYNC1LI1/BUB1B/CDC7/TFDP1/TAF10/RAD50/UBE2S/CHEK2/BARD1/POLE/ESPL1/THOC5/E2F1/ATR/ERCC3/ZW10/CDK7/MAD2L2/RINT1/AKAP8L/DCUN1D3
GOBP_POSITIVE_REGULATION_OF_GENE_EXPRESSION	GOBP_POSITIVE_REGULATION_OF_GENE_EXPRESSION	GOBP_POSITIVE_REGULATION_OF_GENE_EXPRESSION	217	-0.203518247	-2.039937797	0.000237424	0.005444582	0.004359463	1120	tags=47%, list=35%, signal=33%	WNT11/LUM/RPS27L/RARG/INHBA/CLDN5/RBMS3/AGR2/ARRB2/FFAR2/PLXNB2/CYP1B1/LARP4/HAND2/

										ORM2/WNT3A/IL1R1/ETS1/HSPB1/UNC93B1/MDM2/NFATC4/ADIPOQ/CCL5/CD4/CD6/ITGB8/INS/HLA-A/CD34/CITED2/SPN/MEN1/MAZ/HLA-F/C3AR1/KIR2DL4/HLA-E/XBP1/RELA/FXR2/TMEM119/OAS2/CTCF/PYCARD/CEBPB/MYC/LRP3/PLSCR1/YAP1/GPSM3/TNFRSF4/AIF1/BMP4/H19/EDA/ISG15/CLDN3/AIRE/AGER/SMO/RAB1A/CDH3/TYROBP/FGF1/CEBPA/PQBP1/PLAG1/ATF4/AKT1/SLC11A1/CLNS1A/PTPRC/ELAVL1/IL17A/MAF/FCER1G/NAT8/IQGAP3/PPP3CA/ITGA3/NFE2L2/EIF2AK2/LARP6/MSN/IRAK1/HMGB1/PHB2/MMP14/HAVCR2/MEFV/TRUB2/IFNGR1/NKX2-5/TYK2/HEG1/STAT3/ZMPSTE24/EIF2AK4/C14orf93/SH3BGRL/EIF4G1/ABL1
GOBP_POSITIVE_REGULATION_OF_CELL_POPULATION_PROLIFERATION	GOBP_POSITIVE_REGULATION_OF_CELL_POPULATION_PROLIFERATION	GOBP_POSITIVE_REGULATION_OF_CELL_POPULATION_PROLIFERATION	248	-0.194156132	-2.028737354	0.000237848	0.005444582	0.004359463	1082	tags=45%, list=34%, signal=32%	NF1/RARG/CLDN5/FXN/PLXNB2/CDC25B/MAP3K5/CDC42EP5/TRAK1/BCL2/GDI1/TGFA/WNT1/MALAT1/ZNF703/WNT3A/NRP2/PLEK2/MDM2/HTR1B/CCL5/EMP2/BAD/CD6/PRKCH/ILK/SSBP3/INS/HLA-A/HIPK2/SPN/MAZ/FOSL2/TIMP1/PRDX3/CDH4/FRS2/HLA-E/XBP1/RELA/CCL21/VEGFB/TMEM119/FBXW8/RHOG/FOLR2/PYCARD/MYC/YAP1/CUL7/TNFRSF4/NRP1/AIF1/HCLS1/BMP4/TEK/GHSR/LRG1/RAPGEF1/ANG/LYN/HLA-DMB/AGER/PITX3/SMO/FZD4/CDH3/PLXNA1/NTF3/FGF1/CHRD/HAS2/IGF2/BCL2L1/PLAG1/AKT1/DBF4B/RALA/RAC1/ARHGEF7/PTPRC/IQGAP3/PPP3CA/ITGA3/NFE2L2/PDGFRB/CD248/ITGB1/TSPAN31/SCARB2/NAP1L1/CDK4/APLN/IRAK1/MEF2D/FLT3LG/HMGB1/HAVCR2/CHRNB2/NKX2-5/TYK2/SLC25A5/PPP2R5B/PURA/STAT3/FGFR1/PTN/TSPO/SHC1/EFNB1/ITGB1BP1/ABL1

GOBP_CHEMICAL_HOMEOSTASIS	GOBP_CHEMICAL_HOMEOSTASIS	GOBP_CHEMICAL_HOMEOSTASIS	203	-0.202267485	-2.003948247	0.000240766	0.005485373	0.004392124	1370	tags=55%, list=43%, signal=34%	PNPLA2/JAGN1/CCL8/SLC17A7/SRI/SYPL2/GATA4/CYP7A1/DIAPH1/MT1H/RAB20/ATP1A2/CLN8/CREG1/PTPN2/THY1/KCNE3/CCR1/ATP2A1/SV2A/MT1E/GPR12/PNPLA4/ANGPTL4/GPR21/ATP2B3/NUBP1/FXN/MT1B/AQP6/FFAR2/NR1H2/GCLC/ATP7B/FKBP1A/ATP6V0A1/ATP1B1/BCL2/SLC12A8/RPH3AL/ABCB7/HTR1B/ADIPOQ/CCL5/IBTK/PDK2/LIPA/CCR5/OR10J5/BAD/STIM1/INS/TPCN1/RBP1/CNNM2/ACOX1/FOSL2/ATP13A1/ALOX5/XBP1/CCL21/SLC9A8/ABHD4/CYBRD1/GSTO1/MYC/UBTF/WFS1/ITPR1/BAX/CALM3/FECH/LYN/SCARA5/SLC12A9/AGER/LIME1/ADIPOR2/SLC25A23/ANXA6/GRM1/SLC16A1/CEBPA/XCR1/OSBP/TRPM2/HAS2/ATF4/AKT1/SLC11A1/CLN3/SLC34A2/AKR1C1/PTPRC/ATP1A3/PPP3CA/SLC29A1/SLC31A1/BACE2/SLC30A9/KCNB1/F2RL3/ABCD1/ADIPOR1/STAT3/ATP2A3/CCDC115/TSPO/NADK/ACO1/ABL1/EIF2AK1
GOBP_RIBONUCLEOPROTEIN_COMPLEX_BIOGENESIS	GOBP_RIBONUCLEOPROTEIN_COMPLEX_BIOGENESIS	GOBP_RIBONUCLEOPROTEIN_COMPLEX_BIOGENESIS	69	0.285298476	2.289994747	0.000242821	0.005506222	0.004408817	668	tags=41%, list=21%, signal=33%	SRPK1/DDX20/NUP88/PWP1/BUD13/SETX/GEMIN4/EIF5/ZNHIT3/DHX30/EBNA1BP2/RPL26L1/PRPF18/GEMIN6/EXOSC8/SF3A1/GLUL/RUVBL1/GTPBP4/UTP18/NIP7/DDX52/PA2G4/BOP1/EXOSC4/ATR/EIF5B/NOL6
GOBP_TELOMERE_MAINTENANCE	GOBP_TELOMERE_MAINTENANCE	GOBP_TELOMERE_MAINTENANCE	28	0.436249386	2.448022125	0.000251687	0.005654178	0.004527286	818	tags=64%, list=26%, signal=48%	RAD51/PCNA/RECQL4/BLM/RUVBL1/TFIP11/MAPK3/PINX1/WRN/RAD50/SMC6/YLPM1/ATR/RFC1/MAD2L2/POT1/PARP1/MAP3K4
GOCC_GERM_CELL_NUCLEUS	GOCC_GERM_CELL_NUCLEUS	GOCC_GERM_CELL_NUCLEUS	20	0.50228802	2.440720918	0.000251655	0.005654178	0.004527286	330	tags=50%, list=10%, signal=45%	TBPL1/TSN/TCFL5/DAZAP1/SPAG8/RAD51/PCNA/ACTL7A/HSPA2/TRIP13
GOBP_SUBSTRATE_ADHESION_DEPENDENT_CELL_SPREADING	GOBP_SUBSTRATE_ADHESION_DEPENDENT_CELL_SPREADING	GOBP_SUBSTRATE_ADHESION_DEPENDENT_CELL_SPREADING	24	-0.470014298	-2.307832073	0.000263498	0.005892101	0.00471779	1385	tags=88%, list=43%, signal=50%	MERTK/LIMS2/MYOC/PARVB/TRIOBP/ILK/PARVG/MYADM/NRP1/TEK/PKP2/DAB2/FZD4/RAB1A/HAS2/RAC1/ARHGEF7/ITGB7/PARVA/ITGB1BP1/ABL1

GOBP_REGULATION_OF_RHO_PROTEIN_SIGNAL_TRANSDUCTION	GOBP_REGULATION_OF_RHO_PROTEIN_SIGNAL_TRANSDUCTION	GOBP_REGULATION_OF_RHO_PROTEIN_SIGNAL_TRANSDUCTION	23	-0.459144794	-2.209425669	0.000268912	0.00598546	0.004792543	396	tags=57%, list=12%, signal=50%	NET1/NRP1/COL3A1/ARHGDIB/RAC1/ITGA3/ITGB1/F2RL3/ARHGEF3/ARHGDIA/TNFAIP1/HEG1/ABL1
GOBP_REGULATION_OF_CHROMOSOME_ORGANIZATION	GOBP_REGULATION_OF_CHROMOSOME_ORGANIZATION	GOBP_REGULATION_OF_CHROMOSOME_ORGANIZATION	52	0.327942148	2.379311104	0.000285817	0.006332557	0.005070462	825	tags=54%, list=26%, signal=41%	CENPE/CDCA8/TPR/RUVBL1/ATF7IP/TRIP13/BRD7/MAPK3/PINX1/CDK5RAP2/MAD2L1BP/DYNC1LI1/TRIM28/BUB1B/GTF2H2/RAD50/ESPL1/SMC6/YLPM1/ATR/ZW10/PPHLN1/MAD2L2/SMARCC1/POT1/PARP1/MAP3K4/CDC16
GOBP_REGULATION_OF_DEFENSE_RESPONSE	GOBP_REGULATION_OF_DEFENSE_RESPONSE	GOBP_REGULATION_OF_DEFENSE_RESPONSE	138	-0.226652513	-2.037777362	0.000289689	0.006389029	0.005115679	1382	tags=59%, list=43%, signal=35%	EXTL3/MICA/MMRN2/IER3/PJA2/UCN/FYN/LTF/TNF/CYLD/IL15/PTPN2/MGST2/SYK/GPRC5B/NMI/ARRB2/FFAR2/BCR/NR1H2/TRAFD1/FGL2/IL1R1/ETS1/UNC93B1/ADIPOQ/CCL5/PPARA/COLEC11/INS/HLA-A/ABHD12/SPN/HLA-F/KIR2DL4/ALOX5/HLA-E/OTOP1/RELA/HLA-B/PYCARD/CEBPB/PLSCR1/GPSM3/PUM1/RHBDF2/TEK/GHSR/ISG15/SIRT2/LYN/TRIM41/AGER/ZNFX1/FLOT2/TYROBP/LAG3/CEBPA/RNF185/PQBP1/CTSC/AKT1/SCGB1A1/PTPRC/IL17A/CASP4/NFE2L2/EIF2AK2/ALOX5AP/IRAK1/HMGB1/NFKB1/PHB2/MAPKAPK3/HAVCR2/MEFV/NFKBIL1/STAT3/ZMPSTE24/EIF2AK4/PTGES/LSM14A
HP_DECREASED_FERTILITY	HP_DECREASED_FERTILITY	HP_DECREASED_FERTILITY	48	0.340952568	2.390066097	0.000295429	0.006472252	0.005182316	483	tags=42%, list=15%, signal=36%	TTC21A/DNALI1/DZIP1/IFT74/STAG3/CATSPER2/RAD51/FKBP6/ZMYND10/KLHL10/DNAH17/UBE2T/AURKC/DNAH8/BLM/TRIP13/GGPS1/FOXJ1/DNAI1/WRN
GOCC_RNA_POLYMERASE_II_HOLOENZYME	GOCC_RNA_POLYMERASE_II_HOLOENZYME	GOCC_RNA_POLYMERASE_II_HOLOENZYME	18	0.509551093	2.365496679	0.000296142	0.006472252	0.005182316	755	tags=67%, list=24%, signal=51%	TBPL1/CCNH/SUPT3H/PAF1/TAF6/TAF10/GTF2H2/GTF2H1/ERCC3/CDK7/GTF2E1/TAF9
GOBP_TUBE_DEVELOPMENT	GOBP_TUBE_DEVELOPMENT	GOBP_TUBE_DEVELOPMENT	230	-0.196535461	-1.999481332	0.000298526	0.00649495	0.005200489	1087	tags=45%, list=34%, signal=32%	ANGPTL4/PODXL/NF1/RARG/CLDN5/AGR2/ADAMTSL2/MTHFD1/PLXNB2/PHACTR4/CYP1B1/ELK1/GPR15/HAND2/BCL2/TGFA/WNT1/TSC1/WNT3A/NRP2/ETS1/CXCL13/HSPB1/C

											XCR3/NFATC4/CLCN2/LIPA/EMP2/OR10J5/STIM1/TTC8/ILK/ITGB8/SSBP3/HIPK2/COL15A1/CD34/CITED2/CAT/C3AR1/LOXL2/FOSL2/SMAD7/KLF2/ALOX5/XBP1/VEGFB/TGFBI/SERPINE2/MYC/YAP1/CUL7/NRP1/COL4A2/BMP4/TEK/GHSR/EDA/GJB1/BAX/LRG1/PCSK5/ANG/STAB1/MIXL1/SMO/ADIPOR2/FZD4/ADAM12/FGF1/CEBPA/CHRD/COL3A1/HAS2/AKT1/RALA/COL27A1/HS6ST1/ADRA2B/ITGA3/SLC31A1/GLI2/NFE2L2/NFIA/PDGFRB/ITGB1/MED12/ADAM15/PGK1/APLN/PHB2/MMP14/NKX2-5/HEG1/PARVA/STAT3/MAGED1/GADD45A/ITPK1/SHC1/ITGB1BP1/EIF4G1/ABL1
GOBP_REGULATION_OF_MYELOID_LEUKOCYTE_DIFFERENTIATION	GOBP_REGULATION_OF_MYELOID_LEUKOCYTE_DIFFERENTIATION	GOBP_REGULATION_OF_MYELOID_LEUKOCYTE_DIFFERENTIATION	27	-0.438469213	-2.266919095	0.00030118	0.006523317	0.005223203	1259	tags=78%, list=39%, signal=48%	LTF/TNF/PTPN2/CCR1/NF1/INHBA/PIAS3/C1QC/ADIPOQ/CD4/MAFB/TCTA/CEBPB/MYC/HCLS1/LYN/TYROBP/IL17A/TFE3/PPP3CA/FBN1
GOCC_EXTERNAL_SIDE_OF_PLASMA_MEMBRANE	GOCC_EXTERNAL_SIDE_OF_PLASMA_MEMBRANE	GOCC_EXTERNAL_SIDE_OF_PLASMA_MEMBRANE	78	-0.284631973	-2.138560592	0.000310348	0.006691871	0.005358164	1717	tags=79%, list=54%, signal=38%	CD209/IL2RG/ADA/CLEC14A/ABCG2/CD83/ECE1/TRPV1/CX3CR1/UMODL1/IL21R/ATP1B2/CD1A/ITGA5/CHRNA7/IL9R/IGSF21/MICA/PDGFRA/ENG/BCAM/CD276/TNF/THY1/CCR1/BTN1A1/BTNL2/CD1B/HLA-C/MAP3K5/SLC22A11/IL1R1/PLAU/CXCR3/CD4/CCR5/COLEC11/CD79A/HLA-A/CD34/SPN/HLA-F/BTN2A2/HLA-E/HLA-B/ITGAM/FOLR2/TNFRSF4/LAG3/XCR1/GFRA3/PTPRC/F10/IL17A/FCER1G/ITGA3/CD248/ITGB1/ITGA9/CTSK/CHRNB2/HEG1
GOBP_MYOTUBE_DIFFERENTIATION	GOBP_MYOTUBE_DIFFERENTIATION	GOBP_MYOTUBE_DIFFERENTIATION	23	-0.455521905	-2.19199216	0.000314684	0.006725316	0.005384943	1048	tags=70%, list=33%, signal=47%	MYOG/MYOD1/BCL2/WNT1/ADAMTS5/XBP1/TMEM119/CNTNAP1/SMO/ADAM12/P2RX2/PPP3CA/ITGB1/MMP14/NKX2-5/MAML1
GOBP_ERK1_AND_ERK2_CASCADE	GOBP_ERK1_AND_ERK2_CASCADE	GOBP_ERK1_AND_ERK2_CASCADE	59	-0.316878997	-2.171685406	0.000314156	0.006725316	0.005384943	1352	tags=68%, list=42%, signal=40%	CCL8/PDGFRA/GATA4/TNF/PTPN2/SYK/CCL22/CCR1/FSHR/NF1/INHBA/ARRB2/HAND2/DUSP9/ADIPOQ/CCL5/CD4/RGS14/BTN2A2/FRS2/CCL21

											/PYCARD/MYC/NRP1/BMP4/TEK/DAB2/LYN/AGER/FGF1/CSK/PTPRC/IQGAP3/PDGFRB/HMGB1/PHB2/HAVCR2/SHC1/ITGB1BP1/ABL1
GOMF_OXIDOREDUCTASE_ACTIVITY_ACTING_ON_CH_OH_GROUP_OF_DONORS	GOMF_OXIDOREDUCTASE_ACTIVITY_ACTING_ON_CH_OH_GROUP_OF_DONORS	GOMF_OXIDOREDUCTASE_ACTIVITY_ACTING_ON_CH_OH_GROUP_OF_DONORS	28	-0.432400177	-2.265433333	0.000322839	0.006869207	0.005500156	1469	tags=82%, list=46%, signal=45%	NSDHL/GPD1/KCNAB3/DCXR/PGD/RDH5/DHRS4/LDHA/IDH3B/KCNAB2/HSD17B1/SORD/HSD17B8/AKR7A2/AKR1C1/CBR3/AKR7A3/ADHFE1/AKR1A1/CBR1/IMPDH2/IDH3G/CTBP1
GOMF_MONOATOMIC_ION_TRANSMEMBRANE_TRANSPORTER_ACTIVITY	GOMF_MONOATOMIC_ION_TRANSMEMBRANE_TRANSPORTER_ACTIVITY	GOMF_MONOATOMIC_ION_TRANSMEMBRANE_TRANSPORTER_ACTIVITY	133	-0.231237424	-2.065826978	0.000328103	0.006950613	0.005565338	1824	tags=76%, list=57%, signal=34%	KCNJ9/KCNG1/SLC4A1/AQP1/HCN4/HTR3A/SLC6A1/RASA3/SLC5A7/KCNMB1/SLC5A11/SLC25A22/KCNH7/TRPV1/KCNMB3/CLCA4/SLC12A3/KCNQ3/GABRR1/TRPV5/KCNS3/SLC41A3/GJC1/HCN3/CHRNA7/SLC17A8/SLC34A3/NIPA2/KCNJ6/SLC25A37/GRIA4/SLC17A7/KCNAB3/CLDN4/ATP1A2/TMEM63A/ATP6V1G2/CLCN6/KCNE3/ATP2A1/SLC26A5/CHRNA2/CACNA1B/CACNA1E/ATP2B3/SLC10A5/ATP7B/KCNS2/ATP6V0A1/SLC12A8/CACNB3/KCNAB2/HTR1B/CLCN2/TPCN1/TMCO3/CNNM2/TMEM109/SCN2B/ATP13A1/OTOP1/SLC9A8/KCNJ1/SLC25A12/SLC32A1/ITPR1/KCNH4/CLCC1/CLIC5/SLC12A9/OTOP2/COX7B/KCNB2/ANXA6/SLC16A1/SLC2A13/COX15/TRPM2/P2RX2/GRIK5/KCNK5/SLC11A1/SLC34A2/SLC26A1/ATP1A3/SFXN3/OTOP3/SLC29A1/SLC31A1/CCDC51/SLC30A9/KCNB1/LASP1/SEC61A1/CLIC1/CHRNB2/COX7A1/SLC25A5/ATP2A3/COX8A/VDAC1
GOMF_TRANSPORTER_ACTIVITY	GOMF_TRANSPORTER_ACTIVITY	GOMF_TRANSPORTER_ACTIVITY	226	-0.190847431	-1.937051664	0.000330745	0.00697598	0.005585649	1826	tags=71%, list=57%, signal=33%	SLC16A12/KCNJ9/KCNG1/SLC4A1/SEC63/RALBP1/AQP1/HCN4/SLC7A4/HTR3A/SLC6A1/RASA3/ABCD3/SLC5A7/KCNMB1/ABCG2/SLC5A11/SLC23A3/SLC25A22/KCNH7/SLC7A14/TRPV1/KCNMB3/CLCA4/SLC12A3/KCNQ3/SLC22A2/TOMM40/GABRR1/

											TRPV5/KCNS3/SLC41A3/GJC1/HCN3/CHRNA7/SLC35A1/TAP2/SLC17A8/SLC34A3/NIPA2/KCNJ6/MFSD1/SLC25A37/GRIA4/SLC16A9/SLC17A7/KCNAB3/STARD4/CLDN4/SLC25A36/ATP1A2/TMEM63A/ATP6V1G2/LRP6/CLCN6/KCNE3/PLTP/ATP2A1/MPV17/SV2A/SLC35A4/SLC26A5/APOD/ATAD1/NDUFS1/SLC25A20/CHRNA2/CACNA1B/CACNA1E/ATP2B3/SLC10A5/AQP6/ATP7B/KCNS2/ATP8B1/ATP6V0A1/BCL2/SLC12A8/SLC22A11/CACNB3/SLC29A2/ABCB7/NDUFB7/KCNAB2/HTR1B/CLCN2/SLC22A8/SLC22A7/SIDT1/TPCN1/SLC16A11/ABCD4/TMCO3/CNNM2/TMEM109/SCN2B/ATP13A1/OTOP1/SLC9A8/KCNJ1/ABCC10/CYBRD1/PLSCR1/SLC25A12/SLC32A1/RHBDF2/GJB1/ITPR1/SLC44A1/KCNH4/BAX/CLCC1/SLC29A3/CLIC5/SLC12A9/OTOP2/SLC35B2/COX7B/SLC25A23/GJA4/SLC6A17/KCNB2/ANXA6/SLC16A1/SLC2A13/COX15/OSBP/TRPM2/GRAMD1A/P2RX2/GRIK5/KCNK5/TAP1/SLC11A1/SLC34A2/SLC26A1/ATP1A3/SFXN3/OTOP3/SLC29A1/SLC31A1/TIMM17B/CCDC51/TOMM20/SLC30A9/KCNB1/ABCD1/LASP1/SEC61A1/CLIC1/OSBPL5/CHRNB2/COX7A1/SLC25A5/SLC35D2/ATP2A3/SLC14A2/TSPO/COX8A/OSBPL1A/VDAC1
GOBP_APOPTOTIC_SIGNALING_PATHWAY	GOBP_APOPTOTIC_SIGNALING_PATHWAY	GOBP_APOPTOTIC_SIGNALING_PATHWAY	138	-0.224833371	-2.021421899	0.000341353	0.007168403	0.005739721	1350	tags=58%, list=42%, signal=35%	SFN/LTB/IER3/CRIP1/GATA4/APAF1/FYN/TNF/CYLD/PTPN2/IFI27/ATP2A1/PPP1R15A/SSTR3/RPS27L/TMEM14A/NF1/INHBA/FXN/GCLC/MAP3K5/SRPX/DIDO1/CYP1B1/BCL2/WNT1/SNAI2/FASTK/HSPB1/MDM2/NFATC4/PDK2/BAD/INS/HIPK2/TMEM109/SPN/MAZ/PHLDA3/XBP1/RELA/BAG5/MKNK2/ITM2C/PYCARD/CEBPB/MYC/YAP1/NRP1/BMP4/WFS1/ITPR

											1/BAX/TFPT/TNFSF10/BBC3/ANXA6/CTSC/BCL2L1/ATF4/AKT1/DEDD/IFI6/PPIF/PTPRC/LTBR/PERP/MNT/CTTN/PPP2R1A/CASP4/NFE2L2/TRAF1/CTSK/SLC25A5/ATP2A3/MAGED1/FGFR1/BCL2L2/ABL1
GOBP_REGULATION_OF_CELL_CYCLE_CHECKPOINT	GOBP_REGULATION_OF_CELL_CYCLE_CHECKPOINT	GOBP_REGULATION_OF_CELL_CYCLE_CHECKPOINT	12	0.613788918	2.438928105	0.000343011	0.007172038	0.005742632	612	tags=75%, list=19%, signal=61%	RAD51/CDCA8/CRY1/TPR/CDK5RAP2/MAD2L1BP/DYNC1LI1/BARD1/THOC5
GOBP_RESPONSE_TO_HORMONE	GOBP_RESPONSE_TO_HORMONE	GOBP_RESPONSE_TO_HORMONE	195	-0.207614547	-2.028811552	0.000347569	0.007236031	0.005793871	1178	tags=50%, list=37%, signal=34%	TRIM16/UGCG/SLC26A5/SAFB2/CYP11A1/SSTR3/VPS18/BCHE/FSHR/CSHL1/RARG/ACTN2/INHBA/GPR21/NR1H2/GCLC/SOCS2/SOCS1/CYP1B1/MYOD1/BCL2/NQO1/WNT1/TSC1/ARPC1B/MDM2/HTR1B/ADIPOQ/PDK2/VPS11/DHCR24/PPARA/GPR83/INS/RXRB/FAM107A/CITED2/CAT/AANAT/BTG2/FOSL2/TIMP1/TSHB/OTOP1/XBP1/RELA/CCL21/GRB7/UCN3/YAP1/INPPL1/GNB1/GNAI1/MSI1/HCLS1/TEK/GHSR/GLB1/TFPI/ALAD/CRHR2/DAB2/FECH/ANG/LYN/TNFSF10/DDX54/ADIPOR2/MAOB/COL3A1/IGF2/AKT1/SCGB1A1/EIF2B1/CSK/SLC34A2/AKR1C1/PRMT2/PTPRC/ATP1A3/PPP3CA/ITGA3/MAS1L/NFE2L2/NFKB1/PHB2/ZBTB7A/MMP14/ADIPOR1/CTSK/UBE2L3/DENND4C/KLF9/TYK2/FBN1/STAT3/TSPO/SHC1
GOBP_COLLAGEN_METABOLIC_PROCESS	GOBP_COLLAGEN_METABOLIC_PROCESS	GOBP_COLLAGEN_METABOLIC_PROCESS	18	-0.514956983	-2.257688711	0.000356983	0.007400117	0.005925255	1437	tags=94%, list=45%, signal=52%	SERPINH1/UCN/ENG/CYP7A1/TNXB/INHBA/MMP15/FOSL2/MMP17/BMP4/PCOLCE/ITGB1/ADAM15/LARP6/MMP23B/MMP14/CTSK
GOMF_TUBULIN_BINDING	GOMF_TUBULIN_BINDING	GOMF_TUBULIN_BINDING	73	0.281909794	2.277117434	0.000361599	0.0074637	0.005976221	565	tags=36%, list=18%, signal=30%	KIF3A/EFHC1/SPAG5/IFT74/CACYBP/STRBP/SPAG8/CENPE/PAFAH1B1/MAST2/POLB/KATNA1/KATNB1/RAE1/NDE1/TPR/HSPH1/SMC3/CEP350/KIF23/CDK5RAP2/SSNA1/KIFC3/BBS4/MAP6D1/KIF4A
GOCC_9PLUS2_MOTILE_CILIUM	GOCC_9PLUS2_MOTILE_CILIUM	GOCC_9PLUS2_MOTILE_CILIUM	23	0.459731009	2.371459677	0.000365246	0.007468638	0.005980119	484	tags=52%, list=15%, signal=45%	DNALI1/SPA17/SPATA6/AKAP4/SPACA3/TCP11/DNAH17/DNAH8/LYZL6/CAPZB/DNAI1/IQCG

GOBP_PRODUCTION_OF_MOLECULAR_MEDIATOR_INVOLVED_IN_INFLAMMATORY_RESPONSE	GOBP_PRODUCTION_OF_MOLECULAR_MEDIATOR_INVOLVED_IN_INFLAMMATORY_RESPONSE	GOBP_PRODUCTION_OF_MOLECULAR_MEDIATOR_INVOLVED_IN_INFLAMMATORY_RESPONSE	23	-0.452096434	-2.17550864	0.000364269	0.007468638	0.005980119	1477	tags=87%, list=46%, signal=47%	CHIA/EXTL3/TNF/SYK/APOD/CUEDC2/LIPA/CD6/PPARA/INS/ALOX5/PYCARD/GPSM3/H19/LYN/IL17A/ALOX5AP/ABCD1/MEFV/STAT3
GOBP_POSITIVE_REGULATION_OF_INTRACELLULAR_SIGNAL_TRANSDUCTION	GOBP_POSITIVE_REGULATION_OF_INTRACELLULAR_SIGNAL_TRANSDUCTION	GOBP_POSITIVE_REGULATION_OF_INTRACELLULAR_SIGNAL_TRANSDUCTION	191	-0.207273714	-2.022103468	0.000366474	0.007468638	0.005980119	1382	tags=55%, list=43%, signal=34%	EXTL3/AUTS2/CCL8/LTB/PJA2/UCN/MAP2K3/MYOC/PDGFRA/ENG/GATA4/AVPI1/FYN/LTF/PTP4A3/TNF/PTPN2/SYK/CCL22/CANT1/GPRC5B/CCR1/RPS15/NMI/MOS/IGFBP6/WNT11/FSHR/INHBA/ARRB2/MAP3K5/CC2D1A/FKBP1A/VWF/HAND2/TGFA/ADIPOQ/CCL5/CD4/BAD/ILK/INS/HIPK2/MAZ/MINK1/FRS2/NET1/RELA/CCL21/IGFBP4/PYCARD/MYC/S100A4/MIER1/NRP1/HCLS1/BMP4/H19/TEK/EDA/IQGAP1/DOK4/BAX/DAB2/LYN/AGER/TNFSF10/SLC35B2/BBC3/GRM1/NTF3/FGF1/COL3A1/P2RX2/IGF2/NEK6/AKT1/RAC1/PTPRC/F10/LTBR/VAPA/ADRA2B/IQGAP3/PPP3CA/PDGFRB/MID1/ITGB1/EIF2AK2/LMCD1/F2RL3/FBXW11/IRAK1/HMGB1/ARHGEF3/PHB2/HAVCR2/RHOC/TRIM8/FGFR1/GADD45A/TRIP6/SHC1/ING4/ITGB1BP1/ABL1
GOBP_ORGANIC_ACID_METABOLIC_PROCESS	GOBP_ORGANIC_ACID_METABOLIC_PROCESS	GOBP_ORGANIC_ACID_METABOLIC_PROCESS	212	-0.196180351	-1.950460303	0.000382019	0.007752737	0.006207596	937	tags=41%, list=29%, signal=31%	CYP2S1/PSAT1/ASNSD1/DAO/LDHA/CYP2U1/IDH3B/ABHD6/ELOVL1/ADIPOQ/GNPDA1/PDK2/LIPA/FMO1/OAT/PYCR2/PPARA/INS/BPHL/ABHD12/ABCD4/RBP1/KMO/ECHDC1/ACOX1/GCSH/ICMT/ALOX5/ALDH4A1/XBP1/ABCC10/SORD/PLA2G4C/GLUD2/PYCARD/GSTO1/SLC25A12/GHSR/PIPOX/SIRT2/GNE/ADI1/ADIPOR2/SLC16A1/HSD17B8/ECHS1/OSBP/PRKAB2/HYI/ATF4/SDSL/AKT1/SCP2/PTGES2/ACY1/CLN3/UGT2A3/AKR1C1/CYP4B1/CYP2W1/SUCLG2/ADHFE1/AKR1A1/ALDH1A3/CBR1/GOT2/ACAA1/PGK1/ALOX5AP/ABCD

											1/IDH3G/ZBTB7A/ADIPOR1/ILVBL/STAT3/AIG1/ECH1/LTC4S/PTGES/BCAT2/ACO1/LARS2/DHTKD1/OSBPL1A/HMGCL/VDAC1
GOMF_EXONUCLEASE_ACTIVITY	GOMF_EXONUCLEASE_ACTIVITY	GOMF_EXONUCLEASE_ACTIVITY	13	0.580552662	2.378145748	0.000393005	0.007878339	0.006308165	691	tags=77%, list=22%, signal=61%	CPSF3/PNPT1/EXOSC8/POLRMT/WRN/CNOT7/RAD50/POLE/EXOSC4/CNOT6
GOBP_REGULATION_OF_LEUKOCYTE_MIGRATION	GOBP_REGULATION_OF_LEUKOCYTE_MIGRATION	GOBP_REGULATION_OF_LEUKOCYTE_MIGRATION	40	-0.379512602	-2.264023015	0.000393101	0.007878339	0.006308165	1317	tags=72%, list=41%, signal=43%	OXSR1/TNF/THY1/CCR1/APOD/NF1/BCR/IL1R1/CXCL13/CXCR3/CCL5/SPN/C3AR1/CCL21/VEGFB/PYCARD/GPSM3/AIF1/LYN/AIRE/AGER/AKT1/RAC1/MSN/ST3GAL4/HMGB1/MMP14/PTN/ABL1
GOBP_POSITIVE_REGULATION_OF_CELL_DIFFERENTIATION	GOBP_POSITIVE_REGULATION_OF_CELL_DIFFERENTIATION	GOBP_POSITIVE_REGULATION_OF_CELL_DIFFERENTIATION	174	-0.214308023	-2.022128042	0.000392185	0.007878339	0.006308165	1301	tags=55%, list=41%, signal=35%	ENG/CYFIP1/TNXB/LTF/TNF/CBFB/IL15/SYK/HMG20B/GPRC5B/CCR1/TRIM16/ZNF488/JUND/IRX3/INHBA/CLDN5/ARRB2/MYOG/PLXNB2/MAP3K5/SOCS2/SOCS1/TRAK1/MYOD1/BCL2/GDI1/SNAI2/ZNF703/WNT3A/ETS1/MDM2/ADIPOQ/CCDC3/CLCN2/EMP2/CD4/BAD/PRKCH/ILK/INS/CD34/RGS14/ADD1/LOXL2/BTN2A2/CDH4/SMAD7/XBP1/RELA/TMEM119/FBXW8/FAM20C/SERPINE2/CEBPB/LRP3/YAP1/CUL7/PTCH2/NRP1/HCLS1/BMP4/ISG15/LRG1/SIRT2/DAB2/LYN/DAB1/IFITM1/AGER/SMO/FZD4/PLXNA1/TYROBP/CEBPA/PLAG1/AKT1/PTPRC/IL17A/TFE3/ADRA2B/PPP3CA/GLI2/ITGB1/NAP1L1/HMGB1/NFKB1/MMP14/CLIC1/NKX2-5/STAT3/FGFR1/PTN/MAML1/TSPO/EIF4G1
GOBP_EXTRINSIC_APOPTOTIC_SIGNALING_PATHWAY	GOBP_EXTRINSIC_APOPTOTIC_SIGNALING_PATHWAY	GOBP_EXTRINSIC_APOPTOTIC_SIGNALING_PATHWAY	45	-0.352690967	-2.192722233	0.00041642	0.0083112	0.006654756	1249	tags=67%, list=39%, signal=41%	TNF/CYLD/IFI27/NF1/INHBA/GCLC/SRPX/BCL2/SNAI2/BAD/RELA/MKNK2/ITM2C/PYCARD/YAP1/NRP1/BMP4/BAX/TNFSF10/BCL2L1/AKT1/DEDD/IFI6/PTPRC/LTBR/CTTN/PPP2R1A/TRAF1/FGFR1/BCL2L2
GOBP_ESTABLISHMENT_OF_RNA_LOCALIZATION	GOBP_ESTABLISHMENT_OF_RNA_LOCALIZATION	GOBP_ESTABLISHMENT_OF_RNA_LOCALIZATION	34	0.379553757	2.29608758	0.000419976	0.008347668	0.006683956	958	tags=65%, list=30%, signal=46%	NUP88/NCBP2/NUP155/AHCTF1/SNUPN/RAE1/PNPT1/TPR/IWS1/PARP11/KPNB1/NUP93/THOC5/ATR/NOL6/

											AKAP8L/NUP107/RANBP2/SMG7/XPOT/ENY2/NUP37
GOBP_ADAPTIVE_IMMUNE_RESPONSE	GOBP_ADAPTIVE_IMMUNE_RESPONSE	GOBP_ADAPTIVE_IMMUNE_RESPONSE	93	-0.265711148	-2.122648438	0.000426483	0.008442269	0.006759703	976	tags=48%, list=30%, signal=35%	TNFRSF21/C1QC/TSC1/IL1R1/CXCL13/UNC93B1/EMP2/CD4/CD6/HLA-DMA/CD79A/HLA-A/SPN/HLA-F/SMAD7/HLA-E/HLA-B/PYCARD/EOMES/HLA-DOB/HLA-DRB5/LYN/HLA-DMB/TFEB/AIRE/AGER/LIME1/LAG3/HLA-DQB1/CTSC/TAP1/SLC11A1/CSK/PTPRC/IL17A/TFE3/FCER1G/HLA-DRB3/SUPT6H/C8A/HMGB1/HAVCR2/TYK2/STAT3/EIF2AK4
GOBP_TISSUE_REMODELING	GOBP_TISSUE_REMODELING	GOBP_TISSUE_REMODELING	25	-0.462623846	-2.319137991	0.000431285	0.008502478	0.006807912	1653	tags=96%, list=52%, signal=47%	ATG5/UMOD/FOXC1/ROCK2/CBS/IL15/SYK/EFNA2/NF1/BCR/HAND2/MDM2/HTR1B/LIPA/FOSL2/TIMP1/TMEM119/BAX/CSK/PPP3CA/NFKB1/MMP14/CTSK/PTN
GOBP_WOUND_HEALING	GOBP_WOUND_HEALING	GOBP_WOUND_HEALING	94	-0.261938291	-2.099822649	0.000434607	0.008518986	0.00682113	922	tags=46%, list=29%, signal=34%	WNT3A/PLAU/HSPB1/PPARA/SPRR3/ILK/INS/CHMP6/CD34/TIMP1/ALOX5/XBP1/SERPINE2/PLSCR1/YAP1/VPS4A/TFPI/SERPINA1/LRG1/DCBLD2/CLDN3/ENTPD2/LYN/ADIPOR2/ANXA6/FGF1/COL3A1/SLC11A1/CHMP7/GNA12/F10/ADRA2B/FCER1G/PPP3CA/NFE2L2/ITGB1/F2RL3/ST3GAL4/CLIC1/RHOC/TOR1A/HPS6/ITPK1
GOBP_REGULATION_OF_CELL_DIFFERENTIATION	GOBP_REGULATION_OF_CELL_DIFFERENTIATION	GOBP_REGULATION_OF_CELL_DIFFERENTIATION	295	-0.175554421	-1.899258664	0.00043565	0.008518986	0.00682113	1259	tags=49%, list=39%, signal=33%	LTF/CLASP2/TNF/CBFB/TBX6/IL15/PTPN2/THY1/SYK/HMG20B/GPRC5B/CCR1/TRIM16/MAFG/ZNF488/FOXE3/JUND/IRX3/NF1/RARG/INHBA/CLDN5/ARRB2/MYOG/PLXNB2/NR1H2/MAP3K5/PIAS3/SOCS2/SOCS1/TRAK1/HAND2/MYOD1/BCL2/TNFRSF21/GDI1/WNT1/C1QC/SNAI2/CCDC85B/FGL2/ZNF703/WNT3A/OCIAD1/ETS1/MDM2/NFATC4/ADIPOQ/CCDC3/GCM1/CLCN2/EMP2/CD4/OR10J5/BAD/PRKCH/PPARA/ILK/INS/MAFB/CD34/RGS14/ADD1/MEN1/LOXL2/BTG2/FOSL2/BTN2A2/CDH4/SMAD7/ALOX5/FRS2/XBP1/RELA/HLA-B/T

GOBP_NEGATIVE_REGULATION_OF_CELL_CYCLE	GOBP_NEGATIVE_REGULATION_OF_CELL_CYCLE	GOBP_NEGATIVE_REGULATION_OF_CELL_CYCLE	86	0.268675095	2.324165457	0.000449994	0.008764002	0.007017313	711	tags=43%, list=22%, signal=34%	MEM119/TCTA/FBXW8/FAM20C/SERPINE2/CEBPB/MYC/LRP3/YAP1/CUL7/PTCH2/NRP1/HCLS1/BMP4/PKP2/ISG15/LRG1/SIRT2/DAB2/LYN/DAB1/MIXL1/IFITM1/AGER/PITX3/SMO/FZD4/PLXNA1/FLOT2/TYROBP/NTF3/LAG3/CEBPA/TRIO/CHRD/IGF2/PLAG1/ATF4/JDP2/AKT1/PTPRC/IL17A/TFE3/ADRA2B/MAF/PPP3CA/PPP2R1A/GLI2/NFE2L2/SUPT6H/ITGB1/CDK5/EIF2AK2/NAP1L1/HMGB1/NFKB1/MMP14/CLIC1/ADIPOR1/NKX2-5/FBN1/STAT3/FGFR1/EIF2AK4/PTN/MAML1/TSPO/ISL2/EIF4G1/ABL1GMNN/DONSON/FBXO7/INTS7/MDC1/RAD51/CDC14B/CDCA8/DOT1L/CEP63/CRY1/TPR/BLM/TRIP13/AURKAIP1/RAD17/BRD7/PINX1/CDK5RAP2/TAF6/MAD2L1BP/TIMELESS/FZR1/DYNC1LI1/BUB1B/RAD50/CHEK2/RBM14/BARD1/ESPL1/THOC5/ZBTB17/E2F1/ATR/ZW10/MAD2L2/RINT1
GOBP_REGULATION_OF_DNA_REPAIR	GOBP_REGULATION_OF_DNA_REPAIR	GOBP_REGULATION_OF_DNA_REPAIR	46	0.329777345	2.279417412	0.000454465	0.008780261	0.007030332	996	tags=61%, list=31%, signal=43%	SMCHD1/USP1/RAD51/PCNA/RUVBL1/SUPT3H/UBE2N/TFIP11/BRD7/CCDC117/TAF6/TIMELESS/TRIM28/CEBPG/TAF10/BARD1/ATR/MAD2L2/TAF9/SMARCC1/POT1/PARP1/TP53BP1/RIF1/SIRT1/ENY2/EP400/TWIST1
GOMF_SALT_TRANSMEMBRANE_TRANSPORTER_ACTIVITY	GOMF_SALT_TRANSMEMBRANE_TRANSPORTER_ACTIVITY	GOMF_SALT_TRANSMEMBRANE_TRANSPORTER_ACTIVITY	106	-0.254377402	-2.085151398	0.000453155	0.008780261	0.007030332	1950	tags=82%, list=61%, signal=33%	SLC34A1/SLC4A5/SLC6A8/SLC12A1/CACNA1C/GRIN1/SLC13A2/KCNJ9/KCNG1/SLC4A1/AQP1/HCN4/SLC6A1/RASA3/SLC5A7/KCNMB1/ABCG2/SLC5A11/SLC23A3/SLC25A22/KCNH7/TRPV1/KCNMB3/CLCA4/SLC12A3/KCNQ3/SLC22A2/GABRR1/TRPV5/KCNS3/SLC41A3/HCN3/CHRNA7/SLC17A8/SLC34A3/KCNJ6/SLC17A7/KCNAB3/CLDN4/ATP1A2/CLCN6/KCNE3/ATP2A1/SLC26A5/CACNA1B/CACNA1E/ATP2B3/SLC10A5/AQP6/A

											TP7B/KCNS2/SLC12A8/CACNB3/KCNAB2/HTR1B/CLCN2/TPCN1/SLC16A11/TMCO3/SCN2B/SLC9A8/KCNJ1/SLC25A12/ITPR1/SLC44A1/KCNH4/CLCC1/CLIC5/SLC12A9/SLC25A23/KCNB2/ANXA6/SLC16A1/TRPM2/GRIK5/KCNK5/SLC34A2/SLC26A1/ATP1A3/SLC29A1/CCDC51/SLC30A9/KCNB1/SEC61A1/CLIC1/SLC25A5/ATP2A3
GOBP_ACROSOME_ASSEMBLY	GOBP_ACROSOME_ASSEMBLY	GOBP_ACROSOME_ASSEMBLY	10	0.658590081	2.369305236	0.000483374	0.009301585	0.007447755	365	tags=70%, list=11%, signal=62%	TBPL1/RFX2/ZPBP2/PAFAH1B1/ACTL7A/ACRBP/CCDC42
GOBP_SPINDLE_ORGANIZATION	GOBP_SPINDLE_ORGANIZATION	GOBP_SPINDLE_ORGANIZATION	51	0.320472626	2.294410197	0.000489563	0.00938329	0.007513175	632	tags=43%, list=20%, signal=35%	EFHC1/SPAG5/CCNB2/SUGT1/CENPE/WDR62/CDCA8/CEP63/RAE1/AURKC/CENPH/TPR/SMC3/CEP72/KIF23/TUBG1/CHEK2/KIF4A/ESPL1/KPNB1/TPX2/TUBG2
GOCC_SPINDLE_POLE	GOCC_SPINDLE_POLE	GOCC_SPINDLE_POLE	42	0.347435197	2.305827101	0.000500304	0.009551251	0.007647662	673	tags=48%, list=21%, signal=38%	EFHC1/SPAG5/TBCCD1/CDC14B/WDR62/KATNA1/KATNB1/CEP63/RAE1/NDE1/AURKC/MAPKBP1/SMC3/CDK5RAP2/DYNC1LI1/TUBG1/SMC6/TPX2/RASSF1/ZW10
GOBP_POSITIVE_REGULATION_OF_BIOMINERAL_TISSUE_DEVELOPMENT	GOBP_POSITIVE_REGULATION_OF_BIOMINERAL_TISSUE_DEVELOPMENT	GOBP_POSITIVE_REGULATION_OF_BIOMINERAL_TISSUE_DEVELOPMENT	11	-0.60901883	-2.201904922	0.000512665	0.009672542	0.007744779	787	tags=82%, list=25%, signal=62%	RXRB/ALOX5/TMEM119/FAM20C/CEBPB/BMP4/ISG15/ATF4/PTN
GOBP_BEHAVIOR	GOBP_BEHAVIOR	GOBP_BEHAVIOR	131	-0.234174817	-2.070759163	0.000508897	0.009672542	0.007744779	1799	tags=76%, list=56%, signal=35%	NTAN1/ID2/EHMT2/CDH23/SLC6A1/PAK6/GPR37/HCRTR1/GLP1R/SHANK1/ADA/NR3C1/NPAS4/BRSK1/NEUROG1/AAAS/TRPV1/MAPK8IP2/KCNQ3/CX3CR1/EGR1/S100B/ATP1B2/ITGA5/CHRNA7/MCHR1/PJA2/UCN/DLG4/FYN/ATP1A2/GHRH/CLN8/NPTX2/MAFG/ATAD1/LGI4/SGIP1/BCHE/CEND1/ATXN1/NF1/CLDN5/ARRB2/FXN/HAND2/BCL2/TSC1/NFATC4/HTR1B/PPARA/GPR83/INS/ABHD12/HIPK2/RGS14/BTG2/NHLH2/VWA1/PBX3/SERPINE2/MMP17/SEZ6/PUM1/TMOD1/GHSR/WFS1/SLURP1/NAV2/DAB1/AGER/PITX3/MTNR1A/FZD4/GRM1/SLC16A1/NTF3/P2RX2/PI3

GOCC_ORGANELLE_SUBCOMPARTMENT	GOCC_ORGANELLE_SUBCOMPARTMENT	GOCC_ORGANELLE_SUBCOMPARTMENT	303	-0.171399156	-1.866427902	0.000512079	0.009672542	0.007744779	1505	tags=57%, list=47%, signal=33%	/AKT1/CLN3/APRT/ITGA3/ALDH1A3/ARF4/ITGB1/CDK5/GNG7/APLN/CHRNB2/STAT3/ZMPSTE24/EIF2AK4/PTN/TSPO/SGSH/EIF4G1/ABL1/MBD2/VDAC1 GORASP2/GJC1/ATXN2/MLANA/B4GALNT4/VPS13C/SEC23A/TAP2/NSDHL/PTPN1/UGT3A1/TBC1D20/CYP17A1/AP1S2/YIPF4/RAB9A/FMO5/CYP4X1/EXTL3/ALG2/PNPLA2/PCSK1N/JAGN1/ZDHHC4/GALNT1/SRI/PJA2/PDGFRA/AUP1/FUT6/CLSTN1/CYP7A1/CYP2A13/CLASP2/CLN8/IFI27/MGST2/CANT1/ANXA7/DDN/ATP2A1/ACSL5/SULF1/PPP1R15A/ZDHHC9/TXNDC11/REEP2/TMEM14A/TMEM86B/PIGG/RDH5/EXTL2/HLA-C/MAP3K5/LCTL/ATP7B/EMD/ATP8B1/FKBP1A/CYP1B1/DHRS4/BCL2/CREB3L4/TGFA/CCDC91/SPCS2/CYP2S1/CLSTN3/CYP2U1/CYP3A43/ZDHHC6/UNC93B1/PIGW/ELOVL1/CD4/TGOLN2/FMO1/STIM1/DHCR24/HLA-A/ABHD12/SLC16A11/ABCD4/TMEM109/M6PR/HLA-F/COPZ2/RHOBTB3/ATP13A1/ICMT/HLA-E/XBP1/HLA-B/SLC9A8/TMEM119/RHOG/ABHD4/PLA2G4C/TGFBI/B4GALT7/WIPI1/DNAJC14/GAL3ST4/RHBDF2/EDA/GJB1/WFS1/ITPR1/TMEM68/BAX/RNF5/PCSK5/CLCC1/ENTPD2/HLA-DRB5/APH1A/LLGL1/SCAMP3/TBC1D23/IGF2R/RAB11A/RAB11FIP3/ANTXR2/RNF185/OSBP/GRAMD1A/HAS2/STT3A/HLA-DQB1/TAP1/IFI6/CLN3/RAC1/NFE2L1/BFAR/VAPA/NAT8/CYP4B1/HLA-DRB3/CASP4/CYP2W1/BACE2/PDIA6/CALU/SCARB2/HM13/CNIH3/MBTPS1/ALOX5AP/ABCD1/ZFYVE1/ST3GAL4/MMP23B/SEC61A1/EXTL1/RHOC/SSR4/TOR1A/OSBPL5/WDR81/ILVBL/ZMPSTE24/ATP2A

											3/PDIA5/TMEM129/CLTB/PLOD1/DERL1/LTC4S/PTGES/COPZ1/SEC22B/MGAT4B
GOBP_DNA_CONFORMATION_CHANGE	GOBP_DNA_CONFORMATION_CHANGE	GOBP_DNA_CONFORMATION_CHANGE	28	0.417961229	2.345397766	0.000522266	0.009739552	0.007798434	908	tags=68%, list=28%, signal=49%	RFC4/SETX/RAD51/DHX30/TOP1MT/RECQL4/BLM/RUVBL1/WRN/CHD1L/TOP3A/GTF2H2/RAD50/ERCC3/MCM5/POT1/MCM3/HMGA1/HMGB3
GOBP_ENZYME_LINKED_RECEPTOR_PROTEIN_SIGNALING_PATHWAY	GOBP_ENZYME_LINKED_RECEPTOR_PROTEIN_SIGNALING_PATHWAY	GOBP_ENZYME_LINKED_RECEPTOR_PROTEIN_SIGNALING_PATHWAY	199	-0.204473989	-2.015380329	0.000520565	0.009739552	0.007798434	1319	tags=55%, list=41%, signal=34%	MYOC/PDGFRA/ENG/GATA4/FYN/CYFIP1/TNXB/PTP4A3/GHRH/PTPN2/COL4A1/SYK/SULF1/APOD/IGFBP6/EFNA2/KALRN/CSHL1/INHBA/GPR21/CLDN5/AGR2/COL4A6/ARRB2/MPZL1/ADAMTSL2/FKBP1A/SOCS2/SOCS1/SAMD10/TGFA/WNT1/ZNF703/NRP2/HSPB1/UBE2D1/NFATC4/ADIPOQ/PDK2/CD4/PPARA/ILK/ITGB8/INS/FNTA/HIPK2/CHMP6/CITED2/RGS14/MEN1/TGFBRAP1/COL4A5/SMAD7/FRS2/RELA/VEGFB/GRB7/FAM20C/IGFBP4/INPPL1/CPNE3/NRP1/COL4A2/RHBDF2/BMP4/TEK/GHSR/IQGAP1/DOK4/HTRA1/LRG1/DAB2/RAPGEF1/LYN/FZD4/CDH3/IGF2R/NTF3/FGF1/TRIO/CHRD/COL3A1/IGF2/AKT1/IFI6/RALA/RAC1/ARHGEF7/RGMB/ITGA3/SLC31A1/NFIA/PDGFRB/STUB1/ITGB1/GNG7/APLN/ZBTB7A/MAPKAPK3/ADIPOR1/TYK2/PPP2R5B/FBN1/STAT3/FGFR1/PTN/SHC1/EFNB1/ABL1
GOBP_RESPONSE_TO_ORGANIC_CYCLIC_COMPOUND	GOBP_RESPONSE_TO_ORGANIC_CYCLIC_COMPOUND	GOBP_RESPONSE_TO_ORGANIC_CYCLIC_COMPOUND	177	-0.206565326	-1.958494445	0.000521641	0.009739552	0.007798434	1159	tags=49%, list=36%, signal=33%	SLC26A5/SAFB2/SSTR3/VPS18/BCHE/INHBA/ARRB2/MYOG/GCLC/DUOX2/SOCS2/CYP1B1/ELK1/HAND2/MYOD1/BCL2/NQO1/SNAI2/ZNF703/ARPC1B/MDM2/HTR1B/ADIPOQ/LIPA/CCR5/VPS11/PPARA/GPR83/RXRB/FAM107A/MMP15/CAT/AANAT/BTG2/FOSL2/KLF2/RELA/IGFBP4/FOLR2/UCN3/YAP1/GNB1/GNAI1/TEK/GHSR/GLB1/TFPI/ALAD/HRH3/PMVK/SIRT2/DAB2/FECH/RAPGEF1/LYN/PITX3/SMO/DDX54/SLC16A1/CEB

											PA/MAOB/TRPM2/GRAMD1A/P2RX2/PQBP1/BCL2L1/SCGB1A1/PRMT2/PTPRC/ATP1A3/TOMM20/CDK5/MSN/CDK4/NFKB1/PHB2/ZBTB7A/MMP14/CTSK/UBE2L3/CHRNB2/KLF9/STAT3/TSPO/BCL2L2/ABL1/MBD2
GOMF_MICROTUBULE_MOTOR_ACTIVITY	GOMF_MICROTUBULE_MOTOR_ACTIVITY	GOMF_MICROTUBULE_MOTOR_ACTIVITY	13	0.569049368	2.331024255	0.000528343	0.009814981	0.00785883	721	tags=77%, list=23%, signal=60%	KIF3A/DYNLRB2/CENPE/DNAH17/DNAH8/SMC3/KIF23/KIFC3/KIF4A/KIF22
GOBP_TUBE_MORPHOGENESIS	GOBP_TUBE_MORPHOGENESIS	GOBP_TUBE_MORPHOGENESIS	187	-0.209257529	-2.017265777	0.000536871	0.009935192	0.007955082	1374	tags=56%, list=43%, signal=34%	WNT2B/ZFPM2/MMRN2/CCM2/RNH1/PDGFRA/ENG/GATA4/E2F7/APAF1/TNF/TBX6/COL4A1/THY1/SYK/SULF1/APOD/WNT11/IRX3/ANGPTL4/PODXL/NF1/RARG/CLDN5/AGR2/MTHFD1/PLXNB2/PHACTR4/CYP1B1/GPR15/HAND2/BCL2/TGFA/WNT1/TSC1/WNT3A/NRP2/ETS1/CXCL13/HSPB1/CXCR3/NFATC4/EMP2/OR10J5/STIM1/ILK/ITGB8/HIPK2/COL15A1/CD34/CITED2/C3AR1/LOXL2/SMAD7/KLF2/ALOX5/XBP1/VEGFB/TGFBI/MYC/YAP1/CUL7/NRP1/COL4A2/BMP4/TEK/GHSR/EDA/BAX/LRG1/ANG/STAB1/SMO/ADIPOR2/FZD4/ADAM12/FGF1/COL3A1/HAS2/AKT1/RALA/COL27A1/HS6ST1/ADRA2B/SLC31A1/GLI2/NFE2L2/PDGFRB/ITGB1/MED12/ADAM15/PGK1/APLN/PHB2/MMP14/NKX2-5/HEG1/PARVA/STAT3/MAGED1/GADD45A/SHC1/ITGB1BP1/ABL1
HP_ABNORMALITY_OF_HAIR_TEXTURE	HP_ABNORMALITY_OF_HAIR_TEXTURE	HP_ABNORMALITY_OF_HAIR_TEXTURE	39	-0.365582638	-2.186608288	0.000560523	0.0102986 9	0.008246134	605	tags=49%, list=19%, signal=40%	HR/EDA/HEATR3/KRT85/CDH3/COL3A1/PQBP1/PERP/ITGA3/PRR12/MED12/ABCD1/KREMEN1/SUMF1/TRIM8/ZMPSTE24/SGSH/RNF113A/EFNB1
GOBP_CIRCULATORY_SYSTEM_PROCESS	GOBP_CIRCULATORY_SYSTEM_PROCESS	GOBP_CIRCULATORY_SYSTEM_PROCESS	118	-0.230262604	-1.95023088	0.000560778	0.01029869	0.008246134	1949	tags=81%, list=61%, signal=33%	SLC4A5/BDKRB1/CACNA1C/FGF13/ABCB1/SNTA1/GNA11/SLC16A12/ID2/HCN4/SLC6A1/GLP1R/LRP1/ADA/KCNMB1/ABCG2/ADM/RAMP2/ATG5/ECE1/UMOD/FOXC1/ROCK2/TRPV1/CBS/SLC22A2/NDST2/ATP1B2/R

											ENBP/GJC1/CAV3/CHRNA7/ADD3/SVEP1/IER3/SRI/UCN/MAP2K3/GATA4/FYN/PTP4A3/TNF/ATP1A2/KCNE3/POPDC2/CTSG/ATP2A1/IRX3/CLDN5/ATP2B3/BCR/GCLC/ATP1B1/SLC29A2/MDM2/KLK1/HTR1B/ADIPOQ/EMP2/SLC22A8/PPARA/INS/ELN/C3AR1/SCN2B/CORO2B/SMAD7/KLF2/VEGFB/GSTO1/LRP3/YAP1/TEK/PKP2/NAV2/SLC44A1/CPA3/CALM3/PCSK5/AGER/SLC6A17/SLC16A1/SLC2A13/P2RX2/GNA12/ATP1A3/ADRA2B/SLC29A1/APLN/NKX2-5/HEG1/ZMPSTE24/ATP2A3/ITGB1BP1/ABL1
GOBP_SPERM_MOTILITY	GOBP_SPERM_MOTILITY	GOBP_SPERM_MOTILITY	14	0.561914064	2.405027269	0.000568931	0.010408852	0.00833434	484	tags=64%, list=15%, signal=55%	TTC21A/LZTFL1/DZIP1/ROPN1L/AKAP4/CATSPER2/IQCF1/DNAI1/IQCG
HP_PROMINENT_FOREHEAD	HP_PROMINENT_FOREHEAD	HP_PROMINENT_FOREHEAD	63	-0.304936491	-2.140965863	0.000574883	0.010478058	0.008389754	829	tags=48%, list=26%, signal=36%	DHCR24/B3GAT3/ELN/NANS/KCNJ1/INPPL1/H19/EDA/GNE/IGF2/PLAG1/RALA/PRPS1/COL27A1/ITGA3/PDGFRB/MID1/AP2M1/MED12/SH3PXD2B/MBTPS1/SETBP1/ZBTB7A/SUMF1/FBN1/PURA/STAT3/DPH5/FGFR1/FAM50A
GOMF_INORGANIC_MOLECULAR_ENTITY_TRANSMEMBRANE_TRANSPORTER_ACTIVITY	GOMF_INORGANIC_MOLECULAR_ENTITY_TRANSMEMBRANE_TRANSPORTER_ACTIVITY	GOMF_INORGANIC_MOLECULAR_ENTITY_TRANSMEMBRANE_TRANSPORTER_ACTIVITY	128	-0.233394146	-2.045151178	0.000587744	0.010672202	0.008545204	1824	tags=81%, list=57%, signal=36%	SLC34A1/SLC4A5/SLC6A8/SLC12A1/CACNA1C/GRIN1/SLC13A2/KCNJ9/KCNG1/SLC4A1/AQP1/HCN4/SLC6A1/RASA3/SLC5A7/KCNMB1/SLC5A11/SLC25A22/KCNH7/TRPV1/KCNMB3/CLCA4/SLC12A3/KCNQ3/GABRR1/TRPV5/KCNS3/SLC41A3/HCN3/CHRNA7/SLC17A8/SLC34A3/NIPA2/KCNJ6/SLC25A37/GRIA4/SLC17A7/KCNAB3/CLDN4/ATP1A2/TMEM63A/ATP6V1G2/CLCN6/KCNE3/ATP2A1/SLC26A5/CACNA1B/CACNA1E/ATP2B3/SLC10A5/AQP6/ATP7B/KCNS2/ATP6V0A1/SLC12A8/SLC22A11/CACNB3/KCNAB2/HTR1B/CLCN2/SLC22A8/TPCN1/TMCO3/CNNM2/SCN2B/ATP13A1/OTOP1/SLC9A8/KCNJ1/ABCC10/SLC25A12/SLC32A1/ITPR1/KCNH4/CLCC1/CLIC5/SLC12A9/OTOP2

											/COX7B/SLC25A23/KCNB2/ANXA6/SLC16A1/SLC2A13/COX15/TRPM2/GRIK5/KCNK5/SLC11A1/SLC34A2/SLC26A1/ATP1A3/OTOP3/SLC29A1/SLC31A1/CCDC51/SLC30A9/KCNB1/SEC61A1/CLIC1/COX7A1/SLC25A5/ATP2A3/COX8A
GOBP_NEGATIVE_REGULATION_OF_MYELOID_LEUKOCYTE_DIFFERENTIATION	GOBP_NEGATIVE_REGULATION_OF_MYELOID_LEUKOCYTE_DIFFERENTIATION	GOBP_NEGATIVE_REGULATION_OF_MYELOID_LEUKOCYTE_DIFFERENTIATION	12	-0.609404389	-2.279343608	0.000604682	0.010938626	0.00875853	1259	tags=100%, list=39%, signal=61%	PTPN2/NF1/INHBA/PIAS3/C1QC/ADIPOQ/MAFB/TCTA/MYC/LYN/FBN1
HP_THICKENED_SKIN	HP_THICKENED_SKIN	HP_THICKENED_SKIN	79	-0.275611168	-2.086206419	0.000607434	0.010947418	0.008765569	1082	tags=53%, list=34%, signal=36%	NF1/ACTN2/ADAMTSL2/ELMO2/EMD/PAX4/HAND2/PSAT1/LDHA/ELOVL1/FHL1/SERPINB8/STIM1/INS/COL4A5/PEX11B/HLA-B/BAG5/RHBDF2/GLB1/COL6A3/SLURP1/PMVK/SLC29A3/CDH3/ANTXR2/PROKR2/CTSC/AKT1/HS6ST1/PERP/TFE3/PDGFRB/SH3PXD2B/SUMF1/GMPPA/FBN1/ZMPSTE24/FGFR1/KRT14/PLOD1/RNF113A
GOBP_SINGLE_FERTILIZATION	GOBP_SINGLE_FERTILIZATION	GOBP_SINGLE_FERTILIZATION	20	0.47590585	2.312524523	0.000616278	0.011065519	0.008860133	618	tags=55%, list=19%, signal=45%	SPA17/HSPA1L/SPAG8/AKAP4/SPACA3/IQCF1/ZPBP2/ACTL7A/LYZL6/UBE3A/TNP2
GOBP_CELL_MATRIX_ADHESION	GOBP_CELL_MATRIX_ADHESION	GOBP_CELL_MATRIX_ADHESION	61	-0.318116225	-2.206752169	0.000619602	0.011083991	0.008874922	1319	tags=66%, list=41%, signal=39%	MYOC/BCAM/TNXB/CLASP2/THY1/APOD/NF1/ACTN2/BCR/BCL2/TSC1/PLAU/EMP2/ILK/ITGB8/PARVG/FAM107A/CD34/CORO2B/MINK1/CCL21/ITGAM/NRP1/TEK/EDA/CDH11/COL3A1/RAC1/ARHGEF7/CTTN/ITGA3/ITGB1/CDK5/ADAM15/MMP14/ITGA9/ITGB7/TRIP6/ITGB1BP1/ABL1
GOBP_NEGATIVE_REGULATION_OF_CELL_ADHESION	GOBP_NEGATIVE_REGULATION_OF_CELL_ADHESION	GOBP_NEGATIVE_REGULATION_OF_CELL_ADHESION	66	-0.297831632	-2.125445597	0.000623378	0.011105899	0.008892464	1251	tags=64%, list=39%, signal=40%	CLASP2/CBFB/PTPN2/CTSG/APOD/PODXL/NF1/PLXNB2/SOCS1/CYP1B1/TNFRSF21/WNT1/SNAI2/FGL2/ZNF703/FXYD5/ADIPOQ/PPARA/FAM107A/SPN/BTN2A2/CORO2B/SMAD7/CCL21/TGFBI/SERPINE2/CEBPB/MYADM/BMP4/DAB1/FZD4/PLXNA1/LAG3/AKT1/SCGB1A1/PTPRC/ADAM15/HMGB1/MMP14/HAVCR2/ITGB1

											BP1/ABL1
HP_IMPAIRMENT_IN_PERSONALITY_FUNCTIONING	HP_IMPAIRMENT_IN_PERSONALITY_FUNCTIONING	HP_IMPAIRMENT_IN_PERSONALITY_FUNCTIONING	143	-0.216356104	-1.967734767	0.000625425	0.011105899	0.008892464	782	tags=37%, list=24%, signal=29%	FKBP5/MEN1/DNAJC13/ELN/NFASC/NHLH2/TSHB/ALDH4A1/HLA-B/ITGAM/GLUD2/BAZ1B/GNB1/CCNF/WFS1/SERPINA1/ALAD/HTRA1/ANG/GALC/SMO/SLC6A17/TYROBP/PROKR2/HLA-DQB1/PQBP1/AKT1/SLC11A1/CLN3/EIF2B1/TBC1D2B/DNAJC5/ATP1A3/HS6ST1/CDC42BPB/GLI2/PRR12/PDGFRB/GALT/STUB1/UBQLN2/MED12/IMPDH2/PGK1/SETBP1/IRAK1/TOR1A/UBE2L3/CHRNB2/PURA/FGFR1/EIF4G1/EIF2AK1
GOBP_CELL_CHEMOTAXIS	GOBP_CELL_CHEMOTAXIS	GOBP_CELL_CHEMOTAXIS	52	-0.330155822	-2.178867338	0.00064134	0.011346788	0.009085344	1352	tags=69%, list=42%, signal=41%	CCL8/OXSR1/PDGFRA/SYK/CCL22/CCR1/CTSG/ARRB2/FFAR2/ELMO2/CXCL13/HSPB1/CXCR3/CCL5/CCR5/C3AR1/ALOX5/CCL21/VEGFB/RHOG/FOLR2/GPSM3/NRP1/AIF1/LYN/FGF1/XCR1/TRPM2/RAC1/FCER1G/PDGFRB/HMGB1/ITGA9/PARVA/FGFR1/PTN
HP_CNS_DEMYELINATION	HP_CNS_DEMYELINATION	HP_CNS_DEMYELINATION	11	-0.599893428	-2.168912071	0.000649911	0.011456461	0.009173159	1065	tags=91%, list=33%, signal=61%	PIGG/PYCR2/ACOX1/HTRA1/GALC/HLA-DQB1/PRPS1/EIF2B1/ABCD1/SUMF1
HP_MULTICYSTIC_KIDNEY_DYSPLASIA	HP_MULTICYSTIC_KIDNEY_DYSPLASIA	HP_MULTICYSTIC_KIDNEY_DYSPLASIA	22	0.458133052	2.322184584	0.000663198	0.011648169	0.009326659	1414	tags=91%, list=44%, signal=51%	LZTFL1/IFT74/PIGN/WNT3/SMC3/TRIP13/BUB1B/BBS4/MKKS/CEP290/ACTG2/PEX16/PEX10/ROR2/NPHP3/SMC1A/RAD21/MYH11/GNA11/LHX1
GOBP_REGULATION_OF_CELL_CYCLE_PROCESS	GOBP_REGULATION_OF_CELL_CYCLE_PROCESS	GOBP_REGULATION_OF_CELL_CYCLE_PROCESS	170	0.189562332	1.949129287	0.000668793	0.011703884	0.009371269	725	tags=35%, list=23%, signal=29%	SPAG5/DONSON/FBXO7/SH3GLB1/CCNH/INTS7/MDC1/CSNK2A2/CPSF3/CENPE/RAD51/CDC14B/AHCTF1/WDR62/CDCA8/RANBP1/DOT1L/CDC25C/CEP63/RAE1/CRY1/AURKC/HSPA2/TPR/PAXIP1/PIWIL2/CEP250/BLM/TRIP13/AURKAIP1/RAD17/BRD7/PAF1/PINX1/KIF23/CDK5RAP2/MAD2L1BP/TIMELESS/FZR1/DYNC1LI1/BUB1B/BBS4/CDC7/TFDP1/RAD50/CHEK2/RBM14/BARD1/ESPL1/SMC6/THOC5/TPX2/E2F1/ATR/ERCC3/ZW10/CDK7/MAD2L2/RINT1/PRPF4

											0A
GOBP_IMMUNE_RESPONSE_REGULATING_SIGNALING_PATHWAY	GOBP_IMMUNE_RESPONSE_REGULATING_SIGNALING_PATHWAY	GOBP_IMMUNE_RESPONSE_REGULATING_SIGNALING_PATHWAY	89	-0.261659541	-2.060333553	0.000689172	0.012016971	0.009621958	1291	tags=61%, list=40%, signal=37%	FYN/PRAM1/LTF/CD276/TNF/CYLD/PTPN2/THY1/SYK/BTN1A1/NMI/BTNL2/ARRB2/FFAR2/BCL2/TNFRSF21/CACNB3/UNC93B1/LIPA/PRKCH/COLEC11/CD79A/HLA-A/C3AR1/FOSL2/BTN2A2/RELA/ELF1/PYCARD/PLSCR1/PUM1/BAX/SIRT2/LYN/LIME1/FLOT2/TYROBP/HLA-DQB1/EIF2B1/CSK/PTPRC/FCER1G/HLA-DRB3/EIF2AK2/IRAK1/HMGB1/NFKB1/PHB2/MAPKAPK3/HAVCR2/MEFV/NFKBIL1/ABL1/LSM14A
GOCC_MICROTUBULE	GOCC_MICROTUBULE	GOCC_MICROTUBULE	86	0.260410186	2.252670126	0.000702202	0.012200135	0.009768617	787	tags=45%, list=25%, signal=35%	KIF3A/EFHC1/SPAG5/DYNLRB2/DYNLL2/SPAG8/CENPE/PAFAH1B1/POLB/KATNA1/DNAH17/KATNB1/NDE1/AURKC/DNAH8/HSPH1/ARFGEF2/FKBP4/MAP2K2/KIF23/CDK5RAP2/DNAI1/DYNC1LI1/TUBG1/KIFC3/NME7/MAP6D1/KIF4A/RASSF3/TPX2/TUBG2/RASSF1/KIFAP3/ZW10/HOOK2/KIF22/NUSAP1/PACRG/TUBB
GOBP_CELLULAR_ALDEHYDE_METABOLIC_PROCESS	GOBP_CELLULAR_ALDEHYDE_METABOLIC_PROCESS	GOBP_CELLULAR_ALDEHYDE_METABOLIC_PROCESS	17	-0.51177394	-2.179896128	0.000723061	0.012472805	0.009986943	1573	tags=100%, list=49%, signal=51%	BMP2/TPI1/PNPO/CYP1B1/DHRS4/CLCN2/RELA/ALDH2/DAB2/HYI/AKR7A2/AKR1C1/AKR7A3/AKR1A1/ALDH1A3/PDXK
GOBP_MONOATOMIC_ION_HOMEOSTASIS	GOBP_MONOATOMIC_ION_HOMEOSTASIS	GOBP_MONOATOMIC_ION_HOMEOSTASIS	116	-0.235931324	-1.990354698	0.000722423	0.012472805	0.009986943	1949	tags=83%, list=61%, signal=34%	SLC4A5/SLC12A1/BDKRB1/CACNA1C/TMEM106B/GP1BB/VAPB/SCO1/GRIN1/BCAP31/SLC4A1/CA2/CDH23/S100A14/HCRTR1/RASA3/BOK/ATG5/UMOD/NPTN/TRPV1/GPR3/FIS1/SLC12A3/ATP1B2/TRPV5/CAV3/CHRNA7/KLHL3/CCL8/SRI/SYPL2/DIAPH1/MT1H/RAB20/ATP1A2/CREG1/THY1/KCNE3/CCR1/ATP2A1/SV2A/MT1E/GPR12/ATP2B3/NUBP1/FXN/MT1B/ATP7B/FKBP1A/ATP6V0A1/ATP1B1/BCL2/SLC12A8/ABCB7/HTR1B/CCL5/IBTK/PDK2/CCR5/STIM1/TPCN1/CNNM2/ATP13A1/CCL21/SLC9A8/CYBRD1/GSTO1/MYC/WFS1/ITPR1/BAX/CALM3/FECH/LYN/SCARA

											5/SLC12A9/LIME1/SLC25A23/ANXA6/GRM1/XCR1/TRPM2/ATF4/SLC11A1/CLN3/PTPRC/ATP1A3/SLC31A1/SLC30A9/F2RL3/ATP2A3/CCDC115/ACO1/ABL1/EIF2AK1
GOBP_REGULATION_OF_REACTIVE_OXYGEN_SPECIES_METABOLIC_PROCESS	GOBP_REGULATION_OF_REACTIVE_OXYGEN_SPECIES_METABOLIC_PROCESS	GOBP_REGULATION_OF_REACTIVE_OXYGEN_SPECIES_METABOLIC_PROCESS	42	-0.356697519	-2.184554365	0.000738562	0.012694848	0.010164732	1335	tags=71%, list=42%, signal=42%	IER3/FYN/TNF/SYK/MPV17/DCXR/BCR/CYP1B1/DHRS4/BCL2/ABCB7/PPARA/INS/ALOX5/HP/ITGAM/H19/SIRT2/TYROBP/CD177/AKR1C1/RAC1/NFE2L2/PDGFRB/CBR1/ARF4/ABCD1/GADD45A/TSPO/VDAC1
GOBP_MICROTUBULE_CYTOSKELETON_ORGANIZATION_INVOLVED_IN_MITOSIS	GOBP_MICROTUBULE_CYTOSKELETON_ORGANIZATION_INVOLVED_IN_MITOSIS	GOBP_MICROTUBULE_CYTOSKELETON_ORGANIZATION_INVOLVED_IN_MITOSIS	44	0.334638681	2.289183764	0.000745524	0.012723954	0.010188037	923	tags=61%, list=29%, signal=44%	EFHC1/CENPE/PAFAH1B1/WDR62/CDCA8/RAE1/NDE1/AURKC/CENPH/TPR/SMC3/KIF23/CDK5RAP2/TUBG1/CHEK2/KIF4A/ESPL1/KPNB1/TPX2/TUBG2/ZW10/NUSAP1/VCP/SPAST/BCCIP/PDCD6IP/MAPRE1
GOMF_METAL_ION_TRANSMEMBRANE_TRANSPORTER_ACTIVITY	GOMF_METAL_ION_TRANSMEMBRANE_TRANSPORTER_ACTIVITY	GOMF_METAL_ION_TRANSMEMBRANE_TRANSPORTER_ACTIVITY	85	-0.262219697	-2.036736785	0.000745266	0.012723954	0.010188037	1950	tags=84%, list=61%, signal=34%	SLC34A1/SLC4A5/SLC6A8/SLC12A1/CACNA1C/GRIN1/SLC13A2/KCNJ9/KCNG1/AQP1/HCN4/SLC6A1/RASA3/SLC5A7/KCNMB1/SLC5A11/KCNH7/TRPV1/KCNMB3/SLC12A3/KCNQ3/TRPV5/KCNS3/SLC41A3/HCN3/CHRNA7/SLC17A8/SLC34A3/NIPA2/KCNJ6/SLC25A37/SLC17A7/KCNAB3/ATP1A2/KCNE3/ATP2A1/CACNA1B/CACNA1E/ATP2B3/SLC10A5/ATP7B/KCNS2/SLC12A8/CACNB3/KCNAB2/HTR1B/TPCN1/TMCO3/CNNM2/SCN2B/ATP13A1/SLC9A8/KCNJ1/ITPR1/KCNH4/SLC12A9/KCNB2/ANXA6/TRPM2/GRIK5/KCNK5/SLC11A1/SLC34A2/ATP1A3/SLC29A1/SLC31A1/CCDC51/SLC30A9/KCNB1/SEC61A1/ATP2A3
HP_SLOPING_FOREHEAD	HP_SLOPING_FOREHEAD	HP_SLOPING_FOREHEAD	40	0.338776695	2.223560127	0.000751112	0.01277362	0.010227804	784	tags=52%, list=24%, signal=40%	ASNS/SLC25A19/CENPE/RAD51/WDR62/KATNB1/CEP63/UBE2T/NDE1/TRIP13/POMT1/CDK5RAP2/BUB1B/ATR/NHEJ1/MAD2L2/DMXL2/CEP290/FANCC/PIGP/NUP107
GOBP_ORGANIC_HYDROXY_COMPO	GOBP_ORGANIC_HYDROXY_COMPOU	GOBP_ORGANIC_HYDROXY_COMPOUND_T	58	-0.302362301	-2.049685628	0.000756368	0.01277362	0.010227804	572	tags=40%, list=18%, signal=33%	GHSR/VPS4A/SLC44A1/HRH3/SYT12/DAB2/SLC29A3/SLC16A1/SLC2A1

UND_TRANSPORT	ND_TRANSPORT	RANSPORT									3/MAOB/OSBP/GRAMD1A/SCP2/AKR1C1/VAPA/ADRA2B/KCNB1/NFKB1/TOR1A/OSBPL5/CHRNB2/TSPO/OSBPL1A
GOCC_SUPRAMOLECULAR_COMPLEX	GOCC_SUPRAMOLECULAR_COMPLEX	GOCC_SUPRAMOLECULAR_COMPLEX	260	0.163743166	1.823091858	0.000756153	0.01277362	0.010227804	787	tags=35%, list=25%, signal=29%	TDRD7/KIF3A/EFHC1/SPAG5/C1orf112/DYNLRB2/SUGT1/PSMA6/DYNLL2/SPAG8/CSRP2/AKAP4/CENPE/CENPM/NARF/AHCTF1/PAFAH1B1/DHX30/POLB/KATNA1/DNAH17/KATNB1/SPECC1/PAWR/NDE1/MYO9A/AURKC/CENPH/EDC3/ITGB3BP/TPR/DNAH8/HSPH1/PIWIL2/ARFGEF2/PPP1CC/DNAJB6/GGPS1/EIF2S1/FKBP4/TDRKH/BRD7/MAP2K2/PINX1/KIF23/CDK5RAP2/PRPH/MYH10/CAPZB/SSNA1/DNAI1/CPEB1/DYNC1LI1/MYOM1/TUBG1/KIFC3/TIAL1/NME7/BUB1B/CAB39/MYOM2/CNOT7/EDC4/CAPN3/MAP6D1/KIF4A/BARD1/SLC1A4/KPNB1/ACTL8/BFSP1/RASSF3/TPX2/TUBG2/RASSF1/PPP3CB/KIFAP3/ZW10/KCNN2/HOOK2/KIF22/LSM3/KRT86/ACTG2/OGFOD1/KRTAP8-1/NUSAP1/NUP107/PACRG/SMARCC1/TUBB
GOBP_CELLULAR_DEFENSE_RESPONSE	GOBP_CELLULAR_DEFENSE_RESPONSE	GOBP_CELLULAR_DEFENSE_RESPONSE	11	-0.595451155	-2.152851053	0.000762938	0.012795101	0.010245004	863	tags=82%, list=27%, signal=60%	KIR2DS3/CCR5/SPN/KIR2DL4/RELA/TYROBP/LSP1/ITGB1/LGALS3BP
GOBP_CELL_CELL_SIGNALING	GOBP_CELL_CELL_SIGNALING	GOBP_CELL_CELL_SIGNALING	333	-0.164509296	-1.820436813	0.00076282	0.012795101	0.010245004	1931	tags=73%, list=60%, signal=32%	DVL2/CACNA1C/LHB/GRM6/TNFSF9/FGF13/RBX1/FBXW4/BRSK2/GRIN1/CASK/DHH/DDX3X/FGF4/GAL/SYTL4/GNA11/PTPN23/GIPR/TNKS2/CA2/AQP1/HCN4/HTR3A/LHX1/SYN1/SIGLEC6/SLC6A1/HCRTR1/DRP2/GLP1R/TBL1X/SHANK1/LRP1/NR3C1/NPAS4/SLC5A7/RAB8A/KCNMB1/WNT10A/FER1L4/SLC25A22/BRSK1/RPS6KA1/PPM1B/CHMP2B/NPTN/TRPV1/KPNA1/MAPK8IP2/TSPAN32/KCNQ3/CNR2/CX3CR1/BMP2/EGR1/S100B/APCDD1/GABRR1/TMED1/NPFF/LTBP1/GJC1/LZTS2/CHRNA7/SLC17

											A8/LTA/KLF10/PRKAR1A/NOTUM/MDFI/MERTK/WNT2B/JRK/JAGN1/CCL8/GRIA4/TM2D3/LTB/SLC17A7/SRI/UCN/MYOC/DLG4/AMPH/GATA4/FYN/CYFIP1/CLSTN1/TNXB/PCDHB14/TNF/TLE2/CYLD/ATP1A2/GHRH/IL15/LRP6/THY1/SYK/CCL22/GPRC5B/CCR1/NPTX2/FAM53B/SULF1/SV2A/GPR119/SYP/ATAD1/IGFBP6/EFNA2/SSTR3/VPS18/BCHE/STX3/WNT11/SQSTM1/CHRNA2/NF1/RARG/CACNA1B/INHBA/CACNA1E/RBMS3/ARRB2/MPZL1/TM7SF3/FFAR2/BCR/EMD/ANGPTL2/CACNB3/NQO1/WNT1/RPH3AL/PCDHB4/SNAI2/CLSTN3/ZNF703/WNT3A/CXCL13/TSHZ3/ABHD6/NFATC4/HTR1B/CPLX2/ADIPOQ/CCL5/CCR5/BAD/ILK/ITGB8/INS/PSMD9/FAM107A/CD34/RGS14/PCDHB16/KMO/SCN2B/MINK1/TSHB/TMEM25/ALOX5/RELA/CCL21/SNCB/IGFBP4/SERPINE2/SEZ6/UCN3/YAP1/GNB1/GAL3ST4/SLC32A1/NRP1/BMP4/TEK/GHSR/EDA/GJB1/ITPR1/PKP2/CRHR2/HRH3/SYT12/DAB2/CDH11/PCSK5/LYN/STAB1/AGER/SMO/TNFSF10/FZD4/RAB1A/CDH3/GJA4/GRM1/SLC16A1/RGS10/TYROBP/NTF3/TRIO/RAB11FIP3/MAOB/OSBP/P2RX2/PLEKHA4/ATF4/GRIK5/AKT1/UBE2B/CLN3/FCHSD1/RAC1/VGLL4/DNAJC5/IL17A/ADRA2B/PPP3CA/SLC29A1/ITGA3/PPP2R1A/KCNB1/ITGB1/CDK5/MED12/FBXW11/NFKB1/TOR1A/KREMEN1/CHRNB2/NKX2-5/EIF2AK4/PTN/PTGES/TSPO/NADK/EFNB1/ABL1/MBD2/VDAC1
GOCC_BASEMENT_MEMBRANE	GOCC_BASEMENT_MEMBRANE	GOCC_BASEMENT_MEMBRANE	17	-0.508735977	-2.166955954	0.000772642	0.012913013	0.010339416	795	tags=94%, list=25%, signal=71%	COL8A2/MMRN2/COL4A1/COL4A6/COL15A1/SPN/LOXL2/TIMP1/COL4A5/VWA1/TGFBI/COL4A2/ENTPD2/ANG/LAMC3/FBN1
GOBP_LONG_TER	GOBP_LONG_TER	GOBP_LONG_TERM_S	23	-0.436871192	-2.102244081	0.00077813	0.0129599	0.0103770	1124	tags=70%, list=35%,	STX3/SQSTM1/NF1/TSHZ3/NFATC4/

M_SYNAPTIC_POTENTIATION	M_SYNAPTIC_POTENTIATION	YNAPTIC_POTENTIATION				4	6	06		signal=45%	INS/FAM107A/RGS14/SERPINE2/CRHR2/SYT12/AGER/TYROBP/EIF2AK4/PTN/ABL1
GOBP_REGULATION_OF_MITOTIC_SISTER_CHROMATID_SEGREGATION	GOBP_REGULATION_OF_MITOTIC_SISTER_CHROMATID_SEGREGATION	GOBP_REGULATION_OF_MITOTIC_SISTER_CHROMATID_SEGREGATION	12	0.58722456	2.333372993	0.00078271	0.01299138	0.010402164	696	tags=75%, list=22%, signal=59%	CDCA8/TPR/TRIP13/CDK5RAP2/MAD2L1BP/DYNC1LI1/BUB1B/ZW10/MAD2L2
GOMF_AMIDE_BINDING	GOMF_AMIDE_BINDING	GOMF_AMIDE_BINDING	92	-0.253408809	-2.010945335	0.000805408	0.013322328	0.010667153	1362	tags=61%, list=43%, signal=36%	MICA/MTHFS/GRIA4/CRIP1/CLSTN1/PPIH/MRGPRX2/MCCC1/CLN8/PLTP/SSTR3/BCHE/CD1B/CACNA1B/INHBA/HLA-C/GCLC/FKBP1A/CLSTN3/DHCR24/HLA-DMA/HLA-A/FKBP5/HLA-F/HLA-E/RELA/HLA-B/ITGAM/FOLR2/ITM2C/PPIC/GHSR/HLA-DOB/CRHR2/PCSK5/ANG/HLA-DRB5/HLA-DMB/AGER/FZD4/HLA-DQB1/SCP2/TAP1/PPIF/ATP1A3/PPP3CA/HLA-DRB3/TOMM20/GPRASP2/AP2M1/PFDN1/SEC61A1/PHB2/CLTB/MAML1/VDAC1
GOBP_REGULATION_OF_PROTEIN_LOCALIZATION_TO_MEMBRANE	GOBP_REGULATION_OF_PROTEIN_LOCALIZATION_TO_MEMBRANE	GOBP_REGULATION_OF_PROTEIN_LOCALIZATION_TO_MEMBRANE	39	-0.359188507	-2.148363971	0.000831345	0.013704419	0.010973093	1291	tags=72%, list=40%, signal=43%	FYN/TNF/STOM/STX3/SQSTM1/AGR2/GDI1/WNT3A/PRKCH/INS/ABI3/ITGAM/RHOG/GHSR/VPS4A/DAB2/BBC3/BCL2L1/AKT1/CLN3/CSK/ITGA3/AP2M1/KCNB1/ITGB1/CDK5/MMP14/ITGB1BP1
GOCC_VACUOLAR_MEMBRANE	GOCC_VACUOLAR_MEMBRANE	GOCC_VACUOLAR_MEMBRANE	101	-0.256157934	-2.088933102	0.000840904	0.013814852	0.011061516	855	tags=46%, list=27%, signal=34%	VPS11/HLA-DMA/TPCN1/CHMP6/ABCD4/MANBA/M6PR/DNAJC13/HLA-F/C3AR1/ATG16L2/ABCC10/CYBRD1/ITM2C/WIPI1/CPNE3/GNB1/GNAI1/VPS4A/HLA-DOB/DAB2/HLA-DRB5/LYN/HLA-DMB/TFEB/SLC29A3/IFITM1/ANXA6/TRPM2/HLA-DQB1/SLC11A1/CLN3/CHMP7/TM9SF1/DNAJC5/VAPA/TFE3/HLA-DRB3/AP2M1/SCARB2/ABCD1/WDR81/IFITM3/BLOC1S1/HPS6/CCDC115
GOBP_FIBROBLAST_MIGRATION	GOBP_FIBROBLAST_MIGRATION	GOBP_FIBROBLAST_MIGRATION	15	-0.541422751	-2.201890723	0.000844647	0.013829306	0.011073089	552	tags=60%, list=17%, signal=50%	IQGAP1/AKT1/CLN3/RAC1/ARHGEF7/GNA12/CD248/ITGB1/ITGB1BP1
HP_ABNORMAL_EYEBROW_MORPHOLOGY	HP_ABNORMAL_EYEBROW_MORPHOLOGY	HP_ABNORMAL_EYEBROW_MORPHOLOGY	129	-0.225434393	-1.987078388	0.000860887	0.014047581	0.011247862	888	tags=43%, list=28%, signal=33%	PIGW/KCNAB2/FHL1/UBE3B/THUMPD1/PYCR2/TTC8/COLEC11/B3GAT3/ELN/NANS/CTCF/FAM20C/B4GAL

											T7/CUL7/HR/EDA/ZNF462/HEATR3/CDH11/GNE/SLC29A3/KRT85/COX7B/CDH3/SLC6A17/PLXNA1/TRIO/COL3A1/ANKRD11/PQBP1/RALA/RAC1/ATP1A3/TFE3/MAF/CDC42BPB/ITGA3/PRR12/GPRASP2/MED12/SETBP1/MMP23B/KREMEN1/SUMF1/TRIM8/CCDC8/FBN1/PURA/ZMPSTE24/DPH5/FGFR1/SGSH/RNF113A/CTBP1/ABL1
GOBP_MICROTUBULE_ORGANIZING_CENTER_ORGANIZATION	GOBP_MICROTUBULE_ORGANIZING_CENTER_ORGANIZATION	GOBP_MICROTUBULE_ORGANIZING_CENTER_ORGANIZATION	36	0.360175441	2.208438508	0.000875038	0.01423042	0.011394261	563	tags=44%, list=18%, signal=37%	KIF3A/DZIP1/PARD6A/PAFAH1B1/WDR62/RANBP1/CEP63/NDE1/CEP250/CEP72/CCDC42/FOXJ1/CDK5RAP2/KIAA0753/BBS4/RBM14
HP_UPPER_LIMB_UNDERGROWTH	HP_UPPER_LIMB_UNDERGROWTH	HP_UPPER_LIMB_UNDERGROWTH	17	0.496762325	2.294242213	0.000886955	0.014279976	0.0114339 4	747	tags=71%, list=23%, signal=54%	DONSON/RAD51/PSMD12/UBE2T/RECQL4/TRIP11/AFF4/ATR/MAD2L2/LBR/FANCC/CHN1
GOBP_NEGATIVE_REGULATION_OF_DNA_METABOLIC_PROCESS	GOBP_NEGATIVE_REGULATION_OF_DNA_METABOLIC_PROCESS	GOBP_NEGATIVE_REGULATION_OF_DNA_METABOLIC_PROCESS	25	0.422959344	2.263649834	0.000884819	0.014279976	0.0114339 4	915	tags=68%, list=29%, signal=49%	GMNN/SMCHD1/BLM/GTPBP4/RAD17/TFIP11/PINX1/TIMELESS/TRIM28/RAD50/TTF1/ATR/MAD2L2/POT1/PARP1/TP53BP1/RIF1
GOBP_POSITIVE_REGULATION_OF_DEFENSE_RESPONSE	GOBP_POSITIVE_REGULATION_OF_DEFENSE_RESPONSE	GOBP_POSITIVE_REGULATION_OF_DEFENSE_RESPONSE	79	-0.270719064	-2.049176211	0.000883023	0.014279976	0.0114339 4	1357	tags=63%, list=42%, signal=37%	MMRN2/PJA2/UCN/FYN/LTF/TNF/CYLD/IL15/MGST2/SYK/GPRC5B/NMI/ARRB2/FFAR2/ETS1/UNC93B1/CCL5/COLEC11/HLA-F/KIR2DL4/HLA-E/RELA/PYCARD/CEBPB/PLSCR1/GPSM3/PUM1/SIRT2/LYN/TRIM41/ZNFX1/FLOT2/TYROBP/LAG3/CEBPA/RNF185/PQBP1/CTSC/IL17A/CASP4/EIF2AK2/ALOX5AP/IRAK1/HMGB1/PHB2/MAPKAPK3/HAVCR2/MEFV/NFKBIL1/LSM14A
HP_ABNORMALITY_OF_THE_HAND	HP_ABNORMALITY_OF_THE_HAND	HP_ABNORMALITY_OF_THE_HAND	316	-0.164861895	-1.809732591	0.000896401	0.014384107	0.011517317	614	tags=29%, list=19%, signal=26%	INPPL1/B4GALT7/CUL7/GNB1/GNAI1/PTCH2/SLC25A12/PUM1/SLC32A1/RHBDF2/BMP4/H19/EDA/GLB1/GJB1/WFS1/COL6A3/SLURP1/ZNF462/HEATR3/CDH11/LFNG/GNE/TPM2/SLC29A3/CNTNAP1/SMO/PRX/SLC35B2/COX7B/PNKP/CDH3/SLC6A17/TYROBP/TRIO/ANTXR2/COL3A1/PROKR2/ANKRD11/PQBP1/IGF2/CTSC/PLAG1/AKT1/MGP/OSGEP/SLC34A2/

GOCC_CILIARY_BASAL_BODY	GOCC_CILIARY_BASAL_BODY	GOCC_CILIARY_BASAL_BODY	26	0.411491475	2.249402603	0.000917663	0.014613895	0.011701308	756	tags=58%, list=24%, signal=44%	TBC1D2B/RAC1/COL27A1/PERP/TFE3/MAF/CDC42BPB/PPP3CA/PPP2R1A/GLI2/PRR12/PDGFRB/STUB1/CDK5/MED12/FBXW11/SH3PXD2B/MBTPS1/ABCD1/SETBP1/MMP23B/MMP14/MEFV/TOR1A/CTSK/NFKBIL1/NKX2-5/SUMF1/TRIM8/WDR81/CCDC8/GMPPA/FBN1/ZMPSTE24/DPH5/FGFR1/EIF2AK4/KRT14/PLOD1/CTBP1/FAM50A/EFNB1/ABL1/PDXK SPAG5/DZIP1/IQCD/CCDC96/SPATA7/IFT122/CEP250/SSNA1/TUBG1/IFT20/BBS4/MKKS/KIFAP3/CEP290/RAB28
GOBP_SCHWANN_CELL_DEVELOPMENT	GOBP_SCHWANN_CELL_DEVELOPMENT	GOBP_SCHWANN_CELL_DEVELOPMENT	11	-0.59041053	-2.134626693	0.000916809	0.014613895	0.011701308	1319	tags=100%, list=41%, signal=59%	LGI4/NF1/NDRG1/ILK/SIRT2/CNTNAP1/PRX/AKT1/CDK5/MED12
GOBP_INNATE_IMMUNE_RESPONSE	GOBP_INNATE_IMMUNE_RESPONSE	GOBP_INNATE_IMMUNE_RESPONSE	166	-0.20298834	-1.907161768	0.000919798	0.014613895	0.011701308	1057	tags=46%, list=33%, signal=32%	TRIM4/ARRB2/FFAR2/HLA-C/NR1H2/MAP3K5/ATP1B1/TRAFD1/NQO1/C1QC/FBXO9/UNC93B1/CCL5/MPEG1/CD6/COLEC11/INS/HLA-A/PRDX1/HLA-F/UBL7/KIR2DL4/FOSL2/HLA-E/OTOP1/RELA/CCL21/HLA-B/OAS2/ITGAM/CFHR5/PYCARD/PLSCR1/PUM1/AIF1/ISG15/SIRT2/ANG/LYN/TFEB/TRIM41/IFITM1/ZNFX1/IFIT3/FLOT2/TYROBP/LAG3/LY86/RNF185/CD177/PQBP1/PI3/AKT1/DEFB105A/IFI6/SLC11A1/IL17A/FCER1G/CASP4/NFE2L2/EIF2AK2/C8A/ADAM15/IRAK1/HMGB1/NFKB1/PHB2/MAPKAPK3/HAVCR2/MEFV/IFNGR1/NFKBIL1/TYK2/TRIM8/IFITM3/LSM14A
GOBP_RESPONSE_TO_GROWTH_FACTOR	GOBP_RESPONSE_TO_GROWTH_FACTOR	GOBP_RESPONSE_TO_GROWTH_FACTOR	146	-0.218612542	-1.990800964	0.000931961	0.014758589	0.011817164	1723	tags=70%, list=54%, signal=34%	ADAMTS7/NR3C1/CDKN1C/FGF19/WNT10A/RASL11B/RAMP2/ADAM17/FOXC1/ROCK2/NPTN/TRPV1/ATF2/CX3CR1/BMP2/EGR1/DLX1/LTBP1/ITGA5/CAV3/PTPN1/ASCL1/CAD/MMRN2/MAP2K3/PDGFRA/ENG/GATA4/APAF1/FYN/CYFIP1/TNXB/PTP4A3/SULF1/EHD4/CLDN5/ARRB2/ADAMTSL2/MYOG/GCLC/EMD/FKBP1

											A/ELK1/WNT1/SNAI2/ZNF703/NRP2/CXCL13/HSPB1/MDM2/UBE2D1/CCL5/PPARA/ILK/ITGB8/FNTA/HIPK2/CITED2/MEN1/CAT/TGFBRAP1/SMAD7/FRS2/RELA/VEGFB/FAM20C/MYC/CPNE3/NRP1/COL4A2/BMP4/IQGAP1/HTRA1/LRG1/DAB2/RAPGEF1/PITX3/FZD4/NTF3/FGF1/CHRD/COL3A1/HAS2/AKT1/SCGB1A1/PAX9/RGMB/ITGA3/NFIA/PDGFRB/STUB1/ITGB1/APLN/ZBTB7A/CTSK/PPP2R5B/FBN1/STAT3/FGFR1/SHC1/ITGB1BP1/ABL1
GOBP_LEUKOCYTE_DIFFERENTIATION	GOBP_LEUKOCYTE_DIFFERENTIATION	GOBP_LEUKOCYTE_DIFFERENTIATION	122	-0.23106937	-1.97233431	0.000942441	0.014875782	0.011911	1259	tags=54%, list=39%, signal=34%	LTF/TNF/CBFB/IL15/PTPN2/SYK/CCR1/EFNA2/NF1/INHBA/PIAS3/SOCS1/BCL2/WNT1/C1QC/FGL2/TSC1/ADIPOQ/LIPA/CD4/BAD/ITGB8/CD79A/MAFB/CITED2/SPN/MEN1/FOSL2/BTN2A2/SMAD7/XBP1/HLA-B/TCTA/FAM20C/CEBPB/MYC/HCLS1/BMP4/EOMES/BAX/LYN/LFNG/AIRE/AGER/TYROBP/LAG3/CEBPA/TRPM2/PTPRC/LTBR/IL17A/TFE3/FCER1G/PPP3CA/GLI2/ITGB1/HMGB1/ZBTB7A/MMP14/CTSK/SLC25A5/FBN1/STAT3/SH3PXD2A/ABL1/EIF2AK1
GOBP_REGULATION_OF_TRANSPORT	GOBP_REGULATION_OF_TRANSPORT	GOBP_REGULATION_OF_TRANSPORT	356	-0.158358898	-1.775778486	0.000951132	0.014964062	0.011981685	1337	tags=51%, list=42%, signal=34%	SLC17A7/IER3/SRI/UCN/KCNAB3/OXSR1/DLG4/FYN/CYFIP1/SNX17/LLGL2/DIAPH1/PRAM1/CLASP2/TNF/ATP1A2/GHRH/IL15/STOM/THY1/IFI27/SYK/PROM2/KCNE3/PLTP/CCR1/ATP2A1/ACSL5/SV2A/SYP/SLC26A5/APOD/ATAD1/VPS18/SGIP1/EHD4/REEP2/AAK1/TMEM14A/NF1/CACNA1B/ACTN2/INHBA/CACNA1E/ARRB2/TM7SF3/FFAR2/BCR/NR1H2/GCLC/EMD/KCNS2/ATP8B1/FKBP1A/LGI3/ATP1B1/BCL2/GDI1/CACNB3/RPH3AL/WNT3A/FXYD5/MDM2/KCNAB2/HTR1B/CPLX2/ADIPOQ/FHL1/CCL5/CLCN2/CD4/VPS11/BAD/STIM1/PPARA/COLEC11/INS/TPCN1/CHMP6

												/PSMD9/B3GAT3/TMEM109/DNAJC13/HLA-F/KMO/SCN2B/MINK1/ALOX5/XBP1/CCL21/OAS2/ITGAM/KCNJ1/PYCARD/SERPINE2/CEBPB/GSTO1/PLSCR1/UCN3/NRP1/HCLS1/RHBDF2/BMP4/GHSR/VPS4A/WFS1/ITPR1/PKP2/KCNH4/BAX/HRH3/SYT12/CLDN3/CDK5R2/DAB2/CALM3/ANG/LYN/CLIC5/LLGL1/LIME1/SMO/CDH3/KCNB2/BBC3/SLC16A1/NTF3/RAB11A/RAB11FIP3/MAOB/OSBP/CD177/P2RX2/ATF4/GRIK5/KCNK5/AKT1/SCP2/PPIF/SLC11A1/RALA/CLN3/CSK/RAC1/CABP5/PTPRC/DNAJC5/ADRA2B/FCER1G/PPP3CA/PRAP1/NFE2L2/SUPT6H/PDGFRB/AP2M1/KCNB1/ITGB1/CDK5/UBQLN2/F2RL3/MSN/CNIH3/MBTPS1/BET1L/APLN/HMGB1/NFKB1/PHB2/ARHGDIA/CLIC1/TOR1A/UBE2L3/CHRNB2/NKX2-5/SLC25A5/RHOU/PTGES/TSPO/NADK/ITGB1BP1/ABL1/VDAC1
GOMF_ATP_HYDROLYSIS_ACTIVITY	GOMF_ATP_HYDROLYSIS_ACTIVITY	GOMF_ATP_HYDROLYSIS_ACTIVITY	80	0.258176709	2.165258513	0.000962462	0.015093153	0.012085048	803	tags=44%, list=25%, signal=34%		DDX20/KIF3A/RFC4/SMCHD1/HSPA1L/DHX16/HSPA14/ATAD2/RAD51/SMC1B/CLPB/DHX30/KATNA1/RECQL4/HSPA2/DNAH8/SMC3/BLM/RUVBL1/ATF7IP/TRIP13/KIF23/WRN/DDX52/CHD1L/RAD50/CLPX/ERCC3/RFC1/CHD5/KIF22/ATP8A2/MCM5/TOR2A/VCP
GOBP_REGULATION_OF_CELL_CYCLE	GOBP_REGULATION_OF_CELL_CYCLE	GOBP_REGULATION_OF_CELL_CYCLE	245	0.164785132	1.822950119	0.000968128	0.015132875	0.012116854	679	tags=31%, list=21%, signal=26%		ASNS/GMNN/SPAG5/DONSON/MDM4/CCNB2/FBXO7/SH3GLB1/CCNH/INTS7/MDC1/CSNK2A2/CKS2/CPSF3/CENPE/RAD51/CDC14B/AHCTF1/PAFAH1B1/WDR62/CDCA8/RANBP1/DOT1L/CDC25C/CEP63/RAE1/CRY1/DR1/AURKC/NF2/PNPT1/HSPA2/TPR/PAXIP1/PIWIL2/ANAPC10/CEP250/BLM/RUVBL1/TRIP13/GTPBP4/AURKAIP1/RAD17/C6orf89/BRD7/PAF1/MAGEA4/PINX1/KIF23/CDK5RAP2/TAF6/MAD2L1BP/TIMELESS/FZR1/

											DYNC1LI1/BUB1B/BBS4/CDC7/TFDP1/RAD50/CHEK2/RBM14/BARD1/BOP1/ESPL1/SMC6/BIRC8/GTF2H1/THOC5/ZBTB17/TPX2/E2F1/ATR/ERCC3/ZW10/CDK7
GOBP_NEGATIVE_REGULATION_OF_HEMOPOIESIS	GOBP_NEGATIVE_REGULATION_OF_HEMOPOIESIS	GOBP_NEGATIVE_REGULATION_OF_HEMOPOIESIS	25	-0.44290196	-2.220271979	0.000976157	0.015209152	0.012177929	1259	tags=84%, list=39%, signal=51%	LTF/CBFB/PTPN2/NF1/INHBA/PIAS3/SOCS1/C1QC/SNAI2/FGL2/ADIPOQ/MAFB/SMAD7/TCTA/MYC/BMP4/LYN/LAG3/CEBPA/HMGB1/FBN1
GOMF_PEPTIDE_BINDING	GOMF_PEPTIDE_BINDING	GOMF_PEPTIDE_BINDING	75	-0.272619826	-2.041179316	0.000994577	0.01544632	0.012367828	1782	tags=79%, list=56%, signal=36%	CD209/GPR37/HCRTR1/GLP1R/LRP1/NPBWR2/RAMP2/ECE1/KPNA1/MAPK8IP2/CD1A/CHRNA7/TAP2/MCHR1/MICA/GRIA4/CRIP1/CLSTN1/PPIH/MRGPRX2/SSTR3/BCHE/CD1B/CACNA1B/INHBA/HLA-C/CLSTN3/DHCR24/HLA-DMA/HLA-A/HLA-F/HLA-E/RELA/HLA-B/ITGAM/ITM2C/PPIC/GHSR/HLA-DOB/CRHR2/PCSK5/ANG/HLA-DRB5/HLA-DMB/AGER/FZD4/HLA-DQB1/TAP1/PPIF/ATP1A3/PPP3CA/HLA-DRB3/TOMM20/GPRASP2/AP2M1/PFDN1/SEC61A1/CLTB/MAML1
GOBP_SIGNAL_TRANSDUCTION_IN_ABSENCE_OF_LIGAND	GOBP_SIGNAL_TRANSDUCTION_IN_ABSENCE_OF_LIGAND	GOBP_SIGNAL_TRANSDUCTION_IN_ABSENCE_OF_LIGAND	18	-0.488517056	-2.141770046	0.001007037	0.015589707	0.012482638	1082	tags=78%, list=34%, signal=52%	NF1/INHBA/SRPX/BCL2/SNAI2/BAD/MKNK2/BAX/BCL2L1/AKT1/IFI6/PP2R1A/FGFR1/BCL2L2
GOBP_REGULATION_OF_FAT_CELL_DIFFERENTIATION	GOBP_REGULATION_OF_FAT_CELL_DIFFERENTIATION	GOBP_REGULATION_OF_FAT_CELL_DIFFERENTIATION	29	-0.417065058	-2.243212906	0.001014435	0.015654063	0.012534168	949	tags=62%, list=30%, signal=44%	WNT1/SNAI2/CCDC85B/WNT3A/ADIPOQ/CCDC3/INS/ALOX5/XBP1/CEBPB/LRP3/YAP1/SIRT2/CEBPA/TRIO/JDP2/AKT1/TFE3
GOBP_CELL_ACTIVATION_INVOLVED_IN_IMMUNE_RESPONSE	GOBP_CELL_ACTIVATION_INVOLVED_IN_IMMUNE_RESPONSE	GOBP_CELL_ACTIVATION_INVOLVED_IN_IMMUNE_RESPONSE	47	-0.335703363	-2.119315767	0.00102639	0.015737977	0.012601357	1330	tags=68%, list=42%, signal=40%	POU2AF1/MRGPRX2/PRAM1/TNF/SYK/NMI/BCR/FGL2/TSC1/CPLX2/SPN/MEN1/HLA-F/SMAD7/XBP1/ITGAM/PYCARD/EOMES/LYN/HLA-DMB/LFNG/TYROBP/CD177/SLC11A1/PTPRC/FCER1G/SUPT6H/HMGB1/HAVCR2/STAT3/EIF2AK4/ABL1
GOBP_TRANSMEMBRANE_RECEPTOR_PROTEIN_TYROSINE_KINASE_SIG	GOBP_TRANSMEMBRANE_RECEPTOR_PROTEIN_TYROSINE_KINASE_SIGNA	GOBP_TRANSMEMBRANE_RECEPTOR_PROTEIN_TYROSINE_KINASE_SIGNALING_PATHW	134	-0.218145043	-1.95018461	0.001026053	0.015737977	0.012601357	1319	tags=57%, list=41%, signal=35%	MYOC/PDGFRA/FYN/CYFIP1/TNXB/PTP4A3/GHRH/PTPN2/COL4A1/SYK/SULF1/APOD/IGFBP6/EFNA2/KALRN/CSHL1/GPR21/AGR2/COL4A6/M

NALING_PATHWAY	LING_PATHWAY	AY									PZL1/SOCS2/SOCS1/SAMD10/TGFA/WNT1/NRP2/HSPB1/NFATC4/ADIPOQ/PDK2/CD4/INS/CHMP6/RGS14/COL4A5/FRS2/RELA/VEGFB/GRB7/FAM20C/IGFBP4/INPPL1/CPNE3/NRP1/COL4A2/RHBDF2/TEK/GHSR/IQGAP1/DOK4/RAPGEF1/LYN/FZD4/CDH3/IGF2R/NTF3/FGF1/IGF2/AKT1/IFI6/RALA/RAC1/ARHGEF7/SLC31A1/PDGFRB/STUB1/APLN/MAPKAPK3/ADIPOR1/TYK2/PPP2R5B/STAT3/FGFR1/SHC1/EFNB1/ABL1
GOMF_ANTIGEN_BINDING	GOMF_ANTIGEN_BINDING	GOMF_ANTIGEN_BINDING	25	-0.4394365	-2.202899593	0.001054248	0.015962434	0.012781079	829	tags=68%, list=26%, signal=51%	CD1B/HLA-C/DHCR24/HLA-DMA/HLA-A/HLA-F/HLA-E/HLA-B/HLA-DOB/HLA-DRB5/HLA-DMB/LAG3/HLA-DQB1/TAP1/HLA-DRB3/PPP2R1A/MAML1
GOBP_CELL_JUNCTION_ASSEMBLY	GOBP_CELL_JUNCTION_ASSEMBLY	GOBP_CELL_JUNCTION_ASSEMBLY	100	-0.249040871	-2.025059772	0.001052683	0.015962434	0.012781079	1325	tags=58%, list=41%, signal=35%	SH3BP1/MYOC/CLSTN1/PCDHB14/CLDN4/CLASP2/TNF/CDH9/THY1/APOD/AMIGO3/WNT11/IRX3/ACTN2/CLDN5/PLXNB2/BCR/BCL2/PCDHB4/SNAI2/CLSTN3/ZNF703/TSC1/WNT3A/PRKCH/LRRN3/FAM107A/PCDHB16/ABI3/CDH4/CORO2B/SMAD7/NRP1/TEK/GHSR/GJB1/PKP2/CLDN3/CDH11/RAPGEF1/CNTNAP1/CDH3/GJA4/AMIGO2/RAC1/ARHGEF7/IL17A/LRFN3/CTTN/CDK5/MMP14/RHOC/CHRNB2/CLDN15/TRIP6/ITGB1BP1/EIF4G1/ABL1
GOCC_NUCLEOLUS	GOCC_NUCLEOLUS	GOCC_NUCLEOLUS	198	0.18356791	1.962079794	0.001053979	0.015962434	0.012781079	965	tags=46%, list=30%, signal=34%	PWP1/SURF2/ZNF692/CCDC86/LRRC34/PRKRIP1/STAG3/SETX/MTX2/GEMIN4/RAD51/CDC14B/NARF/ELL3/CDCA8/TRAIP/EBNA1BP2/UBE2T/RAE1/SPECC1/NF2/CENPH/EXOSC8/MAPKBP1/BLM/PPP1CC/SUZ12/GTPBP4/RAD17/C6orf89/ACTR6/TFIP11/UTP18/PAF1/PINX1/ZFP91/VRK1/SRP19/NIP7/SENP5/MAD2L1BP/ATXN3/AFF4/CARF/WRN/DDX52/TEX10/PNMA1/PA2G4/CAPN3/DNTTIP1/RBM14/BOP1/TTF1/RGS22/TNP2/EXOS

											C4/NOL6/NHEJ1/CDK7/MAP3K14/AKAP11/DNTTIP2/WDR33/MRPS31/NUSAP1/ZNF415/PARP1/ERGIC2/UBLCP1/CDCA7L/PIM1/KIT/ARL2/PDK3/RBBP6/IPPK/KLHL7/CUL2/MCM3/FBXO11/POP4/NANOG/ZCCHC9/SIRT1/PLRG1/POLR1C/ZNF330/DHX15/RNMT/EN2
GOCC_NUCLEAR_BODY	GOCC_NUCLEAR_BODY	GOCC_NUCLEAR_BODY	163	0.189101186	1.898961646	0.001053097	0.015962434	0.012781079	772	tags=36%, list=24%, signal=29%	SRPK1/DDX20/SPAG5/KLF11/SURF2/DZIP1/BRD1/INTS7/MDC1/SETX/GEMIN4/RAD51/AHCTF1/PCNA/ZNF473/ELL3/TESK2/PHF7/PRPF18/GEMIN6/PRKAA1/PRPF40B/SF3A1/UBN1/BLM/PPP1CC/ATF7IP/SUZ12/TFIP11/MAGEA2B/BANP/ZC3H8/SRP19/USPL1/WRN/LPXN/CNOT7/TOP3A/DGKZ/GTF2H2/S100PBP/CHEK2/RBM14/PARP11/BARD1/SMC6/CBX1/YLPM1/ATR/RMI1/CHD5/RCHY1/KIF22/PRPF40A/AKAP8L/MBD4/KLHDC2/SAP130/SETD1A
GOBP_REGULATION_OF_LEUKOCYTE_PROLIFERATION	GOBP_REGULATION_OF_LEUKOCYTE_PROLIFERATION	GOBP_REGULATION_OF_LEUKOCYTE_PROLIFERATION	51	-0.317116683	-2.084437864	0.001060198	0.01598599	0.012799941	978	tags=55%, list=31%, signal=39%	BCL2/TNFRSF21/WNT3A/CCL5/CD6/HLA-A/SPN/BTN2A2/HLA-E/PYCARD/CEBPB/TNFRSF4/AIF1/BMP4/LYN/HLA-DMB/AGER/TYROBP/IGF2/BCL2L1/SCGB1A1/PTPRC/PPP3CA/HMGB1/HAVCR2/CHRNB2/TYK2/EFNB1
GOBP_CELLULAR_HOMEOSTASIS	GOBP_CELLULAR_HOMEOSTASIS	GOBP_CELLULAR_HOMEOSTASIS	160	-0.206511805	-1.931503599	0.001062423	0.01598599	0.012799941	1356	tags=55%, list=42%, signal=33%	JAGN1/CCL8/SRI/SYPL2/OXSR1/GATA4/CYP7A1/DIAPH1/MT1H/RAB20/ATP1A2/ATP6V1G2/CREG1/THY1/CLCN6/KCNE3/CCR1/CROCC/ATP2A1/SV2A/MT1E/GPR12/ATP2B3/NUBP1/FXN/MT1B/GCLC/ATP7B/FKBP1A/ATP6V0A1/ATP1B1/BCL2/SLC12A8/NQO1/ABCB7/HTR1B/CCL5/IBTK/CCR5/BAD/STIM1/TPCN1/PRDX1/ATP13A1/PRDX3/XBP1/CCL21/SLC9A8/CYBRD1/GSTO1/MYC/UBTF/WFS1/ITPR1/BAX/CALM3/LYN/SCARA5/SLC12A9/AGER/LIME1/SLC25A23/ANXA6/GRM1/XCR1/OSBP/TRPM2/ATF

											4/SLC11A1/CLN3/SLC34A2/CLNS1A/PTPRC/ATP1A3/PPP3CA/SLC29A1/SLC31A1/NFE2L2/CCDC51/SLC30A9/KCNB1/CFL2/F2RL3/ATP2A3/CCDC115/NADK/ACO1/ABL1
GOBP_CELLULAR_CATABOLIC_PROCESS	GOBP_CELLULAR_CATABOLIC_PROCESS	GOBP_CELLULAR_CATABOLIC_PROCESS	316	-0.162760897	-1.786669379	0.001071305	0.016069573	0.012866865	898	tags=36%, list=28%, signal=29%	FASTK/HSPB1/ABHD6/ADIPOQ/GNPDA1/VPS11/BAD/FMO1/OAT/PPARA/INS/TPCN1/ABHD12/CHMP6/ABCD4/PRDX1/CAT/KMO/ECHDC1/ACOX1/BTG2/TIMP1/TGFBRAP1/ATG16L2/GCSH/PRDX3/HP/NTHL1/ALDH4A1/XBP1/FXR2/OAS2/SORD/PLA2G4C/GLUD2/PYCARD/GSTO1/ALDH2/WIPI1/PUM1/VPS4A/GLB1/PIPOX/ITPR1/BAX/BLVRA/RNF5/SIRT2/ENTPD2/ANG/TFEB/GALC/ZNF418/RAB1A/SLC16A1/ECHS1/MAOB/RNF185/GRAMD1A/FYCO1/SDSL/AKT1/SCGB1A1/SCP2/SLC11A1/WDR6/CLN3/CHMP7/ITPA/TM9SF1/DHRSX/ELAVL1/CTTN/CYP2W1/SUCLG2/ADHFE1/NFE2L2/AKR1A1/STUB1/GOT2/ACAA1/CDK5/UBQLN2/PNRC1/DNASE1L2/ABCD1/ZFYVE1/HMGB1/DCPS/PHB2/ATG4A/MEFV/CTSK/ENTPD8/NUDT16/TRIM8/WDR81/SLC25A5/ENTPD6/ILVBL/STAT3/ZMPSTE24/AIG1/ECH1/BCAT2/TSPO/SGSH/EFNB1/NT5C/GTPBP1/EIF4G1/ABL1/SEC22B/HMGCL/VDAC1
GOMF_HELICASE_ACTIVITY	GOMF_HELICASE_ACTIVITY	GOMF_HELICASE_ACTIVITY	34	0.356489716	2.156563054	0.001090059	0.01625464	0.013015048	1072	tags=68%, list=33%, signal=45%	DDX20/RFC4/DHX16/SETX/RAD51/DHX30/RECQL4/BLM/RUVBL1/WRN/DDX52/CHD1L/RAD50/ERCC3/CHD5/MCM5/MCM3/DHX35/DDX49/DHX15/EP400/DDX11/EIF4A1
GOBP_REGULATION_OF_CELL_DEVELOPMENT	GOBP_REGULATION_OF_CELL_DEVELOPMENT	GOBP_REGULATION_OF_CELL_DEVELOPMENT	155	-0.207764388	-1.928511453	0.001090373	0.01625464	0.013015048	1259	tags=54%, list=39%, signal=34%	LTF/TNF/CBFB/TBX6/IL15/PTPN2/THY1/SYK/CCR1/ZNF488/NF1/RARG/INHBA/CLDN5/MYOG/PLXNB2/PIAS3/SOCS1/TRAK1/MYOD1/BCL2/GDI1/C1QC/SNAI2/FGL2/WNT3A/ETS1/NFATC4/ADIPOQ/CLCN2/CD4/BAD/PRKCH/INS/MAFB/RGS14/ADD1/BT

GOCC_TRANSFERASE_COMPLEX_TRANSFERRING_PHOSPHORUS_CONTAINING_GROUPS	GOCC_TRANSFERASE_COMPLEX_TRANSFERRING_PHOSPHORUS_CONTAINING_GROUPS	GOCC_TRANSFERASE_COMPLEX_TRANSFERRING_PHOSPHORUS_CONTAINING_GROUPS	75	0.257417702	2.11070642	0.001108123	0.016468411	0.013186214	755	tags=41%, list=24%, signal=32%	G2/BTN2A2/CDH4/SMAD7/XBP1/RELA/HLA-B/TCTA/FBXW8/SERPINE2/CEBPB/MYC/YAP1/CUL7/NRP1/HCLS1/BMP4/ISG15/SIRT2/LYN/DAB1/AGER/PITX3/SMO/FZD4/PLXNA1/TYROBP/LAG3/CEBPA/PLAG1/AKT1/PTPRC/IL17A/TFE3/PPP3CA/GLI2/ITGB1/CDK5/NAP1L1/HMGB1/MMP14/FBN1/STAT3/PTN/TSPO/ABL1 TBPL1/CCNB2/CCNH/POLG2/AKAP4/PARD6A/CSNK2A2/CKS2/TSSK2/PCNA/PRKAA1/PRIM1/POLRMT/SUPT3H/POLA2/PAF1/PRKAG2/TAF6/CAB39/TAF10/GTF2H2/POLE/TBK1/GTF2H1/PIK3CG/POLR3E/ERCC3/CDK7/MAD2L2/GTF2E1/TAF9
GOMF_3_5_EXONUCLEASE_ACTIVITY	GOMF_3_5_EXONUCLEASE_ACTIVITY	GOMF_3_5_EXONUCLEASE_ACTIVITY	10	0.632786263	2.276474927	0.001117297	0.016553817	0.013254598	860	tags=90%, list=27%, signal=66%	PNPT1/POLRMT/WRN/CNOT7/RAD50/POLE/EXOSC4/CNOT6/ANGEL1
HP_SUBCUTANEOUS_NODULE	HP_SUBCUTANEOUS_NODULE	HP_SUBCUTANEOUS_NODULE	21	-0.464496713	-2.140573108	0.001139942	0.01678634	0.013440779	1241	tags=81%, list=39%, signal=50%	CYLD/CCR1/BTNL2/TSC1/MDM2/HLA-B/TEK/ANTXR2/COL3A1/AKT1/PDGFRB/CDK4/MMP14/MEFV/IFNGR1/FBN1/FGFR1
HP_ABNORMALITY_OF_LIMB_BONE	HP_ABNORMALITY_OF_LIMB_BONE	HP_ABNORMALITY_OF_LIMB_BONE	339	-0.159625772	-1.777243343	0.001138929	0.01678634	0.013440779	614	tags=28%, list=19%, signal=26%	INPPL1/B4GALT7/CUL7/GNB1/GNAI1/PTCH2/SLC25A12/PUM1/SLC32A1/RHBDF2/BMP4/H19/EDA/GLB1/WFS1/COL6A3/SLURP1/ZNF462/HEATR3/CDH11/LFNG/GNE/TPM2/SLC29A3/CNTNAP1/SMO/PRX/SLC35B2/COX7B/PNKP/CDH3/SLC6A17/TRIO/ANTXR2/COL3A1/PROKR2/ANKRD11/PQBP1/IGF2/CTSC/PLAG1/AKT1/RALA/MGP/OSGEP/SLC34A2/TBC1D2B/RAC1/COL27A1/HS6ST1/TFE3/MAF/CDC42BPB/PPP3CA/SLC31A1/PPP2R1A/GLI2/PRR12/PDGFRB/STUB1/AP2M1/MED12/CFL2/FBXW11/SH3PXD2B/IMPDH2/MBTPS1/ABCD1/SETBP1/MMP23B/DCPS/MMP14/SSR4/TOR1A/CTSK/IFNGR1/NFKBIL1/NKX2-5/SUMF1/TRIM8/WDR81/CCDC8/FBN1/PURA/ZMPSTE24/DPH5/FGFR1/EI

GOMF_INTRAMOLECULAR_OXIDOREDUCTASE_ACTIVITY	GOMF_INTRAMOLECULAR_OXIDOREDUCTASE_ACTIVITY	GOMF_INTRAMOLECULAR_OXIDOREDUCTASE_ACTIVITY	12	-0.590654143	-2.209212424	0.001150084	0.016884208	0.013519142	418	tags=67%, list=13%, signal=58%	F2AK4/KRT14/PLOD1/RNF113A/CTBP1/FAM50A/EFNB1/ABL1/PDXK ECHS1/HYI/PTGES2/PDIA6/PDIA5/TXNDC5/ECH1/PTGES
GOBP_RESPONSE_TO_CYTOKINE	GOBP_RESPONSE_TO_CYTOKINE	GOBP_RESPONSE_TO_CYTOKINE	189	-0.196078342	-1.893579316	0.00115557	0.016913345	0.013542472	1254	tags=51%, list=39%, signal=33%	PTP4A3/RAB20/TNF/CYLD/IL15/PTPN2/IFI27/SYK/CCL22/CCR1/CTSG/UGCG/SELPLG/RARG/INHBA/MYOG/NR1H2/GCLC/MRAS/MAP3K5/PIAS3/DUOX2/SOCS1/CYP1B1/MYOD1/BCL2/TNFRSF21/NRP2/IL1R1/CXCL13/CXCR3/ADIPOQ/CCDC3/CCL5/CD4/CCR5/BAD/B3GNT2/ILK/ABCD4/AANAT/KMO/FOSL2/TIMP1/SMAD7/KLF2/OTOP1/XBP1/RELA/CCL21/OAS2/PYCARD/PLSCR1/YAP1/TNFRSF4/AIF1/HCLS1/ALAD/ISG15/LYN/TRIM41/IFITM1/ADIPOR2/FZD4/CEBPA/XCR1/RNF185/COL3A1/HAS2/BCL2L1/AKT1/SCGB1A1/SLC11A1/LSP1/PTPRC/IL17A/FCER1G/CASP4/NFE2L2/EIF2AK2/IMPDH2/CDK4/IRAK1/NFKB1/MAPKAPK3/TRAF1/MEFV/ADIPOR1/CTSK/IFNGR1/TYK2/SLC25A5/IFITM3/STAT3/BCAT2/RNF113A/LSM14A
GOCC_ORGANELLE_MEMBRANE_CONTACT_SITE	GOCC_ORGANELLE_MEMBRANE_CONTACT_SITE	GOCC_ORGANELLE_MEMBRANE_CONTACT_SITE	11	-0.585304659	-2.116166438	0.001160203	0.016929857	0.013555693	508	tags=64%, list=16%, signal=54%	CLCC1/GRAMD1A/CHCHD3/TOMM20/ZFYVE1/OSBPL5/OSBPL1A
GOBP_SCHWANN_CELL_DIFFERENTIATION	GOBP_SCHWANN_CELL_DIFFERENTIATION	GOBP_SCHWANN_CELL_DIFFERENTIATION	12	-0.590595611	-2.208993497	0.001166397	0.016968964	0.013587006	1319	tags=100%, list=41%, signal=59%	LGI4/NF1/NDRG1/ILK/RELA/SIRT2/CNTNAP1/PRX/AKT1/CDK5/MED12
GOBP_POSITIVE_REGULATION_OF_ERK1_AND_ERK2_CASCADE	GOBP_POSITIVE_REGULATION_OF_ERK1_AND_ERK2_CASCADE	GOBP_POSITIVE_REGULATION_OF_ERK1_AND_ERK2_CASCADE	36	-0.371249332	-2.173149376	0.001188164	0.017233737	0.013799008	1683	tags=86%, list=53%, signal=41%	FGF19/CARD9/BMP2/MFHAS1/CHRNA7/CCL8/PDGFRA/GATA4/TNF/CCL22/CCR1/FSHR/INHBA/ARRB2/HAND2/CCL5/CD4/CCL21/PYCARD/NRP1/BMP4/TEK/AGER/FGF1/PTPRC/PDGFRB/HMGB1/PHB2/HAVCR2/SHC1/ABL1
GOBP_SEQUESTERING_OF_CALCIUM	GOBP_SEQUESTERING_OF_CALCIUM	GOBP_SEQUESTERING_OF_CALCIUM_ION	31	-0.393750835	-2.14567781	0.00121043	0.017504121	0.014015504	1331	tags=74%, list=42%, signal=44%	SRI/DIAPH1/ATP1A2/THY1/ATP7B/FKBP1A/IBTK/CCR5/TPCN1/CCL21/G

_ION	_ION									STO1/ITPR1/BAX/CALM3/LYN/LIME1/SLC25A23/ANXA6/XCR1/TRPM2/PTPRC/F2RL3/ABL1
GOBP_REGULATION_OF_METAL_ION_TRANSPORT	GOBP_REGULATION_OF_METAL_ION_TRANSPORT	GOBP_REGULATION_OF_METAL_ION_TRANSPORT	70	-0.277639813	-2.018465178	0.001226811	0.017688045	0.014162771	1331	tags=63%, list=42%, signal=38%	SRI/UCN/KCNAB3/OXSR1/FYN/DIAPH1/ATP1A2/THY1/KCNE3/CCR1/ATP2A1/ACTN2/KCNS2/FKBP1A/ATP1B1/BCL2/CACNB3/FXYD5/KCNAB2/FHL1/CCL5/CD4/STIM1/SCN2B/SERPINE2/GSTO1/WFS1/PKP2/BAX/CALM3/LYN/LIME1/P2RX2/ATF4/AKT1/CABP5/PPP3CA/PDGFRB/ITGB1/F2RL3/NKX2-5/TSPO/ABL1/VDAC1
GOCC_RECEPTOR_COMPLEX	GOCC_RECEPTOR_COMPLEX	GOCC_RECEPTOR_COMPLEX	74	-0.274178942	-2.039047734	0.001235885	0.017765853	0.014225072	1912	tags=81%, list=60%, signal=33%	GP1BB/PIGR/GRIN1/INSRR/LRP1B/HTR3A/AIP/IL2RG/GPR37/LRP1/RAMP2/TSPAN32/BMP2/GABRR1/ITGA5/CHRNA7/TRAF5/CR2/MERTK/GRIA4/PDGFRA/DLG4/ENG/SYK/GPRC5B/GPR119/FSHR/CHRNA2/PLXNB2/AMN/IMPG2/NRP2/ABHD6/HTR1B/CD4/ITGB8/CD79A/ABHD12/ITGAM/NRP1/TEK/LYN/LIME1/MTNR1A/PLXNA1/GRM1/P2RX2/GRIK5/GFRA3/ITGA3/PDGFRB/ITGB1/CNIH3/TRAF1/ITGA9/ITGB7/CHRNB2/TYK2/KCTD12/FGFR1
GOBP_POSITIVE_REGULATION_OF_REACTIVE_OXYGEN_SPECIES_METABOLIC_PROCESS	GOBP_POSITIVE_REGULATION_OF_REACTIVE_OXYGEN_SPECIES_METABOLIC_PROCESS	GOBP_POSITIVE_REGULATION_OF_REACTIVE_OXYGEN_SPECIES_METABOLIC_PROCESS	16	-0.517832004	-2.156178096	0.00125534	0.017991963	0.014406118	1216	tags=88%, list=38%, signal=55%	SYK/DCXR/CYP1B1/DHRS4/ITGAM/H19/TYROBP/CD177/AKR1C1/NFE2L2/PDGFRB/CBR1/GADD45A/TSPO
GOBP_DEFENSE_RESPONSE_TO_OTHER_ORGANISM	GOBP_DEFENSE_RESPONSE_TO_OTHER_ORGANISM	GOBP_DEFENSE_RESPONSE_TO_OTHER_ORGANISM	211	-0.181015735	-1.805901165	0.00126325	0.018051775	0.014454009	1057	tags=44%, list=33%, signal=31%	TRIM4/ARRB2/FFAR2/HLA-C/NR1H2/MAP3K5/ATP1B1/COTL1/TRAFD1/BCL2/NQO1/C1QC/FBXO9/FGL2/CXCL13/UNC93B1/CCL5/ADAMTS5/CD4/MPEG1/CD6/COLEC11/INS/HLA-A/PRDX1/SPN/HLA-F/UBL7/KIR2DL4/FOSL2/HP/HLA-E/OTOP1/RELA/CCL21/HLA-B/OAS2/ITGAM/CFHR5/PYCARD/CEBPB/PLSCR1/PUM1/AIF1/ISG15/SIRT2/ANG/LYN/TFEB/TRIM41/STAB1/IFITM1/ZNFX1/RAB1A/IFIT3/FLOT2/TYROBP/LAG3/LY86/RNF18

											5/CD177/PQBP1/BCL2L1/PI3/AKT1/DEFB105A/IFI6/SLC11A1/PTPRC/IL17A/FCER1G/CASP4/NFE2L2/EIF2AK2/C8A/ADAM15/IRAK1/HMGB1/NFKB1/PHB2/MAPKAPK3/HAVCR2/MEFV/IFNGR1/NFKBIL1/TYK2/TRIM8/IFITM3/ZMPSTE24/EIF2AK4/SHC1/LSM14A
GOBP_MONOATOMIC_CATION_TRANSMEMBRANE_TRANSPORT	GOBP_MONOATOMIC_CATION_TRANSMEMBRANE_TRANSPORT	GOBP_MONOATOMIC_CATION_TRANSMEMBRANE_TRANSPORT	164	-0.205166331	-1.929120972	0.001268391	0.018071765	0.014470015	1657	tags=66%, list=52%, signal=34%	SLC25A22/KCNH7/ATG5/TRPV1/KCNMB3/SLC12A3/KCNQ3/TMEM163/ATP1B2/TRPV5/KCNS3/SLC41A3/CAV3/HCN3/CHRNA7/SLC17A8/SLC34A3/NIPA2/KCNJ6/SLC25A37/SLC17A7/SRI/KCNAB3/OXSR1/FYN/DIAPH1/LTF/ATP1A2/TMEM63A/ATP6V1G2/THY1/KCNE3/ATP2A1/CACNA1B/ACTN2/CACNA1E/ATP2B3/FXN/ATP7B/KCNS2/FKBP1A/ATP6V0A1/ATP1B1/BCL2/SLC12A8/CACNB3/WNT3A/FXYD5/ABCB7/KCNAB2/FHL1/IBTK/CCR5/STIM1/TPCN1/TMCO3/CNNM2/SCN2B/ATP13A1/OTOP1/CCL21/SLC9A8/KCNJ1/GSTO1/SLC25A12/SLC32A1/ITPR1/KCNH4/BAX/CALM3/LYN/SCARA5/SLC12A9/OTOP2/LIME1/COX7B/SLC25A23/SLC6A17/KCNB2/ANXA6/SLC16A1/XCR1/COX15/TRPM2/P2RX2/KCNK5/PPIF/SLC11A1/SLC34A2/CABP5/PTPRC/ATP1A3/OTOP3/PPP3CA/SLC31A1/CCDC51/SLC30A9/KCNB1/ITGB1/F2RL3/SEC61A1/PHB2/STEAP3/COX7A1/SLC25A5/ATP2A3/COX8A/ABL1/VDAC1
GOMF_MICROTUBULE_BINDING	GOMF_MICROTUBULE_BINDING	GOMF_MICROTUBULE_BINDING	53	0.28950803	2.116225286	0.001273345	0.018088986	0.014483804	729	tags=43%, list=23%, signal=34%	KIF3A/SPAG5/STRBP/SPAG8/CENPE/PAFAH1B1/MAST2/POLB/KATNA1/KATNB1/RAE1/NDE1/CEP350/KIF23/CDK5RAP2/SSNA1/KIFC3/MAP6D1/KIF4A/KRIT1/HOOK2/KIF22/CEP290
GOBP_OSTEOBLAST_DIFFERENTIATION	GOBP_OSTEOBLAST_DIFFERENTIATION	GOBP_OSTEOBLAST_DIFFERENTIATION	49	-0.331048313	-2.129385011	0.001297508	0.018378194	0.014715372	1319	tags=67%, list=41%, signal=40%	MYOC/LTF/TNF/CBFB/RPS15/JUND/WNT11/NF1/HAND2/SNAI2/WNT3A/ILK/MEN1/CAT/DNAJC13/FOSL2/TMEM119/FAM20C/CEBPB/LRP3/YA

											P1/BMP4/IFITM1/SMO/CEBPA/CHRD/IGF2/ATF4/AKT1/PPP3CA/GLI2/MEF2D/CLIC1
GOBP_RESPONSE_TO_ALCOHOL	GOBP_RESPONSE_TO_ALCOHOL	GOBP_RESPONSE_TO_ALCOHOL	65	-0.290839736	-2.06484283	0.001327937	0.018754195	0.015016435	1076	tags=55%, list=34%, signal=38%	INHBA/CLDN5/CYP1B1/NQO1/MDM2/HTR1B/ADIPOQ/LIPA/CCR5/PPARA/CAT/AANAT/KLF2/CCL21/UCN3/GNB1/GNAI1/GLB1/ALAD/CLDN3/PMVK/FECH/SMO/MAOB/GRAMD1A/BCL2L1/AKT1/PTPRC/GOT2/ADAM15/CDK4/CTSK/CHRNB2/KLF9/TSPO/EIF4G1
GOBP_ORGANIC_ACID_CATABOLIC_PROCESS	GOBP_ORGANIC_ACID_CATABOLIC_PROCESS	GOBP_ORGANIC_ACID_CATABOLIC_PROCESS	63	-0.290268337	-2.037980429	0.001336688	0.018822744	0.015071322	871	tags=49%, list=27%, signal=37%	DAO/ADIPOQ/GNPDA1/FMO1/OAT/PPARA/ABCD4/KMO/ECHDC1/ACOX1/GCSH/ALDH4A1/SORD/GLUD2/PIPOX/SLC16A1/ECHS1/SDSL/AKT1/SCP2/CYP2W1/ADHFE1/AKR1A1/GOT2/ACAA1/ABCD1/ILVBL/AIG1/ECH1/BCAT2/HMGCL
GOBP_SKELETAL_SYSTEM_DEVELOPMENT	GOBP_SKELETAL_SYSTEM_DEVELOPMENT	GOBP_SKELETAL_SYSTEM_DEVELOPMENT	96	-0.250783208	-2.014579808	0.001379726	0.019372309	0.015511357	860	tags=44%, list=27%, signal=33%	LIPA/OGN/ITGB8/HSD17B1/CITED2/PRDX1/LOXL2/FOSL2/TIMP1/SMAD7/TMEM119/FAM20C/TGFBI/INPPL1/BMP4/TEK/CDH11/PCSK5/ETS2/ANXA6/TYROBP/CHRD/COL3A1/HAS2/OTOR/ANKRD11/MGP/PTPRC/COL27A1/MAF/GLI2/NFIA/MED12/SH3PXD2B/MEF2D/MMP14/EXTL1/CTSK/PRELP/FBN1/ZMPSTE24/FGFR1
GOBP_NEGATIVE_REGULATION_OF_MITOTIC_CELL_CYCLE_PHASE_TRANSITION	GOBP_NEGATIVE_REGULATION_OF_MITOTIC_CELL_CYCLE_PHASE_TRANSITION	GOBP_NEGATIVE_REGULATION_OF_MITOTIC_CELL_CYCLE_PHASE_TRANSITION	43	0.327144595	2.208140287	0.001399097	0.01958736	0.015683548	742	tags=51%, list=23%, signal=40%	DONSON/FBXO7/CDC14B/CDCA8/TPR/BLM/TRIP13/RAD17/BRD7/PINX1/CDK5RAP2/MAD2L1BP/FZR1/DYNC1LI1/BUB1B/RAD50/CHEK2/BARD1/ZW10/MAD2L2/RINT1/DCUN1D3
GOBP_SIGNAL_RELEASE	GOBP_SIGNAL_RELEASE	GOBP_SIGNAL_RELEASE	81	-0.26328131	-2.011752181	0.001424962	0.01989181	0.015927321	1356	tags=62%, list=42%, signal=37%	JAGN1/SRI/UCN/TNF/GHRH/SYK/SV2A/GPR119/SYP/VPS18/STX3/NF1/INHBA/TM7SF3/FFAR2/RPH3AL/HTR1B/CPLX2/ADIPOQ/CCL5/BAD/INS/PSMD9/KMO/ALOX5/UCN3/SLC32A1/GHSR/ITPR1/HRH3/SYT12/DAB2/PCSK5/LYN/FZD4/RAB1A/SLC16A1/RAB11FIP3/MAOB/OSBP/GRIK5/DNAJC5/ADRA2B/PPP3CA/KCNB1/CDK5/CHRNB2/PTGES/TSPO/NADK

GOCC_ENDOCYTIC_VESICLE	GOCC_ENDOCYTIC_VESICLE	GOCC_ENDOCYTIC_VESICLE	75	-0.265046365	-1.98447474	0.001443251	0.020089053	0.016085252	1129	tags=56%, list=35%, signal=37%	SGIP1/EHD4/ARRB2/HLA-C/ATP6V0A1/TGFA/AMN/WNT1/WNT3A/SCGB3A2/UNC93B1/MDM2/CD4/VPS11/TGOLN2/MPEG1/HLA-A/M6PR/HLA-F/HP/HLA-E/HLA-B/HCLS1/HLA-DRB5/LYN/STAB1/SMO/FZD4/IGF2R/FLOT2/RAB11A/RAB11FIP3/HLA-DQB1/TAP1/SLC11A1/RALA/CTTN/HLA-DRB3/AP2M1/SCARB2/CLTB/SEC22B
GOBP_NEGATIVE_REGULATION_OF_CHROMOSOME_ORGANIZATION	GOBP_NEGATIVE_REGULATION_OF_CHROMOSOME_ORGANIZATION	GOBP_NEGATIVE_REGULATION_OF_CHROMOSOME_ORGANIZATION	22	0.433203682	2.195822606	0.001462987	0.020305248	0.016258359	808	tags=64%, list=25%, signal=48%	CDCA8/TPR/TRIP13/PINX1/CDK5RAP2/MAD2L1BP/DYNC1LI1/BUB1B/RAD50/ESPL1/ZW10/MAD2L2/POT1/PARP1
HP_X_LINKED_INHERITANCE	HP_X_LINKED_INHERITANCE	HP_X_LINKED_INHERITANCE	60	-0.294758358	-2.037938546	0.001497593	0.020726001	0.016595255	1988	tags=87%, list=62%, signal=33%	OPN1LW/NKAP/SLC6A8/SMC1A/FGF13/CASK/DDX3X/BCAP31/LAS1L/SYN1/IL2RG/F8/TBL1X/DKC1/TIMM8A/HUWE1/CFP/ARR3/STAG2/BRWD3/WAS/NSDHL/AP1S2/SLITRK2/LAGE3/FTSJ1/SYP/ZDHHC9/ATP2B3/COL4A6/EMD/GDI1/ABCB7/FHL1/COL4A5/EDA/GJB1/COX7B/PQBP1/PRPS1/TFE3/MID1/GPRASP2/UBQLN2/MED12/MSN/PGK1/ABCD1/SSR4/RNF113A/FAM50A/EFNB1
HP_NAIL_DYSTROPHY	HP_NAIL_DYSTROPHY	HP_NAIL_DYSTROPHY	24	-0.427831017	-2.100706609	0.001577207	0.021580487	0.01727944	493	tags=50%, list=15%, signal=43%	AIRE/KRT85/COX7B/PQBP1/CTSC/PERP/MAF/ITGA3/STAT3/ZMPSTE24/KRT14/RNF113A
GOMF_GROWTH_FACTOR_BINDING	GOMF_GROWTH_FACTOR_BINDING	GOMF_GROWTH_FACTOR_BINDING	24	-0.427821946	-2.100662072	0.001577207	0.021580487	0.01727944	1515	tags=83%, list=47%, signal=44%	LTBP1/IL9R/PDGFRA/ENG/COL4A1/TRIM16/IGFBP6/NRP2/IL1R1/CXCL13/IGFBP4/NRP1/RHBDF2/TEK/HTRA1/IGF2R/COL3A1/PDGFRB/FGFR1/SHC1
GOBP_REGULATION_OF_EXTRINSIC_APOPTOTIC_SIGNALING_PATHWAY	GOBP_REGULATION_OF_EXTRINSIC_APOPTOTIC_SIGNALING_PATHWAY	GOBP_REGULATION_OF_EXTRINSIC_APOPTOTIC_SIGNALING_PATHWAY	35	-0.358078755	-2.063356144	0.001577207	0.021580487	0.01727944	1249	tags=69%, list=39%, signal=42%	TNF/CYLD/NF1/INHBA/GCLC/SRPX/BCL2/SNAI2/RELA/ITM2C/PYCARD/YAP1/NRP1/BMP4/TNFSF10/BCL2L1/AKT1/IFI6/PTPRC/LTBR/CTTN/PPP2R1A/TRAF1/FGFR1
GOBP_POSITIVE_REGULATION_OF_TUMOR_NECROSIS_FACTOR_SUPERFA	GOBP_POSITIVE_REGULATION_OF_TUMOR_NECROSIS_FACTOR_SUPERFA	GOBP_POSITIVE_REGULATION_OF_TUMOR_NECROSIS_FACTOR_SUPERFAMILY_CYTO	23	-0.417234543	-2.007751629	0.001563954	0.021580487	0.01727944	928	tags=65%, list=29%, signal=47%	ORM2/HSPB1/NFATC4/SPN/HLA-E/OAS2/PYCARD/AGER/TYROBP/PTPRC/IL17A/HMGB1/HAVCR2/IFNGR1/STAT3

MILY_CYTOKINE_PRODUCTION	MILY_CYTOKINE_PRODUCTION	KINE_PRODUCTION									
HP_ABNORMALITY_OF_THE_METOPIC_SUTURE	HP_ABNORMALITY_OF_THE_METOPIC_SUTURE	HP_ABNORMALITY_OF_THE_METOPIC_SUTURE	20	-0.455157695	-2.090852784	0.001603192	0.021874067	0.017514508	540	tags=50%, list=17%, signal=42%	ZNF462/IGF2/MAF/PPP2R1A/NFIA/MID1/SETBP1/PURA/STAT3/FGFR1
GOBP_NEGATIVE_REGULATION_OF_MOLECULAR_FUNCTION	GOBP_NEGATIVE_REGULATION_OF_MOLECULAR_FUNCTION	GOBP_NEGATIVE_REGULATION_OF_MOLECULAR_FUNCTION	135	-0.211977495	-1.898753447	0.001609387	0.021896723	0.017532649	1437	tags=59%, list=45%, signal=34%	SERPINH1/PRKAR1A/MDFI/PALM/ANXA4/SFN/SRI/OXSR1/ZNF593/MITD1/LTF/TNF/TBX6/CYLD/ATP1A2/LRP6/THY1/KCNE3/PPP1R15A/ANGPTL4/NF1/ACTN2/SERPINB10/ARRB2/NEIL1/FKBP1A/CYP1B1/SERPINA6/TSC1/HSPB1/ADIPOQ/SERPINB8/DHCR24/PPARA/INS/CHMP6/RGS14/MEN1/CSN2/TIMP1/PRDX3/SMAD7/HP/GNL3L/BAG5/CFHR5/PYCARD/SERPINE2/GSTO1/RWDD3/TNFRSF4/H19/WFS1/SERPINA1/EOMES/BAX/DAB2/CALM3/LYN/SMO/PNKP/CEBPA/AKT1/IFI6/PPIF/CLN3/PRMT2/PTPRC/ADAM15/IRAK1/NFKB1/PHB2/HAVCR2/NFKBIL1/HEG1/ATP2A3/EIF2AK4/GADD45A/ITGB1BP1/ABL1
GOBP_GLIAL_CELL_DIFFERENTIATION	GOBP_GLIAL_CELL_DIFFERENTIATION	GOBP_GLIAL_CELL_DIFFERENTIATION	50	-0.317895082	-2.064791605	0.00162589	0.0220591 3	0.017662688	1729	tags=84%, list=54%, signal=39%	BOK/LRP1/NR3C1/DUSP15/BMP2/DLX1/PRDM8/ASCL1/TPPP/CDKN2C/MYOC/TNF/ZNF488/LGI4/NF1/NDRG1/TNFRSF21/GCM1/CLCN2/ILK/EMX1/RELA/SERPINE2/EOMES/SIRT2/LYN/CNTNAP1/DAB1/AGER/SMO/PRX/AKT1/EIF2B1/CSK/NFIA/CDK5/MED12/LAMC3/IFNGR1/STAT3/PTN/ABL1
GOBP_SMOOTH_MUSCLE_CELL_PROLIFERATION	GOBP_SMOOTH_MUSCLE_CELL_PROLIFERATION	GOBP_SMOOTH_MUSCLE_CELL_PROLIFERATION	29	-0.408192649	-2.195492044	0.001640318	0.022130544	0.017719869	1249	tags=69%, list=39%, signal=42%	TNF/APOD/NF1/MAP3K5/MDM2/HTR1B/ADIPOQ/CCL5/OGN/ELN/FRS2/AIF1/BMP4/ANG/AKT1/GNA12/PDGFRB/APLN/IRAK1/MEF2D
GOCC_MEMBRANE_PROTEIN_COMPLEX	GOCC_MEMBRANE_PROTEIN_COMPLEX	GOCC_MEMBRANE_PROTEIN_COMPLEX	248	-0.17770974	-1.856889015	0.001636293	0.022130544	0.017719869	1040	tags=42%, list=32%, signal=31%	HLA-C/MAP3K5/KCNS2/DUOX2/FKBP1A/SOCS2/SOCS1/ATP6V0A1/ATP1B1/BCL2/CACNB3/CLINT1/SPCS2/WNT3A/ABHD6/NDUFB7/KCNAB2/CPLX2/CLCN2/CD4/VPS11/HLA-DMA/ITGB8/CD79A/HLA-A/ABHD12/CHMP6/ABCD4/CD34/HLA-F/COPZ2/S

											CN2B/CDH4/HLA-E/HLA-B/ITGAM/GNB1/GNAI1/GJB1/HLA-DOB/KCNH4/BAX/CDH11/CALM3/CLCC1/HLA-DRB5/LYN/HLA-DMB/CLIC5/APH1A/LIME1/COX7B/CDH3/GJA4/KCNB2/IGF2R/GRM1/FLOT2/NDUFA4L2/STT3A/HLA-DQB1/GRIK5/TAP1/PPIF/CHMP7/RAC1/DNAJC11/GNA12/ATP1A3/FCER1G/HLA-DRB3/ITGA3/TIMM17B/CCDC51/CHCHD3/TOMM20/AP2M1/KCNB1/ITGB1/C8A/GNG7/HM13/CNIH3/BET1L/HMGB1/SEC61A1/PHB2/CLIC1/TRAF1/SSR4/ITGA9/ITGB7/CHRNB2/DENND4C/COX7A1/TYK2/SLC25A5/CLTB/DERL1/CCDC115/SHC1/COPZ1/COX8A/SEC22B/VDAC1
GOBP_ANATOMICAL_STRUCTURE_FORMATION_INVOLVED_IN_MORPHOGENESIS	GOBP_ANATOMICAL_STRUCTURE_FORMATION_INVOLVED_IN_MORPHOGENESIS	GOBP_ANATOMICAL_STRUCTURE_FORMATION_INVOLVED_IN_MORPHOGENESIS	260	-0.169848463	-1.802655842	0.001649936	0.022198304	0.017774124	1120	tags=45%, list=35%, signal=32%	WNT11/CEND1/IRX3/ANGPTL4/PODXL/NF1/RARG/ACTN2/INHBA/CLDN5/MYOG/MTHFD1/PLXNB2/PHACTR4/ATP8B1/FKBP1A/CYP1B1/OBSCN/GPR15/HAND2/MYOD1/TGFA/WNT1/SNAI2/TSC1/WNT3A/NRP2/ETS1/CXCL13/HSPB1/CXCR3/NFATC4/GCM1/ADAMTS5/EMP2/OR10J5/STIM1/PPARA/ITGB8/SSBP3/HIPK2/MAFB/COL15A1/CD34/CITED2/MMP15/C3AR1/LOXL2/KLF2/ALOX5/FRS2/XBP1/SLC9A8/VEGFB/TCTA/FAM20C/TGFBI/YAP1/NRP1/COL4A2/BMP4/TMOD1/TEK/GHSR/EDA/EOMES/WDR74/LRG1/CDK5R2/CDH11/ANG/LFNG/ETS2/CNTNAP1/STAB1/MIXL1/SMO/ADIPOR2/FZD4/PLXNA1/TYROBP/ADAM12/FGF1/COL3A1/AHDC1/AKT1/RALA/COL27A1/HS6ST1/PERP/ADRA2B/ITGA3/SLC31A1/GLI2/NFE2L2/SUPT6H/PDGFRB/ALDH1A3/ITGB1/HSBP1/CDK5/MED12/ADAM15/CFL2/TMOD4/PGK1/APLN/MMP14/NKX2-5/PARVA/STAT3/SH3PXD2A/GADD45A/SHC1/ITGB1BP1/ABL1

GOBP_REGULATION_OF_PROTEIN_BINDING	GOBP_REGULATION_OF_PROTEIN_BINDING	GOBP_REGULATION_OF_PROTEIN_BINDING	29	-0.408017984	-2.194552596	0.001670977	0.022340217	0.017887754	1068	tags=69%, list=33%, signal=46%	CLDN5/ARRB2/FKBP1A/PRMT8/WNT3A/ADIPOQ/HIPK2/ADD1/GNL3L/CFHR5/NRP1/BMP4/BAX/DAB2/AKT1/ADAM15/ATP2A3/DERL1/ITGB1BP1/ABL1
GOBP_NEGATIVE_REGULATION_OF_CHROMOSOME_SEGREGATION	GOBP_NEGATIVE_REGULATION_OF_CHROMOSOME_SEGREGATION	GOBP_NEGATIVE_REGULATION_OF_CHROMOSOME_SEGREGATION	13	0.529827668	2.170358521	0.00169272	0.022340217	0.017887754	696	tags=69%, list=22%, signal=54%	CDCA8/TPR/TRIP13/CDK5RAP2/MAD2L1BP/DYNC1LI1/BUB1B/ZW10/MAD2L2
GOBP_MAMMARY_GLAND_DEVELOPMENT	GOBP_MAMMARY_GLAND_DEVELOPMENT	GOBP_MAMMARY_GLAND_DEVELOPMENT	30	-0.395068172	-2.130516425	0.001685181	0.022340217	0.017887754	1027	tags=63%, list=32%, signal=43%	ATP7B/SOCS2/TGFA/ZNF703/WNT3A/CSN2/XBP1/OAS2/CEBPB/BMP4/BAX/SMO/AKT1/IQGAP3/SLC29A1/GLI2/APLN/NFKB1/PHB2
GOBP_POSITIVE_REGULATION_OF_INFLAMMATORY_RESPONSE	GOBP_POSITIVE_REGULATION_OF_INFLAMMATORY_RESPONSE	GOBP_POSITIVE_REGULATION_OF_INFLAMMATORY_RESPONSE	20	-0.453832917	-2.084767165	0.001667801	0.022340217	0.017887754	1507	tags=90%, list=47%, signal=48%	S100A12/LTA/TNF/IL15/MGST2/GPRC5B/NMI/FFAR2/ETS1/HLA-E/PYCARD/CEBPB/GPSM3/CEBPA/CTSC/CASP4/ALOX5AP/MEFV
GOBP_DENDRITIC_CELL_DIFFERENTIATION	GOBP_DENDRITIC_CELL_DIFFERENTIATION	GOBP_DENDRITIC_CELL_DIFFERENTIATION	11	-0.571232637	-2.065289105	0.001679608	0.022340217	0.017887754	818	tags=73%, list=26%, signal=54%	ITGB8/HLA-B/CEBPB/LYN/AGER/TRPM2/LTBR/HMGB1
HP_OSTEOLYSIS	HP_OSTEOLYSIS	HP_OSTEOLYSIS	23	-0.415303526	-1.998459488	0.001687827	0.022340217	0.017887754	1106	tags=74%, list=35%, signal=49%	SQSTM1/NF1/ELMO2/MAFB/MEN1/SLC29A3/ANTXR2/COL3A1/CTSC/PERP/PDGFRB/SCARB2/SH3PXD2B/MMP14/CTSK/ZMPSTE24/FGFR1
GOBP_CIRCULATORY_SYSTEM_DEVELOPMENT	GOBP_CIRCULATORY_SYSTEM_DEVELOPMENT	GOBP_CIRCULATORY_SYSTEM_DEVELOPMENT	244	-0.173200827	-1.793909654	0.001692861	0.022340217	0.017887754	1511	tags=57%, list=47%, signal=33%	ADPRHL1/ITGA5/GJC1/CAV3/CHRNA7/COL8A2/NSDHL/SVEP1/PRKAR1A/ASCL1/CDX4/ELK3/CAD/ZFPM2/MMRN2/CCM2/RNH1/SRI/SYPL2/MAP2K3/CRIP1/PDGFRA/ENG/GATA4/E2F7/SNX17/MEF2B/TNF/TBX6/COL4A1/THY1/SYK/POPDC2/TCF25/SULF1/APOD/WNT11/IRX3/ANGPTL4/NF1/ACTN2/INHBA/CLDN5/ARRB2/MTHFD1/FKBP1A/CYP1B1/GPR15/HAND2/TGFA/SNAI2/TSC1/WNT3A/NRP2/PDLIM2/ETS1/CXCL13/HSPB1/MDM2/CXCR3/NFATC4/ADAMTS5/LIPA/EMP2/OR10J5/STIM1/PPARA/ILK/ITGB8/HIPK2/COL15A1/CD34/CITED2/ELN/C3AR1/LOXL2/SMAD7/KLF2/ALOX5/FRS2/XBP1/VEGFB/FBXW8/TGFBI/YAP1/CUL7/NRP1/COL4

											A2/BMP4/TEK/GHSR/EOMES/PKP2/BAX/LRG1/CDH11/PCSK5/ANG/STAB1/MIXL1/SMO/ADIPOR2/FZD4/GJA4/ADAM12/FGF1/COL3A1/HAS2/AKT1/COL27A1/VGLL4/HS6ST1/ADRA2B/ITGA3/SLC31A1/GLI2/NFE2L2/PDGFRB/ITGB1/MED12/ADAM15/SH3PXD2B/PGK1/APLN/MEF2D/GALNT11/MMP14/NKX2-5/FBN1/HEG1/PARVA/STAT3/ZMPSTE24/GADD45A/MAML1/SHC1/ITGB1BP1/ABL1/MBD2
GOCC_REPLICATION_FORK	GOCC_REPLICATION_FORK	GOCC_REPLICATION_FORK	18	0.467242829	2.169088394	0.001704448	0.022431838	0.017961115	891	tags=72%, list=28%, signal=52%	DONSON/RFC4/PCNA/PRIM1/BLM/POLA2/TIMELESS/WRN/RFC1/TEX264/PARP1/TP53BP1/MCM3
HP_ABNORMALITY_OF_CRANIAL_SUTURES	HP_ABNORMALITY_OF_CRANIAL_SUTURES	HP_ABNORMALITY_OF_CRANIAL_SUTURES	85	-0.25033859	-1.944452765	0.00170954	0.022437708	0.017965814	825	tags=44%, list=26%, signal=33%	COLEC11/INS/B3GAT3/ELN/TSHB/CTCF/FAM20C/B4GALT7/BMP4/H19/ZNF462/CDH11/SMO/AHDC1/ANKRD11/IGF2/AKT1/MAF/PPP3CA/PPP2R1A/NFIA/PDGFRB/MID1/MED12/SH3PXD2B/SETBP1/MMP23B/MMP14/CTSK/FBN1/PURA/STAT3/ZMPSTE24/FGFR1/KRT14/RNF113A/EFNB1
GOBP_POSITIVE_REGULATION_OF_PROTEIN_LOCALIZATION_TO_MEMBRANE	GOBP_POSITIVE_REGULATION_OF_PROTEIN_LOCALIZATION_TO_MEMBRANE	GOBP_POSITIVE_REGULATION_OF_PROTEIN_LOCALIZATION_TO_MEMBRANE	22	-0.453232198	-2.144131159	0.001734468	0.022641844	0.018129265	1291	tags=82%, list=40%, signal=49%	FYN/TNF/STOM/STX3/SQSTM1/AGR2/WNT3A/PRKCH/ITGAM/RHOG/BBC3/AKT1/CLN3/ITGA3/KCNB1/ITGB1/CDK5/ITGB1BP1
GOBP_ANTIGEN_PROCESSING_AND_PRESENTATION_OF_EXOGENOUS_PEPTIDE_ANTIGEN	GOBP_ANTIGEN_PROCESSING_AND_PRESENTATION_OF_EXOGENOUS_PEPTIDE_ANTIGEN	GOBP_ANTIGEN_PROCESSING_AND_PRESENTATION_OF_EXOGENOUS_PEPTIDE_ANTIGEN	15	-0.516911134	-2.1022054	0.001730599	0.022641844	0.018129265	894	tags=73%, list=28%, signal=53%	UNC93B1/HLA-DMA/HLA-A/HLA-F/HLA-E/HLA-DOB/HLA-DRB5/HLA-DMB/HLA-DQB1/FCER1G/HLA-DRB3
GOBP_MRNA_EXPORT_FROM_NUCLEUS	GOBP_MRNA_EXPORT_FROM_NUCLEUS	GOBP_MRNA_EXPORT_FROM_NUCLEUS	16	0.488086268	2.178029124	0.001755802	0.022858558	0.018302788	840	tags=69%, list=26%, signal=51%	NUP88/NCBP2/NUP155/RAE1/TPR/IWS1/NUP93/THOC5/AKAP8L/NUP107/SMG7
GOBP_PROTEIN_MODIFICATION_BY_SMALL_PROTEIN_REMOVAL	GOBP_PROTEIN_MODIFICATION_BY_SMALL_PROTEIN_REMOVAL	GOBP_PROTEIN_MODIFICATION_BY_SMALL_PROTEIN_REMOVAL	23	0.419330107	2.163057138	0.001790806	0.023251598	0.018617494	946	tags=70%, list=30%, signal=49%	ITCH/USP1/SENP1/USP6/USP30/USP37/SENP5/USPL1/ATXN3/USP31/USP8/VCP/USP10/USP48/ZRANB1/ENY2
GOBP_AMEBOIDAL_TYPE_CELL_MI	GOBP_AMEBOIDAL_TYPE_CELL_MIG	GOBP_AMEBOIDAL_TYPE_CELL_MIGRATIO	91	-0.24239384	-1.927891479	0.001863539	0.024131084	0.019321696	1024	tags=47%, list=32%, signal=33%	PHACTR4/CYP1B1/HAND2/SNAI2/NRP2/ETS1/CXCL13/HSPB1/EMP2/ILK

GRATION	RATION	N									/LOXL2/TIMP1/NRP1/BMP4/TEK/IQGAP1/LRG1/MIXL1/SMO/ANXA6/FGF1/RAB11A/HAS2/AKT1/ARHGDIB/CLN3/RAC1/ARHGEF7/GNA12/ITGA3/NFE2L2/CD248/ITGB1/HMGB1/ADIPOR1/CAP1/ITGB7/FGFR1/PTN/GADD45A/EFNB1/ITGB1BP1/ABL1
HP_PRIMARY_MICROCEPHALY	HP_PRIMARY_MICROCEPHALY	HP_PRIMARY_MICROCEPHALY	26	0.392811112	2.147287105	0.001887533	0.024311423	0.019466093	460	tags=42%, list=14%, signal=37%	ASNS/SLC25A19/CENPE/CLPB/KATNB1/CEP63/NDE1/SMC3/TPRKB/CDK5RAP2/FZR1
GOBP_REGULATION_OF_APOPTOTIC_SIGNALING_PATHWAY	GOBP_REGULATION_OF_APOPTOTIC_SIGNALING_PATHWAY	GOBP_REGULATION_OF_APOPTOTIC_SIGNALING_PATHWAY	88	-0.258256504	-2.02487677	0.001885592	0.024311423	0.019466093	1345	tags=61%, list=42%, signal=37%	LTB/IER3/GATA4/APAF1/FYN/TNF/CYLD/PTPN2/TMEM14A/NF1/INHBA/FXN/GCLC/SRPX/BCL2/WNT1/SNAI2/HSPB1/MDM2/NFATC4/BAD/INS/MAZ/XBP1/RELA/BAG5/ITM2C/PYCARD/MYC/YAP1/NRP1/BMP4/WFS1/BAX/TNFSF10/BBC3/CTSC/BCL2L1/ATF4/AKT1/IFI6/PPIF/PTPRC/LTBR/MNT/CTTN/PPP2R1A/NFE2L2/TRAF1/CTSK/SLC25A5/MAGED1/FGFR1/BCL2L2
HP_KYPHOSIS	HP_KYPHOSIS	HP_KYPHOSIS	91	-0.241699318	-1.922367565	0.001923171	0.024704566	0.019780881	637	tags=37%, list=20%, signal=31%	BAZ1B/B4GALT7/CUL7/UBTF/GLB1/GJB1/COL6A3/HTRA1/CDH11/LFNG/TPM2/PRX/PNKP/TRIO/ANKRD11/AKT1/PRPS1/CFL2/SCARB2/SH3PXD2B/MBTPS1/SETBP1/MMP23B/MMP14/TOR1A/CTSK/WDR81/CCDC8/FBN1/ZMPSTE24/PLOD1/RNF113A/CTBP1/ABL1
GOMF_RNA_POLYMERASE_II_GENERAL_TRANSCRIPTION_INITIATION_FACTOR_ACTIVITY	GOMF_RNA_POLYMERASE_II_GENERAL_TRANSCRIPTION_INITIATION_FACTOR_ACTIVITY	GOMF_RNA_POLYMERASE_II_GENERAL_TRANSCRIPTION_INITIATION_FACTOR_ACTIVITY	10	0.613004965	2.205310883	0.001931756	0.024749027	0.019816482	755	tags=80%, list=24%, signal=61%	TBPL1/CCNH/DR1/TAF6/TAF10/GTF2H2/GTF2E1/TAF9
GOBP_MITOTIC_SPINDLE_ORGANIZATION	GOBP_MITOTIC_SPINDLE_ORGANIZATION	GOBP_MITOTIC_SPINDLE_ORGANIZATION	34	0.345343832	2.089136697	0.00196618	0.025057123	0.020063173	632	tags=47%, list=20%, signal=38%	EFHC1/CENPE/WDR62/CDCA8/RAE1/AURKC/CENPH/TPR/SMC3/KIF23/TUBG1/CHEK2/KIF4A/KPNB1/TPX2/TUBG2
GOBP_REGULATION_OF_OSTEOCLAST_DIFFERENTIATION	GOBP_REGULATION_OF_OSTEOCLAST_DIFFERENTIATION	GOBP_REGULATION_OF_OSTEOCLAST_DIFFERENTIATION	15	-0.512561502	-2.084516053	0.001961339	0.025057123	0.020063173	1259	tags=87%, list=39%, signal=53%	LTF/TNF/CCR1/NF1/PIAS3/MAFB/TCTA/CEBPB/TYROBP/IL17A/TFE3/PPP3CA/FBN1

HP_ABNORMAL_RENAL_MEDULLA_MORPHOLOGY	HP_ABNORMAL_RENAL_MEDULLA_MORPHOLOGY	HP_ABNORMAL_RENAL_MEDULLA_MORPHOLOGY	10	0.610588972	2.19661925	0.001973524	0.025084525	0.020085113	1253	tags=100%, list=39%, signal=61%	DHX16/MAPKBP1/TRIP11/KIAA0753/IQCB1/CEP290/ANKS6/PDCD6IP/CLCN7/NPHP3
GOBP_AXONEME_ASSEMBLY	GOBP_AXONEME_ASSEMBLY	GOBP_AXONEME_ASSEMBLY	10	0.60947149	2.192599058	0.001994407	0.025283431	0.020244378	484	tags=70%, list=15%, signal=60%	CLUAP1/ZMYND10/DNAH17/DNAH8/FOXJ1/DNAI1/IQCG
GOBP_MRNA_TRANSPORT	GOBP_MRNA_TRANSPORT	GOBP_MRNA_TRANSPORT	24	0.418443947	2.210159271	0.002013848	0.025463047	0.020388195	1572	tags=92%, list=49%, signal=47%	NUP88/NCBP2/NUP155/AHCTF1/RAE1/TPR/IWS1/PARP11/NUP93/THOC5/AKAP8L/NUP107/RANBP2/SMG7/ENY2/NUP37/PEG10/KHSRP/RBM15B/SMG1/MCM3AP/AAAS
GOMF_PASSIVE_TRANSMEMBRANE_TRANSPORTER_ACTIVITY	GOMF_PASSIVE_TRANSMEMBRANE_TRANSPORTER_ACTIVITY	GOMF_PASSIVE_TRANSMEMBRANE_TRANSPORTER_ACTIVITY	83	-0.254401343	-1.956890671	0.002066111	0.0259878	0.020808363	1824	tags=78%, list=57%, signal=35%	KCNJ9/KCNG1/AQP1/HCN4/HTR3A/RASA3/KCNMB1/KCNH7/TRPV1/KCNMB3/CLCA4/KCNQ3/TOMM40/GABRR1/TRPV5/KCNS3/GJC1/HCN3/CHRNA7/SLC17A8/KCNJ6/GRIA4/SLC17A7/KCNAB3/CLDN4/TMEM63A/CLCN6/KCNE3/MPV17/CHRNA2/CACNA1B/CACNA1E/AQP6/KCNS2/BCL2/CACNB3/KCNAB2/HTR1B/CLCN2/TPCN1/TMEM109/SCN2B/OTOP1/KCNJ1/GJB1/ITPR1/KCNH4/BAX/CLCC1/CLIC5/OTOP2/GJA4/KCNB2/ANXA6/TRPM2/P2RX2/GRIK5/KCNK5/OTOP3/CCDC51/KCNB1/SEC61A1/CLIC1/CHRNB2/VDAC1
HP_ABNORMALITY_OF_IMMUNE_SYSTEM_PHYSIOLOGY	HP_ABNORMALITY_OF_IMMUNE_SYSTEM_PHYSIOLOGY	HP_ABNORMALITY_OF_IMMUNE_SYSTEM_PHYSIOLOGY	354	-0.154756238	-1.729522188	0.002061822	0.0259878	0.020808363	1082	tags=42%, list=34%, signal=31%	NF1/CACNA1B/PIGG/AGR2/COL4A6/ADAMTSL2/MTHFD1/PAX1/HLA-C/BCR/GCLC/ATP7B/ATP8B1/PAX4/SOCS1/ATP6V0A1/LGI3/OBSCN/GALM/BCL2/AMN/C1QC/TSC1/SCGB3A2/FOXD3/IL1R1/ARPC1B/ETS1/LDHA/UNC93B1/ZNF341/FHL1/EMP2/CD4/UBE3B/MPEG1/STIM1/INS/CD79A/HLA-A/MANBA/CITED2/MEN1/CAT/ELN/NFASC/COL4A5/TSHB/ALOX5/RELA/HLA-B/ITGAM/CTCF/CFHR5/CFHR3/BAZ1B/MYC/INPPL1/GNB1/TNFRSF4/SLC25A12/BMP4/TEK/VPS4A/EDA/GLB1/WFS1/SERPINA1/EOMES/COL6A3/SLURP1/ISG15/FECH/LFNG/AIRE/TPM2/SLC29A3/GALC/SMO/ZNFX1/CDH3/TBC1D23/IGF2R/TR

											IO/ANTXR2/COL3A1/ANKRD11/HLA-DQB1/IGF2/CTSC/PLAG1/AKT1/TAP1/SLC11A1/MGP/OSGEP/PRPS1/CLN3/TBC1D2B/RAC1/PTPRC/APRT/TFE3/MAF/CDC42BPB/ITGA3/GLI2/NFE2L2/MID1/TRMU/GALT/STUB1/GOT2/HPS1/MED12/C8A/SCARB2/SH3PXD2B/MSN/ABCD1/SETBP1/IRAK1/SEC61A1/NFKB1/ZBTB7A/ARHGDIA/HAVCR2/MEFV/IFNGR1/UBE2L3/NFKBIL1/NKX2-5/TYK2/TRIM8/FBN1/PURA/HPS6/STAT3/FGFR1/EIF2AK4/KRT14/PLOD1/SGSH/RNF113A/CTBP1/COX8A/HMGCL
HP_SIMPLIFIED_GYRAL_PATTERN	HP_SIMPLIFIED_GYRAL_PATTERN	HP_SIMPLIFIED_GYRAL_PATTERN	19	0.468144112	2.279529846	0.002072006	0.025994252	0.02081353	410	tags=47%, list=13%, signal=42%	ASNS/GMNN/DONSON/CENPE/WDR62/KATNB1/TRAIP/NDE1/CDK5RAP2
GOMF_POTASSIUM_ION_TRANSMEMBRANE_TRANSPORTER_ACTIVITY	GOMF_POTASSIUM_ION_TRANSMEMBRANE_TRANSPORTER_ACTIVITY	GOMF_POTASSIUM_ION_TRANSMEMBRANE_TRANSPORTER_ACTIVITY	35	-0.352934581	-2.033713887	0.002108133	0.026378975	0.021121576	1824	tags=89%, list=57%, signal=39%	KCNJ9/KCNG1/AQP1/HCN4/KCNMB1/KCNH7/KCNMB3/SLC12A3/KCNQ3/KCNS3/HCN3/KCNJ6/SLC17A7/KCNAB3/ATP1A2/KCNE3/KCNS2/SLC12A8/KCNAB2/TMCO3/SCN2B/SLC9A8/KCNJ1/KCNH4/SLC12A9/KCNB2/GRIK5/KCNK5/ATP1A3/CCDC51/KCNB1
HP_JOINT_CONTRACTURE_OF_THE_HAND	HP_JOINT_CONTRACTURE_OF_THE_HAND	HP_JOINT_CONTRACTURE_OF_THE_HAND	58	-0.287433757	-1.948486435	0.002115304	0.026400304	0.021138655	1067	tags=55%, list=33%, signal=37%	PTRH2/ADAMTSL2/BCR/GPKOW/NDRG1/MYOD1/HSPB1/KCNAB2/FHL1/DHCR24/MAFB/CTCF/COL6A3/LFNG/TPM2/SLC29A3/CDH3/ANTXR2/PQBP1/OSGEP/TBC1D2B/PDGFRB/MED12/FBXW11/SH3PXD2B/MMP23B/FBN1/ZMPSTE24/FGFR1/KRT14/EFNB1/PDXK
GOBP_CELLULAR_RESPONSE_TO_BIOTIC_STIMULUS	GOBP_CELLULAR_RESPONSE_TO_BIOTIC_STIMULUS	GOBP_CELLULAR_RESPONSE_TO_BIOTIC_STIMULUS	41	-0.341872093	-2.074615457	0.002142997	0.026608415	0.021305289	1322	tags=71%, list=41%, signal=42%	MAP2K3/APAF1/LTF/TNF/SYK/CTSG/BCR/CXCL13/CCR5/CD6/KMO/XBP1/RELA/PYCARD/GHSR/WFS1/LYN/TRIM41/LY86/AKT1/CDK4/IRAK1/HMGB1/NFKB1/HAVCR2/NFKBIL1/ZMPSTE24/TSPO/ABL1
HP_SKIN_NODULE	HP_SKIN_NODULE	HP_SKIN_NODULE	24	-0.420708413	-2.06573369	0.002139364	0.026608415	0.021305289	1241	tags=75%, list=39%, signal=46%	CYLD/CCR1/BTNL2/BCL2/TSC1/MDM2/HLA-B/TEK/ANTXR2/COL3A1/AKT1/PDGFRB/CDK4/MMP14/MEFV/I

GOBP_GLAND_MORPHOGENESIS	GOBP_GLAND_MORPHOGENESIS	GOBP_GLAND_MORPHOGENESIS	31	-0.37905539	-2.065597495	0.002181689	0.027019377	0.021634344	1373	tags=77%, list=43%, signal=45%	FNGR1/FBN1/FGFR1 LIMS2/CRIP1/TNF/SULF1/RARG/BCL2/TGFA/SNAI2/WNT3A/LIPA/FRS2/XBP1/CEBPB/NRP1/BMP4/EDA/BAX/PLXNA1/FGF1/PLAG1/MSN/NFKB1/PHB2/PTN
HP_DENTAL_CROWDING	HP_DENTAL_CROWDING	HP_DENTAL_CROWDING	27	-0.392787989	-2.030743699	0.002192896	0.027019613	0.021634533	576	tags=48%, list=18%, signal=40%	H19/TPM2/TRIO/COL3A1/IGF2/MED12/SETBP1/ZBTB7A/FBN1/ZMPSTE24/FGFR1/PLOD1/ABL1
GOBP_REGULATION_OF_OSTEOBLAST_DIFFERENTIATION	GOBP_REGULATION_OF_OSTEOBLAST_DIFFERENTIATION	GOBP_REGULATION_OF_OSTEOBLAST_DIFFERENTIATION	27	-0.392031666	-2.026833456	0.002192896	0.027019613	0.021634533	1259	tags=70%, list=39%, signal=43%	LTF/TNF/JUND/HAND2/SNAI2/ILK/MEN1/TMEM119/FAM20C/CEBPB/LRP3/YAP1/BMP4/IFITM1/CEBPA/CHRD/ATF4/PPP3CA/CLIC1
GOCC_U2_TYPE_SPLICEOSOMAL_COMPLEX	GOCC_U2_TYPE_SPLICEOSOMAL_COMPLEX	GOCC_U2_TYPE_SPLICEOSOMAL_COMPLEX	16	0.480351319	2.143512799	0.00226108	0.027776624	0.022240671	959	tags=75%, list=30%, signal=53%	BUD13/DHX16/PRPF18/PRPF40B/SF3A1/TFIP11/PRPF38A/LSM3/PRPF40A/PLRG1/PRPF6/LUC7L
GOCC_NUCLEAR_PERIPHERY	GOCC_NUCLEAR_PERIPHERY	GOCC_NUCLEAR_PERIPHERY	37	0.3330523	2.056891891	0.002274854	0.027776624	0.022240671	824	tags=54%, list=26%, signal=41%	SRPK1/PSMA6/NARF/AHCTF1/PCNA/EBNA1BP2/TPR/PAXIP1/SMC3/BLM/RUVBL1/BRD7/ATXN3/KIF4A/NUP93/PRPF40A/AKAP8L/NUP107/SMARCC1/HAT1
HP_ICHTHYOSIS	HP_ICHTHYOSIS	HP_ICHTHYOSIS	20	-0.445553815	-2.046735546	0.002283089	0.027776624	0.022240671	1023	tags=70%, list=32%, signal=48%	EMD/PSAT1/ELOVL1/FHL1/STIM1/COL4A5/PEX11B/SLURP1/SLC29A3/PROKR2/HS6ST1/SUMF1/FGFR1/RNF113A
GOBP_POSITIVE_REGULATION_OF_PEPTIDYL_TYROSINE_PHOSPHORYLATION	GOBP_POSITIVE_REGULATION_OF_PEPTIDYL_TYROSINE_PHOSPHORYLATION	GOBP_POSITIVE_REGULATION_OF_PEPTIDYL_TYROSINE_PHOSPHORYLATION	28	-0.390214099	-2.044411808	0.002275861	0.027776624	0.022240671	1504	tags=79%, list=47%, signal=42%	ITGA5/PTPN1/DLG4/FYN/TNF/IL15/SYK/GPRC5B/EHD4/CSHL1/TGFA/ADIPOQ/CCL5/CD4/NRP1/HCLS1/LYN/NTF3/OSBP/IGF2/PTPRC/ABL1
GOBP_CALCIUM_ION_TRANSMEMBRANE_TRANSPORT	GOBP_CALCIUM_ION_TRANSMEMBRANE_TRANSPORT	GOBP_CALCIUM_ION_TRANSMEMBRANE_TRANSPORT	62	-0.286979606	-2.00101398	0.00228037	0.027776624	0.022240671	1331	tags=61%, list=42%, signal=37%	SRI/FYN/DIAPH1/ATP1A2/THY1/ATP2A1/CACNA1B/CACNA1E/ATP2B3/FKBP1A/ATP1B1/BCL2/CACNB3/WNT3A/IBTK/CCR5/STIM1/TPCN1/CCL21/GSTO1/ITPR1/BAX/CALM3/LYN/LIME1/SLC25A23/ANXA6/XCR1/TRPM2/P2RX2/CABP5/PTPRC/PPP3CA/F2RL3/SEC61A1/ATP2A3/ABL1/VDAC1
GOBP_STRIATED_MUSCLE_CELL_DIFFERENTIATION	GOBP_STRIATED_MUSCLE_CELL_DIFFERENTIATION	GOBP_STRIATED_MUSCLE_CELL_DIFFERENTIATION	59	-0.282099152	-1.93332666	0.002334244	0.028327632	0.022681861	1105	tags=54%, list=35%, signal=36%	IRX3/ACTN2/ARRB2/MYOG/OBSCN/MYOD1/BCL2/WNT1/TSC1/WNT3A/ADAMTS5/PPARA/FRS2/XBP1/TME

											M119/BMP4/TMOD1/CNTNAP1/SMO/ADAM12/P2RX2/IGF2/AKT1/PPP3CA/PDGFRB/ITGB1/CFL2/TMOD4/MMP14/NKX2-5/ZMPSTE24/MAML1
GOBP_DNA_TEMPLATED_TRANSCRIPTION_ELONGATION	GOBP_DNA_TEMPLATED_TRANSCRIPTION_ELONGATION	GOBP_DNA_TEMPLATED_TRANSCRIPTION_ELONGATION	20	0.442618078	2.150772384	0.002361855	0.028519404	0.022835413	823	tags=65%, list=26%, signal=49%	NCBP2/INTS7/ELL3/ZMYND11/IWS1/PAF1/INTS6/ADRM1/THOC5/ERCC3/MED4/PARP1/SUPT5H
GOMF_MONOATOMIC_CATION_TRANSMEMBRANE_TRANSPORTER_ACTIVITY	GOMF_MONOATOMIC_CATION_TRANSMEMBRANE_TRANSPORTER_ACTIVITY	GOMF_MONOATOMIC_CATION_TRANSMEMBRANE_TRANSPORTER_ACTIVITY	114	-0.222469468	-1.871453813	0.002359687	0.028519404	0.022835413	1950	tags=81%, list=61%, signal=33%	SLC34A1/SLC4A5/SLC6A8/SLC12A1/CACNA1C/GRIN1/SLC13A2/KCNJ9/KCNG1/AQP1/HCN4/HTR3A/SLC6A1/RASA3/SLC5A7/KCNMB1/SLC5A11/SLC25A22/KCNH7/TRPV1/KCNMB3/SLC12A3/KCNQ3/TRPV5/KCNS3/SLC41A3/HCN3/CHRNA7/SLC17A8/SLC34A3/NIPA2/KCNJ6/SLC25A37/SLC17A7/KCNAB3/ATP1A2/TMEM63A/ATP6V1G2/KCNE3/ATP2A1/CHRNA2/CACNA1B/CACNA1E/ATP2B3/SLC10A5/ATP7B/KCNS2/ATP6V0A1/SLC12A8/CACNB3/KCNAB2/HTR1B/TPCN1/TMCO3/CNNM2/TMEM109/SCN2B/ATP13A1/OTOP1/SLC9A8/KCNJ1/SLC25A12/SLC32A1/ITPR1/KCNH4/SLC12A9/OTOP2/COX7B/KCNB2/ANXA6/SLC16A1/SLC2A13/COX15/TRPM2/P2RX2/GRIK5/KCNK5/SLC11A1/SLC34A2/ATP1A3/OTOP3/SLC29A1/SLC31A1/CCDC51/SLC30A9/KCNB1/SEC61A1/CHRNB2/COX7A1/SLC25A5/ATP2A3/COX8A
GOBP_REGULATION_OF_RESPONSE_TO_STRESS	GOBP_REGULATION_OF_RESPONSE_TO_STRESS	GOBP_REGULATION_OF_RESPONSE_TO_STRESS	269	-0.164100139	-1.748673864	0.002381765	0.028688092	0.022970048	938	tags=38%, list=29%, signal=30%	SNAI2/FGL2/IL1R1/ETS1/PLAU/UNC93B1/MDM2/ADIPOQ/CCL5/BAD/PPARA/COLEC11/INS/HLA-A/ABHD12/HIPK2/PRDX1/SPN/MEN1/HLA-F/KIR2DL4/CORO2B/MINK1/ALOX5/HLA-E/OTOP1/XBP1/RELA/CCL21/HLA-B/PYCARD/SERPINE2/CEBPB/MYC/PLSCR1/GPSM3/PUM1/RHBDF2/BMP4/H19/TEK/GHSR/WFS1/TFPI/BAX/ISG15/CLDN3/SIRT2/FECH/TFPT/LYN/TRIM41/AGER/ZNFX1/SLC25A23/

											PNKP/FLOT2/TYROBP/LAG3/CEBPA/RNF185/PQBP1/CTSC/BCL2L1/AKT1/SCGB1A1/CLN3/PTPRC/BFAR/LTBR/IL17A/PPP3CA/CASP4/NFE2L2/MID1/NCOA7/ITGB1/EIF2AK2/UBQLN2/ALOX5AP/ABCD1/ST3GAL4/IRAK1/HMGB1/NFKB1/PHB2/MAPKAPK3/HAVCR2/MEFV/KREMEN1/NFKBIL1/STAT3/ZMPSTE24/EIF2AK4/PTN/GADD45A/PTGES/ING4/EIF4G1/ABL1/LSM14A/EIF2AK1/VDAC1
GOBP_NEGATIVE_REGULATION_OF_NUCLEAR_DIVISION	GOBP_NEGATIVE_REGULATION_OF_NUCLEAR_DIVISION	GOBP_NEGATIVE_REGULATION_OF_NUCLEAR_DIVISION	15	0.497880689	2.20016484	0.002391729	0.028736444	0.023009196	696	tags=67%, list=22%, signal=52%	CDCA8/TPR/TRIP13/AURKAIP1/CDK5RAP2/MAD2L1BP/DYNC1LI1/BUB1B/ZW10/MAD2L2
GOBP_POSITIVE_REGULATION_OF_CELL_JUNCTION_ASSEMBLY	GOBP_POSITIVE_REGULATION_OF_CELL_JUNCTION_ASSEMBLY	GOBP_POSITIVE_REGULATION_OF_CELL_JUNCTION_ASSEMBLY	22	-0.443050124	-2.095962247	0.002434604	0.029179	0.023363549	1411	tags=82%, list=44%, signal=46%	SLITRK2/MYOC/CLSTN1/THY1/AMIGO3/IRX3/CLDN5/CLSTN3/TSC1/LRRN3/NRP1/TEK/CLDN3/AMIGO2/RAC1/IL17A/ITGB1BP1/ABL1
GOBP_RNA_EXPORT_FROM_NUCLEUS	GOBP_RNA_EXPORT_FROM_NUCLEUS	GOBP_RNA_EXPORT_FROM_NUCLEUS	19	0.460268542	2.241181405	0.002474325	0.029443698	0.023575492	946	tags=74%, list=30%, signal=52%	NUP88/NCBP2/NUP155/RAE1/TPR/IWS1/NUP93/THOC5/NOL6/AKAP8L/NUP107/SMG7/XPOT/ENY2
GOBP_POSITIVE_REGULATION_OF_CHEMOTAXIS	GOBP_POSITIVE_REGULATION_OF_CHEMOTAXIS	GOBP_POSITIVE_REGULATION_OF_CHEMOTAXIS	26	-0.40563429	-2.058470372	0.002474977	0.029443698	0.023575492	1317	tags=77%, list=41%, signal=46%	OXSR1/PDGFRA/CCR1/STX3/CXCL13/HSPB1/CCL5/C3AR1/CCL21/VEGFB/GPSM3/NRP1/AIF1/AGER/NTF3/RAC1/PDGFRB/HMGB1/FGFR1/PTN
GOBP_MAPK_CASCADE	GOBP_MAPK_CASCADE	GOBP_MAPK_CASCADE	135	-0.205486309	-1.840609721	0.002465737	0.029443698	0.023575492	1352	tags=56%, list=42%, signal=34%	CCL8/CCM2/PJA2/MAP2K3/MYOC/PDGFRA/GATA4/AVPI1/TNF/CYLD/PTPN2/SYK/CCL22/CCR1/MOS/IGFBP6/ZDHHC9/FSHR/NF1/INHBA/ARRB2/MAP3K5/CDC42EP5/HAND2/MAPK4/TGFA/DUSP9/ADIPOQ/CCL5/CD4/CCR5/INS/HIPK2/RGS14/PRDX1/MEN1/BTN2A2/MINK1/FRS2/CCL21/IGFBP4/PYCARD/MYC/NRP1/BMP4/TEK/IQGAP1/DOK4/DAB2/LYN/AGER/GRM1/NTF3/FGF1/SLA/IGF2/CSK/PTPRC/LTBR/ADRA2B/IQGAP3/PPP2R1A/PDGFRB/MID1/EIF2AK2/IRAK1/HMGB1/NFKB1/PHB2/MAPKAPK3/HAVCR2/FGFR1/GADD45A/SHC1/ITGB1BP1/ABL1

GOBP_MITOTIC_NUCLEAR_DIVISION	GOBP_MITOTIC_NUCLEAR_DIVISION	GOBP_MITOTIC_NUCLEAR_DIVISION	75	0.24417678	2.002136969	0.002486902	0.029512863	0.023630872	926	tags=52%, list=29%, signal=38%	SPAG5/CENPE/CDC14B/CDCA8/RANBP1/KATNB1/CDC25C/NDE1/AURKC/TPR/SMC3/TRIP13/AURKAIP1/PINX1/KIF23/CDK5RAP2/MAD2L1BP/DYNC1LI1/TUBG1/BUB1B/UBE2S/CHEK2/KIF4A/ESPL1/KPNB1/TPX2/TUBG2/ZW10/MAD2L2/KIF22/AKAP8L/NUSAP1/CDC16/SPAST/BCCIP/UBE2C/PDCD6IP/MAPRE1/REEP3
GOBP_NON_MEMBRANE_BOUNDED_ORGANELLE_ASSEMBLY	GOBP_NON_MEMBRANE_BOUNDED_ORGANELLE_ASSEMBLY	GOBP_NON_MEMBRANE_BOUNDED_ORGANELLE_ASSEMBLY	92	0.226124509	1.98973842	0.002508476	0.029695926	0.023777451	591	tags=34%, list=18%, signal=28%	SPAG5/CCNB2/SUGT1/CSRP2/CENPE/EIF5/WDR62/CDCA8/DHX30/CEP63/AURKC/CENPH/PRKAA1/EDC3/TPR/SMC3/CEP72/EIF2S1/KIF23/CDK5RAP2/NIP7/KIAA0753/MYOM2/CNOT7/CHEK2/CAPN3/RBM14/KIF4A/BOP1/KPNB1/ACTL8
GOMF_PROTEIN_FOLDING_CHAPERONE	GOMF_PROTEIN_FOLDING_CHAPERONE	GOMF_PROTEIN_FOLDING_CHAPERONE	13	0.518149584	2.122521023	0.002514652	0.02969626	0.023777718	333	tags=54%, list=10%, signal=48%	HSPA1L/HSPA14/ZMYND10/DNAJB8/HSPA2/HSPH1/DNAJB6
GOBP_EPITHELIUM_DEVELOPMENT	GOBP_EPITHELIUM_DEVELOPMENT	GOBP_EPITHELIUM_DEVELOPMENT	257	-0.167106658	-1.761252297	0.002562539	0.03018796	0.024171421	1512	tags=74%, list=47%, signal=43%	VASP/F11R/NOTCH4/NPHP3/SLC4A5/SEC24B/DVL2/C3/LMO4/KRT32/ERRFI1/GPR161/TAGLN/BRSK2/GAL/SDC4/LTA4H/HS3ST3B1/CA2/AQP1/LSR/ID2/LHX1/HEYL/CDH23/FOXH1/ABI1/NR3C1/KRT3/CTSH/CDKN1C/WNT10A/ADM/ECE1/BRSK1/ADAM17/UMOD/FOXC1/ROCK2/TMIGD1/MTSS1/SIX3/BMP2/DEAF1/APCDD1/KRT27/PHLDB2/ITGA5/FOXQ1/CAV3/LZTS2/NSDHL/KLHL3/TBC1D20/LBH/NCOA3/ASCL1/CNFN/EXTL3/WNT2B/MMRN2/ANXA4/SFN/CCM2/SH3BP1/ENG/GATA4/E2F7/SOX21/APAF1/LCE1A/CLDN4/CLASP2/TNF/TBX6/LRP6/COL4A1/ANXA7/KRT38/TRIM16/SULF1/UGCG/MAFG/SAFB2/FOXE3/WNT11/IRX3/PODXL/NF1/RARG/INHBA/CLDN5/AGR2/ADAMTSL2/MTHFD1/PLXNB2/BCR/TRIOBP/PHACTR4/HAND2/BCL2/WNT1/SNAI2/ZNF703/TSC1/WNT3A/NFATC4/ELOVL1/ADIPOQ/GCM1/CLCN2/LIP

											A/BAD/TTC8/PRKCH/SPRR3/ILK/SSBP3/MAFB/CD34/CITED2/ADD1/CAT/EMX1/FOSL2/SMAD7/KLF2/FRS2/RELA/FAM20C/SERPINE2/CEBPB/MYC/YAP1/MYADM/PTCH2/MSI1/NRP1/BMP4/TMOD1/EDA/IQGAP1/BAX/LRG1/CLDN3/DAB2/RAPGEF1/LFNG/AIRE/KRT85/PITX3/SMO/FZD4/RAB1A/CDH3/GJA4/PLXNA1/FGF1/CEBPA/CHRD/PLEKHA4/ATF4/AKT1/RALA/AKR1C1/RAC1/COL27A1/IL17A/MAF/IQGAP3/PPP3CA/GLI2/ALDH1A3/CBR1/ITGB1/MED12/DNASE1L2/MSN/PGK1/PHB2/MMP14/RHOC/TOR1A/NKX2-5/HEG1/ZMPSTE24/MAGED1/KRT14/ITPK1/ABL1/VDAC1
GOBP_REGULATION_OF_CELL_SUBSTRATE_ADHESION	GOBP_REGULATION_OF_CELL_SUBSTRATE_ADHESION	GOBP_REGULATION_OF_CELL_SUBSTRATE_ADHESION	53	-0.293930518	-1.95257991	0.002648038	0.031119283	0.024917128	1251	tags=60%, list=39%, signal=37%	CLASP2/THY1/APOD/NF1/AGR2/TRIOBP/BCL2/ABI3BP/WNT1/TSC1/PLAU/EMP2/ILK/FAM107A/CORO2B/MINK1/CCL21/MYADM/NRP1/TEK/PKP2/DAB2/FZD4/RSU1/HAS2/RAC1/ARHGEF7/ITGA3/ADAM15/MMP14/ITGB1BP1/ABL1
GOBP_SYNCYTIUM_FORMATION	GOBP_SYNCYTIUM_FORMATION	GOBP_SYNCYTIUM_FORMATION	12	-0.564340761	-2.110792982	0.002704971	0.031711191	0.025391068	1048	tags=83%, list=33%, signal=56%	MYOG/MYOD1/WNT1/GCM1/ADAMTS5/TCTA/TYROBP/ADAM12/ITGB1/SH3PXD2A
HP_FEMALE_HYPOGONADISM	HP_FEMALE_HYPOGONADISM	HP_FEMALE_HYPOGONADISM	11	-0.556292639	-2.011273609	0.002723222	0.031841595	0.025495482	763	tags=73%, list=24%, signal=56%	MEN1/NHLH2/BMP4/AIRE/PROKR2/HS6ST1/ZMPSTE24/FGFR1
GOBP_POSITIVE_REGULATION_OF_LEUKOCYTE_MIGRATION	GOBP_POSITIVE_REGULATION_OF_LEUKOCYTE_MIGRATION	GOBP_POSITIVE_REGULATION_OF_LEUKOCYTE_MIGRATION	27	-0.385512304	-1.993127859	0.00272928	0.031841595	0.025495482	1317	tags=74%, list=41%, signal=44%	OXSR1/TNF/THY1/CCR1/IL1R1/CXCL13/CCL5/SPN/C3AR1/CCL21/VEGFB/PYCARD/GPSM3/AIF1/AGER/RAC1/HMGB1/MMP14/PTN/ABL1
GOBP_SIGNAL_TRANSDUCTION_IN_RESPONSE_TO_DNA_DAMAGE	GOBP_SIGNAL_TRANSDUCTION_IN_RESPONSE_TO_DNA_DAMAGE	GOBP_SIGNAL_TRANSDUCTION_IN_RESPONSE_TO_DNA_DAMAGE	46	0.29355155	2.029025113	0.002739102	0.031879191	0.025525585	711	tags=46%, list=22%, signal=36%	DONSON/MDM4/INTS7/MDC1/RAD51/CDC14B/DOT1L/CEP63/CRY1/PAXIP1/BLM/RAD17/MAPK3/FZR1/CHEK2/BARD1/THOC5/E2F1/ATR/MAD2L2/RINT1
GOBP_NEGATIVE_REGULATION_OF_MONOATOMIC_ION_TRANSPORT	GOBP_NEGATIVE_REGULATION_OF_MONOATOMIC_ION_TRANSPORT	GOBP_NEGATIVE_REGULATION_OF_MONOATOMIC_ION_TRANSPORT	22	-0.439194627	-2.077722832	0.002752849	0.031937919	0.025572609	1331	tags=82%, list=42%, signal=48%	SRI/OXSR1/ATP1A2/KCNE3/SLC26A5/ACTN2/FKBP1A/BCL2/HTR1B/SERPINE2/GSTO1/HRH3/CALM3/MAOB/ATF4/PPIF/PPP3CA/VDAC1
GOBP_BLOOD_VE	GOBP_BLOOD_VES	GOBP_BLOOD_VESSE	26	-0.401845025	-2.039241005	0.00276239	0.0319379	0.0255726	1357	tags=73%, list=42%,	MMRN2/SH3BP1/MAP2K3/TNF/NF1/

SSEL_ENDOTHELIAL_CELL_MIGRATION	SEL_ENDOTHELIAL_CELL_MIGRATION	L_ENDOTHELIAL_CELL_MIGRATION					19	09		signal=42%	CYP1B1/ETS1/HSPB1/EMP2/NRP1/AKT1/CLN3/NFE2L2/ITGB1/HMGB1/FGFR1/GADD45A/ITGB1BP1/ABL1
GOBP_ORGANIC_HYDROXY_COMPOUND_METABOLIC_PROCESS	GOBP_ORGANIC_HYDROXY_COMPOUND_METABOLIC_PROCESS	GOBP_ORGANIC_HYDROXY_COMPOUND_METABOLIC_PROCESS	99	-0.235102382	-1.903891397	0.002763986	0.031937919	0.025572609	1469	tags=63%, list=46%, signal=35%	NSDHL/GPD1/TPI1/FMO5/ALG2/PNPLA2/PNPO/STARD4/SNX17/CYP7A1/TNF/CLN8/PLTP/CYP11A1/PNPLA4/RDH5/DUOX2/ATP8B1/CYP1B1/DHRS4/HAND2/BCL2/NQO1/SNAI2/PSAT1/DAO/LDHA/IDH3B/SEC14L2/CLCN2/LIPA/DHCR24/HSD17B1/CAT/SNCB/ITGAM/SORD/ALDH2/PMVK/DAB2/FECH/CDH3/FGF1/CEBPA/MAOB/OSBP/AKR7A2/SCP2/AKR1C1/NFE2L1/AKR1A1/ALDH1A3/ACAA1/MBTPS1/IDH3G/NFKB1/CTSK/OSBPL5/CHRNB2/ITPK1/OSBPL1A/PDXK
GOBP_POSTREPLICATION_REPAIR	GOBP_POSTREPLICATION_REPAIR	GOBP_POSTREPLICATION_REPAIR	10	0.598557785	2.15333655	0.002791281	0.032099732	0.025702172	892	tags=90%, list=28%, signal=65%	USP1/PCNA/UBE2N/MAD2L2/RCHY1/WDR33/VCP/USP10/POLI
GOBP_GLIOGENESIS	GOBP_GLIOGENESIS	GOBP_GLIOGENESIS	68	-0.274200996	-1.975551449	0.002785061	0.032099732	0.025702172	1729	tags=79%, list=54%, signal=37%	BOK/LRP1/NR3C1/DUSP15/ETV5/CX3CR1/BMP2/ATP1B2/DLX1/APCDD1/PRDM8/LTA/ASCL1/TPPP/CDKN2C/MYOC/TNF/ZNF488/LGI4/NF1/NDRG1/TNFRSF21/GCM1/CLCN2/PRKCH/ILK/EMX1/RELA/SERPINE2/EOMES/CDK5R2/SIRT2/LYN/CNTNAP1/DAB1/AGER/PITX3/SMO/PRX/COL3A1/PLAG1/AKT1/EIF2B1/CSK/NFIA/CDK5/MED12/LAMC3/MMP14/IFNGR1/STAT3/PTN/TSPO/ABL1
GOBP_FAT_CELL_DIFFERENTIATION	GOBP_FAT_CELL_DIFFERENTIATION	GOBP_FAT_CELL_DIFFERENTIATION	52	-0.296869614	-1.959194608	0.002805376	0.032185197	0.025770603	1041	tags=54%, list=33%, signal=37%	FFAR2/SOCS1/WNT1/FBXO9/SNAI2/CCDC85B/WNT3A/ADIPOQ/CCDC3/TTC8/INS/MAFB/FOSL2/ALOX5/XBP1/CEBPB/LRP3/YAP1/LRG1/SIRT2/CEBPA/TRIO/JDP2/AKT1/TFE3/EBF2/ZBTB7A/CTBP1
GOBP_NCRNA_PROCESSING	GOBP_NCRNA_PROCESSING	GOBP_NCRNA_PROCESSING	71	0.245748444	1.995113766	0.002847762	0.032594054	0.026097974	578	tags=37%, list=18%, signal=31%	TDRD7/TSN/PWP1/NCBP2/INTS7/NUP155/GEMIN4/FKBP6/ZNHIT3/EBNA1BP2/PNPT1/EXOSC8/PIWIL2/TRPT1/GTPBP4/TDRKH/TPRKB/UTP18/SMAD2/INTS6/METTL6/DDX52/DUS1L/PA2G4/DUS3L/BOP1
GOBP_POSITIVE_R	GOBP_POSITIVE_R	GOBP_POSITIVE_REG	64	-0.281340951	-1.989580826	0.00286871	0.0326790	0.0261659	1357	tags=64%, list=42%,	MMRN2/PJA2/FYN/LTF/TNF/CYLD/

EGULATION_OF_RESPONSE_TO_BIOTIC_STIMULUS	EGULATION_OF_RESPONSE_TO_BIOTIC_STIMULUS	ULATION_OF_RESPONSE_TO_BIOTIC_STIMULUS				6	01	91		signal=38%	SYK/NMI/ARRB2/FFAR2/UNC93B1/CCL5/COLEC11/HLA-F/KIR2DL4/HLA-E/RELA/PYCARD/PLSCR1/PUM1/SIRT2/LYN/TRIM41/ZNFX1/FLOT2/TYROBP/LAG3/LY86/RNF185/PQBP1/IL17A/HLA-DRB3/EIF2AK2/IRAK1/HMGB1/PHB2/MAPKAPK3/HAVCR2/MEFV/NFKBIL1/LSM14A
GOBP_REGULATION_OF_HYDROLASE_ACTIVITY	GOBP_REGULATION_OF_HYDROLASE_ACTIVITY	GOBP_REGULATION_OF_HYDROLASE_ACTIVITY	159	-0.198976812	-1.852382901	0.002863997	0.032679001	0.026165991	1352	tags=54%, list=42%, signal=33%	CCL8/SFN/SH3BP1/PDGFRA/APAF1/FYN/CLDN4/LTF/TNF/THY1/SYK/CCL22/PROM2/PPP1R15A/TBC1D17/WNT11/RPS27L/ANGPTL4/NF1/SERPINB10/NEIL1/PLXNB2/BCR/NR1H2/MAP3K5/FKBP1A/SERPINA6/TSC1/CXCL13/CCL5/SERPINB8/BAD/DHCR24/ARHGAP27/PRSS22/TBC1D16/FNTA/CSN2/TIMP1/NET1/CCL21/OAS2/RHOG/PYCARD/SERPINE2/MYC/GRTP1/SERPINA1/BAX/RGS11/CLDN3/CALM3/RAPGEF1/ANG/LYN/APH1A/AGER/TNFSF10/PLXNA1/BBC3/RGS10/NTF3/RSU1/RALGDS/AKT1/IFI6/TBC1D2B/ARHGEF7/PTPRC/GNA12/RGS6/PERP/PDGFRB/ARF4/ITGB1/SCARB2/HMGB1/PHB2/MEFV/RHOC/CTSK/STAT3/ATP2A3/FGFR1/ITGB1BP1/ABL1
GOBP_POST_TRANSLATIONAL_PROTEIN_MODIFICATION	GOBP_POST_TRANSLATIONAL_PROTEIN_MODIFICATION	GOBP_POST_TRANSLATIONAL_PROTEIN_MODIFICATION	231	0.158228059	1.743873047	0.002910743	0.033079741	0.026486862	836	tags=38%, list=26%, signal=30%	MDM4/ITCH/FBXO7/SAE1/TRIM17/USP1/SENP1/CDC14B/UBE2D3/FBXO24/KLHL10/RNF141/DOT1L/DZIP3/TRAIP/CEP63/UBE2T/USP6/RNF138/CRY1/AURKC/ASB1/PAXIP1/USP30/ANAPC10/RUVBL1/MAGEC2/SUPT3H/GTPBP4/UBE2N/PCGF6/UBE2D2/RNF182/MAGEA2B/PINX1/ZFP91/KBTBD2/USP37/SENP5/USPL1/ATXN3/NFYB/FZR1/FBXL2/UBE3A/TRIM28/CAMLG/KCTD10/TAF10/USP31/PINK1/SHARPIN/UBE2S/CAPN3/PARP11/BARD1/SMC6/BIRC8/TRAF2/MIER2/PARP12/MKRN2/KBTBD8/RNF10/MAD2L2/ZC3HC1/HERC5/RCHY1/G

											SK3B/DDA1/USP8/ATG12/DCUN1D3/RMND5A/TAF9/KLHDC2/KLHL8/SETD1A/RNF7/DCUN1D5/RANBP2/VCP/PARP1/RNF139/CDC16/ANKRD9/USP10/USP48
GOBP_IMMUNE_EFFECTOR_PROCESS	GOBP_IMMUNE_EFFECTOR_PROCESS	GOBP_IMMUNE_EFFECTOR_PROCESS	119	-0.210943229	-1.784387835	0.002942704	0.033364456	0.026714833	1589	tags=66%, list=50%, signal=34%	CFP/CD300A/CX3CR1/NDST2/IL21R/C9/CD1A/WAS/TAP2/LTA/SVEP1/CR2/IL9R/MICA/JAGN1/POU2AF1/FYN/MRGPRX2/PRAM1/TNF/SYK/GPRC5B/CTSG/NMI/CD1B/ARRB2/FFAR2/HLA-C/BCR/C1QC/FGL2/TSC1/IL1R1/UNC93B1/CUEDC2/CPLX2/LIPA/EMP2/COLEC11/INS/HLA-A/PRDX1/SPN/MEN1/HLA-F/KIR2DL4/SMAD7/HLA-E/XBP1/HLA-B/ITGAM/CFHR5/CFHR3/PYCARD/TNFRSF4/EOMES/LYN/HLA-DMB/LFNG/AIRE/AGER/TYROBP/LAG3/CD177/CTSC/SLC11A1/PTPRC/IL17A/FCER1G/HLA-DRB3/SUPT6H/C8A/HMGB1/PHB2/HAVCR2/STAT3/EIF2AK4/ABL1
GOBP_RNA_SPLICING_VIA_TRANSESTERIFICATION_REACTIONS	GOBP_RNA_SPLICING_VIA_TRANSESTERIFICATION_REACTIONS	GOBP_RNA_SPLICING_VIA_TRANSESTERIFICATION_REACTIONS	50	0.280127955	1.995690917	0.002965469	0.033543835	0.026858461	406	tags=30%, list=13%, signal=27%	SRPK1/DDX20/BUD13/KHDRBS3/DAZAP1/NCBP2/DHX16/SETX/GEMIN4/PRPF18/GEMIN6/PRPF40B/SF3A1/TFIP11/PRPF38A
GOBP_RENAL_SYSTEM_DEVELOPMENT	GOBP_RENAL_SYSTEM_DEVELOPMENT	GOBP_RENAL_SYSTEM_DEVELOPMENT	74	-0.260993247	-1.940986729	0.002995821	0.033807978	0.02706996	1858	tags=80%, list=58%, signal=34%	SDC4/HS3ST3B1/AQP1/LHX1/HMGCS2/HEYL/CTSH/CDKN1C/UMOD/FOXC1/MTSS1/BMP2/EGR1/RRM2B/LZTS2/KLHL3/WNT2B/PDGFRA/APAF1/SNX17/COL4A1/SULF1/MPV17/WNT11/IRX3/PODXL/NF1/BCL2/WNT1/TSC1/ADIPOQ/TTC8/ILK/CD34/CAT/SMAD7/MMP17/MYC/YAP1/NRP1/BMP4/TEK/WFS1/IQGAP1/BAX/PCSK5/APH1A/SMO/FGF1/HAS2/PPP3CA/ITGA3/GLI2/NFIA/PDGFRB/SEC61A1/FBN1/ZMPSTE24/MAGED1
GOBP_NEGATIVE_REGULATION_OF_TOR_SIGNALING	GOBP_NEGATIVE_REGULATION_OF_TOR_SIGNALING	GOBP_NEGATIVE_REGULATION_OF_TOR_SIGNALING	17	0.455715583	2.104672348	0.003009681	0.033885224	0.027131811	1011	tags=76%, list=32%, signal=53%	TSSK2/PRKAA1/UBE2N/MAPK3/ATXN3/UBE3A/TBK1/ENDOG/YWHAZ/KPTN/TBC1D7/SIRT1/NUAK1
GOBP_NEGATIVE_REGULATION_OF_	GOBP_NEGATIVE_REGULATION_OF_	GOBP_NEGATIVE_REGULATION_OF_DEVELO	174	-0.194280444	-1.833155513	0.003030077	0.034035513	0.027252146	1259	tags=51%, list=39%, signal=32%	LTF/TNF/CBFB/TBX6/PTPN2/THY1/CCR1/SULF1/FOXE3/WNT11/IRX3/N

DEVELOPMENTAL_PROCESS	DEVELOPMENTAL_PROCESS	PMENTAL_PROCESS									F1/RARG/INHBA/CLDN5/FXN/BCR/NR1H2/PIAS3/SOCS2/SOCS1/HAND2/BCL2/GDI1/WNT1/C1QC/SNAI2/CCDC85B/FGL2/WNT3A/NFATC4/ADIPOQ/PPARA/MAFB/CITED2/MEN1/LOXL2/BTG2/SMAD7/KLF2/ALOX5/FRS2/XBP1/TMEM119/TCTA/MYC/LRP3/YAP1/NRP1/COL4A2/BMP4/TEK/PKP2/SIRT2/LYN/STAB1/DAB1/MIXL1/PITX3/SMO/CDH3/LAG3/CEBPA/TRIO/CHRD/IGF2/BCL2L1/JDP2/UBE2B/VGLL4/PPP3CA/NFE2L2/ITGB1/CDK5/PGK1/HMGB1/ADIPOR1/KREMEN1/CTSK/NKX2-5/FBN1/STAT3/EIF2AK4/PTN/GADD45A/TSPO/ISL2/WWC3
GOCC_RIBONUCLEOPROTEIN_COMPLEX	GOCC_RIBONUCLEOPROTEIN_COMPLEX	GOCC_RIBONUCLEOPROTEIN_COMPLEX	126	0.193727087	1.841274	0.003063315	0.034329028	0.027487163	755	tags=36%, list=24%, signal=28%	BUD13/PRKRIP1/DAZAP1/PSMA6/DHX16/SECISBP2/GEMIN4/CPSF3/RPL39L/ZNHIT3/MRPS17/EBNA1BP2/RPL26L1/PRPF18/PNPT1/PRPF40B/SF3A1/MRPL39/RUVBL1/SUZ12/AURKAIP1/EIF2S1/TFIP11/UTP18/PRPF38A/SRP19/MYH10/NIP7/CPEB1/MRPS16/ELAVL4/PDCD7/MRPS10/PA2G4/RBM14/BOP1/MRPS11/NOL6/LSM3/PRPF40A/DNTTIP2/AKAP8L/MRPS14/MRPS31/TAF9
GOBP_HETEROTYPIC_CELL_CELL_ADHESION	GOBP_HETEROTYPIC_CELL_CELL_ADHESION	GOBP_HETEROTYPIC_CELL_CELL_ADHESION	14	-0.507609257	-1.998393129	0.003071095	0.034336549	0.027493185	616	tags=86%, list=19%, signal=70%	TNF/THY1/ADIPOQ/MYADM/PKP2/AGER/FLOT2/PTPRC/PERP/ITGB1/ITGB7/PARVA
GOBP_MICROTUBULE_BUNDLE_FORMATION	GOBP_MICROTUBULE_BUNDLE_FORMATION	GOBP_MICROTUBULE_BUNDLE_FORMATION	15	0.489856266	2.164704431	0.003098833	0.034420793	0.02756064	484	tags=53%, list=15%, signal=45%	CLUAP1/ZMYND10/DNAH17/DNAH8/FOXJ1/CDK5RAP2/DNAI1/IQCG
GOCC_NUCLEAR_PORE	GOCC_NUCLEAR_PORE	GOCC_NUCLEAR_PORE	26	0.376771048	2.0596047	0.003100009	0.034420793	0.02756064	958	tags=65%, list=30%, signal=46%	NUP88/NUP155/AHCTF1/RANBP1/SNUPN/RAE1/TPR/RGPD5/PARP11/KPNB1/XPO4/NUP93/NUP107/RANBP2/XPOT/ENY2/NUP37
GOBP_REGULATION_OF_CALCIUM_ION_TRANSPORT	GOBP_REGULATION_OF_CALCIUM_ION_TRANSPORT	GOBP_REGULATION_OF_CALCIUM_ION_TRANSPORT	43	-0.328478007	-2.012055698	0.003091601	0.034420793	0.02756064	1331	tags=67%, list=42%, signal=40%	SRI/UCN/FYN/DIAPH1/ATP1A2/THY1/CCR1/ATP2A1/FKBP1A/ATP1B1/BCL2/CACNB3/CCL5/CD4/STIM1/GSTO1/WFS1/BAX/CALM3/LYN/LIME1/P2RX2/CABP5/PPP3CA/PDGFRB/F2

											RL3/TSPO/ABL1/VDAC1
GOBP_CELL_CYCLE_DNA_REPLICATION	GOBP_CELL_CYCLE_DNA_REPLICATION	GOBP_CELL_CYCLE_DNA_REPLICATION	11	0.532005568	2.017040068	0.00314138	0.034750847	0.027824913	523	tags=64%, list=16%, signal=53%	GMNN/DONSON/RAD51/PCNA/BLM/WRN/CDC7
HP_ABSENCE_OF_SECONDARY_SEX_CHARACTERISTICS	HP_ABSENCE_OF_SECONDARY_SEX_CHARACTERISTICS	HP_ABSENCE_OF_SECONDARY_SEX_CHARACTERISTICS	11	-0.550297712	-1.989598977	0.003147191	0.034750847	0.027824913	1447	tags=100%, list=45%, signal=55%	DHX37/ZFPM2/GATA4/CYP11A1/KISS1R/NHLH2/PROKR2/HS6ST1/GLI2/FGFR1
HP_ABNORMAL_FINGER_MORPHOLOGY	HP_ABNORMAL_FINGER_MORPHOLOGY	HP_ABNORMAL_FINGER_MORPHOLOGY	233	-0.176499925	-1.807578434	0.003151319	0.034750847	0.027824913	614	tags=30%, list=19%, signal=26%	INPPL1/B4GALT7/CUL7/GNB1/GNAI1/PTCH2/SLC25A12/PUM1/SLC32A1/BMP4/H19/EDA/WFS1/COL6A3/ZNF462/HEATR3/CDH11/LFNG/TPM2/SLC29A3/SMO/SLC35B2/PNKP/TRIO/ANTXR2/COL3A1/PROKR2/ANKRD11/PQBP1/IGF2/CTSC/PLAG1/AKT1/MGP/OSGEP/SLC34A2/TBC1D2B/RAC1/COL27A1/TFE3/MAF/CDC42BPB/PPP2R1A/GLI2/PRR12/PDGFRB/STUB1/MED12/FBXW11/SH3PXD2B/ABCD1/SETBP1/MMP23B/TOR1A/CTSK/NFKBIL1/NKX2-5/SUMF1/TRIM8/CCDC8/FBN1/ZMPSTE24/DPH5/FGFR1/EIF2AK4/KRT14/PLOD1/CTBP1/EFNB1/ABL1
GOCC_SITE_OF_DNA_DAMAGE	GOCC_SITE_OF_DNA_DAMAGE	GOCC_SITE_OF_DNA_DAMAGE	26	0.375483542	2.052566595	0.003186721	0.035057503	0.028070452	915	tags=62%, list=29%, signal=44%	SMCHD1/RAD51/TRAIP/RNF138/TIMELESS/CHD1L/RAD50/SMC6/ARPC2/CBX1/NHEJ1/MAD2L2/VCP/PARP1/TP53BP1/RIF1
GOBP_DNA_TEMPLATED_TRANSCRIPTION_INITIATION	GOBP_DNA_TEMPLATED_TRANSCRIPTION_INITIATION	GOBP_DNA_TEMPLATED_TRANSCRIPTION_INITIATION	29	0.36399677	2.043373362	0.003193644	0.035057503	0.028070452	755	tags=55%, list=24%, signal=43%	TBPL1/CCNH/MDC1/SETX/DR1/PAXIP1/POLRMT/ATF7IP/TAF6/TAF10/GTF2H2/ERCC3/CDK7/MED4/GTF2E1/TAF9
GOBP_MONOAMINE_TRANSPORT	GOBP_MONOAMINE_TRANSPORT	GOBP_MONOAMINE_TRANSPORT	15	-0.494268185	-2.010119686	0.003213935	0.035200238	0.028184739	572	tags=60%, list=18%, signal=50%	GHSR/HRH3/SYT12/SLC29A3/MAOB/ADRA2B/KCNB1/TOR1A/CHRNB2
GOBP_REGULATION_OF_CELL_JUNCTION_ASSEMBLY	GOBP_REGULATION_OF_CELL_JUNCTION_ASSEMBLY	GOBP_REGULATION_OF_CELL_JUNCTION_ASSEMBLY	48	-0.320826045	-2.04235725	0.003227292	0.035246591	0.028221854	1319	tags=65%, list=41%, signal=39%	MYOC/CLSTN1/CLASP2/TNF/THY1/APOD/AMIGO3/IRX3/CLDN5/SNAI2/CLSTN3/TSC1/WNT3A/PRKCH/LRRN3/FAM107A/ABI3/NRP1/TEK/GHSR/CLDN3/RAPGEF1/AMIGO2/RAC1/IL17A/LRFN3/MMP14/CHRNB2/ITGB1BP1/EIF4G1/ABL1
HP_ABNORMALIT	HP_ABNORMALIT	HP_ABNORMALITY_O	24	-0.413620194	-2.0309296	0.00323276	0.0352465	0.0282218	613	tags=50%, list=19%,	B4GALT7/EDA/COL3A1/CTSC/PAX9

Y_OF_PRIMARY_TEETH	Y_OF_PRIMARY_TEETH	F_PRIMARY_TEETH				2	91	54		signal=41%	/ZBTB7A/CTSK/PURA/STAT3/ZMPSTE24/FGFR1/RNF113A
GOMF_ODORANT_BINDING	GOMF_ODORANT_BINDING	GOMF_ODORANT_BINDING	14	-0.505809911	-1.991309334	0.003272476	0.035599237	0.028504216	1472	tags=93%, list=46%, signal=50%	OR5D18/OR5AS1/OR11L1/OR6B2/OR8A1/OR8D1/OR10T2/OR8G1/OR6K2/OR8B8/OR9K2/OR8H2/OR5F1
GOBP_TUMOR_NECROSIS_FACTOR_SUPERFAMILY_CYTOKINE_PRODUCTION	GOBP_TUMOR_NECROSIS_FACTOR_SUPERFAMILY_CYTOKINE_PRODUCTION	GOBP_TUMOR_NECROSIS_FACTOR_SUPERFAMILY_CYTOKINE_PRODUCTION	33	-0.358541105	-2.023024761	0.003341312	0.036250102	0.029025362	928	tags=58%, list=29%, signal=41%	ORM2/HSPB1/NFATC4/ADIPOQ/SPN/HLA-E/OAS2/PYCARD/GHSR/AGER/TYROBP/PTPRC/IL17A/HMGB1/HAVCR2/IFNGR1/NFKBIL1/STAT3/TSPO
HP_ABNORMALITY_OF_UPPER_LIMB_JOINT	HP_ABNORMALITY_OF_UPPER_LIMB_JOINT	HP_ABNORMALITY_OF_UPPER_LIMB_JOINT	123	-0.207788655	-1.776574169	0.003347318	0.036250102	0.029025362	1082	tags=49%, list=34%, signal=34%	NF1/PTRH2/ADAMTSL2/BCR/MRAS/EMD/GPKOW/NDRG1/MYOD1/HSPB1/KCNAB2/FHL1/DHCR24/COLEC11/MAFB/B3GAT3/ELN/NANS/CTCF/BAZ1B/B4GALT7/CUL7/GNB1/GLB1/COL6A3/HEATR3/LFNG/GNE/TPM2/SLC29A3/SLC35B2/CDH3/ANTXR2/PQBP1/AKT1/OSGEP/SLC34A2/TBC1D2B/COL27A1/TFE3/MAF/PDGFRB/MED12/FBXW11/SH3PXD2B/MBTPS1/SETBP1/MMP23B/MMP14/TOR1A/CCDC8/FBN1/ZMPSTE24/FGFR1/KRT14/PLOD1/LARS2/CTBP1/EFNB1/PDXK
GOBP_CARDIAC_CHAMBER_MORPHOGENESIS	GOBP_CARDIAC_CHAMBER_MORPHOGENESIS	GOBP_CARDIAC_CHAMBER_MORPHOGENESIS	27	-0.379690894	-1.963030726	0.003392805	0.036660513	0.029353977	1775	tags=89%, list=55%, signal=40%	HEYL/FOXH1/FOXC1/BMP2/ADPRHL1/CAV3/ZFPM2/ENG/GATA4/WNT11/FKBP1A/HAND2/NRP2/MDM2/CITED2/SMAD7/NRP1/BMP4/TEK/PKP2/SMO/NKX2-5/HEG1/PARVA
GOBP_NEPHRON_DEVELOPMENT	GOBP_NEPHRON_DEVELOPMENT	GOBP_NEPHRON_DEVELOPMENT	40	-0.338769277	-2.020964351	0.003444424	0.0371352	0.029734058	1831	tags=88%, list=57%, signal=38%	HS3ST3B1/AQP1/LHX1/HEYL/UMOD/FOXC1/MTSS1/BMP2/EGR1/LZTS2/KLHL3/WNT2B/PDGFRA/SULF1/MPV17/WNT11/IRX3/PODXL/BCL2/WNT1/ADIPOQ/ILK/CD34/MYC/YAP1/BMP4/TEK/IQGAP1/SMO/FGF1/PPP3CA/ITGA3/PDGFRB/SEC61A1/MAGED1
GOBP_REGULATION_OF_HORMONE_LEVELS	GOBP_REGULATION_OF_HORMONE_LEVELS	GOBP_REGULATION_OF_HORMONE_LEVELS	100	-0.228343223	-1.856758187	0.0034683	0.037309327	0.029873481	1370	tags=59%, list=43%, signal=35%	PNPLA2/PCSK1N/JAGN1/SRI/UCN/PDGFRA/TNF/GHRH/CTSG/GPR119/CYP11A1/BCHE/PNPLA4/INHBA/RDH5/TM7SF3/FFAR2/DUOX2/CYP1B1/DHRS4/RPH3AL/CYP2S1/ADIPOQ/CC

											L5/CLCN2/BAD/INS/RBP1/HSD17B1/PSMD9/AANAT/ALOX5/UCN3/GHSR/ITPR1/CPA3/DAB2/PCSK5/LYN/FZD4/RAB1A/SLC16A1/HSD17B8/RAB11FIP3/OSBP/UGT2A3/AKR1C1/PPP3CA/CYP2W1/STUB1/ALDH1A3/BACE2/KCNB1/NFKB1/CTSK/ZMPSTE24/BCAT2/TSPO/NADK
GOBP_REGULATION_OF_MITOTIC_CELL_CYCLE_PHASE_TRANSITION	GOBP_REGULATION_OF_MITOTIC_CELL_CYCLE_PHASE_TRANSITION	GOBP_REGULATION_OF_MITOTIC_CELL_CYCLE_PHASE_TRANSITION	82	0.226478953	1.910529049	0.00348674	0.037424344	0.029965575	711	tags=39%, list=22%, signal=31%	DONSON/FBXO7/CCNH/CPSF3/CENPE/CDC14B/CDCA8/CDC25C/HSPA2/TPR/BLM/TRIP13/RAD17/BRD7/PINX1/CDK5RAP2/MAD2L1BP/FZR1/DYNC1LI1/BUB1B/CDC7/TFDP1/RAD50/CHEK2/BARD1/ESPL1/E2F1/ERCC3/ZW10/CDK7/MAD2L2/RINT1
GOMF_LIPID_BINDING	GOMF_LIPID_BINDING	GOMF_LIPID_BINDING	159	-0.195892866	-1.823672776	0.003502526	0.037510427	0.030034501	1306	tags=54%, list=41%, signal=34%	STARD4/MITD1/AMPH/SBF2/SNX17/PLA2G4D/PRAM1/LTF/ATP1A2/CLN8/THY1/PROM2/ANXA7/PLTP/GPR119/SYP/APOD/GPR12/SGIP1/STX3/PHF12/CD1B/NF1/ACTN2/FFAR2/ATP8B1/PHLDA1/OBSCN/SERPINA6/CLINT1/SNX24/PLEK2/SEC14L2/TRIP10/CD4/BAD/CD6/PPARA/SIDT1/TPCN1/RBP1/HSD17B1/ACOX1/PHLDA3/KCNJ1/GRB7/PLA2G4C/WIPI1/CPNE3/ITPR1/IQGAP1/BAX/SYT12/CDK5R2/LYN/IGF2R/ANXA6/OSBP/GRAMD1A/PLEKHA4/AKT1/SCP2/CLN3/FCHSD1/NFE2L1/F10/ATP1A3/CASP4/PRAP1/CYP2W1/AP2M1/SCARB2/SH3PXD2B/ALOX5AP/ZFYVE1/HMGB1/PHB2/OSBPL5/STARD7/NME4/SH3PXD2A/LTC4S/TSPO/SHC1/OSBPL1A/VDAC1
GOBP_ORGANOPHOSPHATE_ESTER_TRANSPORT	GOBP_ORGANOPHOSPHATE_ESTER_TRANSPORT	GOBP_ORGANOPHOSPHATE_ESTER_TRANSPORT	23	-0.396648469	-1.908690502	0.003516809	0.037580065	0.03009026	621	tags=52%, list=19%, signal=42%	PLSCR1/GJB1/SLC35B2/SLC25A23/OSBP/SCP2/VAPA/PRAP1/SCARB2/ABCD1/OSBPL5/SLC25A5
HP_GRAY_MATTER_HETEROTOPIA	HP_GRAY_MATTER_HETEROTOPIA	HP_GRAY_MATTER_HETEROTOPIA	21	0.410082352	2.044067294	0.003537058	0.037713001	0.030196702	478	tags=48%, list=15%, signal=41%	DHX16/CENPE/PAFAH1B1/WDR62/KATNB1/CEP63/ARFGEF2/POMT1/CDK5RAP2/TUBG1
GOBP_REGULATION_OF_DNA_TEMPL	GOBP_REGULATION_OF_DNA_TEMPL	GOBP_REGULATION_OF_DNA_TEMPLATED	10	-0.604891593	-2.100336907	0.003585138	0.038141446	0.030539756	777	tags=80%, list=24%, signal=61%	CITED2/RGS14/KLF2/RELA/CEBPB/SIRT2/ATF4/NFE2L2

ATED_TRANSCRIPTION_IN_RESPONSE_TO_STRESS	ATED_TRANSCRIPTION_IN_RESPONSE_TO_STRESS	_TRANSCRIPTION_IN_RESPONSE_TO_STRESS									
GOBP_CHROMOSOME_SEPARATION	GOBP_CHROMOSOME_SEPARATION	GOBP_CHROMOSOME_SEPARATION	21	0.408305676	2.035211401	0.003621777	0.03834227	0.030700556	696	tags=57%, list=22%, signal=45%	CSNK2A2/CDCA8/TPR/TRIP13/CDK5RAP2/MAD2L1BP/DYNC1LI1/BUB1B/TOP3A/ESPL1/ZW10/MAD2L2
GOBP_ENDOTHELIUM_DEVELOPMENT	GOBP_ENDOTHELIUM_DEVELOPMENT	GOBP_ENDOTHELIUM_DEVELOPMENT	26	-0.392750966	-1.993091431	0.003626519	0.03834227	0.030700556	860	tags=58%, list=27%, signal=43%	LIPA/CD34/ADD1/MYADM/NRP1/BMP4/CLDN3/RAPGEF1/FZD4/RAB1A/GJA4/FGF1/COL27A1/MSN/HEG1
GOBP_REGULATION_OF_PROTEIN_LOCALIZATION_TO_PLASMA_MEMBRANE	GOBP_REGULATION_OF_PROTEIN_LOCALIZATION_TO_PLASMA_MEMBRANE	GOBP_REGULATION_OF_PROTEIN_LOCALIZATION_TO_PLASMA_MEMBRANE	28	-0.379048313	-1.985911958	0.00362783	0.03834227	0.030700556	1124	tags=68%, list=35%, signal=44%	STX3/SQSTM1/AGR2/WNT3A/PRKCH/INS/ABI3/RHOG/VPS4A/DAB2/BCL2L1/AKT1/CLN3/CSK/ITGA3/AP2M1/ITGB1/CDK5/MMP14
GOBP_DETOXIFICATION	GOBP_DETOXIFICATION	GOBP_DETOXIFICATION	33	-0.356385571	-2.010862425	0.003653576	0.038446126	0.030783713	1258	tags=67%, list=39%, signal=41%	MT1H/MGST2/MT1E/GPX5/MT1B/ATP7B/DUOX2/NQO1/PRDX1/CAT/PRDX3/HP/GSTO1/H19/SLC11A1/NFE2L2/AKR1A1/MUC2/ALOX5AP/CCS/LTC4S/PTGES
GOBP_REGULATION_OF_PROTEIN_LOCALIZATION_TO_CELL_PERIPHERY	GOBP_REGULATION_OF_PROTEIN_LOCALIZATION_TO_CELL_PERIPHERY	GOBP_REGULATION_OF_PROTEIN_LOCALIZATION_TO_CELL_PERIPHERY	33	-0.355971669	-2.008527033	0.003653576	0.038446126	0.030783713	1124	tags=67%, list=35%, signal=44%	STX3/SQSTM1/AGR2/WNT3A/PRKCH/INS/ABI3/RHOG/GNAI1/GHSR/VPS4A/DAB2/BCL2L1/AKT1/CLN3/CSK/ITGA3/AP2M1/ITGB1/CDK5/MMP14/EIF4G1
GOBP_MYELOID_LEUKOCYTE_ACTIVATION	GOBP_MYELOID_LEUKOCYTE_ACTIVATION	GOBP_MYELOID_LEUKOCYTE_ACTIVATION	49	-0.312603459	-2.010743122	0.003672153	0.038543676	0.030861821	1618	tags=78%, list=51%, signal=39%	TRPV1/TSPAN32/CD300A/CNR2/CX3CR1/MFHAS1/S100A12/PJA2/MRGPRX2/PRAM1/TNF/IL15/SYK/CTSG/NMI/BCR/NDRG1/CPLX2/CCL5/ITGB8/ITGAM/PYCARD/PLSCR1/AIF1/LYN/AGER/TYROBP/CEBPA/CD177/CTSC/SLC11A1/PTPRC/LTBR/FCER1G/HMGB1/HAVCR2/IFNGR1/CRTC3
GOBP_MUSCLE_CELL_PROLIFERATION	GOBP_MUSCLE_CELL_PROLIFERATION	GOBP_MUSCLE_CELL_PROLIFERATION	43	-0.322307276	-1.974257567	0.003678806	0.038543676	0.030861821	1153	tags=58%, list=36%, signal=38%	APOD/NF1/MYOG/MAP3K5/MDM2/HTR1B/ADIPOQ/CCL5/OGN/CITED2/ELN/FRS2/YAP1/AIF1/BMP4/ANG/AKT1/GNA12/VGLL4/PDGFRB/APLN/IRAK1/MEF2D/NKX2-5/ABL1
GOBP_REGULATION_OF_SISTER_CHROMATID_SEGREGATION	GOBP_REGULATION_OF_SISTER_CHROMATID_SEGREGATION	GOBP_REGULATION_OF_SISTER_CHROMATID_SEGREGATION	23	0.401199645	2.069533622	0.003689846	0.038551913	0.030868417	903	tags=65%, list=28%, signal=47%	CENPE/CDCA8/TPR/TRIP13/BRD7/CDK5RAP2/MAD2L1BP/DYNC1LI1/BUB1B/ESPL1/ZW10/MAD2L2/SMARCC1/CDC16/UBE2C
GOMF_SULFUR_C	GOMF_SULFUR_C	GOMF_SULFUR_COMP	50	-0.301461208	-1.958050334	0.00370353	0.0385519	0.0308684	1272	tags=64%, list=40%,	TNXB/MCCC1/LTF/FTSJ1/CTSG/FX

OMPOUND_BINDING	OMPOUND_BINDING	OUND_BINDING				8	13	17		signal=39%	N/PRMT8/IMPG2/NRP2/CXCL13/ADAMTS5/CD34/VEGFB/SERPINE2/GAL3ST4/NRP1/BMP4/NAV2/PCOLCE/ANG/ANXA6/FGF1/SCP2/PTPRC/MUC2/PRELP/SUMF1/ILVBL/FBN1/FGFR1/PTN/DHTKD1
GOBP_MONONUCLEAR_CELL_DIFFERENTIATION	GOBP_MONONUCLEAR_CELL_DIFFERENTIATION	GOBP_MONONUCLEAR_CELL_DIFFERENTIATION	87	-0.242671873	-1.888164887	0.003697324	0.038551913	0.030868417	1007	tags=47%, list=31%, signal=33%	SOCS1/BCL2/WNT1/FGL2/TSC1/LIPA/CD4/BAD/ITGB8/CD79A/MAFB/SPN/MEN1/FOSL2/BTN2A2/SMAD7/XBP1/HLA-B/CEBPB/MYC/BMP4/EOMES/BAX/LYN/LFNG/AIRE/AGER/LAG3/TRPM2/PTPRC/LTBR/FCER1G/GLI2/ITGB1/HMGB1/ZBTB7A/MMP14/CTSK/SLC25A5/STAT3/ABL1
GOBP_NEGATIVE_REGULATION_OF_TORC1_SIGNALING	GOBP_NEGATIVE_REGULATION_OF_TORC1_SIGNALING	GOBP_NEGATIVE_REGULATION_OF_TORC1_SIGNALING	11	0.522923513	1.982606466	0.003773864	0.039150335	0.031347571	918	tags=82%, list=29%, signal=59%	PRKAA1/UBE2N/MAPK3/ATXN3/UBE3A/TBK1/YWHAZ/KPTN/TBC1D7
HP_JUVENILE_ONSET	HP_JUVENILE_ONSET	HP_JUVENILE_ONSET	152	-0.192668849	-1.76817415	0.003777237	0.039150335	0.031347571	1309	tags=53%, list=41%, signal=33%	MECR/SBF2/DIAPH1/MCCC1/CBFB/ATP1A2/HPS3/CLN8/MPV17/KISS1R/FSHR/REEP2/CHRNA2/ACTN2/ATP2B3/FXN/ATP7B/EMD/ATP8B1/SOCS1/ATP6V0A1/OBSCN/C1QC/IMPG2/FOXD3/LDHA/CYP2U1/ABCB7/FHL1/CLCN2/LIPA/CD4/STIM1/ABHD12/MAFB/CNNM2/NHLH2/PRDX3/TMEM126B/BAG5/TGFBI/TNFRSF4/RHBDF2/GLB1/WFS1/ITPR1/COL6A3/HTRA1/PMVK/TPM2/DAB1/ZNFX1/PNKP/SLC16A1/P2RX2/STT3A/SCP2/CLN3/EIF2B1/PAX9/SLC34A2/APRT/ATP1A3/MAF/PDGFRB/SLC30A9/EIF2AK2/UBQLN2/SCARB2/PGK1/LAMC3/SEC61A1/NFKB1/HAVCR2/MEFV/FBN1/STAT3/EIF2AK4/DHTKD1/PDXK
GOBP_POSITIVE_REGULATION_OF_CELL_DEVELOPMENT	GOBP_POSITIVE_REGULATION_OF_CELL_DEVELOPMENT	GOBP_POSITIVE_REGULATION_OF_CELL_DEVELOPMENT	92	-0.22864192	-1.814405762	0.003800835	0.039310565	0.031475867	1249	tags=55%, list=39%, signal=35%	TNF/CBFB/IL15/SYK/CCR1/ZNF488/CLDN5/MYOG/PLXNB2/SOCS1/TRAK1/MYOD1/BCL2/GDI1/WNT3A/ADIPOQ/CLCN2/CD4/BAD/PRKCH/RGS14/ADD1/BTN2A2/CDH4/SMAD7/XBP1/RELA/FBXW8/SERPINE2/CUL7/N

											RP1/HCLS1/BMP4/SIRT2/LYN/AGER/SMO/FZD4/PLXNA1/TYROBP/PLAG1/PTPRC/IL17A/PPP3CA/GLI2/ITGB1/NAP1L1/HMGB1/MMP14/PTN/TSPO
GOBP_CELLULAR_RESPONSE_TO_ORGANIC_CYCLIC_COMPOUND	GOBP_CELLULAR_RESPONSE_TO_ORGANIC_CYCLIC_COMPOUND	GOBP_CELLULAR_RESPONSE_TO_ORGANIC_CYCLIC_COMPOUND	103	-0.219911548	-1.798398315	0.003811577	0.039337432	0.031497379	1151	tags=50%, list=36%, signal=33%	SAFB2/SSTR3/VPS18/INHBA/ARRB2/MYOG/GCLC/CYP1B1/ELK1/HAND2/MYOD1/SNAI2/ZNF703/MDM2/HTR1B/ADIPOQ/VPS11/PPARA/RXRB/FAM107A/AANAT/KLF2/FOLR2/YAP1/GNB1/GNAI1/TFPI/HRH3/SIRT2/DAB2/FECH/RAPGEF1/SMO/DDX54/SLC16A1/CEBPA/TRPM2/GRAMD1A/PQBP1/PRMT2/ATP1A3/MSN/CDK4/NFKB1/PHB2/ZBTB7A/UBE2L3/KLF9/BCL2L2/ABL1/MBD2
GOBP_MULTICELLULAR_ORGANISMAL_LEVEL_HOMEOSTASIS	GOBP_MULTICELLULAR_ORGANISMAL_LEVEL_HOMEOSTASIS	GOBP_MULTICELLULAR_ORGANISMAL_LEVEL_HOMEOSTASIS	159	-0.193624756	-1.802557716	0.003867999	0.039834613	0.03189547	1259	tags=52%, list=39%, signal=33%	LTF/TNF/EHMT1/CLN8/IL15/PTPN2/SYK/CROCC/SGIP1/PRR4/SQSTM1/IRX3/NF1/INHBA/CLDN5/MTHFD1/AQP6/BCR/NR1H2/BCL2/OCIAD1/ETS1/HSPB1/ABHD6/ADIPOQ/ADAMTS5/LIPA/FMO1/HIPK2/MAFB/CD34/CITED2/PRDX1/EMX1/FOSL2/ARMCX1/KLF2/TMEM119/CYBRD1/CEBPB/YAP1/HCLS1/BMP4/WFS1/BAX/ISG15/HEATR3/NOVA1/CLDN3/FECH/LYN/SMO/ADIPOR2/CDH3/TRPM2/COL3A1/HAS2/PRKAB2/ATF4/AKT1/SLC11A1/CSK/IL17A/TFE3/PPP2R1A/GPRASP2/MUC2/ITGB1/EBF2/HMGB1/ZBTB7A/ADIPOR1/TFF3/CTSK/OCIAD2/CRTC3/SLC25A5/STAT3/PTGES/EIF4G1/ABL1/EIF2AK1
GOBP_RHO_PROTEIN_SIGNAL_TRANSDUCTION	GOBP_RHO_PROTEIN_SIGNAL_TRANSDUCTION	GOBP_RHO_PROTEIN_SIGNAL_TRANSDUCTION	34	-0.352891225	-2.013347632	0.003896816	0.040046001	0.032064729	1038	tags=59%, list=32%, signal=40%	BCR/PHACTR4/CDC42EP5/RHOBTB2/NET1/RHOG/NRP1/COL3A1/ARHGDIB/RAC1/GNA12/ITGA3/ITGB1/F2RL3/ARHGEF3/ARHGDIA/RHOU/TNFAIP1/HEG1/ABL1
GOCC_UBIQUITIN_LIGASE_COMPLEX	GOCC_UBIQUITIN_LIGASE_COMPLEX	GOCC_UBIQUITIN_LIGASE_COMPLEX	86	0.231821651	2.005365902	0.00392586	0.040173528	0.032166839	903	tags=49%, list=28%, signal=36%	FBXO15/SUGT1/FBXO7/CACYBP/CKS2/DERL2/RAD51/UBE2D3/FBXO24/FBXL18/ASB1/ANAPC10/UBE2N/PCGF6/UBE2D2/RMND5B/WDR26/FB

											XO39/FZR1/FBXL2/BUB1B/KCTD10/SHARPIN/UBE2S/BARD1/DERL3/TRAF2/KBTBD8/RCHY1/DDA1/DCUN1D3/RMND5A/KLHDC2/KLHL8/RNF7/DCUN1D5/CDC16/ANKRD9/KLHL7/CUL2/FBXO11/UBE2C
HP_ABNORMALITY_OF_THE_CHIN	HP_ABNORMALITY_OF_THE_CHIN	HP_ABNORMALITY_OF_THE_CHIN	85	-0.239205668	-1.857980112	0.003922459	0.040173528	0.032166839	659	tags=38%, list=21%, signal=31%	FAM20C/BAZ1B/CUL7/PTCH2/H19/EDA/GLB1/CDH11/COX7B/SLC6A17/TRIO/ANKRD11/STT3A/PQBP1/IGF2/RALA/TBC1D2B/MAF/CDC42BPB/NFIA/PDGFRB/MED12/FBXW11/SH3PXD2B/SETBP1/MMP23B/CCDC8/FBN1/ZMPSTE24/FGFR1/RNF113A/ABL1
GOBP_NEGATIVE_REGULATION_OF_CELL_DIFFERENTIATION	GOBP_NEGATIVE_REGULATION_OF_CELL_DIFFERENTIATION	GOBP_NEGATIVE_REGULATION_OF_CELL_DIFFERENTIATION	123	-0.20347569	-1.739698709	0.003961489	0.040452412	0.032390141	1259	tags=52%, list=39%, signal=33%	LTF/TNF/CBFB/TBX6/PTPN2/THY1/FOXE3/IRX3/NF1/RARG/INHBA/NR1H2/PIAS3/SOCS1/HAND2/GDI1/WNT1/C1QC/SNAI2/CCDC85B/FGL2/WNT3A/NFATC4/ADIPOQ/PPARA/MAFB/MEN1/BTG2/SMAD7/FRS2/XBP1/TMEM119/TCTA/MYC/LRP3/YAP1/NRP1/BMP4/PKP2/SIRT2/LYN/DAB1/MIXL1/PITX3/SMO/LAG3/CEBPA/TRIO/CHRD/IGF2/JDP2/PPP3CA/NFE2L2/ITGB1/CDK5/HMGB1/ADIPOR1/NKX2-5/FBN1/STAT3/EIF2AK4/PTN/TSPO/ISL2
GOBP_CARDIAC_SEPTUM_MORPHOGENESIS	GOBP_CARDIAC_SEPTUM_MORPHOGENESIS	GOBP_CARDIAC_SEPTUM_MORPHOGENESIS	15	-0.486503409	-1.978541427	0.0040083	0.040844067	0.032703738	1363	tags=87%, list=43%, signal=50%	ZFPM2/ENG/GATA4/WNT11/NRP2/MDM2/CITED2/SMAD7/NRP1/BMP4/SMO/NKX2-5/PARVA
HP_APLASIA_HYPOPLASIA_OF_THE_SPLEEN	HP_APLASIA_HYPOPLASIA_OF_THE_SPLEEN	HP_APLASIA_HYPOPLASIA_OF_THE_SPLEEN	13	0.505166157	2.069336389	0.004078356	0.04147044	0.033205273	469	tags=54%, list=15%, signal=46%	ZMYND10/KATNB1/WNT3/NDE1/SMAD2/FOXJ1/DNAI1
GOBP_PROTEIN_DNA_COMPLEX_ORGANIZATION	GOBP_PROTEIN_DNA_COMPLEX_ORGANIZATION	GOBP_PROTEIN_DNA_COMPLEX_ORGANIZATION	148	0.177266833	1.761493197	0.004122946	0.041835776	0.033497797	786	tags=36%, list=25%, signal=28%	SRPK1/GMNN/SUGT1/SMCHD1/BRD1/CHAF1B/CENPE/RAD51/SFMBT1/HDGFL1/DOT1L/SUV39H2/DR1/CENPH/PRKAA1/ITGB3BP/TPR/UBN1/PAXIP1/RUVBL1/ATF7IP/SUZ12/ZMYND11/PCGF6/C6orf89/ACTR6/IWS1/BRD7/BANP/VRK1/NAP1L4/CGGBP1/TAF6/PHF21A/TRIM28/CHD1L/TAF10/RBM14/SPHK2/SMC6/TNP2/MIE

											R2/CBX1/PPHLN1/CHD5/MED4/ARID4B/GTF2E1/BRD9/BAZ1A/TAF9/SETD1A/SMARCC1
GOBP_RESPONSE_TO_XENOBIOTIC_STIMULUS	GOBP_RESPONSE_TO_XENOBIOTIC_STIMULUS	GOBP_RESPONSE_TO_XENOBIOTIC_STIMULUS	88	-0.242859667	-1.90415688	0.004159537	0.042118578	0.033724236	1788	tags=82%, list=56%, signal=37%	SLC34A1/UGT1A6/ABCB1/GRIN1/GAL/CYP2C18/AIP/EHMT2/PDE4A/HMGCS2/ABCD3/NNMT/ADA/ACSL1/CARD9/ATG5/ADAM17/NAT2/UMOD/KCNQ3/S100A12/ADD3/LTA/FMO5/CAD/OXSR1/ENG/GATA4/CYP2A13/TNF/SLC26A5/BCHE/GCLC/CYP1B1/BCL2/NQO1/CYP2S1/CYP2U1/MDM2/HTR1B/ADIPOQ/LIPA/FMO1/SLC22A7/BPHL/PARP4/CAT/EMX1/FOSL2/RELA/GSTO1/MYC/ALAD/FECH/LYN/SLC29A3/CDH3/MAOB/SCGB1A1/UBE2B/AKR1C1/GNA12/SLC29A1/ITGA3/CBR3/CYP2W1/NFE2L2/CBR1/ACAA1/CDK4/TSPO/ABL1
GOCC_SM_LIKE_PROTEIN_FAMILY_COMPLEX	GOCC_SM_LIKE_PROTEIN_FAMILY_COMPLEX	GOCC_SM_LIKE_PROTEIN_FAMILY_COMPLEX	18	0.434900524	2.018945227	0.004191723	0.042355692	0.033914092	282	tags=39%, list=9%, signal=36%	DDX20/BUD13/GEMIN4/PRPF18/GEMIN6/PRPF40B/SF3A1
GOBP_ENDOTHELIAL_CELL_PROLIFERATION	GOBP_ENDOTHELIAL_CELL_PROLIFERATION	GOBP_ENDOTHELIAL_CELL_PROLIFERATION	36	-0.343205643	-2.008992514	0.004242335	0.042777612	0.034251922	919	tags=56%, list=29%, signal=40%	NRP2/LIPA/CD34/LOXL2/ALOX5/VEGFB/NRP1/BMP4/TEK/GHSR/LRG1/ANG/IGF2/AKT1/APLN/HMGB1/MMP14/STAT3/FGFR1/ITGB1BP1
GOBP_MONONUCLEAR_CELL_MIGRATION	GOBP_MONONUCLEAR_CELL_MIGRATION	GOBP_MONONUCLEAR_CELL_MIGRATION	38	-0.335306846	-1.999734444	0.004256571	0.042831742	0.034295264	1352	tags=71%, list=42%, signal=42%	CCL8/OXSR1/TNF/CCL22/CCR1/CTSG/APOD/GPR15/CXCL13/CCL5/CCR5/SPN/C3AR1/ALOX5/CCL21/FOLR2/PYCARD/AIF1/LYN/AIRE/AGER/TRPM2/AKT1/MSN/HMGB1/ITGB7/ABL1
GOBP_MITOTIC_SISTER_CHROMATID_SEPARATION	GOBP_MITOTIC_SISTER_CHROMATID_SEPARATION	GOBP_MITOTIC_SISTER_CHROMATID_SEPARATION	17	0.438069493	2.023175817	0.004320809	0.043105287	0.034514291	903	tags=71%, list=28%, signal=51%	CDCA8/TPR/TRIP13/CDK5RAP2/MAD2L1BP/DYNC1LI1/BUB1B/ESPL1/ZW10/MAD2L2/CDC16/UBE2C
GOBP_ANTIGEN_RECEPTOR_MEDIATED_SIGNALING_PATHWAY	GOBP_ANTIGEN_RECEPTOR_MEDIATED_SIGNALING_PATHWAY	GOBP_ANTIGEN_RECEPTOR_MEDIATED_SIGNALING_PATHWAY	42	-0.318927388	-1.95323539	0.004318709	0.043105287	0.034514291	1291	tags=69%, list=40%, signal=42%	FYN/PRAM1/CD276/PTPN2/THY1/SYK/BTN1A1/BTNL2/BCL2/TNFRSF21/CACNB3/LIPA/PRKCH/CD79A/HLA-A/FOSL2/BTN2A2/RELA/ELF1/BAX/LYN/LIME1/HLA-DQB1/EIF2B1/CSK/PTPRC/HLA-DRB3/NFKB1/ABL1
GOBP_ENDOCHONDRAL_BONE_MOR	GOBP_ENDOCHONDRAL_BONE_MOR	GOBP_ENDOCHONDRAL_BONE_MORPHOGE	18	-0.440625113	-1.931800859	0.004328378	0.043105287	0.034514291	732	tags=61%, list=23%, signal=47%	FOSL2/TMEM119/INPPL1/BMP4/TEK/ANXA6/COL3A1/COL27A1/MEF2

PHOGENESIS	PHOGENESIS	NESIS									D/MMP14/ZMPSTE24
GOCC_NUCLEAR_SPECK	GOCC_NUCLEAR_SPECK	GOCC_NUCLEAR_SPECK	77	0.229613852	1.909712961	0.004320869	0.043105287	0.034514291	1020	tags=55%, list=32%, signal=38%	SRPK1/SURF2/DZIP1/BRD1/ELL3/PHF7/PRPF18/PRKAA1/PRPF40B/SF3A1/PPP1CC/TFIP11/WRN/LPXN/CNOT7/DGKZ/GTF2H2/S100PBP/RBM14/BARD1/SMC6/YLPM1/CHD5/RCHY1/KIF22/PRPF40A/AKAP8L/MBD4/SAP130/SETD1A/RBBP6/HIPK1/POLI/PIP5K1A/PLRG1/PRPF6/CSNK1A1/DHX15/HP1BP3/EP400/PPP1R8/PRPF4B
GOBP_REGULATION_OF_BODY_FLUID_LEVELS	GOBP_REGULATION_OF_BODY_FLUID_LEVELS	GOBP_REGULATION_OF_BODY_FLUID_LEVELS	81	-0.246847474	-1.886180011	0.004294384	0.043105287	0.034514291	1062	tags=49%, list=33%, signal=34%	AGR2/AQP6/NR1H2/ATP7B/SOCS2/VWF/WNT3A/PLAU/HSPB1/EMP2/ILK/CSN2/FOSL2/CORO2B/XBP1/OAS2/SERPINE2/PLSCR1/WFS1/TFPI/SERPINA1/ENTPD2/LYN/COL3A1/HAS2/GNA12/F10/ADRA2B/FCER1G/PPP3CA/SLC29A1/NFE2L2/MUC2/F2RL3/ST3GAL4/APLN/CLIC1/HEG1/HPS6/ITPK1
GOBP_REGULATION_OF_MYELOID_CELL_DIFFERENTIATION	GOBP_REGULATION_OF_MYELOID_CELL_DIFFERENTIATION	GOBP_REGULATION_OF_MYELOID_CELL_DIFFERENTIATION	37	-0.335256818	-1.988927778	0.004529267	0.044968511	0.03600617	1259	tags=68%, list=39%, signal=41%	LTF/TNF/PTPN2/CCR1/NF1/RARG/INHBA/PIAS3/C1QC/ETS1/ADIPOQ/CD4/MAFB/TCTA/CEBPB/MYC/HCLS1/ISG15/LYN/TYROBP/IL17A/TFE3/PPP3CA/FBN1/STAT3
GOBP_NON_MOTILE_CILIUM_ASSEMBLY	GOBP_NON_MOTILE_CILIUM_ASSEMBLY	GOBP_NON_MOTILE_CILIUM_ASSEMBLY	11	0.513561378	1.947110967	0.004534092	0.044968511	0.03600617	1253	tags=91%, list=39%, signal=56%	IFT74/IFT122/CEP250/CEP350/BBS4/MKKS/CEP290/MAPRE1/TMEM80/NPHP3
GOBP_REGULATION_OF_EXTRINSIC_APOPTOTIC_SIGNALING_PATHWAY_IN_ABSENCE_OF_LIGAND	GOBP_REGULATION_OF_EXTRINSIC_APOPTOTIC_SIGNALING_PATHWAY_IN_ABSENCE_OF_LIGAND	GOBP_REGULATION_OF_EXTRINSIC_APOPTOTIC_SIGNALING_PATHWAY_IN_ABSENCE_OF_LIGAND	12	-0.546392474	-2.043661347	0.004579983	0.045330574	0.036296073	1249	tags=92%, list=39%, signal=56%	TNF/NF1/INHBA/SRPX/BCL2/SNAI2/BCL2L1/AKT1/IFI6/PPP2R1A/FGFR1
GOBP_CHEMOKINE_PRODUCTION	GOBP_CHEMOKINE_PRODUCTION	GOBP_CHEMOKINE_PRODUCTION	21	-0.424046255	-1.954162398	0.004604912	0.045484104	0.036419004	1671	tags=90%, list=52%, signal=44%	CARD9/ADAM17/EGR1/CHIA/TNF/SYK/APOD/FFAR2/SNAI2/ADIPOQ/PYCARD/AIF1/AIRE/AGER/IL17A/EIF2AK2/HMGB1/HAVCR2/MEFV
GOMF_PROTEIN_CONTAINING_COMPLEX_BINDING	GOMF_PROTEIN_CONTAINING_COMPLEX_BINDING	GOMF_PROTEIN_CONTAINING_COMPLEX_BINDING	357	-0.147482217	-1.656449813	0.004694194	0.046271341	0.037049342	826	tags=34%, list=26%, signal=28%	PPARA/HLA-DMA/IFRD2/ITGB8/INS/HLA-A/MAFB/CHMP6/CITED2/ADD1/M6PR/MEN1/CORO6/ABI3/FOSL2/REPIN1/DET1/CORO2B/SMAD7/HLA-E/RELA/ITGAM/CTCF/TGFBI/RB

											M23/CEBPB/MYC/YAP1/GNB1/HR/GNAI1/UBTF/CCNF/AIF1/HCLS1/VPS4A/WFS1/CBX6/EOMES/PKP2/HLA-DOB/IQGAP1/ALAD/ISG15/PCOLCE/RNF5/SIRT2/CENPB/TFPT/HLA-DRB5/LYN/HLA-DMB/AIRE/TPM2/STAB1/AGER/FZD4/IGF2R/ANXA6/FGF1/CEBPA/GTPBP6/RNF185/CD177/PODN/COL3A1/HLA-DQB1/PQBP1/IGF2/ATF4/JDP2/RAC1/PRMT2/PTPRC/GNA12/MNT/CDC42BPB/FCER1G/IQGAP3/PPP3CA/HLA-DRB3/ITGA3/GLI2/SUCLG2/SUPT6H/NFIA/SLC30A9/ITGB1/NAP1L3/MED12/C8A/ADAM15/EBF2/CFL2/NAP1L1/FBXW11/PES1/ALOX5AP/LASP1/HMGB1/SEC61A1/NFKB1/MMP14/ITGA9/CTSK/ITGB7/CHRNB2/NKX2-5/FBN1/STAT3/PTN/KRT14/GADD45A/DERL1/SHC1/CTBP1/BCL2L2/ITGB1BP1/ABL1/MBD2
GOBP_REGULATION_OF_SUBSTRATE_ADHESION_DEPENDENT_CELL_SPREADING	GOBP_REGULATION_OF_SUBSTRATE_ADHESION_DEPENDENT_CELL_SPREADING	GOBP_REGULATION_OF_SUBSTRATE_ADHESION_DEPENDENT_CELL_SPREADING	15	-0.477367751	-1.941387982	0.004737066	0.046598838	0.037311568	1373	tags=87%, list=43%, signal=50%	LIMS2/MYOC/TRIOBP/ILK/MYADM/NRP1/PKP2/DAB2/HAS2/RAC1/ARHGEF7/ITGB1BP1/ABL1
HP_BONE_MARROW_HYPOCELLULARITY	HP_BONE_MARROW_HYPOCELLULARITY	HP_BONE_MARROW_HYPOCELLULARITY	12	0.526711222	2.092919515	0.004755685	0.046686911	0.037382088	738	tags=67%, list=23%, signal=51%	MDM4/VPS33A/RAD51/CLPB/UBE2T/MAD2L2/LBR/FANCC
HP_UPPER_LIMB_JOINT_CONTRACTURE	HP_UPPER_LIMB_JOINT_CONTRACTURE	HP_UPPER_LIMB_JOINT_CONTRACTURE	73	-0.247396469	-1.840268987	0.004779703	0.046827521	0.037494674	1135	tags=53%, list=35%, signal=35%	ZDHHC9/LGI4/PTRH2/ADAMTSL2/BCR/EMD/GPKOW/NDRG1/MYOD1/HSPB1/KCNAB2/FHL1/DHCR24/MAFB/B3GAT3/CTCF/COL6A3/LFNG/TPM2/SLC29A3/CDH3/ANTXR2/PQBP1/IGF2/OSGEP/TBC1D2B/PDGFRB/MED12/FBXW11/SH3PXD2B/MMP23B/TOR1A/FBN1/ZMPSTE24/FGFR1/KRT14/PLOD1/EFNB1/PDXK
GOBP_POSITIVE_REGULATION_OF_HYDROLASE_ACTIVITY	GOBP_POSITIVE_REGULATION_OF_HYDROLASE_ACTIVITY	GOBP_POSITIVE_REGULATION_OF_HYDROLASE_ACTIVITY	116	-0.210448187	-1.775374843	0.004882748	0.047740233	0.03822548	1325	tags=55%, list=41%, signal=34%	SH3BP1/PDGFRA/APAF1/FYN/CLDN4/TNF/THY1/SYK/CCL22/PPP1R15A/TBC1D17/WNT11/RPS27L/NF1/BCR/NR1H2/MAP3K5/TSC1/CXCL13/CCL5/BAD/ARHGAP27/PRSS22/TBC1

											D16/FNTA/NET1/CCL21/RHOG/PYCARD/MYC/GRTP1/BAX/RGS11/CLDN3/CALM3/RAPGEF1/ANG/LYN/APH1A/AGER/TNFSF10/BBC3/RGS10/NTF3/RSU1/RALGDS/AKT1/TBC1D2B/ARHGEF7/PTPRC/GNA12/RGS6/PERP/PDGFRB/ARF4/ITGB1/HMGB1/PHB2/MEFV/RHOC/CTSK/STAT3/ATP2A3/FGFR1
GOBP_POSITIVE_REGULATION_OF_PROTEIN_MODIFICATION_BY_SMALL_PROTEIN_CONJUGATION_OR_REMOVAL	GOBP_POSITIVE_REGULATION_OF_PROTEIN_MODIFICATION_BY_SMALL_PROTEIN_CONJUGATION_OR_REMOVAL	GOBP_POSITIVE_REGULATION_OF_PROTEIN_MODIFICATION_BY_SMALL_PROTEIN_CONJUGATION_OR_REMOVAL	34	0.323728181	1.958374124	0.004947139	0.048272083	0.038651331	918	tags=62%, list=29%, signal=45%	SAE1/CDC14B/CRY1/PAXIP1/MAGEC2/UBE2N/MAGEA2B/FZR1/UBE3A/PINK1/UBE2S/BIRC8/RCHY1/GSK3B/DCUN1D3/DCUN1D5/VCP/TRAF6/HSPBP1/UBE2C/TBC1D7
GOCC_CELL_SUBSTRATE_JUNCTION	GOCC_CELL_SUBSTRATE_JUNCTION	GOCC_CELL_SUBSTRATE_JUNCTION	107	-0.219545111	-1.802447602	0.004989036	0.048582749	0.038900081	911	tags=43%, list=28%, signal=32%	ARPC1B/PLAU/HSPB1/FHL1/ILK/ITGB8/PARVG/FAM107A/ADD1/CAT/NFASC/CORO2B/GRB7/RHOG/TSPAN9/CPNE3/NRP1/TEK/IQGAP1/DAB2/IGF2R/ANXA6/FLOT2/RSU1/RALA/ALKBH6/RAC1/ARHGEF7/PTPRC/GNA12/CTTN/ITGA3/PDGFRB/ITGB1/SCARB2/MSN/LASP1/MMP14/CAP1/ITGB7/RHOU/GDI2/PARVA/TRIP6/SHC1/ITGB1BP1
HP_ABNORMAL_ORAL_MUCOSA_MORPHOLOGY	HP_ABNORMAL_ORAL_MUCOSA_MORPHOLOGY	HP_ABNORMAL_ORAL_MUCOSA_MORPHOLOGY	88	-0.239102882	-1.874701562	0.005022241	0.048807693	0.039080192	763	tags=40%, list=24%, signal=31%	MEN1/CAT/ELN/RELA/HLA-B/ITGAM/FAM20C/BAZ1B/B4GALT7/RHBDF2/EDA/GLB1/SLC29A3/CNTNAP1/ANTXR2/COL3A1/HLA-DQB1/CTSC/TBC1D2B/F10/PERP/PDGFRB/HPS1/SCARB2/SH3PXD2B/ABCD1/SETBP1/IRAK1/MMP14/MEFV/IFNGR1/UBE2L3/TRIM8/STAT3/KRT14
GOBP_GLIAL_CELL_DEVELOPMENT	GOBP_GLIAL_CELL_DEVELOPMENT	GOBP_GLIAL_CELL_DEVELOPMENT	27	-0.370814668	-1.917139963	0.005041605	0.048897495	0.039152097	1412	tags=78%, list=44%, signal=44%	ASCL1/TPPP/MYOC/TNF/ZNF488/LGI4/NF1/NDRG1/ILK/SIRT2/LYN/CNTNAP1/AGER/SMO/PRX/AKT1/EIF2B1/CDK5/MED12/LAMC3/IFNGR1
GOMF_UBIQUITIN_LIKE_PROTEIN_PEPTIDASE_ACTIVITY	GOMF_UBIQUITIN_LIKE_PROTEIN_PEPTIDASE_ACTIVITY	GOMF_UBIQUITIN_LIKE_PROTEIN_PEPTIDASE_ACTIVITY	19	0.436300572	2.124474391	0.005053949	0.048918984	0.039169303	836	tags=63%, list=26%, signal=47%	USP1/SENP1/USP6/USP30/USP37/SENP5/USPL1/ATXN3/USP31/USP8/USP10/USP48

HP_DISPLACEMENT_OF_THE_URETHRAL_MEATUS	HP_DISPLACEMENT_OF_THE_URETHRAL_MEATUS	HP_DISPLACEMENT_OF_THE_URETHRAL_MEATUS	72	-0.246430296	-1.815265821	0.00508031	0.049075794	0.03929486	532	tags=35%, list=17%, signal=30%	HEATR3/CDH11/LFNG/COX7B/COL3A1/ANKRD11/PQBP1/IGF2/PLAG1/PRPS1/RAC1/CDC42BPB/SLC31A1/MID1/MED12/SETBP1/MMP23B/SSR4/TRIM8/CCDC8/ZMPSTE24/FGFR1/CTBP1/EFNB1/ABL1
GOCC_CILIARY_TIP	GOCC_CILIARY_TIP	GOCC_CILIARY_TIP	12	0.523963463	2.082001122	0.005092363	0.049094034	0.039309465	208	tags=50%, list=6%, signal=47%	KIF3A/DYNLRB2/IFT74/DYNLL2/CLUAP1/IFT122
HP_ABNORMALITY_OF_THE_DENTITION	HP_ABNORMALITY_OF_THE_DENTITION	HP_ABNORMALITY_OF_THE_DENTITION	196	-0.180289334	-1.761856095	0.005172679	0.049769005	0.039849913	613	tags=30%, list=19%, signal=26%	B4GALT7/CUL7/PTCH2/BMP4/H19/EDA/GLB1/CDH11/AIRE/TPM2/SLC29A3/KRT85/COX7B/CDH3/PLXNA1/TRIO/ANTXR2/COL3A1/PROKR2/ANKRD11/HLA-DQB1/PQBP1/IGF2/CTSC/AKT1/OSGEP/PAX9/TBC1D2B/ATP1A3/HS6ST1/PERP/MAF/CDC42BPB/PPP3CA/GLI2/PRR12/PDGFRB/MID1/KCNB1/MED12/SH3PXD2B/SETBP1/ZBTB7A/SSR4/KREMEN1/CTSK/CCDC8/FBN1/PURA/STAT3/ZMPSTE24/DPH5/FGFR1/KRT14/PLOD1/RNF113A/CTBP1/EFNB1/ABL1
GOBP_POSITIVE_REGULATION_OF_OSTEOBLAST_DIFFERENTIATION	GOBP_POSITIVE_REGULATION_OF_OSTEOBLAST_DIFFERENTIATION	GOBP_POSITIVE_REGULATION_OF_OSTEOBLAST_DIFFERENTIATION	17	-0.457690571	-1.949528542	0.005199688	0.04992069	0.039971367	822	tags=65%, list=26%, signal=48%	ILK/TMEM119/FAM20C/CEBPB/LRP3/YAP1/BMP4/IFITM1/CEBPA/PPP3CA/CLIC1
HP_LIMB_UNDERGROWTH	HP_LIMB_UNDERGROWTH	HP_LIMB_UNDERGROWTH	47	0.275671062	1.915366477	0.005219451	0.04992069	0.039971367	747	tags=47%, list=23%, signal=36%	DONSON/COL9A3/RAD51/PSMD12/CLPB/IFT122/UBE2T/RECQL4/GLUL/SMC3/TRIP11/GNPAT/AFF4/KIAA0753/POLE/ATR/COMP/ALG9/MAD2L2/LBR/FANCC/CHN1
GOBP_MYELOID_LEUKOCYTE_DIFFERENTIATION	GOBP_MYELOID_LEUKOCYTE_DIFFERENTIATION	GOBP_MYELOID_LEUKOCYTE_DIFFERENTIATION	56	-0.279058222	-1.874954154	0.005214901	0.04992069	0.039971367	1259	tags=59%, list=39%, signal=36%	LTF/TNF/IL15/PTPN2/CCR1/EFNA2/NF1/INHBA/PIAS3/SOCS1/C1QC/ADIPOQ/CD4/ITGB8/MAFB/CITED2/FOSL2/TCTA/FAM20C/CEBPB/MYC/HCLS1/BMP4/LYN/TYROBP/CEBPA/LTBR/IL17A/TFE3/PPP3CA/FBN1/SH3PXD2A/EIF2AK1
GOBP_CALCIUM_ION_TRANSMEMBRANE_IMPORT_INTO_CYTOSOL	GOBP_CALCIUM_ION_TRANSMEMBRANE_IMPORT_INTO_CYTOSOL	GOBP_CALCIUM_ION_TRANSMEMBRANE_IMPORT_INTO_CYTOSOL	42	-0.315841851	-1.934338359	0.00525079	0.049993532	0.040029691	1331	tags=64%, list=42%, signal=38%	SRI/FYN/DIAPH1/ATP1A2/THY1/CACNA1B/CACNA1E/FKBP1A/BCL2/CACNB3/IBTK/CCR5/TPCN1/CCL21/GSTO1/ITPR1/BAX/CALM3/LYN/LIME1/XCR1/TRPM2/P2RX2/PTPRC/PPP

										3CA/F2RL3/ABL1
GOBP_POSITIVE_REGULATION_OF_INTERLEUKIN_1_PRODUCTION	GOBP_POSITIVE_REGULATION_OF_INTERLEUKIN_1_PRODUCTION	GOBP_POSITIVE_REGULATION_OF_INTERLEUKIN_1_PRODUCTION	16	-0.46301428	-1.927924969	0.005267371	0.049993532	0.040029691	1249	tags=81%, list=39%, signal=50%	TNF/TRIM16/ORM2/HSPB1/RELA/PYCARD/AGER/TYROBP/IL17A/HMGB1/HAVCR2/MEFV/STAT3
GOBP_LEUKOCYTE_ADHESION_TO_VASCULAR_ENDOTHELIAL_CELL	GOBP_LEUKOCYTE_ADHESION_TO_VASCULAR_ENDOTHELIAL_CELL	GOBP_LEUKOCYTE_ADHESION_TO_VASCULAR_ENDOTHELIAL_CELL	15	-0.473130609	-1.924156118	0.005267079	0.049993532	0.040029691	1249	tags=80%, list=39%, signal=49%	TNF/SELPLG/ETS1/SPN/ALOX5/RELA/CCL21/LRG1/ITGB1/ST3GAL4/IRAK1/ITGB7
GOBP_POTASSIUM_ION_TRANSPORT	GOBP_POTASSIUM_ION_TRANSPORT	GOBP_POTASSIUM_ION_TRANSPORT	47	-0.300704251	-1.898364243	0.00526847	0.049993532	0.040029691	1866	tags=85%, list=58%, signal=36%	GAL/KCNJ9/KCNG1/AQP1/HCN4/KCNMB1/KCNH7/KCNMB3/SLC12A3/KCNQ3/CDK2/ATP1B2/KCNS3/CAV3/HCN3/KCNJ6/SLC17A7/KCNAB3/OXSR1/ATP1A2/KCNE3/ACTN2/KCNS2/ATP1B1/SLC12A8/TSC1/KCNAB2/FHL1/TMCO3/SLC9A8/KCNJ1/KCNH4/SLC12A9/KCNB2/ATF4/KCNK5/ATP1A3/CCDC51/KCNB1/ITGB1

TableS6: GSEA results of SHC1

	ID	Description	setSize	enrichmentScore	NES	pvalue	p.adjust	qvalue	rank	leading_edge	core_enrichment
GOBP_MALE_GAMETE_GENERATION	GOBP_MALE_GAMETE_GENERATION	GOBP_MALE_GAMETE_GENERATION	106	-0.48010934	-4.338284416	4.72E-19	2.27E-15	1.72E-15	598	tags=57%, list=19%, signal=47%	IQCG/PACRG/INHBB/TNP2/SPAG4/IFT20/HSF2/PLEKHA1/BOLL/SPOCD1/WDR33/PARP11/AFF4/TRIP13/RUVBL1/PIWIL2/PPP1CC/DZIP1/MAST2/TESK2/TDRKH/PRSS21/CSNK2A2/ELL3/ACTL7A/TSSK2/FKBP6/AKAP4/SFMBT1/MYCBP/TCP11/SRPK1/KLHL10/SETX/CCDC42/ACRBP/SPATA6/HSPA2/CCIN/IQCF1/CDC25C/MYBL1/TDRD7/SPA17/DAZAP1/ZPBP2/ROPN1L/FOXJ1/LZTFL1/TCFL5/STRBP/CATSPER2/RPL39L/PAFAH1B1/PTTG1/MYCBPAP/RFX2/SPAG8/TTC21A/TBPL1
GOBP_SEXUAL_REPRODUCTION	GOBP_SEXUAL_REPRODUCTION	GOBP_SEXUAL_REPRODUCTION	180	-0.362979976	-3.770046202	8.59E-16	2.07E-12	1.57E-12	541	tags=43%, list=17%, signal=38%	TNP2/SPAG4/IFT20/SMC3/TIAL1/HSF2/TUBG2/PLEKHA1/BOLL/ESPL1/SPOCD1/WDR33/ANAPC10/PARP11/UBE3A/AFF4/TRIP13/RUVBL1/PIWIL2/TUBG1/PPP1CC/DZIP1/MAST2/LYZL6/TESK2/SMC1B/TDRKH/WNT3/FZR1/PRSS21/SPACA3/CSNK2A2/ELL3/ACTL7A/TSSK2/STAG3/RAD51/FKBP6/CKS2/AKAP4/SFMBT1/MYCBP/TCP11/AURKC/SRPK1/KLHL10/SETX/CCDC42/ACRBP/SPATA6/HSPA2/CCIN/IQCF1/CDC25C/MYBL1/TDRD7/SPA17/DAZAP1/HSPA1L/CCNB2/ZPBP2/ROPN1L/FOXJ1/LZTFL1/TCFL5/DDX20/STRBP/CATSPER2/RPL39L/PAFAH1B1/PTTG1/MYCBPAP/RFX2/SPAG8/PTTG2/TTC21A/TBPL1
GOBP_GAMETE_GENERATION	GOBP_GAMETE_GENERATION	GOBP_GAMETE_GENERATION	136	-0.382276814	-3.720219803	1.30E-14	2.09E-11	1.58E-11	598	tags=47%, list=19%, signal=40%	IQCG/PACRG/INHBB/TNP2/SPAG4/IFT20/TIAL1/HSF2/PLEKHA1/BOLL/SPOCD1/WDR33/PARP11/AFF4/TRIP13/RUVBL1/PIWIL2/PPP1CC/DZIP1/MAST2/TESK2/TDRKH/WNT3/PRSS21/CSNK2A2/ELL3/ACTL7A/TSSK2/FKBP6/AKAP4/SFMBT1/MYCBP/TCP11/SRPK1/KLHL10/SETX/CCDC42/ACRBP/SPATA6/HSPA2/CCIN/IQCF1/CDC25C/MYBL1/TDRD7/SPA17/DAZAP1/CCNB2/ZPBP2/ROPN1L/

GOCC_MICROTUBULE_CYTOSKELETON	GOCC_MICROTUBULE_CYTOSKELETON	GOCC_MICROTUBULE_CYTOSKELETON	275	-0.263012463	-2.978924607	5.88E-12	7.07E-09	5.36E-09	679	tags=39%, list=22%, signal=33%	FOXJ1/LZTFL1/TCFL5/DDX20/STRBP/CATSPER2/RPL39L/PAFAH1B1/PTTG1/MYCBPAP/RFX2/SPAG8/TTC21A/TBPL1HIPK1/DCUN1D5/KIAA0753/ARL2/RAB11FIP4/WRN/HOOK2/TUBB/E2F1/CDK5RAP2/NME7/KIF22/KIF4A/RASSF1/IQCG/SLC1A4/PACRG/KIFAP3/CDC7/NUP93/LYST/CCDC117/ZNF415/RASSF7/IQCB1/IFT20/MARK1/SMC3/MAD2L1BP/PINX1/TUBG2/FKBP4/CEP350/ESPL1/MAD2L2/DYNC1LI1/PCNA/KBTBD8/HSPH1/RASSF3/VPS37A/CEP63/MAP2K2/TPX2/DNAI1/SMC6/SSNA1/MAP6D1/KIFC3/CEP250/NDE1/UBN1/RUVBL1/IFT74/KIF23/MAPKAPK2/SPATA7/TUBG1/DZIP1/MAST2/CENPE/KIF3A/BUB1B/CLUAP1/CEP72/KATNB1/CCDC77/RAB3IP/RRAGD/TBCCD1/C1orf112/EFHC1/CCHCR1/ARFGEF2/POLB/TSSK2/DNAH17/RAD51/TPR/DNAH8/AURKC/SPECC1/PARD6A/DYNLRB2/CDCA8/HSPA2/RANBP1/CDC14B/HMMR/DNALI1/ZMYND10/IFT122/KATNA1/SPAG5/CCNB2/RAE1/CCDC96/DYNLL2/LZTFL1/DR1/STRBP/PAFAH1B1/MAPKBP1/SPAG8/IQCD/CCDC112/WDR62
GOCC_NUCLEAR_PROTEIN_CONTAINING_COMPLEX	GOCC_NUCLEAR_PROTEIN_CONTAINING_COMPLEX	GOCC_NUCLEAR_PROTEIN_CONTAINING_COMPLEX	250	-0.272651784	-3.01257206	1.58E-11	1.52E-08	1.15E-08	807	tags=46%, list=26%, signal=37%	NHEJ1/MED4/LSM3/CTNNBL1/ATR/CREB3/BCCIP/SIRT1/CEBPG/XPO4/BRMS1/ERCC3/CHD5/TIMELESS/PDCD7/TAF9/SETD1A/MCM5/RAD50/SYMPK/TAF6/GTF2E1/BOP1/FANCC/POT1/SMARCC1/TAF10/ZC3H8/NFYB/SMG7/CPSF1/RCOR3/SUPT5H/E2F1/BRD9/POLR3E/RGPD5/GTF2H1/BAZ1A/TFDP1/CPSF2/NUP93/GTF2H2/CEBPZ/SMAD2/SPAG4/DNTTIP1/CSTF3/THOC5/SAP130/POLE/BRD7/NOL6/PRPF40A/TRIM28/SUZ12/SNUPN/BARD1/WDR33/ACTR6/PCNA/ANAPC10/PARP11/KPNB1/PCGF6/PRIM1/TEX10/CDK7/AFF4/UTP18/PAXIP1/PHF21A/SF3A1/RUVBL1/SAP30/GEMIN4/POLA2/EXOSC8/EXOSC4/PRPF18/PAF1/INTS7/

GOBP_CHROMOSOME_ORGANIZATION	GOBP_CHROMOSOME_ORGANIZATION	GOBP_CHROMOSOME_ORGANIZATION	130	-0.339731757	-3.269419372	1.06E-10	8.47E-08	6.42E-08	933	tags=59%, list=30%, signal=43%	CSTF1/BRD1/UBE2S/PRPF40B/BUB1B/FZR1/RFC4/NR1H3/INTS6/STAT4/ELL3/TFIP11/RAD51/SAE1/TPR/CLMN/DHX16/CCNH/SUPT3H/RANBP1/NUP88/PRPF38A/BUD13/TLE4/RAE1/DDX20/DR1/NUP155/AHCTF1/PRKRIP1/TBPL1/CPSF3NUP107/HMGA1/MAPRE1/SMC4/CHEK2/LCMT1/CDC16/RFC1/HMGB3/UBE2C/NUSAP1/PARP1/ATR/MAP3K4/BCCIP/ERCC3/MCM5/RAD50/ZW10/POT1/SMARCC1/TOP3A/WRN/AKAP8L/CDK5RAP2/YLPM1/PPHLN1/KIF22/KIF4A/GTF2H2/TOP1MT/SMC3/MAD2L1BP/PINX1/TUBG2/BRD7/TRIM28/ESPL1/MAD2L2/DYNC1LI1/PCNA/MAPK3/CHD1L/KPNB1/CENPH/TPX2/BLM/SMC6/ITGB3BP/TRIP13/RUVBL1/RECQL4/KIF23/TUBG1/CENPE/SMC1B/BUB1B/RFC4/ATF7IP/KATNB1/SUGT1/TFIP11/DHX30/STAG3/RAD51/TPR/AURKC/SETX/CDCA8/HSPA2/DOT1L/SPAG5/RAE1/SMCHD1/NUP155/PTTG1/PTTG2
GOBP_DNA_REPAIR	GOBP_DNA_REPAIR	GOBP_DNA_REPAIR	128	-0.340769348	-3.275997648	1.63E-10	1.12E-07	8.49E-08	866	tags=56%, list=27%, signal=43%	RFC1/RMI1/VCP/PPP4C/RCHY1/NHEJ1/POLI/PARP1/ATR/BCCIP/CIB1/SIRT1/CEBPG/ERCC3/TIMELESS/TAF9/MCM5/RAD50/TAF6/TP53BP1/FANCC/POT1/SMARCC1/TAF10/TOP3A/USP10/WRN/KIF22/ATXN3/GTF2H1/CDC7/GTF2H2/CCDC117/TEX264/SMC3/POLE/BRD7/TRIM28/BARD1/MAD2L2/WDR33/PCNA/CHD1L/CDK7/BLM/RNF138/SMC6/PAXIP1/TRIP13/RUVBL1/USP1/RECQL4/UBE2D3/CHAF1B/UBE2N/FZR1/RFC4/MDC1/NUDT1/UBE2T/TRAIP/C1orf112/TFIP11/POLB/RAD51/SETX/DOT1L/SUPT3H/CDC14B/RAD17/SMCHD1/PTTG1
GOBP_MULTICELLULAR_ORGANISM_REPRODUCTION	GOBP_MULTICELLULAR_ORGANISM_REPRODUCTION	GOBP_MULTICELLULAR_ORGANISM_REPRODUCTION	169	-0.304344091	-3.08836734	2.12E-10	1.27E-07	9.66E-08	598	tags=40%, list=19%, signal=34%	IQCG/PACRG/INHBB/KPNA6/TNP2/SPAG4/IFT20/TIAL1/HSF2/PLEKHA1/BOLL/SPOCD1/WDR33/PCNA/PARP11/AFF4/TRIP13/RUVBL1/PIWIL2/PPP1CC/DZIP1/MAST2/LYZL6/TESK2/TDRKH/WNT3/PRSS21/SPACA3/CSNK2A2/ELL3/ACT

											L7A/TSSK2/FKBP6/AKAP4/SFMBT1/MYCBP/TCP11/SRPK1/KLHL10/SETX/CCDC42/ACRBP/SPATA6/HSPA2/CCIN/IQCF1/CDC25C/MYBL1/TDRD7/SPA17/DAZAP1/CCNB2/ZPBP2/ROPN1L/FOXJ1/LZTFL1/TCFL5/DDX20/STRBP/CATSPER2/RPL39L/PAFAH1B1/PTTG1/MYCBPAP/RFX2/SPAG8/TTC21A/TBPL1
GOBP_CELL_CYCLE	GOBP_CELL_CYCLE	GOBP_CELL_CYCLE	375	-0.223342968	-2.658545149	2.46E-10	1.31E-07	9.95E-08	776	tags=40%, list=25%, signal=34%	CDK5R1/RABGAP1/BBS4/BCCIP/CIB1/SIRT1/ERCC3/TIMELESS/MCM5/PPP2R3B/RAD50/TAF6/TP53BP1/BOP1/ZW10/SMARCC1/DCUN1D3/TAF10/KIAA0753/TOP3A/ARL2/RAB11FIP4/WRN/AKAP8L/TUBB/E2F1/USP8/CDK5RAP2/KIF22/KIF4A/GTF2H1/RASSF1/TP53INP1/TFDP1/GAK/ECD/CDC7/BIRC8/ZBTB17/HERC5/SMC3/RBM14/EPB41L2/MAD2L1BP/PINX1/THOC5/TUBG2/POLE/BRD7/BANP/PRPF40A/BOLL/MAGEA4/PDCD2L/ZC3HC1/ESPL1/CAPN3/BARD1/MAD2L2/DYNC1LI1/PNPT1/PCNA/MAPK3/ANAPC10/MAPK6/KPNB1/MYH10/CEP63/CENPH/CDK7/USP37/TPX2/BLM/C6orf89/SMC6/SSNA1/PAXIP1/SENP5/CEP250/ITGB3BP/TRIP13/NDE1/RUVBL1/PIWIL2/KIF23/VRK1/PPP6C/PAF1/DONSON/ZMYND11/CHAF1B/INTS7/TUBG1/PPP1CC/UBE2S/FBXO7/CENPE/KIF3A/ASNS/SMC1B/TDRKH/BUB1B/FZR1/CEP72/KATNB1/SUGT1/MDC1/GTPBP4/CSNK2A2/C1orf112/EFHC1/STAG3/RAD51/FKBP6/GMNN/CKS2/CENPM/TPR/CRY1/AURKC/SRPK1/PARD6A/CCNH/CDCA8/CCDC42/HSPA2/DOT1L/RANBP1/CDC14B/CDC25C/MYBL1/NF2/RAD17/KATNA1/SPAG5/CCNB2/RAE1/SUV39H2/SH3GLB1/AURKAIP1/DR1/PAFAH1B1/KLF11/PTTG1/AHCTF1/SPAG8/PTTG2/WDR62/MDM4/CPSF3
GOMF_MOLECULAR_TRANSDUCER_ACTIVITY	GOMF_MOLECULAR_TRANSDUCER_ACTIVITY	GOMF_MOLECULAR_TRANSDUCER_ACTIVITY	239	0.288019692	2.824770673	3.38E-10	1.63E-07	1.23E-07	1925	tags=85%, list=61%, signal=36%	ANTXR2/LAG3/FGFR1/PDGFRB/SCARB2/IFNGR1/SMO/HLA-DRB3/HLA-DQB1/RGMB/TSPO/FOLR2/PLXNA1/STAT3/

											PTPRC/C3AR1/ITGB1/MRGPRF/OR51A7/OR5V1/OR52E4/IGF2R/TAS2R39/OR9K2/OR13C4/MAS1L/OR10T2/RAMP2/TEK/MRGPRX3/HLA-DOB/OR6V1/OR8G1/CHRNB2/CTSH/OR10J5/TAS2R9/OR6C4/GRIA4/DERL1/HRH3/OR1E2/NRP2/OR5F1/GPR39/OR52D1/FZD4/GPR161/GPR83/OR8A1/OR10H4/FCER1G/OR4K17/OR8H2/GRIK5/F2RL3/NR3C1/GPR135/BTN1A1/LTBP1/GRM1/GPR15/KIR2DL4/GPR78/CCR5/OR6X1/GFRA3/ENG/SIGLEC7/PROKR2/AGER/MTNR1A/CXCR3/OR8D1/GPR21/OR52B6/TAS2R40/TMEM63A/SQSTM1/NRP1/CRHR2/TAS2R10/CCR1/NR1H2/CNTNAP1/OR4K14/OR7G2/CD4/OR6Y1/SPN/OR3A3/GABRR1/SEC63/TAS2R41/DCBLD2/PTCH2/OR5AS1/OR5D18/OR4S1/OR2T4/ADIPOR2/OR7D2/FSHR/OR6M1/OR6K2/KISS1R/GPR3/OR2A12/CD7/OR8B8/OR8S1/MRGPRX2/CLDN4/OR6B2/RXFP3/GHSR/CX3CR1/OR51S1/OR2J2/OR1D4/OR51L1/OR52M1/REG4/ADIPOR1/ADRA2B/HTR1B/CLEC1A/OR13C8/EPOR/TAS2R60/GPR119/OR4D5/OR1D2/OR4M1/OR1F1/CHRNA2/TAAR6/XCR1/FFAR2/TAAR5/TRPV1/CD79A/P2RX2/SSTR3/TNFRSF4/OR4D1/PDGFRA/NOTCH4/RARG/OR10A4/IL9R/THRA/OR10H3/OR4P4/NPBWR2/GLP1R/OR2B11/BCAM/PAQR8/CNR2/PPARA/GRM6/IL1R1/CR2/OR51D1/HTR3A/IL2RG/BDKRB1/PROCR/RXRB/IL13RA1/TAS2R16/GPR12/M6PR/CLEC2D/OR52W1/PLXNB2/IL21R/TAS2R13/P2RY10/VAC14/OR11L1/P2RY8/GP1BB/ROR2/GPR152/CD300A/RRH/TSPAN12/CLDN3/CHRNA7/GIPR/MERTK/OR52N2/OR4A47/MCHR1/HCRTR1/RYK/EXTL3/GPR45/LRP6/GPR157/OR4E2
GOBP_DEVELOPMENTAL_PROCESS_INVOLVED_IN_	GOBP_DEVELOPMENTAL_PROCESS_INVOLVED_IN_RE	GOBP_DEVELOPMENTAL_PROCESS_INVOLVED_IN_REPRODUCTION	187	-0.28738262	-3.020800532	4.09E-10	1.75E-07	1.33E-07	541	tags=36%, list=17%, signal=32%	TNP2/SPAG4/IFT20/TIAL1/HSF2/PLEKHA1/FKBP4/BOLL/SPOCD1/WDR33/PARP11/UBE3A/AFF4/DNAJB6/NCOA4/TR

REPRODUCTION	PRODUCTION									IP13/RUVBL1/PIWIL2/SPINT2/PPP1CC/DZIP1/EIF2B4/MAST2/TESK2/TDRKH/ASB1/NUDT1/TESC/PRSS21/CSNK2A2/ELL3/ACTL7A/TSSK2/FKBP6/AKAP4/SFMBT1/MYCBP/TCP11/SRPK1/KLHL10/SETX/CCDC42/ACRBP/SPATA6/HSPA2/CCIN/IQCF1/CDC25C/MYBL1/TDRD7/SPA17/DAZAP1/ZPBP2/ROPN1L/FOXJ1/LZTFL1/TCFL5/DDX20/STRBP/CATSPER2/RPL39L/PAFAH1B1/PTTG1/MYCBPAP/RFX2/SPAG8/TTC21A/TBPL1
GOBP_G_PROTEIN_COUPLED_RECEPTOR_SIGNALING_PATHWAY	GOBP_G_PROTEIN_COUPLED_RECEPTOR_SIGNALING_PATHWAY	GOBP_G_PROTEIN_COUPLED_RECEPTOR_SIGNALING_PATHWAY	202	0.308377774	2.920293814	4.37E-10	1.75E-07	1.33E-07	1497	tags=70%, list=47%, signal=39%	UBQLN2/PDGFRB/RGS10/SMO/ITPR1/PPP3CA/KCTD12/RAC1/C3AR1/MRGPRF/OR51A7/OR5V1/OR52E4/IGF2R/TAS2R39/CCL21/OR9K2/OR13C4/MAS1L/OR10T2/RAMP2/GHRH/PALM/MRGPRX3/OR6V1/CCL5/OR8G1/OR10J5/TAS2R9/OR6C4/ABHD6/RPH3AL/HRH3/OR1E2/OR5F1/GPR39/OR52D1/FZD4/GPR161/GPR83/OR8A1/OR10H4/OR4K17/OR8H2/RELA/F2RL3/GNA12/GPR135/GRM1/GPR15/GPR78/APLN/GNAI1/CCR5/OR6X1/YWHAB/FRS2/ADM/RGS11/PROKR2/MTNR1A/CXCR3/OR8D1/GPR21/OR52B6/TAS2R40/CRTC3/CRHR2/RGS6/TAS2R10/CCR1/UCN3/OR4K14/CALM3/INS/OR7G2/OR6Y1/OR3A3/TAS2R41/OR5AS1/OR5D18/OR4S1/OR2T4/OR7D2/PRKAR1A/ROCK2/FSHR/OR6M1/OR6K2/GNB1/KISS1R/GPR3/OR2A12/ARRB2/OR8B8/OR8S1/MRGPRX2/CCL8/OR6B2/GNG7/ACTN2/RXFP3/GHSR/CX3CR1/RGS14/OR51S1/GRK1/OR2J2/OR1D4/OR51L1/PDCL/TSHB/OR52M1/ADRA2B/HTR1B/ADA/OR13C8/TAS2R60/GPR119/OR4D5/OR1D2/OR4M1/OR1F1/TAAR6/XCR1/FFAR2/TAAR5/CCL22/SSTR3/OR4D1/AKT1/ENTPD2/C3/CA2/OR10A4/OR10H3/OR4P4/NPBWR2/GLP1R/OR2B11/PCSK1N
GOMF_G_PROTEIN_COUPLED_RECEPTOR_ACTIVITY	GOMF_G_PROTEIN_COUPLED_RECEPTOR_ACTIVITY	GOMF_G_PROTEIN_COUPLED_RECEPTOR_ACTIVITY	130	0.353399735	3.009445194	5.08E-10	1.88E-07	1.43E-07	1487	tags=75%, list=47%, signal=41%	PDGFRB/SMO/C3AR1/MRGPRF/OR51A7/OR5V1/OR52E4/TAS2R39/OR9K2/OR13C4/MAS1L/OR10T2/RAMP2/MRGPRX3

											/OR6V1/OR8G1/OR10J5/TAS2R9/OR6C4/HRH3/OR1E2/OR5F1/GPR39/OR52D1/FZD4/GPR161/GPR83/OR8A1/OR10H4/OR4K17/OR8H2/F2RL3/GPR135/GRM1/GPR15/GPR78/CCR5/OR6X1/PROKR2/MTNR1A/CXCR3/OR8D1/GPR21/OR52B6/TAS2R40/CRHR2/TAS2R10/CCR1/OR4K14/OR7G2/OR6Y1/OR3A3/TAS2R41/OR5AS1/OR5D18/OR4S1/OR2T4/OR7D2/FSHR/OR6M1/OR6K2/KISS1R/GPR3/OR2A12/OR8B8/OR8S1/MRGPRX2/OR6B2/RXFP3/GHSR/CX3CR1/OR51S1/OR2J2/OR1D4/OR51L1/OR52M1/ADRA2B/HTR1B/OR13C8/TAS2R60/GPR119/OR4D5/OR1D2/OR4M1/OR1F1/TAAR6/XCR1/FFAR2/TAAR5/SSTR3/OR4D1/OR10A4/OR10H3/OR4P4/NPBWR2/GLP1R/OR2B11
HP_ABNORMAL_MALE_GERM_CELL_MORPHOLOGY	HP_ABNORMAL_MALE_GERM_CELL_MORPHOLOGY	HP_ABNORMAL_MALE_GERM_CELL_MORPHOLOGY	10	-0.894686605	-3.308679968	7.78E-10	2.66E-07	2.01E-07	342	tags=100%, list=11%, signal=89%	DZIP1/DNAH17/FKBP6/DNAH8/AURKC/KLHL10/DNALI1/CATSPER2/TTC21A
GOBP_DNA_METABOLIC_PROCESS	GOBP_DNA_METABOLIC_PROCESS	GOBP_DNA_METABOLIC_PROCESS	202	-0.270671685	-2.894118106	9.35E-10	2.66E-07	2.01E-07	866	tags=50%, list=27%, signal=38%	RFC1/RMI1/HMGB3/ARID4B/VCP/PPP4C/RCHY1/CIDEA/ENDOG/NHEJ1/POLI/PARP1/ATR/MAP3K4/BCCIP/CIB1/SIRT1/CEBPG/ERCC3/TIMELESS/TAF9/MCM5/RAD50/TAF6/TP53BP1/FANCC/POT1/SMARCC1/TAF10/TOP3A/USP10/WRN/YLPM1/TTF1/KIF22/ATXN3/GTF2H1/BAZ1A/TFDP1/CDC7/GTF2H2/CCDC117/TEX264/TOP1MT/SMC3/PINX1/POLE/BRD7/TRIM28/BARD1/MAD2L2/SPOCD1/WDR33/PCNA/MAPK3/CHD1L/RLF/PRIM1/POLRMT/CDK7/USP37/BLM/RNF138/SMC6/PAXIP1/TRIP13/RUVBL1/POLA2/EXOSC4/USP1/RECQL4/UBE2D3/GFER/DONSON/CHAF1B/UBE2N/FZR1/RFC4/ATF7IP/POLG2/MDC1/NUDT1/GTPBP4/UBE2T/FAF1/TRAIP/C1orf112/TFIP11/POLB/RAD51/GMNN/SETX/DOT1L/SUPT3H/CDC14B/RAD17/SMCHD1/TSN/KPNA2/PTTG1
GOBP_REPRODU	GOBP_REPRODUC	GOBP_REPRODUCTION	272	-0.242123664	-2.746804277	9.39E-10	2.66E-07	2.01E-07	541	tags=32%,	TNP2/SPAG4/IFT20/SMC3/TIAL1/HSF2/

CTION	TION									list=17%, signal=29%	TUBG2/PLEKHA1/FKBP4/BOLL/TRIM28/ESPL1/SPOCD1/WDR33/PCNA/ANAPC10/PARP11/UBE3A/AFF4/DNAI1/DNAJB6/NCOA4/TRIP13/RUVBL1/PIWIL2/SPINT2/TUBG1/PPP1CC/DZIP1/EIF2B4/MAST2/LYZL6/TESK2/SMC1B/TDRKH/WNT3/FZR1/ASB1/NUDT1/TESC/PRSS21/SPACA3/CSNK2A2/ELL3/ACTL7A/TSSK2/STAG3/RAD51/FKBP6/CKS2/AKAP4/SFMBT1/MYCBP/TCP11/AURKC/SRPK1/KLHL10/SETX/CCDC42/ACRBP/SPATA6/HSPA2/CCIN/IQCF1/CDC25C/MYBL1/TDRD7/SPA17/DAZAP1/HSPA1L/CCNB2/ZPBP2/ROPN1L/FOXJ1/LZTFL1/TCFL5/DDX20/STRBP/CATSPER2/RPL39L/PAFAH1B1/PTTG1/MYCBPAP/RFX2/SPAG8/PTTG2/TTC21A/TBPL1
GOBP_CELL_ADHESION	GOBP_CELL_ADHESION	GOBP_CELL_ADHESION	314	0.257048878	2.672286797	8.46E-10	2.66E-07	2.01E-07	1263	tags=56%, list=40%, signal=37%	SHC1/FBN1/TYK2/MSN/LAG3/PCDHGA8/PARVA/EFNB1/CDH11/DAB2/PYCARD/IGF2/CEBPB/TFE3/XBP1/HLA-DMB/LGALS3BP/HMGB1/ABL1/EMP2/TGFBI/HAVCR2/CHRD/AIF1/HLA-DRB3/CYP1B1/RAB1A/GLI2/HLA-DQB1/RGMB/PPP3CA/ABI3BP/ST3GAL4/CLIC1/PERP/FOLR2/LAMC3/COL3A1/TRIP6/PLXNA1/RAC1/ITGB1BP1/CD34/HLA-E/RSU1/COL6A3/ILK/STAB1/HLA-DRB5/PTPRC/TRO/FXYD5/GNE/ITGB1/HLA-DMA/HLA-A/COL4A6/FLOT2/BMP4/SRPX/ALOX5/MYOC/CLDN15/TRIOBP/CITED2/CBFB/MAGI1/SNAI2/CCL21/PARVG/OMG/IL15/PCDHB16/ITGA9/TSC1/CD177/ADAM15/ITGB7/CDK5/IRAK1/VWF/MYADM/ADAM12/TOR1A/BMP2/TEK/ROBO3/FGL2/COL15A1/PCDHB14/HLA-DOB/EDA/CCL5/SIGLEC12/BCL2/BTN2A2/PODXL/HAS2/NRP2/AMIGO2/ARHGEF7/PHLDB2/PTPN2/MMP14/FZD4/SVEP1/LYN/SCGB1A1/PLAU/RELA/CLDN5/F2RL3/AOC3/FAM107A/CXCL13/CORO2B/LSAMP/ITGA3/CDH3/PPP2R1A/ITGA5/APOD/LRG1/ENG/CD276/PCDHB4/NFA

											SC/SIGLEC7/HSPB1/AGER/SLURP1/CXCR3/SERPINE2/PKP2/NRP1/CLASP2/BAD/CCR1/CNTNAP1/INPPL1/CD4/SPN/LRFN3/ITGAM/MMRN2/CD83/CDH4/MINK1/SLITRK2/ITGB8/PRKAR1A/ROCK2/TNFSF13B/AGR2/S100B/PAG1/CASK/CLDN4/PARVB/SOCS1/CDH9/TNFRSF21/SMAD7/ETS1/ADIPOQ/SERPINB8/CGREF1/ACTN2/TNXB/CX3CR1/ZNF703/CSK/PSG2/DLC1/ADA/CTTN
GOBP_SPERMATID_DIFFERENTIATION	GOBP_SPERMATID_DIFFERENTIATION	GOBP_SPERMATID_DIFFERENTIATION	41	-0.534760937	-3.549465241	1.59E-09	4.24E-07	3.21E-07	799	tags=71%, list=25%, signal=54%	MKKS/BBS4/CIB1/CHD5/IQCG/PACRG/TNP2/AFF4/TRIP13/DZIP1/MAST2/ACTL7A/TSSK2/AKAP4/TCP11/SRPK1/KLHL10/CCDC42/ACRBP/HSPA2/IQCF1/ZPBP2/ROPN1L/STRBP/CATSPER2/PAFAH1B1/RFX2/TTC21A/TBPL1
GOCC_LYSOSOMAL_LUMEN	GOCC_LYSOSOMAL_LUMEN	GOCC_LYSOSOMAL_LUMEN	16	0.756590222	3.15847205	3.12E-09	7.91E-07	5.99E-07	685	tags=94%, list=22%, signal=74%	CTSK/TXNDC5/SGSH/PDGFRB/SCARB2/OGN/GLB1/MAN2B2/LIPA/MANBA/PRELP/PPT2/LUM/SDC2/GALC
GOCC_COLLAGEN_CONTAINING_EXTRACELLULAR_MATRIX	GOCC_COLLAGEN_CONTAINING_EXTRACELLULAR_MATRIX	GOCC_COLLAGEN_CONTAINING_EXTRACELLULAR_MATRIX	85	0.3916754	2.979530291	3.41E-09	8.19E-07	6.21E-07	1202	tags=69%, list=38%, signal=44%	FBN1/PODN/HTRA1/MGP/COL4A2/LGALS3BP/TGFBI/OGN/ANGPTL2/ABI3BP/PLSCR1/LAMC3/COL3A1/COL4A5/C1QC/LOXL2/COL6A3/PCOLCE/S100A4/ADAMTS5/ANXA7/SULF1/COL4A6/ANXA6/PRELP/NAV2/ANXA4/MUC2/LUM/SRPX/MYOC/MMP23B/VWF/FGL2/COL15A1/ITIH5/SDC2/CTSH/ANGPTL4/ELN/CTSC/COL4A1/LTBP1/COL27A1/IMPG2/SERPINA1/SERPINE2/SPN/CLEC14A/MMRN2/PZP/CASK/CPA3/EMILIN3/CBLN4/WNT2B/ADIPOQ/SERPINB8/TNXB
GOBP_MICROTUBULE_BASED_PROCESS	GOBP_MICROTUBULE_BASED_PROCESS	GOBP_MICROTUBULE_BASED_PROCESS	174	-0.279616169	-2.853370092	3.84E-09	8.79E-07	6.67E-07	777	tags=48%, list=25%, signal=38%	SPAST/WDR73/NUSAP1/MKKS/FUZ/CDK5R1/BBS4/BCCIP/SLK/CIB1/SIRT1/GSK3B/ZW10/KIAA0753/ARL2/HOOK2/TUBB/CDK5RAP2/KIF22/KIF4A/ATXN3/IQCG/KIFAP3/CNTN2/IFT20/MARK1/SMC3/RBM14/TUBG2/FKBP4/CEP350/ESPL1/DYNC1LI1/KPNB1/CEP63/CENPH/TPX2/DNAI1/SSNA1/MAP6D1/KIFC3/CEP250/NDE1/IFT74/KIF23/SPATA7/TUBG1/DZIP1/CENPE/KIF3A/CLUAP1/CEP72/KATNB1/SUGT1/EFHC1/DNAH17/PRK

GOCC_CILIUM	GOCC_CILIUM	GOCC_CILIUM	107	-0.337561753	-3.059016084	6.11E-09	1.34E-06	1.01E-06	671	tags=45%, list=21%, signal=37%	AA1/AKAP4/TPR/DNAH8/AURKC/PARD6A/DYNLRB2/CDCA8/CCDC42/IQCF1/RANBP1/CDC14B/SPA17/ZMYND10/IFT122/KATNA1/SPAG5/CCNB2/RAE1/ROPN1L/FOXJ1/DYNLL2/LZTFL1/CATSPER2/PAFAH1B1/TTC21A/WDR62 IQCE/ARL2/NME7/IQCG/PACRG/KIFAP3/GPI/IQCB1/SPAG4/IFT20/CAPZB/STARD10/DNAI1/SSNA1/CEP250/IFT74/TULP2/SPATA7/TUBG1/DZIP1/LYZL6/KIF3A/CLUAP1/WDR54/SPACA3/EFHC1/ARFGEF2/ACTL7A/DNAH17/AKAP4/DNAH8/TCP11/DYNLRB2/SPATA6/DNALI1/SPA17/IFT122/SPAG5/C20orf85/ROPN1L/CCDC96/DYNLL2/LZTFL1/CATSPER2/PAFAH1B1/SPAG8/IQCD/TTC21A
GOBP_CELL_CYCLE_PROCESS	GOBP_CELL_CYCLE_PROCESS	GOBP_CELL_CYCLE_PROCESS	275	-0.230844819	-2.614588317	9.11E-09	1.90E-06	1.44E-06	686	tags=37%, list=22%, signal=32%	SMARCC1/DCUN1D3/TAF10/KIAA0753/TOP3A/ARL2/RAB11FIP4/WRN/AKAP8L/TUBB/E2F1/USP8/CDK5RAP2/KIF22/KIF4A/TFDP1/ECD/CDC7/ZBTB17/SMC3/RBM14/MAD2L1BP/PINX1/THOC5/TUBG2/POLE/BRD7/PRPF40A/ESPL1/CAPN3/BARD1/MAD2L2/DYNC1LI1/PCNA/KPNB1/MYH10/CEP63/CENPH/CDK7/USP37/TPX2/BLM/SMC6/PAXIP1/CEP250/ITGB3BP/TRIP13/NDE1/PIWIL2/KIF23/VRK1/PPP6C/PAF1/DONSON/INTS7/TUBG1/UBE2S/FBXO7/CENPE/KIF3A/SMC1B/TDRKH/BUB1B/FZR1/CEP72/KATNB1/SUGT1/MDC1/CSNK2A2/C1orf112/EFHC1/STAG3/RAD51/GMNN/CKS2/CENPM/TPR/CRY1/AURKC/SRPK1/PARD6A/CCNH/CDCA8/CCDC42/HSPA2/DOT1L/RANBP1/CDC14B/CDC25C/MYBL1/RAD17/SPAG5/CCNB2/RAE1/SH3GLB1/AURKAIP1/PAFAH1B1/KLF11/PTTG1/AHCTF1/PTTG2/WDR62/CPSF3
GOCC_MHC_PROTEIN_COMPLEX	GOCC_MHC_PROTEIN_COMPLEX	GOCC_MHC_PROTEIN_COMPLEX	11	0.83418205	2.996772093	1.06E-08	2.13E-06	1.61E-06	532	tags=100%, list=17%, signal=83%	HLA-DMB/HLA-DRB3/HLA-DQB1/HLA-B/HLA-E/HLA-F/HLA-DRB5/HLA-DMA/HLA-A/HLA-C/HLA-DOB
GOCC_CATALYTIC_COMPLEX	GOCC_CATALYTIC_COMPLEX	GOCC_CATALYTIC_COMPLEX	369	-0.203486255	-2.404768246	1.44E-08	2.77E-06	2.10E-06	972	tags=47%, list=31%,	APC2/ACVR2B/CASP9/PRPF6/DAD1/MCRS1/LIG3/BCKDHB/ASH2L/POLR1C/S

										signal=37%	ENP3/POP4/PPP3CB/NDUFC2/CUL2/NDUFS6/KLHDC2/NDUFA12/FBXO11/GNG4/SF3B2/PIGH/CDC16/RFC1/RMI1/ARID4B/CSNK1A1/ANKRD9/COX6A1/VCP/RANBP2/PPP4C/RCHY1/UBE2C/NHEJ1/PMPCA/CAB39/ATG12/LSM3/PIGP/CDK5R1/PPP2R2B/BCCIP/BRMS1/ERCC3/CHD5/TAF9/SETD1A/PPP2R3B/PIK3CG/RMND5A/GSK3B/NDUFB6/TAF6/GTF2E1/TP53BP1/PDHX/SMARCC1/DCUN1D3/TAF10/PRKAG2/PMPCB/DCUN1D5/RNF7/SMG7/TOP3A/RCOR3/BRD9/PSENEN/KLHL7/BCKDHA/POLR3E/TRAF2/GTF2H1/DDA1/NDUFS7/UXS1/BAZ1A/TBK1/GTF2H2/KRTCAP2/ENO3/ATP6V0A2/DERL3/PSMD8/DNTTIP1/SAP130/SHARPIN/POLE/ADRM1/BRD7/SUZ12/BARD1/MAD2L2/DYNC1LI1/PNPT1/ACTR6/PCNA/KCTD10/ANAPC10/KBTBD8/PCGF6/CACYBP/PRIM1/CLPX/UBE3A/TEX10/PSMF1/POLRMT/CDK7/FBXL2/BLM/DNAI1/SMC6/PAXIP1/PHF21A/NOXO1/SF3A1/RUVBL1/SAP30/POLA2/EXOSC8/EXOSC4/UBE2D2/UBE2D3/PAF1/RMND5B/FBXO39/UBE2N/BRD1/ATP6V1F/PPP1CC/UBE2S/FBXO7/BUB1B/FZR1/RFC4/WDR26/POLG2/ASB1/SUGT1/FBXL18/RRAGD/DERL2/CSNK2A2/ATP6V1E2/TFIP11/TSSK2/DNAH17/PSMD12/RAD51/SAE1/PRKAA1/FBXO15/CKS2/AKAP4/TPR/DNAH8/PSMA6/COX5A/PARD6A/DYNLRB2/CCNH/APH1B/SUPT3H/FBXO24/DNALI1/C15orf48/CCNB2/DYNLL2/TSN/DR1/PAFAH1B1/TBPL1
GOCC_CHROMOSOMAL_REGION	GOCC_CHROMOSOMAL_REGION	GOCC_CHROMOSOMAL_REGION	77	-0.388187606	-3.148543699	1.53E-08	2.83E-06	2.15E-06	992	tags=69%, list=31%, signal=48%	HMBOX1/RIF1/HAT1/XPO1/NUP107/SMC4/CHEK2/NUP37/CSNK1A1/PARP1/ATR/CBX1/MCM5/RAD50/TP53BP1/ZW10/POT1/SMARCC1/WRN/KIF22/BAZ1A/SMC3/PINX1/THOC5/BRD7/DYNC1LI1/PCNA/CENPH/BLM/SMC6/ITGB3BP/NDE1/RECQL4/PPP1CC/CENPE/SMC1B/BUB1B/SUGT1/C1orf112/TFIP11/STAG3/

											RAD51/CENPM/TPR/AURKC/SETX/CDCA8/RAD17/SPAG5/SUV39H2/SMCHD1/PAFAH1B1/AHCTF1
GOCC_MOTILE_CILIUM	GOCC_MOTILE_CILIUM	GOCC_MOTILE_CILIUM	35	-0.537982504	-3.373421939	2.43E-08	4.17E-06	3.16E-06	598	tags=66%, list=19%, signal=54%	IQCG/PACRG/SPAG4/IFT20/CAPZB/STARD10/DNAI1/SSNA1/IFT74/LYZL6/SPACA3/ACTL7A/DNAH17/AKAP4/DNAH8/TCP11/SPATA6/DNALI1/SPA17/ROPN1L/CATSPER2/PAFAH1B1/IQCD
GOMF_SIGNALING_RECEPTOR_BINDING	GOMF_SIGNALING_RECEPTOR_BINDING	GOMF_SIGNALING_RECEPTOR_BINDING	277	0.254220546	2.566305145	2.41E-08	4.17E-06	3.16E-06	1415	tags=60%, list=45%, signal=36%	SHC1/FBN1/TYK2/MSN/LAG3/EFNB1/TNFSF10/RALA/FGFR1/PDGFRB/SLA/GALNT11/DAB2/IGFBP4/PYCARD/IGF2/HMGB1/TYROBP/ABL1/EMP2/RABEP2/TGFBI/SMO/OGN/VEGFB/GPRASP2/TRAF1/ANGPTL2/PLSCR1/SOCS2/COL3A1/TRIP6/STAT3/ITGB1BP1/HLA-B/HLA-E/INHBA/HLA-F/PTPRC/S100A4/FKBP1A/ADAMTS5/ITGB1/BTNL2/ANXA7/HLA-A/IGFBP6/HLA-C/TRAK2/TGFA/BMP4/PTN/TGFBRAP1/MYOC/CCL21/WIPI1/IL15/ITGA9/CD177/ADAM15/ITGB7/CDK5/VWF/PRMT2/GHRH/PALM/BMP2/EDA/CCL5/GMFG/ISG15/DERL1/BTN2A2/CPNE3/LYNX1/SRI/ARF4/PTPN2/MMP14/FNTA/FLT3LG/AP2M1/LYN/TRAF5/GNA12/CXCL13/BTN1A1/ITGA3/ITGA5/APLN/GNAI1/FGF1/GFRA3/NTF3/LRG1/ENG/FAM3C/FRS2/ANKRA2/CD276/IL17A/ADM/WNT11/ERN1/SLURP1/SERPINE2/SCP2/SQSTM1/MDM2/NR1H2/UCN3/STUB1/INS/CD4/DVL2/ITGAM/PTCH2/FGF19/GUCA2A/ITGB8/FGF13/TSPAN8/TNFSF13B/AGR2/S100B/TAP1/CASK/ARRB2/NFATC4/SOCS1/SNX17/CCL8/DDX54/WNT2B/SMAD7/ADIPOQ/DIAPH1/ACTN2/TNXB/CX3CR1/AQP1/TSHB/REEP2/UNC93B1/CLSTN3/LTA/DNAJC14/TIMP1/CCL22/ANG/THY1/SSTR3/SELPLG/CSHL1/PDGFRA/NOTCH4/S100A12/TAP2/DLG4/AAK1/ZBTB16/SH2B2/C3/WNT3A/LTB/S100A14/F11R
HP_DECREASED_FERTILITY_IN_M	HP_DECREASED_FERTILITY_IN_M	HP_DECREASED_FERTILITY_IN_MALES	29	-0.568226107	-3.286157026	2.73E-08	4.52E-06	3.43E-06	459	tags=62%, list=15%,	MAD2L2/BLM/DNAI1/IFT74/DZIP1/UBE2T/STAG3/DNAH17/RAD51/FKBP6/D

ALES	ALES									signal=54%	NAH8/AURKC/KLHL10/DNALI1/ZMYND10/FOXJ1/CATSPER2/TTC21A
GOBP_MICROTUBULE_CYTOSKELETON_ORGANIZATION	GOBP_MICROTUBULE_CYTOSKELETON_ORGANIZATION	GOBP_MICROTUBULE_CYTOSKELETON_ORGANIZATION	134	-0.291699894	-2.838821961	3.70E-08	5.64E-06	4.28E-06	776	tags=50%, list=25%, signal=39%	VCP/SPAST/WDR73/NUSAP1/CDK5R1/BBS4/BCCIP/SLK/CIB1/SIRT1/GSK3B/ZW10/KIAA0753/ARL2/HOOK2/TUBB/CDK5RAP2/KIF4A/ATXN3/IQCG/CNTN2/MARK1/SMC3/RBM14/TUBG2/FKBP4/CEP350/ESPL1/DYNC1LI1/KPNB1/CEP63/CENPH/TPX2/DNAI1/SSNA1/MAP6D1/CEP250/NDE1/KIF23/SPATA7/TUBG1/DZIP1/CENPE/KIF3A/CLUAP1/CEP72/KATNB1/SUGT1/EFHC1/DNAH17/PRKAA1/TPR/DNAH8/AURKC/PARD6A/CDCA8/CCDC42/RANBP1/CDC14B/ZMYND10/KATNA1/SPAG5/CCNB2/RAE1/FOXJ1/PAFAH1B1/WDR62
GOBP_POSITIVE_REGULATION_OF_CELL_ADHESION	GOBP_POSITIVE_REGULATION_OF_CELL_ADHESION	GOBP_POSITIVE_REGULATION_OF_CELL_ADHESION	106	0.349556457	2.810888751	3.61E-08	5.64E-06	4.28E-06	625	tags=45%, list=20%, signal=38%	TYK2/EFNB1/DAB2/PYCARD/IGF2/TFE3/XBP1/HLA-DMB/HMGB1/ABL1/EMP2/HAVCR2/CHRD/AIF1/HLA-DRB3/GLI2/HLA-DQB1/PPP3CA/ABI3BP/ST3GAL4/RAC1/ITGB1BP1/HLA-E/RSU1/ILK/HLA-DRB5/PTPRC/HLA-DMA/HLA-A/FLOT2/ALOX5/MYOC/TRIOBP/CITED2/CBFB/MAGI1/CCL21/IL15/TSC1/IRAK1/MYADM/TEK/HLA-DOB/CCL5/BTN2A2/PODXL/HAS2/ARHGEF7
GOBP_REGULATION_OF_CELL_ADHESION	GOBP_REGULATION_OF_CELL_ADHESION	GOBP_REGULATION_OF_CELL_ADHESION	171	0.2880499	2.65464638	3.75E-08	5.64E-06	4.28E-06	1171	tags=58%, list=37%, signal=38%	TYK2/LAG3/EFNB1/DAB2/PYCARD/IGF2/CEBPB/TFE3/XBP1/HLA-DMB/HMGB1/ABL1/EMP2/TGFBI/HAVCR2/CHRD/AIF1/HLA-DRB3/CYP1B1/GLI2/HLA-DQB1/PPP3CA/ABI3BP/ST3GAL4/PLXNA1/RAC1/ITGB1BP1/HLA-E/RSU1/ILK/HLA-DRB5/PTPRC/FXYD5/HLA-DMA/HLA-A/FLOT2/BMP4/ALOX5/MYOC/TRIOBP/CITED2/CBFB/MAGI1/SNAI2/CCL21/IL15/TSC1/ADAM15/IRAK1/MYADM/BMP2/TEK/FGL2/HLA-DOB/CCL5/BCL2/BTN2A2/PODXL/HAS2/ARHGEF7/PHLDB2/PTPN2/MMP14/FZD4/LYN/SCGB1A1/PLAU/RELA/FAM107A/CXCL13/CORO2B/ITGA3/PPP2R1A/ITGA5/APOD/CD276/AGER/CXCR3/SERPINE2/PKP2/

GOBP_RESPONSE_TO_ENDOGENOUS_STIMULUS	GOBP_RESPONSE_TO_ENDOGENOUS_STIMULUS	GOBP_RESPONSE_TO_ENDOGENOUS_STIMULUS	352	0.23056632	2.440431191	4.49E-08	6.55E-06	4.97E-06	1375	tags=57%, list=44%, signal=36%	NRP1/CLASP2/BAD/CD4/SPN/LRFN3/CD83/MINK1/PRKAR1A/ROCK2/TNFSF13B/AGR2/PAG1/CASK/SOCS1/TNFRSF21/SMAD7/ETS1/ADIPOQ SHC1/FBN1/NFKB1/TYK2/CTSK/CASP4/KLF9/AKR1C1/NFIA/TNFSF10/TFPI/FGFR1/PDGFRB/PHB2/DAB2/MAOB/HTRA1/RGS10/IGF2/CEBPB/XBP1/IQGAP1/COL4A2/ZMPSTE24/ABL1/HCLS1/VEGFB/CHRD/CYP1B1/ITPR1/GLB1/RGMB/CAT/PPP3CA/SOCS2/BTG2/TSPO/ATP1A2/FOLR2/COL3A1/STAT3/ITGB1BP1/INHBA/ILK/MYC/PTPRC/NFE2L2/FKBP1A/ITGB1/CYFIP1/SULF1/COL4A6/HIPK2/BMP4/PPP2R5B/NQO1/TGFBRAP1/CITED2/EHD4/SNAI2/CCL21/FECH/TSC1/MAS1L/CDK5/BCL2L1/PRMT2/RAMP2/PALM/BMP2/TEK/DHCR24/ZBTB7A/CCL5/ALAD/FOXC1/CHRNB2/SLC34A2/CTSH/CYP11A1/BCL2L2/BCL2/HRH3/AANAT/UBE2D1/HAS2/NRP2/VPS13C/CPNE3/RAPGEF1/PAX9/PTPN2/MMP14/FZD4/FNTA/DENND4C/LYN/GPR83/TRPM2/SCGB1A1/EIF2B1/FCER1G/RELA/COL4A1/CLDN5/BCHE/FAM107A/NR3C1/CXCL13/LTBP1/SIRT2/YAP1/CDKN1C/ITGA3/ITGA5/APLN/GNAI1/FGF1/NTF3/EMD/LRG1/ENG/FRS2/FOSL2/ADM/TRIM41/HSPB1/ERN1/NCOA3/GCLC/AGER/GPR21/LTA4H/LMO2/ARPC1B/MDM2/KLF2/NRP1/CRHR2/NR1H2/UCN3/MAP2K3/INPPL1/RRAGA/SLC26A5/STUB1/INS/ACSL1/OTOP1/PDK2/MMRN2/GRB7/FGF19/ADIPOR2/ITGB8/PRKAR1A/ROCK2/FSHR/SMC1A/GNB1/MYOG/VPS11/FAM20C/CASK/ARRB2/NPAS4/CLDN4/SOCS1/DDX54/SMAD7/PITX3/ADIPOQ/DIAPH1/UGCG/ACTN2/ATF2/TNXB/GHSR/CX3CR1/ZNF703/UBE2L3/AQP1/EGR1/ASCL1/TSHB/ADIPOR1/CSK/HTR1B/TOP2B/UMODL1/ELK1/TIMP1/VPS18/MEN1/TRPV1/ANG/HCN4/SSTR3/CSH

											L1/PDGFRA/AKT1/CCBE1
GOBP_DNA_DAMAGE_RESPONSE	GOBP_DNA_DAMAGE_RESPONSE	GOBP_DNA_DAMAGE_RESPONSE	190	-0.253398579	-2.670745126	5.70E-08	7.84E-06	5.94E-06	868	tags=48%, list=28%, signal=37%	RINT1/RFC1/RMI1/VCP/PPP4C/RCHY1/ENDOG/NHEJ1/POLI/PARP1/ATR/BCCIP/CIB1/SIRT1/CEBPG/CBX1/ERCC3/TIMELESS/TAF9/SETD1A/MCM5/RAD50/TAF6/TP53BP1/TRAF6/FANCC/POT1/SMARCC1/TAF10/HIPK1/DCUN1D5/TOP3A/USP10/WRN/E2F1/KIF22/ATXN3/GTF2H1/CDC7/GTF2H2/CCDC117/TEX264/SMC3/THOC5/POLE/BRD7/TRIM28/BARD1/MAD2L2/WDR33/PCNA/MAPK3/CHD1L/CEP63/CDK7/BLM/RNF138/SMC6/PAXIP1/TRIP13/RUVBL1/USP1/RECQL4/UBE2D3/VRK1/DONSON/MAPKAPK2/CHAF1B/INTS7/UBE2N/GNL1/FZR1/RFC4/MDC1/NUDT1/UBE2T/TRAIP/C1orf112/ELL3/TFIP11/POLB/RAD51/CRY1/SETX/DOT1L/SUPT3H/CDC14B/RAD17/SMCHD1/PTTG1/MDM4
GOCC_VACUOLE	GOCC_VACUOLE	GOCC_VACUOLE	185	0.285739087	2.667468271	5.62E-08	7.84E-06	5.94E-06	902	tags=48%, list=29%, signal=36%	FUCA2/CCDC115/CTSK/TXNDC5/WDR81/UBQLN2/SGSH/CYBRD1/PDGFRB/IFITM3/FYCO1/SCARB2/TM9SF1/DAB2/PYCARD/TFE3/HLA-DMB/IFITM1/CAP1/OGN/BLOC1S1/HLA-DRB3/GLB1/HLA-DQB1/MAN2B2/LIPA/TMEM25/CD34/HLA-F/PYGB/HLA-DRB5/MANBA/TPCN1/HPS6/C3AR1/VAPA/HLA-DMA/TFEB/ATG16L2/ANXA6/PRELP/PPT2/HPS1/LUM/SRPX/PTGES2/GDI2/WIPI1/TSC1/MFSD1/SIDT1/CLN3/SBF2/CTBS/RAMP2/HLA-DOB/SDC2/SLC11A1/SLC2A13/CTSH/CTSO/ABHD6/MEFV/HAS2/VPS13C/CPNE3/CTSC/AP2M1/UVRAG/DNAJC5/LYN/GALC/TRPM2/TRAPPC1/DNAJC13/BBC3/SLC29A3/OCIAD2/GNAI1/STOM/YWHAB/RAB9A/OSTM1/SLC7A14/CHMP2B/TMEM63A/SQSTM1/TBC1D17
GOCC_MICROTUBULE_ORGANIZING_CENTER	GOCC_MICROTUBULE_ORGANIZING_CENTER	GOCC_MICROTUBULE_ORGANIZING_CENTER	175	-0.265092844	-2.714628185	6.21E-08	8.30E-06	6.29E-06	849	tags=47%, list=27%, signal=36%	FLII/CSNK1A1/PPP4C/SPAST/TBC1D7/MKKS/CTNNBL1/SFI1/RABGAP1/BBS4/BCCIP/CIB1/GSK3B/RAB28/HIPK1/KIAA0753/ARL2/RAB11FIP4/WRN/HOOK2/E2F1/CDK5RAP2/NME7/RASSF1/SLC1

											A4/KIFAP3/NUP93/RASSF7/IQCB1/IFT20/TUBG2/CEP350/ESPL1/DYNC1LI1/PCNA/VPS37A/CEP63/DNAI1/SSNA1/KIFC3/CEP250/NDE1/UBN1/RUVBL1/IFT74/KIF23/MAPKAPK2/SPATA7/TUBG1/DZIP1/KIF3A/BUB1B/CLUAP1/CEP72/KATNB1/CCDC77/RAB3IP/RRAGD/TBCCD1/EFHC1/CCHCR1/ARFGEF2/TSSK2/RAD51/AURKC/SPECC1/PARD6A/DYNLRB2/RANBP1/CDC14B/HMMR/ZMYND10/IFT122/KATNA1/SPAG5/CCNB2/CCDC96/DYNLL2/PAFAH1B1/IQCD/CCDC112/WDR62
GOBP_CIRCULATORY_SYSTEM_DEVELOPMENT	GOBP_CIRCULATORY_SYSTEM_DEVELOPMENT	GOBP_CIRCULATORY_SYSTEM_DEVELOPMENT	239	0.259215216	2.542269026	6.82E-08	8.87E-06	6.72E-06	1418	tags=62%, list=45%, signal=37%	SHC1/FBN1/PARVA/SH3PXD2B/PDGFRB/GALNT11/CDH11/MED12/XBP1/COL4A2/PGK1/ZMPSTE24/ABL1/EMP2/TGFBI/SMO/VEGFB/CYP1B1/GLI2/COL3A1/LIPA/STAT3/ITGB1BP1/CD34/LOXL2/INHBA/ILK/STAB1/MBD2/HEG1/GADD45A/C3AR1/NFE2L2/HAND2/FKBP1A/ADAMTS5/ITGB1/SULF1/HIPK2/TGFA/BMP4/NKX2-5/MTHFD1/ALOX5/HS6ST1/CITED2/ZFPM2/SNAI2/GJA4/TSC1/ADAM15/ADAM12/RAMP2/BMP2/TEK/COL15A1/FOXC1/CTSH/OR10J5/ANGPTL4/ELN/MEF2D/SLC31A1/HAS2/NRP2/SRI/ID2/MMP14/FZD4/SVEP1/MAML1/COL4A1/CLDN5/CXCL13/COL27A1/YAP1/GPR15/ITGA3/ITGA5/APOD/APLN/FGF1/LRG1/ENG/FRS2/EOMES/ADM/WNT11/HSPB1/ADPRHL1/MIXL1/VGLL4/CXCR3/NSDHL/MDM2/KLF2/PKP2/NRP1/MAP2K3/BAX/DVL2/CLEC14A/MMRN2/CUL7/FGF19/ADIPOR2/ERRFI1/ITGB8/PRKAR1A/ROCK2/FBXW8/ARRB2/NFATC4/MEF2B/SNX17/PDLIM3/SMAD7/ETS1/PDLIM2/ACTN2/ATF2/GHSR/CX3CR1/CRIP1/SYPL2/AQP1/LMO4/EGR1/ASCL1/DLC1/ADRA2B/EPOR/PCSK5/TBX6/RNH1/ANG/THY1/HCN4/PDGFRA/NOTCH4/AKT1/CCBE1/NPHP3/SEC24B/E2F7/C3/CAV3/WNT3A/ATG5

GOCC_CILIARY_PLASM	GOCC_CILIARY_PLASM	GOCC_CILIARY_PLASM	17	-0.686512452	-3.232639667	9.36E-08	1.14E-05	8.65E-06	626	tags=82%, list=20%, signal=66%	NME7/PACRG/KIFAP3/DNAI1/SSNA1/SPATA7/WDR54/EFHC1/ARFGEF2/DNAH17/DNAH8/DNALI1/CCDC96/SPAG8
GOBP_POSITIVE_REGULATION_OF_LOCOMOTION	GOBP_POSITIVE_REGULATION_OF_LOCOMOTION	GOBP_POSITIVE_REGULATION_OF_LOCOMOTION	108	0.343475669	2.796204799	9.49E-08	1.14E-05	8.65E-06	1412	tags=72%, list=45%, signal=41%	F10/FGFR1/PDGFRB/DAB2/PYCARD/XBP1/HMGB1/ABL1/SMO/VEGFB/AIF1/CYP1B1/PPP3CA/TRIP6/STAT3/RAC1/ITGB1BP1/PTPRC/C3AR1/NFE2L2/ITGB1/BMP4/PTN/MYOC/SNAI2/CCL21/MYADM/BMP2/TEK/CCL5/CTSH/RAB11A/BCL2/MALAT1/PODXL/HAS2/NRP2/CPNE3/ARHGEF7/RHOC/MMP14/FZD4/LYN/PLAU/FAM107A/CXCL13/ITGA3/ITGA5/FGF1/NTF3/HSPB1/AGER/MDM2/NRP1/CLASP2/CCR1/MAP2K3/SLC26A5/INS/SPN/MAZ/GRB7/DUOX2/PTP4A1/ROCK2/CLDN4/ETS1/CX3CR1/ZNF703/AQP1/EPB41L4B/THY1/PDGFRA/AKT1/CCBE1/OXSR1/GPSM3/S100A14
GOBP_BLOOD_VESSEL_MORPHOGENESIS	GOBP_BLOOD_VESSEL_MORPHOGENESIS	GOBP_BLOOD_VESSEL_MORPHOGENESIS	135	0.31376878	2.698364847	9.19E-08	1.14E-05	8.65E-06	1399	tags=67%, list=44%, signal=39%	SHC1/PARVA/PDGFRB/XBP1/COL4A2/PGK1/ABL1/EMP2/TGFBI/SMO/VEGFB/CYP1B1/COL3A1/STAT3/ITGB1BP1/CD34/LOXL2/STAB1/HEG1/GADD45A/C3AR1/NFE2L2/HAND2/ITGB1/SULF1/HIPK2/TGFA/BMP4/NKX2-5/ALOX5/HS6ST1/CITED2/ZFPM2/ADAM15/ADAM12/RAMP2/TEK/COL15A1/FOXC1/CTSH/OR10J5/ANGPTL4/SLC31A1/HAS2/NRP2/MMP14/FZD4/COL4A1/CLDN5/CXCL13/COL27A1/YAP1/GPR15/ITGA5/APOD/APLN/FGF1/LRG1/ENG/ADM/WNT11/HSPB1/CXCR3/KLF2/NRP1/BAX/CLEC14A/MMRN2/CUL7/ADIPOR2/ITGB8/ROCK2/NFATC4/SMAD7/ETS1/ATF2/GHSR/CX3CR1/AQP1/ADRA2B/TBX6/RNH1/ANG/THY1/PDGFRA/NOTCH4/AKT1/CCBE1/SEC24B/E2F7/C3
GOCC_EXTERNAL_ENCAPSULATING_STRUCTURE	GOCC_EXTERNAL_ENCAPSULATING_STRUCTURE	GOCC_EXTERNAL_ENCAPSULATING_STRUCTURE	103	0.343235292	2.750837541	9.99E-08	1.17E-05	8.88E-06	1202	tags=64%, list=38%, signal=41%	FBN1/PODN/HTRA1/MGP/COL4A2/LGALS3BP/TGFBI/OGN/ANGPTL2/CD248/ABI3BP/PLSCR1/LAMC3/COL3A1/COL4A5/C1QC/LOXL2/COL6A3/PCOLCE/S100A4/ADAMTS5/ANXA7/SULF1/COL4A6/ANXA6/PRELP/NAV2/ANXA4/MUC2/

											LUM/SRPX/MYOC/MMP23B/VWF/FGL2/COL15A1/ITIH5/SDC2/CTSH/ANGPTL4/ELN/MMP14/CTSC/PI3/COL4A1/FBN3/LTBP1/COL27A1/FGF1/IMPG2/SERPINA1/WNT11/SERPINE2/SPN/CLEC14A/MMRN2/PZP/CASK/CPA3/MMP15/EMILIN3/CBLN4/WNT2B/ADIPOQ/SERPINB8/TNXB
GOCC_INTRACELLULAR_PROTEIN_CONTAINING_COMPLEX	GOCC_INTRACELLULAR_PROTEIN_CONTAINING_COMPLEX	GOCC_INTRACELLULAR_PROTEIN_CONTAINING_COMPLEX	191	-0.247227247	-2.602804285	1.15E-07	1.31E-05	9.96E-06	749	tags=40%, list=24%, signal=33%	CNOT6/ERCC3/TAF9/RMND5A/GSK3B/TAF6/GTF2E1/DCUN1D3/TAF10/PRKAG2/DCUN1D5/RNF7/TRAPPC3/KLHL7/POLR3E/TRAF2/GTF2H1/DDA1/GTF2H2/CNOT7/DERL3/PSMD8/SHARPIN/POLE/ADRM1/BARD1/MAD2L2/KCTD10/ANAPC10/KBTBD8/PCGF6/CACYBP/PRIM1/UBE3A/PSMF1/POLRMT/CDK7/FBXL2/RUVBL1/POLA2/UBE2D2/UBE2D3/KIF23/PAF1/RMND5B/FBXO39/UBE2N/BRD1/DZIP1/UBE2S/FBXO7/WDR41/BUB1B/FZR1/WDR26/POLG2/ASB1/SUGT1/FBXL18/DERL2/CSNK2A2/TSSK2/PSMD12/RAD51/PRKAA1/FBXO15/CKS2/AKAP4/PSMA6/CCNH/SUPT3H/FBXO24/CNOT10/TSN/DR1/NCBP2/TBPL1
GOCC_SPINDLE	GOCC_SPINDLE	GOCC_SPINDLE	107	-0.314858859	-2.853280339	1.20E-07	1.34E-05	1.01E-05	700	tags=46%, list=22%, signal=37%	ZW10/DCUN1D5/RAB11FIP4/TUBB/CDK5RAP2/KIF22/KIF4A/RASSF1/KIFAP3/CDC7/CCDC117/IQCB1/SMC3/MAD2L1BP/PINX1/TUBG2/CEP350/ESPL1/MAD2L2/DYNC1LI1/KBTBD8/CEP63/TPX2/SMC6/NDE1/KIF23/TUBG1/CENPE/KIF3A/BUB1B/KATNB1/TBCCD1/C1orf112/EFHC1/POLB/TPR/AURKC/CDCA8/HSPA2/CDC14B/HMMR/KATNA1/SPAG5/RAE1/DR1/PAFAH1B1/MAPKBP1/SPAG8/WDR62
GOCC_CONDENSED_CHROMOSOME	GOCC_CONDENSED_CHROMOSOME	GOCC_CONDENSED_CHROMOSOME	59	-0.405839263	-3.044939115	1.32E-07	1.40E-05	1.06E-05	728	tags=56%, list=23%, signal=44%	RAD50/TP53BP1/ZW10/SMARCC1/KIF22/KIFAP3/SMC3/PINX1/BRD7/NOL6/DYNC1LI1/CENPH/BLM/SMC6/ITGB3BP/NDE1/TUBG1/PPP1CC/CENPE/SMC1B/BUB1B/SUGT1/C1orf112/STAG3/RAD51/FKBP6/CENPM/TPR/AURKC/HSPA2/SPAG5/PAFAH1B1/AHCTF1

GOCC_VACUOLAR_LUMEN	GOCC_VACUOLAR_LUMEN	GOCC_VACUOLAR_LUMEN	37	0.511095287	2.939938785	1.34E-07	1.40E-05	1.06E-05	707	tags=62%, list=22%, signal=49%	FUCA2/CTSK/TXNDC5/SGSH/PDGFRB/SCARB2/PYCARD/CAP1/OGN/GLB1/MAN2B2/LIPA/PYGB/MANBA/PRELP/PPT2/LUM/PTGES2/GDI2/SDC2/CTSC/GALC/TRAPPC1
GOBP_NERVOUS_SYSTEM_PROCESS	GOBP_NERVOUS_SYSTEM_PROCESS	GOBP_NERVOUS_SYSTEM_PROCESS	252	0.252835667	2.511009206	1.31E-07	1.40E-05	1.06E-05	1525	tags=65%, list=48%, signal=37%	ALDH1A3/SCARB2/CBR3/ZMPSTE24/ABL1/EIF2AK4/GSTO1/TGFBI/NOB1/VDAC1/TSHZ3/ITPR1/PPP3CA/ST3GAL4/BTG2/ATP1A2/LAMC3/TMEM25/ITGB1/OR51A7/CYFIP1/OR5V1/OR52E4/HIPK2/SLC29A1/PTN/NAV2/HPS1/LUM/TRIOBP/OTOR/SNAI2/TAS2R39/WFS1/PTGES/OR9K2/TSC1/OR13C4/CDK5/CLN3/OR10T2/OR6V1/OR8G1/CHRNB2/OR10J5/TAS2R9/OR6C4/HRH3/OR1E2/OR5F1/SEZ6/ARF4/OR52D1/FZD4/CTSC/OAT/OR8A1/OR10H4/OR4K17/OR8H2/GRIK5/CLDN5/BCHE/FAM107A/GRM1/ITGA3/CDH3/ITGA5/OR6X1/IMPG2/NTF3/PRR4/AGER/ATP8B1/SLURP1/CRYGA/SERPINE2/OR8D1/CHMP2B/OR52B6/TAS2R40/TAS2R10/CNTNAP1/OR4K14/CLIC5/SLC26A5/INS/OR7G2/OR6Y1/OR3A3/GABRR1/TAS2R41/LCTL/OR5AS1/OR5D18/OR4S1/OR2T4/OR7D2/FGF13/ROCK2/S100B/OR6M1/OR6K2/GNB1/OR2A12/TTC8/ARRB2/OR8B8/NPAS4/OR8S1/NFATC4/MRGPRX2/TNFRSF21/OR6B2/DIAPH1/PJA2/GHSR/CX3CR1/RGS14/SLC6A1/OR51S1/GRK1/AQP1/ASCL1/OR2J2/OR1D4/OR51L1/PDCL/OR52M1/REEP2/OR13C8/TAS2R60/TPPP/OR4D5/OR1D2/OR4M1/OR1F1/CHRNA2/TAAR5/TRPV1/FXN/P2RX2/ATXN1/NTAN1/OR4D1/AKT1/DLG4/ZNF488/PBX3/CAV3/NPTX2/EML2/OR10A4/THRA/OR10H3/OR4P4/GLP1R/OR2B11/KCNQ3/BCR/GJC1/LMX1A/SEMA5B/CLN8/B3GNT2
GOBP_POSITIVE_REGULATION_OF_SIGNALING	GOBP_POSITIVE_REGULATION_OF_SIGNALING	GOBP_POSITIVE_REGULATION_OF_SIGNALING	335	0.227901423	2.400347651	1.37E-07	1.40E-05	1.06E-05	1462	tags=60%, list=46%, signal=36%	SHC1/NFKB1/TYK2/CTSK/CASP4/F10/TNFSF10/RALA/FGFR1/PDGFRB/PHB2/LSM14A/DAB2/IGFBP4/PYCARD/IGF2/XBP1/IQGAP1/HMGB1/TYROBP/TSPAN5

											/ABL1/EMP2/EIF2AK4/SMO/HAVCR2/HCLS1/H19/CYP1B1/EIF2AK2/TSHZ3/ITPR1/TRIM8/NADK/PPP3CA/COL3A1/TRIP6/LIPA/STAT3/RAC1/ITGB1BP1/PUM1/INHBA/ILK/MBD2/MYC/GADD45A/PTPRC/ARHGEF3/LMCD1/HAND2/LTBR/S100A4/FKBP1A/ITGB1/VAPA/FBXW11/MID1/CYFIP1/SULF1/ING4/IGFBP6/HIPK2/MAP3K5/TGFA/BMP4/PTN/PPP2R5B/PLEKHA4/SRPX/NMI/MYOC/CITED2/PRKCH/CCL21/RNF185/CYLD/IRAK1/VWF/GHRH/BMP2/TEK/EDA/CCL5/CTSH/RPH3AL/LY86/SRI/MIER1/RHOC/PTPN2/CTSC/LYN/SYT12/UBE2B/KMO/BRCC3/RELA/BBC3/F2RL3/TRAF5/FAM107A/TTC23/OCIAD2/LFNG/YAP1/GRM1/CDKN1C/CDH3/PPP2R1A/ITGA5/SLC35B2/FGF1/NTF3/LRG1/ENG/FRS2/TRIM41/WNT11/ERN1/AGER/MAGED1/NEK6/NET1/SERPINE2/SQSTM1/KLF2/RPS15/NRP1/CRHR2/BAD/CCR1/OSBP/UCN3/MAP2K3/RRAGA/INS/CD4/BAX/TM7SF3/DVL2/MAZ/AVPI1/MINK1/FGF19/TM2D3/RWDD3/PRKAR1A/ROCK2/DDX3X/FSHR/AGR2/S100B/GPR3/TNKS2/ARRB2/NFATC4/FAM53B/CCL8/PSMD9/IQGAP3/ADIPOQ/TRIM44/PJA2/TNXB/GHSR/CX3CR1/RGS14/ASCL1/PDCL/ADIPOR1/ADRA2B/ADA/CLSTN3/OCIAD1/MFHAS1/MEN1/FFAR2/CCL22/P2RX2/CSHL1/PDGFRA/AKT1/S100A12/AUTS2/CCBE1/DLG4/AAK1/C3/MT3/WNT3A/LTB/F11R/CA2/LTF/FIS1/PRRX1/CC2D1A/PTPN23/PLA2G1B
GOBP_VASCULATURE_DEVELOPMENT	GOBP_VASCULATURE_DEVELOPMENT	GOBP_VASCULATURE_DEVELOPMENT	154	0.298998651	2.677833155	1.54E-07	1.54E-05	1.17E-05	1399	tags=66%, list=44%, signal=38%	SHC1/PARVA/PDGFRB/XBP1/COL4A2/PGK1/ABL1/EMP2/TGFBI/SMO/VEGFB/CYP1B1/COL3A1/LIPA/STAT3/ITGB1BP1/CD34/LOXL2/STAB1/HEG1/GADD45A/C3AR1/NFE2L2/HAND2/ITGB1/SULF1/HIPK2/TGFA/BMP4/NKX2-5/ALOX5/HS6ST1/CITED2/ZFPM2/GJA4/ADAM15/ADAM12/RAMP2/BMP2/TEK/COL15A1

											/FOXC1/CTSH/OR10J5/ANGPTL4/SLC31A1/HAS2/NRP2/MMP14/FZD4/SVEP1/COL4A1/CLDN5/CXCL13/COL27A1/YAP1/GPR15/ITGA5/APOD/APLN/FGF1/LRG1/ENG/ADM/WNT11/HSPB1/CXCR3/NSDHL/MDM2/KLF2/NRP1/BAX/CLEC14A/MMRN2/CUL7/ADIPOR2/ERRFI1/ITGB8/ROCK2/FBXW8/NFATC4/SNX17/SMAD7/ETS1/ATF2/GHSR/CX3CR1/AQP1/EGR1/ADRA2B/TBX6/RNH1/ANG/THY1/PDGFRA/NOTCH4/AKT1/CCBE1/SEC24B/E2F7/C3
GOBP_MICROTUBULE_BASED_MOVEMENT	GOBP_MICROTUBULE_BASED_MOVEMENT	GOBP_MICROTUBULE_BASED_MOVEMENT	62	-0.386960841	-2.925779737	1.80E-07	1.77E-05	1.34E-05	620	tags=50%, list=20%, signal=41%	KIF22/KIF4A/IQCG/KIFAP3/IFT20/DYNC1LI1/KPNB1/DNAI1/SSNA1/KIFC3/NDE1/IFT74/KIF23/DZIP1/CENPE/KIF3A/CLUAP1/KATNB1/DNAH17/AKAP4/DNAH8/DYNLRB2/IQCF1/SPA17/ZMYND10/IFT122/ROPN1L/LZTFL1/CATSPER2/PAFAH1B1/TTC21A
GOCC_ACROSOMAL_VESICLE	GOCC_ACROSOMAL_VESICLE	GOCC_ACROSOMAL_VESICLE	33	-0.521294944	-3.184004179	2.37E-07	2.23E-05	1.69E-05	562	tags=61%, list=18%, signal=50%	ATP6V0A2/IFT20/TRIP11/DPEP3/IFT74/POMT1/LYZL6/SH3GL3/NUDT1/SPACA3/CSNK2A2/MORN3/ATP6V1E2/ACTL7A/TSSK2/TCP11/ACRBP/IQCF1/ZPBP2/SPAG8
GOBP_SENSORY_PERCEPTION_OF_CHEMICAL_STIMULUS	GOBP_SENSORY_PERCEPTION_OF_CHEMICAL_STIMULUS	GOBP_SENSORY_PERCEPTION_OF_CHEMICAL_STIMULUS	80	0.376983103	2.803377077	2.33E-07	2.23E-05	1.69E-05	1487	tags=79%, list=47%, signal=43%	OR51A7/OR5V1/OR52E4/NAV2/TAS2R39/OR9K2/OR13C4/OR10T2/OR6V1/OR8G1/OR10J5/TAS2R9/OR6C4/OR1E2/OR5F1/OR52D1/OR8A1/OR10H4/OR4K17/OR8H2/OR6X1/OR8D1/OR52B6/TAS2R40/TAS2R10/OR4K14/OR7G2/OR6Y1/OR3A3/TAS2R41/OR5AS1/OR5D18/OR4S1/OR2T4/OR7D2/OR6M1/OR6K2/GNB1/OR2A12/TTC8/OR8B8/OR8S1/OR6B2/OR51S1/OR2J2/OR1D4/OR51L1/OR52M1/REEP2/OR13C8/TAS2R60/OR4D5/OR1D2/OR4M1/OR1F1/TAAR5/TRPV1/P2RX2/OR4D1/OR10A4/OR10H3/OR4P4/OR2B11
GOCC_LUMENAL_SIDE_OF_ENDOPLASMIC_RETICULUM_MEMBRANE	GOCC_LUMENAL_SIDE_OF_ENDOPLASMIC_RETICULUM_MEMBRANE	GOCC_LUMENAL_SIDE_OF_ENDOPLASMIC_RETICULUM_MEMBRANE	11	0.791529362	2.843543687	2.56E-07	2.36E-05	1.79E-05	348	tags=82%, list=11%, signal=73%	HLA-DRB3/HLA-DQB1/HLA-B/HLA-E/HLA-F/HLA-DRB5/HM13/HLA-A/HLA-C

GOCC_CHROMOSOME	GOCC_CHROMOSOME	GOCC_CHROMOSOME	352	-0.196920091	-2.316321137	2.83E-07	2.56E-05	1.94E-05	771	tags=37%, list=24%, signal=31%	CREB3/HMX1/SIRT1/CEBPG/CBX1/ASF1B/BRMS1/CHD5/TIMELESS/GMEB1/TAF9/SETD1A/MCM5/RAD50/TAF6/TP53BP1/BOP1/ZW10/FANCC/POT1/SMARCC1/TAF10/ZC3H8/NFYB/TOP3A/WRN/ZGPAT/AKAP8L/E2F1/BRD9/PPHLN1/KIF22/KIF4A/PINK1/KIFAP3/BAZ1A/TFDP1/TEX264/TNP2/GRHL1/SMAD2/DNTTIP1/TOP1MT/PAWR/SMC3/PINX1/THOC5/PRRX2/SAP130/HSF2/ARPC2/POLE/BRD7/NOL6/TRIM28/SUZ12/MAD2L2/DYNC1LI1/SPOCD1/ACTR6/PCNA/CHD1L/PRIM1/SPHK2/CENPH/ZBTB32/ZHX3/EBNA1BP2/AFF4/BLM/RNF138/SMC6/PAXIP1/ITGB3BP/TRIP13/NDE1/PWP1/RUVBL1/SAP30/POLA2/EXOSC8/EXOSC4/RECQL4/DONSON/ZMYND11/CHAF1B/INTS7/TUBG1/PPP1R7/BRD1/PPP1CC/NAP1L4/CENPE/SMC1B/BUB1B/RFC4/RFX4/NR1H3/SUGT1/MDC1/CSNK2A2/STAT4/TRAIP/C1orf112/ELL3/TFIP11/STAG3/RAD51/FKBP6/PRKAA1/CENPM/TPR/AURKC/SRPK1/SETX/CDCA8/HSPA2/SUPT3H/RAD17/SPAG5/CCDC86/FOXJ1/SUV39H2/SMCHD1/TCFL5/DR1/PAFAH1B1/KLF11/AHCTF1/RFX2
GOBP_ORGANELLE_ASSEMBLY	GOBP_ORGANELLE_ASSEMBLY	GOBP_ORGANELLE_ASSEMBLY	196	-0.239491438	-2.551215459	3.37E-07	3.01E-05	2.28E-05	799	tags=43%, list=25%, signal=35%	MKKS/ATG12/LSM3/TBC1D10B/FNBP1L/FUZ/RABGAP1/BBS4/BCCIP/CNOT6/GSK3B/BOP1/TRAF6/KIAA0753/TBC1D1/USP10/TUBB/CDK5RAP2/KIF4A/PINK1/IQCG/TP53INP1/CNOT7/ACTL8/ATG9A/IQCB1/IFT20/SMC3/RBM14/CEP350/CAPN3/CSRP2/MYOM2/LRFN4/KPNB1/VPS37A/CEP63/CENPH/TPX2/DNAI1/CEP250/IFT74/EIF2S1/KIF23/STX18/NIP7/RAB8B/DZIP1/CENPE/KIF3A/CLUAP1/CEP72/WDR54/RFX4/EDC3/SUGT1/TBC1D3/RAB3IP/DHX30/ACTL7A/DNAH17/PRKAA1/AKAP4/TPR/DNAH8/EIF5/AURKC/CDCA8/CCDC42/ACRBP/SPATA6/CDC14B/NF2/ZMYND10/IFT122/SPAG5/CCNB2/ZPBP2/CCDC96/FOXJ1/SH3GLB1

											/PAFAH1B1/RFX2/WDR62/TBPL1
GOBP_LOCOMOTION	GOBP_LOCOMOTION	GOBP_LOCOMOTION	233	0.251169445	2.455620029	3.65E-07	3.13E-05	2.38E-05	1414	tags=62%, list=45%, signal=37%	MSN/PARVA/F10/ARHGDIB/RALA/PODN/FGFR1/PDGFRB/CDH11/DAB2/PYCARD/XBP1/HMGB1/ABL1/EMP2/IFITM1/SMO/VEGFB/CHRD/AIF1/CYP1B1/PLEKHG3/PPP3CA/ST3GAL4/ATP1A2/FOLR2/COL3A1/TRIP6/PLXNA1/STAT3/RAC1/ITGB1BP1/LSP1/GADD45A/PTPRC/C3AR1/NFE2L2/S100A4/ITGB1/SULF1/BMP4/PTN/ALOX5/MYOC/CITED2/SNAI2/CCL21/ITGA9/ADAM15/CDK5/MYADM/CLN3/BMP2/TEK/ROBO3/CCL5/CTSH/OR10J5/ABHD6/RAB11A/BCL2/AIRE/MALAT1/PODXL/HAS2/NRP2/CPNE3/ARHGEF7/PHLDB2/RHOC/PTPN2/MMP14/FZD4/LYN/TRPM2/PLAU/FCER1G/CLDN5/FAM107A/GNA12/CXCL13/ITGA3/ITGA5/APOD/FGF1/CCR5/NTF3/ENG/WNT11/HSPB1/AGER/SLURP1/CXCR3/SERPINE2/MDM2/NRP1/CLASP2/CCR1/MAP2K3/SLC26A5/INS/SPN/RHOG/MAZ/MMRN2/GRB7/DUOX2/MINK1/FGF19/PTP4A1/ROCK2/ARRB2/CLDN4/CCL8/SMAD7/ETS1/ADIPOQ/ABI3/TNXB/GHSR/CX3CR1/ZNF703/AQP1/LMO4/ADIPOR1/DLC1/ADA/RALBP1/EPB41L4B/OR1D2/TIMP1/XCR1/FFAR2/CCL22/THY1/PDGFRA/AKT1/S100A12/CCBE1/OXSR1/GPSM3/WNT3A/S100A14/SPATA13
GOBP_POSITIVE_REGULATION_OF_MULTICELLULAR_ORGANISMAL_PROCESS	GOBP_POSITIVE_REGULATION_OF_MULTICELLULAR_ORGANISMAL_PROCESS	GOBP_POSITIVE_REGULATION_OF_MULTICELLULAR_ORGANISMAL_PROCESS	315	0.227861827	2.361856665	3.60E-07	3.13E-05	2.38E-05	1418	tags=59%, list=45%, signal=36%	TYK2/EFNB1/DAB2/PYCARD/IGF2/CEBPB/XBP1/PQBP1/HLA-DMB/HMGB1/IFNGR1/TYROBP/ABL1/EMP2/GSTO1/SMO/HAVCR2/HCLS1/H19/VEGFB/AIF1/HLA-DRB3/CYP1B1/RAB1A/EIF2AK2/GLI2/HLA-DQB1/PPP3CA/ST3GAL4/NAP1L1/TSPO/ATP1A2/PLXNA1/STAT3/CD34/HLA-E/LOXL2/INHBA/HLA-F/HLA-DRB5/HEG1/MYC/PTPRC/CEBPA/C3AR1/NFE2L2/HAND2/ITGB1/HLA-DMA/CYFIP1/SULF1/HLA-A/HIPK2/FLOT2/BMP4/PTN/ATF4/NKX2-5/LUM/ALOX5/CITED2/PRKCH/CBFB/ZFPM2/CCL21/IL15/

											CD177/IRAK1/ADAM12/RAMP2/GHRH/BMP2/EBF2/TEK/HLA-DOB/CCL5/FOXC1/SLC11A1/CHRNB2/CTSH/ANGPTL4/ISG15/BCL2/MEFV/AIRE/PRAP1/BTN2A2/AMIGO2/ID2/PLAG1/MMP14/FZD4/CTSC/LYN/PLAU/FCER1G/RELA/PRKAB2/YAP1/KIR2DL4/ITGA5/APLN/FGF1/LRG1/ENG/FRS2/CD276/IL17A/ADM/WNT11/TMEM119/HSPB1/NCOA3/ADD3/AGER/MAGED1/SERPINE2/GPR21/KLF10/NRP1/BAD/CCR1/NR1H2/OMA1/INS/ACSL1/CD4/SPN/ITGAM/OAS2/MMRN2/CD83/EHMT1/CUL7/CDH4/SLITRK2/ADIPOR2/ITGB8/ROCK2/DDX3X/TNFSF13B/MYOG/GPR3/FAM20C/FBXW8/ARRB2/NFATC4/SOCS1/GDI1/WNT2B/SMAD7/ETS1/IQGAP3/ADIPOQ/ATF2/TNXB/GHSR/CX3CR1/RGS14/ZNF703/SGIP1/AQP1/EGR1/ASCL1/ADIPOR1/CSK/ADRA2B/ADA/CTTN/TPPP/UNC93B1/CLSTN3/LTA/FFAR2/CHIA/THY1/TNFRSF4/AKT1/CCBE1/ZNF488/ZBTB16/LRRN3/C3/GPSM3/WNT3A/LTB/ATG5
GOBP_CELLULAR_PROCESS_INVOLVED_IN_REPRODUCTION_IN_MULTICELLULAR_ORGANISM	GOBP_CELLULAR_PROCESS_INVOLVED_IN_REPRODUCTION_IN_MULTICELLULAR_ORGANISM	GOBP_CELLULAR_PROCESS_INVOLVED_IN_REPRODUCTION_IN_MULTICELLULAR_ORGANISM	84	-0.330872464	-2.759164587	4.28E-07	3.61E-05	2.74E-05	389	tags=36%, list=12%, signal=32%	AFF4/TRIP13/PIWIL2/DZIP1/MAST2/LYZL6/TDRKH/SPACA3/ACTL7A/TSSK2/AKAP4/TCP11/SRPK1/KLHL10/CCDC42/ACRBP/HSPA2/IQCF1/MYBL1/TDRD7/CCNB2/ZPBP2/ROPN1L/DDX20/STRBP/CATSPER2/PAFAH1B1/RFX2/TTC21A/TBPL1
GOBP_CELL_ACTIVATION	GOBP_CELL_ACTIVATION	GOBP_CELL_ACTIVATION	204	0.262334745	2.49690509	4.57E-07	3.79E-05	2.87E-05	1418	tags=62%, list=45%, signal=37%	TYK2/MSN/LAG3/EFNB1/PHB2/PYCARD/IGF2/CEBPB/XBP1/HLA-DMB/HMGB1/IFNGR1/TYROBP/ABL1/EMP2/EIF2AK4/SMO/HAVCR2/AIF1/HLA-DRB3/MAFB/GLI2/HLA-DQB1/PPP3CA/PLSCR1/CLIC1/SLC25A5/IMPDH2/COL3A1/LIPA/STAT3/HLA-E/INHBA/ILK/HLA-F/HLA-DRB5/PTPRC/CEBPA/LTBR/FKBP1A/ITGB1/HLA-DMA/HLA-A/FLOT2/BMP4/NMI/CBFB/CCL21/IL15/PURA/TSC1/CD177/VWF/PRDX1/ZBTB7A/FGL2/HLA-DOB/CCL5/SLC11A1/CHRNB2/KIF13B/B

											CL2/AIRE/BTN2A2/ID2/PTPN2/HSH2D/MMP14/CTSC/LYN/SCGB1A1/FCER1G/F2RL3/LFNG/FOSL2/EOMES/CD276/IL17A/HSPB1/GCLC/AGER/SLURP1/SERPINE2/BATF2/CRTC3/MDM2/BAD/INS/CD4/BAX/SPN/ITGAM/CD83/ITGB8/PRKAR1A/TNFSF13B/PAG1/CD7/MRGPRX2/SOCS1/TNFRSF21/SMAD7/PJA2/ATF2/CX3CR1/LMO4/EGR1/CSK/ADRA2B/ADA/TOP2B/MFHAS1/MEN1/TRPV1/CD79A/THY1/SUPT6H/SELPLG/TNFRSF4/PDGFRA/AKT1/S100A12/ZBTB16/ENTPD2/WNT3A/F11R/ATG5
GOMF_ATP_DEPENDENT_ACTIVITY	GOMF_ATP_DEPENDENT_ACTIVITY	GOMF_ATP_DEPENDENT_ACTIVITY	103	-0.30991554	-2.751934865	5.04E-07	4.11E-05	3.12E-05	448	tags=47%, list=14%, signal=41%	ERCC3/CHD5/MCM5/RAD50/TOR2A/WRN/DDX52/KIF22/KIF4A/ATP6V0A2/SMC3/ATP8A2/CHD1L/HSPH1/MYH10/CLPX/CDK7/BLM/KIFC3/TRIP13/RUVBL1/RECQL4/KIF23/ATP6V1F/CENPE/KIF3A/SMC1B/RFC4/ATF7IP/MYO9A/HSPA14/ATP6V1E2/DHX30/CLPB/DNAH17/RAD51/SAE1/DNAH8/DYNLRB2/DHX16/SETX/HSPA2/RAD17/HSPA1L/KATNA1/SMCHD1/DDX20/ATAD2
GOCC_CELL_SURFACE	GOCC_CELL_SURFACE	GOCC_CELL_SURFACE	172	0.270003291	2.481991841	5.61E-07	4.50E-05	3.41E-05	1617	tags=72%, list=51%, signal=37%	CTSK/ANTXR2/MSN/LAG3/F10/EFNB1/RALA/TFPI/PHB2/HMGB1/TYROBP/EMP2/HAVCR2/LAYN/CD248/FOLR2/CD34/HLA-B/HLA-E/HLA-F/HEG1/PTPRC/HM13/SCARA5/ITGB1/BTNL2/HLA-DMA/SULF1/HLA-A/MAP3K5/HLA-C/TGFA/ENTPD6/ANXA4/SRPX/IGF2R/COLEC11/ITGA9/ADAM15/ITGB7/IRAK1/KCNB1/RAMP2/BMP2/TEK/CYP2W1/SDC2/CHRNB2/BTN2A2/FZD4/FLT3LG/LYN/PLAU/FCER1G/AOC3/BTN1A1/ITGA3/ITGA5/CCR5/GFRA3/ENG/CD276/IL17A/AGER/CXCR3/CCR1/CD4/ABCB1/SPN/SLC22A11/MPZL1/LRFN3/ITGAM/DCBLD2/CLEC14A/CD1A/CD83/DUOX2/PROM2/VAMP5/ITGB8/TSPAN8/KISS1R/ADIPOQ/GHSR/CX3CR1/SLC6A1/CD1B/PSG2/ADRA2B/ADA/EPOR/UMODL1/CLSTN3/XCR1/TRPV1/CD79A/THY1/IGSF21/T

										NFRSF4/PDGFRA/NOTCH4/C3/PSG8/CAV3/WNT3A/LTF/IL9R/TSPAN32/PLA2G1B/SLC32A1/WNT1/KCNQ3/CD6/BCAM/MICA/ABCG2/IL1R1/MCAM/CD209/IL2RG/TMIGD1/CLSTN1
GOBP_MONOATOMIC_ION_TRANSPORT	GOBP_MONOATOMIC_ION_TRANSPORT	GOBP_MONOATOMIC_ION_TRANSPORT	239	0.244667611	2.399592499	5.85E-07	4.62E-05	3.50E-05	1595	tags=67%, list=51%, signal=36%	LASP1/COX7A1/CYBRD1/PDGFRB/PHB2/MAOB/ZMPSTE24/ABL1/GSTO1/COX7B/VDAC1/COX8A/ITPR1/TMCO3/PPP3CA/SLC25A23/CLIC1/CLCC1/SLC25A5/TSPO/ATP1A2/CLNS1A/PTPRC/TPCN1/SLC30A9/SCARA5/CCDC51/FXYD5/FKBP1A/ITGB1/STEAP3/ABCB7/ANXA6/SLC25A12/ATF4/NKX2-5/KCNK5/CLDN15/SLC12A9/KCNE3/CCL21/WFS1/GJA4/TSC1/CDK5/TMEM109/KCNB1/CSN2/SLC25A37/RAMP2/OTOP3/CCL5/SLC11A1/CHRNB2/SLC34A2/SLC9A8/SLC26A1/GRIA4/BCL2/SLC31A1/HRH3/SRI/IBTK/LYN/TRPM2/ATP2A3/GRIK5/F2RL3/COX15/SEC61A1/STOM/CCR5/OSTM1/SLC6A17/ATP8B1/SERPINE2/TMEM63A/PLLP/SLC22A8/PKP2/CCR1/CLIC5/CALM3/SLC26A5/PPIF/LIME1/CD4/ABCB1/BAX/OTOP1/SLC22A11/KCNH4/FHL1/GABRR1/SLC24A3/CNNM2/OTOP2/ABCC10/KCNB2/KCNJ1/NIPA2/FGF13/CABP5/CASK/CLDN4/SLC10A5/CCL8/KCNAB2/DIAPH1/SFXN3/ACTN2/ATP6V1G2/SLC6A1/AQP1/HTR1B/KCNJ6/KCNH7/PXK/CHRNA2/SLC5A7/XCR1/TRPV1/FXN/P2RX2/SLC22A7/THY1/HCN4/AKT1/OXSR1/SLC22A2/MT3/CAV3/WNT3A/CLCN6/CA2/ATG5/CDK2/CACNA2D3/LTF/HCN3/ATP13A1/PLA2G1B/SLC32A1/ATP6V0A1/CACNA1B/KCNQ3/GJC1/SLC34A3/KCNS2/CACNA1E/SLC12A8/SLC25A13/ATP1B1/SLC12A3/CACNB3/SLC17A7/ATP1A3/GRM6/ATP2A1/TRPV5/HTR3A
GOMF_OXIDOREDUCTASE_ACTIVITY	GOMF_OXIDOREDUCTASE_ACTIVITY	GOMF_OXIDOREDUCTASE_ACTIVITY	152	0.285674575	2.55129662	6.81E-07	5.28E-05	4.00E-05	958	tags=49%, list=30%, signal=36%	PLOD1/ALDH1A3/COX7A1/SUMF1/TXNDC5/AKR1A1/AKR1C1/CYBRD1/CBR1/MSRB2/MAOB/CBR3/PGK1/CTBP1/GSTO1/COX7B/RSAD1/LTC4S/CYP1B1/A

											LOX5AP/COX8A/CAT/PAM/IMPDH2/DHRSX/LOXL2/CYP2U1/STAB1/ADHFE1/ADI1/STEAP3/PDIA6/NQO1/MTHFD1/HSDL2/ALDH2/ALOX5/DHTKD1/FMO1/PTGES2/ACAA1/PIPOX/PTGES/PRDX1/CCS/DHCR24/ALKBH4/CYP2W1/IDH3B/IDH3G/CYP11A1/PPOX/PHF2/CYP17A1/CBS/ALKBH6/CYP4B1/KMO/BLVRA/AOC3/PRDX3/COX15/PDIA5/CYP2S1/LDHA/AKR7A2/RRM2B/NSDHL/ADH1C/ACOX1/AKR7A3/FADS6/HSD17B8/ACAD11/CYP3A43
HP_ABNORMAL_SPERM_MOTILITY	HP_ABNORMAL_SPERM_MOTILITY	HP_ABNORMAL_SPERM_MOTILITY	16	-0.674319024	-3.049048387	6.93E-07	5.29E-05	4.01E-05	382	tags=69%, list=12%, signal=61%	DNAI1/IFT74/DZIP1/DNAH17/DNAH8/KLHL10/DNALI1/ZMYND10/FOXJ1/CATSPER2/TTC21A
GOMF_OLFACTORY_RECEPTOR_ACTIVITY	GOMF_OLFACTORY_RECEPTOR_ACTIVITY	GOMF_OLFACTORY_RECEPTOR_ACTIVITY	60	0.409881664	2.803956273	7.06E-07	5.30E-05	4.02E-05	1487	tags=83%, list=47%, signal=45%	OR51A7/OR5V1/OR52E4/OR9K2/OR13C4/OR10T2/OR6V1/OR8G1/OR10J5/OR6C4/OR1E2/OR5F1/OR52D1/OR8A1/OR10H4/OR4K17/OR8H2/OR6X1/OR8D1/OR52B6/OR4K14/OR7G2/OR6Y1/OR3A3/OR5AS1/OR5D18/OR4S1/OR2T4/OR7D2/OR6M1/OR6K2/OR2A12/OR8B8/OR8S1/OR6B2/OR51S1/OR2J2/OR1D4/OR51L1/OR52M1/OR13C8/OR4D5/OR1D2/OR4M1/OR1F1/OR4D1/OR10A4/OR10H3/OR4P4/OR2B11
GOBP_RNA_3_END_PROCESSING	GOBP_RNA_3_END_PROCESSING	GOBP_RNA_3_END_PROCESSING	19	-0.636930163	-3.118381535	7.42E-07	5.49E-05	4.16E-05	723	tags=84%, list=23%, signal=65%	SYMPK/CPSF1/CPSF2/CSTF3/BARD1/PNPT1/WDR33/EXOSC8/EXOSC4/PAF1/INTS7/CSTF1/INTS6/ZNF473/NCBP2/CPSF3
GOCC_CENTROSOME	GOCC_CENTROSOME	GOCC_CENTROSOME	134	-0.268315091	-2.611241166	7.95E-07	5.79E-05	4.39E-05	939	tags=53%, list=30%, signal=39%	TUBGCP2/RBBP6/MAPRE1/ACTR1A/CEP290/MAP1S/CDC16/FLII/CSNK1A1/SPAST/MKKS/CTNNBL1/RABGAP1/BBS4/BCCIP/CIB1/GSK3B/HIPK1/KIAA0753/ARL2/WRN/HOOK2/E2F1/CDK5RAP2/NME7/SLC1A4/KIFAP3/NUP93/RASSF7/IQCB1/IFT20/TUBG2/CEP350/ESPL1/DYNC1LI1/PCNA/VPS37A/CEP63/DNAI1/SSNA1/KIFC3/CEP250/NDE1/UBN1/IFT74/KIF23/MAPKAPK2/TUBG1/DZIP1/KIF3A/CLUAP1/CEP72/KATNB1/CCDC77/RRAGD/TBCCD1/EFHC1/AURKC/PARD6

											A/DYNLRB2/RANBP1/CDC14B/HMMR/ZMYND10/KATNA1/SPAG5/CCNB2/DYNLL2/PAFAH1B1/CCDC112/WDR62
GOBP_TUBE_DEVELOPMENT	GOBP_TUBE_DEVELOPMENT	GOBP_TUBE_DEVELOPMENT	226	0.24483883	2.394026572	1.12E-06	8.04E-05	6.10E-05	1500	tags=63%, list=48%, signal=36%	SHC1/PARVA/NFIA/RALA/PDGFRB/PHB2/MED12/XBP1/COL4A2/PGK1/ABL1/EMP2/TGFBI/SMO/VEGFB/CHRD/CYP1B1/GLI2/CAT/COL3A1/LIPA/STAT3/ITGB1BP1/CD34/LOXL2/ILK/STAB1/HEG1/MYC/GADD45A/ITPK1/CEBPA/C3AR1/NFE2L2/HAND2/EIF4G1/ITGB1/SULF1/HIPK2/TGFA/BMP4/NKX2-5/MTHFD1/ALOX5/HS6ST1/CITED2/ZFPM2/TSC1/ADAM15/ADAM12/RAMP2/BMP2/TEK/COL15A1/EDA/FOXC1/CTSH/OR10J5/ANGPTL4/BCL2/SLC31A1/PODXL/HAS2/NRP2/ID2/MMP14/FZD4/GPR161/COL4A1/CLDN5/NR3C1/CXCL13/COL27A1/YAP1/GPR15/ITGA3/ITGA5/APOD/APLN/FGF1/LRG1/ENG/FOSL2/ADM/WNT11/HSPB1/MAGED1/MIXL1/CXCR3/SERPINE2/LTA4H/KLF2/NRP1/BAX/DVL2/CLEC14A/MMRN2/SSBP3/CUL7/ADIPOR2/ERRFI1/ITGB8/ROCK2/AGR2/TTC8/NFATC4/WNT2B/SMAD7/ETS1/ATF2/GHSR/CX3CR1/AQP1/LMO4/ASCL1/DLC1/ADRA2B/ADA/PCSK5/TBC1D20/ELK1/TBX6/RNH1/ANG/THY1/PDGFRA/NOTCH4/AKT1/CCBE1/NPHP3/SEC24B/E2F7/C3/CAV3/WNT3A/RARG/ELK3/PRRX1/THRA/PHACTR4/IRX3/WNT1/GJC1
GOBP_LYMPHOCYTE_ACTIVATION	GOBP_LYMPHOCYTE_ACTIVATION	GOBP_LYMPHOCYTE_ACTIVATION	147	0.288026881	2.544941869	1.16E-06	8.08E-05	6.12E-05	701	tags=41%, list=22%, signal=33%	TYK2/MSN/LAG3/EFNB1/PHB2/PYCARD/IGF2/CEBPB/XBP1/HLA-DMB/HMGB1/TYROBP/ABL1/EMP2/EIF2AK4/HAVCR2/AIF1/HLA-DRB3/MAFB/GLI2/HLA-DQB1/PPP3CA/SLC25A5/IMPDH2/LIPA/STAT3/HLA-E/INHBA/HLA-F/HLA-DRB5/PTPRC/FKBP1A/ITGB1/HLA-DMA/HLA-A/FLOT2/BMP4/CBFB/CCL21/IL15/PURA/TSC1/PRDX1/ZBTB7A/FGL2/HLA-DOB/CCL5/SLC11A1/CHRNB2/KIF13B/BCL2/AIRE/BTN2A2/ID2/PTPN2/HSH2D/MMP14/LYN/SCGB1A1/FCER1G

GOBP_REGULATION_OF_MULTICELLULAR_ORGANISMAL_DEVELOPMENT	GOBP_REGULATION_OF_MULTICELLULAR_ORGANISMAL_DEVELOPMENT	GOBP_REGULATION_OF_MULTICELLULAR_ORGANISMAL_DEVELOPMENT	271	0.231644185	2.32567706	1.15E-06	8.08E-05	6.12E-05	1443	tags=60%, list=46%, signal=36%	FBN1/CTSK/MAF/LAG3/CEBPB/TFE3/XBP1/MGP/COL4A2/HMGB1/PGK1/ZMPSTE24/TYROBP/ABL1/EMP2/SMO/HCLS1/VEGFB/CYP1B1/MAFB/GLI2/PPP3CA/NAP1L1/BTG2/TSPO/PLXNA1/STAT3/C1QC/CD34/HLA-B/LOXL2/INHBA/STAB1/MYC/GADD45A/PTPRC/CEBPA/C3AR1/NFE2L2/ITGB1/CYFIP1/SULF1/HIPK2/BMP4/PTN/ATF4/NKX2-5/ADD1/TFPT/ALOX5/CITED2/PRKCH/CBFB/ZFPM2/SNAI2/IL15/PIAS3/CDK5/ADAM12/RAMP2/BMP2/TEK/FGL2/FOXC1/CTSH/OR10J5/ANGPTL4/ISG15/BTN2A2/AMIGO2/ID2/PHLDB2/PLAG1/PTPN2/MMP14/FZD4/CTSC/LYN/TDG/MAFG/RELA/CLDN5/TCTA/LGI4/CXCL13/SIRT2/YAP1/CDKN1C/CDH3/ITGA5/FGF1/LRG1/ENG/IL17A/ADM/WNT11/TMEM119/HSPB1/NCOA3/AGER/VGLL4/SERPINE2/KLF10/KLF2/NRP1/CLASP2/BAD/CCR1/CD4/OAS2/PTCH2/MMRN2/CD83/EHMT1/CUL7/CDH4/ULK1/SLITRK2/PRMT6/ERRFI1/ITGB8/FGF13/ROCK2/FAM20C/FBXW8/NOTUM/NFATC4/SOCS1/TNFRSF21/GDI1/WNT2B/SMAD7/ETS1/PITX3/ADIPOQ/ATF2/GHSR/CX3CR1/RGS14/AQP1/ASCL1/ADA/TPPP/CLSTN3/LTA/RNH1/THY1/NOTCH4/AKT1/CCBE1/NPHP3/ZNF488/ZBTB16/LRRN3/C3/MT3/CAV3/WNT3A/F11R/RARG/LTF/DAB1/FOXE3
GOBP_GERM_CELL_DEVELOPMENT	GOBP_GERM_CELL_DEVELOPMENT	GOBP_GERM_CELL_DEVELOPMENT	67	-0.363766193	-2.815515366	1.20E-06	8.24E-05	6.25E-05	799	tags=54%, list=25%, signal=41%	MKKS/BBS4/CIB1/CHD5/FANCC/PAQR7/IQCG/PACRG/INHBB/TNP2/TIAL1/AFF4/TRIP13/PIWIL2/DZIP1/TDRKH/ACTL7A/TSSK2/AKAP4/TCP11/SRPK1/KLHL10/CCDC42/ACRBP/HSPA2/IQCF1/TDRD7/ZPBP2/ROPN1L/DDX20/STRBP/CATSPER2/PAFAH1B1/RFX2/TTC21A/TBPL1
GOCC_NUCLEAR_CHROMOSOME	GOCC_NUCLEAR_CHROMOSOME	GOCC_NUCLEAR_CHROMOSOME	44	-0.431814277	-2.978699921	1.38E-06	9.32E-05	7.06E-05	750	tags=61%, list=24%, signal=47%	PARP1/BRMS1/TIMELESS/MCM5/RAD50/KIFAP3/BAZ1A/SMC3/PINX1/SAP130/POLE/NOL6/ACTR6/PCNA/PRIM1/BL

											M/RUVBL1/SAP30/POLA2/TUBG1/SMC1B/STAG3/RAD51/FKBP6/SETX/HSPA2/SMCHD1
GOBP_MRNA_TRANSPORT	GOBP_MRNA_TRANSPORT	GOBP_MRNA_TRANSPORT	26	-0.536603959	-2.999107588	1.42E-06	9.49E-05	7.19E-05	1236	tags=92%, list=39%, signal=57%	NXF1/RBM15B/MCM3AP/AAAS/NUTF2/KHSRP/XPO1/NUP107/IGF2BP1/NUP37/PABPN1/RANBP2/IWS1/SMG7/AKAP8L/NUP93/THOC5/PARP11/TPR/NUP88/RAE1/NUP155/AHCTF1/NCBP2
GOBP_MRNA_METABOLIC_PROCESS	GOBP_MRNA_METABOLIC_PROCESS	GOBP_MRNA_METABOLIC_PROCESS	137	-0.261387346	-2.558857906	1.66E-06	0.000109172	8.28E-05	792	tags=44%, list=25%, signal=34%	LSM3/CTNNBL1/SAFB/CEBPG/CNOT6/TAF9/SYMPK/TAF6/FASTKD1/TAF10/IWS1/SMG7/CPSF1/AKAP8L/E2F1/PAIP1/CPEB1/TRAF2/ECD/CPSF2/CNOT7/CSTF3/RBM14/ELAVL4/THOC5/PRPF40A/BOLL/EDC4/BARD1/PNPT1/WDR33/DUS3L/SF3A1/GEMIN4/EXOSC8/EXOSC4/GEMIN6/PRPF18/PAF1/MAPKAPK2/CSTF1/PRPF40B/EDC3/NR1H3/SECISBP2/ZRANB2/TFIP11/SRPK1/DHX16/SETX/PRPF38A/ZNF473/BUD13/DAZAP1/CNOT10/DDX20/KHDRBS3/NCBP2/PRKRIP1/CPSF3
GOBP_POSITIVE_REGULATION_OF_LEUKOCYTE_CELL_CELL_ADHESION	GOBP_POSITIVE_REGULATION_OF_LEUKOCYTE_CELL_CELL_ADHESION	GOBP_POSITIVE_REGULATION_OF_LEUKOCYTE_CELL_CELL_ADHESION	63	0.382249514	2.665962463	1.80E-06	0.000115109	8.73E-05	711	tags=51%, list=23%, signal=40%	TYK2/EFNB1/PYCARD/IGF2/XBP1/HLA-DMB/HMGB1/ABL1/HAVCR2/AIF1/HLA-DRB3/GLI2/HLA-DQB1/PPP3CA/ST3GAL4/HLA-E/HLA-DRB5/PTPRC/HLA-DMA/HLA-A/FLOT2/ALOX5/CBFB/CCL21/IL15/IRAK1/HLA-DOB/CCL5/BTN2A2/HAS2/LYN/RELA
GOBP_POSITIVE_REGULATION_OF_RESPONSE_TO_EXTERNAL_STIMULUS	GOBP_POSITIVE_REGULATION_OF_RESPONSE_TO_EXTERNAL_STIMULUS	GOBP_POSITIVE_REGULATION_OF_RESPONSE_TO_EXTERNAL_STIMULUS	99	0.331387487	2.619072571	1.79E-06	0.000115109	8.73E-05	1427	tags=72%, list=45%, signal=41%	CASP4/LAG3/FGFR1/PDGFRB/PHB2/LSM14A/PYCARD/CEBPB/PQBP1/HMGB1/TYROBP/HAVCR2/VEGFB/AIF1/ALOX5AP/EIF2AK2/MAPKAPK3/PLSCR1/RAC1/PUM1/HLA-E/HLA-F/CEBPA/C3AR1/FLOT2/PTN/NMI/CCL21/COLEC11/RNF185/IL15/CYLD/IRAK1/CCL5/LY86/MEFV/CTSC/LYN/PLAU/BRCC3/RELA/CXCL13/SIRT2/KIR2DL4/NTF3/IL17A/TRIM41/HSPB1/AGER/ZNFX1/NFKBIL1/NRP1/CCR1/MMRN2/DDX3X/ARRB2/ETS1/PJA2/GHSR/CX3CR1/UNC93B1/LTA/MFHAS1/FFAR2/PDGFRA/S100A12/OXSR1/C3/GPSM3/S100A14/LTF

GOBP_EPITHELIAL_CELL_PROLIFERATION	GOBP_EPITHELIAL_CELL_PROLIFERATION	GOBP_EPITHELIAL_CELL_PROLIFERATION	84	0.348461955	2.622118918	1.98E-06	0.000124999	9.48E-05	924	tags=55%, list=29%, signal=40%	KLF9/CDK4/FGFR1/PHB2/DAB2/IGFBP4/IGF2/CEBPB/XBP1/HMGB1/SMO/VEGFB/LIPA/STAT3/ITGB1BP1/CD34/LOXL2/MYC/SULF1/TGFA/BMP4/PTN/NKX2-5/ALOX5/SNAI2/PURA/BCL2L1/TEK/BCL2L2/HAS2/NRP2/ID2/MMP14/YAP1/CDKN1C/CDH3/APLN/FGF1/LRG1/FRS2/IL17A/ERN1/MAGED1/SLURP1/NRP1/BAD
GOCC_LUMENAL_SIDE_OF_MEMBRANE	GOCC_LUMENAL_SIDE_OF_MEMBRANE	GOCC_LUMENAL_SIDE_OF_MEMBRANE	12	0.734791314	2.695948866	2.03E-06	0.000126091	9.56E-05	348	tags=75%, list=11%, signal=67%	HLA-DRB3/HLA-DQB1/HLA-B/HLA-E/HLA-F/HLA-DRB5/HM13/HLA-A/HLA-C
GOBP_POSITIVE_REGULATION_OF_IMMUNE_RESPONSE	GOBP_POSITIVE_REGULATION_OF_IMMUNE_RESPONSE	GOBP_POSITIVE_REGULATION_OF_IMMUNE_RESPONSE	137	0.284295841	2.449602	2.05E-06	0.000126091	9.56E-05	837	tags=47%, list=27%, signal=36%	NFKB1/TYK2/LAG3/PHB2/LSM14A/PYCARD/XBP1/PQBP1/HLA-DMB/HMGB1/TYROBP/ABL1/EIF2AK4/HAVCR2/CFHR3/HLA-DRB3/EIF2AK2/HLA-DQB1/MAPKAPK3/PLSCR1/LIPA/C1QC/PUM1/HLA-B/HLA-E/HLA-F/HLA-DRB5/PTPRC/C3AR1/BTNL2/HLA-DMA/HLA-A/FLOT2/HLA-C/CFHR5/NMI/PRKCH/COLEC11/RNF185/IL15/CYLD/CD177/IRAK1/HLA-DOB/CCL5/SLC11A1/BCL2/MEFV/BTN2A2/PTPN2/SVEP1/LYN/EIF2B1/FCER1G/BRCC3/RELA/BTN1A1/SIRT2/KIR2DL4/FOSL2/CD276/IL17A/TRIM41/C8A
GOBP_CILIUM_ORGANIZATION	GOBP_CILIUM_ORGANIZATION	GOBP_CILIUM_ORGANIZATION	72	-0.34356782	-2.69178733	2.08E-06	0.000126899	9.62E-05	817	tags=56%, list=26%, signal=42%	TBC1D7/MKKS/TBC1D10B/FNBP1L/FUZ/RABGAP1/BBS4/GSK3B/KIAA0753/TBC1D1/TTC17/IQCG/KIFAP3/IQCB1/IFT20/CEP350/DNAI1/SSNA1/CEP250/IFT74/RAB8B/DZIP1/KIF3A/CLUAP1/WDR54/RFX4/TBC1D3/RAB3IP/DNAH17/AKAP4/DNAH8/CCDC42/SPATA6/CDC14B/ZMYND10/IFT122/CCDC96/FOXJ1/RFX2/TTC21A
GOBP_INFLAMMATORY_RESPONSE	GOBP_INFLAMMATORY_RESPONSE	GOBP_INFLAMMATORY_RESPONSE	148	0.280183426	2.482958242	2.15E-06	0.000129317	9.80E-05	1415	tags=65%, list=45%, signal=38%	NFKB1/CASP4/RHBDF2/PYCARD/CEBPB/HMGB1/IFNGR1/TYROBP/SMO/HAVCR2/H19/AIF1/ALOX5AP/EIF2AK2/EIF2AK1/PLSCR1/FOLR2/LIPA/STAT3/RAC1/HLA-E/STAB1/PTPRC/CEBPA/HP/C3AR1/NFE2L2/ITGB1/PTN/ALOX5/NMI/ABHD12/CCL21/PTGES/IL15/CYLD/BM

											P2/TEK/PARP4/CCL5/SLC11A1/LY86/MEFV/PTPN2/CTSC/LYN/SCGB1A1/BRCC3/RELA/AOC3/NR3C1/CXCL13/IER3/SIRT2/PLA2G4C/APOD/CCR5/SERPINA1/FOSL2/IL17A/ADM/AGER/CXCR3/CCR1/MAP2K3/INS/SPN/ITGAM/DDX3X/AGR2/NFATC4/CCL8/ETS1/ADIPOQ/PJA2/CUEDC2/GHSR/CX3CR1/ADA/LTA/PXK/TIMP1/MFHAS1/XCR1/FFAR2/TRPV1/CCL22/CHIA/TNFRSF4/ABCD1/AKT1/S100A12/C3/GPSM3/F8/F11R
GOBP_SKELETAL_SYSTEM_DEVELOPMENT	GOBP_SKELETAL_SYSTEM_DEVELOPMENT	GOBP_SKELETAL_SYSTEM_DEVELOPMENT	94	0.325514547	2.52687272	2.31E-06	0.000137279	0.000104059	842	tags=49%, list=27%, signal=37%	FBN1/CTSK/MAF/NFIA/FGFR1/SH3PXD2B/CDH11/MED12/MGP/ZMPSTE24/TYROBP/TGFBI/OGN/ETS2/CHRD/GLI2/COL3A1/LIPA/EXTL1/LOXL2/PTPRC/HAND2/SULF1/ANXA6/BMP4/PRELP/MTHFD1/MYOC/CITED2/OTOR/SNAI2/PRDX1/BMP2/TEK/FOXC1/MEF2D/HAS2/MMP14/CBS/PLS3/COL27A1/CDKN1C/ENG/FOSL2/WNT11/TMEM119
GOBP_TUBE_MORPHOGENESIS	GOBP_TUBE_MORPHOGENESIS	GOBP_TUBE_MORPHOGENESIS	185	0.261080881	2.437275822	2.36E-06	0.000138435	0.000104935	1500	tags=65%, list=48%, signal=36%	SHC1/PARVA/RALA/PDGFRB/PHB2/MED12/XBP1/COL4A2/PGK1/ABL1/EMP2/TGFBI/SMO/VEGFB/CYP1B1/GLI2/COL3A1/STAT3/ITGB1BP1/CD34/LOXL2/ILK/STAB1/HEG1/MYC/GADD45A/C3AR1/NFE2L2/HAND2/ITGB1/SULF1/HIPK2/TGFA/BMP4/NKX2-5/MTHFD1/ALOX5/HS6ST1/CITED2/ZFPM2/TSC1/ADAM15/ADAM12/RAMP2/BMP2/TEK/COL15A1/EDA/FOXC1/CTSH/OR10J5/ANGPTL4/BCL2/SLC31A1/PODXL/HAS2/NRP2/ID2/MMP14/FZD4/COL4A1/CLDN5/NR3C1/CXCL13/COL27A1/YAP1/GPR15/ITGA5/APOD/APLN/FGF1/LRG1/ENG/ADM/WNT11/HSPB1/MAGED1/CXCR3/KLF2/NRP1/BAX/DVL2/CLEC14A/MMRN2/CUL7/ADIPOR2/ITGB8/ROCK2/AGR2/NFATC4/WNT2B/SMAD7/ETS1/ATF2/GHSR/CX3CR1/AQP1/LMO4/DLC1/ADRA2B/TBX6/RNH1/ANG/THY1/PDGFRA/NOTCH4/AKT1/CCBE1/NPHP3/SEC24B/E2F7/C3/CAV3/WNT3A/RARG/ELK3/PRRX

											1/PHACTR4/IRX3/WNT1/GJC1
GOBP_LEUKOCYTE_CELL_CELL_ADHESION	GOBP_LEUKOCYTE_CELL_CELL_ADHESION	GOBP_LEUKOCYTE_CELL_CELL_ADHESION	92	0.337893105	2.611145318	2.77E-06	0.000159773	0.00012111	1158	tags=62%, list=37%, signal=40%	TYK2/MSN/LAG3/EFNB1/PYCARD/IGF2/CEBPB/XBP1/HLA-DMB/HMGB1/ABL1/HAVCR2/AIF1/HLA-DRB3/GLI2/HLA-DQB1/PPP3CA/ST3GAL4/HLA-E/HLA-DRB5/PTPRC/ITGB1/HLA-DMA/HLA-A/FLOT2/BMP4/ALOX5/CBFB/CCL21/IL15/CD177/ITGB7/IRAK1/FGL2/HLA-DOB/CCL5/BTN2A2/HAS2/PTPN2/LYN/SCGB1A1/RELA/ITGA5/LRG1/CD276/AGER/BAD/CD4/SPN/CD83/PRKAR1A/TNFSF13B/PAG1/SOCS1/TNFRSF21/SMAD7/ETS1
GOBP_T_CELL_ACTIVATION	GOBP_T_CELL_ACTIVATION	GOBP_T_CELL_ACTIVATION	110	0.306460171	2.482493868	2.79E-06	0.000159773	0.00012111	1157	tags=58%, list=37%, signal=38%	TYK2/MSN/LAG3/EFNB1/PYCARD/IGF2/CEBPB/XBP1/HLA-DMB/HMGB1/ABL1/EIF2AK4/HAVCR2/AIF1/HLA-DRB3/MAFB/GLI2/HLA-DQB1/PPP3CA/LIPA/STAT3/HLA-E/HLA-DRB5/PTPRC/FKBP1A/HLA-DMA/HLA-A/FLOT2/BMP4/CBFB/CCL21/IL15/TSC1/FGL2/HLA-DOB/CCL5/SLC11A1/KIF13B/BCL2/AIRE/BTN2A2/PTPN2/HSH2D/LYN/SCGB1A1/FCER1G/LFNG/FOSL2/EOMES/CD276/AGER/BAD/INS/CD4/BAX/SPN/CD83/PRKAR1A/TNFSF13B/PAG1/CD7/SOCS1/TNFRSF21/SMAD7
GOBP_MRNA_3_END_PROCESSING	GOBP_MRNA_3_END_PROCESSING	GOBP_MRNA_3_END_PROCESSING	12	-0.702368138	-2.812922802	2.93E-06	0.000165919	0.000125768	723	tags=92%, list=23%, signal=71%	SYMPK/CPSF1/CPSF2/CSTF3/BARD1/WDR33/PAF1/CSTF1/ZNF473/NCBP2/CPSF3
GOBP_CELL_MOTILITY	GOBP_CELL_MOTILITY	GOBP_CELL_MOTILITY	316	0.212280172	2.204360873	3.05E-06	0.000170606	0.000129321	1421	tags=59%, list=45%, signal=36%	MSN/PARVA/F10/EFNB1/ARHGDIB/PODN/FGFR1/PDGFRB/PHB2/CDH11/DAB2/PYCARD/XBP1/IQGAP1/HMGB1/ABL1/EMP2/IFITM1/CAP1/SMO/VEGFB/CHRD/AIF1/CYP1B1/RAB1A/PLEKHG3/CD248/PPP3CA/WWC3/ST3GAL4/TSPO/FOLR2/COL3A1/TRIP6/PLXNA1/STAT3/RAC1/ITGB1BP1/CD34/LOXL2/ILK/GADD45A/PTPRC/C3AR1/PLTP/NFE2L2/HAND2/ITGB1/SULF1/IGFBP6/ANXA6/CORO6/BMP4/PTN/APCDD1/ALOX5/MYOC/CITED2/SNAI2/CCL21/ITGA9/TNFAIP1/CD177/ADAM15/ITGB7/CDK5/MYA

										DM/CLN3/TOR1A/BMP2/TEK/CCL5/FOXC1/SDC2/CTSH/ABHD6/RAB11A/BCL2/AIRE/MALAT1/PODXL/HAS2/NRP2/CPNE3/ARHGEF7/ARF4/PHLDB2/RHOC/MMP14/FZD4/LYN/LIMA1/UBE2B/TRPM2/PIK3C2B/PLAU/FCER1G/CLDN5/FAM107A/GNA12/CXCL13/GPR15/ITGA3/ITGA5/APOD/FGF1/CCR5/GFRA3/NTF3/LRG1/ENG/EOMES/IL17A/WNT11/HSPB1/AGER/MIXL1/SLURP1/NET1/CXCR3/SERPINE2/MDM2/CEND1/NRP1/CLASP2/CDK5R2/CCR1/NHLH2/MAP2K3/SLC26A5/INS/BAX/SPN/RHOG/MAZ/CLEC14A/MMRN2/GRB7/DUOX2/MINK1/CDC42BPB/FGF19/PTP4A1/ITGB8/FGF13/ROCK2/ARRB2/CLDN4/CCL8/SMAD7/ETS1/ADIPOQ/ABI3/TNXB/CX3CR1/ZNF703/AQP1/LMO4/ASCL1/ADIPOR1/PSG2/DLC1/ADA/CTTN/TOP2B/JAGN1/EPB41L4B/TIMP1/XCR1/FFAR2/RNH1/CCL22/ANG/THY1/SELPLG/PDGFRA/AKT1/S100A12/AUTS2/CCBE1/OXSR1/GPSM3/S100A14/SPATA13/F11R/RND3
GOCC_MICROTUBULE_ASSOCIATED_COMPLEX	GOCC_MICROTUBULE_ASSOCIATED_COMPLEX	GOCC_MICROTUBULE_ASSOCIATED_COMPLEX	34	-0.47303641	-2.902907838	3.21E-06	0.000177693	0.000134692	620	tags=59%, list=20%, signal=48%	KIF22/KIF4A/KIFAP3/DYNC1LI1/DNAI1/KIFC3/NDE1/KIF23/KIF3A/KATNB1/DNAH17/TPR/DNAH8/AURKC/DYNLRB2/CDCA8/DNALI1/KATNA1/DYNLL2/PAFAH1B1
GOMF_EXTRACELLULAR_MATRIX_STRUCTURAL_CONSTITUENT	GOMF_EXTRACELLULAR_MATRIX_STRUCTURAL_CONSTITUENT	GOMF_EXTRACELLULAR_MATRIX_STRUCTURAL_CONSTITUENT	37	0.465927602	2.680123773	3.33E-06	0.000180079	0.000136502	798	tags=65%, list=25%, signal=49%	FBN1/PODN/MGP/COL4A2/TGFBI/OGN/ABI3BP/COL3A1/COL4A5/COL6A3/PCOLCE/COL4A6/PRELP/LUM/SRPX/VWF/FGL2/COL15A1/ELN/COL4A1/FBN3/LTBP1/COL27A1/IMPG2
GOBP_REGULATION_OF_LOCOMOTION	GOBP_REGULATION_OF_LOCOMOTION	GOBP_REGULATION_OF_LOCOMOTION	199	0.246634731	2.336104211	3.30E-06	0.000180079	0.000136502	1414	tags=62%, list=45%, signal=36%	MSN/F10/ARHGDIB/PODN/FGFR1/PDGFRB/CDH11/DAB2/PYCARD/XBP1/HMGB1/ABL1/EMP2/IFITM1/SMO/VEGFB/CHRD/AIF1/CYP1B1/PLEKHG3/PPP3CA/ST3GAL4/COL3A1/TRIP6/PLXNA1/STAT3/RAC1/ITGB1BP1/GADD45A/PTPRC/C3AR1/NFE2L2/ITGB1/SULF1/BMP4/PTN/MYOC/CITED2/SNAI2/CCL21/ADAM15/CDK5/MYADM/CLN3/BMP2/TEK

											/ROBO3/CCL5/CTSH/ABHD6/RAB11A/BCL2/AIRE/MALAT1/PODXL/HAS2/NRP2/CPNE3/ARHGEF7/PHLDB2/RHOC/PTPN2/MMP14/FZD4/LYN/PLAU/CLDN5/FAM107A/GNA12/CXCL13/ITGA3/ITGA5/APOD/FGF1/NTF3/ENG/WNT11/HSPB1/AGER/SLURP1/CXCR3/SERPINE2/MDM2/NRP1/CLASP2/CCR1/MAP2K3/SLC26A5/INS/SPN/MAZ/MMRN2/GRB7/DUOX2/MINK1/FGF19/PTP4A1/ROCK2/CLDN4/SMAD7/ETS1/ADIPOQ/ABI3/TNXB/GHSR/CX3CR1/ZNF703/AQP1/LMO4/ADIPOR1/DLC1/ADA/EPB41L4B/TIMP1/THY1/PDGFRA/AKT1/CCBE1/OXSR1/GPSM3/WNT3A/S100A14/SPATA13
GOBP_CHROMOSOME_SEGREGATION	GOBP_CHROMOSOME_SEGREGATION	GOBP_CHROMOSOME_SEGREGATION	90	-0.308267567	-2.637828054	3.48E-06	0.000185696	0.000140759	700	tags=58%, list=22%, signal=46%	TUBGCP2/MAPRE1/SMC4/CHEK2/MAP1S/NUP37/LCMT1/CDC16/UBE2C/NUSAP1/BCCIP/ZW10/SMARCC1/TOP3A/AKAP8L/TUBB/CDK5RAP2/KIF22/KIF4A/SMC3/MAD2L1BP/PINX1/TUBG2/BRD7/ESPL1/MAD2L2/DYNC1LI1/KPNB1/CEP63/CENPH/TPX2/SMC6/ITGB3BP/TRIP13/NDE1/KIF23/TUBG1/CENPE/BUB1B/KATNB1/CSNK2A2/C1orf112/STAG3/CENPM/TPR/AURKC/SRPK1/CDCA8/SPAG5/CCNB2/PTTG1/PTTG2
GOBP_MRNA_PROCESSING	GOBP_MRNA_PROCESSING	GOBP_MRNA_PROCESSING	87	-0.304753053	-2.568510566	3.54E-06	0.000187084	0.000141811	675	tags=60%, list=21%, signal=48%	LUC7L2/KHSRP/ADARB2/RBM26/LUC7L/TSEN54/PRPF6/SF3B4/RBBP6/SF3B2/PABPN1/RNGTT/LSM3/CTNNBL1/SAFB/SYMPK/IWS1/CPSF1/AKAP8L/CPEB1/ECD/CPSF2/CSTF3/RBM14/ELAVL4/THOC5/PRPF40A/BARD1/PNPT1/WDR33/DUS3L/SF3A1/GEMIN4/GEMIN6/PRPF18/PAF1/CSTF1/PRPF40B/ZRANB2/TFIP11/SRPK1/DHX16/SETX/PRPF38A/ZNF473/BUD13/DAZAP1/DDX20/KHDRBS3/NCBP2/PRKRIP1/CPSF3
GOBP_ORGANELLE_FISSION	GOBP_ORGANELLE_FISSION	GOBP_ORGANELLE_FISSION	108	-0.281400335	-2.543002399	3.63E-06	0.000189601	0.000143719	649	tags=41%, list=21%, signal=34%	AKAP8L/CDK5RAP2/KIF22/KIF4A/PINK1/SMC3/MAD2L1BP/PINX1/TUBG2/ESPL1/MAD2L2/DYNC1LI1/KPNB1/TPX2/TRIP13/NDE1/PIWIL2/KIF23/TUBG1/UBE2S/CENPE/TDRKH/BUB1B/FZR1/KAT

											NB1/MTFR1/DDHD1/PEX11G/STAG3/RAD51/CKS2/TPR/AURKC/CDCA8/HSPA2/RANBP1/CDC14B/CDC25C/MYBL1/SPAG5/CCNB2/AURKAIP1/PTTG1/PTTG2
GOBP_SMALL_MOLECULE_METABOLIC_PROCESS	GOBP_SMALL_MOLECULE_METABOLIC_PROCESS	GOBP_SMALL_MOLECULE_METABOLIC_PROCESS	372	0.205812749	2.19382219	3.73E-06	0.000193122	0.000146388	980	tags=42%, list=31%, signal=33%	FUCA2/NFKB1/ALDH1A3/DCTD/AKR1A1/OSBPL5/AKR1C1/CBR1/GALT/DAB2/IGFBP4/PYCARD/IGF2/XBP1/PGK1/HYI/ZMPSTE24/GSTO1/ACOT9/NUDT16/LTC4S/H19/VDAC1/CYP1B1/ALOX5AP/GLB1/OSBPL1A/NADK/CAT/NT5C/GCSH/TSPO/PGLS/ATP1A2/CA14/MAN2B2/IMPDH2/LIPA/APRT/STAT3/ACSL5/ECHDC1/CYP2U1/MBTPS1/ITPK1/CEBPA/ADHFE1/ACO1/PLTP/ADI1/GNE/GALM/NEIL1/RBP1/SLC25A12/AIG1/ATF4/ENTPD6/NQO1/MTHFD1/DERA/ALDH2/PPT2/NFE2L1/ALOX5/DHTKD1/FMO1/ABHD12/PTGES2/ACAA1/SNAI2/PIPOX/COQ10A/BPHL/PTGES/HMGCL/ACY1/FECH/AMPD2/CLN3/SUCLG2/EXTL2/CA11/BMP2/DHCR24/ZBTB7A/PDXK/CYP2W1/IDH3B/IDH3G/BCAT2/HNMT/TFF3/ABHD6/CYP11A1/SLC16A9/NTHL1/GTPBP1/PNPLA4/LARS2/ASNSD1/PTPN2/CYP17A1/STARD4/UGT2A3/CBS/NME4/OAT/PGM2/LIMA1/CYP4B1/TDG/KMO/NR3C1/PRPS1/PRKAB2/NNMT/IER3/SIRT2/PLA2G4C/APOD/CYP2S1/FGF1/GNPDA1/ALG2/NME6/ADM/ILVBL/LDHA/GCLC/ATP8B1/AKR7A2/SCP2/RRM2B/NSDHL/ADH1C/ENTPD8/LTA4H/ECH1/PMVK/BAD/UCKL1/ACOX1/NR1H2/OSBP/FADS6/INPPL1/OMA1/NUDT8/HSD17B8/ACAD11/INS/ACSL1/PDK2/TALDO1/SLC22A11
GOBP_HEMOPOIESIS	GOBP_HEMOPOIESIS	GOBP_HEMOPOIESIS	178	0.254549281	2.367344475	4.39E-06	0.000224826	0.00017042	1427	tags=62%, list=45%, signal=36%	SH3PXD2A/FBN1/CTSK/MAF/LAG3/CEBPB/TFE3/XBP1/HMGB1/TYROBP/ABL1/HCLS1/EIF2AK2/EIF2AK1/MAFB/GLI2/PPP3CA/WDR7/SLC25A5/LIPA/STAT3/C1QC/CD34/HLA-B/INHBA/MYC/PTPRC/CEBPA/NFE2L2/LTBR/ITGB1/HIPK2/

											BMP4/PTN/ATF4/NKX2-5/DHTKD1/CITED2/CBFB/SNAI2/IL15/FECH/TSC1/PIAS3/BMP2/TEK/ZBTB7A/FGL2/FOXC1/ISG15/BCL2/AIRE/BTN2A2/ID2/PTPN2/MMP14/FLT3LG/LYN/TRPM2/FCER1G/TCTA/NR3C1/HERC6/PRDX3/LFNG/CDKN1C/FOSL2/EOMES/IL17A/OSTM1/AGER/MIXL1/BATF2/KLF10/KLF2/BAD/CCR1/CD4/BAX/HEATR3/SPN/CD83/SSBP3/PRMT6/ITGB8/TNFSF13B/FAM20C/ALAS1/SOCS1/WNT2B/SMAD7/ETS1/ADIPOQ/ATF2/MKNK2/EGR1/ADA/EPOR/TOP2B/JAGN1/MFHAS1/MEN1/CD79A/PDGFRA/NOTCH4/ZBTB16/WNT3A/ATG5/RARG/LTF
GOBP_NEGATIVE_REGULATION_OF_MULTICELLULAR_ORGANISMAL_PROCESS	GOBP_NEGATIVE_REGULATION_OF_MULTICELLULAR_ORGANISMAL_PROCESS	GOBP_NEGATIVE_REGULATION_OF_MULTICELLULAR_ORGANISMAL_PROCESS	215	0.238819539	2.303343201	4.52E-06	0.000226523	0.000171706	1443	tags=61%, list=46%, signal=36%	FBN1/NFKB1/CTSK/LAG3/ARHGDIB/TFPI/PHB2/PYCARD/CEBPB/TFE3/COL4A2/HMGB1/PGK1/IFNGR1/TYROBP/SMO/HAVCR2/MAFB/PPP3CA/WWC3/SOCS2/BTG2/TSPO/ATP1A2/RAC1/C1QC/ITGB1BP1/CD34/LOXL2/INHBA/HLA-F/STAB1/MYC/GADD45A/PTPRC/CEBPA/ADAMTS5/SULF1/BMP4/PTN/ATF4/ANXA4/ALOX5/NMI/CITED2/CBFB/ZFPM2/SNAI2/IL15/CYLD/PIAS3/CDK5/BMP2/TEK/FGL2/FOXC1/SLC11A1/ABHD6/BCL2/MEFV/BTN2A2/SRI/ID2/PTPN2/CTSC/LYN/NOVA1/SCGB1A1/PLAU/CLDN5/TCTA/CXCL13/SIRT2/CDH3/APOD/FRS2/ADM/WNT11/TMEM119/GCLC/AGER/VGLL4/SERPINE2/KLF2/NFKBIL1/NRP1/CCR1/NR1H2/INS/SPN/MMRN2/CD83/ULK1/FGF13/TSPAN8/PRKAR1A/ROCK2/PAG1/ARRB2/NFATC4/SOCS1/TNFRSF21/GDI1/SMAD7/PITX3/ADIPOQ/CUEDC2/ATF2/GHSR/CX3CR1/ADIPOR1/CSK/ADA/KREMEN1/TIMP1/TRPV1/FXN/THY1/ABCD1/CYP51A1/PDGFRA/F11/MT3/CAV3/WNT3A/ATG5/ACOT11/RARG/LTF/DAB1/TSPAN32/FOXE3
GOBP_ANIMAL_ORGAN_MORPHOG	GOBP_ANIMAL_ORGAN_MORPHOG	GOBP_ANIMAL_ORGAN_MORPHOGENESIS	205	0.241078433	2.300699525	4.53E-06	0.000226523	0.000171706	1006	tags=46%, list=32%,	FBN1/NFKB1/MSN/ALDH1A3/PARVA/FGFR1/PHB2/DAB2/IGF2/MED12/CEBPB

ENESIS	ENESIS									signal=34%	/XBP1/MGP/ZMPSTE24/ABL1/EMP2/SMO/ZNF22/MAFB/GLI2/PPP3CA/PERP/COL3A1/LIPA/PLXNA1/STAT3/RAC1/CD34/INHBA/ILK/HEG1/MYC/HAND2/FKBP1A/ADAMTS5/AHDC1/FBXW11/SULF1/HIPK2/ANXA6/TGFA/BMP4/PTN/ATF4/NKX2-5/MTHFD1/PLEKHA4/APCDD1/TRIOBP/CITED2/ZFPM2/OTOR/SNAI2/ADAM15/BMP2/TEK/EDA/FOXC1/CTSH/ELN/MEF2D/BCL2/HAS2/NRP2/PAX9/ID2/PLAG1/MMP14/CBS/RELA/CLDN5/NR3C1/LFNG/COL27A1/YAP1/FGF1/IMPG2/ENG/FRS2/FOSL2/ADM/WNT11/TMEM119/ADPRHL1/MAGED1/MDM2/PKP2/NRP1/INPPL1/BAX/OTOP1/FHL1/DVL2/ETV7/LCTL
GOBP_HOMEOSTATIC_PROCESS	GOBP_HOMEOSTATIC_PROCESS	GOBP_HOMEOSTATIC_PROCESS	337	0.21008649	2.211303074	4.57E-06	0.000226523	0.000171706	1501	tags=60%, list=48%, signal=35%	CCDC115/CTSK/AKR1C1/CYBRD1/CEBPB/TFE3/XBP1/HMGB1/ABL1/GSTO1/SMO/HCLS1/GPRASP2/EIF2AK1/ITPR1/MAFB/NADK/PPP3CA/ARMCX1/SLC25A23/SLC25A5/TSPO/ATP1A2/COL3A1/LIPA/STAT3/CD34/INHBA/CLNS1A/MYC/PTPRC/TPCN1/CEBPA/ACO1/SLC30A9/NFE2L2/SCARA5/CCDC51/EIF4G1/FKBP1A/ADAMTS5/ITGB1/RBP1/ABCB7/HIPK2/ANXA6/SLC29A1/BMP4/ATF4/NQO1/MTHFD1/MUC2/BACE2/ALOX5/FMO1/CITED2/SLC12A9/KCNE3/CCL21/WFS1/MT1B/PTGES/IL15/FECH/AMPD2/CLN3/KCNB1/MT1E/PRDX1/EBF2/ZBTB7A/CCL5/FOXC1/SLC11A1/MPV17/SLC34A2/CTSH/SLC9A8/OR10J5/TFF3/ABHD6/ANGPTL4/RPH3AL/ISG15/PNPLA4/BCL2/SLC31A1/HAS2/MT1H/SRI/ID2/IBTK/PTPN2/LYN/LIMA1/NOVA1/TRPM2/ATP2A3/CLDN5/F2RL3/PRKAB2/PRDX3/OCIAD2/YAP1/GRM1/CDH3/PPP2R1A/CCR5/FOSL2/PRR4/IL17A/WDR37/TMEM119/HSPB1/ERN1/GCLC/AGER/GPR21/CHMP2B/SQSTM1/CRTC3/KLF2/BAD/ACOX1/CCR1/NR1H2/OSBP/UBTF/RRAGA/CALM3/OMA1/INS/LIME1/ACSL1/B

											AX/HEATR3/PDK2/CREG1/SLC24A3/CNNM2/EHMT1/ADIPOR2/DDX3X/TNFSF13B/GPR3/CNBP/ALAS1/CCL8/ETS1/APBB2/CROCC/ADIPOQ/DIAPH1/ATP6V1G2/VAPB/CX3CR1/SGIP1/TSC22D3/SYPL2/AQP1/EGR1/ADIPOR1/CSK/HTR1B/ADA/AQP6/CFL2/NUBP1/JAGN1/OCIAD1/MFHAS1/XCR1/FFAR2/EMX1/TRPV1/ANG/FXN/THY1/ABCD1/AKT1/NPHP3/OXSR1/SH2B2/MT3/CAV3/S100A14/CLCN6/F11R/CA2/ATG5/ACOT11/LTF/FIS1/THRA/ATP13A1/TMEM106B/ATP6V0A1/IRX3/PCSK1N/BCR/SLC34A3
GOBP_SENSORY_PERCEPTION_OF_SMELL	GOBP_SENSORY_PERCEPTION_OF_SMELL	GOBP_SENSORY_PERCEPTION_OF_SMELL	66	0.375178072	2.640596654	4.62E-06	0.000226603	0.000171767	1525	tags=80%, list=48%, signal=42%	OR51A7/OR5V1/OR52E4/NAV2/OR9K2/OR13C4/OR10T2/OR6V1/OR8G1/OR10J5/OR6C4/OR1E2/OR5F1/OR52D1/OR8A1/OR10H4/OR4K17/OR8H2/OR6X1/OR8D1/OR52B6/OR4K14/OR7G2/OR6Y1/OR3A3/OR5AS1/OR5D18/OR4S1/OR2T4/OR7D2/OR6M1/OR6K2/OR2A12/TTC8/OR8B8/OR8S1/OR6B2/OR51S1/OR2J2/OR1D4/OR51L1/OR52M1/OR13C8/OR4D5/OR1D2/OR4M1/OR1F1/OR4D1/OR10A4/OR10H3/OR4P4/OR2B11/B3GNT2
GOCC_CHROMOSOME_CENTROMERIC_REGION	GOCC_CHROMOSOME_CENTROMERIC_REGION	GOCC_CHROMOSOME_CENTROMERIC_REGION	52	-0.382045461	-2.694990507	5.12E-06	0.000246175	0.000186603	708	tags=56%, list=22%, signal=44%	CBX1/TP53BP1/ZW10/SMARCC1/KIF22/BAZ1A/SMC3/PINX1/BRD7/DYNC1LI1/CENPH/SMC6/ITGB3BP/NDE1/PPP1CC/CENPE/SMC1B/BUB1B/SUGT1/C1orf112/STAG3/CENPM/TPR/AURKC/CDCA8/SPAG5/SUV39H2/PAFAH1B1/AHCTF1
GOBP_RESPONSE_TO_GROWTH_FACTOR	GOBP_RESPONSE_TO_GROWTH_FACTOR	GOBP_RESPONSE_TO_GROWTH_FACTOR	145	0.272401041	2.392867431	5.08E-06	0.000246175	0.000186603	1235	tags=57%, list=39%, signal=36%	SHC1/FBN1/CTSK/NFIA/FGFR1/PDGFRB/DAB2/HTRA1/IQGAP1/COL4A2/ABL1/VEGFB/CHRD/RGMB/CAT/COL3A1/STAT3/ITGB1BP1/ILK/MYC/FKBP1A/ITGB1/CYFIP1/SULF1/HIPK2/BMP4/PPP2R5B/TGFBRAP1/CITED2/EHD4/SNAI2/RAMP2/BMP2/ZBTB7A/CCL5/FOXC1/UBE2D1/HAS2/NRP2/CPNE3/RAPGEF1/PAX9/FZD4/FNTA/SCGB1A1/RELA/CLDN5/NR3C1/CXCL13/LTBP1/CDKN1C/ITGA3/ITGA5/APLN/FGF1/NTF3/EMD/LRG1/ENG/FRS2/HSPB1/ERN1/GCLC/MD

											M2/NRP1/MAP2K3/STUB1/MMRN2/FGF19/ITGB8/ROCK2/MYOG/FAM20C/CASK/ARRB2/SMAD7/PITX3/ATF2/TNXB/CX3CR1/ZNF703/EGR1/ASCL1
GOBP_REGULATION_OF_IMMUNE_SYSTEM_PROCESS	GOBP_REGULATION_OF_IMMUNE_SYSTEM_PROCESS	GOBP_REGULATION_OF_IMMUNE_SYSTEM_PROCESS	275	0.220591627	2.23093801	5.26E-06	0.000250328	0.000189751	1427	tags=60%, list=45%, signal=36%	FBN1/NFKB1/TYK2/MSN/LAG3/EFNB1/PHB2/LSM14A/RHBDF2/PYCARD/IGF2/CEBPB/TFE3/XBP1/PQBP1/HLA-DMB/HMGB1/TYROBP/ABL1/EIF2AK4/HAVCR2/HCLS1/VEGFB/CFHR3/AIF1/HLA-DRB3/EIF2AK2/MAFB/GLI2/HLA-DQB1/MAPKAPK3/PPP3CA/PLSCR1/ST3GAL4/COL3A1/LIPA/STAT3/RAC1/C1QC/PUM1/HLA-B/HLA-E/INHBA/HLA-F/HLA-DRB5/MYC/PTPRC/CEBPA/C3AR1/NFE2L2/FKBP1A/BTNL2/HLA-DMA/HLA-A/FLOT2/HLA-C/BMP4/PTN/CFHR5/NMI/PRKCH/CBFB/SNAI2/CCL21/COLEC11/RNF185/IL15/CYLD/CD177/PIAS3/IRAK1/FGL2/HLA-DOB/CCL5/SLC11A1/CHRNB2/CTSH/ISG15/BCL2/MEFV/AIRE/BTN2A2/ID2/PTPN2/TAPBPL/MMP14/CTSC/SVEP1/LYN/SCGB1A1/EIF2B1/FCER1G/BRCC3/RELA/TCTA/CXCL13/BTN1A1/SIRT2/KIR2DL4/APOD/FOSL2/CD276/IL17A/TRIM41/C8A/AGER/CXCR3/ZNFX1/KLF10/NFKBIL1/BAD/CCR1/NR1H2/INPPL1/INS/LIME1/CD4/BAX/OTOP1/TRAFD1/SPN/ITGAM/CD1A/CD83/PRMT6/PRKAR1A/DDX3X/TNFSF13B/PAG1/ARRB2/SOCS1/TNFRSF21/SMAD7/ETS1/ADIPOQ/PJA2/CUEDC2/CX3CR1/CD1B/TSC22D3/CSK/ADA/UNC93B1/STX7/LTA/MFHAS1/FFAR2/CD79A/THY1/SUPT6H/TNFRSF4/AKT1/TAP2/OXSR1/ZBTB16/SH2B2/C3/GPSM3/PSG8/WNT3A/S100A14/ATG5/RARG/LTF
GOCC_CHROMOSOME_TELOMERIC_REGION	GOCC_CHROMOSOME_TELOMERIC_REGION	GOCC_CHROMOSOME_TELOMERIC_REGION	30	-0.489585666	-2.858952025	5.85E-06	0.000273309	0.00020717	992	tags=80%, list=31%, signal=55%	HMBOX1/RIF1/HAT1/CHEK2/PARP1/ATR/CBX1/MCM5/RAD50/TP53BP1/POT1/WRN/PINX1/THOC5/PCNA/BLM/SMC6/RECQL4/PPP1CC/TFIP11/RAD51/SETX/RAD17/SMCHD1
GOCC_MICROTU	GOCC_MICROTUB	GOCC_MICROTUBULE	87	-0.297517613	-2.507529045	5.83E-06	0.00027330	0.00020717	648	tags=43%,	HOOK2/TUBB/CDK5RAP2/NME7/KIF22

BULE	ULE						9			list=21%, signal=35%	/KIF4A/RASSF1/PACRG/KIFAP3/TUBG2/FKBP4/DYNC1LI1/HSPH1/RASSF3/MAP2K2/TPX2/DNAI1/MAP6D1/KIFC3/NDE1/KIF23/TUBG1/CENPE/KIF3A/KATNB1/EFHC1/ARFGEF2/POLB/DNAH17/DNAH8/AURKC/DYNLRB2/KATNA1/SPAG5/DYNLL2/PAFAH1B1/SPAG8
GOBP_RNA_PROCESSING	GOBP_RNA_PROCESSING	GOBP_RNA_PROCESSING	174	-0.226559392	-2.311947111	5.96E-06	0.00027554	0.000208862	792	tags=41%, list=25%, signal=32%	LSM3/DDX49/CTNNBL1/SAFB/PDCD7/TAF9/YRDC/SYMPK/FASTKD1/BOP1/TAF10/PA2G4/IWS1/CPSF1/AKAP8L/DDX52/CPEB1/TRPT1/ECD/CPSF2/SMAD2/CSTF3/RBM14/ELAVL4/THOC5/NOL6/PRPF40A/DUS1L/BARD1/PNPT1/WDR33/EBNA1BP2/UTP18/DUS3L/PWP1/TPRKB/SF3A1/METTL6/GEMIN4/PIWIL2/EXOSC8/EXOSC4/GEMIN6/PRPF18/PAF1/INTS7/CSTF1/ZNHIT3/TDRKH/PRPF40B/GTPBP4/INTS6/ZRANB2/TFIP11/FKBP6/SRPK1/DHX16/SETX/SUPT3H/PRPF38A/TDRD7/ZNF473/BUD13/DAZAP1/TSN/DDX20/KHDRBS3/NUP155/NCBP2/PRKRIP1/CPSF3
GOBP_RESPONSE_TO_OXYGEN_CONTAINING_COMPOUND	GOBP_RESPONSE_TO_OXYGEN_CONTAINING_COMPOUND	GOBP_RESPONSE_TO_OXYGEN_CONTAINING_COMPOUND	361	0.202328975	2.15478309	6.06E-06	0.000277649	0.00021046	1428	tags=57%, list=45%, signal=35%	SHC1/FBN1/NFKB1/TYK2/CTSK/CASP4/MSN/AKR1A1/KLF9/TOMM20/AKR1C1/CDK4/TNFSF10/PHB2/MAOB/PYCARD/RGS10/IGF2/CEBPB/XBP1/IQGAP1/HMGB1/ABL1/SMO/HAVCR2/H19/CYP1B1/ALOX5AP/ITPR1/GLB1/NADK/CAT/MAPKAPK3/PPP3CA/SLC25A23/SOCS2/BTG2/TSPO/ATP1A2/FOLR2/COL3A1/LIPA/STAT3/COL6A3/INHBA/MBD2/PTPRC/CEBPA/HP/NFE2L2/HAND2/EIF4G1/CYFIP1/COL4A6/MAP3K5/SLC29A1/SLC25A12/ENTPD6/NQO1/ADD1/FMO1/IGF2R/SNAI2/CCL21/FECH/TSC1/MAS1L/ADAM15/CDK5/IRAK1/BCL2L1/KCNB1/PRDX1/PALM/CCS/TEK/ALAD/SLC11A1/MPV17/CHRNB2/CTSH/CYP11A1/BCL2L2/LY86/BCL2/HRH3/AANAT/VPS13C/CPNE3/RAPGEF1/SRI/PTPN2/FZD4/DENND4C/CBS/LYN/SYT12/TRPM2/KMO/SCGB1A1/EIF2B1/RELA/COL4A1/C

											LDN5/BCHE/NR3C1/CXCL13/PRDX3/SIRT2/YAP1/ANKZF1/APOD/GNAI1/CCR5/FOSL2/ALG2/ADM/TRIM41/WNT11/ERN1/NCOA3/GCLC/AGER/NET1/GPR21/SQSTM1/LTA4H/ARPC1B/MDM2/KLF2/NFKBIL1/CRHR2/PMVK/BAD/OSBP/UBTF/UCN3/MAP2K3/INPPL1/RRAGA/CALM3/SLC26A5/PPIF/INS/ACSL1/OTOP1/PDK2/GRB7/DUOX2/FGF19/PRKAR1A/ROCK2/GNB1/MYOG/ARRB2/NPAS4/MMP15/NFATC4/ALAS1/CLDN4/SOCS1/GYS2/GDI1/PITX3/ADIPOQ/ACTN2/GRAMD1A/GHSR/CX3CR1/SLC6A1/ZNF703/AQP1/EGR1/ASCL1/TSHB/ADIPOR1/CSK/HTR1B/TOP2B/UMODL1/JAGN1/LTA/ELK1/CHRNA2/TIMP1/FFAR2/JUND/TRPV1/FXN/P2RX2/HCN4/SSTR3/CSHL1/PDGFRA/AKT1/DLG4/SH2B2/MT3/WNT3A/S100A14/CA2/ATG5/CDK2/RARG/LTF/HCN3
GOBP_CELL_CYCLE_CHECKPOINT_SIGNALING	GOBP_CELL_CYCLE_CHECKPOINT_SIGNALING	GOBP_CELL_CYCLE_CHECKPOINT_SIGNALING	45	-0.408761057	-2.820483497	6.25E-06	0.000283467	0.000214871	787	tags=67%, list=25%, signal=51%	CHEK2/LCMT1/RINT1/ATR/TIMELESS/RAD50/TP53BP1/ZW10/E2F1/CDK5RAP2/MAD2L1BP/THOC5/BARD1/MAD2L2/DYNC1LI1/CEP63/BLM/TRIP13/DONSON/INTS7/BUB1B/FZR1/MDC1/RAD51/TPR/CRY1/CDCA8/DOT1L/CDC14B/RAD17
GOBP_POSITIVE_REGULATION_OF_IMMUNE_SYSTEM_PROCESS	GOBP_POSITIVE_REGULATION_OF_IMMUNE_SYSTEM_PROCESS	GOBP_POSITIVE_REGULATION_OF_IMMUNE_SYSTEM_PROCESS	194	0.248759754	2.350687232	6.49E-06	0.000291856	0.00022123	1016	tags=48%, list=32%, signal=35%	NFKB1/TYK2/LAG3/EFNB1/PHB2/LSM14A/PYCARD/IGF2/XBP1/PQBP1/HLA-DMB/HMGB1/TYROBP/ABL1/EIF2AK4/HAVCR2/HCLS1/VEGFB/CFHR3/AIF1/HLA-DRB3/EIF2AK2/GLI2/HLA-DQB1/MAPKAPK3/PPP3CA/PLSCR1/LIPA/RAC1/C1QC/PUM1/HLA-B/HLA-E/HLA-F/HLA-DRB5/PTPRC/CEBPA/C3AR1/BTNL2/HLA-DMA/HLA-A/FLOT2/HLA-C/PTN/CFHR5/NMI/PRKCH/CBFB/CCL21/COLEC11/RNF185/IL15/CYLD/CD177/IRAK1/HLA-DOB/CCL5/SLC11A1/CHRNB2/BCL2/MEFV/BTN2A2/ID2/PTPN2/MMP14/CTSC/SVEP1/LYN/EIF2B1/FCER1G/BRCC3/RELA/CXCL13/BTN1A1/SIRT2/

											KIR2DL4/FOSL2/CD276/IL17A/TRIM41/C8A/AGER/ZNFX1/KLF10/NFKBIL1/BAD/CCR1/LIME1/CD4/BAX/SPN/ITGAM/CD1A/CD83
GOBP_CELL_SUBSTRATE_ADHESION	GOBP_CELL_SUBSTRATE_ADHESION	GOBP_CELL_SUBSTRATE_ADHESION	85	0.32098636	2.441788743	6.82E-06	0.000303749	0.000230244	1263	tags=64%, list=40%, signal=39%	PARVA/CDH11/DAB2/ABL1/EMP2/RAB1A/ABI3BP/COL3A1/TRIP6/RAC1/ITGB1BP1/CD34/RSU1/ILK/ITGB1/MYOC/TRIOBP/CCL21/PARVG/ITGA9/TSC1/ADAM15/ITGB7/CDK5/VWF/MYADM/TEK/EDA/BCL2/HAS2/ARHGEF7/PHLDB2/MMP14/FZD4/PLAU/FAM107A/CORO2B/ITGA3/ITGA5/APOD/PKP2/NRP1/CLASP2/ITGAM/MINK1/ITGB8/ROCK2/AGR2/CASK/PARVB/ACTN2/TNXB/DLC1/CTTN
GOBP_RNA_LOCALIZATION	GOBP_RNA_LOCALIZATION	GOBP_RNA_LOCALIZATION	39	-0.422463052	-2.756889213	6.93E-06	0.000305742	0.000231755	1176	tags=79%, list=37%, signal=50%	RBM15B/MCM3AP/AAAS/NUTF2/KHSRP/PRPF6/XPO1/NUP107/IGF2BP1/NUP37/PABPN1/RANBP2/ATR/IWS1/SMG7/AKAP8L/NUP93/THOC5/NOL6/SNUPN/PNPT1/PARP11/KPNB1/RUVBL1/ZNHIT3/TPR/NUP88/RAE1/NUP155/AHCTF1/NCBP2
GOBP_MUSCLE_CELL_MIGRATION	GOBP_MUSCLE_CELL_MIGRATION	GOBP_MUSCLE_CELL_MIGRATION	22	0.560250791	2.623299146	7.07E-06	0.000309053	0.000234265	919	tags=77%, list=29%, signal=55%	PARVA/PDGFRB/SMO/AIF1/CYP1B1/PLXNA1/ITGB1BP1/NFE2L2/BMP4/CCL5/BCL2/HAS2/PLAU/GNA12/NET1/MDM2/NRP1
GOBP_CILIUM_MOVEMENT	GOBP_CILIUM_MOVEMENT	GOBP_CILIUM_MOVEMENT	21	-0.561698968	-2.887539894	7.22E-06	0.000312812	0.000237114	382	tags=57%, list=12%, signal=51%	DNAI1/DZIP1/DNAH17/AKAP4/DNAH8/IQCF1/SPA17/ZMYND10/ROPN1L/LZTFL1/CATSPER2/TTC21A
GOBP_POSITIVE_REGULATION_OF_CELL_ACTIVATION	GOBP_POSITIVE_REGULATION_OF_CELL_ACTIVATION	GOBP_POSITIVE_REGULATION_OF_CELL_ACTIVATION	77	0.336793603	2.481661171	7.95E-06	0.000341542	0.000258892	674	tags=45%, list=21%, signal=37%	TYK2/EFNB1/PYCARD/IGF2/XBP1/HLA-DMB/HMGB1/TYROBP/ABL1/HAVCR2/AIF1/HLA-DRB3/GLI2/HLA-DQB1/PPP3CA/HLA-E/HLA-F/HLA-DRB5/PTPRC/CEBPA/HLA-DMA/HLA-A/FLOT2/CBFB/CCL21/IL15/CD177/HLA-DOB/CCL5/CHRNB2/BCL2/BTN2A2/MMP14/CTSC/LYN
GOBP_POSITIVE_REGULATION_OF_LYMPHOCYTE_ACTIVATION	GOBP_POSITIVE_REGULATION_OF_LYMPHOCYTE_ACTIVATION	GOBP_POSITIVE_REGULATION_OF_LYMPHOCYTE_ACTIVATION	66	0.368030944	2.590293386	8.29E-06	0.000352918	0.000267515	674	tags=48%, list=21%, signal=39%	TYK2/EFNB1/PYCARD/IGF2/XBP1/HLA-DMB/HMGB1/TYROBP/ABL1/HAVCR2/AIF1/HLA-DRB3/GLI2/HLA-DQB1/PPP3CA/HLA-E/HLA-F/HLA-DRB5/PTPRC/HLA-DMA/HLA-A/FLOT2/CBFB/CCL

											21/IL15/HLA-DOB/CCL5/CHRNB2/BCL2/BTN2A2/MMP14/LYN
GOBP_CELL_CELL_ADHESION	GOBP_CELL_CELL_ADHESION	GOBP_CELL_CELL_ADHESION	191	0.241793096	2.274106758	8.71E-06	0.000367632	0.000278668	1536	tags=66%, list=49%, signal=36%	SHC1/TYK2/MSN/LAG3/PCDHGA8/PARVA/EFNB1/CDH11/PYCARD/IGF2/CEBPB/XBP1/HLA-DMB/HMGB1/ABL1/HAVCR2/AIF1/HLA-DRB3/CYP1B1/GLI2/HLA-DQB1/PPP3CA/ST3GAL4/CLIC1/PERP/CD34/HLA-E/ILK/HLA-DRB5/PTPRC/TRO/FXYD5/ITGB1/HLA-DMA/HLA-A/FLOT2/BMP4/ALOX5/CLDN15/CITED2/CBFB/MAGI1/CCL21/IL15/PCDHB16/ITGA9/CD177/ITGB7/IRAK1/MYADM/BMP2/ROBO3/FGL2/PCDHB14/HLA-DOB/CCL5/BCL2/BTN2A2/PODXL/HAS2/AMIGO2/PTPN2/LYN/SCGB1A1/RELA/CLDN5/F2RL3/CXCL13/ITGA3/CDH3/ITGA5/LRG1/CD276/PCDHB4/NFASC/HSPB1/AGER/SERPINE2/PKP2/BAD/CD4/SPN/LRFN3/ITGAM/CD83/CDH4/MINK1/SLITRK2/PRKAR1A/TNFSF13B/PAG1/CLDN4/SOCS1/CDH9/TNFRSF21/SMAD7/ETS1/ADIPOQ/SERPINB8/TNXB/CX3CR1/ZNF703/CSK/PSG2/ADA/CLSTN3/TMEM47/THY1/IGSF21/SELPLG/PDGFRA/NOTCH4/AKT1/DLG4/ZBTB16/WNT3A/F11R/DAB1/TSPAN32/PTPN23/WNT1/LIMS2/CD6/COL8A2/ARID1B/VEZT
GOBP_FERTILIZATION	GOBP_FERTILIZATION	GOBP_FERTILIZATION	28	-0.492390142	-2.864278983	9.13E-06	0.000381705	0.000289335	259	tags=46%, list=8%, signal=43%	LYZL6/TDRKH/SPACA3/ACTL7A/AKAP4/KLHL10/ACRBP/IQCF1/SPA17/HSPA1L/ZPBP2/CATSPER2/SPAG8
HP_INFERTILITY	HP_INFERTILITY	HP_INFERTILITY	32	-0.464624778	-2.787903704	9.80E-06	0.00040616	0.000307873	388	tags=50%, list=12%, signal=44%	BLM/DNAI1/TRIP13/IFT74/DZIP1/STAG3/DNAH17/FKBP6/DNAH8/AURKC/KLHL10/DNALI1/ZMYND10/FOXJ1/CATSPER2/TTC21A
GOBP_REACTIVE_OXYGEN_SPECIES_METABOLIC_PROCESS	GOBP_REACTIVE_OXYGEN_SPECIES_METABOLIC_PROCESS	GOBP_REACTIVE_OXYGEN_SPECIES_METABOLIC_PROCESS	59	0.379649207	2.57905926	1.01E-05	0.000413239	0.000313239	1022	tags=61%, list=32%, signal=42%	SH3PXD2A/AKR1C1/SH3PXD2B/CBR1/PDGFRB/MAOB/TYROBP/H19/VDAC1/CYP1B1/CAT/TSPO/LIPA/RAC1/GADD45A/HP/NFE2L2/ABCB7/NQO1/ALOX5/CD177/PRDX1/CCS/MPV17/BCL2/ARF4/CBS/PRDX3/IER3/SIRT2/IFI6/ACOX1/INS/ITGAM/NOXA1/DUOX2
GOBP_RESPONSE	GOBP_RESPONSE_	GOBP_RESPONSE_TO_O	176	0.252026662	2.335947291	1.06E-05	0.00043007	0.000326	1347	tags=60%,	NFKB1/CTSK/MSN/KLF9/TOMM20/CD

_TO_ORGANIC_CYCLIC_COMPOUND	TO_ORGANIC_CYCLIC_COMPOUND	RGANIC_CYCLIC_COMPOUND					4			list=43%, signal=36%	K4/TFPI/PHB2/DAB2/IGFBP4/MAOB/PQBP1/ABL1/SMO/CYP1B1/GLB1/CAT/SOCS2/BTG2/TSPO/ATP1A2/FOLR2/LIPA/STAT3/INHBA/MBD2/PTPRC/CEBPA/HAND2/NQO1/SNAI2/FECH/CDK5/BCL2L1/PRMT2/PALM/BMP2/TEK/ZBTB7A/ALAD/CHRNB2/BCL2L2/BCL2/HRH3/AANAT/RAPGEF1/MMP14/CBS/LYN/GPR83/TRPM2/SCGB1A1/RELA/BCHE/FAM107A/NR3C1/SIRT2/YAP1/GNAI1/CCR5/FOSL2/ADM/NCOA3/GCLC/ARPC1B/MDM2/KLF2/PMVK/UCN3/SLC26A5/ACSL1/CD83/DUOX2/FGF19/ROCK2/GNB1/MYOG/VPS11/ARRB2/NPAS4/MMP15/ALAS1/CLDN4/DDX54/PITX3/ADIPOQ/DIAPH1/ATF2/GRAMD1A/GHSR/SLC6A1/ZNF703/UBE2L3/AQP1/EGR1/ASCL1/HTR1B/ADA/TOP2B/ELK1/VPS18/TRPV1/P2RX2/HCN4/SSTR3
GOBP_CELLULAR_RESPONSE_TO_OXYGEN_CONTAINING_COMPOUND	GOBP_CELLULAR_RESPONSE_TO_OXYGEN_CONTAINING_COMPOUND	GOBP_CELLULAR_RESPONSE_TO_OXYGEN_CONTAINING_COMPOUND	244	0.223431306	2.209404182	1.20E-05	0.000486384	0.000368683	1428	tags=60%, list=45%, signal=35%	SHC1/FBN1/NFKB1/TYK2/CASP4/MSN/AKR1A1/KLF9/AKR1C1/CDK4/PHB2/PYCARD/RGS10/IGF2/CEBPB/XBP1/IQGAP1/HMGB1/ABL1/SMO/HAVCR2/H19/CYP1B1/ALOX5AP/ITPR1/NADK/CAT/PPP3CA/SLC25A23/SOCS2/TSPO/FOLR2/COL3A1/STAT3/INHBA/CEBPA/NFE2L2/HAND2/CYFIP1/COL4A6/MAP3K5/SLC29A1/NQO1/ADD1/SNAI2/FECH/MAS1L/ADAM15/CDK5/IRAK1/BCL2L1/KCNB1/PRDX1/PALM/CCS/MPV17/CHRNB2/CYP11A1/BCL2L2/LY86/HRH3/AANAT/CPNE3/RAPGEF1/SRI/PTPN2/FZD4/DENND4C/LYN/SYT12/TRPM2/KMO/RELA/COL4A1/NR3C1/CXCL13/PRDX3/SIRT2/YAP1/ANKZF1/GNAI1/CCR5/TRIM41/WNT11/ERN1/NCOA3/GCLC/AGER/NET1/GPR21/SQSTM1/MDM2/KLF2/NFKBIL1/CRHR2/BAD/OSBP/UBTF/MAP2K3/RRAGA/PPIF/INS/OTOP1/PDK2/GRB7/PRKAR1A/ROCK2/GNB1/MYOG/ARRB2/NPAS4/NFATC4/SOCS1/ADIPOQ/ACTN2/GRAMD1A/GHSR/CX3CR1/ZNF

											703/AQP1/EGR1/ADIPOR1/CSK/HTR1B/TOP2B/UMODL1/JAGN1/ELK1/FFAR2/JUND/TRPV1/FXN/HCN4/SSTR3/CSHL1/PDGFRA/AKT1/DLG4/SH2B2/MT3/WNT3A/CA2/CDK2/RARG/LTF/HCN3
HP_REDUCED_SPERM_MOTILITY	HP_REDUCED_SPERM_MOTILITY	HP_REDUCED_SPERM_MOTILITY	11	-0.724512416	-2.73650517	1.25E-05	0.000501445	0.0003801	154	tags=64%, list=5%, signal=61%	DNAH17/DNAH8/KLHL10/DNALI1/ZMYND10/CATSPER2/TTC21A
GOBP_MEIOSIS_I_CELL_CYCLE_PROCESS	GOBP_MEIOSIS_I_CELL_CYCLE_PROCESS	GOBP_MEIOSIS_I_CELL_CYCLE_PROCESS	21	-0.549080314	-2.822670866	1.30E-05	0.000515456	0.00039072	466	tags=57%, list=15%, signal=49%	ESPL1/TRIP13/PIWIL2/STAG3/RAD51/CKS2/HSPA2/CDC25C/MYBL1/CCNB2/PTTG1/PTTG2
GOBP_BIOLOGICAL_PROCESS_INVOLVED_IN_INTERSPECIES_INTERACTION_BETWEEN_ORGANISMS	GOBP_BIOLOGICAL_PROCESS_INVOLVED_IN_INTERSPECIES_INTERACTION_BETWEEN_ORGANISMS	GOBP_BIOLOGICAL_PROCESS_INVOLVED_IN_INTERSPECIES_INTERACTION_BETWEEN_ORGANISMS	298	0.204652697	2.096879728	1.31E-05	0.000515456	0.00039072	1427	tags=58%, list=45%, signal=35%	SHC1/FUCA2/NFKB1/TYK2/CASP4/LAG3/CDK4/IFITM3/PHB2/LSM14A/SCARB2/MAOB/PYCARD/IFIT3/CEBPB/XBP1/PQBP1/HMGB1/IFNGR1/ZMPSTE24/TYROBP/ABL1/EIF2AK4/IFITM1/HAVCR2/H19/AIF1/RAB1A/EIF2AK2/TRIM8/MAPKAPK3/PLSCR1/TSPO/C1QC/PUM1/HLA-B/HLA-E/HLA-F/STAB1/PTPRC/TPCN1/HP/NFE2L2/ADAMTS5/ITGB1/VAPA/TFEB/HLA-A/IFI44/FLOT2/MAP3K5/HLA-C/NQO1/CFHR5/MUC2/NMI/FMO1/IGF2R/CCL21/COLEC11/RNF185/IL15/CYLD/CD177/ADAM15/ITGB7/IRAK1/BCL2L1/PRDX1/BMP2/ZDHHC9/FGL2/CCL5/ALAD/SLC11A1/ISG15/LY86/BCL2/MEFV/TRIM4/PTPN2/UVRAG/LYN/KMO/SCGB1A1/FCER1G/BRCC3/RELA/PI3/ZBED1/HERC6/CXCL13/PRDX3/IER3/OCIAD2/SIRT2/GPR15/KIR2DL4/ITGA5/STOM/CCR5/LRG1/FOSL2/RAB9A/IL17A/ADM/TRIM41/C8A/IFI6/HSPB1/CHMP2B/BATF2/ZNFX1/RNF5/NFKBIL1/NRP1/NR1H2/MAP2K3/INS/CD4/BAX/OTOP1/TRAFD1/ZFYVE1/SPN/ITGAM/OAS2/MMRN2/DUOX2/CHMP4C/FBXO9/FGF19/ITGB8/ROCK2/DDX3X/MPEG1/ARRB2/CCL8/DEFB105A/VWCE/ADIPOQ/CHMP6/CHMP7/VPS4A/TRIM44/PJA2/ATF2/GHSR/CX3CR1/AQP1/REG4/UNC93B1/JAGN1/LTA/TBC1D20/VPS18/MFHAS1/FFAR2/CCL22/ANG/SELPLG/TNFRSF4/U

GOBP_CARDIAC_CHAMBER_MORPHOGENESIS	GOBP_CARDIAC_CHAMBER_MORPHOGENESIS	GOBP_CARDIAC_CHAMBER_MORPHOGENESIS	27	0.519741632	2.655440054	1.36E-05	0.000531945	0.000403219	919	tags=70%, list=29%, signal=50%	BL7/AKT1/S100A12/FKBP5/TAP2/COTL1/C3/S100A14/F11R/ATG5/LTF PARVA/SMO/HEG1/HAND2/FKBP1A/BMP4/NKX2-5/CITED2/ZFPM2/BMP2/TEK/FOXC1/NRP2/ENG/WNT11/ADPRHL1/MDM2/PKP2/NRP1
GOCC_MITOTIC_SPINDLE	GOCC_MITOTIC_SPINDLE	GOCC_MITOTIC_SPINDLE	42	-0.413292489	-2.764046505	1.42E-05	0.000546102	0.00041395	647	tags=52%, list=21%, signal=42%	TUBB/CDK5RAP2/KIF22/CDC7/CCDC117/IQCB1/SMC3/ESPL1/TPX2/SMC6/KIF23/TUBG1/CENPE/EFHC1/TPR/CDC14B/KATNA1/SPAG5/RAE1/DR1/MAPKBP1/WDR62
GOBP_ACROSOME_ASSEMBLY	GOBP_ACROSOME_ASSEMBLY	GOBP_ACROSOME_ASSEMBLY	10	-0.725422245	-2.682715979	1.43E-05	0.000546102	0.00041395	158	tags=70%, list=5%, signal=67%	ACTL7A/CCDC42/ACRBP/ZPBP2/PAFAH1B1/RFX2/TBPL1
GOMF_PEPTIDE_ANTIGEN_BINDING	GOMF_PEPTIDE_ANTIGEN_BINDING	GOMF_PEPTIDE_ANTIGEN_BINDING	17	0.599467637	2.571179059	1.42E-05	0.000546102	0.00041395	532	tags=71%, list=17%, signal=59%	HLA-DMB/HLA-DRB3/HLA-DQB1/HLA-B/HLA-E/HLA-F/HLA-DRB5/HLA-DMA/HLA-A/HLA-C/DHCR24/HLA-DOB
GOBP_HEART_DEVELOPMENT	GOBP_HEART_DEVELOPMENT	GOBP_HEART_DEVELOPMENT	134	0.2699627	2.30781219	1.48E-05	0.000559448	0.000424066	1418	tags=64%, list=45%, signal=37%	SHC1/FBN1/PARVA/SH3PXD2B/PDGFRB/GALNT11/CDH11/MED12/ZMPSTE24/ABL1/EMP2/SMO/VEGFB/GLI2/COL3A1/INHBA/ILK/MBD2/HEG1/HAND2/FKBP1A/ADAMTS5/ITGB1/BMP4/NKX2-5/MTHFD1/CITED2/ZFPM2/SNAI2/TSC1/ADAM15/RAMP2/BMP2/TEK/FOXC1/ELN/MEF2D/HAS2/NRP2/SRI/ID2/MAML1/CLDN5/YAP1/ITGA3/APLN/ENG/FRS2/EOMES/ADM/WNT11/ADPRHL1/MIXL1/VGLL4/MDM2/PKP2/NRP1/MAP2K3/DVL2/FGF19/PRKAR1A/ROCK2/ARRB2/NFATC4/MEF2B/SNX17/PDLIM3/SMAD7/PDLIM2/ACTN2/ATF2/CRIP1/SYPL2/LMO4/ASCL1/DLC1/EPOR/PCSK5/TBX6/HCN4/PDGFRA/NPHP3/SEC24B/CAV3/WNT3A/ATG5
GOBP_MEIOTIC_CELL_CYCLE	GOBP_MEIOTIC_CELL_CYCLE	GOBP_MEIOTIC_CELL_CYCLE	51	-0.37516714	-2.658462577	1.50E-05	0.000564732	0.000428071	520	tags=43%, list=16%, signal=37%	SMC3/TUBG2/BOLL/ESPL1/ANAPC10/TRIP13/PIWIL2/TUBG1/SMC1B/TDRKH/FZR1/STAG3/RAD51/FKBP6/CKS2/AURKC/HSPA2/CDC25C/MYBL1/CCNB2/PTTG1/PTTG2
GOBP_RECOMBINATIONAL_REPAIR	GOBP_RECOMBINATIONAL_REPAIR	GOBP_RECOMBINATIONAL_REPAIR	28	-0.479581139	-2.789767831	1.57E-05	0.000582059	0.000441206	983	tags=75%, list=31%, signal=52%	RIF1/LIG3/SENP3/RMI1/PPP4C/PARP1/TIMELESS/MCM5/TP53BP1/TOP3A/WRN/CDC7/MAD2L2/BLM/RNF138/SMC6/

										RUVBL1/RECQL4/UBE2N/RAD51/SMCHD1
GOBP_POSITIVE_REGULATION_OF_CELL_CELL_ADHESION	GOBP_POSITIVE_REGULATION_OF_CELL_CELL_ADHESION	GOBP_POSITIVE_REGULATION_OF_CELL_CELL_ADHESION	74	0.344194492	2.501245811	1.57E-05	0.000582059	0.000441206	611	tags=45%, list=19%, signal=37%	TYK2/EFNB1/PYCARD/IGF2/XBP1/HLA-DMB/HMGB1/ABL1/HAVCR2/AIF1/HLA-DRB3/GLI2/HLA-DQB1/PPP3CA/ST3GAL4/HLA-E/HLA-DRB5/PTPRC/HLA-DMA/HLA-A/FLOT2/ALOX5/CITED2/CBFB/MAGI1/CCL21/IL15/IRAK1/HLA-DOB/CCL5/BTN2A2/PODXL/HAS2
GOBP_REGULATION_OF_IMMUNE_RESPONSE	GOBP_REGULATION_OF_IMMUNE_RESPONSE	GOBP_REGULATION_OF_IMMUNE_RESPONSE	177	0.24826714	2.301490936	1.61E-05	0.000592679	0.000449256	1011	tags=49%, list=32%, signal=35%	NFKB1/TYK2/LAG3/PHB2/LSM14A/RHBDF2/PYCARD/XBP1/PQBP1/HLA-DMB/HMGB1/TYROBP/ABL1/EIF2AK4/HAVCR2/CFHR3/HLA-DRB3/EIF2AK2/HLA-DQB1/MAPKAPK3/PLSCR1/COL3A1/LIPA/C1QC/PUM1/HLA-B/HLA-E/HLA-F/HLA-DRB5/PTPRC/C3AR1/NFE2L2/FKBP1A/BTNL2/HLA-DMA/HLA-A/FLOT2/HLA-C/CFHR5/NMI/PRKCH/COLEC11/RNF185/IL15/CYLD/CD177/IRAK1/FGL2/HLA-DOB/CCL5/SLC11A1/CTSH/ISG15/BCL2/MEFV/BTN2A2/PTPN2/SVEP1/LYN/EIF2B1/FCER1G/BRCC3/RELA/CXCL13/BTN1A1/SIRT2/KIR2DL4/FOSL2/CD276/IL17A/TRIM41/C8A/AGER/ZNFX1/NFKBIL1/NR1H2/INPPL1/INS/LIME1/CD4/BAX/OTOP1/TRAFD1/SPN/ITGAM/CD1A
GOCC_TRANSFERASE_COMPLEX	GOCC_TRANSFERASE_COMPLEX	GOCC_TRANSFERASE_COMPLEX	195	-0.212878601	-2.263652693	1.75E-05	0.00063274	0.000479622	797	tags=42%, list=25%, signal=33%	ANKRD9/RANBP2/RCHY1/UBE2C/CAB39/ATG12/PIGP/CDK5R1/BCCIP/ERCC3/TAF9/SETD1A/PIK3CG/RMND5A/TAF6/GTF2E1/DCUN1D3/TAF10/PRKAG2/DCUN1D5/RNF7/KLHL7/POLR3E/TRAF2/GTF2H1/DDA1/TBK1/GTF2H2/KRTCAP2/DERL3/SHARPIN/POLE/SUZ12/BARD1/MAD2L2/PCNA/KCTD10/ANAPC10/KBTBD8/PCGF6/CACYBP/PRIM1/TEX10/POLRMT/CDK7/FBXL2/SMC6/PAXIP1/RUVBL1/POLA2/UBE2D2/UBE2D3/PAF1/RMND5B/FBXO39/UBE2N/BRD1/UBE2S/FBXO7/BUB1B/FZR1/WDR26/POLG2/ASB1/SUGT1/FBXL18/DERL2/CSNK2A2/TSSK2/RAD51/PRKAA1/FBXO15/CKS

										2/AKAP4/PARD6A/CCNH/SUPT3H/FBXO24/CCNB2/DR1/TBPL1
GOBP_POSITIVE_REGULATION_OF_GENE_EXPRESSION	GOBP_POSITIVE_REGULATION_OF_GENE_EXPRESSION	GOBP_POSITIVE_REGULATION_OF_GENE_EXPRESSION	216	0.22882429	2.21480711	1.75E-05	0.00063274	0.000479622	856	tags=41%, list=27%, signal=32%	TYK2/MSN/MAF/SH3BGRL/LRP3/PHB2/LARP6/PYCARD/CEBPB/XBP1/PQBP1/HMGB1/IFNGR1/ZMPSTE24/TYROBP/ABL1/EIF2AK4/SMO/C14orf93/HAVCR2/H19/AIF1/CYP1B1/RAB1A/EIF2AK2/PPP3CA/PLSCR1/STAT3/CD34/HLA-E/INHBA/RBMS3/HLA-F/CLNS1A/HEG1/MYC/PTPRC/CEBPA/RPS27L/C3AR1/NFE2L2/HAND2/EIF4G1/SULF1/HLA-A/BMP4/ATF4/NKX2-5/NAT8/LUM/CITED2/IL15/IRAK1/RAMP2/BMP2/CTCF/EDA/CCL5/FOXC1/SLC11A1/CTSH/ISG15/MEFV/AIRE/PHF2/ID2/PLAG1/MMP14/MRPS27/MAFG/FCER1G/RELA/CLDN5/TRAF5/LARP4/TRUB2/YAP1/ITGA3/KIR2DL4/CDH3/FGF1/ENG/CD276/IL17A/WNT11/TMEM119/HSPB1/ERN1/AGER
GOBP_DEFENSE_RESPONSE	GOBP_DEFENSE_RESPONSE	GOBP_DEFENSE_RESPONSE	305	0.20393507	2.100122229	1.79E-05	0.000641313	0.000486121	1427	tags=57%, list=45%, signal=35%	SHC1/NFKB1/TYK2/CASP4/LAG3/IFITM3/PHB2/LSM14A/RHBDF2/PYCARD/IFIT3/CEBPB/PQBP1/LGALS3BP/HMGB1/IFNGR1/ZMPSTE24/TYROBP/EIF2AK4/IFITM1/SMO/HAVCR2/H19/VDAC1/AIF1/RAB1A/ALOX5AP/EIF2AK2/EIF2AK1/TRIM8/MAPKAPK3/PLSCR1/ATP1A2/FOLR2/LIPA/STAT3/RAC1/C1QC/PUM1/HLA-B/HLA-E/INHBA/RBMS3/HLA-F/LSP1/STAB1/PTPRC/CEBPA/HP/C3AR1/NFE2L2/EIF4G1/ADAMTS5/ITGB1/TFEB/HLA-A/FLOT2/MAP3K5/HLA-C/PTN/NQO1/CFHR5/ALOX5/NMI/ABHD12/CCL21/COLEC11/PTGES/RNF185/IL15/CYLD/CD177/ADAM15/IRAK1/BCL2L1/PRDX1/BMP2/TEK/FGL2/PARP4/CCL5/SLC11A1/ISG15/LY86/BCL2/MEFV/TRIM4/PTPN2/CTSC/LYN/SCGB1A1/FCER1G/BRCC3/RELA/PI3/AOC3/NR3C1/CXCL13/IER3/SIRT2/PLA2G4C/KIR2DL4/APOD/CCR5/SERPINA1/KIR2DS3/FOSL2/IL17A/ADM/TRIM41/C8A/IFI6/AGER/CXCR3/BATF2/ZNFX1/NFKBIL1/CCR1/NR

											1H2/MAP2K3/INS/CD4/OTOP1/TRAFD1/SPN/ITGAM/OAS2/MMRN2/CD83/DUOX2/FBXO9/DDX3X/AGR2/MPEG1/TAP1/ARRB2/NFATC4/CCL8/ETS1/DEFB105A/GNG7/ADIPOQ/ABI3/TRIM44/PJA2/CUEDC2/GHSR/CX3CR1/AQP1/ADA/UNC93B1/JAGN1/LTA/PXK/TIMP1/MFHAS1/XCR1/FFAR2/TRPV1/CCL22/ANG/CHIA/TNFRSF4/ABCD1/UBL7/AKT1/S100A12/COTL1/C3/GPSM3/F8/S100A14/F11R/ATG5/LTF
GOBP_REGULATION_OF_CELL_CELL_ADHESION	GOBP_REGULATION_OF_CELL_CELL_ADHESION	GOBP_REGULATION_OF_CELL_CELL_ADHESION	106	0.286994313	2.307807704	1.82E-05	0.000648775	0.000491777	1171	tags=58%, list=37%, signal=38%	TYK2/LAG3/EFNB1/PYCARD/IGF2/CEBPB/XBP1/HLA-DMB/HMGB1/ABL1/HAVCR2/AIF1/HLA-DRB3/GLI2/HLA-DQB1/PPP3CA/ST3GAL4/HLA-E/HLA-DRB5/PTPRC/FXYD5/HLA-DMA/HLA-A/FLOT2/BMP4/ALOX5/CITED2/CBFB/MAGI1/CCL21/IL15/IRAK1/MYADM/BMP2/FGL2/HLA-DOB/CCL5/BTN2A2/PODXL/HAS2/PTPN2/LYN/SCGB1A1/RELA/CXCL13/CD276/AGER/SERPINE2/BAD/CD4/SPN/LRFN3/CD83/MINK1/PRKAR1A/TNFSF13B/PAG1/SOCS1/TNFRSF21/SMAD7/ETS1/ADIPOQ
GOBP_DNA_REPLICATION	GOBP_DNA_REPLICATION	GOBP_DNA_REPLICATION	65	-0.337599192	-2.609071303	1.90E-05	0.000671809	0.000509237	954	tags=62%, list=30%, signal=44%	MCRS1/LIG3/RBBP6/HMGA1/RFC1/RMI1/ENDOG/POLI/PARP1/ATR/TIMELESS/MCM5/RAD50/WRN/TTF1/BAZ1A/CDC7/TOP1MT/SMC3/POLE/BARD1/PCNA/PRIM1/POLRMT/USP37/BLM/RUVBL1/POLA2/RECQL4/DONSON/CHAF1B/RFC4/POLG2/GTPBP4/FAF1/TRAIP/POLB/RAD51/GMNN/RAD17
GOBP_CYTOKINE_PRODUCTION	GOBP_CYTOKINE_PRODUCTION	GOBP_CYTOKINE_PRODUCTION	156	0.254399148	2.290642477	2.07E-05	0.000728142	0.000551938	1427	tags=63%, list=45%, signal=37%	NFKB1/TYK2/LAG3/PYCARD/CEBPB/XBP1/PQBP1/IQGAP1/HMGB1/IFNGR1/TYROBP/ABL1/HAVCR2/H19/AIF1/CYP1B1/RAB1A/EIF2AK2/TSPO/COL3A1/STAT3/RAC1/CD34/HLA-B/HLA-E/INHBA/HLA-F/HEG1/PTPRC/C3AR1/BTNL2/SULF1/HLA-A/ATF4/ANXA4/LUM/ALOX5/NMI/SNAI2/IL15/CYLD/IRAK1/SLC11A1/ISG15/MEFV/AIRE/BTN2A2/LYN/SCGB1A1/FCER1G/RELA/BTN1A1/LTBP

										1/KIR2DL4/CDH3/APOD/CD276/IL17A/WNT11/HSPB1/AGER/KLF2/NFKBIL1/MAP2K3/INS/CD4/SPN/OAS2/CD83/ITGB8/ROCK2/DDX3X/ARRB2/NFATC4/SOCS1/TNFRSF21/SMAD7/ADIPOQ/CUEDC2/ATF2/TNXB/GHSR/CX3CR1/EGR1/CSK/UNC93B1/PCSK5/LTA/FFAR2/CHIA/ABCD1/CCBE1/C3/GPSM3/WNT3A/LTB/F11R/ATG5/LTF
GOBP_RESPONSE_TO_XENOBIOTIC_STIMULUS	GOBP_RESPONSE_TO_XENOBIOTIC_STIMULUS	GOBP_RESPONSE_TO_XENOBIOTIC_STIMULUS	83	0.323580181	2.425460426	2.17E-05	0.000756287	0.000573272	965	tags=53%, list=31%, signal=38%	AKR1C1/CDK4/CBR1/MAOB/CBR3/ABL1/GSTO1/CYP1B1/CAT/TSPO/LIPA/CYP2U1/MYC/NFE2L2/SLC29A1/NQO1/FMO1/ACAA1/BPHL/FECH/CYP2W1/PARP4/ALAD/BCL2/LYN/UBE2B/SCGB1A1/RELA/BCHE/SLC29A3/GNA12/NNMT/ITGA3/CDH3/CYP2S1/ENG/FOSL2/NAT2/ADD3/GCLC/MDM2/SLC26A5/ACSL1/ABCB1
GOCC_CONDENSED_CHROMOSOME_CENTROMERIC_REGION	GOCC_CONDENSED_CHROMOSOME_CENTROMERIC_REGION	GOCC_CONDENSED_CHROMOSOME_CENTROMERIC_REGION	37	-0.418463655	-2.673251365	2.56E-05	0.000887042	0.000672386	708	tags=57%, list=22%, signal=45%	TP53BP1/ZW10/SMARCC1/KIF22/PINX1/BRD7/DYNC1LI1/CENPH/ITGB3BP/NDE1/PPP1CC/CENPE/BUB1B/SUGT1/C1orf112/CENPM/TPR/AURKC/SPAG5/PAFAH1B1/AHCTF1
GOBP_CILIUM_OR_FLAGELLUM_DEPENDENT_CELL_MOTILITY	GOBP_CILIUM_OR_FLAGELLUM_DEPENDENT_CELL_MOTILITY	GOBP_CILIUM_OR_FLAGELLUM_DEPENDENT_CELL_MOTILITY	19	-0.568575986	-2.783722547	2.73E-05	0.000938407	0.00071132	382	tags=74%, list=12%, signal=65%	MKKS/BBS4/IQCG/DNAI1/DZIP1/EFHC1/DNAH17/AKAP4/DNAH8/IQCF1/ROPN1L/LZTFL1/CATSPER2/TTC21A
GOBP_RESPONSE_TO_TOXIC_SUBSTANCE	GOBP_RESPONSE_TO_TOXIC_SUBSTANCE	GOBP_RESPONSE_TO_TOXIC_SUBSTANCE	54	0.374374626	2.441634871	2.77E-05	0.000943865	0.000715457	1024	tags=61%, list=32%, signal=42%	AKR1A1/MAOB/GSTO1/LTC4S/H19/CYP1B1/ALOX5AP/CAT/HP/NFE2L2/ATF4/NQO1/MUC2/BPHL/MT1B/PTGES/FECH/MT1E/PRDX1/CCS/CCL5/ALAD/SLC11A1/BCL2/MT1H/LYN/PRDX3/SLC22A8/MDM2/ABCB1/BAX/DUOX2/EHMT1
GOBP_TRANSMEMBRANE_TRANSPORT	GOBP_TRANSMEMBRANE_TRANSPORT	GOBP_TRANSMEMBRANE_TRANSPORT	280	0.212850775	2.158130398	2.81E-05	0.00095077	0.000720691	1574	tags=64%, list=50%, signal=35%	LASP1/COX7A1/SLC44A1/TOMM20/CYBRD1/PHB2/ABL1/GSTO1/COX7B/VDAC1/COX8A/ITPR1/TMCO3/PPP3CA/SLC25A23/CLIC1/CLCC1/SLC25A5/ATP1A2/ACSL5/PTPRC/TPCN1/SLC30A9/NFE2L2/SCARA5/CCDC51/FXYD5/FKBP1A/ITGB1/SLC35D2/STEAP3/ABCB7/ANXA6/SLC29A1/SLC25A12/PRAF2/KCNK5/SLC12A9/KCNE3/CCL21/GJA4/RNF185/TS

											C1/MFSD1/CLN3/TMEM109/KCNB1/SLC25A37/OTOP3/SLC11A1/MPV17/CHRNB2/SLC2A13/SLC34A2/SLC9A8/SLC26A1/SLC16A9/GRIA4/BCL2/SLC31A1/SRI/IBTK/CNIH3/LYN/TRPM2/ATP2A3/SLC25A20/GRIK5/F2RL3/SLC29A3/COX15/GRM1/SLC35B2/SEC61A1/STOM/SLC16A11/CCR5/SLC6A17/ATP8B1/SLC14A2/SLC7A14/TMEM63A/SLC22A8/RNF5/SLC29A2/CLIC5/CALM3/SLC26A5/PPIF/INS/LIME1/ACSL1/TIMM17B/ABCB1/BAX/OTOP1/SLC22A11/KCNH4/FHL1/GABRR1/SEC63/SLC24A3/CNNM2/ABCD4/OTOP2/ABCC10/MINK1/FGF19/KCNB2/KCNJ1/NIPA2/FGF13/CABP5/TAP1/CLDN4/SLC10A5/ADIPOQ/KCNAB2/DIAPH1/SFXN3/ACTN2/SLC35A1/SLC16A12/ATP6V1G2/SLC25A36/SLC6A1/SLC35A4/AQP1/AQP6/RALBP1/KCNJ6/KCNH7/CHRNA2/SLC5A7/XCR1/TRPV1/FXN/P2RX2/SLC22A7/THY1/HCN4/ABCD1/AKT1/TAP2/DLG4/OXSR1/SLC22A2/C3/CAV3/WNT3A/CLCN6/CA2/ATG5/CACNA2D3/LTF/HCN3/ATP13A1/PLA2G1B/SLC32A1/GLP1R/ATP6V0A1/CACNA1B/KCNQ3/BCR/GJC1/SLC34A3/KCNS2/CACNA1E/CLN8/SLC12A8/SLC25A13/ATP1B1/SLC12A3/CACNB3/SLC17A7/ATP1A3/GRM6/ABCG2/ATP2A1
GOBP_MEIOTIC_CELL_CYCLE_PROCESS	GOBP_MEIOTIC_CELL_CYCLE_PROCESS	GOBP_MEIOTIC_CELL_CYCLE_PROCESS	33	-0.447051397	-2.730533897	2.86E-05	0.00096048	0.000728052	498	tags=48%, list=16%, signal=41%	TUBG2/ESPL1/TRIP13/PIWIL2/TUBG1/TDRKH/FZR1/STAG3/RAD51/CKS2/HSPA2/CDC25C/MYBL1/CCNB2/PTTG1/PTTG2
GOMF_INTEGRIN_BINDING	GOMF_INTEGRIN_BINDING	GOMF_INTEGRIN_BINDING	35	0.459182617	2.575319191	2.97E-05	0.000992621	0.000752415	791	tags=66%, list=25%, signal=50%	FBN1/IGF2/HMGB1/EMP2/TGFBI/COL3A1/ITGB1BP1/ADAMTS5/ITGB1/ANXA7/PTN/ITGA9/CD177/ADAM15/ITGB7/VWF/ISG15/PTPN2/MMP14/LYN/ITGA3/ITGA5/FGF1
GOCC_EXTERNAL_SIDE_OF_PLASMA_MEMBRANE	GOCC_EXTERNAL_SIDE_OF_PLASMA_MEMBRANE	GOCC_EXTERNAL_SIDE_OF_PLASMA_MEMBRANE	77	0.322701639	2.377824634	3.08E-05	0.00102098	0.000773912	1719	tags=83%, list=55%, signal=39%	CTSK/LAG3/F10/CD248/FOLR2/CD34/HLA-B/HLA-E/HLA-F/HEG1/PTPRC/ITGB1/BTNL2/HLA-A/MAP3K5/HLA-C/COLEC11/ITGA9/CHRNB2/BTN2A2/PLAU/F

											CER1G/BTN1A1/ITGA3/ITGA5/CCR5/GFRA3/ENG/CD276/IL17A/CXCR3/CCR1/CD4/ABCB1/SPN/SLC22A11/ITGAM/CLEC14A/CD1A/CD83/CX3CR1/CD1B/ADA/EPOR/UMODL1/XCR1/TRPV1/CD79A/THY1/IGSF21/TNFRSF4/PDGFRA/IL9R/BCAM/MICA/ABCG2/IL1R1/MCAM/CD209/IL2RG/IL13RA1/TAS2R16/CLEC2D/IL21R
GOBP_MITOTIC_CELL_CYCLE	GOBP_MITOTIC_CELL_CYCLE	GOBP_MITOTIC_CELL_CYCLE	198	-0.202103514	-2.157988162	3.12E-05	0.001026095	0.000777789	768	tags=39%, list=24%, signal=32%	BBS4/BCCIP/CIB1/SIRT1/ERCC3/RAD50/ZW10/SMARCC1/DCUN1D3/TAF10/AKAP8L/TUBB/E2F1/USP8/CDK5RAP2/KIF22/KIF4A/TFDP1/ECD/CDC7/SMC3/MAD2L1BP/PINX1/TUBG2/POLE/BRD7/ESPL1/BARD1/MAD2L2/DYNC1LI1/PCNA/ANAPC10/KPNB1/MYH10/CENPH/CDK7/USP37/TPX2/BLM/CEP250/TRIP13/NDE1/KIF23/VRK1/PPP6C/DONSON/TUBG1/UBE2S/FBXO7/CENPE/ASNS/BUB1B/FZR1/KATNB1/MDC1/C1orf112/EFHC1/RAD51/GMNN/CKS2/TPR/AURKC/CCNH/CDCA8/HSPA2/RANBP1/CDC14B/CDC25C/MYBL1/RAD17/SPAG5/CCNB2/RAE1/AURKAIP1/PAFAH1B1/KLF11/WDR62/CPSF3
HP_DENTAL_CROWDING	HP_DENTAL_CROWDING	HP_DENTAL_CROWDING	27	0.499968118	2.554414126	3.20E-05	0.001047507	0.00079402	740	tags=63%, list=23%, signal=49%	FBN1/PLOD1/SETBP1/FGFR1/IGF2/MED12/ZMPSTE24/ABL1/H19/COL3A1/TPM2/BMP2/ZDHHC9/ZBTB7A/CBS/TRIO/LGI4
GOBP_PROTEIN_RNA_COMPLEX_ORGANIZATION	GOBP_PROTEIN_RNA_COMPLEX_ORGANIZATION	GOBP_PROTEIN_RNA_COMPLEX_ORGANIZATION	30	-0.458451511	-2.677143073	3.49E-05	0.001132621	0.000858536	349	tags=43%, list=11%, signal=39%	SF3A1/RUVBL1/GEMIN4/GEMIN6/PRPF18/ZNHIT3/TFIP11/DHX30/EIF5/SRPK1/SETX/BUD13/DDX20
GOBP_NUCLEAR_CHROMOSOME_SEGREGATION	GOBP_NUCLEAR_CHROMOSOME_SEGREGATION	GOBP_NUCLEAR_CHROMOSOME_SEGREGATION	67	-0.321379161	-2.487443808	3.51E-05	0.001134458	0.000859929	921	tags=58%, list=29%, signal=42%	MAPRE1/SMC4/CHEK2/MAP1S/LCMT1/CDC16/UBE2C/NUSAP1/BCCIP/ZW10/SMARCC1/AKAP8L/CDK5RAP2/KIF22/KIF4A/SMC3/MAD2L1BP/PINX1/TUBG2/BRD7/ESPL1/MAD2L2/DYNC1LI1/KPNB1/TPX2/TRIP13/KIF23/TUBG1/CENPE/BUB1B/KATNB1/STAG3/TPR/AURKC/CDCA8/SPAG5/CCNB2/PTTG1/PTTG2
GOBP_DNA_RECOMBINATION	GOBP_DNA_RECOMBINATION	GOBP_DNA_RECOMBINATION	51	-0.361397778	-2.560891845	3.59E-05	0.001144335	0.000867416	983	tags=65%, list=31%,	RIF1/MCRS1/LIG3/SENP3/RMI1/HMGB3/PPP4C/ENDOG/NHEJ1/PARP1/TIMEL

										signal=45%	ESS/MCM5/RAD50/TP53BP1/TOP3A/WRN/CDC7/BARD1/MAD2L2/BLM/RNF138/SMC6/PAXIP1/TRIP13/RUVBL1/RECQL4/UBE2N/POLB/RAD51/SETX/SMCHD1/TSN/KPNA2
GOCC_ANCHORING_JUNCTION	GOCC_ANCHORING_JUNCTION	GOCC_ANCHORING_JUNCTION	201	0.22093907	2.092370659	3.58E-05	0.001144335	0.000867416	1166	tags=51%, list=37%, signal=34%	SHC1/SH3PXD2A/LASP1/MSN/PARVA/CDK4/RALA/SH3PXD2B/PDGFRB/CDH11/SCARB2/DAB2/IQGAP1/TSPAN9/CAP1/FHOD1/HAVCR2/LAYN/CAT/PPP3CA/PERP/ATP1A2/TRIP6/RAI14/RAC1/ITGB1BP1/RSU1/ILK/HEG1/PTPRC/RHOU/ITGB1/VAPA/CYFIP1/FLOT2/ANXA6/ADD1/CLDN15/TRIOBP/PRKCH/IGF2R/MAGI1/GDI2/GJA4/PARVG/ADAM15/ITGB7/MYADM/TEK/PRX/LLGL1/SLC31A1/PODXL/CPNE3/ARHGEF7/PHLDB2/MMP14/FZD4/ALKBH6/LYN/LIMA1/PLAU/CLDN5/FAM107A/GNA12/SIRT2/CORO2B/YAP1/ITGA3/CDH3/ITGA5/YWHAB/ENG/ARHGAP21/FRS2/NFASC/HSPB1/ADD3/ARPC1B/PKP2/RPS15/NRP1/CLASP2/CNTNAP1/FHL1/MPZL1/RHOG/GRB7/DUOX2/CDH4/CDC42BPB/VAMP5/ITGB8/FGF13/TNFSF13B/CASK/CLDN4/PARVB/CDH9/PDLIM3/SMAD7/IQGAP3/PDLIM2
GOBP_DOUBLE_STRAND_BREAK_REPAIR	GOBP_DOUBLE_STRAND_BREAK_REPAIR	GOBP_DOUBLE_STRAND_BREAK_REPAIR	56	-0.343494436	-2.521682513	3.64E-05	0.001153067	0.000874035	948	tags=61%, list=30%, signal=43%	LIG3/SENP3/CHEK2/RMI1/VCP/PPP4C/NHEJ1/PARP1/ATR/CIB1/SIRT1/TIMELESS/MCM5/RAD50/TP53BP1/POT1/SMARCC1/TOP3A/WRN/CDC7/BRD7/MAD2L2/BLM/RNF138/SMC6/TRIP13/RUVBL1/RECQL4/UBE2N/TFIP11/POLB/RAD51/SETX/SMCHD1
GOMF_CYTOSKELETAL_MOTOR_ACTIVITY	GOMF_CYTOSKELETAL_MOTOR_ACTIVITY	GOMF_CYTOSKELETAL_MOTOR_ACTIVITY	18	-0.582351827	-2.787562441	3.76E-05	0.001181213	0.00089537	620	tags=72%, list=20%, signal=58%	KIF22/KIF4A/SMC3/MYH10/DNAI1/KIFC3/KIF23/CENPE/KIF3A/MYO9A/DNAH17/DNAH8/DYNLRB2
GOBP_ENZYME_LINKED_RECEPTOR_PROTEIN_SIGNALING_PATHWAY	GOBP_ENZYME_LINKED_RECEPTOR_PROTEIN_SIGNALING_PATHWAY	GOBP_ENZYME_LINKED_RECEPTOR_PROTEIN_SIGNALING_PATHWAY	202	0.231668616	2.193868961	3.78E-05	0.001181213	0.00089537	1233	tags=53%, list=39%, signal=35%	SHC1/FBN1/TYK2/EFNB1/NFIA/RALA/FGFR1/PDGFRB/RHBDF2/DAB2/IGFBP4/HTRA1/IGF2/IQGAP1/COL4A2/ABL1/VEGFB/CHRD/RGMB/MAPKAPK3/SOCS2/COL3A1/STAT3/COL4A5/RAC1/INHBA/ILK/FKBP1A/ITGB1/CYFIP1/SULF1/

											COL4A6/IGFBP6/HIPK2/TGFA/BMP4/PTN/PPP2R5B/TGFBRAP1/MYOC/CITED2/IGF2R/GHRH/BMP2/TEK/ZBTB7A/FOXC1/SLC31A1/UBE2D1/NRP2/CPNE3/RAPGEF1/ARHGEF7/PTPN2/FZD4/FNTA/SVEP1/LYN/RELA/TRIO/COL4A1/CLDN5/LTBP1/CDKN1C/ITGA3/CDH3/ITGA5/APOD/APLN/FGF1/NTF3/LRG1/ENG/FRS2/IFI6/HSPB1/GPR21/RBPMS/NRP1/INPPL1/STUB1/INS/CD4/PDK2/MPZL1/CLEC14A/MMRN2/GRB7/FGF19/ERRFI1/ITGB8/AGR2/FAM20C/ARRB2/CD7/NFATC4/SOCS1/KALRN/SMAD7/GNG7/ADIPOQ/CHMP6/ATF2/TNXB/GHSR/RGS14/ZNF703/EGR1
GOBP_LEUKOCYTE_DIFFERENTIATION	GOBP_LEUKOCYTE_DIFFERENTIATION	GOBP_LEUKOCYTE_DIFFERENTIATION	122	0.272783207	2.287174041	3.96E-05	0.001228168	0.000930962	977	tags=48%, list=31%, signal=35%	SH3PXD2A/FBN1/CTSK/LAG3/CEBPB/TFE3/XBP1/HMGB1/TYROBP/ABL1/HCLS1/EIF2AK1/MAFB/GLI2/PPP3CA/SLC25A5/LIPA/STAT3/C1QC/HLA-B/INHBA/MYC/PTPRC/CEBPA/LTBR/ITGB1/BMP4/CITED2/CBFB/IL15/TSC1/PIAS3/BMP2/ZBTB7A/FGL2/BCL2/AIRE/BTN2A2/ID2/PTPN2/MMP14/LYN/TRPM2/FCER1G/TCTA/NR3C1/LFNG/FOSL2/EOMES/IL17A/OSTM1/AGER/BATF2/KLF10/BAD/CCR1/CD4/BAX/SPN
GOBP_DNA_TEMPLATED_DNA_REPLICATION_MAINTENANCE_OF_FIDELITY	GOBP_DNA_TEMPLATED_DNA_REPLICATION_MAINTENANCE_OF_FIDELITY	GOBP_DNA_TEMPLATED_DNA_REPLICATION_MAINTENANCE_OF_FIDELITY	15	-0.614908394	-2.729881574	4.09E-05	0.001261285	0.000956065	804	tags=80%, list=25%, signal=60%	PARP1/ATR/TIMELESS/RAD50/WRN/POLE/BARD1/PCNA/BLM/DONSON/TRAIP/RAD51
GOBP_MYELOID_LEUKOCYTE_DIFFERENTIATION	GOBP_MYELOID_LEUKOCYTE_DIFFERENTIATION	GOBP_MYELOID_LEUKOCYTE_DIFFERENTIATION	53	0.368736603	2.403156861	4.14E-05	0.001264991	0.000958874	1171	tags=66%, list=37%, signal=42%	SH3PXD2A/FBN1/CEBPB/TFE3/TYROBP/HCLS1/EIF2AK1/MAFB/PPP3CA/C1QC/INHBA/MYC/CEBPA/LTBR/BMP4/CITED2/IL15/PIAS3/BMP2/ID2/PTPN2/LYN/TCTA/NR3C1/FOSL2/IL17A/OSTM1/BATF2/KLF10/CCR1/CD4/ITGB8/FAM20C/SOCS1/ADIPOQ
GOBP_REGULATION_OF_LYMPHOCYTE_ACTIVATION	GOBP_REGULATION_OF_LYMPHOCYTE_ACTIVATION	GOBP_REGULATION_OF_LYMPHOCYTE_ACTIVATION	92	0.305430519	2.360283351	4.16E-05	0.001264991	0.000958874	694	tags=43%, list=22%, signal=35%	TYK2/LAG3/EFNB1/PYCARD/IGF2/CEBPB/XBP1/HLA-DMB/HMGB1/TYROBP/ABL1/HAVCR2/AIF1/HLA-DRB3/GLI2/HLA-DQB1/PPP3CA/HLA-E/INHBA/HL

											A-F/HLA-DRB5/PTPRC/HLA-DMA/HLA-A/FLOT2/BMP4/CBFB/CCL21/IL15/FGL2/HLA-DOB/CCL5/CHRNB2/BCL2/BTN2A2/ID2/PTPN2/MMP14/LYN/SCGB1A1
GOCC_ENDOSOME	GOCC_ENDOSOME	GOCC_ENDOSOME	221	0.221305846	2.138413918	4.18E-05	0.001264991	0.000958874	777	tags=38%, list=25%, signal=31%	CCDC115/CTSK/ANTXR2/WDR81/SLA/IFITM3/FYCO1/TBC1D2B/SCARB2/F8A1/HLA-DMB/HMGB1/TBC1D16/ZMPSTE24/RABEP2/SMO/HAVCR2/BLOC1S1/HLA-DRB3/RAB1A/OSBPL1A/HLA-DQB1/ATP1A2/TMEM25/RAC1/HLA-B/HLA-E/HLA-F/HLA-DRB5/TPCN1/HPS6/FCHSD1/RHOU/ITGB1/STEAP3/BET1L/HLA-DMA/HLA-A/FLOT2/HLA-C/ANXA6/TRAK2/PRAF2/TGFBRAP1/BACE2/EHD4/IGF2R/WIPI1/IL15/TNFAIP1/IRAK1/CLN3/SBF2/AP1G2/HLA-DOB/SLC11A1/SLC2A13/CTSH/SLC9A8/MITD1/ABHD6/RAB11A/DERL1/LLGL1/SLC31A1/VPS13C/RAPGEF1/PTPN2/MMP14/SCAMP3/GPR161/AP2M1/UVRAG/TRAPPC1/DNAJC13/AOC3/SLC29A3/CLINT1/PRDX3/GPR135/OCIAD2/GPR15/MAGEL2/KIR2DL4
GOBP_ESTABLISHMENT_OF_RNA_LOCALIZATION	GOBP_ESTABLISHMENT_OF_RNA_LOCALIZATION	GOBP_ESTABLISHMENT_OF_RNA_LOCALIZATION	35	-0.422050732	-2.646471194	4.24E-05	0.001273313	0.000965182	1236	tags=80%, list=39%, signal=49%	RBM15B/MCM3AP/AAAS/NUTF2/KHSRP/XPO1/NUP107/IGF2BP1/NUP37/PABPN1/RANBP2/ATR/IWS1/SMG7/AKAP8L/NUP93/THOC5/NOL6/SNUPN/PNPT1/PARP11/KPNB1/TPR/NUP88/RAE1/NUP155/AHCTF1/NCBP2
GOBP_DNA_TEMPLATED_DNA_REPLICATION	GOBP_DNA_TEMPLATED_DNA_REPLICATION	GOBP_DNA_TEMPLATED_DNA_REPLICATION	41	-0.392386086	-2.604454957	4.35E-05	0.001298117	0.000983984	811	tags=66%, list=26%, signal=50%	LIG3/HMGA1/RFC1/ENDOG/PARP1/ATR/TIMELESS/MCM5/RAD50/WRN/BAZ1A/CDC7/POLE/BARD1/PCNA/PRIM1/POLRMT/BLM/POLA2/RECQL4/DONSON/RFC4/POLG2/TRAIP/POLB/RAD51/GMNN
GOBP_REGULATION_OF_PROGRAMMED_CELL_DEATH	GOBP_REGULATION_OF_PROGRAMMED_CELL_DEATH	GOBP_REGULATION_OF_PROGRAMMED_CELL_DEATH	297	0.199759066	2.047316784	4.44E-05	0.001317313	0.000998535	875	tags=39%, list=28%, signal=31%	SHC1/NFKB1/CTSK/CASP4/ALDH1A3/TXNDC5/AKR1A1/TNFSF10/FGFR1/PHB2/DAB2/HTRA1/PYCARD/IFIT3/CEBPB/XBP1/HMGB1/ABL1/SMO/HCLS1/H19/VDAC1/CYP1B1/EIF2AK2/TRAF1/CAT/CD248/SOCS2/BTG2/SLC25A5/TSPO/I

											NHBA/MYC/GADD45A/PTPRC/NFE2L2/HAND2/LTBR/BFAR/ITGB1/ARHGDIA/ING4/HIPK2/MAP3K5/BMP4/ATF4/NQO1/NKX2-5/TMEM14A/ANXA4/PHLDA3/SRPX/MNT/TFPT/CITED2/PRKCH/IGF2R/SNAI2/CCL21/WFS1/CYLD/CDK5/CLN3/BCL2L1/TMEM109/KCNB1/PRMT2/RAMP2/BMP2/TEK/DHCR24/ZBTB7A/CCL5/FOXC1/CTSH/CCNL2/ANGPTL4/GRIA4/BCL2L2/BCL2/PRAP1/AMIGO2/ARHGEF7/ARF4/PTPN2/HSH2D/CTSC/CBS/DNAJC5/LYN/UBE2B/RELA/BBC3/DEDD/TRAF5/NR3C1/PRDX3/IER3/RGL2/SIRT2/YAP1/PPP2R1A/ITGA5/CCR5/NTF3/ENG/FRS2/ADM/WNT11/IFI6/HSPB1/GCLC/MAGED1/NET1/RRM2B
GOBP_DETECTION_OF_STIMULUS_INVOLVED_IN_SENSORY_PERCEPTION	GOBP_DETECTION_OF_STIMULUS_INVOLVED_IN_SENSORY_PERCEPTION	GOBP_DETECTION_OF_STIMULUS_INVOLVED_IN_SENSORY_PERCEPTION	80	0.323073941	2.402489858	4.57E-05	0.001347938	0.001021748	1508	tags=74%, list=48%, signal=39%	OR51A7/OR5V1/OR52E4/TAS2R39/OR9K2/OR13C4/OR10T2/OR6V1/OR8G1/OR10J5/TAS2R9/OR6C4/OR1E2/OR5F1/OR52D1/OR8A1/OR10H4/OR4K17/OR8H2/OR6X1/SERPINE2/OR8D1/OR52B6/TAS2R40/TAS2R10/OR4K14/OR7G2/OR6Y1/OR3A3/TAS2R41/OR5AS1/OR5D18/OR4S1/OR2T4/OR7D2/OR6M1/OR6K2/OR2A12/OR8B8/OR8S1/OR6B2/OR51S1/OR2J2/OR1D4/OR51L1/OR52M1/OR13C8/TAS2R60/OR4D5/OR1D2/OR4M1/OR1F1/TRPV1/OR4D1/OR10A4/OR10H3/OR4P4/OR2B11/SEMA5B
GOBP_POSITIVE_REGULATION_OF_T_CELL_PROLIFERATION	GOBP_POSITIVE_REGULATION_OF_T_CELL_PROLIFERATION	GOBP_POSITIVE_REGULATION_OF_T_CELL_PROLIFERATION	24	0.513377528	2.494599348	4.81E-05	0.001408433	0.001067604	535	tags=58%, list=17%, signal=49%	TYK2/EFNB1/PYCARD/IGF2/HLA-DMB/HMGB1/HAVCR2/AIF1/PPP3CA/HLA-E/PTPRC/HLA-A/IL15/CCL5
GOBP_CELL_MATRIX_ADHESION	GOBP_CELL_MATRIX_ADHESION	GOBP_CELL_MATRIX_ADHESION	60	0.356086074	2.435946441	4.83E-05	0.001408433	0.001067604	1263	tags=68%, list=40%, signal=42%	CDH11/ABL1/EMP2/COL3A1/TRIP6/RAC1/ITGB1BP1/CD34/ILK/ITGB1/MYOC/CCL21/PARVG/ITGA9/TSC1/ADAM15/ITGB7/CDK5/TEK/EDA/BCL2/ARHGEF7/PHLDB2/MMP14/PLAU/FAM107A/CORO2B/ITGA3/ITGA5/APOD/NRP1/CLASP2/ITGAM/MINK1/ITGB8/ROCK2/CASK/ACTN2/TNXB/DLC1/CTTN
GOBP_MUSCLE_C	GOBP_MUSCLE_C	GOBP_MUSCLE_CELL_	38	0.428578224	2.478856834	4.99E-05	0.00144531	0.00109555	905	tags=63%,	PDGFRB/ABL1/OGN/AIF1/MAP3K5/BM

ELL_PROLIFERATION	ELL_PROLIFERATION	PROLIFERATION					1	8		list=29%, signal=46%	P4/NKX2-5/CITED2/ZFPM2/IRAK1/BMP2/CCL5/FOXC1/ELN/MEF2D/ID2/GNA12/YAP1/APOD/APLN/FRS2/ERN1/VGLL4/MDM2
GOBP_REGULATION_OF_RESPONSE_TO_EXTERNAL_STIMULUS	GOBP_REGULATION_OF_RESPONSE_TO_EXTERNAL_STIMULUS	GOBP_REGULATION_OF_RESPONSE_TO_EXTERNAL_STIMULUS	192	0.232747015	2.192461438	5.20E-05	0.001496209	0.001134139	1508	tags=64%, list=48%, signal=35%	NFKB1/CASP4/LAG3/TFPI/FGFR1/PDGFRB/PHB2/LSM14A/RHBDF2/PYCARD/CEBPB/PQBP1/HMGB1/ZMPSTE24/TYROBP/EIF2AK4/HAVCR2/VEGFB/AIF1/ALOX5AP/EIF2AK2/MAPKAPK3/PLSCR1/ST3GAL4/STAT3/RAC1/PUM1/HLA-B/HLA-E/HLA-F/PTPRC/CEBPA/C3AR1/NFE2L2/HLA-A/FLOT2/PTN/ALOX5/NMI/ABHD12/SNAI2/CCL21/COLEC11/PTGES/RNF185/IL15/CYLD/IRAK1/TEK/ROBO3/FGL2/CCL5/ISG15/LY86/BCL2/MEFV/PHLDB2/PTPN2/CTSC/LYN/SCGB1A1/PLAU/BRCC3/RELA/CXCL13/IER3/SIRT2/KIR2DL4/FGF1/NTF3/IL17A/TRIM41/HSPB1/AGER/SERPINE2/ZNFX1/NFKBIL1/NRP1/CLASP2/CCR1/NR1H2/INS/BAX/OTOP1/TRAFD1/SPN/MMRN2/TSPAN8/DDX3X/CASK/ARRB2/ETS1/ADIPOQ/TRIM44/PJA2/GHSR/CX3CR1/GRK1/ADA/UNC93B1/LTA/KREMEN1/MFHAS1/FFAR2/PDGFRA/AKT1/S100A12/OXSR1/F11/C3/MT3/GPSM3/WNT3A/S100A14/ATG5/LTF/TSPAN32/MYOD1/OTUD4/BCR/STX3/SEMA5B
GOBP_POSITIVE_REGULATION_OF_INTRACELLULAR_SIGNAL_TRANSDUCTION	GOBP_POSITIVE_REGULATION_OF_INTRACELLULAR_SIGNAL_TRANSDUCTION	GOBP_POSITIVE_REGULATION_OF_INTRACELLULAR_SIGNAL_TRANSDUCTION	193	0.230257321	2.16467927	5.24E-05	0.001500845	0.001137653	1184	tags=50%, list=38%, signal=33%	SHC1/F10/TNFSF10/FGFR1/PDGFRB/PHB2/DAB2/IGFBP4/PYCARD/IGF2/IQGAP1/HMGB1/ABL1/HAVCR2/HCLS1/H19/EIF2AK2/TRIM8/PPP3CA/COL3A1/TRIP6/RAC1/ITGB1BP1/INHBA/ILK/MYC/GADD45A/PTPRC/ARHGEF3/LMCD1/HAND2/LTBR/S100A4/FKBP1A/ITGB1/VAPA/FBXW11/MID1/ING4/IGFBP6/HIPK2/MAP3K5/TGFA/BMP4/NMI/MYOC/CCL21/IRAK1/VWF/BMP2/TEK/EDA/CCL5/MIER1/RHOC/PTPN2/LYN/RELA/BBC3/F2RL3/TRAF5/GRM1/SLC35B2/FGF1/NTF3/ENG/FRS2/WNT11/ERN1/AGER/NEK6/NET1/RPS15/NRP1/BAD/CCR1/MAP

											2K3/RRAGA/INS/CD4/BAX/DVL2/MAZ/AVPI1/MINK1/FGF19/ROCK2/DDX3X/FSHR/S100B/GPR3/ARRB2/CCL8/IQGAP3/ADIPOQ/TRIM44/PJA2
GOBP_RESPONSE_TO_WOUNDING	GOBP_RESPONSE_TO_WOUNDING	GOBP_RESPONSE_TO_WOUNDING	120	0.274416889	2.279877156	5.37E-05	0.00152931	0.00115923	1445	tags=65%, list=46%, signal=37%	F10/NFIA/TFPI/XBP1/TYROBP/PPP3CA/PLSCR1/ST3GAL4/CLIC1/TSPO/COL3A1/STAT3/RAC1/CD34/INHBA/ILK/ITPK1/HPS6/NFE2L2/ITGB1/ANXA6/PTN/ALOX5/VWF/KCNB1/TOR1A/SLC11A1/BCL2/PHLDB2/RHOC/LYN/PLAU/FCER1G/F2RL3/GNA12/YAP1/ITGA5/APOD/FGF1/SERPINA1/LRG1/IL17A/HSPB1/AGER/SERPINE2/CHMP2B/NRP1/CLASP2/CCR1/INS/BAX/DCBLD2/DUOX2/CHMP4C/ADIPOR2/TSPAN8/CASK/CLDN4/CHMP6/CHMP7/VPS4A/CX3CR1/AQP1/ADRA2B/EPB41L4B/KREMEN1/TIMP1/PDGFRA/NOTCH4/ENTPD2/F11/CAV3/WNT3A/F8/F11R/SPRR3/TSPAN32/ELK3
GOBP_MITOTIC_SISTER_CHROMATID_SEGREGATION	GOBP_MITOTIC_SISTER_CHROMATID_SEGREGATION	GOBP_MITOTIC_SISTER_CHROMATID_SEGREGATION	49	-0.370505541	-2.589323292	5.48E-05	0.001551073	0.001175726	921	tags=65%, list=29%, signal=47%	MAPRE1/SMC4/CHEK2/LCMT1/CDC16/UBE2C/NUSAP1/BCCIP/ZW10/AKAP8L/CDK5RAP2/KIF22/KIF4A/SMC3/MAD2L1BP/PINX1/TUBG2/ESPL1/MAD2L2/DYNC1LI1/KPNB1/TPX2/TRIP13/KIF23/TUBG1/CENPE/BUB1B/KATNB1/TPR/AURKC/CDCA8/SPAG5
GOBP_REGULATION_OF_CELL_SUBSTRATE_ADHESION	GOBP_REGULATION_OF_CELL_SUBSTRATE_ADHESION	GOBP_REGULATION_OF_CELL_SUBSTRATE_ADHESION	52	0.372682579	2.441174753	5.65E-05	0.001587628	0.001203435	1116	tags=65%, list=35%, signal=43%	DAB2/ABL1/EMP2/ABI3BP/RAC1/ITGB1BP1/RSU1/ILK/MYOC/TRIOBP/CCL21/TSC1/ADAM15/MYADM/TEK/BCL2/HAS2/ARHGEF7/PHLDB2/MMP14/FZD4/PLAU/FAM107A/CORO2B/ITGA3/ITGA5/APOD/PKP2/NRP1/CLASP2/MINK1/ROCK2/AGR2/CASK
GOBP_SUBSTRATE_ADHESION_DEPENDENT_CELL_SPREADING	GOBP_SUBSTRATE_ADHESION_DEPENDENT_CELL_SPREADING	GOBP_SUBSTRATE_ADHESION_DEPENDENT_CELL_SPREADING	26	0.492720399	2.491316578	5.77E-05	0.00161209	0.001221978	649	tags=62%, list=21%, signal=49%	PARVA/DAB2/ABL1/RAB1A/RAC1/ITGB1BP1/ILK/MYOC/TRIOBP/PARVG/ITGB7/MYADM/TEK/HAS2/ARHGEF7/FZD4
GOBP_REGULATION_OF_T_CELL_ACTIVATION	GOBP_REGULATION_OF_T_CELL_ACTIVATION	GOBP_REGULATION_OF_T_CELL_ACTIVATION	74	0.325204454	2.363246063	6.16E-05	0.001703875	0.001291552	1157	tags=61%, list=37%, signal=39%	TYK2/LAG3/EFNB1/PYCARD/IGF2/CEBPB/XBP1/HLA-DMB/HMGB1/ABL1/HAVCR2/AIF1/HLA-DRB3/GLI2/HLA-DQB1/PPP3CA/HLA-E/HLA-DRB5/PTPRC/HLA-DMA/HLA-A/FLOT2/BMP4/CBFB/

GOBP_REGULATION_OF_CELL_CYCLE_PHASE_TRANSITION	GOBP_REGULATION_OF_CELL_CYCLE_PHASE_TRANSITION	GOBP_REGULATION_OF_CELL_CYCLE_PHASE_TRANSITION	100	-0.254501708	-2.232856604	6.16E-05	0.001703875	0.001291552	748	tags=44%, list=24%, signal=35%	CCL21/IL15/FGL2/HLA-DOB/CCL5/BTN2A2/PTPN2/LYN/SCGB1A1/CD276/AGER/BAD/CD4/SPN/CD83/PRKAR1A/TNFSF13B/PAG1/SOCS1/TNFRSF21/SMAD7ERCC3/TIMELESS/RAD50/TP53BP1/ZW10/SMARCC1/DCUN1D3/E2F1/CDK5RAP2/TFDP1/ECD/CDC7/MAD2L1BP/PINX1/THOC5/BRD7/ESPL1/BARD1/MAD2L2/DYNC1LI1/CEP63/CDK7/BLM/PAXIP1/TRIP13/PAF1/DONSON/INTS7/FBXO7/CENPE/BUB1B/FZR1/MDC1/RAD51/TPR/CRY1/CCNH/CDCA8/HSPA2/DOT1L/CDC14B/CDC25C/RAD17/CPSF3
GOBP_POSITIVE_REGULATION_OF_DEVELOPMENTAL_PROCESS	GOBP_POSITIVE_REGULATION_OF_DEVELOPMENTAL_PROCESS	GOBP_POSITIVE_REGULATION_OF_DEVELOPMENTAL_PROCESS	263	0.203831641	2.041831526	6.54E-05	0.001795995	0.001361379	1491	tags=60%, list=47%, signal=35%	NFKB1/RALA/FGFR1/LRP3/DAB2/IGF2/CEBPB/TFE3/XBP1/HMGB1/TYROBP/ABL1/EMP2/IFITM1/SMO/HCLS1/VEGFB/CYP1B1/GLI2/PPP3CA/NAP1L1/CLIC1/SOCS2/TSPO/PLXNA1/STAT3/CD34/LOXL2/INHBA/ILK/MYC/PTPRC/CEBPA/C3AR1/NFE2L2/EIF4G1/ITGB1/CYFIP1/HIPK2/MAP3K5/BMP4/PTN/ATF4/NKX2-5/ADD1/ALOX5/CITED2/PRKCH/CBFB/ZFPM2/SNAI2/IL15/CCDC3/ADAM12/RAMP2/GHRH/BMP2/TEK/FOXC1/CTSH/ANGPTL4/ISG15/BCL2/BTN2A2/AMIGO2/ARHGEF7/ID2/PHLDB2/PLAG1/MMP14/FZD4/LYN/RELA/MAML1/CLDN5/SIRT2/YAP1/ITGA5/FGF1/LRG1/ENG/FRS2/ZDHHC6/IL17A/ADM/WNT11/TMEM119/HSPB1/NCOA3/AGER/MAGED1/MIXL1/SERPINE2/GPR21/MDM2/KLF10/NRP1/CLASP2/BAD/CCR1/INS/CD4/BAX/DVL2/PTCH2/MMRN2/CD83/SSBP3/CUL7/CDH4/SLITRK2/ITGB8/TNFSF13B/AGR2/S100B/MYOG/GCM1/FAM20C/FBXW8/ARRB2/SOCS1/GDI1/WNT2B/SMAD7/ETS1/IQGAP3/ADIPOQ/TNXB/GHSR/CX3CR1/RGS14/ZNF703/AQP1/ASCL1/ADRA2B/ADA/RALBP1/CLSTN3/LTA/JUND/AKT1/CCBE1/DLG4/ZNF488/ZBTB16/LRRN3/C3/CAV3/WNT3A/F11R/LTF/DAB1/FIS1/MYOD1/TMEM106B/H

											OXD3/IRX3/WNT1
GOBP_DETECTION_OF_STIMULUS	GOBP_DETECTION_OF_STIMULUS	GOBP_DETECTION_OF_STIMULUS	105	0.283927875	2.276557959	6.70E-05	0.001830983	0.001387901	1508	tags=69%, list=48%, signal=37%	SMO/TSPO/HLA-B/OR51A7/OR5V1/OR52E4/HLA-A/SRPX/TAS2R39/OR9K2/FECH/OR13C4/OR10T2/OR6V1/OR8G1/OR10J5/TAS2R9/OR6C4/OR1E2/OR5F1/OR52D1/OR8A1/OR10H4/OR4K17/OR8H2/YAP1/OR6X1/SERPINE2/OR8D1/OR52B6/TAS2R40/TAS2R10/OR4K14/CALM3/OR7G2/OTOP1/OR6Y1/OR3A3/TAS2R41/OR5AS1/OR5D18/OR4S1/OR2T4/OR7D2/OR6M1/OR6K2/OR2A12/OR8B8/OR8S1/OR6B2/ATF2/OR51S1/GRK1/OR2J2/OR1D4/OR51L1/OR52M1/OR13C8/TAS2R60/OR4D5/OR1D2/OR4M1/OR1F1/TRPV1/P2RX2/OR4D1/CAV3/OR10A4/OR10H3/OR4P4/OR2B11/SEMA5B
GOCC_CELL_SUBSTRATE_JUNCTION	GOCC_CELL_SUBSTRATE_JUNCTION	GOCC_CELL_SUBSTRATE_JUNCTION	106	0.272951421	2.194884584	6.91E-05	0.001877766	0.001423362	1020	tags=52%, list=32%, signal=36%	SHC1/LASP1/MSN/PARVA/RALA/PDGFRB/SCARB2/DAB2/IQGAP1/TSPAN9/CAP1/LAYN/CAT/TRIP6/RAC1/ITGB1BP1/RSU1/ILK/PTPRC/RHOU/ITGB1/CYFIP1/FLOT2/ANXA6/ADD1/TRIOBP/IGF2R/GDI2/PARVG/ITGB7/TEK/CPNE3/ARHGEF7/PHLDB2/MMP14/ALKBH6/LIMA1/PLAU/FAM107A/GNA12/CORO2B/ITGA3/ITGA5/YWHAB/ENG/NFASC/HSPB1/ARPC1B/RPS15/NRP1/CLASP2/FHL1/MPZL1/RHOG/GRB7
GOBP_MYELOID_CELL_DIFFERENTIATION	GOBP_MYELOID_CELL_DIFFERENTIATION	GOBP_MYELOID_CELL_DIFFERENTIATION	88	0.300587401	2.296528646	7.09E-05	0.001915402	0.001451891	1171	tags=58%, list=37%, signal=37%	SH3PXD2A/FBN1/MAF/CEBPB/TFE3/TYROBP/HCLS1/EIF2AK1/MAFB/PPP3CA/SLC25A5/LIPA/STAT3/C1QC/INHBA/MYC/CEBPA/LTBR/HIPK2/BMP4/CITED2/CBFB/IL15/FECH/PIAS3/BMP2/ZBTB7A/ISG15/ID2/PTPN2/LYN/TCTA/NR3C1/PRDX3/CDKN1C/FOSL2/IL17A/OSTM1/BATF2/KLF10/KLF2/CCR1/CD4/HEATR3/PRMT6/ITGB8/FAM20C/ALAS1/SOCS1/ETS1/ADIPOQ
GOBP_REGULATION_OF_MYELOID_LEUKOCYTE_DIFFERENTIATION	GOBP_REGULATION_OF_MYELOID_LEUKOCYTE_DIFFERENTIATION	GOBP_REGULATION_OF_MYELOID_LEUKOCYTE_DIFFERENTIATION	26	0.488231348	2.468618823	7.33E-05	0.001948609	0.001477062	963	tags=73%, list=31%, signal=51%	FBN1/CEBPB/TFE3/TYROBP/HCLS1/MAFB/PPP3CA/C1QC/INHBA/MYC/PIAS3/ID2/PTPN2/LYN/TCTA/IL17A/KLF10/CCR1/CD4
GOBP_OUTFLOW	GOBP_OUTFLOW_	GOBP_OUTFLOW_TRAC	19	0.547259706	2.418020538	7.29E-05	0.00194860	0.00147706	986	tags=79%,	PARVA/ILK/HAND2/BMP4/NKX2-5/CIT

_TRACT_MORPHOGENESIS	TRACT_MORPHOGENESIS	T_MORPHOGENESIS					9	2		list=31%, signal=55%	ED2/ZFPM2/FOXC1/ELN/NRP2/CLDN5/ENG/WNT11/NRP1/DVL2
GOBP_CELL_DIVISION	GOBP_CELL_DIVISION	GOBP_CELL_DIVISION	143	-0.227283468	-2.237223608	7.31E-05	0.001948609	0.001477062	520	tags=31%, list=16%, signal=28%	SMC3/EPB41L2/TIAL1/PRPF40A/ZC3HC1/ESPL1/MAD2L2/DYNC1LI1/ANAPC10/MYH10/CEP63/CDK7/USP37/TPX2/BLM/SSNA1/SENP5/ITGB3BP/NDE1/RUVBL1/KIF23/VRK1/PPP1CC/UBE2S/CENPE/BUB1B/FZR1/KATNB1/EFHC1/CKS2/TPR/AURKC/PARD6A/CDCA8/CDC14B/CDC25C/KATNA1/SPAG5/CCNB2/RAE1/SH3GLB1/DR1/PAFAH1B1/PTTG1/AHCTF1
GOBP_POSITIVE_REGULATION_OF_CELL_POPULATION_PROLIFERATION	GOBP_POSITIVE_REGULATION_OF_CELL_POPULATION_PROLIFERATION	GOBP_POSITIVE_REGULATION_OF_CELL_POPULATION_PROLIFERATION	244	0.209655705	2.073183926	7.77E-05	0.00205327	0.001556396	1513	tags=62%, list=48%, signal=35%	SHC1/TYK2/EFNB1/CDK4/RALA/FGFR1/PDGFRB/SCARB2/PYCARD/IGF2/XBP1/HLA-DMB/HMGB1/ABL1/EMP2/SMO/HAVCR2/HCLS1/VEGFB/CHRD/AIF1/CD248/PPP3CA/NAP1L1/SLC25A5/TSPO/FOLR2/PLXNA1/STAT3/RAC1/ITGB1BP1/HLA-E/ILK/MYC/PTPRC/NFE2L2/ITGB1/CYFIP1/HLA-A/HIPK2/TSPAN31/MAP3K5/TGFA/BMP4/PTN/PPP2R5B/NKX2-5/PRKCH/ZFPM2/CCL21/PLEK2/IL15/PURA/IRAK1/BCL2L1/CDC42EP5/GHRH/PALM/BMP2/TEK/ROBO3/CCL5/CHRNB2/CTSH/CCPG1/MEF2D/BCL2/MALAT1/CDC25B/HAS2/NRP2/RAPGEF1/ARHGEF7/ID2/PLAG1/FZD4/FLT3LG/LYN/RELA/CLDN5/TRAF5/PRDX3/YAP1/ITGA3/CDH3/APLN/FGF1/NTF3/LRG1/FRS2/FOSL2/CD276/ADM/TMEM119/ERN1/AGER/MDM2/NRP1/BAD/INS/SPN/DVL2/RHOG/MAZ/SSBP3/CUL7/CDH4/PROM2/FGF19/TNFSF13B/S100B/FBXW8/CNBP/GDI1/IQGAP3/PITX3/CROCC/DBF4B/ATF2/TNXB/GHSR/CX3CR1/ZNF703/AQP1/EGR1/ASCL1/HTR1B/ADA/EPOR/LTA/TIMP1/ANG/FXN/TNFRSF4/PDGFRA/AKT1/AUTS2/DLG4/CAV3/WNT3A/CDK2/RARG/LTF/FOXE3/PRRX1/PLA2G1B/TMEM106B/WNT1/DNM3/STX3/LBH/CD6
GOBP_PERIPHER	GOBP_PERIPHERA	GOBP_PERIPHERAL_NE	21	0.525824153	2.406898897	7.89E-05	0.00207458	0.00157255	937	tags=76%,	MED12/ILK/HAND2/ISL2/MYOC/CITED

AL_NERVOUS_SYSTEM_DEVELOPMENT	L_NERVOUS_SYSTEM_DEVELOPMENT	RVOUS_SYSTEM_DEVELOPMENT					7	5		list=30%, signal=54%	2/CDK5/PRX/RELA/LGI4/SIRT2/GFRA3/NTF3/NFASC/CNTNAP1/NHLH2
GOBP_NEGATIVE_REGULATION_OF_DNA_METABOLIC_PROCESS	GOBP_NEGATIVE_REGULATION_OF_DNA_METABOLIC_PROCESS	GOBP_NEGATIVE_REGULATION_OF_DNA_METABOLIC_PROCESS	26	-0.454167138	-2.538363889	8.36E-05	0.002143082	0.001624475	983	tags=77%, list=31%, signal=53%	RIF1/FLCN/LIG3/SENP3/PARP1/ATR/TIMELESS/RAD50/TP53BP1/POT1/TTF1/PINX1/TRIM28/MAD2L2/BLM/GTPBP4/TFIP11/GMNN/RAD17/SMCHD1
GOBP_SMOOTH_MUSCLE_CELL_MIGRATION	GOBP_SMOOTH_MUSCLE_CELL_MIGRATION	GOBP_SMOOTH_MUSCLE_CELL_MIGRATION	18	0.564788462	2.456635322	8.35E-05	0.002143082	0.001624475	919	tags=78%, list=29%, signal=55%	PARVA/PDGFRB/AIF1/CYP1B1/PLXNA1/NFE2L2/BMP4/CCL5/BCL2/HAS2/PLAU/GNA12/MDM2/NRP1
GOBP_TAXIS	GOBP_TAXIS	GOBP_TAXIS	73	0.322062593	2.338610523	8.38E-05	0.002143082	0.001624475	1508	tags=74%, list=48%, signal=40%	PARVA/RALA/FGFR1/PDGFRB/HMGB1/VEGFB/AIF1/FOLR2/RAC1/LSP1/C3AR1/S100A4/BMP4/PTN/ALOX5/CCL21/ITGA9/ROBO3/CCL5/OR10J5/NRP2/PTPN2/LYN/TRPM2/PLAU/FCER1G/CXCL13/FGF1/CCR5/NTF3/HSPB1/AGER/CXCR3/NRP1/CCR1/SPN/RHOG/ARRB2/CCL8/CX3CR1/RALBP1/OR1D2/XCR1/FFAR2/CCL22/PDGFRA/S100A12/OXSR1/GPSM3/WNT3A/S100A14/PLA2G1B/STX3/SEMA5B
GOBP_REGULATION_OF_HEMOPOIESIS	GOBP_REGULATION_OF_HEMOPOIESIS	GOBP_REGULATION_OF_HEMOPOIESIS	74	0.321488014	2.336238862	8.35E-05	0.002143082	0.001624475	1171	tags=61%, list=37%, signal=39%	FBN1/LAG3/CEBPB/TFE3/XBP1/HMGB1/TYROBP/ABL1/HCLS1/MAFB/GLI2/PPP3CA/STAT3/C1QC/HLA-B/INHBA/MYC/PTPRC/CEBPA/BMP4/PTN/CBFB/SNAI2/IL15/PIAS3/FGL2/ISG15/BTN2A2/ID2/PTPN2/MMP14/LYN/TCTA/IL17A/AGER/KLF10/BAD/CCR1/CD4/CD83/PRMT6/SOCS1/SMAD7/ETS1/ADIPOQ
GOBP_MONOATOMIC_CATION_TRANSPORT	GOBP_MONOATOMIC_CATION_TRANSPORT	GOBP_MONOATOMIC_CATION_TRANSPORT	196	0.231158158	2.179456988	8.28E-05	0.002143082	0.001624475	1574	tags=65%, list=50%, signal=35%	COX7A1/CYBRD1/PDGFRB/PHB2/ZMPSTE24/ABL1/GSTO1/COX7B/VDAC1/COX8A/ITPR1/TMCO3/PPP3CA/SLC25A23/SLC25A5/TSPO/ATP1A2/PTPRC/TPCN1/SLC30A9/SCARA5/CCDC51/FXYD5/FKBP1A/ITGB1/STEAP3/ABCB7/ANXA6/SLC25A12/ATF4/NKX2-5/KCNK5/SLC12A9/KCNE3/CCL21/WFS1/GJA4/TSC1/CDK5/KCNB1/CSN2/SLC25A37/RAMP2/OTOP3/CCL5/SLC11A1/CHRNB2/SLC34A2/SLC9A8/BCL2/SLC31A1/SRI/IBTK/LYN/TRPM2/ATP2A3/F2RL3/COX15/SEC61A1/CCR5/SLC6A17/SERPINE2/TMEM

										63A/PKP2/CCR1/CALM3/PPIF/LIME1/CD4/BAX/OTOP1/KCNH4/FHL1/SLC24A3/CNNM2/OTOP2/KCNB2/KCNJ1/NIPA2/FGF13/CABP5/CASK/SLC10A5/CCL8/KCNAB2/DIAPH1/ACTN2/ATP6V1G2/SLC6A1/AQP1/KCNJ6/KCNH7/SLC5A7/XCR1/TRPV1/FXN/P2RX2/THY1/HCN4/AKT1/OXSR1/SLC22A2/MT3/CAV3/WNT3A/ATG5/CDK2/CACNA2D3/LTF/HCN3/ATP13A1/PLA2G1B/SLC32A1/ATP6V0A1/CACNA1B/KCNQ3/SLC34A3/KCNS2/CACNA1E/SLC12A8/SLC25A13/ATP1B1/SLC12A3/CACNB3/SLC17A7/ATP1A3/GRM6/ATP2A1
GOCC_SIDE_OF_MEMBRANE	GOCC_SIDE_OF_MEMBRANE	GOCC_SIDE_OF_MEMBRANE	143	0.252767859	2.218485679	8.60E-05	0.002188043	0.001658555	1382	tags=61%, list=44%, signal=36%	TYK2/CTSK/LAG3/F10/TFPI/IQGAP1/BLOC1S1/HLA-DRB3/TRAF1/HLA-DQB1/RGMB/CD248/PPP3CA/FOLR2/CD34/HLA-B/HLA-E/HLA-F/HLA-DRB5/HEG1/PTPRC/HM13/FKBP1A/ITGB1/BTNL2/HLA-A/MAP3K5/HLA-C/PLEKHA4/COLEC11/OMG/CYLD/ITGA9/CD177/PALM/CHRNB2/BTN2A2/LYNX1/ACP1/AP2M1/LYN/PLAU/FCER1G/TRAF5/GNA12/BTN1A1/LSAMP/ITGA3/ITGA5/GNAI1/CCR5/GFRA3/ENG/CD276/IL17A/CXCR3/PRMT8/CCR1/CD4/ABCB1/SPN/SLC22A11/ITGAM/CLEC14A/CD1A/CD83/CHMP4C/PTP4A1/GNB1/GNG7/CHMP7/KCNAB2/CX3CR1/CD1B/MMP17/ADA/EPOR/UMODL1/CD52/XCR1/TRPV1/CD79A/THY1/IGSF21/TNFRSF4/PDGFRA/DLG4
GOBP_REGULATION_OF_LEUKOCYTE_DIFFERENTIATION	GOBP_REGULATION_OF_LEUKOCYTE_DIFFERENTIATION	GOBP_REGULATION_OF_LEUKOCYTE_DIFFERENTIATION	60	0.348012048	2.380712901	8.90E-05	0.002251877	0.001706942	963	tags=57%, list=31%, signal=40%	FBN1/LAG3/CEBPB/TFE3/XBP1/HMGB1/TYROBP/ABL1/HCLS1/MAFB/GLI2/PPP3CA/C1QC/HLA-B/INHBA/MYC/PTPRC/BMP4/CBFB/IL15/PIAS3/FGL2/BTN2A2/ID2/PTPN2/MMP14/LYN/TCTA/IL17A/AGER/KLF10/BAD/CCR1/CD4
GOCC_RNA_POLYMERASE_COMPLEX	GOCC_RNA_POLYMERASE_COMPLEX	GOCC_RNA_POLYMERASE_COMPLEX	24	-0.491805476	-2.669327955	9.18E-05	0.002310565	0.001751428	748	tags=67%, list=24%, signal=51%	ERCC3/TAF9/TAF6/GTF2E1/TAF10/POLR3E/GTF2H1/GTF2H2/PRIM1/POLRMT/CDK7/POLA2/PAF1/CCNH/SUPT3H/TBPL1
HP_FETAL_ONSE	HP_FETAL_ONSET	HP_FETAL_ONSET	39	-0.384352906	-2.508191842	9.29E-05	0.00232728	0.00176409	418	tags=44%,	PRIM1/CHRNG/PYCR1/RECQL4/VRK1/

T							9		list=13%, signal=38%	CENPE/ASNS/WNT3/FZR1/TRAIP/DEF6/SLC25A19/CLPB/PSMD12/NUP88/NUP155/CPSF3
GOBP_POSITIVE_REGULATION_OF_REACTIVE_OXYGEN_SPECIES_METABOLIC_PROCESS	GOBP_POSITIVE_REGULATION_OF_REACTIVE_OXYGEN_SPECIES_METABOLIC_PROCESS	GOBP_POSITIVE_REGULATION_OF_REACTIVE_OXYGEN_SPECIES_METABOLIC_PROCESS	17	0.563704682	2.417788024	9.63E-05	0.002387985	0.001810113	465	tags=59%, list=15%, signal=50%	AKR1C1/CBR1/PDGFRB/TYROBP/H19/CYP1B1/TSPO/GADD45A/NFE2L2/CD177
GOBP_EPITHELIUM_DEVELOPMENT	GOBP_EPITHELIUM_DEVELOPMENT	GOBP_EPITHELIUM_DEVELOPMENT	249	0.203984539	2.0159415	9.63E-05	0.002387985	0.001810113	1516	tags=60%, list=48%, signal=34%	MSN/MAF/ALDH1A3/AKR1C1/RALA/CBR1/PHB2/DAB2/MED12/CEBPB/IQGAP1/PGK1/ZMPSTE24/ABL1/SMO/VDAC1/KRT14/CHRD/RAB1A/MAFB/GLI2/CAT/PPP3CA/TMOD1/LIPA/PLXNA1/RAC1/CD34/INHBA/ILK/HEG1/MYC/ITPK1/CEBPA/HAND2/ITGB1/ANXA7/SULF1/BMP4/ATF4/NKX2-5/MTHFD1/PLEKHA4/ANXA4/APCDD1/ADD1/TRIOBP/CITED2/PRKCH/SNAI2/GJA4/DNASE1L2/TSC1/MYADM/TOR1A/BMP2/EDA/FOXC1/CTSH/BCL2/AIRE/PODXL/RAPGEF1/ID2/PHLDB2/RHOC/MMP14/FZD4/GPR161/KRT85/MAFG/RELA/COL4A1/CLDN5/NR3C1/LFNG/COL27A1/YAP1/CDKN1C/CDH3/ITGA5/FGF1/LRG1/ENG/FRS2/FOSL2/IL17A/ADM/WNT11/NCOA3/MAGED1/SERPINE2/NSDHL/KRT27/LTA4H/KLF2/NRP1/CLASP2/BAD/BAX/DVL2/PTCH2/MMRN2/SSBP3/ERRFI1/ROCK2/AGR2/GCM1/FAM20C/TTC8/NFATC4/CLDN4/WNT2B/SMAD7/IQGAP3/KRT38/PITX3/ADIPOQ/UGCG/ZNF703/AQP1/LMO4/ASCL1/DLC1/TBC1D20/CNFN/TBX6/EMX1/NOTCH4/AKT1/NPHP3/SEC24B/E2F7/C3/CAV3/WNT3A/F11R/CA2/RARG/ELOVL1/SPRR3/FOXE3/THRA/PHACTR4/KRT32/IRX3/WNT1/BCR/LBH/SH3BP1
GOMF_ATP_HYDROLYSIS_ACTIVITY	GOMF_ATP_HYDROLYSIS_ACTIVITY	GOMF_ATP_HYDROLYSIS_ACTIVITY	75	-0.281578052	-2.259082227	9.71E-05	0.002394541	0.001815082	748	tags=49%, list=24%, signal=39%	RFC1/VCP/SPAST/DDX49/ERCC3/CHD5/MCM5/RAD50/TOR2A/WRN/DDX52/KIF22/SMC3/ATP8A2/CHD1L/CLPX/BLM/TRIP13/RUVBL1/RECQL4/KIF23/KIF3A

											/SMC1B/RFC4/ATF7IP/HSPA14/DHX30/CLPB/RAD51/DNAH8/DHX16/HSPA2/HSPA1L/KATNA1/SMCHD1/DDX20/ATAD2
GOBP_REGULATION_OF_ANATOMICAL_STRUCTURE_MORPHOGENESIS	GOBP_REGULATION_OF_ANATOMICAL_STRUCTURE_MORPHOGENESIS	GOBP_REGULATION_OF_ANATOMICAL_STRUCTURE_MORPHOGENESIS	187	0.228387419	2.135698513	0.000107988	0.002636111	0.001998195	1442	tags=61%, list=46%, signal=35%	MSN/PARVA/RALA/PHB2/DAB2/MED12/XBP1/PQBP1/COL4A2/PGK1/ZMPSTE24/ABL1/EMP2/SMO/VEGFB/CYP1B1/PPP3CA/PLXNA1/STAT3/RAC1/CD34/STAB1/GADD45A/C3AR1/NFE2L2/RHOU/ITGB1/CYFIP1/SULF1/HIPK2/BMP4/PLEKHA4/APCDD1/ALOX5/CITED2/SNAI2/CDK5/ADAM12/CDC42EP5/RAMP2/PALM/BMP2/TEK/FOXC1/SDC2/CHRNB2/CTSH/OR10J5/ANGPTL4/KIF13B/PAX9/ARHGEF7/PHLDB2/FZD4/CLDN5/LARP4/GNA12/CXCL13/LFNG/ITGA5/FGF1/LRG1/ENG/FRS2/ZDHHC6/ADM/WNT11/HSPB1/MAGED1/ZMYM3/KLF2/NRP1/CLASP2/SLC26A5/OMA1/DVL2/RHOG/MMRN2/HUWE1/CUL7/CDH4/ULK1/ITGB8/FGF13/ROCK2/TNFSF13B/FBXW8/NFATC4/CLDN4/PARVB/GDI1/WNT2B/ETS1/DIAPH1/ABI3/ATF2/GHSR/CX3CR1/AQP1/DLC1/ADA/RALBP1/RNH1/THY1/CCBE1/DLG4/NPHP3/SEC24B/C3/MT3/CAV3/WNT3A/F11R/DAB1/FIS1
GOBP_RESPONSE_TO_HORMONE	GOBP_RESPONSE_TO_HORMONE	GOBP_RESPONSE_TO_HORMONE	194	0.226002956	2.135643949	0.000107664	0.002636111	0.001998195	1576	tags=65%, list=50%, signal=35%	SHC1/FBN1/NFKB1/TYK2/CTSK/KLF9/AKR1C1/TNFSF10/TFPI/PHB2/DAB2/MAOB/IGF2/XBP1/HCLS1/CYP1B1/GLB1/CAT/PPP3CA/SOCS2/BTG2/TSPO/ATP1A2/COL3A1/STAT3/INHBA/PTPRC/NFE2L2/CYFIP1/NQO1/CITED2/CCL21/FECH/TSC1/MAS1L/PRMT2/RAMP2/TEK/DHCR24/ZBTB7A/ALAD/SLC34A2/CTSH/CYP11A1/BCL2/AANAT/VPS13C/PTPN2/MMP14/DENND4C/LYN/GPR83/SCGB1A1/EIF2B1/RELA/BCHE/FAM107A/NR3C1/YAP1/ITGA3/GNAI1/FOSL2/ADM/NCOA3/GCLC/GPR21/LTA4H/LMO2/ARPC1B/MDM2/CRHR2/NR1H2/UCN3/INPPL1/SLC26A5/INS/ACSL1/OTOP1/PDK2/GRB7/ADIPOR2/PRKAR1A/ROCK2/FS

											HR/GNB1/VPS11/NPAS4/CLDN4/SOCS1/DDX54/ADIPOQ/UGCG/ACTN2/GHSR/UBE2L3/AQP1/EGR1/TSHB/ADIPOR1/CSK/HTR1B/UMODL1/TIMP1/VPS18/TRPV1/ANG/SSTR3/CSHL1/AKT1/SH2B2/MT3/CA2/RARG/THRA/MYOD1/PLA2G1B/GLP1R/WNT1/LBH/CAD/ARID1B/SLC12A3/PAQR8/RWDD1/ATP1A3/PPARA/MSI1
GOMF_3_5_EXONUCLEASE_ACTIVITY	GOMF_3_5_EXONUCLEASE_ACTIVITY	GOMF_3_5_EXONUCLEASE_ACTIVITY	10	-0.673041028	-2.489002689	0.00011092	0.002680482	0.002031828	749	tags=90%, list=24%, signal=69%	CNOT6/RAD50/ANGEL1/WRN/CNOT7/POLE/PNPT1/POLRMT/EXOSC4
GOBP_POSITIVE_REGULATION_OF_CYTOKINE_PRODUCTION	GOBP_POSITIVE_REGULATION_OF_CYTOKINE_PRODUCTION	GOBP_POSITIVE_REGULATION_OF_CYTOKINE_PRODUCTION	93	0.28722542	2.226708982	0.000110582	0.002680482	0.002031828	1411	tags=66%, list=45%, signal=37%	TYK2/PYCARD/CEBPB/XBP1/PQBP1/HMGB1/IFNGR1/TYROBP/ABL1/HAVCR2/H19/AIF1/CYP1B1/RAB1A/EIF2AK2/STAT3/CD34/HLA-E/HLA-F/HEG1/PTPRC/C3AR1/SULF1/HLA-A/ATF4/LUM/IL15/IRAK1/SLC11A1/ISG15/MEFV/AIRE/FCER1G/RELA/KIR2DL4/CD276/IL17A/WNT11/HSPB1/AGER/INS/CD4/SPN/OAS2/CD83/ROCK2/DDX3X/NFATC4/ADIPOQ/ATF2/TNXB/EGR1/UNC93B1/LTA/FFAR2/CHIA/CCBE1/C3/GPSM3/WNT3A/LTB
GOBP_SISTER_CHROMATID_SEGREGATION	GOBP_SISTER_CHROMATID_SEGREGATION	GOBP_SISTER_CHROMATID_SEGREGATION	57	-0.326611188	-2.415848137	0.000111653	0.002684701	0.002035027	921	tags=60%, list=29%, signal=43%	MAPRE1/SMC4/CHEK2/LCMT1/CDC16/UBE2C/NUSAP1/BCCIP/ZW10/SMARCC1/AKAP8L/CDK5RAP2/KIF22/KIF4A/SMC3/MAD2L1BP/PINX1/TUBG2/BRD7/ESPL1/MAD2L2/DYNC1LI1/KPNB1/TPX2/TRIP13/KIF23/TUBG1/CENPE/BUB1B/KATNB1/TPR/AURKC/CDCA8/SPAG5
HP_PROMINENT_FOREHEAD	HP_PROMINENT_FOREHEAD	HP_PROMINENT_FOREHEAD	60	0.344466207	2.356456186	0.000117095	0.002801544	0.002123594	800	tags=50%, list=25%, signal=38%	FBN1/SUMF1/SETBP1/RALA/FGFR1/SH3PXD2B/PDGFRB/FAM50A/DPH5/IGF2/MED12/H19/STAT3/MBTPS1/GNE/MID1/CBFB/PURA/DHCR24/ZBTB7A/EDA/FOXC1/ELN/PLAG1/AP2M1/PRPS1/COL27A1/CDKN1C/ITGA3/PTDSS1
GOBP_NEGATIVE_REGULATION_OF_HEMOPOIESIS	GOBP_NEGATIVE_REGULATION_OF_HEMOPOIESIS	GOBP_NEGATIVE_REGULATION_OF_HEMOPOIESIS	26	0.477351848	2.413609375	0.000121449	0.002891323	0.002191648	722	tags=65%, list=23%, signal=51%	FBN1/LAG3/HMGB1/MAFB/C1QC/INHBA/MYC/CEBPA/BMP4/CBFB/SNAI2/PIAS3/FGL2/ID2/PTPN2/LYN/TCTA
GOBP_REGULATI	GOBP_REGULATI	GOBP_REGULATION_O	30	0.446784138	2.371883967	0.00012225	0.00289610	0.00219527	860	tags=63%,	PHB2/DAB2/MED12/ABL1/SMO/RAC1/

ON_OF_ANIMAL_ORGAN_MORPHOGENESIS	ON_OF_ANIMAL_ORGAN_MORPHOGENESIS	F_ANIMAL_ORGAN_MORPHOGENESIS				2	3			list=27%, signal=47%	CD34/SULF1/BMP4/PLEKHA4/APCDD1/CITED2/SNAI2/BMP2/PAX9/FGF1/FRS2/WNT11/MAGED1
GOBP_PROTEIN_DNA_COMPLEX_ASSEMBLY	GOBP_PROTEIN_DNA_COMPLEX_ASSEMBLY	GOBP_PROTEIN_DNA_COMPLEX_ASSEMBLY	25	-0.469219759	-2.603833251	0.000123206	0.00290441	0.002201567	806	tags=68%, list=26%, signal=51%	MED4/ASF1B/TAF9/TAF6/TAF10/BAZ1A/CENPH/ITGB3BP/UBN1/CHAF1B/NAP1L4/CENPE/ATF7IP/SUGT1/RAD51/GMNN/DR1
GOBP_REGULATION_OF_DNA_METABOLIC_PROCESS	GOBP_REGULATION_OF_DNA_METABOLIC_PROCESS	GOBP_REGULATION_OF_DNA_METABOLIC_PROCESS	113	-0.247965216	-2.31574981	0.000124277	0.002915362	0.00220987	833	tags=46%, list=26%, signal=35%	PPP4C/CIDEA/ENDOG/PARP1/ATR/MAP3K4/SIRT1/CEBPG/TIMELESS/TAF9/MCM5/RAD50/TAF6/TP53BP1/POT1/SMARCC1/TAF10/WRN/YLPM1/TTF1/BAZ1A/TFDP1/CDC7/CCDC117/SMC3/PINX1/BRD7/TRIM28/BARD1/MAD2L2/PCNA/MAPK3/RLF/USP37/BLM/SMC6/PAXIP1/RUVBL1/USP1/GFER/UBE2N/RFC4/POLG2/GTPBP4/FAF1/TFIP11/RAD51/GMNN/SUPT3H/RAD17/SMCHD1/KPNA2
GOBP_MORPHOGENESIS_OF_A_BRANCHING_STRUCTURE	GOBP_MORPHOGENESIS_OF_A_BRANCHING_STRUCTURE	GOBP_MORPHOGENESIS_OF_A_BRANCHING_STRUCTURE	37	0.406426977	2.337862362	0.000127373	0.002973482	0.002253925	1153	tags=70%, list=37%, signal=45%	PHB2/ABL1/SMO/GLI2/PLXNA1/ILK/MYC/SULF1/BMP4/SNAI2/BMP2/CTSH/BCL2/MMP14/COL4A1/YAP1/FGF1/ENG/FRS2/ADM/MAGED1/NRP1/FGF13/GCM1/NFATC4/WNT2B
GOBP_HEART_MORPHOGENESIS	GOBP_HEART_MORPHOGENESIS	GOBP_HEART_MORPHOGENESIS	51	0.372865107	2.418066716	0.000139077	0.003231016	0.002449138	1407	tags=75%, list=45%, signal=42%	PARVA/ZMPSTE24/EMP2/SMO/ILK/HEG1/HAND2/FKBP1A/ADAMTS5/BMP4/NKX2-5/CITED2/ZFPM2/SNAI2/ADAM15/BMP2/TEK/FOXC1/ELN/HAS2/NRP2/CLDN5/YAP1/ENG/WNT11/ADPRHL1/MDM2/PKP2/NRP1/DVL2/SMAD7/ATF2/DLC1/TBX6/NPHP3/SEC24B/CAV3/WNT3A
GOBP_ORGANIC_HYDROXY_COMPOUND_METABOLIC_PROCESS	GOBP_ORGANIC_HYDROXY_COMPOUND_METABOLIC_PROCESS	GOBP_ORGANIC_HYDROXY_COMPOUND_METABOLIC_PROCESS	97	0.282843576	2.228367491	0.000142476	0.003294083	0.002496943	1051	tags=52%, list=33%, signal=35%	NFKB1/CTSK/ALDH1A3/AKR1A1/OSBPL5/AKR1C1/DAB2/MAOB/CYP1B1/OSBPL1A/CAT/LIPA/MBTPS1/ITPK1/CEBPA/PLTP/HAND2/NQO1/ALDH2/NFE2L1/ACAA1/SNAI2/FECH/BMP2/DHCR24/PDXK/IDH3B/IDH3G/CHRNB2/CYP11A1/PNPLA4/BCL2/STARD4/LIMA1/CDH3/FGF1/ALG2/LDHA/ATP8B1/AKR7A2/SCP2/NSDHL/ADH1C/PMVK/OSBP/DAO/ITGAM/DUOX2/FMO5/FGF19
GOBP_NEGATIVE_REGULATION_O	GOBP_NEGATIVE_REGULATION_O	GOBP_NEGATIVE_REGULATION_OF_CELL_CY	69	-0.290059516	-2.272881285	0.000144575	0.003326612	0.002521601	744	tags=48%, list=24%,	TIMELESS/RAD50/TP53BP1/ZW10/DCUN1D3/E2F1/CDK5RAP2/RBM14/MAD2L

F_CELL_CYCLE_PROCESS	F_CELL_CYCLE_PROCESS	CLE_PROCESS								signal=37%	1BP/PINX1/THOC5/BRD7/ESPL1/BARD1/MAD2L2/DYNC1LI1/CEP63/BLM/TRIP13/DONSON/INTS7/FBXO7/BUB1B/FZR1/MDC1/RAD51/TPR/CRY1/CDCA8/DOT1L/CDC14B/RAD17/AURKAIP1
HP_ABNORMAL_MAXILLA_MORPHOLOGY	HP_ABNORMAL_MAXILLA_MORPHOLOGY	HP_ABNORMAL_MAXILLA_MORPHOLOGY	38	0.411774507	2.381665691	0.00014544	0.003329332	0.002523662	539	tags=47%, list=17%, signal=40%	FBN1/CTSK/FGFR1/PDGFRB/CDH11/TBC1D2B/MED12/PQBP1/ABL1/MAFB/GLI2/BMP4/CBFB/COLEC11/BMP2/ZDHHC9/EDA/FOXC1
GOBP_LEUKOCYTE_PROLIFERATION	GOBP_LEUKOCYTE_PROLIFERATION	GOBP_LEUKOCYTE_PROLIFERATION	66	0.333182525	2.345021541	0.000146078	0.003329332	0.002523662	694	tags=45%, list=22%, signal=36%	TYK2/MSN/EFNB1/PYCARD/IGF2/CEBPB/HLA-DMB/HMGB1/TYROBP/ABL1/EMP2/HAVCR2/AIF1/PPP3CA/IMPDH2/LIPA/HLA-E/PTPRC/HLA-A/BMP4/IL15/PURA/BCL2L1/CCL5/SLC11A1/CHRNB2/BCL2/BTN2A2/LYN/SCGB1A1
GOMF_DNA_HELICASE_ACTIVITY	GOMF_DNA_HELICASE_ACTIVITY	GOMF_DNA_HELICASE_ACTIVITY	16	-0.551416715	-2.493324654	0.000151745	0.003419777	0.00259222	748	tags=75%, list=24%, signal=57%	ERCC3/MCM5/RAD50/WRN/CHD1L/BLM/RUVBL1/RECQL4/RFC4/DHX30/RAD51/SETX
GOMF_TUBULIN_BINDING	GOMF_TUBULIN_BINDING	GOMF_TUBULIN_BINDING	73	-0.29373501	-2.333157464	0.000152066	0.003419777	0.00259222	939	tags=55%, list=30%, signal=39%	TUBGCP2/SLC6A2/MAPRE1/CEP290/MAP1S/REEP4/SPAST/NUSAP1/CDK5R1/RABGAP1/BBS4/KRIT1/HOOK2/CDK5RAP2/KIF22/KIF4A/PACRG/SMC3/CEP350/HSPH1/CACYBP/SSNA1/MAP6D1/KIFC3/NDE1/IFT74/KIF23/MAST2/CENPE/KIF3A/KATNB1/EFHC1/POLB/TPR/KATNA1/SPAG5/RAE1/STRBP/PAFAH1B1/SPAG8
GOBP_RENAL_SYSTEM_DEVELOPMENT	GOBP_RENAL_SYSTEM_DEVELOPMENT	GOBP_RENAL_SYSTEM_DEVELOPMENT	73	0.314642858	2.284733194	0.00015218	0.003419777	0.00259222	1389	tags=66%, list=44%, signal=38%	FBN1/NFIA/PDGFRB/IQGAP1/ZMPSTE24/SMO/GLI2/CAT/PPP3CA/CD34/ILK/MYC/SULF1/BMP4/WFS1/TSC1/BMP2/TEK/FOXC1/MPV17/CTSH/BCL2/PODXL/HAS2/COL4A1/YAP1/CDKN1C/ITGA3/SEC61A1/FGF1/WNT11/MAGED1/RRM2B/NRP1/BAX/APH1A/TTC8/SNX17/WNT2B/SMAD7/ADIPOQ/AQP1/MMP17/EGR1/PCSK5/PDGFRA/NPHP3/ZBTB16
GOBP_MULTICELLULAR_ORGANISMAL_LEVEL_HOMEOSTASIS	GOBP_MULTICELLULAR_ORGANISMAL_LEVEL_HOMEOSTASIS	GOBP_MULTICELLULAR_ORGANISMAL_LEVEL_HOMEOSTASIS	161	0.230272554	2.09060749	0.000155543	0.003479099	0.002637187	1427	tags=60%, list=45%, signal=35%	CTSK/CYBRD1/CEBPB/TFE3/HMGB1/ABL1/SMO/HCLS1/GPRASP2/EIF2AK1/MAFB/ARMCX1/SLC25A5/COL3A1/LIPA/STAT3/CD34/INHBA/EIF4G1/ADAMTS5/ITGB1/HIPK2/BMP4/ATF4/MTHFD1/MUC2/FMO1/CITED2/WFS1/PTGES/IL15/

HP_X_LINKED_INHERITANCE	HP_X_LINKED_INHERITANCE	HP_X_LINKED_INHERITANCE	53	0.349131114	2.275382547	0.000157714	0.003511316	0.002661608	1151	tags=58%, list=37%, signal=38%	FECH/AMPD2/PRDX1/EBF2/ZBTB7A/FOXC1/SLC11A1/CTSH/TFF3/ABHD6/ISG15/BCL2/HAS2/ID2/PTPN2/LYN/NOVA1/TRPM2/CLDN5/PRKAB2/OCIAD2/YAP1/CDH3/PPP2R1A/FOSL2/PRR4/IL17A/WDR37/TMEM119/HSPB1/SQSTM1/CRTC3/KLF2/NR1H2/OMA1/ACSL1/BAX/HEATR3/EHMT1/ADIPOR2/TNFSF13B/GPR3/ALAS1/ETS1/APBB2/CROCC/ADIPOQ/CX3CR1/SGIP1/TSC22D3/AQP1/EGR1/ADIPOR1/CSK/ADA/AQP6/OCIAD1/MFHAS1/EMX1/TRPV1/AKT1/NPHP3/SH2B2/F11R/ACOT11/LTF	RNF113A/MSN/UBQLN2/EFNB1/FAM50A/MED12/TFE3/PQBP1/PGK1/COX7B/GPRASP2/COL4A5/SSR4/MID1/COL4A6/ABCB7/ZDHHC9/EDA/PRPS1/LAGE3/EMD/STAG2/NSDHL/FHL1/HUWE1/SLITRK2/FGF13/DDX3X/SMC1A/CASK/GDI1
GOBP_NEGATIVE_REGULATION_OF_MONOATOMIC_ION_TRANSPORT	GOBP_NEGATIVE_REGULATION_OF_MONOATOMIC_ION_TRANSPORT	GOBP_NEGATIVE_REGULATION_OF_MONOATOMIC_ION_TRANSPORT	23	0.48433048	2.302464861	0.000159293	0.003513951	0.002663605	1405	tags=87%, list=45%, signal=49%	MAOB/GSTO1/VDAC1/PPP3CA/ATP1A2/FKBP1A/ATF4/KCNE3/BCL2/HRH3/SRI/SERPINE2/CALM3/SLC26A5/PPIF/ACTN2/HTR1B/PXK/OXSR1/CAV3	
GOBP_REGULATION_OF_MONOATOMIC_ION_TRANSPORT	GOBP_REGULATION_OF_MONOATOMIC_ION_TRANSPORT	GOBP_REGULATION_OF_MONOATOMIC_ION_TRANSPORT	104	0.275045298	2.20791425	0.000158903	0.003513951	0.002663605	1574	tags=70%, list=50%, signal=36%	PDGFRB/PHB2/MAOB/ABL1/GSTO1/VDAC1/PPP3CA/CLIC1/TSPO/ATP1A2/TPCN1/FXYD5/FKBP1A/ITGB1/ATF4/NKX2-5/KCNK5/KCNE3/WFS1/TMEM109/KCNB1/CCL5/BCL2/HRH3/SRI/LYN/F2RL3/STOM/ATP8B1/SERPINE2/PKP2/CCR1/CLIC5/CALM3/SLC26A5/PPIF/LIME1/CD4/ABCB1/BAX/KCNH4/FHL1/KCNB2/KCNJ1/FGF13/CABP5/CASK/KCNAB2/DIAPH1/ACTN2/HTR1B/KCNJ6/KCNH7/PXK/P2RX2/THY1/HCN4/AKT1/OXSR1/CAV3/CA2/ATG5/CACNA2D3/HCN3/PLA2G1B/CACNA1B/KCNQ3/KCNS2/CACNA1E/ATP1B1/CACNB3/GRM6/ATP2A1	
GOBP_POSITIVE_REGULATION_OF	GOBP_POSITIVE_REGULATION_OF	GOBP_POSITIVE_REGULATION_OF_LEUKOCY	34	0.415295554	2.320264537	0.000160923	0.0035337	0.002678575	856	tags=59%, list=27%,	TYK2/EFNB1/PYCARD/IGF2/HLA-DMB/HMGB1/HAVCR2/AIF1/PPP3CA/HLA-E	

_LEUKOCYTE_PROLIFERATION	_LEUKOCYTE_PROLIFERATION	TE_PROLIFERATION								signal=43%	/PTPRC/HLA-A/IL15/BCL2L1/CCL5/CHRNB2/BCL2/LYN/CD276/AGER
GOCC_9PLUS2_MOTILE_CILIUM	GOCC_9PLUS2_MOTILE_CILIUM	GOCC_9PLUS2_MOTILE_CILIUM	21	-0.493702338	-2.537987921	0.000164563	0.003561915	0.002699962	598	tags=62%, list=19%, signal=51%	IQCG/PACRG/CAPZB/DNAI1/LYZL6/SPACA3/DNAH17/AKAP4/DNAH8/TCP11/SPATA6/DNALI1/SPA17
GOBP_TELOMERE_ORGANIZATION	GOBP_TELOMERE_ORGANIZATION	GOBP_TELOMERE_ORGANIZATION	31	-0.415307538	-2.474695354	0.000165559	0.003561915	0.002699962	992	tags=74%, list=31%, signal=51%	HMBOX1/RIF1/HAT1/MCRS1/RFC1/PARP1/ATR/MAP3K4/RAD50/POT1/WRN/YLPM1/PINX1/MAD2L2/PCNA/MAPK3/BLM/SMC6/RUVBL1/RECQL4/TFIP11/RAD51/DOT1L
GOBP_EXTERNAL_ENCAPSULATING_STRUCTURE_ORGANIZATION	GOBP_EXTERNAL_ENCAPSULATING_STRUCTURE_ORGANIZATION	GOBP_EXTERNAL_ENCAPSULATING_STRUCTURE_ORGANIZATION	63	0.319625377	2.229196445	0.000163507	0.003561915	0.002699962	810	tags=49%, list=26%, signal=37%	CTSK/SH3PXD2B/COL4A2/ABL1/TGFBI/CYP1B1/ABI3BP/COL3A1/COL4A5/LOXL2/ADAMTS5/ITGB1/SULF1/COL4A6/LUM/MMP23B/ADAM15/RAMP2/BMP2/COL15A1/FOXC1/MPV17/ELN/PAPLN/HAS2/PHLDB2/MMP14/COL4A1/COL27A1/IMPG2/ENG
GOBP_SENSORY_PERCEPTION	GOBP_SENSORY_PERCEPTION	GOBP_SENSORY_PERCEPTION	154	0.243698246	2.182562499	0.000165439	0.003561915	0.002699962	1525	tags=65%, list=48%, signal=35%	SCARB2/TGFBI/NOB1/LAMC3/OR51A7/OR5V1/OR52E4/NAV2/HPS1/LUM/TRIOBP/OTOR/SNAI2/TAS2R39/WFS1/PTGES/OR9K2/OR13C4/CDK5/OR10T2/OR6V1/OR8G1/CHRNB2/OR10J5/TAS2R9/OR6C4/OR1E2/OR5F1/OR52D1/FZD4/OAT/OR8A1/OR10H4/OR4K17/OR8H2/GRM1/CDH3/OR6X1/IMPG2/PRR4/ATP8B1/CRYGA/SERPINE2/OR8D1/OR52B6/TAS2R40/TAS2R10/OR4K14/CLIC5/SLC26A5/OR7G2/OR6Y1/OR3A3/TAS2R41/LCTL/OR5AS1/OR5D18/OR4S1/OR2T4/OR7D2/OR6M1/OR6K2/GNB1/OR2A12/TTC8/OR8B8/OR8S1/MRGPRX2/OR6B2/DIAPH1/OR51S1/GRK1/AQP1/OR2J2/OR1D4/OR51L1/PDCL/OR52M1/REEP2/OR13C8/TAS2R60/OR4D5/OR1D2/OR4M1/OR1F1/TAAR5/TRPV1/FXN/P2RX2/OR4D1/EML2/OR10A4/OR10H3/OR4P4/OR2B11/KCNQ3/GJC1/SEMA5B/CLN8/B3GNT2
GOBP_ANATOMICAL_STRUCTURE_FORMATION_INVOLVED_IN_MORPHOGENESIS	GOBP_ANATOMICAL_STRUCTURE_FORMATION_INVOLVED_IN_MORPHOGENESIS	GOBP_ANATOMICAL_STRUCTURE_FORMATION_INVOLVED_IN_MORPHOGENESIS	256	0.197658582	1.962889868	0.000165912	0.003561915	0.002699962	1407	tags=57%, list=45%, signal=34%	SHC1/SH3PXD2A/ALDH1A3/PARVA/RALA/PDGFRB/CDH11/MED12/XBP1/COL4A2/PGK1/TYROBP/ABL1/EMP2/TGFBI/SMO/ETS2/VEGFB/CYP1B1/MAFB/GLI2/PERP/TMOD1/COL3A1/PLXNA1/ST

											AT3/ITGB1BP1/CD34/LOXL2/INHBA/STAB1/GADD45A/C3AR1/NFE2L2/HAND2/FKBP1A/ADAMTS5/ITGB1/AHDC1/SULF1/HIPK2/TGFA/BMP4/NKX2-5/MTHFD1/ALOX5/HS6ST1/HSBP1/CITED2/SNAI2/TSC1/ADAM15/CDK5/ADAM12/RAMP2/BMP2/TEK/COL15A1/EDA/FOXC1/CTSH/SLC9A8/OR10J5/ANGPTL4/SLC31A1/PODXL/NRP2/PHLDB2/MMP14/FZD4/TMOD4/COL4A1/CLDN5/TCTA/CXCL13/LFNG/COL27A1/YAP1/GPR15/ITGA3/ITGA5/APOD/APLN/FGF1/LRG1/ENG/FRS2/EOMES/ADM/WNT11/HSPB1/ADPRHL1/ATP8B1/MIXL1/CXCR3/KLF2/CEND1/NRP1/CLASP2/CDK5R2/CNTNAP1/WDR74/DVL2/CLEC14A/MMRN2/SSBP3/ADIPOR2/ITGB8/PRKAR1A/ROCK2/TNFSF13B/MYOG/GCM1/FAM20C/MMP15/NFATC4/WNT2B/ETS1/OBSCN/ACTN2/ATF2/GHSR/CX3CR1/AQP1/LMO4/DLC1/ADRA2B/ADA/CFL2/TPPP/TBC1D20/TBX6/RNH1/ANG/THY1/SUPT6H/PDGFRA/NOTCH4/AKT1/CCBE1/NPHP3/SEC24B/E2F7/C3/CAV3/WNT3A
GOBP_OSSIFICATION	GOBP_OSSIFICATION	GOBP_OSSIFICATION	80	0.308073946	2.290944688	0.000170015	0.003617708	0.002742253	1452	tags=70%, list=46%, signal=39%	CTSK/LRP3/CDH11/IGF2/CEBPB/MGP/ZMPSTE24/IFITM1/SMO/CHRD/GLI2/CAT/PPP3CA/CLIC1/ILK/CEBPA/HAND2/BMP4/PTN/ATF4/ALOX5/MYOC/CBFB/SNAI2/BMP2/TEK/FOXC1/ISG15/MEF2D/BCL2/MMP14/CBS/DNAJC13/YAP1/FOSL2/WNT11/TMEM119/KLF10/RPS15/CCR1/INPPL1/SLC24A3/MYOG/OSTF1/FAM20C/NOTUM/SMAD7/KREMEN1/MEN1/JUND/AKT1/ZBTB16/WNT3A/LTF/FIGNL1/THRA
GOBP_MITOTIC_CELL_CYCLE_PROCESS	GOBP_MITOTIC_CELL_CYCLE_PROCESS	GOBP_MITOTIC_CELL_CYCLE_PROCESS	177	-0.203426449	-2.093712008	0.000169545	0.003617708	0.002742253	700	tags=37%, list=22%, signal=30%	ZW10/SMARCC1/DCUN1D3/TAF10/AKAP8L/E2F1/USP8/CDK5RAP2/KIF22/KIF4A/TFDP1/ECD/CDC7/SMC3/MAD2L1BP/PINX1/TUBG2/POLE/BRD7/ESPL1/BARD1/MAD2L2/DYNC1LI1/PCNA/KPNB1/MYH10/CENPH/CDK7/USP37/TPX2/BLM/TRIP13/NDE1/KIF23/VRK1/PPP6C/D

											ONSON/TUBG1/UBE2S/FBXO7/CENPE/BUB1B/FZR1/KATNB1/MDC1/EFHC1/RAD51/CKS2/TPR/AURKC/CCNH/CDCA8/HSPA2/RANBP1/CDC14B/CDC25C/RAD17/SPAG5/CCNB2/RAE1/AURKAIP1/PAFAH1B1/KLF11/WDR62/CPSF3
GOMF_ATP_DEPENDENT_ACTIVITY_ACTING_ON_DNA	GOMF_ATP_DEPENDENT_ACTIVITY_ACTING_ON_DNA	GOMF_ATP_DEPENDENT_ACTIVITY_ACTING_ON_DNA	23	-0.474439706	-2.514503124	0.000180727	0.003828715	0.002902198	748	tags=65%, list=24%, signal=50%	ERCC3/CHD5/MCM5/RAD50/WRN/CHD1L/CDK7/BLM/RUVBL1/RECQL4/RFC4/DHX30/RAD51/SETX/RAD17
GOBP_DETOXIFICATION	GOBP_DETOXIFICATION	GOBP_DETOXIFICATION	32	0.430617976	2.340518305	0.00018789	0.00396299	0.00300398	613	tags=53%, list=19%, signal=43%	AKR1A1/GSTO1/LTC4S/H19/ALOX5AP/CAT/HP/NFE2L2/NQO1/MUC2/MT1B/PTGES/MT1E/PRDX1/CCS/SLC11A1/MT1H
GOBP_POSITIVE_REGULATION_OF_CELL_DIFFERENTIATION	GOBP_POSITIVE_REGULATION_OF_CELL_DIFFERENTIATION	GOBP_POSITIVE_REGULATION_OF_CELL_DIFFERENTIATION	171	0.222693475	2.052326446	0.00019056	0.00400176	0.003033369	1262	tags=54%, list=40%, signal=34%	NFKB1/FGFR1/LRP3/DAB2/CEBPB/TFE3/XBP1/HMGB1/TYROBP/EMP2/IFITM1/SMO/HCLS1/GLI2/PPP3CA/NAP1L1/CLIC1/SOCS2/TSPO/PLXNA1/STAT3/CD34/LOXL2/INHBA/ILK/PTPRC/CEBPA/EIF4G1/ITGB1/CYFIP1/MAP3K5/BMP4/PTN/NKX2-5/ADD1/PRKCH/CBFB/SNAI2/IL15/CCDC3/RAMP2/BMP2/FOXC1/ISG15/BCL2/BTN2A2/ID2/PLAG1/MMP14/FZD4/LYN/RELA/MAML1/CLDN5/SIRT2/YAP1/LRG1/ENG/IL17A/ADM/TMEM119/NCOA3/AGER/SERPINE2/MDM2/KLF10/NRP1/BAD/CCR1/INS/CD4/PTCH2/CD83/CUL7/CDH4/S100B/MYOG/FAM20C/FBXW8/ARRB2/SOCS1/GDI1/SMAD7/ETS1/ADIPOQ/TNXB/CX3CR1/RGS14/ZNF703/ASCL1/ADRA2B/ADA
GOBP_INORGANIC_ION_HOMEOSTASIS	GOBP_INORGANIC_ION_HOMEOSTASIS	GOBP_INORGANIC_ION_HOMEOSTASIS	109	0.265493421	2.157715503	0.000192122	0.004017023	0.003044938	1758	tags=73%, list=56%, signal=34%	CCDC115/CYBRD1/ABL1/GSTO1/EIF2AK1/ITPR1/SLC25A23/ATP1A2/MYC/PTPRC/TPCN1/ACO1/SLC30A9/SCARA5/FKBP1A/ABCB7/ANXA6/ATF4/SLC12A9/KCNE3/CCL21/WFS1/MT1B/FECH/CLN3/MT1E/CCL5/SLC11A1/SLC34A2/BCL2/SLC31A1/MT1H/SRI/IBTK/LYN/TRPM2/ATP2A3/F2RL3/GRM1/CCR5/CCR1/CALM3/LIME1/BAX/SLC24A3/CNNM2/GPR3/CCL8/DIAPH1/VAPB/SYPL2/HTR1B/NUBP1/XCR1/TRPV1/FXN/THY1/MT3/

											CAV3/S100A14/ATG5/FIS1/ATP13A1/SLC34A3/SLC12A8/ATP1B1/SLC12A3/SLC17A7/ATP1A3/ATP2A1/TRPV5/BDKRB1/SLC17A8/SV2A/GPR12/SLC12A1/ATP7B/STIM1/ATP2B3/GP1BB
GOCC_MALE_GERM_CELL_NUCLEUS	GOCC_MALE_GERM_CELL_NUCLEUS	GOCC_MALE_GERM_CELL_NUCLEUS	18	-0.541639751	-2.592684622	0.00019492	0.004057888	0.003075913	686	tags=72%, list=22%, signal=57%	SMARCC1/TAF10/PCNA/CDK7/TRIP13/ACTL7A/RAD51/HSPA2/DAZAP1/TSN/TCFL5/SPAG8/TBPL1
GOBP_POSITIVE_REGULATION_OF_CELL_SUBSTRATE_ADHESION	GOBP_POSITIVE_REGULATION_OF_CELL_SUBSTRATE_ADHESION	GOBP_POSITIVE_REGULATION_OF_CELL_SUBSTRATE_ADHESION	30	0.434450415	2.306406797	0.000200696	0.00416012	0.003153407	781	tags=60%, list=25%, signal=46%	DAB2/ABL1/EMP2/ABI3BP/RAC1/ITGB1BP1/RSU1/ILK/MYOC/TRIOBP/CCL21/TSC1/MYADM/TEK/HAS2/ARHGEF7/ITGA3/ITGA5
HP_ABNORMALITY_OF_PRIMARY_TEETH	HP_ABNORMALITY_OF_PRIMARY_TEETH	HP_ABNORMALITY_OF_PRIMARY_TEETH	23	0.476563296	2.265540345	0.000202706	0.004183758	0.003171324	654	tags=57%, list=21%, signal=45%	RNF113A/CTSK/FGFR1/ZMPSTE24/COL3A1/STAT3/TGFA/CBFB/PURA/ZBTB7A/EDA/PAX9/CTSC
HP_HEMOPTYSIS	HP_HEMOPTYSIS	HP_HEMOPTYSIS	20	0.514353054	2.320783989	0.000212162	0.004340202	0.00328991	930	tags=75%, list=29%, signal=53%	FBN1/IFNGR1/EIF2AK4/COL3A1/HLA-B/BTNL2/TSC1/SLC11A1/SLC34A2/ELN/MEFV/SERPINA1/ENG/GCLC/CCR1
GOBP_GLAND_MORPHOGENESIS	GOBP_GLAND_MORPHOGENESIS	GOBP_GLAND_MORPHOGENESIS	30	0.433028226	2.29885669	0.00021195	0.004340202	0.00328991	966	tags=67%, list=31%, signal=47%	NFKB1/MSN/PHB2/CEBPB/XBP1/LIPA/PLXNA1/SULF1/TGFA/BMP4/PTN/SNAI2/EDA/BCL2/PLAG1/NR3C1/FGF1/FRS2/NRP1/BAX
GOBP_ACTIVATION_OF_IMMUNE_RESPONSE	GOBP_ACTIVATION_OF_IMMUNE_RESPONSE	GOBP_ACTIVATION_OF_IMMUNE_RESPONSE	95	0.276582788	2.164411887	0.000212994	0.004340202	0.00328991	966	tags=51%, list=31%, signal=36%	NFKB1/PHB2/LSM14A/PYCARD/PQBP1/HMGB1/TYROBP/ABL1/HAVCR2/CFHR3/HLA-DRB3/EIF2AK2/HLA-DQB1/MAPKAPK3/PLSCR1/LIPA/C1QC/PUM1/PTPRC/C3AR1/BTNL2/HLA-A/FLOT2/CFHR5/NMI/PRKCH/COLEC11/CYLD/IRAK1/BCL2/MEFV/BTN2A2/PTPN2/SVEP1/LYN/EIF2B1/FCER1G/BRCC3/RELA/BTN1A1/SIRT2/FOSL2/CD276/C8A/ZNFX1/NFKBIL1/LIME1/BAX
GOCC_ER_TO_GOLGI_TRANSPORT_VESICLE_MEMBRANE	GOCC_ER_TO_GOLGI_TRANSPORT_VESICLE_MEMBRANE	GOCC_ER_TO_GOLGI_TRANSPORT_VESICLE_MEMBRANE	19	0.523105899	2.311298993	0.000216426	0.00438015	0.003320191	353	tags=53%, list=11%, signal=47%	SEC22B/HLA-DRB3/HLA-DQB1/HLA-B/HLA-E/HLA-F/HLA-DRB5/HLA-A/HLA-C/TGFA
HP_ABNORMAL_GASTROINTESTINAL_MOTILITY	HP_ABNORMAL_GASTROINTESTINAL_MOTILITY	HP_ABNORMAL_GASTROINTESTINAL_MOTILITY	22	0.489197414	2.290601241	0.000216776	0.00438015	0.003320191	905	tags=64%, list=29%, signal=46%	CDK4/GMPPA/SMO/ATP1A2/STAT3/COL4A5/COL4A6/WFS1/SLC11A1/PPOX/SERPINA1/GCLC/RRM2B/MDM2
GOMF_GROWTH_FACTOR_BINDING	GOMF_GROWTH_FACTOR_BINDING	GOMF_GROWTH_FACTOR_BINDING	25	0.469466055	2.324390485	0.000223944	0.004492726	0.003405524	919	tags=64%, list=29%, signal=46%	SHC1/FGFR1/PDGFRB/RHBDF2/IGFBP4/HTRA1/COL3A1/IGFBP6/IGF2R/TEK/NRP2/COL4A1/CXCL13/LTBP1/ENG/NRP

										1
GOBP_MONOATOMIC_ION_TRANSMEMBRANE_TRANSPORT	GOBP_MONOATOMIC_ION_TRANSMEMBRANE_TRANSPORT	GOBP_MONOATOMIC_ION_TRANSMEMBRANE_TRANSPORT	191	0.214152316	2.01414035	0.000224216	0.004492726	0.003405524	1595	tags=65%, list=51%, signal=34%	LASP1/COX7A1/PHB2/ABL1/GSTO1/COX7B/VDAC1/COX8A/ITPR1/TMCO3/PPP3CA/SLC25A23/CLIC1/CLCC1/SLC25A5/ATP1A2/PTPRC/TPCN1/SLC30A9/SCARA5/CCDC51/FXYD5/FKBP1A/ITGB1/STEAP3/ABCB7/ANXA6/SLC25A12/KCNK5/SLC12A9/KCNE3/CCL21/TMEM109/KCNB1/SLC25A37/OTOP3/SLC11A1/CHRNB2/SLC34A2/SLC9A8/SLC26A1/GRIA4/BCL2/SLC31A1/SRI/IBTK/LYN/TRPM2/ATP2A3/GRIK5/F2RL3/COX15/SEC61A1/STOM/CCR5/SLC6A17/ATP8B1/TMEM63A/CLIC5/CALM3/SLC26A5/PPIF/LIME1/ABCB1/BAX/OTOP1/KCNH4/FHL1/GABRR1/SLC24A3/CNNM2/OTOP2/ABCC10/KCNB2/KCNJ1/NIPA2/FGF13/CABP5/CLDN4/KCNAB2/DIAPH1/SFXN3/ACTN2/ATP6V1G2/SLC6A1/AQP1/KCNJ6/KCNH7/CHRNA2/XCR1/TRPV1/FXN/P2RX2/THY1/HCN4/OXSR1/CAV3/WNT3A/CLCN6/ATG5/CACNA2D3/LTF/HCN3/ATP13A1/PLA2G1B/SLC32A1/ATP6V0A1/CACNA1B/KCNQ3/GJC1/SLC34A3/KCNS2/CACNA1E/SLC12A8/SLC25A13/ATP1B1/SLC12A3/CACNB3/SLC17A7/ATP1A3/GRM6/ATP2A1/TRPV5/HTR3A
GOBP_TELOMERE_MAINTENANCE	GOBP_TELOMERE_MAINTENANCE	GOBP_TELOMERE_MAINTENANCE	28	-0.425864301	-2.477292012	0.000225555	0.0045008	0.003411645	992	tags=75%, list=31%, signal=52%	HMBOX1/RIF1/MCRS1/RFC1/PARP1/ATR/MAP3K4/RAD50/POT1/WRN/YLPM1/PINX1/MAD2L2/PCNA/MAPK3/BLM/SMC6/RUVBL1/RECQL4/TFIP11/RAD51
GOMF_MICROTUBULE_MOTOR_ACTIVITY	GOMF_MICROTUBULE_MOTOR_ACTIVITY	GOMF_MICROTUBULE_MOTOR_ACTIVITY	13	-0.615983943	-2.492463888	0.000236374	0.004658693	0.00353133	620	tags=77%, list=20%, signal=62%	KIF22/KIF4A/SMC3/KIFC3/KIF23/CENPE/KIF3A/DNAH17/DNAH8/DYNLRB2
GOBP_TRANSMEMBRANE_RECEPTOR_PROTEIN_TYROSINE_KINASE_SIGNALING_PATHWAY	GOBP_TRANSMEMBRANE_RECEPTOR_PROTEIN_TYROSINE_KINASE_SIGNALING_PATHWAY	GOBP_TRANSMEMBRANE_RECEPTOR_PROTEIN_TYROSINE_KINASE_SIGNALING_PATHWAY	135	0.244224694	2.100296046	0.000234472	0.004658693	0.00353133	1209	tags=55%, list=38%, signal=35%	SHC1/TYK2/EFNB1/RALA/FGFR1/PDGFRB/RHBDF2/IGFBP4/IGF2/IQGAP1/COL4A2/ABL1/VEGFB/MAPKAPK3/SOCS2/STAT3/COL4A5/RAC1/CYFIP1/SULF1/COL4A6/IGFBP6/TGFA/PPP2R5B/MYOC/IGF2R/GHRH/BMP2/TEK/FOXC1/SLC31A1/NRP2/CPNE3/RAPGEF1/ARHGEF7/PTPN2/FZD4/SVEP1/LYN/RELA/COL4

											A1/CDH3/ITGA5/APOD/APLN/FGF1/NTF3/FRS2/IFI6/HSPB1/GPR21/NRP1/INPPL1/STUB1/INS/CD4/PDK2/MPZL1/CLEC14A/MMRN2/GRB7/FGF19/ERRFI1/AGR2/FAM20C/CD7/NFATC4/SOCS1/KALRN/ADIPOQ/CHMP6/TNXB/GHSR/RGS14
GOBP_GLAND_DEVELOPMENT	GOBP_GLAND_DEVELOPMENT	GOBP_GLAND_DEVELOPMENT	106	0.260098464	2.091530082	0.000235961	0.004658693	0.00353133	1520	tags=69%, list=48%, signal=37%	NFKB1/MSN/ALDH1A3/PHB2/IGF2/CEBPB/XBP1/ZMPSTE24/ABL1/SMO/CYP1B1/MAFB/GLI2/SOCS2/TSPO/LIPA/PLXNA1/CEBPA/HAND2/SULF1/HIPK2/SLC29A1/TGFA/BMP4/PTN/NKX2-5/CITED2/IGF2R/SNAI2/CSN2/GHRH/BMP2/EDA/FOXC1/BCL2/AIRE/PHF2/ID2/PLAG1/RELA/NR3C1/CDKN1C/APLN/FGF1/FRS2/WNT11/SERPINE2/NRP1/BAX/SEC63/OAS2/IQGAP3/ATF2/ZNF703/CRIP1/LMO4/ASCL1/ADA/ELK1/PDGFRA/NOTCH4/AKT1/NPHP3/E2F7/CAV3/WNT3A/RARG/THRA/HOXD3/WNT1/LIMS2/LBH/CAD
GOBP_T_CELL_PROLIFERATION	GOBP_T_CELL_PROLIFERATION	GOBP_T_CELL_PROLIFERATION	45	0.369797674	2.280206596	0.000241401	0.004738348	0.003591708	602	tags=47%, list=19%, signal=38%	TYK2/MSN/EFNB1/PYCARD/IGF2/CEBPB/HLA-DMB/HMGB1/ABL1/HAVCR2/AIF1/PPP3CA/LIPA/HLA-E/PTPRC/HLA-A/BMP4/IL15/CCL5/SLC11A1/BTN2A2
GOBP_POSITIVE_REGULATION_OF_MAPK_CASCADE	GOBP_POSITIVE_REGULATION_OF_MAPK_CASCADE	GOBP_POSITIVE_REGULATION_OF_MAPK_CASCADE	86	0.295420961	2.230583275	0.000243035	0.004751034	0.003601325	1184	tags=57%, list=38%, signal=37%	SHC1/FGFR1/PDGFRB/PHB2/IGFBP4/PYCARD/IGF2/IQGAP1/HMGB1/ABL1/HAVCR2/EIF2AK2/INHBA/GADD45A/PTPRC/HAND2/LTBR/MID1/IGFBP6/HIPK2/MAP3K5/TGFA/BMP4/CCL21/IRAK1/BMP2/TEK/CCL5/GRM1/FGF1/NTF3/FRS2/ERN1/AGER/NRP1/CCR1/MAP2K3/INS/CD4/DVL2/AVPI1/MINK1/FGF19/ROCK2/FSHR/ARRB2/CCL8/IQGAP3/PJA2
GOMF_ANTIGEN_BINDING	GOMF_ANTIGEN_BINDING	GOMF_ANTIGEN_BINDING	24	0.47439623	2.305181785	0.000247346	0.004815742	0.003650374	780	tags=62%, list=25%, signal=47%	LAG3/HLA-DMB/HLA-DRB3/HLA-DQB1/HLA-B/HLA-E/HLA-F/HLA-DRB5/HLA-DMA/HLA-A/HLA-C/DHCR24/HLA-DOB/MAML1/PPP2R1A
HP_NEVUS	HP_NEVUS	HP_NEVUS	39	0.376602277	2.189632548	0.000250317	0.004852151	0.003677972	571	tags=49%, list=18%, signal=40%	FBN1/SETBP1/CDK4/FGFR1/CDH11/IGF2/SMO/H19/KRT14/CYP1B1/COL3A1/BAZ1B/MRAS/GTF2IRD2/HPS1/MYOC/TSC1/TEK/ELN
HP_ABNORMAL_	HP_ABNORMAL_	HP_ABNORMAL_VASC	222	0.206773704	2.011569148	0.00025123	0.00485215	0.00367797	1092	tags=46%,	FBN1/NFKB1/PLOD1/MAF/F10/NFIA/F

VASCULAR_MORPHOLOGY	VASCULAR_MORPHOLOGY	ULAR_MORPHOLOGY				4	1	2		list=35%, signal=32%	GFR1/CCDC8/SH3PXD2B/PDGFRB/OSGEP/DPH5/HTRA1/IGF2/MED12/MGP/PQBP1/COL4A2/IFNGR1/ZMPSTE24/ABL1/EIF2AK4/SMO/COX7B/CYP1B1/ALOX5AP/ITPR1/GLB1/GLI2/CAT/COL3A1/LIPA/PLXNA1/BAZ1B/STAT3/RAC1/HLA-B/SSR4/STT3A/MID1/MRAS/GTF2IRD2/NKX2-5/PLAGL1/MYOC/CITED2/PRKCH/MMP23B/ZFPM2/TPM2/WFS1/TSC1/VWF/BMP2/TEK/DHCR24/ZBTB7A/CTCF/FOXC1/SLC34A2/ELN/MEFV/AIRE/LARS2/ZNF462/MMP14/FZD4/CBS/DNAJC5/GALC/BRCC3/TRIO/COL4A1/NR3C1/SLC29A3/LTBP1/LFNG/PPP2R1A/SEC61A1/PTDSS1/ENG/ALG2/STAG2/WDR37/CHMP2B/ARPC1B/PKP2/NFKBIL1/CCR1/CALM3/HEATR3/TALDO1/SEC63/ABCD4/EHMT1/CUL7/CDC42BPB/UBE3B/NIPA2/PRKAR1A/DDX3X/SMC1A
GOBP_REGULATION_OF_MITOTIC_SISTER_CHROMATID_SEGREGATION	GOBP_REGULATION_OF_MITOTIC_SISTER_CHROMATID_SEGREGATION	GOBP_REGULATION_OF_MITOTIC_SISTER_CHROMATID_SEGREGATION	12	-0.595956286	-2.386752664	0.000254772	0.004881265	0.003700041	700	tags=83%, list=22%, signal=65%	LCMT1/ZW10/CDK5RAP2/MAD2L1BP/MAD2L2/DYNC1LI1/TRIP13/BUB1B/TPR/CDCA8
GOBP_OSTEOBLAST_DIFFERENTIATION	GOBP_OSTEOBLAST_DIFFERENTIATION	GOBP_OSTEOBLAST_DIFFERENTIATION	46	0.361701465	2.253297664	0.000254475	0.004881265	0.003700041	842	tags=54%, list=27%, signal=40%	LRP3/IGF2/CEBPB/IFITM1/SMO/CHRD/GLI2/CAT/PPP3CA/CLIC1/ILK/CEBPA/HAND2/BMP4/ATF4/MYOC/CBFB/SNAI2/BMP2/MEF2D/DNAJC13/YAP1/FOSL2/WNT11/TMEM119
GOBP_NATURAL_KILLER_CELL_ACTIVATION	GOBP_NATURAL_KILLER_CELL_ACTIVATION	GOBP_NATURAL_KILLER_CELL_ACTIVATION	15	0.583805498	2.338273818	0.000258531	0.004933627	0.003739731	494	tags=60%, list=16%, signal=51%	TYK2/TYROBP/EMP2/HAVCR2/HLA-E/HLA-F/PTPRC/IL15/PRDX1
GOBP_AMEBOIDAL_TYPE_CELL_MIGRATION	GOBP_AMEBOIDAL_TYPE_CELL_MIGRATION	GOBP_AMEBOIDAL_TYPE_CELL_MIGRATION	88	0.282059112	2.154969999	0.00026478	0.005032905	0.003814985	1077	tags=53%, list=34%, signal=36%	EFNB1/ARHGDIB/FGFR1/IQGAP1/HMGB1/ABL1/EMP2/CAP1/SMO/CYP1B1/CD248/RAC1/ITGB1BP1/LOXL2/ILK/GADD45A/NFE2L2/HAND2/ITGB1/ANXA6/BMP4/PTN/SNAI2/ITGB7/CLN3/TEK/CTSH/RAB11A/HAS2/NRP2/ARHGEF7/GNA12/CXCL13/ITGA3/FGF1/LRG1/ENG/WNT11/HSPB1/MIXL1/NRP1/CLASP2/MAP2K3/CLEC14A/MMRN2/FGF19/ROCK2
GOCC_VACUOLA	GOCC_VACUOLA	GOCC_VACUOLAR_ME	105	0.267529401	2.145073595	0.00028503	0.00539661	0.00409068	1214	tags=57%,	CCDC115/WDR81/CYBRD1/IFITM3/SC

R_MEMBRANE	R_MEMBRANE	MBRANE				7	8	3		list=39%, signal=36%	ARB2/TM9SF1/DAB2/TFE3/HLA-DMB/IFITM1/BLOC1S1/HLA-DRB3/HLA-DQB1/HLA-F/HLA-DRB5/MANBA/TPCN1/HPS6/C3AR1/VAPA/HLA-DMA/TFEB/ATG16L2/ANXA6/WIPI1/TSC1/MFSD1/CLN3/SBF2/HLA-DOB/SLC11A1/ABHD6/VPS13C/CPNE3/AP2M1/UVRAG/DNAJC5/LYN/TRPM2/DNAJC13/SLC29A3/GNAI1/STOM/YWHAB/OSTM1/SLC7A14/CHMP2B/TMEM63A/RRAGA/ABCD4/CHMP4C/ABCC10/ULK1/GNB1/VPS11/CHMP6/CHMP7/VPS4A/ATP6V1G2/CD1B
HP_ABNORMALITY_OF_THE_CHIN	HP_ABNORMALITY_OF_THE_CHIN	HP_ABNORMALITY_OF_THE_CHIN	84	0.286659375	2.157064665	0.000287353	0.005419134	0.00410775	416	tags=33%, list=13%, signal=30%	RNF113A/FBN1/MAF/SETBP1/NFIA/RALA/FGFR1/CCDC8/SH3PXD2B/PDGFRB/CDH11/TBC1D2B/IGF2/MED12/PQBP1/ZMPSTE24/ABL1/COX7B/H19/GLB1/BAZ1B/STT3A/PIGW/FBXW11/MRAS/GTF2IRD2/MMP23B/CBFB
GOBP_REGULATION_OF_CELL_CYCLE_CHECKPOINT	GOBP_REGULATION_OF_CELL_CYCLE_CHECKPOINT	GOBP_REGULATION_OF_CELL_CYCLE_CHECKPOINT	12	-0.589822365	-2.362186851	0.000295916	0.005515745	0.004180982	636	tags=75%, list=20%, signal=60%	CDK5RAP2/MAD2L1BP/THOC5/BARD1/DYNC1LI1/RAD51/TPR/CRY1/CDCA8
GOMF_CATALYTIC_ACTIVITY_ACTING_ON_A_NUCLEIC_ACID	GOMF_CATALYTIC_ACTIVITY_ACTING_ON_A_NUCLEIC_ACID	GOMF_CATALYTIC_ACTIVITY_ACTING_ON_A_NUCLEIC_ACID	105	-0.235481635	-2.122771412	0.000295303	0.005515745	0.004180982	840	tags=50%, list=27%, signal=38%	LIG3/POLR1C/POP4/MAP1S/MBD4/RFC1/TRIT1/RNGTT/ENDOG/POLI/DDX49/CNOT6/ERCC3/CHD5/MCM5/RAD50/ANGEL1/TOP3A/WRN/DDX52/TRPT1/CNOT7/TOP1MT/SMC3/POLE/DUS1L/PNPT1/PCNA/CHD1L/PRIM1/POLRMT/CDK7/NUDT4/BLM/DUS3L/RUVBL1/METTL6/PIWIL2/EXOSC8/EXOSC4/RECQL4/RFC4/POLG2/NUDT1/DHX30/POLB/RAD51/CRY1/DHX16/SETX/RAD17/DDX20/CPSF3
GOBP_CIRCULATORY_SYSTEM_PROCESS	GOBP_CIRCULATORY_SYSTEM_PROCESS	GOBP_CIRCULATORY_SYSTEM_PROCESS	117	0.249614859	2.072584124	0.000295793	0.005515745	0.004180982	1955	tags=85%, list=62%, signal=33%	SLC44A1/LRP3/ZMPSTE24/ABL1/EMP2/GSTO1/VEGFB/ATP1A2/ITGB1BP1/HEG1/C3AR1/SLC29A1/NKX2-5/NAV2/KCNE3/RAMP2/TEK/FOXC1/SLC2A13/ELN/SRI/ID2/SVEP1/CBS/ATP2A3/CLDN5/GNA12/IER3/CORO2B/YAP1/APLN/ADM/ADD3/GCLC/AGER/SLC6A17/SLC22A8/MDM2/KLF2/PKP2/SLC29A2/MAP2K3/CALM3/INS/ABCB1/SLC24A3/KLK1

											/FGF13/ROCK2/CPA3/SMAD7/ADIPOQ/SLC16A12/SLC6A1/ADRA2B/HTR1B/ADA/NDST2/PCSK5/TRPV1/P2RX2/HCN4/SLC22A2/CAV3/ATG5/THRA/GLP1R/IRX3/BCR/GJC1/ATP1B1/RENBP/ATP1A3/PPARA/ABCG2/ATP2A1/BDKRB1/SCN2B/CTSG/POPDC2/DES/DSP/SLC16A7/SLC16A1/ZC3H12A/ATP2B3/GATA4/KCND2/UMOD/KCNMB2/CHRNA7/UCN/ECE1/KCNMB1/FYN/PTP4A3/ATP1B2/LRP1/TNF
GOMF_HELICASE_ACTIVITY	GOMF_HELICASE_ACTIVITY	GOMF_HELICASE_ACTIVITY	28	-0.417187196	-2.426816496	0.000299376	0.005558687	0.004213532	791	tags=61%, list=25%, signal=46%	DDX49/ERCC3/CHD5/MCM5/RAD50/WRN/DDX52/CHD1L/BLM/RUVBL1/RECQL4/RFC4/DHX30/RAD51/DHX16/SETX/DDX20
GOBP_TISSUE_MORPHOGENESIS	GOBP_TISSUE_MORPHOGENESIS	GOBP_TISSUE_MORPHOGENESIS	130	0.24919574	2.122075505	0.000303443	0.005604665	0.004248384	923	tags=45%, list=29%, signal=33%	MSN/ALDH1A3/RALA/PHB2/DAB2/MED12/ABL1/SMO/GLI2/COL3A1/PLXNA1/RAC1/INHBA/ILK/HEG1/MYC/HAND2/FKBP1A/ADAMTS5/ITGB1/AHDC1/SULF1/BMP4/NKX2-5/MTHFD1/PLEKHA4/CITED2/ZFPM2/SNAI2/TSC1/TOR1A/BMP2/FOXC1/CTSH/BCL2/AIRE/PODXL/NRP2/PHLDB2/RHOC/MMP14/COL4A1/NR3C1/YAP1/ITGA3/ITGA5/FGF1/LRG1/ENG/FRS2/EOMES/ADM/WNT11/MAGED1/KRT27/MDM2/PKP2/NRP1/CLASP2
HP_ABNORMALITY_OF_THE_ABDOMINAL_WALL	HP_ABNORMALITY_OF_THE_ABDOMINAL_WALL	HP_ABNORMALITY_OF_THE_ABDOMINAL_WALL	110	0.260736888	2.112110439	0.000304183	0.005604665	0.004248384	800	tags=43%, list=25%, signal=33%	RNF113A/FBN1/PLOD1/MAF/SETBP1/SGSH/EFNB1/NFIA/FGFR1/SH3PXD2B/CDH11/IGF2/MED12/TFE3/ZMPSTE24/H19/GLB1/TRIM8/HLA-DQB1/ATP1A2/COL3A1/BAZ1B/RAC1/MBTPS1/MANBA/PIGW/GNE/FBXW11/MID1/MRAS/GTF2IRD2/NKX2-5/PLAGL1/COLEC11/TOR1A/ZBTB7A/CTCF/ELN/PLAG1/MMP14/CBS/LTBP1/LFNG/CDKN1C/PPP2R1A/PRR12/PTDSS1
GOBP_REGULATION_OF_CELL_ACTIVATION	GOBP_REGULATION_OF_CELL_ACTIVATION	GOBP_REGULATION_OF_CELL_ACTIVATION	117	0.249401265	2.070810629	0.000306549	0.005626687	0.004265077	701	tags=38%, list=22%, signal=31%	TYK2/LAG3/EFNB1/PYCARD/IGF2/CEBPB/XBP1/HLA-DMB/HMGB1/TYROBP/ABL1/HAVCR2/AIF1/HLA-DRB3/GLI2/HLA-DQB1/PPP3CA/PLSCR1/HLA-E/INHBA/HLA-F/HLA-DRB5/PTPRC/CEBPA/HLA-DMA/HLA-A/FLOT2/BMP4/CBFB

											/CCL21/IL15/CD177/FGL2/HLA-DOB/CCL5/CHRNB2/BCL2/BTN2A2/ID2/PTPN2/MMP14/CTSC/LYN/SCGB1A1/FCER1G
GOBP_REGULATION_OF_CELL_DIFFERENTIATION	GOBP_REGULATION_OF_CELL_DIFFERENTIATION	GOBP_REGULATION_OF_CELL_DIFFERENTIATION	292	0.183772958	1.879072291	0.000308591	0.005642641	0.00427717	1443	tags=56%, list=46%, signal=33%	FBN1/NFKB1/MAF/LAG3/FGFR1/LRP3/DAB2/IGF2/CEBPB/TFE3/XBP1/HMGB1/TYROBP/ABL1/EMP2/EIF2AK4/IFITM1/SMO/HCLS1/JDP2/CHRD/EIF2AK2/MAFB/GLI2/PPP3CA/NAP1L1/CLIC1/SOCS2/BTG2/TSPO/PLXNA1/STAT3/C1QC/CD34/HLA-B/LOXL2/INHBA/ILK/MYC/PTPRC/CEBPA/NFE2L2/HAND2/EIF4G1/ISL2/ITGB1/CYFIP1/FLOT2/MAP3K5/BMP4/PTN/ATF4/NKX2-5/ADD1/ALOX5/PRKCH/CBFB/ZFPM2/SNAI2/IL15/CCDC3/PIAS3/CDK5/RAMP2/BMP2/FGL2/FOXC1/OR10J5/ISG15/BCL2/BTN2A2/ID2/PHLDB2/PLAG1/PTPN2/MMP14/FZD4/LYN/MAFG/RELA/MAML1/TRIO/CLDN5/TCTA/SIRT2/YAP1/CDKN1C/PPP2R1A/NTF3/LRG1/ENG/FRS2/FOSL2/IL17A/ADM/TMEM119/NCOA3/AGER/MIXL1/SERPINE2/MDM2/KLF10/PKP2/NRP1/CLASP2/BAD/CCR1/NR1H2/INS/CD4/PTCH2/CD83/CUL7/CDH4/ULK1/PRMT6/ERRFI1/FGF13/ROCK2/S100B/MYOG/GCM1/FAM20C/FBXW8/ARRB2/NFATC4/SOCS1/TNFRSF21/GDI1/SMAD7/ETS1/PITX3/ADIPOQ/TNXB/CX3CR1/RGS14/ZNF703/ASCL1/ADIPOR1/ADRA2B/ADA/LTA/OCIAD1/MEN1/TBX6/JUND/TRPV1/THY1/SUPT6H/PDGFRA/NOTCH4/AKT1/NPHP3/ZNF488/ZBTB16/MT3/CAV3/WNT3A/F11R/RARG/LTF/DAB1/FOXE3
GOCC_CONDENSED_NUCLEAR_CHROMOSOME	GOCC_CONDENSED_NUCLEAR_CHROMOSOME	GOCC_CONDENSED_NUCLEAR_CHROMOSOME	16	-0.534257167	-2.415734829	0.000315979	0.005755837	0.004362974	728	tags=69%, list=23%, signal=53%	RAD50/KIFAP3/SMC3/NOL6/BLM/TUBG1/SMC1B/STAG3/RAD51/FKBP6/HSPA2
GOBP_POSITIVE_REGULATION_OF_EPITHELIAL_CELL_PROLIFERATION	GOBP_POSITIVE_REGULATION_OF_EPITHELIAL_CELL_PROLIFERATION	GOBP_POSITIVE_REGULATION_OF_EPITHELIAL_CELL_PROLIFERATION	36	0.398057676	2.243052348	0.000323427	0.005869287	0.00444897	1443	tags=81%, list=46%, signal=44%	FGFR1/IGF2/XBP1/HMGB1/SMO/VEGFB/STAT3/MYC/TGFA/BMP4/PTN/NKX2-5/TEK/HAS2/NRP2/YAP1/CDH3/APLN/FGF1/LRG1/NRP1/BAD/IQGAP3/GHSR/ZNF703/ANG/AKT1/WNT3A/FOXE3

GOBP_ANTIGEN_PROCESSING_AND_PRESENTATION_OF_EXOGENOUS_PEPTIDE_ANTIGEN	GOBP_ANTIGEN_PROCESSING_AND_PRESENTATION_OF_EXOGENOUS_PEPTIDE_ANTIGEN	GOBP_ANTIGEN_PROCESSING_AND_PRESENTATION_OF_EXOGENOUS_PEPTIDE_ANTIGEN	14	0.568976678	2.223651396	0.000343928	0.00621785	0.004713184	701	tags=71%, list=22%, signal=56%	HLA-DMB/HLA-DRB3/HLA-DQB1/HLA-E/HLA-F/HLA-DRB5/HLA-DMA/HLA-A/HLA-DOB/FCER1G
GOBP_REGULATION_OF_CHROMOSOME_ORGANIZATION	GOBP_REGULATION_OF_CHROMOSOME_ORGANIZATION	GOBP_REGULATION_OF_CHROMOSOME_ORGANIZATION	55	-0.313101427	-2.294157618	0.00035158	0.00632268	0.004792646	920	tags=58%, list=29%, signal=42%	MCRS1/SMC4/LCMT1/CDC16/UBE2C/PARP1/ATR/MAP3K4/RAD50/ZW10/POT1/SMARCC1/CDK5RAP2/YLPM1/PPHLN1/GTF2H2/MAD2L1BP/PINX1/BRD7/TRIM28/ESPL1/MAD2L2/DYNC1LI1/MAPK3/SMC6/TRIP13/RUVBL1/CENPE/BUB1B/ATF7IP/TPR/CDCA8
GOBP_WOUND_HEALING	GOBP_WOUND_HEALING	GOBP_WOUND_HEALING	92	0.280407005	2.166908494	0.000352356	0.00632268	0.004792646	1445	tags=66%, list=46%, signal=37%	F10/TFPI/XBP1/PPP3CA/PLSCR1/ST3GAL4/CLIC1/COL3A1/CD34/ILK/ITPK1/HPS6/NFE2L2/ITGB1/ANXA6/ALOX5/VWF/TOR1A/SLC11A1/PHLDB2/RHOC/LYN/PLAU/FCER1G/F2RL3/GNA12/YAP1/ITGA5/FGF1/SERPINA1/LRG1/HSPB1/SERPINE2/CHMP2B/CLASP2/INS/DCBLD2/DUOX2/CHMP4C/ADIPOR2/TSPAN8/CASK/CLDN4/CHMP6/CHMP7/VPS4A/AQP1/ADRA2B/EPB41L4B/TIMP1/PDGFRA/NOTCH4/ENTPD2/F11/CAV3/WNT3A/F8/F11R/SPRR3/TSPAN32/ELK3
GOBP_NEPHRON_DEVELOPMENT	GOBP_NEPHRON_DEVELOPMENT	GOBP_NEPHRON_DEVELOPMENT	39	0.371109362	2.157695762	0.000354151	0.00633127	0.004799157	860	tags=54%, list=27%, signal=40%	PDGFRB/IQGAP1/SMO/PPP3CA/CD34/ILK/MYC/SULF1/BMP4/BMP2/TEK/FOXC1/MPV17/BCL2/PODXL/YAP1/ITGA3/SEC61A1/FGF1/WNT11/MAGED1
HP_ABNORMALITY_OF_SKIN_ADNEXA_MORPHOLOGY	HP_ABNORMALITY_OF_SKIN_ADNEXA_MORPHOLOGY	HP_ABNORMALITY_OF_SKIN_ADNEXA_MORPHOLOGY	293	0.191426786	1.957094085	0.000359253	0.006398692	0.004850263	1122	tags=45%, list=36%, signal=32%	RNF113A/FBN1/NFKB1/CTSK/ANTXR2/MAF/SUMF1/SETBP1/WDR81/SGSH/EFNB1/CDK4/RALA/FGFR1/CCDC8/PDGFRB/CDH11/DPH5/HTRA1/IGF2/MED12/TFE3/MGP/PQBP1/CTBP1/GMPPA/ZMPSTE24/ABL1/SMO/COX7B/KRT14/GPRASP2/GLB1/MAFB/TRIM8/GLI2/HLA-DQB1/PERP/ATP1A2/COL3A1/PLXNA1/BAZ1B/STAT3/RAC1/HLA-B/HPS6/SLC30A9/STT3A/PIGW/GNE/BTNL2/MID1/MRAS/HLA-C/SLC25A12/BMP4/GTF2IRD2/PLAGL1/HPS1/APCDD1/HS6ST1/MMP23B/ZFPM2/SNAI2/COLEC11/PURA/TS

											C1/CDK5/IRAK1/CLN3/BMP2/CTCF/EDA/ELN/PPOX/AIRE/PAX9/PLAG1/ZNF462/MMP14/CYP17A1/CTSC/CBS/AP2M1/OAT/PIGG/KRT85/BRCC3/TRIO/NR3C1/PRPS1/SLC29A3/LTBP1/CDKN1C/MAGEL2/ITGA3/CDH3/PPP2R1A/PRR12/PTDSS1/ENG/STAG2/SEC23A/PROKR2/SLC6A17/SLURP1/NSDHL/THUMPD1/MDM2/NHLH2/INPPL1/STUB1/HEATR3/TALDO1/FHL1/PNKP/ITGAM/HUWE1/EHMT1/CUL7/CDC42BPB/UBE3B/PRKAR1A/DDX3X/FSHR/SMC1A/PYCR2/KISS1R/FAM20C/TTC8/CASK/CNBP
GOBP_SMOOTH_MUSCLE_CELL_PROLIFERATION	GOBP_SMOOTH_MUSCLE_CELL_PROLIFERATION	GOBP_SMOOTH_MUSCLE_CELL_PROLIFERATION	26	0.456123744	2.306274811	0.000363349	0.006447763	0.00488746	905	tags=65%, list=29%, signal=47%	PDGFRB/OGN/AIF1/MAP3K5/BMP4/IRAK1/BMP2/CCL5/ELN/MEF2D/ID2/GNA12/APOD/APLN/FRS2/ERN1/MDM2
GOCC_GERM_CELL_NUCLEUS	GOCC_GERM_CELL_NUCLEUS	GOCC_GERM_CELL_NUCLEUS	19	-0.505289617	-2.473875322	0.000364748	0.006448797	0.004888243	437	tags=68%, list=14%, signal=59%	SMARCC1/TAF10/PCNA/CDK7/TRIP13/ACTL7A/RAD51/HSPA2/DAZAP1/TSN/TCFL5/SPAG8/TBPL1
HP_DECREASED_FERTILITY	HP_DECREASED_FERTILITY	HP_DECREASED_FERTILITY	50	-0.31576937	-2.222142385	0.000373358	0.006576842	0.004985303	388	tags=40%, list=12%, signal=36%	GGPS1/MAD2L2/BLM/DNAI1/TRIP13/IFT74/DZIP1/UBE2T/STAG3/DNAH17/RAD51/FKBP6/DNAH8/AURKC/KLHL10/DNALI1/ZMYND10/FOXJ1/CATSPER2/TTC21A
GOBP_CARDIAC_SEPTUM_MORPHOGENESIS	GOBP_CARDIAC_SEPTUM_MORPHOGENESIS	GOBP_CARDIAC_SEPTUM_MORPHOGENESIS	14	0.565537785	2.210211655	0.000378903	0.006650163	0.00504088	919	tags=79%, list=29%, signal=56%	PARVA/SMO/BMP4/NKX2-5/CITED2/ZFPM2/NRP2/ENG/WNT11/MDM2/NRP1
GOCC_SPINDLE_POLE	GOCC_SPINDLE_POLE	GOCC_SPINDLE_POLE	43	-0.334701672	-2.269586652	0.000381975	0.006679693	0.005063264	828	tags=53%, list=26%, signal=40%	SPAST/WDR73/BCCIP/ZW10/CDK5RAP2/RASSF1/SMC3/DYNC1LI1/CEP63/TPX2/SMC6/NDE1/TUBG1/KATNB1/TBCCD1/EFHC1/AURKC/CDC14B/KATNA1/SPAG5/RAE1/MAPKBP1/WDR62
GOBP_APOPTOTIC_PROCESS	GOBP_APOPTOTIC_PROCESS	GOBP_APOPTOTIC_PROCESS	389	0.170923288	1.832242484	0.000391645	0.006823988	0.005172641	977	tags=39%, list=31%, signal=31%	SHC1/NFKB1/CTSK/CASP4/ALDH1A3/TXNDC5/AKR1A1/TNFSF10/FGFR1/PHB2/DAB2/HTRA1/PYCARD/IFIT3/EMP3/CEBPB/XBP1/HMGB1/ZMPSTE24/ABL1/EMP2/SMO/HCLS1/H19/VDAC1/EMP1/CYP1B1/EIF2AK2/ITPR1/TRAF1/CAT/CD248/PLSCR1/SOCS2/BTG2/PERP/SLC25A5/TSPO/LIPA/INHBA/MYC/GADD45A/PTPRC/RPS27L/NFE2L2/HAND2/LTBR/BFAR/ITGB1/ARHGDIA/STEAP3/SUL

											F1/RNF130/ING4/HIPK2/MAP3K5/ANXA6/BMP4/ATF4/NQO1/NKX2-5/TMEM14A/PLAGL1/ANXA4/PHLDA3/SRPX/MNT/TFPT/BLCAP/CITED2/PRKCH/IGF2R/SNAI2/CCL21/WFS1/COLEC11/CYLD/TNFAIP1/ADAM15/CDK5/CLN3/BCL2L1/TMEM109/KCNB1/PRMT2/RAMP2/BMP2/TEK/DHCR24/ZBTB7A/CCL5/FOXC1/CTSH/CCNL2/ANGPTL4/GRIA4/BCL2L2/MEF2D/BCL2/PRAP1/ARHGEF7/ARF4/PTPN2/HSH2D/CTSC/CBS/DNAJC5/LYN/UBE2B/ATP2A3/RELA/BBC3/DEDD/TRAF5/NR3C1/PRDX3/IER3/RGL2/SIRT2/YAP1/PPP2R1A/ITGA5/CCR5/NTF3/ENG/FRS2/FOSL2/IL17A/NME6/ADM/WNT11/IFI6/HSPB1/ERN1/GCLC/MAGED1/NEK6/NET1/CXCR3/RRM2B/SLFN12/SQSTM1/MDM2/NRP1/BAD/NHLH2/RRAGA/PPIF/OMA1/INS/BAX/PDK2/SPN
HP_SUBCUTANEOUS_NODULE	HP_SUBCUTANEOUS_NODULE	HP_SUBCUTANEOUS_NODULE	25	0.455820975	2.256831833	0.000404712	0.007026216	0.005325932	647	tags=56%, list=21%, signal=45%	FBN1/ANTXR2/CDK4/FGFR1/PDGFRB/IFNGR1/COL3A1/HLA-B/BTNL2/CYLD/TSC1/TEK/MEFV/MMP14
GOCC_NUCLEAR_BODY	GOCC_NUCLEAR_BODY	GOCC_NUCLEAR_BODY	166	-0.191663313	-1.955493954	0.000417053	0.007214418	0.005468591	966	tags=48%, list=31%, signal=35%	TOLLIP/PRPF6/XPO1/RBBP6/GON4L/SMC4/CHEK2/FIBP/KLHDC2/MBD4/ELL/SF3B2/PABPN1/RMI1/CSNK1A1/ELF2/RCHY1/POLI/PARP1/ATR/SIRT1/CBX1/CHD5/SETD1A/SYMPK/TP53BP1/PIP5K1A/HIPK1/ZC3H8/TOP3A/WRN/AKAP8L/YLPM1/KIF22/TP53INP1/GTF2H2/CNOT7/SRP19/LPXN/RBM14/SAP130/USPL1/BANP/PRPF40A/MAGEA2B/SUZ12/BARD1/S100PBP/DGKZ/PCNA/PARP11/BLM/SMC6/UBN1/SF3A1/GEMIN4/GEMIN6/PRPF18/INTS7/BRD1/PPP1CC/DZIP1/TESK2/PRPF40B/ATF7IP/MDC1/ELL3/TFIP11/RAD51/PHF7/PRKAA1/SRPK1/SETX/SURF2/ZNF473/SPAG5/DDX20/KLF11/AHCTF1
GOBP_POSITIVE_REGULATION_OF_RESPONSE_TO_BIOTIC_STIMULU	GOBP_POSITIVE_REGULATION_OF_RESPONSE_TO_BIOTIC_STIMULUS	GOBP_POSITIVE_REGULATION_OF_RESPONSE_TO_BIOTIC_STIMULUS	62	0.323530467	2.231785468	0.000423044	0.007291819	0.005527261	910	tags=53%, list=29%, signal=39%	LAG3/PHB2/LSM14A/PYCARD/PQBP1/HMGB1/TYROBP/HAVCR2/HLA-DRB3/EIF2AK2/MAPKAPK3/PLSCR1/PUM1/HLA-E/HLA-F/FLOT2/NMI/COLEC11/RN

S											F185/CYLD/IRAK1/CCL5/LY86/MEFV/LYN/BRCC3/RELA/SIRT2/KIR2DL4/IL17A/TRIM41/ZNFX1/NFKBIL1
GOBP_POSITIVE_REGULATION_OF_VASCULATURE_DEVELOPMENT	GOBP_POSITIVE_REGULATION_OF_VASCULATURE_DEVELOPMENT	GOBP_POSITIVE_REGULATION_OF_VASCULATURE_DEVELOPMENT	40	0.372502486	2.180492888	0.000436418	0.007469361	0.00566184	1229	tags=70%, list=39%, signal=43%	XBP1/ABL1/EMP2/VEGFB/CYP1B1/STAT3/CD34/C3AR1/NFE2L2/ITGB1/HIPK2/ADAM12/RAMP2/TEK/CTSH/ANGPTL4/ITGA5/FGF1/LRG1/ENG/ADM/HSPB1/NRP1/ITGB8/ETS1/GHSR/CX3CR1/AQP1
GOBP_REGULATION_OF_CELL_CYCLE_PROCESS	GOBP_REGULATION_OF_CELL_CYCLE_PROCESS	GOBP_REGULATION_OF_CELL_CYCLE_PROCESS	166	-0.191172496	-1.950486273	0.000436451	0.007469361	0.00566184	519	tags=29%, list=16%, signal=25%	RBM14/MAD2L1BP/PINX1/THOC5/BRD7/PRPF40A/ESPL1/BARD1/MAD2L2/DYNC1LI1/CEP63/CDK7/TPX2/BLM/SMC6/PAXIP1/CEP250/TRIP13/PIWIL2/KIF23/PAF1/DONSON/INTS7/FBXO7/CENPE/BUB1B/FZR1/MDC1/CSNK2A2/RAD51/TPR/CRY1/AURKC/CCNH/CDCA8/HSPA2/DOT1L/RANBP1/CDC14B/CDC25C/RAD17/SPAG5/RAE1/SH3GLB1/AURKAIP1/AHCTF1/WDR62/CPSF3
GOBP_ANTIGEN_PROCESSING_AND_PRESENTATION_OF_PEPTIDE_ANTIGEN	GOBP_ANTIGEN_PROCESSING_AND_PRESENTATION_OF_PEPTIDE_ANTIGEN	GOBP_ANTIGEN_PROCESSING_AND_PRESENTATION_OF_PEPTIDE_ANTIGEN	25	0.451658197	2.236221351	0.000442535	0.007519966	0.005700199	701	tags=60%, list=22%, signal=47%	PYCARD/HLA-DMB/HLA-DRB3/HLA-DQB1/HLA-B/HLA-E/HLA-F/HLA-DRB5/HLA-DMA/HLA-A/HLA-C/HLA-DOB/SLC11A1/TAPBPL/FCER1G
GOBP_CELL_JUNCTION_ASSEMBLY	GOBP_CELL_JUNCTION_ASSEMBLY	GOBP_CELL_JUNCTION_ASSEMBLY	95	0.265264924	2.075843398	0.000442481	0.007519966	0.005700199	1516	tags=68%, list=48%, signal=37%	CDH11/ABL1/TRIP6/RAC1/ITGB1BP1/EIF4G1/MYOC/CLDN15/PRKCH/SNAI2/GJA4/PCDHB16/TSC1/CDK5/LGI2/RAMP2/TEK/PCDHB14/CHRNB2/BCL2/AMIGO2/RAPGEF1/ARHGEF7/PHLDB2/RHOC/MMP14/CLDN5/FAM107A/CORO2B/CDH3/ITGA5/APOD/IL17A/PCDHB4/WNT11/PKP2/NRP1/CLASP2/CNTNAP1/LRFN3/CDH4/SLITRK2/FGF13/ROCK2/NPAS4/CLDN4/CDH9/CBLN4/SMAD7/ABI3/ACTN2/GHSR/ZNF703/DLC1/CTTN/CLSTN3/THY1/LRRN3/WNT3A/F11R/IRX3/DNM3/BCR/GJC1/SH3BP1
GOBP_SINGLE_FERTILIZATION	GOBP_SINGLE_FERTILIZATION	GOBP_SINGLE_FERTILIZATION	19	-0.500073559	-2.448337737	0.000445802	0.007548804	0.005722058	541	tags=58%, list=17%, signal=48%	TNP2/UBE3A/LYZL6/SPACA3/ACTL7A/AKAP4/IQCF1/SPA17/HSPA1L/ZPBP2/SPAG8
GOCC_CENTRIOLE	GOCC_CENTRIOLE	GOCC_CENTRIOLE	26	-0.415178488	-2.320454285	0.000447555	0.007551904	0.005724408	784	tags=62%, list=25%,	SFI1/BBS4/BCCIP/KIAA0753/IQCB1/IFT20/CEP350/CEP63/SSNA1/CEP250/TUB

										signal=47%	G1/DZIP1/KIF3A/CCHCR1/TSSK2/WDR62
GOCC_RNA_POLYMERASE_II_HOLOENZYME	GOCC_RNA_POLYMERASE_II_HOLOENZYME	GOCC_RNA_POLYMERASE_II_HOLOENZYME	17	-0.521068287	-2.453598633	0.000451139	0.007585769	0.005750078	748	tags=71%, list=24%, signal=54%	ERCC3/TAF9/TAF6/GTF2E1/TAF10/GTF2H1/GTF2H2/CDK7/PAF1/CCNH/SUPT3H/TBPL1
HP_ANTENATAL_ONSET	HP_ANTENATAL_ONSET	HP_ANTENATAL_ONSET	49	-0.338428675	-2.365150193	0.000456817	0.007627894	0.005782009	418	tags=39%, list=13%, signal=34%	PRIM1/CHRNG/PYCR1/RECQL4/VRK1/GLUL/CENPE/ASNS/WNT3/BUB1B/FZR1/TRAIP/DEF6/SLC25A19/CLPB/PSMD12/NUP88/NUP155/CPSF3
HP_ABNORMAL_UMBILICUS_MORPHOLOGY	HP_ABNORMAL_UMBILICUS_MORPHOLOGY	HP_ABNORMAL_UMBILICUS_MORPHOLOGY	67	0.315335601	2.234967692	0.000455433	0.007627894	0.005782009	513	tags=39%, list=16%, signal=33%	RNF113A/FBN1/PLOD1/SETBP1/SGSH/EFNB1/SH3PXD2B/CDH11/IGF2/MED12/TFE3/ZMPSTE24/H19/TRIM8/COL3A1/BAZ1B/RAC1/PIGW/FBXW11/MID1/GTF2IRD2/NKX2-5/PLAGL1/COLEC11/TOR1A/ZBTB7A
HP_SKIN_NODULE	HP_SKIN_NODULE	HP_SKIN_NODULE	28	0.432949422	2.232450164	0.000464458	0.007728649	0.005858382	647	tags=54%, list=21%, signal=43%	FBN1/ANTXR2/CDK4/FGFR1/PDGFRB/IFNGR1/COL3A1/HLA-B/BTNL2/CYLD/TSC1/TEK/BCL2/MEFV/MMP14
GOBP_PROTEIN_LOCALIZATION_TO_ORGANELLE	GOBP_PROTEIN_LOCALIZATION_TO_ORGANELLE	GOBP_PROTEIN_LOCALIZATION_TO_ORGANELLE	201	-0.190270222	-2.033001702	0.000473097	0.007845247	0.005946764	700	tags=34%, list=22%, signal=28%	ZW10/POT1/PMPCB/KIAA0753/ARL2/WRN/PPHLN1/YWHAZ/CAMLG/RGPD5/PINK1/GAK/NUP93/SRP19/KPNA6/MON1A/IFT20/OSBPL8/ARFRP1/PINX1/BANP/TIMM23/TRIM28/RER1/CEP350/SNUPN/BARD1/KPNB1/VPS37A/ZFAND6/DNAJB6/NCOA4/CEP250/POLA2/TULP2/HK1/LAMP3/UBE2D3/VRK1/PAF1/DNAJC15/SPATA7/GLUL/RAB8B/DZIP1/FBXO7/BUB1B/CEP72/CSE1L/HACL1/CSNK2A2/SAE1/PRKAA1/TPR/BCAP29/MTX2/PARD6A/NUP88/NF2/ZMYND10/IFT122/HSPA1L/SPAG5/LZTFL1/SH3GLB1/NUP155/KPNA2/TTC21A
GOBP_MONONUCLEAR_CELL_MIGRATION	GOBP_MONONUCLEAR_CELL_MIGRATION	GOBP_MONONUCLEAR_CELL_MIGRATION	37	0.37867731	2.178239828	0.000478045	0.007900071	0.005988321	1415	tags=78%, list=45%, signal=44%	MSN/PYCARD/HMGB1/ABL1/AIF1/FOLR2/C3AR1/ALOX5/CCL21/ITGB7/CCL5/AIRE/LYN/TRPM2/CXCL13/GPR15/APOD/CCR5/AGER/CCR1/SPN/CCL8/CX3CR1/CCL22/AKT1/S100A12/OXSR1/S100A14/F11R
GOCC_ENDOPLASMIC_RETICULUM_LUMEN	GOCC_ENDOPLASMIC_RETICULUM_LUMEN	GOCC_ENDOPLASMIC_RETICULUM_LUMEN	66	0.315244841	2.218771656	0.000481492	0.007929774	0.006010837	1716	tags=80%, list=54%, signal=37%	FUCA2/FBN1/SUMF1/TXNDC5/F10/IGFBP4/COL4A2/CALU/TSPAN5/COL3A1/COL4A5/COL6A3/MBTPS1/ADAMTS5/COL4A6/BMP4/PDIA6/FMO1/WFS1/TOR1

											A/CYP2W1/COL15A1/SDC2/CTSC/COL4A1/BCHE/LTBP1/COL27A1/PDIA5/SERPINA1/INS/CD4/DNAJC10/FAM20C/NOTUM/P4HA1/TIMP1/TGOLN2/MEN1/COL21A1/C3/WNT3A/F8/ADAMTSL1/VWA1/WNT1/COL8A2/FOXRED2/CFP/ARSI/RDH5/IL27/SERPINH1
HP_OLIGOZOOSPERMIA	HP_OLIGOZOOSPERMIA	HP_OLIGOZOOSPERMIA	10	-0.626452715	-2.316712398	0.0005011	0.00822454	0.006234271	388	tags=70%, list=12%, signal=62%	BLM/IFT74/DZIP1/DNAH17/FKBP6/KLHL10/CATSPER2
GOBP_REGULATION_OF_OSTEOBLAST_DIFFERENTIATION	GOBP_REGULATION_OF_OSTEOBLAST_DIFFERENTIATION	GOBP_REGULATION_OF_OSTEOBLAST_DIFFERENTIATION	26	0.445908211	2.254622544	0.000505585	0.008269926	0.006268674	503	tags=50%, list=16%, signal=42%	LRP3/CEBPB/IFITM1/CHRD/PPP3CA/CLIC1/ILK/CEBPA/HAND2/BMP4/ATF4/SNAI2/BMP2
HP_COARSE_FACIAL_FEATURES	HP_COARSE_FACIAL_FEATURES	HP_COARSE_FACIAL_FEATURES	52	0.337397726	2.210049133	0.000531685	0.008667374	0.006569943	369	tags=37%, list=12%, signal=33%	RNF113A/ANTXR2/SUMF1/SETBP1/WDR81/SGSH/SH3PXD2B/TBC1D2B/IGF2/MED12/TFE3/GLB1/PPP3CA/BAZ1B/STAT3/PIGW/GNE/GTF2IRD2/NKX2-5
GOCC_NUCLEOLUS	GOCC_NUCLEOLUS	GOCC_NUCLEOLUS	191	-0.184655521	-1.944050208	0.000545626	0.008864578	0.006719426	955	tags=46%, list=30%, signal=34%	XPO1/MCRS1/WDR3/BCKDHB/POLR1C/RBBP6/RGS22/SENP3/POP4/CUL2/MAP1S/FBXO11/KIT/IPPK/ERGIC2/ETV4/MAP3K14/DNTTIP2/NUSAP1/NHEJ1/PARP1/PDK3/CDCA7L/SIRT1/MRPS31/BOP1/PA2G4/ARL2/WRN/DDX52/TTF1/KLHL7/ATXN3/CARF/SRP19/PNMA1/ZNF415/TNP2/DNTTIP1/RBM14/MAD2L1BP/PINX1/NOL6/SUZ12/CAPN3/WDR33/ACTR6/TEX10/CENPH/CDK7/EBNA1BP2/AFF4/BLM/UTP18/C6orf89/SENP5/PWP1/GEMIN4/EXOSC8/EXOSC4/ZFP91/VRK1/PAF1/NIP7/PPP1CC/NARF/GTPBP4/ZNF692/UBE2T/TRAIP/ELL3/TFIP11/STAG3/RAD51/MTX2/SPECC1/SETX/CDCA8/CDC14B/SURF2/NF2/RAD17/LRRC34/RAE1/CCDC86/MAPKBP1/PRKRIP1
GOBP_REGULATION_OF_CELL_DEVELOPMENT	GOBP_REGULATION_OF_CELL_DEVELOPMENT	GOBP_REGULATION_OF_CELL_DEVELOPMENT	156	0.219957151	1.980522328	0.000560492	0.009075441	0.006879262	1438	tags=60%, list=46%, signal=34%	FBN1/LAG3/CEBPB/TFE3/XBP1/HMGB1/TYROBP/ABL1/SMO/HCLS1/MAFB/GLI2/PPP3CA/NAP1L1/BTG2/TSPO/PLXNA1/STAT3/C1QC/HLA-B/INHBA/MYC/PTPRC/CEBPA/ITGB1/CYFIP1/BMP4/PTN/ADD1/PRKCH/CBFB/SNAI2/IL15/PIAS3/CDK5/BMP2/FGL2/ISG15/BCL2/BTN

											2A2/ID2/PLAG1/PTPN2/MMP14/FZD4/LYN/RELA/CLDN5/TCTA/SIRT2/YAP1/IL17A/AGER/SERPINE2/KLF10/NRP1/BAD/CCR1/INS/CD4/CD83/CUL7/CDH4/ULK1/PRMT6/FGF13/ROCK2/MYOG/FBXW8/NFATC4/SOCS1/GDI1/SMAD7/ETS1/PITX3/ADIPOQ/CX3CR1/RGS14/ASCL1/ADA/LTA/TBX6/TRPV1/THY1/AKT1/ZNF488/ZBTB16/MT3/CAV3/WNT3A/F11R/RARG/LTF/DAB1
GOCC_VESICLE_MEMBRANE	GOCC_VESICLE_MEMBRANE	GOCC_VESICLE_MEMBRANE	251	0.19240833	1.913928493	0.000564342	0.009076655	0.006880182	749	tags=35%, list=24%, signal=29%	ANTXR2/COPZ1/WDR81/RALA/IFITM3/SCARB2/DAB2/HLA-DMB/IQGAP1/SEC22B/ZMPSTE24/TYROBP/SMO/HLA-DRB3/RAB1A/ITPR1/HLA-DQB1/PAM/RAC1/COPZ2/HLA-B/HLA-E/HLA-F/STAB1/HLA-DRB5/PTPRC/MANBA/TPCN1/HPS6/C3AR1/RHOU/ITGB1/VAPA/STEAP3/HLA-DMA/ANXA7/HLA-A/FLOT2/HLA-C/ANXA6/TGFA/CLTB/PRAF2/ANXA4/BACE2/EHD4/IGF2R/WFS1/WIPI1/CD177/IRAK1/CLN3/BCL2L1/SBF2/TOR1A/AP1G2/HLA-DOB/SLC11A1/SLC9A8/MITD1/ABHD6/RAB11A/GRIA4/RPH3AL/PHACTR2/LLGL1/SLC31A1/VPS13C/CPNE3/SRI/FZD4/SCAMP3/DENND4C/GPR161/AP2M1/FER1L4/CNIH3/DNAJC5/LYN/SYT12/TRPM2/PLAU/FCER1G/DNAJC13/SLC29A3/CLINT1/GPR135
GOBP_REGULATION_OF_CELL_CYCLE	GOBP_REGULATION_OF_CELL_CYCLE	GOBP_REGULATION_OF_CELL_CYCLE	236	-0.171223975	-1.857613065	0.000563924	0.009076655	0.006880182	776	tags=37%, list=25%, signal=30%	CDK5R1/BBS4/BCCIP/SIRT1/ERCC3/TIMELESS/PPP2R3B/RAD50/TAF6/TP53BP1/BOP1/ZW10/SMARCC1/DCUN1D3/RAB11FIP4/E2F1/CDK5RAP2/GTF2H1/TP53INP1/TFDP1/ECD/CDC7/BIRC8/ZBTB17/HERC5/RBM14/MAD2L1BP/PINX1/THOC5/BRD7/PRPF40A/MAGEA4/ESPL1/BARD1/MAD2L2/DYNC1LI1/PNPT1/ANAPC10/CEP63/CDK7/TPX2/BLM/C6orf89/SMC6/PAXIP1/CEP250/TRIP13/RUVBL1/PIWIL2/KIF23/PAF1/DONSON/INTS7/FBXO7/CENPE/ASNS/BUB1B/FZR1/MDC1/GTPBP4/CSNK2A2/RAD51/GMNN/CKS2/TPR/CRY1/AURKC/CCNH/CDCA

											8/HSPA2/DOT1L/RANBP1/CDC14B/CDC25C/NF2/RAD17/SPAG5/CCNB2/RAE1/SH3GLB1/AURKAIP1/DR1/PAFAH1B1/AHCTF1/WDR62/MDM4/CPSF3
GOMF_EXONUCLEASE_ACTIVITY	GOMF_EXONUCLEASE_ACTIVITY	GOMF_EXONUCLEASE_ACTIVITY	15	-0.537747357	-2.387325685	0.000571526	0.00916156	0.006944541	749	tags=73%, list=24%, signal=56%	CNOT6/RAD50/ANGEL1/WRN/CNOT7/POLE/PNPT1/POLRMT/EXOSC8/EXOSC4/CPSF3
GOBP_MRNA_EXPORT_FROM_NUCLEUS	GOBP_MRNA_EXPORT_FROM_NUCLEUS	GOBP_MRNA_EXPORT_FROM_NUCLEUS	18	-0.508230217	-2.432762117	0.000581051	0.00918147	0.006959633	1236	tags=89%, list=39%, signal=54%	NXF1/RBM15B/MCM3AP/XPO1/NUP107/PABPN1/IWS1/SMG7/AKAP8L/NUP93/THOC5/TPR/NUP88/RAE1/NUP155/NCBP2
GOBP_POSTREPLICATION_REPAIR	GOBP_POSTREPLICATION_REPAIR	GOBP_POSTREPLICATION_REPAIR	10	-0.62321113	-2.304724549	0.000580848	0.00918147	0.006959633	841	tags=90%, list=27%, signal=66%	VCP/RCHY1/POLI/USP10/MAD2L2/WDR33/PCNA/USP1/UBE2N
GOBP_SPINDLE_ORGANIZATION	GOBP_SPINDLE_ORGANIZATION	GOBP_SPINDLE_ORGANIZATION	52	-0.313012507	-2.20802449	0.000582314	0.00918147	0.006959633	520	tags=38%, list=16%, signal=33%	SMC3/TUBG2/ESPL1/KPNB1/CEP63/CENPH/TPX2/KIF23/TUBG1/CENPE/CEP72/SUGT1/EFHC1/TPR/AURKC/CDCA8/SPAG5/CCNB2/RAE1/WDR62
GOBP_VASCULOGENESIS	GOBP_VASCULOGENESIS	GOBP_VASCULOGENESIS	23	0.453110739	2.154048937	0.000574946	0.00918147	0.006959633	1067	tags=74%, list=34%, signal=49%	PDGFRB/EMP2/SMO/CD34/HEG1/NKX2-5/CITED2/ZFPM2/RAMP2/HAS2/FZD4/YAP1/ENG/ADM/NRP1/CUL7/ITGB8
GOBP_CELL_CYCLE_PHASE_TRANSITION	GOBP_CELL_CYCLE_PHASE_TRANSITION	GOBP_CELL_CYCLE_PHASE_TRANSITION	131	-0.214158879	-2.075537482	0.000577862	0.00918147	0.006959633	748	tags=40%, list=24%, signal=32%	ERCC3/TIMELESS/RAD50/TP53BP1/ZW10/SMARCC1/DCUN1D3/TAF10/AKAP8L/E2F1/CDK5RAP2/TFDP1/ECD/CDC7/MAD2L1BP/PINX1/THOC5/POLE/BRD7/ESPL1/BARD1/MAD2L2/DYNC1LI1/CEP63/CDK7/USP37/BLM/PAXIP1/TRIP13/PPP6C/PAF1/DONSON/INTS7/UBE2S/FBXO7/CENPE/BUB1B/FZR1/MDC1/RAD51/CKS2/TPR/CRY1/CCNH/CDCA8/HSPA2/DOT1L/CDC14B/CDC25C/RAD17/CCNB2/KLF11/CPSF3
GOBP_HEMATOPOIETIC_PROGENITOR_CELL_DIFFERENTIATION	GOBP_HEMATOPOIETIC_PROGENITOR_CELL_DIFFERENTIATION	GOBP_HEMATOPOIETIC_PROGENITOR_CELL_DIFFERENTIATION	23	0.452351335	2.150438798	0.000590176	0.009275022	0.007030546	862	tags=65%, list=27%, signal=48%	ABL1/EIF2AK2/WDR7/LIPA/INHBA/PTPRC/NFE2L2/BMP4/DHTKD1/CITED2/FOXC1/BCL2/LYN/HERC6/MIXL1
GOCC_CILIARY_BASAL_BODY	GOCC_CILIARY_BASAL_BODY	GOCC_CILIARY_BASAL_BODY	27	-0.401679988	-2.290326254	0.000592773	0.009285485	0.007038477	921	tags=67%, list=29%, signal=48%	MAPRE1/CEP290/CSNK1A1/TBC1D7/MKKS/BBS4/RAB28/KIFAP3/IFT20/SSNA1/CEP250/SPATA7/TUBG1/DZIP1/IFT122/SPAG5/CCDC96/IQCD
GOBP_REGULATION_OF_VASCULA	GOBP_REGULATION_OF_VASCULA	GOBP_REGULATION_OF_VASCULATURE_DEV	68	0.300272733	2.133898948	0.000601154	0.009386205	0.007114824	919	tags=50%, list=29%,	XBP1/COL4A2/PGK1/ABL1/EMP2/VEGFB/CYP1B1/STAT3/CD34/STAB1/GADD

TURE_DEVELOPMENT	TURE_DEVELOPMENT	ELOPMENT								signal=36%	45A/C3AR1/NFE2L2/ITGB1/SULF1/HIPK2/ALOX5/ADAM12/RAMP2/TEK/FOXC1/CTSH/OR10J5/ANGPTL4/CLDN5/CXCL13/ITGA5/FGF1/LRG1/ENG/ADM/HSPB1/KLF2/NRP1
HP_ABNORMAL_PLEURA_MORPHOLOGY	HP_ABNORMAL_PLEURA_MORPHOLOGY	HP_ABNORMAL_PLEURA_MORPHOLOGY	41	0.371935255	2.20390019	0.000612	0.009524615	0.00721974	930	tags=59%, list=29%, signal=42%	FBN1/SCARB2/IFNGR1/ABL1/EIF2AK4/COL3A1/HLA-B/GPKOW/BTNL2/TSC1/IRAK1/BMP2/SLC11A1/SLC34A2/ELN/BCL2/MEFV/SLC31A1/LTBP1/SERPINA1/ENG/GCLC/ZNFX1/CCR1
GOBP_SPERM_MOTILITY	GOBP_SPERM_MOTILITY	GOBP_SPERM_MOTILITY	14	-0.553270797	-2.357538982	0.000616569	0.009564772	0.007250179	382	tags=64%, list=12%, signal=57%	IQCG/DNAI1/DZIP1/AKAP4/IQCF1/ROPN1L/LZTFL1/CATSPER2/TTC21A
GOBP_RIBONUCLEOPROTEIN_COMPLEX_BIOGENESIS	GOBP_RIBONUCLEOPROTEIN_COMPLEX_BIOGENESIS	GOBP_RIBONUCLEOPROTEIN_COMPLEX_BIOGENESIS	73	-0.271525385	-2.156744878	0.00062645	0.009686812	0.007342686	392	tags=30%, list=12%, signal=27%	EBNA1BP2/UTP18/PWP1/SF3A1/RUVBL1/GEMIN4/EXOSC8/EXOSC4/GEMIN6/PRPF18/NIP7/GLUL/ZNHIT3/GTPBP4/DHX30/RPL26L1/EIF5/SRPK1/SETX/NUP88/BUD13/DDX20
HP_RESTRICTIVE_VENTILATORY_DEFECT	HP_RESTRICTIVE_VENTILATORY_DEFECT	HP_RESTRICTIVE_VENTILATORY_DEFECT	13	0.593989531	2.268239454	0.000628767	0.009691471	0.007346218	554	tags=69%, list=18%, signal=57%	NFKB1/PLOD1/GLB1/HLA-B/BTNL2/HPS1/TSC1/TOR1A/SLC34A2
GOBP_REGULATION_OF_EPITHELIAL_CELL_PROLIFERATION	GOBP_REGULATION_OF_EPITHELIAL_CELL_PROLIFERATION	GOBP_REGULATION_OF_EPITHELIAL_CELL_PROLIFERATION	64	0.316854083	2.203830355	0.000657412	0.010068448	0.00763197	1013	tags=55%, list=32%, signal=38%	KLF9/CDK4/FGFR1/PHB2/DAB2/IGF2/XBP1/HMGB1/SMO/VEGFB/STAT3/MYC/SULF1/TGFA/BMP4/PTN/NKX2-5/ALOX5/SNAI2/TEK/HAS2/NRP2/YAP1/CDKN1C/CDH3/APLN/FGF1/LRG1/FRS2/MAGED1/SLURP1/NRP1/BAD/BAX/MMRN2
GOBP_CARTILAGE_DEVELOPMENT	GOBP_CARTILAGE_DEVELOPMENT	GOBP_CARTILAGE_DEVELOPMENT	40	0.367494746	2.151179418	0.00065652	0.010068448	0.00763197	1472	tags=78%, list=47%, signal=42%	CTSK/MAF/NFIA/MGP/ZMPSTE24/TGFBI/OGN/COL3A1/LOXL2/HAND2/SULF1/ANXA6/BMP4/OTOR/SNAI2/BMP2/MEF2D/CBS/COL27A1/FOSL2/WNT11/ITGB8/WNT2B/SMAD7/ATF2/TIMP1/ZBTB16/RARG/PRRX1/THRA/HOXD3
GOBP_CONNECTIVE_TISSUE_DEVELOPMENT	GOBP_CONNECTIVE_TISSUE_DEVELOPMENT	GOBP_CONNECTIVE_TISSUE_DEVELOPMENT	57	0.317125971	2.117706331	0.000672574	0.010267968	0.007783208	1472	tags=72%, list=47%, signal=39%	CTSK/MAF/NFIA/SH3PXD2B/PDGFRB/XBP1/MGP/ZMPSTE24/TGFBI/OGN/COL3A1/LIPA/CD34/LOXL2/HAND2/SULF1/ANXA6/BMP4/OTOR/SNAI2/BMP2/EBF2/MEF2D/CBS/COL27A1/FOSL2/WNT11/ITGB8/WNT2B/SMAD7/ATF2/CRIP1/EGR1/HSD17B1/UMODL1/TIMP1/ZBTB16/RARG/PRRX1/THRA/HOXD3

GOBP_CALCIUM_ION_TRANSPORT	GOBP_CALCIUM_ION_TRANSPORT	GOBP_CALCIUM_ION_TRANSPORT	81	0.283778935	2.118246206	0.000702052	0.010684073	0.008098619	1620	tags=72%, list=51%, signal=36%	PDGFRB/ZMPSTE24/ABL1/GSTO1/VDAC1/ITPR1/PPP3CA/SLC25A23/TSPO/ATP1A2/PTPRC/TPCN1/FKBP1A/ANXA6/CCL21/WFS1/GJA4/CDK5/CSN2/RAMP2/CCL5/CHRNB2/BCL2/SRI/IBTK/LYN/TRPM2/ATP2A3/F2RL3/SEC61A1/CCR5/CCR1/CALM3/LIME1/CD4/BAX/SLC24A3/CABP5/CASK/CCL8/DIAPH1/XCR1/TRPV1/P2RX2/THY1/CAV3/WNT3A/ATG5/CACNA2D3/PLA2G1B/CACNA1B/CACNA1E/ATP1B1/CACNB3/GRM6/ATP2A1/TRPV5/BDKRB1
GOCC_NUCLEAR_SPECK	GOCC_NUCLEAR_SPECK	GOCC_NUCLEAR_SPECK	78	-0.246786428	-2.018024276	0.000711266	0.010790158	0.008179032	936	tags=51%, list=30%, signal=37%	RBBP6/SMC4/FIBP/MBD4/ELL/SF3B2/PABPN1/CSNK1A1/RCHY1/POLI/CHD5/SETD1A/PIP5K1A/HIPK1/WRN/AKAP8L/YLPM1/KIF22/GTF2H2/CNOT7/LPXN/RBM14/SAP130/PRPF40A/BARD1/S100PBP/DGKZ/SMC6/SF3A1/PRPF18/BRD1/PPP1CC/DZIP1/PRPF40B/ELL3/TFIP11/PHF7/PRKAA1/SRPK1/SURF2
GOBP_CELLULAR_ALDEHYDE_METABOLIC_PROCESS	GOBP_CELLULAR_ALDEHYDE_METABOLIC_PROCESS	GOBP_CELLULAR_ALDEHYDE_METABOLIC_PROCESS	18	0.515893701	2.24395995	0.00071714	0.010845052	0.008220642	973	tags=72%, list=31%, signal=50%	ALDH1A3/AKR1A1/AKR1C1/DAB2/HYI/CYP1B1/ALDH2/BMP2/PDXK/RELA/AKR7A2/AKR7A3/TALDO1
GOCC_SUPRAMOLECULAR_COMPLEX	GOCC_SUPRAMOLECULAR_COMPLEX	GOCC_SUPRAMOLECULAR_COMPLEX	263	-0.162862442	-1.832902432	0.000757803	0.011424051	0.008659529	648	tags=30%, list=21%, signal=26%	HOOK2/TUBB/CDK5RAP2/CPEB1/NME7/KIF22/KIF4A/KRT86/RASSF1/SLC1A4/PACRG/KIFAP3/CNOT7/ACTL8/PAWR/GGPS1/TIAL1/PINX1/TUBG2/FKBP4/BRD7/MYOM1/EDC4/CAPN3/CSRP2/BARD1/DYNC1LI1/MYOM2/HSPH1/RASSF3/KPNB1/MYH10/CAPZB/CENPH/MAP2K2/TPX2/DNAI1/DNAJB6/SSNA1/MAP6D1/KIFC3/ITGB3BP/NDE1/PIWIL2/EIF2S1/PRPH/KIF23/TUBG1/PPP1CC/CENPE/KIF3A/TDRKH/BUB1B/NARF/KATNB1/EDC3/SUGT1/MYO9A/C1orf112/EFHC1/DHX30/ARFGEF2/POLB/DNAH17/AKAP4/CENPM/TPR/DNAH8/PSMA6/AURKC/SPECC1/DYNLRB2/TDRD7/KATNA1/SPAG5/DYNLL2/PAFAH1B1/AHCTF1/SPAG8
GOBP_BONE_DEV	GOBP_BONE_DEV	GOBP_BONE_DEVELOP	47	0.350547116	2.204251099	0.00076781	0.01153875	0.00874647	842	tags=53%,	FBN1/SH3PXD2B/ZMPSTE24/TYROBP/

ELOPMENT	ELOPMENT	MENT				1	6	6		list=27%, signal=40%	OGN/COL3A1/LIPA/PTPRC/SULF1/ANXA6/BMP4/MYOC/CITED2/BMP2/TEK/FOXC1/MEF2D/HAS2/MMP14/CBS/PLS3/COL27A1/ENG/FOSL2/TMEM119
GOBP_MACROPHAGE_DIFFERENTIATION	GOBP_MACROPHAGE_DIFFERENTIATION	GOBP_MACROPHAGE_DIFFERENTIATION	17	0.51170219	2.194743927	0.000782077	0.01170449	0.008872104	1171	tags=82%, list=37%, signal=52%	HCLS1/EIF2AK1/C1QC/INHBA/CEBPA/BMP4/IL15/ID2/PTPN2/NR3C1/FOSL2/CD4/SOCS1/ADIPOQ
GOBP_POSITIVE_REGULATION_OF_DEFENSE_RESPONSE	GOBP_POSITIVE_REGULATION_OF_DEFENSE_RESPONSE	GOBP_POSITIVE_REGULATION_OF_DEFENSE_RESPONSE	77	0.281219437	2.07216334	0.000788575	0.01170449	0.008872104	910	tags=48%, list=29%, signal=35%	CASP4/LAG3/PHB2/LSM14A/PYCARD/CEBPB/PQBP1/HMGB1/TYROBP/HAVCR2/ALOX5AP/EIF2AK2/MAPKAPK3/PLSCR1/PUM1/HLA-E/HLA-F/CEBPA/FLOT2/NMI/COLEC11/RNF185/IL15/CYLD/IRAK1/CCL5/MEFV/CTSC/LYN/BRCC3/RELA/SIRT2/KIR2DL4/IL17A/TRIM41/ZNFX1/NFKBIL1
GOBP_REGULATION_OF_LEUKOCYTE_MIGRATION	GOBP_REGULATION_OF_LEUKOCYTE_MIGRATION	GOBP_REGULATION_OF_LEUKOCYTE_MIGRATION	39	0.355905769	2.069299375	0.00078648	0.01170449	0.008872104	1628	tags=82%, list=52%, signal=40%	MSN/PYCARD/HMGB1/ABL1/VEGFB/AIF1/ST3GAL4/RAC1/C3AR1/PTN/CCL21/CCL5/AIRE/MMP14/LYN/CXCL13/APOD/AGER/CXCR3/CCR1/SPN/CX3CR1/ADA/THY1/AKT1/OXSR1/GPSM3/S100A14/BCR/IL1R1/BDKRB1/NF1
GOBP_REGULATION_OF_RESPONSE_TO_STRESS	GOBP_REGULATION_OF_RESPONSE_TO_STRESS	GOBP_REGULATION_OF_RESPONSE_TO_STRESS	274	0.181714001	1.830390235	0.000784112	0.01170449	0.008872104	518	tags=27%, list=16%, signal=24%	NFKB1/CASP4/LAG3/UBQLN2/TFPI/PHB2/LSM14A/RHBDF2/PYCARD/CEBPB/XBP1/PQBP1/HMGB1/ZMPSTE24/TYROBP/ABL1/EIF2AK4/HAVCR2/H19/VDAC1/ALOX5AP/EIF2AK2/EIF2AK1/MAPKAPK3/PPP3CA/PLSCR1/SLC25A23/ST3GAL4/STAT3/PUM1/HLA-B/HLA-E/HLA-F/MYC/GADD45A/PTPRC/CEBPA/NFE2L2/HAND2/EIF4G1/LTBR/BFAR/ITGB1/MID1/ING4/HLA-A/IGFBP6/HIPK2/FLOT2/MAP3K5/BMP4/PTN/TFPT/ALOX5/NMI/ABHD12/SNAI2/CCL21/WFS1/COLEC11/PTGES/RNF185/IL15/CYLD/FECH/IRAK1/CLN3/BCL2L1/DEK/PRDX1/BMP2/TEK/FGL2
GOBP_DNA_CONFORMATION_CHANGE	GOBP_DNA_CONFORMATION_CHANGE	GOBP_DNA_CONFORMATION_CHANGE	27	-0.392894131	-2.240230459	0.000818585	0.012112539	0.009181408	748	tags=59%, list=24%, signal=46%	ERCC3/MCM5/RAD50/POT1/TOP3A/WRN/GTF2H2/TOP1MT/CHD1L/BLM/RUVBL1/RECQL4/RFC4/DHX30/RAD51/SETX
GOCC_UBIQUITIN_LIGASE_COMPL	GOCC_UBIQUITIN_LIGASE_COMPLE	GOCC_UBIQUITIN_LIGASE_COMPLEX	86	-0.239474062	-2.017131749	0.000868001	0.012804344	0.009705803	684	tags=38%, list=22%,	DCUN1D3/DCUN1D5/RNF7/KLHL7/TRAF2/DDA1/DERL3/SHARPIN/BARD1/K

EX	X									signal=31%	CTD10/ANAPC10/KBTBD8/PCGF6/CACYBP/FBXL2/UBE2D2/UBE2D3/RMND5B/FBXO39/UBE2N/UBE2S/FBXO7/BUB1B/FZR1/WDR26/ASB1/SUGT1/FBXL18/DERL2/RAD51/FBXO15/CKS2/FBXO24
GOMF_ODORANT_BINDING	GOMF_ODORANT_BINDING	GOMF_ODORANT_BINDING	14	0.544209907	2.126858915	0.000871283	0.012813449	0.009712704	1148	tags=86%, list=36%, signal=55%	OR9K2/OR10T2/OR8G1/OR5F1/OR8A1/OR8H2/OR8D1/OR5AS1/OR5D18/OR6K2/OR8B8/OR6B2
GOBP_BRANCHING_MORPHOGENESIS_OF_AN_EPITHELIAL_TUBE	GOBP_BRANCHING_MORPHOGENESIS_OF_AN_EPITHELIAL_TUBE	GOBP_BRANCHING_MORPHOGENESIS_OF_AN_EPITHELIAL_TUBE	28	0.415024694	2.14002352	0.000887968	0.012979445	0.00983853	919	tags=61%, list=29%, signal=43%	PHB2/ABL1/SMO/GLI2/ILK/MYC/BMP4/BMP2/CTSH/BCL2/MMP14/COL4A1/YAP1/FGF1/ENG/MAGED1/NRP1
GOBP_NEGATIVE_REGULATION_OF_MOLECULAR_FUNCTION	GOBP_NEGATIVE_REGULATION_OF_MOLECULAR_FUNCTION	GOBP_NEGATIVE_REGULATION_OF_MOLECULAR_FUNCTION	133	0.23535605	2.007785369	0.000887469	0.012979445	0.00983853	1405	tags=61%, list=45%, signal=35%	NFKB1/PHB2/DAB2/PYCARD/ABL1/EIF2AK4/GSTO1/SMO/HAVCR2/H19/CYP1B1/ATP1A2/ITGB1BP1/HEG1/GADD45A/PTPRC/CEBPA/HP/FKBP1A/NEIL1/CFHR5/ANXA4/KCNE3/WFS1/GNL3L/CYLD/TSC1/ADAM15/IRAK1/CLN3/CSN2/PRMT2/PALM/BMP2/DHCR24/MITD1/ANGPTL4/SRI/ID2/LYN/SERPINA6/ATP2A3/AOC3/PPP2R5A/PRDX3/CDKN1C/SERPINA1/EOMES/IFI6/HSPB1/SERPINE2/NFKBIL1/CALM3/PPIF/INS/BAX/PNKP/RWDD3/ERRFI1/PRKAR1A/ROCK2/DDX3X/ARRB2/SMAD7/ADIPOQ/CHMP6/SERPINB8/ACTN2/RGS14/AQP1/SERPINB10/PXK/TIMP1/MEN1/TBX6/THY1/TNFRSF4/AKT1/OXSR1/MT3/CAV3
GOBP_RNA_SPLICING_VIA_TRANSESTERIFICATION_REACTIONS	GOBP_RNA_SPLICING_VIA_TRANSESTERIFICATION_REACTIONS	GOBP_RNA_SPLICING_VIA_TRANSESTERIFICATION_REACTIONS	50	-0.300120569	-2.112018139	0.000892209	0.013001916	0.009855564	349	tags=30%, list=11%, signal=27%	SF3A1/GEMIN4/GEMIN6/PRPF18/PRPF40B/TFIP11/SRPK1/DHX16/SETX/PRPF38A/BUD13/DAZAP1/DDX20/KHDRBS3/NCBP2
GOBP_INTEGRIN_MEDIATED_SIGNALING_PATHWAY	GOBP_INTEGRIN_MEDIATED_SIGNALING_PATHWAY	GOBP_INTEGRIN_MEDIATED_SIGNALING_PATHWAY	28	0.4142475	2.136016016	0.000903146	0.013121544	0.009946243	1503	tags=82%, list=48%, signal=43%	DAB2/ABL1/EMP2/COL3A1/ITGB1BP1/ILK/ITGB1/PTN/ITGA9/CD177/ADAM15/ITGB7/ISG15/ITGA3/ITGA5/NRP1/ITGAM/ITGB8/TIMP1/THY1/TSPAN32/PHACTR4/LIMS2
GOBP_ANTIGEN_PROCESSING_AND_PRESENTATION	GOBP_ANTIGEN_PROCESSING_AND_PRESENTATION	GOBP_ANTIGEN_PROCESSING_AND_PRESENTATION	34	0.378062264	2.112241402	0.000907949	0.013151583	0.009969012	701	tags=53%, list=22%, signal=42%	PYCARD/HLA-DMB/HLA-DRB3/HLA-DQB1/HLA-B/HLA-E/HLA-F/HLA-DRB5/HLA-DMA/HLA-A/HLA-C/CCL21/FGL2/HLA-DOB/SLC11A1/CTSH/TAPBPL/FCER1G

HP_INGUINAL_HERNIA	HP_INGUINAL_HERNIA	HP_INGUINAL_HERNIA	65	0.306159993	2.140440765	0.000916806	0.013240007	0.010036038	800	tags=48%, list=25%, signal=36%	FBN1/PLOD1/MAF/SETBP1/SGSH/NFIA/FGFR1/SH3PXD2B/IGF2/MED12/H19/GLB1/ATP1A2/COL3A1/BAZ1B/MBTPS1/PIGW/GNE/FBXW11/MID1/GTF2IRD2/TOR1A/CTCF/ELN/PLAG1/CBS/LTBP1/LFNG/CDKN1C/PPP2R1A/PTDSS1
GOBP_DNA_TEMPLATED_TRANSCRIPTION_ELONGATION	GOBP_DNA_TEMPLATED_TRANSCRIPTION_ELONGATION	GOBP_DNA_TEMPLATED_TRANSCRIPTION_ELONGATION	20	-0.468903948	-2.341337981	0.000919918	0.013245161	0.010039945	887	tags=70%, list=28%, signal=51%	ELL/MED4/PARP1/ERCC3/IWS1/SUPT5H/THOC5/ADRM1/PAF1/ZMYND11/INTS7/INTS6/ELL3/NCBP2
GOBP_POSITIVE_REGULATION_OF_ERK1_AND_ERK2_CASCADE	GOBP_POSITIVE_REGULATION_OF_ERK1_AND_ERK2_CASCADE	GOBP_POSITIVE_REGULATION_OF_ERK1_AND_ERK2_CASCADE	38	0.377113545	2.181189886	0.000931475	0.013371533	0.010135736	1402	tags=74%, list=44%, signal=41%	SHC1/PDGFRB/PHB2/PYCARD/HMGB1/ABL1/HAVCR2/INHBA/PTPRC/HAND2/BMP4/CCL21/BMP2/TEK/CCL5/FGF1/AGER/NRP1/CCR1/CD4/FGF19/FSHR/ARRB2/CCL8/MFHAS1/CCL22/PDGFRA/MT3
GOBP_MITOTIC_SISTER_CHROMATID_SEPARATION	GOBP_MITOTIC_SISTER_CHROMATID_SEPARATION	GOBP_MITOTIC_SISTER_CHROMATID_SEPARATION	17	-0.492876898	-2.320851438	0.000951236	0.01361457	0.010319961	886	tags=76%, list=28%, signal=55%	LCMT1/CDC16/UBE2C/ZW10/CDK5RAP2/MAD2L1BP/ESPL1/MAD2L2/DYNC1LI1/TRIP13/BUB1B/TPR/CDCA8
HP_ECTRODACTYLY	HP_ECTRODACTYLY	HP_ECTRODACTYLY	15	0.541707813	2.169663017	0.000964641	0.013736371	0.010412287	779	tags=67%, list=25%, signal=50%	FGFR1/IGF2/MED12/CTBP1/SBF2/PRX/PLAG1/PIGG/CDKN1C/CDH3
GOBP_POSITIVE_REGULATION_OF_CHEMOTAXIS	GOBP_POSITIVE_REGULATION_OF_CHEMOTAXIS	GOBP_POSITIVE_REGULATION_OF_CHEMOTAXIS	25	0.433711606	2.147365333	0.000965459	0.013736371	0.010412287	1504	tags=88%, list=48%, signal=46%	FGFR1/PDGFRB/HMGB1/VEGFB/AIF1/RAC1/C3AR1/PTN/CCL21/CCL5/CXCL13/NTF3/HSPB1/AGER/NRP1/CCR1/CX3CR1/PDGFRA/OXSR1/GPSM3/S100A14/STX3
GOBP_NEGATIVE_REGULATION_OF_MYELOID_LEUKOCYTE_DIFFERENTIATION	GOBP_NEGATIVE_REGULATION_OF_MYELOID_LEUKOCYTE_DIFFERENTIATION	GOBP_NEGATIVE_REGULATION_OF_MYELOID_LEUKOCYTE_DIFFERENTIATION	12	0.581153435	2.132251585	0.000972372	0.013793908	0.0104559	722	tags=75%, list=23%, signal=58%	FBN1/MAFB/C1QC/INHBA/MYC/PIAS3/PTPN2/LYN/TCTA
GOBP_CARDIAC_CHAMBER_DEVELOPMENT	GOBP_CARDIAC_CHAMBER_DEVELOPMENT	GOBP_CARDIAC_CHAMBER_DEVELOPMENT	38	0.374004622	2.163208165	0.000990149	0.01400479	0.010615751	919	tags=58%, list=29%, signal=42%	PARVA/ZMPSTE24/SMO/HEG1/HAND2/FKBP1A/BMP4/NKX2-5/CITED2/ZFPM2/BMP2/TEK/FOXC1/NRP2/MAML1/ENG/FRS2/WNT11/ADPRHL1/MDM2/PKP2/NRP1
GOBP_NERVE_DEVELOPMENT	GOBP_NERVE_DEVELOPMENT	GOBP_NERVE_DEVELOPMENT	17	0.504438035	2.163587208	0.000998885	0.014086911	0.010677999	919	tags=71%, list=29%, signal=50%	MAFB/PLXNA1/ILK/SULF1/NAV2/CITED2/CHRNB2/NRP2/NTF3/ATP8B1/SERPINE2/NRP1
GOBP_CHEMOKINE_PRODUCTION	GOBP_CHEMOKINE_PRODUCTION	GOBP_CHEMOKINE_PRODUCTION	19	0.486323073	2.148777202	0.001016048	0.014203993	0.010766748	1341	tags=84%, list=43%,	PYCARD/HMGB1/HAVCR2/AIF1/EIF2AK2/SNAI2/MEFV/AIRE/APOD/IL17A/A

										signal=49%	GER/DDX3X/ADIPOQ/EGR1/FFAR2/CHIA
GOBP_ENDOTHELIAL_CELL_APOPTOTIC_PROCESS	GOBP_ENDOTHELIAL_CELL_APOPTOTIC_PROCESS	GOBP_ENDOTHELIAL_CELL_APOPTOTIC_PROCESS	10	0.612345309	2.109623259	0.001014237	0.014203993	0.010766748	568	tags=70%, list=18%, signal=58%	ABL1/CD248/NFE2L2/MAP3K5/RAMP2/TEK/ANGPTL4
GOBP_RESPONSE_TO_LIPID	GOBP_RESPONSE_TO_LIPID	GOBP_RESPONSE_TO_LIPID	181	0.204934814	1.915678578	0.001010271	0.014203993	0.010766748	1564	tags=63%, list=50%, signal=34%	NFKB1/MSN/KLF9/AKR1C1/CDK4/TFPI/PHB2/DAB2/MAOB/PYCARD/CEBPB/XBP1/HMGB1/ABL1/SMO/HAVCR2/CYP1B1/GLB1/CAT/MAPKAPK3/SOCS2/TSPO/ATP1A2/LIPA/STAT3/INHBA/MBD2/PTPRC/HAND2/NQO1/FMO1/IGF2R/SNAI2/CCL21/FECH/ADAM15/IRAK1/PRMT2/ZBTB7A/ALAD/SLC11A1/CTSH/BCL2L2/LY86/BCL2/AANAT/FZD4/LYN/GPR83/KMO/SCGB1A1/RELA/BCHE/FAM107A/NR3C1/CXCL13/PRDX3/YAP1/GNAI1/CCR5/FOSL2/ADM/TRIM41/WNT11/NCOA3/ARPC1B/MDM2/NFKBIL1/PMVK/BAD/UCN3/MAP2K3/ACSL1/ROCK2/GNB1/MYOG/VPS11/NPAS4/MMP15/ALAS1/CLDN4/DDX54/ADIPOQ/GRAMD1A/GHSR/CX3CR1/SLC6A1/ZNF703/UBE2L3/AQP1/ASCL1/TSHB/HTR1B/LTA/ELK1/VPS18/FFAR2/SSTR3/AKT1/WNT3A/S100A14/RARG/LTF/MYOD1/BCR/LBH/CD6/CAD/SLC12A3/PAQR8/CNR2/RWDD1/ATP1A3/PPARA
GOBP_RESPONSE_TO_BACTERIUM	GOBP_RESPONSE_TO_BACTERIUM	GOBP_RESPONSE_TO_BACTERIUM	117	0.233739398	1.940768146	0.001021085	0.014233043	0.010788768	1427	tags=62%, list=45%, signal=35%	SHC1/FUCA2/NFKB1/CASP4/CDK4/MAOB/PYCARD/CEBPB/XBP1/HMGB1/ABL1/HAVCR2/RAB1A/MAPKAPK3/TSPO/HLA-B/HLA-E/STAB1/HP/ADAMTS5/TFEB/HLA-A/IFI44/NQO1/FMO1/IRAK1/BMP2/ALAD/SLC11A1/ISG15/LY86/LYN/KMO/SCGB1A1/FCER1G/RELA/PI3/HERC6/CXCL13/PRDX3/OCIAD2/CCR5/LRG1/FOSL2/IL17A/ADM/TRIM41/ZNFX1/RNF5/NFKBIL1/MAP2K3/CD4/SPN/OAS2/MMRN2/FGF19/MPEG1/DEFB105A/ADIPOQ/GHSR/CX3CR1/AQP1/REG4/LTA/ANG/AKT1/S100A12/FKBP5/TAP2/C3/S100A14/LTF
GOBP_IMMUNE_	GOBP_IMMUNE_R	GOBP_IMMUNE_RESPO	87	0.267665733	2.033765144	0.00103085	0.01432771	0.01086053	823	tags=45%,	NFKB1/PHB2/LSM14A/PYCARD/HMGB

RESPONSE_REGULATING_SIGNALING_PATHWAY	ESPONSE_REGULATING_SIGNALING_PATHWAY	NSE_REGULATING_SIGNALING_PATHWAY				7	5			list=26%, signal=34%	1/TYROBP/ABL1/HAVCR2/HLA-DRB3/EIF2AK2/HLA-DQB1/MAPKAPK3/PLSCR1/LIPA/PUM1/PTPRC/C3AR1/BTNL2/HLA-A/FLOT2/NMI/PRKCH/COLEC11/CYLD/IRAK1/CTSH/BCL2/MEFV/BTN2A2/PTPN2/LYN/EIF2B1/FCER1G/BRCC3/RELA/BTN1A1/SIRT2/FOSL2/CD276
GOBP_NEGATIVE_REGULATION_OF_CELL_CYCLE	GOBP_NEGATIVE_REGULATION_OF_CELL_CYCLE	GOBP_NEGATIVE_REGULATION_OF_CELL_CYCLE	82	-0.247706217	-2.058745257	0.001036701	0.014367417	0.010890625	759	tags=46%, list=24%, signal=36%	ATR/SIRT1/TIMELESS/RAD50/TAF6/TP53BP1/ZW10/DCUN1D3/E2F1/CDK5RAP2/ZBTB17/RBM14/MAD2L1BP/PINX1/THOC5/BRD7/ESPL1/BARD1/MAD2L2/DYNC1LI1/CEP63/BLM/TRIP13/DONSON/INTS7/FBXO7/BUB1B/FZR1/MDC1/RAD51/GMNN/TPR/CRY1/CDCA8/DOT1L/CDC14B/RAD17/AURKAIP1
GOMF_CATALYTIC_ACTIVITY_ACTING_ON_DNA	GOMF_CATALYTIC_ACTIVITY_ACTING_ON_DNA	GOMF_CATALYTIC_ACTIVITY_ACTING_ON_DNA	49	-0.323791879	-2.262859149	0.001070194	0.014772437	0.011197633	897	tags=57%, list=28%, signal=42%	MAP1S/MBD4/RFC1/ENDOG/POLI/ERCC3/CHD5/MCM5/RAD50/TOP3A/WRN/TOP1MT/SMC3/POLE/PCNA/CHD1L/CDK7/BLM/RUVBL1/RECQL4/RFC4/POLG2/DHX30/POLB/RAD51/CRY1/SETX/RAD17
HP_ABNORMALITY_OF_BLOOD_CIRCULATION	HP_ABNORMALITY_OF_BLOOD_CIRCULATION	HP_ABNORMALITY_OF_BLOOD_CIRCULATION	84	0.269046741	2.02453249	0.001072069	0.014772437	0.011197633	720	tags=40%, list=23%, signal=32%	FBN1/PLOD1/F10/NFIA/RHBDF2/MED12/COL4A2/IFNGR1/ZMPSTE24/EIF2AK4/SMO/CFHR3/COL3A1/STAT3/HLA-B/MYC/STT3A/NKX2-5/HPS1/CITED2/WFS1/TSC1/VWF/TEK/CTCF/ELN/CYP11A1/MEFV/FZD4/CBS/CDKN2C/PLAU/BRCC3/COL4A1
GOBP_REGULATION_OF_SISTER_CHROMATID_SEGREGATION	GOBP_REGULATION_OF_SISTER_CHROMATID_SEGREGATION	GOBP_REGULATION_OF_SISTER_CHROMATID_SEGREGATION	24	-0.433073588	-2.350554219	0.001097712	0.01493457	0.011320532	886	tags=67%, list=28%, signal=48%	LCMT1/CDC16/UBE2C/ZW10/SMARCC1/CDK5RAP2/MAD2L1BP/BRD7/ESPL1/MAD2L2/DYNC1LI1/TRIP13/CENPE/BUB1B/TPR/CDCA8
GOBP_REGULATION_OF_SUBSTRATE_ADHESION_DEPENDENT_CELL_SPREADING	GOBP_REGULATION_OF_SUBSTRATE_ADHESION_DEPENDENT_CELL_SPREADING	GOBP_REGULATION_OF_SUBSTRATE_ADHESION_DEPENDENT_CELL_SPREADING	16	0.527870456	2.203655338	0.001097363	0.01493457	0.011320532	919	tags=75%, list=29%, signal=53%	DAB2/ABL1/RAC1/ITGB1BP1/ILK/MYOC/TRIOBP/MYADM/HAS2/ARHGEF7/PKP2/NRP1
HP_ABNORMAL_CORNEAL_EPITHELIUM_MORPHOLOGY	HP_ABNORMAL_CORNEAL_EPITHELIUM_MORPHOLOGY	HP_ABNORMAL_CORNEAL_EPITHELIUM_MORPHOLOGY	24	0.435762793	2.117454544	0.001090707	0.01493457	0.011320532	596	tags=50%, list=19%, signal=41%	RNF113A/FBN1/IFNGR1/ZMPSTE24/TGFBI/COL4A5/HLA-B/BTNL2/COL4A6/MPV17/MEFV/AIRE
HP_LOCALIZED_S	HP_LOCALIZED_S	HP_LOCALIZED_SKIN_	201	0.195716738	1.853506302	0.00109209	0.01493457	0.01132053	930	tags=40%,	FBN1/NFKB1/PLOD1/ANTXR2/SETBP1/

KIN_LESION	KIN_LESION	LESION				4		2		list=29%, signal=30%	CDK4/FGFR1/SH3PXD2B/PDGFRB/CDH11/IGF2/MED12/MGP/IFNGR1/CTBP1/ZMPSTE24/ABL1/EIF2AK4/SMO/COX7B/HAVCR2/H19/KRT14/CYP1B1/MAFB/HLA-DQB1/PERP/COL3A1/BAZ1B/STAT3/HLA-B/COL6A3/STEAP3/BTNL2/MID1/MRAS/GTF2IRD2/NKX2-5/HPS1/MYOC/CITED2/MMP23B/ZFPM2/SNAI2/CYLD/TSC1/SBF2/TEK/DHCR24/IDH3B/CTCF/EDA/MPV17/ELN/PPOX/BCL2/MEFV/AIRE/PLAG1/ZNF462/MMP14/CTSC/CDKN2C/GALC/PIGG/NR3C1/SLC29A3/CDKN1C/CDH3/PRR12/IMPG2/PTDSS1/STAG2/LDHA/SLURP1/SLC7A14/NSDHL/MDM2/PMVK/CCR1
GOBP_LIPID_METABOLIC_PROCESS	GOBP_LIPID_METABOLIC_PROCESS	GOBP_LIPID_METABOLIC_PROCESS	269	0.182735047	1.832300959	0.001099363	0.01493457	0.011320532	972	tags=41%, list=31%, signal=31%	NFKB1/ALDH1A3/SUMF1/SLC44A1/AKR1A1/OSBPL5/AKR1C1/ST6GALNAC6/CBR1/PDGFRB/PHB2/DAB2/XBP1/ZMPSTE24/LTC4S/CYP1B1/ALOX5AP/GLB1/OSBPL1A/CAT/PLSCR1/ST3GAL4/PAM/SOCS2/ATG4A/TSPO/LIPA/ACSL5/ECHDC1/CYP2U1/MBTPS1/CEBPA/ADHFE1/PLTP/PIGW/VAPA/RBP1/AIG1/PPT2/NFE2L1/ALOX5/FMO1/ABHD12/PTGES2/ACAA1/SNAI2/CCL21/PTGES/HMGCL/FECH/CCDC3/CLN3/BMP2/TEK/DHCR24/CYP2W1/BCAT2/ABHD6/ANGPTL4/CYP11A1/PNPLA4/ID2/CYP17A1/STARD4/UGT2A3/UVRAG/SERPINA6/LIMA1/CYP4B1/GALC/PIGG/PIK3C2B/NR3C1/PRKAB2/PPP2R5A/SIRT2/PLA2G4C/PPP2R1A/APOD/CYP2S1/FGF1/SLC16A11/PTDSS1/ALG2/ADM/ILVBL/ATP8B1/AKR7A2/SCP2/NSDHL/ADH1C/LTA4H/CRTC3/ECH1/PMVK/ACOX1/NR1H2/OSBP/FADS6/INPPL1/STUB1/OMA1/NUDT8/HSD17B8/ACAD11/INS/CYP3A43/ACSL1/BAX/PDK2
GOBP_MICROTUBULE_CYTOSKELETON_ORGANIZATION_INVOLVED_I	GOBP_MICROTUBULE_CYTOSKELETON_ORGANIZATION_INVOLVED_I	GOBP_MICROTUBULE_CYTOSKELETON_ORGANIZATION_INVOLVED_IN_MITOSIS	45	-0.325121446	-2.243363587	0.001120659	0.01516263	0.011493403	921	tags=60%, list=29%, signal=43%	MAPRE1/CHEK2/MAP1S/VCP/SPAST/NUSAP1/BCCIP/ZW10/CDK5RAP2/KIF4A/SMC3/TUBG2/ESPL1/KPNB1/CENPH/TPX2/NDE1/KIF23/TUBG1/CENPE/EFHC

N_MITOSIS	N_MITOSIS										1/TPR/AURKC/CDCA8/RAE1/PAFAH1B1/WDR62
GOMF_OXIDOREDUCTASE_ACTIVITY_ACTING_ON_A_SULFUR_GROUP_OF_DONORS	GOMF_OXIDOREDUCTASE_ACTIVITY_ACTING_ON_A_SULFUR_GROUP_OF_DONORS	GOMF_OXIDOREDUCTASE_ACTIVITY_ACTING_ON_A_SULFUR_GROUP_OF_DONORS	13	0.577753569	2.206239963	0.001122457	0.01516263	0.011493403	763	tags=77%, list=24%, signal=59%	SUMF1/TXNDC5/MSRB2/PGK1/GSTO1/STAB1/PDIA6/CCS/PRDX3/PDIA5
GOCC_U2_TYPE_SPLICEOSOMAL_COMPLEX	GOCC_U2_TYPE_SPLICEOSOMAL_COMPLEX	GOCC_U2_TYPE_SPLICEOSOMAL_COMPLEX	17	-0.487387127	-2.295001286	0.001129244	0.015211576	0.011530505	1029	tags=82%, list=33%, signal=56%	LUC7L2/LUC7L/PRPF6/SF3B4/SF3B2/LSM3/PRPF40A/SF3A1/PRPF18/PRPF40B/TFIP11/DHX16/PRPF38A/BUD13
GOBP_REGULATION_OF_DNA_REPAIR	GOBP_REGULATION_OF_DNA_REPAIR	GOBP_REGULATION_OF_DNA_REPAIR	49	-0.322297213	-2.252413496	0.00114037	0.01531854	0.011611584	1138	tags=69%, list=36%, signal=45%	SUPT7L/ATXN7/NUDT16L1/DDX11/ACTL6B/CBX8/RIF1/MCRS1/SENP3/PPP4C/PARP1/ATR/SIRT1/CEBPG/TIMELESS/TAF9/TAF6/TP53BP1/POT1/SMARCC1/TAF10/CCDC117/BRD7/TRIM28/BARD1/MAD2L2/PCNA/RUVBL1/USP1/UBE2N/TFIP11/RAD51/SUPT3H/SMCHD1
GOBP_LEUKOCYTE_MIGRATION	GOBP_LEUKOCYTE_MIGRATION	GOBP_LEUKOCYTE_MIGRATION	74	0.287583223	2.089854276	0.001153445	0.015451027	0.011712011	1628	tags=76%, list=52%, signal=37%	MSN/PYCARD/HMGB1/ABL1/EMP2/VEGFB/AIF1/ST3GAL4/FOLR2/RAC1/CD34/C3AR1/ITGB1/PTN/ALOX5/CCL21/ITGA9/CD177/ITGB7/CCL5/AIRE/MMP14/LYN/TRPM2/FCER1G/CXCL13/GPR15/ITGA3/APOD/CCR5/IL17A/AGER/CXCR3/CCR1/SPN/CCL8/CX3CR1/ADA/JAGN1/FFAR2/CCL22/THY1/SELPLG/AKT1/S100A12/OXSR1/GPSM3/S100A14/F11R/PLA2G1B/BCR/CNR2/IL1R1/EPS8/BDKRB1/NF1
GOBP_NEGATIVE_REGULATION_OF_EPITHELIAL_CELL_APOPTOTIC_PROCESS	GOBP_NEGATIVE_REGULATION_OF_EPITHELIAL_CELL_APOPTOTIC_PROCESS	GOBP_NEGATIVE_REGULATION_OF_EPITHELIAL_CELL_APOPTOTIC_PROCESS	14	0.532893674	2.082633277	0.001169552	0.015623272	0.011842574	764	tags=71%, list=24%, signal=54%	ABL1/NFE2L2/HAND2/NKX2-5/WFS1/RAMP2/TEK/ANGPTL4/BCL2/YAP1
GOMF_PROTEIN_FOLDING_CHAPERONE	GOMF_PROTEIN_FOLDING_CHAPERONE	GOMF_PROTEIN_FOLDING_CHAPERONE	13	-0.564462875	-2.283993517	0.001173429	0.015631636	0.011848914	427	tags=62%, list=14%, signal=53%	HSPH1/CLPX/DNAJB6/HSPA14/DNAJB8/HSPA2/ZMYND10/HSPA1L
GOBP_MITOTIC_NUCLEAR_DIVISION	GOBP_MITOTIC_NUCLEAR_DIVISION	GOBP_MITOTIC_NUCLEAR_DIVISION	74	-0.253766671	-2.036272072	0.001199456	0.015934205	0.012078264	921	tags=54%, list=29%, signal=39%	MAPRE1/SMC4/CHEK2/LCMT1/CDC16/REEP4/SPAST/UBE2C/NUSAP1/BCCIP/ZW10/AKAP8L/CDK5RAP2/KIF22/KIF4A/SMC3/MAD2L1BP/PINX1/TUBG2/ESPL1/MAD2L2/DYNC1LI1/KPNB1/TPX2/TRIP13/NDE1/KIF23/TUBG1/UBE2S/CE

											NPE/BUB1B/KATNB1/TPR/AURKC/CDCA8/RANBP1/CDC14B/CDC25C/SPAG5/AURKAIP1
GOBP_REGULATION_OF_RESPONSE_TO_BIOTIC_STIMULUS	GOBP_REGULATION_OF_RESPONSE_TO_BIOTIC_STIMULUS	GOBP_REGULATION_OF_RESPONSE_TO_BIOTIC_STIMULUS	97	0.25159233	1.982156282	0.00121426	0.016086432	0.012193654	977	tags=48%, list=31%, signal=35%	LAG3/PHB2/LSM14A/PYCARD/PQBP1/HMGB1/ZMPSTE24/TYROBP/EIF2AK4/HAVCR2/HLA-DRB3/EIF2AK2/MAPKAPK3/PLSCR1/PUM1/HLA-B/HLA-E/HLA-F/NFE2L2/HLA-A/FLOT2/NMI/COLEC11/RNF185/IL15/CYLD/IRAK1/FGL2/CCL5/ISG15/LY86/MEFV/PTPN2/LYN/BRCC3/RELA/SIRT2/KIR2DL4/IL17A/TRIM41/ZNFX1/NFKBIL1/NR1H2/INS/OTOP1/TRAFD1/SPN
GOBP_REGULATION_OF_DEFENSE_RESPONSE	GOBP_REGULATION_OF_DEFENSE_RESPONSE	GOBP_REGULATION_OF_DEFENSE_RESPONSE	135	0.22750135	1.95647778	0.001221921	0.016143452	0.012236875	977	tags=45%, list=31%, signal=33%	NFKB1/CASP4/LAG3/PHB2/LSM14A/RHBDF2/PYCARD/CEBPB/PQBP1/HMGB1/ZMPSTE24/TYROBP/EIF2AK4/HAVCR2/ALOX5AP/EIF2AK2/MAPKAPK3/PLSCR1/STAT3/PUM1/HLA-B/HLA-E/HLA-F/PTPRC/CEBPA/NFE2L2/HLA-A/FLOT2/ALOX5/NMI/ABHD12/COLEC11/PTGES/RNF185/IL15/CYLD/IRAK1/TEK/FGL2/CCL5/ISG15/MEFV/PTPN2/CTSC/LYN/SCGB1A1/BRCC3/RELA/IER3/SIRT2/KIR2DL4/IL17A/TRIM41/AGER/ZNFX1/NFKBIL1/NR1H2/INS/OTOP1/TRAFD1/SPN
GOBP_RNA_EXPORT_FROM_NUCLEUS	GOBP_RNA_EXPORT_FROM_NUCLEUS	GOBP_RNA_EXPORT_FROM_NUCLEUS	20	-0.458863702	-2.291204883	0.001236269	0.016288273	0.01234665	1236	tags=70%, list=39%, signal=43%	XPO1/NUP107/PABPN1/IWS1/SMG7/AKAP8L/NUP93/THOC5/NOL6/TPR/NUP88/RAE1/NUP155/NCBP2
HP_ABNORMAL_BLOOD_VESSEL_MORPHOLOGY	HP_ABNORMAL_BLOOD_VESSEL_MORPHOLOGY	HP_ABNORMAL_BLOOD_VESSEL_MORPHOLOGY	188	0.200103667	1.870780903	0.001245125	0.016360124	0.012401114	1092	tags=46%, list=35%, signal=32%	FBN1/NFKB1/PLOD1/MAF/NFIA/FGFR1/SH3PXD2B/PDGFRB/OSGEP/DPH5/HTRA1/IGF2/MED12/MGP/PQBP1/IFNGR1/ZMPSTE24/ABL1/EIF2AK4/COX7B/CYP1B1/ITPR1/GLB1/GLI2/CAT/COL3A1/LIPA/BAZ1B/STAT3/RAC1/HLA-B/SSR4/MID1/MRAS/GTF2IRD2/NKX2-5/PLAGL1/MYOC/CITED2/MMP23B/ZFPM2/TPM2/TSC1/BMP2/TEK/DHCR24/ZBTB7A/CTCF/FOXC1/SLC34A2/ELN/MEFV/LARS2/ZNF462/MMP14/FZD4/CBS/GALC/BRCC3/TRIO/COL4A1/SLC29A3/LTBP1/LFNG/PPP2R1A/SEC61A1/PTDSS1/ENG

											/STAG2/WDR37/ARPC1B/PKP2/NFKBIL1/CCR1/CALM3/HEATR3/TALDO1/SEC63/ABCD4/EHMT1/CDC42BPB/UBE3B/NIPA2/PRKAR1A/DDX3X/SMC1A
GOBP_REGULATION_OF_LEUKOCYTE_PROLIFERATION	GOBP_REGULATION_OF_LEUKOCYTE_PROLIFERATION	GOBP_REGULATION_OF_LEUKOCYTE_PROLIFERATION	49	0.33875973	2.168883752	0.001265846	0.01649801	0.012505633	694	tags=47%, list=22%, signal=37%	TYK2/EFNB1/PYCARD/IGF2/CEBPB/HLA-DMB/HMGB1/TYROBP/HAVCR2/AIF1/PPP3CA/HLA-E/PTPRC/HLA-A/BMP4/IL15/BCL2L1/CCL5/CHRNB2/BCL2/BTN2A2/LYN/SCGB1A1
GOBP_POSITIVE_REGULATION_OF_PROTEIN_MODIFICATION_BY_SMALL_PROTEIN_CONJUGATION_OR_REMOVAL	GOBP_POSITIVE_REGULATION_OF_PROTEIN_MODIFICATION_BY_SMALL_PROTEIN_CONJUGATION_OR_REMOVAL	GOBP_POSITIVE_REGULATION_OF_PROTEIN_MODIFICATION_BY_SMALL_PROTEIN_CONJUGATION_OR_REMOVAL	36	-0.332515336	-2.125390317	0.001264158	0.01649801	0.012505633	841	tags=58%, list=27%, signal=43%	VCP/RCHY1/UBE2C/TBC1D7/HSPBP1/GSK3B/TRAF6/DCUN1D3/DCUN1D5/PINK1/BIRC8/MAGEC2/MAGEA2B/UBE3A/PAXIP1/UBE2N/UBE2S/FZR1/SAE1/CRY1/CDC14B
GOBP_CELLULAR_OXIDANT_DETOXIFICATION	GOBP_CELLULAR_OXIDANT_DETOXIFICATION	GOBP_CELLULAR_OXIDANT_DETOXIFICATION	20	0.465615999	2.100880216	0.001265911	0.01649801	0.012505633	505	tags=55%, list=16%, signal=46%	GSTO1/LTC4S/H19/ALOX5AP/CAT/HP/NFE2L2/NQO1/PTGES/PRDX1/CCS
HP_IMPAIRMENT_IN_PERSONALITY_FUNCTIONING	HP_IMPAIRMENT_IN_PERSONALITY_FUNCTIONING	HP_IMPAIRMENT_IN_PERSONALITY_FUNCTIONING	133	0.230940524	1.970117218	0.001283522	0.016682321	0.012645342	1101	tags=49%, list=35%, signal=33%	SETBP1/UBQLN2/FGFR1/PDGFRB/GALT/TBC1D2B/HTRA1/MED12/PQBP1/PGK1/TYROBP/SMO/EIF2AK1/GLI2/HLA-DQB1/ATP1A2/IMPDH2/BAZ1B/HLA-B/EIF4G1/GTF2IRD2/HS6ST1/WFS1/PURA/TSC1/IRAK1/CLN3/TOR1A/ALAD/SLC11A1/CHRNB2/ELN/PPOX/PODXL/VPS13C/CBS/DNAJC5/CDKN2C/GALC/EIF2B1/DNAJC13/NR3C1/PRR12/SERPINA1/STAG2/NFASC/GCLC/PROKR2/SLC6A17/ATP8B1/RRM2B/ADH1C/CHMP2B/SQSTM1/NHLH2/STUB1/DAO/ITGAM/EHMT1/SLITRK2/CDC42BPB/PRKAR1A/SMC1A/GNB1/KISS1R
HP_PRIMARY_MICROCEPHALY	HP_PRIMARY_MICROCEPHALY	HP_PRIMARY_MICROCEPHALY	26	-0.390566935	-2.182899026	0.00129473	0.016782637	0.012721382	726	tags=62%, list=23%, signal=48%	NUP37/PIGH/YRDC/TUBB/CDK5RAP2/SLC1A4/SMC3/CEP63/NDE1/TPRKB/CENPE/ASNS/FZR1/KATNB1/SLC25A19/CLPB
GOCC_TRANSPORT_VESICLE_MEMBRANE	GOCC_TRANSPORT_VESICLE_MEMBRANE	GOCC_TRANSPORT_VESICLE_MEMBRANE	53	0.313177247	2.041061406	0.001310799	0.016945251	0.012844645	679	tags=42%, list=22%, signal=33%	SEC22B/HLA-DRB3/RAB1A/ITPR1/HLA-DQB1/PAM/HLA-B/HLA-E/HLA-F/HLA-DRB5/HLA-A/HLA-C/TGFA/CLTB/WFS1/BCL2L1/AP1G2/RPH3AL/FER1L4/CNIH3/DNAJC5/SYT12

GOBP_CHROMOSOME_SEPARATION	GOBP_CHROMOSOME_SEPARATION	GOBP_CHROMOSOME_SEPARATION	22	-0.437090962	-2.2524575	0.001317912	0.016946091	0.012845282	920	tags=73%, list=29%, signal=52%	SMC4/LCMT1/CDC16/UBE2C/ZW10/TOP3A/CDK5RAP2/MAD2L1BP/ESPL1/MAD2L2/DYNC1LI1/TRIP13/BUB1B/CSNK2A2/TPR/CDCA8
GOBP_MONONUCLEAR_CELL_DIFFERENTIATION	GOBP_MONONUCLEAR_CELL_DIFFERENTIATION	GOBP_MONONUCLEAR_CELL_DIFFERENTIATION	88	0.261158149	1.995283796	0.001316992	0.016946091	0.012845282	1336	tags=59%, list=42%, signal=35%	CTSK/LAG3/CEBPB/XBP1/HMGB1/ABL1/MAFB/GLI2/SLC25A5/LIPA/STAT3/HLA-B/INHBA/MYC/PTPRC/LTBR/ITGB1/BMP4/CBFB/IL15/TSC1/ZBTB7A/FGL2/BCL2/AIRE/BTN2A2/ID2/PTPN2/MMP14/LYN/TRPM2/FCER1G/LFNG/FOSL2/EOMES/AGER/BATF2/BAD/CD4/BAX/SPN/CD83/ITGB8/TNFSF13B/SOCS1/SMAD7/ATF2/EGR1/ADA/TOP2B/MEN1/CD79A
GOBP_HEMATOPOIETIC_OR_LYMPHOID_ORGAN_DEVELOPMENT	GOBP_HEMATOPOIETIC_OR_LYMPHOID_ORGAN_DEVELOPMENT	GOBP_HEMATOPOIETIC_OR_LYMPHOID_ORGAN_DEVELOPMENT	23	0.431604782	2.051811492	0.00135454	0.017370619	0.013167078	596	tags=57%, list=19%, signal=46%	ZMPSTE24/ABL1/MAFB/CD248/LIPA/PTPRC/HAND2/LTBR/NKX2-5/CITED2/IL15/BCL2/AIRE
GOBP_ORGANIC_HYDROXY_COMPOUND_TRANSPORT	GOBP_ORGANIC_HYDROXY_COMPOUND_TRANSPORT	GOBP_ORGANIC_HYDROXY_COMPOUND_TRANSPORT	56	0.306794863	2.035444578	0.001381999	0.017675614	0.013398267	1282	tags=62%, list=41%, signal=38%	NFKB1/SLC44A1/OSBPL5/AKR1C1/DAB2/MAOB/OSBPL1A/TSPO/LIPA/PLTP/VAPA/KCNB1/TOR1A/CHRNB2/SLC2A13/HRH3/STARD4/LIMA1/SYT12/SLC29A3/ATP8B1/SCP2/NR1H2/OSBP/FGF19/SLC10A5/ADIPOQ/VPS4A/VAPB/GRAMD1A/GHSR/AQP1/ADRA2B/HTR1B/RALBP1
GOBP_POSITIVE_REGULATION_OF_EPITHELIAL_CELL_MIGRATION	GOBP_POSITIVE_REGULATION_OF_EPITHELIAL_CELL_MIGRATION	GOBP_POSITIVE_REGULATION_OF_EPITHELIAL_CELL_MIGRATION	28	0.40362408	2.081237667	0.001404041	0.017909908	0.013575864	1375	tags=79%, list=44%, signal=45%	FGFR1/HMGB1/ABL1/RAC1/ITGB1BP1/NFE2L2/TEK/CTSH/RAB11A/HAS2/NRP2/ITGA3/FGF1/HSPB1/NRP1/CLASP2/MAP2K3/ROCK2/ETS1/EPB41L4B/AKT1/CCBE1
GOBP_MICROTUBULE_BUNDLE_FORMATION	GOBP_MICROTUBULE_BUNDLE_FORMATION	GOBP_MICROTUBULE_BUNDLE_FORMATION	15	-0.507836576	-2.254536977	0.001427812	0.018117007	0.013732846	1122	tags=80%, list=36%, signal=52%	RP1L1/MAPRE1/MAP1S/SPAST/CDK5RAP2/IQCG/DNAI1/CLUAP1/DNAH17/DNAH8/ZMYND10/FOXJ1
GOMF_TRANSPORTER_ACTIVITY	GOMF_TRANSPORTER_ACTIVITY	GOMF_TRANSPORTER_ACTIVITY	221	0.191528387	1.850683008	0.001427035	0.018117007	0.013732846	1723	tags=68%, list=55%, signal=33%	LASP1/COX7A1/SLC44A1/OSBPL5/TOMM20/CYBRD1/RHBDF2/COX7B/VDAC1/COX8A/ITPR1/OSBPL1A/TMCO3/PLSCR1/SLC25A23/CLIC1/CLCC1/SLC25A5/TSPO/ATP1A2/TPCN1/SLC30A9/PLTP/CCDC51/SLC35D2/ABCB7/ANXA6/SLC29A1/SLC25A12/KCNK5/SLC12A9/KCNE3/GJA4/MFSD1/SIDT1/TMEM109/KCN

											B1/SLC25A37/OTOP3/SLC11A1/MPV17/CHRNB2/SLC2A13/SLC34A2/SLC9A8/SLC26A1/SLC16A9/GRIA4/BCL2/SLC31A1/STARD4/TRPM2/ATP2A3/SLC25A20/GRIK5/SLC29A3/COX15/APOD/SLC35B2/SEC61A1/SLC16A11/SLC6A17/ATP8B1/SLC14A2/SLC7A14/TMEM63A/SLC22A8/SLC29A2/OSBP/CLIC5/SLC26A5/TIMM17B/ABCB1/BAX/OTOP1/SLC22A11/KCNH4/GABRR1/SEC63/SLC24A3/CNNM2/ABCD4/OTOP2/ABCC10/KCNB2/KCNJ1/NIPA2/TAP1/CLDN4/SLC10A5/KCNAB2/SFXN3/SLC35A1/SLC16A12/ATP6V1G2/GRAMD1A/SLC25A36/SLC6A1/SLC35A4/AQP1/HTR1B/AQP6/RALBP1/KCNJ6/KCNH7/CHRNA2/SLC5A7/TRPV1/P2RX2/SLC22A7/HCN4/ABCD1/TAP2/SLC22A2/CLCN6/CACNA2D3/HCN3/ATP13A1/SLC32A1/ATP6V0A1/CACNA1B/KCNQ3/GJC1/SLC34A3/KCNS2/CACNA1E/SLC12A8/SLC25A13/SLC12A3/NDUFB7/CACNB3/SLC17A7/ATP1A3/ABCG2/ATP2A1/TRPV5/HTR3A/GJB1/SLC17A8/ATAD1/PCTP/SCN2B/SV2A/SLC23A3/SLC12A1/KCNMB3/SLC4A1/ATP7B/SLC16A7/SLC16A1
HP_ABNORMALITY_OF_HAND_JOINT_MOBILITY	HP_ABNORMALITY_OF_HAND_JOINT_MOBILITY	HP_ABNORMALITY_OF_HAND_JOINT_MOBILITY	11	0.588747552	2.115056581	0.001468435	0.018534661	0.014049432	854	tags=82%, list=27%, signal=60%	FBN1/MED12/COL3A1/COL6A3/PIGG/PRR12/PTDSS1/NFASC/PROKR2
GOBP_STEM_CELL_DIFFERENTIATION	GOBP_STEM_CELL_DIFFERENTIATION	GOBP_STEM_CELL_DIFFERENTIATION	45	0.337754691	2.0826266	0.001466574	0.018534661	0.014049432	818	tags=53%, list=26%, signal=40%	EFNB1/ABL1/SMO/KRT14/EIF2AK2/STAT3/PUM1/PTPRC/NFE2L2/HAND2/ITGB1/ANXA6/BMP4/PTN/NKX2-5/CITED2/SNAI2/FOXC1/BCL2/NRP2/YAP1/MSI2/ENG/EOMES
GOBP_POSITIVE_REGULATION_OF_STRESS_ACTIVATED_PROTEIN_KINASE_SIGNALING_CASCADE	GOBP_POSITIVE_REGULATION_OF_STRESS_ACTIVATED_PROTEIN_KINASE_SIGNALING_CASCADE	GOBP_POSITIVE_REGULATION_OF_STRESS_ACTIVATED_PROTEIN_KINASE_SIGNALING_CASCADE	27	0.413390257	2.112074495	0.001479453	0.01861308	0.014108874	503	tags=48%, list=16%, signal=41%	PYCARD/HMGB1/EIF2AK2/GADD45A/HAND2/LTBR/MID1/IGFBP6/HIPK2/MAP3K5/BMP4/CCL21/BMP2
HP_OSTEOLYSIS	HP_OSTEOLYSIS	HP_OSTEOLYSIS	25	0.42177664	2.088273686	0.001482389	0.01861308	0.014108874	216	tags=40%, list=7%,	CTSK/ANTXR2/FGFR1/SH3PXD2B/PDGFRB/SCARB2/ZMPSTE24/MAFB/PERP/

										signal=38%	COL3A1
HP_ABDOMINAL_PAIN	HP_ABDOMINAL_PAIN	HP_ABDOMINAL_PAIN	72	0.275501367	2.006445928	0.001501548	0.018804547	0.014254008	593	tags=38%, list=19%, signal=31%	GYPC/SCARB2/MED12/IFNGR1/EMP2/SMO/H19/HLA-DQB1/BAZ1B/APRT/STAT3/COL4A5/HLA-B/MYC/GNE/ARHGDIA/COL4A6/GTF2IRD2/NKX2-5/HPS1/TSC1/IRAK1/BMP2/ALAD/ELN/PPOX/MEFV
HP_BRACHYDACTYLY	HP_BRACHYDACTYLY	HP_BRACHYDACTYLY	71	0.288483063	2.091777547	0.001528286	0.019040221	0.01443265	1108	tags=55%, list=35%, signal=36%	FBN1/CTSK/ANTXR2/MAF/EFNB1/FGFR1/SH3PXD2B/PDGFRB/CDH11/IGF2/MED12/MAFB/PPP3CA/MBTPS1/FBXW11/MRAS/BMP4/NKX2-5/CITED2/MMP23B/ZFPM2/EPB41L1/BMP2/ZDHHC9/TRIO/LTBP1/DCPS/MAGEL2/PTDSS1/SLURP1/INPPL1/HEATR3/PTCH2/HUWE1/EHMT1/PRKAR1A/DDX3X/SMC1A/FAM20C
HP_ABNORMALITY_OF_UPPER_LIMB_JOINT	HP_ABNORMALITY_OF_UPPER_LIMB_JOINT	HP_ABNORMALITY_OF_UPPER_LIMB_JOINT	129	0.227104342	1.920887981	0.001525689	0.019040221	0.01443265	805	tags=40%, list=26%, signal=31%	FBN1/PLOD1/ANTXR2/MAF/SETBP1/EFNB1/FGFR1/CCDC8/SH3PXD2B/PDGFRB/OSGEP/TBC1D2B/MED12/TFE3/PQBP1/CTBP1/ZMPSTE24/KRT14/GLB1/MAFB/BAZ1B/COL6A3/GPKOW/MBTPS1/GNE/FBXW11/MRAS/GTF2IRD2/MMP23B/CBFB/TPM2/COLEC11/TOR1A/DHCR24/ZDHHC9/PDXK/CTCF/SLC34A2/ELN/LARS2/PTPN2/MMP14/SLC29A3/LGI4/LFNG/COL27A1/LAGE3/CDH3/SLC35B2/PTDSS1/EMD
HP_CAPILLARY_MALFORMATION	HP_CAPILLARY_MALFORMATION	HP_CAPILLARY_MALFORMATION	18	0.492500592	2.142207982	0.00155163	0.019151613	0.014517087	571	tags=61%, list=18%, signal=50%	SETBP1/FGFR1/IGF2/EIF2AK4/H19/CYP1B1/BAZ1B/GTF2IRD2/MYOC/TEK/ELN
GOBP_FOCAL_ADHESION_ASSEMBLY	GOBP_FOCAL_ADHESION_ASSEMBLY	GOBP_FOCAL_ADHESION_ASSEMBLY	29	0.394391807	2.076155378	0.001553156	0.019151613	0.014517087	1342	tags=76%, list=43%, signal=44%	CDH11/ABL1/TRIP6/RAC1/ITGB1BP1/MYOC/TSC1/TEK/BCL2/ARHGEF7/PHLDB2/MMP14/FAM107A/CORO2B/APOD/NRP1/CLASP2/ROCK2/ACTN2/DLC1/CTTN/THY1
GOBP_REGULATION_OF_HORMONE_LEVELS	GOBP_REGULATION_OF_HORMONE_LEVELS	GOBP_REGULATION_OF_HORMONE_LEVELS	99	0.251083116	1.984398716	0.001547615	0.019151613	0.014517087	1326	tags=58%, list=42%, signal=34%	NFKB1/CTSK/ALDH1A3/AKR1C1/DAB2/ZMPSTE24/CYP1B1/RAB1A/ITPR1/NADK/PPP3CA/TSPO/INHBA/RBP1/BACE2/ALOX5/KCNB1/GHRH/BMP2/CYP2W1/CCL5/BCAT2/CYP11A1/RPH3AL/PNPLA4/AANAT/SRI/FZD4/CYP17A1/UGT2A3/LYN/BCHE/NR3C1/CYP2S1/ADM/

											ADH1C/BAD/OSBP/UCN3/STUB1/HSD17B8/INS/TM7SF3/DUOX2/PRKAR1A/CPA3/PSMD9/ADIPOQ/GHSR/AQP1/EGR1/NDST2/HSD17B1/GPR119/JAGN1/PCSK5/FFAR2
GOBP_REGULATION_OF_INNATE_IMMUNE_RESPONSE	GOBP_REGULATION_OF_INNATE_IMMUNE_RESPONSE	GOBP_REGULATION_OF_INNATE_IMMUNE_RESPONSE	79	0.262707744	1.941841347	0.001550619	0.019151613	0.014517087	971	tags=49%, list=31%, signal=35%	LAG3/PHB2/LSM14A/PYCARD/PQBP1/HMGB1/TYROBP/HAVCR2/EIF2AK2/MAPKAPK3/PLSCR1/PUM1/HLA-B/HLA-E/HLA-F/NFE2L2/HLA-A/FLOT2/NMI/COLEC11/RNF185/CYLD/IRAK1/CCL5/ISG15/MEFV/PTPN2/LYN/BRCC3/RELA/SIRT2/KIR2DL4/TRIM41/ZNFX1/NFKBIL1/NR1H2/INS/OTOP1/TRAFD1
HP_CRANIOSYNOSTOSIS	HP_CRANIOSYNOSTOSIS	HP_CRANIOSYNOSTOSIS	46	0.3308842	2.061314831	0.001576376	0.01938822	0.014696436	356	tags=35%, list=11%, signal=31%	RNF113A/FBN1/CTSK/MAF/EFNB1/NFIA/FGFR1/CDH11/MED12/SMO/KRT14/PPP3CA/STAT3/AHDC1/MID1/BMP4
GOCC_SITE_OF_DNA_DAMAGE	GOCC_SITE_OF_DNA_DAMAGE	GOCC_SITE_OF_DNA_DAMAGE	24	-0.424115694	-2.301934269	0.001588976	0.019448519	0.014742144	841	tags=62%, list=27%, signal=46%	VCP/NHEJ1/PARP1/CBX1/TIMELESS/RAD50/TP53BP1/ARPC2/MAD2L2/CHD1L/RNF138/SMC6/TRAIP/RAD51/SMCHD1
GOBP_MUSCLE_ORGAN_MORPHOGENESIS	GOBP_MUSCLE_ORGAN_MORPHOGENESIS	GOBP_MUSCLE_ORGAN_MORPHOGENESIS	14	0.526549668	2.05783989	0.001593412	0.019448519	0.014742144	909	tags=71%, list=29%, signal=51%	COL3A1/HEG1/FKBP1A/BMP4/NKX2-5/ZFPM2/BMP2/FOXC1/ENG/PKP2
GOMF_CALCIUM_ION_BINDING	GOMF_CALCIUM_ION_BINDING	GOMF_CALCIUM_ION_BINDING	116	0.23287527	1.925736599	0.001591533	0.019448519	0.014742144	1624	tags=67%, list=52%, signal=34%	FBN1/PCDHGA8/F10/CDH11/MGP/IQGAP1/CALU/AIF1/ITPR1/CD248/PPP3CA/PLSCR1/SLC25A23/PAM/LOXL2/STAB1/HEG1/S100A4/ANXA7/SULF1/ANXA6/SLC25A12/ANXA4/EHD4/COLEC11/PCDHB16/DNASE1L2/CSN2/PCDHB14/SRI/SVEP1/FER1L4/SYT12/TRPM2/PLS3/AOC3/FBN3/LTBP1/PLA2G4C/CDH3/PCDHB4/CALM3/DUOX2/CDH4/S100B/CABP5/FAM20C/PRRG3/CDH9/VWCE/CGREF1/ACTN2/LRP1B/S100Z/REG4/UMODL1/BNIP2/CLSTN3/SNCB/NOTCH4/S100A12/CCBE1/SPTAN1/S100A14/PLA2G1B/CACNA1B/CACNA1E/SLC25A13/S100A5/PCP4/ATP2A1/ITSN2/TNNC2/RAB11FIP3/MEGF6/CLSTN1/PLA2G4D/RHOT1
HP_TELECANTHUS	HP_TELECANTHUS	HP_TELECANTHUS	31	0.380646638	2.033271107	0.00160432	0.019532084	0.014805487	539	tags=48%, list=17%,	FBN1/EFNB1/FGFR1/CDH11/TBC1D2B/PQBP1/ZMPSTE24/GPRASP2/MAFB/CO

										signal=41%	L3A1/MID1/SNAI2/COLEC11/PURA/FOXC1
HP_HERNIA	HP_HERNIA	HP_HERNIA	127	0.23435297	1.975974885	0.001609387	0.019544292	0.014814741	800	tags=41%, list=25%, signal=32%	RNF113A/FBN1/PLOD1/MAF/SETBP1/SGSH/EFNB1/NFIA/FGFR1/SH3PXD2B/OSGEP/CDH11/IGF2/MED12/TFE3/CTBP1/ABL1/COX7B/H19/GLB1/MAFB/TRIM8/ATP1A2/COL3A1/BAZ1B/RAC1/MBTPS1/PIGW/GNE/FBXW11/MID1/GTF2IRD2/NKX2-5/PLAGL1/ZFPM2/COLEC11/TOR1A/ZBTB7A/CTCF/ELN/PLAG1/MMP14/CBS/PIGG/SLC29A3/LTBP1/LFNG/CDKN1C/LAGE3/PPP2R1A/PRR12/PTDSS1
GOBP_XENOBIOTIC_METABOLIC_PROCESS	GOBP_XENOBIOTIC_METABOLIC_PROCESS	GOBP_XENOBIOTIC_METABOLIC_PROCESS	32	0.383480974	2.084316705	0.001616116	0.019576583	0.014839217	1520	tags=81%, list=48%, signal=43%	AKR1C1/CBR1/CBR3/GSTO1/CYP1B1/CYP2U1/SLC29A1/NQO1/FMO1/ACAA1/BPHL/CYP2W1/BCHE/SLC29A3/CYP2S1/NAT2/ACSL1/ABCB1/FMO5/ADA/SLC22A7/S100A12/CYP2C18/CYP2A13/UGT1A6/CAD
GOBP_POSITIVE_REGULATION_OF_SUBSTRATE_ADHESION_DEPENDENT_CELL_SPREADING	GOBP_POSITIVE_REGULATION_OF_SUBSTRATE_ADHESION_DEPENDENT_CELL_SPREADING	GOBP_POSITIVE_REGULATION_OF_SUBSTRATE_ADHESION_DEPENDENT_CELL_SPREADING	14	0.525115681	2.052235643	0.001624963	0.019634293	0.014882962	625	tags=64%, list=20%, signal=52%	DAB2/ABL1/RAC1/ILK/MYOC/TRIOBP/MYADM/HAS2/ARHGEF7
HP_CORNEAL_OPACITY	HP_CORNEAL_OPACITY	HP_CORNEAL_OPACITY	55	0.321585135	2.118747661	0.001629473	0.019639434	0.014886859	764	tags=47%, list=24%, signal=36%	MAF/SUMF1/FGFR1/PDGFRB/CTBP1/ZMPSTE24/TGFBI/COX7B/KRT14/CYP1B1/GLB1/MAFB/BAZ1B/BMP4/GTF2IRD2/FOXC1/MPV17/ELN/AIRE/PTPN2/MMP14/FZD4/PIGG/COL4A1/SLC29A3/YAP1
GOBP_ANTIGEN_PROCESSING_AND_PRESENTATION_OF_PEPTIDE_OR_POLYSACCHARIDE_ANTIGEN_VIA_MHC_CLASS_II	GOBP_ANTIGEN_PROCESSING_AND_PRESENTATION_OF_PEPTIDE_OR_POLYSACCHARIDE_ANTIGEN_VIA_MHC_CLASS_II	GOBP_ANTIGEN_PROCESSING_AND_PRESENTATION_OF_PEPTIDE_OR_POLYSACCHARIDE_ANTIGEN_VIA_MHC_CLASS_II	11	0.584239274	2.098860739	0.001639669	0.019663762	0.0149053	701	tags=73%, list=22%, signal=57%	PYCARD/HLA-DMB/HLA-DRB3/HLA-DQB1/HLA-DRB5/HLA-DMA/HLA-DOB/FCER1G
GOBP_MORPHOGENESIS_OF_AN_EPITHELIUM	GOBP_MORPHOGENESIS_OF_AN_EPITHELIUM	GOBP_MORPHOGENESIS_OF_AN_EPITHELIUM	102	0.246350945	1.962851321	0.001639543	0.019663762	0.0149053	923	tags=45%, list=29%, signal=33%	MSN/ALDH1A3/RALA/PHB2/DAB2/MED12/ABL1/SMO/GLI2/PLXNA1/RAC1/ILK/MYC/HAND2/ITGB1/SULF1/BMP4/NKX2-5/MTHFD1/PLEKHA4/CITED2/SN

											AI2/TSC1/TOR1A/BMP2/CTSH/BCL2/AIRE/PODXL/PHLDB2/RHOC/MMP14/COL4A1/NR3C1/YAP1/ITGA5/FGF1/LRG1/ENG/FRS2/ADM/WNT11/MAGED1/KRT27/NRP1/CLASP2
GOBP_REGULATION_OF_MONONUCLEAR_CELL_MIGRATION	GOBP_REGULATION_OF_MONONUCLEAR_CELL_MIGRATION	GOBP_REGULATION_OF_MONONUCLEAR_CELL_MIGRATION	23	0.424693311	2.018954963	0.001645795	0.019688125	0.014923767	1412	tags=83%, list=45%, signal=46%	MSN/PYCARD/HMGB1/ABL1/AIF1/C3AR1/CCL21/CCL5/AIRE/LYN/CXCL13/APOD/AGER/CCR1/SPN/CX3CR1/AKT1/OXSR1/S100A14
GOMF_MICROTUBULE_BINDING	GOMF_MICROTUBULE_BINDING	GOMF_MICROTUBULE_BINDING	53	-0.28882583	-2.072658243	0.001651171	0.01970343	0.014935368	939	tags=55%, list=30%, signal=39%	TUBGCP2/MAPRE1/CEP290/MAP1S/REEP4/SPAST/NUSAP1/KRIT1/HOOK2/CDK5RAP2/KIF22/KIF4A/CEP350/SSNA1/MAP6D1/KIFC3/NDE1/KIF23/MAST2/CENPE/KIF3A/KATNB1/POLB/KATNA1/SPAG5/RAE1/STRBP/PAFAH1B1/SPAG8
GOBP_NEGATIVE_REGULATION_OF_CELL_ADHESION	GOBP_NEGATIVE_REGULATION_OF_CELL_ADHESION	GOBP_NEGATIVE_REGULATION_OF_CELL_ADHESION	65	0.29636751	2.071979078	0.001665775	0.0198285	0.015030173	1254	tags=65%, list=40%, signal=40%	LAG3/CEBPB/HMGB1/ABL1/TGFBI/HAVCR2/CYP1B1/PLXNA1/ITGB1BP1/PTPRC/FXYD5/BMP4/MYOC/CBFB/SNAI2/CCL21/ADAM15/MYADM/BMP2/FGL2/BTN2A2/PODXL/PHLDB2/PTPN2/MMP14/FZD4/SCGB1A1/FAM107A/CORO2B/APOD/SERPINE2/CLASP2/SPN/PRKAR1A/PAG1/CASK/SOCS1/TNFRSF21/SMAD7/ADIPOQ/ZNF703/DLC1
GOBP_SEQUESTERING_OF_CALCIUM_ION	GOBP_SEQUESTERING_OF_CALCIUM_ION	GOBP_SEQUESTERING_OF_CALCIUM_ION	32	0.381169386	2.071752633	0.001706737	0.020265923	0.015361743	966	tags=59%, list=31%, signal=42%	ABL1/GSTO1/ITPR1/SLC25A23/ATP1A2/PTPRC/TPCN1/FKBP1A/ANXA6/CCL21/SRI/IBTK/LYN/TRPM2/F2RL3/CCR5/CALM3/LIME1/BAX
GOCC_LATE_ENDOSOME_MEMBRANE	GOCC_LATE_ENDOSOME_MEMBRANE	GOCC_LATE_ENDOSOME_MEMBRANE	39	0.340808518	1.981521273	0.001731268	0.020506568	0.015544154	614	tags=46%, list=19%, signal=38%	WDR81/IFITM3/SCARB2/HLA-DMB/ZMPSTE24/HLA-DRB3/HLA-DQB1/HLA-DRB5/HLA-DMA/ANXA6/CLN3/HLA-DOB/SLC11A1/SLC9A8/MITD1/ABHD6/SLC31A1/VPS13C
GOBP_RENAL_SYSTEM_PROCESS	GOBP_RENAL_SYSTEM_PROCESS	GOBP_RENAL_SYSTEM_PROCESS	34	0.36219396	2.023584873	0.001735849	0.020510312	0.015546992	1589	tags=82%, list=50%, signal=41%	EMP2/PPP3CA/SLC25A23/CD34/SULF1/BMP4/WFS1/CLN3/CHRNB2/BCL2/HAS2/CORO2B/ITGA3/ADM/RRM2B/CLDN4/ADIPOQ/AQP1/AQP6/PCSK5/TRPV1/OXSR1/AMN/BCR/SLC12A3/ABCG2/MCAM/TRPV5
GOBP_CELL_FATE_COMMITMENT	GOBP_CELL_FATE_COMMITMENT	GOBP_CELL_FATE_COMMITMENT	43	0.335911339	2.025591566	0.001749764	0.020624058	0.015633212	1491	tags=74%, list=47%,	NFIA/SMO/ETS2/GLI2/STAT3/ISL2/ITGB1/BMP4/NKX2-5/BMP2/EBF2/BCL2/ID

										signal=40%	2/EOMES/WNT11/NRP1/SPN/PTCH2/TM2D3/FGF13/MYOG/GCM1/WNT2B/TNXB/LMO4/ASCL1/TBX6/NOTCH4/WNT3A/PRRX1/MYOD1/WNT1
GOBP_SECONDARY_METABOLIC_PROCESS	GOBP_SECONDARY_METABOLIC_PROCESS	GOBP_SECONDARY_METABOLIC_PROCESS	12	0.563462426	2.067343284	0.001757274	0.020659652	0.015660193	861	tags=75%, list=27%, signal=55%	AKR1A1/AKR1C1/CYP1B1/NFE2L2/FMO1/CYP2W1/BCL2/CDH3/AKR7A2
GOMF_GLYCOSAMINOGLYCAN_BINDING	GOMF_GLYCOSAMINOGLYCAN_BINDING	GOMF_GLYCOSAMINOGLYCAN_BINDING	39	0.339694601	1.975044763	0.001761376	0.020659652	0.015660193	919	tags=56%, list=29%, signal=40%	FBN1/FGFR1/VEGFB/LAYN/STAB1/PCOLCE/PTPRC/ADAMTS5/SULF1/ANXA6/BMP4/PTN/PRELP/NAV2/EXTL2/NRP2/CXCL13/FGF1/IMPG2/ENG/SERPINE2/NRP1
GOBP_SPLICEOSOMAL_COMPLEX_ASSEMBLY	GOBP_SPLICEOSOMAL_COMPLEX_ASSEMBLY	GOBP_SPLICEOSOMAL_COMPLEX_ASSEMBLY	11	-0.580762637	-2.193557934	0.001769073	0.020699448	0.015690358	1029	tags=91%, list=33%, signal=61%	LUC7L2/LUC7L/PRPF6/SF3B4/SF3B2/SF3A1/GEMIN6/SRPK1/SETX/BUD13
GOBP_GLOMERULUS_DEVELOPMENT	GOBP_GLOMERULUS_DEVELOPMENT	GOBP_GLOMERULUS_DEVELOPMENT	19	0.469293098	2.073531702	0.001788848	0.020880026	0.015827239	606	tags=58%, list=19%, signal=47%	PDGFRB/IQGAP1/PPP3CA/CD34/SULF1/BMP4/TEK/FOXC1/MPV17/BCL2/PODXL
GOBP_PORPHYRIN_CONTAINING_COMPOUND_METABOLIC_PROCESS	GOBP_PORPHYRIN_CONTAINING_COMPOUND_METABOLIC_PROCESS	GOBP_PORPHYRIN_CONTAINING_COMPOUND_METABOLIC_PROCESS	16	0.516234739	2.155080713	0.001798872	0.020895598	0.015839042	759	tags=69%, list=24%, signal=52%	RSAD1/EIF2AK1/TSPO/ABCB7/TMEM14A/NFE2L1/FECH/ALAD/PPOX/BLVRA/COX15
GOBP_REGULATION_OF_APOPTOTIC_SIGNALING_PATHWAY	GOBP_REGULATION_OF_APOPTOTIC_SIGNALING_PATHWAY	GOBP_REGULATION_OF_APOPTOTIC_SIGNALING_PATHWAY	88	0.257666683	1.968608519	0.001798795	0.020895598	0.015839042	966	tags=48%, list=31%, signal=34%	CTSK/TNFSF10/FGFR1/PYCARD/XBP1/TRAF1/SLC25A5/INHBA/MYC/PTPRC/NFE2L2/LTBR/BMP4/ATF4/TMEM14A/SRPX/MNT/SNAI2/WFS1/CYLD/BCL2L1/CTSH/BCL2L2/BCL2/PTPN2/CTSC/RELA/BBC3/IER3/YAP1/PPP2R1A/IFI6/HSPB1/GCLC/MAGED1/RRM2B/MDM2/NRP1/BAD/PPIF/INS/BAX
GOMF_CELL_ADHESION_MOLECULE_BINDING	GOMF_CELL_ADHESION_MOLECULE_BINDING	GOMF_CELL_ADHESION_MOLECULE_BINDING	112	0.241229262	1.964262463	0.001809764	0.020971459	0.015896545	909	tags=45%, list=29%, signal=33%	FBN1/LASP1/MSN/PARVA/CDH11/IGF2/IQGAP1/HMGB1/EMP2/TGFBI/RAB1A/CLIC1/COL3A1/ITGB1BP1/FXYD5/ADAMTS5/ITGB1/VAPA/ANXA7/PTN/ADD1/EHD4/ITGA9/CD177/ADAM15/ITGB7/VWF/PRDX1/CCS/ROBO3/ISG15/PHLDB2/PTPN2/MMP14/LYN/LIMA1/CLINT1/LRRFIP1/ITGA3/CDH3/ITGA5/FGF1/EMD/YWHAB/NFASC/LDHA/AGER/SLC14A2/CHMP2B/PKP2
GOBP_REGULATI	GOBP_REGULATI	GOBP_REGULATION_O	15	0.518747206	2.077700563	0.00184267	0.02130145	0.01614668	930	tags=73%,	FBN1/CEBPB/TFE3/TYROBP/MAFB/PP

ON_OF_OSTEOCLAST_DIFFERENTIATION	ON_OF_OSTEOCLAST_DIFFERENTIATION	F_OSTEOCLAST_DIFFERENTIATION				1	5	5		list=29%, signal=52%	P3CA/PIAS3/TCTA/IL17A/KLF10/CCR1
GOBP_CHEMICAL_HOMEOSTASIS	GOBP_CHEMICAL_HOMEOSTASIS	GOBP_CHEMICAL_HOMEOSTASIS	201	0.191472641	1.813313219	0.001887403	0.021766239	0.016498996	1810	tags=70%, list=57%, signal=32%	CCDC115/AKR1C1/CYBRD1/XBP1/ABL1/GSTO1/EIF2AK1/ITPR1/NADK/PPP3CA/SLC25A23/TSPO/ATP1A2/LIPA/STAT3/MYC/PTPRC/TPCN1/CEBPA/ACO1/SLC30A9/SCARA5/FKBP1A/RBP1/ABCB7/ANXA6/SLC29A1/ATF4/BACE2/ALOX5/SLC12A9/KCNE3/CCL21/WFS1/MT1B/FECH/CLN3/KCNB1/MT1E/CCL5/SLC11A1/SLC34A2/CTSH/SLC9A8/OR10J5/ANGPTL4/RPH3AL/PNPLA4/BCL2/SLC31A1/HAS2/MT1H/SRI/IBTK/PTPN2/LYN/LIMA1/TRPM2/ATP2A3/F2RL3/GRM1/CCR5/FOSL2/ERN1/GCLC/AGER/GPR21/BAD/ACOX1/CCR1/NR1H2/OSBP/UBTF/RRAGA/CALM3/INS/LIME1/BAX/PDK2/CREG1/SLC24A3/CNNM2/ADIPOR2/DDX3X/GPR3/CNBP/CCL8/ADIPOQ/DIAPH1/VAPB/SYPL2/AQP1/ADIPOR1/HTR1B/AQP6/NUBP1/JAGN1/XCR1/FFAR2/TRPV1/FXN/THY1/ABCD1/AKT1/MT3/CAV3/S100A14/CA2/ATG5/FIS1/ATP13A1/TMEM106B/ATP6V0A1/SLC34A3/CLN8/SLC12A8/ATP1B1/SLC12A3/SLC17A7/ATP1A3/ATP2A1/TRPV5/BDKRB1/SLC17A8/SV2A/GPR12/SLC12A1/GLS/SLC4A1/ATP7B/SLC16A1/STIM1/HKDC1/ATP2B3/PNPLA2/GP1BB/GATA4/ENPP1/RAB20/UMOD/HDAC9
GOBP_NEGATIVE_REGULATION_OF_CHROMOSOME_ORGANIZATION	GOBP_NEGATIVE_REGULATION_OF_CHROMOSOME_ORGANIZATION	GOBP_NEGATIVE_REGULATION_OF_CHROMOSOME_ORGANIZATION	22	-0.42554609	-2.192963402	0.001936744	0.022281826	0.016889815	886	tags=68%, list=28%, signal=49%	LCMT1/PARP1/RAD50/ZW10/POT1/CDK5RAP2/MAD2L1BP/PINX1/ESPL1/MAD2L2/DYNC1LI1/TRIP13/BUB1B/TPR/CDCA8
GOBP_NEGATIVE_REGULATION_OF_NUCLEAR_DIVISION	GOBP_NEGATIVE_REGULATION_OF_NUCLEAR_DIVISION	GOBP_NEGATIVE_REGULATION_OF_NUCLEAR_DIVISION	15	-0.497054715	-2.206670978	0.001943104	0.022289569	0.016895684	700	tags=67%, list=22%, signal=52%	ZW10/CDK5RAP2/MAD2L1BP/MAD2L2/DYNC1LI1/TRIP13/BUB1B/TPR/CDCA8/AURKAIP1
GOBP_ERK1_AND_ERK2_CASCADE	GOBP_ERK1_AND_ERK2_CASCADE	GOBP_ERK1_AND_ERK2_CASCADE	61	0.297240445	2.044907524	0.001946687	0.022289569	0.016895684	1402	tags=66%, list=44%, signal=37%	SHC1/PDGFRB/PHB2/DAB2/PYCARD/HMGB1/ABL1/HAVCR2/ITGB1BP1/INHBA/MYC/PTPRC/HAND2/BMP4/CCL21/B

											MP2/TEK/CCL5/CTSH/BTN2A2/PTPN2/LYN/FGF1/FRS2/AGER/NRP1/CCR1/CD4/FGF19/FSHR/ARRB2/CCL8/IQGAP3/ADIPOQ/RGS14/CSK/MFHAS1/CCL22/PDGFRA/MT3
GOBP_NEGATIVE_REGULATION_OF_CHROMOSOME_SEGREGATION	GOBP_NEGATIVE_REGULATION_OF_CHROMOSOME_SEGREGATION	GOBP_NEGATIVE_REGULATION_OF_CHROMOSOME_SEGREGATION	13	-0.544440314	-2.202975967	0.001984941	0.022668861	0.017183191	700	tags=77%, list=22%, signal=60%	LCMT1/ZW10/CDK5RAP2/MAD2L1BP/MAD2L2/DYNC1LI1/TRIP13/BUB1B/TPR/CDCA8
GOCC_ENDOSOME_MEMBRANE	GOCC_ENDOSOME_MEMBRANE	GOCC_ENDOSOME_MEMBRANE	111	0.237606078	1.923428623	0.001989241	0.022668861	0.017183191	777	tags=41%, list=25%, signal=32%	ANTXR2/WDR81/IFITM3/SCARB2/HLA-DMB/ZMPSTE24/HLA-DRB3/HLA-DQB1/RAC1/HLA-B/HLA-E/HLA-F/HLA-DRB5/TPCN1/HPS6/RHOU/ITGB1/STEAP3/HLA-DMA/HLA-A/HLA-C/ANXA6/PRAF2/EHD4/IGF2R/WIPI1/IRAK1/CLN3/SBF2/AP1G2/HLA-DOB/SLC11A1/SLC9A8/MITD1/ABHD6/RAB11A/LLGL1/SLC31A1/VPS13C/SCAMP3/AP2M1/DNAJC13/SLC29A3/GPR135/KIR2DL4
GOCC_BASEMENT_MEMBRANE	GOCC_BASEMENT_MEMBRANE	GOCC_BASEMENT_MEMBRANE	19	0.46477565	2.053571744	0.00201047	0.022802712	0.017284651	1532	tags=89%, list=49%, signal=46%	FBN1/COL4A2/TGFBI/LAMC3/COL4A5/LOXL2/COL4A6/COL15A1/COL4A1/SPN/MMRN2/CASK/TIMP1/ANG/ENTPD2/VWA1/COL8A2
GOMF_ALCOHOL_BINDING	GOMF_ALCOHOL_BINDING	GOMF_ALCOHOL_BINDING	20	0.447192244	2.017751409	0.002007021	0.022802712	0.017284651	874	tags=65%, list=28%, signal=47%	OSBPL5/SCARB2/VDAC1/ITPR1/OSBPL1A/TSPO/RBP1/ANXA6/SIDT1/CYP2W1/STARD4/APOD/SCP2
GOBP_MAMMARY_GLAND_DEVELOPMENT	GOBP_MAMMARY_GLAND_DEVELOPMENT	GOBP_MAMMARY_GLAND_DEVELOPMENT	30	0.377986631	2.006652324	0.002025409	0.022884612	0.017346732	1520	tags=80%, list=48%, signal=42%	NFKB1/PHB2/CEBPB/XBP1/SMO/GLI2/SOCS2/SLC29A1/TGFA/BMP4/CSN2/ID2/NR3C1/APLN/BAX/OAS2/IQGAP3/ZNF703/NOTCH4/AKT1/CAV3/WNT3A/LBH/CAD
GOCC_SECRETORY_GRANULE	GOCC_SECRETORY_GRANULE	GOCC_SECRETORY_GRANULE	182	0.195054461	1.815629088	0.002027208	0.022884612	0.017346732	714	tags=35%, list=23%, signal=29%	FUCA2/NFKB1/TXNDC5/PYCARD/IGF2/IQGAP1/LGALS3BP/HMGB1/TYROBP/CAP1/VEGFB/ITPR1/GLB1/CAT/PAM/IMPDH2/APRT/RAC1/HLA-B/PYGB/PTPRC/MANBA/HP/C3AR1/VAPA/CYFIP1/ANXA7/FLOT2/HLA-C/DERA/ALOX5/IGF2R/PTGES2/ACAA1/WFS1/GDI2/CD177/ADAM15/TUBA8/VWF/TOR1A/PDXK/FGL2/ALAD/SLC11A1/GMFG/CTSH/SLC9A8/TFF3/PHACTR2/VPS13C/CPNE3/SRI/CTSC/DENND4C/DNAJC5/PGM2/T

											RPM2/KLK10/SCGB1A1/PLAU/FCER1G/TRAPPC1/DNAJC13
GOBP_POSITIVE_REGULATION_OF_NON_CANONICAL_NF_KAPPAB_SIGNAL_TRANSDUCTION	GOBP_POSITIVE_REGULATION_OF_NON_CANONICAL_NF_KAPPAB_SIGNAL_TRANSDUCTION	GOBP_POSITIVE_REGULATION_OF_NON_CANONICAL_NF_KAPPAB_SIGNAL_TRANSDUCTION	17	0.482973168	2.071522162	0.00203876	0.02296112	0.017404725	1183	tags=76%, list=38%, signal=48%	PHB2/PYCARD/HAVCR2/EIF2AK2/TRIP6/ILK/NMI/IRAK1/EDA/RELA/AGER/DDX3X/TRIM44
GOBP_REGULATION_OF_CHROMOSOME_SEGREGATION	GOBP_REGULATION_OF_CHROMOSOME_SEGREGATION	GOBP_REGULATION_OF_CHROMOSOME_SEGREGATION	30	-0.36593719	-2.136902575	0.002043752	0.022963554	0.017406571	920	tags=63%, list=29%, signal=45%	SMC4/LCMT1/CDC16/UBE2C/ZW10/SMARCC1/CDK5RAP2/MAD2L1BP/BRD7/ESPL1/MAD2L2/DYNC1LI1/SMC6/TRIP13/CENPE/BUB1B/CSNK2A2/TPR/CDCA8
GOBP_MYELOID_CELL_HOMEOSTASIS	GOBP_MYELOID_CELL_HOMEOSTASIS	GOBP_MYELOID_CELL_HOMEOSTASIS	38	0.358187216	2.071721752	0.002078524	0.02329982	0.017661463	674	tags=47%, list=21%, signal=38%	HMGB1/HCLS1/MAFB/SLC25A5/LIPA/STAT3/INHBA/HIPK2/BMP4/MTHFD1/CITED2/FECH/PRDX1/ZBTB7A/ISG15/ID2/PTPN2/LYN
HP_ABNORMALITY_OF_CHROMOSOME_STABILITY	HP_ABNORMALITY_OF_CHROMOSOME_STABILITY	HP_ABNORMALITY_OF_CHROMOSOME_STABILITY	10	-0.571976561	-2.115251729	0.002088506	0.023357271	0.017705011	728	tags=80%, list=23%, signal=62%	RAD50/FANCC/TOP3A/MAD2L2/BLM/UBE2T/RAD51/NF2
GOCC_NUCLEAR_PERIPHERY	GOCC_NUCLEAR_PERIPHERY	GOCC_NUCLEAR_PERIPHERY	39	-0.314145431	-2.050035256	0.002103942	0.023475304	0.017794481	686	tags=46%, list=22%, signal=37%	SMARCC1/AKAP8L/KIF4A/ATXN3/NUP93/SMC3/BRD7/PRPF40A/PCNA/EBNA1BP2/BLM/PAXIP1/RUVBL1/NARF/TPR/PSMA6/SRPK1/AHCTF1
GOBP_RESPONSE_TO_NITROGEN_COMPOUND	GOBP_RESPONSE_TO_NITROGEN_COMPOUND	GOBP_RESPONSE_TO_NITROGEN_COMPOUND	227	0.187347511	1.833904525	0.002115647	0.02355127	0.017852064	1428	tags=56%, list=45%, signal=33%	SHC1/FBN1/NFKB1/TYK2/CTSK/CASP4/UBQLN2/TOMM20/TNFSF10/RGS10/IGF2/CEBPB/XBP1/PQBP1/ABL1/TMEM129/CYP1B1/ITPR1/CAT/PPP3CA/SOCS2/BTG2/TSPO/FOLR2/COL3A1/LIPA/STAT3/INHBA/HM13/NFE2L2/ITGB1/CYFIP1/COL4A6/MAP3K5/NQO1/WFS1/RNF185/TSC1/MAS1L/CDK5/BCL2L1/KCNB1/TOR1A/PALM/TEK/ALAD/CHRNB2/CYP11A1/BCL2L2/BCL2/DERL1/HRH3/AANAT/VPS13C/RAPGEF1/PTPN2/DENND4C/CBS/LYN/TRPM2/EIF2B1/FCER1G/RELA/COL4A1/BCHE/NNMT/SIRT2/ANKZF1/GNAI1/FOSL2/ADM/TRIM41/GCLC/AGER/RRM2B/GPR21/LTA4H/MDM2/RNF5/KLF2/CRHR2/FAM8A1/DDI2/INPPL1/RRAGA/STUB1/INS/OTOP1/PDK2/DNAJC10/GRB7/DUOX2/PRKAR1A/RO

											CK2/GNB1/ARRB2/SOCS1/PITX3/ADIPOQ/DIAPH1/ACTN2/TMUB1/ATF2/GHSR/SLC6A1/AQP1/EGR1/ASCL1/ADIPOR1/CSK/HTR1B/ADA/TOP2B/UMODL1/CHRNA2/TIMP1/TRPV1/P2RX2/HCN4/CSHL1/PDGFRA/AKT1/SH2B2/CAV3/CA2/CDK2/HCN3
GOBP_RESPONSE_TO_CYTOKINE	GOBP_RESPONSE_TO_CYTOKINE	GOBP_RESPONSE_TO_CYTOKINE	184	0.196155519	1.835074314	0.002137817	0.023743103	0.017997476	1474	tags=59%, list=47%, signal=34%	RNF113A/NFKB1/TYK2/CTSK/CASP4/CDK4/IFITM3/LSM14A/PYCARD/XBP1/IFNGR1/IFITM1/HCLS1/AIF1/CYP1B1/EIF2AK2/TRAF1/MAPKAPK3/PLSCR1/SLC25A5/IMPDH2/COL3A1/STAT3/INHBA/ILK/LSP1/PTPRC/CEBPA/NFE2L2/MRAS/MAP3K5/CCL21/RNF185/IL15/CYLD/CCDC3/PIAS3/IRAK1/BCL2L1/CCL5/ALAD/FOXC1/SLC11A1/BCAT2/ISG15/BCL2/MEFV/AANAT/HAS2/NRP2/PTPN2/FZD4/LYN/KMO/SCGB1A1/FCER1G/RELA/TRAF5/CXCL13/YAP1/CCR5/FOSL2/IL17A/TRIM41/GCLC/CXCR3/KLF2/BAD/CCR1/NR1H2/RRAGA/ACSL1/CD4/OTOP1/OAS2/DUOX2/ABCD4/ULK1/ADIPOR2/TNFSF13B/MYOG/SOCS1/TNFRSF21/CCL8/SMAD7/ADIPOQ/TRIM44/UGCG/CX3CR1/EGR1/ADIPOR1/EPOR/JAGN1/TIMP1/XCR1/TRPV1/CCL22/SELPLG/TNFRSF4/AKT1/SRM/OXSR1/SH2B2/MT3/RARG/IL9R/MYOD1/OTUD4/PELI3
GOMF_AMIDE_BINDING	GOMF_AMIDE_BINDING	GOMF_AMIDE_BINDING	90	0.250282043	1.910567357	0.002154726	0.023875757	0.018098028	1640	tags=71%, list=52%, signal=35%	TOMM20/PHB2/HLA-DMB/VDAC1/HLA-DRB3/GPRASP2/HLA-DQB1/PPIC/PPP3CA/FOLR2/HLA-B/HLA-E/INHBA/HLA-F/HLA-DRB5/PLTP/FKBP1A/HLA-DMA/HLA-A/HLA-C/CLTB/RAMP2/DHCR24/HLA-DOB/GRIA4/FZD4/AP2M1/RELA/MAML1/BCHE/SEC61A1/GCLC/AGER/SCP2/CRHR2/PPIF/ITGAM/CD1A/PFDN1/TAP1/MRGPRX2/APBB2/GHSR/CD1B/CRIP1/MCCC1/CLSTN3/PCSK5/NPEPPS/ANG/SSTR3/FKBP5/TAP2/NPBWR2/GLP1R/CACNA1B/CLN8/MICA/ATP1A3/CD209/NQO2/CLSTN1/BDKRB1/ITM2

											C
GOBP_BEHAVIOR	GOBP_BEHAVIOR	GOBP_BEHAVIOR	135	0.220665349	1.897689187	0.002165273	0.023937464	0.018144803	1521	tags=62%, list=48%, signal=34%	ALDH1A3/SGSH/ZMPSTE24/ABL1/EIF2AK4/VDAC1/BTG2/TMOD1/TSPO/ATP1A2/APRT/STAT3/PUM1/MBD2/HAND2/EIF4G1/ITGB1/HIPK2/PTN/NAV2/ABHD12/WFS1/TSC1/CDK5/CLN3/GHRH/CHRNB2/BCL2/ID2/SEZ6/ARF4/FZD4/GPR83/MAFG/PI3/CLDN5/BCHE/NR3C1/LGI4/GRM1/ITGA3/ITGA5/APLN/NTF3/AGER/MTNR1A/SLURP1/SERPINE2/CEND1/NHLH2/INS/FGF13/S100B/ARRB2/NPAS4/NFATC4/PITX3/GNG7/PJA2/GHSR/CX3CR1/RGS14/SLC6A1/SGIP1/MMP17/EGR1/HTR1B/ADA/TRPV1/FXN/P2RX2/ATXN1/NTAN1/AKT1/DLG4/PBX3/NPTX2/DAB1/THRA/GLP1R/VWA1/KCNQ3/LMX1A/CLN8
GOBP_POST_TRANSLATIONAL_PROTEIN_MODIFICATION	GOBP_POST_TRANSLATIONAL_PROTEIN_MODIFICATION	GOBP_POST_TRANSLATIONAL_PROTEIN_MODIFICATION	244	-0.15434347	-1.684806593	0.002207639	0.024349851	0.018457395	973	tags=45%, list=31%, signal=33%	WDR24/USP20/TOLLIP/FLCN/PEX10/MCRS1/CNOT4/RFFL/ASH2L/RBBP6/SENP3/CUL2/KLHDC2/FBXO11/OTUD3/LCMT1/UBE2Q1/CDC16/ZRANB1/PARP12/RNF10/ANKRD9/VCP/RANBP2/RCHY1/UBE2C/TBC1D7/HSPBP1/PARP1/ATG12/SIRT1/TAF9/SETD1A/RMND5A/GSK3B/TRAF6/DCUN1D3/TAF10/MKRN2/DCUN1D5/NFYB/RNF139/RNF7/USP10/USP8/CAMLG/KLHL7/MIER2/TRAF2/ATXN3/PINK1/DDA1/BIRC8/USP31/HERC5/PINX1/MAGEC2/USPL1/SHARPIN/MAGEA2B/TRIM28/ZC3HC1/CAPN3/BARD1/MAD2L2/KBTBD2/KCTD10/ANAPC10/KBTBD8/PARP11/PCGF6/CEP63/UBE3A/FBXL2/USP37/RNF138/SMC6/PAXIP1/SENP5/RNF182/RUVBL1/USP1/UBE2D2/ZFP91/UBE2D3/UBE2N/UBE2S/FBXO7/FZR1/USP6/ASB1/GTPBP4/UBE2T/TRAIP/RNF141/SAE1/CRY1/AURKC/KLHL10/DOT1L/SUPT3H/TRIM17/CDC14B/FBXO24/DZIP3/USP30/SENP1/MDM4/ITCH
GOBP_MITOTIC_CELL_CYCLE_CHECKPOINT_SIGN	GOBP_MITOTIC_CELL_CYCLE_CHECKPOINT_SIGNAL	GOBP_MITOTIC_CELL_CYCLE_CHECKPOINT_SIGNALING	34	-0.342862005	-2.104059608	0.00222583	0.024438388	0.018524508	728	tags=59%, list=23%, signal=46%	CHEK2/LCMT1/RINT1/RAD50/ZW10/CDK5RAP2/MAD2L1BP/BARD1/MAD2L2/DYNC1LI1/BLM/TRIP13/DONSON/BU

ALING	ING										B1B/FZR1/MDC1/TPR/CDCA8/CDC14B/RAD17
GOMF_OXIDOREDUCTASE_ACTIVITY_ACTING_ON_THE_CH_CH_GROUP_OF_DONORS	GOMF_OXIDOREDUCTASE_ACTIVITY_ACTING_ON_THE_CH_CH_GROUP_OF_DONORS	GOMF_OXIDOREDUCTASE_ACTIVITY_ACTING_ON_THE_CH_CH_GROUP_OF_DONORS	14	0.515365213	2.01412926	0.002224448	0.024438388	0.018524508	955	tags=79%, list=30%, signal=55%	AKR1C1/RSAD1/PTGES2/ACAA1/DHCR24/PPOX/BLVRA/COX15/CYP2S1/ACOX1/ACAD11
GOBP_APOPTOTIC_SIGNALING_PATHWAY	GOBP_APOPTOTIC_SIGNALING_PATHWAY	GOBP_APOPTOTIC_SIGNALING_PATHWAY	140	0.218177181	1.895948267	0.002233081	0.024462153	0.018542522	1007	tags=45%, list=32%, signal=32%	CTSK/CASP4/TNFSF10/FGFR1/PYCARD/CEBPB/XBP1/ABL1/CYP1B1/ITPR1/TRAF1/PERP/SLC25A5/INHBA/MYC/PTPRC/RPS27L/NFE2L2/LTBR/HIPK2/MAP3K5/ANXA6/BMP4/ATF4/TMEM14A/PHLDA3/SRPX/MNT/TFPT/SNAI2/WFS1/CYLD/BCL2L1/TMEM109/CTSH/BCL2L2/BCL2/PTPN2/CTSC/ATP2A3/RELA/BBC3/DEDD/IER3/YAP1/PPP2R1A/IFI6/HSPB1/ERN1/GCLC/MAGED1/RRM2B/SLFN12/MDM2/NRP1/BAD/PPIF/INS/BAX/PDK2/SPN/MAZ/DNAJC10
GOBP_STRIATED_MUSCLE_TISSUE_DEVELOPMENT	GOBP_STRIATED_MUSCLE_TISSUE_DEVELOPMENT	GOBP_STRIATED_MUSCLE_TISSUE_DEVELOPMENT	47	0.328840973	2.067762204	0.002246031	0.024492432	0.018565473	1500	tags=70%, list=48%, signal=37%	PDGFRB/ZMPSTE24/ABL1/HEG1/FKBP1A/ITGB1/BMP4/NKX2-5/CITED2/ZFPM2/TSC1/BMP2/FOXC1/ID2/MAML1/SIRT2/YAP1/ENG/FRS2/ADPRHL1/VGLL4/PKP2/PRKAR1A/ARRB2/SMAD7/ACTN2/HCN4/PDGFRA/CAV3/WNT3A/ATG5/IRX3/GJC1
HP_DEPRESSION	HP_DEPRESSION	HP_DEPRESSION	91	0.257542716	1.977355855	0.002243886	0.024492432	0.018565473	1335	tags=60%, list=42%, signal=36%	UBQLN2/FGFR1/PDGFRB/GALT/GLI2/HLA-DQB1/IMPDH2/BAZ1B/HLA-B/EIF4G1/GTF2IRD2/HS6ST1/WFS1/TSC1/IRAK1/CLN3/TOR1A/ALAD/SLC11A1/CHRNB2/ELN/PODXL/VPS13C/CBS/DNAJC5/CDKN2C/DNAJC13/NR3C1/SERPINA1/STAG2/GCLC/PROKR2/ATP8B1/RRM2B/ADH1C/CHMP2B/SQSTM1/NHLH2/STUB1/DAO/ITGAM/EHMT1/PRKAR1A/SMC1A/KISS1R/CCNF/ETS1/VAPB/UBE2L3/TSHB/ALDH4A1/PXK/CHRNA2/MEN1/ANG
GOBP_T_CELL_MIGRATION	GOBP_T_CELL_MIGRATION	GOBP_T_CELL_MIGRATION	16	0.508311162	2.122002839	0.002268133	0.02463188	0.018671176	784	tags=69%, list=25%, signal=52%	MSN/PYCARD/ABL1/AIF1/CCL21/ITGB7/CCL5/AIRE/CXCL13/GPR15/APOD
GOBP_ERYTHRO	GOBP_ERYTHROC	GOBP_ERYTHROCYTE_	30	0.374817876	1.989830065	0.00226906	0.02463188	0.01867117	674	tags=50%,	HCLS1/MAFB/SLC25A5/STAT3/INHBA/

CYTE_HOMEOSTASIS	YTE_HOMEOSTASIS	HOMEOSTASIS				3		6		list=21%, signal=40%	HIPK2/BMP4/CITED2/FECH/PRDX1/ZBTB7A/ISG15/ID2/PTPN2/LYN
GOBP_ADAPTIVE_IMMUNE_RESPONSE	GOBP_ADAPTIVE_IMMUNE_RESPONSE	GOBP_ADAPTIVE_IMMUNE_RESPONSE	91	0.25745413	1.976675716	0.002303326	0.024947509	0.018910425	1399	tags=63%, list=44%, signal=36%	TYK2/LAG3/PYCARD/TFE3/HLA-DMB/HMGB1/EMP2/EIF2AK4/HAVCR2/HLA-DRB3/HLA-DQB1/STAT3/C1QC/HLA-B/HLA-E/HLA-F/HLA-DRB5/PTPRC/HLA-DMA/TFEB/HLA-A/HLA-C/TSC1/HLA-DOB/SLC11A1/CTSH/AIRE/CTSC/SVEP1/LYN/FCER1G/CXCL13/EOMES/IL17A/C8A/AGER/LIME1/CD4/SPN/CD1A/TNFSF13B/PAG1/TAP1/CD7/TNFRSF21/SMAD7/CX3CR1/CD1B/CSK/ADA/UNC93B1/STX7/LTA/CD79A/SUPT6H/TAP2/C3
GOCC_POLYMERIC_CYTOSKELETAL_FIBER	GOCC_POLYMERIC_CYTOSKELETAL_FIBER	GOCC_POLYMERIC_CYTOSKELETAL_FIBER	139	-0.189926037	-1.871702573	0.002310338	0.02496723	0.018925374	648	tags=32%, list=21%, signal=27%	HOOK2/TUBB/CDK5RAP2/NME7/KIF22/KIF4A/KRT86/RASSF1/SLC1A4/PACRG/KIFAP3/ACTL8/PAWR/TUBG2/FKBP4/DYNC1LI1/HSPH1/RASSF3/MAP2K2/TPX2/DNAI1/MAP6D1/KIFC3/NDE1/PRPH/KIF23/TUBG1/CENPE/KIF3A/NARF/KATNB1/MYO9A/EFHC1/ARFGEF2/POLB/DNAH17/DNAH8/AURKC/SPECC1/DYNLRB2/KATNA1/SPAG5/DYNLL2/PAFAH1B1/SPAG8
HP_COARCTATION_OF_AORTA	HP_COARCTATION_OF_AORTA	HP_COARCTATION_OF_AORTA	27	0.398921226	2.03814999	0.002326437	0.025084833	0.019014518	1078	tags=67%, list=34%, signal=44%	FGFR1/OSGEP/MED12/ABL1/GLI2/MID1/NKX2-5/CTCF/ELN/MMP14/STAG2/HEATR3/TALDO1/ABCD4/EHMT1/UBE3B/NIPA2/DDX3X
GOBP_DNA_INTEGRITY_CHECKPOINT_SIGNALING	GOBP_DNA_INTEGRITY_CHECKPOINT_SIGNALING	GOBP_DNA_INTEGRITY_CHECKPOINT_SIGNALING	30	-0.362329786	-2.115837015	0.002343961	0.025160953	0.019072217	787	tags=63%, list=25%, signal=48%	CHEK2/RINT1/ATR/TIMELESS/TP53BP1/E2F1/THOC5/BARD1/CEP63/BLM/DONSON/INTS7/FZR1/MDC1/RAD51/CRY1/DOT1L/CDC14B/RAD17
GOBP_STEROID_METABOLIC_PROCESS	GOBP_STEROID_METABOLIC_PROCESS	GOBP_STEROID_METABOLIC_PROCESS	71	0.282142223	2.045800402	0.002343351	0.025160953	0.019072217	958	tags=49%, list=30%, signal=35%	NFKB1/OSBPL5/AKR1C1/CBR1/DAB2/CYP1B1/OSBPL1A/CAT/TSPO/LIPA/MBTPS1/CEBPA/NFE2L1/ACAA1/SNAI2/FECH/BMP2/DHCR24/CYP11A1/CYP17A1/STARD4/UGT2A3/SERPINA6/LIMA1/NR3C1/FGF1/ADM/ATP8B1/SCP2/NSDHL/PMVK/OSBP/STUB1/HSD17B8/CYP3A43
HP_ABNORMAL_ORAL_MUCOSA_MORPHOLOGY	HP_ABNORMAL_ORAL_MUCOSA_MORPHOLOGY	HP_ABNORMAL_ORAL_MUCOSA_MORPHOLOGY	83	0.261707256	1.961679452	0.002363543	0.025301596	0.019178826	736	tags=41%, list=23%, signal=32%	ANTXR2/SETBP1/F10/SH3PXD2B/PDGFRB/TBC1D2B/SCARB2/RHBDF2/IFNGR1/KRT14/GLB1/TRIM8/HLA-DQB1/CAT

GOCC_MEMBRANE_PROTEIN_COMPLEX	GOCC_MEMBRANE_PROTEIN_COMPLEX	GOCC_MEMBRANE_PROTEIN_COMPLEX	247	0.176890237	1.752080739	0.002367585	0.025301596	0.019178826	1175	tags=46%, list=37%, signal=31%	/PERP/COL3A1/BAZ1B/STAT3/HLA-B/GTF2IRD2/PLAGL1/HPS1/TSC1/IRAK1/DHCR24/EDA/ELN/MEFV/MMP14/CTSC/CDKN2C/RELA/NR3C1/SLC29A3 SHC1/CCDC115/TYK2/COPZ1/COX7A1/TOMM20/PHB2/CDH11/HLA-DMB/HMGB1/SEC22B/COX7B/VDAC1/HLA-DRB3/COX8A/CHCHD3/TRAF1/HLA-DQB1/CLIC1/CLCC1/SOCS2/SLC25A5/ATP1A2/RAC1/COPZ2/CD34/HLA-B/HLA-E/HLA-F/HLA-DRB5/SSR4/HM13/STT3A/CCDC51/FKBP1A/ITGB1/BET1L/HLA-DMA/HLA-A/FLOT2/MAP3K5/HLA-C/CLTB/ABHD12/IGF2R/GJA4/NDUFA4L2/ITGA9/DNAJC11/ITGB7/KCNB1/RAMP2/BMP2/SPCS2/AP1G2/HLA-DOB/CHRNB2/ABHD6/GRIA4/BCL2/DERL1/TAPBPL/DENND4C/AP2M1/CNIH3/UVRAG/LYN/PIK3C2B/FCER1G/GRIK5/TRAF5/GNA12/CLINT1/GRM1/ITGA3/CDH3/ITGA5/SEC61A1/GNAI1/SEC23A/C8A/ERN1/OSTM1/CHMP2B/FAM8A1/CLIC5/CALM3/PPIF/LIME1/TIMM17B/CD4/BAX/KCNH4/GABRR1/SEC63/ITGAM/NOXA1/DUOX2/ABCD4/CHMP4C/CDH4/APH1A/KCNB2/ITGB8/GNB1/VPS11/TAP1/CLDN4/SOCS1/CDH9/GNG7/CHMP6/CHMP7/KCNAB2
GOBP_NON_CANONICAL_WNT_SIGNALING_PATHWAY	GOBP_NON_CANONICAL_WNT_SIGNALING_PATHWAY	GOBP_NON_CANONICAL_WNT_SIGNALING_PATHWAY	11	0.567103748	2.037301914	0.002420444	0.025800936	0.01955733	986	tags=82%, list=31%, signal=56%	DAB2/MED12/ABL1/RAC1/PLEKHA4/MYOC/FZD4/WNT11/DVL2
GOBP_STEM_CELL_DEVELOPMENT	GOBP_STEM_CELL_DEVELOPMENT	GOBP_STEM_CELL_DEVELOPMENT	21	0.43953335	2.011912783	0.002430406	0.025800936	0.01955733	1051	tags=71%, list=33%, signal=48%	EFNB1/SMO/PTPRC/HAND2/ANXA6/BMP4/CITED2/SNAI2/FOXC1/BCL2/NRP2/MSI2/ENG/NRP1/FGF19
GOBP_CALCIUM_ION_HOMEOSTASIS	GOBP_CALCIUM_ION_HOMEOSTASIS	GOBP_CALCIUM_ION_HOMEOSTASIS	70	0.273382149	1.963315633	0.002428425	0.025800936	0.01955733	1758	tags=74%, list=56%, signal=34%	ABL1/GSTO1/ITPR1/SLC25A23/ATP1A2/PTPRC/TPCN1/FKBP1A/ANXA6/ATF4/CCL21/WFS1/CLN3/CCL5/BCL2/SRI/IBTK/LYN/TRPM2/ATP2A3/F2RL3/GRM1/CCR5/CCR1/CALM3/LIME1/BAX/SLC24A3/GPR3/CCL8/DIAPH1/VAPB/SYPL2/HTR1B/XCR1/TRPV1/THY1/CAV3/S100

											A14/ATG5/FIS1/ATP13A1/ATP1B1/ATP2A1/TRPV5/BDKRB1/SV2A/GPR12/ATP7B/STIM1/ATP2B3/GP1BB
GOBP_CELLULAR_RESPONSE_TO_TOXIC_SUBSTANCE	GOBP_CELLULAR_RESPONSE_TO_TOXIC_SUBSTANCE	GOBP_CELLULAR_RESPONSE_TO_TOXIC_SUBSTANCE	27	0.397312183	2.029929137	0.00244969	0.025891341	0.019625858	545	tags=48%, list=17%, signal=40%	AKR1A1/GSTO1/LTC4S/H19/ALOX5AP/CAT/HP/NFE2L2/NQO1/PTGES/PRDX1/CCS/SLC11A1
HP_MEGALOCORNEA	HP_MEGALOCORNEA	HP_MEGALOCORNEA	14	0.512563078	2.003178074	0.002445312	0.025891341	0.019625858	689	tags=71%, list=22%, signal=56%	FBN1/MAF/SH3PXD2B/CDH11/CTBP1/BAZ1B/GTF2IRD2/TEK/ELN/PIGG
GOBP_REGULATION_OF_BODY_FLUID_LEVELS	GOBP_REGULATION_OF_BODY_FLUID_LEVELS	GOBP_REGULATION_OF_BODY_FLUID_LEVELS	79	0.254444279	1.880760775	0.002483977	0.02619615	0.019856906	1441	tags=63%, list=46%, signal=35%	F10/TFPI/XBP1/EMP2/PPP3CA/PLSCR1/ST3GAL4/CLIC1/SOCS2/COL3A1/ILK/HEG1/ITPK1/HPS6/NFE2L2/SLC29A1/MUC2/WFS1/VWF/CSN2/HAS2/LYN/PLAU/FCER1G/F2RL3/GNA12/CORO2B/APLN/SERPINA1/FOSL2/ADM/HSPB1/SERPINE2/NR1H2/OAS2/TSPAN8/AGR2/AQP1/ADRA2B/ADA/AQP6/PDGFRA/SLC22A2/ENTPD2/F11/WNT3A/F8/F11R/ATG5/TSPAN32
GOMF_PEPTIDE_BINDING	GOMF_PEPTIDE_BINDING	GOMF_PEPTIDE_BINDING	71	0.280944931	2.03711889	0.002518008	0.026496941	0.020084908	1640	tags=75%, list=52%, signal=37%	TOMM20/HLA-DMB/HLA-DRB3/GPRASP2/HLA-DQB1/PPIC/PPP3CA/HLA-B/HLA-E/INHBA/HLA-F/HLA-DRB5/HLA-DMA/HLA-A/HLA-C/CLTB/RAMP2/DHCR24/HLA-DOB/GRIA4/FZD4/AP2M1/RELA/MAML1/BCHE/SEC61A1/AGER/CRHR2/PPIF/ITGAM/CD1A/PFDN1/TAP1/MRGPRX2/APBB2/GHSR/CD1B/CRIP1/CLSTN3/PCSK5/NPEPPS/ANG/SSTR3/TAP2/NPBWR2/GLP1R/CACNA1B/MICA/ATP1A3/CD209/CLSTN1/BDKRB1/ITM2C
GOBP_REGULATION_OF_FAT_CELL_DIFFERENTIATION	GOBP_REGULATION_OF_FAT_CELL_DIFFERENTIATION	GOBP_REGULATION_OF_FAT_CELL_DIFFERENTIATION	30	0.372932533	1.979821172	0.002543174	0.026703325	0.020241349	764	tags=50%, list=24%, signal=38%	LRP3/CEBPB/TFE3/XBP1/JDP2/CEBPA/ALOX5/ZFPM2/SNAI2/CCDC3/BMP2/ID2/TRIO/SIRT2/YAP1
GOBP_STRESS_ACTIVATED_PROTEIN_KINASE_SIGNALING_CASCADE	GOBP_STRESS_ACTIVATED_PROTEIN_KINASE_SIGNALING_CASCADE	GOBP_STRESS_ACTIVATED_PROTEIN_KINASE_SIGNALING_CASCADE	50	0.316034557	2.042351699	0.002587499	0.026847083	0.020350318	503	tags=40%, list=16%, signal=34%	NFKB1/PYCARD/HMGB1/ZMPSTE24/EIF2AK2/MYC/GADD45A/HAND2/LTBR/MID1/IGFBP6/HIPK2/MAP3K5/BMP4/CCL21/CYLD/IRAK1/PRDX1/CDC42EP5/BMP2
GOBP_COLLAGE	GOBP_COLLAGEN	GOBP_COLLAGEN_MET	19	0.457303214	2.020555417	0.00258035	0.02684708	0.02035031	1231	tags=74%,	CTSK/LARP6/INHBA/PCOLCE/ITGB1/B

N_METABOLIC_PROCESS	_METABOLIC_PROCESS	ABOLIC_PROCESS				6	3	8		list=39%, signal=45%	MP4/MMP23B/ADAM15/MMP14/ENG/FOSL2/MMP15/TNXB/MMP17
HP_CHRONIC_CONSTIPATION	HP_CHRONIC_CONSTIPATION	HP_CHRONIC_CONSTIPATION	21	0.437483724	2.002530858	0.002589254	0.026847083	0.020350318	780	tags=57%, list=25%, signal=43%	TFE3/ABL1/SMO/TRIM8/EIF4G1/ELN/MEFV/DNAJC13/TRIO/DCPS/MAGEL2/PPP2R1A
GOBP_CELL_SUBSTRATE_JUNCTION_ORGANIZATION	GOBP_CELL_SUBSTRATE_JUNCTION_ORGANIZATION	GOBP_CELL_SUBSTRATE_JUNCTION_ORGANIZATION	31	0.36865778	1.969231141	0.002582167	0.026847083	0.020350318	1342	tags=74%, list=43%, signal=43%	CDH11/ABL1/TRIP6/RAC1/ITGB1BP1/MYOC/TSC1/TEK/BCL2/ARHGEF7/PHLDB2/MMP14/FAM107A/CORO2B/ITGA5/APOD/NRP1/CLASP2/ROCK2/ACTN2/DLC1/CTTN/THY1
GOBP_NEGATIVE_REGULATION_OF_CELL_DEVELOPMENT	GOBP_NEGATIVE_REGULATION_OF_CELL_DEVELOPMENT	GOBP_NEGATIVE_REGULATION_OF_CELL_DEVELOPMENT	58	0.283769669	1.921991228	0.002574958	0.026847083	0.020350318	1438	tags=69%, list=46%, signal=38%	FBN1/LAG3/HMGB1/MAFB/PPP3CA/BTG2/TSPO/C1QC/INHBA/MYC/CEBPA/BMP4/PTN/CBFB/SNAI2/PIAS3/CDK5/FGL2/ID2/PTPN2/LYN/TCTA/SIRT2/NRP1/ULK1/FGF13/NFATC4/SOCS1/GDI1/SMAD7/PITX3/ADIPOQ/TBX6/TRPV1/THY1/MT3/CAV3/WNT3A/LTF/DAB1
GOBP_NEGATIVE_REGULATION_OF_PROGRAMMED_CELL_DEATH	GOBP_NEGATIVE_REGULATION_OF_PROGRAMMED_CELL_DEATH	GOBP_NEGATIVE_REGULATION_OF_PROGRAMMED_CELL_DEATH	179	0.195008525	1.816365969	0.002590361	0.026847083	0.020350318	853	tags=38%, list=27%, signal=29%	SHC1/NFKB1/TXNDC5/AKR1A1/PHB2/DAB2/IFIT3/CEBPB/XBP1/ABL1/SMO/HCLS1/VDAC1/EIF2AK2/CAT/SOCS2/BTG2/SLC25A5/MYC/NFE2L2/HAND2/BFAR/ITGB1/ARHGDIA/HIPK2/BMP4/ATF4/NQO1/NKX2-5/TMEM14A/ANXA4/MNT/CITED2/PRKCH/SNAI2/CCL21/WFS1/CLN3/BCL2L1/TMEM109/RAMP2/TEK/DHCR24/CCL5/FOXC1/CTSH/ANGPTL4/GRIA4/BCL2L2/BCL2/PRAP1/AMIGO2/ARF4/HSH2D/CBS/DNAJC5/UBE2B/RELA/PRDX3/IER3/RGL2/YAP1/ITGA5/CCR5/NTF3/IFI6/HSPB1/GCLC
GOBP_REGULATION_OF_ACTIN_FILAMENT_BASED_PROCESS	GOBP_REGULATION_OF_ACTIN_FILAMENT_BASED_PROCESS	GOBP_REGULATION_OF_ACTIN_FILAMENT_BASED_PROCESS	85	0.248328986	1.889073796	0.002601228	0.02690173	0.020391742	1415	tags=61%, list=45%, signal=35%	RALA/PDGFRB/PYCARD/ABL1/FHOD1/HCLS1/TMOD1/ATP1A2/RAC1/ITGB1BP1/FCHSD1/CYFIP1/ADD1/MYOC/TRIOBP/CCL21/TSC1/MYADM/CDC42EP5/DSTN/GMFG/ELN/SRI/PHLDB2/RHOC/LIMA1/TRPM2/TMOD4/FAM107A/CORO2B/MAGEL2/WNT11/ADD3/PKP2/NRP1/CLASP2/RHOG/FGF13/ROCK2/TTC8/ACTN2/DLC1/CTTN/CFL2/SSH3/RNH1/HCN4/PDGFRA/SPTAN1/COTL1/CAV3/F11R
GOBP_GLIAL_CE	GOBP_GLIAL_CEL	GOBP_GLIAL_CELL_DI	50	0.315858275	2.041212484	0.00261741	0.02699726	0.02046415	1438	tags=70%,	NFIA/MED12/IFNGR1/ABL1/SMO/LAM

LL_DIFFERENTIATION	L_DIFFERENTIATION	FFERENTIATION				2	4	7		list=46%, signal=39%	C3/STAT3/ILK/PTN/MYOC/CDK5/BMP2/PRX/ID2/LYN/CDKN2C/EIF2B1/RELA/NR3C1/LGI4/SIRT2/EOMES/AGER/SERPINE2/CNTNAP1/GCM1/TNFRSF21/PRDM8/ASCL1/CSK/TPPP/EMX1/AKT1/ZNF488/DAB1
GOCC_TRANSFERASE_COMPLEX_TRANSFERRING_PHOSPHORUS_CONTAINING_GROUPS	GOCC_TRANSFERASE_COMPLEX_TRANSFERRING_PHOSPHORUS_CONTAINING_GROUPS	GOCC_TRANSFERASE_COMPLEX_TRANSFERRING_PHOSPHORUS_CONTAINING_GROUPS	71	-0.255900705	-2.000069002	0.002621693	0.026997264	0.020464157	797	tags=46%, list=25%, signal=36%	CAB39/CDK5R1/BCCIP/ERCC3/TAF9/PIK3CG/TAF6/GTF2E1/TAF10/PRKAG2/POLR3E/GTF2H1/TBK1/GTF2H2/POLE/MAD2L2/PCNA/PRIM1/POLRMT/CDK7/POLA2/PAF1/POLG2/CSNK2A2/TSSK2/PRKAA1/CKS2/AKAP4/PARD6A/CCNH/SUPT3H/CCNB2/TBPL1
GOBP_REGULATION_OF_CELL_SUBSTRATE_JUNCTION_ORGANIZATION	GOBP_REGULATION_OF_CELL_SUBSTRATE_JUNCTION_ORGANIZATION	GOBP_REGULATION_OF_CELL_SUBSTRATE_JUNCTION_ORGANIZATION	18	0.478120787	2.079660783	0.002706998	0.027642873	0.020953534	1342	tags=83%, list=43%, signal=48%	ABL1/RAC1/ITGB1BP1/MYOC/TSC1/TEK/PHLDB2/MMP14/FAM107A/APOD/NRP1/CLASP2/ROCK2/DLC1/THY1
GOBP_DNA_TEMPLATED_TRANSCRIPTION_INITIATION	GOBP_DNA_TEMPLATED_TRANSCRIPTION_INITIATION	GOBP_DNA_TEMPLATED_TRANSCRIPTION_INITIATION	26	-0.371042322	-2.073774943	0.00269602	0.027642873	0.020953534	806	tags=62%, list=26%, signal=46%	MED4/ERCC3/TAF9/TAF6/GTF2E1/TAF10/GTF2H2/POLRMT/CDK7/PAXIP1/ATF7IP/MDC1/CCNH/SETX/DR1/TBPL1
HP_ABNORMALITY_OF_THE_NAIL	HP_ABNORMALITY_OF_THE_NAIL	HP_ABNORMALITY_OF_THE_NAIL	98	0.237328824	1.872737861	0.002708937	0.027642873	0.020953534	810	tags=40%, list=26%, signal=31%	RNF113A/CTSK/MAF/SETBP1/EFNB1/FGFR1/IGF2/PQBP1/CTBP1/ZMPSTE24/COX7B/KRT14/TRIM8/PERP/BAZ1B/STAT3/HLA-B/PIGW/HLA-C/BMP4/GTF2IRD2/PLAGL1/APCDD1/TSC1/BMP2/EDA/ELN/AIRE/PAX9/PLAG1/ZNF462/CTSC/KRT85/CDKN1C/ITGA3/CDH3/PRR12/PTDSS1/ENG
GOCC_ORGANELLE_SUBCOMPARTMENT	GOCC_ORGANELLE_SUBCOMPARTMENT	GOCC_ORGANELLE_SUBCOMPARTMENT	299	0.169130307	1.734252657	0.002703458	0.027642873	0.020953534	1048	tags=41%, list=33%, signal=30%	PLOD1/ANTXR2/CASP4/COPZ1/WDR81/OSBPL5/SCARB2/RHBDF2/XBP1/SEC22B/CALU/ZMPSTE24/TGFBI/LTC4S/TMEM129/HLA-DRB3/CYP1B1/ALOX5AP/ITPR1/RHOBTB3/HLA-DQB1/MGAT4B/ST3GAL4/CLCC1/ACSL5/RAC1/COPZ2/HLA-B/HLA-E/EXTL1/CYP2U1/MBTPS1/HLA-F/HLA-DRB5/SSR4/HM13/STT3A/BFAR/PIGW/FKBP1A/VAPA/ANXA7/SULF1/HLA-A/MAP3K5/HLA-C/TGFA/CLTB/PDIA6/TMEM14A/NAT8/NFE2L1/BACE2/FMO1/ABHD12/MMP23B/IGF2R

											/WFS1/PTGES/WIPI1/RNF185/CLN3/TMEM109/EXTL2/TOR1A/DHCR24/ZDHHC9/TBC1D23/SPCS2/CYP2W1/EDA/SLC9A8/RAB11A/BCL2/DERL1/LLGL1/HAS2/VPS13C/SRI/RHOC/TAPBPL/CYP17A1/SCAMP3/CNIH3/CYP4B1/PIGG/ATP2A3/PDIA5/PLA2G4C/CYP2S1/SEC61A1/SLC16A11/PTDSS1/EMD/CCDC91/ZDHHC6/RAB9A/ALG2/SEC23A/ILVBL/TMEM119/IFI6/ERN1/ATP8B1/NSDHL/RNF5/YIPF4/CLASP2/FAM8A1/OSBP/CYP3A43/ACSL1/CD4/BAX/ZFYVE1/PREB/SEC63/RHOG/LCTL/ABCD4/ULK1/FMO5/APH1A
GOMF_PROTEIN_CONTAINING_COMPLEX_BINDING	GOMF_PROTEIN_CONTAINING_COMPLEX_BINDING	GOMF_PROTEIN_CONTAINING_COMPLEX_BINDING	356	0.16294395	1.728418645	0.002713129	0.027642873	0.020953534	909	tags=37%, list=29%, signal=30%	SHC1/FBN1/NFKB1/LASP1/CTSK/NFIA/PODN/IGF2/MED12/CEBPB/PQBP1/HLA-DMB/IQGAP1/HMGB1/CTBP1/ABL1/EMP2/TGFBI/FHOD1/HCLS1/JDP2/KRT14/AIF1/HLA-DRB3/ALOX5AP/TSHZ3/MAFB/GLI2/HLA-DQB1/PPP3CA/NAP1L1/COL3A1/STAT3/RAC1/DET1/ITGB1BP1/HLA-E/INHBA/STAB1/REPIN1/HLA-DRB5/PCOLCE/MBD2/MYC/GADD45A/PTPRC/CEBPA/SLC30A9/PLTP/ADAMTS5/ITGB1/HLA-DMA/FBXW11/CYFIP1/ANXA7/HLA-A/ANXA6/CORO6/PTN/ATF4/NKX2-5/LUM/MNT/ADD1/TFPT/TRIOBP/CITED2/IGF2R/SNAI2/TPM2/WFS1/RNF185/ITGA9/CD177/ADAM15/ITGB7/VWF/SUCLG2/PRMT2/EBF2/CTCF/HLA-DOB/ALAD/FOXC1/DSTN/RBM23/GMFG/CHRNB2/BCL2L2/ISG15/IFRD2/DERL1/AIRE/MYO1F/PTPN2/TAPBPL/MMP14/FZD4/SVEP1/LYN/LIMA1/MRPS27/FCER1G/NAP1L3/PLS3/RELA/GNA12/SIRT2/CORO2B/YAP1/CENPB/CDKN1C/ITGA3/ITGA5/SEC61A1/GNAI1/FGF1/ENG/FOSL2/EOMES/STAG2/C8A/GTPBP6/ADD3/GCLC/AGER/SLFN12/SQSTM1/ARPC1B/MDM2/RNF5/PKP2
HP_ABNORMALITY_ON_PULMONA	HP_ABNORMALITY_ON_PULMONA	HP_ABNORMALITY_ON_PULMONARY_FUNCTI	30	0.371991717	1.974826573	0.002725915	0.027714427	0.021007773	853	tags=57%, list=27%,	NFKB1/PLOD1/EIF2AK4/SMO/GLB1/HLA-B/BTNL2/HPS1/TPM2/TSC1/SBF2/TO

RY_FUNCTION_TESTING	RY_FUNCTION_TESTING	ON_TESTING								signal=42%	R1A/SLC11A1/SLC34A2/SERPINA1/ENG/GCLC
GOBP_CELLULAR_DEFENSE_RESPONSE	GOBP_CELLULAR_DEFENSE_RESPONSE	GOBP_CELLULAR_DEFENSE_RESPONSE	11	0.562057782	2.019174443	0.002763987	0.028042218	0.021256241	1208	tags=91%, list=38%, signal=56%	LGALS3BP/TYROBP/LSP1/ITGB1/RELA/KIR2DL4/CCR5/KIR2DS3/SPN/CX3CR1
HP_ABNORMALITY_OF_THE_PULMONARY_ARTERY	HP_ABNORMALITY_OF_THE_PULMONARY_ARTERY	HP_ABNORMALITY_OF_THE_PULMONARY_ARTERY	23	0.411315407	1.955357572	0.002814743	0.028497053	0.02160101	423	tags=48%, list=13%, signal=42%	FBN1/FGFR1/MED12/MGP/ITPR1/COL3A1/BAZ1B/MRAS/GTF2IRD2/NKX2-5/SNAI2
GOBP_TETRAPYRROLE_BIOSYNTHETIC_PROCESS	GOBP_TETRAPYRROLE_BIOSYNTHETIC_PROCESS	GOBP_TETRAPYRROLE_BIOSYNTHETIC_PROCESS	13	0.54653415	2.087023859	0.002823936	0.028509066	0.021610116	759	tags=69%, list=24%, signal=53%	RSAD1/TSPO/ABCB7/TMEM14A/NFE2L1/FECH/ALAD/PPOX/COX15
HP_TOOTH_MALPOSITION	HP_TOOTH_MALPOSITION	HP_TOOTH_MALPOSITION	80	0.267667945	1.990471657	0.002833715	0.028509066	0.021610116	787	tags=42%, list=25%, signal=33%	RNF113A/FBN1/CTSK/PLOD1/SETBP1/FGFR1/SH3PXD2B/CDH11/TBC1D2B/IGF2/MED12/ZMPSTE24/ABL1/H19/GLB1/GLI2/COL3A1/BAZ1B/SSR4/TGFA/BMP4/GTF2IRD2/TPM2/BMP2/ZDHHC9/ZBTB7A/EDA/ELN/PAX9/CBS/TRIO/LGI4/CDH3/PRR12
HP_APATHY	HP_APATHY	HP_APATHY	22	0.422147981	1.97665127	0.002831325	0.028509066	0.021610116	1092	tags=68%, list=35%, signal=45%	FGFR1/HTRA1/GLI2/HLA-DQB1/EIF4G1/HMGCL/ALAD/PODXL/VPS13C/DNAJC13/STAG2/CHMP2B/SQSTM1/EHMT1/SMC1A
GOBP_OSTEOCLAST_DIFFERENTIATION	GOBP_OSTEOCLAST_DIFFERENTIATION	GOBP_OSTEOCLAST_DIFFERENTIATION	25	0.406638462	2.013322504	0.002887745	0.028931597	0.021930398	930	tags=60%, list=29%, signal=43%	SH3PXD2A/FBN1/CEBPB/TFE3/TYROBP/MAFB/PPP3CA/PIAS3/BMP2/TCTA/FOSL2/IL17A/OSTM1/KLF10/CCR1
GOBP_GLIOGENESIS	GOBP_GLIOGENESIS	GOBP_GLIOGENESIS	67	0.281907162	1.998040804	0.002882771	0.028931597	0.021930398	1385	tags=66%, list=44%, signal=38%	NFIA/MED12/IFNGR1/ABL1/SMO/TSPO/LAMC3/COL3A1/STAT3/ILK/PTN/APCDD1/MYOC/PRKCH/CDK5/BMP2/PRX/ID2/PLAG1/MMP14/LYN/CDKN2C/EIF2B1/RELA/NR3C1/LGI4/SIRT2/EOMES/AGER/SERPINE2/CDK5R2/CNTNAP1/GCM1/TNFRSF21/PITX3/PRDM8/CX3CR1/ASCL1/CSK/TPPP/LTA/EMX1/AKT1/ZNF488
GOBP_NEGATIVE_REGULATION_OF_DEVELOPMENTAL_PROCESS	GOBP_NEGATIVE_REGULATION_OF_DEVELOPMENTAL_PROCESS	GOBP_NEGATIVE_REGULATION_OF_DEVELOPMENTAL_PROCESS	177	0.19748508	1.830730085	0.002894278	0.028936767	0.021934317	1512	tags=60%, list=48%, signal=33%	FBN1/CTSK/LAG3/LRP3/IGF2/XBP1/COL4A2/HMGB1/PGK1/EIF2AK4/SMO/JDP2/CHRD/MAFB/PPP3CA/WWC3/SOCS2/BTG2/TSPO/STAT3/C1QC/LOXL2/INHBA/STAB1/MYC/GADD45A/CEBPA/NFE2L2/HAND2/ISL2/ITGB1/SULF1/BMP4/PTN/NKX2-5/ALOX5/CITED2/CBFB/ZFPM2/SNAI2/PIAS3/CDK5/BCL2L1/BMP2/

											TEK/FGL2/FOXC1/BCL2/ID2/PTPN2/LYN/UBE2B/TRIO/CLDN5/TCTA/SIRT2/YAP1/CDH3/FRS2/WNT11/TMEM119/MIXL1/VGLL4/KLF2/PKP2/NRP1/CCR1/NR1H2/OMA1/HUWE1/ULK1/FGF13/ROCK2/NFATC4/SOCS1/GDI1/SMAD7/PITX3/ADIPOQ/ATF2/ASCL1/ADIPOR1/KREMEN1/MEN1/TBX6/TRPV1/FXN/THY1/NOTCH4/NPHP3/ZBTB16/MT3/CAV3/WNT3A/RARG/LTF/DAB1/FOXE3/IRX3/WNT1/DNM3/BCR/LMX1A/SEMA5B/DDB1/LBH
GOBP_MICROTUBULE_ORGANIZING_CENTER_ORGANIZATION	GOBP_MICROTUBULE_ORGANIZING_CENTER_ORGANIZATION	GOBP_MICROTUBULE_ORGANIZING_CENTER_ORGANIZATION	34	-0.334680984	-2.053854699	0.002926023	0.02909262	0.022052455	768	tags=53%, list=24%, signal=40%	BBS4/SIRT1/KIAA0753/ARL2/CDK5RAP2/RBM14/CEP63/CEP250/NDE1/DZIP1/KIF3A/CEP72/PARD6A/CCDC42/RANBP1/FOXJ1/PAFAH1B1/WDR62
GOBP_REGULATION_OF_T_CELL_MIGRATION	GOBP_REGULATION_OF_T_CELL_MIGRATION	GOBP_REGULATION_OF_T_CELL_MIGRATION	11	0.560562138	2.01380139	0.002920142	0.02909262	0.022052455	977	tags=82%, list=31%, signal=57%	PYCARD/ABL1/AIF1/CCL21/CCL5/AIRE/CXCL13/APOD/SPN
GOBP_CELL_CELL_SIGNALING	GOBP_CELL_CELL_SIGNALING	GOBP_CELL_CELL_SIGNALING	328	0.162036049	1.700140939	0.002928016	0.02909262	0.022052455	1510	tags=56%, list=48%, signal=33%	NFKB1/EFNB1/TNFSF10/CDH11/DAB2/IGFBP4/MAOB/RGS10/MED12/TYROBP/ABL1/EIF2AK4/SMO/VDAC1/RAB1A/TSHZ3/ITPR1/NADK/ANGPTL2/PPP3CA/TSPO/ATP1A2/TMEM25/RAC1/CD34/INHBA/RBMS3/ILK/STAB1/MBD2/FCHSD1/ITGB1/FBXW11/CYFIP1/SULF1/IGFBP6/SLC29A1/BMP4/PTN/ATF4/NQO1/NKX2-5/PLEKHA4/APCDD1/ALOX5/MYOC/SNAI2/CCL21/GJA4/PTGES/IL15/PCDHB16/CYLD/CDK5/CLN3/KCNB1/TOR1A/GHRH/BMP2/TEK/PCDHB14/EDA/CCL5/CHRNB2/ABHD6/GRIA4/RPH3AL/HRH3/SRI/SEZ6/FZD4/FER1L4/DNAJC5/LYN/SYT12/UBE2B/KMO/RELA/TRIO/GRIK5/BCHE/FAM107A/NR3C1/CXCL13/LTBP1/YAP1/GRM1/ITGA3/CDH3/PPP2R1A/CCR5/NTF3/EMD/IL17A/PCDHB4/WNT11/AGER/VGLL4/SERPINE2/CHMP2B/SQSTM1/KLF10/PKP2/NRP1/CRHR2/BAD/CCR1/OSBP/UCN3/INS/TM7SF3/GABRR1/DVL2/MPZL1/MINK1/TM2D3/ITGB8/FGF13/PRKAR1A/DDX3X/S100B

											/GNB1/TNKS2/CASK/ARRB2/NOTUM/NPAS4/NFATC4/FAM53B/CCL8/PSMD9/WNT2B/ADIPOQ/TNXB/GHSR/CX3CR1/RGS14/SLC6A1/ZNF703/AQP1/EGR1/TSHB/ADRA2B/HTR1B/GPR119/JAGN1/CLSTN3/PCSK5/LTA/PXK/KREMEN1/CHRNA2/SLC5A7/VPS18/FFAR2/TRPV1/CCL22/P2RX2/THY1/HCN4/SSTR3/SNCB/AKT1/DRP2/DLG4/NPHP3/WNT3A/LTB/NPTX2/CA2/RARG/GAL3ST4/TSPAN32/PTPN23/SLC32A1/GLP1R/CACNA1B/WNT1/SIGLEC6/KCNQ3/BCR/GJC1/STX3/CACNA1E/DDB1
HP_X_LINKED_DOMINANT_INHERITANCE	HP_X_LINKED_DOMINANT_INHERITANCE	HP_X_LINKED_DOMINANT_INHERITANCE	17	0.472264016	2.025589494	0.002941403	0.029165372	0.022107601	1151	tags=76%, list=37%, signal=49%	RNF113A/UBQLN2/EFNB1/MED12/COX7B/COL4A5/EDA/NSDHL/FHL1/DDX3X/SMC1A/CASK/GDI1
GOBP_ESTABLISHMENT_OF_PROTEIN_LOCALIZATION_TO_ORGANELLE	GOBP_ESTABLISHMENT_OF_PROTEIN_LOCALIZATION_TO_ORGANELLE	GOBP_ESTABLISHMENT_OF_PROTEIN_LOCALIZATION_TO_ORGANELLE	101	-0.208741196	-1.845517771	0.002987434	0.029560847	0.022407375	604	tags=33%, list=19%, signal=27%	RGPD5/PINK1/NUP93/SRP19/KPNA6/MON1A/TIMM23/TRIM28/SNUPN/KPNB1/VPS37A/ZFAND6/NCOA4/POLA2/HK1/LAMP3/UBE2D3/DNAJC15/RAB8B/FBXO7/CSE1L/HACL1/CSNK2A2/SAE1/PRKAA1/TPR/MTX2/NUP88/IFT122/HSPA1L/SH3GLB1/NUP155/KPNA2
GOBP_PIGMENT_METABOLIC_PROCESS	GOBP_PIGMENT_METABOLIC_PROCESS	GOBP_PIGMENT_METABOLIC_PROCESS	17	0.471838229	2.02376325	0.003003654	0.029633352	0.022462334	779	tags=65%, list=25%, signal=49%	TSPO/ABCB7/TMEM14A/NFE2L1/FECH/ALAD/PPOX/BCL2/BLVRA/COX15/CDH3
GOBP_FIBROBLAST_MIGRATION	GOBP_FIBROBLAST_MIGRATION	GOBP_FIBROBLAST_MIGRATION	14	0.508884612	1.988802043	0.003013248	0.029633352	0.022462334	741	tags=64%, list=24%, signal=49%	IQGAP1/CD248/RAC1/ITGB1BP1/ILK/ITGB1/CLN3/ARHGEF7/GNA12
GOBP_REGULATION_OF_CELL_JUNCTION_ASSEMBLY	GOBP_REGULATION_OF_CELL_JUNCTION_ASSEMBLY	GOBP_REGULATION_OF_CELL_JUNCTION_ASSEMBLY	45	0.322243922	1.98698577	0.003009363	0.029633352	0.022462334	1415	tags=69%, list=45%, signal=39%	ABL1/RAC1/ITGB1BP1/EIF4G1/MYOC/PRKCH/SNAI2/TSC1/TEK/CHRNB2/AMIGO2/RAPGEF1/PHLDB2/MMP14/CLDN5/FAM107A/APOD/IL17A/NRP1/CLASP2/LRFN3/SLITRK2/ROCK2/ABI3/GHSR/DLC1/CLSTN3/THY1/LRRN3/WNT3A/F11R
GOCC_PIGMENT_GRANULE	GOCC_PIGMENT_GRANULE	GOCC_PIGMENT_GRANULE	26	0.406021789	2.052946896	0.003071092	0.030079379	0.022800427	821	tags=58%, list=26%, signal=43%	SEC22B/CALU/RAB1A/NAP1L1/RAC1/ITGB1/ANXA6/PDIA6/BACE2/PRDX1/MMP14/DNAJC5/STOM/YWHAB/RAB9A
GOBP_REGULATION_OF_DNA_REPLICATION	GOBP_REGULATION_OF_DNA_REPLICATION	GOBP_REGULATION_OF_DNA_REPLICATION	34	-0.333168314	-2.044571816	0.003071112	0.030079379	0.022800427	954	tags=62%, list=30%, signal=44%	MCRS1/LIG3/RBBP6/ENDOG/ATR/TIMELESS/MCM5/TTF1/BAZ1A/CDC7/SMC3/PCNA/USP37/BLM/RUVBL1/RFC4/PO

GOBP_REGULATION_OF_CHEMOTAXIS	GOBP_REGULATION_OF_CHEMOTAXIS	GOBP_REGULATION_OF_CHEMOTAXIS	35	0.364415972	2.043821805	0.003156435	0.030755038	0.023312582	1508	tags=80%, list=48%, signal=42%	LG2/GTPBP4/FAF1/GMNN/RAD17 FGFR1/PDGFRB/HMGB1/VEGFB/AIF1/RAC1/C3AR1/PTN/CCL21/ROBO3/CCL5/PTPN2/LYN/CXCL13/FGF1/NTF3/HSPB1/AGER/NRP1/CCR1/CX3CR1/PDGFRA/OXSR1/GPSM3/WNT3A/S100A14/STX3/SEMA5B
GOBP_POSITIVE_REGULATION_OF_OSTEOBLAST_DIFFERENTIATION	GOBP_POSITIVE_REGULATION_OF_OSTEOBLAST_DIFFERENTIATION	GOBP_POSITIVE_REGULATION_OF_OSTEOBLAST_DIFFERENTIATION	17	0.470979702	2.020080936	0.003159282	0.030755038	0.023312582	842	tags=65%, list=27%, signal=48%	LRP3/CEBPB/IFITM1/PPP3CA/CLIC1/ILK/CEBPA/BMP4/BMP2/YAP1/TMEM119
HP_CEREBRAL_ISCHEMIA	HP_CEREBRAL_ISCHEMIA	HP_CEREBRAL_ISCHEMIA	30	0.368478309	1.956174623	0.003152611	0.030755038	0.023312582	810	tags=53%, list=26%, signal=40%	FBN1/HTRA1/IFNGR1/ZMPSTE24/COL3A1/BAZ1B/HLA-B/GTF2IRD2/NKX2-5/CITED2/ELN/MEFV/CBS/BRCC3/COL4A1/ENG
GOBP_CELL_CHEMOTAXIS	GOBP_CELL_CHEMOTAXIS	GOBP_CELL_CHEMOTAXIS	51	0.31946406	2.0717557	0.003292712	0.031580434	0.023938239	1462	tags=73%, list=46%, signal=40%	PARVA/FGFR1/PDGFRB/HMGB1/VEGFB/AIF1/FOLR2/RAC1/C3AR1/PTN/ALOX5/CCL21/ITGA9/CCL5/LYN/TRPM2/FCER1G/CXCL13/FGF1/CCR5/HSPB1/CXCR3/NRP1/CCR1/RHOG/ARRB2/CCL8/CX3CR1/XCR1/FFAR2/CCL22/PDGFRA/S100A12/OXSR1/GPSM3/S100A14/PLA2G1B
GOBP_POSITIVE_REGULATION_OF_PROTEIN_UBIQUITINATION	GOBP_POSITIVE_REGULATION_OF_PROTEIN_UBIQUITINATION	GOBP_POSITIVE_REGULATION_OF_PROTEIN_UBIQUITINATION	28	-0.353438756	-2.055985926	0.003310311	0.031580434	0.023938239	826	tags=61%, list=26%, signal=45%	RCHY1/UBE2C/TBC1D7/HSPBP1/GSK3B/TRAF6/PINK1/BIRC8/MAGEC2/MAGEA2B/UBE3A/PAXIP1/UBE2N/UBE2S/FZR1/CRY1/CDC14B
HP_WORMIAN_BONES	HP_WORMIAN_BONES	HP_WORMIAN_BONES	12	0.54370812	1.994864749	0.003301014	0.031580434	0.023938239	126	tags=50%, list=4%, signal=48%	CTSK/SETBP1/SH3PXD2B/PDGFRB/ZMPSTE24/SMO
GOBP_REGULATION_OF_NON_CANONICAL_NF_KAPPAB_SIGNAL_TRANSDUCTION	GOBP_REGULATION_OF_NON_CANONICAL_NF_KAPPAB_SIGNAL_TRANSDUCTION	GOBP_REGULATION_OF_NON_CANONICAL_NF_KAPPAB_SIGNAL_TRANSDUCTION	24	0.410173268	1.99311016	0.003296836	0.031580434	0.023938239	1249	tags=71%, list=40%, signal=43%	PHB2/PYCARD/HAVCR2/EIF2AK2/TRIP6/ILK/NMI/CYLD/IRAK1/PRDX1/EDA/RELA/AGER/LIME1/DDX3X/TRIM44/ADIPOR1
HP_STENOSIS_OF_THE_EXTERNAL_AUDITORY_CANAL	HP_STENOSIS_OF_THE_EXTERNAL_AUDITORY_CANAL	HP_STENOSIS_OF_THE_EXTERNAL_AUDITORY_CANAL	11	0.551743265	1.982119875	0.003295532	0.031580434	0.023938239	170	tags=45%, list=5%, signal=43%	MAF/MED12/CTBP1/GPRASP2/MAFB
GOBP_NEGATIVE_REGULATION_OF_CALCIUM_ION_	GOBP_NEGATIVE_REGULATION_OF_CALCIUM_ION_	GOBP_NEGATIVE_REGULATION_OF_CALCIUM_ION_TRANSPORT	11	0.551721202	1.982040615	0.003295532	0.031580434	0.023938239	622	tags=64%, list=20%, signal=51%	GSTO1/VDAC1/PPP3CA/ATP1A2/FKBP1A/BCL2/SRI

TRANSPORT	TRANSPORT										
GOBP_RESPONSE_TO_COPPER_ION	GOBP_RESPONSE_TO_COPPER_ION	GOBP_RESPONSE_TO_COPPER_ION	10	0.572626875	1.972787176	0.003322874	0.031580434	0.023938239	613	tags=70%, list=19%, signal=57%	LOXL2/NFE2L2/MUC2/MT1B/MT1E/AANAT/MT1H
HP_JOINT_CONTRACTURE_OF_THE_HAND	HP_JOINT_CONTRACTURE_OF_THE_HAND	HP_JOINT_CONTRACTURE_OF_THE_HAND	62	0.285294402	1.968024551	0.003255537	0.031580434	0.023938239	528	tags=35%, list=17%, signal=30%	FBN1/ANTXR2/EFNB1/FGFR1/SH3PXD2B/PDGFRB/OSGEP/TBC1D2B/MED12/PQBP1/ZMPSTE24/KRT14/MAFB/COL6A3/GPKOW/FBXW11/MMP23B/TPM2/DHCR24/ZDHHC9/PDXK/CTCF
HP_WEIGHT_LOSS	HP_WEIGHT_LOSS	HP_WEIGHT_LOSS	62	0.284246525	1.960796061	0.00331527	0.031580434	0.023938239	956	tags=50%, list=30%, signal=36%	RHBDF2/IFNGR1/ZMPSTE24/SMO/HAVCR2/H19/MAFB/HLA-DQB1/LIPA/STAT3/COL4A5/HLA-B/EIF4G1/BTNL2/COL4A6/TPM2/HMGCL/SLC11A1/SLC34A2/BCL2/MEFV/PTPN2/CDKN2C/GALC/DNAJC13/RRM2B/NFKBIL1/CCR1/CNTNAP1/STUB1/INS
HP_SLOPING_FOREHEAD	HP_SLOPING_FOREHEAD	HP_SLOPING_FOREHEAD	43	-0.288958528	-1.959405862	0.003282384	0.031580434	0.023938239	939	tags=58%, list=30%, signal=41%	TSEN54/TUBGCP2/NUP107/CEP290/NUP37/DMXL2/WDR73/NHEJ1/ATR/PIGP/FANCC/CDK5RAP2/MAD2L2/CEP63/TRIP13/NDE1/POMT1/CENPE/ASNS/BUB1B/KATNB1/UBE2T/SLC25A19/RAD51/WDR62
GOBP_POSITIVE_REGULATION_OF_INFLAMMATORY_RESPONSE	GOBP_POSITIVE_REGULATION_OF_INFLAMMATORY_RESPONSE	GOBP_POSITIVE_REGULATION_OF_INFLAMMATORY_RESPONSE	20	0.433517144	1.956048747	0.003271577	0.031580434	0.023938239	1403	tags=80%, list=44%, signal=45%	CASP4/PYCARD/CEBPB/ALOX5AP/HLA-E/CEBPA/NMI/IL15/MEFV/CTSC/ETS1/LTA/FFAR2/S100A12/C3/GPSM3
GOBP_ACTIN_FILAMENT_ORGANIZATION	GOBP_ACTIN_FILAMENT_ORGANIZATION	GOBP_ACTIN_FILAMENT_ORGANIZATION	106	0.225940264	1.816853708	0.003321431	0.031580434	0.023938239	1421	tags=59%, list=45%, signal=34%	MSRB2/PYCARD/IQGAP1/ABL1/EMP2/CAP1/FHOD1/HCLS1/AIF1/TMOD1/RAC1/ITGB1BP1/FCHSD1/RHOU/ITGB1/CYFIP1/CORO6/ADD1/MYOC/TRIOBP/TPM2/CCL21/TNFAIP1/TSC1/MYADM/CDC42EP5/DSTN/GMFG/ELN/BCL2/MYO1F/PHLDB2/RHOC/LIMA1/PLS3/TMOD4/FAM107A/CORO2B/MAGEL2/WNT11/ADD3/ARPC1B/NRP1/CLASP2/INPPL1/RHOG/ROCK2/TTC8/IQGAP3/DIAPH1/ACTN2/GHSR/DLC1/CTTN/CFL2/SSH3/RNH1/ANG/SPTAN1/COTL1/CAV3/F11R/RND3
GOBP_POSITIVE_REGULATION_OF_DNA_METABOLI	GOBP_POSITIVE_REGULATION_OF_DNA_METABOLI	GOBP_POSITIVE_REGULATION_OF_DNA_METABOLIC_PROCESS	64	-0.255063635	-1.9547422	0.00336129	0.031882527	0.024167229	811	tags=47%, list=26%, signal=36%	ENDOG/PARP1/ATR/MAP3K4/SIRT1/CEBPG/TIMELESS/RAD50/TP53BP1/POT1/SMARCC1/WRN/BAZ1A/CDC7/CCDC

C_PROCESS	C_PROCESS										117/BRD7/TRIM28/MAD2L2/PCNA/MAPK3/PAXIP1/RUVBL1/USP1/GFER/UBE2N/RFC4/POLG2/FAF1/RAD51/SMCHD1
HP_APLASIA_HYPOPLASIA_OF_THE_SPLEEN	HP_APLASIA_HYPOPLASIA_OF_THE_SPLEEN	HP_APLASIA_HYPOPLASIA_OF_THE_SPLEEN	11	-0.557457001	-2.105531844	0.003376722	0.031965855	0.024230392	533	tags=64%, list=17%, signal=53%	SMAD2/DNAI1/NDE1/WNT3/KATNB1/ZMYND10/FOXJ1
GOCC_CILIARY_TIP	GOCC_CILIARY_TIP	GOCC_CILIARY_TIP	12	-0.506773884	-2.029584966	0.003405368	0.032173706	0.024387944	591	tags=67%, list=19%, signal=54%	KIFAP3/IFT20/IFT74/KIF3A/CLUAP1/DYNLRB2/IFT122/DYNLL2
HP_UPPER_LIMB_JOINT_CONTRACTURE	HP_UPPER_LIMB_JOINT_CONTRACTURE	HP_UPPER_LIMB_JOINT_CONTRACTURE	77	0.259907071	1.915123323	0.003423691	0.032283396	0.02447109	845	tags=43%, list=27%, signal=32%	FBN1/PLOD1/ANTXR2/EFNB1/FGFR1/SH3PXD2B/PDGFRB/OSGEP/TBC1D2B/IGF2/MED12/PQBP1/ZMPSTE24/KRT14/MAFB/COL6A3/GPKOW/FBXW11/MMP23B/TPM2/TOR1A/DHCR24/ZDHHC9/PDXK/CTCF/SLC29A3/LGI4/LFNG/LAGE3/CDH3/PTDSS1/EMD/HSPB1
GOBP_CELLULAR_RESPONSE_TO_NITROGEN_COMPOUND	GOBP_CELLULAR_RESPONSE_TO_NITROGEN_COMPOUND	GOBP_CELLULAR_RESPONSE_TO_NITROGEN_COMPOUND	136	0.211963182	1.82423047	0.003444468	0.032415749	0.024571415	1428	tags=59%, list=45%, signal=34%	SHC1/FBN1/NFKB1/TYK2/CASP4/RGS10/IGF2/CEBPB/XBP1/PQBP1/ABL1/CYP1B1/ITPR1/PPP3CA/SOCS2/FOLR2/COL3A1/STAT3/INHBA/NFE2L2/CYFIP1/COL4A6/MAP3K5/MAS1L/CDK5/BCL2L1/PALM/CHRNB2/CYP11A1/BCL2L2/HRH3/AANAT/RAPGEF1/PTPN2/DENND4C/LYN/TRPM2/RELA/COL4A1/SIRT2/TRIM41/GCLC/AGER/GPR21/MDM2/KLF2/CRHR2/DDI2/RRAGA/INS/OTOP1/PDK2/GRB7/PRKAR1A/ROCK2/GNB1/ARRB2/SOCS1/ADIPOQ/DIAPH1/ACTN2/ATF2/GHSR/AQP1/EGR1/ADIPOR1/CSK/HTR1B/TOP2B/UMODL1/TRPV1/HCN4/CSHL1/PDGFRA/AKT1/SH2B2/CAV3/CA2/CDK2/HCN3
GOBP_MESENCHYMAL_CELL_DIFFERENTIATION	GOBP_MESENCHYMAL_CELL_DIFFERENTIATION	GOBP_MESENCHYMAL_CELL_DIFFERENTIATION	50	0.308185346	1.991626706	0.003457039	0.032470509	0.024612924	1249	tags=64%, list=40%, signal=39%	EFNB1/FGFR1/DAB2/EMP2/SMO/LOXL2/HAND2/S100A4/ANXA6/BMP4/CITED2/SNAI2/ADAM15/BMP2/FOXC1/BCL2/HAS2/NRP2/PHLDB2/LRG1/ENG/EOMES/WNT11/NRP1/CLASP2/CUL7/FGF19/ROCK2/SMAD7/TNXB/ZNF703/ADIPOR1
GOBP_REGULATION_OF_CELL_MATRIX_ADHESION	GOBP_REGULATION_OF_CELL_MATRIX_ADHESION	GOBP_REGULATION_OF_CELL_MATRIX_ADHESION	31	0.361556124	1.931296763	0.003470152	0.032482768	0.024622216	1116	tags=65%, list=35%, signal=42%	ABL1/EMP2/RAC1/ITGB1BP1/MYOC/CCL21/TSC1/ADAM15/TEK/BCL2/PHLDB2/MMP14/PLAU/FAM107A/APOD/NRP1/CLASP2/MINK1/ROCK2/CASK

GOBP_MUSCLE_TISSUE_DEVELOPMENT	GOBP_MUSCLE_TISSUE_DEVELOPMENT	GOBP_MUSCLE_TISSUE_DEVELOPMENT	83	0.25417622	1.905229052	0.003479634	0.032482768	0.024622216	1564	tags=66%, list=50%, signal=34%	PDGFRB/IGF2/ZMPSTE24/ABL1/SMO/PPP3CA/BTG2/COL3A1/HEG1/FKBP1A/ITGB1/BMP4/NKX2-5/CITED2/ZFPM2/TSC1/CDK5/BMP2/FOXC1/ELN/MEF2D/BCL2/ID2/MAML1/SIRT2/YAP1/EMD/ENG/FRS2/FOSL2/EOMES/ADPRHL1/VGLL4/PKP2/CNTNAP1/VAMP5/PRKAR1A/MYOG/ARRB2/SMAD7/ACTN2/EGR1/CFL2/HSD17B1/P2RX2/HCN4/PDGFRA/CAV3/WNT3A/ATG5/MYOD1/IRX3/GJC1/HMG20B/PPARA
GOBP_CYTOKINE_MEDIATED_SIGNALING_PATHWAY	GOBP_CYTOKINE_MEDIATED_SIGNALING_PATHWAY	GOBP_CYTOKINE_MEDIATED_SIGNALING_PATHWAY	107	0.23201355	1.875497054	0.003481209	0.032482768	0.024622216	1474	tags=64%, list=47%, signal=35%	RNF113A/TYK2/CASP4/IFITM3/LSM14A/PYCARD/IFNGR1/IFITM1/TRAF1/STAT3/INHBA/ILK/PTPRC/CEBPA/CCL21/RNF185/IL15/CYLD/CCDC3/PIAS3/IRAK1/CCL5/FOXC1/ISG15/PTPN2/FZD4/LYN/FCER1G/RELA/TRAF5/CXCL13/YAP1/CCR5/IL17A/TRIM41/CXCR3/BAD/CCR1/NR1H2/RRAGA/ACSL1/CD4/OTOP1/OAS2/DUOX2/ULK1/ADIPOR2/TNFSF13B/SOCS1/CCL8/ADIPOQ/TRIM44/UGCG/CX3CR1/EGR1/ADIPOR1/EPOR/JAGN1/XCR1/CCL22/TNFRSF4/AKT1/OXSR1/SH2B2/MT3/IL9R/OTUD4/PELI3
GOBP_REGULATION_OF_INTRACELLULAR_SIGNAL_TRANSDUCTION	GOBP_REGULATION_OF_INTRACELLULAR_SIGNAL_TRANSDUCTION	GOBP_REGULATION_OF_INTRACELLULAR_SIGNAL_TRANSDUCTION	339	0.160536006	1.691974939	0.003485362	0.032482768	0.024622216	1184	tags=45%, list=38%, signal=31%	SHC1/F10/ARHGDIB/TNFSF10/FGFR1/PDGFRB/SLA/PHB2/DAB2/IGFBP4/PYCARD/IGF2/XBP1/IQGAP1/HMGB1/ZMPSTE24/ABL1/HAVCR2/HCLS1/H19/PLEKHG3/EIF2AK2/ITPR1/TRIM8/TRAF1/PPP3CA/WWC3/COL3A1/TRIP6/RAC1/ITGB1BP1/INHBA/ILK/HEG1/MYC/GADD45A/PTPRC/ARHGEF3/NFE2L2/LMCD1/HAND2/LTBR/S100A4/FKBP1A/RHOU/ITGB1/ARHGDIA/VAPA/FBXW11/MID1/ING4/IGFBP6/HIPK2/MAP3K5/TGFA/BMP4/ATF4/PHLDA3/NMI/MYOC/SNAI2/CCL21/WFS1/CYLD/TNFAIP1/TSC1/IRAK1/VWF/MYADM/BCL2L1/PRDX1/BMP2/TEK/EDA/CCL5/BCL2L2/BCL2/PRAP1/BTN2A2/UBE2D1/MIER1/SEZ6/RHOC/PTPN2/DENND4C/CBS/LYN/UBE2B/BRCC3/RELA/TRIO/BBC3/F2RL3/TRA

HP_PNEUMOTHORAX	HP_PNEUMOTHORAX	HP_PNEUMOTHORAX	19	0.450299341	1.9896094	0.00353173	0.032851241	0.024901522	853	tags=63%, list=27%, signal=46%	F5/GNA12/RGL2/GRM1/ITGA3/PPP2R1A/SLC35B2/GNAI1/FGF1/NTF3/ENG/ARHGAP21/FRS2/WNT11/HSPB1/ERN1/AGER/NEK6/VGLL4/NET1/RRM2B/SQSTM1/MDM2/RPS15/NRP1/BAD/CCR1/MAP2K3/RRAGA/CALM3/PPIF/INS/LIME1/CD4/BAX/PDK2/DVL2/MAZ/AVPI1/ARHGEF17/MINK1/FBXO9/FGF19/PRMT6/ROCK2/DDX3X/FSHR/S100B/GPR3/ARRB2/CCL8/KALRN/SMAD7/IQGAP3/ADIPOQ/OBSCN/DYRK2/TRIM44/PJA2 FBN1/ABL1/COL3A1/BTNL2/TSC1/SLC11A1/SLC34A2/ELN/SLC31A1/LTBP1/SERPINA1/GCLC
GOCC_VESICLE_LUMEN	GOCC_VESICLE_LUMEN	GOCC_VESICLE_LUMEN	77	0.259350062	1.911019008	0.003542773	0.032890342	0.02493116	1427	tags=64%, list=45%, signal=36%	FUCA2/NFKB1/TXNDC5/PYCARD/IGF2/LGALS3BP/HMGB1/CAP1/VEGFB/GLB1/CAT/IMPDH2/APRT/PYGB/HP/CYFIP1/DERA/ALOX5/PTGES2/ACAA1/GDI2/VWF/PDXK/ALAD/GMFG/CTSH/CTSC/PGM2/TRAPPC1/SERPINA1/LRG1/FAM3C/PSMD7/INS/CREG1/HUWE1/HEBP2/DDX3X/OSTF1/ACTN2/ADA/TIMP1/NHLRC2/S100A12/SPTAN1/COTL1/C3/F8/LTF
GOCC_NUCLEAR_PORE	GOCC_NUCLEAR_PORE	GOCC_NUCLEAR_PORE	25	-0.381482407	-2.11695385	0.003579103	0.033163594	0.025138288	1312	tags=60%, list=42%, signal=35%	NUP107/NUP37/RANBP2/XPO4/RGPD5/NUP93/SNUPN/PARP11/KPNB1/TPR/RANBP1/NUP88/RAE1/NUP155/AHCTF1
GOCC_RECYCLING_ENDOSOME_MEMBRANE	GOCC_RECYCLING_ENDOSOME_MEMBRANE	GOCC_RECYCLING_ENDOSOME_MEMBRANE	22	0.414763608	1.942074933	0.003598954	0.033283407	0.025229107	653	tags=50%, list=21%, signal=40%	RAC1/HLA-B/HLA-E/HLA-F/TPCN1/HLA-A/HLA-C/EHD4/RAB11A/SLC31A1/SCAMP3
GOBP_CELLULAR_RESPONSE_TO_ORGANIC_CYCLIC_COMPOUND	GOBP_CELLULAR_RESPONSE_TO_ORGANIC_CYCLIC_COMPOUND	GOBP_CELLULAR_RESPONSE_TO_ORGANIC_CYCLIC_COMPOUND	100	0.235419327	1.872312459	0.003633765	0.033540841	0.025424244	1347	tags=58%, list=43%, signal=34%	NFKB1/MSN/KLF9/CDK4/TFPI/PHB2/DAB2/PQBP1/ABL1/SMO/CYP1B1/ATP1A2/FOLR2/INHBA/MBD2/CEBPA/HAND2/SNAI2/FECH/PRMT2/PALM/BMP2/ZBTB7A/BCL2L2/HRH3/AANAT/RAPGEF1/TRPM2/FAM107A/NR3C1/SIRT2/YAP1/GNAI1/NCOA3/GCLC/MDM2/KLF2/ROCK2/GNB1/MYOG/VPS11/ARRB2/NPAS4/DDX54/ADIPOQ/DIAPH1/GRAMD1A/ZNF703/UBE2L3/AQP1/EGR1/HTR1B/TOP2B/ELK1/VPS18/TRPV1/HCN4/SSTR3

GOBP_MITOTIC_SPINDLE_ORGANIZATION	GOBP_MITOTIC_SPINDLE_ORGANIZATION	GOBP_MITOTIC_SPINDLE_ORGANIZATION	35	-0.323334229	-2.027468872	0.003651906	0.033643707	0.025502218	520	tags=57%, list=16%, signal=48%	CHEK2/MAP1S/VCP/SPAST/BCCIP/KIF4A/SMC3/TUBG2/KPNB1/CENPH/TPX2/KIF23/TUBG1/CENPE/EFHC1/TPR/AURKC/CDCA8/RAE1/WDR62
HP_MULTICYSTIC_KIDNEY_DYSPLASIA	HP_MULTICYSTIC_KIDNEY_DYSPLASIA	HP_MULTICYSTIC_KIDNEY_DYSPLASIA	22	-0.40285477	-2.076028398	0.003662287	0.033674832	0.025525811	956	tags=68%, list=30%, signal=48%	PEX10/FIBP/PEX16/CEP290/SF3B2/ACTG2/MKKS/BBS4/SMC3/PIGN/TRIP13/IFT74/WNT3/BUB1B/LZTFL1
GOBP_PROTEIN_MODIFICATION_BY_SMALL_PROTEIN_REMOVAL	GOBP_PROTEIN_MODIFICATION_BY_SMALL_PROTEIN_REMOVAL	GOBP_PROTEIN_MODIFICATION_BY_SMALL_PROTEIN_REMOVAL	25	-0.380513076	-2.111574761	0.003671465	0.033694804	0.02554095	968	tags=68%, list=31%, signal=48%	USP20/SENP3/OTUD3/ZRANB1/VCP/USP10/USP8/ATXN3/USP31/USPL1/USP37/SENP5/USP1/USP6/USP30/SENP1/ITCH
HP_ABNORMALITY_OF_SKIN_PIGMENTATION	HP_ABNORMALITY_OF_SKIN_PIGMENTATION	HP_ABNORMALITY_OF_SKIN_PIGMENTATION	111	0.227694808	1.843196581	0.003697523	0.033869309	0.025673226	779	tags=38%, list=25%, signal=30%	RNF113A/FBN1/ANTXR2/SETBP1/CDK4/FGFR1/SH3PXD2B/PDGFRB/IGF2/MED12/TFE3/GMPPA/ZMPSTE24/SMO/COX7B/KRT14/MAFB/CAT/COL3A1/HPS6/STEAP3/BTNL2/MRAS/HPS1/SNAI2/TSC1/BMP2/EDA/CYP11A1/PPOX/AIRE/PLAG1/CTSC/CBS/CDKN2C/GALC/NR3C1/SLC29A3/DCPS/CDKN1C/MAGEL2/CDH3
HP_GRAY_MATTER_HETEROTOPIA	HP_GRAY_MATTER_HETEROTOPIA	HP_GRAY_MATTER_HETEROTOPIA	23	-0.389949287	-2.066708765	0.00372647	0.034069571	0.025825027	972	tags=70%, list=31%, signal=48%	APC2/TUBGCP2/SPEN/DCHS1/NUP37/TUBB/CDK5RAP2/CEP63/POMT1/TUBG1/CENPE/KATNB1/ARFGEF2/DHX16/PAFAH1B1/WDR62
HP_BONE_MARROW_HYPOCELLULARITY	HP_BONE_MARROW_HYPOCELLULARITY	HP_BONE_MARROW_HYPOCELLULARITY	11	-0.551781967	-2.084097072	0.003779239	0.034231462	0.025947741	692	tags=64%, list=22%, signal=50%	FANCC/MAD2L2/UBE2T/CLPB/RAD51/VPS33A/MDM4
GOCC_HISTONE_METHYLTRANSFERASE_COMPLEX	GOCC_HISTONE_METHYLTRANSFERASE_COMPLEX	GOCC_HISTONE_METHYLTRANSFERASE_COMPLEX	14	-0.481127847	-2.050131075	0.003769074	0.034231462	0.025947741	991	tags=79%, list=31%, signal=54%	C17orf49/MCRS1/ASH2L/SENP3/TAF9/SETD1A/TAF6/SUZ12/TEX10/PAXIP1/RUVBL1
GOCC_COATED_VESICLE_MEMBRANE	GOCC_COATED_VESICLE_MEMBRANE	GOCC_COATED_VESICLE_MEMBRANE	47	0.316565347	1.990572685	0.003777237	0.034231462	0.025947741	672	tags=45%, list=21%, signal=36%	COPZ1/SCARB2/DAB2/SEC22B/HLA-DRB3/HLA-DQB1/COPZ2/HLA-B/HLA-E/HLA-F/HLA-DRB5/HLA-A/HLA-C/TGFA/CLTB/IGF2R/AP1G2/FZD4/AP2M1/CNIH3/DNAJC5
GOBP_CRANIAL_NERVE_DEVELOPMENT	GOBP_CRANIAL_NERVE_DEVELOPMENT	GOBP_CRANIAL_NERVE_DEVELOPMENT	10	0.567621704	1.955543597	0.003774513	0.034231462	0.025947741	919	tags=80%, list=29%, signal=57%	MAFB/PLXNA1/NAV2/CITED2/CHRNB2/NRP2/ATP8B1/NRP1
GOBP_CELLULAR_RESPONSE_TO_CARBOHYDRATE_STIMULUS	GOBP_CELLULAR_RESPONSE_TO_CARBOHYDRATE_STIMULUS	GOBP_CELLULAR_RESPONSE_TO_CARBOHYDRATE_STIMULUS	20	0.428807495	1.934798599	0.003779768	0.034231462	0.025947741	939	tags=65%, list=30%, signal=46%	XBP1/NADK/PPP3CA/SLC29A1/KCNB1/SRI/ERN1/GCLC/AGER/BAD/OSBP/UBTF/MAP2K3

HP_SPARSE_BODY_HAIR	HP_SPARSE_BODY_HAIR	HP_SPARSE_BODY_HAIR	13	0.538166302	2.055069958	0.003836712	0.034681853	0.026289141	1101	tags=85%, list=35%, signal=55%	FGFR1/APCDD1/HS6ST1/EDA/CYP17A1/CTSC/KRT85/CDH3/PROKR2/NHLH2/KISS1R
GOBP_REGULATION_OF_REACTIVE_OXYGEN_SPECIES_METABOLIC_PROCESS	GOBP_REGULATION_OF_REACTIVE_OXYGEN_SPECIES_METABOLIC_PROCESS	GOBP_REGULATION_OF_REACTIVE_OXYGEN_SPECIES_METABOLIC_PROCESS	42	0.336386669	2.015174476	0.003851429	0.034749569	0.026340471	758	tags=48%, list=24%, signal=37%	AKR1C1/CBR1/PDGFRB/TYROBP/H19/VDAC1/CYP1B1/TSPO/RAC1/GADD45A/HP/NFE2L2/ABCB7/ALOX5/CD177/MPV17/BCL2/ARF4/IER3/SIRT2
GOCC_CILIARY_TRANSITION_ZONE	GOCC_CILIARY_TRANSITION_ZONE	GOCC_CILIARY_TRANSITION_ZONE	10	-0.544054518	-2.01199199	0.003896273	0.035088345	0.026597266	1101	tags=90%, list=35%, signal=59%	TMEM80/RP1L1/CEP290/BBS4/KIFAP3/IQCB1/IFT20/SPATA7/IFT122
GOBP_PROTEIN_DNA_COMPLEX_ORGANIZATION	GOBP_PROTEIN_DNA_COMPLEX_ORGANIZATION	GOBP_PROTEIN_DNA_COMPLEX_ORGANIZATION	147	-0.182828589	-1.816344398	0.004002499	0.035977605	0.027271333	759	tags=37%, list=24%, signal=29%	SIRT1/CBX1/ASF1B/CHD5/TAF9/SETD1A/TAF6/CGGBP1/GTF2E1/SMARCC1/TAF10/IWS1/SUPT5H/BRD9/PPHLN1/MIER2/BAZ1A/TNP2/RBM14/BRD7/BANP/TRIM28/SUZ12/ACTR6/CHD1L/PCGF6/SPHK2/CENPH/C6orf89/SMC6/PAXIP1/PHF21A/ITGB3BP/UBN1/RUVBL1/VRK1/ZMYND11/CHAF1B/BRD1/NAP1L4/CENPE/ATF7IP/SUGT1/RAD51/PRKAA1/GMNN/SFMBT1/TPR/SRPK1/DOT1L/HDGFL1/SUV39H2/SMCHD1/DR1
HP_ABSENT_SEPTUM_PELLUCIDUM	HP_ABSENT_SEPTUM_PELLUCIDUM	HP_ABSENT_SEPTUM_PELLUCIDUM	16	0.491566544	2.052100521	0.00405565	0.03631959	0.02753056	1050	tags=81%, list=33%, signal=54%	FGFR1/CTBP1/COX7B/GLI2/SSR4/DHCR24/GPR161/COL4A1/PTDSS1/STAG2/PROKR2/EHMT1/CDC42BPB
GOBP_LONG_TERM_SYNAPTIC_POTENTIATION	GOBP_LONG_TERM_SYNAPTIC_POTENTIATION	GOBP_LONG_TERM_SYNAPTIC_POTENTIATION	21	0.422064075	1.931949207	0.00405473	0.03631959	0.02753056	1209	tags=71%, list=38%, signal=44%	TYROBP/ABL1/EIF2AK4/TSHZ3/PTN/SYT12/FAM107A/AGER/SERPINE2/SQSTM1/CRHR2/INS/NFATC4/CX3CR1/RGS14
HP_MULTIPLE_CAFE_AU_LAIT_SPOTS	HP_MULTIPLE_CAFE_AU_LAIT_SPOTS	HP_MULTIPLE_CAFE_AU_LAIT_SPOTS	12	-0.499787003	-2.001603118	0.004073899	0.036415207	0.027603038	761	tags=67%, list=24%, signal=51%	CIB1/FANCC/MAD2L2/MAP2K2/TRIP13/BUB1B/UBE2T/RAD51
GOBP_REGULATION_OF_LEUKOCYTE_MEDIATED_CYTOTOXICITY	GOBP_REGULATION_OF_LEUKOCYTE_MEDIATED_CYTOTOXICITY	GOBP_REGULATION_OF_LEUKOCYTE_MEDIATED_CYTOTOXICITY	22	0.410281322	1.921087229	0.004091108	0.036501182	0.027668209	1379	tags=82%, list=44%, signal=46%	LAG3/TYROBP/HAVCR2/HLA-B/HLA-E/HLA-F/PTPRC/HLA-A/HLA-C/KIR2DL4/AGER/ITGAM/CD1A/ARRB2/CX3CR1/CD1B/STX7/TAP2
GOBP_MONOATOMIC_CATION_TRANSMEMBRANE_TRANSPORT	GOBP_MONOATOMIC_CATION_TRANSMEMBRANE_TRANSPORT	GOBP_MONOATOMIC_CATION_TRANSMEMBRANE_TRANSPORT	162	0.201557705	1.826236875	0.004150007	0.036958119	0.028014571	1589	tags=64%, list=50%, signal=33%	COX7A1/PHB2/ABL1/GSTO1/COX7B/VDAC1/COX8A/ITPR1/TMCO3/PPP3CA/SLC25A23/SLC25A5/ATP1A2/PTPRC/TPCN1/SLC30A9/SCARA5/CCDC51/FXYD5/FKBP1A/ITGB1/STEAP3/ABCB7/ANXA6/SLC25A12/KCNK5/SLC12A9/KCNE3

											/CCL21/KCNB1/SLC25A37/OTOP3/SLC11A1/SLC34A2/SLC9A8/BCL2/SLC31A1/SRI/IBTK/LYN/TRPM2/ATP2A3/F2RL3/COX15/SEC61A1/CCR5/SLC6A17/TMEM63A/CALM3/PPIF/LIME1/BAX/OTOP1/KCNH4/FHL1/SLC24A3/CNNM2/OTOP2/KCNB2/KCNJ1/NIPA2/FGF13/CABP5/KCNAB2/DIAPH1/ACTN2/ATP6V1G2/SLC6A1/AQP1/KCNJ6/KCNH7/XCR1/TRPV1/FXN/P2RX2/THY1/HCN4/OXSR1/CAV3/WNT3A/ATG5/CACNA2D3/LTF/HCN3/ATP13A1/PLA2G1B/SLC32A1/ATP6V0A1/CACNA1B/KCNQ3/SLC34A3/KCNS2/CACNA1E/SLC12A8/SLC25A13/ATP1B1/SLC12A3/CACNB3/SLC17A7/ATP1A3/GRM6/ATP2A1/TRPV5
GOMF_HEPARIN_BINDING	GOMF_HEPARIN_BINDING	GOMF_HEPARIN_BINDING	29	0.375724153	1.977885208	0.004212685	0.03730903	0.028280564	1427	tags=79%, list=45%, signal=44%	FBN1/FGFR1/VEGFB/PCOLCE/PTPRC/ADAMTS5/BMP4/PTN/PRELP/NAV2/NRP2/CXCL13/FGF1/IMPG2/SERPINE2/NRP1/ZNF146/CCL8/TNXB/REG4/ANG/F11/LTF
GOCC_PLASMA_MEMBRANE_PROTEIN_COMPLEX	GOCC_PLASMA_MEMBRANE_PROTEIN_COMPLEX	GOCC_PLASMA_MEMBRANE_PROTEIN_COMPLEX	128	0.215532225	1.823866313	0.004197726	0.03730903	0.028280564	1608	tags=65%, list=51%, signal=33%	SHC1/TYK2/CDH11/HLA-DMB/HMGB1/HLA-DRB3/TRAF1/HLA-DQB1/ATP1A2/RAC1/HLA-B/HLA-E/HLA-F/HLA-DRB5/FKBP1A/ITGB1/HLA-DMA/HLA-A/FLOT2/HLA-C/CLTB/ABHD12/GJA4/ITGA9/ITGB7/KCNB1/RAMP2/BMP2/HLA-DOB/CHRNB2/ABHD6/GRIA4/AP2M1/CNIH3/LYN/FCER1G/GRIK5/TRAF5/GNA12/GRM1/ITGA3/CDH3/ITGA5/GNAI1/C8A/CALM3/LIME1/CD4/KCNH4/ITGAM/NOXA1/DUOX2/ABCD4/CDH4/APH1A/KCNB2/ITGB8/GNB1/CDH9/GNG7/KCNAB2/SGIP1/KCNJ6/CHRNA2/CD79A/HCN4/DLG4/CAV3/WNT3A/CACNA2D3/HCN3/TSPAN32/CACNA1B/KCNQ3/GJC1/KCNS2/CACNA1E/ATP1B1/CACNB3/ATP1A3/HTR3A/EPS8/GJB1
GOMF_MONOATOMIC_ION_TRANSMEMBRANE_TRANSPORTER_ACT	GOMF_MONOATOMIC_ION_TRANSMEMBRANE_TRANSPORTER_ACTIV	GOMF_MONOATOMIC_ION_TRANSMEMBRANE_TRANSPORTER_ACTIVITY	132	0.210837255	1.793385831	0.004211267	0.03730903	0.028280564	1723	tags=70%, list=55%, signal=33%	LASP1/COX7A1/COX7B/VDAC1/COX8A/ITPR1/TMCO3/CLIC1/CLCC1/SLC25A5/ATP1A2/TPCN1/SLC30A9/CCDC51/ANXA6/SLC29A1/SLC25A12/KCNK5/SLC

IVITY	ITY										12A9/KCNE3/TMEM109/KCNB1/SLC25A37/OTOP3/SLC11A1/CHRNB2/SLC2A13/SLC34A2/SLC9A8/SLC26A1/GRIA4/SLC31A1/TRPM2/ATP2A3/GRIK5/COX15/SEC61A1/TMEM63A/CLIC5/SLC26A5/OTOP1/KCNH4/GABRR1/SLC24A3/CNNM2/OTOP2/KCNB2/KCNJ1/NIPA2/CLDN4/SLC10A5/KCNAB2/SFXN3/ATP6V1G2/SLC6A1/AQP1/HTR1B/KCNJ6/KCNH7/CHRNA2/SLC5A7/TRPV1/P2RX2/HCN4/CLCN6/CACNA2D3/HCN3/ATP13A1/SLC32A1/ATP6V0A1/CACNA1B/KCNQ3/GJC1/SLC34A3/KCNS2/CACNA1E/SLC12A8/SLC25A13/SLC12A3/CACNB3/SLC17A7/ATP1A3/ATP2A1/TRPV5/HTR3A/SLC17A8/SCN2B/SLC12A1/KCNMB3/SLC4A1/ATP7B/SLC16A1
GOBP_CELL_ADHESION_MEDIATED_BY_INTEGRIN	GOBP_CELL_ADHESION_MEDIATED_BY_INTEGRIN	GOBP_CELL_ADHESION_MEDIATED_BY_INTEGRIN	26	0.394328796	1.993824225	0.00422652	0.037313098	0.028283648	781	tags=58%, list=25%, signal=44%	FBN1/CYP1B1/ITGB1BP1/ITGB1/SNAI2/CCL21/ITGA9/ITGB7/CCL5/PODXL/LYN/PLAU/CXCL13/ITGA3/ITGA5
GOBP_REGULATION_OF_MITOTIC_CELL_CYCLE_PHASE_TRANSITION	GOBP_REGULATION_OF_MITOTIC_CELL_CYCLE_PHASE_TRANSITION	GOBP_REGULATION_OF_MITOTIC_CELL_CYCLE_PHASE_TRANSITION	79	-0.224444561	-1.838360027	0.004228663	0.037313098	0.028283648	748	tags=42%, list=24%, signal=33%	ERCC3/RAD50/ZW10/SMARCC1/DCUN1D3/E2F1/CDK5RAP2/TFDP1/ECD/CDC7/MAD2L1BP/PINX1/BRD7/ESPL1/BARD1/MAD2L2/DYNC1LI1/CDK7/BLM/TRIP13/DONSON/FBXO7/CENPE/BUB1B/FZR1/TPR/CCNH/CDCA8/HSPA2/CDC14B/CDC25C/RAD17/CPSF3
HP_ABNORMAL_MORPHOLOGY_OF_THE_ABDOMINAL_MUSCULATURE	HP_ABNORMAL_MORPHOLOGY_OF_THE_ABDOMINAL_MUSCULATURE	HP_ABNORMAL_MORPHOLOGY_OF_THE_ABDOMINAL_MUSCULATURE	12	0.533864212	1.958747456	0.004258071	0.037459082	0.028394305	436	tags=58%, list=14%, signal=50%	FBN1/IGF2/H19/COL3A1/MANBA/MRAS/COLEC11
GOBP_CYTOSOLIC_PATTERN_RECOGNITION_RECEPTOR_SIGNALING_PATHWAY	GOBP_CYTOSOLIC_PATTERN_RECOGNITION_RECEPTOR_SIGNALING_PATHWAY	GOBP_CYTOSOLIC_PATTERN_RECOGNITION_RECEPTOR_SIGNALING_PATHWAY	25	0.391094965	1.936364527	0.004268575	0.037459082	0.028394305	758	tags=56%, list=24%, signal=43%	PHB2/LSM14A/PYCARD/HMGB1/HAVCR2/EIF2AK2/PUM1/FLOT2/CYLD/IRAK1/MEFV/BRCC3/RELA/SIRT2
HP_ABNORMALITY_OF_THE_DENTITION	HP_ABNORMALITY_OF_THE_DENTITION	HP_ABNORMALITY_OF_THE_DENTITION	198	0.18672529	1.762860537	0.004262437	0.037459082	0.028394305	800	tags=35%, list=25%, signal=28%	RNF113A/FBN1/CTSK/PLOD1/ANTXR2/MAF/SETBP1/EFNB1/FGFR1/CCDC8/SH3PXD2B/PDGFRB/OSGEP/CDH11/DPH5/TBC1D2B/IGF2/MED12/PQBP1/CTBP1/ZMPSTE24/ABL1/COX7B/H19/KRT14/G

											LB1/GLI2/HLA-DQB1/CAT/PPP3CA/PERP/ATP1A2/COL3A1/PLXNA1/BAZ1B/STAT3/SSR4/MID1/TGFA/BMP4/GTF2IRD2/APCDD1/HS6ST1/CBFB/TPM2/PURA/TSC1/KCNB1/BMP2/ZDHHC9/ZBTB7A/CTCF/EDA/FOXC1/ELN/AIRE/PAX9/CTSC/CBS/PIGG/KRT85/TRIO/SLC29A3/LGI4/LAGE3/MAGEL2/CDH3/PRR12/PTDSS1
GOBP_ENDOCHONDRAL_BONE_MORPHOGENESIS	GOBP_ENDOCHONDRAL_BONE_MORPHOGENESIS	GOBP_ENDOCHONDRAL_BONE_MORPHOGENESIS	18	0.46647016	2.028984564	0.004422596	0.038717933	0.029348524	946	tags=72%, list=30%, signal=51%	ZMPSTE24/COL3A1/ANXA6/BMP4/TEK/FOXC1/MEF2D/MMP14/CBS/COL27A1/FOSL2/TMEM119/INPPL1
GOMF_OXIDOREDUCTASE_ACTIVITY_ACTING_ON_CH_OH_GROUP_OF_DONORS	GOMF_OXIDOREDUCTASE_ACTIVITY_ACTING_ON_CH_OH_GROUP_OF_DONORS	GOMF_OXIDOREDUCTASE_ACTIVITY_ACTING_ON_CH_OH_GROUP_OF_DONORS	27	0.376499746	1.923595193	0.004428127	0.038717933	0.029348524	954	tags=56%, list=30%, signal=39%	AKR1A1/AKR1C1/CBR1/CBR3/CTBP1/IMPDH2/ADHFE1/IDH3B/IDH3G/LDHA/AKR7A2/NSDHL/ADH1C/AKR7A3/HSD17B8
GOCC_SECRETORY_GRANULE_MEMBRANE	GOCC_SECRETORY_GRANULE_MEMBRANE	GOCC_SECRETORY_GRANULE_MEMBRANE	61	0.282782744	1.945443731	0.0044502	0.038840311	0.029441288	1178	tags=59%, list=37%, signal=38%	IQGAP1/TYROBP/ITPR1/PAM/RAC1/HLA-B/PTPRC/MANBA/C3AR1/VAPA/ANXA7/FLOT2/HLA-C/IGF2R/CD177/SLC11A1/PHACTR2/VPS13C/CPNE3/SRI/DNAJC5/TRPM2/PLAU/FCER1G/DNAJC13/STOM/NFASC/DCST2/TMEM63A/ITGAM/RHOG/MS4A3/MCEMP1/FCAR/KCNAB2/DIAPH1
GOBP_CELLULAR_RESPONSE_TO_VASCULAR_ENDOTHELIAL_GROWTH_FACTOR_STIMULUS	GOBP_CELLULAR_RESPONSE_TO_VASCULAR_ENDOTHELIAL_GROWTH_FACTOR_STIMULUS	GOBP_CELLULAR_RESPONSE_TO_VASCULAR_ENDOTHELIAL_GROWTH_FACTOR_STIMULUS	17	0.463322881	1.987240036	0.004473007	0.03896864	0.029538563	1375	tags=88%, list=44%, signal=50%	PDGFRB/VEGFB/ITGB1BP1/RAMP2/FOXC1/NRP2/RELA/HSPB1/ERN1/NRP1/MAP2K3/TNXB/PDGFRA/AKT1/CCBE1
GOBP_SIGNAL_TRANSDUCTION_IN_RESPONSE_TO_DNA_DAMAGE	GOBP_SIGNAL_TRANSDUCTION_IN_RESPONSE_TO_DNA_DAMAGE	GOBP_SIGNAL_TRANSDUCTION_IN_RESPONSE_TO_DNA_DAMAGE	44	-0.286786608	-1.978283935	0.004539551	0.039334599	0.029815963	787	tags=50%, list=25%, signal=38%	ATR/SIRT1/TP53BP1/USP10/E2F1/THOC5/BARD1/MAD2L2/MAPK3/CEP63/BLM/PAXIP1/DONSON/INTS7/FZR1/MDC1/RAD51/CRY1/DOT1L/CDC14B/RAD17/MDM4
GOBP_PIGMENT_BIOSYNTHETIC_PROCESS	GOBP_PIGMENT_BIOSYNTHETIC_PROCESS	GOBP_PIGMENT_BIOSYNTHETIC_PROCESS	14	0.494217728	1.931481527	0.004534281	0.039334599	0.029815963	779	tags=64%, list=25%, signal=49%	TSPO/ABCB7/TMEM14A/NFE2L1/FECH/ALAD/PPOX/COX15/CDH3
GOCC_NUCLEAR_OUTER_MEMBRANE_ENDOPLAS	GOCC_NUCLEAR_OUTER_MEMBRANE_ENDOPLASMI	GOCC_NUCLEAR_OUTER_MEMBRANE_ENDOPLASMIC_RETICULUM_	251	0.172803375	1.718913637	0.004527725	0.039334599	0.029815963	1048	tags=42%, list=33%, signal=30%	PLOD1/ANTXR2/CASP4/COPZ1/WDR81/OSBPL5/SCARB2/RHBDF2/XBP1/SEC22B/CALU/ZMPSTE24/LTC4S/TMEM129/

MIC_RETICULUM_MEMBRANE_NETWORK	C_RETICULUM_MEMBRANE_NETWORK	MEMBRANE_NETWORK									HLA-DRB3/CYP1B1/ALOX5AP/ITPR1/HLA-DQB1/CLCC1/ACSL5/RAC1/COPZ2/HLA-B/HLA-E/EXTL1/CYP2U1/MBTPS1/HLA-F/HLA-DRB5/SSR4/HM13/STT3A/BFAR/PIGW/FKBP1A/VAPA/ANXA7/HLA-A/MAP3K5/HLA-C/TGFA/PDIA6/TMEM14A/NAT8/NFE2L1/FMO1/ABHD12/MMP23B/WFS1/PTGES/RNF185/CLN3/TMEM109/EXTL2/TOR1A/DHCR24/ZDHHC9/SPCS2/CYP2W1/EDA/BCL2/DERL1/HAS2/VPS13C/SRI/RHOC/TAPBPL/CYP17A1/CNIH3/CYP4B1/PIGG/ATP2A3/PDIA5/PLA2G4C/CYP2S1/SEC61A1/SLC16A11/PTDSS1/EMD/ZDHHC6/RAB9A/ALG2/SEC23A/ILVBL/TMEM119/IFI6/ERN1/NSDHL/RNF5/FAM8A1/OSBP/CYP3A43/ACSL1/CD4/BAX/ZFYVE1/PREB/SEC63/RHOG/LCTL/ABCD4/ULK1/FMO5/APH1A
GOBP_REGULATION_OF_BIOLOGICAL_PROCESS_INVOLVED_IN_SYMBIOTIC_INTERACTION	GOBP_REGULATION_OF_BIOLOGICAL_PROCESS_INVOLVED_IN_SYMBIOTIC_INTERACTION	GOBP_REGULATION_OF_BIOLOGICAL_PROCESS_INVOLVED_IN_SYMBIOTIC_INTERACTION	11	0.542501205	1.94891807	0.00455094	0.039362357	0.029837004	172	tags=45%, list=5%, signal=43%	FUCA2/IFITM3/HMGB1/IFITM1/TRIM8
GOBP_POSITIVE_REGULATION_OF_BINDING	GOBP_POSITIVE_REGULATION_OF_BINDING	GOBP_POSITIVE_REGULATION_OF_BINDING	27	0.375129302	1.916593382	0.00461392	0.039835439	0.030195604	758	tags=52%, list=24%, signal=40%	LARP6/HMGB1/ABL1/HAND2/EIF4G1/FKBP1A/HIPK2/BMP4/ADD1/BMP2/FOXC1/DERL1/CLDN5/SIRT2
GOMF_CORECEPTOR_ACTIVITY	GOMF_CORECEPTOR_ACTIVITY	GOMF_CORECEPTOR_ACTIVITY	10	0.560498872	1.931004353	0.004677796	0.040170569	0.030449635	963	tags=80%, list=31%, signal=56%	RGMB/ITGB1/RAMP2/GPR15/CCR5/ENG/NRP1/CD4
GOBP_ANTIGEN_PROCESSING_AND_PRESENTATION_OF_EXOGENOUS_PEPTIDE_ANTIGEN_VIA_MHC_CLASS_II	GOBP_ANTIGEN_PROCESSING_AND_PRESENTATION_OF_EXOGENOUS_PEPTIDE_ANTIGEN_VIA_MHC_CLASS_II	GOBP_ANTIGEN_PROCESSING_AND_PRESENTATION_OF_EXOGENOUS_PEPTIDE_ANTIGEN_VIA_MHC_CLASS_II	10	0.560362783	1.930535505	0.004677796	0.040170569	0.030449635	701	tags=70%, list=22%, signal=55%	HLA-DMB/HLA-DRB3/HLA-DQB1/HLA-DRB5/HLA-DMA/HLA-DOB/FCER1G
GOBP_LYMPHOCYTE_MIGRATION	GOBP_LYMPHOCYTE_MIGRATION	GOBP_LYMPHOCYTE_MIGRATION	20	0.422997485	1.908583573	0.004669107	0.040170569	0.030449635	1415	tags=85%, list=45%, signal=47%	MSN/PYCARD/ABL1/AIF1/CCL21/ITGB7/CCL5/AIRE/CXCL13/GPR15/APOD/SPN/CCL8/CCL22/AKT1/OXSR1/F11R
GOBP_FAT_CELL	GOBP_FAT_CELL_	GOBP_FAT_CELL_DIFF	50	0.300152489	1.93971492	0.00471958	0.04045716	0.03066687	1407	tags=66%,	LRP3/CEBPB/TFE3/XBP1/CTBP1/JDP2/

_DIFFERENTIATION	DIFFERENTIATION	ERENTIATION				2	7	9		list=45%, signal=37%	MAFB/CEBPA/ALOX5/ZFPM2/SNAI2/CDC3/BMP2/EBF2/ZBTB7A/ID2/TRIO/SIRT2/YAP1/LRG1/FOSL2/INS/FBXO9/TTC8/SOCS1/ADIPOQ/ATF2/FFAR2/PDGFRA/AKT1/ZBTB16/SH2B2/WNT3A
GOBP_ACTIN_FILAMENT_BUNDLE_ORGANIZATION	GOBP_ACTIN_FILAMENT_BUNDLE_ORGANIZATION	GOBP_ACTIN_FILAMENT_BUNDLE_ORGANIZATION	39	0.316657772	1.841104547	0.004750429	0.040649135	0.030812392	923	tags=51%, list=29%, signal=37%	ABL1/FHOD1/AIF1/RAC1/ITGB1BP1/ITGB1/ADD1/MYOC/TNFAIP1/TSC1/ELN/PHLDB2/RHOC/LIMA1/PLS3/FAM107A/CORO2B/WNT11/NRP1/CLASP2
GOBP_RESPONSE_TO_SALT	GOBP_RESPONSE_TO_SALT	GOBP_RESPONSE_TO_SALT	78	0.249318154	1.836030589	0.004795273	0.040959975	0.031048011	1338	tags=59%, list=42%, signal=35%	TOMM20/DAB2/RGS10/IQGAP1/ALOX5AP/ITPR1/CAT/PPP3CA/SLC25A23/TSPO/CEBPA/SLC25A12/ENTPD6/ADD1/CDK5/KCNB1/ALAD/CHRNB2/HRH3/AANAT/CPNE3/SYT12/TRPM2/ALG2/GCLC/MDM2/CALM3/SLC26A5/PPIF/ROCK2/GNB1/MYOG/NFATC4/GDI1/PITX3/ADIPOQ/GHSR/SLC6A1/EGR1/TSHB/HTR1B/TOP2B/CHRNA2/JUND/TRPV1/P2RX2
GOCC_REPLICATION_FORK	GOCC_REPLICATION_FORK	GOCC_REPLICATION_FORK	17	-0.443540662	-2.088537702	0.004832679	0.041206298	0.031234726	866	tags=71%, list=27%, signal=51%	RFC1/PARP1/TIMELESS/TP53BP1/WRN/TEX264/PCNA/PRIM1/BLM/POLA2/DONSON/RFC4
GOBP_TYPE_II_INTERFERON_PRODUCTION	GOBP_TYPE_II_INTERFERON_PRODUCTION	GOBP_TYPE_II_INTERFERON_PRODUCTION	18	0.464034161	2.018388807	0.00486803	0.041243579	0.031262986	823	tags=61%, list=26%, signal=45%	TYK2/PYCARD/HMGB1/ABL1/HAVCR2/INHBA/HLA-A/SLC11A1/ISG15/SCGB1A1/CD276
GOBP_RESPONSE_TO_TUMOR_CELL	GOBP_RESPONSE_TO_TUMOR_CELL	GOBP_RESPONSE_TO_TUMOR_CELL	10	0.558698899	1.924803169	0.004871356	0.041243579	0.031262986	711	tags=70%, list=23%, signal=54%	HMGB1/HAVCR2/HLA-DRB3/RBMS3/HLA-A/ADAM15/RELA
GOBP_POSITIVE_REGULATION_OF_CELL_MATRIX_ADHESION	GOBP_POSITIVE_REGULATION_OF_CELL_MATRIX_ADHESION	GOBP_POSITIVE_REGULATION_OF_CELL_MATRIX_ADHESION	14	0.492034771	1.92295018	0.004851358	0.041243579	0.031262986	507	tags=57%, list=16%, signal=48%	ABL1/EMP2/RAC1/ITGB1BP1/MYOC/CCL21/TSC1/TEK
GOBP_REGULATION_OF_EXTRINSIC_APOPTOTIC_SIGNALING_PATHWAY	GOBP_REGULATION_OF_EXTRINSIC_APOPTOTIC_SIGNALING_PATHWAY	GOBP_REGULATION_OF_EXTRINSIC_APOPTOTIC_SIGNALING_PATHWAY	34	0.343476767	1.919011542	0.004860494	0.041243579	0.031262986	919	tags=56%, list=29%, signal=40%	TNFSF10/FGFR1/PYCARD/TRAF1/INHBA/PTPRC/LTBR/BMP4/SRPX/SNAI2/CYLD/BCL2L1/BCL2/RELA/YAP1/PPP2R1A/IFI6/GCLC/NRP1
GOBP_TORC1_SIGNALING	GOBP_TORC1_SIGNALING	GOBP_TORC1_SIGNALING	23	-0.376134576	-1.993491593	0.004916621	0.041480755	0.031442767	1181	tags=78%, list=37%, signal=49%	NPC1/SYK/RRAGC/RPS6KB1/EP300/WDR24/FLCN/KPTN/CSNK1A1/TBC1D7/YWHAZ/ATXN3/TBK1/MAPK3/UBE3A/UBE2N/RRAGD/PRKAA1
GOCC_AZUROPHI	GOCC_AZUROPHI	GOCC_AZUROPHIL_GR	36	0.33721614	1.900210703	0.00491590	0.04148075	0.03144276	792	tags=47%,	FUCA2/TXNDC5/PYCARD/CAP1/GLB1/

L_GRANULE	L_GRANULE	ANULE				7	5	7		list=25%, signal=36%	PYGB/MANBA/C3AR1/VAPA/PTGES2/GDI2/CPNE3/CTSC/DNAJC5/TRAPPC1/DNAJC13/STOM
GOMF_UBIQUITIN_LIKE_PROTEIN_PEPTIDASE_ACTIVITY	GOMF_UBIQUITIN_LIKE_PROTEIN_PEPTIDASE_ACTIVITY	GOMF_UBIQUITIN_LIKE_PROTEIN_PEPTIDASE_ACTIVITY	22	-0.392195538	-2.021098259	0.004953496	0.041700985	0.031609704	968	tags=68%, list=31%, signal=48%	USP20/SENP3/OTUD3/ZRANB1/USP10/USP8/ATXN3/USP31/USPL1/USP37/SENP5/USP1/USP6/USP30/SENP1
HP_ABNORMAL_SYSTEMIC_BLOOD_PRESSURE	HP_ABNORMAL_SYSTEMIC_BLOOD_PRESSURE	HP_ABNORMAL_SYSTEMIC_BLOOD_PRESSURE	112	0.223617523	1.82085499	0.004960067	0.041700985	0.031609704	732	tags=37%, list=23%, signal=29%	FBN1/OSGEP/HTRA1/MED12/MGP/GMPPA/ZMPSTE24/H19/CFHR3/MAFB/GLI2/IMPDH2/COL3A1/LIPA/BAZ1B/APRT/COL4A5/HLA-B/SLC30A9/EIF4G1/BTNL2/GTF2IRD2/NKX2-5/ADD1/HMGCL/TSC1/IRAK1/EDA/ELN/CYP11A1/PPOX/LARS2/MMP14/CYP17A1/CBS/SERPINA6/CDKN2C/BRCC3/SLC25A20/DNAJC13/NR3C1
GOBP_HOMEOSTASIS_OF_NUMBER_OF_CELLS	GOBP_HOMEOSTASIS_OF_NUMBER_OF_CELLS	GOBP_HOMEOSTASIS_OF_NUMBER_OF_CELLS	65	0.27207298	1.902129971	0.005007153	0.042023387	0.031854087	1391	tags=65%, list=44%, signal=37%	HMGB1/ABL1/SMO/HCLS1/GPRASP2/MAFB/ARMCX1/SLC25A5/LIPA/STAT3/INHBA/HIPK2/BMP4/MTHFD1/MUC2/CITED2/FECH/PRDX1/ZBTB7A/ISG15/BCL2/ID2/PTPN2/LYN/OCIAD2/PPP2R1A/FOSL2/WDR37/KLF2/BAX/HEATR3/TNFSF13B/ALAS1/ETS1/CX3CR1/TSC22D3/ADA/OCIAD1/MFHAS1/EMX1/AKT1/SH2B2
GOBP_POSITIVE_REGULATION_OF_PROGRAMMED_CELL_DEATH	GOBP_POSITIVE_REGULATION_OF_PROGRAMMED_CELL_DEATH	GOBP_POSITIVE_REGULATION_OF_PROGRAMMED_CELL_DEATH	111	0.223106989	1.806058046	0.00508031	0.042563084	0.032263182	966	tags=44%, list=31%, signal=32%	CTSK/ALDH1A3/TNFSF10/HTRA1/PYCARD/HMGB1/ABL1/H19/CYP1B1/CD248/TSPO/INHBA/MYC/GADD45A/PTPRC/LTBR/ITGB1/ING4/MAP3K5/BMP4/ATF4/NQO1/PHLDA3/SRPX/TFPT/IGF2R/CYLD/CDK5/PRMT2/BMP2/CCL5/CTSH/BCL2/ARHGEF7/PTPN2/CTSC/LYN/BBC3/NR3C1/SIRT2/PPP2R1A/ADM/WNT11/MAGED1/NET1/SQSTM1/BAD/OMA1/BAX
GOBP_RESPONSE_TO_INORGANIC_SUBSTANCE	GOBP_RESPONSE_TO_INORGANIC_SUBSTANCE	GOBP_RESPONSE_TO_INORGANIC_SUBSTANCE	129	0.210591597	1.781220316	0.005090845	0.042577172	0.032273861	951	tags=43%, list=30%, signal=31%	CYBRD1/MAOB/IQGAP1/ABL1/H19/CYP1B1/ALOX5AP/CUTA/CAT/PPP3CA/PLSCR1/SLC25A23/PAM/TSPO/LOXL2/CEBPA/ACO1/HP/NFE2L2/MAP3K5/SLC25A12/ATF4/ENTPD6/NQO1/MUC2/ADD1/MT1B/FECH/KCNB1/MT1E/PRDX1/CCS/ALAD/SLC11A1/BCL2/AANAT/MT1H/

											CPNE3/SYT12/TRPM2/SCGB1A1/RELA/PRDX3/ANKZF1/ALG2/ERN1/GCLC/NET1/LTA4H/MDM2/KLF2/DDI2/CALM3/SLC26A5/PPIF
GOBP_FAT_SOLUBLE_VITAMIN_METABOLIC_PROCESS	GOBP_FAT_SOLUBLE_VITAMIN_METABOLIC_PROCESS	GOBP_FAT_SOLUBLE_VITAMIN_METABOLIC_PROCESS	13	0.52791908	2.015939379	0.005105021	0.042621604	0.032307541	573	tags=69%, list=18%, signal=57%	NFKB1/CBR1/LIPA/PLTP/RBP1/NQO1/SNAI2/CYP2W1/CYP11A1
HP_SHORT_CHIN	HP_SHORT_CHIN	HP_SHORT_CHIN	16	0.483860556	2.019930998	0.005124562	0.0427106	0.032375001	142	tags=44%, list=5%, signal=42%	NFIA/IGF2/MED12/ZMPSTE24/ABL1/COX7B/H19
GOBP_REGULATION_OF_CELL_KILLING	GOBP_REGULATION_OF_CELL_KILLING	GOBP_REGULATION_OF_CELL_KILLING	24	0.398184122	1.934852614	0.005145484	0.042810787	0.032450943	1379	tags=79%, list=44%, signal=45%	LAG3/TYROBP/HAVCR2/HLA-B/HLA-E/HLA-F/PTPRC/HLA-A/HLA-C/BCL2L1/KIR2DL4/AGER/ITGAM/CD1A/ARRB2/CX3CR1/CD1B/STX7/TAP2
GOBP_ANTIGEN_PROCESSING_AND_PRESENTATION_OF_EXOGENOUS_ANTIGEN	GOBP_ANTIGEN_PROCESSING_AND_PRESENTATION_OF_EXOGENOUS_ANTIGEN	GOBP_ANTIGEN_PROCESSING_AND_PRESENTATION_OF_EXOGENOUS_ANTIGEN	16	0.48157325	2.010382379	0.005187439	0.043040491	0.03262506	701	tags=62%, list=22%, signal=49%	HLA-DMB/HLA-DRB3/HLA-DQB1/HLA-E/HLA-F/HLA-DRB5/HLA-DMA/HLA-A/HLA-DOB/FCER1G
GOBP_BIOMINERAL_TISSUE_DEVELOPMENT	GOBP_BIOMINERAL_TISSUE_DEVELOPMENT	GOBP_BIOMINERAL_TISSUE_DEVELOPMENT	33	0.347444935	1.90923463	0.005190993	0.043040491	0.03262506	1119	tags=61%, list=35%, signal=40%	CEBPB/MGP/ZMPSTE24/ITGB1BP1/BMP4/PTN/ATF4/ALOX5/BMP2/ISG15/FOSL2/WNT11/TMEM119/KLF10/CCR1/OTOP1/SLC24A3/ROCK2/FAM20C/NOTUM
GOCC_SITE_OF_DOUBLE_STRAND_BREAK	GOCC_SITE_OF_DOUBLE_STRAND_BREAK	GOCC_SITE_OF_DOUBLE_STRAND_BREAK	22	-0.390440273	-2.012052862	0.005231254	0.043151115	0.032708914	841	tags=59%, list=27%, signal=44%	VCP/NHEJ1/PARP1/TIMELESS/RAD50/TP53BP1/ARPC2/MAD2L2/CHD1L/RNF138/SMC6/RAD51/SMCHD1
GOBP_NEGATIVE_REGULATION_OF_MITOTIC_CELL_CYCLE_PHASE_TRANSITION	GOBP_NEGATIVE_REGULATION_OF_MITOTIC_CELL_CYCLE_PHASE_TRANSITION	GOBP_NEGATIVE_REGULATION_OF_MITOTIC_CELL_CYCLE_PHASE_TRANSITION	42	-0.293316563	-1.961663087	0.005218208	0.043151115	0.032708914	728	tags=48%, list=23%, signal=37%	RAD50/ZW10/DCUN1D3/CDK5RAP2/MAD2L1BP/PINX1/BRD7/BARD1/MAD2L2/DYNC1LI1/BLM/TRIP13/DONSON/FBXO7/BUB1B/FZR1/TPR/CDCA8/CDC14B/RAD17
GOBP_DEFENSE_RESPONSE_TO_OTHER_ORGANISM	GOBP_DEFENSE_RESPONSE_TO_OTHER_ORGANISM	GOBP_DEFENSE_RESPONSE_TO_OTHER_ORGANISM	209	0.182463048	1.749834267	0.005222901	0.043151115	0.032708914	1013	tags=42%, list=32%, signal=30%	SHC1/NFKB1/TYK2/CASP4/LAG3/IFITM3/PHB2/LSM14A/PYCARD/IFIT3/CEBPB/PQBP1/HMGB1/IFNGR1/ZMPSTE24/TYROBP/EIF2AK4/IFITM1/HAVCR2/AIF1/RAB1A/EIF2AK2/TRIM8/MAPKAPK3/PLSCR1/C1QC/PUM1/HLA-B/HLA-E/HLA-F/STAB1/PTPRC/HP/NFE2L2/ADAMTS5/TFEB/HLA-A/FLOT2/MAP3K5/HLA-C/NQO1/CFHR5/NMI/CCL21/COLE

											C11/RNF185/IL15/CYLD/CD177/ADAM15/IRAK1/BCL2L1/PRDX1/FGL2/CCL5/SLC11A1/ISG15/LY86/BCL2/MEFV/TRIM4/PTPN2/LYN/FCER1G/BRCC3/RELA/PI3/CXCL13/SIRT2/KIR2DL4/FOSL2/IL17A/TRIM41/C8A/IFI6/BATF2/ZNFX1/NFKBIL1/NR1H2/INS/CD4/OTOP1/TRAFD1/SPN/ITGAM/OAS2/MMRN2
GOBP_SCHWANN_CELL_DIFFERENTIATION	GOBP_SCHWANN_CELL_DIFFERENTIATION	GOBP_SCHWANN_CELL_DIFFERENTIATION	12	0.525895034	1.929508546	0.005314317	0.043757782	0.033168773	935	tags=75%, list=30%, signal=53%	MED12/ILK/MYOC/CDK5/PRX/RELA/LGI4/SIRT2/CNTNAP1
GOBP_T_CELL_SELECTION	GOBP_T_CELL_SELECTION	GOBP_T_CELL_SELECTION	10	0.554235818	1.909427174	0.005322999	0.043757782	0.033168773	977	tags=80%, list=31%, signal=55%	STAT3/PTPRC/IL15/BCL2/AIRE/PTPN2/CD4/SPN
HP_HEMIPLEGIA_HEMIPARESIS	HP_HEMIPLEGIA_HEMIPARESIS	HP_HEMIPLEGIA_HEMIPARESIS	47	0.304805818	1.916628407	0.005374088	0.043952363	0.033316267	930	tags=53%, list=29%, signal=38%	FGFR1/PDGFRB/OSGEP/HTRA1/COL4A2/IFNGR1/SMO/CFHR3/HLA-DQB1/ATP1A2/COL3A1/HLA-B/MMP23B/HMGCL/TSC1/IRAK1/MEFV/CBS/GALC/COL4A1/LAGE3/ENG/PROKR2/RRM2B/CCR1
GOMF_IMMUNE_RECEPTOR_ACTIVITY	GOMF_IMMUNE_RECEPTOR_ACTIVITY	GOMF_IMMUNE_RECEPTOR_ACTIVITY	25	0.384164549	1.902051094	0.005374088	0.043952363	0.033316267	1719	tags=88%, list=55%, signal=40%	IFNGR1/HLA-DRB3/HLA-DQB1/C3AR1/HLA-DOB/CTSH/FZD4/FCER1G/KIR2DL4/CCR5/CXCR3/CCR1/CD4/CX3CR1/EPOR/XCR1/IL9R/IL1R1/CR2/IL2RG/IL13RA1/IL21R
GOBP_REGULATION_OF_PROTEIN_BINDING	GOBP_REGULATION_OF_PROTEIN_BINDING	GOBP_REGULATION_OF_PROTEIN_BINDING	30	0.356805103	1.894204003	0.005356322	0.043952363	0.033316267	966	tags=60%, list=31%, signal=42%	DAB2/ABL1/ITGB1BP1/FKBP1A/HIPK2/BMP4/CFHR5/ADD1/GNL3L/ADAM15/BMP2/MITD1/DERL1/ATP2A3/CLDN5/PRMT8/NRP1/BAX
GOMF_SIGNALING_RECEPTOR_REGULATOR_ACTIVITY	GOMF_SIGNALING_RECEPTOR_REGULATOR_ACTIVITY	GOMF_SIGNALING_RECEPTOR_REGULATOR_ACTIVITY	80	0.256326392	1.906131937	0.005397229	0.044066681	0.033402922	1411	tags=61%, list=45%, signal=35%	FBN1/TNFSF10/IGF2/HMGB1/RABEP2/OGN/VEGFB/INHBA/S100A4/FKBP1A/TGFA/BMP4/PTN/CCL21/IL15/CDK5/GHRH/BMP2/EDA/CCL5/GMFG/LYNX1/FNTA/FLT3LG/CXCL13/APLN/FGF1/NTF3/FAM3C/IL17A/ADM/WNT11/SLURP1/UCN3/INS/FGF19/GUCA2A/FGF13/TNFSF13B/CCL8/WNT2B/ADIPOQ/TSHB/LTA/TIMP1/CCL22/CSHL1/WNT3A/LTB
GOBP_MOLTING_CYCLE	GOBP_MOLTING_CYCLE	GOBP_MOLTING_CYCLE	25	0.383787306	1.900183311	0.005435506	0.044303977	0.033582793	1009	tags=56%, list=32%, signal=38%	ZMPSTE24/SMO/KRT14/GLI2/INHBA/APCDD1/DNASE1L2/EDA/BCL2/RELA/CDH3/NSDHL/KRT27/PTCH2
GOBP_NUCLEAR_EXPORT	GOBP_NUCLEAR_EXPORT	GOBP_NUCLEAR_EXPORT	39	-0.291753846	-1.90391332	0.005460349	0.044431163	0.033679202	752	tags=54%, list=24%,	XPO1/NUP107/PABPN1/RANBP2/XPO4/GSK3B/IWS1/SMG7/AKAP8L/NUP93/T

										signal=42%	HOC5/NOL6/BARD1/CSE1L/CCHCR1/FRAT1/TPR/NUP88/RAE1/NUP155/NCBP2
GOBP_POSITIVE_REGULATION_OF_MIRNA_TRANSCRIPTION	GOBP_POSITIVE_REGULATION_OF_MIRNA_TRANSCRIPTION	GOBP_POSITIVE_REGULATION_OF_MIRNA_TRANSCRIPTION	10	0.553701832	1.907587509	0.00551656	0.044757585	0.033926632	1233	tags=90%, list=39%, signal=55%	STAT3/MYC/BMP2/RELA/NR3C1/APLN/NR1H2/ETS1/EGR1
GOBP_NEUROTRANSMITTER_TRANSPORT	GOBP_NEUROTRANSMITTER_TRANSPORT	GOBP_NEUROTRANSMITTER_TRANSPORT	36	0.333300351	1.878145257	0.005519078	0.044757585	0.033926632	1670	tags=83%, list=53%, signal=40%	ATP1A2/ITGB1/SLC29A1/CDK5/TOR1A/RPH3AL/HRH3/FER1L4/DNAJC5/SYT12/KMO/GRIK5/SLC29A3/SLC6A17/SLC29A2/CASK/SLC6A1/HTR1B/SLC5A7/VPS18/SLC22A2/SLC32A1/STX3/CLN8/SLC17A7/CPLX2/SLC17A8/NF1/SYP/SV2A
GOBP_NEGATIVE_REGULATION_OF_MONOATOMIC_ION_TRANSMEMBRANE_TRANSPORT	GOBP_NEGATIVE_REGULATION_OF_MONOATOMIC_ION_TRANSMEMBRANE_TRANSPORT	GOBP_NEGATIVE_REGULATION_OF_MONOATOMIC_ION_TRANSMEMBRANE_TRANSPORT	17	0.455602018	1.954124452	0.005599062	0.044853027	0.033998978	1405	tags=82%, list=45%, signal=46%	GSTO1/VDAC1/PPP3CA/ATP1A2/FKBP1A/KCNE3/BCL2/SRI/CALM3/SLC26A5/PPIF/ACTN2/OXSR1/CAV3
GOBP_REGULATION_OF_INFLAMMATORY_RESPONSE	GOBP_REGULATION_OF_INFLAMMATORY_RESPONSE	GOBP_REGULATION_OF_INFLAMMATORY_RESPONSE	60	0.279999211	1.915444415	0.005567176	0.044853027	0.033998978	1403	tags=65%, list=44%, signal=37%	NFKB1/CASP4/RHBDF2/PYCARD/CEBPB/ALOX5AP/STAT3/HLA-E/PTPRC/CEBPA/ALOX5/NMI/ABHD12/PTGES/IL15/CYLD/TEK/CCL5/MEFV/PTPN2/CTSC/LYN/SCGB1A1/RELA/IER3/IL17A/AGER/INS/SPN/ETS1/ADIPOQ/GHSR/ADA/LTA/MFHAS1/FFAR2/S100A12/C3/GPSM3
GOMF_CYTOKINE_RECEPTOR_BINDING	GOMF_CYTOKINE_RECEPTOR_BINDING	GOMF_CYTOKINE_RECEPTOR_BINDING	44	0.310244975	1.886288655	0.005555271	0.044853027	0.033998978	813	tags=48%, list=26%, signal=36%	SHC1/TYK2/TNFSF10/PYCARD/VEGFB/TRAF1/SOCS2/TRIP6/FKBP1A/TGFBRAP1/CCL21/IL15/EDA/CCL5/TRAF5/CXCL13/ITGA5/NTF3/LRG1/ENG/FRS2
GOBP_CALCIUM_ION_TRANSMEMBRANE_TRANSPORT	GOBP_CALCIUM_ION_TRANSMEMBRANE_TRANSPORT	GOBP_CALCIUM_ION_TRANSMEMBRANE_TRANSPORT	62	0.270942875	1.86902451	0.005585135	0.044853027	0.033998978	1620	tags=71%, list=51%, signal=35%	ABL1/GSTO1/VDAC1/ITPR1/PPP3CA/SLC25A23/ATP1A2/PTPRC/TPCN1/FKBP1A/ANXA6/CCL21/BCL2/SRI/IBTK/LYN/TRPM2/ATP2A3/F2RL3/SEC61A1/CCR5/CALM3/LIME1/BAX/SLC24A3/CABP5/DIAPH1/XCR1/TRPV1/P2RX2/THY1/CAV3/WNT3A/ATG5/CACNA2D3/PLA2G1B/CACNA1B/CACNA1E/ATP1B1/CACNB3/GRM6/ATP2A1/TRPV5/BDKRB1
HP_ABNORMAL_SPUTUM	HP_ABNORMAL_SPUTUM	HP_ABNORMAL_SPUTUM	23	0.392319743	1.865053842	0.005595458	0.044853027	0.033998978	930	tags=65%, list=29%,	FBN1/IFNGR1/EIF2AK4/COL3A1/HLA-B/BTNL2/TSC1/SLC11A1/SLC34A2/ELN

										signal=46%	/MEFV/SERPINA1/ENG/GCLC/CCR1
HP_ABSENT_SPEECH	HP_ABSENT_SPEECH	HP_ABSENT_SPEECH	89	0.23290164	1.782788783	0.005605156	0.044853027	0.033998978	1180	tags=53%, list=37%, signal=34%	SETBP1/RALA/FGFR1/DPH5/MED12/TFE3/EIF2AK2/TRIM8/GLI2/PPP3CA/ATP1A2/RAC1/PIGW/SLC25A12/BMP4/MMP23B/PURA/AMPD2/TUBA8/KCNB1/TOR1A/GRIA4/PIGG/TRIO/PRPS1/DCPS/GRM1/MAGEL2/PPP2R1A/SLC35B2/GNAI1/STAG2/WDR37/SLC6A17/ACOX1/UBTF/CNNM2/HUWE1/SLITRK2/UBE3B/FGF13/SMC1A/PYCR2/VPS11/CASK/KCNAB2/VPS4A
GOBP_ORGANIC_ACID_METABOLIC_PROCESS	GOBP_ORGANIC_ACID_METABOLIC_PROCESS	GOBP_ORGANIC_ACID_METABOLIC_PROCESS	204	0.179309886	1.706673539	0.005605462	0.044853027	0.033998978	1060	tags=43%, list=34%, signal=30%	ALDH1A3/AKR1A1/AKR1C1/CBR1/PYCARD/XBP1/PGK1/HYI/GSTO1/LTC4S/VDAC1/CYP1B1/ALOX5AP/OSBPL1A/GCSH/LIPA/STAT3/ACSL5/ECHDC1/CYP2U1/ADHFE1/ACO1/ADI1/GNE/RBP1/SLC25A12/AIG1/ATF4/MTHFD1/ALOX5/DHTKD1/FMO1/ABHD12/PTGES2/ACAA1/PIPOX/BPHL/PTGES/HMGCL/ACY1/CLN3/SUCLG2/ZBTB7A/CYP2W1/IDH3B/IDH3G/BCAT2/HNMT/ABHD6/LARS2/ASNSD1/STARD4/UGT2A3/CBS/OAT/CYP4B1/KMO/PRKAB2/IER3/SIRT2/PLA2G4C/CYP2S1/GNPDA1/ILVBL/LDHA/GCLC/ATP8B1/SCP2/ADH1C/LTA4H/ECH1/ACOX1/NR1H2/OSBP/FADS6/NUDT8/HSD17B8/ACAD11/INS/ACSL1/PDK2/DAO/ACOT12/ABCD4/ABCC10/FGF19/ADIPOR2
HP_ABNORMAL_LUNG_MORPHOLOGY	HP_ABNORMAL_LUNG_MORPHOLOGY	HP_ABNORMAL_LUNG_MORPHOLOGY	225	0.174929744	1.701396599	0.005563568	0.044853027	0.033998978	603	tags=29%, list=19%, signal=25%	RNF113A/FBN1/NFKB1/TYK2/PLOD1/MSN/SETBP1/SGSH/FGFR1/PDGFRB/SCARB2/MED12/MGP/IFNGR1/CTBP1/ZMPSTE24/ABL1/EMP2/EIF2AK4/SMO/H19/ITPR1/GLB1/GLI2/HLA-DQB1/PERP/COL3A1/BAZ1B/STAT3/COL4A5/RAC1/HLA-B/COL6A3/GPKOW/MANBA/HPS6/NFE2L2/ARHGDIA/BTNL2/MID1/COL4A6/MRAS/GTF2IRD2/NKX2-5/MTHFD1/HPS1/CITED2/ZFPM2/SNAI2/TPM2/PURA/TSC1/IRAK1/BMP2/DHCR24/TBC1D23/ZBTB7A/CTCF/EDA/SLC11A1/SLC34A2/ELN/BCL2/MEFV/SLC31A1

GOBP_DENDRITIC_CELL_DIFFERENTIATION	GOBP_DENDRITIC_CELL_DIFFERENTIATION	GOBP_DENDRITIC_CELL_DIFFERENTIATION	11	0.532934783	1.914551011	0.005618042	0.044879007	0.034018672	1067	tags=82%, list=34%, signal=54%	CEBPB/HMGB1/HLA-B/LTBR/LYN/TRPM2/AGER/BATF2/ITGB8
GOBP_OLEFINIC_COMPOUND_METABOLIC_PROCESS	GOBP_OLEFINIC_COMPOUND_METABOLIC_PROCESS	GOBP_OLEFINIC_COMPOUND_METABOLIC_PROCESS	40	0.320227598	1.874494875	0.005638459	0.044906553	0.034039552	884	tags=50%, list=28%, signal=36%	ALDH1A3/AKR1C1/CBR1/DAB2/CYP1B1/CYP2U1/ALOX5/ABHD12/PTGES2/ACAA1/BMP2/ABHD6/CYP11A1/PNPLA4/CYP17A1/PLA2G4C/CYP2S1/ADM/SCP2/ADH1C
GOBP_NON_MEMBRANE_BOUNDED_ORGANELLE_ASSEMBLY	GOBP_NON_MEMBRANE_BOUNDED_ORGANELLE_ASSEMBLY	GOBP_NON_MEMBRANE_BOUNDED_ORGANELLE_ASSEMBLY	89	-0.212279476	-1.804556125	0.005640166	0.044906553	0.034039552	703	tags=44%, list=22%, signal=35%	OGFOD1/FLII/EIF5B/LSM3/BCCIP/CNOT6/BOP1/KIAA0753/USP10/TUBB/CDK5RAP2/KIF4A/CNOT7/ACTL8/SMC3/RBM14/CAPN3/CSRP2/MYOM2/KPNB1/CEP63/CENPH/TPX2/EIF2S1/KIF23/NIP7/CENPE/CEP72/EDC3/SUGT1/DHX30/PRKAA1/TPR/EIF5/AURKC/CDCA8/SPAG5/CCNB2/WDR62
HP_AORTIC_ANEURYSM	HP_AORTIC_ANEURYSM	HP_AORTIC_ANEURYSM	38	0.338600094	1.958431647	0.005658965	0.044928602	0.034056265	462	tags=39%, list=15%, signal=34%	FBN1/PLOD1/DPH5/MED12/ZMPSTE24/ABL1/COL3A1/BAZ1B/HLA-B/MID1/GTF2IRD2/NKX2-5/MMP23B/TPM2/TSC1
HP_KERATITIS	HP_KERATITIS	HP_KERATITIS	17	0.454928726	1.951236633	0.005661621	0.044928602	0.034056265	596	tags=53%, list=19%, signal=43%	RNF113A/FBN1/IFNGR1/COL4A5/HLA-B/BTNL2/COL4A6/MEFV/AIRE
HP_ABNORMAL_MORPHOLOGY_OF_BONY_ORBIT_OF_SKULL	HP_ABNORMAL_MORPHOLOGY_OF_BONY_ORBIT_OF_SKULL	HP_ABNORMAL_MORPHOLOGY_OF_BONY_ORBIT_OF_SKULL	13	0.522423458	1.994953512	0.005739177	0.045097552	0.034184331	111	tags=46%, list=4%, signal=45%	FBN1/MAF/SETBP1/FGFR1/PDGFRB/ZMPSTE24
GOCC_TERTIARY_GRANULE_MEMBRANE	GOCC_TERTIARY_GRANULE_MEMBRANE	GOCC_TERTIARY_GRANULE_MEMBRANE	13	0.521784052	1.992511841	0.005739177	0.045097552	0.034184331	1175	tags=85%, list=37%, signal=53%	CD177/SLC11A1/TRPM2/PLAU/FCER1G/STOM/TMEM63A/ITGAM/MCEMP1/FCAR/KCNAB2
GOCC_MIDBODY	GOCC_MIDBODY	GOCC_MIDBODY	47	-0.275157511	-1.914032619	0.005708456	0.045097552	0.034184331	420	tags=32%, list=13%, signal=28%	MYH10/C6orf89/SSNA1/STK17B/KIF23/PPP1CC/CENPE/KATNB1/C1orf112/AURKC/CDCA8/KATNA1/SPAG5/SH3GLB1/SCCPDH
GOBP_REGULATION_OF_DNA_TEMPLATED_TRANSCRIPTION_IN_RESPONSE_TO_STRESS	GOBP_REGULATION_OF_DNA_TEMPLATED_TRANSCRIPTION_IN_RESPONSE_TO_STRESS	GOBP_REGULATION_OF_DNA_TEMPLATED_TRANSCRIPTION_IN_RESPONSE_TO_STRESS	10	0.55293061	1.904930531	0.005710122	0.045097552	0.034184331	1233	tags=90%, list=39%, signal=55%	CEBPB/NFE2L2/ATF4/CITED2/RELA/SIRT2/KLF2/RGS14/EGR1
GOMF_SALT_TRANSMEMBRANE_TRANSPORTER_AC	GOMF_SALT_TRANSMEMBRANE_TRANSPORTER_AC	GOMF_SALT_TRANSMEMBRANE_TRANSPORTER_ACTIVITY	106	0.21728136	1.747224852	0.005731623	0.045097552	0.034184331	2040	tags=83%, list=65%, signal=30%	SLC44A1/ITPR1/TMCO3/SLC25A23/CLIC1/CLCC1/SLC25A5/ATP1A2/TPCN1/SLC30A9/CCDC51/ANXA6/SLC29A1/SLC2

TIVITY	TIVITY									5A12/KCNK5/SLC12A9/KCNE3/KCNB1/SLC34A2/SLC9A8/SLC26A1/TRPM2/ATP2A3/GRIK5/SEC61A1/SLC16A11/CLIC5/SLC26A5/KCNH4/GABRR1/SLC24A3/KCNB2/KCNJ1/CLDN4/SLC10A5/KCNAB2/SLC6A1/AQP1/HTR1B/AQP6/KCNJ6/KCNH7/SLC5A7/TRPV1/HCN4/SLC22A2/CLCN6/CACNA2D3/HCN3/CACNA1B/KCNQ3/SLC34A3/KCNS2/CACNA1E/SLC12A8/SLC25A13/SLC12A3/CACNB3/SLC17A7/ATP1A3/ABCG2/ATP2A1/TRPV5/SLC17A8/SCN2B/SLC23A3/SLC12A1/KCNMB3/SLC4A1/ATP7B/SLC16A7/SLC16A1/ATP2B3/KCND2/KCNMB2/CHRNA7/KCNMB1/KCNAB3/KCNS3/SLC25A22/CLCN2/RASA3/CHRNA10/SLC5A11/KCNN1/GRIN1/GRIN2D/SLC25A1
HP_GENERALIZED_ABNORMALITY_OF_SKIN	HP_GENERALIZED_ABNORMALITY_OF_SKIN	HP_GENERALIZED_ABNORMALITY_OF_SKIN	217	0.17799643	1.718887478	0.005721636	0.045097552	0.034184331	810	tags=35%, list=26%, signal=28%	CCDC115/RNF113A/FBN1/NFKB1/PLOD1/ANTXR2/SLC44A1/F10/NFIA/FGFR1/GYPC/PDGFRB/CDH11/GALT/SCARB2/MED12/ZMPSTE24/ABL1/EIF2AK4/COX7B/KRT14/CFHR3/GLB1/HLA-DQB1/PERP/ATP1A2/COL3A1/LIPA/BAZ1B/STAT3/HLA-B/MANBA/HPS6/PIGW/GALM/MRAS/GTF2IRD2/NKX2-5/ALDH2/HPS1/CITED2/MMP23B/SNAI2/HMGCL/CYLD/FECH/TSC1/IRAK1/VWF/TOR1A/BMP2/CTCF/EDA/MPV17/SLC34A2/ELN/PPOX/MEFV/FZD4/CBS/AP2M1/KRT85/PLAU/BRCC3/SLC25A20/NR3C1/PRPS1/SLC29A3/MAGEL2/ITGA3/TRMU/CDH3/PTDSS1/SERPINA1/ENG
HP_POINTED_CHIN	HP_POINTED_CHIN	HP_POINTED_CHIN	24	0.3954223	1.921432392	0.005761703	0.045200703	0.03426252	416	tags=46%, list=13%, signal=40%	SETBP1/RALA/CCDC8/PDGFRB/CDH11/ABL1/BAZ1B/MRAS/GTF2IRD2/MMP23B/CBFB
GOBP_REGULATION_OF_MYELOID_CELL_DIFFERENTIATION	GOBP_REGULATION_OF_MYELOID_CELL_DIFFERENTIATION	GOBP_REGULATION_OF_MYELOID_CELL_DIFFERENTIATION	38	0.337740884	1.953462057	0.005777476	0.045250625	0.034300361	1171	tags=63%, list=37%, signal=40%	FBN1/CEBPB/TFE3/TYROBP/HCLS1/MAFB/PPP3CA/STAT3/C1QC/INHBA/MYC/PIAS3/ISG15/ID2/PTPN2/LYN/TCTA/IL17A/KLF10/CCR1/CD4/PRMT6/ETS1/ADIPOQ
HP_TOE_DEFORMITY	HP_TOE_DEFORMITY	HP_TOE_DEFORMITY	18	0.457273105	1.988980541	0.005822533	0.04546164	0.034460313	588	tags=50%, list=19%,	FBN1/SETBP1/MED12/TPM2/SBF2/TOR1A/PDXK/MPV17/PRX

										signal=41%	
GOBP_CALCIUM_ION_TRANSMEMBRANE_IMPORT_INTO_CYTOSOL	GOBP_CALCIUM_ION_TRANSMEMBRANE_IMPORT_INTO_CYTOSOL	GOBP_CALCIUM_ION_TRANSMEMBRANE_IMPORT_INTO_CYTOSOL	42	0.325816777	1.951853963	0.005823325	0.04546164	0.034460313	1620	tags=76%, list=51%, signal=38%	ABL1/GSTO1/ITPR1/PPP3CA/ATP1A2/PTPRC/TPCN1/FKBP1A/CCL21/BCL2/SRI/IBTK/LYN/TRPM2/F2RL3/CCR5/CALM3/LIME1/BAX/DIAPH1/XCR1/TRPV1/P2RX2/THY1/ATG5/PLA2G1B/CACNA1B/CACNA1E/CACNB3/GRM6/TRPV5/BDKRB1
GOCC_CORTICAL_CYTOSKELETON	GOCC_CORTICAL_CYTOSKELETON	GOCC_CORTICAL_CYTOSKELETON	25	0.382002753	1.891347745	0.005926846	0.046194815	0.035016065	1388	tags=80%, list=44%, signal=45%	LASP1/GYPC/IQGAP1/CAP1/TMOD1/FCHSD1/FLOT2/MYADM/DSTN/LLGL1/CLDN5/GYS2/PDLIM2/ACTN2/DLC1/CTTN/SPTAN1/COTL1/DLG4/LLGL2
GOBP_POSITIVE_REGULATION_OF_LEUKOCYTE_MIGRATION	GOBP_POSITIVE_REGULATION_OF_LEUKOCYTE_MIGRATION	GOBP_POSITIVE_REGULATION_OF_LEUKOCYTE_MIGRATION	26	0.383101682	1.937057148	0.00605089	0.047085327	0.035691081	1412	tags=77%, list=45%, signal=43%	PYCARD/HMGB1/ABL1/VEGFB/AIF1/RAC1/C3AR1/PTN/CCL21/CCL5/MMP14/CXCL13/AGER/CCR1/SPN/CX3CR1/THY1/OXSR1/GPSM3/S100A14
GOBP_MAPK_CASCADE	GOBP_MAPK_CASCADE	GOBP_MAPK_CASCADE	139	0.212474193	1.84655653	0.006114029	0.047499784	0.036005243	1209	tags=51%, list=38%, signal=33%	SHC1/NFKB1/FGFR1/PDGFRB/SLA/PHB2/DAB2/IGFBP4/PYCARD/IGF2/IQGAP1/HMGB1/ABL1/HAVCR2/EIF2AK2/MAPKAPK3/ITGB1BP1/INHBA/MYC/GADD45A/PTPRC/HAND2/LTBR/MID1/IGFBP6/HIPK2/MAP3K5/TGFA/BMP4/MYOC/CCL21/CYLD/IRAK1/PRDX1/CDC42EP5/BMP2/TEK/ZDHHC9/CCL5/MAPK4/CTSH/BTN2A2/PTPN2/LYN/GRM1/PPP2R1A/FGF1/CCR5/NTF3/FRS2/ERN1/AGER/NRP1/CCR1/MAP2K3/INS/CD4/DVL2/AVPI1/MINK1/FGF19/FGF13/ROCK2/FSHR/ARRB2/CCL8/IQGAP3/ADIPOQ/PJA2/ATF2/RGS14
HP_HAMMERTOE	HP_HAMMERTOE	HP_HAMMERTOE	10	0.549105204	1.891751423	0.006161765	0.047793433	0.036227831	588	tags=60%, list=19%, signal=49%	FBN1/SBF2/TOR1A/PDXK/MPV17/PRX
GOBP_RESPONSE_TO_REACTIVE_OXYGEN_SPECIES	GOBP_RESPONSE_TO_REACTIVE_OXYGEN_SPECIES	GOBP_RESPONSE_TO_REACTIVE_OXYGEN_SPECIES	45	0.308077407	1.899633727	0.006172516	0.047799728	0.036232603	972	tags=56%, list=31%, signal=39%	ABL1/H19/CYP1B1/CAT/HP/NFE2L2/MAP3K5/NQO1/PRDX1/CCS/MPV17/BCL2/TRPM2/SCGB1A1/RELA/PRDX3/ANKZF1/APOD/ERN1/NET1/SQSTM1/MDM2/KLF2/PPIF/PDK2
GOBP_BIOLOGICAL_PROCESS_INVOLVED_IN_INTERACTION_WITH_H	GOBP_BIOLOGICAL_PROCESS_INVOLVED_IN_INTERACTION_WITH_HOS	GOBP_BIOLOGICAL_PROCESS_INVOLVED_IN_INTERACTION_WITH_HOST	43	0.310642504	1.873217017	0.006193264	0.047883293	0.036295946	1594	tags=79%, list=51%, signal=40%	FUCA2/IFITM3/SCARB2/HMGB1/IFITM1/TRIM8/PLSCR1/TPCN1/ITGB1/VAPA/ITGB7/BCL2L1/UVRAG/GPR15/ITGA5/CCR5/CHMP2B/NRP1/CD4/CHMP4C/CH

OST	T										MP6/CHMP7/VPS4A/VPS18/SELPLG/TNFRSF4/AKT1/F11R/LTF/DDB1/PPARA/CD209/CR2/TMPRSS2
HP_ABNORMALITY_OF_THE_METOPIC_SUTURE	HP_ABNORMALITY_OF_THE_METOPIC_SUTURE	HP_ABNORMALITY_OF_THE_METOPIC_SUTURE	19	0.436023545	1.926533008	0.006204335	0.047891886	0.036302459	780	tags=58%, list=25%, signal=44%	MAF/SETBP1/NFIA/FGFR1/IGF2/STAT3/MID1/PURA/ZNF462/CDKN1C/PPP2R1A
GOBP_POSITIVE_REGULATION_OF_CELL_KILLING	GOBP_POSITIVE_REGULATION_OF_CELL_KILLING	GOBP_POSITIVE_REGULATION_OF_CELL_KILLING	16	0.473953159	1.978571441	0.006256354	0.048061988	0.036431398	348	tags=50%, list=11%, signal=45%	LAG3/TYROBP/HLA-B/HLA-E/HLA-F/PTPRC/HLA-A/HLA-C
HP_DIABETES_INSIPIDUS	HP_DIABETES_INSIPIDUS	HP_DIABETES_INSIPIDUS	14	0.483170764	1.888308232	0.006246503	0.048061988	0.036431398	854	tags=64%, list=27%, signal=47%	FGFR1/GLI2/HLA-B/BTNL2/WFS1/GPR161/PRPS1/STAG2/PROKR2
HP_ABNORMAL_CORNEA_MORPHOLOGY	HP_ABNORMAL_CORNEA_MORPHOLOGY	HP_ABNORMAL_CORNEA_MORPHOLOGY	133	0.209932248	1.790898926	0.006252689	0.048061988	0.036431398	798	tags=37%, list=25%, signal=29%	RNF113A/FBN1/PLOD1/MAF/SUMF1/SETBP1/FGFR1/SH3PXD2B/PDGFRB/CDH11/TBC1D2B/TFE3/IFNGR1/CTBP1/ZMPSTE24/TGFBI/COX7B/KRT14/CYP1B1/GLB1/MAFB/COL3A1/BAZ1B/COL4A5/HLA-B/BTNL2/COL4A6/BMP4/GTF2IRD2/TEK/ZBTB7A/ROBO3/IDH3B/CTCF/FOXC1/MPV17/ELN/MEFV/AIRE/PTPN2/MMP14/FZD4/PIGG/COL4A1/SLC29A3/PRDX3/YAP1/PRR12/IMPG2
GOBP_MOLTING_CYCLE_PROCESS	GOBP_MOLTING_CYCLE_PROCESS	GOBP_MOLTING_CYCLE_PROCESS	23	0.389627468	1.852255002	0.006275562	0.048132663	0.03648497	887	tags=52%, list=28%, signal=38%	ZMPSTE24/SMO/GLI2/INHBA/APCDD1/DNASE1L2/EDA/BCL2/RELA/CDH3/NSDHL/KRT27
GOBP_REGULATION_OF_LYMPHOCYTE_MIGRATION	GOBP_REGULATION_OF_LYMPHOCYTE_MIGRATION	GOBP_REGULATION_OF_LYMPHOCYTE_MIGRATION	13	0.520166592	1.986335325	0.006309918	0.048319104	0.036626294	977	tags=77%, list=31%, signal=53%	MSN/PYCARD/ABL1/AIF1/CCL21/CCL5/AIRE/CXCL13/APOD/SPN
GOBP_NON_MOTILE_CILIUM_ASSEMBLY	GOBP_NON_MOTILE_CILIUM_ASSEMBLY	GOBP_NON_MOTILE_CILIUM_ASSEMBLY	12	-0.485697108	-1.945174326	0.006340496	0.048476063	0.036745271	921	tags=75%, list=29%, signal=53%	MAPRE1/CEP290/MKKS/FUZ/BBS4/CEP350/CEP250/IFT74/IFT122
GOBP_MULTICELLULAR_ORGANISMAL_LEVEL_CHEMICAL_HOMEOSTASIS	GOBP_MULTICELLULAR_ORGANISMAL_LEVEL_CHEMICAL_HOMEOSTASIS	GOBP_MULTICELLULAR_ORGANISMAL_LEVEL_CHEMICAL_HOMEOSTASIS	14	0.482676681	1.886377275	0.00643675	0.049133857	0.037243884	611	tags=57%, list=19%, signal=46%	CYBRD1/EIF2AK1/LIPA/WFS1/FECH/SLC11A1/CTSH/HAS2
GOMF_RNA_EXONUCLEASE_ACTIVITY	GOMF_RNA_EXONUCLEASE_ACTIVITY	GOMF_RNA_EXONUCLEASE_ACTIVITY	11	-0.528460576	-1.996011479	0.006517123	0.049209094	0.037300914	749	tags=73%, list=24%, signal=56%	CNOT6/ANGEL1/CNOT7/PNPT1/POLRMT/EXOSC8/EXOSC4/CPSF3
GOCC_LATE_ENDOSOME	GOCC_LATE_ENDOSOME	GOCC_LATE_ENDOSOME	71	0.267152919	1.937113641	0.006526437	0.049209094	0.037300914	614	tags=38%, list=19%,	WDR81/IFITM3/FYCO1/SCARB2/HLA-DMB/ZMPSTE24/SMO/HLA-DRB3/OSB

										signal=31%	PL1A/HLA-DQB1/TMEM25/HLA-DRB5/STEAP3/HLA-DMA/ANXA6/IGF2R/CLN3/HLA-DOB/SLC11A1/CTSH/SLC9A8/MITD1/ABHD6/RAB11A/DERL1/SLC31A1/VPS13C
HP_NAIL_DYSTROPHY	HP_NAIL_DYSTROPHY	HP_NAIL_DYSTROPHY	24	0.392297922	1.906250442	0.006502381	0.049209094	0.037300914	776	tags=54%, list=25%, signal=41%	RNF113A/MAF/PQBP1/ZMPSTE24/COX7B/KRT14/PERP/STAT3/HLA-B/AIRE/CTSC/KRT85/ITGA3
HP_ABNORMAL_VASCULAR_PHYSIOLOGY	HP_ABNORMAL_VASCULAR_PHYSIOLOGY	HP_ABNORMAL_VASCULAR_PHYSIOLOGY	73	0.260871423	1.894279769	0.006470175	0.049209094	0.037300914	736	tags=41%, list=23%, signal=32%	FBN1/PLOD1/FGFR1/SCARB2/HTRA1/MED12/MGP/IFNGR1/ZMPSTE24/EIF2AK4/COX7B/COX8A/COL3A1/BAZ1B/HLA-B/BTNL2/GTF2IRD2/NKX2-5/CITED2/IRAK1/CTCF/SLC34A2/ELN/MEFV/LARS2/CBS/BRCC3/COL4A1/NR3C1/SLC29A3
GOBP_REGULATION_OF_G_PROTEIN_COUPLED_RECEPTOR_SIGNALING_PATHWAY	GOBP_REGULATION_OF_G_PROTEIN_COUPLED_RECEPTOR_SIGNALING_PATHWAY	GOBP_REGULATION_OF_G_PROTEIN_COUPLED_RECEPTOR_SIGNALING_PATHWAY	32	0.347897674	1.890912412	0.006528468	0.049209094	0.037300914	1262	tags=69%, list=40%, signal=42%	UBQLN2/RGS10/PPP3CA/KCTD12/RAMP2/CCL5/ABHD6/RPH3AL/APLN/YWHAB/ADM/RGS11/CRTC3/RGS6/ROCK2/ARRB2/GNG7/RGS14/GRK1/PDCL/HTR1B/ADA
GOBP_DNA_BIOSYNTHETIC_PROCESS	GOBP_DNA_BIOSYNTHETIC_PROCESS	GOBP_DNA_BIOSYNTHETIC_PROCESS	39	-0.289167359	-1.887034545	0.006504913	0.049209094	0.037300914	866	tags=56%, list=27%, signal=41%	RFC1/VCP/RCHY1/POLI/ATR/MAP3K4/SIRT1/RAD50/POT1/USP10/WRN/TFDP1/PINX1/POLE/MAD2L2/PCNA/MAPK3/USP1/GFER/RFC4/POLG2/POLB
HP_ABNORMAL_FINGERNAIL_MORPHOLOGY	HP_ABNORMAL_FINGERNAIL_MORPHOLOGY	HP_ABNORMAL_FINGERNAIL_MORPHOLOGY	40	0.316173524	1.850763813	0.006502381	0.049209094	0.037300914	800	tags=50%, list=25%, signal=38%	RNF113A/EFNB1/IGF2/ZMPSTE24/KRT14/PERP/BAZ1B/STAT3/HLA-B/GTF2IRD2/PLAGL1/ELN/AIRE/PAX9/PLAG1/ZNF462/CTSC/CDKN1C/CDH3/PTDSS1
HP_CONSTITUTIONAL_SYMPTOM	HP_CONSTITUTIONAL_SYMPTOM	HP_CONSTITUTIONAL_SYMPTOM	235	0.169379323	1.658255111	0.006509026	0.049209094	0.037300914	606	tags=28%, list=19%, signal=24%	FBN1/NFKB1/CTSK/ANTXR2/SLC44A1/UBQLN2/FGFR1/GYPC/PDGFRB/DPH5/SCARB2/HTRA1/MED12/PGK1/IFNGR1/ZMPSTE24/TYROBP/ABL1/EMP2/EIF2AK4/TGFBI/SMO/HAVCR2/H19/KRT14/EIF2AK2/MAFB/GLI2/HLA-DQB1/CAT/IMPDH2/COL3A1/BAZ1B/APRT/STAT3/COL4A5/HLA-B/MYC/GNE/ARHGDIA/STEAP3/BTNL2/COL4A6/TMEM126B/GTF2IRD2/NKX2-5/MTHFD1/HPS1/CITED2/TPM2/HMGCL/TSC1/IRAK1/BMP2/TEK/ALAD/SLC11A1/CHRNB2/SLC34A2/SLC26A1/ELN/PPOX/BCL2/MEFV/PODXL

HP_ABNORMALITY_OF_THE_GINGIVA	HP_ABNORMALITY_OF_THE_GINGIVA	HP_ABNORMALITY_OF_THE_GINGIVA	56	0.276602981	1.835135158	0.006560734	0.049374912	0.037426606	676	tags=41%, list=21%, signal=33%	ANTXR2/SETBP1/F10/SH3PXD2B/PDGFRB/TBC1D2B/SCARB2/GLB1/TRIM8/CAT/PERP/COL3A1/BAZ1B/STAT3/GTF2IRD2/PLAGL1/HPS1/TSC1/DHCR24/ELN/MMP14/CTSC/CDKN2C
GOBP_NEGATIVE_REGULATION_OF_TOR_SIGNALING	GOBP_NEGATIVE_REGULATION_OF_TOR_SIGNALING	GOBP_NEGATIVE_REGULATION_OF_TOR_SIGNALING	20	-0.415218068	-2.07327287	0.006585021	0.04941614	0.037457857	973	tags=70%, list=31%, signal=49%	WDR24/FLCN/KPTN/TBC1D7/ENDOG/SIRT1/YWHAZ/ATXN3/TBK1/MAPK3/UBE3A/UBE2N/TSSK2/PRKAA1
HP_CHIARI_MALFORMATION	HP_CHIARI_MALFORMATION	HP_CHIARI_MALFORMATION	19	0.433295307	1.91447852	0.006586763	0.04941614	0.037457857	368	tags=47%, list=12%, signal=42%	FBN1/CTSK/MAF/SETBP1/NFIA/FGFR1/SMO/BAZ1B/GTF2IRD2
GOBP_REGULATORY_NCRNA_MEDIATED_GENE_SILENCING	GOBP_REGULATORY_NCRNA_MEDIATED_GENE_SILENCING	GOBP_REGULATORY_NCRNA_MEDIATED_GENE_SILENCING	25	-0.365713942	-2.029450175	0.006636642	0.049712787	0.037682718	568	tags=52%, list=18%, signal=43%	CNOT7/SMAD2/TIAL1/SPOCD1/PIWIL2/EXOSC4/TDRKH/FKBP6/TDRD7/CNOT10/TSN/NUP155/NCBP2
GOBP_ACTIVATED_T_CELL_PROLIFERATION	GOBP_ACTIVATED_T_CELL_PROLIFERATION	GOBP_ACTIVATED_T_CELL_PROLIFERATION	10	0.546844609	1.883963327	0.006678565	0.049949019	0.037861784	1076	tags=80%, list=34%, signal=53%	PYCARD/IGF2/HMGB1/ABL1/PPP3CA/BTN2A2/AGER/PRKAR1A
